# Common Genomic Aberrations in Mouse and Human Breast Cancers with Concurrent P53 Deficiency and Activated PTEN-PI3K-AKT Pathway

**DOI:** 10.7150/ijbs.65763

**Published:** 2022-01-01

**Authors:** Jarrod D. Martinez, Qianxing Mo, Yixiang Xu, Li Qin, Yi Li, Jianming Xu

**Affiliations:** 1 Department of Molecular and Cellular Biology, Baylor College of Medicine, Houston, TX 77030.; 2 Dan L. Duncan Comprehensive Cancer Center, Baylor College of Medicine, Houston, TX 77030.

**Keywords:** breast cancer, PTEN, P53, mouse model, genome sequencing, genomic aberration

## Abstract

Simultaneous P53 loss and activation of the PTEN-restricted PI3K-AKT pathway frequently occur in aggressive breast cancers. P53 loss causes genome instability, while PTEN loss and/or activating mutations of PIK3CA and AKT promote cancer cell proliferation that also increases incidences of genomic aberrations. However, the genomic alterations associated with P53 loss and activated PTEN-PI3K-AKT signaling in breast cancer have not been defined. Spatiotemporally controlled breast cancer models with inactivation of both P53 and Pten in adult mice have not been established for studying genomic alterations. Herein, we deleted both floxed *Pten* and *Tp53* genes in the mammary gland epithelial cells in adult mice using a RCAS virus-mediated Cre-expressing system. These mice developed small tumors in 21 weeks, and poorly differentiated larger tumors in 26 weeks. In these tumors, we identified 360 genes mutated by nonsynonymous point mutations and small insertions and deletions (NSPMs/InDels), 435 genes altered by copy number amplifications (CNAs), and 450 genes inactivated by copy number deletions (CNDs). Importantly, 22.2%, 75.9% and 27.3% of these genes were also altered in human breast tumors with P53 and PTEN losses or P53 loss and activated PI3K-AKT signaling by NSPMs/InDels, CNAs and CNDs, respectively. Therefore, inactivation of P53 and Pten in adult mice causes rapid-growing breast tumors, and these tumors recapitulate a significant number of genetic aberrations in human breast tumors with inactivated P53 and activated PTEN-PI3K-AKT signaling. Further characterization of these commonly altered genes in breast cancer should help to identify novel cancer-driving genes and molecular targets for developing therapeutics.

## Introduction

Genome stability is crucial for maintaining normal cellular division, differentiation, growth, and function. Interference with genome stability usually causes the cell to undergo cell cycle arrest or senescence, allowing time for the cell to repair DNA damage. If the damage is repaired, the cell may continue with the cell cycle. If the damage cannot be repaired, the cell will undergo apoptosis. If the damage is too severe and cannot be repaired but enough advantageous genetic aberrations have accrued from increased genome instability, a cell can transform into a cancer cell with uncontrolled proliferation 1-5. Several proteins including P53 that protect genome stability are known as *Guardians of the Genome* 1-5. Acting as a transcription factor, P53 is a critical modulator of cell division, survival and apoptosis in response to DNA damage and other cellular stress responses 6-8. Being a potent tumor suppressor, P53 has many known mutations concentrated in its central DNA binding domain that inactivate its transcriptional activity 6, 9, 10. Many other mutations also act as dominant negative forms to inhibit P53 function 6, 9, 10. Homozygous *TP53* losses are uncommon in breast cancers possibly due to essential genes in its adjacent chromosomal loci 6, 11. The loss of P53 function in cancer is usually attributed to heterozygous deletion of one *TP53* allele plus heterozygous mutation of the other *TP53* allele 6, 12. The loss of P53 function frequently increases large-scale chromosomal instability such as gene copy number variations (CNVs) in cancer cells 6. Disruption of *Tp53* or expression of mutant P53 can slowly induce mammary tumorigenesis with various kinetics and penetrance in mouse models 13, 14. Heterozygous knockout of *Tp53* in mice causes a Li-Fraumeni syndrome phenotype, which is characterized by the development of different cancers 15. Mice with homozygous knockout of *Tp53* survive for about 3-6 months due to the development of lymphomas 15, 16. Transplant of mammary epithelial cells from BALB/c-*Trp53*^-/-^ mice into the cleared fat pads of wild type mice leads to mammary tumor development in about 8-12 months in roughly 60% of the recipient mice 17-21. This incomplete penetrance and the long latency suggest that accumulation of additional genetic aberrations is required for P53 deficiency-triggered mammary tumorigenesis.

The PTEN-PI3K-AKT pathway is also frequently altered in breast cancers. In the METABRIC-TCGA data sets, *PTEN* is inactivated in 8% of breast cancers, and activating mutations in *PIK3CA*, the gene for the p110a catalytic subunit of PI3K, and *AKT1* are detected in 40% and 5% of breast cancers, respectively 22, 23. Homozygous *Pten* knockout in mice results in embryonic lethality. Heterozygous *Pten* knockout mice are viable, but develop hyperplasia or tumors with a long latency in multiple tissues including breast, prostate, and endometrium 24-26. Mammary gland specific knockout of *Pten* has a long tumorigenic latency, suggesting that additional genetic alternations are required to work with Pten deficiency to produce aggressive breast cancers 24, 27. Expression of the constitutively active *AKT1* (E17K) mutant in mice is able to induce premalignant lesions. Expression of the *PIK3CA* (H1047R or E545K) mutant in mice is sufficient to induce mammary tumorigenesis 22, 23, 28-31. Therefore, activation of the PTEN-PI3K-AKT1 pathway by either PTEN loss or PIK3CA or AKT1 activating mutations induces breast cancer development.

Loss of PTEN activates PIK3CA and AKT1, which inhibit apoptosis and increase Cyclin D expression to promote cell cycle progression. Activation of the PTEN-PI3K-AKT1 pathway also enhances P53 and MDM2 interaction, leading to P53 ubiquitination and degradation 32-36. PTEN can also associate with P53 to enhance P53 stability and regulate its DNA-binding capability for modulating the transcriptional activity of P53 to maintain cellular homeostasis 34, 35, 37, 38. These interactions between PTEN and P53 are likely correlated with the concurrent activation of the PTEN-PI3K-AKT1 pathway and inactivation of the P53 pathway. However, the spectrum of *TP53* loss combined with *PTEN* loss and/or PIK3CA or AKT1 activation has not been modeled in spontaneous breast tumors developed from the mammary gland epithelial cells of adult mice. The genetic aberrations associated with both the loss of *TP53* function and the activation of the PTEN-PI3K-AKT1 pathway also have not been analyzed in human breast cancers and compared between human and mouse breast cancers.

In this study, we generated a unique breast cancer mouse model induced by dual *Pten* and *Tp53* inactivation (PtenI/Tp53I) in the mammary gland epithelial cells (MGECs) of adult mice, and sequenced the whole genome DNA of these mouse breast tumors. We found that these mice developed fast-growing breast tumors containing hundreds of somatically mutated, amplified or deleted genes, and many of these altered genes are also mutated, amplified or deleted in human breast cancers with both deficient P53 and activated PTEN-PI3K-AKT pathway.

## Materials and Methods

### Induction of mouse mammary gland tumorigenesis

A Cre-coding DNA fragment with Not1 and Asc1 sites was amplified by PCR and subcloned into the RCAS-PyMT avian-viral vector to replace the PyMT coding sequence 39. The resulted RCAS-Cre vector was used to transfect DF-1 chicken fibroblast cells for producing RCAS-Cre avian virus as described previously 39, 40. MMTV-TVA mice with FVB strain background 39 were crossbred with Gt(ROSA)26Sortm4(ACTB-tdTomato,-EGFP)Luo/J (designated as *mT/mG*) mice 41 to generate heterozygous MMTV-TVA^+/-^; mT/mG^+/-^ mice. These mice were further crossbred with *Pten^F/F^* and *Tp53^F/F^* mice 42-44 to generate MMTV-TVA^+/-^; mT/mG^+/-^; *Tp53^F/F^*; *Pten^F/F^* mice. The *Tp53^F/F^* mice with a backcrossed FVB background were obtained from Dr. Jonker's lab 45-48. MMTV-TVA^+/-^; *mT/mG^+/-^*; *Pten^+/+^*; *Tp53^+/+^* mice from the same breeding colony were used as control mice. Genotype analysis was carried out by PCR using DNA samples prepared from a small piece of mouse ear tissue. PCR primers 5'-caagcactctgcgaactgag and 5'-gcatcttgccttcaaaaactt were used to detect *Pten* wild type (WT) (156 bp) and floxed alleles (328 bp). PCR primers 5'-aaggggtatgagggacaagg and 5'-gaagacagaaaaggggaggg were used to detect *Tp53* WT (431 bp) and floxed (584 bp) alleles. The Rosa26R mT/mG and the MMTV-TVA transgenes were genotyped as described previously 39, 49, 50. For inducing tumorigenesis, RCAS-Cre virus was introduced via intraductal nipple injection into the 4^th^ mammary gland(s) of 7-week-old mice as described previously 39, 40. The injected mice were examined once a week for detecting mammary tumor by palpation. Once a palpable tumor was detected, the tumor length (L) and width (W) were measured once a week to calculate tumor volume by 0.5×L×W^2^. MMTV-TVA^+/-^; *mT/mG^+/-^*; *Pten^F/F^*; *Tp53^F/F^* mice were sacrificed at 4 days, 2 months, and 6 months post viral introduction. Cre-mediated deletions of the floxed *Pten* and *Tp53* alleles were assayed by PCR as described previously 42-44. DNA samples were prepared from the tumors collected at 6 months. The PCR primers 5'-actcaaggcagggatgagc and 5'-gcttgatatcgaattcctgcagc were used to detect the deleted *Pten* allele (400 bp). The PCR primers 5'-cacaaaaacaggttaaaccca and 5'-gaagacagaaaaggggaggg were used to detect the deleted *Tp53* allele (612 bp). The animal protocol was approved by Institutional Animal Care and Use Committee at Baylor College of Medicine.

### Hematoxylin and Eosin (H&E) and immunofluorescence (IF) staining

Mammary glands and tumors were dissected from euthanized mice. Tissues were fixed overnight in 4% paraformaldehyde (PFA) in phosphate-buffered saline (PBS) at 4°C, washed in PBS, dehydrated in ethanol solution series, and embedded in paraffin. Five-μm thick sections were prepared and used for H&E and IF staining as described previously 51-53. For antibodies with a mouse origin, the M.O.M Mouse-on-Mouse Immunodetection kit (Vector Laboratories) was used to block immunostaining background caused by the endogenous mouse IgG. This study used primary antibodies against estrogen receptor alpha (ERα) (ab108398, abcam), progesterone receptor (PR) (A0098, Agilent DaKo), HER2 (2165, Cell Signaling), and green fluorescent protein (GFP) (632381, Clontech). Double IF was performed using the Tyramide Signal Amplification kit (Life technologies) according to the manufacturer's instructions. Stained tissue slides were examined and imaged under microscopes.

### Mouse genomic DNA preparation and DNA sequencing

Genomic DNA samples were prepared from mouse mammary gland tumors and normal healthy liver from the mice bearing the tumors as described previously 54, 55. The prepared DNA samples were analyzed by PCR to confirm the expected knockout of *Tp53* and* Pten* in tumors. DNA was quantified using the Thermo Scientific Nanodrop One. The prepared genomic DNA samples were sent to BGI sequencing company (Cambridge, MA) where the whole genomic DNA was fragmented randomly. After electrophoresis, the extracted DNA fragments with expected lengths was purified, the adapter primers were ligated to DNA fragments, and DNA was amplified into cluster by PCR, followed by sequencing on the HiSeq\reference (TM) sequencing system.

### Mouse DNA sequencing analysis and detection of mutations and InDels

The bioinformatics analysis of the sequencing data starts with the raw data. Clean sequence data was obtained by filtering the raw data by deleting contaminations and low-quality reads (rich in N or low-quality nucleotides). The cleaned sequence reads were mapped to the mouse reference Genome mm10 using the software Burrows-Wheeler Aligner (BWA) 56, and the mapped data was saved as formatted bam files. We then processed the bam files for labeling the repeated reads produced by PCR and recalibrating base quality score. After processing, the bam files were used for detecting mutations by using the Genome Analysis Toolkit (GATK) software, which discoveries variants in high throughput sequencing data for single nucleotide polymorphisms (SNPs), and short (typically less than 20 base pairs that alter protein) insertions and deletions (InDels) 57, 58. Somatic mutations were called by using GATK muTect2 in which non-tumor DNA samples from the livers were used as normal controls. ANNOVAR was used to annotate the variants. Identified SNPs were compared against the snp137 database 59 to identify previously reported SNPs. To determine whether a nonsynonymous point mutation (NSPM) or InDel that results in amino acid change in a protein has a significant effect on protein folding, stability or function, three independent mutation assessor algorithms including Sorting Tolerant From Intolerant (SIFT), Polymorphism Phenotyping-2 (PolyPhen-2), and Protein Variation Effect Analyzer (PROVEAN) were implemented to determine the potential functional impact of all mutations identified in the mouse breast tumor genomes 60-62.

### Mouse gene copy number variation (CNV) analysis

The bam files were used for detecting CNVs using the SOAPcnv software for mapping short reads against large genomic regions 63. The number of reads falling in 1000 bp genomic windows were counted and then normalized to the GC percentages and sequencing depth. The log2 ratio data (log2 normalized count of tumor - log2 normalized count of control) were used for the CNV analysis. CBS algorithm 56, 64, 65 was used for segmentation of the data. A region was called “amp” if the segmental mean value minus the global median value was 3-fold greater than the median absolute deviation (MAD) of the log2 ratios, and a region was called “del” if the segmental mean value minus the global median value was 3-fold less than the MAD of the log2 ratios.

### Analysis of human breast cancer datasets and identification of breast cancers with both P53 loss and PTEN loss or with P53 loss and activated PI3K-AKT pathway

Breast cancer genomic DNA sequence datasets were collected from the TCGA-Cell 2015 report and the METABRIC breast cancer datasets 66, 67. Only breast tumors with complete information of genomic mutations and gene CNVs were used for analysis. Breast tumors with both P53 and PTEN losses or with P53 loss and activated PI3K-AKT pathway, which was designated as PPAPA/TP53I cohort, were identified based on the genetic aberration profiles of the *PTEN* and *TP53* genes as well as the *PIK3CA (H1047R & E545K)* and *AKT1(E17K)* activating mutations. Activating point mutations were identified using the OncoKB, CIViC, and Cancer Hot Spot web interfaces 68, 69. The NSPM and InDel profiles for each human breast tumor were downloaded from the Cbioportal interface 22, 23, 66, 67. The NSPM/InDel mutations in human PPAPA/TP53I breast tumors were analyzed in same ways as the mutations in mouse Tp53I/PtenI breast tumors using SIFT, PolyPhen, and PROVEAN algorithms 60-62. CNAs and CNDs in human breast tumors were counted as whole gene amplifications and deletions or amplified and deleted genomic regions in genes longer than 1000 base pairs, respectively. The CNA and CND profiles of human breast tumors were downloaded from the Cbioportal interface 22, 23, 66, 67.

### Comparison of the genetic aberrations in the mouse mammary tumors and human breast cancers

Common genes affected by NSPMs/InDels, CNAs and CNDs in both mouse Tp53I/PtenI and human PPAPA/TP53I breast tumors were identified and subsequently sorted and compared using Microsoft Excel software. Due to the large numbers of CNV-affected genes in human breast cancers, the criteria for inclusion of CNVs and CNV-affected genes were that the CNV had to happen in two or more tumors in the cohort. The identified CNVs and CNV-affected genes in the human PPAPA/TP53I breast cancer cohort were compared to that in the mouse PtenI/Tp53I tumor cohort to determine the commonly altered genes. These commonly altered genes were subjected to Gene Ontology (GO) enrichment analysis and KEGG pathway analysis using DAVID Bioinformatics Resources (Version 6.8) 70, 71.

### Statistical analysis

The data sets of mouse tumor volumes were analyzed and plotted by using Prism 8 Software (GraphPad). Quantitative data were presented as Mean ± SEM. The statistical differences between the defined categories were analyzed by Chi-square or Two-tailed Student's t test by using Prism 8 Software (GraphPad). In all tests, p < 0.05 was considered significant.

## Results

### Dual inactivation of *Pten* and *Tp53* in the mouse MGECs causes rapid growing triple negative breast tumors

**Figure 1:**
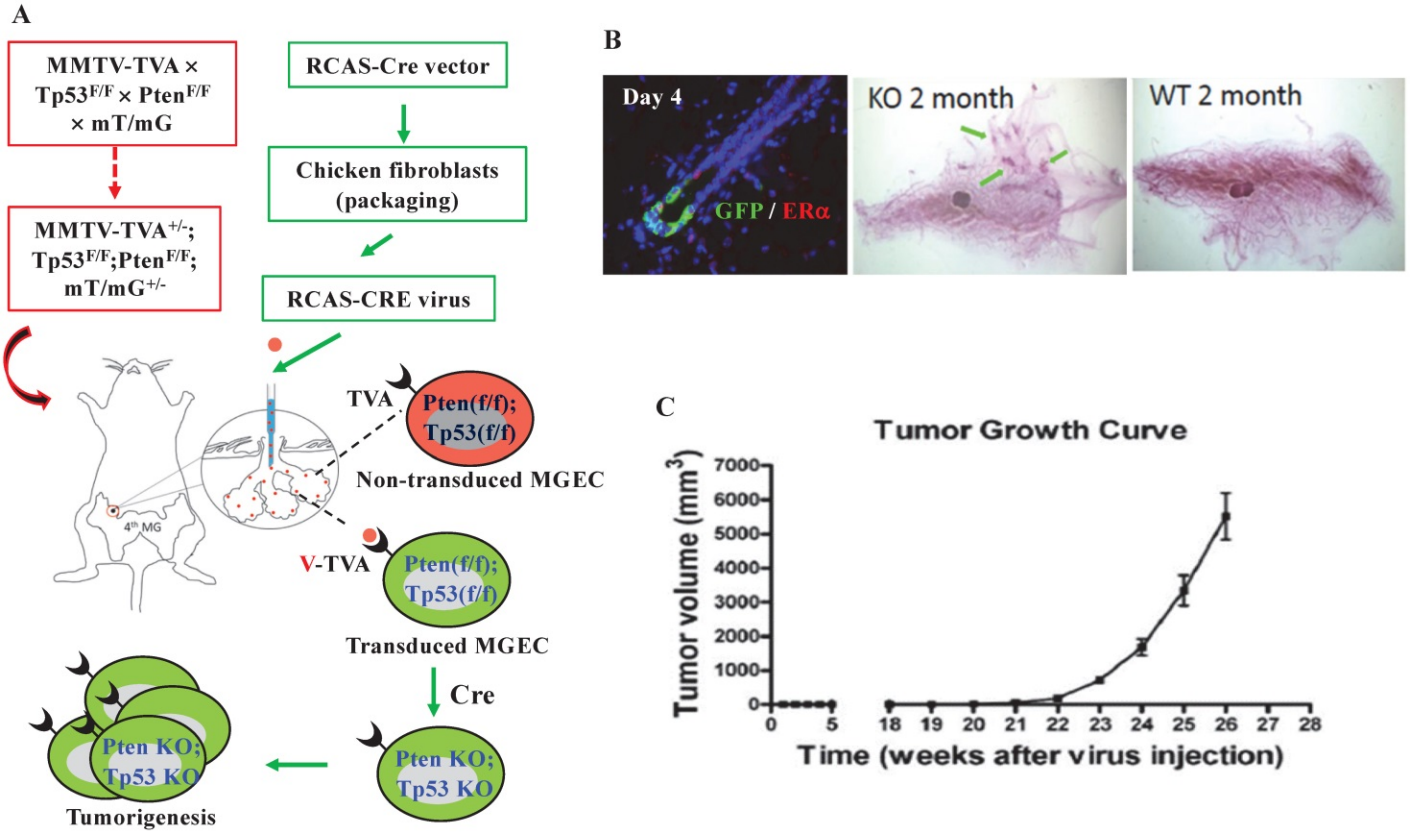
** Knockout of both *Pten* and *Tp53* in MGECs of adult mice causes fast-growing mammary gland tumors. A.** The combined RCAS-Cre virus and MMTV-TVA^+/-^; mT/mG^+/-^; *Pten*^f/f^; *Tp53*^f/f^ mouse model system. In this system, MGECs express TVA to accept RCAS-Cre virus. The virus-mediated Cre expression excises the floxed *Pten* and *Tp53* alleles as well as the floxed RFP at the Rosa locus to generate GFP-expressing MGECs with double knockout of *Pten* and *Tp53*. **B.** Double immunofluorescent (IF) staining for GFP and ERα on the mammary gland (MG) section of a MMTV-TVA^+/-^; mT/mG^+/-^; *Pten*^f/f^; *Tp53*^f/f^ mouse at day 4 post RCAS-Cre virus injection (left). Whole-mounted MG images, showing hyperplastic nodules in a MMTV-TVA^+/-^; mT/mG^+/-^; *Pten*^f/f^; *Tp53*^f/f^ mouse (central) and normal MG ducts in a MMTV-TVA^+/-^ control mouse (right) at 2 months post RCAS-Cre-viral injection. **C.** The tumor growth profile (n=8) in MMTV-TVA^+/-^; mT/mG^+/-^; *Pten*^f/f^; *Tp53*^f/f^ mice after receiving RCAS-Cre virus.

To get a mouse model that can closely mimic breast tumorigenesis triggered by somatic losses of both *Pten* and *Tp53* in adulthood, we generated MMTV-TVA^+/-^; mT/mG^+/-^; *Pten*^f/f^; *Tp53*^f/f^ mice, and injected RCAS-Cre virus into the lumens of the mammary gland ducts of these mice at 7 weeks of age. In these mice, the TVA protein is expressed in the luminal epithelial cells (LECs) of the mammary gland and serves as the receptor for the RCAS-Cre virus 39, allowing LEC-specific Cre expression to excise the floxed *Pten* and *Tp53* for inducing LEC-specific tumorigenesis. The Cre also converts the mT/mG reporter from expressing RFP to expressing GFP by deleting the floxed RFP-coding sequence preceding the GFP-coding sequence 41 (Fig. 1A). Four days after viral injection, about 0.9% of the mammary gland LECs expressed GFP, and GFP-positive LECs could be either positive or negative to ERα expression. By two months, ductal hyperplastic lesions were observed in the whole-mounted mammary glands. As negative controls, we also introduced the RCAS-Cre virus into the lumens of female MMTV-TVA mice with wild type *Pten* and *Tp53* alleles that do not respond to Cre expression. We did not detect any GFP-positive cells at day 4 or any hyperplasia at month 2 in the mammary glands of these control mice, indicating that the mammary tumorigenesis was dependent on the RCAS-Cre-mediated deletion of the floxed *Pten* and *Tp53* genes (Fig. 1B). By 21 weeks, palpable mammary tumors were detected in MMTV-TVA^+/-^; mT/mG^+/-^; *Pten*^f/f^; *Tp53*^f/f^ mice. By 26 weeks (or 6 months), the average of tumor size of the 8 tumors collected reached an average volume of 5.5 cm^3^, which was designated as experimental end point (Fig. 1C). These results demonstrate that a small number of RCAS-Cre virus-transduced LECs in the mammary glands of MMTV-TVA^+/-^; mT/mG^+/-^; *Pten*^f/f^; *Tp53*^f/f^ mice developed rapid growing tumors.

**Figure 2:**
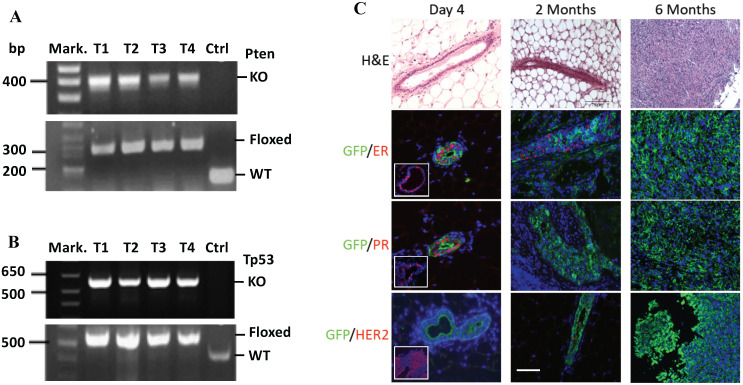
** MGECs with double Pten and Tp53 deficiency developed triple-negative mammary gland tumor. A.** PCR-based genotype analysis of the *Pten* alleles. DNA samples were prepared from four tumors (T1-T4) of MMTV-TVA^+/-^; mT/mG^+/-^; *Pten*^f/f^; *Tp53*^f/f^ mice and an ear tissue piece of a wild-type control (Ctrl) mouse. Specific primers were used to detect the knockout (KO) (400 bp), LoxP-containing (floxed) (328 bp), and wild type (WT) (156 bp) *Pten* alleles in the indicated samples. Mark., DNA size markers. **B.** PCR-based genotype analysis of the *Tp53* alleles. The same DNA samples used for *Pten* genotyping were used. Specific primers were used to detect the KO (612 bp), floxed (584 bp), and WT *Tp53* (413 bp) alleles in the indicated samples. **C.** H&E and double IF staining for GFP and ERα, PR, or HER2 on the mammary gland or tumor sections, which were prepared from MMT-TVA^+/-^; mTmG^+/-^; Tp53^f/f^; Pten^f/f^ mice at 4 days, 2 months, and 6 months after RCAS-Cre virus injection. The inserted photos in the left panels of the 2^nd^ and the 3^rd^ rows are for double IF of GFP & ERα and double IF of GFP and PR on mammary gland sections from MMT-TVA^+/-^; mTmG^+/-^; Tp53^f/f^; Pten^f/f^ mice without viral injection. The inserted photo in the left panel of the 4^th^ row is a positive control for HER2 IF staining on a HER2-expressing mouse tumor section. Scale bar represents 50 μm for all image panels.

PCR-based genotype analysis using DNA samples prepared from the mammary gland tumors containing both cancer and non-cancer cells detected both the knockout *Pten* and *Tp53* alleles in cancer cells infected by RCAS-Cre virus, and the floxed *Pten* and *Tp53* alleles in non-cancer cells that were not infected by RCAS-Cre virus (Fig. 2A-B). On the H&E-stained tumor sections, the mammary ducts exhibited normal morphology at day 4 and intraductal epithelial hyperplasia at month 2 post viral injection. By 6 months, solid tumors with poorly differentiated tumor cells developed. Double immunofluorescent staining revealed that more GFP-expressing cells transduced by the RCAS-Cre virus were ERα and PR positive and fewer numbers of these cells were ERα and PR negative on day 4 after viral injection. By 2 months, PR-positive cells were rare although some cells were still ERα positive. In the tumors at 6 months, all GFP-expressing tumor cells were negative to both ERα and PR. In addition, HER2 overexpression was not detected in these tumors (Fig. 2C). These results demonstrate that at 6 months the tumors induced by double *Pten* and *Tp53* deficiencies can be classified as triple negative breast tumors.

### Identification of genetic aberrations in the dual *Pten* and *Tp53* inactivation-induced mouse mammary tumors by whole-genome sequencing

**Figure 3:**
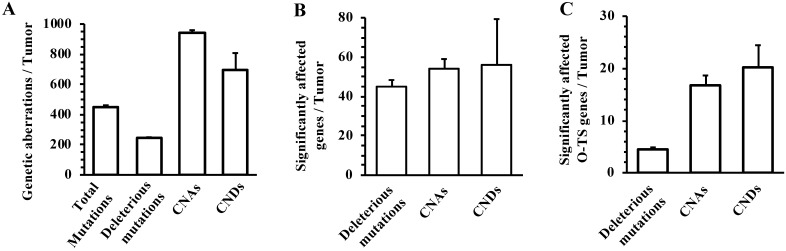
** The frequencies of NSPM/InDel, CNAs and CNDs in the mouse mammary tumors with double *Pten* and *Tp53* knockout. A.** The average numbers of total NSPM/InDel mutations, deleterious NSPM/InDel mutations, CNAs and CNDs per mouse tumor. **B.** The average numbers of significantly affected genes per mouse tumor by deleterious NSPM/InDel mutations, CNAs and CNDs. **C.** The average numbers of known oncogenes and tumor suppressor (O-TS) genes per tumor affected by deleterious NSPM/InDel mutations, CNAs and CNDs. The data in all panels are presented as Mean ± SEM (n=8).

We prepared tumor genomic DNA samples from 8 mammary tumors collected at 6 months after RCAS-Cre virus injection from MMTV-TVA^+/-^; mT/mG^+/-^; *Pten*^f/f^; *Tp53*^f/f^ mice and non-tumor genomic DNA samples from the tumor-free liver tissues collected at the same time from the same mice. We performed whole-genome sequencing and obtained specific NSPM/InDel and CNV profiles in the tumors by comparing the genomic DNA sequences of tumors to that of the livers. The eight mouse tumors had a total of 3614 site mutations and small InDels, which yielded an astonishing frequency of 451.7 such mutation events per tumor (Fig. 3A). From these total mutations, SIFT and PolyPhen analyses identified 1934 deleterious mutations that were distributed in 361 individual genes (Supplementary Table S1), providing frequencies of 241.8 such mutations and 45.1 mutated genes per mouse tumor (Fig. 3A and B). There were 82 and 71 genes that were mutated in ≥ 37.5% (3 out of 8) and ≥ 50% (4 out of 8) of mouse tumors, respectively (Supplementary Table S1). In addition to the experimentally deleted *Tp53* and *Pten* genes, other known oncogenes such as *Eef1a1*, *Chd4*, *Ret*, *Sf1*, and *Cdk4*, and tumor suppressor genes such as *Eef2*, *Kmt2c*, *Kmt2d*, and *Scaf4* were among the mutated genes. The number of deleterious mutation-affected oncogenes and tumor suppressor genes per tumor is shown in Fig. 3C. These results indicate that the breast cancer cells developed from *Tp53* and *Pten* deficient mouse MGECs contain many other mutated genes and the accumulation of these gene mutations may play an essential role in breast cancer initiation and progression induced by P53 and Pten deficiencies.

We identified 7519 CNAs from the 8 mouse tumors, with a frequency of 939.8 CNAs per tumor (Fig. 3A). These CNAs affected 435 genes with an average of 54.4 amplified genes per tumor (Fig. 3B). Among these 435 genes, 293 and 260 genes were amplified in ≥ 37.5% (3 out of 8) and ≥ 50% (4 out of 8) of mouse tumors, respectively (Supplementary Table S2). We also noticed a number of known oncogenes in the list of CNA-affected genes, including *Cdkn1a*, *Dach1*, *Erbb4*, *Klf5*, *Myc*, and *Ppp6c*. The number of CNA-affected oncogenes and tumor suppressor genes per tumor is shown in Fig. 3C. Furthermore, we found 5556 CNDs from the 8 mouse tumors, with a frequency of 694.5 CNDs per tumor (Fig. 3A). These CNDs affected 450 genes with a frequency of 56.3 deleted genes per tumor (Fig. 3B). Among these 450 genes, 311 and 261 genes were amplified in ≥ 37.5% (3 out of 8) and ≥ 50% (4 out of 8) of mouse tumors, respectively (Supplementary Table S3). In addition to the experimentally deleted *Tp53* and *Pten* genes, a number of other known tumor suppressor genes including *Pbrm1*, *Smad2*, *Smad4*, and *Tcf7l2* were found in the list of CND-affected genes. The number of CND-affected oncogenes and tumor suppressor genes per tumor is shown in Fig. 3C. These results indicate that inactivation of *Tp53* and *Pten* causes many CNAs and CNDs, resulting in acquisition and loss of many gene functions during breast cancer development and progression.

### Identification of human breast cancers with both inactivated *TP53* and activated PTEN-PI3K-AKT pathway

We searched and examined point mutations and InDels within the amino acid coding sequences, CNAs, and CNDs of the *TP53* and *PTEN* genes, and *PIK3CA*(H1047R & E545K) and *AKT1*(E17K) activating mutations in the METABRIC and TCGA human breast cancer datasets (MB-TCGA) from 3326 tumors 22, 23, 66, 72, 73. We grouped tumors with the inactivated *PTEN* gene or *PIK3CA*(H1047R & E545K) and *AKT1*(E17K) activating mutations in one PTEN-PI3K-AKT pathway activation (PPAPA) category, because all of these genetic alterations activate the PI3K-AKT pathway and are known to induce mammary gland tumorigenesis 28-31. We used SIFT and PolyPhen, two commonly used algorithm tools 60-62, to predict the mutation impact on protein function. We excluded the tumors carrying only synonymous or non-significant point mutations and InDels of *TP53*, *PTEN*, *PIK3CA* and *AKT1* genes. In these datasets, *TP53* and *PTEN* are the second and the 25^th^ most frequently mutated genes with NSPMs/InDels in 34.4% and 4.1% of these breast cancers, respectively. *PIK3CA* appears the most frequently mutated gene in 37.8% of these breast cancers. The *AKT1* (E17K) activating mutation is also detected in 3.6% of these breast cancers. *PTEN* is the 6^th^ most frequently deleted gene, with homozygous copy number deletions (CNDs) in 2.8% of these breast cancers. Homozygous and heterozygous CNDs of *TP53* occur in less than 1% and as many as 48.4% of these breast cancers, respectively. Together, about 83% of these individual breast tumors contain one or more genetic aberrations in the *TP53*, *PTEN*, *PIK3CA* and/or *AKT1* genes. Analysis of these breast cancer datasets also revealed that the two *TP53* alleles are completely inactivated in a significant number of breast tumors by a heterozygous CND and an inactivating NSPM/InDel mutation. The two *PTEN* alleles are usually inactivated by NSPM/InDel mutations and/or CNDs. The PIK3CA and AKT1 functions are constitutively activated by *PIK3CA* (H1047R or E545K) and *AKT1* (E17K) activating mutations.

**Figure 4:**
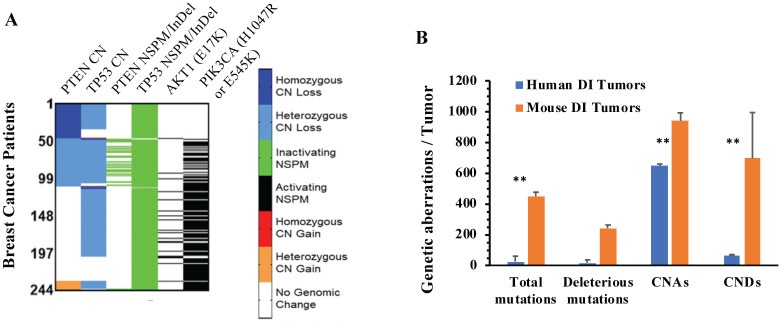
** The genomic aberrations of the *PTEN*, *TP53*, *PIK3CA* and *AKT1* genes in individual human PPAPA/TP53I breast tumors, and the average incidences of genomic aberrations in human PPAPA/TP53I breast tumors and mouse PtenI/Tp53I breast tumors. A.** A color-coded map showing the copy number (CN) loss or gain of the *PTEN* and *TP53* genes, the inactivating NSPMs/Indels for *PTEN* and *TP53*, and the activating *PIK3CA* (H1047R & E545K) and *AKT1* (E17K) mutations in individual tumors of the human PAPPA/TP53I breast tumors (n=244). **B.** Comparison of the incidences of the indicated genetic aberrations detected in PtenI/Tp53I mouse breast tumors (Mouse DI Tumors, n=8) with that in human PAPPA/TP53I breast tumors (Human DI Tumors, n=244). **, p < 0.01 by two-tailed Student's t test.

From analyzing the genomic sequence datasets of 3326 breast tumors, we identified 244 tumors that have both PPAPA and TP53 functional inactivation (TP53I). We designated this tumor group as the PPAPA/TP53I cohort. In agreement with all analyzed breast tumors, the loss of the *TP53* function in this cohort is mostly attributed to heterozygous CNDs and inactivating NSPMs/InDels. In a small number of tumors in this cohort, the *PTEN* gene is disrupted by inactivating NSPMs/InDels, while in most tumors of this cohort, the PTEN-PI3K-AKT1 pathway is activated by the *PIK3CA* (H1047R & E545K) activating mutations (Fig. 4A). The detailed genetic alterations of *TP53*, *PTEN*, *PIK3CA* (H1047R or E545K), and *AKT1* (E17K) in individual tumors of this cohort are documented in Supplementary Table S4.

We summed the events of total and deleterious NSPMs/InDels, CNAs and CNDs in all 244 specimens of the PPAPA/TP53I human breast cancer cohort, and compared the average NSPM/InDel, CNA and CND events per human specimen with the average NSPM/InDel, CNA and CND events per PtenI/Tp53I mouse breast tumor. As shown in Fig. 4B, the average NSPM/InDel, CNA and CND events per mouse tumor are significantly higher than that per human specimen.

### The Tp53 and Pten inactivation-induced mouse breast tumors recapitulate many genetic aberrations in human breast cancers

We compared the genes mutated by deleterious NSPMs/InDels in the PtenI/Tp53I mouse breast tumors with that in the PPAPA/TP53I human breast tumors. From the 360 mutated mouse genes, we found 80 (22.2%) genes that were commonly mutated in both PtenI/Tp53I mouse tumors and PPAPA/TP53I human breast tumors (Table 1, and Supplementary Table S5). Among these 80 genes, the genes with top mutation rates in human PPAPA/TP53I breast tumors include *KMT2C* (11%), *KMT2D* (6%), *NOTCH1* (2.9%), *COL22A1* (2.9%), *MUC4* (2.5%), and *PTPRM* (2.1%), most of which are known breast cancer related genes 22, 23.

Due to the sheer number of CNV-affected genes in human breast cancers, only human genes affected by CNVs that occurred in two or more patients were used to compare with mouse genes affected by CNVs in the mouse PtenI/Tp53I tumors. We found that as many as 330 (75.9%) of the 435 genes that were amplified in the PtenI/Tp53I mouse tumors were also amplified in the human PPAPA/TP53I breast cancer cohort (Table 1, and Supplementary Table S5). Importantly, we also found that many of these 330 genes exhibited very high amplification frequencies in both mouse and human tumors. For example, five genes including *TRPS1*, *CSMD3*, *EFR3A*, *PKHD1L1*, and TMEM74 that were amplified in 100% of mouse tumors were found amplified in 35.5%, 33.8%, 32.2%, 31.6% and 31.6% of human PPAPA/TP53I tumors, respectively. *Myc* was amplified in 25% of mouse tumors and 37.2% of PPAPA/TP53I breast cancer. We found that 123 (27.3%) of the 450 genes that were deleted in at least one of mouse PtenI/Tp53I tumors were deleted in at least two tumors in the human PPAPA/TP53I cohort. There were 19 of the 123 genes that were deleted in more than 5% of the human PPAPA/TP53I breast cancers, including *POLR3A* (8.6%), *SH2D4B* (7.9%), *ANXA11* (7.5%), *SFTPD* (7.5%), *TSPAN14* (7.2%), *ADK* (6.6%), and *DYDC2* (6.5%) (Table 1, and Supplementary Table S5).

Collectively, these results demonstrate that CNAs, CNDs and NSPMs/InDels are the first, second and third causes responsible for the commonly altered genes in mouse PtenI/Tp53I and human PPAPA/TP53I breast tumors. The majority of the genes altered by CNAs in mouse PtenI/Tp53I are also altered by CNAs in human PPAPA/TP53I breast tumors in very high frequencies.

### Pathways involving the genes altered commonly in both mouse PtenI/Tp53I tumors and human PPAPA/TP53I breast cancers

**Figure 5:**
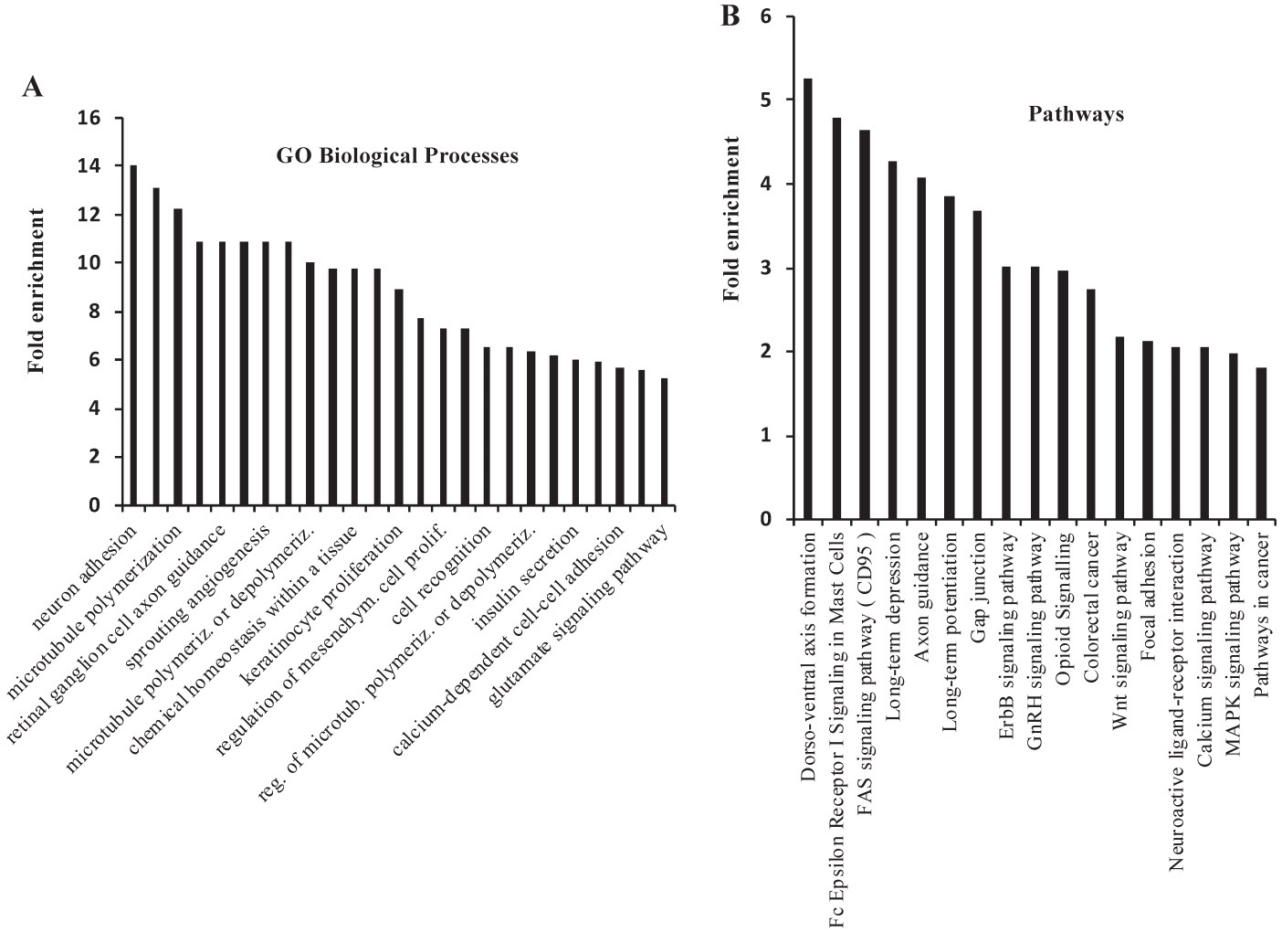
** GO Biological Process and KEGG Pathway analyses for the genes that are commonly altered in the mouse PtenI/Tp53I mammary tumors and the human PPAPA/Tp53I breast tumors.** A total of 558 genes that were altered by NSPMs/InDels, CNAs and CNDs in mouse PtenI/Tp53I mammary tumors and 1% or more human PPAPA/TP53I breast cancers were subjected to GO Biological Process and KEGG Pathway analyses. The most significant 25 GO Biological Processes (Panel A) (p < 0.05; fold enrichment > 6.3) and 17 KEGG Pathways (Panel B) (p < 0.05) are presented.

There were 620 genes that were commonly altered by NSPMs/InDels, CNAs and CNDs in the mouse PtenI/Tp53I tumors and human PPAPA/TP53I breast cancers. 558 of these 620 genes were altered in more than 1% of human PPAPA/TP53I breast cancers (Supplementary Table S5). By performing GO annotation enrichment analysis, we found that these 558 genes are enriched in many cancer-related biological processes, such as regulation of microtubule polymerization, angiogenesis, cell proliferation, cell recognition, and calcium-dependent cell-cell adhesion (Fig. 5A). We also performed KEGG pathway analysis and found that these 558 genes are enriched in multiple cancer-related pathways, such as FAS signaling pathway, GAP junction, Erbb signaling pathway, Wnt signaling pathway, focal adhesion, calcium and MAPK signaling pathways, as well as pathways in cancer (Fig. 5B). Interestingly, many of the most significant 25 GO biological processes and 17 KEGG pathways are related to neural development and/or signaling pathways. However, to date the neuronal aspect of cancer cell biology is not well understood. We identified 21 commonly altered genes including *CADM1, CDC42, CDH2, CNTN4, CNTNAP2, DAB2, EFNA5 EPHA7, ERBB4, GJA1, GNAI2, HGF, NFAT5, NFATC1, NOTCH1, PTEN, PTPRM, SLIT2, TNC, Tp53,* and *WNT2* that are known to be expressed and functionally important in both neural and breast cancer cells 74, 75. We also found 67 other commonly altered genes that are known to be involved in embryonic neural development but have not been studied for their roles in breast cancer (Supplementary Table S6) 74. These results may suggest an important role of these neuronal genes in breast cancer initiation and progression.

## Discussion

Genome instability is a hallmark of cancer that yields multiple types of genetic aberrations, including NSPMs, InDels, CNAs and CNDs. These genomic aberrations are both instrumental and unavoidable in the initiation and progression of breast cancer. P53 and PTEN are two prominent tumor suppressors known to guard the genome and they are often among the first to succumb to detrimental genomic alterations 14, 34, 35. From analysis of the 820 human breast tumor datasets with clear genomic DNA sequences, we identified 136 (16.6%) tumors with wild-type *TP53* and *PTEN* genes, 345 (42.1%) PPAPA tumors, 95 (11.6%) TP53I tumors, and 244 (29.7%) PPAPA/TP53I tumors. The *TP53* function is completely lost in 41.3% of these breast tumors that include 11.6% TP53I and 29.7% PAPPA/TP53I tumors. In most cases, the two *TP53* alleles are inactivated by a heterozygous CND and an inactivating NSPM/InDel mutation. This might be due to the essential function of the adjacent genes near the *TP53* locus. The *PTEN* function is usually inactivated by NSPM/InDel mutations and/or CNDs, while the PTEN-PI3K-AKT pathway is more frequently activated by the activating mutations of *PIK3CA* (H1047R & E545K), and *AKT1* (E17K). When counted together, 71.8% of breast cancers, which include 42.1% PPAPA and 29.7% PPAPA/TP53I tumors, contain the PTEN-PI3K-AKT1 pathway activated by genomic alterations of *Pten*, *PIK3CA* (H1047R & E545K), and *AKT1* (E17K). These analytical data suggest that complete loss of *TP53* function in combination with activation of the PTEN-PI3K-AKT cancer-driving pathway occurs in a significant percentage (29.8%) of human breast cancers.

P53 and the PTEN-PI3K-AKT1 signaling pathway in human and in mice function similarly. Specific knockout of *Pten* in the mouse MGECs causes precocious lobuloalveolar development, epithelial hyperproliferation and slow-growing mammary tumors in 50% of 290-day-old female mice 44. Conventional P53 knockout mice do not develop breast tumor because of their short lifespans caused by lymphomas and sarcomas 16, 76. However, 62% of wild type mice that orthotopically receive *Tp53* knockout mammary gland tissue transplants develop tumors in 8-12 months, demonstrating P53 loss is sufficient to induce mammary tumorigenesis after a long latency 21. Specific knockout of *Tp53* in MGECs also induces detectable tumors in 50% of mice after a long latency of 288 days 46. Previous studies also showed that conditional knockout of both *Pten* and *Tp53* in MGECs of MMTV-Cre; *Pten^F/F^*; *Tp53^F/F^* female mice and WAP-Cre; *Pten^F/F^*; *Tp53^F/F^* female mice resulted in tumor development in 9 and 11 months, respectively, which were faster than their single gene deletion counterparts, suggesting a synergistic role of double Pten and P53 deficiencies in breast tumorigenesis 77. Since P53 loss and activation of the PTEN-PI3K-AKT pathway occur in a significant proportion of breast cancers, in this study we generated MMTV-TVA^+/-^; mT/mG^+/-^; *Tp53^F/F^*; *Pten^F/F^* mice, and used RCAS-Cre virus to knock out both *Tp53* and *Pten* genes in MGECs in these mice when they were sexually matured. We demonstrated that these mice developed palpable fast-growing breast tumors after only a 21-week latency. In comparison with other conditional *Tp53* and *Pten* knockout breast cancer models, our model specifically induces breast tumors from a small proportion of the mammary luminal epithelial cells in adult mice, allowing better modeling of the breast cancer development timing and tissue environment in adult women.

Genomic alterations are highly heterogenous in individual tumors. One explanation is that many genomic alterations caused by genome instability in cancer cells are random in nature and may not associate with cancer development and progression. However, those genomic alterations driving cancer cell growth should be enriched and occur more frequently. Identification of the commonly altered genes in mouse and human breast tumors could help to find key cancer-driving genomic alterations and signaling pathways. Our results demonstrated that the mouse PtenI/Tp53I tumors recapitulate many genetic aberrations that are also detected in human breast cancers. Specifically, the PtenI/Tp53I mouse tumors have 80, 330 and 123 genes altered by NSPMs/InDels, CNAs, and CNDs respectively, that are also altered in human PPAPA/TP53I breast cancers. These represent 22%, 76% and 27% of the total NSPM/InDel-, CNA- and CND-altered genes in the PtenI/Tp53I mouse breast tumors, respectively, and 2.2%, 2.0% and 3.9% of the total NSPM/InDel-, CNA- and CND-altered genes in human PPAPA/TP53I breast cancers, respectively. Furthermore, these genes altered by NSPMs/InDels, CNAs and CNDs in both mouse PtenI/Tp53I tumors and human PPAPA/Tp53I breast cancers are involved in many cancer-related biological processes and pathways. These results suggest that many of these commonly altered genes may play important roles in promoting the growth and progression of breast cancer initiated by TP53 inactivation and PTEN-PI3K-AKT1 pathway activation. Further functional analysis of these genes may lead to identification of novel breast cancer-driving genes as molecular targets of the aggressive PPAPA/TP53I breast cancers.

The high prevalence of commonly altered genes involved in neuronal signaling and development in the mouse PtenI/Tp53I tumors and human PPAPA/TP53I breast cancers suggests an important role of these genes in breast cancer biology and a connection between the neuronal axis and cancer progression. Previous studies suggest that non-neuronal cancer cells such as liver, prostate, colon, and breast cancer cells have many more signaling networks similar to that in neural progenitor cells versus that in normal epithelial or mesenchymal cells. During the process of cancer initiation and progression, tumor cells can lose their classic epithelial signaling networks and gain certain neuronal signaling networks 74, 75. Future studies of the novel neuronal axis genes involved in breast cancer could possibly yield novel and valuable therapeutic targets for human breast cancers.

Our findings have important basic and translational research implications. Firstly, our PtenI/Tp53I mouse tumors are purely induced by somatic inactivation of both *Pten* and *Tp53* in adult mice, and these tumors can recapitulate many genetic aberrations in human PPAPA/TP53I breast cancers. This clinically relevant mouse model should be a good model for understanding molecular mechanisms responsible for Tp53 and Pten loss-induced breast cancer initiation, progression and metastasis. Secondly, the commonly altered genes in both mouse PtenI/Tp53I and human PPAPA/TP53I breast tumors include both well-studied genes known to be related to breast cancers and unstudied genes with unknown functions in breast cancer. Further characterization of these genes with unknown functions will provide new insights into understanding of the genetic networks involving breast cancer development and progression. Thirdly, our PtenI/Tp53I mouse model is a useful model to test therapeutic drugs for treating TNBCs with P53 deficiency and PTEN-PI3K-AKT1 pathway activation.

## Supplementary Material

Supplementary tables.

## Figures and Tables

**Table 1:** The number and frequency of genes mutated in both mouse PtenI/Tp53I breast tumors and human PPAPA/TP53I breast cancer cohort.

Genomic Alteration	Tumor Category	Altered Genes^ a^	Number of Common Genes (Percentage)^ b^
NSPMs/InDels	m-PtenI/Tp53I h-PPAP/TP53I	360 3643	80 (22.2%)
CNAs	m-PtenI/Tp53I h-PPAP/TP53I	435 16385	330 (75.9%)
CNDs	m-PtenI/Tp53I h-PPAP/TP53I	450 3151	123 (27.3%)

***^a^*** the gene numbers altered by NSPMs/InDels, CNAs and CNDs detected in 2 or more tumors; ***^b^*** the % is calculated by (common gene #/total altered gene # in the mouse PtenI/Tp53I mouse tumors) x 100.
