# Supplementary material for: Common Genomic Aberrations in Mouse and Human Breast Cancers with Concurrent P53 Deficiency and Activated PTEN-PI3K-AKT Pathway
Source: Int J Biol Sci. 2022 Jan 1;18(1):229–41. doi: 10.7150/ijbs.65763 (PMC8692138; doi:10.7150/ijbs.65763)
Supplement: Supplementary file 1 — Supplementary tables. [file ijbsv18p0229s1.pdf]

Supplementary Materials for:

Common Genomic Aberrations in Mouse and Human Breast Cancers with Double PTEN and TP53 Deficiencies

Jarrod D. Martinez, Qianxing Mo, Yixiang Xu, Li Qin, Yi Li, and Jianming Xu

Contents of the Supplemental Data Tables

Supplementary Table S1. Profiles of NSPMs/InDels and Mutated Genes in the Mouse PtenI/Tp53I Mammary Gland Tumors (Pages 1-123)

Supplementary Table S2. The Profiles of CNAs and CNA-affected Genes in the Mouse PtenI/Tp53I Mammary Tumors (Pages 1-154)

Supplementary Table S3. The Profiles of CNDs and CND-affected Genes in the Mouse PtenI/Tp53I Mammary Tumors (Pages 1-121)

Supplementary Table S4. Selection of PPAPA/TP53I Patient Cohort (Pages 1-7)

Supplementary Table S5. The Genes that Are Altered in both Mouse PtenI/Tp53I Mammary Tumors (n = 8) and Human PAPPA/TP53I Breast Cancer Cohort (n = 244) (Pages 1-9)

Supplementary Table S6. Commonly altered genes between mouse PtenI/Tp53I tumors and human PPAPA/TP53I breast cancers that have a neural connection (Page 1)

(Of note: Each Supplementary Table has its specific Header on all of its pages)

Supplementary Table S1. Profiles of NSPMs/InDels and Mutated Genes in the Mouse PtenI/Tp53I Mammary Gland Tumors

There are 1934 deleterious mutations in 361 Individual Genes

Samples	Chr	**Peak Score**	**Freq (%)**	Start	End	Ref	Alt	Func.refGene	**Gene.refGene**	ENSMUSP ID	GeneDetail.refGene	ExonicFunc.refGene	AAChange.refGene	**AA Change**	Provean Format (Protein)	Provean Format (Genomic)	cytoBand	snp137	Otherinfo	Unknw1	Unknw2	CHROM	POS	ID	REF	ALT	QUAL	FILTER	INFO	FORMAT	TUMOR	NORMAL	% Altered	SIFT/PolyPHEN Deleterious/Tolerated	SCORE	PREDICTION (cutoff=-2.5)	#SEQ	#CLUSTER	SCORE	PREDICTION (cutoff=0.05)	MEDIAN_INFO	#SEQ
T12,T1,T22,T2,T32,T3,T42,T4	chr6	**8**	**100**	121636338	121636338	C	T	exonic	**A2m**	ENSMUSP00000032203	.	nonsynonymous SNV	A2m:NM_175628:exon1:c.C50T:p.P17L	**P17L**	ENSMUSP00000032203,17,P,L	6,121636338,C,A	6qF1	rs52342553	0.25	.	45	chr6	121636338	rs52342553	C	A	.	clustered_events;homologous_mapping_event	DB;ECNT=7;HCNT=8;MAX_ED=177;MIN_ED=71;NLOD=14.45;TLOD=112.63	GT:AD:AF:ALT_F1R2:ALT_F2R1:FOXOG:PGT:PID:QSS:REF_F1R2:REF_F2R1	0/1:18,29:0.609:20:9:0.690:0|1:121639360_T_C:497,841:11:7	0/0:54,0:0.00:0:0:.:0|1:121639360_T_C:1517,0:24:30	2.2	Damaging	-0.27	Neutral	124	30	0.048	Damaging	3.5	18
T12,T1,T22,T2,T32,T3,T42,T4	chr6	**8**	**100**	121661523	121661523	C	A	exonic	**A2m**	ENSMUSP00000032203	.	nonsynonymous SNV	A2m:NM_175628:exon21:c.C2693A:p.T898K	**T898K**	ENSMUSP00000032203,898,T,K	6,121661523,C,C	6qF1	rs30949689	0.25	.	44	chr6	121661523	rs30949689	C	C	.	PASS	ECNT=1;HCNT=1;MAX_ED=.;MIN_ED=.;NLOD=9.58;TLOD=8.42	GT:AD:AF:ALT_F1R2:ALT_F2R1:FOXOG:QSS:REF_F1R2:REF_F2R1	0/1:33,5:0.114:4:1:0.800:927,149:17:16	0/0:39,0:0.00:0:0:.:1097,0:16:23	2.1	Deleterious	-5.88	Deleterious	124	30	0.001	Damaging	2.81	129
T12,T1,T22,T2,T32,T3,T42,T4	chr6	**8**	**100**	121677995	121677995	T	C	exonic	**A2m**	ENSMUSP00000032203	.	nonsynonymous SNV	A2m:NM_175628:exon34:c.T4277C:p.M1426T	**M1426T**	ENSMUSP00000032203,1426,M,T	6,121677995,T,A	6qF1	rs30709786	0.25	.	41	chr6	121677995	rs30709786	T	A	.	t_lod_fstar	ECNT=1;HCNT=2;MAX_ED=.;MIN_ED=.;NLOD=12.59;TLOD=4.63	GT:AD:AF:ALT_F1R2:ALT_F2R1:FOXOG:QSS:REF_F1R2:REF_F2R1	0/1:54,3:0.037:0:3:.:1510,89:26:28	0/0:47,0:0.00:0:0:.:1313,0:20:27	23	Tolerated	4.59	Neutral	124	30	1	Tolerated	2.8	129
T12,T1,T22,T2,T32,T3,T42,T4	chr6	**8**	**100**	121639361	121639361	G	A	exonic	**A2m**	ENSMUSP00000032203	.	nonsynonymous SNV	A2m:NM_175628:exon3:c.G447A:p.M149I	**M149I**	ENSMUSP00000032203,149,M,I	6,121639361,G,A	6qF1	rs247075986	0.25	.	54	chr6	121639361	rs247075986	G	A	.	clustered_events;homologous_mapping_event	DB;ECNT=3;HCNT=2;MAX_ED=92;MIN_ED=75;NLOD=11.14;TLOD=60.40	GT:AD:AF:ALT_F1R2:ALT_F2R1:FOXOG:PGT:PID:QSS:REF_F1R2:REF_F2R1	0/1:21,17:0.457:4:13:0.235:0|1:121657999_G_A:582,475:15:6	0/0:38,0:0.00:0:0:.:0|1:121657999_G_A:1076,0:17:21	2.2	Tolerated	-0.6	Neutral	124	30	0.089	Tolerated	2.81	122
T12,T1,T22,T2,T32,T3,T42,T4	chr6	**8**	**100**	121639360	121639360	T	C	exonic	**A2m**	ENSMUSP00000032203	.	nonsynonymous SNV	A2m:NM_175628:exon3:c.T446C:p.M149T	**M149T**	ENSMUSP00000032203,149,M,T	6,121639360,T,C	6qF1	rs46877575	0.25	.	54	chr6	121639360	rs46877575	T	C	.	clustered_events;homologous_mapping_event	DB;ECNT=5;HCNT=16;MAX_ED=183;MIN_ED=2;NLOD=9.93;TLOD=47.13	GT:AD:AF:ALT_F1R2:ALT_F2R1:FOXOG:QSS:REF_F1R2:REF_F2R1	0/1:24,23:0.452:12:11:0.522:680,608:17:7	0/0:36,0:0.00:0:0:.:1009,0:22:14	2.2	Tolerated	2.5	Neutral	124	30	1	Tolerated	2.81	122
T12,T1,T22,T2,T32,T3,T42,T4	chr6	**8**	**100**	121654513	121654513	T	C	exonic	**A2m**	ENSMUSP00000032203	.	nonsynonymous SNV	A2m:NM_175628:exon15:c.T1768C:p.F590L	**F590L**	ENSMUSP00000032203,590,F,L	6,121654513,T,T	6qF1	rs45860830	0.25	.	36	chr6	121654513	rs45860830	T	T	.	PASS	DB;ECNT=1;HCNT=1;MAX_ED=.;MIN_ED=.;NLOD=11.43;TLOD=68.96	GT:AD:AF:ALT_F1R2:ALT_F2R1:FOXOG:QSS:REF_F1R2:REF_F2R1	0/1:20,30:0.587:14:16:0.533:563,798:10:10	0/0:49,0:0.00:0:0:.:1367,0:26:23	2.2	Tolerated	-1.45	Neutral	124	30	0.118	Tolerated	2.81	130
T12,T1,T22,T2,T32,T3,T42,T4	chr6	**8**	**100**	121657999	121657999	G	A	exonic	**A2m**	ENSMUSP00000032203	.	nonsynonymous SNV	A2m:NM_175628:exon17:c.G2104A:p.V702I	**V702I**	ENSMUSP00000032203,702,V,I	6,121657999,G,A	6qF1	rs48872830	0.25	.	38	chr6	121657999	rs48872830	G	A	.	clustered_events;homologous_mapping_event	DB;ECNT=3;HCNT=2;MAX_ED=87;MIN_ED=67;NLOD=12.28;TLOD=67.31	GT:AD:AF:ALT_F1R2:ALT_F2R1:FOXOG:QSS:REF_F1R2:REF_F2R1	0/1:9,27:0.719:16:11:0.407:227,761:4:5	0/0:44,0:0.00:0:0:.:1103,0:25:19	2.2	Tolerated	-0.28	Neutral	124	30	0.397	Tolerated	2.81	121
T12,T1,T22,T2,T32,T3,T42,T4	chr6	**8**	**100**	121660099	121660099	C	T	exonic	**A2m**	ENSMUSP00000032203	.	nonsynonymous SNV	A2m:NM_175628:exon19:c.C2420T:p.A807V	**A807V**	ENSMUSP00000032203,807,A,V	6,121660099,C,C	6qF1	rs30127457	0.25	.	49	chr6	121660099	rs30127457	C	C	.	clustered_events	DB;ECNT=2;HCNT=2;MAX_ED=47;MIN_ED=47;NLOD=10.83;TLOD=38.98	GT:AD:AF:ALT_F1R2:ALT_F2R1:FOXOG:QSS:REF_F1R2:REF_F2R1	0/1:20,17:0.472:8:9:0.471:572,418:9:11	0/0:41,0:0.00:0:0:.:1177,0:13:28	2.2	Tolerated	-2.06	Neutral	124	30	0.2	Tolerated	2.81	130
T12,T1,T22,T2,T32,T3,T42,T4	chr6	**8**	**100**	121636301	121636301	T	A	exonic	**A2m**	ENSMUSP00000032203	.	nonsynonymous SNV	A2m:NM_175628:exon1:c.T13A:p.W5R	**W5R**	ENSMUSP00000032203,5,W,R	6,121636301,T,C	6qF1	rs52414787	0.25	.	49	chr6	121636301	rs52414787	T	C	.	clustered_events;homologous_mapping_event	DB;ECNT=7;HCNT=8;MAX_ED=177;MIN_ED=71;NLOD=14.15;TLOD=117.34	GT:AD:AF:ALT_F1R2:ALT_F2R1:FOXOG:PGT:PID:QSS:REF_F1R2:REF_F2R1	0/1:19,28:0.630:20:8:0.714:0|1:121639360_T_C:529,787:12:7	0/0:54,0:0.00:0:0:.:0|1:121639360_T_C:1524,0:24:30	2.2	Unknown	0.53	Neutral	124	30	NA	NA	NA	NA

T12,T1,T22,T2,T32,T3,T42,T4	chr6	**8**	**100**	128544930	128544930	T	C	exonic	**A2ml1**	ENSMUSP00000115324	.	nonsynonymous SNV	A2ml1:NM_001001179:exon30:c.A3928G:p.I1310V	**I1310V**	ENSMUSP00000115324,1310,I,V	6,128544930,T,A	6qF3	rs31800809	0.25	.	46	chr6	128544930	rs31800809	T	A	.	PASS	DB;ECNT=1;HCNT=1;MAX_ED=.;MIN_ED=.;NLOD=10.21;TLOD=61.70	GT:AD:AF:ALT_F1R2:ALT_F2R1:FOXOG:QSS:REF_F1R2:REF_F2R1	0/1:18,26:0.579:12:14:0.462:475,741:7:11	0/0:38,0:0.00:0:0:.:970,0:16:22	2.2	Damaging	-2.46	Neutral	135	30	0.007	Damaging	2.79	100
T12,T1,T22,T2,T32,T3,T42,T4	chr6	**8**	**100**	128545065	128545065	T	C	exonic	**A2ml1**	ENSMUSP00000115324	.	nonsynonymous SNV	A2ml1:NM_001001179:exon30:c.A3793G:p.I1265V	**I1265V**	ENSMUSP00000115324,1265,I,V	6,128545065,T,G	6qF3	rs31801845	0.25	.	39	chr6	128545065	rs31801845	T	G	.	clustered_events;homologous_mapping_event	DB;ECNT=4;HCNT=2;MAX_ED=69;MIN_ED=1;NLOD=11.74;TLOD=59.24	GT:AD:AF:ALT_F1R2:ALT_F2R1:FOXOG:PGT:PID:QSS:REF_F1R2:REF_F2R1	0/1:24,17:0.421:11:6:0.353:0|1:128570827_A_G:665,487:13:11	0/0:44,0:0.00:0:0:.:0|1:128570827_A_G:1214,0:27:17	2.2	Damaging	-1.42	Neutral	135	30	0.026	Damaging	2.79	99
T12,T1,T22,T2,T32,T3,T42,T4	chr6	**8**	**100**	128541019	128541019	A	G	exonic	**A2ml1**	ENSMUSP00000115324	.	nonsynonymous SNV	A2ml1:NM_001001179:exon34:c.T4303C:p.Y1435H	**Y1435H**	ENSMUSP00000115324,1435,Y,H	6,128541019,A,T	6qF3	rs31801889	0.25	.	42	chr6	128541019	rs31801889	A	T	.	clustered_events;homologous_mapping_event	DB;ECNT=3;HCNT=4;MAX_ED=81;MIN_ED=15;NLOD=11.13;TLOD=55.30	GT:AD:AF:ALT_F1R2:ALT_F2R1:FOXOG:QSS:REF_F1R2:REF_F2R1	0/1:21,22:0.513:12:10:0.455:590,635:11:10	0/0:41,0:0.00:0:0:.:1157,0:21:20	2.2	Deleterious	-6.16	Deleterious	135	30	0.003	Damaging	2.8	90
T12,T1,T22,T2,T32,T3,T42,T4	chr6	**8**	**100**	128570827	128570827	A	G	exonic	**A2ml1**	ENSMUSP00000115324	.	nonsynonymous SNV	A2ml1:NM_001001179:exon9:c.T947C:p.V316A	**V316A**	ENSMUSP00000115324,316,V,A	6,128570827,A,T	6qF3	rs31807364	0.25	.	44	chr6	128570827	rs31807364	A	T	.	clustered_events;homologous_mapping_event	DB;ECNT=8;HCNT=8;MAX_ED=204;MIN_ED=44;NLOD=9.93;TLOD=24.20	GT:AD:AF:ALT_F1R2:ALT_F2R1:FOXOG:PGT:PID:QSS:REF_F1R2:REF_F2R1	0/1:26,7:0.219:3:4:0.429:0|1:128572014_T_C:679,200:9:17	0/0:35,0:0.00:0:0:.:0|1:128572014_T_C:817,0:21:14	2.2	Deleterious	-5.67	Deleterious	135	30	0	Damaging	2.79	97
T12,T1,T22,T2,T32,T3,T42,T4	chr6	**8**	**100**	128575567	128575567	C	T	exonic	**A2ml1**	ENSMUSP00000115324	.	nonsynonymous SNV	A2ml1:NM_001001179:exon7:c.G707A:p.S236N	**S236N**	ENSMUSP00000115324,236,S,N	6,128575567,C,A	6qF3	rs31800940	0.25	.	34	chr6	128575567	rs31800940	C	A	.	clustered_events;homologous_mapping_event	DB;ECNT=5;HCNT=4;MAX_ED=102;MIN_ED=4;NLOD=12.74;TLOD=65.74	GT:AD:AF:ALT_F1R2:ALT_F2R1:FOXOG:QSS:REF_F1R2:REF_F2R1	0/1:26,26:0.511:11:15:0.423:624,741:12:14	0/0:48,0:0.00:0:0:.:1106,0:25:23	2.2	Deleterious	-3.51	Deleterious	135	30	0.005	Damaging	2.79	101
T12,T1,T22,T2,T32,T3,T42,T4	chr6	**8**	**100**	128572018	128572018	G	T	exonic	**A2ml1**	ENSMUSP00000115324	.	nonsynonymous SNV	A2ml1:NM_001001179:exon8:c.C800A:p.P267Q	**P267Q**	ENSMUSP00000115324,267,P,Q	6,128572018,G,A	6qF3	rs31802583	0.25	.	35	chr6	128572018	.	G	A	.	clustered_events;homologous_mapping_event	DB;ECNT=5;HCNT=6;MAX_ED=102;MIN_ED=4;NLOD=11.74;TLOD=102.75	GT:AD:AF:ALT_F1R2:ALT_F2R1:FOXOG:PGT:PID:QSS:REF_F1R2:REF_F2R1	0/1:19,29:0.591:13:16:0.448:0|1:128576175_T_C:468,825:9:10	0/0:42,0:0.00:0:0:.:0|1:128576175_T_C:1031,0:24:18	2.2	Deleterious	-2.77	Deleterious	135	30	0.577	Tolerated	2.79	100
T12,T1,T22,T2,T32,T3,T42,T4	chr6	**8**	**100**	128540349	128540349	C	T	exonic	**A2ml1**	ENSMUSP00000115324	.	nonsynonymous SNV	A2ml1:NM_001001179:exon35:c.G4319A:p.G1440D	**G1440D**	ENSMUSP00000115324,1440,G,D	6,128540349,C,T	6qF3	rs31800766	0.25	.	39	chr6	128540349	rs31800766	C	T	.	clustered_events;homologous_mapping_event	DB;ECNT=9;HCNT=2;MAX_ED=119;MIN_ED=6;NLOD=11.14;TLOD=82.16	GT:AD:AF:ALT_F1R2:ALT_F2R1:FOXOG:PGT:PID:QSS:REF_F1R2:REF_F2R1	0/1:24,24:0.512:11:13:0.542:0|1:128540973_C_T:693,673:13:11	0/0:47,0:0.00:0:0:.:0|1:128540973_C_T:1348,0:27:20	2.2	Tolerated	4.01	Neutral	135	30	1	Tolerated	2.79	76
T12,T1,T22,T2,T32,T3,T42,T4	chr6	**8**	**100**	128541006	128541006	T	C	exonic	**A2ml1**	ENSMUSP00000115324	.	nonsynonymous SNV	A2ml1:NM_001001179:exon34:c.A4316G:p.K1439R	**K1439R**	ENSMUSP00000115324,1439,K,R	6,128541006,T,G	6qF3	rs49741801	0.25	.	47	chr6	128541006	rs49741801	T	G	.	clustered_events;homologous_mapping_event	DB;ECNT=9;HCNT=2;MAX_ED=119;MIN_ED=6;NLOD=10.54;TLOD=77.66	GT:AD:AF:ALT_F1R2:ALT_F2R1:FOXOG:PGT:PID:QSS:REF_F1R2:REF_F2R1	0/1:23,23:0.488:10:13:0.565:0|1:128540973_C_T:656,639:12:11	0/0:42,0:0.00:0:0:.:0|1:128540973_C_T:1169,0:24:18	2.2	Tolerated	1.98	Neutral	135	30	1	Tolerated	2.79	76
T12,T1,T22,T2,T32,T3,T42,T4	chr6	**8**	**100**	128541010	128541010	C	T	exonic	**A2ml1**	ENSMUSP00000115324	.	nonsynonymous SNV	A2ml1:NM_001001179:exon34:c.G4312A:p.E1438K	**E1438K**	ENSMUSP00000115324,1438,E,K	6,128541010,C,C	6qF3	rs47299044	0.25	.	47	chr6	128541010	rs47299044	C	C	.	PASS	DB;ECNT=2;HCNT=1;MAX_ED=2;MIN_ED=2;NLOD=10.54;TLOD=93.70	GT:AD:AF:ALT_F1R2:ALT_F2R1:FOXOG:PGT:PID:QSS:REF_F1R2:REF_F2R1	0/1:27,23:0.481:13:10:0.435:0|1:128543315_A_C:754,629:13:14	0/0:40,0:0.00:0:0:.:0|1:128543315_A_C:1109,0:18:22	2.2	Tolerated	0	Neutral	135	30	1	Tolerated	2.79	76
T12,T1,T22,T2,T32,T3,T42,T4	chr6	**8**	**100**	128543315	128543315	A	C	exonic	**A2ml1**	ENSMUSP00000115324	.	nonsynonymous SNV	A2ml1:NM_001001179:exon32:c.T4085G:p.V1362G	**V1362G**	ENSMUSP00000115324,1362,V,G	6,128543315,A,C	6qF3	rs47311957	0.25	.	40	chr6	128543315	rs47311957	A	C	.	clustered_events;homologous_mapping_event	DB;ECNT=6;HCNT=18;MAX_ED=166;MIN_ED=45;NLOD=12.29;TLOD=47.31	GT:AD:AF:ALT_F1R2:ALT_F2R1:FOXOG:QSS:REF_F1R2:REF_F2R1	0/1:23,20:0.425:7:13:0.350:663,558:12:11	0/0:46,0:0.00:0:0:.:1287,0:24:22	2.2	Tolerated	2.86	Neutral	135	30	0.062	Tolerated	2.79	99

T12,T1,T22,T2,T32,T3,T42,T4	chr6	**8**	**100**	128543975	128543975	A	T	exonic	**A2ml1**	ENSMUSP00000115324	.	nonsynonymous SNV	A2ml1:NM_001001179:exon31:c.T4015A:p.S1339T	**S1339T**	ENSMUSP00000115324,1339,S,T	6,128543975,A,C	6qF3	rs31798958	0.25	.	41	chr6	128543975	rs31798958	A	C	.	clustered_events	DB;ECNT=2;HCNT=2;MAX_ED=38;MIN_ED=38;NLOD=11.13;TLOD=39.15	GT:AD:AF:ALT_F1R2:ALT_F2R1:FOXOG:QSS:REF_F1R2:REF_F2R1	0/1:31,20:0.396:10:10:0.500:874,492:17:14	0/0:39,0:0.00:0:0:.:1113,0:18:21	2.2	Tolerated	-0.6	Neutral	135	30	0.614	Tolerated	2.79	94
T12,T1,T22,T2,T32,T3,T42,T4	chr6	**8**	**100**	128547276	128547276	G	A	exonic	**A2ml1**	ENSMUSP00000115324	.	nonsynonymous SNV	A2ml1:NM_001001179:exon28:c.C3392T:p.S1131L	**S1131L**	ENSMUSP00000115324,1131,S,L	6,128547276,G,G	6qF3	rs30122649	0.25	.	38	chr6	128547276	rs30122649	G	G	.	clustered_events;homologous_mapping_event	DB;ECNT=4;HCNT=2;MAX_ED=69;MIN_ED=1;NLOD=11.14;TLOD=56.65	GT:AD:AF:ALT_F1R2:ALT_F2R1:FOXOG:PGT:PID:QSS:REF_F1R2:REF_F2R1	0/1:22,16:0.405:10:6:0.625:0|1:128570827_A_G:621,456:12:10	0/0:43,0:0.00:0:0:.:0|1:128570827_A_G:1175,0:26:17	2.2	Tolerated	0	Neutral	135	30	0.753	Tolerated	2.79	99
T12,T1,T22,T2,T32,T3,T42,T4	chr6	**8**	**100**	128570833	128570833	T	G	exonic	**A2ml1**	ENSMUSP00000115324	.	nonsynonymous SNV	A2ml1:NM_001001179:exon9:c.A941C:p.E314A	**E314A**	ENSMUSP00000115324,314,E,A	6,128570833,T,T	6qF3	rs31807366	0.25	.	43	chr6	128570833	rs31807366	T	T	.	clustered_events;homologous_mapping_event	DB;ECNT=10;HCNT=16;MAX_ED=215;MIN_ED=34;NLOD=8.12;TLOD=14.26	GT:AD:AF:ALT_F1R2:ALT_F2R1:FOXOG:QSS:REF_F1R2:REF_F2R1	0/1:15,6:0.300:4:2:0.333:417,167:8:7	0/0:34,0:0.00:0:0:.:959,0:11:23	2.2	Tolerated	0	Neutral	135	30	0.065	Tolerated	2.79	97
T12,T1,T22,T2,T32,T3,T42,T4	chr6	**8**	**100**	128576179	128576179	G	A	exonic	**A2ml1**	ENSMUSP00000115324	.	nonsynonymous SNV	A2ml1:NM_001001179:exon6:c.C614T:p.T205M	**T205M**	ENSMUSP00000115324,205,T,M	6,128576179,G,A	6qF3	rs223840017	0.25	.	42	chr6	128576179	rs223840017	G	A	.	clustered_events;homologous_mapping_event	DB;ECNT=3;HCNT=2;MAX_ED=100;MIN_ED=26;NLOD=8.98;TLOD=97.81	GT:AD:AF:ALT_F1R2:ALT_F2R1:FOXOG:PGT:PID:QSS:REF_F1R2:REF_F2R1	0/1:12,27:0.676:15:12:0.556:0|1:128580299_GTTT_G:329,777:5:7	0/0:35,0:0.00:0:0:.:0|1:128580299_GTTT_G:939,0:15:20	2.2	Tolerated	0.16	Neutral	135	30	0.171	Tolerated	2.8	100
T12,T1,T22,T2,T32,T3,T42,T4	chr6	**8**	**100**	128576230	128576230	G	A	exonic	**A2ml1**	ENSMUSP00000115324	.	nonsynonymous SNV	A2ml1:NM_001001179:exon6:c.C563T:p.P188L	**P188L**	ENSMUSP00000115324,188,P,L	6,128576230,G,G	6qF3	rs30059409	0.25	.	48	chr6	128576230	rs30059409	G	G	.	clustered_events;homologous_mapping_event;t_lod_fstar	DB;ECNT=7;HCNT=16;MAX_ED=204;MIN_ED=44;NLOD=10.78;TLOD=5.93	GT:AD:AF:ALT_F1R2:ALT_F2R1:FOXOG:QSS:REF_F1R2:REF_F2R1	0/1:21,3:0.136:1:2:0.667:588,83:10:11	0/0:39,0:0.00:0:0:.:1088,0:19:20	2.2	Tolerated	0	Neutral	135	30	0.193	Tolerated	2.8	100
T12,T1,T22,T2,T32,T3,T42,T4	chr6	**8**	**100**	128580325	128580325	G	A	exonic	**A2ml1**	ENSMUSP00000115324	.	nonsynonymous SNV	A2ml1:NM_001001179:exon3:c.C404T:p.T135I	**T135I**	ENSMUSP00000115324,135,T,I	6,128580325,G,G	6qF3	rs31808790	0.25	.	35	chr6	128580325	rs31808790	G	G	.	str_contraction;t_lod_fstar	ECNT=1;HCNT=1;MAX_ED=.;MIN_ED=.;NLOD=14.92;RPA=6,5;RU=GCA;STR;TLOD=4.27	GT:AD:AF:ALT_F1R2:ALT_F2R1:FOXOG:QSS:REF_F1R2:REF_F2R1	0/1:40,3:0.050:0:3:.:968,78:20:20	0/0:61,1:0.019:1:0:.:1533,6:30:31	1	Tolerated	0	Neutral	135	30	1	Tolerated	2.79	99
T12,T1,T22,T2,T32,T3,T42,T4	chr6	**8**	**100**	125053619	125053619	G	A	exonic	**Acrbp**	ENSMUSP00000112994	.	nonsynonymous SNV	Acrbp:NM_001127340:exon5:c.G602A:p.R201H,Acrbp:NM_016845:exon5:c.G602A:p.R201H	**R201H**	ENSMUSP00000112994,201,R,H	6,125053619,G,A	6qF2	rs30967950	0.25	.	36	chr6	125053619	rs30967950	G	A	.	alt_allele_in_normal;t_lod_fstar	ECNT=1;HCNT=1;MAX_ED=.;MIN_ED=.;NLOD=11.75;TLOD=4.29	GT:AD:AF:ALT_F1R2:ALT_F2R1:FOXOG:QSS:REF_F1R2:REF_F2R1	0/1:80,5:0.071:2:3:.:2061,141:48:32	0/0:42,2:0.048:2:0:.:1015,55:29:13	1.5	Tolerated	0.73	Neutral	50	10	0.726	Tolerated	3.57	35
T12,T1,T22,T2,T32,T3,T42,T4	chr6	**8**	**100**	118506575	118506575	C	G	exonic	**Ankrd26**	ENSMUSP00000097809	.	nonsynonymous SNV	Ankrd26:NM_001081112:exon32:c.G4667C:p.S1556T	**S1556T**	ENSMUSP00000097809,1556,S,T	6,118506575,C,A	6qF1	rs263215838	0.25	.	33	chr6	118506575	rs263215838	C	A	.	PASS	DB;ECNT=1;HCNT=1;MAX_ED=.;MIN_ED=.;NLOD=9.62;TLOD=68.84	GT:AD:AF:ALT_F1R2:ALT_F2R1:FOXOG:QSS:REF_F1R2:REF_F2R1	0/1:23,26:0.543:14:12:0.538:593,736:10:13	0/0:37,0:0.00:0:0:.:946,0:17:20	2	Damaging	-2.23	Neutral	69	30	0.002	Damaging	2.9	73
T12,T1,T22,T2,T32,T3,T42,T4	chr6	**8**	**100**	118508560	118508560	G	A	exonic	**Ankrd26**	ENSMUSP00000097809	.	nonsynonymous SNV	Ankrd26:NM_001081112:exon30:c.C4352T:p.A1451V	**A1451V**	ENSMUSP00000097809,1451,A,V	6,118508560,G,T	6qF1	rs31571560	0.25	.	37	chr6	118508560	rs31571560	G	T	.	PASS	DB;ECNT=1;HCNT=1;MAX_ED=.;MIN_ED=.;NLOD=10.53;TLOD=30.92	GT:AD:AF:ALT_F1R2:ALT_F2R1:FOXOG:QSS:REF_F1R2:REF_F2R1	0/1:17,12:0.414:7:5:0.417:485,338:5:12	0/0:40,0:0.00:0:0:.:1132,0:18:22	2	Damaging	1.32	Neutral	69	30	0.028	Damaging	2.89	74
T12,T1,T22,T2,T32,T3,T42,T4	chr6	**8**	**100**	118532301	118532301	G	A	exonic	**Ankrd26**	ENSMUSP00000097809	.	nonsynonymous SNV	Ankrd26:NM_001081112:exon18:c.C1802T:p.A601V	**A601V**	ENSMUSP00000097809,601,A,V	6,118532301,G,T	6qF1	rs229836234	0.25	.	35	chr6	118532301	rs229836234	G	T	.	clustered_events;homologous_mapping_event	DB;ECNT=4;HCNT=8;MAX_ED=208;MIN_ED=78;NLOD=9.28;TLOD=23.91	GT:AD:AF:ALT_F1R2:ALT_F2R1:FOXOG:QSS:REF_F1R2:REF_F2R1	0/1:12,10:0.450:4:6:0.600:350,284:7:5	0/0:38,0:0.00:0:0:.:1077,0:19:19	2	Damaging	-1.27	Neutral	69	30	0.033	Damaging	3.32	45

T12,T1,T22,T2,T32,T3,T42,T4	chr6	**8**	**100**	118525013	118525013	C	T	exonic	**Ankrd26**	ENSMUSP00000097809	.	nonsynonymous SNV	Ankrd26:NM_001081112:exon23:c.G2545A:p.G849R	**G849R**	ENSMUSP00000097809,849,G,R	6,118525013,C,T	6qF1	rs31572531	0.25	.	40	chr6	118525013	rs31572531	C	T	.	clustered_events;homologous_mapping_event	DB;ECNT=9;HCNT=16;MAX_ED=203;MIN_ED=17;NLOD=10.54;TLOD=41.35	GT:AD:AF:ALT_F1R2:ALT_F2R1:FOXOG:PGT:PID:QSS:REF_F1R2:REF_F2R1	0/1:18,12:0.393:4:8:0.667:0|1:118532276_G_A:523,340:10:8	0/0:39,0:0.00:0:0:.:0|1:118532276_G_A:1078,0:21:18	2	Deleterious	-3.94	Deleterious	69	30	0.203	Tolerated	2.97	59
T12,T1,T22,T2,T32,T3,T42,T4	chr6	**8**	**100**	118559290	118559290	C	T	exonic	**Ankrd26**	ENSMUSP00000097809	.	nonsynonymous SNV	Ankrd26:NM_001081112:exon2:c.G341A:p.S114N	**S114N**	ENSMUSP00000097809,114,S,N	NA	6qF1	.	0.25	.	38	chr6	118559290	.	C	T	.	alt_allele_in_normal;clustered_events	ECNT=7;HCNT=3;MAX_ED=31;MIN_ED=1;NLOD=4.83;TLOD=8.98	GT:AD:AF:ALT_F1R2:ALT_F2R1:FOXOG:PGT:PID:QSS:REF_F1R2:REF_F2R1	0/1:35,3:0.081:3:0:1.00:0|1:110764148_A_ATTTTTAAT:975,73:14:21	0/0:31,1:0.032:1:0:1.00:0|1:110764148_A_ATTTTTAAT:865,28:16:15	1.4	Tolerated	0.23	Neutral	114	30	0.07	Tolerated	4.32	9
T12,T1,T22,T2,T32,T3,T42,T4	chr6	**8**	**100**	118503074	118503074	G	T	exonic	**Ankrd26**	ENSMUSP00000097809	.	nonsynonymous SNV	Ankrd26:NM_001081112:exon34:c.C4995A:p.D1665E	**D1665E**	ENSMUSP00000097809,1665,D,E	6,118503074,G,G	6qF1	rs31574016	0.25	.	53	chr6	118503074	rs31574016	G	G	.	clustered_events;homologous_mapping_event	DB;ECNT=5;HCNT=8;MAX_ED=213;MIN_ED=4;NLOD=10.22;TLOD=55.14	GT:AD:AF:ALT_F1R2:ALT_F2R1:FOXOG:QSS:REF_F1R2:REF_F2R1	0/1:31,23:0.420:8:15:0.652:854,641:23:8	0/0:34,0:0.00:0:0:.:919,0:18:16	2	Tolerated	0	Neutral	69	30	0.181	Tolerated	3.1	43
T12,T1,T22,T2,T32,T3,T42,T4	chr6	**8**	**100**	118503097	118503097	C	G	exonic	**Ankrd26**	ENSMUSP00000097809	.	nonsynonymous SNV	Ankrd26:NM_001081112:exon34:c.G4972C:p.A1658P	**A1658P**	ENSMUSP00000097809,1658,A,P	6,118503097,C,G	6qF1	rs31574018	0.25	.	52	chr6	118503097	rs31574018	C	G	.	clustered_events	DB;ECNT=2;HCNT=8;MAX_ED=33;MIN_ED=33;NLOD=9.63;TLOD=64.99	GT:AD:AF:ALT_F1R2:ALT_F2R1:FOXOG:QSS:REF_F1R2:REF_F2R1	0/1:28,26:0.472:11:15:0.577:824,719:16:12	0/0:33,0:0.00:0:0:.:945,0:18:15	2	Tolerated	-1.08	Neutral	69	30	0.26	Tolerated	3.16	43
T12,T1,T22,T2,T32,T3,T42,T4	chr6	**8**	**100**	118506486	118506486	A	G	exonic	**Ankrd26**	ENSMUSP00000097809	.	nonsynonymous SNV	Ankrd26:NM_001081112:exon32:c.T4756C:p.F1586L	**F1586L**	ENSMUSP00000097809,1586,F,L	6,118506486,A,C	6qF1	.	0.25	.	34	chr6	118506486	.	A	C	.	PASS	DB;ECNT=1;HCNT=1;MAX_ED=.;MIN_ED=.;NLOD=11.43;TLOD=25.46	GT:AD:AF:ALT_F1R2:ALT_F2R1:FOXOG:QSS:REF_F1R2:REF_F2R1	0/1:18,10:0.400:9:1:0.900:509,280:5:13	0/0:43,0:0.00:0:0:.:1224,0:16:27	2	Tolerated	-0.49	Neutral	69	30	0.081	Tolerated	2.9	71
T12,T1,T22,T2,T32,T3,T42,T4	chr6	**8**	**100**	118507746	118507746	T	C	exonic	**Ankrd26**	ENSMUSP00000097809	.	nonsynonymous SNV	Ankrd26:NM_001081112:exon31:c.A4529G:p.Y1510C	**Y1510C**	ENSMUSP00000097809,1510,Y,C	6,118507746,T,T	6qF1	.	0.25	.	43	chr6	118507746	.	T	T	.	PASS	DB;ECNT=1;HCNT=1;MAX_ED=.;MIN_ED=.;NLOD=13.49;TLOD=19.40	GT:AD:AF:ALT_F1R2:ALT_F2R1:FOXOG:QSS:REF_F1R2:REF_F2R1	0/1:16,8:0.333:6:2:0.750:422,222:9:7	0/0:54,0:0.00:0:0:.:1437,0:32:22	2	Tolerated	0	Neutral	69	30	0.209	Tolerated	2.88	74
T12,T1,T22,T2,T32,T3,T42,T4	chr6	**8**	**100**	118511605	118511605	G	T	exonic	**Ankrd26**	ENSMUSP00000097809	.	nonsynonymous SNV	Ankrd26:NM_001081112:exon29:c.C4092A:p.H1364Q	**H1364Q**	ENSMUSP00000097809,1364,H,Q	6,118511605,G,C	6qF1	.	0.25	.	54	chr6	118511605	.	G	C	.	PASS	DB;ECNT=1;HCNT=1;MAX_ED=.;MIN_ED=.;NLOD=12.29;TLOD=36.00	GT:AD:AF:ALT_F1R2:ALT_F2R1:FOXOG:QSS:REF_F1R2:REF_F2R1	0/1:16,15:0.500:7:8:0.533:444,379:7:9	0/0:48,0:0.00:0:0:.:1348,0:27:21	2	Tolerated	0.1	Neutral	69	30	1	Tolerated	2.96	61
T12,T1,T22,T2,T32,T3,T42,T4	chr6	**8**	**100**	118525855	118525855	A	C	exonic	**Ankrd26**	ENSMUSP00000097809	.	nonsynonymous SNV	Ankrd26:NM_001081112:exon22:c.T2353G:p.S785A	**S785A**	ENSMUSP00000097809,785,S,A	6,118525855,A,A	6qF1	.	0.25	.	48	chr6	118525855	.	A	A	.	clustered_events;homologous_mapping_event	DB;ECNT=9;HCNT=16;MAX_ED=203;MIN_ED=17;NLOD=12.04;TLOD=41.14	GT:AD:AF:ALT_F1R2:ALT_F2R1:FOXOG:PGT:PID:QSS:REF_F1R2:REF_F2R1	0/1:22,11:0.379:4:7:0.364:0|1:118532276_G_A:564,313:11:11	0/0:35,0:0.00:0:0:.:0|1:118532276_G_A:852,0:22:13	2	Tolerated	0	Neutral	69	30	0.317	Tolerated	2.91	88
T12,T1,T22,T2,T32,T3,T42,T4	chr6	**8**	**100**	118532293	118532293	C	T	exonic	**Ankrd26**	ENSMUSP00000097809	.	nonsynonymous SNV	Ankrd26:NM_001081112:exon18:c.G1810A:p.A604T	**A604T**	ENSMUSP00000097809,604,A,T	6,118532293,C,G	6qF1	rs218938688	0.25	.	39	chr6	118532293	rs218938688	C	G	.	clustered_events;homologous_mapping_event	DB;ECNT=9;HCNT=16;MAX_ED=203;MIN_ED=17;NLOD=8.38;TLOD=25.45	GT:AD:AF:ALT_F1R2:ALT_F2R1:FOXOG:QSS:REF_F1R2:REF_F2R1	0/1:19,10:0.357:3:7:0.300:544,286:9:10	0/0:29,0:0.00:0:0:.:803,0:16:13	2	Tolerated	2.27	Neutral	69	30	1	Tolerated	3.32	45
T12,T1,T22,T2,T32,T3,T42,T4	chr6	**8**	**100**	118532353	118532353	T	G	exonic	**Ankrd26**	ENSMUSP00000097809	.	nonsynonymous SNV	Ankrd26:NM_001081112:exon18:c.A1750C:p.M584L	**M584L**	ENSMUSP00000097809,584,M,L	6,118532353,T,C	6qF1	.	0.25	.	29	chr6	118532353	.	T	C	.	clustered_events	DB;ECNT=2;HCNT=2;MAX_ED=38;MIN_ED=38;NLOD=13.14;TLOD=50.70	GT:AD:AF:ALT_F1R2:ALT_F2R1:FOXOG:QSS:REF_F1R2:REF_F2R1	0/1:23,21:0.452:9:12:0.429:634,598:9:14	0/0:48,0:0.00:0:0:.:1351,0:26:22	2	Tolerated	-1.89	Neutral	69	30	0.08	Tolerated	3.44	42

T12,T1,T22,T2,T32,T3,T42,T4	chr6	**8**	**100**	118540510	118540510	C	T	exonic	**Ankrd26**	ENSMUSP00000097809	.	nonsynonymous SNV	Ankrd26:NM_001081112:exon13:c.G1349A:p.S450N	**S450N**	ENSMUSP00000097809,450,S,N	6,118540510,C,T	6qF1	.	0.25	.	38	chr6	118540510	.	C	T	.	clustered_events	DB;ECNT=2;HCNT=2;MAX_ED=56;MIN_ED=56;NLOD=10.74;TLOD=21.10	GT:AD:AF:ALT_F1R2:ALT_F2R1:FOXOG:QSS:REF_F1R2:REF_F2R1	0/1:11,8:0.421:5:3:0.375:318,229:8:3	0/0:38,0:0.00:0:0:.:1057,0:17:21	2	Tolerated	1.72	Neutral	69	30	1	Tolerated	3.28	38
T12,T1,T22,T2,T32,T3,T42,T4	chr6	**8**	**100**	118548334	118548334	G	C	exonic	**Ankrd26**	ENSMUSP00000097809	.	nonsynonymous SNV	Ankrd26:NM_001081112:exon11:c.C1195G:p.H399D	**H399D**	ENSMUSP00000097809,399,H,D	6,118548334,G,T	6qF1	rs31578998	0.25	.	48	chr6	118548334	rs31578998	G	T	.	clustered_events;homologous_mapping_event	DB;ECNT=7;HCNT=8;MAX_ED=118;MIN_ED=43;NLOD=9.33;TLOD=32.93	GT:AD:AF:ALT_F1R2:ALT_F2R1:FOXOG:QSS:REF_F1R2:REF_F2R1	0/1:19,15:0.406:10:5:0.333:536,417:6:13	0/0:38,0:0.00:0:0:.:1089,0:16:22	2	Tolerated	0.25	Neutral	69	30	0.055	Tolerated	3.37	37
T12,T1,T22,T2,T32,T3,T42,T4	chr6	**8**	**100**	118554253	118554253	C	T	exonic	**Ankrd26**	ENSMUSP00000097809	.	nonsynonymous SNV	Ankrd26:NM_001081112:exon5:c.G654A:p.M218I	**M218I**	ENSMUSP00000097809,218,M,I	6,118554253,C,C	6qF1	.	0.25	.	38	chr6	118554253	.	C	C	.	clustered_events	DB;ECNT=2;HCNT=2;MAX_ED=15;MIN_ED=15;NLOD=10.77;TLOD=82.73	GT:AD:AF:ALT_F1R2:ALT_F2R1:FOXOG:QSS:REF_F1R2:REF_F2R1	0/1:32,31:0.500:16:15:0.516:817,866:13:19	0/0:41,0:0.00:0:0:.:984,0:18:23	2	Tolerated	0	Neutral	69	30	0.311	Tolerated	3.12	42
T12,T1,T22,T2,T32,T3,T42,T4	chr6	**8**	**100**	118561947	118561947	G	C	exonic	**Ankrd26**	ENSMUSP00000097809	.	nonsynonymous SNV	Ankrd26:NM_001081112:exon1:c.C111G:p.H37Q	**H37Q**	ENSMUSP00000097809,37,H,Q	6,118561947,G,A	6qF1	rs31576214	0.25	.	41	chr6	118561947	rs31576214	G	A	.	alt_allele_in_normal;clustered_events	ECNT=7;HCNT=3;MAX_ED=31;MIN_ED=1;NLOD=4.23;TLOD=9.02	GT:AD:AF:ALT_F1R2:ALT_F2R1:FOXOG:PGT:PID:QSS:REF_F1R2:REF_F2R1	0/1:33,3:0.083:3:0:0.00:0|1:110764148_A_ATTTTTAAT:891,66:15:18	0/0:30,1:0.034:1:0:0.00:0|1:110764148_A_ATTTTTAAT:801,28:15:15	1.4	Tolerated	0	Neutral	69	30	0.54	Tolerated	3.04	56
T12,T1,T22,T2,T32,T3,T42,T4	chr6	**8**	**100**	113765797	113765797	C	A	exonic	**Atp2b2**	ENSMUSP00000047449	.	nonsynonymous SNV	Atp2b2:NM_001036684:exon13:c.G2193T:p.E731D,Atp2b2:NM_009723:exon15:c.G2193T:p.E731D,Atp2b2:NM_001347365:exon17:c.G2328T:p.E776D	**E731D**	ENSMUSP00000047449,731,E,D	6,113765797,C,G	6qE3	rs30756873	0.25	.	41	chr6	113765797	rs30756873	C	G	.	alt_allele_in_normal;clustered_events	ECNT=5;HCNT=1;MAX_ED=24;MIN_ED=1;NLOD=5.43;RPA=3,1;RU=C;STR;TLOD=11.81	GT:AD:AF:ALT_F1R2:ALT_F2R1:FOXOG:PGT:PID:QSS:REF_F1R2:REF_F2R1	0/1:49,4:0.074:4:0:.:0|1:1136373432_T_TTTTTAATGATACG:1370,115:23:26	0/0:38,1:0.030:1:0:.:0|1:1136373432_T_TTTTTAATGATACG:1066,27:14:24	1.4	Tolerated	-2.23	Neutral	366	30	0.131	Tolerated	3.13	387
T12,T1,T22,T2,T32,T3,T42,T4	chr6	**8**	**100**	113765797	113765797	C	A	exonic	**Atp2b2**	ENSMUSP00000047449	.	nonsynonymous SNV	Atp2b2:NM_001036684:exon13:c.G2193T:p.E731D,Atp2b2:NM_009723:exon15:c.G2193T:p.E731D,Atp2b2:NM_001347365:exon17:c.G2328T:p.E776D	**E776D**	ENSMUSP00000047449,776,E,D	6,113765797,C,C	6qE3	rs30756873	0.25	.	41	chr6	113765797	rs30756873	C	C	.	alt_allele_in_normal;clustered_events	ECNT=5;HCNT=1;MAX_ED=24;MIN_ED=1;NLOD=5.43;TLOD=11.84	GT:AD:AF:ALT_F1R2:ALT_F2R1:FOXOG:PGT:PID:QSS:REF_F1R2:REF_F2R1	0/1:50,4:0.075:4:0:.:0|1:1136373432_T_TTTTTAATGATACG:1253,57:21:29	0/0:37,1:0.030:1:0:.:0|1:1136373432_T_TTTTTAATGATACG:938,22:13:24	1.4	Tolerated	0	Neutral	366	30	1	Tolerated	3.13	387
T12,T1,T22,T2,T32,T3,T42,T4	chr6	**8**	**100**	118383658	118383658	T	C	exonic	**Bms1**	ENSMUSP00000037870	.	nonsynonymous SNV	Bms1:NM_194339:exon23:c.A3848G:p.D1283G	**D1283G**	ENSMUSP00000037870,1283,D,G	6,118383658,T,T	6qF1	.	0.25	.	37	chr6	118383658	.	T	T	.	clustered_events;homologous_mapping_event	DB;ECNT=3;HCNT=4;MAX_ED=77;MIN_ED=39;NLOD=11.94;TLOD=68.29	GT:AD:AF:ALT_F1R2:ALT_F2R1:FOXOG:QSS:REF_F1R2:REF_F2R1	0/1:34,30:0.448:14:16:0.533:926,838:18:16	0/0:43,0:0.00:0:0:.:1195,0:14:29	1.2	Tolerated	0	Neutral	99	30	0.263	Tolerated	4.32	11
T12,T1,T22,T2,T32,T3,T42,T4	chr6	**8**	**100**	118404663	118404663	A	C	exonic	**Bms1**	ENSMUSP00000037870	.	nonsynonymous SNV	Bms1:NM_194339:exon10:c.T1913G:p.V638G	**V638G**	ENSMUSP00000037870,638,V,G	6,118404663,A,G	6qF1	.	0.25	.	42	chr6	118404663	.	A	G	.	clustered_events;homologous_mapping_event	DB;ECNT=3;HCNT=2;MAX_ED=70;MIN_ED=50;NLOD=8.34;TLOD=83.60	GT:AD:AF:ALT_F1R2:ALT_F2R1:FOXOG:PGT:PID:QSS:REF_F1R2:REF_F2R1	0/1:35,23:0.411:9:14:0.609:0|1:118404904_A_G:935,658:17:18	0/0:30,0:0.00:0:0:.:0|1:118404904_A_G:825,0:11:19	1.2	Tolerated	-0.16	Neutral	99	30	0.81	Tolerated	2.97	58
T12,T1,T22,T2,T32,T3,T42,T4	chr6	**8**	**100**	118404702	118404702	A	T	exonic	**Bms1**	ENSMUSP00000037870	.	nonsynonymous SNV	Bms1:NM_194339:exon10:c.T1874A:p.L625Q	**L625Q**	ENSMUSP00000037870,625,L,Q	6,118404702,A,C	6qF1	rs30315116	0.25	.	43	chr6	118404702	rs30315116	A	C	.	clustered_events;homologous_mapping_event	DB;ECNT=3;HCNT=2;MAX_ED=70;MIN_ED=50;NLOD=9.24;TLOD=77.61	GT:AD:AF:ALT_F1R2:ALT_F2R1:FOXOG:PGT:PID:QSS:REF_F1R2:REF_F2R1	0/1:35,22:0.389:9:13:0.409:0|1:118404904_A_G:972,615:17:18	0/0:35,0:0.00:0:0:.:0|1:118404904_A_G:907,0:14:21	1.2	Tolerated	-0.07	Neutral	99	30	0.692	Tolerated	3.28	44
T12,T1,T22,T2,T32,T3,T42,T4	chr6	**8**	**100**	118404904	118404904	A	G	exonic	**Bms1**	ENSMUSP00000037870	.	nonsynonymous SNV	Bms1:NM_194339:exon10:c.T1672C:p.S558P	**S558P**	ENSMUSP00000037870,558,S,P	6,118404904,A,G	6qF1	rs216199045	0.25	.	30	chr6	118404904	rs216199045	A	G	.	clustered_events	DB;ECNT=2;HCNT=2;MAX_ED=23;MIN_ED=23;NLOD=9.92;TLOD=66.77	GT:AD:AF:ALT_F1R2:ALT_F2R1:FOXOG:QSS:REF_F1R2:REF_F2R1	0/1:42,26:0.413:10:16:0.615:1209,731:21:21	0/0:35,0:0.00:0:0:.:960,0:18:17	1.2	Tolerated	-0.42	Neutral	99	30	0.282	Tolerated	3.49	43

T12,T1,T22,T2,T32,T3,T42,T4	chr6	**8**	**100**	118404924	118404924	T	C	exonic	**Bms1**	ENSMUSP00000037870	.	nonsynonymous SNV	Bms1:NM_194339:exon10:c.A1652G:p.E551G	**E551G**	ENSMUSP00000037870,551,E,G	6,118404924,T,A	6qF1	rs31557210	0.25	.	35	chr6	118404924	rs31557210	T	A	.	clustered_events;homologous_mapping_event	DB;ECNT=3;HCNT=2;MAX_ED=70;MIN_ED=13;NLOD=13.17;TLOD=43.70	GT:AD:AF:ALT_F1R2:ALT_F2R1:FOXOG:QSS:REF_F1R2:REF_F2R1	0/1:17,20:0.500:11:9:0.550:415,575:6:11	0/0:50,0:0.00:0:0:.:1208,0:26:24	1.2	Tolerated	-0.58	Neutral	99	30	0.086	Tolerated	3.48	42
T12,T1,T22,T2,T32,T3,T42,T4	chr6	**8**	**100**	118405014	118405014	C	G	exonic	**Bms1**	ENSMUSP00000037870	.	nonsynonymous SNV	Bms1:NM_194339:exon10:c.G1562C:p.G521A	**G521A**	ENSMUSP00000037870,521,G,A	6,118405014,C,A	6qF1	rs31557212	0.25	.	35	chr6	118405014	rs31557212	C	A	.	str_contraction;t_lod_fstar	ECNT=1;HCNT=8;MAX_ED=.;MIN_ED=.;NLOD=8.42;RPA=7,6;RU=CAG;STR;TLOD=4.61	GT:AD:AF:ALT_F1R2:ALT_F2R1:FOXOG:QSS:REF_F1R2:REF_F2R1	0/1:59,2:0.033:0:2:.:1662,57:28:31	0/0:31,0:0.00:0:0:.:872,0:20:11	18	Tolerated	0.49	Neutral	99	30	0.103	Tolerated	3.1	49
T12,T1,T22,T2,T32,T3,T42,T4	chr6	**8**	**100**	118411483	118411483	G	A	exonic	**Bms1**	ENSMUSP00000037870	.	nonsynonymous SNV	Bms1:NM_194339:exon8:c.C1094T:p.A365V	**A365V**	ENSMUSP00000037870,365,A,V	6,118411483,G,A	6qF1	rs31563226	0.25	.	50	chr6	118411483	rs31563226	G	A	.	str_contraction;t_lod_fstar	ECNT=1;HCNT=8;MAX_ED=.;MIN_ED=.;NLOD=8.42;RPA=7,6;RU=CAG;STR;TLOD=4.61	GT:AD:AF:ALT_F1R2:ALT_F2R1:FOXOG:QSS:REF_F1R2:REF_F2R1	0/1:59,2:0.033:0:2:.:1662,57:28:31	0/0:31,0:0.00:0:0:.:872,0:20:11	18	Tolerated	0.12	Neutral	99	30	0.279	Tolerated	2.82	35
T12,T1,T22,T2,T32,T3,T42,T4	chr6	**8**	**100**	118593983	118593983	G	A	exonic	**Cacna1c**	ENSMUSP00000075021	.	nonsynonymous SNV	5:exon42:c.C5519T:p.A1840V,Cacna1c:NM_001159533:exon47:c.C6329T:p.A2110V,Cacna1c:NM_001159534:exon47:c.C6329T:p.A2110V,Cacna1c:NM_001159535:exon47:c.C6323T:p.A2108V,Cacna1c:NM_001255997:exon47:c.C6419T:p.A2140V,Cacna1c:NM_001255999:exon47:c.C6419T:p.A2140V,Cacna1c:NM_001256000:exon47:c.C6371T:p.A2124V,Cacna1c:NM_001256001:exon47:c.C6380T:p.A2127V,Cacna1c:NM_009781:exon47:c.C6419T:p.A2140V,Cacn	**A2165V**	ENSMUSP00000075021,2165,A,V	6,118593983,G,T	6qF1	rs3675286	0.25	.	38	chr6	118593983	rs3675286	G	T	.	str_contraction;t_lod_fstar	ECNT=1;HCNT=2;MAX_ED=.;MIN_ED=.;NLOD=8.98;RPA=9,8;RU=TGC;STR;TLOD=4.35	GT:AD:AF:ALT_F1R2:ALT_F2R1:FOXOG:QSS:REF_F1R2:REF_F2R1	0/1:30,2:0.067:1:1:.:861,58:10:20	0/0:33,0:0.00:0:0:.:929,0:15:18	20	Damaging	0.09	Neutral	323	30	0.013	Damaging	3.87	182
T12,T1,T22,T2,T32,T3,T42,T4	chr6	**8**	**100**	118593983	118593983	G	A	exonic	**Cacna1c**	ENSMUSP00000066598	.	nonsynonymous SNV	5:exon42:c.C5519T:p.A1840V,Cacna1c:NM_001159533:exon47:c.C6329T:p.A2110V,Cacna1c:NM_001159534:exon47:c.C6329T:p.A2110V,Cacna1c:NM_001159535:exon47:c.C6323T:p.A2108V,Cacna1c:NM_001255997:exon47:c.C6419T:p.A2140V,Cacna1c:NM_001255999:exon47:c.C6419T:p.A2140V,Cacna1c:NM_001256000:exon47:c.C6371T:p.A2124V,Cacna1c:NM_001256001:exon47:c.C6380T:p.A2127V,Cacna1c:NM_009781:exon47:c.C6419T:p.A2140V,Cacn	**A2193V**	ENSMUSP00000066598,2193,A,V	6,118593983,G,T	6qF1	rs3675286	0.25	.	38	chr6	118593983	rs3675286	G	T	.	t_lod_fstar	ECNT=1;HCNT=1;MAX_ED=.;MIN_ED=.;NLOD=7.51;TLOD=5.00	GT:AD:AF:ALT_F1R2:ALT_F2R1:FOXOG:QSS:REF_F1R2:REF_F2R1	0/1:66,2:0.030:1:1:.:1883,56:40:26	0/0:26,0:0.00:0:0:.:742,0:15:11	1.8	Damaging	0.09	Neutral	323	30	0.013	Damaging	3.87	182
T12,T1,T22,T2,T32,T3,T42,T4	chr6	**8**	**100**	118593983	118593983	G	A	exonic	**Cacna1c**	ENSMUSP00000046229	.	nonsynonymous SNV	5:exon42:c.C5519T:p.A1840V,Cacna1c:NM_001159533:exon47:c.C6329T:p.A2110V,Cacna1c:NM_001159534:exon47:c.C6329T:p.A2110V,Cacna1c:NM_001159535:exon47:c.C6323T:p.A2108V,Cacna1c:NM_001255997:exon47:c.C6419T:p.A2140V,Cacna1c:NM_001255999:exon47:c.C6419T:p.A2140V,Cacna1c:NM_001256000:exon47:c.C6371T:p.A2124V,Cacna1c:NM_001256001:exon47:c.C6380T:p.A2127V,Cacna1c:NM_009781:exon47:c.C6419T:p.A2140V,Cacn	**A1840V**	ENSMUSP00000046229,1840,A,V	6,118593983,G,A	6qF1	rs3675286	0.25	.	38	chr6	118593983	rs3675286	G	A	.	PASS	DB;ECNT=1;HCNT=1;MAX_ED=.;MIN_ED=.;NLOD=10.48;TLOD=64.39	GT:AD:AF:ALT_F1R2:ALT_F2R1:FOXOG:QSS:REF_F1R2:REF_F2R1	0/1:27,26:0.490:14:12:0.538:763,719:20:7	0/0:38,0:0.00:0:0:.:1036,0:16:22	4	Tolerated	-1.08	Neutral	323	30	0.095	Tolerated	3.87	182

T12,T1,T22,T2,T32,T3,T42,T4	chr6	**8**	**100**	118593983	118593983	G	A	exonic	**Cacna1c**	ENSMUSP00000046229	.	nonsynonymous SNV	5:exon42:c.C5519T:p.A1840V,Cacna1c:NM_001159533:exon47:c.C6329T:p.A2110V,Cacna1c:NM_001159534:exon47:c.C6329T:p.A2110V,Cacna1c:NM_001159535:exon47:c.C6323T:p.A2108V,Cacna1c:NM_001255997:exon47:c.C6419T:p.A2140V,Cacna1c:NM_001255999:exon47:c.C6419T:p.A2140V,Cacna1c:NM_001256000:exon47:c.C6371T:p.A2124V,Cacna1c:NM_001256001:exon47:c.C6380T:p.A2127V,Cacna1c:NM_009781:exon47:c.C6419T:p.A2140V,Cacn	**A2108V**	ENSMUSP00000046229,2108,A,V	6,118593983,G,A	6qF1	rs3675286	0.25	.	38	chr6	118593983	rs3675286	G	A	.	PASS	DB;ECNT=1;HCNT=1;MAX_ED=.;MIN_ED=.;NLOD=10.48;TLOD=64.39	GT:AD:AF:ALT_F1R2:ALT_F2R1:FOXOG:QSS:REF_F1R2:REF_F2R1	0/1:27,26:0.490:14:12:0.538:763,719:20:7	0/0:38,0:0.00:0:0:.:1036,0:16:22	4	Tolerated	-1.08	Neutral	323	30	0.095	Tolerated	3.87	182
T12,T1,T22,T2,T32,T3,T42,T4	chr6	**8**	**100**	118593983	118593983	G	A	exonic	**Cacna1c**	ENSMUSP00000046229	.	nonsynonymous SNV	5:exon42:c.C5519T:p.A1840V,Cacna1c:NM_001159533:exon47:c.C6329T:p.A2110V,Cacna1c:NM_001159534:exon47:c.C6329T:p.A2110V,Cacna1c:NM_001159535:exon47:c.C6323T:p.A2108V,Cacna1c:NM_001255997:exon47:c.C6419T:p.A2140V,Cacna1c:NM_001255999:exon47:c.C6419T:p.A2140V,Cacna1c:NM_001256000:exon47:c.C6371T:p.A2124V,Cacna1c:NM_001256001:exon47:c.C6380T:p.A2127V,Cacna1c:NM_009781:exon47:c.C6419T:p.A2140V,Cacn	**A2110V**	ENSMUSP00000046229,2110,A,V	6,118593983,G,A	6qF1	rs3675286	0.25	.	38	chr6	118593983	rs3675286	G	A	.	PASS	DB;ECNT=1;HCNT=1;MAX_ED=.;MIN_ED=.;NLOD=10.48;TLOD=64.39	GT:AD:AF:ALT_F1R2:ALT_F2R1:FOXOG:QSS:REF_F1R2:REF_F2R1	0/1:27,26:0.490:14:12:0.538:763,719:20:7	0/0:38,0:0.00:0:0:.:1036,0:16:22	4	Tolerated	-1.08	Neutral	323	30	0.095	Tolerated	3.87	182
T12,T1,T22,T2,T32,T3,T42,T4	chr6	**8**	**100**	118593983	118593983	G	A	exonic	**Cacna1c**	ENSMUSP00000046229	.	nonsynonymous SNV	Cacna1c:NM_001290335:exon42:c.C5519T:p.A1840V,Cacna1c:NM_001159533:exon47:c.C6329T:p.A2110V,Cacna1c:NM_001159534:exon47:c.C6329T:p.A2110V,Cacna1c:NM_001159535:exon47:c.C6323T:p.A2108V,Cacna1c:NM_001255997:exon47:c.C6419T:p.A2140V,Cacna1c:NM_001255999:exon47:c.C6419T:p.A2140V,Cacna1c:NM_001256000:exon47:c.C6371T:p.A2124V,Cacna1c:NM_001256001:exon47:c.C6380T:p.A2127V,Cacna1c:NM_009781:exon47:c.	**A2124V**	ENSMUSP00000046229,2124,A,V	6,118593983,G,A	6qF1	rs3675286	0.25	.	38	chr6	118593983	rs3675286	G	A	.	PASS	DB;ECNT=1;HCNT=1;MAX_ED=.;MIN_ED=.;NLOD=10.48;TLOD=64.39	GT:AD:AF:ALT_F1R2:ALT_F2R1:FOXOG:QSS:REF_F1R2:REF_F2R1	0/1:27,26:0.490:14:12:0.538:763,719:20:7	0/0:38,0:0.00:0:0:.:1036,0:16:22	4	Tolerated	-1.08	Neutral	323	30	0.095	Tolerated	3.87	182
T12,T1,T22,T2,T32,T3,T42,T4	chr6	**8**	**100**	118593983	118593983	G	A	exonic	**Cacna1c**	ENSMUSP00000046229	.	nonsynonymous SNV	Cacna1c:NM_001290335:exon42:c.C5519T:p.A1840V,Cacna1c:NM_001159533:exon47:c.C6329T:p.A2110V,Cacna1c:NM_001159534:exon47:c.C6329T:p.A2110V,Cacna1c:NM_001159535:exon47:c.C6323T:p.A2108V,Cacna1c:NM_001255997:exon47:c.C6419T:p.A2140V,Cacna1c:NM_001255999:exon47:c.C6419T:p.A2140V,Cacna1c:NM_001256000:exon47:c.C6371T:p.A2124V,Cacna1c:NM_001256001:exon47:c.C6380T:p.A2127V,Cacna1c:NM_009781:exon47:c.C6419T:p.A2140V,Cacn	**A2127V**	ENSMUSP00000046229,2127,A,V	6,118593983,G,A	6qF1	rs3675286	0.25	.	38	chr6	118593983	rs3675286	G	A	.	PASS	DB;ECNT=1;HCNT=1;MAX_ED=.;MIN_ED=.;NLOD=10.48;TLOD=64.39	GT:AD:AF:ALT_F1R2:ALT_F2R1:FOXOG:QSS:REF_F1R2:REF_F2R1	0/1:27,26:0.490:14:12:0.538:763,719:20:7	0/0:38,0:0.00:0:0:.:1036,0:16:22	4	Tolerated	-1.08	Neutral	323	30	0.095	Tolerated	3.87	182

T12,T1,T22,T2,T32,T3,T42,T4	chr6	**8**	**100**	118593983	118593983	G	A	exonic	**Cacna1c**	ENSMUSP00000046229	.	nonsynonymous SNV	_5:exon42:c.C5519T:p.A1840V,Cacna1c:NM_001159533:exon47:c.C6329T:p.A2110V,Cacna1c:NM_001159534:exon47:c.C6329T:p.A2110V,Cacna1c:NM_001159535:exon47:c.C6323T:p.A2108V,Cacna1c:NM_001255997:exon47:c.C6419T:p.A2140V,Cacna1c:NM_001255999:exon47:c.C6419T:p.A2140V,Cacna1c:NM_001256000:exon47:c.C6371T:p.A2124V,Cacna1c:NM_001256001:exon47:c.C6380T:p.A2127V,Cacna1c:NM_009781:exon47:c.C6419T:p.A2140V,Cacn	**A2140V**	ENSMUSP00000046229,2140,A,V	6,118593983,G,A	6qF1	rs3675286	0.25	.	38	chr6	118593983	rs3675286	G	A	.	PASS	DB;ECNT=1;HCNT=1;MAX_ED=.;MIN_ED=.;NLOD=10.48;TLOD=64.39	GT:AD:AF:ALT_F1R2:ALT_F2R1:FOXOG:QSS:REF_F1R2:REF_F2R1	0/1:27,26:0.490:14:12:0.538:763,719:20:7	0/0:38,0:0.00:0:0:.:1036,0:16:22	4	Tolerated	-1.08	Neutral	323	30	0.095	Tolerated	3.87	182
T12,T1,T22,T2,T32,T3,T42,T4	chr6	**8**	**100**	125122006	125122006	A	T	exonic	**Chd4**	ENSMUSP00000125742	.	nonsynonymous SNV	Chd4:NM_001346610:exon32:c.A4735T:p.T1579S,Chd4:NM_145979:exon32:c.A4735T:p.T1579S	**T1579S**	ENSMUSP00000125742,1579,T,S	6,125122006,A,CTCCCTG	6qF2	rs13463121	0.25	.	38	chr6	125122006	rs13463121	A	CTCCCTG	.	clustered_events	ECNT=2;HCNT=1;MAX_ED=9;MIN_ED=9;NLOD=9.05;TLOD=61.92	GT:AD:AF:ALT_F1R2:ALT_F2R1:FOXOG:PGT:PID:QSS:REF_F1R2:REF_F2R1	0/1:30,21:0.391:10:11:.:0|1:125122132_C_CTCCCTG:837,603:18:12	0/0:32,0:0.00:0:0:.:0|1:125122132_C_CTCCCTG:896,0:17:15	3	Neutral	-2.45	Neutral	190	30	NA	NA	NA	NA
T12,T1,T22,T2,T32,T3,T42,T4	chr6	**8**	**100**	125122141	125122141	C	A	exonic	**Chd4**	ENSMUSP00000125742	.	nonsynonymous SNV	Chd4:NM_001346610:exon33:c.C4777A:p.P1593T,Chd4:NM_145979:exon33:c.C4777A:p.P1593T	**P1593T**	ENSMUSP00000125742,1593,P,T	6,125122141,C,T	6qF2	rs30618367	0.25	.	31	chr6	125122141	.	C	T	.	alt_allele_in_normal;clustered_events	ECNT=12;HCNT=8;MAX_ED=164;MIN_ED=9;NLOD=10.26;TLOD=24.36	GT:AD:AF:ALT_F1R2:ALT_F2R1:FOXOG:PGT:PID:QSS:REF_F1R2:REF_F2R1	0/1:87,9:0.091:6:3:0.333:0|1:37423406_C_A:2426,262:44:43	0/0:63,2:0.031:1:1:0.500:0|1:37423406_C_A:1727,59:34:29	0.8	Tolerated	-1.46	Neutral	190	30	0.137	Tolerated	3.43	161
T12,T1,T22,T2,T32,T3,T42,T4	chr6	**8**	**100**	125122132	125122132	-	TCCCTG	exonic	**Chd4**	ENSMUSP00000125742	.	nonframeshift insertion	Chd4:NM_001346610:exon33:c.4768_4769insTCCCTG:p.P1590delinsLPA,Chd4:NM_145979:exon33:c.4768_4769insTCCCTG:p.P1590delinsLPA	**P1590delinsLPA**	ENSMUSP00000125742,1590,P,PA	6,125122132,C,A	6qF2	.	0.25	.	32	chr6	125122132	.	C	A	.	alt_allele_in_normal;clustered_events	ECNT=12;HCNT=8;MAX_ED=164;MIN_ED=9;NLOD=10.78;TLOD=14.98	GT:AD:AF:ALT_F1R2:ALT_F2R1:FOXOG:QSS:REF_F1R2:REF_F2R1	0/1:77,8:0.104:6:2:0.750:2059,228:44:33	0/0:65,2:0.034:1:1:0.500:1674,57:31:34	0.8	Tolerated	-0.43	Neutral	190	30	0.26	Tolerated	3.42	161
T12,T1,T22,T2,T32,T3,T42,T4	chr6	**8**	**100**	129376260	129376260	G	T	exonic	**Clec12b**	ENSMUSP00000060054	.	nonsynonymous SNV	Clec12b:NM_027709:exon6:c.C763A:p.R255S,Clec12b:NM_001204223:exon7:c.C763A:p.R255S	**R255S**	ENSMUSP00000060054,255,R,S	6,129376260,G,A	6qF3	rs38552283	0.25	.	35	chr6	129376260	rs38552283	G	A	.	clustered_events;homologous_mapping_event	DB;ECNT=8;HCNT=38;MAX_ED=199;MIN_ED=18;NLOD=10.83;TLOD=28.80	GT:AD:AF:ALT_F1R2:ALT_F2R1:FOXOG:QSS:REF_F1R2:REF_F2R1	0/1:14,12:0.440:3:9:0.750:391,339:7:7	0/0:42,0:0.00:0:0:.:1194,0:22:20	2	Damaging	-1.96	Neutral	166	30	0.024	Damaging	2.79	44
T12,T1,T22,T2,T32,T3,T42,T4	chr6	**8**	**100**	129376229	129376229	C	G	exonic	**Clec12b**	ENSMUSP00000060054	.	nonsynonymous SNV	Clec12b:NM_027709:exon6:c.G794C:p.R265T,Clec12b:NM_001204223:exon7:c.G794C:p.R265T	**R265T**	ENSMUSP00000060054,265,R,T	NA	6qF3	.	0.25	.	32	chr6	129376229	.	C	C	.	clustered_events;homologous_mapping_event	DB;ECNT=8;HCNT=42;MAX_ED=199;MIN_ED=18;NLOD=9.28;TLOD=36.48	GT:AD:AF:ALT_F1R2:ALT_F2R1:FOXOG:QSS:REF_F1R2:REF_F2R1	0/1:19,15:0.387:6:9:0.400:525,382:9:10	0/0:38,0:0.00:0:0:.:1084,0:18:20	2	Deleterious	-3.27	Deleterious	190	30	0.003	Damaging	3.43	163
T12,T1,T22,T2,T32,T3,T42,T4	chr6	**8**	**100**	129376211	129376211	A	G	exonic	**Clec12b**	ENSMUSP00000060054	.	nonsynonymous SNV	Clec12b:NM_027709:exon6:c.T812C:p.I271T,Clec12b:NM_001204223:exon7:c.T812C:p.I271T	**I271T**	ENSMUSP00000060054,271,I,T	6,129376211,A,T	6qF3	rs37466921	0.25	.	34	chr6	129376211	rs37466921	A	T	.	clustered_events;homologous_mapping_event	DB;ECNT=8;HCNT=42;MAX_ED=199;MIN_ED=18;NLOD=8.41;TLOD=41.03	GT:AD:AF:ALT_F1R2:ALT_F2R1:FOXOG:QSS:REF_F1R2:REF_F2R1	0/1:22,15:0.394:5:10:0.333:575,431:11:11	0/0:35,0:0.00:0:0:.:890,0:17:18	2	Deleterious	-3.2	Deleterious	166	30	0.003	Damaging	2.79	44
T12,T1,T22,T2,T32,T3,T42,T4	chr6	**8**	**100**	129376272	129376272	T	C	exonic	**Clec12b**	ENSMUSP00000060054	.	nonsynonymous SNV	Clec12b:NM_027709:exon6:c.A751G:p.T251A,Clec12b:NM_001204223:exon7:c.A751G:p.T251A	**T251A**	ENSMUSP00000060054,251,T,A	6,129376272,T,A	6qF3	rs36331045	0.25	.	38	chr6	129376272	rs36331045	T	A	.	clustered_events;homologous_mapping_event	DB;ECNT=8;HCNT=38;MAX_ED=199;MIN_ED=18;NLOD=11.39;TLOD=33.70	GT:AD:AF:ALT_F1R2:ALT_F2R1:FOXOG:QSS:REF_F1R2:REF_F2R1	0/1:16,13:0.464:4:9:0.308:466,360:7:9	0/0:40,0:0.00:0:0:.:1138,0:18:22	2	Tolerated	0.16	Neutral	166	30	0.128	Tolerated	2.79	44
T12,T1,T22,T2,T32,T3,T42,T4	chr6	**8**	**100**	129376303	129376303	C	A	exonic	**Clec12b**	ENSMUSP00000060054	.	nonsynonymous SNV	Clec12b:NM_027709:exon6:c.G720T:p.E240D,Clec12b:NM_001204223:exon7:c.G720T:p.E240D	**E240D**	ENSMUSP00000060054,240,E,D	6,129376303,C,T	6qF3	rs229478585	0.25	.	42	chr6	129376303	rs229478585	C	T	.	clustered_events;germline_risk;homologous_mapping_event	DB;ECNT=7;HCNT=8;MAX_ED=161;MIN_ED=10;NLOD=5.36;TLOD=53.33	GT:AD:AF:ALT_F1R2:ALT_F2R1:FOXOG:QSS:REF_F1R2:REF_F2R1	0/1:28,21:0.426:9:12:0.571:703,607:12:16	0/0:21,0:0.00:0:0:.:511,0:12:9	2	Tolerated	0	Neutral	166	30	0.343	Tolerated	2.91	33

T12,T1,T22,T2,T32,T3,T42,T4	chr6	**8**	**100**	129376344	129376344	G	A	exonic	**Clec12b**	ENSMUSP00000060054	.	nonsynonymous SNV	Clec12b:NM_027709:exon6:c.C679T:p.L227F,Clec12b:NM_001204223:exon7:c.C679T:p.L227F	**L227F**	ENSMUSP00000060054,227,L,F	6,129376344,G,A	6qF3	rs38008129	0.25	.	40	chr6	129376344	rs38008129	G	A	.	PASS	DB;ECNT=1;HCNT=1;MAX_ED=.;MIN_ED=.;NLOD=9.57;TLOD=31.19	GT:AD:AF:ALT_F1R2:ALT_F2R1:FOXOG:QSS:REF_F1R2:REF_F2R1	0/1:19,12:0.400:7:5:0.417:505,342:12:7	0/0:34,0:0.00:0:0:.:854,0:11:23	2.1	Tolerated	2.41	Neutral	166	30	1	Tolerated	2.81	43
T12,T1,T22,T2,T32,T3,T42,T4	chr6	**8**	**100**	129385426	129385426	C	T	exonic	**Clec12b**	ENSMUSP00000108009	.	nonsynonymous SNV	Clec12b:NM_027709:exon1:c.G65A:p.R22Q,Clec12b:NM_001204223:exon2:c.G65A:p.R22Q	**R22Q**	ENSMUSP00000108009,22,R,Q	6,129385426,C,C	6qF3	rs225232537	0.25	.	21	chr6	129385426	rs225232537	C	C	.	clustered_events;homologous_mapping_event	DB;ECNT=4;HCNT=2;MAX_ED=66;MIN_ED=64;NLOD=11.14;TLOD=107.88	GT:AD:AF:ALT_F1R2:ALT_F2R1:FOXOG:PGT:PID:QSS:REF_F1R2:REF_F2R1	0/1:38,29:0.446:19:10:0.655:0|1:129400088_G_A:1053,735:13:25	0/0:42,0:0.00:0:0:.:0|1:129400088_G_A:1180,0:19:23	2.2	Tolerated	0	Neutral	166	30	0.884	Tolerated	2.82	49
T12,T1,T22,T2,T32,T3,T42,T4	chr6	**8**	**100**	129401547	129401547	T	C	exonic	**Clec1b**	ENSMUSP00000108011	.	nonsynonymous SNV	Clec1b:NM_001204239:exon2:c.T140C:p.F47S,Clec1b:NM_001204253:exon2:c.T140C:p.F47S,Clec1b:NM_019985:exon3:c.T236C:p.F79S	**F47S**	ENSMUSP00000108011,47,F,S	6,129401547,T,A	6qF3	rs36867419	0.25	.	49	chr6	129401547	rs36867419	T	A	.	PASS	ECNT=1;HCNT=6;MAX_ED=.;MIN_ED=.;NLOD=8.07;TLOD=13.51	GT:AD:AF:ALT_F1R2:ALT_F2R1:FOXOG:QSS:REF_F1R2:REF_F2R1	0/1:36,6:0.146:4:2:0.667:940,171:15:21	0/0:31,0:0.00:0:0:.:771,0:13:18	1.5	Damaging	-1.43	Neutral	155	30	0.006	Damaging	2.78	64
T12,T1,T22,T2,T32,T3,T42,T4	chr6	**8**	**100**	129401547	129401547	T	C	exonic	**Clec1b**	ENSMUSP00000120704	.	nonsynonymous SNV	Clec1b:NM_001204239:exon2:c.T140C:p.F47S,Clec1b:NM_001204253:exon2:c.T140C:p.F47S,Clec1b:NM_019985:exon3:c.T236C:p.F79S	**F79S**	ENSMUSP00000120704,79,F,S	6,129401547,T,A	6qF3	rs36867419	0.25	.	49	chr6	129401547	rs36867419	T	A	.	PASS	DB;ECNT=1;HCNT=1;MAX_ED=.;MIN_ED=.;NLOD=10.48;TLOD=64.39	GT:AD:AF:ALT_F1R2:ALT_F2R1:FOXOG:QSS:REF_F1R2:REF_F2R1	0/1:27,26:0.490:14:12:0.538:763,719:20:7	0/0:38,0:0.00:0:0:.:1036,0:16:22	4	Deleterious	-7.32	Deleterious	181	30	0	Damaging	2.92	223
T12,T1,T22,T2,T32,T3,T42,T4	chr6	**8**	**100**	129400089	129400089	A	G	exonic	**Clec1b**	ENSMUSP00000108011	.	nonsynonymous SNV	Clec1b:NM_019985:exon2:c.A94G:p.M32V	**M32V**	ENSMUSP00000108011,32,M,V	6,129400089,A,C	6qF3	rs38698453	0.25	.	42	chr6	129400089	rs38698453	A	C	.	clustered_events;homologous_mapping_event	DB;ECNT=6;HCNT=8;MAX_ED=100;MIN_ED=60;NLOD=13.49;TLOD=25.06	GT:AD:AF:ALT_F1R2:ALT_F2R1:FOXOG:QSS:REF_F1R2:REF_F2R1	0/1:19,11:0.345:6:5:0.545:534,310:10:9	0/0:49,0:0.00:0:0:.:1350,0:28:21	2.2	Tolerated	-0.55	Neutral	155	30	0.338	Tolerated	2.8	49
T12,T1,T22,T2,T32,T3,T42,T4	chr6	**8**	**100**	129400090	129400090	T	C	exonic	**Clec1b**	ENSMUSP00000108011	.	nonsynonymous SNV	Clec1b:NM_019985:exon2:c.T95C:p.M32T	**M32T**	ENSMUSP00000108011,32,M,T	6,129400090,T,C	6qF3	rs251777061	0.25	.	42	chr6	129400090	rs251777061	T	C	.	clustered_events;homologous_mapping_event	DB;ECNT=6;HCNT=8;MAX_ED=100;MIN_ED=60;NLOD=13.49;TLOD=25.06	GT:AD:AF:ALT_F1R2:ALT_F2R1:FOXOG:QSS:REF_F1R2:REF_F2R1	0/1:19,11:0.345:6:5:0.545:534,310:10:9	0/0:49,0:0.00:0:0:.:1350,0:28:21	2.2	Tolerated	-1.32	Neutral	155	30	0.322	Tolerated	2.8	49
T12,T1,T22,T2,T32,T3,T42,T4	chr6	**8**	**100**	129184190	129184190	C	G	exonic	**Clec2d**	ENSMUSP00000143151	.	nonsynonymous SNV	Clec2d:NM_053109:exon3:c.C249G:p.N83K	**N83K**	ENSMUSP00000143151,83,N,K	6,129184190,C,G	6qF3	.	0.25	.	32	chr6	129184190	.	C	G	.	clustered_events;homologous_mapping_event	ECNT=22;HCNT=32;MAX_ED=181;MIN_ED=8;NLOD=9.63;TLOD=15.63	GT:AD:AF:ALT_F1R2:ALT_F2R1:FOXOG:PGT:PID:QSS:REF_F1R2:REF_F2R1	0/1:42,5:0.109:2:3:0.600:0|1:129184154_CCCAG_C:1139,142:19:23	0/0:32,0:0.00:0:0:.:0|1:129184154_CCCAG_C:891,0:18:14	2.1	Deleterious	-2.74	Deleterious	104	30	0.094	Tolerated	2.79	86
T12,T1,T22,T2,T32,T3,T42,T4	chr6	**8**	**100**	129184248	129184248	T	G	exonic	**Clec2d**	ENSMUSP00000143151	.	nonsynonymous SNV	Clec2d:NM_053109:exon3:c.T307G:p.F103V	**F103V**	ENSMUSP00000143151,103,F,V	6,129184248,T,G	6qF3	.	0.25	.	36	chr6	129184248	.	T	G	.	clustered_events;homologous_mapping_event	ECNT=22;HCNT=32;MAX_ED=181;MIN_ED=8;NLOD=10.23;TLOD=60.97	GT:AD:AF:ALT_F1R2:ALT_F2R1:FOXOG:PGT:PID:QSS:REF_F1R2:REF_F2R1	0/1:43,17:0.283:6:11:0.353:0|1:129184230_T_A:1197,478:18:25	0/0:36,0:0.00:0:0:.:0|1:129184230_T_A:975,0:18:18	2.1	Deleterious	-5.5	Deleterious	104	30	0.066	Tolerated	2.79	87
T12,T1,T22,T2,T32,T3,T42,T4	chr6	**8**	**100**	129184261	129184261	T	G	exonic	**Clec2d**	ENSMUSP00000143151	.	nonsynonymous SNV	Clec2d:NM_053109:exon3:c.T320G:p.F107C	**F107C**	ENSMUSP00000143151,107,F,C	6,129184261,T,G	6qF3	.	0.25	.	39	chr6	129184261	.	T	G	.	clustered_events;homologous_mapping_event	ECNT=22;HCNT=32;MAX_ED=181;MIN_ED=8;NLOD=11.04;TLOD=60.97	GT:AD:AF:ALT_F1R2:ALT_F2R1:FOXOG:PGT:PID:QSS:REF_F1R2:REF_F2R1	0/1:44,18:0.283:8:10:0.444:0|1:129184230_T_A:1224,530:19:25	0/0:39,0:0.00:0:0:.:0|1:129184230_T_A:1046,0:22:17	2.1	Deleterious	-4.08	Deleterious	104	30	0.051	Tolerated	2.79	87
T12,T1,T22,T2,T32,T3,T42,T4	chr6	**8**	**100**	129184296	129184296	G	C	exonic	**Clec2d**	ENSMUSP00000143151	.	nonsynonymous SNV	Clec2d:NM_053109:exon3:c.G355C:p.D119H	**D119H**	ENSMUSP00000143151,119,D,H	6,129184296,G,C	6qF3	.	0.25	.	40	chr6	129184296	.	G	C	.	clustered_events;homologous_mapping_event	ECNT=22;HCNT=32;MAX_ED=181;MIN_ED=8;NLOD=10.23;TLOD=84.53	GT:AD:AF:ALT_F1R2:ALT_F2R1:FOXOG:PGT:PID:QSS:REF_F1R2:REF_F2R1	0/1:47,25:0.338:12:13:0.480:0|1:129184292_G_A:1351,735:17:30	0/0:40,0:0.00:0:0:.:0|1:129184292_G_A:1130,0:21:19	2.1	Deleterious	-3.27	Deleterious	104	30	0.098	Tolerated	2.79	87

T12,T1,T22,T2,T32,T3,T42,T4	chr6	**8**	**100**	129184185	129184185	C	A	exonic	**Clec2d**	ENSMUSP00000143151	.	nonsynonymous SNV	Clec2d:NM_053109:exon3:c.C244A:p.Q82K	**Q82K**	ENSMUSP00000143151,82,Q,K	6,129184185,C,A	6qF3	.	0.25	.	34	chr6	129184185	.	C	A	.	clustered_events;homologous_mapping_event	DB;ECNT=22;HCNT=32;MAX_ED=181;MIN_ED=8;NLOD=9.33;TLOD=15.58	GT:AD:AF:ALT_F1R2:ALT_F2R1:FOXOG:PGT:PID:QSS:REF_F1R2:REF_F2R1	0/1:41,5:0.106:2:3:0.600:0|1:129184154_CCCAG_C:1206,140:19:22	0/0:34,0:0.00:0:0:.:0|1:129184154_CCCAG_C:991,0:19:15	2.1	Tolerated	1.58	Neutral	104	30	1	Tolerated	2.79	86
T12,T1,T22,T2,T32,T3,T42,T4	chr6	**8**	**100**	129184204	129184204	A	G	exonic	**Clec2d**	ENSMUSP00000143151	.	nonsynonymous SNV	Clec2d:NM_053109:exon3:c.A263G:p.E88G	**E88G**	ENSMUSP00000143151,88,E,G	6,129184204,A,G	6qF3	rs221331319	0.25	.	34	chr6	129184204	.	A	G	.	clustered_events;homologous_mapping_event	ECNT=22;HCNT=32;MAX_ED=181;MIN_ED=8;NLOD=8.98;TLOD=9.61	GT:AD:AF:ALT_F1R2:ALT_F2R1:FOXOG:QSS:REF_F1R2:REF_F2R1	0/1:42,6:0.128:2:4:0.667:1154,163:21:21	0/0:34,0:0.00:0:0:.:945,0:18:16	2.1	Tolerated	6.51	Neutral	104	30	1	Tolerated	2.79	87
T12,T1,T22,T2,T32,T3,T42,T4	chr6	**8**	**100**	129184230	129184230	T	A	exonic	**Clec2d**	ENSMUSP00000143151	.	nonsynonymous SNV	Clec2d:NM_053109:exon3:c.T289A:p.Y97N	**Y97N**	ENSMUSP00000143151,97,Y,N	6,129184230,T,A	6qF3	.	0.25	.	35	chr6	129184230	.	T	A	.	clustered_events;homologous_mapping_event	ECNT=22;HCNT=32;MAX_ED=181;MIN_ED=8;NLOD=9.63;TLOD=49.20	GT:AD:AF:ALT_F1R2:ALT_F2R1:FOXOG:PGT:PID:QSS:REF_F1R2:REF_F2R1	0/1:45,14:0.246:6:8:0.429:0|1:129184230_T_A:1253,385:19:26	0/0:35,0:0.00:0:0:.:0|1:129184230_T_A:973,0:19:16	2.1	Tolerated	1	Neutral	104	30	0.339	Tolerated	2.79	87
T12,T1,T22,T2,T32,T3,T42,T4	chr6	**8**	**100**	129184233	129184233	C	T	exonic	**Clec2d**	ENSMUSP00000143151	.	nonsynonymous SNV	Clec2d:NM_053109:exon3:c.C292T:p.P98S	**P98S**	ENSMUSP00000143151,98,P,S	6,129184233,C,T	6qF3	.	0.25	.	37	chr6	129184233	.	C	T	.	clustered_events;homologous_mapping_event	ECNT=22;HCNT=32;MAX_ED=181;MIN_ED=8;NLOD=10.54;TLOD=49.08	GT:AD:AF:ALT_F1R2:ALT_F2R1:FOXOG:PGT:PID:QSS:REF_F1R2:REF_F2R1	0/1:44,14:0.241:6:8:0.571:0|1:129184230_T_A:1215,403:18:26	0/0:37,0:0.00:0:0:.:0|1:129184230_T_A:988,0:20:17	2.1	Tolerated	1.02	Neutral	104	30	0.432	Tolerated	2.79	87
T12,T1,T22,T2,T32,T3,T42,T4	chr6	**8**	**100**	129184237	129184237	G	A	exonic	**Clec2d**	ENSMUSP00000143151	.	nonsynonymous SNV	Clec2d:NM_053109:exon3:c.G296A:p.S99N	**S99N**	ENSMUSP00000143151,99,S,N	6,129184237,G,A	6qF3	.	0.25	.	39	chr6	129184237	.	G	A	.	clustered_events;homologous_mapping_event	ECNT=22;HCNT=32;MAX_ED=181;MIN_ED=8;NLOD=10.84;TLOD=53.23	GT:AD:AF:ALT_F1R2:ALT_F2R1:FOXOG:PGT:PID:QSS:REF_F1R2:REF_F2R1	0/1:45,15:0.263:6:9:0.400:0|1:129184230_T_A:1255,417:18:27	0/0:39,0:0.00:0:0:.:0|1:129184230_T_A:1094,0:21:18	2.1	Tolerated	-0.1	Neutral	104	30	0.446	Tolerated	2.79	87
T12,T1,T22,T2,T32,T3,T42,T4	chr6	**8**	**100**	129184238	129184238	T	A	exonic	**Clec2d**	ENSMUSP00000143151	.	nonsynonymous SNV	Clec2d:NM_053109:exon3:c.T297A:p.S99R	**S99R**	ENSMUSP00000143151,99,S,R	6,129184238,T,A	6qF3	.	0.25	.	38	chr6	129184238	.	T	A	.	clustered_events;homologous_mapping_event	ECNT=22;HCNT=32;MAX_ED=181;MIN_ED=8;NLOD=10.84;TLOD=53.23	GT:AD:AF:ALT_F1R2:ALT_F2R1:FOXOG:PGT:PID:QSS:REF_F1R2:REF_F2R1	0/1:45,15:0.263:6:9:0.400:0|1:129184230_T_A:1258,413:18:27	0/0:38,0:0.00:0:0:.:0|1:129184230_T_A:1064,0:20:18	2.1	Tolerated	1.31	Neutral	104	30	0.908	Tolerated	2.79	87
T12,T1,T22,T2,T32,T3,T42,T4	chr6	**8**	**100**	129184257	129184257	G	A	exonic	**Clec2d**	ENSMUSP00000143151	.	nonsynonymous SNV	Clec2d:NM_053109:exon3:c.G316A:p.A106T	**A106T**	ENSMUSP00000143151,106,A,T	6,129184257,G,A	6qF3	.	0.25	.	35	chr6	129184257	.	G	A	.	clustered_events;homologous_mapping_event	ECNT=22;HCNT=32;MAX_ED=181;MIN_ED=8;NLOD=10.74;TLOD=64.78	GT:AD:AF:ALT_F1R2:ALT_F2R1:FOXOG:PGT:PID:QSS:REF_F1R2:REF_F2R1	0/1:45,18:0.290:8:10:0.444:0|1:129184230_T_A:1280,511:19:26	0/0:35,0:0.00:0:0:.:0|1:129184230_T_A:950,0:18:17	2.1	Tolerated	2.21	Neutral	104	30	1	Tolerated	2.79	87
T12,T1,T22,T2,T32,T3,T42,T4	chr6	**8**	**100**	129184258	129184258	C	A	exonic	**Clec2d**	ENSMUSP00000143151	.	nonsynonymous SNV	Clec2d:NM_053109:exon3:c.C317A:p.A106D	**A106D**	ENSMUSP00000143151,106,A,D	6,129184258,C,A	6qF3	.	0.25	.	35	chr6	129184258	.	C	A	.	clustered_events;homologous_mapping_event	ECNT=22;HCNT=32;MAX_ED=181;MIN_ED=8;NLOD=11.34;TLOD=60.83	GT:AD:AF:ALT_F1R2:ALT_F2R1:FOXOG:PGT:PID:QSS:REF_F1R2:REF_F2R1	0/1:44,18:0.279:8:10:0.556:0|1:129184230_T_A:1254,503:19:25	0/0:35,0:0.00:0:0:.:0|1:129184230_T_A:970,0:18:17	2.1	Tolerated	-0.92	Neutral	104	30	0.142	Tolerated	2.79	87
T12,T1,T22,T2,T32,T3,T42,T4	chr6	**8**	**100**	129419607	129419607	T	C	exonic	**Clec9a**	ENSMUSP00000062049	.	nonsynonymous SNV	Clec9a:NM_001205364:exon4:c.T436C:p.F146L,Clec9a:NM_172732:exon4:c.T439C:p.F147L,Clec9a:NM_001205363:exon5:c.T517C:p.F173L	**N144S**	ENSMUSP00000062049,144,N,S	6,129419607,T,A	6qF3	rs37860376	0.25	.	59	chr6	129419607	rs37860376	T	A	.	clustered_events;multi_event_alt_allele_in_normal;t_lod_fstar	ECNT=4;HCNT=1;MAX_ED=19;MIN_ED=1;NLOD=7.84;TLOD=5.90	GT:AD:AF:ALT_F1R2:ALT_F2R1:FOXOG:PGT:PID:QSS:REF_F1R2:REF_F2R1	0/1:26,2:0.074:2:0:.:0|1:131129021_T_TTTTA:610,17:14:12	0/0:44,1:0.024:1:0:.:0|1:131129021_T_TTTTA:1068,11:26:18	23	Damaging	1.57	Neutral	158	30	0.008	Damaging	2.82	65
T12,T1,T22,T2,T32,T3,T42,T4	chr6	**8**	**100**	129419521	129419521	A	G	exonic	**Clec9a**	ENSMUSP00000062049	.	nonsynonymous SNV	Clec9a:NM_001205364:exon4:c.A350G:p.N117S,Clec9a:NM_172732:exon4:c.A353G:p.N118S,Clec9a:NM_001205363:exon5:c.A431G:p.N144S	**N117S**	ENSMUSP00000062049,117,N,S	6,129419521,A,C	6qF3	rs36360114	0.25	.	51	chr6	129419521	rs36360114	A	C	.	clustered_events;homologous_mapping_event	DB;ECNT=5;HCNT=16;MAX_ED=183;MIN_ED=66;NLOD=14.39;TLOD=54.84	GT:AD:AF:ALT_F1R2:ALT_F2R1:FOXOG:QSS:REF_F1R2:REF_F2R1	0/1:15,24:0.571:9:15:0.375:427,662:6:9	0/0:59,0:0.00:0:0:.:1654,0:27:32	2.1	Deleterious	-3.24	Deleterious	158	30	0.013	Damaging	2.82	65

T12,T1,T22,T2,T32,T3,T42,T4	chr6	8	100	129419607	129419607	T	C	exonic	Clec9a	ENSMUSP00000078469	.	nonsynonymous SNV	Clec9a:NM_001205364:exon4:c.T436C:p.F146L,Clec9a:NM_172732:exon4:c.T439C:p.F147L,Clec9a:NM_001205363:exon5:c.T517C:p.F173L	F173L	ENSMUSP00000078469,173,F,L	6,129419607,T,C	6qF3	rs37860376	0.25	.	59	chr6	129419607	rs37860376	T	C	.	clustered_events;multi_event_alt_allele_in_normal;t_lod_fstar	ECNT=3;HCNT=35;MAX_ED=6;MIN_ED=5;NLOD=19.58;TLOD=5.49	GT:AD:AF:ALT_F1R2:ALT_F2R1:FOXOG:PGT:PID:QSS:REF_F1R2:REF_F2R1	0/1:41,2:0.047:0:2:1.00:0|1:37321427_A_G:1143,53:19:22	0/0:82,1:0.013:0:1:1.00:0|1:37321427_A_G:2307,30:41:41	0.7	Tolerated	3	Neutral	158	30	1	Tolerated	2.82	65
T12,T1,T22,T2,T32,T3,T42,T4	chr6	8	100	129408962	129408962	C	A	exonic	Clec9a	ENSMUSP00000062049	.	nonsynonymous SNV	Clec9a:NM_001205363:exon1:c.C8A:p.A3E,Clec9a:NM_001205364:exon1:c.C8A:p.A3E,Clec9a:NM_001205365:exon1:c.C8A:p.A3E,Clec9a:NM_172732:exon1:c.C8A:p.A3E	A3E	ENSMUSP00000062049,3,A,E	6,129408962,C,C	6qF3	rs37921689	0.25	.	34	chr6	129408962	rs37921689	C	C	.	clustered_events;homologous_mapping_event	DB;ECNT=5;HCNT=16;MAX_ED=183;MIN_ED=66;NLOD=14.39;TLOD=54.84	GT:AD:AF:ALT_F1R2:ALT_F2R1:FOXOG:QSS:REF_F1R2:REF_F2R1	0/1:15,24:0.571:9:15:0.375:427,662:6:9	0/0:59,0:0.00:0:0:.:1654,0:27:32	2.1	Tolerated	0	Neutral	158	30	0.226	Tolerated	3.66	29
T12,T1,T22,T2,T32,T3,T42,T4	chr6	8	100	129419607	129419607	T	C	exonic	Clec9a	ENSMUSP00000062049	.	nonsynonymous SNV	Clec9a:NM_001205364:exon4:c.T436C:p.F146L,Clec9a:NM_172732:exon4:c.T439C:p.F147L,Clec9a:NM_001205363:exon5:c.T517C:p.F173L	N118S	ENSMUSP00000062049,118,N,S	6,129419607,T,C	6qF3	rs37860376	0.25	.	59	chr6	129419607	rs37860376	T	C	.	clustered_events;homologous_mapping_event	DB;ECNT=5;HCNT=16;MAX_ED=183;MIN_ED=66;NLOD=14.39;TLOD=54.84	GT:AD:AF:ALT_F1R2:ALT_F2R1:FOXOG:QSS:REF_F1R2:REF_F2R1	0/1:15,24:0.571:9:15:0.375:427,662:6:9	0/0:59,0:0.00:0:0:.:1654,0:27:32	2.1	Tolerated	3	Neutral	158	30	1	Tolerated	2.82	65
T12,T1,T22,T2,T32,T3,T42,T4	chr6	8	100	129419607	129419607	T	C	exonic	Clec9a	ENSMUSP00000062049	.	nonsynonymous SNV	Clec9a:NM_001205364:exon4:c.T436C:p.F146L,Clec9a:NM_172732:exon4:c.T439C:p.F147L,Clec9a:NM_001205363:exon5:c.T517C:p.F173L	F146L	ENSMUSP00000062049,146,F,L	6,129419607,T,C	6qF3	rs37860376	0.25	.	59	chr6	129419607	rs37860376	T	C	.	clustered_events;homologous_mapping_event	DB;ECNT=5;HCNT=16;MAX_ED=183;MIN_ED=66;NLOD=14.39;TLOD=54.84	GT:AD:AF:ALT_F1R2:ALT_F2R1:FOXOG:QSS:REF_F1R2:REF_F2R1	0/1:15,24:0.571:9:15:0.375:427,662:6:9	0/0:59,0:0.00:0:0:.:1654,0:27:32	2.1	Tolerated	3	Neutral	158	30	1	Tolerated	2.82	65
T12,T1,T22,T2,T32,T3,T42,T4	chr6	8	100	129419607	129419607	T	C	exonic	Clec9a	ENSMUSP00000062049	.	nonsynonymous SNV	Clec9a:NM_001205364:exon4:c.T436C:p.F146L,Clec9a:NM_172732:exon4:c.T439C:p.F147L,Clec9a:NM_001205363:exon5:c.T517C:p.F173L	F147L	ENSMUSP00000062049,147,F,L	6,129419607,T,C	6qF3	rs37860376	0.25	.	59	chr6	129419607	rs37860376	T	C	.	clustered_events;homologous_mapping_event	DB;ECNT=5;HCNT=16;MAX_ED=183;MIN_ED=66;NLOD=14.39;TLOD=54.84	GT:AD:AF:ALT_F1R2:ALT_F2R1:FOXOG:QSS:REF_F1R2:REF_F2R1	0/1:15,24:0.571:9:15:0.375:427,662:6:9	0/0:59,0:0.00:0:0:.:1654,0:27:32	2.1	Tolerated	3	Neutral	158	30	1	Tolerated	2.82	65
T12,T1,T22,T2,T32,T3,T42,T4	chr6	8	100	104804380	104804380	T	A	exonic	Cntn6	ENSMUSP00000032261	.	nonsynonymous SNV	Cntn6:NM_017383:exon12:c.T1426A:p.S476T	S476T	ENSMUSP00000032261,476,S,T	6,104804380,T,A	6qE1	.	0.25	.	57	chr6	104804380	.	T	A	.	clustered_events	DB;ECNT=2;HCNT=1;MAX_ED=5;MIN_ED=5;NLOD=14	GT:AD:AF:ALT_F1R2:ALT_F2R1:FOXOG:PGT:PID:QSS:	0/1:15,9:0.391:6:3:0.667:0|1:104804380_T_A:419,261:6	0/0:57,0:0.00:0:0:.:0|1:104804380_T_A:1548,0:33:24	2.3	Tolerated	-0.78	Neutral	303	30	0.508	Tolerated	2.83	228
T12,T1,T22,T2,T32,T3,T42,T4	chr6	8	100	104863180	104863180	A	G	exonic	Cntn6	ENSMUSP00000134875	.	nonsynonymous SNV	Cntn6:NM_017383:exon23:c.A3040G:p.M1014V	M1014V	ENSMUSP00000134875,1014,M,V	6,104863180,A,C	6qE1	.	0.25	.	49	chr6	104863180	.	A	C	PASS		ECNT=1;HCNT=1;MAX_ED=.;MIN_ED=.;NLOD=9.58;TLOD=8.42	GT:AD:AF:ALT_F1R2:ALT_F2R1:FOXOG:QSS:REF_F1R2:REF_F2R1	0/1:33,5:0.114:4:1:0.800:927,149:17:16	0/0:39,0:0.00:0:0:.:1097,0:16:23	2.1	Tolerated	0.1	Neutral	303	30	0.69	Tolerated	3.03	117
T12,T1,T22,T2,T32,T3,T42,T4	chr6	8	100	113304584	113304584	C	T	exonic	Cpne9	ENSMUSP00000082533	.	nonsynonymous SNV	Cpne9:NM_170673:exon20:c.C1625T:p.A542V	A542V	ENSMUSP00000082533,542,A,V	6,113304584,C,G	6qE3	rs225698057	0.25	.	38	chr6	113304584	rs225698057	C	G	PASS		ECNT=1;HCNT=1;MAX_ED=.;MIN_ED=.;NLOD=16.50;TLOD=8.19	GT:AD:AF:ALT_F1R2:ALT_F2R1:FOXOG:QSS:REF_F1R2:REF_F2R1	0/1:65,3:0.045:0:3:.:1792,86:36:29	0/0:60,0:0.00:0:0:.:1656,0:22:38	2.2	Tolerated	-1.62	Neutral	259	30	0.057	Tolerated	2.9	230
T12,T1,T22,T2,T32,T3,T42,T4	chr15	8	100	78349046	78349046	T	A	exonic	Csf2rb	ENSMUSP00000004646	.	nonsynonymous SNV	Csf2rb:NM_007780:exon14:c.T2552A:p.V851D	V851D	ENSMUSP00000004646,851,V,D	15,78349046,T,G	15qE1	rs231639694	0.25	.	55	chr15	78349046	rs231639694	T	G	.	alt_allele_in_normal;clustered_events;germline_risk	ECNT=2;HCNT=2;MAX_ED=8;MIN_ED=8;NLOD=0.964;TLOD=11.32	GT:AD:AF:ALT_F1R2:ALT_F2R1:FOXOG:PGT:PID:QSS:REF_F1R2:REF_F2R1	0/1:74,4:0.056:2:2:0.500:0|1:3344581_A_G:2118,18:40:34	0/0:66,4:0.063:3:1:0.250:0|1:3344581_A_G:1817,105:39:27	1.9	Damaging	0.21	Neutral	85	30	0.041	Damaging	3.17	42
T12,T1,T22,T2,T32,T3,T42,T4	chr6	8	100	118109113	118109113	T	C	exonic	Csgalnact2	ENSMUSP00000118341	.	nonsynonymous SNV	Csgalnact2:NM_030165:exon8:c.A1618G:p.T540A	T540A	ENSMUSP00000118341,540,T,A	6,118109113,T,A	6qF1	.	0.25	.	40	chr6	118109113	.	T	A	.	clustered_events;homologous_mapping_event;t_lod_fstar	ECNT=9;HCNT=1;MAX_ED=18;MIN_ED=2;NLOD=9.93;TLOD=5.49	GT:AD:AF:ALT_F1R2:ALT_F2R1:FOXOG:PGT:PID:QSS:REF_F1R2:REF_F2R1	0/1:42,2:0.047:0:2:1.00:0|1:75184494_A_T:1010,24:24:18	0/0:34,0:0.00:0:0:.:0|1:75184494_A_T:882,0:16:18	0.4	Tolerated	-0.03	Neutral	123	30	0.277	Tolerated	3.6	41
T12,T1,T22,T2,T32,T3,T42,T4	chr6	8	100	126876410	126876410	A	G	exonic	Dyrk4	ENSMUSP00000107819	.	nonsynonymous SNV	Dyrk4:NM_207210:exon15:c.T1810C:p.S604P	S604P	ENSMUSP00000107819,604,S,P	6,126876410,A,C	6qF3	rs47087308	0.25	.	42	chr6	126876410	rs47087308	A	C	.	alt_allele_in_normal;clustered_events;germline_risk	ECNT=2;HCNT=1;MAX_ED=5;MIN_ED=5;NLOD=1.49;TLOD=7.19	GT:AD:AF:ALT_F1R2:ALT_F2R1:FOXOG:PGT:PID:QSS:REF_F1R2:REF_F2R1	0/1:43,3:0.067:3:0:1.00:0|1:141859064_T_C:1200,83:19:24	0/0:39,2:0.057:0:2:0.00:0|1:141859064_T_C:1107,59:16:23	1.6	Damaging	-0.15	Neutral	91	30	0.032	Damaging	3.35	43

T12,T1,T22,T2,T32,T3,T42,T4	chr6	**8**	**100**	134248465	134248465	G	T	exonic	**Etv6**	ENSMUSP00000073453	.	nonsynonymous SNV	Etv6:NM_007961:exon5:c.G658T:p.A220S,Etv6:NM_001303102:exon6:c.G529T:p.A177S	**A177S**	ENSMUSP00000073453,177,A,S	NA	6qG1	rs31921736	0.25	.	47	chr6	134248465	rs31921736	G	A	.	str_contraction	ECNT=1;HCNT=1;MAX_ED=.;MIN_ED=.;NLOD=6.59;RPA=8,7;RU=CAG;STR;TLOD=7.63	GT:AD:AF:ALT_F1R2:ALT_F2R1:FOXOG:QSS:REF_F1R2:REF_F2R1	0/1:41,3:0.070:1:2:.:1149,84:16:25	0/0:22,0:0.00:0:0:.:608,0:9:13	0.7	Damaging	-2.32	Neutral	107	30	0	Damaging	2.8	60
T12,T1,T22,T2,T32,T3,T42,T4	chr6	**8**	**100**	134248465	134248465	G	T	exonic	**Etv6**	ENSMUSP00000073453	.	nonsynonymous SNV	Etv6:NM_007961:exon5:c.G658T:p.A220S,Etv6:NM_001303102:exon6:c.G529T:p.A177S	**A220S**	ENSMUSP00000073453,220,A,S	6,134248465,G,T	6qG1	rs31921736	0.25	.	47	chr6	134248465	rs31921736	G	T	.	alt_allele_in_normal;t_lod_fstar	ECNT=1;HCNT=8;MAX_ED=.;MIN_ED=.;NLOD=4.18;TLOD=4.81	GT:AD:AF:ALT_F1R2:ALT_F2R1:FOXOG:QSS:REF_F1R2:REF_F2R1	0/1:38,3:0.083:3:0:0.00:1049,81:28:10	0/0:39,2:0.056:1:1:0.500:1099,57:19:20	1.3	Tolerated	-0.74	Neutral	101	30	0.494	Tolerated	3.2	55
T12,T1,T22,T2,T32,T3,T42,T4	chr6	**8**	**100**	128365822	128365822	G	A	exonic	**Foxm1**	ENSMUSP00000029143	.	nonsynonymous SNV	Foxm1:NM_008021:exon2:c.G118A:p.E40K	**E40K**	ENSMUSP00000029143,40,E,K	6,128365822,G,C	6qF3	rs30222042	0.25	.	27	chr6	128365822	rs30222042	G	C	.	alt_allele_in_normal	ECNT=1;HCNT=4;MAX_ED=.;MIN_ED=.;NLOD=6.28;TLOD=7.84	GT:AD:AF:ALT_F1R2:ALT_F2R1:FOXOG:QSS:REF_F1R2:REF_F2R1	0/1:37,5:0.128:2:3:0.400:1019,143:19:18	0/0:60,4:0.069:2:2:0.500:1670,100:30:30	0.9	Damaging	-1.02	Neutral	89	30	0.011	Damaging	3.17	67
T12,T1,T22,T2,T32,T3,T42,T4	chr6	**8**	**100**	125145335	125145335	C	A	exonic	**Iffo1**	ENSMUSP00000028252	.	nonsynonymous SNV	Iffo1:NM_001039669:exon1:c.C79A:p.L27M,Iffo1:NM_001302778:exon1:c.C79A:p.L27M,Iffo1:NM_001302779:exon1:c.C79A:p.L27M,Iffo1:NM_178787:exon1:c.C79A:p.L27M	**L27M**	ENSMUSP00000028252,27,L,M	6,125145335,C,C	6qF2	rs235927984	0.25	.	45	chr6	125145335	rs235927984	C	C	.	t_lod_fstar	ECNT=1;HCNT=1;MAX_ED=.;MIN_ED=.;NLOD=10.89;TLOD=4.98	GT:AD:AF:ALT_F1R2:ALT_F2R1:FOXOG:QSS:REF_F1R2:REF_F2R1	0/1:37,3:0.079:2:1:0.667:1038,81:16:21	0/0:44,1:0.025:1:0:1.00:1160,10:22:22	0.8	Tolerated	0	Neutral	267	30	1	Tolerated	4.05	50
T12,T1,T22,T2,T32,T3,T42,T4	chr6	**8**	**100**	113464792	113464792	G	A	exonic	**Il17re**	ENSMUSP00000082682	.	nonsynonymous SNV	Il17re:NM_001034029:exon6:c.G44A:p.R15H,Il17re:NM_145826:exon7:c.G647A:p.R216H,Il17re:NM_001034031:exon8:c.G44A:p.R15H	**R15H**	ENSMUSP00000082682,15,R,H	6,113464792,G,C	6qE3	rs36479566	0.25	.	30	chr6	113464792	rs36479566	G	C	.	clustered_events;homologous_mapping_event;t_lod_fstar	ECNT=9;HCNT=2;MAX_ED=27;MIN_ED=4;NLOD=10.23;TLOD=5.11	GT:AD:AF:ALT_F1R2:ALT_F2R1:FOXOG:PGT:PID:QSS:REF_F1R2:REF_F2R1	0/1:67,2:0.030:0:2:0.00:0|1:90396389_TGGAA_T:1861,50:34:33	0/0:39,0:0.00:0:0:.:0|1:90396389_TGGAA_T:1061,0:24:15	19	Deleterious	-2.79	Deleterious	120	30	0.078	Tolerated	2.91	81
T12,T1,T22,T2,T32,T3,T42,T4	chr6	**8**	**100**	113464792	113464792	G	A	exonic	**Il17re**	ENSMUSP00000082682	.	nonsynonymous SNV	Il17re:NM_001034029:exon6:c.G44A:p.R15H,Il17re:NM_145826:exon7:c.G647A:p.R216H,Il17re:NM_001034031:exon8:c.G44A:p.R15H	**R216H**	ENSMUSP00000082682,216,R,H	6,113464792,G,T	6qE3	rs36479566	0.25	.	30	chr6	113464792	rs36479566	G	T	.	clustered_events;homologous_mapping_event;t_lod_fstar	ECNT=9;HCNT=2;MAX_ED=27;MIN_ED=4;NLOD=10.23;TLOD=5.12	GT:AD:AF:ALT_F1R2:ALT_F2R1:FOXOG:PGT:PID:QSS:REF_F1R2:REF_F2R1	0/1:65,2:0.031:0:2:0.00:0|1:90396389_TGGAA_T:1846,54:31:34	0/0:38,0:0.00:0:0:.:0|1:90396389_TGGAA_T:1059,0:22:16	19	Tolerated	-1.85	Neutral	120	30	0.109	Tolerated	2.91	81
T12,T1,T22,T2,T32,T3,T42,T4	chr19	**8**	**100**	8897542	8897542	T	G	exonic	**Ints5**	ENSMUSP00000098807	.	nonsynonymous SNV	Ints5:NM_176843:exon2:c.T2864G:p.L955W	**L955W**	ENSMUSP00000098807,955,L,W	19,8897542,T,G	19qA	.	0.25	.	39	chr19	8897542	.	T	G	.	PASS	DB;ECNT=1;HCNT=1;MAX_ED=.;MIN_ED=.;NLOD=9.27;TLOD=81.35	GT:AD:AF:ALT_F1R2:ALT_F2R1:FOXOG:QSS:REF_F1R2:REF_F2R1	0/1:29,32:0.545:16:16:0.500:720,892:14:15	0/0:34,0:0.00:0:0:.:825,0:21:13	1.6	Damaging	-1.52	Neutral	81	30	0.019	Damaging	2.87	39
T12,T1,T22,T2,T32,T3,T42,T4	chr19	**8**	**100**	8897538	8897538	C	A	exonic	**Ints5**	ENSMUSP00000098807	.	nonsynonymous SNV	Ints5:NM_176843:exon2:c.C2860A:p.P954T	**P954T**	ENSMUSP00000098807,954,P,T	19,8897538,C,G	19qA	.	0.25	.	41	chr19	8897538	.	C	G	.	PASS	DB;ECNT=1;HCNT=1;MAX_ED=.;MIN_ED=.;NLOD=9.27;TLOD=81.35	GT:AD:AF:ALT_F1R2:ALT_F2R1:FOXOG:QSS:REF_F1R2:REF_F2R1	0/1:29,32:0.545:16:16:0.500:720,892:14:15	0/0:34,0:0.00:0:0:.:825,0:21:13	1.6	Deleterious	-5.33	Deleterious	81	30	0	Damaging	2.87	39
T12,T1,T22,T2,T32,T3,T42,T4	chr6	**8**	**100**	113675727	113675727	C	G	exonic	**Irak2**	ENSMUSP00000058308	.	nonsynonymous SNV	Irak2:NM_001113553:exon6:c.C735G:p.H245Q,Irak2:NM_172161:exon7:c.C879G:p.H293Q	**H245Q**	ENSMUSP00000058308,245,H,Q	6,113675727,C,G	6qE3	rs36894093	0.25	.	34	chr6	113675727	rs36894093	C	G	.	PASS	ECNT=1;HCNT=1;MAX_ED=.;MIN_ED=.;NLOD=11.39;TLOD=7.36	GT:AD:AF:ALT_F1R2:ALT_F2R1:FOXOG:QSS:REF_F1R2:REF_F2R1	0/1:46,3:0.067:0:3:.:1323,87:20:26	0/0:45,0:0.00:0:0:.:1229,0:28:17	16	Tolerated	1.53	Neutral	179	30	0.827	Tolerated	2.82	86
T12,T1,T22,T2,T32,T3,T42,T4	chr6	**8**	**100**	113675727	113675727	C	G	exonic	**Irak2**	ENSMUSP00000058308	.	nonsynonymous SNV	Irak2:NM_001113553:exon6:c.C735G:p.H245Q,Irak2:NM_172161:exon7:c.C879G:p.H293Q	**H293Q**	ENSMUSP00000058308,293,H,Q	6,113675727,C,AG	6qE3	rs36894093	0.25	.	34	chr6	113675727	rs36894093	C	AG	.	alt_allele_in_normal;clustered_events	ECNT=6;HCNT=6;MAX_ED=60;MIN_ED=38;NLOD=2.45;RPA=5,6;RU=G;STR;TLOD=25.98	GT:AD:AF:ALT_F1R2:ALT_F2R1:FOXOG:QSS:REF_F1R2:REF_F2R1	0/1:141,19:0.123:10:9:.:3978,543:67:74	0/0:141,17:0.105:8:9:.:3908,470:71:70	20	Unknown	NA	NA	179	30	NA	NA	NA	NA
T12,T1,T22,T2,T32,T3,T42,T4	chr1	**8**	**100**	171531090	171531090	G	A	exonic	**Itln1**	ENSMUSP00000116671	.	nonsynonymous SNV	Itln1:NM_010584:exon5:c.C508T:p.R170C	**R170C**	ENSMUSP00000116671,170,R,C	1,171531090,G,G	1qH3	rs31569041	0.25	.	154	chr1	171531090	.	G	G	.	alt_allele_in_normal;clustered_events	ECNT=6;HCNT=8;MAX_ED=60;MIN_ED=38;NLOD=8.23;TLOD=22.54	GT:AD:AF:ALT_F1R2:ALT_F2R1:FOXOG:PGT:PID:QSS:REF_F1R2:REF_F2R1	0/1:147,8:0.058:6:2:0.750:0|1:171530510_G_A:3873,222:69:78	0/0:148,8:0.062:3:5:0.375:0|1:171530510_G_A:3869,227:73:75	20	Tolerated	0	Neutral	144	30	1	Tolerated	2.85	162

T12,T1,T22,T2,T32,T3,T42,T4	chr1	**8**	**100**	171531105	171531105	A	T	exonic	**Itln1**	ENSMUSP00000116671	.	nonsynonymous SNV	Itln1:NM_010584:exon5:c.T493A:p.S165T	**S165T**	ENSMUSP00000116671,165,S,T	1,171531105,A,A	1qH3	.	0.25	.	162	chr1	171531105	.	A	A	.	alt_allele_in_normal;clustered_events	ECNT=8;HCNT=4;MAX_ED=146;MIN_ED=15;NLOD=18.71;TLOD=17.06	GT:AD:AF:ALT_F1R2:ALT_F2R1:FOXOG:PGT:PID:QSS:REF_F1R2:REF_F2R1	0/1:94,6:0.058:3:3:0.500:0|1:171531166_A_T:2638,169:44:50	0/0:110,4:0.037:1:3:0.750:0|1:171531166_A_T:3072,113:55:55	20	Tolerated	0	Neutral	144	30	1	Tolerated	2.85	162
T12,T1,T22,T2,T32,T3,T42,T4	chr6	**8**	**100**	128710587	128710587	C	A	exonic	**Klrb1**	ENSMUSP00000117070	.	nonsynonymous SNV	Klrb1:NM_001099918:exon4:c.G343T:p.G115C	**G115C**	ENSMUSP00000117070,115,G,C	6,128710587,C,CACGGTTTCTTCCGGCCTTGTCCATCCGGCCATGGCTGTGTCGACGAGGCCAGCGGAACCACCAAAGCTGTGGCTACCTCCTGCTAGAT	6qF3	rs31814824	0.25	.	43	chr6	128710587	rs31814824	C	CACGGTTTCTTCCGGCCTTGTCCATCCGGCCATGGCTGTGTCGACGAGGCCAGCGGAACCACCAAAGCTGTGGCTACCTCCTGCTAGAT	.	PASS	ECNT=1;HCNT=1;MAX_ED=.;MIN_ED=.;NLOD=10.01;TLOD=7.48	GT:AD:AF:ALT_F1R2:ALT_F2R1:FOXOG:QSS:REF_F1R2:REF_F2R1	0/1:36,1:0.077:1:0:.:1038,28:13:23	0/0:36,0:0.00:0:0:.:1034,0:15:21	0.5	Unknown	NA	NA	116	30	NA	NA	NA	NA
T12,T1,T22,T2,T32,T3,T42,T4	chr6	**8**	**100**	129677202	129677202	A	C	exonic	**Klrc1**	ENSMUSP00000049584	.	nonsynonymous SNV	Klrc1:NM_001288664:exon3:c.T359G:p.V120G,Klrc1:NM_010652:exon3:c.T410G:p.V137G,Klrc1:NM_001136068:exon4:c.T461G:p.V154G	**V120G**	ENSMUSP00000049584,120,V,G	6,129677202,A,G	6qF3	.	0.25	.	43	chr6	129677202	.	A	G	.	clustered_events	DB;ECNT=2;HCNT=2;MAX_ED=32;MIN_ED=32;NLOD=10.23;TLOD=24.06	GT:AD:AF:ALT_F1R2:ALT_F2R1:FOXOG:QSS:REF_F1R2:REF_F2R1	0/1:13,9:0.409:3:6:0.667:353,262:8:5	0/0:38,0:0.00:0:0:.:1053,0:20:18	2.4	Deleterious	-6.29	Deleterious	198	30	0	Damaging	2.9	216
T12,T1,T22,T2,T32,T3,T42,T4	chr6	**8**	**100**	129677170	129677170	A	G	exonic	**Klrc1**	ENSMUSP00000049584	.	nonsynonymous SNV	Klrc1:NM_001288664:exon3:c.T391C:p.Y131H,Klrc1:NM_010652:exon3:c.T442C:p.Y148H,Klrc1:NM_001136068:exon4:c.T493C:p.Y165H	**Y148H**	ENSMUSP00000049584,148,Y,H	6,129677170,A,C	6qF3	rs30949894	0.25	.	38	chr6	129677170	rs30949894	A	C	.	clustered_events	DB;ECNT=2;HCNT=2;MAX_ED=32;MIN_ED=32;NLOD=11.69;TLOD=22.53	GT:AD:AF:ALT_F1R2:ALT_F2R1:FOXOG:QSS:REF_F1R2:REF_F2R1	0/1:13,9:0.409:5:4:0.444:369,247:9:4	0/0:43,0:0.00:0:0:.:1206,0:23:20	2.4	Deleterious	-6.69	Deleterious	195	30	0.158	Tolerated	2.82	219
T12,T1,T22,T2,T32,T3,T42,T4	chr6	**8**	**100**	129677170	129677170	A	G	exonic	**Klrc1**	ENSMUSP00000049584	.	nonsynonymous SNV	Klrc1:NM_001288664:exon3:c.T391C:p.Y131H,Klrc1:NM_010652:exon3:c.T442C:p.Y148H,Klrc1:NM_001136068:exon4:c.T493C:p.Y165H	**Y165H**	ENSMUSP00000049584,165,Y,H	6,129677170,A,C	6qF3	rs30949894	0.25	.	38	chr6	129677170	rs30949894	A	C	.	clustered_events	DB;ECNT=2;HCNT=2;MAX_ED=32;MIN_ED=32;NLOD=11.69;TLOD=22.53	GT:AD:AF:ALT_F1R2:ALT_F2R1:FOXOG:QSS:REF_F1R2:REF_F2R1	0/1:13,9:0.409:5:4:0.444:369,247:9:4	0/0:43,0:0.00:0:0:.:1206,0:23:20	2.4	Deleterious	-6.69	Deleterious	195	30	0.158	Tolerated	2.82	219
T12,T1,T22,T2,T32,T3,T42,T4	chr6	**8**	**100**	129677170	129677170	A	G	exonic	**Klrc1**	ENSMUSP00000119110	.	nonsynonymous SNV	Klrc1:NM_001288664:exon3:c.T391C:p.Y131H,Klrc1:NM_010652:exon3:c.T442C:p.Y148H,Klrc1:NM_001136068:exon4:c.T493C:p.Y165H	**Y131H**	ENSMUSP00000119110,131,Y,H	6,129677170,A,T	6qF3	rs30949894	0.25	.	38	chr6	129677170	rs30949894	A	T	.	PASS	DB;ECNT=1;HCNT=1;MAX_ED=.;MIN_ED=.;NLOD=14.65;TLOD=70.26	GT:AD:AF:ALT_F1R2:ALT_F2R1:FOXOG:QSS:REF_F1R2:REF_F2R1	0/1:27,27:0.500:14:13:0.481:750,736:18:9	0/0:58,0:0.00:0:0:.:1574,0:34:24	2.4	Deleterious	-5.61	Deleterious	195	30	0.227	Tolerated	2.82	219
T12,T1,T22,T2,T32,T3,T42,T4	chr6	**8**	**100**	129677202	129677202	A	C	exonic	**Klrc1**	ENSMUSP00000049584	.	nonsynonymous SNV	Klrc1:NM_001288664:exon3:c.T359G:p.V120G,Klrc1:NM_010652:exon3:c.T410G:p.V137G,Klrc1:NM_001136068:exon4:c.T461G:p.V154G	**V137G**	ENSMUSP00000049584,137,V,G	6,129677202,A,A	6qF3	.	0.25	.	43	chr6	129677202	.	A	A	.	clustered_events	ECNT=2;HCNT=2;MAX_ED=55;MIN_ED=55;NLOD=11.13;TLOD=19.13	GT:AD:AF:ALT_F1R2:ALT_F2R1:FOXOG:QSS:REF_F1R2:REF_F2R1	0/1:27,8:0.194:1:7:.:743,230:10:17	0/0:41,0:0.00:0:0:.:1139,0:16:25	2.3	Tolerated	0	Neutral	195	30	0.326	Tolerated	2.82	219
T12,T1,T22,T2,T32,T3,T42,T4	chr6	**8**	**100**	129677202	129677202	A	C	exonic	**Klrc1**	ENSMUSP00000049584	.	nonsynonymous SNV	Klrc1:NM_001288664:exon3:c.T359G:p.V120G,Klrc1:NM_010652:exon3:c.T410G:p.V137G,Klrc1:NM_001136068:exon4:c.T461G:p.V154G	**V154G**	ENSMUSP00000049584,154,V,G	6,129677202,A,G	6qF3	.	0.25	.	43	chr6	129677202	.	A	G	.	clustered_events;t_lod_fstar	ECNT=2;HCNT=2;MAX_ED=55;MIN_ED=55;NLOD=9.32;TLOD=5.65	GT:AD:AF:ALT_F1R2:ALT_F2R1:FOXOG:QSS:REF_F1R2:REF_F2R1	0/1:26,3:0.111:0:3:1.00:708,87:13:13	0/0:34,0:0.00:0:0:.:953,0:11:23	2.3	Tolerated	-1.45	Neutral	195	30	0.225	Tolerated	2.82	219
T12,T1,T22,T2,T32,T3,T42,T4	chr6	**8**	**100**	129678755	129678755	A	T	exonic	**Klrc1**	ENSMUSP00000049584	.	nonsynonymous SNV	Klrc1:NM_001136068:exon1:c.T156A:p.H52Q,Klrc1:NM_001288664:exon1:c.T156A:p.H52Q,Klrc1:NM_010652:exon1:c.T156A:p.H52Q	**H52Q**	ENSMUSP00000049584,52,H,Q	6,129678755,A,G	6qF3	rs50315572	0.25	.	58	chr6	129678755	rs50315572	A	G	.	clustered_events	DB;ECNT=2;HCNT=2;MAX_ED=32;MIN_ED=32;NLOD=10.23;TLOD=24.06	GT:AD:AF:ALT_F1R2:ALT_F2R1:FOXOG:QSS:REF_F1R2:REF_F2R1	0/1:13,9:0.409:3:6:0.667:353,262:8:5	0/0:38,0:0.00:0:0:.:1053,0:20:18	2.4	Tolerated	0	Neutral	195	30	0.718	Tolerated	2.82	213
T12,T1,T22,T2,T32,T3,T42,T4	chr6	**8**	**100**	129660543	129660548	AGCAGG	-	exonic	**Klrc2**		.	nonframeshift deletion	Klrc2:NM_001098669:exon1:c.9_14del:p.3_5del,Klrc2:NM_010653:exon1:c.9_14del:p.3_5del	**3_5del**			6qF3	.	0.25	.	41	chr6	129660542	.	AAGCAGG	A	.	clustered_events	ECNT=2;HCNT=2;MAX_ED=55;MIN_ED=55;NLOD=11.13;TLOD=19.13	GT:AD:AF:ALT_F1R2:ALT_F2R1:FOXOG:QSS:REF_F1R2:REF_F2R1	0/1:27,8:0.194:1:7:.:743,230:10:17	0/0:41,0:0.00:0:0:.:1139,0:16:25	2.3	Unknown	.	.	.	.	.	.	.	.

T12,T1,T22,T2,T32,T3,T42,T4	chr6	**8**	**100**	129639089	129639089	A	T	exonic	**Klrc3**	ENSMUSP00000126656	.	nonsynonymous SNV	Klrc3:NM_021378:exon5:c.T662A:p.I221N	**I221N**	ENSMUSP00000126656,221,I,N	6,129639089,A,G	6qF3	rs51282507	0.25	.	28	chr6	129639089	rs51282507	A	G	.	PASS	DB;ECNT=1;HCNT=1;MAX_ED=.;MIN_ED=.;NLOD=10.74;TLOD=35.72	GT:AD:AF:ALT_F1R2:ALT_F2R1:FOXOG:QSS:REF_F1R2:REF_F2R1	0/1:28,15:0.350:9:6:0.400:783,429:14:14	0/0:39,0:0.00:0:0:.:1058,0:20:19	2.4	Damaging	0.22	Neutral	194	30	0.003	Damaging	3.66	21
T12,T1,T22,T2,T32,T3,T42,T4	chr6	**8**	**100**	129639093	129639093	G	T	exonic	**Klrc3**	ENSMUSP00000126656	.	nonsynonymous SNV	Klrc3:NM_021378:exon5:c.C658A:p.P220T	**P220T**	ENSMUSP00000126656,220,P,T	6,129639093,G,T	6qF3	rs218646162	0.25	.	27	chr6	129639093	rs218646162	G	T	.	PASS	DB;ECNT=1;HCNT=1;MAX_ED=.;MIN_ED=.;NLOD=10.83;TLOD=38.72	GT:AD:AF:ALT_F1R2:ALT_F2R1:FOXOG:QSS:REF_F1R2:REF_F2R1	0/1:23,15:0.417:7:8:0.533:637,422:14:9	0/0:39,0:0.00:0:0:.:1094,0:23:16	2.4	Damaging	-0.36	Neutral	194	30	0.015	Damaging	3.66	21
T12,T1,T22,T2,T32,T3,T42,T4	chr6	**8**	**100**	129641419	129641419	A	G	exonic	**Klrc3**	ENSMUSP00000126656	.	nonsynonymous SNV	Klrc3:NM_021378:exon3:c.T424C:p.Y142H	**Y142H**	ENSMUSP00000126656,142,Y,H	6,129641419,A,A	6qF3	rs3694883	0.25	.	39	chr6	129641419	rs3694883	A	A	.	PASS	ECNT=1;HCNT=1;MAX_ED=.;MIN_ED=.;NLOD=11.14;TLOD=25.22	GT:AD:AF:ALT_F1R2:ALT_F2R1:FOXOG:QSS:REF_F1R2:REF_F2R1	0/1:19,7:0.292:2:5:.:538,198:11:8	0/0:39,0:0.00:0:0:.:1101,0:16:23	2.4	Tolerated	0	Neutral	194	30	1	Tolerated	2.8	208
T12,T1,T22,T2,T32,T3,T42,T4	chr6	**8**	**100**	129641532	129641532	A	T	exonic	**Klrc3**	ENSMUSP00000126656	.	nonsynonymous SNV	Klrc3:NM_021378:exon3:c.T311A:p.L104H	**L104H**	ENSMUSP00000126656,104,L,H	6,129641532,A,A	6qF3	rs47378319	0.25	.	39	chr6	129641532	rs47378319	A	A	.	clustered_events;homologous_mapping_event	DB;ECNT=4;HCNT=8;MAX_ED=80;MIN_ED=16;NLOD=7.78;TLOD=35.23	GT:AD:AF:ALT_F1R2:ALT_F2R1:FOXOG:QSS:REF_F1R2:REF_F2R1	0/1:27,15:0.389:7:8:0.467:771,419:18:9	0/0:29,0:0.00:0:0:.:833,0:17:12	2.4	Tolerated	0	Neutral	194	30	0.388	Tolerated	2.81	200
T12,T1,T22,T2,T32,T3,T42,T4	chr6	**8**	**100**	129643275	129643280	AGCAGG	-	exonic	**Klrc3**	ENSMUSP00000126656	.	nonframeshift deletion	Klrc3:NM_021378:exon1:c.9_14del:p.3_5del	**3_5del**	NA	6,129643275,AAGCAGG,T	6qF3	.	0.25	.	39	chr6	129643274	.	AAGCAGG	T	.	str_contraction;t_lod_fstar	ECNT=1;HCNT=1;MAX_ED=.;MIN_ED=.;NLOD=8.90;RPA=10,9;RU=TGC;STR;TLOD=4.53	GT:AD:AF:ALT_F1R2:ALT_F2R1:FOXOG:QSS:REF_F1R2:REF_F2R1	0/1:45,3:0.068:0:3:.:1267,82:17:28	0/0:34,0:0.00:0:0:.:945,0:14:20	13	Unknown	.	.	.	.	.	.	.	.
T12,T1,T22,T2,T32,T3,T42,T4	chr6	**8**	**100**	129622546	129622546	G	A	exonic	**Klrk1**	ENSMUSP00000040548	.	nonsynonymous SNV	Klrk1:NM_033078:exon1:c.C28T:p.H10Y	**H10Y**	ENSMUSP00000040548,10,H,Y	6,129622546,G,T	6qF3	rs30798578	0.25	.	29	chr6	129622546	rs30798578	G	T	.	str_contraction	ECNT=1;HCNT=3;MAX_ED=.;MIN_ED=.;NLOD=9.21;RPA=11,10;RU=TGC;STR;TLOD=6.71	GT:AD:AF:ALT_F1R2:ALT_F2R1:FOXOG:QSS:REF_F1R2:REF_F2R1	0/1:66,7:0.106:4:3:.:1842,197:39:27	0/0:36,0:0.00:0:0:.:1004,0:20:16	7	Tolerated	-0.31	Neutral	145	30	0.132	Tolerated	3.74	22
T12,T1,T22,T2,T32,T3,T42,T4	chr6	**8**	**100**	119320973	119320973	T	G	exonic	**Lrtm2**	ENSMUSP00000032271	.	nonsynonymous SNV	Lrtm2:NM_001172207:exon4:c.A106C:p.I36L,Lrtm2:NM_172492:exon4:c.A106C:p.I36L	**I36L**	ENSMUSP00000032271,36,I,L	6,119320973,T,G	6qF1	rs31661638	0.25	.	45	chr6	119320973	rs31661638	T	G	.	PASS	ECNT=1;HCNT=1;MAX_ED=.;MIN_ED=.;NLOD=11.38;TLOD=11.28	GT:AD:AF:ALT_F1R2:ALT_F2R1:FOXOG:QSS:REF_F1R2:REF_F2R1	0/1:24,5:0.185:4:1:0.200:673,145:13:11	0/0:41,0:0.00:0:0:.:1132,0:18:23	0.7	Tolerated	0.32	Neutral	389	30	1	Tolerated	2.97	171
T12,T1,T22,T2,T32,T3,T42,T4	chr16	**8**	**100**	32752299	32752299	C	A	exonic	**Muc4**	ENSMUSP00000059471	.	nonsynonymous SNV	Muc4:NM_080457:exon2:c.C2176A:p.Q726K	**Q726K**	ENSMUSP00000059471,726,Q,K	16,32752299,C,G	16qB3	.	0.25	.	76	chr16	32752299	.	C	G	.	alt_allele_in_normal;clustered_events	ECNT=5;HCNT=1;MAX_ED=52;MIN_ED=4;NLOD=16.30;TLOD=10.03	GT:AD:AF:ALT_F1R2:ALT_F2R1:FOXOG:PGT:PID:QSS:REF_F1R2:REF_F2R1	0/1:147,3:0.027:2:1:0.333:0|1:32754579_C_A:4200,86:75:72	0/0:101,3:0.040:3:0:0.00:0|1:32754579_C_A:2890,90:52:49	4	Tolerated	-0.02	Neutral	56	30	0.298	Tolerated	3.55	11
T12,T1,T22,T2,T32,T3,T42,T4	chr7	**8**	**100**	49597280	49597280	C	A	exonic	**Nav2**	ENSMUSP00000065353	.	nonsynonymous SNV	Nav2:NM_175272:exon33:c.C6552A:p.N2184K,Nav2:NM_001111016:exon34:c.C6369A:p.N2123K	**N2123K**	ENSMUSP00000065353,2123,N,K	7,49597280,C,T	7qB4	.	0.25	.	30	chr7	49597280	.	C	T	.	clustered_events;homologous_mapping_event	DB;ECNT=9;HCNT=8;MAX_ED=197;MIN_ED=30;NLOD=15.34;TLOD=58.94	GT:AD:AF:ALT_F1R2:ALT_F2R1:FOXOG:QSS:REF_F1R2:REF_F2R1	0/1:24,24:0.500:12:12:0.500:663,641:14:10	0/0:59,0:0.00:0:0:.:1608,0:32:27	2.7	Tolerated	0	Neutral	198	30	1	Tolerated	2.95	326
T12,T1,T22,T2,T32,T3,T42,T4	chr7	**8**	**100**	49597280	49597280	C	A	exonic	**Nav2**	ENSMUSP00000065353	.	nonsynonymous SNV	Nav2:NM_175272:exon33:c.C6552A:p.N2184K,Nav2:NM_001111016:exon34:c.C6369A:p.N2123K	**N2184K**	ENSMUSP00000065353,2184,N,K	7,49597280,C,T	7qB4	.	0.25	.	30	chr7	49597280	.	C	T	.	PASS	DB;ECNT=1;HCNT=1;MAX_ED=.;MIN_ED=.;NLOD=14.99;TLOD=84.76	GT:AD:AF:ALT_F1R2:ALT_F2R1:FOXOG:QSS:REF_F1R2:REF_F2R1	0/1:37,34:0.493:19:15:0.441:1031,931:19:18	0/0:57,0:0.00:0:0:.:1574,0:26:31	2.7	Tolerated	0	Neutral	198	30	1	Tolerated	2.95	326

T12,T1,T22,T2,T32,T3,T42,T4	chr6	**8**	**100**	125169856	125169856	C	T	exonic	**Ncapd2**	ENSMUSP00000099465	.	nonsynonymous SNV	Ncapd2:NM_146171:exon29:c.G3766A:p.V1256I	**V1256I**	ENSMUSP00000099465,1256,V,I	6,125169856,C,A	6qF2	.	0.25	.	57	chr6	125169856	.	C	A	.	clustered_events;multi_event_alt_allele_in_normal;t_lod_fstar	ECNT=7;HCNT=2;MAX_ED=26;MIN_ED=10;NLOD=4.79;TLOD=4.50	GT:AD:AF:ALT_F1R2:ALT_F2R1:FOXOG:PGT:PID:QSS:REF_F1R2:REF_F2R1	0/1:33,3:0.061:0:3:0.00:0|1:134227483_G_A:889,71:11:22	0/0:33,1:0.032:0:1:0.00:0|1:134227483_G_A:901,11:17:16	0.7	Deleterious	-2.83	Deleterious	105	30	0.005	Damaging	2.87	64
T12,T1,T22,T2,T32,T3,T42,T4	chr6	**8**	**100**	125177531	125177531	C	T	exonic	**Ncapd2**	ENSMUSP00000099465	.	nonsynonymous SNV	Ncapd2:NM_146171:exon15:c.G1721A:p.G574D	**G574D**	ENSMUSP00000099465,574,G,D	6,125177531,C,A	6qF2	rs46901660	0.25	.	59	chr6	125177531	rs46901660	C	A	.	clustered_events;multi_event_alt_allele_in_normal;t_lod_fstar	ECNT=7;HCNT=1;MAX_ED=26;MIN_ED=10;NLOD=4.53;TLOD=5.75	GT:AD:AF:ALT_F1R2:ALT_F2R1:FOXOG:PGT:PID:QSS:REF_F1R2:REF_F2R1	0/1:35,2:0.063:0:2:1.00:0|1:134227483_G_A:933,37:15:20	0/0:32,1:0.033:0:1:1.00:0|1:134227483_G_A:832,13:17:15	0.7	Tolerated	-0.94	Neutral	105	30	0.144	Tolerated	3.07	61
T12,T1,T22,T2,T32,T3,T42,T4	chr6	**8**	**100**	125136964	125136964	G	A	exonic	**Nop2**	ENSMUSP00000093813	.	nonsynonymous SNV	Nop2:NM_138747:exon6:c.G478A:p.A160T	**A160T**	ENSMUSP00000093813,160,A,T	6,125136964,G,A	6qF2	rs51670718	0.25	.	36	chr6	125136964	rs51670718	G	A	.	clustered_events	DB;ECNT=2;HCNT=2;MAX_ED=75;MIN_ED=75;NLOD=8.12;TLOD=54.92	GT:AD:AF:ALT_F1R2:ALT_F2R1:FOXOG:QSS:REF_F1R2:REF_F2R1	0/1:27,22:0.438:10:12:0.455:740,622:12:15	0/0:28,0:0.00:0:0:.:782,0:12:16	2.8	Tolerated	0.1	Neutral	84	30	0.14	Tolerated	3.27	65
T12,T1,T22,T2,T32,T3,T42,T4	chr6	**8**	**100**	125144447	125144447	G	A	exonic	**Nop2**	ENSMUSP00000041963	.	nonsynonymous SNV	Nop2:NM_138747:exon16:c.G2191A:p.E731K	**E731K**	ENSMUSP00000041963,731,E,K	6,125144447,G,A	6qF2	rs30890525	0.25	.	28	chr6	125144447	rs30890525	G	A	.	alt_allele_in_normal	ECNT=1;HCNT=2;MAX_ED=.;MIN_ED=.;NLOD=8.82;TLOD=10.55	GT:AD:AF:ALT_F1R2:ALT_F2R1:FOXOG:QSS:REF_F1R2:REF_F2R1	0/1:55,6:0.102:2:4:0.333:1438,163:27:28	0/0:62,3:0.052:1:2:0.333:1534,85:28:34	0.7	Tolerated	0.6	Neutral	84	30	1	Tolerated	3.73	40
T12,T1,T22,T2,T32,T3,T42,T4	chr6	**8**	**100**	125144372	125144372	A	G	exonic	**Nop2**	ENSMUSP00000041963	.	nonsynonymous SNV	Nop2:NM_138747:exon16:c.A2116G:p.R706G	**R706G**	ENSMUSP00000041963,706,R,G	6,125144372,A,T	6qF2	rs29978723	0.25	.	33	chr6	125144372	rs29978723	A	T	.	PASS	DB;ECNT=1;HCNT=1;MAX_ED=.;MIN_ED=.;NLOD=10.48;TLOD=85.18	GT:AD:AF:ALT_F1R2:ALT_F2R1:FOXOG:QSS:REF_F1R2:REF_F2R1	0/1:28,33:0.534:15:18:0.545:787,937:13:15	0/0:39,0:0.00:0:0:.:1072,0:20:19	5	Unknown	NA	NA	84	30	NA	NA	NA	NA
T12,T1,T22,T2,T32,T3,T42,T4	chr6	**8**	**100**	128400210	128400210	A	G	exonic	**Nrip2**	ENSMUSP00000083810	.	nonsynonymous SNV	Nrip2:NM_001162858:exon1:c.A98G:p.Q33R,Nrip2:NM_021717:exon1:c.A98G:p.Q33R	**Q33R**	ENSMUSP00000083810,33,Q,R	6,128400210,A,G	6qF3	rs31795869	0.25	.	42	chr6	128400210	rs31795869	A	G	.	clustered_events;homologous_mapping_event;t_lod_fstar	ECNT=4;HCNT=4;MAX_ED=10;MIN_ED=2;NLOD=5.07;TLOD=5.55	GT:AD:AF:ALT_F1R2:ALT_F2R1:FOXOG:QSS:REF_F1R2:REF_F2R1	0/1:39,2:0.050:0:2:0.00:1108,56:6:33	0/0:22,0:0.00:0:0:.:593,0:0:22	1.9	Tolerated	2.1	Neutral	92	30	1	Tolerated	2.86	62
T12,T1,T22,T2,T32,T3,T42,T4	chr6	**8**	**100**	128407403	128407403	C	T	exonic	**Nrip2**	ENSMUSP00000083810	.	nonsynonymous SNV	Nrip2:NM_001162858:exon4:c.C472T:p.P158S,Nrip2:NM_021717:exon4:c.C472T:p.P158S	**P158S**	ENSMUSP00000083810,158,P,S	6,128407403,C,GCAGAA	6qF3	rs252012303	0.25	.	39	chr6	128407403	rs252012303	C	GCAGAA	.	clustered_events;homologous_mapping_event;t_lod_fstar	ECNT=4;HCNT=2;MAX_ED=10;MIN_ED=2;NLOD=5.42;TLOD=5.60	GT:AD:AF:ALT_F1R2:ALT_F2R1:FOXOG:PGT:PID:QSS:REF_F1R2:REF_F2R1	0/1:38,1:0.053:0:1:.:0|1:72666798_G_GCAGAA:1043,26:6:32	0/0:22,0:0.00:0:0:.:0|1:72666798_G_GCAGAA:625,0:0:22	1.9	Unknown	NA	NA	92	30	NA	NA	NA	NA
T12,T1,T22,T2,T32,T3,T42,T4	chr6	**8**	**100**	113333642	113333642	C	T	exonic	**Ogg1**	ENSMUSP00000047763	.	nonsynonymous SNV	Ogg1:NM_010957:exon6:c.C910T:p.R304W	**R304W**	ENSMUSP00000047763,304,R,W	6,113333642,C,C	6qE3	rs251017737	0.25	.	48	chr6	113333642	rs251017737	C	C	.	alt_allele_in_normal;t_lod_fstar	ECNT=1;HCNT=1;MAX_ED=.;MIN_ED=.;NLOD=2.39;TLOD=5.56	GT:AD:AF:ALT_F1R2:ALT_F2R1:FOXOG:QSS:REF_F1R2:REF_F2R1	0/1:41,2:0.050:1:1:.:1094,54:21:20	0/0:27,1:0.043:1:0:.:743,28:13:14	3	Tolerated	0	Neutral	109	30	1	Tolerated	2.84	122
T12,T1,T22,T2,T32,T3,T42,T4	chr6	**8**	**100**	113333900	113333900	G	A	exonic	**Ogg1**	ENSMUSP00000047763	.	nonsynonymous SNV	Ogg1:NM_010957:exon7:c.G1007A:p.R336H	**R336H**	ENSMUSP00000047763,336,R,H	6,113333900,G,GT	6qE3	rs257746873	0.25	.	32	chr6	113333900	rs257746873	G	GT	.	clustered_events;multi_event_alt_allele_in_normal	ECNT=3;HCNT=6;MAX_ED=74;MIN_ED=67;NLOD=7.54;TLOD=9.26	GT:AD:AF:ALT_F1R2:ALT_F2R1:FOXOG:PGT:PID:QSS:REF_F1R2:REF_F2R1	0/1:28,3:0.100:2:1:.:0|1:123283707_T_C:814,74:16:12	0/0:44,1:0.024:1:0:.:0|1:123283707_T_C:1238,26:26:18	1.4	Unknown	NA	NA	109	30	NA	NA	NA	NA
T12,T1,T22,T2,T32,T3,T42,T4	chr4	**8**	**100**	123283640	123283640	C	T	exonic	**Pabpc4**	ENSMUSP00000020163	.	nonsynonymous SNV	Pabpc4:NM_001356377:exon1:c.C26T:p.P9L,Pabpc4:NM_130881:exon1:c.C26T:p.P9L,Pabpc4:NM_148917:exon1:c.C26T:p.P9L	**P9L**	ENSMUSP00000020163,9,P,L	4,123283640,C,A	4qD2.2	.	0.25	.	48	chr4	123283640	.	C	A	.	alt_allele_in_normal;germline_risk	ECNT=2;HCNT=6;MAX_ED=2;MIN_ED=2;NLOD=0.824;TLOD=27.80	GT:AD:AF:ALT_F1R2:ALT_F2R1:FOXOG:PGT:PID:QSS:REF_F1R2:REF_F2R1	0/1:27,8:0.235:4:4:0.500:0|1:119455790_G_A:668,237:15:12	0/0:34,2:0.065:1:1:0.500:0|1:119455790_G_A:833,57:18:16	2.3	Deleterious	-5.62	Deleterious	290	30	0.007	Damaging	2.96	249
T12,T1,T22,T2,T32,T3,T42,T4	chr19	**8**	**100**	40222708	40222708	A	T	exonic	**Pdlim1**	ENSMUSP00000020732	.	stopgain	Pdlim1:NM_016861:exon7:c.T861A:p.C287X	**C287X**	ENSMUSP00000020732,287,C,X	NA	19qC3	.	0.25	.	43	chr19	40222708	.	A	A	.	clustered_events	ECNT=2;HCNT=1;MAX_ED=11;MIN_ED=11;NLOD=19.77;TLOD=6.81	GT:AD:AF:ALT_F1R2:ALT_F2R1:FOXOG:PGT:PID:QSS:REF_F1R2:REF_F2R1	0/1:94,4:0.043:0:4:0.00:0|1:171356584_G_A:2715,111:56:38	0/0:74,0:0.00:0:0:.:0|1:171356584_G_A:2099,0:36:38	20	Deletarious	.	.	.	.	.	.	.	.

T12,T1,T22,T2,T32,T3,T42,T4	chr6	**8**	**100**	101151237	101151237	C	A	exonic	**Pdzrn3**	ENSMUSP00000032309	.	nonsynonymous SNV	Pdzrn3:NM_018884:exon10:c.G2467T:p.A823S	**A823S**	ENSMUSP00000032309,823,A,S	6,101151237,C,T	6qD3	.	0.25	.	44	chr6	101151237	.	C	T	.	t_lod_fstar	ECNT=1;HCNT=1;MAX_ED=.;MIN_ED=.;NLOD=11.13;TLOD=5.53	GT:AD:AF:ALT_F1R2:ALT_F2R1:FOXOG:QSS:REF_F1R2:REF_F2R1	0/1:32,3:0.088:1:2:0.667:938,86:16:16	0/0:44,0:0.00:0:0:.:1246,0:20:24	0.4	Tolerated	-0.18	Neutral	196	30	0.483	Tolerated	3	177
T12,T1,T22,T2,T32,T3,T42,T4	chr6	**8**	**100**	122323581	122323581	-	TGCTGC	exonic	**Phc1**	ENSMUSP00000032406	.	nonframeshift insertion	Phc1:NM_001042623:exon7:c.1181_1182insGCAGCA:p.Q394delinsQQQ,Phc1:NM_001271579:exon8:c.1337_1338insGCAGCA:p.Q446delinsQQQ,Phc1:NM_007905:exon8:c.1337_1338insGCAGCA:p.Q446delinsQQQ,Phc1:NM_001355215:exon9:c.1385_138	**Q394delinsQQQ**	ENSMUSP00000032406,394,Q,QQQ	6,122323581,T,TTGCTGC	6qF1	.	0.25	.	37	chr6	122323581	.	T	TTGCTGC	.	PASS	ECNT=1;HCNT=1;MAX_ED=.;MIN_ED=.;NLOD=10.90;RPA=6,8;RU=TGC;STR;TLOD=119.17	GT:AD:AF:ALT_F1R2:ALT_F2R1:FOXOG:QSS:REF_F1R2:REF_F2R1	0/1:33,34:0.492:20:14:.:929,948:12:21	0/0:37,0:0.00:0:0:.:1025,0:16:21	.	Neutral	-1.09	Neutral	187	30	NA	NA	NA	NA
T12,T1,T22,T2,T32,T3,T42,T4	chr6	**8**	**100**	122321022	122321022	T	C	exonic	**Phc1**	ENSMUSP00000122181	.	nonsynonymous SNV	Phc1:NM_001042623:exon10:c.A2146G:p.K716E,Phc1:NM_001271579:exon11:c.A2302G:p.K768E,Phc1:NM_007905:exon11:c.A2302G:p.K768E,Phc1:NM_001355215:exon12:c.A2350G:p.K784E	**K768E**	ENSMUSP00000122181,768,K,E	6,122321022,T,TTGCTGC	6qF1	rs30350773	0.25	.	37	chr6	122321022	rs30350773	T	TTGCTGC	.	PASS	ECNT=1;HCNT=1;MAX_ED=.;MIN_ED=.;NLOD=10.90;RPA=6,8;RU=TGC;STR;TLOD=119.17	GT:AD:AF:ALT_F1R2:ALT_F2R1:FOXOG:QSS:REF_F1R2:REF_F2R1	0/1:33,34:0.492:20:14:.:929,948:12:21	0/0:37,0:0.00:0:0:.:1025,0:16:21	2	Tolerated	-1.72	Neutral	56	30	0.205	Tolerated	2.82	44
T12,T1,T22,T2,T32,T3,T42,T4	chr6	**8**	**100**	122321022	122321022	T	C	exonic	**Phc1**	ENSMUSP00000122181	.	nonsynonymous SNV	Phc1:NM_001042623:exon10:c.A2146G:p.K716E,Phc1:NM_001271579:exon11:c.A2302G:p.K768E,Phc1:NM_007905:exon11:c.A2302G:p.K768E,Phc1:NM_001355215:exon12:c.A2350G:p.K784E	**K784E**	ENSMUSP00000122181,784,K,E	6,122321022,T,TTGCTGC	6qF1	rs30350773	0.25	.	37	chr6	122321022	rs30350773	T	TTGCTGC	.	PASS	ECNT=1;HCNT=1;MAX_ED=.;MIN_ED=.;NLOD=10.90;RPA=6,8;RU=TGC;STR;TLOD=119.17	GT:AD:AF:ALT_F1R2:ALT_F2R1:FOXOG:QSS:REF_F1R2:REF_F2R1	0/1:33,34:0.492:20:14:.:929,948:12:21	0/0:37,0:0.00:0:0:.:1025,0:16:21	2	Tolerated	-2.23	Neutral	56	30	0.236	Tolerated	2.82	44
T12,T1,T22,T2,T32,T3,T42,T4	chr6	**8**	**100**	122321022	122321022	T	C	exonic	**Phc1**	ENSMUSP00000032406	.	nonsynonymous SNV	Phc1:NM_001042623:exon10:c.A2146G:p.K716E,Phc1:NM_001271579:exon11:c.A2302G:p.K768E,Phc1:NM_007905:exon11:c.A2302G:p.K768E,Phc1:NM_001355215:exon12:c.A2350G:p.K784E	**K716E**	ENSMUSP00000032406,716,K,E	6,122321022,T,C	6qF1	rs30350773	0.25	.	37	chr6	122321022	rs30350773	T	C	.	PASS	DB;ECNT=1;HCNT=1;MAX_ED=.;MIN_ED=.;NLOD=10.23;TLOD=47.80	GT:AD:AF:ALT_F1R2:ALT_F2R1:FOXOG:QSS:REF_F1R2:REF_F2R1	0/1:38,21:0.368:8:13:0.381:1056,544:23:15	0/0:37,0:0.00:0:0:.:1027,0:21:16	2	Tolerated	-0.56	Neutral	187	30	1	Tolerated	3.59	208
T12,T1,T22,T2,T32,T3,T42,T4	chr6	**8**	**100**	122323581	122323581	-	TGCTGC	exonic	**Phc1**	ENSMUSP00000032406	.	nonframeshift insertion	[illegible]xon7:c.1181_1182insGCAGCA:p.Q394delinsQQQ,Phc1:NM_001271579:exon8:c.1337_1338insGCAGCA:p.Q446delinsQQQ,Phc1:NM_007905:exon8:c.1337_1338insGCAGCA:p.Q446delinsQQQ,Phc1:NM_001355215:exon9:c.1385_138	**Q462delinsQQQ**	ENSMUSP00000032406,462,Q,QQQ	6,122323581,T,C	6qF1	.	0.25	.	37	chr6	122323581	.	T	C	.	str_contraction;t_lod_fstar	ECNT=1;HCNT=1;MAX_ED=.;MIN_ED=.;NLOD=8.93;RPA=10,9;RU=GCA;STR;TLOD=4.75	GT:AD:AF:ALT_F1R2:ALT_F2R1:FOXOG:QSS:REF_F1R2:REF_F2R1	0/1:53,3:0.057:1:2:.:1316,70:24:29	0/0:33,0:0.00:0:0:.:783,0:16:17	1	Tolerated	0	Neutral	187	30	1	Tolerated	4.32	30
T12,T1,T22,T2,T32,T3,T42,T4	chr6	**8**	**100**	122323581	122323581	-	TGCTGC	exonic	**Phc1**	ENSMUSP00000032406	.	nonframeshift insertion	[illegible]xon7:c.1181_1182insGCAGCA:p.Q394delinsQQQ,Phc1:NM_001271579:exon8:c.1337_1338insGCAGCA:p.Q446delinsQQQ,Phc1:NM_007905:exon8:c.1337_1338insGCAGCA:p.Q446delinsQQQ,Phc1:NM_001355215:exon9:c.1385_138	**Q446delinsQQQ**	ENSMUSP00000032406,446,Q,QQQ	6,122323581,T,T	6qF1	.	0.25	.	37	chr6	122323581	.	T	T	.	t_lod_fstar	ECNT=1;HCNT=1;MAX_ED=.;MIN_ED=.;NLOD=11.33;TLOD=4.67	GT:AD:AF:ALT_F1R2:ALT_F2R1:FOXOG:QSS:REF_F1R2:REF_F2R1	0/1:65,3:0.046:3:0:0.00:1598,85:42:23	0/0:41,0:0.00:0:0:.:999,0:13:28	0.8	Tolerated	0	Neutral	187	30	1	Tolerated	4.32	30
T12,T1,T22,T2,T32,T3,T42,T4	chr6	**8**	**100**	125363136	125363136	G	A	exonic	**Plekhg6**	ENSMUSP00000052717	.	nonsynonymous SNV	Plekhg6:NM_198604:exon16:c.C2260T:p.P754S	**P754S**	ENSMUSP00000052717,754,P,S	6,125363136,G,T	6qF3	rs30168962	0.25	.	29	chr6	125363136	rs30168962	G	T	.	clustered_events;homologous_mapping_event	DB;ECNT=3;HCNT=8;MAX_ED=51;MIN_ED=23;NLOD=7.22;TLOD=64.79	GT:AD:AF:ALT_F1R2:ALT_F2R1:FOXOG:QSS:REF_F1R2:REF_F2R1	0/1:38,28:0.410:10:18:0.643:1080,787:19:19	0/0:28,0:0.00:0:0:.:789,0:15:13	2.8	Damaging	-0.77	Neutral	123	30	0.014	Damaging	3.48	30

T12,T1,T22,T2,T32,T3,T42,T4	chr6	**8**	**100**	125372138	125372138	C	T	exonic	**Plekhg6**	ENSMUSP00000052717	.	nonsynonymous SNV	Plekhg6:NM_198604:exon12:c.G1195A:p.V399I	**V399I**	ENSMUSP00000052717,399,V,I	6,125372138,C,C	6qF3	rs3683773	0.25	.	28	chr6	125372138	rs3683773	C	C	.	clustered_events;homologous_mapping_event	DB;ECNT=4;HCNT=16;MAX_ED=200;MIN_ED=55;NLOD=9.63;TLOD=128.69	GT:AD:AF:ALT_F1R2:ALT_F2R1:FOXOG:PGT:PID:QSS:REF_F1R2:REF_F2R1	0/1:28,33:0.541:14:19:0.424:0|1:125375774_T_C:774,950:22:6	0/0:31,0:0.00:0:0:.:0|1:125375774_T_C:884,0:16:15	2.8	Damaging	0	Neutral	123	30	0.033	Damaging	2.84	165
T12,T1,T22,T2,T32,T3,T42,T4	chr6	**8**	**100**	125374626	125374626	C	T	exonic	**Plekhg6**	ENSMUSP00000052717	.	nonsynonymous SNV	Plekhg6:NM_198604:exon8:c.G659A:p.R220Q	**R220Q**	ENSMUSP00000052717,220,R,Q	6,125374626,C,G	6qF3	rs46159311	0.25	.	28	chr6	125374626	rs46159311	C	G	.	str_contraction	ECNT=1;HCNT=1;MAX_ED=.;MIN_ED=.;NLOD=9.31;RPA=8,7;RU=CA;STR;TLOD=8.79	GT:AD:AF:ALT_F1R2:ALT_F2R1:FOXOG:QSS:REF_F1R2:REF_F2R1	0/1:45,5:0.104:3:2:.:1271,148:20:25	0/0:33,0:0.00:0:0:.:930,0:21:12	3	Deleterious	-3.78	Deleterious	123	30	0.123	Tolerated	2.79	154
T12,T1,T22,T2,T32,T3,T42,T4	chr6	**8**	**100**	125363622	125363622	C	T	exonic	**Plekhg6**	ENSMUSP00000052717	.	nonsynonymous SNV	Plekhg6:NM_198604:exon16:c.G1774A:p.G592S	**G592S**	ENSMUSP00000052717,592,G,S	6,125363622,C,T	6qF3	rs30617015	0.25	.	33	chr6	125363622	rs30617015	C	T	.	clustered_events	DB;ECNT=2;HCNT=3;MAX_ED=47;MIN_ED=47;NLOD=7.81;TLOD=42.91	GT:AD:AF:ALT_F1R2:ALT_F2R1:FOXOG:QSS:REF_F1R2:REF_F2R1	0/1:21,16:0.444:11:5:0.313:532,461:7:14	0/0:28,0:0.00:0:0:.:702,0:15:13	2.8	Tolerated	-1.18	Neutral	123	30	0.119	Tolerated	3.41	69
T12,T1,T22,T2,T32,T3,T42,T4	chr6	**8**	**100**	125375774	125375774	T	C	exonic	**Plekhg6**	ENSMUSP00000052717	.	nonsynonymous SNV	Plekhg6:NM_198604:exon5:c.A409G:p.S137G	**S137G**	ENSMUSP00000052717,137,S,G	6,125375774,T,C	6qF3	rs6334042	0.25	.	31	chr6	125375774	rs6334042	T	C	.	PASS	ECNT=1;HCNT=2;MAX_ED=.;MIN_ED=.;NLOD=18.00;TLOD=18.45	GT:AD:AF:ALT_F1R2:ALT_F2R1:FOXOG:QSS:REF_F1R2:REF_F2R1	0/1:16,8:0.364:6:2:0.750:463,209:6:10	0/0:67,0:0.00:0:0:.:1890,0:39:28	1.7	Tolerated	-1.25	Neutral	123	30	0.139	Tolerated	2.99	130
T12,T1,T22,T2,T32,T3,T42,T4	chr6	**8**	**100**	124914453	124914453	-	TCT	exonic	**Ptms**	ENSP00000310088	.	nonframeshift insertion	Ptms:NM_026988:exon4:c.209_210insAGA:p.E70delinsEE	**E70delinsEE**	ENSP00000310088,70,E,EE	6,124914453,C,CTCT	6qF2	.	0.25	.	28	chr6	124914453	.	C	CTCT	.	PASS	ECNT=1;HCNT=1;MAX_ED=.;MIN_ED=.;NLOD=8.15;RPA=2,3;RU=TCT;STR;TLOD=72.51	GT:AD:AF:ALT_F1R2:ALT_F2R1:FOXOG:QSS:REF_F1R2:REF_F2R1	0/1:20,20:0.487:5:15:.:558,565:6:14	0/0:28,0:0.00:0:0:.:795,0:14:14	2.6	Tolerated	9.82	Neutral	31	8	NA	NA	NA	NA
T12,T1,T22,T2,T32,T3,T42,T4	chr11	**8**	**100**	86689871	86689871	C	T	exonic	**Ptrh2**	ENSMUSP00000116194	.	stopgain	Ptrh2:NM_001098810:exon2:c.C313T:p.Q105X,Ptrh2:NM_175004:exon3:c.C316T:p.Q106X	**Q105X**	ENSMUSP00000116194,105,Q,X	NA	11qC	.	0.25	.	52	chr11	86689871	.	C	T	.	PASS	ECNT=1;HCNT=1;MAX_ED=.;MIN_ED=.;NLOD=14.95;TLOD=39.08	GT:AD:AF:ALT_F1R2:ALT_F2R1:FOXOG:QSS:REF_F1R2:REF_F2R1	0/1:30,19:0.370:11:8:0.421:865,513:16:14	0/0:52,0:0.00:0:0:.:1455,0:32:20	10	Deletarious	.	.	.	.	.	.	.	.
T12,T1,T22,T2,T32,T3,T42,T4	chr11	**8**	**100**	86689871	86689871	C	T	exonic	**Ptrh2**	ENSMUSP00000116194	.	stopgain	Ptrh2:NM_001098810:exon2:c.C313T:p.Q105X,Ptrh2:NM_175004:exon3:c.C316T:p.Q106X	**Q106X**	ENSMUSP00000116194,106,Q,X	NA	11qC	.	0.25	.	52	chr11	86689871	.	C	C	.	PASS	DB;ECNT=1;HCNT=2;MAX_ED=.;MIN_ED=.;NLOD=13.19;TLOD=25.32	GT:AD:AF:ALT_F1R2:ALT_F2R1:FOXOG:QSS:REF_F1R2:REF_F2R1	0/1:13,15:0.478:6:9:0.400:374,376:5:8	0/0:50,0:0.00:0:0:.:1382,0:27:23	2.2	Deletarious	.	.	.	.	.	.	.	.
T12,T1,T22,T2,T32,T3,T42,T4	chr11	**8**	**100**	86689778	86689778	C	T	exonic	**Ptrh2**	ENSMUSP00000116194	.	stopgain	Ptrh2:NM_001098810:exon2:c.C220T:p.R74X,Ptrh2:NM_175004:exon3:c.C223T:p.R75X	**R74X**	ENSMUSP00000116194,74,R,X	NA	11qC	.	0.25	.	52	chr11	86689778	.	C	T	.	PASS	ECNT=1;HCNT=2;MAX_ED=.;MIN_ED=.;NLOD=14.07;TLOD=39.12	GT:AD:AF:ALT_F1R2:ALT_F2R1:FOXOG:QSS:REF_F1R2:REF_F2R1	0/1:36,16:0.327:12:4:0.250:901,449:17:19	0/0:52,0:0.00:0:0:.:1276,0:30:22	10	Deletarious	.	.	.	.	.	.	.	.
T12,T1,T22,T2,T32,T3,T42,T4	chr11	**8**	**100**	86689778	86689778	C	T	exonic	**Ptrh2**	ENSMUSP00000116194	.	stopgain	Ptrh2:NM_001098810:exon2:c.C220T:p.R74X,Ptrh2:NM_175004:exon3:c.C223T:p.R75X	**R75X**	ENSMUSP00000116194,74,R,X	NA	11qC	.	0.25	.	52	chr11	86689778	.	C	A	.	clustered_events;homologous_mapping_event	DB;ECNT=5;HCNT=8;MAX_ED=118;MIN_ED=9;NLOD=12.04;TLOD=37.72	GT:AD:AF:ALT_F1R2:ALT_F2R1:FOXOG:PGT:PID:QSS:REF_F1R2:REF_F2R1	0/1:34,11:0.268:9:2:0.818:0|1:128489836_T_C:988,306:13:21	0/0:46,0:0.00:0:0:.:0|1:128489836_T_C:1285,0:29:17	2.2	Deletarious	.	.	.	.	.	.	.	.
T12,T1,T22,T2,T32,T3,T42,T4	chr6	**8**	**100**	128525258	128525258	C	T	exonic	**Pzp**	ENSMUSP00000076911	.	nonsynonymous SNV	Pzp:NM_007376:exon2:c.G127A:p.V43I	**V43I**	ENSMUSP00000076911,43,V,I	6,128525258,C,A	6qF3	rs13460967	0.25	.	39	chr6	128525258	.	C	A	.	PASS	DB;ECNT=1;HCNT=1;MAX_ED=.;MIN_ED=.;NLOD=6.62;TLOD=41.59	GT:AD:AF:ALT_F1R2:ALT_F2R1:FOXOG:QSS:REF_F1R2:REF_F2R1	0/1:21,18:0.444:9:9:0.500:582,506:8:13	0/0:23,0:0.00:0:0:.:657,0:13:10	1	Damaging	-2.15	Neutral	125	30	0.002	Damaging	2.82	118

T12,T1,T22,T2,T32,T3,T42,T4	chr6	**8**	**100**	128489852	128489852	G	A	exonic	**Pzp**	ENSMUSP00000076911	.	nonsynonymous SNV	Pzp:NM_007376:exon28:c.C3398T:p.A1133V	**A1133V**	ENSMUSP00000076911,1133,A,V	6,128489852,G,T	6qF3	rs46929612	0.25	.	46	chr6	128489852	rs46929612	G	T	.	PASS	DB;ECNT=1;HCNT=1;MAX_ED=.;MIN_ED=.;NLOD=9.79;TLOD=45.10	GT:AD:AF:ALT_F1R2:ALT_F2R1:FOXOG:QSS:REF_F1R2:REF_F2R1	0/1:29,18:0.383:5:13:0.722:801,501:14:15	0/0:39,0:0.00:0:0:.:1065,0:12:27	2.2	Deleterious	-2.76	Deleterious	125	30	0.001	Damaging	2.81	127
T12,T1,T22,T2,T32,T3,T42,T4	chr6	**8**	**100**	128506813	128506813	T	C	exonic	**Pzp**	ENSMUSP00000076911	.	nonsynonymous SNV	Pzp:NM_007376:exon14:c.A1621G:p.I541V	**I541V**	ENSMUSP00000076911,541,I,V	6,128506813,T,C	6qF3	rs30855079	0.25	.	50	chr6	128506813	rs30855079	T	C	.	PASS	ECNT=1;HCNT=1;MAX_ED=.;MIN_ED=.;NLOD=12.50;TLOD=7.62	GT:AD:AF:ALT_F1R2:ALT_F2R1:FOXOG:QSS:REF_F1R2:REF_F2R1	0/1:46,4:0.087:2:2:0.500:1309,116:24:22	0/0:49,0:0.00:0:0:.:1315,0:31:18	3	Tolerated	-0.21	Neutral	125	30	1	Tolerated	2.81	127
T12,T1,T22,T2,T32,T3,T42,T4	chr6	**8**	**100**	126939497	126939497	A	G	exonic	**Rad51ap1**	ENSMUSP00000064413	.	nonsynonymous SNV	Rad51ap1:NM_001347455:exon1:c.T14C:p.I5T,Rad51ap1:NM_009013:exon1:c.T14C:p.I5T	**I5T**	ENSMUSP00000064413,5,I,T	NA	6qF3	rs30235850	0.25	.	40	chr6	126939497	rs30235850	A	GATCTGCTGAGATGGCCGCCCAGGAAGAGCCGCAGATCCAATTCAAGC	.	alt_allele_in_normal;clustered_events	ECNT=5;HCNT=1;MAX_ED=22;MIN_ED=3;NLOD=7.56;TLOD=26.06	GT:AD:AF:ALT_F1R2:ALT_F2R1:FOXOG:PGT:PID:QSS:REF_F1R2:REF_F2R1	0/1:50,8:0.145:4:4:.:0|1:129020257_G_A:1356,222:18:32	0/0:58,2:0.036:2:0:.:0|1:129020257_G_A:1526,53:35:23	0.5	Damaging	-0.78	Neutral	170	30	0.007	Damaging	3.71	33
T12,T1,T22,T2,T32,T3,T42,T4	chr6	**8**	**100**	126928176	126928176	T	C	exonic	**Rad51ap1**	ENSMUSP00000064413	.	nonsynonymous SNV	Rad51ap1:NM_001347455:exon5:c.A371G:p.H124R,Rad51ap1:NM_009013:exon5:c.A371G:p.H124R	**H124R**	ENSMUSP00000064413,124,H,R	6,126928176,T,C	6qF3	rs13463008	0.25	.	46	chr6	126928176	.	T	C	.	clustered_events;homologous_mapping_event	DB;ECNT=3;HCNT=8;MAX_ED=30;MIN_ED=12;NLOD=12.33;TLOD=42.05	GT:AD:AF:ALT_F1R2:ALT_F2R1:FOXOG:QSS:REF_F1R2:REF_F2R1	0/1:29,19:0.370:11:8:0.579:829,528:10:19	0/0:45,0:0.00:0:0:.:1290,0:20:25	3	Deleterious	-3.77	Deleterious	63	22	0.514	Tolerated	3.07	50
T12,T1,T22,T2,T32,T3,T42,T4	chr6	**8**	**100**	126928188	126928188	C	T	exonic	**Rad51ap1**	ENSMUSP00000064413	.	nonsynonymous SNV	Rad51ap1:NM_001347455:exon5:c.G359A:p.G120D,Rad51ap1:NM_009013:exon5:c.G359A:p.G120D	**G120D**	ENSMUSP00000064413,120,G,D	6,126928188,C,C	6qF3	rs30556096	0.25	.	45	chr6	126928188	rs30556096	C	C	.	clustered_events;homologous_mapping_event	DB;ECNT=3;HCNT=2;MAX_ED=53;MIN_ED=2;NLOD=10.53;TLOD=31.00	GT:AD:AF:ALT_F1R2:ALT_F2R1:FOXOG:PGT:PID:QSS:REF_F1R2:REF_F2R1	0/1:2,9:0.818:3:6:0.333:0|1:126934806_A_C:56,251:2:0	0/0:37,0:0.00:0:0:.:0|1:126934806_A_C:1012,0:16:21	3	Tolerated	0	Neutral	63	22	0.208	Tolerated	3.07	49
T12,T1,T22,T2,T32,T3,T42,T4	chr6	**8**	**100**	126928206	126928206	T	C	exonic	**Rad51ap1**	ENSMUSP00000064413	.	nonsynonymous SNV	Rad51ap1:NM_001347455:exon5:c.A341G:p.E114G,Rad51ap1:NM_009013:exon5:c.A341G:p.E114G	**E114G**	ENSMUSP00000064413,114,E,G	6,126928206,T,G	6qF3	rs30704455	0.25	.	45	chr6	126928206	rs30704455	T	G	.	clustered_events;homologous_mapping_event	DB;ECNT=7;HCNT=32;MAX_ED=208;MIN_ED=71;NLOD=10.69;TLOD=67.13	GT:AD:AF:ALT_F1R2:ALT_F2R1:FOXOG:QSS:REF_F1R2:REF_F2R1	0/1:31,27:0.473:11:16:0.593:873,738:19:12	0/0:40,0:0.00:0:0:.:1059,0:22:18	3	Tolerated	0.52	Neutral	63	22	0.688	Tolerated	2.92	52
T12,T1,T22,T2,T32,T3,T42,T4	chr6	**8**	**100**	126934808	126934808	T	C	exonic	**Rad51ap1**	ENSMUSP00000064413	.	nonsynonymous SNV	Rad51ap1:NM_001347455:exon3:c.A115G:p.T39A,Rad51ap1:NM_009013:exon3:c.A115G:p.T39A	**T39A**	ENSMUSP00000064413,39,T,A	6,126934808,T,T	6qF3	rs45926821	0.25	.	37	chr6	126934808	rs45926821	T	T	.	str_contraction;t_lod_fstar	ECNT=1;HCNT=1;MAX_ED=.;MIN_ED=.;NLOD=9.00;RPA=7,6;RU=TGC;STR;TLOD=4.81	GT:AD:AF:ALT_F1R2:ALT_F2R1:FOXOG:QSS:REF_F1R2:REF_F2R1	0/1:45,2:0.043:0:2:.:1267,43:25:20	0/0:31,0:0.00:0:0:.:853,0:15:16	1	Tolerated	0	Neutral	63	22	1	Tolerated	2.84	54
T12,T1,T22,T2,T32,T3,T42,T4	chr6	**8**	**100**	118175843	118175843	T	C	exonic	**Ret**	ENSMUSP00000006171	.	nonsynonymous SNV	Ret:NM_001080780:exon8:c.A1534G:p.T512A,Ret:NM_009050:exon8:c.A1534G:p.T512A	**T512A**	ENSMUSP00000006171,512,T,A	6,118175843,T,A	6qF1	rs36505070	0.25	.	37	chr6	118175843	rs36505070	T	A	.	PASS	DB;ECNT=1;HCNT=2;MAX_ED=.;MIN_ED=.;NLOD=9.61;TLOD=93.40	GT:AD:AF:ALT_F1R2:ALT_F2R1:FOXOG:QSS:REF_F1R2:REF_F2R1	0/1:36,37:0.493:18:19:0.514:892,1039:19:17	0/0:36,0:0.00:0:0:.:872,0:16:20	1.5	Tolerated	0.31	Neutral	107	30	0.691	Tolerated	3	71
T12,T1,T22,T2,T32,T3,T42,T4	chr6	**8**	**100**	118176264	118176264	G	T	exonic	**Ret**	ENSMUSP00000006171	.	nonsynonymous SNV	Ret:NM_001080780:exon7:c.C1442A:p.T481N,Ret:NM_009050:exon7:c.C1442A:p.T481N	**T481N**	ENSMUSP00000006171,481,T,N	6,118176264,G,C	6qF1	rs225841295	0.25	.	39	chr6	118176264	rs225841295	G	C	.	clustered_events;homologous_mapping_event;t_lod_fstar	ECNT=6;HCNT=19;MAX_ED=45;MIN_ED=6;NLOD=9.93;TLOD=5.29	GT:AD:AF:ALT_F1R2:ALT_F2R1:FOXOG:PGT:PID:QSS:REF_F1R2:REF_F2R1	0/1:52,2:0.037:1:1:0.500:0|1:38088265_T_C:1487,59:27:25	0/0:36,0:0.00:0:0:.:0|1:38088265_T_C:972,0:21:15	0.5	Tolerated	-0.34	Neutral	107	30	0.37	Tolerated	2.9	79
T12,T1,T22,T2,T32,T3,T42,T4	chr6	**8**	**100**	118178614	118178614	C	A	exonic	**Ret**	ENSMUSP00000006171	.	nonsynonymous SNV	Ret:NM_001080780:exon6:c.G1120T:p.A374S,Ret:NM_009050:exon6:c.G1120T:p.A374S	**A374S**	ENSMUSP00000006171,374,A,S	6,118178614,C,T	6qF1	.	0.25	.	36	chr6	118178614	.	C	T	.	clustered_events;homologous_mapping_event;t_lod_fstar	ECNT=6;HCNT=19;MAX_ED=45;MIN_ED=6;NLOD=9.63;TLOD=5.25	GT:AD:AF:ALT_F1R2:ALT_F2R1:FOXOG:PGT:PID:QSS:REF_F1R2:REF_F2R1	0/1:53,2:0.036:1:1:0.500:0|1:38088265_T_C:1542,56:30:23	0/0:34,0:0.00:0:0:.:0|1:38088265_T_C:980,0:20:14	0.5	Tolerated	0.03	Neutral	107	30	0.585	Tolerated	2.94	76

T12,T1,T22,T2,T32,T3,T42,T4	chr6	**8**	**100**	128358145	128358145	G	A	exonic	**Rhno1**	ENSMUSP00000138433	.	nonsynonymous SNV	Rhno1:NM_001326583:exon3:c.C214T:p.R72W,Rhno1:NM_001326585:exon3:c.C214T:p.R72W,Rhno1:NM_001326586:exon4:c.C214T:p.R72W,Rhno1:NM_001326587:exon4:c.C214T:p.R72W	**R72W**	ENSMUSP00000138433,72,R,W	6,128358145,G,T	6qF3	rs13459788	0.25	.	22	chr6	128358145	.	G	T	.	alt_allele_in_normal;clustered_events	ECNT=3;HCNT=12;MAX_ED=72;MIN_ED=70;NLOD=3.34;TLOD=8.91	GT:AD:AF:ALT_F1R2:ALT_F2R1:FOXOG:PGT:PID:QSS:REF_F1R2:REF_F2R1	0/1:39,3:0.077:3:0:0.00:0|1:128359115_G_T:1013,87:18:21	0/0:41,2:0.049:1:1:0.500:0|1:128359115_G_T:997,56:19:22	3	Tolerated	0	Neutral	43	14	1	Tolerated	2.86	39
T12,T1,T22,T2,T32,T3,T42,T4	chr6	**8**	**100**	100232874	100232874	T	A	exonic	**Rybp**	ENSP00000419494	.	nonsynonymous SNV	Rybp:NM_019743:exon4:c.A427T:p.T143S	**T143S**	ENSP00000419494,143,T,S	6,100232874,T,A	6qD3	.	0.25	.	54	chr6	100232874	.	T	A	.	PASS	DB;ECNT=1;HCNT=1;MAX_ED=.;MIN_ED=.;NLOD=13.70;TLOD=29.80	GT:AD:AF:ALT_F1R2:ALT_F2R1:FOXOG:QSS:REF_F1R2:REF_F2R1	0/1:15,13:0.444:5:8:0.385:421,356:10:5	0/0:54,0:0.00:0:0:.:1445,0:17:37	0.5	Tolerated	-1.6	Neutral	128	30	0.635	Tolerated	3.46	58
T12,T1,T22,T2,T32,T3,T42,T4	chr6	**8**	**100**	113143653	113143653	T	A	exonic	**Setd5**	ENSMUSP00000068835	.	nonsynonymous SNV	Setd5:NM_173005:exon17:c.T2822A:p.F941Y,Setd5:NM_028385:exon18:c.T2765A:p.F922Y	**F922Y**	ENSMUSP00000068835,922,F,Y	6,113143653,T,G	6qE3	rs37126472	0.25	.	42	chr6	113143653	rs37126472	T	G	.	str_contraction;t_lod_fstar	ECNT=1;HCNT=1;MAX_ED=.;MIN_ED=.;NLOD=10.52;RPA=6,5;RU=GCA;STR;TLOD=5.42	GT:AD:AF:ALT_F1R2:ALT_F2R1:FOXOG:QSS:REF_F1R2:REF_F2R1	0/1:44,2:0.045:0:2:.:1252,59:22:22	0/0:38,0:0.00:0:0:.:1025,0:18:20	0.7	Damaging	-1.31	Neutral	134	30	0.001	Damaging	3.39	61
T12,T1,T22,T2,T32,T3,T42,T4	chr6	**8**	**100**	113143782	113143782	G	A	exonic	**Setd5**	ENSMUSP00000068835	.	nonsynonymous SNV	Setd5:NM_173005:exon17:c.G2951A:p.G984D,Setd5:NM_028385:exon18:c.G2894A:p.G965D	**G984D**	ENSMUSP00000068835,984,G,D	6,113143782,G,A	6qE3	rs36992713	0.25	.	49	chr6	113143782	rs36992713	G	A	.	PASS	DB;ECNT=1;HCNT=1;MAX_ED=.;MIN_ED=.;NLOD=12.58;TLOD=38.52	GT:AD:AF:ALT_F1R2:ALT_F2R1:FOXOG:QSS:REF_F1R2:REF_F2R1	0/1:23,15:0.395:8:7:0.533:611,424:11:12	0/0:49,0:0.00:0:0:.:1283,0:21:28	2	Damaging	0.44	Neutral	134	30	0.005	Damaging	3.47	58
T12,T1,T22,T2,T32,T3,T42,T4	chr6	**8**	**100**	113143653	113143653	T	A	exonic	**Setd5**	ENSMUSP00000068835	.	nonsynonymous SNV	Setd5:NM_173005:exon17:c.T2822A:p.F941Y,Setd5:NM_028385:exon18:c.T2765A:p.F922Y	**F941Y**	ENSMUSP00000068835,941,F,Y	6,113143653,T,A	6qE3	rs37126472	0.25	.	42	chr6	113143653	rs37126472	T	A	.	PASS	DB;ECNT=1;HCNT=1;MAX_ED=.;MIN_ED=.;NLOD=11.43;TLOD=25.91	GT:AD:AF:ALT_F1R2:ALT_F2R1:FOXOG:QSS:REF_F1R2:REF_F2R1	0/1:29,14:0.306:8:6:0.571:811,387:17:12	0/0:42,0:0.00:0:0:.:1171,0:23:19	2	Tolerated	-0.19	Neutral	134	30	1	Tolerated	3.39	61
T12,T1,T22,T2,T32,T3,T42,T4	chr6	**8**	**100**	113143782	113143782	G	A	exonic	**Setd5**	ENSMUSP00000068835	.	nonsynonymous SNV	Setd5:NM_173005:exon17:c.G2951A:p.G984D,Setd5:NM_028385:exon18:c.G2894A:p.G965D	**G965D**	ENSMUSP00000068835,965,G,D	6,113143782,G,T	6qE3	rs36992713	0.25	.	49	chr6	113143782	rs36992713	G	T	.	alt_allele_in_normal;clustered_events	ECNT=5;HCNT=19;MAX_ED=162;MIN_ED=16;NLOD=132.24;TLOD=145.04	GT:AD:AF:ALT_F1R2:ALT_F2R1:FOXOG:QSS:REF_F1R2:REF_F2R1	0/1:1672,97:0.053:47:50:0.515:47343,2750:855:817	0/0:1140,68:0.055:39:29:0.426:31847,1852:574:566	1	Tolerated	0.26	Neutral	134	30	0.186	Tolerated	3.47	58
T12,T1,T22,T2,T32,T3,T42,T4	chr11	**8**	**100**	3136008	3136008	G	A	exonic	**Sfi1**	ENSMUSP00000098766	.	nonsynonymous SNV	Sfi1:NM_030207:exon25:c.C2428T:p.R810W	**R810W**	ENSMUSP00000098766,810,R,W	11,3136008,G,T	11qA1	.	0.25	.	1436	chr11	3136008	.	G	T	.	alt_allele_in_normal	ECNT=1;HCNT=19;MAX_ED=.;MIN_ED=.;NLOD=244.11;TLOD=45.35	GT:AD:AF:ALT_F1R2:ALT_F2R1:FOXOG:QSS:REF_F1R2:REF_F2R1	0/1:1415,40:0.027:24:16:0.600:41496,1102:720:695	0/0:1347,45:0.032:22:23:0.489:38507,1250:668:679	1	Deleterious	-7.27	Deleterious	210	30	0	Damaging	2.92	224
T12,T1,T22,T2,T32,T3,T42,T4	chr11	**8**	**100**	3135767	3135767	C	A	exonic	**Sfi1**	ENSMUSP00000038485	.	nonsynonymous SNV	Sfi1:NM_030207:exon26:c.G2557T:p.A853S	**A853S**	ENSMUSP00000038485,853,A,S	11,3135767,C,T	11qA1	.	0.25	.	1234	chr11	3135767	.	C	T	.	alt_allele_in_normal;clustered_events	ECNT=2;HCNT=20;MAX_ED=110;MIN_ED=110;NLOD=81.14;TLOD=147.57	GT:AD:AF:ALT_F1R2:ALT_F2R1:FOXOG:QSS:REF_F1R2:REF_F2R1	0/1:1641,98:0.057:47:51:0.520:46779,2723:799:842	0/0:1254,100:0.074:50:50:0.500:36101,2737:615:639	1	Deleterious	-3.37	Deleterious	83	30	0	Damaging	2.9	69
T12,T1,T22,T2,T32,T3,T42,T4	chr11	**8**	**100**	3136953	3136953	C	T	exonic	**Sfi1**	ENSMUSP00000038485	.	nonsynonymous SNV	Sfi1:NM_030207:exon23:c.G2336A:p.R779K	**R779K**	ENSMUSP00000038485,779,R,K	11,3136953,C,A	11qA1	.	0.25	.	1354	chr11	3136953	.	C	A	.	alt_allele_in_normal	ECNT=1;HCNT=11;MAX_ED=.;MIN_ED=.;NLOD=289.25;TLOD=88.41	GT:AD:AF:ALT_F1R2:ALT_F2R1:FOXOG:QSS:REF_F1R2:REF_F2R1	0/1:1950,68:0.033:34:34:0.500:50114,1939:937:1013	0/0:1489,39:0.028:15:24:0.385:37529,1061:753:736	1	Deleterious	-5.85	Deleterious	83	30	0.001	Damaging	3.09	67
T12,T1,T22,T2,T32,T3,T42,T4	chr11	**8**	**100**	3142342	3142342	G	T	exonic	**Sfi1**	ENSMUSP00000038485	.	nonsynonymous SNV	Sfi1:NM_030207:exon21:c.C2051A:p.A684E	**A684E**	ENSMUSP00000038485,684,A,E	11,3142342,G,A	11qA1	.	0.25	.	1392	chr11	3142342	.	G	A	.	alt_allele_in_normal;clustered_events	ECNT=2;HCNT=14;MAX_ED=71;MIN_ED=71;NLOD=21.93;TLOD=185.12	GT:AD:AF:ALT_F1R2:ALT_F2R1:FOXOG:QSS:REF_F1R2:REF_F2R1	0/1:1739,118:0.062:59:59:0.500:43508,3377:862:877	0/0:1292,117:0.087:60:57:0.513:32080,3260:635:657	1	Deleterious	-2.57	Deleterious	83	30	0.027	Damaging	2.86	65

T12,T1,T22,T2,T32,T3,T42,T4	chr11	**8**	**100**	3160011	3160011	C	T	exonic	**Sfi1**	ENSMUSP00000038485	.	nonsynonymous SNV	Sfi1:NM_030207:exon10:c.G986A:p.R329Q	**R329Q**	ENSMUSP00000038485,329,R,Q	11,3160011,C,A	11qA1	.	0.25	.	1268	chr11	3160011	.	C	A	.	alt_allele_in_normal;clustered_events	ECNT=2;HCNT=22;MAX_ED=156;MIN_ED=156;NLOD=121.65;TLOD=60.74	GT:AD:AF:ALT_F1R2:ALT_F2R1:FOXOG:QSS:REF_F1R2:REF_F2R1	0/1:1908,49:0.025:24:25:0.490:54070,1390:986:922	0/0:1245,77:0.060:38:39:0.494:34893,2168:611:634	1	Deleterious	-4.03	Deleterious	83	30	0.003	Damaging	2.98	63
T12,T1,T22,T2,T32,T3,T42,T4	chr11	**8**	**100**	3177508	3177508	T	G	exonic	**Sfi1**	ENSMUSP00000038485	.	nonsynonymous SNV	Sfi1:NM_030207:exon6:c.A391C:p.N131H	**N131H**	ENSMUSP00000038485,131,N,H	11,3177508,T,A	11qA1	.	0.25	.	1522	chr11	3177508	.	T	A	.	alt_allele_in_normal;clustered_events	ECNT=7;HCNT=2;MAX_ED=59;MIN_ED=13;NLOD=572.76;TLOD=275.48	GT:AD:AF:ALT_F1R2:ALT_F2R1:FOXOG:QSS:REF_F1R2:REF_F2R1	0/1:2507,272:0.119:145:127:.:65621,7275:1229:1278	0/0:3371,186:0.067:88:98:.:90984,5101:1678:1693	1	Deleterious	-4	Deleterious	83	30	0.006	Damaging	3.05	58
T12,T1,T22,T2,T32,T3,T42,T4	chr11	**8**	**100**	3164673	3164673	C	T	exonic	**Sfi1**	ENSMUSP00000038485	.	nonsynonymous SNV	Sfi1:NM_030207:exon9:c.G734A:p.R245Q	**R245Q**	ENSMUSP00000038485,245,R,Q	11,3164673,C,G	11qA1	.	0.25	.	1358	chr11	3164673	.	C	G	.	alt_allele_in_normal;clustered_events	ECNT=2;HCNT=10;MAX_ED=58;MIN_ED=58;NLOD=270.88;TLOD=35.42	GT:AD:AF:ALT_F1R2:ALT_F2R1:FOXOG:QSS:REF_F1R2:REF_F2R1	0/1:1574,32:0.020:13:19:0.406:44074,904:786:788	0/0:1473,49:0.031:23:26:0.469:40619,1344:716:757	1	Deleterious	-2.83	Deleterious	83	30	0.058	Tolerated	3	61
T12,T1,T22,T2,T32,T3,T42,T4	chr11	**8**	**100**	3134341	3134341	G	A	exonic	**Sfi1**	ENSMUSP00000038485	.	nonsynonymous SNV	Sfi1:NM_030207:exon29:c.C3196T:p.H1066Y	**H1066Y**	ENSMUSP00000038485,1066,H,Y	11,3134341,G,A	11qA1	.	0.25	.	1130	chr11	3134341	.	G	A	.	alt_allele_in_normal;clustered_events	ECNT=2;HCNT=20;MAX_ED=241;MIN_ED=241;NLOD=116.88;TLOD=118.37	GT:AD:AF:ALT_F1R2:ALT_F2R1:FOXOG:QSS:REF_F1R2:REF_F2R1	0/1:1528,76:0.049:45:31:0.592:37974,2159:744:784	0/0:1346,90:0.063:50:40:0.556:32737,2519:683:663	1	Tolerated	-1.32	Neutral	83	30	0.556	Tolerated	2.95	64
T12,T1,T22,T2,T32,T3,T42,T4	chr11	**8**	**100**	3146172	3146172	G	A	exonic	**Sfi1**	ENSMUSP00000038485	.	nonsynonymous SNV	Sfi1:NM_030207:exon15:c.C1462T:p.R488C	**R488C**	ENSMUSP00000038485,488,R,C	11,3146172,G,T	11qA1	.	0.25	.	1528	chr11	3146172	.	G	T	.	alt_allele_in_normal	ECNT=1;HCNT=6;MAX_ED=.;MIN_ED=.;NLOD=89.47;TLOD=198.66	GT:AD:AF:ALT_F1R2:ALT_F2R1:FOXOG:QSS:REF_F1R2:REF_F2R1	0/1:1725,129:0.066:66:63:0.488:43005,3688:855:870	0/0:1179,89:0.069:39:50:0.562:28742,2490:529:650	1	Tolerated	-2.16	Neutral	83	30	0.062	Tolerated	2.96	67
T12,T1,T22,T2,T32,T3,T42,T4	chr11	**8**	**100**	3157883	3157883	G	A	exonic	**Sfi1**	ENSMUSP00000038485	.	nonsynonymous SNV	Sfi1:NM_030207:exon11:c.C1066T:p.R356C	**R356C**	ENSMUSP00000038485,356,R,C	11,3157883,G,T	11qA1	.	0.25	.	1409	chr11	3157883	.	G	T	.	alt_allele_in_normal;clustered_events	ECNT=2;HCNT=20;MAX_ED=64;MIN_ED=64;NLOD=249.60;TLOD=81.30	GT:AD:AF:ALT_F1R2:ALT_F2R1:FOXOG:QSS:REF_F1R2:REF_F2R1	0/1:1437,58:0.039:32:26:0.448:36493,1618:690:747	0/0:1315,43:0.030:22:21:0.488:33117,1202:658:657	1	Tolerated	-1.05	Neutral	83	30	0.062	Tolerated	2.99	63
T12,T1,T22,T2,T32,T3,T42,T4	chr11	**8**	**100**	3165620	3165620	G	A	exonic	**Sfi1**	ENSMUSP00000038485	.	nonsynonymous SNV	Sfi1:NM_030207:exon8:c.C649T:p.H217Y	**H217Y**	ENSMUSP00000038485,217,H,Y	11,3165620,G,A	11qA1	.	0.25	.	1322	chr11	3165620	.	G	A	.	alt_allele_in_normal	ECNT=1;HCNT=18;MAX_ED=.;MIN_ED=.;NLOD=227.55;TLOD=32.27	GT:AD:AF:ALT_F1R2:ALT_F2R1:FOXOG:QSS:REF_F1R2:REF_F2R1	0/1:1062,28:0.028:15:13:0.536:29904,763:489:573	0/0:1278,43:0.033:17:26:0.395:35059,1129:548:730	1	Tolerated	-1.51	Neutral	83	30	0.279	Tolerated	3	61
T12,T1,T22,T2,T32,T3,T42,T4	chr11	**8**	**100**	3187319	3187319	G	A	exonic	**Sfi1**	ENSMUSP00000038485	.	nonsynonymous SNV	Sfi1:NM_030207:exon3:c.C115T:p.P39S	**P39S**	ENSMUSP00000038485,39,P,S	11,3187319,G,C	11qA1	.	0.25	.	1321	chr11	3187319	.	G	C	.	PASS	ECNT=1;HCNT=2;MAX_ED=.;MIN_ED=.;NLOD=8.08;TLOD=12.82	GT:AD:AF:ALT_F1R2:ALT_F2R1:FOXOG:QSS:REF_F1R2:REF_F2R1	0/1:38,7:0.150:4:3:0.571:1070,203:22:16	0/0:31,0:0.00:0:0:.:860,0:11:20	0.2	Tolerated	-0.1	Neutral	83	30	0.073	Tolerated	3.56	44
T12,T1,T22,T2,T32,T3,T42,T4	chr11	**8**	**100**	3133099	3133099	C	T	exonic	**Sfi1**	ENSMUSP00000038485	.	nonsynonymous SNV	Sfi1:NM_030207:exon30:c.G3328A:p.E1110K	**E1110K**	ENSMUSP00000038485,1110,E,K	11,3133099,C,A	11qA1	.	0.25	.	1208	chr11	3133099	.	C	A	.	alt_allele_in_normal;clustered_events	ECNT=2;HCNT=21;MAX_ED=241;MIN_ED=241;NLOD=232.57;TLOD=84.06	GT:AD:AF:ALT_F1R2:ALT_F2R1:FOXOG:QSS:REF_F1R2:REF_F2R1	0/1:2152,69:0.031:33:36:0.522:60838,1902:1030:1122	0/0:1198,36:0.030:20:16:0.444:33141,978:597:601	1	Unknown	NA	NA	83	30	NA	NA	NA	NA
T12,T1,T22,T2,T32,T3,T42,T4	chr1	**8**	**100**	85596055	85596055	G	T	exonic	**Sp110**	ENSMUSP00000040248	.	stopgain	Sp110:NM_030194:exon2:c.C54A:p.Y18X,Sp110:NM_175397:exon3:c.C54A:p.Y18X	**Y18X**	ENSMUSP00000040248,18,Y,X	NA	1qC5	.	0.25	.	556	chr1	85596055	.	G	A	.	alt_allele_in_normal;clustered_events	ECNT=3;HCNT=10;MAX_ED=60;MIN_ED=52;NLOD=4.10;TLOD=13.91	GT:AD:AF:ALT_F1R2:ALT_F2R1:FOXOG:QSS:REF_F1R2:REF_F2R1	0/1:271,10:0.037:3:7:0.300:7430,280:135:136	0/0:344,36:0.085:16:20:0.444:9380,1029:171:173	0.3	Deletarious	.	.	.	.	.	.	.	.

T12,T1,T22,T2,T32,T3,T42,T4	chr1	**8**	**100**	85589118	85589118	C	G	exonic	**Sp110**	ENSMUSP00000040248	.	nonsynonymous SNV	Sp110:NM_030194:exon5:c.G657C:p.E219D,Sp110:NM_175397:exon6:c.G657C:p.E219D	**E219D**	ENSMUSP00000040248,219,E,D	1,85589118,C,T	1qC5	rs108286035	0.25	.	634	chr1	85589118	.	C	T	.	alt_allele_in_normal;clustered_events	ECNT=4;HCNT=2;MAX_ED=248;MIN_ED=196;NLOD=16.93;TLOD=97.41	GT:AD:AF:ALT_F1R2:ALT_F2R1:FOXOG:QSS:REF_F1R2:REF_F2R1	0/1:816,36:0.043:24:12:0.333:22779,1005:382:434	0/0:661,43:0.062:20:23:0.535:17952,1175:321:340	0.3	Tolerated	0	Neutral	104	30	1	Tolerated	2.9	54
T12,T1,T22,T2,T32,T3,T42,T4	chr1	**8**	**100**	85589173	85589173	T	G	exonic	**Sp110**	ENSMUSP00000040248	.	nonsynonymous SNV	Sp110:NM_030194:exon5:c.A602C:p.Q201P,Sp110:NM_175397:exon6:c.A602C:p.Q201P	**Q201P**	ENSMUSP00000040248,201,Q,P	1,85589173,T,T	1qC5	rs108283636	0.25	.	579	chr1	85589173	.	T	T	.	alt_allele_in_normal;clustered_events	ECNT=5;HCNT=2;MAX_ED=83;MIN_ED=14;NLOD=72.25;TLOD=121.58	GT:AD:AF:ALT_F1R2:ALT_F2R1:FOXOG:PGT:PID:QSS:REF_F1R2:REF_F2R1	0/1:813,44:0.053:22:22:0.500:0|1:85591668_C_A:22212,1215:405:408	0/0:743,32:0.046:16:16:0.500:0|1:85591668_C_A:19913,853:371:372	0.3	Tolerated	0	Neutral	104	30	1	Tolerated	2.84	59
T12,T1,T22,T2,T32,T3,T42,T4	chr1	**8**	**100**	85591568	85591568	C	T	exonic	**Sp110**	ENSMUSP00000040248	.	nonsynonymous SNV	Sp110:NM_030194:exon4:c.G538A:p.A180T,Sp110:NM_175397:exon5:c.G538A:p.A180T	**A180T**	ENSMUSP00000040248,180,A,T	1,85591568,C,G	1qC5	.	0.25	.	704	chr1	85591568	.	C	G	.	alt_allele_in_normal;clustered_events	ECNT=18;HCNT=6;MAX_ED=213;MIN_ED=3;NLOD=48.27;TLOD=113.27	GT:AD:AF:ALT_F1R2:ALT_F2R1:FOXOG:PGT:PID:QSS:REF_F1R2:REF_F2R1	0/1:724,40:0.058:20:20:0.500:0|1:85591774_T_G:20132,1170:381:343	0/0:676,36:0.053:17:19:0.472:0|1:85591774_T_G:18474,1005:336:340	0.3	Tolerated	-2.17	Neutral	104	30	0.075	Tolerated	2.9	57
T12,T1,T22,T2,T32,T3,T42,T4	chr1	**8**	**100**	85591682	85591682	G	T	exonic	**Sp110**	ENSMUSP00000040248	.	nonsynonymous SNV	Sp110:NM_030194:exon4:c.C424A:p.P142T,Sp110:NM_175397:exon5:c.C424A:p.P142T	**P142T**	ENSMUSP00000040248,142,P,T	1,85591682,G,T	1qC5	rs108261062	0.25	.	775	chr1	85591682	.	G	T	.	alt_allele_in_normal;clustered_events	ECNT=18;HCNT=3;MAX_ED=213;MIN_ED=3;NLOD=114.17;TLOD=22.38	GT:AD:AF:ALT_F1R2:ALT_F2R1:FOXOG:QSS:REF_F1R2:REF_F2R1	0/1:743,19:0.024:8:11:0.579:18592,534:391:352	0/0:678,30:0.041:20:10:0.333:16760,827:331:347	0.3	Tolerated	-1.72	Neutral	104	30	0.176	Tolerated	2.92	66
T12,T1,T22,T2,T32,T3,T42,T4	chr1	**8**	**100**	85591774	85591774	T	G	exonic	**Sp110**	ENSMUSP00000040248	.	nonsynonymous SNV	Sp110:NM_030194:exon4:c.A332C:p.E111A,Sp110:NM_175397:exon5:c.A332C:p.E111A	**E111A**	ENSMUSP00000040248,111,E,A	1,85591774,T,T	1qC5	.	0.25	.	712	chr1	85591774	.	T	T	.	alt_allele_in_normal;clustered_events	ECNT=18;HCNT=6;MAX_ED=213;MIN_ED=3;NLOD=54.94;TLOD=112.84	GT:AD:AF:ALT_F1R2:ALT_F2R1:FOXOG:PGT:PID:QSS:REF_F1R2:REF_F2R1	0/1:733,43:0.058:23:20:0.465:0|1:85591774_T_G:20027,1202:380:353	0/0:678,37:0.050:20:17:0.459:0|1:85591774_T_G:18121,1018:340:338	0.3	Tolerated	0	Neutral	104	30	1	Tolerated	2.89	74
T12,T1,T22,T2,T32,T3,T42,T4	chr1	**8**	**100**	85591775	85591775	C	T	exonic	**Sp110**	ENSMUSP00000040248	.	nonsynonymous SNV	Sp110:NM_030194:exon4:c.G331A:p.E111K,Sp110:NM_175397:exon5:c.G331A:p.E111K	**E111K**	ENSMUSP00000040248,111,E,K	1,85591775,C,C	1qC5	.	0.25	.	708	chr1	85591775	.	C	C	.	alt_allele_in_normal;clustered_events	ECNT=2;HCNT=11;MAX_ED=10;MIN_ED=10;NLOD=46.35;TLOD=42.88	GT:AD:AF:ALT_F1R2:ALT_F2R1:FOXOG:QSS:REF_F1R2:REF_F2R1	0/1:376,29:0.066:17:12:0.586:10357,825:184:192	0/0:493,34:0.067:14:20:0.412:13574,923:265:228	0.3	Tolerated	0	Neutral	104	30	1	Tolerated	2.89	74
T12,T1,T22,T2,T32,T3,T42,T4	chr1	**8**	**100**	85591786	85591786	C	T	exonic	**Sp110**	ENSMUSP00000040248	.	nonsynonymous SNV	Sp110:NM_030194:exon4:c.G320A:p.G107D,Sp110:NM_175397:exon5:c.G320A:p.G107D	**G107D**	ENSMUSP00000040248,107,G,D	1,85591786,C,C	1qC5	.	0.25	.	715	chr1	85591786	.	C	C	.	alt_allele_in_normal;clustered_events	ECNT=5;HCNT=1;MAX_ED=83;MIN_ED=14;NLOD=125.43;RPA=4,3;RU=G;STR;TLOD=19.49	GT:AD:AF:ALT_F1R2:ALT_F2R1:FOXOG:QSS:REF_F1R2:REF_F2R1	0/1:825,22:0.025:10:12:.:21076,513:417:408	0/0:734,26:0.037:13:13:.:18356,685:362:372	0.3	Tolerated	0	Neutral	104	30	0.71	Tolerated	2.88	77
T12,T1,T22,T2,T32,T3,T42,T4	chr1	**8**	**100**	85596045	85596045	T	C	exonic	**Sp110**	ENSMUSP00000040248	.	nonsynonymous SNV	Sp110:NM_030194:exon2:c.A64G:p.N22D,Sp110:NM_175397:exon3:c.A64G:p.N22D	**N22D**	ENSMUSP00000040248,22,N,D	1,85596045,T,T	1qC5	.	0.25	.	527	chr1	85596045	.	T	T	.	alt_allele_in_normal;clustered_events	ECNT=2;HCNT=10;MAX_ED=10;MIN_ED=10;NLOD=48.16;TLOD=34.14	GT:AD:AF:ALT_F1R2:ALT_F2R1:FOXOG:QSS:REF_F1R2:REF_F2R1	0/1:404,25:0.057:13:12:0.520:11580,657:200:204	0/0:525,31:0.057:15:16:0.484:14882,832:272:253	0.3	Tolerated	0	Neutral	104	30	0.178	Tolerated	2.81	106
T12,T1,T22,T2,T32,T3,T42,T4	chr1	**8**	**100**	85591685	85591685	G	-	exonic	**Sp110**	ENSMUSP00000040248	.	frameshift deletion	Sp110:NM_030194:exon4:c.421delC:p.R141fs,Sp110:NM_175397:exon5:c.421delC:p.R141fs	**R141fs**	ENSMUSP00000040248,141,R,.	1,85591685,CG,A	1qC5	.	0.25	.	760	chr1	85591684	.	CG	A	.	alt_allele_in_normal;clustered_events	ECNT=18;HCNT=3;MAX_ED=213;MIN_ED=3;NLOD=70.20;TLOD=48.58	GT:AD:AF:ALT_F1R2:ALT_F2R1:FOXOG:QSS:REF_F1R2:REF_F2R1	0/1:733,35:0.044:18:17:0.514:20065,996:378:355	0/0:673,42:0.060:12:30:0.286:18243,1163:347:326	0.3	Unknown	NA	NA	104	30	NA	NA	NA	NA
T12,T1,T22,T2,T32,T3,T42,T4	chr1	**8**	**100**	85610823	85610823	C	T	exonic	**Sp140**	ENSMUSP00000108782	.	nonsynonymous SNV	Sp140:NM_001013817:exon3:c.C280T:p.R94W	**R94W**	ENSMUSP00000108782,94,R,W	1,85610823,C,G	1qC5	rs108194004	0.25	.	359	chr1	85610823	.	C	G	.	alt_allele_in_normal;clustered_events	ECNT=11;HCNT=5;MAX_ED=129;MIN_ED=1;NLOD=52.82;RPA=3,2;RU=T;STR;TLOD=15.49	GT:AD:AF:ALT_F1R2:ALT_F2R1:FOXOG:PGT:PID:QSS:REF_F1R2:REF_F2R1	0/1:200,6:0.032:4:2:.:0|1:85610790_G_T:5688,173:100:100	0/0:363,10:0.027:5:5:.:0|1:85610790_G_T:10240,279:162:201	0.2	Deleterious	-2.55	Deleterious	93	30	0.119	Tolerated	2.86	66

T12,T1,T22,T2,T32,T3,T42,T4	chr1	**8**	**100**	85621680	85621680	C	A	exonic	**Sp140**	ENSMUSP00000109113	.	nonsynonymous SNV	Sp140:NM_001013817:exon7:c.C631A:p.P211T	**P211T**	ENSMUSP00000109113,211,P,T	1,85621680,C,A	1qC5	.	0.25	.	745	chr1	85621680	.	C	A	.	alt_allele_in_normal;clustered_events	ECNT=11;HCNT=1;MAX_ED=129;MIN_ED=1;NLOD=16.13;TLOD=10.09	GT:AD:AF:ALT_F1R2:ALT_F2R1:FOXOG:QSS:REF_F1R2:REF_F2R1	0/1:178,13:0.041:8:5:0.615:5114,363:91:87	0/0:316,26:0.074:14:12:0.538:8947,722:140:176	0.2	Tolerated	2.38	Neutral	93	30	1	Tolerated	3.12	56
T12,T1,T22,T2,T32,T3,T42,T4	chr1	**8**	**100**	85610817	85610817	T	-	exonic	**Sp140**	ENSMUSP00000108782	.	frameshift deletion	Sp140:NM_001013817:exon3:c.274delT:p.F92fs	**F92fs**	ENSMUSP00000108782,92,F,.	1,85610817,GT,A	1qC5	.	0.25	.	373	chr1	85610816	.	GT	A	.	alt_allele_in_normal;clustered_events	ECNT=11;HCNT=1;MAX_ED=129;MIN_ED=1;NLOD=27.10;TLOD=26.95	GT:AD:AF:ALT_F1R2:ALT_F2R1:FOXOG:PGT:PID:QSS:REF_F1R2:REF_F2R1	0/1:176,11:0.070:4:7:0.364:0|1:85610789_C_T:4526,322:89:87	0/0:320,20:0.067:11:9:0.550:0|1:85610789_C_T:8006,553:141:179	0.2	Unknown	NA	NA	93	30	NA	NA	NA	NA
T12,T1,T22,T2,T32,T3,T42,T4	chr6	**8**	**100**	125226785	125226785	T	C	exonic	**Tapbpl**	ENSMUSP00000088350	.	nonsynonymous SNV	Tapbpl:NM_145391:exon5:c.A931G:p.I311V	**I311V**	ENSMUSP00000088350,311,I,V	6,125226785,T,T	6qF2	rs30807042	0.25	.	41	chr6	125226785	rs30807042	T	T	.	PASS	DB;ECNT=1;HCNT=1;MAX_ED=.;MIN_ED=.;NLOD=11.13;TLOD=77.81	GT:AD:AF:ALT_F1R2:ALT_F2R1:FOXOG:QSS:REF_F1R2:REF_F2R1	0/1:36,33:0.462:20:13:0.394:1018,917:16:20	0/0:39,0:0.00:0:0:.:1042,0:19:20	2.8	Tolerated	0	Neutral	111	30	0.108	Tolerated	2.82	48
T12,T1,T22,T2,T32,T3,T42,T4	chr6	**8**	**100**	125228128	125228128	T	G	exonic	**Tapbpl**	ENSMUSP00000088350	.	nonsynonymous SNV	Tapbpl:NM_145391:exon4:c.A854C:p.N285T	**N285T**	ENSMUSP00000088350,285,N,T	6,125228128,T,T	6qF2	rs29873392	0.25	.	43	chr6	125228128	rs29873392	T	T	.	str_contraction;t_lod_fstar	ECNT=1;HCNT=2;MAX_ED=.;MIN_ED=.;NLOD=6.91;RPA=7,6;RU=ACC;STR;TLOD=5.54	GT:AD:AF:ALT_F1R2:ALT_F2R1:FOXOG:QSS:REF_F1R2:REF_F2R1	0/1:24,2:0.077:0:2:.:643,57:12:12	0/0:24,0:0.00:0:0:.:672,0:12:12	0.7	Tolerated	0	Neutral	111	30	0.083	Tolerated	2.82	48
T12,T1,T22,T2,T32,T3,T42,T4	chr6	**8**	**100**	125228311	125228311	C	T	exonic	**Tapbpl**	ENSMUSP00000088350	.	nonsynonymous SNV	Tapbpl:NM_145391:exon4:c.G671A:p.G224D	**G224D**	ENSMUSP00000088350,224,G,D	6,125228311,C,G	6qF2	rs30672494	0.25	.	39	chr6	125228311	rs30672494	C	G	.	PASS	ECNT=1;HCNT=1;MAX_ED=.;MIN_ED=.;NLOD=12.59;TLOD=6.94	GT:AD:AF:ALT_F1R2:ALT_F2R1:FOXOG:QSS:REF_F1R2:REF_F2R1	0/1:47,4:0.083:0:4:1.00:1331,110:24:23	0/0:46,0:0.00:0:0:.:1292,0:25:21	19	Tolerated	-1.7	Neutral	111	30	0.055	Tolerated	2.82	49
T12,T1,T22,T2,T32,T3,T42,T4	chr6	**8**	**100**	115742369	115742369	G	A	exonic	**Tmem40**	ENSP00000264728	.	nonsynonymous SNV	Tmem40:NM_001168256:exon2:c.C11T:p.S4L,Tmem40:NM_001168257:exon2:c.C11T:p.S4L,Tmem40:NM_144805:exon2:c.C11T:p.S4L	**S4L**	ENSP00000264728,4,S,L	6,115742369,G,A	6qE3	rs31498687	0.25	.	51	chr6	115742369	rs31498687	G	A	.	clustered_events	DB;ECNT=2;HCNT=2;MAX_ED=49;MIN_ED=49;NLOD=12.29;TLOD=53.24	GT:AD:AF:ALT_F1R2:ALT_F2R1:FOXOG:QSS:REF_F1R2:REF_F2R1	0/1:21,21:0.526:12:9:0.571:596,579:11:10	0/0:51,0:0.00:0:0:.:1455,0:24:27	1.3	Deleterious	-2.97	Deleterious	48	17	0.004	Damaging	3.94	33
T12,T1,T22,T2,T32,T3,T42,T4	chr6	**8**	**100**	115741647	115741647	A	T	exonic	**Tmem40**	ENSP00000264728	.	nonsynonymous SNV	Tmem40:NM_001168256:exon3:c.T96A:p.H32Q,Tmem40:NM_001168257:exon3:c.T96A:p.H32Q,Tmem40:NM_144805:exon3:c.T96A:p.H32Q	**H32Q**	ENSP00000264728,32,H,Q	6,115741647,A,T	6qE3	rs31498257	0.25	.	29	chr6	115741647	rs31498257	A	T	.	clustered_events;homologous_mapping_event	DB;ECNT=7;HCNT=2;MAX_ED=90;MIN_ED=3;NLOD=7.52;TLOD=69.76	GT:AD:AF:ALT_F1R2:ALT_F2R1:FOXOG:QSS:REF_F1R2:REF_F2R1	0/1:28,28:0.481:10:18:0.643:791,780:15:13	0/0:29,0:0.00:0:0:.:831,0:16:13	1.3	Tolerated	2.06	Neutral	48	17	1	Tolerated	3.99	32
T12,T1,T22,T2,T32,T3,T42,T4	chr6	**8**	**100**	115734822	115734822	T	C	exonic	**Tmem40**	ENSP00000264728	.	nonsynonymous SNV	Tmem40:NM_001168257:exon5:c.A280G:p.T94A,Tmem40:NM_001168256:exon6:c.A355G:p.T119A,Tmem40:NM_144805:exon6:c.A355G:p.T119A	**T119A**	ENSP00000264728,119,T,A	6,115734822,T,C	6qE3	rs31495179	0.25	.	36	chr6	115734822	rs31495179	T	C	.	PASS	DB;ECNT=1;HCNT=1;MAX_ED=.;MIN_ED=.;NLOD=9.83;TLOD=100.61	GT:AD:AF:ALT_F1R2:ALT_F2R1:FOXOG:QSS:REF_F1R2:REF_F2R1	0/1:28,37:0.574:21:16:0.568:801,1040:17:11	0/0:36,0:0.00:0:0:.:966,0:18:18	1.3	Tolerated	-0.16	Neutral	48	17	0.131	Tolerated	3.88	38
T12,T1,T22,T2,T32,T3,T42,T4	chr6	**8**	**100**	115734822	115734822	T	C	exonic	**Tmem40**	ENSP00000264728	.	nonsynonymous SNV	Tmem40:NM_001168257:exon5:c.A280G:p.T94A,Tmem40:NM_001168256:exon6:c.A355G:p.T119A,Tmem40:NM_144805:exon6:c.A355G:p.T119A	**T94A**	ENSP00000264728,94,T,A	6,115734822,T,C	6qE3	rs31495179	0.25	.	36	chr6	115734822	rs31495179	T	C	.	PASS	DB;ECNT=1;HCNT=1;MAX_ED=.;MIN_ED=.;NLOD=9.83;TLOD=100.61	GT:AD:AF:ALT_F1R2:ALT_F2R1:FOXOG:QSS:REF_F1R2:REF_F2R1	0/1:28,37:0.574:21:16:0.568:801,1040:17:11	0/0:36,0:0.00:0:0:.:966,0:18:18	1.3	Tolerated	-0.16	Neutral	48	17	0.131	Tolerated	3.88	38
T12,T1,T22,T2,T32,T3,T42,T4	chr6	**8**	**100**	129516660	129516660	G	C	exonic	**Tmem52b**	ENSMUSP00000032703	.	nonsynonymous SNV	Tmem52b:NM_001081186:exon4:c.G235C:p.G79R	**G79R**	ENSMUSP00000032703,79,G,R	NA	6qF3	rs50014977	0.25	.	55	chr6	129516660	rs50014977	G	T	.	homologous_mapping_event;t_lod_fstar	ECNT=3;HCNT=1;MAX_ED=2;MIN_ED=1;NLOD=9.63;TLOD=5.43	GT:AD:AF:ALT_F1R2:ALT_F2R1:FOXOG:PGT:PID:QSS:REF_F1R2:REF_F2R1	0/1:46,2:0.043:0:2:1.00:0|1:129516632_C_T:1341,56:23:23	0/0:36,0:0.00:0:0:.:0|1:129516632_C_T:1001,0:17:19	22	Deleterious	-7.2	Deleterious	107	30	0.001	Damaging	2.8	114

T12,T1,T22,T2,T32,T3,T42,T4	chr6	**8**	**100**	127965323	127965323	-	G	splicing	**Tspan9**	ENSMUSP00000003152	.	NM_001356301:exon7:c.565-2->C;NM_175414:exon8:c.565-2->C	.	**NA**	6,127965323,T,A	NA	6qF3	.	0.25	.	32	chr6	127965323	.	T	A	.	str_contraction;t_lod_fstar	ECNT=1;HCNT=1;MAX_ED=.;MIN_ED=.;NLOD=10.22;RPA=6,5;RU=CAG;STR;TLOD=5.62	GT:AD:AF:ALT_F1R2:ALT_F2R1:FOXOG:QSS:REF_F1R2:REF_F2R1	0/1:32,2:0.061:2:0:.:901,58:18:14	0/0:38,0:0.00:0:0:.:1071,0:19:19	0.9	Unkown	.	.	.	.	.	.	.	.
T12,T1,T22,T2,T32,T3,T42,T4	chr6	**8**	**100**	113405403	113405403	G	A	exonic	**Ttll3**	ENSMUSP00000064826	.	nonsynonymous SNV	Ttll3:NM_133923:exon9:c.G1409A:p.R470Q	**R470Q**	ENSMUSP00000064826,470,R,Q	6,113405403,G,A	6qE3	rs38208806	0.25	.	36	chr6	113405403	rs38208806	G	A	.	str_contraction;t_lod_fstar	ECNT=1;HCNT=1;MAX_ED=.;MIN_ED=.;NLOD=10.22;RPA=6,5;RU=CAG;STR;TLOD=5.62	GT:AD:AF:ALT_F1R2:ALT_F2R1:FOXOG:QSS:REF_F1R2:REF_F2R1	0/1:32,2:0.061:2:0:.:901,58:18:14	0/0:38,0:0.00:0:0:.:1071,0:19:19	0.9	Deleterious	-3.13	Deleterious	72	30	0.134	Tolerated	2.77	135
T12,T1,T22,T2,T32,T3,T42,T4	chr4	**8**	**100**	41195171	41195171	C	A	exonic	**Ubap2**	ENSMUSP00000047105	.	nonsynonymous SNV	Ubap2:NM_026872:exon29:c.G3379T:p.A1127S	**A1127S**	ENSMUSP00000047105,1127,A,S	4,41195171,C,CA	4qA5	rs28316596	0.25	.	38	chr4	41195171	rs28316596	C	CA	.	PASS	ECNT=1;HCNT=2;MAX_ED=.;MIN_ED=.;NLOD=8.12;RPA=3,4;RU=A;STR;TLOD=8.71	GT:AD:AF:ALT_F1R2:ALT_F2R1:FOXOG:QSS:REF_F1R2:REF_F2R1	0/1:54,6:0.102:3:3:.:1583,175:26:28	0/0:28,0:0.00:0:0:.:797,0:15:13	1.4	Unknown	NA	NA	169	30	NA	NA	NA	NA
T12,T1,T22,T2,T32,T3,T42,T4	chr12	**8**	**100**	69582737	69582737	T	C	exonic	**Vcpkmt**	ENSMUSP00000106586	.	nonsynonymous SNV	Vcpkmt:NM_001033236:exon1:c.A250G:p.M84V	**M84V**	ENSMUSP00000106586,84,M,V	12,69582737,T,C	12qC2	.	0.25	.	81	chr12	69582737	.	T	C	.	alt_allele_in_normal;clustered_events;germline_risk	ECNT=4;HCNT=4;MAX_ED=9;MIN_ED=2;NLOD=0.648;TLOD=15.06	GT:AD:AF:ALT_F1R2:ALT_F2R1:FOXOG:PGT:PID:QSS:REF_F1R2:REF_F2R1	0/1:56,5:0.085:3:2:0.400:0|1:93609809_G_A:1627,137:25:31	0/0:45,3:0.064:1:2:0.667:0|1:93609809_G_A:1237,68:19:26	1.9	Tolerated	-0.73	Neutral	66	30	0.784	Tolerated	2.81	48
T12,T1,T22,T2,T32,T3,T42,T4	chr6	**8**	**100**	125565870	125565870	C	T	exonic	**Vwf**	ENSMUSP00000072704	.	nonsynonymous SNV	Vwf:NM_011708:exon4:c.C308T:p.T103M	**T103M**	ENSMUSP00000072704,103,T,M	6,125565870,C,AGGATCAACT	6qF3	rs13479016	0.25	.	40	chr6	125565870	rs13479016	C	AGGATCAACT	.	t_lod_fstar	ECNT=1;HCNT=1;MAX_ED=.;MIN_ED=.;NLOD=20.05;RPA=1,2;RU=GGATCAACT;STR;TLOD=5.55	GT:AD:AF:ALT_F1R2:ALT_F2R1:FOXOG:QSS:REF_F1R2:REF_F2R1	0/1:40,1:0.050:0:1:.:1137,28:17:23	0/0:78,0:0.00:0:0:.:2153,0:40:38	1	Deleterious	-8.46	Deleterious	100	30	NA	NA	NA	NA
T12,T1,T22,T2,T32,T3,T42,T4	chr6	**8**	**100**	125566171	125566171	G	T	exonic	**Vwf**	ENSMUSP00000072704	.	nonsynonymous SNV	Vwf:NM_011708:exon5:c.G365T:p.R122L	**R122L**	ENSMUSP00000072704,122,R,L	6,125566171,G,T	6qF3	rs30268241	0.25	.	45	chr6	125566171	rs30268241	G	T	.	clustered_events;multi_event_alt_allele_in_normal	ECNT=5;HCNT=3;MAX_ED=26;MIN_ED=5;NLOD=5.38;TLOD=14.79	GT:AD:AF:ALT_F1R2:ALT_F2R1:FOXOG:QSS:REF_F1R2:REF_F2R1	0/1:63,5:0.075:0:5:1.00:1642,123:31:32	0/0:34,1:0.030:0:1:1.00:920,12:18:16	0.7	Deleterious	-2.82	Deleterious	100	30	0.377	Tolerated	2.84	90
T12,T1,T22,T2,T32,T3,T42,T4	chr6	**8**	**100**	116314763	116314763	A	G	exonic	**Zfand4**	ENSMUSP00000067353	.	nonsynonymous SNV	Zfand4:NM_001290339:exon4:c.A976G:p.I326V,Zfand4:NM_001290338:exon6:c.A1282G:p.I428V,Zfand4:NM_001309446:exon7:c.A1657G:p.I553V	**I428V**	ENSMUSP00000067353,428,I,V	6,116314763,A,T	6qE3	rs31539045	0.25	.	54	chr6	116314763	rs31539045	A	T	.	alt_allele_in_normal;clustered_events;germline_risk;str_contraction	ECNT=3;HCNT=2;MAX_ED=28;MIN_ED=16;NLOD=1.83;RPA=2,1;RU=CTC;STR;T	GT:AD:AF:ALT_F1R2:ALT_F2R1:FOXOG:PGT:PID:QSS:REF_F1R2:REF_F2R1	0/1:71,6:0.079:2:4:.:0|1:21396402_TCTC_T:1992,167:38:33	0/0:37,2:0.056:2:0:.:0|1:21396402_TCTC_T:1059,55:16:21	0.7	Damaging	-1.72	Neutral	80	30	0.037	Damaging	3.06	59
T12,T1,T22,T2,T32,T3,T42,T4	chr6	**8**	**100**	116314763	116314763	A	G	exonic	**Zfand4**	ENSMUSP00000067353	.	nonsynonymous SNV	Zfand4:NM_001290339:exon4:c.A976G:p.I326V,Zfand4:NM_001290338:exon6:c.A1282G:p.I428V,Zfand4:NM_001309446:exon7:c.A1657G:p.I553V	**I553V**	ENSMUSP00000067353,553,I,V	6,116314763,A,C	6qE3	rs31539045	0.25	.	54	chr6	116314763	rs31539045	A	C	.	alt_allele_in_normal;t_lod_fstar	ECNT=1;HCNT=1;MAX_ED=.;MIN_ED=.;NLOD=14.87;TLOD=	GT:AD:AF:ALT_F1R2:ALT_F2R1:FOXOG:QSS:REF_F1R2:REF_F2R1	0/1:101,5:0.048:1:4:.:2751,131:43:58	0/0:53,3:0.056:1:2:.:1426,87:25:28	0.7	Tolerated	-0.3	Neutral	80	30	0.137	Tolerated	3.06	59
T12,T1,T22,T2,T32,T3,T42,T4	chr6	**8**	**100**	116314763	116314763	A	G	exonic	**Zfand4**	ENSMUSP00000067353	.	nonsynonymous SNV	Zfand4:NM_001290339:exon4:c.A976G:p.I326V,Zfand4:NM_001290338:exon6:c.A1282G:p.I428V,Zfand4:NM_001309446:exon7:c.A1657G:p.I553V	**I326V**	ENSMUSP00000067353,326,I,V	6,116314763,A,G	6qE3	rs31539045	0.25	.	54	chr6	116314763	rs31539045	A	G	.	PASS	DB;ECNT=1;HCNT=1;MAX_ED=.;MIN_ED=.;NLOD=14.09;TLOD	GT:AD:AF:ALT_F1R2:ALT_F2R1:FOXOG:QSS:REF_F1R2:REF_F2R1	0/1:21,16:0.455:9:7:0.438:600,456:5:16	0/0:54,0:0.00:0:0:.:1511,0:33:21	1	Tolerated	0.26	Neutral	80	30	1	Tolerated	3.06	59
T12,T1,T22,T2,T3,T42,T4	chr19	**7**	**87.5**	3979259	3979259	A	G	exonic	**Aldh3b2**	ENSP00000255084	.	nonsynonymous SNV	Aldh3b2:NM_001177438:exon7:c.A631G:p.T211A	**T211A**	ENSP00000255084,211,T,A	19,3979259,A,G	19qA	.	0.25	.	41	chr19	3979259	.	A	G	.	germline_risk	ECNT=1;HCNT=1;MAX_ED=.;MIN_ED=.;NLOD=0.734;TLOD=7.40	GT:AD:AF:ALT_F1R2:ALT_F2R1:FOXOG:QSS:REF_F1R2:REF_F2R1	0/1:26,4:0.133:1:3:0.750:736,106:14:12	0/0:40,1:0.028:1:0:0.00:1109,27:27:13	6	Tolerated	-0.87	Neutral	216	30	0.969	Tolerated	2.8	248

T12, T1, T22, T2, T3, T42, T4	chr4	**7**	**87.5**	84292918	84292918	T	C	exonic	**Bnc2**	ENSMUSP00000135375	.	nonsynonymous SNV	Bnc2:NM_172870:exon5:c.A1213G:p.T405A	**T405A**	ENSMUSP00000135375,405,T,A	4,84292918,T,A	4qC4	.	0.25	.	39	chr4	84292918	.	T	C	.	PASS	ECNT=1;HCNT=1;MAX_ED=.;MIN_ED=.;NLOD=9.58;TLOD=8.42	GT:AD:AF:ALT_F1R2:ALT_F2R1:FOXOG:QSS:REF_F1R2:REF_F2R1	0/1:33,5:0.114:4:1:0.800:927,149:17:16	0/0:39,0:0.00:0:0:.:1097,0:16:23	2.1	Deleterious	-3.98	Deleterious	120	30	0.019	Damaging	2.87	168
T12,T1,T22,T2,T3,T42,T4	chr10	**7**	**87.5**	110764148	110764148	-	TTTTTTAAT	exonic	**E2f7**	ENSMUSP00000047881	.	stopgain	E2f7:NM_178609:exon5:c.763_764insTTTTTTAAT:p.R255delinsIFX	**R255delinsIFX**	ENSMUSP00000047881,255,R,IFX	NA	10qD1	.	0.25	.	32	chr10	110764148	.	A	A	.	alt_allele_in_normal;t_lod_fstar	ECNT=2;HCNT=2;MAX_ED=2;MIN_ED=2;NLOD=2.44;TLOD=5.41	GT:AD:AF:ALT_F1R2:ALT_F2R1:FOXOG:PGT:PID:QSS:REF_F1R2:REF_F2R1	0/1:46,2:0.043:1:1:0.500:0|1:78346447_T_C:1219,53:24:22	0/0:34,1:0.053:0:1:0.00:0|1:78346447_T_C:847,29:17:17	0.7	Deletarious	.	.	.	.	.	.	.	.
T12,T1,T22,T2,T3,T42,T4	chr10	**7**	**87.5**	110764154	110764154	G	T	exonic	**E2f7**	ENSMUSP00000047881	.	nonsynonymous SNV	E2f7:NM_178609:exon5:c.G769T:p.D257Y	**D257Y**	ENSMUSP00000047881,257,D,Y	10,110764154,G,T	10qD1	.	0.25	.	32	chr10	110764154	.	G	T	.	alt_allele_in_normal;clustered_events	ECNT=7;HCNT=3;MAX_ED=31;MIN_ED=1;NLOD=3.63;TLOD=8.94	GT:AD:AF:ALT_F1R2:ALT_F2R1:FOXOG:PGT:PID:QSS:REF_F1R2:REF_F2R1	0/1:35,3:0.079:3:0:0.00:0|1:110764148_A_ATTTTTTAAT:946,74:16:19	0/0:29,1:0.037:1:0:0.00:0|1:110764148_A_ATTTTTTAAT:805,28:15:14	1.4	Deleterious	-3.27	Deleterious	107	30	0.014	Damaging	2.81	61
T12,T1,T22,T2,T3,T42,T4	chr10	**7**	**87.5**	110764170	110764170	C	A	exonic	**E2f7**	ENSMUSP00000047881	.	nonsynonymous SNV	E2f7:NM_178609:exon5:c.C785A:p.P262H	**P262H**	ENSMUSP00000047881,262,P,H	10,110764170,C,G	10qD1	.	0.25	.	31	chr10	110764170	.	C	G	.	alt_allele_in_normal;clustered_events	ECNT=7;HCNT=3;MAX_ED=31;MIN_ED=1;NLOD=4.23;TLOD=8.87	GT:AD:AF:ALT_F1R2:ALT_F2R1:FOXOG:PGT:PID:QSS:REF_F1R2:REF_F2R1	0/1:36,3:0.075:3:0:.:0|1:110764148_A_ATTTTTTAAT:971,50:16:20	0/0:31,1:0.034:1:0:.:0|1:110764148_A_ATTTTTTAAT:842,27:17:14	1.4	Tolerated	0.04	Neutral	107	30	0.382	Tolerated	2.98	56
T12,T1,T22,T2,T3,T42,T4	chr10	**7**	**87.5**	110764179	110764179	C	T	exonic	**E2f7**	ENSMUSP00000047881	.	nonsynonymous SNV	E2f7:NM_178609:exon5:c.C794T:p.P265L	**P265L**	ENSMUSP00000047881,265,P,L	10,110764179,C,C	10qD1	.	0.25	.	30	chr10	110764179	.	C	C	.	alt_allele_in_normal;clustered_events	ECNT=7;HCNT=3;MAX_ED=31;MIN_ED=1;NLOD=4.53;TLOD=8.81	GT:AD:AF:ALT_F1R2:ALT_F2R1:FOXOG:PGT:PID:QSS:REF_F1R2:REF_F2R1	0/1:35,3:0.071:3:0:.:0|1:110764148_A_ATTTTTTAAT:950,61:16:19	0/0:30,1:0.033:1:0:.:0|1:110764148_A_ATTTTTTAAT:784,22:15:15	1.4	Tolerated	0	Neutral	107	30	0.189	Tolerated	2.94	60
T12,T1,T22,T2,T3,T42,T4	chr10	**7**	**87.5**	110764159	110764160	GT	-	exonic	**E2f7**	ENSMUSP00000047881	.	frameshift deletion	E2f7:NM_178609:exon5:c.774_775del:p.G258fs	**G258fs**	ENSMUSP00000047881,258,G,.	10,110764159,GGT,G	10qD1	.	0.25	.	32	chr10	110764158	.	GGT	G	.	alt_allele_in_normal;clustered_events	ECNT=7;HCNT=3;MAX_ED=31;MIN_ED=1;NLOD=4.83;TLOD=8.98	GT:AD:AF:ALT_F1R2:ALT_F2R1:FOXOG:PGT:PID:QSS:REF_F1R2:REF_F2R1	0/1:35,3:0.081:3:0:.:0|1:110764148_A_ATTTTTTAAT:972,84:14:21	0/0:34,1:0.032:1:0:.:0|1:110764148_A_ATTTTTTAAT:952,28:16:18	1.4	Unknown	NA	NA	107	30	NA	NA	NA	NA
T12,T1,T22,T2,T3,T42,T4	chr10	**7**	**87.5**	110764162	110764165	TCCG	-	exonic	**E2f7**	ENSMUSP00000047881	.	frameshift deletion	E2f7:NM_178609:exon5:c.777_780del:p.S259fs	**S259fs**	ENSMUSP00000047881,259,S,.	10,110764162,CTCCG,ATTTTTTAAT	10qD1	.	0.25	.	31	chr10	110764161	.	CTCCG	ATTTTTTAAT	.	clustered_events;multi_event_alt_allele_in_normal	ECNT=7;HCNT=3;MAX_ED=31;MIN_ED=1;NLOD=4.23;TLOD=8.98	GT:AD:AF:ALT_F1R2:ALT_F2R1:FOXOG:PGT:PID:QSS:REF_F1R2:REF_F2R1	0/1:35,3:0.081:3:0:.:0|1:110764148_A_ATTTTTTAAT:999,70:14:21	0/0:31,1:0.034:1:0:.:0|1:110764148_A_ATTTTTTAAT:878,11:13:18	1.4	Unknown	NA	NA	107	30	NA	NA	NA	NA
T12,T1,T22,T2,T3,T42,T4	chr10	**7**	**87.5**	110764150	110764152	GAA	-	exonic	**E2f7**	ENSMUSP00000047881	.	nonframeshift deletion	E2f7:NM_178609:exon5:c.765_767del:p.255_256del	**255_256del**	NA	10,110764150,GGAA,A	10qD1	.	0.25	.	35	chr10	110764149	.	GGAA	A	.	alt_allele_in_normal;clustered_events;germline_risk	DB;ECNT=2;HCNT=1;MAX_ED=10;MIN_ED=10;NLOD=2.37;TLOD=8.66	GT:AD:AF:ALT_F1R2:ALT_F2R1:FOXOG:PGT:PID:QSS:REF_F1R2:REF_F2R1	0/1:63,4:0.063:1:3:0.250:0|1:78349046_T_A:1779,109:34:29	0/0:51,4:0.080:3:1:0.750:0|1:78349046_T_A:1377,88:28:23	0.7	Unknown	.	.	.	.	.	.	.	.
T12,T1,T22,T2,T32,T3,T42	chr18	**7**	**87.5**	37423519	37423519	T	C	exonic	**Pcdhb11**	ENSMUSP00000125789	.	nonsynonymous SNV	Pcdhb11:NM_053136:exon1:c.T1901C:p.V634A	**V634A**	ENSMUSP00000125789,634,V,A	18,37423519,T,G	18qB3	.	0.25	.	66	chr18	37423519	.	T	G	.	clustered_events;homologous_mapping_event	ECNT=12;HCNT=8;MAX_ED=164;MIN_ED=9;NLOD=17.76;TLOD=8.22	GT:AD:AF:ALT_F1R2:ALT_F2R1:FOXOG:PGT:PID:QSS:REF_F1R2:REF_F2R1	0/1:61,3:0.046:2:1:0.667:0|1:37423559_C_A:1728,84:38:23	0/0:68,0:0.00:0:0:.:0|1:37423559_C_A:1915,0:27:41	0.8	Damaging	-1.24	Neutral	386	30	0.012	Damaging	3.35	392
T12,T1,T22,T2,T32,T3,T42	chr18	**7**	**87.5**	37423489	37423489	G	A	exonic	**Pcdhb11**	ENSMUSP00000125789	.	nonsynonymous SNV	Pcdhb11:NM_053136:exon1:c.G1871A:p.R624H	**R624H**	ENSMUSP00000125789,624,R,H	18,37423489,G,T	18qB3	.	0.25	.	67	chr18	37423489	.	G	T	.	clustered_events;homologous_mapping_event	ECNT=12;HCNT=8;MAX_ED=164;MIN_ED=9;NLOD=17.76;TLOD=10.12	GT:AD:AF:ALT_F1R2:ALT_F2R1:FOXOG:PGT:PID:QSS:REF_F1R2:REF_F2R1	0/1:67,3:0.057:2:1:0.333:0|1:37423559_C_A:1897,85:40:27	0/0:66,0:0.00:0:0:.:0|1:37423559_C_A:1848,0:26:40	0.8	Deleterious	-6.1	Deleterious	386	30	0	Damaging	3.35	392

T12,T1,T22,T2,T32,T3,T42	chr18	**7**	**87.5**	37423441	37423441	C	T	exonic	**Pcdhb11**	ENSMUSP00000125789	.	nonsynonymous SNV	Pcdhb11:NM_053136:exon1:c.C1823T:p.A608V	**A608V**	ENSMUSP00000125789,608,A,V	18,37423441,C,C	18qB3	.	0.25	.	65	chr18	37423441	.	C	C	.	alt_allele_in_normal;clustered_events	ECNT=12;HCNT=8;MAX_ED=164;MIN_ED=9;NLOD=12.10;TLOD=10.21	GT:AD:AF:ALT_F1R2:ALT_F2R1:FOXOG:QSS:REF_F1R2:REF_F2R1	0/1:71,8:0.104:6:2:0.750:2031,175:39:32	0/0:64,2:0.034:1:1:0.500:1830,44:28:36	0.8	Tolerated	0	Neutral	386	30	1	Tolerated	3.34	394
T12,T1,T2,T32,T3,T42,T4	chr18	**7**	**87.5**	37341997	37341997	C	T	exonic	**Pcdhb7**	ENSMUSP00000117225	.	nonsynonymous SNV	Pcdhb7:NM_053132:exon1:c.C185T:p.T62I	**T62I**	ENSMUSP00000117225,62,T,I	18,37341997,C,C	18qB3	.	0.25	.	72	chr18	37341997	.	C	C	.	alt_allele_in_normal;t_lod_fstar	ECNT=1;HCNT=1;MAX_ED=.;MIN_ED=.;NLOD=3.06;TLOD=5.10	GT:AD:AF:ALT_F1R2:ALT_F2R1:FOXOG:QSS:REF_F1R2:REF_F2R1	0/1:67,0:0.030:0:0:.:1829,0:34:33	0/0:57,3:0.055:2:1:0.333:1547,89:16:41	1	Tolerated	0	Neutral	388	30	0.665	Tolerated	3.35	290
T12,T1,T2,T32,T3,T42,T4	chr18	**7**	**87.5**	37342000	37342000	A	G	exonic	**Pcdhb7**	ENSMUSP00000117225	.	nonsynonymous SNV	Pcdhb7:NM_053132:exon1:c.A188G:p.Q63R	**Q63R**	ENSMUSP00000117225,63,Q,R	18,37342000,A,A	18qB3	.	0.25	.	72	chr18	37342000	.	A	A	.	str_contraction;t_lod_fstar	ECNT=1;HCNT=8;MAX_ED=.;MIN_ED=.;NLOD=8.42;RPA=7,6;RU=CAG;STR;TLOD=4.61	GT:AD:AF:ALT_F1R2:ALT_F2R1:FOXOG:QSS:REF_F1R2:REF_F2R1	0/1:59,2:0.033:0:2:.:1662,57:28:31	0/0:31,0:0.00:0:0:.:872,0:20:11	18	Tolerated	0	Neutral	388	30	0.245	Tolerated	3.33	357
T12,T1,T22,T2,T3,T42,T4	chr7	**7**	**87.5**	4632246	4632246	T	C	splicing	**Ppp6r1**	ENSMUSP00000030588	.	NM_172894:exon24:c.2568-2A>G	.	**NA**	7,4632246,T,T	NA	7qA1	.	0.25	.	35	chr7	4632246	.	T	T	.	str_contraction;t_lod_fstar	ECNT=1;HCNT=1;MAX_ED=.;MIN_ED=.;NLOD=8.71;RPA=4,3;RU=TGC;STR;TLOD=4.38	GT:AD:AF:ALT_F1R2:ALT_F2R1:FOXOG:QSS:REF_F1R2:REF_F2R1	0/1:34,3:0.059:0:3:.:958,85:17:17	0/0:33,0:0.00:0:0:.:912,0:19:14	1	Unkown	.	.	.	.	.	.	.	.
T1,T22,T2,T32,T3,T42,T4	chr1	**7**	**87.5**	85591643	85591643	T	A	exonic	**Sp110**	ENSMUSP00000040248	.	stopgain	Sp110:NM_030194:exon4:c.A463T:p.R155X,Sp110:NM_175397:exon5:c.A463T:p.R155X	**R155X**	ENSMUSP00000040248,155,R,X	NA	1qC5	.	0.25	.	699	chr1	85591643	.	T	G	.	alt_allele_in_normal;clustered_events	ECNT=7;HCNT=8;MAX_ED=262;MIN_ED=76;NLOD=74.06;TLOD=8.58	GT:AD:AF:ALT_F1R2:ALT_F2R1:FOXOG:QSS:REF_F1R2:REF_F2R1	0/1:469,12:0.027:9:3:0.250:12957,281:228:241	0/0:555,23:0.041:10:13:0.565:15270,564:295:260	0.3	Deletarious	.	.	.	.	.	.	.	.
T12,T1,T22,T2,T32,T3,T42	chr1	**7**	**87.5**	85610805	85610805	G	A	exonic	**Sp140**	ENSMUSP00000108782	.	nonsynonymous SNV	Sp140:NM_001013817:exon3:c.G262A:p.V88I	**V88I**	ENSMUSP00000108782,88,V,I	1,85610805,G,A	1qC5	rs45667597	0.25	.	388	chr1	85610805	.	G	A	.	alt_allele_in_normal;clustered_events	ECNT=6;HCNT=2;MAX_ED=207;MIN_ED=154;NLOD=96.14;TLOD=37.57	GT:AD:AF:ALT_F1R2:ALT_F2R1:FOXOG:QSS:REF_F1R2:REF_F2R1	0/1:600,26:0.044:14:12:0.462:16879,739:312:288	0/0:712,33:0.045:14:19:0.576:19595,925:355:357	0.2	Damaging	0.55	Neutral	93	30	0.006	Damaging	2.86	66
T12,T1,T22,T32,T3,T42,T4	chr1	**7**	**87.5**	85610828	85610828	T	A	exonic	**Sp140**	ENSMUSP00000108782	.	nonsynonymous SNV	Sp140:NM_001013817:exon3:c.T285A:p.D95E	**D95E**	ENSMUSP00000108782,95,D,E	1,85610828,T,C	1qC5	rs108696072	0.25	.	342	chr1	85610828	.	T	C	.	alt_allele_in_normal;clustered_events	ECNT=5;HCNT=5;MAX_ED=204;MIN_ED=35;NLOD=11.24;TLOD=17.38	GT:AD:AF:ALT_F1R2:ALT_F2R1:FOXOG:QSS:REF_F1R2:REF_F2R1	0/1:144,11:0.070:5:6:0.455:4071,294:73:71	0/0:234,19:0.076:8:11:0.421:6584,496:112:122	0.2	Tolerated	0	Neutral	93	30	0.209	Tolerated	2.86	66
T12,T1,T22,T2,T32,T42,T4	chr4	**7**	**87.5**	41195410	41195410	G	C	exonic	**Ubap2**	ENSMUSP00000047105	.	nonsynonymous SNV	Ubap2:NM_026872:exon28:c.C3227G:p.P1076R	**P1076R**	ENSMUSP00000047105,1076,P,R	4,41195410,G,T	4qA5	.	0.25	.	41	chr4	41195410	.	G	T	.	clustered_events	ECNT=2;HCNT=2;MAX_ED=146;MIN_ED=146;NLOD=19.09;TLOD=32.45	GT:AD:AF:ALT_F1R2:ALT_F2R1:FOXOG:QSS:REF_F1R2:REF_F2R1	0/1:70,13:0.128:7:6:.:1925,367:39:31	0/0:70,0:0.00:0:0:.:1941,0:40:30	1.4	Deleterious	-5.58	Deleterious	169	30	0.001	Damaging	2.83	139
T12,T1,T22,T2,T32,T3,T42	chr4	**7**	**87.5**	41199763	41199763	A	G	exonic	**Ubap2**	ENSMUSP00000047105	.	nonsynonymous SNV	Ubap2:NM_026872:exon20:c.T2366C:p.V789A	**V789A**	ENSMUSP00000047105,789,V,A	4,41199763,A,C	4qA5	.	0.25	.	44	chr4	41199763	.	A	C	.	PASS	ECNT=1;HCNT=4;MAX_ED=.;MIN_ED=.;NLOD=10.52;TLOD=16.53	GT:AD:AF:ALT_F1R2:ALT_F2R1:FOXOG:QSS:REF_F1R2:REF_F2R1	0/1:49,9:0.170:5:4:0.556:1216,227:26:23	0/0:41,0:0.00:0:0:.:1023,0:25:16	1.4	Tolerated	-0.2	Neutral	169	30	0.354	Tolerated	2.89	152
T12,T22,T2,T32,T3,T42,T4	chr17	**7**	**87.5**	33962974	33962974	T	G	exonic	**Vps52**	ENSMUSP00000121814	.	nonsynonymous SNV	Vps52:NM_001357329:exon16:c.T1310G:p.V437G,Vps52:NM_001357330:exon16:c.T929G:p.V310G,Vps52:NM_172620:exon16:c.T1685G:p.V562G	**V310G**	ENSMUSP00000121814,310,V,G	17,33962974,T,G	17qB1	.	0.25	.	42	chr17	33962974	.	T	G	.	clustered_events;multi_event_alt_allele_in_normal	ECNT=4;HCNT=28;MAX_ED=33;MIN_ED=8;NLOD=6.04;TLOD	GT:AD:AF:ALT_F1R2:ALT_F2R1:FOXOG:PGT:PID:QSS:REF_F1R	0/1:53,7:0.127:4:3:0.429:0|1:33962974_T_G:1506,188:25:28	0/0:40,1:0.029:0:1:1.00:0|1:33962974_T_G:1099,28:28:12	1	Deleterious	-6.87	Deleterious	108	30	0.001	Damaging	2.85	107
T12,T22,T2,T32,T3,T42,T4	chr17	**7**	**87.5**	33962974	33962974	T	G	exonic	**Vps52**	ENSMUSP00000121814	.	nonsynonymous SNV	Vps52:NM_001357329:exon16:c.T1310G:p.V437G,Vps52:NM_001357330:exon16:c.T929G:p.V310G,Vps52:NM_172620:exon16:c.T1685G:p.V562G	**V562G**	ENSMUSP00000121814,562,V,G	17,33962974,T,G	17qB1	.	0.25	.	42	chr17	33962974	.	T	G	.	clustered_events;multi_event_alt_allele_in_normal	ECNT=4;HCNT=28;MAX_ED=33;MIN_ED=8;NLOD=6.04;TLOD=21.07	GT:AD:AF:ALT_F1R2:ALT_F2R1:FOXOG:PGT:PID:QSS:REF_F1R2:REF_F2R1	0/1:53,7:0.127:4:3:0.429:0|1:33962974_T_G:1506,188:25:28	0/0:40,1:0.029:0:1:1.00:0|1:33962974_T_G:1099,28:28:12	1	Deleterious	-6.87	Deleterious	108	30	0.001	Damaging	2.85	107

T12,T22,T2,T32,T3,T42,T4	chr17	**7**	**87.5**	33962982	33962982	A	G	exonic	**Vps52**	ENSMUSP00000121814	.	nonsynonymous SNV	Vps52:NM_001357329:exon16:c.A1318G:p.I440V,Vps52:NM_001357330:exon16:c.A937G:p.I313V,Vps52:NM_172620:exon16:c.A1693G:p.I565V	**I313V**	ENSMUSP00000121814,313,I,V	17,33962982,A,T	17qB1	.	0.25	.	41	chr17	33962982	.	A	T	.	alt_allele_in_normal;clustered_events	ECNT=4;HCNT=28;MAX_ED=33;MIN_ED=8;NLOD=5.13;TLOD	GT:AD:AF:ALT_F1R2:ALT_F2R1:FOXOG:PGT:PID:QSS:REF_F1R	0/1:53,7:0.123:4:3:0.571:0|1:33963006_G_T:1319,198:24:29	0/0:37,1:0.031:0:1:0.00:0|1:33963006_G_T:934,25:21:16	1	Deleterious	-3.93	Deleterious	108	30	0	Damaging	2.85	107
T12,T22,T2,T32,T3,T42,T4	chr17	**7**	**87.5**	33962982	33962982	A	G	exonic	**Vps52**	ENSMUSP00000121814	.	nonsynonymous SNV	Vps52:NM_001357329:exon16:c.A1318G:p.I440V,Vps52:NM_001357330:exon16:c.A937G:p.I313V,Vps52:NM_172620:exon16:c.A1693G:p.I565V	**I440V**	ENSMUSP00000121814,440,I,V	17,33962982,A,T	17qB1	.	0.25	.	41	chr17	33962982	.	A	T	.	alt_allele_in_normal;clustered_events	ECNT=4;HCNT=28;MAX_ED=33;MIN_ED=8;NLOD=5.13;TLOD	GT:AD:AF:ALT_F1R2:ALT_F2R1:FOXOG:PGT:PID:QSS:REF_F1R	0/1:53,7:0.123:4:3:0.571:0|1:33963006_G_T:1448,192:24:29	0/0:38,1:0.031:0:1:0.00:0|1:33963006_G_T:1034,25:21:17	1	Deleterious	-3.93	Deleterious	108	30	0	Damaging	2.85	107
T12,T22,T2,T32,T3,T42,T4	chr17	**7**	**87.5**	33962982	33962982	A	G	exonic	**Vps52**	ENSMUSP00000121814	.	nonsynonymous SNV	Vps52:NM_001357329:exon16:c.A1318G:p.I440V,Vps52:NM_001357330:exon16:c.A937G:p.I313V,Vps52:NM_172620:exon16:c.A1693G:p.I565V	**I565V**	ENSMUSP00000121814,565,I,V	17,33962982,A,T	17qB1	.	0.25	.	41	chr17	33962982	.	A	T	.	alt_allele_in_normal;clustered_events	ECNT=4;HCNT=28;MAX_ED=33;MIN_ED=8;NLOD=5.13;TLOD	GT:AD:AF:ALT_F1R2:ALT_F2R1:FOXOG:PGT:PID:QSS:REF_F1R	0/1:53,7:0.123:4:3:0.571:0|1:33963006_G_T:1319,198:24:29	0/0:37,1:0.031:0:1:0.00:0|1:33963006_G_T:934,25:21:16	1	Deleterious	-3.93	Deleterious	108	30	0	Damaging	2.85	107
T12,T22,T2,T32,T3,T42,T4	chr17	**7**	**87.5**	33963006	33963006	G	T	exonic	**Vps52**	ENSMUSP00000121814	.	nonsynonymous SNV	Vps52:NM_001357329:exon16:c.G1342T:p.G448W,Vps52:NM_001357330:exon16:c.G961T:p.G321W,Vps52:NM_172620:exon16:c.G1717T:p.G573W	**G321W**	ENSMUSP00000121814,321,G,W	17,33963006,G,T	17qB1	.	0.25	.	38	chr17	33963006	.	G	T	.	alt_allele_in_normal;clustered_events	ECNT=4;HCNT=28;MAX_ED=33;MIN_ED=8;NLOD=5.13;TLOD	GT:AD:AF:ALT_F1R2:ALT_F2R1:FOXOG:PGT:PID:QSS:REF_F1R	0/1:53,7:0.123:4:3:0.571:0|1:33963006_G_T:1448,192:24:29	0/0:38,1:0.031:0:1:0.00:0|1:33963006_G_T:1034,25:21:17	1	Deleterious	-4.72	Deleterious	108	30	0.002	Damaging	2.85	107
T12,T22,T2,T32,T3,T42,T4	chr17	**7**	**87.5**	33963006	33963006	G	T	exonic	**Vps52**	ENSMUSP00000121814	.	nonsynonymous SNV	Vps52:NM_001357329:exon16:c.G1342T:p.G448W,Vps52:NM_001357330:exon16:c.G961T:p.G321W,Vps52:NM_172620:exon16:c.G1717T:p.G573W	**G573W**	ENSMUSP00000121814,573,G,W	17,33963006,G,T	17qB1	.	0.25	.	38	chr17	33963006	.	G	T	.	alt_allele_in_normal;clustered_events	ECNT=4;HCNT=28;MAX_ED=33;MIN_ED=8;NLOD=5.13;TLOD	GT:AD:AF:ALT_F1R2:ALT_F2R1:FOXOG:PGT:PID:QSS:REF_F1R	0/1:53,7:0.123:4:3:0.571:0|1:33963006_G_T:1448,192:24:29	0/0:38,1:0.031:0:1:0.00:0|1:33963006_G_T:1034,25:21:17	1	Deleterious	-4.72	Deleterious	108	30	0.002	Damaging	2.85	107
T12,T22,T2,T32,T3,T42,T4	chr17	**7**	**87.5**	33962974	33962974	T	G	exonic	**Vps52**	ENSMUSP00000121814	.	nonsynonymous SNV	Vps52:NM_001357329:exon16:c.T1310G:p.V437G,Vps52:NM_001357330:exon16:c.T929G:p.V310G,Vps52:NM_172620:exon16:c.T1685G:p.V562G	**V437G**	ENSMUSP00000121814,437,V,G	17,33962974,T,T	17qB1	.	0.25	.	42	chr17	33962974	.	T	T	.	alt_allele_in_normal;clustered_events	ECNT=4;HCNT=28;MAX_ED=33;MIN_ED=8;NLOD=5.13;TLOD	GT:AD:AF:ALT_F1R2:ALT_F2R1:FOXOG:PGT:PID:QSS:REF_F1R	0/1:53,7:0.123:4:3:0.571:0|1:33963006_G_T:1319,198:24:29	0/0:37,1:0.031:0:1:0.00:0|1:33963006_G_T:934,25:21:16	1	Tolerated	0	Neutral	108	30	1	Tolerated	2.85	107
T12,T22,T2,T32,T3,T42,T4	chr17	**7**	**87.5**	33963006	33963006	G	T	exonic	**Vps52**	ENSMUSP00000121814	.	nonsynonymous SNV	Vps52:NM_001357329:exon16:c.G1342T:p.G448W,Vps52:NM_001357330:exon16:c.G961T:p.G321W,Vps52:NM_172620:exon16:c.G1717T:p.G573W	**G448W**	ENSMUSP00000121814,448,G,W	17,33963006,G,G	17qB1	.	0.25	.	38	chr17	33963006	.	G	G	.	clustered_events;multi_event_alt_allele_in_normal	ECNT=4;HCNT=28;MAX_ED=33;MIN_ED=8;NLOD=6.64;TLOD	GT:AD:AF:ALT_F1R2:ALT_F2R1:FOXOG:PGT:PID:QSS:REF_F1R	0/1:51,6:0.130:3:3:0.500:0|1:33962974_T_G:1447,161:26:25	0/0:41,1:0.027:0:1:0.00:0|1:33962974_T_G:1157,20:28:13	1	Tolerated	0	Neutral	108	30	1	Tolerated	2.85	107
T12,T22,T2,T32,T3,T42,T4	chr17	**7**	**87.5**	33963007	33963007	G	T	exonic	**Vps52**	ENSMUSP00000121814	.	nonsynonymous SNV	Vps52:NM_001357329:exon16:c.G1343T:p.G448V,Vps52:NM_001357330:exon16:c.G962T:p.G321V,Vps52:NM_172620:exon16:c.G1718T:p.G573V	**G321V**	ENSMUSP00000121814,321,G,V	17,33963007,G,C	17qB1	.	0.25	.	39	chr17	33963007	.	G	C	.	clustered_events;homologous_mapping_event;t_lod_fstar	ECNT=5;HCNT=8;MAX_ED=88;MIN_ED=55;NLOD=10.23;TLOD=4.72	GT:AD:AF:ALT_F1R2:ALT_F2R1:FOXOG:QSS:REF_F1R2:REF_F2R1	0/1:51,4:0.073:2:2:0.500:1416,116:32:19	0/0:37,0:0.00:0:0:.:1030,0:22:15	1	Tolerated	-1.91	Neutral	108	30	0.097	Tolerated	2.85	107
T12,T22,T2,T32,T3,T42,T4	chr17	**7**	**87.5**	33963007	33963007	G	T	exonic	**Vps52**	ENSMUSP00000121814	.	nonsynonymous SNV	Vps52:NM_001357329:exon16:c.G1343T:p.G448V,Vps52:NM_001357330:exon16:c.G962T:p.G321V,Vps52:NM_172620:exon16:c.G1718T:p.G573V	**G573V**	ENSMUSP00000121814,573,G,V	17,33963007,G,C	17qB1	.	0.25	.	39	chr17	33963007	.	G	C	.	clustered_events;homologous_mapping_event;t_lod_fstar	ECNT=5;HCNT=8;MAX_ED=88;MIN_ED=55;NLOD=10.23;TLOD=4.72	GT:AD:AF:ALT_F1R2:ALT_F2R1:FOXOG:QSS:REF_F1R2:REF_F2R1	0/1:51,4:0.073:2:2:0.500:1416,116:32:19	0/0:37,0:0.00:0:0:.:1030,0:22:15	1	Tolerated	-1.91	Neutral	108	30	0.097	Tolerated	2.85	107
T12,T22,T2,T32,T3,T42,T4	chr17	**7**	**87.5**	33963007	33963007	G	T	exonic	**Vps52**	ENSMUSP00000121814	.	nonsynonymous SNV	Vps52:NM_001357329:exon16:c.G1343T:p.G448V,Vps52:NM_001357330:exon16:c.G962T:p.G321V,Vps52:NM_172620:exon16:c.G1718T:p.G573V	**G448V**	ENSMUSP00000121814,448,G,V	17,33963007,G,G	17qB1	.	0.25	.	39	chr17	33963007	.	G	G	.	clustered_events;multi_event_alt_allele_in_normal	ECNT=4;HCNT=28;MAX_ED=33;MIN_ED=8;NLOD=6.64;TLOD	GT:AD:AF:ALT_F1R2:ALT_F2R1:FOXOG:PGT:PID:QSS:REF_F1R	0/1:51,6:0.130:3:3:0.500:0|1:33962974_T_G:1447,161:26:25	0/0:41,1:0.027:0:1:0.00:0|1:33962974_T_G:1157,20:28:13	1	Tolerated	0	Neutral	108	30	1	Tolerated	2.85	107
T1,T22,T2,T32,T3,T42,T4	chr13	**7**	**87.5**	21396403	21396405	CTC	-	exonic	**Zkscan3**	ENSMUSP00000022057	.	nonframeshift deletion	Zkscan3:NM_001145778:exon2:c.112_114del:p.38_38del,Zkscan3:NM_023685:exon2:c.112_114del:p.38_38del	**38_38del**	NA	13,21396403,TCTC,C	13qA3.1	.	0.25	.	39	chr13	21396402	.	TCTC	C	.	alt_allele_in_normal;t_lod_fstar	ECNT=1;HCNT=1;MAX_ED=.;MIN_ED=.;NLOD=14.8	GT:AD:AF:ALT_F1R2:ALT_F2R1:FOXOG:QSS:REF_F1R	0/1:101,5:0.048:1:4:.:2751,131:43:58	0/0:53,3:0.056:1:2:.:1426,87:25:28	0.7	Unknown	.	.	.	.	.	.	.	.
T12,T22,T2,T32,T3,T42	chr5	**6**	**75**	21661394	21661394	G	A	exonic	**Armc10**	ENSMUSP00000135168	.	nonsynonymous SNV	Armc10:NM_026034:exon6:c.G697A:p.G233S	**G233S**	ENSMUSP00000135168,233,G,S	5,21661394,G,C	5qA3	.	0.25	.	49	chr5	21661394	.	G	C	.	t_lod_fstar	ECNT=1;HCNT=1;MAX_ED=.;MIN_ED=.;NLOD=10.78;TLOD=4.91	GT:AD:AF:ALT_F1R2:ALT_F2R1:FOXOG:QSS:REF_F1R2:REF_F2R1	0/1:30,4:0.118:2:2:0.500:857,82:14:16	0/0:40,0:0.00:0:0:.:1123,0:15:25	1	Tolerated	-1.78	Neutral	173	30	0.077	Tolerated	2.74	201
T12, T1, T22, T2, T3, T4	chr1	**6**	**75**	140108617	140108617	C	A	exonic	**Cfh**	ENSMUSP00000125668	.	nonsynonymous SNV	Cfh:NM_009888:exon14:c.G2162T:p.C721F	**C721F**	ENSMUSP00000125668,721,C,F	1,140108617,C,A	1qF	rs31728142	0.25	.	36	chr1	140108617	.	C	A	.	clustered_events	ECNT=2;HCNT=1;MAX_ED=9;MIN_ED=9;NLOD=8.73;TLOD=69.84	GT:AD:AF:ALT_F1R2:ALT_F2R1:FOXOG:PGT:PID:QSS:REF_F1R2:REF_F2R1	0/1:31,26:0.450:10:11:0.524:0|1:125122132_C_CTCCCTG:616,600:13:11	0/0:31,0:0.00:0:0:.:0|1:125122132_C_CTCCCTG:664,0:14:10	3	Tolerated	1.08	Neutral	79	30	0.71	Tolerated	2.93	57

T12,T1,T2,T3,T42,T4	chr6	**6**	**75**	129184174	129184174	C	A	exonic	**Clec2d**	ENSMUSP00000143151	.	nonsynonymous SNV	Clec2d:NM_053109:exon3:c.C233A:p.A78D	**A78D**	ENSMUSP00000143151,78,A,D	6,129184174,C,A	6qF3	.	0.25	.	34	chr6	129184174	.	C	A	.	clustered_events;homologous_mapping_event	ECNT=22;HCNT=32;MAX_ED=181;MIN_ED=8;NLOD=9.93;TLOD=8.66	GT:AD:AF:ALT_F1R2:ALT_F2R1:FOXOG:PGT:PID:QSS:REF_F1R2:REF_F2R1	0/1:43,3:0.064:2:1:0.333:0|1:129184154_CCCAG_C:1247,85:19:24	0/0:34,0:0.00:0:0:.:0|1:129184154_CCCAG_C:976,0:20:14	2.1	Deleterious	-4.2	Deleterious	104	30	0.101	Tolerated	2.79	86
T12,T1,T2,T3,T42,T4	chr6	**6**	**75**	129184165	129184165	-	AAC	exonic	**Clec2d**	ENSMUSP00000143151	.	nonframeshift insertion	Clec2d:NM_053109:exon3:c.224_225insAAC:p.K75delinsKT	**K75delinsKT**	NA	6,129184165,A,AAAC	6qF3	.	0.25	.	37	chr6	129184165	.	A	AAAC	.	clustered_events;homologous_mapping_event;t_lod_fstar	ECNT=22;HCNT=32;MAX_ED=181;MIN_ED=8;NLOD=10.54;TLOD=5.45	GT:AD:AF:ALT_F1R2:ALT_F2R1:FOXOG:PGT:PID:QSS:REF_F1R2:REF_F2R1	0/1:44,2:0.044:1:1:.:0|1:129184154_CCCAG_C:1223,55:21:23	0/0:37,0:0.00:0:0:.:0|1:129184154_CCCAG_C:1003,0:23:14	2.1	Tolerated	-2.22	Neutral	104	30	NA	NA	NA	NA
T12,T1,T2,T3,T42,T4	chr6	**6**	**75**	129184166	129184166	G	T	exonic	**Clec2d**	ENSMUSP00000143151	.	nonsynonymous SNV	Clec2d:NM_053109:exon3:c.G225T:p.K75N	**K75N**	ENSMUSP00000143151,75,K,N	6,129184166,G,T	6qF3	.	0.25	.	36	chr6	129184166	.	G	T	.	clustered_events;homologous_mapping_event;t_lod_fstar	DB;ECNT=22;HCNT=32;MAX_ED=181;MIN_ED=8;NLOD=10.54;TLOD=5.47	GT:AD:AF:ALT_F1R2:ALT_F2R1:FOXOG:PGT:PID:QSS:REF_F1R2:REF_F2R1	0/1:44,2:0.045:1:1:0.500:0|1:129184154_CCCAG_C:1249,57:21:23	0/0:36,0:0.00:0:0:.:0|1:129184154_CCCAG_C:999,0:23:13	2.1	Tolerated	0.38	Neutral	104	30	0.6	Tolerated	2.78	81
T12, T1, T2,T3,T42, T4	chr6	**6**	**75**	129184162	129184162	-	C	exonic	**Clec2d**	ENSMUSP00000143151	.	frameshift insertion	Clec2d:NM_053109:exon3:c.222dupC:p.N74fs	**N74fs**	ENSMUSP00000143151,74,N,	6,129184162,A,A	6qF3	.	0.25	.	38	chr6	129184162	.	A	A	.	clustered_events;homologous_mapping_event	ECNT=22;HCNT=32;MAX_ED=181;MIN_ED=8;NLOD=9.93;TLOD=8.66	GT:AD:AF:ALT_F1R2:ALT_F2R1:FOXOG:PGT:PID:QSS:REF_F1R2:REF_F2R1	0/1:43,3:0.064:2:1:0.333:0|1:129184154_CCCAG_C:1247,85:19:24	0/0:34,0:0.00:0:0:.:0|1:129184154_CCCAG_C:976,0:20:14	2.1	Tolerated	0	Neutral	104	30	0.509	Tolerated	2.79	82
T12, T1, T2, T3, T42, T4	chr6	**6**	**75**	129184155	129184158	CCAG	-	exonic	**Clec2d**	ENSMUSP00000143151	.	frameshift deletion	Clec2d:NM_053109:exon3:c.214_217del:p.P72fs	**P72fs**	NA	6,129184154,CCCAG,C	6qF3	.	0.25	.	41	chr6	129184154	.	CCCAG	C	.	clustered_events;homologous_mapping_event;t_lod_fstar	ECNT=22;HCNT=32;MAX_ED=181;MIN_ED=8;NLOD=11.14;TLOD=5.41	GT:AD:AF:ALT_F1R2:ALT_F2R1:FOXOG:PGT:PID:QSS:REF_F1R2:REF_F2R1	0/1:47,2:0.043:1:1:.:0|1:129184154_CCCAG_C:1307,56:24:23	0/0:41,0:0.00:0:0:.:0|1:129184154_CCCAG_C:1121,0:25:16	2.1	Unknown	NA	NA	104	30	NA	NA	NA	NA
T12,T1,T22,T2,T3,T4	chr4	**6**	**75**	126043785	126043785	C	T	exonic	**Csf3r**	ENSMUSP00000129457	.	nonsynonymous SNV	Csf3r:NM_001252651:exon15:c.C2137T:p.P713S,Csf3r:NM_007782:exon17:c.C2461T:p.P821S	**P713S**	ENSMUSP00000129457,713,P,S	4,126043785,C,A	4qD2.2	.	0.25	.	41	chr4	126043785	.	C	A	.	str_contraction;t_lod_fstar	ECNT=1;HCNT=1;MAX_ED=.;MIN_ED=.;NLOD=4.16;RPA=10,9;RU=GAG;STR;TLOD=4.94	GT:AD:AF:ALT_F1R2:ALT_F2R1:FOXOG:QSS:REF_F1R2:REF_F2R1	0/1:33,3:0.086:2:1:.:937,86:17:16	0/0:16,0:0.00:0:0:.:443,0:8:8	1.4	Deleterious	-3.99	Deleterious	121	30	0	Damaging	2.88	34
T12,T1,T22,T2,T3,T4	chr4	**6**	**75**	126043785	126043785	C	T	exonic	**Csf3r**	ENSMUSP00000129457	.	nonsynonymous SNV	Csf3r:NM_001252651:exon15:c.C2137T:p.P713S,Csf3r:NM_007782:exon17:c.C2461T:p.P821S	**P821S**	ENSMUSP00000129457,821,P,S	4,126043785,C,A	4qD2.2	.	0.25	.	41	chr4	126043785	.	C	A	.	alt_allele_in_normal	ECNT=1;HCNT=6;MAX_ED=.;MIN_ED=.;NLOD=8.99	GT:AD:AF:ALT_F1R2:ALT_F2R1:FOXOG:QSS:REF_F1R	0/1:78,6:0.073:4:2:0.333:2201,174:43:35	0/0:78,5:0.052:4:1:0.200:2139,144:47:31	0.2	Deleterious	-3.99	Deleterious	121	30	0	Damaging	2.88	34
T12,T22,T2,T42,T4	chr6	**6**	**75**	129658713	129658713	G	T	exonic	**Klrc2**	ENSMUSP00000038150	.	nonsynonymous SNV	Klrc2:NM_001098669:exon3:c.C376A:p.L126I,Klrc2:NM_010653:exon4:c.C451A:p.L151I	**L126I**	ENSMUSP00000038150,126,L,I	6,129658713,G,T	6qF3	rs212670017	0.25	.	35	chr6	129658713	.	G	T	.	clustered_events;homologous_mapping_event	DB;ECNT=9;HCNT=8;MAX_ED=96;MIN_ED=1;NLOD=7.52;TLOD	GT:AD:AF:ALT_F1R2:ALT_F2R1:FOXOG:PGT:PID:QSS:REF_F1R	0/1:12,22:0.636:14:8:0.364:0|1:129639089_A_T:344,606:4:8	0/0:28,0:0.00:0:0:.:0|1:129639089_A_T:790,0:13:15	2.4	Tolerated	0.36	Neutral	198	30	0.216	Tolerated	2.81	223
T12,T1,T22,T2,T42,T4	chr16	**6**	**75**	32752585	32752585	C	A	exonic	**Muc4**	ENSMUSP00000059471	.	nonsynonymous SNV	Muc4:NM_080457:exon2:c.C2462A:p.T821N	**T821N**	ENSMUSP00000059471,821,T,N	16,32752585,C,A	16qB3	.	0.25	.	66	chr16	32752585	.	C	A	.	alt_allele_in_normal;clustered_events	ECNT=5;HCNT=1;MAX_ED=52;MIN_ED=4;NLOD=10.31;TLOD=22.53	GT:AD:AF:ALT_F1R2:ALT_F2R1:FOXOG:PGT:PID:QSS:REF_F1R2:REF_F2R1	0/1:140,8:0.055:6:2:0.750:0|1:32754579_C_A:3922,215:70:70	0/0:114,5:0.055:2:3:0.400:0|1:32754579_C_A:3144,143:61:53	4	Damaging	-0.42	Neutral	56	30	0.004	Damaging	3.55	11
T12,T1,T2,T32,T3,T4	chr16	**6**	**75**	32752657	32752657	G	C	exonic	**Muc4**	ENSMUSP00000059471	.	nonsynonymous SNV	Muc4:NM_080457:exon2:c.G2534C:p.G845A	**G845A**	ENSMUSP00000059471,845,G,A	16,32752657,G,A	16qB3	.	0.25	.	41	chr16	32752657	.	G	A	.	alt_allele_in_normal;clustered_events	ECNT=5;HCNT=2;MAX_ED=35;MIN_ED=4;NLOD=10.63;TLOD=31.55	GT:AD:AF:ALT_F1R2:ALT_F2R1:FOXOG:PGT:PID:QSS:REF_F1R2:REF_F2R1	0/1:173,9:0.061:5:4:0.556:0|1:32754882_T_C:4662,244:83:90	0/0:132,6:0.048:4:2:0.667:0|1:32754882_T_C:3576,160:63:69	4	Damaging	-0.33	Neutral	56	30	0.01	Damaging	3.55	10
T12,T1,T22,T32,T42,T4	chr16	**6**	**75**	32754595	32754595	C	G	exonic	**Muc4**	ENSMUSP00000059471	.	nonsynonymous SNV	Muc4:NM_080457:exon4:c.C4468G:p.Q1490E	**Q1490E**	ENSMUSP00000059471,1490,Q,E	16,32754595,C,A	16qB3	.	0.25	.	104	chr16	32754595	.	C	A	.	alt_allele_in_normal;clustered_events	ECNT=5;HCNT=1;MAX_ED=52;MIN_ED=4;NLOD=5.21;TLOD=35.99	GT:AD:AF:ALT_F1R2:ALT_F2R1:FOXOG:PGT:PID:QSS:REF_F1R2:REF_F2R1	0/1:144,12:0.082:7:5:0.583:0|1:32754579_C_A:4024,330:74:70	0/0:112,7:0.064:2:5:0.286:0|1:32754579_C_A:3115,196:63:49	4	Damaging	-0.45	Neutral	56	30	0.011	Damaging	3.6	10

T12,T1,T2,T32,T3,T4	chr16	**6**	**75**	32752665	32752665	G	C	exonic	**Muc4**	ENSMUSP00000059471	.	nonsynonymous SNV	Muc4:NM_080457:exon2:c.G2542C:p.G848R	**G848R**	ENSMUSP00000059471,848,G,R	16,32752665,G,T	16qB3	.	0.25	.	41	chr16	32752665	.	G	T	.	alt_allele_in_normal;clustered_events	ECNT=5;HCNT=2;MAX_ED=35;MIN_ED=4;NLOD=25.03;TLOD=12.68	GT:AD:AF:ALT_F1R2:ALT_F2R1:FOXOG:PGT:PID:QSS:REF_F1R2:REF_F2R1	0/1:175,5:0.029:2:3:0.600:0|1:32754882_T_C:5057,136:85:90	0/0:139,3:0.023:1:2:0.667:0|1:32754882_T_C:3976,81:66:73	4	Tolerated	-0.4	Neutral	56	30	0.092	Tolerated	3.55	10
T12,T1,T22,T32,T42,T4	chr16	**6**	**75**	32754616	32754616	G	A	exonic	**Muc4**	ENSMUSP00000059471	.	nonsynonymous SNV	Muc4:NM_080457:exon4:c.G4489A:p.G1497R	**G1497R**	ENSMUSP00000059471,1497,G,R	16,32754616,G,A	16qB3	.	0.25	.	119	chr16	32754616	.	G	A	.	clustered_events;multi_event_alt_allele_in_normal	ECNT=5;HCNT=2;MAX_ED=35;MIN_ED=4;NLOD=34.63;TLOD=6.99	GT:AD:AF:ALT_F1R2:ALT_F2R1:FOXOG:PGT:PID:QSS:REF_F1R2:REF_F2R1	0/1:165,3:0.018:1:2:0.333:0|1:32754882_T_C:4772,83:79:86	0/0:139,1:7.634e-03:0:1:0.00:0|1:32754882_T_C:3953,27:63:76	4	Tolerated	-0.91	Neutral	56	30	0.066	Tolerated	3.54	12
T12,T1,T22,T32,T42,T4	chr16	**6**	**75**	32754631	32754631	G	A	exonic	**Muc4**	ENSMUSP00000059471	.	nonsynonymous SNV	Muc4:NM_080457:exon4:c.G4504A:p.G1502R	**G1502R**	ENSMUSP00000059471,1502,G,R	16,32754631,G,G	16qB3	.	0.25	.	119	chr16	32754631	.	G	G	.	alt_allele_in_normal	ECNT=1;HCNT=3;MAX_ED=.;MIN_ED=.;NLOD=7.04;TLOD=8.74	GT:AD:AF:ALT_F1R2:ALT_F2R1:FOXOG:QSS:REF_F1R2:REF_F2R1	0/1:66,5:0.070:4:1:0.800:1851,142:32:34	0/0:54,4:0.082:2:2:0.500:1471,116:21:33	4	Tolerated	0	Neutral	56	30	0.141	Tolerated	3.54	12
T12,T1,T2,T32,T42,T4	chr11	**6**	**75**	59129846	59129846	-	TGGGGACAAA	splicing	**Obscn**	ENSMUSP00000016094	.	NM_199152:exon7:c.2372-1->TTTGTCCCCA	.	**NA**	11,59129846,C,C	NA	11qB1.3	.	0.25	.	29	chr11	59129846	.	C	C	.	alt_allele_in_normal;clustered_events;germline_risk	DB;ECNT=2;HCNT=4;MAX_ED=40;MIN_ED=40;NLOD=1.87;TLOD=7.14	GT:AD:AF:ALT_F1R2:ALT_F2R1:FOXOG:QSS:REF_F1R2:REF_F2R1	0/1:66,6:0.083:3:3:0.500:1890,134:32:34	0/0:24,2:0.080:2:0:1.00:681,44:17:7	22	Unkown	.	.	.	.	.	.	.	.
T12,T1,T22,T32,T3,T4	chr18	**6**	**75**	37449668	37449668	C	T	exonic	**Pcdhb14**	ENSMUSP00000126428	.	nonsynonymous SNV	Pcdhb14:NM_053139:exon1:c.C1826T:p.T609I	**T609I**	ENSMUSP00000126428,609,T,I	18,37449668,C,G	18qB3	.	0.25	.	76	chr18	37449668	.	C	G	.	PASS	ECNT=1;HCNT=1;MAX_ED=.;MIN_ED=.;NLOD=12.59;TLOD=6.94	GT:AD:AF:ALT_F1R2:ALT_F2R1:FOXOG:QSS:REF_F1R2:REF_F2R1	0/1:47,4:0.083:0:4:1.00:1331,110:24:23	0/0:46,0:0.00:0:0:.:1292,0:25:21	19	Damaging	0.78	Neutral	390	30	0.019	Damaging	3.35	393
T12,T1,T22,T32,T3,T4	chr18	**6**	**75**	37449667	37449667	A	G	exonic	**Pcdhb14**	ENSMUSP00000126428	.	nonsynonymous SNV	Pcdhb14:NM_053139:exon1:c.A1825G:p.T609A	**T609A**	ENSMUSP00000126428,609,T,A	18,37449667,A,G	18qB3	rs13490689	0.25	.	75	chr18	37449667	rs13490689	A	G	.	PASS	ECNT=1;HCNT=1;MAX_ED=.;MIN_ED=.;NLOD=12.59;TLOD=6.94	GT:AD:AF:ALT_F1R2:ALT_F2R1:FOXOG:QSS:REF_F1R2:REF_F2R1	0/1:47,4:0.083:0:4:1.00:1331,110:24:23	0/0:46,0:0.00:0:0:.:1292,0:25:21	19	Tolerated	3.39	Neutral	390	30	1	Tolerated	3.35	393
T12,T1,T22,T32,T32,T42	chr18	**6**	**75**	37309268	37309268	A	G	exonic	**Pcdhb4**	ENSMUSP00000132166	.	nonsynonymous SNV	Pcdhb4:NM_053129:exon1:c.A1630G:p.T544A	**T544A**	ENSMUSP00000132166,544,T,A	18,37309268,A,T	18qB3	.	0.25	.	76	chr18	37309268	.	A	T	.	alt_allele_in_normal;clustered_events;t_lod_fstar	DB;ECNT=21;HCNT=36;MAX_ED=213;MIN_ED=12;NLOD=8.76;TLOD=5.06	GT:AD:AF:ALT_F1R2:ALT_F2R1:FOXOG:PGT:PID:QSS:REF_F1R2:REF_F2R1	0/1:65,2:0.029:0:2:1.00:0|1:37309309_G_A:1718,60:27:38	0/0:59,1:0.034:0:1:1.00:0|1:37309309_G_A:1504,27:27:32	0.7	Damaging	0.5	Neutral	388	30	0.001	Damaging	3.36	390
T12,T1,T22,T32,T32,T42	chr18	**6**	**75**	37309328	37309328	C	A	exonic	**Pcdhb4**	ENSMUSP00000132166	.	nonsynonymous SNV	Pcdhb4:NM_053129:exon1:c.C1690A:p.L564M	**L564M**	ENSMUSP00000132166,564,L,M	18,37309328,C,G	18qB3	.	0.25	.	63	chr18	37309328	.	C	G	.	clustered_events;homologous_mapping_event;t_lod_fstar	ECNT=21;HCNT=36;MAX_ED=213;MIN_ED=12;NLOD=17.16;TLOD=5.08	GT:AD:AF:ALT_F1R2:ALT_F2R1:FOXOG:PGT:PID:QSS:REF_F1R2:REF_F2R1	0/1:66,2:0.029:0:2:1.00:0|1:37309309_G_A:1831,57:29:37	0/0:60,0:0.00:0:0:.:0|1:37309309_G_A:1635,0:28:32	0.7	Damaging	-2.27	Neutral	388	30	0.002	Damaging	3.37	388
T12,T22,T32,T3,T42,T4	chr18	**6**	**75**	37309461	37309461	C	T	exonic	**Pcdhb4**	ENSMUSP00000132166	.	nonsynonymous SNV	Pcdhb4:NM_053129:exon1:c.C1823T:p.A608V	**A608V**	ENSMUSP00000132166,608,A,V	18,37309461,C,A	18qB3	.	0.25	.	66	chr18	37309461	.	C	A	.	clustered_events;homologous_mapping_event;t_lod_fstar	ECNT=3;HCNT=8;MAX_ED=71;MIN_ED=30;NLOD=11.13;TLOD=5.36	GT:AD:AF:ALT_F1R2:ALT_F2R1:FOXOG:QSS:REF_F1R2:REF_F2R1	0/1:31,4:0.114:1:3:0.750:857,97:15:16	0/0:41,0:0.00:0:0:.:1124,0:22:19	1	Deleterious	-5.46	Deleterious	388	30	0.004	Damaging	3.37	391
T12,T1,T22,T32,T32,T42	chr18	**6**	**75**	37309344	37309344	C	T	exonic	**Pcdhb4**	ENSMUSP00000132166	.	nonsynonymous SNV	Pcdhb4:NM_053129:exon1:c.C1706T:p.A569V	**A569V**	ENSMUSP00000132166,569,A,V	18,37309344,C,T	18qB3	rs264254737	0.25	.	60	chr18	37309344	rs264254737	C	T	.	clustered_events;multi_event_alt_allele_in_normal;t_lod_fstar	ECNT=21;HCNT=33;MAX_ED=213;MIN_ED=12;NLOD=13.56;TLOD=5.01	GT:AD:AF:ALT_F1R2:ALT_F2R1:FOXOG:PGT:PID:QSS:REF_F1R2:REF_F2R1	0/1:75,2:0.027:0:2:1.00:0|1:37309453_G_A:2181,56:33:42	0/0:65,1:0.017:0:1:1.00:0|1:37309453_G_A:1828,28:29:36	0.7	Deleterious	-3.12	Deleterious	388	30	0.053	Tolerated	3.37	389

T12,T22,T32,T3,T42,T4	chr6	**6**	**75**	112649642	112649642	-	ACGGCAGTGTCTCTGCAGGTTCATCTGTTCTCATCG	exonic	**Rad18**	ENSMUSP00000027362	.	nonframeshift insertion	Rad18:NM_021385:exon10:c.1222_1223insCGATGAGAACAGATGAACCTGCAGAGACACTGCCGT:p.L408delinsSMRTDEPAETLPL,Rad18:NM_001167730:exon11:c.1300_1301insCGATGAGAACAGATGAACCTGCAGAGACACTGCCGT:p.L434delinsSMRTDEPAETLPL	**L408delinsSMRTDEPAETLPL**	ENSMUSP00000027362,408,L,SMRTDEPAETLPL	6,112649642,A,C	6qE3	.	0.25	.	0	chr6	112649642	.	A	C	.	clustered_events;homologous_mapping_event	DB;ECNT=3;HCNT=8;MAX_ED=30;MIN_ED=12;NLOD=11.13;TLOD=44.57	GT:AD:AF:ALT_F1R2:ALT_F2R1:FOXOG:QSS:REF_F1R2:REF_F2R1	0/1:28,21:0.417:14:7:0.667:802,515:10:18	0/0:46,0:0.00:0:0:.:1321,0:22:24	3	Deleterious	-17.48	Deleterious	151	30	NA	NA	NA	NA
T12,T22,T32,T3,T42,T4	chr6	**6**	**75**	112649642	112649642	-	ACGGCAGTGTCTCTGCAGGTTCATCTGTTCTCATCG	exonic	**Rad18**	ENSMUSP00000027362	.	nonframeshift insertion	Rad18:NM_021385:exon10:c.1222_1223insCGATGAGAACAGATGAACCTGCAGAGACACTGCCGT:p.L408delinsSMRTDEPAETLPL,Rad18:NM_001167730:exon11:c.1300_1301insCGATGAGAACAGATGAACCTGCAGAGACACTGCCGT:p.L434delinsSMRTDEPAETLPL	**L434delinsSMRTDEPAETLPL**	ENSMUSP00000027362,434,L,SMRTDEPAETLPL	6,112649642,A,T	6qE3	.	0.25	.	0	chr6	112649642	.	A	T	.	clustered_events;homologous_mapping_event	DB;ECNT=3;HCNT=8;MAX_ED=30;MIN_ED=12;NLOD=10.83;TLOD=50.05	GT:AD:AF:ALT_F1R2:ALT_F2R1:FOXOG:QSS:REF_F1R2:REF_F2R1	0/1:27,22:0.413:14:8:0.364:731,633:9:18	0/0:45,0:0.00:0:0:.:1256,0:20:25	3	Unknown	NA	NA	80	30	NA	NA	NA	NA
T12,T1,T2,T32,T3,T42	chr18	**6**	**75**	36682151	36682151	-	GCCC	exonic	**Slc35a4**	ENSMUSP00000029888	.	frameshift insertion	Slc35a4:NM_026404:exon3:c.33_34insGCCC:p.L11fs,Slc35a4:NM_001083317:exon4:c.33_34insGCCC:p.L11fs	**L11fs**	ENSMUSP00000029888,11,L,	18,36682151,A,A	18qB2	.	0.25	.	75	chr18	36682151	.	A	A	.	clustered_events;multi_event_alt_allele_in_normal	ECNT=8;HCNT=1;MAX_ED=14;MIN_ED=1;NLOD=6.94;TLOD=	GT:AD:AF:ALT_F1R2:ALT_F2R1:FOXOG:PGT:PID:QSS:REF_F1R	0/1:54,4:0.069:4:0:1.00:0|1:76615201_C_T:1405,78:27:27	0/0:38,1:0.026:1:0:1.00:0|1:76615201_C_T:1019,28:20:18	19	Tolerated	0	Neutral	173	30	1	Tolerated	3.25	32
T12,T22,T2,T32,T3,T42	chr5	**6**	**75**	31042999	31042999	T	A	exonic	**Slc5a6**	ENSMUSP00000021918	.	nonsynonymous SNV	Slc5a6:NM_177870:exon3:c.A341T:p.H114L,Slc5a6:NM_001177621:exon4:c.A341T:p.H114L,Slc5a6:NM_001177622:exon4:c.A341T:p.H114L	**H114L**	ENSMUSP00000021918,114,H,L	5,31042999,T,TACCTGCTTTACAGCCGGCAGGTACCATTAGCAGCATCTTGGGTAGTCCCACTTTTTGTGGCATTTTCAACCATCGGTGCTGCTAATGGC	5qB1	.	0.25	.	48	chr5	31042999	.	T	CTACCTGCTTTACAGCCGGCAGGTACCATTAGCAGCATCTTGGGTAGTCCCACTTTTTGTGGCATTTTCAACCATCGGTGCTGCTAATGG	.	t_lod_fstar	ECNT=1;HCNT=2;MAX_ED=.;MIN_ED=.;NLOD=8.83;TLOD=5.84	GT:AD:AF:ALT_F1R2:ALT_F2R1:FOXOG:QSS:REF_F1R2:REF_F2R1	0/1:44,1:0.067:1:0:.:1252,28:23:21	0/0:32,0:0.00:0:0:.:881,0:14:18	1.5	Unknown	NA	NA	147	30	NA	NA	NA	NA
T12,T1,T2,T32,T3,T4	chr1	**6**	**75**	85596079	85596079	C	G	exonic	**Sp110**	ENSMUSP00000040248	.	nonsynonymous SNV	Sp110:NM_030194:exon2:c.G30C:p.K10N,Sp110:NM_175397:exon3:c.G30C:p.K10N	**K10N**	ENSMUSP00000040248,10,K,N	1,85596079,C,A	1qC5	rs107818046	0.25	.	578	chr1	85596079	.	C	A	.	alt_allele_in_normal;clustered_events	ECNT=10;HCNT=1;MAX_ED=117;MIN_ED=25;NLOD=8	GT:AD:AF:ALT_F1R2:ALT_F2R1:FOXOG:QSS:REF_F1R	0/1:427,9:0.022:7:2:0.222:1496,258:234:193	0/0:535,22:0.040:15:7:0.318:14538,609:286:249	0.3	Damaging	-0.82	Neutral	104	30	0.005	Damaging	3.01	73
T12,T1,T22,T2,T3,T42	chr1	**6**	**75**	85584921	85584921	G	T	exonic	**Sp110**	ENSMUSP00000040248	.	nonsynonymous SNV	Sp110:NM_030194:exon8:c.C952A:p.Q318K,Sp110:NM_175397:exon9:c.C952A:p.Q318K	**Q318K**	ENSMUSP00000040248,318,Q,K	1,85584921,G,G	1qC5	.	0.25	.	437	chr1	85584921	.	G	G	.	alt_allele_in_normal;clustered_events	ECNT=10;HCNT=2;MAX_ED=117;MIN_ED=25;NLOD=43.65;TLO	GT:AD:AF:ALT_F1R2:ALT_F2R1:FOXOG:QSS:REF_F1R2:REF_F2	0/1:427,36:0.081:15:21:0.417:12211,908:234:193	0/0:537,42:0.080:15:27:0.357:15087,1010:285:252	0.3	Tolerated	0	Neutral	104	30	1	Tolerated	2.83	75
T12,T1,T2,T32,T3,T42	chr1	**6**	**75**	85589114	85589114	C	T	exonic	**Sp110**	ENSMUSP00000040248	.	nonsynonymous SNV	Sp110:NM_030194:exon5:c.G661A:p.V221M,Sp110:NM_175397:exon6:c.G661A:p.V221M	**V221M**	ENSMUSP00000040248,221,V,M	1,85589114,C,G	1qC5	rs107738149	0.25	.	637	chr1	85589114	.	C	G	.	alt_allele_in_normal;clustered_events	ECNT=10;HCNT=2;MAX_ED=117;MIN_ED=25;NLOD=75.62;TLOD=58.54	GT:AD:AF:ALT_F1R2:ALT_F2R1:FOXOG:PGT:PID:QSS:REF_F1R2:REF_F2R1	0/1:532,36:0.062:22:14:0.389:0|1:85589100_C_A:14804,1016:294:238	0/0:607,27:0.044:10:17:0.630:0|1:85589100_C_A:17076,781:319:288	0.3	Tolerated	-1.31	Neutral	104	30	0.201	Tolerated	2.94	54
T12,T1,T2,T32,T3,T42	chr1	**6**	**75**	85589120	85589120	C	T	exonic	**Sp110**	ENSMUSP00000040248	.	nonsynonymous SNV	Sp110:NM_030194:exon5:c.G655A:p.E219K,Sp110:NM_175397:exon6:c.G655A:p.E219K	**E219K**	ENSMUSP00000040248,219,E,K	1,85589120,C,T	1qC5	.	0.25	.	630	chr1	85589120	.	C	T	.	alt_allele_in_normal;clustered_events	ECNT=10;HCNT=2;MAX_ED=117;MIN_ED=25;NLOD=111.88;TLOD=40.30	GT:AD:AF:ALT_F1R2:ALT_F2R1:FOXOG:PGT:PID:QSS:REF_F1R2:REF_F2R1	0/1:559,19:0.034:7:12:0.632:0|1:85589114_C_T:15627,518:312:247	0/0:618,19:0.029:12:7:0.368:0|1:85589114_C_T:17573,526:320:298	0.3	Tolerated	-0.45	Neutral	104	30	0.702	Tolerated	2.9	54
T1,T22,T2,T32,T3,T42	chr1	**6**	**75**	85596002	85596002	G	A	exonic	**Sp110**	ENSMUSP00000040248	.	nonsynonymous SNV	Sp110:NM_030194:exon2:c.C107T:p.A36V,Sp110:NM_175397:exon3:c.C107T:p.A36V	**A36V**	ENSMUSP00000040248,36,A,V	1,85596002,G,C	1qC5	rs108278440	0.25	.	400	chr1	85596002	.	G	C	.	alt_allele_in_normal;clustered_events	ECNT=5;HCNT=3;MAX_ED=83;MIN_ED=14;NLOD=101.63;TLO	GT:AD:AF:ALT_F1R2:ALT_F2R1:FOXOG:QSS:REF_F1R2:REF_F2	0/1:766,39:0.048:13:26:0.333:21440,1095:393:373	0/0:709,35:0.047:14:21:0.400:19242,935:349:360	0.3	Tolerated	2.18	Neutral	104	30	1	Tolerated	2.81	106
T12,T1,T2,T32,T3,T42	chr1	**6**	**75**	85589192	85589192	C	A	splicing	**Sp110**	ENSMUSP00000040248	NM_030194:exon5:c.584-1G>T;NM_175397:exon6:c.584-1G>T	.	.	**NA**	1,85589192,C,T	NA	1qC5	.	0.25	.	557	chr1	85589192	.	C	T	.	alt_allele_in_normal;clustered_events	ECNT=3;HCNT=6;MAX_ED=100;MIN_ED=38;NLOD=24.62;TLOD=56.04	GT:AD:AF:ALT_F1R2:ALT_F2R1:FOXOG:QSS:REF_F1R2:REF_F2R1	0/1:407,35:0.078:19:16:0.543:11616,967:199:208	0/0:406,31:0.074:19:12:0.613:11385,852:195:211	0.3	Unkown	.	.	.	.	.	.	.	.

T12,T22,T2,T32,T3,T42	chr1	**6**	**75**	85610808	85610808	C	G	exonic	**Sp140**	ENSMUSP00000108782	.	nonsynonymous SNV	Sp140:NM_001013817:exon3:c.C265G:p.Q89E	**Q89E**	ENSMUSP00000108782,89,Q,E	1,85610808,C,A	1qC5	rs51555481	0.25	.	391	chr1	85610808	.	C	A	.	t_lod_fstar	ECNT=1;HCNT=8;MAX_ED=.;MIN_ED=.;NLOD=11.29;TLOD=4.59	GT:AD:AF:ALT_F1R2:ALT_F2R1:FOXOG:QSS:REF_F1R2:REF_F2R1	0/1:62,4:0.047:1:3:0.250:1737,117:35:27	0/0:41,1:0.024:0:1:0.00:1117,13:18:23	0.2	Damaging	-1.36	Neutral	93	30	0.003	Damaging	2.86	66
T12,T1,T22,T2,T3,T42	chr1	**6**	**75**	85610790	85610790	G	T	exonic	**Sp140**	ENSMUSP00000108782	.	nonsynonymous SNV	Sp140:NM_001013817:exon3:c.G247T:p.D83Y	**D83Y**	ENSMUSP00000108782,83,D,Y	1,85610790,G,T	1qC5	.	0.25	.	399	chr1	85610790	.	G	T	.	alt_allele_in_normal;clustered_events	ECNT=11;HCNT=5;MAX_ED=129;MIN_ED=1;NLOD=54.91;TLOD=15.59	GT:AD:AF:ALT_F1R2:ALT_F2R1:FOXOG:PGT:PID:QSS:REF_F1R2:REF_F2R1	0/1:192,5:0.034:4:1:0.200:0|1:85610790_G_T:4920,146:96:96	0/0:350,9:0.025:5:4:0.444:0|1:85610790_G_T:8736,257:154:196	0.2	Deleterious	-3.98	Deleterious	93	30	0.004	Damaging	2.86	66
T12,T1,T22,T2,T42,T4	chr1	**6**	**75**	85610824	85610824	G	A	exonic	**Sp140**	ENSMUSP00000108782	.	nonsynonymous SNV	Sp140:NM_001013817:exon3:c.G281A:p.R94Q	**R94Q**	ENSMUSP00000108782,94,R,Q	1,85610824,G,G	1qC5	.	0.25	.	340	chr1	85610824	.	G	G	.	alt_allele_in_normal;clustered_events	ECNT=11;HCNT=5;MAX_ED=129;MIN_ED=1;NLOD=38.43;TLOD=21.52	GT:AD:AF:ALT_F1R2:ALT_F2R1:FOXOG:PGT:PID:QSS:REF_F1R2:REF_F2R1	0/1:200,7:0.041:5:2:0.286:0|1:85610790_G_T:5681,202:100:100	0/0:376,15:0.040:7:8:0.533:0|1:85610790_G_T:10589,423:172:204	0.2	Tolerated	0	Neutral	93	30	0.835	Tolerated	2.86	66
T12,T1,T2,T32,T3,T4	chr12	**6**	**75**	80949818	80949823	GGAGCC	-	exonic	**Srsf5**	ENSMUSP00000144120	.	nonframeshift deletion	Srsf5:NM_001079694:exon8:c.650_655del:p.217_219del,Srsf5:NM_001079695:exon8:c.650_655del:p.217_219del,Srsf5:NM_001347415:exon8:c.653_658del:p.218_220del,Srsf5:NM_001347416:exon8:c.653_658del:p.218_220del,Srsf5:NM_009159:exon8:c.650_655del:p.217_219del	**218_220del**	NA	12,80949818,AGGAGCC,C	12qC3	.	0.25	.	30	chr12	80949817	.	AGGAGCC	C	.	str_contraction;t_lod_fstar	ECNT=1;HCNT=2;MAX_ED=.;MIN_ED=.;NLOD=11.36;RPA=6,5;RU=CTG;STR;TLOD=5.65	GT:AD:AF:ALT_F1R2:ALT_F2R1:FOXOG:QSS:REF_F1R2:REF_F2R1	0/1:37,2:0.056:0:2:.:1034,56:19:18	0/0:41,0:0.00:0:0:.:1144,0:21:20	3	Unknown	.	.	.	.	.	.	.	.
T12,T1,T2,T32,T3,T4	chr12	**6**	**75**	80949818	80949823	GGAGCC	-	exonic	**Srsf5**	ENSMUSP00000144120	.	nonframeshift deletion	Srsf5:NM_001079694:exon8:c.650_655del:p.217_219del,Srsf5:NM_001079695:exon8:c.650_655del:p.217_219del,Srsf5:NM_001347415:exon8:c.653_658del:p.218_220del,Srsf5:NM_001347416:exon8:c.653_658del:p.218_220del,Srsf5:NM_009159:exon8:c.650_655del:p.217_219del	**217_219del**	NA	12,80949818,AGGAGCC,GCCTGATCCTCTAAGTGGACCAGGCCACCCGTATCATGGT	12qC3	.	0.25	.	30	chr12	80949817	.	AGGAGCC	GCCTGATCCTCTAAGTGGACCAGGCCACCCGTATCATGGT	.	t_lod_fstar	ECNT=1;HCNT=1;MAX_ED=.;MIN_ED=.;NLOD=8.90;TLOD=5.31	GT:AD:AF:ALT_F1R2:ALT_F2R1:FOXOG:QSS:REF_F1R2:REF_F2R1	0/1:53,2:0.038:0:2:.:1499,53:27:26	0/0:33,0:0.00:0:0:.:897,0:19:14	0.4	Unknown	.	.	.	.	.	.	.	.
T12,T22,T2,T3,T42,T4	chr4	**6**	**75**	119282420	119282420	G	A	exonic	**Ybx1**	ENSMUSP00000133076	.	nonsynonymous SNV	Ybx1:NM_011732:exon5:c.C377T:p.P126L	**P126L**	ENSMUSP00000133076,126,P,L	4,119282420,G,T	4qD2.1	.	0.25	.	41	chr4	119282420	.	G	T	.	clustered_events;multi_event_alt_allele_in_normal;t_lod_fstar	ECNT=4;HCNT=15;MAX_ED=29;MIN_ED=24;NLOD=6.35;TLOD=5.45	GT:AD:AF:ALT_F1R2:ALT_F2R1:FOXOG:PGT:PID:QSS:REF_F1R2:REF_F2R1	0/1:43,2:0.044:2:0:0.00:0|1:119282444_G_A:1187,52:20:23	0/0:37,1:0.028:1:0:0.00:0|1:119282444_G_A:1030,27:17:20	1.9	Deleterious	-7.98	Deleterious	213	30	0	Damaging	2.9	292
T1,T22,T32,T3,T4	chr10	**5**	**62.5**	81178729	81178729	G	A	exonic	**Eef2**	ENSMUSP00000132786	.	nonsynonymous SNV	Eef2:NM_007907:exon4:c.G449A:p.R150H	**R150H**	ENSMUSP00000132786,150,R,H	10,81178729,G,C	10qC1	.	0.25	.	317	chr10	81178729	.	G	C	.	alt_allele_in_normal	ECNT=1;HCNT=6;MAX_ED=.;MIN_ED=.;NLOD=15.86;TLOD=29.37	GT:AD:AF:ALT_F1R2:ALT_F2R1:FOXOG:QSS:REF_F1R2:REF_F2R1	0/1:272,26:0.096:14:12:0.538:7599,596:140:132	0/0:320,28:0.084:17:11:0.607:8831,670:169:151	0.7	Deleterious	-5.91	Deleterious	706	30	0.001	Damaging	3.61	381
T12,T1,T22,T2,T42	chr1	**5**	**62.5**	171530511	171530511	T	G	exonic	**Itln1**	ENSMUSP00000116671	.	nonsynonymous SNV	Itln1:NM_010584:exon6:c.A668C:p.Y223S	**Y223S**	ENSMUSP00000116671,223,Y,S	1,171530511,T,A	1qH3	.	0.25	.	156	chr1	171530511	.	T	A	.	alt_allele_in_normal;clustered_events	ECNT=8;HCNT=4;MAX_ED=146;MIN_ED=15;NLOD=17.81;TLOD=17.17	GT:AD:AF:ALT_F1R2:ALT_F2R1:FOXOG:PGT:PID:QSS:REF_F1R2:REF_F2R1	0/1:93,5:0.061:2:3:0.400:0|1:171531166_A_T:2549,148:43:50	0/0:110,4:0.038:1:3:0.250:0|1:171531166_A_T:3022,113:55:55	20	Deleterious	-2.81	Deleterious	144	30	0.058	Tolerated	2.91	154
T12,T22,T2,T42,T4	chr6	**5**	**62.5**	129658713	129658713	G	T	exonic	**Klrc2**	ENSMUSP00000038150	.	nonsynonymous SNV	Klrc2:NM_001098669:exon3:c.C376A:p.L126I,Klrc2:NM_010653:exon4:c.C451A:p.L151I	**L151I**	ENSMUSP00000038150,151,L,I	6,129658713,G,T	6qF3	rs212670017	0.25	.	35	chr6	129658713	.	G	T	.	clustered_events;homologous_mapping_event	DB;ECNT=9;HCNT=8;MAX_ED=96;MIN_ED=1;NLOD=7.52;TLOD=83.79	GT:AD:AF:ALT_F1R2:ALT_F2R1:FOXOG:PGT:PID:QSS:REF_F1R2:REF_F2R1	0/1:12,23:0.636:15:8:0.652:0|1:129639089_A_T:332,654:4:8	0/0:27,0:0.00:0:0:.:0|1:129639089_A_T:748,0:12:15	2.4	Tolerated	0.36	Neutral	198	30	0.216	Tolerated	2.81	223

T22, T2, T32,T3,T42	chr16	**5**	**62.5**	32751942	32751942	A	T	exonic	**Muc4**	ENSMUSP00000059471	.	nonsynonymous SNV	Muc4:NM_080457:exon2:c.A1819T:p.T607S	**T607S**	ENSMUSP00000059471,607,T,S	16,32751942,A,C	16qB3	.	0.25	.	117	chr16	32751942	.	A	C	.	alt_allele_in_normal	ECNT=2;HCNT=24;MAX_ED=2;MIN_ED=2;NLOD=5.73;TLOD=33.76	GT:AD:AF:ALT_F1R2:ALT_F2R1:FOXOG:PGT:PID:QSS:REF_F1R2:REF_F2R1	0/1:44,10:0.185:5:5:0.500:0|1:32755873_A_C:1231,235:19:25	0/0:50,3:0.059:0:3:1.00:0|1:32755873_A_C:1367,74:24:26	4	Damaging	-0.13	Neutral	56	30	0.004	Damaging	3.45	13
T22,T32,T3,T42,T4	chr7	**5**	**62.5**	141859064	141859064	T	C	exonic	**Muc5b**	ENSMUSP00000107873	.	nonsynonymous SNV	Muc5b:NM_028801:exon31:c.T5746C:p.Y1916H	**Y1916H**	ENSMUSP00000107873,1916,Y,H	7,141859064,T,G	7qF5	rs3142423	0.25	.	41	chr7	141859064	.	T	G	.	alt_allele_in_normal	ECNT=1;HCNT=1;MAX_ED=.;MIN_ED=.;NLOD=11.72;TLOD=7.82	GT:AD:AF:ALT_F1R2:ALT_F2R1:FOXOG:QSS:REF_F1R2:REF_F2R1	0/1:79,7:0.063:4:3:.:2124,198:39:40	0/0:42,4:0.109:0:4:.:1105,93:14:28	19	Deleterious	-5.67	Deleterious	42	30	0.054	Tolerated	2.77	30
T12,T1,T22,T2,T32,	chr14	**5**	**62.5**	54944741	54944741	T	A	exonic	**Myh6**	ENSMUSP00000000505	.	nonsynonymous SNV	Myh6:NM_001164171:exon35:c.A5209T:p.T1737S,Myh6:NM_010856:exon35:c.A5209T:p.T1737S	**T1737S**	ENSMUSP00000000505,1737,T,S	14,54944741,T,G	14qC2	.	0.25	.	51	chr14	54944741	.	T	G	.	clustered_events;homologous_mapping_event	ECNT=3;HCNT=1;MAX_ED=5;MIN_ED=4;NLOD=17.76;TLOD=8.12	GT:AD:AF:ALT_F1R2:ALT_F2R1:FOXOG:PGT:PID:QSS:REF_F1R2:REF_F2R1	0/1:66,3:0.043:1:2:0.333:0|1:54960410_A_C:1876,81:33:33	0/0:65,0:0.00:0:0:.:0|1:54960410_A_C:1800,0:37:28	1.5	Damaging	-1.34	Neutral	529	30	0.023	Damaging	3.89	386
T22,T2,T3,T42,T4	chr4	**5**	**62.5**	128705175	128705175	C	T	exonic	**Phc2**	ENSMUSP00000058320	.	nonsynonymous SNV	Phc2:NM_001195130:exon2:c.C124T:p.R42C,Phc2:NM_018774:exon2:c.C124T:p.R42C	**R42C**	ENSMUSP00000058320,42,R,C	4,128705175,C,T	4qD2.2	.	0.25	.	41	chr4	128705175	.	C	T	.	t_lod_fstar	ECNT=1;HCNT=1;MAX_ED=.;MIN_ED=.;NLOD=11.13;TLOD=6.00	GT:AD:AF:ALT_F1R2:ALT_F2R1:FOXOG:QSS:REF_F1R2:REF_F2R1	0/1:23,3:0.115:1:2:0.667:658,86:15:8	0/0:42,0:0.00:0:0:.:1186,0:17:25	1.1	Deleterious	-3.99	Deleterious	191	30	0	Damaging	3.4	100
T12,T22,T2,T42,T4	chr10	**5**	**62.5**	22181326	22181326	A	T	exonic	**Raet1c;Raet1e**	NONE	.	nonsynonymous SNV	Raet1c:NM_009018:exon5:c.A545T:p.Y182F,Raet1e:NM_198193:exon5:c.A548T:p.Y183F	**Y182F**	NA	NA	10qA3	rs235720530	0.25	.	51	chr10	22181326	.	A	T	.	alt_allele_in_normal;clustered_events;t_lod_fstar	ECNT=2;HCNT=29;MAX_ED=4;MIN_ED=4;NLOD=6.27;TLOD=4.04	GT:AD:AF:ALT_F1R2:ALT_F2R1:FOXOG:PGT:PID:QSS:REF_F1R2:REF_F2R1	0/1:52,1:0.040:0:1:1.00:0|1:22181326_A_T:1491,27:26:26	0/0:50,1:0.043:0:1:1.00:0|1:22181326_A_T:1381,27:28:22	1.4	Unkown	.	.	.	.	.	.	.	.
T12,T22,T2,T42,T4	chr10	**5**	**62.5**	22181330	22181330	C	G	exonic	**Raet1c;Raet1e**	NONE	.	nonsynonymous SNV	Raet1c:NM_009018:exon5:c.C549G:p.F183L,Raet1e:NM_198193:exon5:c.C552G:p.F184L	**F183L**	NA	NA	10qA3	.	0.25	.	53	chr10	22181330	.	C	G	.	alt_allele_in_normal;clustered_events;t_lod_fstar	ECNT=2;HCNT=29;MAX_ED=4;MIN_ED=4;NLOD=6.27;TLOD=4.04	GT:AD:AF:ALT_F1R2:ALT_F2R1:FOXOG:PGT:PID:QSS:REF_F1R2:REF_F2R1	0/1:55,2:0.040:0:2:1.00:0|1:22181326_A_T:1578,56:29:26	0/0:51,2:0.043:0:2:1.00:0|1:22181326_A_T:1466,55:28:23	1.4	Unkown	.	.	.	.	.	.	.	.
T12,T22,T2,T42,T4	chr10	**5**	**62.5**	22181326	22181326	A	T	exonic	**Raet1c;Raet1e**	NONE	.	nonsynonymous SNV	Raet1c:NM_009018:exon5:c.A545T:p.Y182F,Raet1e:NM_198193:exon5:c.A548T:p.Y183F	**Y183F**	NA	NA	10qA3	rs235720530	0.25	.	51	chr10	22181326	.	A	T	.	alt_allele_in_normal;germline_risk;t_lod_fstar	ECNT=1;HCNT=1;MAX_ED=.;MIN_ED=.;NLOD=0.721;TLOD=5.25	GT:AD:AF:ALT_F1R2:ALT_F2R1:FOXOG:QSS:REF_F1R2:REF_F2R1	0/1:49,4:0.078:3:1:0.250:1438,106:21:28	0/0:30,2:0.080:1:1:0.500:822,58:16:14	0.7	Unkown	.	.	.	.	.	.	.	.
T12,T22,T2,T42,T4	chr10	**5**	**62.5**	22181330	22181330	C	G	exonic	**Raet1c;Raet1e**	NONE	.	nonsynonymous SNV	Raet1c:NM_009018:exon5:c.C549G:p.F183L,Raet1e:NM_198193:exon5:c.C552G:p.F184L	**F184L**	NA	NA	10qA3	.	0.25	.	53	chr10	22181330	.	C	A	.	str_contraction	ECNT=1;HCNT=1;MAX_ED=.;MIN_ED=.;NLOD=7.81;RPA=6,5;RU=TG;STR;TLOD=6.52	GT:AD:AF:ALT_F1R2:ALT_F2R1:FOXOG:QSS:REF_F1R2:REF_F2R1	0/1:68,4:0.060:1:3:.:1865,91:33:35	0/0:32,0:0.00:0:0:.:880,0:16:16	1.2	Unkown	.	.	.	.	.	.	.	.
T1,T22,,T2,T3,T42	chr1	**5**	**62.5**	85584906	85584906	G	A	exonic	**Sp110**	ENSMUSP00000040248	.	stopgain	Sp110:NM_030194:exon8:c.C967T:p.Q323X,Sp110:NM_175397:exon9:c.C967T:p.Q323X	**Q323X**	ENSMUSP00000040248,323,Q,X	NA	1qC5	.	0.25	.	446	chr1	85584906	.	G	A	.	alt_allele_in_normal;clustered_events	ECNT=7;HCNT=3;MAX_ED=174;MIN_ED=60;NLOD=58.72;TLOD=91.48	GT:AD:AF:ALT_F1R2:ALT_F2R1:FOXOG:PGT:PID:QSS:REF_F1R2:REF_F2R1	0/1:814,42:0.048:21:21:0.500:0|1:85591643_T_A:22700,1150:403:411	0/0:661,38:0.050:16:22:0.421:0|1:85591643_T_A:18240,1063:318:343	0.3	Deletarious	.	.	.	.	.	.	.	.
T22,T2,T3,T42,T4	chr1	**5**	**62.5**	85585010	85585010	G	T	exonic	**Sp110**	ENSMUSP00000040248	.	nonsynonymous SNV	Sp110:NM_030194:exon8:c.C863A:p.A288E,Sp110:NM_175397:exon9:c.C863A:p.A288E	**A288E**	ENSMUSP00000040248,288,A,E	1,85585010,G,G	1qC5	.	0.25	.	457	chr1	85585010	.	G	G	.	str_contraction;t_lod_fstar	ECNT=1;HCNT=1;MAX_ED=.;MIN_ED=.;NLOD=14.92;RPA=6,5;RU=GCA;STR;TLOD=4.27	GT:AD:AF:ALT_F1R2:ALT_F2R1:FOXOG:QSS:REF_F1R2:REF_F2R1	0/1:40,3:0.050:0:3:.:968,78:20:20	0/0:61,1:0.019:1:0:.:1533,6:30:31	1	Tolerated	0	Neutral	104	30	0.761	Tolerated	2.83	64

T1,T22,T2,T3,T42	chr1	**5**	**62.5**	85610777	85610777	G	T	exonic	**Sp140**	ENSMUSP00000108782	.	nonsynonymous SNV	Sp140:NM_001013817:exon3:c.G234T:p.E78D	**E78D**	ENSMUSP00000108782,78,E,D	1,85610777,G,T	1qC5	.	0.25	.	427	chr1	85610777	.	G	T	.	alt_allele_in_normal;clustered_events	ECNT=15;HCNT=1;MAX_ED=140;MIN_ED=12;NLOD=39.73;TLOD=26.70	GT:AD:AF:ALT_F1R2:ALT_F2R1:FOXOG:PGT:PID:QSS:REF_F1R2:REF_F2R1	0/1:193,10:0.052:7:3:0.300:0|1:85610745_C_T:5493,285:99:94	0/0:442,20:0.042:8:12:0.600:0|1:85610745_C_T:12655,555:222:220	0.2	Tolerated	-1.45	Neutral	93	30	0.171	Tolerated	2.86	66
T12,T22,T2,T3,T42	chr1	**5**	**62.5**	85626148	85626148	T	C	exonic	**Sp140**	ENSMUSP00000108782	.	nonsynonymous SNV	Sp140:NM_001013817:exon8:c.T707C:p.M236T	**M236T**	ENSMUSP00000108782,236,M,T	1,85626148,T,C	1qC5	.	0.25	.	253	chr1	85626148	.	T	C	.	alt_allele_in_normal;clustered_events;str_contraction;triallelic_site	ECNT=15;HCNT=3;MAX_ED=140;MIN_ED=12;NLOD=33.86;RPA=2,1;RU=AGA;STR;TLOD=38.43	GT:AD:AF:ALT_F1R2:ALT_F2R1:FOXOG:QSS:REF_F1R2:REF_F2R1	0/1:174,22:0.120:17:5:.:4990,642:84:90	0/0:343,23:0.068:8:15:.:9680,664:158:185	0.2	Tolerated	-0.49	Neutral	93	30	0.638	Tolerated	2.85	72
T12,T1,T32,T3,T42	chr4	**5**	**62.5**	41196787	41196787	-	A	exonic	**Ubap2**	ENSMUSP00000047105	.	frameshift insertion	Ubap2:NM_026872:exon23:c.2574dupT:p.A859fs	**A859fs**	ENSMUSP00000047105,859,A,	4,41196787,C,T	4qA5	.	0.25	.	28	chr4	41196787	.	C	T	.	clustered_events	ECNT=2;HCNT=2;MAX_ED=146;MIN_ED=146;NLOD=15.19;TLOD=35.59	GT:AD:AF:ALT_F1R2:ALT_F2R1:FOXOG:QSS:REF_F1R2:REF_F2R1	0/1:76,21:0.128:11:10:.:2102,570:32:44	0/0:54,0:0.00:0:0:.:1526,0:32:22	1.4	Tolerated	-0.8	Neutral	169	30	0.125	Tolerated	2.85	167
T12,T22,T2,T32,T42	chr4	**5**	**62.5**	41195672	41195744	CCTAGGAAAAAAAACAGCTGTCACACAGGAGACTACTACTCCAGGTTCCAGTCCCGGAGAGGAGCTGACATTA	-	exonic	**Ubap2**	ENSMUSP00000047105	.	frameshift deletion	Ubap2:NM_026872:exon27:c.3047_3047del:p.G1016fs	**G1016fs**	ENSMUSP00000047105,1016,G,.	4,41195672,TCCTAGGAAAAAAAACAGCTGTCACACAGGAGACTACTACTCCAGGTTCCAGTCCCGGAGAGGAGCTGACATTA,GTTTTTTTTAGCTGCTAGTCACTGAGCCCTTACTGTGATCCGATGAGTTGGAAGAGATGGTTTGTATTTACAGTGT	4qA5	.	0.25	.	54	chr4	41195671	.	TCCTAGGAAAAAAAACAGCTGTCACACAGGAGACTACTACTCCAGGTTCCAGTCCCGGAGAGGAGCTGACATTA	GTTTTTTTTAGCTGCTAGTCACTGAGCCCTTACTGTGATCCGATGAGTTGGAGAGATGGTTTGTATTTACAGTGT	.	PASS	ECNT=1;HCNT=2;MAX_ED=.;MIN_ED=.;NLOD=11.44;TLOD=15.28	GT:AD:AF:ALT_F1R2:ALT_F2R1:FOXOG:QSS:REF_F1R2:REF_F2R1	0/1:49,5:0.086:2:3:.:1382,135:29:20	0/0:40,0:0.00:0:0:.:1097,0:14:26	1.4	Unknown	NA	NA	169	30	NA	NA	NA	NA
T12,T1,T3,T42	chr10	**4**	**50**	80534537	80534537	A	T	exonic	**Atp8b3**	ENSMUSP00000086398	.	nonsynonymous SNV	Atp8b3:NM_026094:exon5:c.T257A:p.I86N	**I86N**	ENSMUSP00000086398,86,I,N	10,80534537,A,C	10qC1	.	0.25	.	27	chr10	80534537	.	A	C	.	alt_allele_in_normal;clustered_events	ECNT=10;HCNT=1;MAX_ED=31;MIN_ED=9;NLOD=3.33;TLOD=18.23	GT:AD:AF:ALT_F1R2:ALT_F2R1:FOXOG:PGT:PID:QSS:REF_F1R2:REF_F2R1	0/1:65,6:0.090:0:6:.:0|1:80534525_G_T:1724,164:41:24	0/0:27,1:0.038:0:1:.:0|1:80534525_G_T:734,27:17:10	0.6	Deleterious	-4.72	Deleterious	145	30	0	Damaging	2.79	175
T12,T1,T3,T42	chr10	**4**	**50**	80534534	80534534	-	T	exonic	**Atp8b3**	ENSMUSP00000140012	.	frameshift insertion	Atp8b3:NM_026094:exon5:c.259_260insA:p.P87fs	**P87fs**	ENSMUSP00000140012,87,P,	10,80534534,G,A	10qC1	.	0.25	.	26	chr10	80534534	.	G	A	.	PASS	DB;ECNT=1;HCNT=1;MAX_ED=.;MIN_ED=.;NLOD=10.48;TLOD=64.39	GT:AD:AF:ALT_F1R2:ALT_F2R1:FOXOG:QSS:REF_F1R2:REF_F2R1	0/1:27,26:0.490:14:12:0.538:763,719:20:7	0/0:38,0:0.00:0:0:.:1036,0:16:22	4	Deleterious	-8.27	Deleterious	145	30	0.001	Damaging	2.81	175
T12,T1,T3,T42	chr10	**4**	**50**	80534525	80534525	G	T	exonic	**Atp8b3**	ENSMUSP00000086398	.	nonsynonymous SNV	Atp8b3:NM_026094:exon5:c.C269A:p.S90Y	**S90Y**	ENSMUSP00000086398,90,S,Y	10,80534525,G,C	10qC1	.	0.25	.	28	chr10	80534525	.	G	C	.	alt_allele_in_normal;clustered_events	ECNT=10;HCNT=1;MAX_ED=31;MIN_ED=9;NLOD=2.72;TLOD=18.15	GT:AD:AF:ALT_F1R2:ALT_F2R1:FOXOG:PGT:PID:QSS:REF_F1R2:REF_F2R1	0/1:62,6:0.087:0:6:0.00:0|1:80534525_G_T:1733,87:41:21	0/0:26,1:0.042:0:1:0.00:0|1:80534525_G_T:702,28:17:9	0.6	Deleterious	-4.02	Deleterious	145	30	0	Damaging	2.81	174
T12,T1,T3,T42	chr10	**4**	**50**	80534538	80534538	T	C	exonic	**Atp8b3**	ENSMUSP00000086398	.	nonsynonymous SNV	Atp8b3:NM_026094:exon5:c.A256G:p.I86V	**I86V**	ENSMUSP00000086398,86,I,V	10,80534538,T,GT	10qC1	.	0.25	.	27	chr10	80534538	.	T	GT	.	alt_allele_in_normal;clustered_events	ECNT=10;HCNT=1;MAX_ED=31;MIN_ED=9;NLOD=3.63;TLOD=18.11	GT:AD:AF:ALT_F1R2:ALT_F2R1:FOXOG:PGT:PID:QSS:REF_F1R2:REF_F2R1	0/1:64,6:0.086:0:6:.:0|1:80534525_G_T:1703,165:39:25	0/0:25,1:0.037:0:1:.:0|1:80534525_G_T:658,27:16:9	0.6	Unknown	NA	NA	145	30	NA	NA	NA	NA
T12,T1,T3,T42	chr10	**4**	**50**	80534542	80534543	CT	-	splicing	**Atp8b3**	ENSMUSP00000086398	.	.	.	**NA**	10,80534542,CCT,C	NA	10qC1	.	0.25	.	28	chr10	80534541	.	CCT	C	.	clustered_events;homologous_mapping_event;t_lod_fstar	ECNT=4;HCNT=4;MAX_ED=108;MIN_ED=6;NLOD=6.66;TLOD=5.35	GT:AD:AF:ALT_F1R2:ALT_F2R1:FOXOG:PGT:PID:QSS:REF_F1R2:REF_F2R1	0/1:50,2:0.040:1:1:0.500:0|1:113844430_G_T:1397,50:29:21	0/0:35,0:0.030:0:0:.:0|1:113844430_G_T:974,0:22:13	0.4	Unkown	.	.	.	.	.	.	.	.

T1,T22,T2,T4	chr4	**4**	**50**	126043744	126043744	A	G	exonic	**Csf3r**	ENSMUSP0000012945 7	.	nonsynonymous SNV	Csf3r:NM_001252651:exon15:c.A2096G:p.N699S,Csf3r:NM_007782:exon17:c.A2420G:p.N807S	**N807S**	ENSMUSP00000129457,807,N,S	4,126043744,A,T	4qD2.2	.	0.25	.	43	chr4	126043744	.	A	T	.	t_lod_fstar	ECNT=1;HCNT=4;MAX_ED=.;MIN_ED=.;NLOD=10.43;TLOD=5.06	GT:AD:AF:ALT_F1R2:ALT_F2R1:FOXOG:QSS:REF_F1R2:REF_F2R1	0/1:30,3:0.097:0:3:1.00:838,83:17:13	0/0:40,1:0.026:0:1:1.00:1108,16:19:21	1	Damaging	-2.44	Neutral	121	30	0.005	Damaging	2.88	34
T1,T22,T2,T4	chr4	**4**	**50**	126043714	126043714	C	A	exonic	**Csf3r**	ENSMUSP0000012945 7	.	nonsynonymous SNV	Csf3r:NM_001252651:exon15:c.C2066A:p.P689H,Csf3r:NM_007782:exon17:c.C2390A:p.P797H	**P689H**	ENSMUSP00000129457,689,P,H	4,126043714,C,A	4qD2.2	.	0.25	.	41	chr4	126043714	.	C	A	.	clustered_events;homologous_mapping_event;t_lod_fstar	ECNT=3;HCNT=8;MAX_ED=71;MIN_ED=30;NLOD=11.13;TLOD=5.36	GT:AD:AF:ALT_F1R2:ALT_F2R1:FOXOG:QSS:REF_F1R2:REF_F2R1	0/1:31,4:0.114:1:3:0.750:857,97:15:16	0/0:41,0:0.00:0:0:.:1124,0:22:19	1	Deleterious	-3.04	Deleterious	121	30	0.011	Damaging	2.88	34
T1,T22,T2,T4	chr4	**4**	**50**	126043714	126043714	C	A	exonic	**Csf3r**	ENSMUSP0000012945 7	.	nonsynonymous SNV	Csf3r:NM_001252651:exon15:c.C2066A:p.P689H,Csf3r:NM_007782:exon17:c.C2390A:p.P797H	**P797H**	ENSMUSP00000129457,797,P,H	4,126043714,C,T	4qD2.2	.	0.25	.	41	chr4	126043714	.	C	T	.	t_lod_fstar	ECNT=1;HCNT=4;MAX_ED=.;MIN_ED=.;NLOD=10.43;TLOD=5.06	GT:AD:AF:ALT_F1R2:ALT_F2R1:FOXOG:QSS:REF_F1R2:REF_F2R1	0/1:30,3:0.097:0:3:1.00:838,83:17:13	0/0:40,1:0.026:0:1:1.00:1108,16:19:21	1	Deleterious	-3.46	Deleterious	121	30	0.005	Damaging	2.88	34
T1,T22,T2,T4	chr4	**4**	**50**	126043744	126043744	A	G	exonic	**Csf3r**	ENSMUSP0000012945 7	.	nonsynonymous SNV	Csf3r:NM_001252651:exon15:c.A2096G:p.N699S,Csf3r:NM_007782:exon17:c.A2420G:p.N807S	**N699S**	ENSMUSP00000129457,699,N,S	4,126043744,A,G	4qD2.2	.	0.25	.	43	chr4	126043744	.	A	G	.	clustered_events;homologous_mapping_event;t_lod_fstar	ECNT=3;HCNT=8;MAX_ED=71;MIN_ED=30;NLOD=11.43;TLOD=5.02	GT:AD:AF:ALT_F1R2:ALT_F2R1:FOXOG:QSS:REF_F1R2:REF_F2R1	0/1:36,3:0.081:1:2:0.667:1001,87:17:19	0/0:43,0:0.00:0:0:.:1155,0:20:23	1	Tolerated	-0.15	Neutral	121	30	0.597	Tolerated	2.88	34
T1,T32,T42,T4	chr5	**4**	**50**	136373432	136373432	-	TTTTAATGATACG	exonic	**Cux1**	ENSMUSP0000003067 3	.	stopgain	Cux1:NM_001291233:exon5:c.583_584insCGTATCATTAAAA:p.Q195_Q196delinsPYHX,Cux1:NM_001291234:exon5:c.328_329insCGTATCATTAAAA:p.Q110_Q111delinsPYHX,Cux1:NM_001291238:exon5:c.328_329insCGTATCATTAAAA:p.Q110_Q111delinsPYHX,Cux1:NM_001291239:exon5:c.583_584insCGTATCATTAAAA:p.Q195_Q196delinsPYHX,Cux1:NM_001291240:exon5:c.583_584insCGTATCATTAAAA:p.Q195_Q196delinsPYHX,Cux1:NM_009986:exon5:c.328_329insCGTATCATTAAAA:p.Q110_Q111delinsPYHX,Cux1:NM_198602:exon5:c.328_329insCGTATCATTAAAA:p.Q110_Q111delinsPYHX	**Q110_Q111delinsPYHX**	ENSMUSP00000030673,110,QQ,PYHX	NA	5qG2	.	0.25	.	38	chr5	136373432	.	T	G	.	PASS	DB;ECNT=1;HCNT=1;MAX_ED=.;MIN_ED=.;NLOD=8.97;TLOD=49.70	GT:AD:AF:ALT_F1R2:ALT_F2R1:FOXOG:QSS:REF_F1R2:REF_F2R1	0/1:14,23:0.600:11:12:0.522:339,550:5:9	0/0:33,0:0.00:0:0:.:819,0:22:11	0.2	Deletarious	.	.	.	.	.	.	.	.
T1,T32,T42,T4	chr5	**4**	**50**	136373432	136373432	-	TTTTAATGATACG	exonic	**Cux1**	ENSMUSP0000003067 3	.	stopgain	Cux1:NM_001291233:exon5:c.583_584insCGTATCATTAAAA:p.Q195_Q196delinsPYHX,Cux1:NM_001291234:exon5:c.328_329insCGTATCATTAAAA:p.Q110_Q111delinsPYHX,Cux1:NM_001291238:exon5:c.328_329insCGTATCATTAAAA:p.Q110_Q111delinsPYHX,Cux1:NM_001291239:exon5:c.583_584insCGTATCATTAAAA:p.Q195_Q196delinsPYHX,Cux1:NM_001291240:exon5:c.583_584insCGTATCATTAAAA:p.Q195_Q196delinsPYHX,Cux1:NM_009986:exon5:c.328_329insCGTATCATTAAAA:p.Q110_Q111delinsPYHX,Cux1:NM_198602:exon5:c.328_329insCGTATCATTAAAA:p.Q110_Q111delinsPYHX	**Q195_Q196delinsPYHX**	ENSMUSP00000030673,195,QQ,PYHX	NA	5qG2	.	0.25	.	38	chr5	136373432	.	T	T	.	t_lod_fstar	ECNT=1;HCNT=1;MAX_ED=.;MIN_ED=.;NLOD=14.95;TLOD=5.00	GT:AD:AF:ALT_F1R2:ALT_F2R1:FOXOG:QSS:REF_F1R2:REF_F2R1	0/1:62,3:0.051:3:0:1.00:1737,87:29:33	0/0:54,1:0.019:0:1:0.00:1561,14:25:29	1.1	Deletarious	.	.	.	.	.	.	.	.

T1,T32,T42,T4	chr5	**4**	**50**	136373443	136373452	CAGGGCTGGT	-	exonic	**Cux1**	ENSMUSP00000039819	.	frameshift deletion	Cux1:NM_001291233:exon5:c.564_573del:p.V188fs,Cux1:NM_001291234:exon5:c.309_318del:p.V103fs,Cux1:NM_001291238:exon5:c.309_318del:p.V103fs,Cux1:NM_001291239:exon5:c.564_573del:p.V188fs,Cux1:NM_001291240:exon5:c.564_573del:p.V188fs,Cux1:NM_009986:exon5:c.309_318del:p.V103fs,Cux1:NM_198602:exon5:c.309_318del:p.V103fs	**V188fs**	ENSMUSP00000039819,188,V,.	5,136373443,CCAGGGCTGGT,C	5qG2	.	0.25	.	39	chr5	136373442	.	CCAGGGCTGGT	C	.	alt_allele_in_normal;clustered_events	ECNT=5;HCNT=1;MAX_ED=24;MIN_ED=1;NLOD=5.43;TLOD=11.84	GT:AD:AF:ALT_F1R2:ALT_F2R1:FOXOG:PGT:PID:QSS:REF_F1R2:REF_F2R1	0/1:50,4:0.075:4:0:.:0|1:136373432_T_TTTTTAATGATACG:1253,57:21:29	0/0:37,1:0.030:1:0:.:0|1:136373432_T_TTTTTAATGATACG:938,22:13:24	1.4	Deleterious	-7.52	Deleterious	123	30	NA	NA	NA	NA
T1,T32,T42,T4	chr5	**4**	**50**	136373456	136373456	-	A	exonic	**Cux1**	ENSMUSP00000030673	.	frameshift insertion	Cux1:NM_001291233:exon5:c.559_560insT:p.P187fs,Cux1:NM_001291234:exon5:c.304_305insT:p.P102fs,Cux1:NM_001291238:exon5:c.304_305insT:p.P102fs,Cux1:NM_001291239:exon5:c.559_560insT:p.P187fs,Cux1:NM_001291240:exon5:c.559_560insT:p.P187fs,Cux1:NM_009986:exon5:c.304_305insT:p.P102fs,Cux1:NM_198602:exon5:c.304_305insT:p.P102fs	**P102fs**	ENSMUSP00000030673,102,P,	5,136373456,G,TTTTTAATGATACG	5qG2	.	0.25	.	38	chr5	136373456	.	G	TTTTTAATGATACG	.	alt_allele_in_normal;clustered_events	ECNT=5;HCNT=1;MAX_ED=24;MIN_ED=1;NLOD=5.13;TLOD=11.88	GT:AD:AF:ALT_F1R2:ALT_F2R1:FOXOG:PGT:PID:QSS:REF_F1R2:REF_F2R1	0/1:48,4:0.077:4:0:.:0|1:136373432_T_TTTTTAATGATACG:1366,103:22:26	0/0:37,1:0.031:1:0:.:0|1:136373432_T_TTTTTAATGATACG:1013,26:13:24	1.4	Unknown	NA	NA	144	30	NA	NA	NA	NA
T1,T32,T42,T4	chr5	**4**	**50**	136373456	136373456	-	A	exonic	**Cux1**	ENSMUSP00000030673	.	frameshift insertion	Cux1:NM_001291233:exon5:c.559_560insT:p.P187fs,Cux1:NM_001291234:exon5:c.304_305insT:p.P102fs,Cux1:NM_001291238:exon5:c.304_305insT:p.P102fs,Cux1:NM_001291239:exon5:c.559_560insT:p.P187fs,Cux1:NM_001291240:exon5:c.559_560insT:p.P187fs,Cux1:NM_009986:exon5:c.304_305insT:p.P102fs,Cux1:NM_198602:exon5:c.304_305insT:p.P102fs	**P187fs**	ENSMUSP00000030673,187,P,	5,136373456,G,TTTTTAATGATACG	5qG2	.	0.25	.	38	chr5	136373456	.	G	TTTTTAATGATACG	.	alt_allele_in_normal;clustered_events	ECNT=5;HCNT=1;MAX_ED=24;MIN_ED=1;NLOD=5.13;TLOD=11.88	GT:AD:AF:ALT_F1R2:ALT_F2R1:FOXOG:PGT:PID:QSS:REF_F1R2:REF_F2R1	0/1:48,4:0.077:4:0:.:0|1:136373432_T_TTTTTAATGATACG:1366,103:22:26	0/0:37,1:0.031:1:0:.:0|1:136373432_T_TTTTTAATGATACG:1013,26:13:24	1.4	Unknown	NA	NA	144	30	NA	NA	NA	NA
T1,T32,T42,T4	chr5	**4**	**50**	136373434	136373435	CC	-	exonic	**Cux1**	ENSMUSP00000030673	.	frameshift deletion	Cux1:NM_001291233:exon5:c.581_582del:p.G194fs,Cux1:NM_001291234:exon5:c.326_327del:p.G109fs,Cux1:NM_001291238:exon5:c.326_327del:p.G109fs,Cux1:NM_001291239:exon5:c.581_582del:p.G194fs,Cux1:NM_001291240:exon5:c.581_582del:p.G194fs,Cux1:NM_009986:exon5:c.326_327del:p.G109fs,Cux1:NM_198602:exon5:c.326_327del:p.G109fs	**G109fs**	ENSMUSP00000030673,109,G,.	5,136373434,GCC,C	5qG2	.	0.25	.	39	chr5	136373433	.	GCC	C	.	clustered_events;multi_event_alt_allele_in_normal	ECNT=5;HCNT=1;MAX_ED=24;MIN_ED=1;NLOD=5.74;TLOD=11.71	GT:AD:AF:ALT_F1R2:ALT_F2R1:FOXOG:PGT:PID:QSS:REF_F1R2:REF_F2R1	0/1:56,4:0.070:4:0:.:0|1:136373432_T_TTTTTAATGATACG:1524,79:26:30	0/0:38,1:0.029:1:0:.:0|1:136373432_T_TTTTTAATGATACG:1057,28:13:25	1.4	Unknown	NA	NA	144	30	NA	NA	NA	NA
T1,T32,T42,T4	chr5	**4**	**50**	136373434	136373435	CC	-	exonic	**Cux1**	ENSMUSP00000030673	.	frameshift deletion	Cux1:NM_001291233:exon5:c.581_582del:p.G194fs,Cux1:NM_001291234:exon5:c.326_327del:p.G109fs,Cux1:NM_001291238:exon5:c.326_327del:p.G109fs,Cux1:NM_001291239:exon5:c.581_582del:p.G194fs,Cux1:NM_001291240:exon5:c.581_582del:p.G194fs,Cux1:NM_009986:exon5:c.326_327del:p.G109fs,Cux1:NM_198602:exon5:c.326_327del:p.G109fs	**G194fs**	ENSMUSP00000030673,194,G,.	5,136373434,GCC,C	5qG2	.	0.25	.	39	chr5	136373433	.	GCC	C	.	clustered_events;multi_event_alt_allele_in_normal	ECNT=5;HCNT=1;MAX_ED=24;MIN_ED=1;NLOD=5.74;TLOD=11.71	GT:AD:AF:ALT_F1R2:ALT_F2R1:FOXOG:PGT:PID:QSS:REF_F1R2:REF_F2R1	0/1:56,4:0.070:4:0:.:0|1:136373432_T_TTTTTAATGATACG:1524,79:26:30	0/0:38,1:0.029:1:0:.:0|1:136373432_T_TTTTTAATGATACG:1057,28:13:25	1.4	Unknown	NA	NA	144	30	NA	NA	NA	NA

T1,T32,T42,T4	chr5	**4**	**50**	136373440	136373441	GT	-	exonic	**Cux1**	ENSMUSP00000030673	.	frameshift deletion	Cux1:NM_001291233:exon5:c.575_576del:p.D192fs,Cux1:NM_001291234:exon5:c.320_321del:p.D107fs,Cux1:NM_001291238:exon5:c.320_321del:p.D107fs,Cux1:NM_001291239:exon5:c.575_576del:p.D192fs,Cux1:NM_001291240:exon5:c.575_576del:p.D192fs,Cux1:NM_009986:exon5:c.320_321del:p.D107fs,Cux1:NM_198602:exon5:c.320_321del:p.D107fs	**D192fs**	ENSMUSP00000030673,192,D,.	5,136373440,CGT,G	5qG2	.	0.25	.	38	chr5	136373439	.	CGT	G	.	alt_allele_in_normal;clustered_events	ECNT=5;HCNT=1;MAX_ED=24;MIN_ED=1;NLOD=5.43;RPA=3,1;RU=C;STR;TLOD=11.81	GT:AD:AF:ALT_F1R2:ALT_F2R1:FOXOG:PGT:PID:QSS:REF_F1R2:REF_F2R1	0/1:49,4:0.074:4:0:.:0|1:136373432_T_TTTTTAATGATACG:1370,115:23:26	0/0:38,1:0.030:1:0:.:0|1:136373432_T_TTTTTAATGATACG:1066,27:14:24	1.4	Unknown	NA	NA	144	30	NA	NA	NA	NA
T1,T32,T42,T4	chr5	**4**	**50**	136373440	136373441	GT	-	exonic	**Cux1**	ENSMUSP00000030673	.	frameshift deletion	Cux1:NM_001291233:exon5:c.575_576del:p.D192fs,Cux1:NM_001291234:exon5:c.320_321del:p.D107fs,Cux1:NM_001291238:exon5:c.320_321del:p.D107fs,Cux1:NM_001291239:exon5:c.575_576del:p.D192fs,Cux1:NM_001291240:exon5:c.575_576del:p.D192fs,Cux1:NM_009986:exon5:c.320_321del:p.D107fs,Cux1:NM_198602:exon5:c.320_321del:p.D107fs	**D107fs**	ENSMUSP00000030673,107,D,.	5,136373440,CGT,GA	5qG2	.	0.25	.	38	chr5	136373439	.	CGT	GA	.	alt_allele_in_normal;clustered_events	ECNT=5;HCNT=1;MAX_ED=24;MIN_ED=1;NLOD=5.13;TLOD=11.84	GT:AD:AF:ALT_F1R2:ALT_F2R1:FOXOG:PGT:PID:QSS:REF_F1R2:REF_F2R1	0/1:50,4:0.075:4:0:.:0|1:136373432_T_TTTTTAATGATACG:1203,95:22:28	0/0:37,1:0.031:1:0:.:0|1:136373432_T_TTTTTAATGATACG:859,26:13:24	1.4	Unknown	NA	NA	144	30	NA	NA	NA	NA
T1,T32,T42,T4	chr5	**4**	**50**	136373443	136373452	CAGGGCTGGT	-	exonic	**Cux1**	ENSMUSP00000030673	.	frameshift deletion	Cux1:NM_001291233:exon5:c.564_573del:p.V188fs,Cux1:NM_001291234:exon5:c.309_318del:p.V103fs,Cux1:NM_001291238:exon5:c.309_318del:p.V103fs,Cux1:NM_001291239:exon5:c.564_573del:p.V188fs,Cux1:NM_001291240:exon5:c.564_573del:p.V188fs,Cux1:NM_009986:exon5:c.309_318del:p.V103fs,Cux1:NM_198602:exon5:c.309_318del:p.V103fs	**V103fs**	ENSMUSP00000030673,103,V,.	5,136373443,CCAGGGCTGGT,GA	5qG2	.	0.25	.	39	chr5	136373442	.	CCAGGGCTGGT	GA	.	alt_allele_in_normal;clustered_events	ECNT=5;HCNT=1;MAX_ED=24;MIN_ED=1;NLOD=5.13;TLOD=11.84	GT:AD:AF:ALT_F1R2:ALT_F2R1:FOXOG:PGT:PID:QSS:REF_F1R2:REF_F2R1	0/1:50,4:0.075:4:0:.:0|1:136373432_T_TTTTTAATGATACG:1203,95:22:28	0/0:37,1:0.031:1:0:.:0|1:136373432_T_TTTTTAATGATACG:859,26:13:24	1.4	Unknown	.	.	.	.	.	.	.	.
T22, T2, T3,T42	chr9	**4**	**50**	66271693	66271693	A	C	exonic	**Dapk2**	ENSMUSP00000135086	.	nonsynonymous SNV	Dapk2:NM_010019:exon12:c.A1099C:p.S367R	**S367R**	ENSMUSP00000135086,367,S,R	9,66271693,A,G	9qC	.	0.25	.	40	chr9	66271693	.	A	G	.	PASS	ECNT=1;HCNT=1;MAX_ED=.;MIN_ED=.;NLOD=12.59;TLOD=6.94	GT:AD:AF:ALT_F1R2:ALT_F2R1:FOXOG:QSS:REF_F1R2:REF_F2R1	0/1:47,4:0.083:0:4:1.00:1331,110:24:23	0/0:46,0:0.00:0:0:.:1292,0:25:21	19	Damaging	-1.28	Neutral	182	30	0	Damaging	3.52	30
T12,T1,T32,T4	chr10	**4**	**50**	81178211	81178211	T	C	exonic	**Eef2**	ENSMUSP00000132786	.	nonsynonymous SNV	Eef2:NM_007907:exon3:c.T377C:p.L126P	**L126P**	ENSMUSP00000132786,126,L,P	10,81178211,T,A	10qC1	.	0.25	.	348	chr10	81178211	.	T	A	.	alt_allele_in_normal;clustered_events;t_lod_fstar	ECNT=3;HCNT=16;MAX_ED=95;MIN_ED=34;NLOD=47.30;TLOD=5.70	GT:AD:AF:ALT_F1R2:ALT_F2R1:FOXOG:QSS:REF_F1R2:REF_F2R1	0/1:202,5:0.024:1:4:0.200:5528,145:103:99	0/0:265,9:0.035:4:5:0.444:6774,255:109:156	0.7	Deleterious	-5.32	Deleterious	706	30	0	Damaging	3.61	380
T12,T1,T42,T4	chr3	**4**	**50**	142572523	142572523	T	C	exonic	**Gbp3**	ENSMUSP00000034859	.	nonsynonymous SNV	Gbp3:NM_001289493:exon11:c.T1694C:p.L565S,Gbp3:NM_018734:exon11:c.T1694C:p.L565S,Gbp3:NM_001289492:exon12:c.T1694C:p.L565S,Gbp3:NM_001355403:exon12:c.T1694C:p.L565S	**L565S**	ENSMUSP00000034859,565,L,S	3,142572523,T,A	3qH1	.	0.25	.	64	chr3	142572523	.	T	A	.	clustered_events;multi_event_alt_allele_in_normal;t_lod_fstar	ECNT=2;HCNT=46;MAX_ED=3;MIN_ED=3;NLOD=10.41;TLOD=4.62	GT:AD:AF:ALT_F1R2:ALT_F2R1:FOXOG:PGT:PID:QSS:REF_F1R2:REF_F2R1	0/1:43,3:0.065:2:1:0.333:0|1:142572539_T_A:1194,83:27:16	0/0:47,1:0.022:0:1:1.00:0|1:142572539_T_A:1280,27:27:20	0.9	Unknown	NA	NA	142	30	NA	NA	NA	NA
T12,T1,T22,T4	chr9	**4**	**50**	107615768	107615768	-	CA	exonic	**Gnai2**	ENSMUSP00000019198	.	frameshift insertion	Gnai2:NM_008138:exon8:c.934_935insTG:p.N312fs	**N312fs**	ENSMUSP00000019198,312,N,	9,107615768,T,C	9qF1	.	0.25	.	38	chr9	107615768	.	T	C	.	alt_allele_in_normal;clustered_events	ECNT=8;HCNT=1;MAX_ED=24;MIN_ED=6;NLOD=4.53;TLOD=21.23	GT:AD:AF:ALT_F1R2:ALT_F2R1:FOXOG:PGT:PID:QSS:REF_F1R2:REF_F2R1	0/1:76,7:0.089:0:7:.:0|1:107615748_CCTT_C:2121,199:43:33	0/0:35,1:0.033:0:1:.:0|1:107615748_CCTT_C:983,29:19:16	0.4	Deleterious	-4.46	Deleterious	573	30	0.003	Damaging	3.66	394
T12,T1,T22,T4	chr9	**4**	**50**	107615770	107615770	-	AAAA	exonic	**Gnai2**	ENSMUSP00000019198	.	frameshift insertion	Gnai2:NM_008138:exon8:c.932_933insTTTT:p.L311fs	**L311fs**	ENSMUSP00000019198,311,L,	9,107615770,T,C	9qF1	.	0.25	.	37	chr9	107615770	.	T	C	.	alt_allele_in_normal;clustered_events	ECNT=8;HCNT=1;MAX_ED=24;MIN_ED=6;NLOD=4.53;TLOD=21.08	GT:AD:AF:ALT_F1R2:ALT_F2R1:FOXOG:PGT:PID:QSS:REF_F1R2:REF_F2R1	0/1:80,7:0.084:0:7:.:0|1:107615748_CCTT_C:2252,111:48:32	0/0:35,1:0.033:0:1:.:0|1:107615748_CCTT_C:988,22:19:16	0.4	Tolerated	0	Neutral	573	30	1	Tolerated	3.66	393

T12,T1,T22,T4	chr9	**4**	**50**	107615765	107615765	T	G	exonic	**Gnai2**	ENSMUSP00000019198	.	nonsynonymous SNV	Gnai2:NM_008138:exon8:c.A938C:p.K313T	**K313T**	ENSMUSP00000019198,313,K,T	9,107615765,T,TG	9qF1	.	0.25	.	37	chr9	107615765	.	T	TG	.	alt_allele_in_normal;clustered_events	ECNT=8;HCNT=1;MAX_ED=24;MIN_ED=6;NLOD=4.23;RPA=1,2;RU=G;STR;TLOD=21.23	GT:AD:AF:ALT_F1R2:ALT_F2R1:FOXOG:PGT:PID:QSS:REF_F1R2:REF_F2R1	0/1:74,7:0.089:0:7:.:0|1:107615748_CCTT_C:2118,199:44:30	0/0:34,1:0.034:0:1:.:0|1:107615748_CCTT_C:960,29:19:15	0.4	Unknown	NA	NA	573	30	NA	NA	NA	NA
T12,T1,T22,T4	chr9	**4**	**50**	107615754	107615754	-	G	exonic	**Gnai2**	ENSMUSP00000019198	.	frameshift insertion	Gnai2:NM_008138:exon8:c.948dupC:p.T317fs	**T317fs**	ENSMUSP00000019198,317,T,	9,107615754,T,TAAAA	9qF1	.	0.25	.	35	chr9	107615754	.	T	TAAAA	.	alt_allele_in_normal;clustered_events	ECNT=8;HCNT=1;MAX_ED=24;MIN_ED=6;NLOD=4.23;RPA=1,5;RU=A;STR;TLOD=21.15	GT:AD:AF:ALT_F1R2:ALT_F2R1:FOXOG:PGT:PID:QSS:REF_F1R2:REF_F2R1	0/1:80,7:0.089:0:7:.:0|1:107615748_CCTT_C:2148,189:48:32	0/0:36,1:0.037:0:1:.:0|1:107615748_CCTT_C:961,28:20:15	0.4	Unknown	NA	NA	573	30	NA	NA	NA	NA
T12,T1,T22,T4	chr9	**4**	**50**	107615763	107615763	G	-	exonic	**Gnai2**	ENSMUSP00000019198	.	frameshift deletion	Gnai2:NM_008138:exon8:c.940delC:p.R314fs	**R314fs**	ENSMUSP00000019198,314,R,.	9,107615763,CG,TCA	9qF1	.	0.25	.	36	chr9	107615762	.	CG	TCA	.	alt_allele_in_normal;clustered_events	ECNT=8;HCNT=1;MAX_ED=24;MIN_ED=6;NLOD=4.23;TLOD=21.11	GT:AD:AF:ALT_F1R2:ALT_F2R1:FOXOG:PGT:PID:QSS:REF_F1R2:REF_F2R1	0/1:82,7:0.085:0:7:.:0|1:107615748_CCTT_C:2267,91:50:32	0/0:37,1:0.034:0:1:.:0|1:107615748_CCTT_C:1003,26:21:16	0.4	Unknown	NA	NA	573	30	NA	NA	NA	NA
T12,T1,T22,T4	chr9	**4**	**50**	107615749	107615751	CTT	-	exonic	**Gnai2**	ENSMUSP00000019198	.	nonframeshift deletion	Gnai2:NM_008138:exon8:c.952_954del:p.318_318del	**318_318del**	NA	9,107615749,CCTT,C	9qF1	.	0.25	.	36	chr9	107615748	.	CCTT	C	.	alt_allele_in_normal;germline_risk	DB;ECNT=1;HCNT=8;MAX_ED=.;MIN_ED=.;NLOD=4.99;TLOD=9.36	GT:AD:AF:ALT_F1R2:ALT_F2R1:FOXOG:QSS:REF_F1R2:REF_F2R1	0/1:27,9:0.250:6:3:0.667:706,146:13:14	0/0:28,3:0.111:1:2:0.333:729,24:10:18	0.3	Unknown	.	.	.	.	.	.	.	.
T12,T1,T22,T4	chr9	**4**	**50**	107615758	107615760	TTT	-	exonic	**Gnai2**	ENSMUSP00000019198	.	nonframeshift deletion	Gnai2:NM_008138:exon8:c.943_945del:p.315_315del	**315_315del**	NA	9,107615758,CTTT,G	9qF1	.	0.25	.	36	chr9	107615757	.	CTTT	G	.	clustered_events;homologous_mapping_event;t_lod_fstar	DB;ECNT=6;HCNT=1;MAX_ED=22;MIN_ED=4;NLOD=11.14;TLOD=5.47	GT:AD:AF:ALT_F1R2:ALT_F2R1:FOXOG:PGT:PID:QSS:REF_F1R2:REF_F2R1	0/1:43,2:0.045:1:1:0.500:0|1:70640673_A_G:1142,42:17:26	0/0:36,0:0.00:0:0:.:0|1:70640673_A_G:937,0:16:20	0.3	Unknown	.	.	.	.	.	.	.	.
T12,T1,T3,T42	chr17	**4**	**50**	27107665	27107665	A	C	exonic	**Itpr3**	ENSMUSP00000151755	.	nonsynonymous SNV	Itpr3:NM_080553:exon32:c.A4280C:p.E1427A	**E1427A**	ENSMUSP00000151755,1427,E,A	17,27107665,A,C	17qA3.3	.	0.25	.	31	chr17	27107665	.	A	C	.	clustered_events;multi_event_alt_allele_in_normal	ECNT=9;HCNT=2;MAX_ED=24;MIN_ED=1;NLOD=3.84;TLOD=8.57	GT:AD:AF:ALT_F1R2:ALT_F2R1:FOXOG:PGT:PID:QSS:REF_F1R2:REF_F2R1	0/1:47,3:0.060:3:0:0.00:0|1:27107648_C_CTTTT:1220,41:21:26	0/0:30,1:0.036:1:0:0.00:0|1:27107648_C_CTTTT:791,17:19:11	1.7	Deleterious	-5.63	Deleterious	190	30	0	Damaging	2.91	224
T12,T1,T3,T42	chr17	**4**	**50**	27107668	27107668	T	C	exonic	**Itpr3**	ENSMUSP00000151755	.	nonsynonymous SNV	Itpr3:NM_080553:exon32:c.T4283C:p.M1428T	**M1428T**	ENSMUSP00000151755,1428,M,T	17,27107668,T,C	17qA3.3	.	0.25	.	29	chr17	27107668	.	T	C	.	clustered_events;multi_event_alt_allele_in_normal	ECNT=9;HCNT=2;MAX_ED=24;MIN_ED=1;NLOD=3.54;TLOD=8.60	GT:AD:AF:ALT_F1R2:ALT_F2R1:FOXOG:PGT:PID:QSS:REF_F1R2:REF_F2R1	0/1:48,3:0.061:3:0:1.00:0|1:27107648_C_CTTTT:1199,77:22:26	0/0:28,1:0.037:1:0:1.00:0|1:27107648_C_CTTTT:648,17:18:10	1.7	Deleterious	-5.46	Deleterious	190	30	0.001	Damaging	2.91	224
T12,T1,T3,T42	chr17	**4**	**50**	27107669	27107669	G	C	exonic	**Itpr3**	ENSMUSP00000151755	.	nonsynonymous SNV	Itpr3:NM_080553:exon32:c.G4284C:p.M1428I	**M1428I**	ENSMUSP00000151755,1428,M,I	17,27107669,G,C	17qA3.3	.	0.25	.	29	chr17	27107669	.	G	C	.	clustered_events;multi_event_alt_allele_in_normal	ECNT=9;HCNT=2;MAX_ED=24;MIN_ED=1;NLOD=3.24;TLOD=8.60	GT:AD:AF:ALT_F1R2:ALT_F2R1:FOXOG:PGT:PID:QSS:REF_F1R2:REF_F2R1	0/1:48,3:0.061:3:0:1.00:0|1:27107648_C_CTTTT:1234,64:22:26	0/0:28,1:0.038:1:0:1.00:0|1:27107648_C_CTTTT:716,11:18:10	1.7	Deleterious	-3.55	Deleterious	190	30	0.002	Damaging	2.91	224
T12,T1,T3,T42	chr17	**4**	**50**	27107672	27107672	G	C	exonic	**Itpr3**	ENSMUSP00000151755	.	nonsynonymous SNV	Itpr3:NM_080553:exon32:c.G4287C:p.K1429N	**K1429N**	ENSMUSP00000151755,1429,K,N	17,27107672,G,C	17qA3.3	.	0.25	.	28	chr17	27107672	.	G	C	.	alt_allele_in_normal;clustered_events	ECNT=9;HCNT=2;MAX_ED=24;MIN_ED=1;NLOD=2.94;TLOD=8.63	GT:AD:AF:ALT_F1R2:ALT_F2R1:FOXOG:PGT:PID:QSS:REF_F1R2:REF_F2R1	0/1:48,3:0.063:3:0:1.00:0|1:27107648_C_CTTTT:1242,50:21:27	0/0:27,1:0.040:1:0:1.00:0|1:27107648_C_CTTTT:694,22:17:10	1.7	Deleterious	-4.69	Deleterious	190	30	0	Damaging	2.91	224
T12,T1,T3,T42	chr17	**4**	**50**	27107649	27107649	G	T	exonic	**Itpr3**	ENSMUSP00000151755	.	nonsynonymous SNV	Itpr3:NM_080553:exon32:c.G4264T:p.V1422L	**V1422L**	ENSMUSP00000151755,1422,V,L	17,27107649,G,T	17qA3.3	.	0.25	.	31	chr17	27107649	.	G	T	.	clustered_events;multi_event_alt_allele_in_normal	ECNT=9;HCNT=2;MAX_ED=24;MIN_ED=1;NLOD=3.54;TLOD=8.60	GT:AD:AF:ALT_F1R2:ALT_F2R1:FOXOG:PGT:PID:QSS:REF_F1R2:REF_F2R1	0/1:48,3:0.061:3:0:1.00:0|1:27107648_C_CTTTT:1175,53:21:27	0/0:30,1:0.037:1:0:1.00:0|1:27107648_C_CTTTT:743,20:19:11	1.7	Tolerated	-2.21	Neutral	190	30	0.167	Tolerated	2.91	224

T12,T1,T3,T42	chr17	**4**	**50**	27107648	27107648	-	TTTT	exonic	**Itpr3**	ENSMUSP00000151755	.	frameshift insertion	Itpr3:NM_080553:exon32:c.4263_4264insTTTT:p.Y1421fs	**Y1421fs**	ENSMUSP00000151755,1421,Y,	17,27107648,C,C	17qA3.3	.	0.25	.	30	chr17	27107648	.	C	C	.	clustered_events;multi_event_alt_allele_in_normal	ECNT=9;HCNT=2;MAX_ED=24;MIN_ED=1;NLOD=4.15;TLOD=8.47	GT:AD:AF:ALT_F1R2:ALT_F2R1:FOXOG:PGT:PID:QSS:REF_F1R2:REF_F2R1	0/1:51,3:0.056:3:0:.:0|1:27107648_C_CTTTT:1114,37:23:28	0/0:31,1:0.034:1:0:.:0|1:27107648_C_CTTTT:703,17:20:11	1.7	Tolerated	0	Neutral	190	30	1	Tolerated	2.91	224
T12,T1,T3,T42	chr17	**4**	**50**	27107651	27107651	-	AT	exonic	**Itpr3**	ENSMUSP00000151755	.	frameshift insertion	Itpr3:NM_080553:exon32:c.4266_4267insAT:p.V1422fs	**V1422fs**	ENSMUSP00000151755,1422,V,	17,27107651,A,T	17qA3.3	.	0.25	.	31	chr17	27107651	.	A	T	.	clustered_events;homologous_mapping_event	ECNT=3;HCNT=2;MAX_ED=3;MIN_ED=1;NLOD=7.87;TLOD=10.16	GT:AD:AF:ALT_F1R2:ALT_F2R1:FOXOG:PGT:PID:QSS:REF_F1R2:REF_F2R1	0/1:49,4:0.075:1:3:0.250:0|1:98003232_G_T:1404,110:25:24	0/0:27,0:0.00:0:0:.:0|1:98003232_G_T:768,0:14:13	9	Tolerated	0	Neutral	190	30	1	Tolerated	2.91	224
T12,T1,T3,T42	chr17	**4**	**50**	27107657	27107662	GGAGGT	-	exonic	**Itpr3**	ENSMUSP00000151755	.	nonframeshift deletion	Itpr3:NM_080553:exon32:c.4272_4277del:p.1424_1426del	**1424_1426del**	NA	17,27107657,CGGAGGT,A	17qA3.3	.	0.25	.	32	chr17	27107656	.	CGGAGGT	A	.	clustered_events;homologous_mapping_event	ECNT=3;HCNT=2;MAX_ED=3;MIN_ED=1;NLOD=7.52;TLOD=8.46	GT:AD:AF:ALT_F1R2:ALT_F2R1:FOXOG:PGT:PID:QSS:REF_F1R2:REF_F2R1	0/1:52,1:0.056:0:1:1.00:0|1:98003232_G_T:1311,26:27:25	0/0:27,0:0.00:0:0:.:0|1:98003232_G_T:633,0:14:13	9	Unknown	.	.	.	.	.	.	.	.
T12,T1,T2,T4-2	chr2	**4**	**50**	90906178	90906178	C	A	exonic	**Kbtbd4**	ENSMUSP00000113088	.	nonsynonymous SNV	Kbtbd4:NM_001311116:exon2:c.C532A:p.L178I,Kbtbd4:NM_025991:exon2:c.C484A:p.L162I	**L178I**	ENSMUSP00000113088,178,L,I	NA	2qE1	.	0.25	.	38	chr2	90906178	.	C	G	.	alt_allele_in_normal;clustered_events;germline_risk	ECNT=6;HCNT=1;MAX_ED=25;MIN_ED=10;NLOD=1.23;TLOD=8.44	GT:AD:AF:ALT_F1R2:ALT_F2R1:FOXOG:PGT:PID:QSS:REF_F1R2:REF_F2R1	0/1:55,4:0.055:4:0:0.00:0|1:90906153_TGCAGA_T:1544,74:20:35	0/0:35,2:0.059:2:0:0.00:0|1:90906153_TGCAGA_T:944,45:16:19	0.8	Damaging	-1.29	Neutral	257	30	0.002	Damaging	2.92	192
T12,T1,T2,T4-2	chr2	**4**	**50**	90906163	90906163	C	T	exonic	**Kbtbd4**	ENSMUSP00000113088	.	nonsynonymous SNV	Kbtbd4:NM_001311116:exon2:c.C517T:p.H173Y,Kbtbd4:NM_025991:exon2:c.C469T:p.H157Y	**H157Y**	ENSMUSP00000113088,157,H,Y	2,90906163,C,A	2qE1	.	0.25	.	38	chr2	90906163	.	C	A	.	alt_allele_in_normal;clustered_events;germline_risk	ECNT=6;HCNT=1;MAX_ED=25;MIN_ED=10;NLOD=0.934;TLOD=11.04	GT:AD:AF:ALT_F1R2:ALT_F2R1:FOXOG:PGT:PID:QSS:REF_F1R2:REF_F2R1	0/1:52,4:0.071:4:0:0.00:0|1:90906153_TGCAGA_T:1466,100:20:32	0/0:36,2:0.061:2:0:0.00:0|1:90906153_TGCAGA_T:979,55:16:20	0.8	Deleterious	-3.69	Deleterious	314	30	0.003	Damaging	2.92	77
T12,T1,T2,T4-2	chr2	**4**	**50**	90906163	90906163	C	T	exonic	**Kbtbd4**	ENSMUSP00000113088	.	nonsynonymous SNV	Kbtbd4:NM_001311116:exon2:c.C517T:p.H173Y,Kbtbd4:NM_025991:exon2:c.C469T:p.H157Y	**H173Y**	ENSMUSP00000113088,173,H,Y	2,90906163,C,A	2qE1	.	0.25	.	38	chr2	90906163	.	C	A	.	alt_allele_in_normal;clustered_events;germline_risk	ECNT=6;HCNT=1;MAX_ED=25;MIN_ED=10;NLOD=0.934;TLOD=11.04	GT:AD:AF:ALT_F1R2:ALT_F2R1:FOXOG:PGT:PID:QSS:REF_F1R2:REF_F2R1	0/1:52,4:0.071:4:0:0.00:0|1:90906153_TGCAGA_T:1466,100:20:32	0/0:36,2:0.061:2:0:0.00:0|1:90906153_TGCAGA_T:979,55:16:20	0.8	Deleterious	-3.69	Deleterious	314	30	0.003	Damaging	2.92	77
T12,T1,T2,T4-2	chr2	**4**	**50**	90906166	90906166	A	G	exonic	**Kbtbd4**	ENSMUSP00000113088	.	nonsynonymous SNV	Kbtbd4:NM_001311116:exon2:c.A520G:p.S174G,Kbtbd4:NM_025991:exon2:c.A472G:p.S158G	**S158G**	ENSMUSP00000113088,158,S,G	2,90906166,A,T	2qE1	.	0.25	.	37	chr2	90906166	.	A	T	.	alt_allele_in_normal;clustered_events;germline_risk	ECNT=6;HCNT=1;MAX_ED=25;MIN_ED=10;NLOD=0.633;TLOD=8.28	GT:AD:AF:ALT_F1R2:ALT_F2R1:FOXOG:PGT:PID:QSS:REF_F1R2:REF_F2R1	0/1:59,3:0.048:3:0:.:0|1:90906153_TGCAGA_T:1654,81:21:38	0/0:35,2:0.063:2:0:.:0|1:90906153_TGCAGA_T:959,55:13:22	0.8	Deleterious	-3.39	Deleterious	314	30	0.001	Damaging	2.92	77
T12,T1,T2,T4-2	chr2	**4**	**50**	90906166	90906166	A	G	exonic	**Kbtbd4**	ENSMUSP00000113088	.	nonsynonymous SNV	Kbtbd4:NM_001311116:exon2:c.A520G:p.S174G,Kbtbd4:NM_025991:exon2:c.A472G:p.S158G	**S174G**	ENSMUSP00000113088,174,S,G	2,90906166,A,T	2qE1	.	0.25	.	37	chr2	90906166	.	A	T	.	alt_allele_in_normal;clustered_events;germline_risk	ECNT=6;HCNT=1;MAX_ED=25;MIN_ED=10;NLOD=0.934;TLOD=10.98	GT:AD:AF:ALT_F1R2:ALT_F2R1:FOXOG:PGT:PID:QSS:REF_F1R2:REF_F2R1	0/1:54,3:0.069:3:0:0.00:0|1:90906153_TGCAGA_T:1462,52:19:35	0/0:36,2:0.061:2:0:0.00:0|1:90906153_TGCAGA_T:980,45:15:21	0.8	Deleterious	-3.39	Deleterious	314	30	0.001	Damaging	2.92	77
T12,T1,T2,T4-2	chr2	**4**	**50**	90906178	90906178	C	A	exonic	**Kbtbd4**	ENSMUSP00000113088	.	nonsynonymous SNV	Kbtbd4:NM_001311116:exon2:c.C532A:p.L178I,Kbtbd4:NM_025991:exon2:c.C484A:p.L162I	**L162I**	ENSMUSP00000113088,162,L,I	2,90906178,C,T	2qE1	.	0.25	.	38	chr2	90906178	.	C	T	.	alt_allele_in_normal;clustered_events;germline_risk	ECNT=6;HCNT=1;MAX_ED=25;MIN_ED=10;NLOD=0.633;TLOD=8.28	GT:AD:AF:ALT_F1R2:ALT_F2R1:FOXOG:PGT:PID:QSS:REF_F1R2:REF_F2R1	0/1:59,3:0.048:3:0:.:0|1:90906153_TGCAGA_T:1654,81:21:38	0/0:35,2:0.063:2:0:.:0|1:90906153_TGCAGA_T:959,55:13:22	0.8	Deleterious	-3.45	Deleterious	314	30	0.003	Damaging	2.92	77
T12,T1,T2,T4-2	chr2	**4**	**50**	90906172	90906172	-	A	exonic	**Kbtbd4**	ENSMUSP00000113088	.	frameshift insertion	Kbtbd4:NM_001311116:exon2:c.526_527insA:p.P176fs,Kbtbd4:NM_025991:exon2:c.478_479insA:p.P160fs	**P160fs**	ENSMUSP00000113088,160,P,	2,90906172,C,T	2qE1	.	0.25	.	36	chr2	90906172	.	C	T	.	clustered_events;t_lod_fstar	ECNT=2;HCNT=1;MAX_ED=6;MIN_ED=6;NLOD=10.78;TLOD=5.62	GT:AD:AF:ALT_F1R2:ALT_F2R1:FOXOG:PGT:PID:QSS:REF_F1R2:REF_F2R1	0/1:34,2:0.054:2:0:1.00:0|1:15312935_C_T:945,58:10:24	0/0:40,0:0.00:0:0:.:0|1:15312935_C_T:1058,0:18:22	15	Tolerated	0.25	Neutral	314	30	0.624	Tolerated	2.92	77

T12,T1,T2,T4-2	chr2	**4**	**50**	90906172	90906172	-	A	exonic	**Kbtbd4**	ENSMUSP00000113088	.	frameshift insertion	Kbtbd4:NM_001311116:exon2:c.526_527insA:p.P176fs,Kbtbd4:NM_025991:exon2:c.478_479insA:p.P160fs	**P176fs**	ENSMUSP00000113088,176,P,	2,90906172,C,T	2qE1	.	0.25	.	36	chr2	90906172	.	C	T	.	clustered_events;t_lod_fstar	ECNT=2;HCNT=1;MAX_ED=6;MIN_ED=6;NLOD=10.48;TLOD=5.62	GT:AD:AF:ALT_F1R2:ALT_F2R1:FOXOG:PGT:PID:QSS:REF_F1R2:REF_F2R1	0/1:35,2:0.054:2:0:0.00:0|1:15312935_C_T:917,58:10:25	0/0:39,0:0.00:0:0:.:0|1:15312935_C_T:935,0:16:23	15	Tolerated	0.25	Neutral	314	30	0.624	Tolerated	2.92	77
T12,T1,T2,T4-2	chr2	**4**	**50**	90906154	90906158	GCAGA	-	exonic	**Kbtbd4**	ENSMUSP00000113088	.	frameshift deletion	Kbtbd4:NM_001311116:exon2:c.508_512del:p.A170fs,Kbtbd4:NM_025991:exon2:c.460_464del:p.A154fs	**A154fs**	ENSMUSP00000113088,154,A,.	2,90906154,TGCAGA,CA	2qE1	.	0.25	.	37	chr2	90906153	.	TGCAGA	CA	.	alt_allele_in_normal;clustered_events;germline_risk	ECNT=6;HCNT=1;MAX_ED=25;MIN_ED=10;NLOD=1.23;TLOD=8.42	GT:AD:AF:ALT_F1R2:ALT_F2R1:FOXOG:PGT:PID:QSS:REF_F1R2:REF_F2R1	0/1:56,4:0.054:4:0:.:0|1:90906153_TGCAGA_T:1529,111:21:35	0/0:34,2:0.059:2:0:.:0|1:90906153_TGCAGA_T:856,58:15:19	0.8	Unknown	NA	NA	314	30	NA	NA	NA	NA
T12,T1,T2,T4-2	chr2	**4**	**50**	90906154	90906158	GCAGA	-	exonic	**Kbtbd4**	ENSMUSP00000113088	.	frameshift deletion	Kbtbd4:NM_001311116:exon2:c.508_512del:p.A170fs,Kbtbd4:NM_025991:exon2:c.460_464del:p.A154fs	**A170fs**	ENSMUSP00000113088,170,A,.	2,90906154,TGCAGA,CA	2qE1	.	0.25	.	37	chr2	90906153	.	TGCAGA	CA	.	alt_allele_in_normal;clustered_events;germline_risk	ECNT=6;HCNT=1;MAX_ED=25;MIN_ED=10;NLOD=1.23;TLOD=8.42	GT:AD:AF:ALT_F1R2:ALT_F2R1:FOXOG:PGT:PID:QSS:REF_F1R2:REF_F2R1	0/1:56,4:0.054:4:0:.:0|1:90906153_TGCAGA_T:1529,111:21:35	0/0:34,2:0.059:2:0:.:0|1:90906153_TGCAGA_T:856,58:15:19	0.8	Unknown	NA	NA	314	30	NA	NA	NA	NA
T12,T22,T2,T42	chr6	**4**	**50**	129660487	129660487	A	G	exonic	**Klrc2**	ENSMUSP00000038150	.	nonsynonymous SNV	Klrc2:NM_001098669:exon1:c.T70C:p.F24L,Klrc2:NM_010653:exon1:c.T70C:p.F24L	**F24L**	ENSMUSP00000038150,24,F,L	6,129660487,A,T	6qF3	rs47730423	0.25	.	34	chr6	129660487	.	A	T	.	t_lod_fstar	ECNT=1;HCNT=1;MAX_ED=.;MIN_ED=.;NLOD=9.32;TLOD=5.70	GT:AD:AF:ALT_F1R2:ALT_F2R1:FOXOG:QSS:REF_F1R2:REF_F2R1	0/1:30,5:0.107:4:1:0.800:799,146:15:15	0/0:35,0:0.00:0:0:.:985,0:14:21	2.3	Tolerated	2.18	Neutral	198	30	0.397	Tolerated	3.14	80
T12,T22,T3,T4	chr8	**4**	**50**	13167805	13167805	A	C	splicing	**Lamp1**	ENSMUSP00000152020	NM_001317353:exon4:c.389-2A>C;NM_010684:exon4:c.386-2A>C	.	.	**NA**	8,13167805,A,A	NA	8qA1.1	.	0.25	.	42	chr8	13167805	.	A	A	.	alt_allele_in_normal;t_lod_fstar	ECNT=1;HCNT=1;MAX_ED=.;MIN_ED=.;NLOD=4.53;TLOD=4.89	GT:AD:AF:ALT_F1R2:ALT_F2R1:FOXOG:QSS:REF_F1R2:REF_F2R1	0/1:49,4:0.077:1:3:0.250:966,98:26:23	0/0:32,3:0.091:1:2:0.333:666,67:14:18	19	Unkown	.	.	.	.	.	.	.	.
T1,T22,T2,T4	chr7	**4**	**50**	48021973	48021973	C	T	exonic	**Mrgprx1**	ENSMUSP00000114308	.	nonsynonymous SNV	Mrgprx1:NM_207540:exon2:c.G25A:p.D9N	**D9N**	ENSMUSP00000114308,9,D,N	7,48021973,C,A	7qB4	.	0.25	.	59	chr7	48021973	.	C	A	.	clustered_events;multi_event_alt_allele_in_normal;t_lod_fstar	ECNT=6;HCNT=2;MAX_ED=20;MIN_ED=1;NLOD=11.45;TLOD=5.60	GT:AD:AF:ALT_F1R2:ALT_F2R1:FOXOG:PGT:PID:QSS:REF_F1R2:REF_F2R1	0/1:36,2:0.053:1:1:0.500:0|1:48021914_G_A:1021,52:16:20	0/0:52,1:0.019:0:1:0.00:0|1:48021914_G_A:1428,27:30:22	1.8	Damaging	-2.04	Neutral	140	30	0.02	Damaging	2.86	120
T12,T1,T2,T4	chr16	**4**	**50**	32754905	32754905	C	T	exonic	**Muc4**	ENSMUSP00000059471	.	nonsynonymous SNV	Muc4:NM_080457:exon4:c.C4778T:p.A1593V	**A1593V**	ENSMUSP00000059471,1593,A,V	16,32754905,C,A	16qB3	.	0.25	.	142	chr16	32754905	.	C	A	.	alt_allele_in_normal;clustered_events	ECNT=3;HCNT=6;MAX_ED=80;MIN_ED=72;NLOD=9.06;TLOD=17.81	GT:AD:AF:ALT_F1R2:ALT_F2R1:FOXOG:QSS:REF_F1R2:REF_F2R1	0/1:72,6:0.077:3:3:0.500:2010,166:33:39	0/0:64,2:0.032:2:0:0.00:1819,56:31:33	4	Damaging	-0.72	Neutral	56	30	0.003	Damaging	3.61	7
T12,T2,T32,T4	chr16	**4**	**50**	32753864	32753864	C	T	exonic	**Muc4**	ENSMUSP00000059471	.	nonsynonymous SNV	Muc4:NM_080457:exon3:c.C3739T:p.P1247S	**P1247S**	ENSMUSP00000059471,1247,P,S	16,32753864,C,T	16qB3	.	0.25	.	124	chr16	32753864	.	C	T	.	alt_allele_in_normal	ECNT=1;HCNT=4;MAX_ED=.;MIN_ED=.;NLOD=6.09;TLOD=10.85	GT:AD:AF:ALT_F1R2:ALT_F2R1:FOXOG:QSS:REF_F1R2:REF_F2R1	0/1:60,6:0.098:4:2:0.333:1687,165:28:32	0/0:42,3:0.070:2:1:0.333:1182,84:19:23	4	Tolerated	-0.61	Neutral	56	30	0.13	Tolerated	3.54	12
T12,T32,T42,T4	chr16	**4**	**50**	32754583	32754583	A	G	exonic	**Muc4**	ENSMUSP00000059471	.	nonsynonymous SNV	Muc4:NM_080457:exon4:c.A4456G:p.T1486A	**T1486A**	ENSMUSP00000059471,1486,T,A	16,32754583,A,A	16qB3	.	0.25	.	104	chr16	32754583	.	A	A	.	alt_allele_in_normal;clustered_events	ECNT=4;HCNT=3;MAX_ED=58;MIN_ED=18;NLOD=22.26;TLOD=16.24	GT:AD:AF:ALT_F1R2:ALT_F2R1:FOXOG:PGT:PID:QSS:REF_F1R2:REF_F2R1	0/1:138,6:0.043:3:3:0.500:0|1:32753806_A_T:3986,164:71:67	0/0:114,3:0.019:2:1:0.333:0|1:32753806_A_T:3242,63:65:49	4	Tolerated	0	Neutral	56	30	1	Tolerated	3.54	11
T12,T1,T2,T4	chr16	**4**	**50**	32754886	32754886	G	A	exonic	**Muc4**	ENSMUSP00000059471	.	nonsynonymous SNV	Muc4:NM_080457:exon4:c.G4759A:p.E1587K	**E1587K**	ENSMUSP00000059471,1587,E,K	16,32754886,G,A	16qB3	.	0.25	.	138	chr16	32754886	.	G	A	.	alt_allele_in_normal	ECNT=1;HCNT=3;MAX_ED=.;MIN_ED=.;NLOD=7.46;TLOD=16.55	GT:AD:AF:ALT_F1R2:ALT_F2R1:FOXOG:QSS:REF_F1R2:REF_F2R1	0/1:44,8:0.151:2:6:0.750:1224,225:17:27	0/0:72,4:0.056:2:2:0.500:1981,113:35:37	4	Tolerated	-0.93	Neutral	56	30	0.83	Tolerated	3.58	8
T12,T1,T3,T42	chr18	**4**	**50**	37423567	37423567	A	T	exonic	**Pcdhb11**	ENSMUSP00000125789	.	nonsynonymous SNV	Pcdhb11:NM_053136:exon1:c.A1949T:p.Q650L	**Q650L**	ENSMUSP00000125789,650,Q,L	18,37423567,A,A	18qB3	.	0.25	.	66	chr18	37423567	.	A	A	.	alt_allele_in_normal;germline_risk	ECNT=1;HCNT=4;MAX_ED=.;MIN_ED=.;NLOD=0.93;TLOD=26.93	GT:AD:AF:ALT_F1R2:ALT_F2R1:FOXOG:QSS:REF_F1R2:REF_F2R1	0/1:47,9:0.161:6:3:0.667:1318,243:23:24	0/0:37,4:0.114:2:2:0.500:1054,113:19:18	2.4	Damaging	0	Neutral	386	30	0.032	Damaging	3.35	391

T12,T1,T3,T42	chr18	**4**	**50**	37423570	37423570	T	G	exonic	**Pcdhb11**	ENSMUSP00000125789	.	nonsynonymous SNV	Pcdhb11:NM_053136:exon1:c.T1952G:p.L651R	**L651R**	ENSMUSP00000125789,651,L,R	18,37423570,T,A	18qB3	.	0.25	.	68	chr18	37423570	.	T	A	.	alt_allele_in_normal;germline_risk	ECNT=1;HCNT=4;MAX_ED=.;MIN_ED=.;NLOD=0.932;TLOD=26.93	GT:AD:AF:ALT_F1R2:ALT_F2R1:FOXOG:QSS:REF_F1R2:REF_F2R1	0/1:47,9:0.161:6:3:0.667:1318,243:23:24	0/0:37,4:0.114:2:2:0.500:1054,113:19:18	2.4	Tolerated	-0.1	Neutral	386	30	0.092	Tolerated	3.35	391
T12,T2,T32,T42	chr18	**4**	**50**	37309350	37309350	A	G	exonic	**Pcdhb4**	ENSMUSP00000132166	.	nonsynonymous SNV	Pcdhb4:NM_053129:exon1:c.A1712G:p.Y571C	**Y571C**	ENSMUSP00000132166,571,Y,C	18,37309350,A,A	18qB3	.	0.25	.	60	chr18	37309350	.	A	A	.	t_lod_fstar	ECNT=1;HCNT=2;MAX_ED=.;MIN_ED=.;NLOD=13.76;TLOD=4.28	GT:AD:AF:ALT_F1R2:ALT_F2R1:FOXOG:QSS:REF_F1R2:REF_F2R1	0/1:73,3:0.040:1:2:0.333:1917,84:38:35	0/0:63,1:0.018:1:0:1.00:1594,28:27:36	0.7	Tolerated	0	Neutral	388	30	0.076	Tolerated	3.37	389
T12,T32,T3,T4	chr1	**4**	**50**	85591571	85591571	G	A	exonic	**Sp110**	ENSMUSP00000040248	.	stopgain	Sp110:NM_030194:exon4:c.C535T:p.R179X,Sp110:NM_175397:exon5:c.C535T:p.R179X	**R179X**	ENSMUSP00000040248,179,R,X	NA	1qC5	.	0.25	.	700	chr1	85591571	.	G	T	.	alt_allele_in_normal;clustered_events	ECNT=4;HCNT=4;MAX_ED=50;MIN_ED=25;NLOD=60.63;TLOD=13.63	GT:AD:AF:ALT_F1R2:ALT_F2R1:FOXOG:QSS:REF_F1R2:REF_F2R1	0/1:165,18:0.098:8:10:0.556:3944,507:83:82	0/0:298,21:0.066:13:8:0.381:7169,602:148:150	0.3	Deletarious	.	.	.	.	.	.	.	.
T12,T3,T42,T4	chr1	**4**	**50**	85594382	85594382	T	A	exonic	**Sp110**	ENSMUSP00000040248	.	nonsynonymous SNV	Sp110:NM_030194:exon3:c.A199T:p.N67Y,Sp110:NM_175397:exon4:c.A199T:p.N67Y	**N67Y**	ENSMUSP00000040248,67,N,Y	1,85594382,T,A	1qC5	.	0.25	.	380	chr1	85594382	.	T	A	.	alt_allele_in_normal;clustered_events	ECNT=4;HCNT=4;MAX_ED=248;MIN_ED=196;NLOD=83.60;TLOD=67.42	GT:AD:AF:ALT_F1R2:ALT_F2R1:FOXOG:QSS:REF_F1R2:REF_F2R1	0/1:812,46:0.051:22:24:0.478:20107,1298:391:421	0/0:661,39:0.055:16:23:0.410:16093,1073:323:338	0.3	Deleterious	-6.77	Deleterious	104	30	0.006	Damaging	2.79	110
T12,T3,T42,T4	chr1	**4**	**50**	85594434	85594434	C	T	splicing	**Sp110**	ENSMUSP00000040248	NM_030194:exon3:c.148-1G>A;NM_175397:exon4:c.148-1G>A	.	.	**NA**	1,85594434,C,T	NA	1qC5	.	0.25	.	438	chr1	85594434	.	C	T	.	alt_allele_in_normal;clustered_events	ECNT=4;HCNT=2;MAX_ED=50;MIN_ED=25;NLOD=39.93;TLOD=16.54	GT:AD:AF:ALT_F1R2:ALT_F2R1:FOXOG:PGT:PID:QSS:REF_F1R2:REF_F2R1	0/1:199,12:0.056:4:8:0.667:0|1:85586225_A_T:5513,347:102:97	0/0:310,21:0.054:8:13:0.619:0|1:85586225_A_T:8420,598:161:149	0.3	Unkown	.	.	.	.	.	.	.	.
T1,T22,T3,T42	chr1	**4**	**50**	85610793	85610793	A	G	exonic	**Sp140**	ENSMUSP00000108782	.	nonsynonymous SNV	Sp140:NM_001013817:exon3:c.A250G:p.I84V	**I84V**	ENSMUSP00000108782,84,I,V	1,85610793,A,T	1qC5	rs36239030	0.25	.	410	chr1	85610793	.	A	T	.	alt_allele_in_normal;clustered_events	ECNT=11;HCNT=5;MAX_ED=129;MIN_ED=1;NLOD=34.54;TLOD=27.83	GT:AD:AF:ALT_F1R2:ALT_F2R1:FOXOG:PGT:PID:QSS:REF_F1R2:REF_F2R1	0/1:197,9:0.051:6:3:0.667:0|1:85610790_G_T:4855,262:92:105	0/0:380,19:0.050:8:11:0.421:0|1:85610790_G_T:9477,551:175:205	0.2	Deleterious	-3.26	Deleterious	93	30	0.001	Damaging	2.86	66
T1,T3,T42,T4	chr18	**4**	**50**	52490056	52490056	G	A	splicing	**Srfbp1**	ENSMUSP00000078795	NM_026040:exon7:c.1141+1G>A	.	.	**NA**	18,52490056,G,T	NA	18qD1	.	0.25	.	101	chr18	52490056	.	G	T	.	t_lod_fstar	ECNT=1;HCNT=1;MAX_ED=.;MIN_ED=.;NLOD=14.95;TLOD=5.00	GT:AD:AF:ALT_F1R2:ALT_F2R1:FOXOG:QSS:REF_F1R2:REF_F2R1	0/1:62,3:0.051:3:0:1.00:1737,87:29:33	0/0:54,1:0.019:0:1:0.00:1561,14:25:29	1.1	Unkown	.	.	.	.	.	.	.	.
T22,T32,T3,T42	chr4	**4**	**50**	41196345	41196345	A	G	exonic	**Ubap2**	ENSMUSP00000047105	.	nonsynonymous SNV	Ubap2:NM_026872:exon24:c.T2821C:p.Y941H	**Y941H**	ENSMUSP00000047105,941,Y,H	4,41196345,A,C	4qA5	.	0.25	.	55	chr4	41196345	.	A	C	.	clustered_events;homologous_mapping_event	ECNT=3;HCNT=2;MAX_ED=6;MIN_ED=2;NLOD=11.44;TLOD=11.35	GT:AD:AF:ALT_F1R2:ALT_F2R1:FOXOG:PGT:PID:QSS:REF_F1R2:REF_F2R1	0/1:66,2:0.061:0:2:.:0|1:41199897_G_GGTTTTTTTTA:1838,56:34:32	0/0:40,0:0.00:0:0:.:0|1:41199897_G_GGTTTTTTTTA:1067,0:14:26	1.4	Deleterious	-8.41	Deleterious	169	30	0	Damaging	2.84	168
T12,T22,T2,T4	chr4	**4**	**50**	119282444	119282444	G	A	exonic	**Ybx1**	ENSMUSP00000133076	.	nonsynonymous SNV	Ybx1:NM_011732:exon5:c.C353T:p.A118V	**A118V**	ENSMUSP00000133076,118,A,V	4,119282444,G,AACCTTTTCT	4qD2.1	.	0.25	.	40	chr4	119282444	.	G	AACCTTTTCT	.	alt_allele_in_normal;clustered_events;germline_risk;t_lod_fstar	ECNT=4;HCNT=15;MAX_ED=29;MIN_ED=24;NLOD=2.14;TLOD=5.43	GT:AD:AF:ALT_F1R2:ALT_F2R1:FOXOG:PGT:PID:QSS:REF_F1R2:REF_F2R1	0/1:43,2:0.043:2:0:.:0|1:119282444_G_A:1213,54:20:23	0/0:38,2:0.054:1:1:.:0|1:119282444_G_A:1082,54:17:21	1.9	Deleterious	-13.82	Deleterious	213	30	NA	NA	NA	NA
T2, T32, T3	chr1	**3**	**37.5**	171573958	171573958	-	AGAA	exonic	**Cd244**	ENSMUSP00000142896	.	frameshift insertion	Cd244:NM_018729:exon2:c.339_340insAGAA:p.N113fs	**N113fs**	ENSMUSP00000142896,113,N,	1,171573958,C,T	1qH3	.	0.25	.	166	chr1	171573958	.	C	T	.	alt_allele_in_normal	ECNT=1;HCNT=2;MAX_ED=.;MIN_ED=.;NLOD=4.49;TLOD=10.24	GT:AD:AF:ALT_F1R2:ALT_F2R1:FOXOG:QSS:REF_F1R2:REF_F2R1	0/1:96,7:0.067:4:3:0.429:2659,187:49:47	0/0:117,10:0.081:4:6:0.600:3262,277:63:54	20	Tolerated	0	Neutral	101	30	1	Tolerated	2.87	61
T2, T3, T32	chr1	**3**	**37.5**	171573962	171573965	GGCG	-	exonic	**Cd244**	ENSMUSP00000142896	.	frameshift deletion	Cd244:NM_018729:exon2:c.343_346del:p.G115fs	**G115fs**	NA	1,171573961,AGGCG,A	1qH3	.	0.25	.	173	chr1	171573961	.	AGGCG	A	.	alt_allele_in_normal;clustered_events	ECNT=2;HCNT=13;MAX_ED=3;MIN_ED=3;NLOD=9.38;TLOD=19.80	GT:AD:AF:ALT_F1R2:ALT_F2R1:FOXOG:PGT:PID:QSS:REF_F1R2:REF_F2R1	0/1:144,8:0.052:3:5:.:0|1:171573958_C_CAGAA:4020,233:77:67	0/0:164,9:0.056:4:5:.:0|1:171573958_C_CAGAA:4612,256:78:86	20	Unknown	NA	NA	101	30	NA	NA	NA	NA

T1, T2, T42	chr1	**3**	**37.5**	171574129	171574144	AGCCCTGGACTAATGG	-	exonic	**Cd244**	ENSMUSP00000142896	.	frameshift deletion	Cd244:NM_018729:exon3:c.422_437del:p.K141fs	**K141fs**	NA	1,171574128,AAGCCCTGGACTAATGG,A	1qH3	.	0.25	.	168	chr1	171574128	.	AAGCCCTGGACTAATGG	A	.	alt_allele_in_normal	ECNT=1;HCNT=2;MAX_ED=.;MIN_ED=.;NLOD=38.26;TLOD=15.17	GT:AD:AF:ALT_F1R2:ALT_F2R1:FOXOG:QSS:REF_F1R2:REF_F2R1	0/1:214,7:0.029:5:2:.:6024,205:114:100	0/0:166,2:0.013:0:2:.:4544,57:85:81	20	Unknown	NA	NA	101	30	NA	NA	NA	NA
T1, T22, T3	chr8	**3**	**37.5**	102673875	102673875	C	T	exonic	**Cdh11**	ENSMUSP00000043753	.	nonsynonymous SNV	Cdh11:NM_009866:exon4:c.G460A:p.D154N	**D154N**	ENSMUSP00000043753,154,D,N	8,102673875,C,C	8qD1	.	0.25	.	39	chr8	102673875	.	C	C	.	t_lod_fstar	ECNT=1;HCNT=2;MAX_ED=.;MIN_ED=.;NLOD=12.29;TLOD=4.30	GT:AD:AF:ALT_F1R2:ALT_F2R1:FOXOG:QSS:REF_F1R2:REF_F2R1	0/1:25,2:0.077:2:0:0.00:688,45:10:15	0/0:51,0:0.00:0:0:.:1397,0:24:27	0.5	Tolerated	0	Neutral	442	30	1	Tolerated	3.03	391
T22, T32, T4	chr8	**3**	**37.5**	72467909	72467914	TGTTGC	-	exonic	**Cherp**	ENSMUSP00000140645	.	nonframeshift deletion	Cherp:NM_138585:exon8:c.1005_1010del:p.335_337del	**335_337del**	NA	8,72467909,TTGTTGC,A	8qB3.3	.	0.25	.	26	chr8	72467908	.	TTGTTGC	A	.	PASS	DB;ECNT=1;HCNT=1;MAX_ED=.;MIN_ED=.;NLOD=10.48;TLOD=64.39	GT:AD:AF:ALT_F1R2:ALT_F2R1:FOXOG:QSS:REF_F1R2:REF_F2R1	0/1:27,26:0.490:14:12:0.538:763,719:20:7	0/0:38,0:0.00:0:0:.:1036,0:16:22	4	Unknown	.	.	.	.	.	.	.	.
T32, T42,T4	chr15	**3**	**37.5**	78346449	78346449	G	A	exonic	**Csf2rb**	ENSMUSP00000129314	.	nonsynonymous SNV	Csf2rb:NM_007780:exon11:c.G1391A:p.R464H	**R464H**	ENSMUSP00000129314,464,R,H	15,78346449,G,A	15qE1	.	0.25	.	35	chr15	78346449	.	G	A	.	alt_allele_in_normal;clustered_events;t_lod_fstar	ECNT=9;HCNT=1;MAX_ED=27;MIN_ED=3;NLOD=4.53;TLOD=5.21	GT:AD:AF:ALT_F1R2:ALT_F2R1:FOXOG:PGT:PID:QSS:REF_F1R2:REF_F2R1	0/1:56,2:0.034:2:0:1.00:0|1:122862086_C_CTTTTT:1516,33:27:29	0/0:32,1:0.033:1:0:1.00:0|1:122862086_C_CTTTTT:866,23:14:18	1.1	Tolerated	-1.09	Neutral	85	30	0.557	Tolerated	2.86	51
T22,T2,T3	chr2	**3**	**37.5**	155829912	155829912	A	T	exonic	**Fam83c**	ENSMUSP00000022296	.	nonsynonymous SNV	Fam83c:NM_027788:exon4:c.T1602A:p.H534Q	**H534Q**	ENSMUSP00000022296,534,H,Q	2,155829912,A,T	2qH1	.	0.25	.	38	chr2	155829912	.	A	T	.	t_lod_fstar	ECNT=1;HCNT=1;MAX_ED=.;MIN_ED=.;NLOD=13.54;TLOD=5.24	GT:AD:AF:ALT_F1R2:ALT_F2R1:FOXOG:QSS:REF_F1R2:REF_F2R1	0/1:44,3:0.071:1:2:0.667:1201,85:26:18	0/0:50,0:0.00:0:0:.:1404,0:20:30	0.5	Tolerated	-0.72	Neutral	160	30	0.057	Tolerated	3.66	31
T12, T2, T3	chr18	**3**	**37.5**	35583876	35583876	A	G	splicing	**Matr3**	ENSMUSP00000016678	.	NM_010771:exon11:c.1735-2A>G	.	**NA**	18,35583876,A,T	NA	18qB2	.	0.25	.	77	chr18	35583876	.	A	T	.	PASS	ECNT=1;HCNT=1;MAX_ED=.;MIN_ED=.;NLOD=11.12;TLOD=13.80	GT:AD:AF:ALT_F1R2:ALT_F2R1:FOXOG:QSS:REF_F1R2:REF_F2R1	0/1:21,6:0.240:3:3:0.500:526,169:13:8	0/0:41,0:0.00:0:0:.:1051,0:19:22	1.3	Unkown	.	.	.	.	.	.	.	.
T22,T2,T3	chr9	**3**	**37.5**	64647900	64647900	G	C	exonic	**Megf11**	ENSMUSP00000030860	.	nonsynonymous SNV	Megf11:NM_172522:exon7:c.G752C:p.C251S,Megf11:NM_001134399:exon8:c.G845C:p.C282S	**C251S**	ENSMUSP00000030860,251,C,S	9,64647900,G,A	9qC	.	0.25	.	31	chr9	64647900	.	G	A	.	t_lod_fstar	ECNT=1;HCNT=1;MAX_ED=.;MIN_ED=.;NLOD=9.63;TLOD=4.12	GT:AD:AF:ALT_F1R2:ALT_F2R1:FOXOG:QSS:REF_F1R2:REF_F2R1	0/1:59,1:0.034:0:1:0.00:1704,16:29:30	0/0:33,0:0.00:0:0:.:948,0:16:17	0.7	Deleterious	-9.57	Deleterious	163	30	0	Damaging	2.89	105
T22,T2,T3	chr9	**3**	**37.5**	64647900	64647900	G	C	exonic	**Megf11**	ENSMUSP00000030860	.	nonsynonymous SNV	Megf11:NM_172522:exon7:c.G752C:p.C251S,Megf11:NM_001134399:exon8:c.G845C:p.C282S	**C282S**	ENSMUSP00000030860,282,C,S	9,64647900,G,A	9qC	.	0.25	.	31	chr9	64647900	.	G	A	.	t_lod_fstar	ECNT=1;HCNT=1;MAX_ED=.;MIN_ED=.;NLOD=9.63;TLOD=4.12	GT:AD:AF:ALT_F1R2:ALT_F2R1:FOXOG:QSS:REF_F1R2:REF_F2R1	0/1:59,1:0.034:0:1:0.00:1704,16:29:30	0/0:33,0:0.00:0:0:.:948,0:16:17	0.7	Deleterious	-9.57	Deleterious	163	30	0	Damaging	2.89	105
T1, T22,T4	chr7	**3**	**37.5**	48021962	48021962	A	-	exonic	**Mrgprx1**	ENSMUSP00000114308	.	frameshift deletion	Mrgprx1:NM_207540:exon2:c.36delT:p.S12fs	**S12fs**	ENSMUSP00000114308,12,S,.	7,48021962,TA,C	7qB4	.	0.25	.	60	chr7	48021961	.	TA	C	.	clustered_events;multi_event_alt_allele_in_normal;t_lod_fstar	ECNT=6;HCNT=2;MAX_ED=20;MIN_ED=1;NLOD=11.15;TLOD=5.62	GT:AD:AF:ALT_F1R2:ALT_F2R1:FOXOG:PGT:PID:QSS:REF_F1R2:REF_F2R1	0/1:36,2:0.054:1:1:0.500:0|1:48021914_G_A:973,53:16:20	0/0:52,1:0.019:0:1:0.00:0|1:48021914_G_A:1434,27:30:22	1.8	Unknown	NA	NA	140	30	NA	NA	NA	NA
T22, T2,T42	chr16	**3**	**37.5**	32754662	32754662	C	T	exonic	**Muc4**	ENSMUSP00000059471	.	nonsynonymous SNV	Muc4:NM_080457:exon4:c.C4535T:p.T1512I	**T1512I**	ENSMUSP00000059471,1512,T,I	16,32754662,C,T	16qB3	.	0.25	.	116	chr16	32754662	.	C	T	.	alt_allele_in_normal;clustered_events	ECNT=2;HCNT=5;MAX_ED=7;MIN_ED=7;NLOD=3.72;TLOD=16.26	GT:AD:AF:ALT_F1R2:ALT_F2R1:FOXOG:PGT:PID:QSS:REF_F1R2:REF_F2R1	0/1:134,6:0.043:4:2:0.333:0|1:32753864_C_T:3778,170:61:73	0/0:123,7:0.059:5:2:0.286:0|1:32753864_C_T:3453,197:62:61	4	Damaging	-0.94	Neutral	56	30	0.002	Damaging	3.54	12
T22,T32, T4	chr16	**3**	**37.5**	32755873	32755873	A	C	exonic	**Muc4**	ENSMUSP00000059471	.	nonsynonymous SNV	Muc4:NM_080457:exon4:c.A5746C:p.S1916R	**S1916R**	ENSMUSP00000059471,1916,S,R	16,32755873,A,T	16qB3	.	0.25	.	53	chr16	32755873	.	A	T	.	t_lod_fstar	ECNT=1;HCNT=6;MAX_ED=.;MIN_ED=.;NLOD=6.92;TLOD=5.47	GT:AD:AF:ALT_F1R2:ALT_F2R1:FOXOG:QSS:REF_F1R2:REF_F2R1	0/1:41,2:0.045:1:1:0.500:1155,56:20:21	0/0:23,0:0.00:0:0:.:623,0:9:14	4	Damaging	-1.11	Neutral	56	30	0.001	Damaging	3.41	18

T1, T3,T42	chr16	**3**	**37.5**	32753580	32753580	G	A	exonic	**Muc4**	ENSMUSP00000059471	.	nonsynonymous SNV	Muc4:NM_080457:exon3:c.G3455A:p.R1152K	**R1152K**	ENSMUSP00000059471,1152,R,K	16,32753580,G,C	16qB3	.	0.25	.	49	chr16	32753580	.	G	C	.	alt_allele_in_normal;clustered_events	ECNT=2;HCNT=3;MAX_ED=16;MIN_ED=16;NLOD=12.45;TLOD=7.72	GT:AD:AF:ALT_F1R2:ALT_F2R1:FOXOG:PGT:PID:QSS:REF_F1R2:REF_F2R1	0/1:93,3:0.032:0:3:0.00:0|1:32753786_G_T:2668,84:49:44	0/0:75,2:0.027:1:1:0.500:0|1:32753786_G_T:2084,56:38:37	4	Tolerated	0.08	Neutral	56	30	0.416	Tolerated	3.74	7
T1,T3,T42	chr16	**3**	**37.5**	32753581	32753581	A	C	exonic	**Muc4**	ENSMUSP00000059471	.	nonsynonymous SNV	Muc4:NM_080457:exon3:c.A3456C:p.R1152S	**R1152S**	ENSMUSP00000059471,1152,R,S	16,32753581,A,C	16qB3	.	0.25	.	49	chr16	32753581	.	A	C	.	clustered_events;homologous_mapping_event	ECNT=3;HCNT=15;MAX_ED=80;MIN_ED=72;NLOD=10.84;TLOD=11.88	GT:AD:AF:ALT_F1R2:ALT_F2R1:FOXOG:PGT:PID:QSS:REF_F1R2:REF_F2R1	0/1:49,4:0.077:4:0:1.00:0|1:32752657_G_C:1371,79:26:23	0/0:41,0:0.00:0:0:.:0|1:32752657_G_C:1172,0:16:25	4	Tolerated	0.14	Neutral	56	30	0.069	Tolerated	3.74	7
T12,T3,T42	chr16	**3**	**37.5**	32754347	32754347	C	T	exonic	**Muc4**	ENSMUSP00000059471	.	nonsynonymous SNV	Muc4:NM_080457:exon4:c.C4220T:p.T1407I	**T1407I**	ENSMUSP00000059471,1407,T,I	16,32754347,C,T	16qB3	.	0.25	.	45	chr16	32754347	.	C	T	.	alt_allele_in_normal;clustered_events	ECNT=4;HCNT=3;MAX_ED=58;MIN_ED=18;NLOD=9.31;TLOD=20.02	GT:AD:AF:ALT_F1R2:ALT_F2R1:FOXOG:PGT:PID:QSS:REF_F1R2:REF_F2R1	0/1:134,8:0.057:3:5:0.625:0|1:32753806_A_T:3640,223:71:63	0/0:108,8:0.067:3:5:0.625:0|1:32753806_A_T:2948,221:60:48	4	Tolerated	-0.66	Neutral	56	30	0.196	Tolerated	3.55	10
T12,T2,T4,	chr16	**3**	**37.5**	32754911	32754911	G	A	exonic	**Muc4**	ENSMUSP00000059471	.	nonsynonymous SNV	Muc4:NM_080457:exon4:c.G4784A:p.S1595N	**S1595N**	ENSMUSP00000059471,1595,S,N	16,32754911,G,G	16qB3	.	0.25	.	142	chr16	32754911	.	G	G	.	alt_allele_in_normal;clustered_events;t_lod_fstar	ECNT=5;HCNT=1;MAX_ED=52;MIN_ED=4;NLOD=16.00;TLOD=4.46	GT:AD:AF:ALT_F1R2:ALT_F2R1:FOXOG:PGT:PID:QSS:REF_F1R2:REF_F2R1	0/1:147,2:0.014:1:1:0.500:0|1:32754579_C_A:4043,56:74:73	0/0:101,3:0.031:3:0:0.00:0|1:32754579_C_A:2688,87:52:49	4	Tolerated	0	Neutral	56	30	1	Tolerated	3.61	7
T12,T2,T32	chr16	**3**	**37.5**	32755721	32755721	T	G	exonic	**Muc4**	ENSMUSP00000059471	.	nonsynonymous SNV	Muc4:NM_080457:exon4:c.T5594G:p.I1865S	**I1865S**	ENSMUSP00000059471,1865,I,S	16,32755721,T,A	16qB3	.	0.25	.	58	chr16	32755721	.	T	A	.	clustered_events;multi_event_alt_allele_in_normal	ECNT=5;HCNT=2;MAX_ED=35;MIN_ED=4;NLOD=34.63;TLOD=12.73	GT:AD:AF:ALT_F1R2:ALT_F2R1:FOXOG:PGT:PID:QSS:REF_F1R2:REF_F2R1	0/1:170,4:0.030:1:3:0.250:0|1:32754882_T_C:4907,109:82:88	0/0:141,1:7.634e-03:0:1:0.00:0|1:32754882_T_C:3983,28:65:76	4	Tolerated	0.59	Neutral	56	30	0.06	Tolerated	3.41	17
T2,T3,T42	chr14	**3**	**37.5**	54960410	54960410	A	C	exonic	**Myh6**	ENSMUSP00000108141	.	nonsynonymous SNV	Myh6:NM_001164171:exon13:c.T1270G:p.S424A,Myh6:NM_010856:exon13:c.T1270G:p.S424A	**S424A**	ENSMUSP00000108141,424,S,A	14,54960410,A,T	14qC3	.	0.25	.	63	chr14	54960410	.	A	T	.	clustered_events;homologous_mapping_event	ECNT=3;HCNT=1;MAX_ED=5;MIN_ED=4;NLOD=18.06;TLOD=9.90	GT:AD:AF:ALT_F1R2:ALT_F2R1:FOXOG:PGT:PID:QSS:REF_F1R2:REF_F2R1	0/1:68,3:0.056:1:2:0.333:0|1:54960410_A_C:1875,84:34:34	0/0:63,0:0.00:0:0:.:0|1:54960410_A_C:1752,0:36:27	1.5	Damaging	-1.24	Neutral	529	30	0.018	Damaging	3.89	395
T2,T3,T42	chr14	**3**	**37.5**	54960414	54960414	G	T	exonic	**Myh6**	ENSMUSP00000000505	.	stopgain	Myh6:NM_001164171:exon13:c.C1266A:p.Y422X,Myh6:NM_010856:exon13:c.C1266A:p.Y422X	**Y422X**	ENSMUSP00000000505,422,Y,X	NA	14qC3	.	0.25	.	63	chr14	54960414	.	G	C	.	alt_allele_in_normal;t_lod_fstar	ECNT=1;HCNT=1;MAX_ED=.;MIN_ED=.;NLOD=10.71;TLOD=4.41	GT:AD:AF:ALT_F1R2:ALT_F2R1:FOXOG:QSS:REF_F1R2:REF_F2R1	0/1:76,10:0.077:4:6:.:1982,266:37:39	0/0:45,6:0.125:3:3:.:1225,159:15:30	1.4	Deletarious	.	.	.	.	.	.	.	.
T2,T3,T42	chr14	**3**	**37.5**	54960415	54960415	T	G	exonic	**Myh6**	ENSMUSP00000000505	.	nonsynonymous SNV	Myh6:NM_001164171:exon13:c.A1265C:p.Y422S,Myh6:NM_010856:exon13:c.A1265C:p.Y422S	**Y422S**	ENSMUSP00000000505,422,Y,S	14,54960415,T,A	14qC3	.	0.25	.	65	chr14	54960415	.	T	A	.	clustered_events;homologous_mapping_event	ECNT=5;HCNT=1;MAX_ED=14;MIN_ED=4;NLOD=12.91;TLOD=11.39	GT:AD:AF:ALT_F1R2:ALT_F2R1:FOXOG:PGT:PID:QSS:REF_F1R2:REF_F2R1	0/1:64,3:0.059:1:2:0.333:0|1:54960410_A_C:1799,76:34:30	0/0:62,0:0.017:0:0:.:0|1:54960410_A_C:1743,0:35:27	1.5	Tolerated	-1.17	Neutral	529	30	0.316	Tolerated	3.89	395
T12,T22,T42	chr5	**3**	**37.5**	134227483	134227483	G	A	exonic	**Ncf1**	ENSMUSP00000072091	.	nonsynonymous SNV	Ncf1:NM_001286037:exon3:c.C196T:p.H66Y,Ncf1:NM_010876:exon3:c.C196T:p.H66Y	**H66Y**	ENSMUSP00000072091,66,H,Y	5,134227483,G,A	5qG2	.	0.25	.	34	chr5	134227483	.	G	A	.	clustered_events;multi_event_alt_allele_in_normal;t_lod_fstar	ECNT=7;HCNT=1;MAX_ED=26;MIN_ED=10;NLOD=5.73;TLOD=5.67	GT:AD:AF:ALT_F1R2:ALT_F2R1:FOXOG:PGT:PID:QSS:REF_F1R2:REF_F2R1	0/1:35,2:0.057:0:2:.:0|1:134227483_G_A:931,54:16:19	0/0:35,1:0.029:0:1:.:0|1:134227483_G_A:906,20:18:17	0.7	Damaging	-2.1	Neutral	182	30	0.002	Damaging	2.78	171
T12,T22,T42	chr5	**3**	**37.5**	134227508	134227508	C	A	exonic	**Ncf1**	ENSMUSP00000072091	.	nonsynonymous SNV	Ncf1:NM_001286037:exon3:c.G171T:p.M57I,Ncf1:NM_010876:exon3:c.G171T:p.M57I	**M57I**	ENSMUSP00000072091,57,M,I	5,134227508,C,A	5qG2	.	0.25	.	33	chr5	134227508	.	C	A	.	clustered_events;multi_event_alt_allele_in_normal;t_lod_fstar	ECNT=7;HCNT=1;MAX_ED=26;MIN_ED=10;NLOD=4.53;TLOD=5.72	GT:AD:AF:ALT_F1R2:ALT_F2R1:FOXOG:PGT:PID:QSS:REF_F1R2:REF_F2R1	0/1:34,2:0.061:0:2:.:0|1:134227483_G_A:943,46:14:20	0/0:32,1:0.033:0:1:.:0|1:134227483_G_A:889,13:17:15	0.7	Deleterious	-2.72	Deleterious	182	30	0.059	Tolerated	2.78	169

T12,T22,T42	chr5	**3**	**37.5**	134227496	134227496	-	GTA	exonic	**Ncf1**	ENSMUSP00000072091	.	nonframeshift insertion	Ncf1:NM_001286037:exon3:c.182_183insTAC:p.E61delinsDT,Ncf1:NM_010876:exon3:c.182_183insTAC:p.E61delinsDT	**E61delinsDT**	ENSMUSP00000072091,61,E,DT	5,134227496,C,G	5qG2	.	0.25	.	32	chr5	134227496	.	C	G	.	alt_allele_in_normal;clustered_events;t_lod_fstar	ECNT=8;HCNT=1;MAX_ED=26;MIN_ED=7;NLOD=5.13;TLOD=5.67	GT:AD:AF:ALT_F1R2:ALT_F2R1:FOXOG:PGT:PID:QSS:REF_F1R2:REF_F2R1	0/1:33,2:0.057:0:2:.:0|1:134227483_G_A:890,44:22:11	0/0:32,1:0.031:0:1:.:0|1:134227483_G_A:832,22:17:15	0.7	Deleterious	-2.66	Deleterious	182	30	0.014	Damaging	2.78	171
T12,T22,T42	chr5	**3**	**37.5**	134227493	134227493	-	TGGTC	exonic	**Ncf1**	ENSMUSP00000072091	.	frameshift insertion	Ncf1:NM_001286037:exon3:c.185_186insGACCA:p.A62fs,Ncf1:NM_010876:exon3:c.185_186insGACCA:p.A62fs	**A62fs**	ENSMUSP00000072091,62,A,	5,134227493,G,CATT	5qG2	.	0.25	.	33	chr5	134227493	.	G	CATT	.	clustered_events;multi_event_alt_allele_in_normal;t_lod_fstar	ECNT=7;HCNT=1;MAX_ED=26;MIN_ED=10;NLOD=4.83;TLOD=5.70	GT:AD:AF:ALT_F1R2:ALT_F2R1:FOXOG:PGT:PID:QSS:REF_F1R2:REF_F2R1	0/1:33,2:0.059:0:2:.:0|1:134227483_G_A:942,43:13:20	0/0:31,1:0.032:0:1:.:0|1:134227483_G_A:856,9:16:15	0.7	Deleterious	-8.91	Deleterious	182	30	NA	NA	NA	NA
T12,T22,T42	chr5	**3**	**37.5**	134227498	134227498	-	ATT	exonic	**Ncf1**	ENSMUSP00000072091	.	nonframeshift insertion	Ncf1:NM_001286037:exon3:c.180_181insAAT:p.E61delinsNE,Ncf1:NM_010876:exon3:c.180_181insAAT:p.E61delinsNE	**E61delinsNE**	ENSMUSP00000072091,61,E,NE	5,134227498,C,A	5qG2	.	0.25	.	32	chr5	134227498	.	C	A	.	clustered_events;multi_event_alt_allele_in_normal	ECNT=2;HCNT=2;MAX_ED=3;MIN_ED=3;NLOD=6.34;TLOD=22.09	GT:AD:AF:ALT_F1R2:ALT_F2R1:FOXOG:PGT:PID:QSS:REF_F1R2:REF_F2R1	0/1:31,7:0.184:2:5:0.286:0|1:52305377_T_A:850,194:12:19	0/0:39,1:0.028:1:0:1.00:0|1:52305377_T_A:1098,28:17:22	1	Unknown	NA	NA	182	30	NA	NA	NA	NA
T12,T22,T42	chr5	**3**	**37.5**	134227510	134227513	TCTC	-	exonic	**Ncf1**	ENSMUSP00000072091	.	frameshift deletion	Ncf1:NM_001286037:exon3:c.166_169del:p.E56fs,Ncf1:NM_010876:exon3:c.166_169del:p.E56fs	**E56fs**	ENSMUSP00000072091,56,E,.	5,134227510,ATCTC,CGTA	5qG2	.	0.25	.	33	chr5	134227509	.	ATCTC	CGTA	.	clustered_events;multi_event_alt_allele_in_normal;t_lod_fstar	ECNT=7;HCNT=1;MAX_ED=26;MIN_ED=10;NLOD=4.53;TLOD=5.70	GT:AD:AF:ALT_F1R2:ALT_F2R1:FOXOG:PGT:PID:QSS:REF_F1R2:REF_F2R1	0/1:33,2:0.059:0:2:.:0|1:134227483_G_A:919,46:13:20	0/0:31,1:0.033:0:1:.:0|1:134227483_G_A:888,8:16:15	0.7	Unknown	NA	NA	182	30	NA	NA	NA	NA
T12,T22,T42	chr5	**3**	**37.5**	134227501	134227505	TGGGG	-	exonic	**Ncf1**	ENSMUSP00000072091	.	frameshift deletion	Ncf1:NM_001286037:exon3:c.174_178del:p.F58fs,Ncf1:NM_010876:exon3:c.174_178del:p.F58fs	**F58fs**	ENSMUSP00000072091,58,F,.	5,134227501,ATGGGG,GTGGTC	5qG2	.	0.25	.	36	chr5	134227500	.	ATGGGG	GTGGTC	.	clustered_events;multi_event_alt_allele_in_normal;t_lod_fstar	ECNT=7;HCNT=1;MAX_ED=26;MIN_ED=10;NLOD=5.13;TLOD=5.72	GT:AD:AF:ALT_F1R2:ALT_F2R1:FOXOG:PGT:PID:QSS:REF_F1R2:REF_F2R1	0/1:33,2:0.061:0:2:.:0|1:134227483_G_A:815,50:13:20	0/0:32,1:0.031:0:1:.:0|1:134227483_G_A:786,9:17:15	0.7	Unknown	.	.	.	.	.	.	.	.
T12,T32,T3	chr2	**3**	**37.5**	52305377	52305377	T	A	exonic	**Neb**	ENSMUSP00000047667	.	nonsynonymous SNV	Neb:NM_010889:exon19:c.A1674T:p.Q558H	**Q558H**	ENSMUSP00000047667,558,Q,H	2,52305377,T,G	2qC1.1	.	0.25	.	40	chr2	52305377	.	T	G	.	t_lod_fstar	ECNT=1;HCNT=1;MAX_ED=.;MIN_ED=.;NLOD=8.72;TLOD=5.71	GT:AD:AF:ALT_F1R2:ALT_F2R1:FOXOG:QSS:REF_F1R2:REF_F2R1	0/1:34,4:0.081:2:2:0.500:955,118:19:15	0/0:34,0:0.00:0:0:.:955,0:11:23	0.8	Tolerated	-0.24	Neutral	79	30	0.239	Tolerated	2.94	36
T2,T42,T4	chr14	**3**	**37.5**	18215329	18215329	G	A	exonic	**Nr1d2**	ENSMUSP00000080538	.	nonsynonymous SNV	Nr1d2:NM_011584:exon5:c.C682T:p.R228W	**R228W**	ENSMUSP00000080538,228,R,W	14,18215329,G,T	14qA2	rs50302967	0.25	.	65	chr14	18215329	.	G	T	.	clustered_events;homologous_mapping_event	DB;ECNT=5;HCNT=16;MAX_ED=200;MIN_ED=53;NLOD=10.53;TLOD=85.44	GT:AD:AF:ALT_F1R2:ALT_F2R1:FOXOG:QSS:REF_F1R2:REF_F2R1	0/1:17,33:0.638:16:17:0.515:491,934:10:7	0/0:39,0:0.00:0:0:.:1085,0:18:21	3	Tolerated	0	Neutral	209	30	0.821	Tolerated	3.28	105
T12,T2,T32	chr2	**3**	**37.5**	92320384	92320386	GCA	-	exonic	**Phf21a**	ENSMUSP00000026520	.	nonframeshift deletion	Phf21a:NM_001109690:exon4:c.309_311del:p.103_104del,Phf21a:NM_001346704:exon7:c.309_311del:p.103_104del,Phf21a:NM_138755:exon7:c.309_311del:p.103_104del,Phf21a:NM_001109691:exon8:c.309_311del:p.103_104del	**103_104del**	NA	2,92320384,CGCA,T	2qE1	.	0.25	.	33	chr2	92320383	.	CGCA	T	.	t_lod_fstar	ECNT=1;HCNT=1;MAX_ED=.;MIN_ED=.;NLOD=9.27;TLOD=4.32	GT:AD:AF:ALT_F1R2:ALT_F2R1:FOXOG:QSS:REF_F1R2:REF_F2R1	0/1:73,3:0.041:3:0:0.00:1875,83:39:34	0/0:37,0:0.00:0:0:.:904,0:22:15	1.2	Unknown	.	.	.	.	.	.	.	.
T1,T32,T42	chr19	**3**	**37.5**	6374816	6374818	GCA	-	exonic	**Sf1**	ENSMUSP00000030134	.	nonframeshift deletion	Sf1:NM_001110791:exon12:c.1500_1502del:p.500_501del,Sf1:NM_011750:exon12:c.1500_1502del:p.500_501del	**500_501del**	NA	19,6374816,GGCA,A	19qA	.	0.25	.	38	chr19	6374815	.	GGCA	A	.	alt_allele_in_normal;clustered_events	ECNT=2;HCNT=8;MAX_ED=5;MIN_ED=5;NLOD=178.15;TLOD=85.99	GT:AD:AF:ALT_F1R2:ALT_F2R1:FOXOG:QSS:REF_F1R2:REF_F2R1	0/1:1501,59:0.038:27:32:0.458:37318,1701:725:776	0/0:1090,40:0.038:21:19:0.525:26744,1120:546:544	1	Unknown	.	.	.	.	.	.	.	.
T12,T1,T32	chr2	**3**	**37.5**	26370819	26370819	G	T	exonic	**Snapc4**	ENSMUSP00000045740	.	nonsynonymous SNV	Snapc4:NM_172339:exon14:c.C1387A:p.Q463K,Snapc4:NM_001290419:exon15:c.C1117A:p.Q373K	**Q463K**	ENSMUSP00000045740,463,Q,K	2,26370819,G,C	2qA3	rs33426179	0.25	.	53	chr2	26370819	rs33426179	G	C	.	clustered_events	DB;ECNT=2;HCNT=2;MAX_ED=37;MIN_ED=37;NLOD=12.03;TLOD=53.67	GT:AD:AF:ALT_F1R2:ALT_F2R1:FOXOG:QSS:REF_F1R2:REF_F2R1	0/1:17,24:0.564:16:8:0.667:483,596:9:8	0/0:46,0:0.00:0:0:.:1306,0:18:28	1.1	Damaging	-1.25	Neutral	112	30	0	Damaging	3.14	102

T12,T1,T32	chr2	**3**	**37.5**	26370819	26370819	G	T	exonic	**Snapc4**	ENSMUSP00000045740	.	nonsynonymous SNV	Snapc4:NM_172339:exon14:c.C1387A:p.Q463K,Snapc4:NM_001290419:exon15:c.C1117A:p.Q373K	**Q373K**	ENSMUSP00000045740,373,Q,K	2,26370819,G,T	2qA3	rs33426179	0.25	.	53	chr2	26370819	rs33426179	G	T	.	PASS	DB;ECNT=1;HCNT=1;MAX_ED=.;MIN_ED=.;NLOD=9.49;TLOD=53.57	GT:AD:AF:ALT_F1R2:ALT_F2R1:FOXOG:QSS:REF_F1R2:REF_F2R1	0/1:37,22:0.393:7:15:0.682:1016,602:17:20	0/0:33,0:0.00:0:0:.:870,0:17:16	1.1	Tolerated	0.47	Neutral	56	30	1	Tolerated	2.77	58
T12,T1,T2	chr1	**3**	**37.5**	85591751	85591751	T	C	exonic	**Sp110**	ENSMUSP00000040248	.	nonsynonymous SNV	Sp110:NM_030194:exon4:c.A355G:p.T119A,Sp110:NM_175397:exon5:c.A355G:p.T119A	**T119A**	ENSMUSP00000040248,119,T,A	1,85591751,T,T	1qC5	.	0.25	.	744	chr1	85591751	.	T	T	.	alt_allele_in_normal;clustered_events	ECNT=10;HCNT=2;MAX_ED=117;MIN_ED=25;NLOD=59.11;TLOD=60.39	GT:AD:AF:ALT_F1R2:ALT_F2R1:FOXOG:QSS:REF_F1R2:REF_F2R1	0/1:529,36:0.066:18:18:0.500:12970,1009:297:232	0/0:590,40:0.060:24:16:0.400:14524,1110:303:287	0.3	Tolerated	0	Neutral	104	30	1	Tolerated	2.89	75
T1,T2,T42	chr1	**3**	**37.5**	85610752	85610752	C	T	exonic	**Sp140**	ENSMUSP00000108782	.	nonsynonymous SNV	Sp140:NM_001013817:exon3:c.C209T:p.T70I	**T70I**	ENSMUSP00000108782,70,T,I	1,85610752,C,T	1qC5	rs49983289	0.25	.	455	chr1	85610752	.	C	T	.	alt_allele_in_normal;clustered_events	ECNT=15;HCNT=1;MAX_ED=140;MIN_ED=12;NLOD=43.57;TLOD=23.46	GT:AD:AF:ALT_F1R2:ALT_F2R1:FOXOG:PGT:PID:QSS:REF_F1R2:REF_F2R1	0/1:195,10:0.046:7:3:0.700:0|1:85610745_C_T:5661,297:100:95	0/0:409,18:0.040:7:11:0.389:0|1:85610745_C_T:11827,517:192:217	0.2	Deleterious	-4.49	Deleterious	134	30	0	Damaging	3.27	81
T1,T2,T42	chr1	**3**	**37.5**	85610759	85610759	G	C	exonic	**Sp140**	ENSMUSP00000108782	.	nonsynonymous SNV	Sp140:NM_001013817:exon3:c.G216C:p.K72N	**K72N**	ENSMUSP00000108782,72,K,N	1,85610759,G,G	1qC5	rs47068727	0.25	.	456	chr1	85610759	.	G	G	.	alt_allele_in_normal;clustered_events	ECNT=15;HCNT=1;MAX_ED=140;MIN_ED=12;NLOD=16.13;TLOD=9.23	GT:AD:AF:ALT_F1R2:ALT_F2R1:FOXOG:QSS:REF_F1R2:REF_F2R1	0/1:202,9:0.046:4:5:0.556:5918,208:104:98	0/0:378,32:0.087:17:15:0.469:10684,780:171:207	0.2	Tolerated	0	Neutral	93	30	0.058	Tolerated	2.86	66
T3,T42,T4	chr1	**3**	**37.5**	85601188	85601188	G	T	exonic	**Sp140**	ENSMUSP00000108782	.	nonsynonymous SNV	Sp140:NM_001013817:exon1:c.G29T:p.S10I	**S10I**	ENSMUSP00000108782,10,S,I	1,85601188,G,G	1qC5	.	0.25	.	296	chr1	85601188	.	G	G	.	alt_allele_in_normal;clustered_events;t_lod_fstar	ECNT=10;HCNT=1;MAX_ED=96;MIN_ED=3;NLOD=60.56;TLOD=4.55	GT:AD:AF:ALT_F1R2:ALT_F2R1:FOXOG:PGT:PID:QSS:REF_F1R2:REF_F2R1	0/1:125,2:0.016:1:1:0.500:0|1:85610874_G_A:3530,60:60:65	0/0:293,9:0.031:3:6:0.667:0|1:85610874_G_A:8133,257:128:165	0.2	Tolerated	0	Neutral	93	30	0.573	Tolerated	3.17	15
T22,T2,T3	chr18	**3**	**37.5**	9975672	9975672	T	C	exonic	**Thoc1**	ENSMUSP00000053430	.	nonsynonymous SNV	Thoc1:NM_153552:exon10:c.T751C:p.C251R	**C251R**	ENSMUSP00000053430,251,C,R	18,9975672,T,G	18qA1	.	0.25	.	67	chr18	9975672	.	T	G	.	alt_allele_in_normal;clustered_events;germline_risk	ECNT=2;HCNT=2;MAX_ED=8;MIN_ED=8;NLOD=0.964;TLOD=11.32	GT:AD:AF:ALT_F1R2:ALT_F2R1:FOXOG:PGT:PID:QSS:REF_F1R2:REF_F2R1	0/1:74,4:0.056:2:2:0.500:0|1:3344581_A_G:2118,116:40:34	0/0:66,4:0.063:3:1:0.250:0|1:3344581_A_G:1817,105:39:27	1.9	Deleterious	-10.56	Deleterious	115	30	0.004	Damaging	2.87	71
T12,T2,T32	chr4	**3**	**37.5**	41195526	41195607	CTGTAGGATAAAGATGGCAATGCCGTCAGGTTGAACTGCACTCTTCGCCCATCTAACTGTGCCCACCTTTGTGCGACCTCAC	-	splicing	**Ubap2**	ENSMUSP00000047105	.	.	.	**NA**	4,41195526,TCTGTAGGATAAAGATGGCAATGCCGTCAGGTTGAACTGCACTCTTCGCCCATCTAACTGTGCCCACCTTTGTGCGACCTCAC,A	NA	4qA5	.	0.25	.	70	chr4	41195525	.	TCTGTAGGATAAAGATGGCAATGCCGTCAGGTTGAACTGCACTCTTCGCCCATCTAACTGTGCCCACCTTTGTGCGACCTCAC	A	.	alt_allele_in_normal	ECNT=1;HCNT=1;MAX_ED=.;MIN_ED=.;NLOD=15.26;TLOD=62.36	GT:AD:AF:ALT_F1R2:ALT_F2R1:FOXOG:QSS:REF_F1R2:REF_F2R1	0/1:131,42:0.287:20:22:.:3609,1117:61:70	0/0:55,2:0.055:1:1:.:1473,52:26:29	1.4	Unkown	.	.	.	.	.	.	.	.
T12,T22,T32	chr14	**3**	**37.5**	18331136	18331136	-	CTCTTTGTGTCTGGCTGCTCCGTGCC	splicing	**Ube2e1**	ENSMUSP00000023486	.	NM_009455:exon2:UTR6	.	**NA**	14,18331136,T,C	NA	14qA2	.	0.25	.	47	chr14	18331136	.	T	C	.	t_lod_fstar	ECNT=1;HCNT=4;MAX_ED=.;MIN_ED=.;NLOD=13.11;TLOD=5.23	GT:AD:AF:ALT_F1R2:ALT_F2R1:FOXOG:QSS:REF_F1R2:REF_F2R1	0/1:58,2:0.034:1:1:.:1637,59:33:25	0/0:49,0:0.00:0:0:.:1335,0:20:29	1.1	Unkown	.	.	.	.	.	.	.	.
T22,T2	chr7	**3**	**37.5**	98853274	98853274	A	G	exonic	**Wnt11**	ENSMUSP00000025706	.	nonsynonymous SNV	Wnt11:NM_001285795:exon5:c.A545G:p.Y182C,Wnt11:NM_009519:exon5:c.A1052G:p.Y351C,Wnt11:NM_001285794:exon6:c.A857G:p.Y286C,Wnt11:NM_001285792:exon7:c.A1052G:p.Y351C	**Y351C**	ENSMUSP00000025706,351,Y,C	7,98853274,A,G	7qE1	.	0.25	.	31	chr7	98853274	.	A	G	.	PASS	ECNT=1;HCNT=1;MAX_ED=.;MIN_ED=.;NLOD=8.42;TLOD=20.44	GT:AD:AF:ALT_F1R2:ALT_F2R1:FOXOG:QSS:REF_F1R2:REF_F2R1	0/1:33,9:0.220:4:5:0.556:918,255:14:19	0/0:31,0:0.00:0:0:.:866,0:9:22	7	Deleterious	-4.82	Deleterious	238	30	0.011	Damaging	2.84	100
T22,T2,T4	chr4	**3**	**37.5**	119282447	119282447	C	A	exonic	**Ybx1**	ENSMUSP00000133076	.	nonsynonymous SNV	Ybx1:NM_011732:exon5:c.G350T:p.G117V	**G117V**	ENSMUSP00000133076,117,G,V	4,119282447,C,A	4qD2.1	.	0.25	.	42	chr4	119282447	.	C	A	.	t_lod_fstar	ECNT=1;HCNT=1;MAX_ED=.;MIN_ED=.;NLOD=6.64;TLOD=5.43	GT:AD:AF:ALT_F1R2:ALT_F2R1:FOXOG:QSS:REF_F1R2:REF_F2R1	0/1:45,2:0.043:1:1:0.500:1132,51:24:21	0/0:44,1:0.027:1:0:1.00:1124,27:30:14	1.9	Deleterious	-7.87	Deleterious	213	30	0	Damaging	2.9	292

T22,T2,T4	chr4	**3**	**37.5**	119282450	119282450	-	TTTCTCCTTCA	splicing	**Ybx1**	ENSMUSP00000133076	.	NM_011732:exon5:c.349-2->TGAAGGAGAAA	.	**NA**	4,119282450,T,CTTCGGAATCG	NA	4qD2.1	.	0.25	.	40	chr4	119282450	.	T	CTTCGGAATCG	.	clustered_events;multi_event_alt_allele_in_normal;t_lod_fstar	ECNT=2;HCNT=3;MAX_ED=11;MIN_ED=11;NLOD=6.96;TLOD=5.62	GT:AD:AF:ALT_F1R2:ALT_F2R1:FOXOG:PGT:PID:QSS:REF_F1R2:REF_F2R1	0/1:36,2:0.054:0:2:.:0|1:119279189_G_A:1020,54:20:16	0/0:44,1:0.026:1:0:.:0|1:119279189_G_A:1229,27:30:14	1.9	Unkown	.	.	.	.	.	.	.	.
T22df	chr6	**2**	**25**	128571970	128571970	A	G	exonic	**A2ml1**	ENSMUSP00000115324	.	nonsynonymous SNV	A2ml1:NM_001001179:exon8:c.T848C:p.V283A	**V283A**	ENSMUSP00000115324,283,V,A	6,128571970,A,C	6qF3	rs31802577	0.25	.	39	chr6	128571970	rs31802577	A	C	.	PASS	ECNT=1;HCNT=1;MAX_ED=.;MIN_ED=.;NLOD=9.58;TLOD=8.42	GT:AD:AF:ALT_F1R2:ALT_F2R1:FOXOG:QSS:REF_F1R2:REF_F2R1	0/1:33,5:0.114:4:1:0.800:927,149:17:16	0/0:39,0:0.00:0:0:.:1097,0:16:23	2.1	Tolerated	-0.03	Neutral	135	30	0.413	Tolerated	2.79	99
T22,T3	chr10	**2**	**25**	80002613	80002613	C	G	exonic	**Abca7**	ENSMUSP00000084523	.	nonsynonymous SNV	Abca7:NM_001347081:exon13:c.C1720G:p.L574V,Abca7:NM_013850:exon13:c.C1720G:p.L574V	**L574V**	ENSMUSP00000084523,574,L,V	10,80002613,C,A	10qC1	.	0.25	.	26	chr10	80002613	.	C	A	.	t_lod_fstar	ECNT=1;HCNT=2;MAX_ED=.;MIN_ED=.;NLOD=12.59;TLOD=4.63	GT:AD:AF:ALT_F1R2:ALT_F2R1:FOXOG:QSS:REF_F1R2:REF_F2R1	0/1:54,3:0.037:0:3:.:1510,89:26:28	0/0:47,0:0.00:0:0:.:1313,0:20:27	23	Damaging	-1.9	Neutral	152	30	0.002	Damaging	2.82	135
T22,T3	chr12	**2**	**25**	112639728	112639728	G	A	exonic	**Adssl1**	ENSMUSP00000108032	.	nonsynonymous SNV	Adssl1:NM_007421:exon12:c.G1288A:p.V430M	**V430M**	ENSMUSP00000108032,430,V,M	12,112639728,G,A	12qF1	.	0.25	.	31	chr12	112639728	.	G	A	.	PASS	DB;ECNT=1;HCNT=1;MAX_ED=.;MIN_ED=.;NLOD=10.48;TLOD=64.39	GT:AD:AF:ALT_F1R2:ALT_F2R1:FOXOG:QSS:REF_F1R2:REF_F2R1	0/1:27,26:0.490:14:12:0.538:763,719:20:7	0/0:38,0:0.00:0:0:.:1036,0:16:22	4	Deleterious	-2.55	Deleterious	147	30	0.001	Damaging	2.78	341
T12,T1	chr8	**2**	**25**	71485789	71485789	T	A	exonic	**Ano8**	ENSMUSP00000122136	.	nonsynonymous SNV	Ano8:NM_001164679:exon1:c.A71T:p.E24V	**E24V**	ENSMUSP00000122136,24,E,V	8,71485789,T,A	8qB3.3	.	0.25	.	20	chr8	71485789	.	T	A	.	clustered_events;homologous_mapping_event;t_lod_fstar	ECNT=4;HCNT=1;MAX_ED=15;MIN_ED=4;NLOD=9.63;TLOD=5.18	GT:AD:AF:ALT_F1R2:ALT_F2R1:FOXOG:PGT:PID:QSS:REF_F1R2:REF_F2R1	0/1:61,2:0.033:0:2:0.00:0|1:46406919_C_A:1716,58:34:27	0/0:33,0:0.00:0:0:.:0|1:46406919_C_A:878,0:14:19	0.9	Damaging	-0.76	Neutral	76	30	0.004	Damaging	3.6	25
T2,T3	chr7	**2**	**25**	6997604	6997604	T	A	exonic	**Aurkc**	ENSMUSP00000035415	.	stopgain	Aurkc:NM_001080965:exon4:c.T423A:p.Y141X,Aurkc:NM_001080966:exon4:c.T306A:p.Y102X,Aurkc:NM_020572:exon4:c.T306A:p.Y102X	**Y102X**	ENSMUSP00000035415,102,Y,X	NA	7qA1	.	0.25	.	41	chr7	6997604	.	T	A	.	alt_allele_in_normal;clustered_events;germline_risk	ECNT=2;HCNT=22;MAX_ED=9;MIN_ED=9;NLOD=0.627;TLOD=53.26	GT:AD:AF:ALT_F1R2:ALT_F2R1:FOXOG:PGT:PID:QSS:REF_F1R2:REF_F2R1	0/1:54,16:0.239:12:4:0.750:0|1:6997633_A_C:1338,452:25:29	0/0:35,6:0.118:3:3:0.500:0|1:6997633_A_C:890,166:18:17	2.4	Deletarious	.	.	.	.	.	.	.	.
T2,T3	chr7	**2**	**25**	6997604	6997604	T	A	exonic	**Aurkc**	ENSMUSP00000035415	.	stopgain	Aurkc:NM_001080965:exon4:c.T423A:p.Y141X,Aurkc:NM_001080966:exon4:c.T306A:p.Y102X,Aurkc:NM_020572:exon4:c.T306A:p.Y102X	**Y141X**	ENSMUSP00000035415,141,Y,X	NA	7qA1	.	0.25	.	41	chr7	6997604	.	T	C	.	alt_allele_in_normal;clustered_events;germline_risk	ECNT=2;HCNT=22;MAX_ED=9;MIN_ED=9;NLOD=0.927;TLOD=48.96	GT:AD:AF:ALT_F1R2:ALT_F2R1:FOXOG:PGT:PID:QSS:REF_F1R2:REF_F2R1	0/1:45,14:0.237:11:3:0.214:0|1:6997633_A_C:1288,404:23:22	0/0:38,6:0.114:3:3:0.500:0|1:6997633_A_C:1066,164:21:17	2.4	Deletarious	.	.	.	.	.	.	.	.
T1, T2	chr8	**2**	**25**	27124170	27124172	CTG	-	exonic	**Brf2**	ENSMUSP00000112474	.	nonframeshift deletion	Brf2:NM_025686:exon4:c.985_987del:p.329_329del	**329_329del**	NA	8,27124170,CCTG,A	8qA2	rs228847142	0.25	.	21	chr8	27124169	.	CCTG	A	.	str_contraction;t_lod_fstar	ECNT=1;HCNT=8;MAX_ED=.;MIN_ED=.;NLOD=8.42;RPA=7,6;RU=CAG;STR;TLOD=4.61	GT:AD:AF:ALT_F1R2:ALT_F2R1:FOXOG:QSS:REF_F1R2:REF_F2R1	0/1:59,2:0.033:0:2:.:1662,57:28:31	0/0:31,0:0.00:0:0:.:872,0:20:11	18	Unknown	.	.	.	.	.	.	.	.
T22, T4	chr5	**2**	**25**	122758957	122758957	C	T	exonic	**Camkk2**	ENSMUSP00000124119	.	nonsynonymous SNV	Camkk2:NM_001199676:exon3:c.G515A:p.G172E,Camkk2:NM_145358:exon3:c.G515A:p.G172E	**G172E**	ENSMUSP00000124119,172,G,E	5,122758957,C,G	5qF	.	0.25	.	50	chr5	122758957	.	C	G	.	PASS	DB;ECNT=1;HCNT=1;MAX_ED=.;MIN_ED=.;NLOD=8.97;TLOD=49.70	GT:AD:AF:ALT_F1R2:ALT_F2R1:FOXOG:QSS:REF_F1R2:REF_F2R1	0/1:14,23:0.600:11:12:0.522:339,550:5:9	0/0:33,0:0.00:0:0:.:819,0:22:11	0.2	Deleterious	-5.64	Deleterious	172	30	0.003	Damaging	2.92	235
T1, T3	chr16	**2**	**25**	93609818	93609818	A	C	exonic	**Cbr1**	ENSMUSP00000107297	.	nonsynonymous SNV	Cbr1:NM_007620:exon3:c.A421C:p.M141L	**M141L**	ENSMUSP00000107297,141,M,L	16,93609818,A,C	16qC4	.	0.25	.	48	chr16	93609818	.	A	C	.	PASS	ECNT=1;HCNT=1;MAX_ED=.;MIN_ED=.;NLOD=9.62;TLOD=20.26	GT:AD:AF:ALT_F1R2:ALT_F2R1:FOXOG:QSS:REF_F1R2:REF_F2R1	0/1:42,10:0.180:5:5:0.500:1186,293:22:20	0/0:36,0:0.00:0:0:.:1006,0:14:22	0.8	Tolerated	-0.66	Neutral	184	30	1	Tolerated	2.8	190
T32, T3	chr7	**2**	**25**	16995954	16995954	T	C	exonic	**Ccdc8**	ENSMUSP00000145329	.	nonsynonymous SNV	Ccdc8:NM_001101535:exon1:c.T1367C:p.V456A	**V456A**	ENSMUSP00000145329,456,V,A	7,16995954,T,C	7qA2	.	0.25	.	39	chr7	16995954	.	T	C	.	alt_allele_in_normal;germline_risk	ECNT=1;HCNT=1;MAX_ED=.;MIN_ED=.;NLOD=1.91;TLOD=12.93	GT:AD:AF:ALT_F1R2:ALT_F2R1:FOXOG:QSS:REF_F1R2:REF_F2R1	0/1:28,16:0.357:7:9:0.438:599,221:19:9	0/0:34,5:0.132:4:1:0.800:837,74:14:20	0.6	Tolerated	-0.38	Neutral	50	30	1	Tolerated	2.94	70

T42, T4	chr16	**2**	**25**	44788755	44788755	G	C	exonic	**Cd200r1**	ENSP00000295863	.	nonsynonymous SNV	Cd200r1:NM_021325:exon2:c.G74C:p.C25S	**C25S**	ENSP00000295863,25,C,S	16,44788755,G,C	16qB4	.	0.25	.	68	chr16	44788755	.	G	C	.	clustered_events;multi_event_alt_allele_in_normal	ECNT=9;HCNT=4;MAX_ED=82;MIN_ED=5;NLOD=13.81;TLOD=9.02	GT:AD:AF:ALT_F1R2:ALT_F2R1:FOXOG:PGT:PID:QSS:REF_F1R2:REF_F2R1	0/1:33,3:0.083:2:1:0.667:0|1:44788673_A_G:903,87:24:9	0/0:67,1:0.016:0:1:0.00:0|1:44788673_A_G:1866,29:39:28	1.1	Deleterious	-2.81	Deleterious	81	30	0.145	Tolerated	3.1	53
T1, T42	chr1	**2**	**25**	171559334	171559334	C	A	exonic	**Cd244**	ENSMUSP00000142896	.	nonsynonymous SNV	Cd244:NM_018729:exon1:c.C10A:p.Q4K	**Q4K**	ENSMUSP00000142896,4,Q,K	1,171559334,C,A	1qH3	rs216356497	0.25	.	182	chr1	171559334	.	C	A	.	alt_allele_in_normal;t_lod_fstar	ECNT=1;HCNT=9;MAX_ED=.;MIN_ED=.;NLOD=30.77;TLOD=4.01	GT:AD:AF:ALT_F1R2:ALT_F2R1:FOXOG:QSS:REF_F1R2:REF_F2R1	0/1:264,4:0.016:3:1:0.250:7648,110:129:135	0/0:176,6:0.036:2:4:0.667:5004,170:100:76	20	Damaging	-0.95	Neutral	101	30	0.021	Damaging	3.33	35
T2, T3	chr1	**2**	**25**	171577335	171577335	A	T	exonic	**Cd244**	ENSMUSP00000142896	.	nonsynonymous SNV	Cd244:NM_018729:exon4:c.A754T:p.R252W	**R252W**	ENSMUSP00000142896,252,R,W	1,171577335,A,T	1qH3	.	0.25	.	127	chr1	171577335	.	A	T	.	alt_allele_in_normal	ECNT=1;HCNT=4;MAX_ED=.;MIN_ED=.;NLOD=3.46;TLOD=18.20	GT:AD:AF:ALT_F1R2:ALT_F2R1:FOXOG:QSS:REF_F1R2:REF_F2R1	0/1:166,14:0.074:7:7:0.500:4732,377:86:80	0/0:116,11:0.096:9:2:0.182:3288,304:61:55	20	Deleterious	-4.29	Deleterious	101	30	0.003	Damaging	2.87	56
T1, T4	chr1	**2**	**25**	171579119	171579119	-	C	exonic	**Cd244**	ENSMUSP00000142896	.	frameshift insertion	Cd244:NM_018729:exon6:c.943dupC:p.Q314fs	**Q314fs**	ENSMUSP00000142896,314,Q,	1,171579119,G,A	1qH3	.	0.25	.	164	chr1	171579119	.	G	A	.	alt_allele_in_normal;t_lod_fstar	ECNT=1;HCNT=9;MAX_ED=.;MIN_ED=.;NLOD=30.77;TLOD=4.01	GT:AD:AF:ALT_F1R2:ALT_F2R1:FOXOG:QSS:REF_F1R2:REF_F2R1	0/1:264,4:0.016:3:1:0.250:7648,110:129:135	0/0:176,6:0.036:2:4:0.667:5004,170:100:76	20	Tolerated	0	Neutral	101	30	0.648	Tolerated	3.29	35
T1, T4	chr1	**2**	**25**	171579128	171579128	-	T	exonic	**Cd244**	ENSMUSP00000142896	.	frameshift insertion	Cd244:NM_018729:exon6:c.951_952insT:p.F317fs	**F317fs**	ENSMUSP00000142896,317,F,	1,171579128,C,T	1qH3	.	0.25	.	166	chr1	171579128	.	C	T	.	alt_allele_in_normal	ECNT=1;HCNT=4;MAX_ED=.;MIN_ED=.;NLOD=3.46;TLOD=18.20	GT:AD:AF:ALT_F1R2:ALT_F2R1:FOXOG:QSS:REF_F1R2:REF_F2R1	0/1:166,14:0.074:7:7:0.500:4732,377:86:80	0/0:116,11:0.096:9:2:0.182:3288,304:61:55	20	Tolerated	0	Neutral	101	30	0.538	Tolerated	3.1	38
T1, T4	chr1	**2**	**25**	171579124	171579125	CT	-	exonic	**Cd244**	ENSMUSP00000142896	.	frameshift deletion	Cd244:NM_018729:exon6:c.947_948del:p.T316fs	**T316fs**	NA	1,171579123,ACT,A	1qH3	.	0.25	.	165	chr1	171579123	.	ACT	A	.	alt_allele_in_normal;clustered_events	ECNT=3;HCNT=1;MAX_ED=9;MIN_ED=4;NLOD=23.04;TLOD=7.27	GT:AD:AF:ALT_F1R2:ALT_F2R1:FOXOG:PGT:PID:QSS:REF_F1R2:REF_F2R1	0/1:147,6:0.033:1:5:.:0|1:171579119_G_GC:4106,172:62:85	0/0:156,9:0.057:1:8:.:0|1:171579119_G_GC:4293,253:84:72	20	Unknown	NA	NA	101	30	NA	NA	NA	NA
T2, T4	chr8	**2**	**25**	119236901	119236901	C	A	exonic	**Cdh13**	ENSMUSP00000101740	.	nonsynonymous SNV	Cdh13:NM_019707:exon9:c.C1219A:p.P407T	**P407T**	ENSMUSP00000101740,407,P,T	8,119236901,C,A	8qE1	.	0.25	.	31	chr8	119236901	.	C	A	.	clustered_events;homologous_mapping_event;t_lod_fstar	ECNT=9;HCNT=1;MAX_ED=31;MIN_ED=4;NLOD=9.33;TLOD=5.39	GT:AD:AF:ALT_F1R2:ALT_F2R1:FOXOG:PGT:PID:QSS:REF_F1R2:REF_F2R1	0/1:46,2:0.042:2:0:1.00:0|1:119236891_C_CTTTTT:1191,47:26:20	0/0:32,0:0.00:0:0:.:0|1:119236891_C_CTTTTTT:884,0:12:20	1.5	Deleterious	-5.24	Deleterious	238	30	0.002	Damaging	2.85	176
T2, T4	chr8	**2**	**25**	119236914	119236914	T	C	exonic	**Cdh13**	ENSMUSP00000101740	.	nonsynonymous SNV	Cdh13:NM_019707:exon9:c.T1232C:p.F411S	**F411S**	ENSMUSP00000101740,411,F,S	8,119236914,T,C	8qE1	.	0.25	.	31	chr8	119236914	.	T	C	.	clustered_events;homologous_mapping_event;t_lod_fstar	ECNT=9;HCNT=1;MAX_ED=31;MIN_ED=4;NLOD=9.03;TLOD=5.43	GT:AD:AF:ALT_F1R2:ALT_F2R1:FOXOG:PGT:PID:QSS:REF_F1R2:REF_F2R1	0/1:44,2:0.043:2:0:.:0|1:119236891_C_CTTTTTT:1141,42:27:17	0/0:31,0:0.00:0:0:.:0|1:119236891_C_CTTTTTT:846,0:10:21	1.5	Deleterious	-6.66	Deleterious	238	30	0	Damaging	2.85	176
T2, T4	chr8	**2**	**25**	119236911	119236911	G	C	exonic	**Cdh13**	ENSMUSP00000101740	.	nonsynonymous SNV	Cdh13:NM_019707:exon9:c.G1229C:p.S410T	**S410T**	ENSMUSP00000101740,410,S,T	NA	8qE1	.	0.25	.	31	chr8	119236911	.	G	C	.	clustered_events;homologous_mapping_event;t_lod_fstar	ECNT=9;HCNT=1;MAX_ED=31;MIN_ED=4;NLOD=9.33;TLOD=5.39	GT:AD:AF:ALT_F1R2:ALT_F2R1:FOXOG:PGT:PID:QSS:REF_F1R2:REF_F2R1	0/1:46,2:0.042:2:0:1.00:0|1:119236891_C_CTTTTT:1198,53:25:21	0/0:31,0:0.00:0:0:.:0|1:119236891_C_CTTTTTT:836,0:11:20	1.5	Tolerated	-0.56	Neutral	114	30	0.572	Tolerated	2.98	87
T2, T4	chr8	**2**	**25**	119236913	119236913	T	A	exonic	**Cdh13**	ENSMUSP00000101740	.	nonsynonymous SNV	Cdh13:NM_019707:exon9:c.T1231A:p.F411I	**F411I**	ENSMUSP00000101740,411,F,I	8,119236913,T,T	8qE1	.	0.25	.	32	chr8	119236913	.	T	T	.	clustered_events;homologous_mapping_event;t_lod_fstar	ECNT=9;HCNT=2;MAX_ED=31;MIN_ED=4;NLOD=9.32;TLOD=5.52	GT:AD:AF:ALT_F1R2:ALT_F2R1:FOXOG:PGT:PID:QSS:REF_F1R2:REF_F2R1	0/1:43,2:0.048:2:0:0.00:0|1:119236891_C_CTTTTT:1134,51:24:19	0/0:33,0:0.00:0:0:.:0|1:119236891_C_CTTTTTT:932,0:12:21	1.5	Tolerated	0	Neutral	238	30	1	Tolerated	2.85	176
T2, T4	chr8	**2**	**25**	119236922	119236922	C	T	exonic	**Cdh13**	ENSMUSP00000101740	.	nonsynonymous SNV	Cdh13:NM_019707:exon9:c.C1240T:p.H414Y	**H414Y**	ENSMUSP00000101740,414,H,Y	8,119236922,C,C	8qE1	.	0.25	.	33	chr8	119236922	.	C	C	.	clustered_events;homologous_mapping_event;t_lod_fstar	ECNT=9;HCNT=1;MAX_ED=31;MIN_ED=4;NLOD=9.33;TLOD=5.41	GT:AD:AF:ALT_F1R2:ALT_F2R1:FOXOG:PGT:PID:QSS:REF_F1R2:REF_F2R1	0/1:44,2:0.043:2:0:.:0|1:119236891_C_CTTTTTT:1173,39:27:17	0/0:32,0:0.00:0:0:.:0|1:119236891_C_CTTTTTT:882,0:10:22	1.5	Tolerated	0	Neutral	238	30	0.396	Tolerated	2.85	175

T2, T4	chr8	**2**	**25**	119236891	119236891	-	TTTTTT	exonic	**Cdh13**	ENSMUSP00000101740	.	nonframeshift insertion	Cdh13:NM_019707:exon9:c.1209_1210insTTTTTT:p.I403delinsIFF	**I403delinsIFF**	ENSMUSP00000101740,403,I,IFF	8,119236891,C,C	8qE1	.	0.25	.	31	chr8	119236891	.	C	C	.	alt_allele_in_normal;clustered_events	ECNT=5;HCNT=1;MAX_ED=24;MIN_ED=1;NLOD=5.43;TLOD=11.84	GT:AD:AF:ALT_F1R2:ALT_F2R1:FOXOG:PGT:PID:QSS:REF_F1R2:REF_F2R1	0/1:50,4:0.075:4:0:.:0|1:136373432_T_TTTTTAATGATACG:1253,57:21:29	0/0:37,1:0.030:1:0:.:0|1:136373432_T_TTTTTAATGATACG:938,22:13:24	1.4	Tolerated	0	Neutral	238	30	1	Tolerated	2.85	176
T2, T4	chr8	**2**	**25**	119236903	119236904	TG	-	exonic	**Cdh13**	ENSMUSP00000101740	.	frameshift deletion	Cdh13:NM_019707:exon9:c.1221_1222del:p.P407fs	**P407fs**	ENSMUSP00000101740,407,P,.	8,119236903,CTG,G	8qE1	.	0.25	.	31	chr8	119236902	.	CTG	G	.	clustered_events;homologous_mapping_event;t_lod_fstar	ECNT=9;HCNT=1;MAX_ED=31;MIN_ED=4;NLOD=9.03;TLOD=5.45	GT:AD:AF:ALT_F1R2:ALT_F2R1:FOXOG:PGT:PID:QSS:REF_F1R2:REF_F2R1	0/1:43,2:0.044:2:0:.:0|1:119236891_C_CTTTTTT:1149,38:27:16	0/0:31,0:0.00:0:0:.:0|1:119236891_C_CTTTTTT:850,0:11:20	1.5	Unknown	NA	NA	238	30	NA	NA	NA	NA
T2, T4	chr8	**2**	**25**	119236908	119236908	A	-	exonic	**Cdh13**	ENSMUSP00000101740	.	frameshift deletion	Cdh13:NM_019707:exon9:c.1226delA:p.Q409fs	**Q409fs**	ENSMUSP00000101740,409,Q,.	8,119236908,CA,CTTTTTT	8qE1	.	0.25	.	32	chr8	119236907	.	CA	CTTTTTT	.	clustered_events;homologous_mapping_event;t_lod_fstar	ECNT=9;HCNT=1;MAX_ED=31;MIN_ED=4;NLOD=9.03;TLOD=5.47	GT:AD:AF:ALT_F1R2:ALT_F2R1:FOXOG:PGT:PID:QSS:REF_F1R2:REF_F2R1	0/1:44,2:0.045:2:0:.:0|1:119236891_C_CTTTTTT:1254,45:26:18	0/0:31,0:0.00:0:0:.:0|1:119236891_C_CTTTTTT:876,0:10:21	1.5	Unknown	NA	NA	238	30	NA	NA	NA	NA
T2, T4	chr8	**2**	**25**	119236896	119236898	GAA	-	exonic	**Cdh13**	ENSMUSP00000101740	.	nonframeshift deletion	Cdh13:NM_019707:exon9:c.1214_1216del:p.405_406del	**405_406del**	NA	8,119236896,GGAA,G	8qE1	.	0.25	.	31	chr8	119236895	.	GGAA	G	.	alt_allele_in_normal;clustered_events	ECNT=5;HCNT=1;MAX_ED=24;MIN_ED=1;NLOD=5.43;RPA=3,1;RU=C;STR;TLOD=11.81	GT:AD:AF:ALT_F1R2:ALT_F2R1:FOXOG:PGT:PID:QSS:REF_F1R2:REF_F2R1	0/1:49,4:0.074:4:0:.:0|1:136373432_T_TTTTTAATGATACG:1370,115:23:26	0/0:38,1:0.030:1:0:.:0|1:136373432_T_TTTTTAATGATACG:1066,27:14:24	1.4	Unknown	.	.	.	.	.	.	.	.
T12, T32	chr2	**2**	**25**	29054987	29054987	C	G	exonic	**Cfap77**	ENSMUSP00000124323	.	nonsynonymous SNV	Cfap77:NM_001166705:exon1:c.G22C:p.G8R	**G8R**	ENSMUSP00000124323,8,G,R	2,29054987,C,A	2qA3	rs212345576	0.25	.	33	chr2	29054987	rs212345576	C	A	.	str_contraction	ECNT=1;HCNT=1;MAX_ED=.;MIN_ED=.;NLOD=6.59;RPA=8,7;RU=CAG;STR;TLOD=7.63	GT:AD:AF:ALT_F1R2:ALT_F2R1:FOXOG:QSS:REF_F1R2:REF_F2R1	0/1:41,3:0.070:1:2:.:1149,84:16:25	0/0:22,0:0.00:0:0:.:608,0:9:13	0.7	Tolerated	-0.67	Neutral	27	10	0.189	Tolerated	3.75	23
T12, T4	chr19	**2**	**25**	5492805	5492805	-	GCCCCCGAGAA	splicing	**Cfl1**	ENSMUSP00000142967	.	.	.	**NA**	19,5492805,G,GGCCCCCGAGAA	NA	19qA	.	0.25	.	46	chr19	5492805	.	G	GGCCCCCGAGAA	.	alt_allele_in_normal	ECNT=1;HCNT=2;MAX_ED=.;MIN_ED=.;NLOD=4.00;TLOD=8.34	GT:AD:AF:ALT_F1R2:ALT_F2R1:FOXOG:QSS:REF_F1R2:REF_F2R1	0/1:57,3:0.051:1:2:.:1574,81:28:29	0/0:44,2:0.047:0:2:.:1200,56:24:20	1.6	Unkown	.	.	.	.	.	.	.	.
T22, T3	chr5	**2**	**25**	115982138	115982138	G	C	exonic	**Cit**	ENSMUSP00000112259	.	nonsynonymous SNV	Cit:NM_007708:exon31:c.G4011C:p.R1337S	**R1337S**	ENSMUSP00000112259,1337,R,S	5,115982138,G,C	5qF	.	0.25	.	36	chr5	115982138	.	G	C	.	str_contraction;t_lod_fstar	ECNT=1;HCNT=2;MAX_ED=.;MIN_ED=.;NLOD=5.71;RPA=8,7;RU=CTG;STR;TLOD=4.67	GT:AD:AF:ALT_F1R2:ALT_F2R1:FOXOG:QSS:REF_F1R2:REF_F2R1	0/1:45,2:0.044:0:2:.:1261,57:18:27	0/0:21,0:0.00:0:0:.:580,0:17:4	14	Damaging	-0.66	Neutral	110	30	0.014	Damaging	3.03	70
T2, T42	chr6	**2**	**25**	129183217	129183217	-	GA	exonic	**Clec2d**	ENSMUSP00000143151	.	frameshift insertion	Clec2d:NM_053109:exon2:c.193_194insGA:p.A65fs	**A65fs**	ENSMUSP00000143151,65,A,	6,129183217,G,A	6qF3	.	0.25	.	44	chr6	129183217	.	G	A	.	alt_allele_in_normal;clustered_events	ECNT=3;HCNT=1;MAX_ED=9;MIN_ED=4;NLOD=23.04;TLOD=7.27	GT:AD:AF:ALT_F1R2:ALT_F2R1:FOXOG:PGT:PID:QSS:REF_F1R2:REF_F2R1	0/1:147,6:0.033:1:5:.:0|1:171579119_G_GC:4106,172:62:85	0/0:156,9:0.057:1:8:.:0|1:17157911 9_G_GC:4293,253:84:72	20	Tolerated	-0.79	Neutral	104	30	0.208	Tolerated	2.79	83
T2, T42	chr6	**2**	**25**	129183215	129183216	CA	-	exonic	**Clec2d**	ENSMUSP00000143151	.	frameshift deletion	Clec2d:NM_053109:exon2:c.191_192del:p.S64fs	**S64fs**	NA	6,129183214,TCA,T	6qF3	.	0.25	.	44	chr6	129183214	.	TCA	T	.	clustered_events;homologous_mapping_event;t_lod_fstar	ECNT=12;HCNT=8;MAX_ED=64;MIN_ED=3;NLOD=11.74;TLOD=5.29	GT:AD:AF:ALT_F1R2:ALT_F2R1:FOXOG:PGT:PID:QSS:REF_F1R2:REF_F2R1	0/1:54,2:0.037:2:0:.:0|1:129183214_TCA_T:1532,56:22:32	0/0:44,0:0.00:0:0:.:0|1:129183214_TCA_T:1243,0:23:21	2.1	Unknown	NA	NA	104	30	NA	NA	NA	NA
T12, T32	chr17	**2**	**25**	43746579	43746579	C	G	exonic	**Cyp39a1**	ENSMUSP00000004097	.	stopgain	Cyp39a1:NM_001285947:exon10:c.C1158G:p.Y386X,Cyp39a1:NM_018887:exon11:c.C1308G:p.Y436X	**Y386X**	ENSMUSP00000004097,386,Y,X	NA	17qB3	.	0.25	.	35	chr17	43746579	.	C	A	.	t_lod_fstar	ECNT=1;HCNT=1;MAX_ED=.;MIN_ED=.;NLOD=12.64;TLOD=4.92	GT:AD:AF:ALT_F1R2:ALT_F2R1:FOXOG:QSS:REF_F1R2:REF_F2R1	0/1:25,1:0.077:0:1:1.00:719,29:13:12	0/0:49,0:0.00:0:0:.:1315,0:28:21	4	Deletarious	.	.	.	.	.	.	.	.

T12, T32	chr17	**2**	**25**	43746579	43746579	C	G	exonic	**Cyp39a1**	ENSMUSP00000004097	.	stopgain	Cyp39a1:NM_001285947:exon10:c.C1158G:p.Y386X,Cyp39a1:NM_018887:exon11:c.C1308G:p.Y436X	**Y436X**	ENSMUSP00000004097,436,Y,X	NA	17qB3	.	0.25	.	35	chr17	43746579	.	C	A	.	alt_allele_in_normal;clustered_events;germline_risk	DB;ECNT=2;HCNT=1;MAX_ED=10;MIN_ED=10;NLOD=2.37;TLOD=8.66	GT:AD:AF:ALT_F1R2:ALT_F2R1:FOXOG:PGT:PID:QSS:REF_F1R2:REF_F2R1	0/1:63,4:0.063:1:3:0.250:0|1:78349046_T_A:1779,109:34:29	0/0:51,4:0.080:3:1:0.750:0|1:78349046_T_A:1377,88:28:23	0.7	Deletarious	.	.	.	.	.	.	.	.
T1,T32	chr3	**2**	**25**	34057981	34057982	CA	-	exonic	**Dnajc19**	ENSMUSP00000052755	.	frameshift deletion	Dnajc19:NM_001026211:exon6:c.413_414del:p.V138fs	**V138fs**	ENSMUSP00000052755,138,V,.	3,34057981,GCA,G	3qA3	.	0.25	.	33	chr3	34057980	.	GCA	G	.	str_contraction;t_lod_fstar	ECNT=1;HCNT=1;MAX_ED=.;MIN_ED=.;NLOD=5.70;RPA=5,4;RU=GCT;STR;TLOD=5.12	GT:AD:AF:ALT_F1R2:ALT_F2R1:FOXOG:QSS:REF_F1R2:REF_F2R1	0/1:67,2:0.031:1:1:.:.:1702,51:41:26	0/0:24,0:0.00:0:0:.:.:626,0:14:10	1.7	Deleterious	-9.35	Deleterious	83	30	NA	NA	NA	NA
T12, T32	chr2	**2**	**25**	26351942	26351942	C	T	exonic	**Dnlz**	ENSMUSP00000131359	.	nonsynonymous SNV	Dnlz:NM_001139503:exon1:c.G71A:p.R24Q,Dnlz:NM_001139504:exon1:c.G71A:p.R24Q,Dnlz:NM_026828:exon1:c.G71A:p.R24Q	**R24Q**	ENSMUSP00000131359,24,R,Q	2,26351942,C,G	2qA3	rs27215938	0.25	.	47	chr2	26351942	rs27215938	C	G	.	homologous_mapping_event;t_lod_fstar	ECNT=3;HCNT=1;MAX_ED=2;MIN_ED=1;NLOD=16.86;TLOD=5.12	GT:AD:AF:ALT_F1R2:ALT_F2R1:FOXOG:PGT:PID:QSS:REF_F1R2:REF_F2R1	0/1:64,2:0.031:2:0:0.00:0|1:37475669_A_T:1609,58:33:31	0/0:67,0:0.00:0:0:.:0|1:37475669_A_T:1668,0:34:33	0.7	Damaging	-1.6	Neutral	58	30	0.014	Damaging	3.39	19
T12, T32	chr2	**2**	**25**	26351863	26351874	CCAGCCCGAGGC	-	exonic	**Dnlz**	ENSMUSP00000131359	.	nonframeshift deletion	Dnlz:NM_001139503:exon1:c.139_150del:p.47_50del,Dnlz:NM_001139504:exon1:c.139_150del:p.47_50del,Dnlz:NM_026828:exon1:c.139_150del:p.47_50del	**47_50del**	NA	2,26351863,GCCAGCCCGAGGC,T	2qA3	rs262480195	0.25	.	32	chr2	26351862	.	GCCAGCCCGAGGC	T	.	homologous_mapping_event;t_lod_fstar	ECNT=3;HCNT=1;MAX_ED=2;MIN_ED=1;NLOD=17.16;TLOD=5.12	GT:AD:AF:ALT_F1R2:ALT_F2R1:FOXOG:PGT:PID:QSS:REF_F1R2:REF_F2R1	0/1:63,2:0.031:2:0:1.00:0|1:37475669_A_T:1614,56:32:31	0/0:66,0:0.00:0:0:.:0|1:37475669_A_T:1663,0:34:32	0.7	Unknown	.	.	.	.	.	.	.	.
T12, T42	chr19	**2**	**25**	41053182	41053182	-	C	exonic	**Dntt**	ENSMUSP00000079295	.	frameshift insertion	Dntt:NM_001043228:exon9:c.1304_1305insC:p.L435fs,Dntt:NM_009345:exon9:c.1304_1305insC:p.L435fs	**L435fs**	ENSMUSP00000079295,435,L,	19,41053182,T,C	19qC3	.	0.25	.	39	chr19	41053182	.	T	C	.	str_contraction	ECNT=1;HCNT=2;MAX_ED=.;MIN_ED=.;NLOD=9.32;RPA=6,5;RU=GCA;STR;TLOD=8.03	GT:AD:AF:ALT_F1R2:ALT_F2R1:FOXOG:QSS:REF_F1R2:REF_F2R1	0/1:69,3:0.042:1:2:.:.:1700,83:37:32	0/0:32,0:0.00:0:0:.:.:792,0:13:19	7	Deleterious	-6.52	Deleterious	111	30	0	Damaging	2.89	92
T12,T42	chr19	**2**	**25**	41053206	41053206	-	TATCATTAAAAA	exonic	**Dntt**	ENSMUSP00000079295	.	nonframeshift insertion	Dntt:NM_001043228:exon9:c.1328_1329insTATCATTAAAAA:p.R443delinsRIIKK,Dntt:NM_009345:exon9:c.1328_1329insTATCATTAAAAA:p.R443delinsRIIKK	**R443delinsRIIKK**	ENSMUSP00000079295,443,R,RIIKK	19,41053206,G,A	19qC3	.	0.25	.	44	chr19	41053206	.	G	A	.	str_contraction;t_lod_fstar	ECNT=1;HCNT=1;MAX_ED=.;MIN_ED=.;NLOD=8.09;RPA=7,6;RU=CAG;STR;TLOD=4.73	GT:AD:AF:ALT_F1R2:ALT_F2R1:FOXOG:QSS:REF_F1R2:REF_F2R1	0/1:62,2:0.032:0:2:.:.:1770,52:31:31	0/0:28,0:0.00:0:0:.:.:792,0:12:16	7	Tolerated	0.02	Neutral	111	30	0.066	Tolerated	2.89	92
T12, T42	chr19	**2**	**25**	41053200	41053200	A	G	exonic	**Dntt**	ENSMUSP00000079295	.	nonsynonymous SNV	Dntt:NM_001043228:exon9:c.A1322G:p.D441G,Dntt:NM_009345:exon9:c.A1322G:p.D441G	**D441G**	ENSMUSP00000079295,441,D,G	19,41053200,A,TC	19qC3	.	0.25	.	41	chr19	41053200	.	A	TC	.	clustered_events;multi_event_alt_allele_in_normal	ECNT=5;HCNT=1;MAX_ED=25;MIN_ED=2;NLOD=6.64;TLOD=11.62	GT:AD:AF:ALT_F1R2:ALT_F2R1:FOXOG:PGT:PID:QSS:REF_F1R2:REF_F2R1	0/1:59,4:0.067:0:4:.:.:0|1:41053182_T_TC:1692,113:30:29	0/0:38,1:0.027:0:1:.:.:0|1:41053182_T_TC:1078,29:23:15	0.4	Unknown	NA	NA	111	30	NA	NA	NA	NA
T12, T42	chr19	**2**	**25**	41053185	41053197	TCATGTGCCCCTA	-	exonic	**Dntt**	ENSMUSP00000079295	.	frameshift deletion	Dntt:NM_001043228:exon9:c.1307_1319del:p.V436fs,Dntt:NM_009345:exon9:c.1307_1319del:p.V436fs	**V436fs**	ENSMUSP00000079295,436,V,.	19,41053185,GTCATGTGCCCCTA,GTATCATTAAAAA	19qC3	.	0.25	.	43	chr19	41053184	.	GTCATGTGCCCCTA	GTATCATTAAAAA	.	clustered_events;multi_event_alt_allele_in_normal	ECNT=5;HCNT=1;MAX_ED=25;MIN_ED=2;NLOD=7.54;TLOD=11.47	GT:AD:AF:ALT_F1R2:ALT_F2R1:FOXOG:PGT:PID:QSS:REF_F1R2:REF_F2R1	0/1:58,4:0.062:0:4:.:.:0|1:41053182_T_TC:1376,49:29:29	0/0:43,1:0.025:0:1:.:.:0|1:41053182_T_TC:1007,11:25:18	0.4	Unknown	NA	NA	111	30	NA	NA	NA	NA
T12,T32	chr9	**2**	**25**	45751097	45751097	-	G	exonic	**Dscaml1**	ENSMUSP00000101915	.	frameshift insertion	Dscaml1:NM_001081270:exon31:c.5361dupG:p.V1787fs	**V1787fs**	ENSMUSP00000101915,1787,V,	9,45751097,T,A	9qA5.2	.	0.25	.	33	chr9	45751097	.	T	A	.	t_lod_fstar	ECNT=2;HCNT=1;MAX_ED=1;MIN_ED=1;NLOD=20.17;TLOD=5.29	GT:AD:AF:ALT_F1R2:ALT_F2R1:FOXOG:PGT:PID:QSS:REF_F1R2:REF_F2R1	0/1:55,2:0.037:2:0:0.00:0|1:121559024_C_A:1517,56:30:25	0/0:71,0:0.00:0:0:.:0|1:121559024_C_A:1893,0:33:38	2	Damaging	-1.81	Neutral	205	30	0.017	Damaging	3.37	91
T12,T32	chr9	**2**	**25**	45751107	45751113	GAATGTG	-	exonic	**Dscaml1**	ENSMUSP00000119959	.	frameshift deletion	Dscaml1:NM_001081270:exon31:c.5370_5376del:p.K1790fs	**K1790fs**	ENSMUSP00000119959,1790,K,.	NA	9qA5.2	.	0.25	.	36	chr9	45751106	.	AGAATGTG	CT	.	clustered_events;homologous_mapping_event;t_lod_fstar	ECNT=8;HCNT=1;MAX_ED=23;MIN_ED=2;NLOD=9.03;TLOD=5.03	GT:AD:AF:ALT_F1R2:ALT_F2R1:FOXOG:PGT:PID:QSS:REF_F1R2:REF_F2R1	0/1:68,2:0.028:0:2:.:.:0|1:45751093_C_CT:1896,56:42:26	0/0:33,0:0.00:0:0:.:0|1:45751093_C_CT:918,0:23:10	0.9	Deleterious	-10.89	Deleterious	144	30	NA	NA	NA	NA

T12,T32	chr9	**2**	**25**	45751117	45751118	TC	-	exonic	**Dscaml1**	ENSMUSP00000081864	.	frameshift deletion	Dscaml1:NM_001081270:exon31:c.5380_5381del:p.S1794fs	**S1794fs**	ENSMUSP00000081864,1794,S,.	NA	9qA5.2	.	0.25	.	37	chr9	45751116	.	ATC	TG	.	clustered_events;homologous_mapping_event;t_lod_fstar	ECNT=8;HCNT=1;MAX_ED=23;MIN_ED=2;NLOD=9.03;RPA=1,2;RU=G;STR;TLOD=5.06	GT:AD:AF:ALT_F1R2:ALT_F2R1:FOXOG:PGT:PID:QSS:REF_F1R2:REF_F2R1	0/1:71,2:0.029:0:2:..:0|1:45751093_C_CT:1903,18:44:27	0/0:33,0:0.00:0:0:.:0|1:45751093_C_CT:895,0:23:10	0.9	Deleterious	-11.88	Deleterious	148	30	NA	NA	NA	NA
T12,T32	chr9	**2**	**25**	45751103	45751103	G	T	exonic	**Dscaml1**	ENSMUSP00000081864	.	nonsynonymous SNV	Dscaml1:NM_001081270:exon31:c.G5366T:p.R1789M	**R1789M**	ENSMUSP00000081864,1789,R,M	9,45751103,G,A	9qA5.2	.	0.25	.	33	chr9	45751103	.	G	A	.	clustered_events;homologous_mapping_event;t_lod_fstar	ECNT=8;HCNT=1;MAX_ED=23;MIN_ED=2;NLOD=10.54;TLOD=5.01	GT:AD:AF:ALT_F1R2:ALT_F2R1:FOXOG:PGT:PID:QSS:REF_F1R2:REF_F2R1	0/1:74,2:0.027:0:2:..:0|1:45751093_C_CT:1926,55:46:28	0/0:36,0:0.00:0:0:.:0|1:45751093_C_CT:971,0:22:14	0.9	Tolerated	-1.55	Neutral	205	30	0.111	Tolerated	3.37	91
T12,T32	chr9	**2**	**25**	45751104	45751104	G	T	exonic	**Dscaml1**	ENSMUSP00000081864	.	nonsynonymous SNV	Dscaml1:NM_001081270:exon31:c.G5367T:p.R1789S	**R1789S**	ENSMUSP00000081864,1789,R,S	9,45751104,G,A	9qA5.2	.	0.25	.	33	chr9	45751104	.	G	A	.	clustered_events;homologous_mapping_event;t_lod_fstar	ECNT=8;HCNT=1;MAX_ED=23;MIN_ED=2;NLOD=10.23;TLOD=5.06	GT:AD:AF:ALT_F1R2:ALT_F2R1:FOXOG:PGT:PID:QSS:REF_F1R2:REF_F2R1	0/1:68,2:0.029:0:2:..:0|1:45751093_C_CT:1866,18:41:27	0/0:37,0:0.00:0:0:.:0|1:45751093_C_CT:1024,0:23:14	0.9	Tolerated	0	Neutral	205	30	1	Tolerated	3.37	91
T12,T32	chr9	**2**	**25**	45751093	45751093	-	T	exonic	**Dscaml1**	ENSMUSP00000081864	.	frameshift insertion	Dscaml1:NM_001081270:exon31:c.5356_5357insT:p.P1786fs	**P1786fs**	ENSMUSP00000081864,1786,P,	9,45751093,C,CGCCGTAT	9qA5.2	.	0.25	.	33	chr9	45751093	.	C	CGCCGTAT	.	clustered_events;homologous_mapping_event;t_lod_fstar	ECNT=8;HCNT=1;MAX_ED=23;MIN_ED=2;NLOD=9.03;TLOD=5.06	GT:AD:AF:ALT_F1R2:ALT_F2R1:FOXOG:PGT:PID:QSS:REF_F1R2:REF_F2R1	0/1:72,2:0.029:0:2:..:0|1:45751093_C_CT:1937,22:45:27	0/0:31,0:0.00:0:0:.:0|1:45751093_C_CT:843,0:21:10	0.9	Unknown	NA	NA	205	30	NA	NA	NA	NA
T12,T32	chr9	**2**	**25**	45751100	45751100	-	GCCGTAT	exonic	**Dscaml1**	ENSMUSP00000081864	.	frameshift insertion	Dscaml1:NM_001081270:exon31:c.5363_5364insGCCGTAT:p.S1788fs	**S1788fs**	ENSMUSP00000081864,1788,S,	9,45751100,C,GA	9qA5.2	.	0.25	.	31	chr9	45751100	.	C	GA	.	t_lod_fstar	ECNT=1;HCNT=2;MAX_ED=.;MIN_ED=.;NLOD=9.04;TLOD=4.44	GT:AD:AF:ALT_F1R2:ALT_F2R1:FOXOG:QSS:REF_F1R2:REF_F2R1	0/1:39,2:0.050:0:2:..:1137,58:15:24	0/0:37,0:0.00:0:0:.:1079,0:20:17	0.3	Unknown	NA	NA	205	30	NA	NA	NA	NA
T42, T4	chr18	**2**	**25**	49755508	49755508	C	T	exonic	**Dtwd2**	ENSMUSP00000110229	.	nonsynonymous SNV	Dtwd2:NM_001170960:exon1:c.G31A:p.G11R,Dtwd2:NM_026854:exon1:c.G31A:p.G11R	**G11R**	ENSMUSP00000110229,11,G,R	18,49755508,C,A	18qC	.	0.25	.	60	chr18	49755508	.	C	A	.	alt_allele_in_normal;clustered_events	ECNT=7;HCNT=2;MAX_ED=33;MIN_ED=10;NLOD=8.16;TLOD=29.35	GT:AD:AF:ALT_F1R2:ALT_F2R1:FOXOG:PGT:PID:QSS:REF_F1R2:REF_F2R1	0/1:56,10:0.145:7:3:0.700:0|1:49755498_G_A:1454,290:22:34	0/0:59,13:0.191:9:4:0.692:0|1:49755498_G_A:1484,365:35:24	0.7	Damaging	-0.99	Neutral	80	30	0.027	Damaging	4.2	20
T42, T4	chr18	**2**	**25**	49755511	49755511	-	CTTTCA	exonic	**Dtwd2**	ENSMUSP00000110229	.	stopgain	Dtwd2:NM_001170960:exon1:c.27_28insTGAAAG:p.L10delinsX,Dtwd2:NM_026854:exon1:c.27_28insTGAAAG:p.L10delinsX	**L10delinsX**	ENSMUSP00000110229,10,L,X	NA	18qC	.	0.25	.	60	chr18	49755511	.	G	A	.	alt_allele_in_normal;t_lod_fstar	ECNT=1;HCNT=5;MAX_ED=.;MIN_ED=.;NLOD=7.89;TLOD=4.59	GT:AD:AF:ALT_F1R2:ALT_F2R1:FOXOG:QSS:REF_F1R2:REF_F2R1	0/1:36,3:0.079:1:2:0.333:1029,77:15:21	0/0:53,2:0.043:1:1:0.500:1543,50:23:30	2.9	Deletarious	.	.	.	.	.	.	.	.
T42, T4	chr18	**2**	**25**	49755520	49755520	C	A	exonic	**Dtwd2**	ENSMUSP00000110229	.	stopgain	Dtwd2:NM_001170960:exon1:c.G19T:p.E7X,Dtwd2:NM_026854:exon1:c.G19T:p.E7X	**E7X**	ENSMUSP00000110229,7,E,X	NA	18qC	.	0.25	.	62	chr18	49755520	.	C	T	.	str_contraction;t_lod_fstar	ECNT=1;HCNT=2;MAX_ED=.;MIN_ED=.;NLOD=6.91;RPA=7,6;RU=ACC;STR;TLOD=5.54	GT:AD:AF:ALT_F1R2:ALT_F2R1:FOXOG:QSS:REF_F1R2:REF_F2R1	0/1:24,2:0.077:0:2:..:643,57:12:12	0/0:24,0:0.00:0:0:.:672,0:12:12	0.7	Deletarious	.	.	.	.	.	.	.	.
T42, T4	chr18	**2**	**25**	49755498	49755498	G	A	exonic	**Dtwd2**	ENSMUSP00000110229	.	nonsynonymous SNV	Dtwd2:NM_001170960:exon1:c.C41T:p.A14V,Dtwd2:NM_026854:exon1:c.C41T:p.A14V	**A14V**	ENSMUSP00000110229,14,A,V	NA	18qC	.	0.25	.	61	chr18	49755498	.	G	T	.	alt_allele_in_normal;clustered_events	ECNT=7;HCNT=2;MAX_ED=33;MIN_ED=10;NLOD=3.66;TLOD=36.83	GT:AD:AF:ALT_F1R2:ALT_F2R1:FOXOG:PGT:PID:QSS:REF_F1R2:REF_F2R1	0/1:54,12:0.175:7:5:0.417:0|1:49755498_G_A:1358,343:23:31	0/0:54,7:0.129:4:3:0.429:0|1:49755498_G_A:1273,194:33:21	0.7	Deleterious	-2.99	Deleterious	146	30	0.058	Tolerated	3.36	215
T42, T4	chr18	**2**	**25**	49755516	49755516	C	T	exonic	**Dtwd2**	ENSMUSP00000110229	.	nonsynonymous SNV	Dtwd2:NM_001170960:exon1:c.G23A:p.R8Q,Dtwd2:NM_026854:exon1:c.G23A:p.R8Q	**R8Q**	ENSMUSP00000110229,8,R,Q	18,49755516,C,GCTTTCA	18qC	.	0.25	.	61	chr18	49755516	.	C	GCTTTCA	.	alt_allele_in_normal;clustered_events	ECNT=7;HCNT=2;MAX_ED=33;MIN_ED=10;NLOD=2.45;TLOD=36.67	GT:AD:AF:ALT_F1R2:ALT_F2R1:FOXOG:PGT:PID:QSS:REF_F1R2:REF_F2R1	0/1:48,12:0.169:7:5:..:0|1:49755498_G_A:1392,349:21:27	0/0:53,7:0.138:3:4:..:0|1:49755498_G_A:1494,195:33:20	0.7	Neutral	-2.41	Neutral	80	30	NA	NA	NA	NA

T42, T4	chr18	**2**	**25**	49755531	49755531	G	A	exonic	**Dtwd2**	ENSMUSP00000011029	.	nonsynonymous SNV	Dtwd2:NM_001170960:exon1:c.C8T:p.P3L,Dtwd2:NM_026854:exon1:c.C8T:p.P3L	**P3L**	ENSMUSP00000011029,3,P,L	18,49755531,G,A	18qC	.	0.25	.	72	chr18	49755531	.	G	A	.	alt_allele_in_normal;clustered_events	ECNT=7;HCNT=2;MAX_ED=33;MIN_ED=10;NLOD=7.86;TLOD=33.18	GT:AD:AF:ALT_F1R2:ALT_F2R1:FOXOG:PGT:PID:QSS:REF_F1R2:REF_F2R1	0/1:53,11:0.164:7:4:0.364:0|1:49755498_G_A:1502,295:22:31	0/0:55,7:0.115:4:3:0.429:0|1:49755498_G_A:1500,189:34:21	0.7	Tolerated	-1.02	Neutral	80	30	0.126	Tolerated	4.32	18
T22,T4	chr9	**2**	**25**	78480072	78480072	C	T	exonic	**Eef1a1**	ENSMUSP00000034592	.	nonsynonymous SNV	Eef1a1:NM_010106:exon4:c.G401A:p.R134H	**R134H**	ENSMUSP00000034592,134,R,H	9,78480072,C,A	9qE1	.	0.25	.	43	chr9	78480072	.	C	A	.	clustered_events;multi_event_alt_allele_in_normal	ECNT=4;HCNT=2;MAX_ED=76;MIN_ED=38;NLOD=5.66;TLOD=13.31	GT:AD:AF:ALT_F1R2:ALT_F2R1:FOXOG:PGT:PID:QSS:REF_F1R2:REF_F2R1	0/1:20,4:0.167:2:2:.:0|1:78480110_ACCAACACCAG_A:556,112:11:9	0/0:35,1:0.029:0:1:.:0|1:78480110_ACCAACACCAG_A:978,29:18:17	1.2	Deleterious	-6.05	Deleterious	15414	30	0	Damaging	4.05	366
T22,T4	chr9	**2**	**25**	78480148	78480148	C	A	exonic	**Eef1a1**	ENSMUSP00000034592	.	nonsynonymous SNV	Eef1a1:NM_010106:exon4:c.G325T:p.A109S	**A109S**	ENSMUSP00000034592,109,A,S	9,78480148,C,G	9qE1	.	0.25	.	30	chr9	78480148	.	C	G	.	PASS	ECNT=1;HCNT=1;MAX_ED=.;MIN_ED=.;NLOD=12.59;TLOD=6.94	GT:AD:AF:ALT_F1R2:ALT_F2R1:FOXOG:QSS:REF_F1R2:REF_F2R1	0/1:47,4:0.083:0:4:1.00:1331,110:24:23	0/0:46,0:0.00:0:0:.:1292,0:25:21	19	Deleterious	-4.31	Deleterious	15414	30	0	Damaging	4.05	366
T22,T4	chr9	**2**	**25**	78480111	78480120	CCAACACCAG	-	exonic	**Eef1a1**	ENSMUSP00000034592	.	frameshift deletion	Eef1a1:NM_010106:exon4:c.353_362del:p.A118fs	**A118fs**	ENSMUSP00000034592,118,A,.	9,78480111,ACCAACACCAG,G	9qE1	.	0.25	.	36	chr9	78480110	.	ACCAACACCAG	G	.	PASS	ECNT=1;HCNT=1;MAX_ED=.;MIN_ED=.;NLOD=12.59;TLOD=6.94	GT:AD:AF:ALT_F1R2:ALT_F2R1:FOXOG:QSS:REF_F1R2:REF_F2R1	0/1:47,4:0.083:0:4:1.00:1331,110:24:23	0/0:46,0:0.00:0:0:.:1292,0:25:21	19	Unknown	NA	NA	15414	30	NA	NA	NA	NA
T32,T3	chr10	**2**	**25**	81178824	81178824	G	A	exonic	**Eef2**	ENSMUSP00000132786	.	nonsynonymous SNV	Eef2:NM_007907:exon4:c.G544A:p.V182M	**V182M**	ENSMUSP00000132786,182,V,M	10,81178824,G,C	10qC1	.	0.25	.	274	chr10	81178824	.	G	C	.	alt_allele_in_normal;clustered_events;t_lod_fstar	ECNT=4;HCNT=4;MAX_ED=108;MIN_ED=6;NLOD=2.75;TLOD=5.39	GT:AD:AF:ALT_F1R2:ALT_F2R1:FOXOG:PGT:PID:QSS:REF_F1R2:REF_F2R1	0/1:46,2:0.042:1:1:0.500:0|1:113844345_G_A:1312,43:22:24	0/0:39,2:0.051:2:0:0.00:0|1:113844345_G_A:1082,46:21:18	0.4	Damaging	-1.23	Neutral	706	30	0.01	Damaging	3.61	381
T1,T3	chr10	**2**	**25**	81178158	81178158	T	G	exonic	**Eef2**	ENSMUSP00000132786	.	nonsynonymous SNV	Eef2:NM_007907:exon3:c.T324G:p.H108Q	**H108Q**	ENSMUSP00000132786,108,H,Q	10,81178158,T,T	10qC1	.	0.25	.	226	chr10	81178158	.	T	T	.	multi_event_alt_allele_in_normal	ECNT=2;HCNT=9;MAX_ED=2;MIN_ED=2;NLOD=47.75;TLOD=9.75	GT:AD:AF:ALT_F1R2:ALT_F2R1:FOXOG:QSS:REF_F1R2:REF_F2R1	0/1:213,8:0.036:3:5:0.625:5995,225:114:99	0/0:183,1:5.814e-03:1:0:0.00:5009,29:87:96	0.7	Tolerated	0	Neutral	706	30	1	Tolerated	3.61	380
T12,T1	chr15	**2**	**25**	76103603	76103603	T	C	exonic	**Eppk1**	ENSMUSP00000103703	.	nonsynonymous SNV	Eppk1:NM_144848:exon2:c.A9077G:p.E3026G	**E3026G**	ENSMUSP00000103703,3026,E,G	15,76103603,T,T	15qD3	rs108382437	0.25	.	196	chr15	76103603	.	T	T	.	alt_allele_in_normal;clustered_events	ECNT=7;HCNT=2;MAX_ED=33;MIN_ED=10;NLOD=3.06;TLOD=37.18	GT:AD:AF:ALT_F1R2:ALT_F2R1:FOXOG:PGT:PID:QSS:REF_F1R2:REF_F2R1	0/1:47,12:0.186:7:5:0.417:0|1:49755498_G_A:1185,349:20:27	0/0:53,7:0.119:3:4:0.571:0|1:49755498_G_A:1266,198:33:20	0.7	Unkown	.	.	.	.	.	.	.	.
T12,T3	chr8	**2**	**25**	35478562	35478562	G	A	exonic	**Eri1**	ENSMUSP00000016670	.	stopgain	Eri1:NM_026067:exon4:c.C568T:p.Q190X	**Q190X**	ENSMUSP00000016670,190,Q,X	NA	8qA4	.	0.25	.	55	chr8	35478562	.	G	C	.	alt_allele_in_normal;clustered_events;germline_risk;t_lod_fstar	DB;ECNT=3;HCNT=1;MAX_ED=5;MIN_ED=1;NLOD=1.82;TLOD=6.09	GT:AD:AF:ALT_F1R2:ALT_F2R1:FOXOG:PGT:PID:QSS:REF_F1R2:REF_F2R1	0/1:18,2:0.100:2:0:0.00:0|1:12395642_A_C:512,17:12:6	0/0:18,1:0.048:1:0:0.00:0|1:12395642_A_C:485,28:7:11	0.9	Deletarious	.	.	.	.	.	.	.	.
T12,T32	chr6	**2**	**25**	97014438	97014438	T	C	exonic	**Fam19a4**	ENSMUSP00000120205	.	nonsynonymous SNV	Fam19a4:NM_177233:exon3:c.A49G:p.T17A	**T17A**	ENSMUSP00000120205,17,T,A	6,97014438,T,T	6qD3	rs30957756	0.25	.	55	chr6	97014438	rs30957756	T	T	.	t_lod_fstar	ECNT=1;HCNT=1;MAX_ED=.;MIN_ED=.;NLOD=12.33;TLOD=5.25	GT:AD:AF:ALT_F1R2:ALT_F2R1:FOXOG:QSS:REF_F1R2:REF_F2R1	0/1:46,3:0.067:3:0:0.00:1317,86:24:22	0/0:46,0:0.00:0:0:.:1276,0:20:26	1.3	Tolerated	0	Neutral	130	30	0.476	Tolerated	3.16	96
T22, T2	chr2	**2**	**25**	64916827	64916827	A	G	splicing	**Grb14**	ENSMUSP00000080093	.	.	.	**NA**	2,64916827,A,T	NA	2qC1.3	.	0.25	.	32	chr2	64916827	.	A	T	.	t_lod_fstar	ECNT=1;HCNT=1;MAX_ED=.;MIN_ED=.;NLOD=12.63;TLOD=4.04	GT:AD:AF:ALT_F1R2:ALT_F2R1:FOXOG:QSS:REF_F1R2:REF_F2R1	0/1:16,2:0.111:0:2:1.00:427,58:9:7	0/0:48,0:0.00:0:0:.:1328,0:24:24	0.6	Unkown	.	.	.	.	.	.	.	.
T12,T3	chr13	**2**	**25**	51202988	51202988	C	T	ncRNA_exonic	**Hist1h2al**	NONE	.	.	.	**NA**	13,51202988,C,G	NA	13qA5	rs13471089	0.25	.	32	chr13	51202988	.	C	G	.	t_lod_fstar	ECNT=1;HCNT=1;MAX_ED=.;MIN_ED=.;NLOD=12.04;TLOD=5.23	GT:AD:AF:ALT_F1R2:ALT_F2R1:FOXOG:QSS:REF_F1R2:REF_F2R1	0/1:43,3:0.071:0:3:.:1203,81:17:26	0/0:45,0:0.00:0:0:.:1228,0:21:24	1.2	Tolerated	0	Neutral	894	30	1	Tolerated	4.13	392

T22,T42	chr1	**2**	**25**	171531185	171531185	C	A	exonic	**Itln1**	ENSMUSP00000120115	.	nonsynonymous SNV	Itln1:NM_010584:exon5:c.G413T:p.G138V	**G138V**	ENSMUSP00000120115,138,G,V	1,171531185,C,T	1qH3	.	0.25	.	114	chr1	171531185	.	C	T	.	alt_allele_in_normal;clustered_events	ECNT=8;HCNT=1;MAX_ED=146;MIN_ED=15;NLOD=8.50;TLOD=7.75	GT:AD:AF:ALT_F1R2:ALT_F2R1:FOXOG:PGT:PID:QSS:REF_F1R2:REF_F2R1	0/1:88,3:0.032:1:2:0.667:0|1:171531181_G_T:2502,78:42:46	0/0:103,5:0.048:4:1:0.200:0|1:171531181_G_T:2913,140:48:55	20	Deleterious	-7.25	Deleterious	168	30	0	Damaging	2.87	264
T22,T42	chr1	**2**	**25**	171531189	171531189	G	A	exonic	**Itln1**	ENSMUSP00000116671	.	nonsynonymous SNV	Itln1:NM_010584:exon5:c.C409T:p.P137S	**P137S**	ENSMUSP00000116671,137,P,S	1,171531189,G,T	1qH3	.	0.25	.	114	chr1	171531189	.	G	T	.	alt_allele_in_normal;clustered_events	ECNT=8;HCNT=1;MAX_ED=146;MIN_ED=15;NLOD=9.84;TLOD=7.61	GT:AD:AF:ALT_F1R2:ALT_F2R1:FOXOG:PGT:PID:QSS:REF_F1R2:REF_F2R1	0/1:100,3:0.029:1:2:0.333:0|1:171531181_G_T:2789,90:46:54	0/0:113,5:0.045:4:1:0.800:0|1:171531181_G_T:3182,140:54:59	20	Deleterious	-7.89	Deleterious	144	30	0.026	Damaging	2.84	161
T12, T22	chr1	**2**	**25**	171530503	171530503	-	G	exonic	**Itln1**	ENSMUSP00000116671	.	frameshift insertion	Itln1:NM_010584:exon6:c.675dupC:p.S226fs	**S226fs**	ENSMUSP00000116671,226,S,	1,171530503,A,T	1qH3	.	0.25	.	158	chr1	171530503	.	A	T	.	alt_allele_in_normal;clustered_events	ECNT=2;HCNT=3;MAX_ED=15;MIN_ED=15;NLOD=11.22;TLOD=10.10	GT:AD:AF:ALT_F1R2:ALT_F2R1:FOXOG:PGT:PID:QSS:REF_F1R2:REF_F2R1	0/1:139,4:0.028:2:2:0.500:0|1:171531090_G_A:3972,111:67:72	0/0:152,10:0.056:4:6:0.600:0|1:171531090_G_A:4193,261:83:69	20	Tolerated	-0.88	Neutral	144	30	0.345	Tolerated	2.91	153
T32, T42	chr7	**2**	**25**	3908702	3908702	G	C	exonic	**Lilra6**	ENSMUSP00000082016	.	nonsynonymous SNV	Lilra6:NM_011090:exon9:c.C1976G:p.P659R	**P659R**	ENSMUSP00000082016,659,P,R	7,3908702,G,C	7qA1	.	0.25	.	36	chr7	3908702	.	G	C	.	alt_allele_in_normal;clustered_events;germline_risk	DB;ECNT=4;HCNT=12;MAX_ED=31;MIN_ED=10;NLOD=2.39;TLOD=15.51	GT:AD:AF:ALT_F1R2:ALT_F2R1:FOXOG:PGT:PID:QSS:REF_F1R2:REF_F2R1	0/1:25,5:0.167:1:4:0.200:0|1:3908692_T_C:716,145:10:15	0/0:40,3:0.077:1:2:0.333:0|1:3908692_T_C:1105,71:21:19	1.9	Tolerated	-0.06	Neutral	179	30	0.115	Tolerated	3.15	81
T32, T42	chr7	**2**	**25**	3908703	3908703	G	A	exonic	**Lilra6**	ENSMUSP00000082016	.	nonsynonymous SNV	Lilra6:NM_011090:exon9:c.C1975T:p.P659S	**P659S**	ENSMUSP00000082016,659,P,S	7,3908703,G,A	7qA1	.	0.25	.	36	chr7	3908703	.	G	A	.	t_lod_fstar	ECNT=1;HCNT=1;MAX_ED=.;MIN_ED=.;NLOD=9.88;TLOD=4.19	GT:AD:AF:ALT_F1R2:ALT_F2R1:FOXOG:QSS:REF_F1R2:REF_F2R1	0/1:80,3:0.038:0:3:0.00:2310,83:42:38	0/0:35,0:0.00:0:0:.:984,0:18:17	1.5	Tolerated	-0.54	Neutral	179	30	0.392	Tolerated	3.15	81
T32, T42	chr7	**2**	**25**	3908723	3908723	T	C	exonic	**Lilra6**	ENSMUSP00000082016	.	nonsynonymous SNV	Lilra6:NM_011090:exon9:c.A1955G:p.Q652R	**Q652R**	ENSMUSP00000082016,652,Q,R	7,3908723,T,T	7qA1	rs49820170	0.25	.	43	chr7	3908723	rs49820170	T	T	.	t_lod_fstar	ECNT=1;HCNT=1;MAX_ED=.;MIN_ED=.;NLOD=10.78;TLOD=4.13	GT:AD:AF:ALT_F1R2:ALT_F2R1:FOXOG:QSS:REF_F1R2:REF_F2R1	0/1:75,3:0.042:2:1:0.667:2111,81:26:49	0/0:45,0:0.00:0:0:.:1204,0:19:26	0.7	Tolerated	0	Neutral	179	30	0.501	Tolerated	3.04	104
T22, T2	chr15	**2**	**25**	91709358	91709358	C	G	exonic	**Lrrk2**	ENSMUSP00000110394	.	nonsynonymous SNV	Lrrk2:NM_025730:exon13:c.C1519G:p.L507V	**L507V**	ENSMUSP00000110394,507,L,V	15,91709358,C,A	15qE3	.	0.25	.	38	chr15	91709358	.	C	A	.	clustered_events;homologous_mapping_event	ECNT=3;HCNT=2;MAX_ED=3;MIN_ED=1;NLOD=7.52;TLOD=8.46	GT:AD:AF:ALT_F1R2:ALT_F2R1:FOXOG:PGT:PID:QSS:REF_F1R2:REF_F2R1	0/1:52,1:0.056:0:1:1.00:0|1:98003232_G_T:1311,26:27:25	0/0:27,0:0.00:0:0:.:0|1:98003232_G_T:633,0:14:13	9	Damaging	-0.7	Neutral	110	30	0.008	Damaging	3.26	48
T12, T32	chrX	**2**	**25**	157576100	157576100	C	T	exonic	**Mbtps2**	ENSMUSP00000025486	.	nonsynonymous SNV	Mbtps2:NM_172307:exon4:c.G514A:p.G172S	**G172S**	ENSMUSP00000025486,172,G,S	X,157576100,C,G	XqF4	.	0.25	.	41	chrX	157576100	.	C	G	.	alt_allele_in_normal;t_lod_fstar	ECNT=1;HCNT=1;MAX_ED=.;MIN_ED=.;NLOD=9.96;TLOD=5.86	GT:AD:AF:ALT_F1R2:ALT_F2R1:FOXOG:QSS:REF_F1R2:REF_F2R1	0/1:83,6:0.114:3:3:.:2357,164:37:46	0/0:64,2:0.032:1:1:.:1759,58:33:31	1.6	Damaging	-2.31	Neutral	84	30	0.004	Damaging	2.85	43
T32,T42	chr3	**2**	**25**	32734260	32734260	-	CCGAGTGAGCAGCGTCCT	splicing	**Mrpl47**	ENSMUSP00000025178	NM_029017:exon2:c.99-1->AGGACGCTGCTCACTCGG	.	.	**NA**	3,32734260,C,C	NA	3qA3	.	0.25	.	47	chr3	32734260	.	C	C	.	str_contraction;t_lod_fstar	ECNT=1;HCNT=1;MAX_ED=.;MIN_ED=.;NLOD=6.02;RPA=7,6;RU=CTG;STR;TLOD=4.73	GT:AD:AF:ALT_F1R2:ALT_F2R1:FOXOG:QSS:REF_F1R2:REF_F2R1	0/1:54,2:0.038:0:2:.:1525,56:29:25	0/0:24,0:0.00:0:0:.:672,0:13:11	2.8	Unkown	.	.	.	.	.	.	.	.
T12, T32	chr2	**2**	**25**	28469580	28469580	C	T	exonic	**Mrps2**	ENSMUSP00000133926	.	nonsynonymous SNV	Mrps2:NM_001166031:exon4:c.C391T:p.H131Y,Mrps2:NM_080452:exon4:c.C448T:p.H150Y	**H150Y**	ENSMUSP00000133926,150,H,Y	2,28469580,C,T	2qA3	rs13476400	0.25	.	28	chr2	28469580	rs13476400	C	T	.	t_lod_fstar	ECNT=1;HCNT=1;MAX_ED=.;MIN_ED=.;NLOD=12.94;TLOD=5.70	GT:AD:AF:ALT_F1R2:ALT_F2R1:FOXOG:QSS:REF_F1R2:REF_F2R1	0/1:33,2:0.059:2:0:.:940,41:21:12	0/0:50,0:0.00:0:0:.:1401,0:24:26	1.3	Deleterious	-4.03	Deleterious	90	30	0.314	Tolerated	2.81	92

T12, T32	chr2	**2**	**25**	28469580	28469580	C	T	exonic	**Mrps2**	ENSMUSP00000133926	.	nonsynonymous SNV	Mrps2:NM_001166031:exon4:c.C391T:p.H131Y,Mrps2:NM_080452:exon4:c.C448T:p.H150Y	**H131Y**	ENSMUSP00000133926,131,H,Y	2,28469580,C,C	2qA3	rs13476400	0.25	.	28	chr2	28469580	rs13476400	C	C	.	str_contraction;t_lod_fstar	ECNT=1;HCNT=1;MAX_ED=.;MIN_ED=.;NLOD=6.61;RPA=7,6;RU=GCT;STR;TLOD=4.81	GT:AD:AF:ALT_F1R2:ALT_F2R1:FOXOG:QSS:REF_F1R2:REF_F2R1	0/1:53,2:0.037:1:1:.:1347,45:26:27	0/0:27,0:0.00:0:0:.:690,0:11:16	2.4	Tolerated	0	Neutral	90	30	1	Tolerated	2.81	92
T1,T3	chr16	**2**	**25**	32753786	32753786	G	T	exonic	**Muc4**	ENSMUSP00000059471	.	nonsynonymous SNV	Muc4:NM_080457:exon3:c.G3661T:p.A1221S	**A1221S**	ENSMUSP00000059471,1221,A,S	16,32753786,G,C	16qB3	.	0.25	.	90	chr16	32753786	.	G	C	.	alt_allele_in_normal;clustered_events	ECNT=3;HCNT=10;MAX_ED=6;MIN_ED=5;NLOD=4.55;TLOD=36.28	GT:AD:AF:ALT_F1R2:ALT_F2R1:FOXOG:PGT:PID:QSS:REF_F1R2:REF_F2R1	0/1:62,11:0.157:4:7:0.636:0|1:32753575_T_C:1722,306:32:30	0/0:47,2:0.044:1:1:0.500:0|1:32753575_T_C:1237,54:27:20	4	Damaging	-0.14	Neutral	56	30	0.017	Damaging	3.53	10
T2, T42	chr16	**2**	**25**	32754674	32754674	C	T	exonic	**Muc4**	ENSMUSP00000059471	.	nonsynonymous SNV	Muc4:NM_080457:exon4:c.C4547T:p.P1516L	**P1516L**	ENSMUSP00000059471,1516,P,L	16,32754674,C,A	16qB3	.	0.25	.	125	chr16	32754674	.	C	A	.	clustered_events;multi_event_alt_allele_in_normal	ECNT=10;HCNT=3;MAX_ED=64;MIN_ED=12;NLOD=29.82;TLOD=7.07	GT:AD:AF:ALT_F1R2:ALT_F2R1:FOXOG:PGT:PID:QSS:REF_F1R2:REF_F2R1	0/1:153,3:0.019:0:3:0.00:0|1:32754674_C_T:4482,84:83:70	0/0:118,1:8.772e-03:1:0:1.00:0|1:32754674_C_T:3344,28:68:50	4	Damaging	-1.06	Neutral	56	30	0.005	Damaging	3.55	11
T12,T2	chr16	**2**	**25**	32754917	32754917	G	A	exonic	**Muc4**	ENSMUSP00000059471	.	nonsynonymous SNV	Muc4:NM_080457:exon4:c.G4790A:p.S1597N	**S1597N**	ENSMUSP00000059471,1597,S,N	16,32754917,G,T	16qB3	.	0.25	.	140	chr16	32754917	.	G	T	.	alt_allele_in_normal;clustered_events	ECNT=4;HCNT=4;MAX_ED=58;MIN_ED=18;NLOD=4.02;TLOD=19.74	GT:AD:AF:ALT_F1R2:ALT_F2R1:FOXOG:QSS:REF_F1R2:REF_F2R1	0/1:121,7:0.055:4:3:0.429:3402,188:69:52	0/0:117,7:0.058:5:2:0.286:3261,178:62:55	4	Damaging	-1.23	Neutral	56	30	0.005	Damaging	3.61	7
T1,T3	chr16	**2**	**25**	32753802	32753802	T	C	exonic	**Muc4**	ENSMUSP00000059471	.	nonsynonymous SNV	Muc4:NM_080457:exon3:c.T3677C:p.I1226T	**I1226T**	ENSMUSP00000059471,1226,I,T	16,32753802,T,C	16qB3	.	0.25	.	77	chr16	32753802	.	T	C	.	clustered_events;multi_event_alt_allele_in_normal	ECNT=3;HCNT=15;MAX_ED=80;MIN_ED=72;NLOD=6.94;TLOD=11.84	GT:AD:AF:ALT_F1R2:ALT_F2R1:FOXOG:PGT:PID:QSS:REF_F1R2:REF_F2R1	0/1:47,4:0.075:4:0:1.00:0|1:32752657_G_C:1325,108:24:23	0/0:40,1:0.026:1:0:1.00:0|1:32752657_G_C:1104,28:16:24	4	Tolerated	1.34	Neutral	56	30	1	Tolerated	3.54	10
T2, T42	chr16	**2**	**25**	32754690	32754690	G	T	exonic	**Muc4**	ENSMUSP00000059471	.	nonsynonymous SNV	Muc4:NM_080457:exon4:c.G4563T:p.M1521I	**M1521I**	ENSMUSP00000059471,1521,M,I	16,32754690,G,G	16qB3	.	0.25	.	122	chr16	32754690	.	G	G	.	clustered_events;homologous_mapping_event	ECNT=10;HCNT=3;MAX_ED=64;MIN_ED=12;NLOD=35.82;TLOD=7.07	GT:AD:AF:ALT_F1R2:ALT_F2R1:FOXOG:PGT:PID:QSS:REF_F1R2:REF_F2R1	0/1:152,3:0.019:0:3:1.00:0|1:32754674_C_T:4398,84:82:70	0/0:125,0:0.00:0:0:.:0|1:32754674_C_T:3565,0:69:56	4	Tolerated	0	Neutral	56	30	0.491	Tolerated	3.55	11
T2, T42	chr16	**2**	**25**	32754703	32754703	G	A	exonic	**Muc4**	ENSMUSP00000059471	.	nonsynonymous SNV	Muc4:NM_080457:exon4:c.G4576A:p.A1526T	**A1526T**	ENSMUSP00000059471,1526,A,T	16,32754703,G,A	16qB3	.	0.25	.	119	chr16	32754703	.	G	A	.	clustered_events;homologous_mapping_event	ECNT=10;HCNT=3;MAX_ED=64;MIN_ED=12;NLOD=36.12;TLOD=7.06	GT:AD:AF:ALT_F1R2:ALT_F2R1:FOXOG:PGT:PID:QSS:REF_F1R2:REF_F2R1	0/1:156,3:0.019:0:3:0.00:0|1:32754674_C_T:4349,81:84:72	0/0:139,0:0.00:0:0:.:0|1:32754674_C_T:3769,0:79:60	4	Tolerated	0.51	Neutral	56	30	1	Tolerated	3.55	11
T2, T42	chr16	**2**	**25**	32754711	32754711	C	G	exonic	**Muc4**	ENSMUSP00000059471	.	nonsynonymous SNV	Muc4:NM_080457:exon4:c.C4584G:p.I1528M	**I1528M**	ENSMUSP00000059471,1528,I,M	16,32754711,C,T	16qB3	.	0.25	.	125	chr16	32754711	.	C	T	.	alt_allele_in_normal;t_lod_fstar	ECNT=1;HCNT=8;MAX_ED=.;MIN_ED=.;NLOD=12.67;TLOD=4.86	GT:AD:AF:ALT_F1R2:ALT_F2R1:FOXOG:QSS:REF_F1R2:REF_F2R1	0/1:90,1:0.023:1:0:0.00:2582,28:47:43	0/0:113,4:0.040:1:3:0.750:3151,83:52:61	4	Tolerated	0	Neutral	56	30	1	Tolerated	3.55	11
T2, T42	chr16	**2**	**25**	32754725	32754725	T	A	exonic	**Muc4**	ENSMUSP00000059471	.	nonsynonymous SNV	Muc4:NM_080457:exon4:c.T4598A:p.I1533N	**I1533N**	ENSMUSP00000059471,1533,I,N	16,32754725,T,T	16qB3	.	0.25	.	139	chr16	32754725	.	T	T	.	alt_allele_in_normal;clustered_events	ECNT=3;HCNT=6;MAX_ED=136;MIN_ED=133;NLOD=7.76;TLOD=28.85	GT:AD:AF:ALT_F1R2:ALT_F2R1:FOXOG:QSS:REF_F1R2:REF_F2R1	0/1:87,14:0.141:8:6:0.429:2481,397:42:45	0/0:109,7:0.067:3:4:0.571:3068,195:65:44	4	Tolerated	0	Neutral	56	30	0.119	Tolerated	3.55	10
T1,T22	chr14	**2**	**25**	54944750	54944753	ACTC	-	exonic	**Myh6**	ENSMUSP00000000505	.	frameshift deletion	Myh6:NM_001164171:exon35:c.5197_5200del:p.E1733fs,Myh6:NM_010856:exon35:c.5197_5200del:p.E1733fs	**E1733fs**	ENSMUSP00000000505,1733,E,.	14,54944750,GACTC,C	14qC2	.	0.25	.	51	chr14	54944749	.	GACTC	C	.	clustered_events;homologous_mapping_event	ECNT=3;HCNT=1;MAX_ED=5;MIN_ED=4;NLOD=17.46;TLOD=9.90	GT:AD:AF:ALT_F1R2:ALT_F2R1:FOXOG:PGT:PID:QSS:REF_F1R2:REF_F2R1	0/1:68,4:0.056:1:3:0.750:0|1:54960410_A_C:1910,113:34:34	0/0:63,0:0.00:0:0:.:0|1:54960410_A_C:1716,0:35:28	1.5	Unknown	NA	NA	529	30	NA	NA	NA	NA

T22,T42	chr5	**2**	**25**	134227491	134227492	CC	-	exonic	**Ncf1**	ENSMUSP00000072091	.	frameshift deletion	Ncf1:NM_001286037:exon3:c.187_188del:p.G63fs,Ncf1:NM_010876:exon3:c.187_188del:p.G63fs	**G63fs**	ENSMUSP00000072091,63,G,.	5,134227491,GCC,TC	5qG2	.	0.25	.	33	chr5	134227490	.	GCC	TC	.	t_lod_fstar	ECNT=1;HCNT=1;MAX_ED=.;MIN_ED=.;NLOD=13.84;RPA=1,2;RU=C;STR;TLOD=5.55	GT:AD:AF:ALT_F1R2:ALT_F2R1:FOXOG:QSS:REF_F1R2:REF_F2R1	0/1:55,3:0.054:3:0:.:1531,84:28:27	0/0:53,0:0.00:0:0:.:1496,0:23:30	0.7	Unknown	NA	NA	182	30	NA	NA	NA	NA
T22,T2	chrX	**2**	**25**	104087588	104087588	A	G	exonic	**Nexmif**	ENSMUSP00000048078	.	nonsynonymous SNV	Nexmif:NM_001077354:exon4:c.T722C:p.L241S	**L241S**	ENSMUSP00000048078,241,L,S	X,104087588,A,C	XqD	.	0.25	.	34	chrX	104087588	.	A	C	.	PASS	ECNT=1;HCNT=2;MAX_ED=.;MIN_ED=.;NLOD=10.39;TLOD=10.60	GT:AD:AF:ALT_F1R2:ALT_F2R1:FOXOG:QSS:REF_F1R2:REF_F2R1	0/1:29,5:0.152:3:2:0.600:790,143:14:15	0/0:40,0:0.00:0:0:.:1025,0:21:19	1.8	Unknown	NA	NA	86	15	NA	NA	NA	NA
T12,T32	chr2	**2**	**25**	26467702	26467702	C	T	exonic	**Notch1**	ENSMUSP00000119029	.	nonsynonymous SNV	Notch1:NM_008714:exon26:c.G4915A:p.A1639T	**A1639T**	ENSMUSP00000119029,1639,A,T	2,26467702,C,G	2qA3	rs27201809	0.25	.	39	chr2	26467702	rs27201809	C	G	.	clustered_events	DB;ECNT=2;HCNT=2;MAX_ED=52;MIN_ED=52;NLOD=11.34;TLOD=77.87	GT:AD:AF:ALT_F1R2:ALT_F2R1:FOXOG:QSS:REF_F1R2:REF_F2R1	0/1:30,33:0.517:14:19:0.576:818,854:16:14	0/0:42,0:0.00:0:0:.:1129,0:20:22	3	Tolerated	-0.67	Neutral	125	30	0.322	Tolerated	3.06	57
T12,T42	chr11	**2**	**25**	59090528	59090528	G	A	exonic	**Obscn**	ENSMUSP00000139045	.	nonsynonymous SNV	Obscn:NM_199152:exon18:c.C5663T:p.S1888F	**S1888F**	ENSMUSP00000139045,1888,S,F	11,59090528,G,G	11qB1.3	.	0.25	.	23	chr11	59090528	.	G	G	.	clustered_events;multi_event_alt_allele_in_normal;t_lod_fstar	ECNT=2;HCNT=10;MAX_ED=20;MIN_ED=20;NLOD=8.99;TLOD=5.07	GT:AD:AF:ALT_F1R2:ALT_F2R1:FOXOG:QSS:REF_F1R2:REF_F2R1	0/1:68,2:0.029:0:2:1.00:1951,52:39:29	0/0:48,1:0.022:0:1:1.00:1362,23:26:22	22	Deleterious	-2.63	Deleterious	198	30	0.235	Tolerated	2.97	243
T12,T42	chr11	**2**	**25**	59090747	59090747	T	C	exonic	**Obscn**	ENSMUSP00000016094	.	nonsynonymous SNV	Obscn:NM_199152:exon18:c.A5444G:p.H1815R	**H1815R**	ENSMUSP00000016094,1815,H,R	11,59090747,T,G	11qB1.3	.	0.25	.	26	chr11	59090747	.	T	G	.	clustered_events;multi_event_alt_allele_in_normal;t_lod_fstar	ECNT=2;HCNT=10;MAX_ED=20;MIN_ED=20;NLOD=8.99;TLOD=5.07	GT:AD:AF:ALT_F1R2:ALT_F2R1:FOXOG:QSS:REF_F1R2:REF_F2R1	0/1:68,2:0.029:0:2:1.00:1951,52:39:29	0/0:48,1:0.022:0:1:1.00:1362,23:26:22	22	Tolerated	-0.84	Neutral	66	30	0.239	Tolerated	2.88	24
T1,T32	chr11	**2**	**25**	59129573	59129573	C	T	exonic	**Obscn**	ENSMUSP00000016094	.	nonsynonymous SNV	Obscn:NM_199152:exon7:c.G2644A:p.A882T	**A882T**	ENSMUSP00000016094,882,A,T	11,59129573,C,A	11qB1.3	.	0.25	.	43	chr11	59129573	.	C	A	.	PASS	ECNT=1;HCNT=6;MAX_ED=.;MIN_ED=.;NLOD=6.92;TLOD=11.65	GT:AD:AF:ALT_F1R2:ALT_F2R1:FOXOG:QSS:REF_F1R2:REF_F2R1	0/1:49,6:0.118:3:3:0.500:1376,168:27:22	0/0:23,0:0.00:0:0:.:646,0:11:12	22	Tolerated	0.31	Neutral	66	30	0.9	Tolerated	2.85	17
T1,T22	chr4	**2**	**25**	123283714	123283714	-	T	exonic	**Pabpc4**	ENSMUSP00000020163	.	frameshift insertion	Pabpc4:NM_001356377:exon1:c.100_101insT:p.A34fs,Pabpc4:NM_130881:exon1:c.100_101insT:p.A34fs,Pabpc4:NM_148917:exon1:c.100_101insT:p.A34fs	**A34fs**	ENSMUSP00000020163,34,A,	4,123283714,G,G	4qD2.2	.	0.25	.	45	chr4	123283714	.	G	G	.	PASS	ECNT=1;HCNT=1;MAX_ED=.;MIN_ED=.;NLOD=11.08;TLOD=9.40	GT:AD:AF:ALT_F1R2:ALT_F2R1:FOXOG:QSS:REF_F1R2:REF_F2R1	0/1:50,6:0.111:1:5:0.833:1418,147:28:22	0/0:41,0:0.00:0:0:.:1124,0:25:16	0.9	Tolerated	0	Neutral	290	30	1	Tolerated	2.84	320
T1,T32	chr13	**2**	**25**	119455790	119455790	G	A	splicing	**Paip1**	ENSMUSP00000118130	.	NM_001079849:exon10:c.1010+1G>A;NM_145457:exon10:c.1109+1G>A	.	**NA**	13,119455790,G,CATATTTGGATGATATCGATGATGAGATAGACCCAGAAATTGAAGAAGCTTAT	NA	13qD2.3	.	0.25	.	36	chr13	119455790	.	G	CATATTTGGATGATATCGATGATGAGATAGACCCAGAAATTGAAGAAGCTTAT	.	t_lod_fstar	ECNT=2;HCNT=2;MAX_ED=2;MIN_ED=2;NLOD=9.28;TLOD=5.98	GT:AD:AF:ALT_F1R2:ALT_F2R1:FOXOG:PGT:PID:QSS:REF_F1R2:REF_F2R1	0/1:24,2:0.083:0:2:.:0|1:119455789_C_CATATTTGGATGATATCGATGATGAGATAGACCCAGAAATTGAAGAAGCTTAT:615,58:14:10	0/0:34,0:0.00:0:0:.:0|1:119455789_C_CATATTTGGATGATATCGATGATGAGATAGACCCAGAAATTGAAGAAGCTTAT:798,0:18:16	2.3	Unkown	.	.	.	.	.	.	.	.
T22,T4	chr1	**2**	**25**	93321393	93321393	-	T	exonic	**Pask**	ENSMUSP00000001561	.	frameshift insertion	Pask:NM_080850:exon10:c.2283_2284insA:p.L762fs	**L762fs**	ENSMUSP00000001561,762,L,	1,93321393,G,A	1qD	.	0.25	.	29	chr1	93321393	.	G	A	.	clustered_events;homologous_mapping_event	ECNT=7;HCNT=1;MAX_ED=22;MIN_ED=3;NLOD=9.33;TLOD=8.27	GT:AD:AF:ALT_F1R2:ALT_F2R1:FOXOG:PGT:PID:QSS:REF_F1R2:REF_F2R1	0/1:36,5:0.111:0:5:0.00:0|1:93321384_G_GGTGGTC:1022,74:23:13	0/0:30,0:0.00:0:0:.:0|1:93321384_G_GGTGGTC:839,0:13:17	0.3	Tolerated	0	Neutral	89	30	1	Tolerated	3.57	59
T12,T1	chr18	**2**	**25**	37449681	37449683	ACT	-	exonic	**Pcdhb14**	ENSMUSP00000126428	.	nonframeshift deletion	Pcdhb14:NM_053139:exon1:c.1839_1841del:p.613_614del	**613_614del**	NA	18,37449681,GACT,T	18qB3	.	0.25	.	76	chr18	37449680	.	GACT	T	.	alt_allele_in_normal;clustered_events	ECNT=4;HCNT=2;MAX_ED=13;MIN_ED=1;NLOD=3.37;TLOD=18.07	GT:AD:AF:ALT_F1R2:ALT_F2R1:FOXOG:PGT:PID:QSS:REF_F1R2:REF_F2R1	0/1:68,5:0.085:4:1:0.200:0|1:37449667_A_G:1875,138:39:29	0/0:73,3:0.056:0:3:1.00:0|1:37449667_A_G:1989,87:39:34	0.7	Unknown	.	.	.	.	.	.	.	.

T3,T4	chr18	**2**	**25**	37321408	37321408	G	C	exonic	**Pcdhb5**	ENSMUSP00000078589	.	nonsynonymous SNV	Pcdhb5:NM_053130:exon1:c.G840C:p.L280F	**L280F**	ENSMUSP00000078589,280,L,F	18,37321408,G,AGGGCACAGAAGATGCACT	18qB3	.	0.25	.	96	chr18	37321408	.	G	AGGGCACAGAAGATGCACT	.	alt_allele_in_normal;clustered_events;germline_risk	ECNT=2;HCNT=33;MAX_ED=3;MIN_ED=3;NLOD=0.740;TLOD=108.08	GT:AD:AF:ALT_F1R2:ALT_F2R1:FOXOG:PGT:PID:QSS:REF_F1R2:REF_F2R1	0/1:34,30:0.484:16:14:.:0|1:52475684_A_AGGGCACAGAAGATGCACT:959,852:16:18	0/0:75,5:0.067:2:3:.:0|1:52475684_A_AGGGCACAGAAGATGCACT:2053,141:39:36	0.5	Deleterious	-12.6	Deleterious	392	30	NA	NA	NA	NA
T12, T3	chr18	**2**	**25**	37341977	37341977	G	T	exonic	**Pcdhb7**	ENSMUSP00000117225	.	nonsynonymous SNV	Pcdhb7:NM_053132:exon1:c.G165T:p.Q55H	**Q55H**	ENSMUSP00000117225,55,Q,H	18,37341977,G,G	18qB3	.	0.25	.	79	chr18	37341977	.	G	G	.	clustered_events;multi_event_alt_allele_in_normal	ECNT=3;HCNT=4;MAX_ED=23;MIN_ED=20;NLOD=14.46;TLOD=15.97	GT:AD:AF:ALT_F1R2:ALT_F2R1:FOXOG:PGT:PID:QSS:REF_F1R2:REF_F2R1	0/1:40,6:0.140:1:5:0.833:0|1:37341997_C_T:1120,134:20:20	0/0:71,1:0.016:0:1:1.00:0|1:37341997_C_T:1968,22:39:32	1	Tolerated	0	Neutral	388	30	0.985	Tolerated	3.32	358
T22,T3	chr5	**2**	**25**	14713531	14713531	G	A	exonic	**Pclo**	ENSMUSP00000124446	.	nonsynonymous SNV	Pclo:NM_001110796:exon7:c.G12017A:p.R4006H,Pclo:NM_011995:exon7:c.G12017A:p.R4006H	**R4006H**	ENSMUSP00000124446,4006,R,H	5,14713531,G,GGGCATTGTTCCACTAGGACCCCACCGATCAGCTGGCAGCAAATT	5qA1	.	0.25	.	45	chr5	14713531	.	G	GGGCATTGTTCCACTAGGACCCCACCGATCAGCTGGCAGCAAATT	.	t_lod_fstar	ECNT=1;HCNT=1;MAX_ED=.;MIN_ED=.;NLOD=9.70;TLOD=5.46	GT:AD:AF:ALT_F1R2:ALT_F2R1:FOXOG:QSS:REF_F1R2:REF_F2R1	0/1:38,1:0.051:0:1:.:1031,28:25:13	0/0:31,0:0.00:0:0:.:850,0:11:20	1	Unknown	NA	NA	98	30	NA	NA	NA	NA
T12,T4	chr8	**2**	**25**	109623606	109623606	T	C	exonic	**Pkd1l3**	ENSMUSP00000054111	.	nonsynonymous SNV	Pkd1l3:NM_001039700:exon5:c.T1082C:p.L361S,Pkd1l3:NM_001286454:exon5:c.T1082C:p.L361S,Pkd1l3:NM_181544:exon5:c.T1082C:p.L361S	**L361S**	ENSMUSP00000054111,361,L,S	8,109623606,T,G	8qD3	.	0.25	.	37	chr8	109623606	.	T	G	.	PASS	ECNT=1;HCNT=1;MAX_ED=.;MIN_ED=.;NLOD=12.63;TLOD=7.42	GT:AD:AF:ALT_F1R2:ALT_F2R1:FOXOG:QSS:REF_F1R2:REF_F2R1	0/1:50,4:0.074:1:3:0.750:1433,117:24:26	0/0:46,0:0.00:0:0:.:1284,0:27:19	22	Unknown	NA	NA	63	30	NA	NA	NA	NA
T42,T4	chr17	**2**	**25**	35930837	35930837	G	A	exonic	**Ppp1r10**	ENSMUSP00000125030	.	nonsynonymous SNV	Ppp1r10:NM_001163818:exon18:c.G2503A:p.G835S	**G835S**	ENSMUSP00000125030,835,G,S	17,35930837,G,T	17qB1	.	0.25	.	26	chr17	35930837	.	G	T	.	clustered_events;homologous_mapping_event;t_lod_fstar	ECNT=3;HCNT=2;MAX_ED=15;MIN_ED=12;NLOD=7.22;TLOD=4.87	GT:AD:AF:ALT_F1R2:ALT_F2R1:FOXOG:PGT:PID:QSS:REF_F1R2:REF_F2R1	0/1:84,2:0.023:1:1:0.500:0|1:35930825_C_T:2123,55:43:41	0/0:26,0:0.00:0:0:.:0|1:35930825_C_T:633,0:15:11	0.9	Damaging	-1.14	Neutral	84	30	0.006	Damaging	4.32	21
T12,T32	chr17	**2**	**25**	35928844	35928844	T	A	exonic	**Ppp1r10**	ENSMUSP00000125030	.	nonsynonymous SNV	Ppp1r10:NM_001163818:exon13:c.T1454A:p.I485N	**I485N**	ENSMUSP00000125030,485,I,N	17,35928844,T,A	17qB1	.	0.25	.	37	chr17	35928844	.	T	A	.	clustered_events;homologous_mapping_event	ECNT=3;HCNT=2;MAX_ED=15;MIN_ED=12;NLOD=7.53;TLOD=7.76	GT:AD:AF:ALT_F1R2:ALT_F2R1:FOXOG:PGT:PID:QSS:REF_F1R2:REF_F2R1	0/1:86,3:0.033:1:2:0.333:0|1:35930825_C_T:2405,75:44:42	0/0:26,0:0.00:0:0:.:0|1:35930825_C_T:738,0:15:11	0.9	Deleterious	-4.66	Deleterious	84	30	0.002	Damaging	2.94	68
T12,T32	chr17	**2**	**25**	35928838	35928838	G	A	exonic	**Ppp1r10**	ENSMUSP00000125030	.	nonsynonymous SNV	Ppp1r10:NM_001163818:exon13:c.G1448A:p.R483Q	**R483Q**	ENSMUSP00000125030,483,R,Q	17,35928838,G,G	17qB1	.	0.25	.	38	chr17	35928838	.	G	G	.	clustered_events;homologous_mapping_event;t_lod_fstar	ECNT=4;HCNT=2;MAX_ED=8;MIN_ED=1;NLOD=10.23;TLOD=5.11	GT:AD:AF:ALT_F1R2:ALT_F2R1:FOXOG:PGT:PID:QSS:REF_F1R2:REF_F2R1	0/1:66,2:0.030:0:2:1.00:0|1:35928837_C_A:1821,58:37:29	0/0:37,0:0.00:0:0:.:0|1:35928837_C_A:985,0:14:23	0.9	Tolerated	0	Neutral	84	30	1	Tolerated	2.94	68
T12,T32	chr17	**2**	**25**	35928845	35928845	C	G	exonic	**Ppp1r10**	ENSMUSP00000125030	.	nonsynonymous SNV	Ppp1r10:NM_001163818:exon13:c.C1455G:p.I485M	**I485M**	ENSMUSP00000125030,485,I,M	17,35928845,C,T	17qB1	.	0.25	.	37	chr17	35928845	.	C	T	.	clustered_events;homologous_mapping_event	ECNT=3;HCNT=2;MAX_ED=15;MIN_ED=12;NLOD=8.13;TLOD=7.76	GT:AD:AF:ALT_F1R2:ALT_F2R1:FOXOG:PGT:PID:QSS:REF_F1R2:REF_F2R1	0/1:88,3:0.033:1:2:0.667:0|1:35930825_C_T:2454,87:44:44	0/0:28,0:0.00:0:0:.:0|1:35930825_C_T:765,0:16:12	0.9	Tolerated	0	Neutral	84	30	1	Tolerated	2.94	68
T42,T4	chr17	**2**	**25**	35930840	35930840	C	T	exonic	**Ppp1r10**	ENSMUSP00000125030	.	nonsynonymous SNV	Ppp1r10:NM_001163818:exon18:c.C2506T:p.H836Y	**H836Y**	ENSMUSP00000125030,836,H,Y	17,35930840,C,C	17qB1	.	0.25	.	28	chr17	35930840	.	C	C	.	alt_allele_in_normal	ECNT=1;HCNT=1;MAX_ED=.;MIN_ED=.;NLOD=3.50;TLOD=21.35	GT:AD:AF:ALT_F1R2:ALT_F2R1:FOXOG:QSS:REF_F1R2:REF_F2R1	0/1:57,10:0.156:5:5:0.500:1601,288:25:32	0/0:25,2:0.083:1:1:0.500:678,59:15:10	0.9	Tolerated	0	Neutral	84	30	1	Tolerated	4.32	21
T12,T32	chr2	**2**	**25**	28453349	28453349	G	A	exonic	**Ppp1r26**	ENSMUSP00000125568	.	nonsynonymous SNV	Ppp1r26:NM_001005420:exon3:c.G2990A:p.G997D	**G997D**	ENSMUSP00000125568,997,G,D	2,28453349,G,T	2qA3	rs212361752	0.25	.	49	chr2	28453349	rs212361752	G	T	.	clustered_events;homologous_mapping_event;t_lod_fstar	ECNT=4;HCNT=1;MAX_ED=6;MIN_ED=1;NLOD=5.35;TLOD=5.60	GT:AD:AF:ALT_F1R2:ALT_F2R1:FOXOG:PGT:PID:QSS:REF_F1R2:REF_F2R1	0/1:37,2:0.053:0:2:1.00:0|1:150455892_A_T:1006,56:18:19	0/0:29,0:0.036:0:0:.:0|1:150455892_A_T:785,0:15:14	20	Damaging	-1.04	Neutral	43	20	0.029	Damaging	3.1	32
T12,T32	chr2	**2**	**25**	28451494	28451494	G	A	exonic	**Ppp1r26**	ENSMUSP00000125568	.	nonsynonymous SNV	Ppp1r26:NM_001005420:exon3:c.G1135A:p.A379T	**A379T**	ENSMUSP00000125568,379,A,T	2,28451494,G,C	2qA3	rs252706057	0.25	.	21	chr2	28451494	rs252706057	G	C	.	t_lod_fstar	ECNT=1;HCNT=1;MAX_ED=.;MIN_ED=.;NLOD=9.84;TLOD=4.30	GT:AD:AF:ALT_F1R2:ALT_F2R1:FOXOG:QSS:REF_F1R2:REF_F2R1	0/1:35,3:0.086:0:3:0.00:983,73:18:17	0/0:35,0:0.00:0:0:.:969,0:20:15	1.9	Deleterious	-3.38	Deleterious	43	20	0.084	Tolerated	2.85	38

T12,T32	chr2	**2**	**25**	28450528	28450528	G	A	exonic	**Ppp1r26**	ENSMUSP00000125568	.	nonsynonymous SNV	Ppp1r26:NM_001005420:exon3:c.G169A:p.D57N	**D57N**	ENSMUSP00000125568,57,D,N	2,28450528,G,A	2qA3	rs33215877	0.25	.	25	chr2	28450528	rs33215877	G	A	.	PASS	DB;ECNT=1;HCNT=1;MAX_ED=.;MIN_ED=.;NLOD=12.03;TLOD=52.82	GT:AD:AF:ALT_F1R2:ALT_F2R1:FOXOG:QSS:REF_F1R2:REF_F2R1	0/1:15,21:0.559:10:11:0.476:431,597:8:7	0/0:49,0:0.00:0:0:.:1380,0:21:28	1.1	Tolerated	-2.05	Neutral	43	20	0.098	Tolerated	2.87	37
T1,T42	chr1	**2**	**25**	162709062	162709064	TGC	-	exonic	**Prrc2c**	ENSMUSP00000076157	.	nonframeshift deletion	Prrc2c:NM_001081290:exon14:c.2004_2006del:p.668_669del	**668_669del**	NA	1,162709062,TTGC,A	1qH2.1	.	0.25	.	33	chr1	162709061	.	TTGC	A	.	t_lod_fstar	ECNT=2;HCNT=1;MAX_ED=1;MIN_ED=1;NLOD=10.84;TLOD=5.01	GT:AD:AF:ALT_F1R2:ALT_F2R1:FOXOG:PGT:PID:QSS:REF_F1R2:REF_F2R1	0/1:77,2:0.027:2:0:0.00:0|1:21799494_T_G:1938,55:32:45	0/0:38,0:0.00:0:0:.:0|1:21799494_T_G:940,0:13:25	0.9	Unknown	.	.	.	.	.	.	.	.
T2,T4	chr17	**2**	**25**	66698439	66698439	T	G	exonic	**Ptprm**	ENSMUSP00000056530	.	nonsynonymous SNV	Ptprm:NM_008984:exon22:c.A3059C:p.K1020T	**K1020T**	ENSMUSP00000056530,1020,K,T	17,66698439,T,G	17qE1.1	.	0.25	.	44	chr17	66698439	.	T	G	.	t_lod_fstar	ECNT=1;HCNT=2;MAX_ED=.;MIN_ED=.;NLOD=9.33;TLOD=5.36	GT:AD:AF:ALT_F1R2:ALT_F2R1:FOXOG:QSS:REF_F1R2:REF_F2R1	0/1:56,2:0.047:0:2:1.00:1580,46:32:24	0/0:37,0:0.00:0:0:.:1001,0:12:25	1.8	Damaging	-0.79	Neutral	281	30	0.001	Damaging	2.9	253
T2,T3	chr2	**2**	**25**	104790150	104790150	G	C	exonic	**Qser1**	ENSMUSP00000051512	.	nonsynonymous SNV	Qser1:NM_001123327:exon3:c.C46G:p.P16A	**P16A**	ENSMUSP00000051512,16,P,A	2,104790150,G,C	2qE2	.	0.25	.	49	chr2	104790150	.	G	C	.	str_contraction;t_lod_fstar	ECNT=1;HCNT=1;MAX_ED=.;MIN_ED=.;NLOD=12.63;RPA=6,5;RU=AG;STR;TLOD=4.02	GT:AD:AF:ALT_F1R2:ALT_F2R1:FOXOG:QSS:REF_F1R2:REF_F2R1	0/1:31,2:0.065:1:1:.:872,59:16:15	0/0:47,0:0.00:0:0:.:1345,0:20:27	0.9	Deleterious	-4.11	Deleterious	96	30	0.006	Damaging	3.26	67
T12,T32	chr2	**2**	**25**	26220745	26220745	G	A	exonic	**Qsox2**	ENSP00000351536	.	nonsynonymous SNV	Qsox2:NM_001302956:exon8:c.C953T:p.T318I,Qsox2:NM_153559:exon8:c.C953T:p.T318I	**T318I**	ENSP00000351536,318,T,I	2,26220745,G,A	2qA3	rs33647617	0.25	.	23	chr2	26220745	rs33647617	G	A	.	PASS	DB;ECNT=1;HCNT=1;MAX_ED=.;MIN_ED=.;NLOD=6.62;TLOD=41.59	GT:AD:AF:ALT_F1R2:ALT_F2R1:FOXOG:QSS:REF_F1R2:REF_F2R1	0/1:21,18:0.444:9:9:0.500:582,506:8:13	0/0:23,0:0.00:0:0:.:657,0:13:10	1	Damaging	-0.12	Neutral	98	30	0.029	Damaging	2.81	104
T12df	chr11	**2**	**25**	109240883	109240883	-	AAA	exonic	**Rgs9**	ENSMUSP00000025253	.	nonframeshift insertion	Rgs9:NM_001165934:exon14:c.1021_1022insTTT:p.S341delinsFS,Rgs9:NM_011268:exon14:c.1021_1022insTTT:p.S341delinsFS	**S341delinsFS**	ENSMUSP00000025253,341,S,FS	11,109240883,G,A	11qE1	.	0.25	.	35	chr11	109240883	.	G	A	.	PASS	DB;ECNT=1;HCNT=1;MAX_ED=.;MIN_ED=.;NLOD=6.47;TLOD=35.36	GT:AD:AF:ALT_F1R2:ALT_F2R1:FOXOG:QSS:REF_F1R2:REF_F2R1	0/1:12,13:0.520:6:7:0.462:286,368:6:6	0/0:22,0:0.00:0:0:.:471,0:9:13	3	Deleterious	-4.16	Deleterious	150	30	0.034	Damaging	2.86	351
T1,T4	chr13	**2**	**25**	53110303	53110305	CCT	-	exonic	**Ror2**	ENSMUSP00000153179	.	nonframeshift deletion	Ror2:NM_013846:exon9:c.2750_2752del:p.917_918del	**917_918del**	NA	13,53110303,CCCT,C	13qB1	.	0.25	.	37	chr13	53110302	.	CCCT	C	.	PASS	ECNT=2;HCNT=2;MAX_ED=2;MIN_ED=2;NLOD=8.40;TLOD=7.31	GT:AD:AF:ALT_F1R2:ALT_F2R1:FOXOG:PGT:PID:QSS:REF_F1R2:REF_F2R1	0/1:42,3:0.068:1:2:.:0|1:90229853_CT_C:1212,76:22:20	0/0:35,0:0.00:0:0:.:0|1:90229853_CT_C:963,0:14:21	1.3	Unknown	.	.	.	.	.	.	.	.
T12,T32	chr2	**2**	**25**	26440020	26440020	T	C	exonic	**Sec16a**	ENSMUSP00000037128	.	nonsynonymous SNV	Sec16a:NM_153125:exon2:c.A1982G:p.Q661R	**Q661R**	ENSMUSP00000037128,661,Q,R	2,26440020,T,A	2qA3	.	0.25	.	41	chr2	26440020	.	T	A	.	str_contraction	ECNT=1;HCNT=1;MAX_ED=.;MIN_ED=.;NLOD=7.80;RPA=6,5;RU=AGC;STR;TLOD=8.45	GT:AD:AF:ALT_F1R2:ALT_F2R1:FOXOG:QSS:REF_F1R2:REF_F2R1	0/1:55,3:0.055:1:2:.:1538,85:31:24	0/0:30,0:0.00:0:0:.:834,0:17:13	0.5	Tolerated	-0.61	Neutral	104	30	0.277	Tolerated	3.3	42
T12,T4	chr9	**2**	**25**	46212058	46212060	CAG	-	exonic	**Sik3**	ENSMUSP00000020920	.	nonframeshift deletion	Sik3:NM_027498:exon21:c.3154_3156del:p.1052_1052del	**1052_1052del**	NA	9,46212058,ACAG,T	9qA5.2	.	0.25	.	39	chr9	46212057	.	ACAG	T	.	PASS	ECNT=1;HCNT=1;MAX_ED=.;MIN_ED=.;NLOD=8.84;TLOD=8.50	GT:AD:AF:ALT_F1R2:ALT_F2R1:FOXOG:QSS:REF_F1R2:REF_F2R1	0/1:56,3:0.054:1:2:.:1543,85:29:27	0/0:37,0:0.00:0:0:.:957,0:20:17	0.7	Unknown	.	.	.	.	.	.	.	.
T1,T4	chr2	**2**	**25**	18046345	18046347	TGC	-	exonic	**Skida1**	ENSMUSP00000054573	.	nonframeshift deletion	Skida1:NM_028317:exon1:c.1993_1995del:p.665_665del	**665_665del**	NA	2,18046345,TTGC,T	2qA3	.	0.25	.	41	chr2	18046344	.	TTGC	T	.	str_contraction;t_lod_fstar	ECNT=1;HCNT=2;MAX_ED=.;MIN_ED=.;NLOD=11.14;RPA=2,1;RU=ACATGGCTCCCACAGCAGGTACAGCGGC;STR;TLOD=4.17	GT:AD:AF:ALT_F1R2:ALT_F2R1:FOXOG:QSS:REF_F1R2:REF_F2R1	0/1:38,2:0.054:0:2:.:1025,59:22:16	0/0:45,0:0.00:0:0:.:1258,0:21:24	0.6	Unknown	.	.	.	.	.	.	.	.

T12,T32	chr2	**2**	**25**	26375651	26375651	C	T	exonic	**Snapc4**	ENSMUSP00000045740	.	nonsynonymous SNV	Snapc4:NM_172339:exon7:c.G589A:p.V197M,Snapc4:NM_001290419:exon8:c.G319A:p.V107M	**V107M**	ENSMUSP00000045740,107,V,M	2,26375651,C,T	2qA3	rs27202042	0.25	.	33	chr2	26375651	rs27202042	C	T	.	alt_allele_in_normal;clustered_events	ECNT=15;HCNT=7;MAX_ED=140;MIN_ED=12;NLOD=43.29;TLOD=24.62	GT:AD:AF:ALT_F1R2:ALT_F2R1:FOXOG:PGT:PID:QSS:REF_F1R2:REF_F2R1	0/1:197,9:0.046:5:4:0.444:0|1:85610752_C_T:5551,246:102:95	0/0:432,23:0.044:9:14:0.609:0|1:85610752_C_T:12189,657:211:221	0.2	Damaging	-0.89	Neutral	112	30	0.003	Damaging	3.31	47
T12,T32	chr2	**2**	**25**	26364769	26364769	T	C	exonic	**Snapc4**	ENSMUSP00000045740	.	nonsynonymous SNV	Snapc4:NM_172339:exon21:c.A3718G:p.I1240V,Snapc4:NM_001290419:exon22:c.A3448G:p.I1150V	**I1240V**	ENSMUSP00000045740,1240,I,V	2,26364769,T,C	2qA3	rs33468892	0.25	.	46	chr2	26364769	rs33468892	T	C	.	clustered_events	DB;ECNT=2;HCNT=2;MAX_ED=37;MIN_ED=37;NLOD=12.03;TLOD=53.67	GT:AD:AF:ALT_F1R2:ALT_F2R1:FOXOG:QSS:REF_F1R2:REF_F2R1	0/1:17,24:0.564:16:8:0.667:483,596:9:8	0/0:46,0:0.00:0:0:.:1306,0:18:28	1.1	Tolerated	0.36	Neutral	56	30	1	Tolerated	3.32	17
T12,T32	chr2	**2**	**25**	26375651	26375651	C	T	exonic	**Snapc4**	ENSMUSP00000045740	.	nonsynonymous SNV	Snapc4:NM_172339:exon7:c.G589A:p.V197M,Snapc4:NM_001290419:exon8:c.G319A:p.V107M	**V197M**	ENSMUSP00000045740,197,V,M	2,26375651,C,T	2qA3	rs27202042	0.25	.	33	chr2	26375651	rs27202042	C	T	.	PASS	DB;ECNT=1;HCNT=1;MAX_ED=.;MIN_ED=.;NLOD=9.49;TLOD=53.57	GT:AD:AF:ALT_F1R2:ALT_F2R1:FOXOG:QSS:REF_F1R2:REF_F2R1	0/1:37,22:0.393:7:15:0.682:1016,602:17:20	0/0:33,0:0.00:0:0:.:870,0:17:16	1.1	Tolerated	-0.96	Neutral	56	30	0.141	Tolerated	2.79	56
T12,T32	chr2	**2**	**25**	26364769	26364769	T	C	exonic	**Snapc4**	ENSMUSP00000045740	.	nonsynonymous SNV	Snapc4:NM_172339:exon21:c.A3718G:p.I1240V,Snapc4:NM_001290419:exon22:c.A3448G:p.I1150V	**I1150V**	ENSMUSP00000045740,1150,I,V	2,26364769,T,CTG	2qA3	rs33468892	0.25	.	46	chr2	26364769	rs33468892	T	CTG	.	t_lod_fstar	ECNT=1;HCNT=1;MAX_ED=.;MIN_ED=.;NLOD=9.94;TLOD=5.65	GT:AD:AF:ALT_F1R2:ALT_F2R1:FOXOG:QSS:REF_F1R2:REF_F2R1	0/1:36,2:0.056:0:2:.:971,58:17:19	0/0:36,0:0.00:0:0:.:869,0:19:17	0.8	Unknown	NA	NA	56	30	NA	NA	NA	NA
T2,T42	chr1	**2**	**25**	85594358	85594358	T	C	exonic	**Sp110**	ENSMUSP00000040248	.	nonsynonymous SNV	Sp110:NM_030194:exon3:c.A223G:p.T75A,Sp110:NM_175397:exon4:c.A223G:p.T75A	**T75A**	ENSMUSP00000040248,75,T,A	1,85594358,T,G	1qC5	rs47718372	0.25	.	284	chr1	85594358	.	T	G	.	alt_allele_in_normal;clustered_events;t_lod_fstar	ECNT=5;HCNT=11;MAX_ED=83;MIN_ED=7;NLOD=49.63;TLOD=4.13	GT:AD:AF:ALT_F1R2:ALT_F2R1:FOXOG:QSS:REF_F1R2:REF_F2R1	0/1:166,4:0.023:3:1:0.250:4780,115:87:79	0/0:285,8:0.031:3:5:0.625:7981,222:138:147	0.3	Deleterious	-4.56	Deleterious	104	30	0.003	Damaging	2.79	110
T2,T4	chr1	**2**	**25**	85586225	85586225	A	T	exonic	**Sp110**	ENSMUSP00000040248	.	nonsynonymous SNV	Sp110:NM_030194:exon7:c.T810A:p.D270E,Sp110:NM_175397:exon8:c.T810A:p.D270E	**D270E**	ENSMUSP00000040248,270,D,E	1,85586225,A,A	1qC5	.	0.25	.	331	chr1	85586225	.	A	A	.	alt_allele_in_normal;clustered_events	ECNT=4;HCNT=2;MAX_ED=50;MIN_ED=25;NLOD=16.82;TLOD=17.55	GT:AD:AF:ALT_F1R2:ALT_F2R1:FOXOG:PGT:PID:QSS:REF_F1R2:REF_F2R1	0/1:140,9:0.095:3:6:0.667:0|1:85586225_A_T:2388,261:56:43	0/0:263,16:0.097:6:10:0.625:0|1:85586225_A_T:3953,458:92:68	0.3	Tolerated	0	Neutral	104	30	1	Tolerated	2.85	32
T2,T4	chr1	**2**	**25**	85586232	85586232	C	T	exonic	**Sp110**	ENSMUSP00000040248	.	nonsynonymous SNV	Sp110:NM_030194:exon7:c.G803A:p.R268H,Sp110:NM_175397:exon8:c.G803A:p.R268H	**R268H**	ENSMUSP00000040248,268,R,H	1,85586232,C,G	1qC5	.	0.25	.	319	chr1	85586232	.	C	G	.	alt_allele_in_normal;clustered_events;t_lod_fstar	ECNT=4;HCNT=5;MAX_ED=127;MIN_ED=7;NLOD=49.95;TLOD=5.02	GT:AD:AF:ALT_F1R2:ALT_F2R1:FOXOG:QSS:REF_F1R2:REF_F2R1	0/1:161,6:0.035:3:3:0.500:4297,124:84:77	0/0:259,6:0.024:1:5:0.167:6875,167:122:137	0.3	Tolerated	0.35	Neutral	104	30	0.297	Tolerated	2.83	55
T2,T4	chr1	**2**	**25**	85586250	85586250	C	A	exonic	**Sp110**	ENSMUSP00000040248	.	nonsynonymous SNV	Sp110:NM_030194:exon7:c.G785T:p.R262L,Sp110:NM_175397:exon8:c.G785T:p.R262L	**R262L**	ENSMUSP00000040248,262,R,L	1,85586250,C,C	1qC5	.	0.25	.	279	chr1	85586250	.	C	C	.	alt_allele_in_normal;clustered_events	ECNT=4;HCNT=2;MAX_ED=127;MIN_ED=7;NLOD=36.61;TLOD=15.53	GT:AD:AF:ALT_F1R2:ALT_F2R1:FOXOG:QSS:REF_F1R2:REF_F2R1	0/1:176,6:0.033:4:2:0.667:4976,179:90:86	0/0:271,13:0.046:5:8:0.385:7407,367:132:139	0.3	Tolerated	0	Neutral	104	30	1	Tolerated	2.83	58
T12,T2	chr1	**2**	**25**	85591760	85591760	G	A	exonic	**Sp110**	ENSMUSP00000040248	.	nonsynonymous SNV	Sp110:NM_030194:exon4:c.C346T:p.P116S,Sp110:NM_175397:exon5:c.C346T:p.P116S	**P116S**	ENSMUSP00000040248,116,P,S	1,85591760,G,T	1qC5	rs107933776	0.25	.	715	chr1	85591760	.	G	T	.	alt_allele_in_normal;clustered_events	ECNT=3;HCNT=13;MAX_ED=60;MIN_ED=52;NLOD=22.30;TLOD=20.02	GT:AD:AF:ALT_F1R2:ALT_F2R1:FOXOG:QSS:REF_F1R2:REF_F2R1	0/1:349,15:0.044:7:8:0.533:9675,427:173:176	0/0:412,26:0.070:16:10:0.385:11473,740:205:207	0.3	Tolerated	-0.4	Neutral	104	30	0.61	Tolerated	2.89	75
T2,T42	chr1	**2**	**25**	85594351	85594351	T	G	exonic	**Sp110**	ENSMUSP00000040248	.	nonsynonymous SNV	Sp110:NM_030194:exon3:c.A230C:p.H77P,Sp110:NM_175397:exon4:c.A230C:p.H77P	**H77P**	ENSMUSP00000040248,77,H,P	1,85594351,T,T	1qC5	rs108624819	0.25	.	265	chr1	85594351	.	T	T	.	alt_allele_in_normal;clustered_events	ECNT=6;HCNT=11;MAX_ED=200;MIN_ED=15;NLOD=42.18;TLOD=32.31	GT:AD:AF:ALT_F1R2:ALT_F2R1:FOXOG:QSS:REF_F1R2:REF_F2R1	0/1:399,28:0.064:17:11:0.607:10522,814:188:211	0/0:430,27:0.064:13:14:0.481:10880,751:227:203	0.3	Tolerated	0	Neutral	104	30	0.129	Tolerated	2.79	110
T1,T42	chr1	**2**	**25**	85610745	85610745	C	T	exonic	**Sp140**	ENSMUSP00000108782	.	stopgain	Sp140:NM_001013817:exon3:c.C202T:p.Q68X	**Q68X**	ENSMUSP00000108782,68,Q,X	NA	1qC5	.	0.25	.	462	chr1	85610745	.	C	A	.	alt_allele_in_normal;clustered_events	ECNT=11;HCNT=5;MAX_ED=129;MIN_ED=1;NLOD=32.14;TLOD=21.40	GT:AD:AF:ALT_F1R2:ALT_F2R1:FOXOG:PGT:PID:QSS:REF_F1R2:REF_F2R1	0/1:198,8:0.040:6:2:0.750:0|1:85610790_G_T:5733,225:96:102	0/0:373,15:0.045:7:8:0.467:0|1:85610790_G_T:10410,424:172:201	0.2	Deletarious	.	.	.	.	.	.	.	.

T22,T32	chr18	**2**	**25**	52475684	52475684	-	GGGCACAGAAGATGCACT	exonic	**Srfbp1**	ENSMUSP00000078795	.	nonframeshift insertion	Srfbp1:NM_026040:exon2:c.155_156insGGGCACAGAAGATGCACT:p.K52delinsKGTEDAL	**K52delinsKGTEDAL**	ENSMUSP00000078795,52,K,KGTEDAL	18,52475684,A,G	18qD1	.	0.25	.	80	chr18	52475684	.	A	G	.	clustered_events;multi_event_alt_allele_in_normal	ECNT=5;HCNT=1;MAX_ED=25;MIN_ED=2;NLOD=6.94;TLOD=11.50	GT:AD:AF:ALT_F1R2:ALT_F2R1:FOXOG:PGT:PID:QSS:REF_F1R2:REF_F2R1	0/1:62,4:0.063:0:4:1.00:0|1:4105318 2_T_TC:1740,49:31:31	0/0:40,1:0.026:0:1:1.00:0|1:4105318 2_T_TC:1092,24:24:16	0.4	Tolerated	-1.64	Neutral	62	30	0.075	Tolerated	2.87	44
T1df	chr10	**2**	**25**	80128027	80128029	GGA	-	exonic	**Stk11**	ENSMUSP00000070610	.	nonframeshift deletion	Stk11:NM_001301853:exon8:c.1044_1046del:p.348_349del,Stk11:NM_001301854:exon8:c.672_674del:p.224_225del,Stk11:NM_011492:exon8:c.1044_1046del:p.348_349del	**348_349del**	NA	10,80128027,CGGA,G	10qC1	.	0.25	.	36	chr10	80128026	.	CGGA	G	.	t_lod_fstar	ECNT=1;HCNT=1;MAX_ED=.;MIN_ED=.;NLOD=9.63;TLOD=5.83	GT:AD:AF:ALT_F1R2:ALT_F2R1:FOXOG:QSS:REF_F1R2:REF_F2R1	0/1:37,3:0.081:0:3:.:975,82:22:15	0/0:36,0:0.00:0:0:.:925,0:18:18	0.8	Unknown	.	.	.	.	.	.	.	.
T22df	chr5	**2**	**25**	33664342	33664485	GAGCCAGTGCTGGAGCCTTCCCATCAGGGACTAGAACCTGTCTTGGAAAGTGAGCTGGTGACCCCTCCTGTTGAGCCAGTGCTGGAGCCTTCCCATCAGGAACTAGAACCTGTCTTGGAAAGTGAGCTGGTGACCCCTCCTATT	-	exonic	**Tacc3**	ENSMUSP00000100070	.	nonframeshift deletion	Tacc3:NM_001040435:exon5:c.445_588del:p.149_196del,Tacc3:NM_001310541:exon5:c.445_588del:p.149_196del	**149_196del**	NA	5,33664342,CGAGCCAGTGCTGGAGCCTTCCCATCAGGGACTAGAACCTGTCTTGGAAAGTGAGCTGGTGACCCCTCCTGTTGAGCCAGTGCTGGAGCCTTCCCATCAGGAACTAGAACCTGTCTTGGAAAGTGAGCTGGTGACCCCTCCTATT,A	5qB2	.	0.25	.	65	chr5	33664341	.	CGAGCCAGTGCTGGAGCCTTCCCATCAGGGACTAGAACCTGTCTTGGAAAGTGAGCTGGTGACCCCTCCTGTTGAGCCAGTGCTGGAGCCTTCCCATCAGGAACTAGAACCTGTCTTGGAAAGTGAGCTGGTGACCCCTCCTATT	A	.	t_lod_fstar	ECNT=1;HCNT=3;MAX_ED=.;MIN_ED=.;NLOD=15.04;TLOD=5.85	GT:AD:AF:ALT_F1R2:ALT_F2R1:FOXOG:QSS:REF_F1R2:REF_F2R1	0/1:23,3:0.120:3:0:1.00:654,84:8:15	0/0:58,0:0.00:0:0:.:1640,0:24:34	2.7	Unknown	.	.	.	.	.	.	.	.
T22df	chr5	**2**	**25**	64955176	64955176	G	C	exonic	**Tlr6**	ENSP00000371376	.	nonsynonymous SNV	Tlr6:NM_011604:exon2:c.C387G:p.C129W	**C129W**	ENSP00000371376,129,C,W	5,64955176,G,C	5qC3.1	.	0.25	.	50	chr5	64955176	.	G	C	.	PASS	ECNT=1;HCNT=1;MAX_ED=.;MIN_ED=.;NLOD=12.93;TLOD=9.58	GT:AD:AF:ALT_F1R2:ALT_F2R1:FOXOG:QSS:REF_F1R2:REF_F2R1	0/1:37,5:0.122:3:2:0.600:1041,138:16:21	0/0:50,0:0.00:0:0:.:1394,0:31:19	0.5	Damaging	0.76	Neutral	362	30	0.009	Damaging	2.85	248
T12,T32	chr2	**2**	**25**	29064947	29064947	A	G	exonic	**Ttf1**	ENSMUSP00000106585	.	nonsynonymous SNV	Ttf1:NM_009442:exon2:c.A322G:p.T108A	**T108A**	ENSMUSP00000106585,108,T,A	2,29064947,A,C	2qA3	rs27211101	0.25	.	37	chr2	29064947	rs27211101	A	C	.	PASS	DB;ECNT=1;HCNT=1;MAX_ED=.;MIN_ED=.;NLOD=13.24;TLOD=50.22	GT:AD:AF:ALT_F1R2:ALT_F2R1:FOXOG:QSS:REF_F1R2:REF_F2R1	0/1:22,23:0.487:10:13:0.435:642,643:10:12	0/0:50,0:0.00:0:0:.:1400,0:23:27	0.7	Damaging	-0.4	Neutral	114	30	0.012	Damaging	4.32	9
T12,T32	chr2	**2**	**25**	29064882	29064882	C	A	exonic	**Ttf1**	ENSMUSP00000106585	.	nonsynonymous SNV	Ttf1:NM_009442:exon2:c.C257A:p.T86N	**T86N**	ENSMUSP00000106585,86,T,N	2,29064882,C,G	2qA3	rs47186598	0.25	.	31	chr2	29064882	rs47186598	C	G	.	clustered_events;homologous_mapping_event	DB;ECNT=6;HCNT=4;MAX_ED=191;MIN_ED=3;NLOD=11.74;TLOD=38.81	GT:AD:AF:ALT_F1R2:ALT_F2R1:FOXOG:PGT:PID:QSS:REF_F1R2:REF_F2R1	0/1:12,14:0.500:8:6:0.571:0|1:29065034_T_G:331,371:7:5	0/0:46,0:0.00:0:0:.:0|1:29065034_T_G:1252,0:20:26	0.7	Tolerated	0.43	Neutral	114	30	1	Tolerated	4.32	8
T12,T32	chr2	**2**	**25**	29065034	29065034	T	G	exonic	**Ttf1**	ENSMUSP00000106585	.	nonsynonymous SNV	Ttf1:NM_009442:exon2:c.T409G:p.S137A	**S137A**	ENSMUSP00000106585,137,S,A	2,29065034,T,G	2qA3	rs27211100	0.25	.	46	chr2	29065034	rs27211100	T	G	.	clustered_events;homologous_mapping_event	ECNT=6;HCNT=4;MAX_ED=191;MIN_ED=3;NLOD=6.92;TLOD=59.10	GT:AD:AF:ALT_F1R2:ALT_F2R1:FOXOG:PGT:PID:QSS:REF_F1R2:REF_F2R1	0/1:13,14:0.536:8:6:.:0|1:29064857_GAA_G:361,375:9:4	0/0:24,0:0.00:0:0:.:0|1:29064857_GAA_G:657,0:13:11	0.7	Tolerated	-0.39	Neutral	114	30	0.551	Tolerated	4.32	9
T12,T32	chr2	**2**	**25**	29064860	29064860	-	GT	exonic	**Ttf1**	ENSMUSP00000106585	.	frameshift insertion	Ttf1:NM_009442:exon2:c.235_236insGT:p.P79fs	**P79fs**	ENSMUSP00000106585,79,P,	2,29064860,C,T	2qA3	.	0.25	.	25	chr2	29064860	.	C	T	.	str_contraction;t_lod_fstar	ECNT=1;HCNT=1;MAX_ED=.;MIN_ED=.;NLOD=10.21;RPA=4,3;RU=TGC;STR;TLOD=4.88	GT:AD:AF:ALT_F1R2:ALT_F2R1:FOXOG:QSS:REF_F1R2:REF_F2R1	0/1:85,2:0.023:1:1:.:2355,56:39:46	0/0:36,0:0.00:0:0:.:1004,0:18:18	8	Tolerated	0.63	Neutral	114	30	1	Tolerated	4.32	8
T12,T32	chr2	**2**	**25**	29084809	29084809	G	C	exonic	**Ttf1**	ENSMUSP00000106585	.	nonsynonymous SNV	Ttf1:NM_009442:exon11:c.G2556C:p.E852D	**E852D**	ENSMUSP00000106585,852,E,D	2,29084809,G,CGT	2qA3	rs29815824	0.25	.	50	chr2	29084809	rs29815824	G	CGT	.	clustered_events;homologous_mapping_event	ECNT=6;HCNT=4;MAX_ED=191;MIN_ED=3;NLOD=6.92;TLOD=59.10	GT:AD:AF:ALT_F1R2:ALT_F2R1:FOXOG:PGT:PID:QSS:REF_F1R2:REF_F2R1	0/1:12,15:0.536:9:6:.:0|1:29064857_GAA_G:327,351:9:3	0/0:25,0:0.00:0:0:.:0|1:29064857_GAA_G:607,0:14:11	0.7	Unknown	NA	NA	114	30	NA	NA	NA	NA

T12,T32	chr2	**2**	**25**	29064858	29064859	AA	-	exonic	**Ttf1**	ENSMUSP00000106585	.	frameshift deletion	Ttf1:NM_009442:exon2:c.233_234del:p.E78fs	**E78fs**	ENSMUSP00000106585,78,E,.	2,29064858,GAA,C	2qA3	.	0.25	.	24	chr2	29064857	.	GAA	C	.	PASS	ECNT=1;HCNT=12;MAX_ED=.;MIN_ED=.;NLOD=18.25;TLOD=13.68	GT:AD:AF:ALT_F1R2:ALT_F2R1:FOXOG:QSS:REF_F1R2:REF_F2R1	0/1:54,11:0.151:9:2:0.818:1494,269:27:27	0/0:81,0:0.00:0:0:.:2193,0:48:33	2.3	Unknown	NA	NA	114	30	NA	NA	NA	NA
T42df	chr3	**2**	**25**	146940053	146940055	CAG	-	exonic	**Ttll7**	ENSMUSP00000110069	.	nonframeshift deletion	Ttll7:NM_001302957:exon15:c.1665_1667del:p.555_556del,Ttll7:NM_001302958:exon15:c.1665_1667del:p.555_556del,Ttll7:NM_001357217:exon15:c.1665_1667del:p.555_556del	**555_556del**	NA	3,146940053,ACAG,C	3qH2	.	0.25	.	38	chr3	146940052	.	ACAG	C	.	alt_allele_in_normal;t_lod_fstar	ECNT=1;HCNT=2;MAX_ED=.;MIN_ED=.;NLOD=2.79;TLOD=5.63	GT:AD:AF:ALT_F1R2:ALT_F2R1:FOXOG:QSS:REF_F1R2:REF_F2R1	0/1:54,8:0.125:3:5:0.375:1375,143:28:26	0/0:24,5:0.192:0:5:0.00:639,29:7:17	1.3	Unknown	.	.	.	.	.	.	.	.
T12,T4	chr4	**2**	**25**	41199898	41199898	-	TTTTTTTTAGCTGCTAGTCACTGAGCCCTTACTGTGATCCGATGAGTTGGAAGAGATGGTTTGTATTTACAGTGT	exonic	**Ubap2**	ENSMUSP00000047105	.	stopgain	Ubap2:NM_026872:exon20:c.2230_2231insACACTGTAAATACAAACCATCTCTTCCAACTCATCGGATCACAGTAAGGGCTCAGTGACTAGCAGCTAAAAAAAA:p.A744delinsDTVNTNHLFQLIGSQX	**A744delinsDTVNTNHLFQLIGSQX**	ENSMUSP00000047105,744,A,DTVNTNHLFQLIGSOX	NA	4qA5	.	0.25	.	40	chr4	41199898	.	G	G	.	PASS	ECNT=1;HCNT=1;MAX_ED=.;MIN_ED=.;NLOD=13.49;TLOD=7.83	GT:AD:AF:ALT_F1R2:ALT_F2R1:FOXOG:QSS:REF_F1R2:REF_F2R1	0/1:41,4:0.093:3:1:0.250:1156,117:26:15	0/0:55,0:0.00:0:0:.:1530,0:17:38	1.4	Deletarious	.	.	.	.	.	.	.	.
T12,T4	chr4	**2**	**25**	41195852	41195941	ACCTAGGATGACAGGGCACAAGAGGCATGTGTGGGCTAAGCAGGTCTCTCTGAGCATGTGCCGCTCAGTACTGGGAGTGATGCAGTCCTT	-	exonic	**Ubap2**	ENSMUSP00000047105	.	frameshift deletion	Ubap2:NM_026872:exon26:c.2939_2940del:p.G980fs	**G980fs**	ENSMUSP00000047105,980,G,.	4,41195852,AACCTAGGATGACAGGGCACAAGAGGCATGTGTGGGCTAAGCAGGTCTCTCTGAGCATGTGCCGCTCAGTACTGGGAGTGATGCAGTCCTT,CAAACATAGTAGGCCCATGCTGGAAGGCACTGGGCACGCCTGTGTAGTAGGGGAGGCCGGTATAG	4qA5	.	0.25	.	57	chr4	41195851	.	AACCTAGGATGACAGGGCACAAGAGGCATGTGTGGGCTAAGCAGGTCTCTCTGAGCATGTGCCGCTCAGTACTGGGAGTGATGCAGTCCTT	CAAACATAGTAGGCCCATGCTGGAAGGCACTGGGCACGCCTGTGTAGTAGGGGAGGCCGGTATAG	.	PASS	ECNT=1;HCNT=1;MAX_ED=.;MIN_ED=.;NLOD=11.54;TLOD=32.29	GT:AD:AF:ALT_F1R2:ALT_F2R1:FOXOG:QSS:REF_F1R2:REF_F2R1	0/1:64,10:0.145:4:6:.:1752,272:33:31	0/0:41,0:0.00:0:0:.:1079,0:20:21	1.4	Unknown	NA	NA	169	30	NA	NA	NA	NA
T42,T4	chr10	**2**	**25**	88807460	88807460	G	C	exonic	**Utp20**	ENSMUSP00000057527	.	nonsynonymous SNV	Utp20:NM_175158:exon16:c.C1799G:p.P600R	**P600R**	ENSMUSP00000057527,600,P,R	10,88807460,G,G	10qC1	.	0.25	.	53	chr10	88807460	.	G	G	.	clustered_events;multi_event_alt_allele_in_normal	ECNT=4;HCNT=28;MAX_ED=33;MIN_ED=8;NLOD=6.64;TLOD=21.13	GT:AD:AF:ALT_F1R2:ALT_F2R1:FOXOG:PGT:PID:QSS:REF_F1R2:REF_F2R1	0/1:51,6:0.130:3:3:0.500:0|1:33962974_T_G:1447,161:26:25	0/0:41,1:0.027:0:1:0.00:0|1:33962974_T_G:1157,20:28:13	1	Tolerated	0	Neutral	71	30	0.435	Tolerated	2.93	43
T22,T2	chr7	**2**	**25**	98853274	98853274	A	G	exonic	**Wnt11**	ENSMUSP00000025706	.	nonsynonymous SNV	Wnt11:NM_001285795:exon5:c.A545G:p.Y182C,Wnt11:NM_009519:exon5:c.A1052G:p.Y351C,Wnt11:NM_001285794:exon6:c.A857G:p.Y286C,Wnt11:NM_001285792:exon7:c.A1052G:p.Y351C	**Y182C**	ENSMUSP00000025706,182,Y,C	7,98853274,A,G	7qE1	.	0.25	.	31	chr7	98853274	.	A	G	.	PASS	ECNT=1;HCNT=1;MAX_ED=.;MIN_ED=.;NLOD=8.42;TLOD=20.44	GT:AD:AF:ALT_F1R2:ALT_F2R1:FOXOG:QSS:REF_F1R2:REF_F2R1	0/1:33,9:0.220:4:5:0.556:918,255:14:19	0/0:31,0:0.00:0:0:.:866,0:9:22	7	Deleterious	-4.82	Deleterious	238	30	0.011	Damaging	2.84	100
T22,T2	chr7	**2**	**25**	98853274	98853274	A	G	exonic	**Wnt11**	ENSMUSP00000025706	.	nonsynonymous SNV	Wnt11:NM_001285795:exon5:c.A545G:p.Y182C,Wnt11:NM_009519:exon5:c.A1052G:p.Y351C,Wnt11:NM_001285794:exon6:c.A857G:p.Y286C,Wnt11:NM_001285792:exon7:c.A1052G:p.Y351C	**Y286C**	ENSMUSP00000025706,286,Y,C	7,98853274,A,A	7qE1	.	0.25	.	31	chr7	98853274	.	A	A	.	alt_allele_in_normal;clustered_events;germline_risk	ECNT=4;HCNT=15;MAX_ED=29;MIN_ED=24;NLOD=1.86;TLOD=8.42	GT:AD:AF:ALT_F1R2:ALT_F2R1:FOXOG:QSS:REF_F1R2:REF_F2R1	0/1:50,5:0.094:3:2:0.600:1346,132:20:30	0/0:38,3:0.077:2:1:0.667:1071,84:16:22	1.9	Tolerated	0	Neutral	238	30	1	Tolerated	2.84	100
T3,T42	chr4	**2**	**25**	119279189	119279189	G	A	exonic	**Ybx1**	ENSMUSP00000133076	.	nonsynonymous SNV	Ybx1:NM_011732:exon7:c.C745T:p.R249C	**R249C**	ENSMUSP00000133076,249,R,C	4,119279189,G,T	4qD2.1	.	0.25	.	45	chr4	119279189	.	G	T	.	str_contraction;t_lod_fstar	ECNT=1;HCNT=1;MAX_ED=.;MIN_ED=.;NLOD=8.11;RPA=5,4;RU=TCC;STR;TLOD=5.21	GT:AD:AF:ALT_F1R2:ALT_F2R1:FOXOG:QSS:REF_F1R2:REF_F2R1	0/1:59,2:0.034:0:2:.:1681,58:28:31	0/0:30,0:0.00:0:0:.:821,0:16:14	2.3	Deleterious	-2.91	Deleterious	213	30	0.315	Tolerated	3.09	107
T1,T3	chr5	**2**	**25**	138125503	138125503	G	A	exonic	**Zscan21**	ENSMUSP00000032503	.	nonsynonymous SNV	Zscan21:NM_001044703:exon2:c.G443A:p.C148Y,Zscan21:NM_001044705:exon2:c.G443A:p.C148Y,Zscan21:NM_001044704:exon3:c.G443A:p.C148Y,Zscan21:NM_011757:exon3:c.G443A:p.C148Y	**C148Y**	ENSMUSP00000032503,148,C,Y	5,138125503,G,T	5qG2	.	0.25	.	45	chr5	138125503	.	G	T	.	alt_allele_in_normal;clustered_events;germline_risk	ECNT=9;HCNT=1;MAX_ED=30;MIN_ED=2;NLOD=1.79;TLOD=10.14	GT:AD:AF:ALT_F1R2:ALT_F2R1:FOXOG:PGT:PID:QSS:REF_F1R2:REF_F2R1	0/1:62,5:0.065:5:0:0.00:0|1:138125496_C_T:1719,123:30:32	0/0:36,2:0.056:2:0:0.00:0|1:138125496_C_T:973,46:20:16	1.4	Deleterious	-9.96	Deleterious	164	30	0.033	Damaging	2.79	114

T1,T3	chr5	**2**	**25**	138125496	138125496	C	T	exonic	**Zscan21**	ENSMUSP00000018651	.	nonsynonymous SNV	Zscan21:NM_001044703:exon2:c.C436T:p.L146F,Zscan21:NM_001044705:exon2:c.C436T:p.L146F,Zscan21:NM_001044704:exon3:c.C436T:p.L146F,Zscan21:NM_011757:exon3:c.C436T:p.L146F	**L146F**	ENSMUSP00000018651,146,L,F	5,138125496,C,G	5qG2	.	0.25	.	47	chr5	138125496	.	C	G	.	alt_allele_in_normal;clustered_events	ECNT=9;HCNT=1;MAX_ED=30;MIN_ED=2;NLOD=3.34;TLOD=11.53	GT:AD:AF:ALT_F1R2:ALT_F2R1:FOXOG:PGT:PID:QSS:REF_F1R2:REF_F2R1	0/1:62,4:0.063:4:0:0.00:0|1:138125496_C_T:1704,93:30:32	0/0:41,2:0.049:2:0:0.00:0|1:138125496_C_T:1078,43:23:18	1.4	Deleterious	-2.54	Deleterious	268	30	0.001	Damaging	2.83	236
T1,T3	chr5	**2**	**25**	138125515	138125515	A	G	exonic	**Zscan21**	ENSMUSP00000018651	.	nonsynonymous SNV	Zscan21:NM_001044703:exon2:c.A455G:p.Q152R,Zscan21:NM_001044705:exon2:c.A455G:p.Q152R,Zscan21:NM_001044704:exon3:c.A455G:p.Q152R,Zscan21:NM_011757:exon3:c.A455G:p.Q152R	**Q152R**	ENSMUSP00000018651,152,Q,R	5,138125515,A,T	5qG2	.	0.25	.	43	chr5	138125515	.	A	T	.	alt_allele_in_normal;clustered_events	ECNT=9;HCNT=1;MAX_ED=30;MIN_ED=2;NLOD=4.85;TLOD=11.56	GT:AD:AF:ALT_F1R2:ALT_F2R1:FOXOG:PGT:PID:QSS:REF_F1R2:REF_F2R1	0/1:62,4:0.065:4:0:.:0|1:138125496_C_T:1820,98:30:32	0/0:44,2:0.043:2:0:.:0|1:138125496_C_T:1239,54:23:21	1.4	Deleterious	-2.92	Deleterious	268	30	0.001	Damaging	2.83	236
T1,T3	chr5	**2**	**25**	138125526	138125526	C	T	exonic	**Zscan21**	ENSMUSP00000018651	.	nonsynonymous SNV	Zscan21:NM_001044703:exon2:c.C466T:p.H156Y,Zscan21:NM_001044705:exon2:c.C466T:p.H156Y,Zscan21:NM_001044704:exon3:c.C466T:p.H156Y,Zscan21:NM_011757:exon3:c.C466T:p.H156Y	**H156Y**	ENSMUSP00000018651,156,H,Y	5,138125526,C,A	5qG2	.	0.25	.	38	chr5	138125526	.	C	A	.	alt_allele_in_normal;clustered_events	ECNT=9;HCNT=1;MAX_ED=30;MIN_ED=2;NLOD=4.25;TLOD=11.50	GT:AD:AF:ALT_F1R2:ALT_F2R1:FOXOG:PGT:PID:QSS:REF_F1R2:REF_F2R1	0/1:63,4:0.063:4:0:.:0|1:138125496_C_T:1810,101:32:31	0/0:44,2:0.045:2:0:.:0|1:138125496_C_T:1260,51:23:21	1.4	Deleterious	-3.17	Deleterious	268	30	0.16	Tolerated	2.83	236
T1,T3	chr5	**2**	**25**	138125517	138125517	-	GACCA	exonic	**Zscan21**	ENSMUSP00000018651	.	frameshift insertion	Zscan21:NM_001044703:exon2:c.457_458insGACCA:p.P153fs,Zscan21:NM_001044705:exon2:c.457_458insGACCA:p.P153fs,Zscan21:NM_001044704:exon3:c.457_458insGACCA:p.P153fs,Zscan21:NM_011757:exon3:c.457_458insGACCA:p.P153fs	**P153fs**	ENSMUSP00000018651,153,P,	5,138125517,C,G	5qG2	.	0.25	.	42	chr5	138125517	.	C	G	.	PASS	ECNT=1;HCNT=2;MAX_ED=.;MIN_ED=.;NLOD=10.13;TLOD=93.84	GT:AD:AF:ALT_F1R2:ALT_F2R1:FOXOG:QSS:REF_F1R2:REF_F2R1	0/1:12,35:0.744:16:19:0.543:307,978:3:9	0/0:35,0:0.00:0:0:.:832,0:22:13	1.7	Deleterious	-6.78	Deleterious	268	30	0.012	Damaging	2.83	236
T1,T3	chr5	**2**	**25**	138125500	138125501	GC	-	exonic	**Zscan21**	ENSMUSP00000032503	.	frameshift deletion	Zscan21:NM_001044703:exon2:c.440_441del:p.C147fs,Zscan21:NM_001044705:exon2:c.440_441del:p.C147fs,Zscan21:NM_001044704:exon3:c.440_441del:p.C147fs,Zscan21:NM_011757:exon3:c.440_441del:p.C147fs	**C147fs**	ENSMUSP00000032503,147,C,.	5,138125500,TGC,TTA	5qG2	.	0.25	.	46	chr5	138125499	.	TGC	TTA	.	alt_allele_in_normal;clustered_events	ECNT=9;HCNT=1;MAX_ED=30;MIN_ED=2;NLOD=3.94;TLOD=11.56	GT:AD:AF:ALT_F1R2:ALT_F2R1:FOXOG:PGT:PID:QSS:REF_F1R2:REF_F2R1	0/1:61,4:0.065:4:0:.:0|1:138125496_C_T:1738,46:30:31	0/0:42,2:0.047:2:0:.:0|1:138125496_C_T:1138,40:22:20	1.4	Deleterious	-17.28	Deleterious	164	30	NA	NA	NA	NA
T1,T3	chr5	**2**	**25**	138125502	138125502	-	TA	exonic	**Zscan21**	ENSMUSP00000018651	.	frameshift insertion	Zscan21:NM_001044703:exon2:c.442_443insTA:p.C148fs,Zscan21:NM_001044705:exon2:c.442_443insTA:p.C148fs,Zscan21:NM_001044704:exon3:c.442_443insTA:p.C148fs,Zscan21:NM_011757:exon3:c.442_443insTA:p.C148fs	**C148fs**	ENSMUSP00000018651,148,C,	5,138125502,T,C	5qG2	.	0.25	.	44	chr5	138125502	.	T	C	.	str_contraction;t_lod_fstar	ECNT=1;HCNT=2;MAX_ED=.;MIN_ED=.;NLOD=5.71;RPA=8,7;RU=CTG;STR;TLOD=4.67	GT:AD:AF:ALT_F1R2:ALT_F2R1:FOXOG:QSS:REF_F1R2:REF_F2R1	0/1:45,2:0.044:0:2:.:1261,57:18:27	0/0:21,0:0.00:0:0:.:580,0:17:4	14	Tolerated	-2.26	Neutral	268	30	0.381	Tolerated	2.83	236
T1,T3	chr5	**2**	**25**	138125507	138125511	GTGGC	-	exonic	**Zscan21**	ENSMUSP00000018651	.	frameshift deletion	Zscan21:NM_001044703:exon2:c.447_451del:p.E149fs,Zscan21:NM_001044705:exon2:c.447_451del:p.E149fs,Zscan21:NM_001044704:exon3:c.447_451del:p.E149fs,Zscan21:NM_011757:exon3:c.447_451del:p.E149fs	**E149fs**	ENSMUSP00000018651,149,E,.	5,138125507,AGTGGC,CGACCA	5qG2	.	0.25	.	46	chr5	138125506	.	AGTGGC	CGACCA	.	clustered_events;multi_event_alt_allele_in_normal	ECNT=9;HCNT=1;MAX_ED=30;MIN_ED=2;NLOD=3.34;TLOD=13.50	GT:AD:AF:ALT_F1R2:ALT_F2R1:FOXOG:PGT:PID:QSS:REF_F1R2:REF_F2R1	0/1:62,4:0.079:4:0:.:0|1:138125496_C_T:1727,44:30:32	0/0:40,2:0.049:2:0:.:0|1:138125496_C_T:1046,13:21:19	1.4	Unknown	.	.	.	.	.	.	.	.
T1df	chr1	**1**	**12.5**	71302724	71302724	T	C	exonic	**Abca12**	ENSMUSP00000115922	.	nonsynonymous SNV	Abca12:NM_175210:exon22:c.A3077G:p.E1026G	**E1026G**	ENSMUSP00000115922,1026,E,G	1,71302724,T,G	1qC3	.	0.25	.	39	chr1	71302724	.	T	G	.	str_contraction	ECNT=1;HCNT=1;MAX_ED=.;MIN_ED=.;NLOD=9.31;RPA=8,7;RU=CA;STR;TLOD=8.79	GT:AD:AF:ALT_F1R2:ALT_F2R1:FOXOG:QSS:REF_F1R2:REF_F2R1	0/1:45,5:0.104:3:2:.:1271,148:20:25	0/0:33,0:0.00:0:0:.:930,0:21:12	3	Deleterious	-5.19	Deleterious	171	30	0.002	Damaging	2.83	117
T1df	chr1	**1**	**12.5**	71302829	71302829	G	T	exonic	**Abca12**	ENSMUSP00000115922	.	nonsynonymous SNV	Abca12:NM_175210:exon22:c.C2972A:p.T991N	**T991N**	ENSMUSP00000115922,991,T,N	1,71302829,G,G	1qC3	.	0.25	.	46	chr1	71302829	.	G	G	.	str_contraction;t_lod_fstar	ECNT=1;HCNT=1;MAX_ED=.;MIN_ED=.;NLOD=14.92;RPA=6,5;RU=GCA;STR;TLOD=4.27	GT:AD:AF:ALT_F1R2:ALT_F2R1:FOXOG:QSS:REF_F1R2:REF_F2R1	0/1:40,3:0.050:0:3:.:968,78:20:20	0/0:61,1:0.019:1:0:.:1533,6:30:31	1	Tolerated	0	Neutral	171	30	0.607	Tolerated	2.83	117

T3df	chr11	**1**	**12.5**	110165931	110165931	-	GCCAGTTCTTGCAGAGAAGAGAGAACAGGCAGATGTGGAACCATACCAGGAGGGTCTCTCTTCTCATCCTGAACTTTCTGG	exonic	**Abca9**	ENSMUSP00000116142	.	nonframeshift insertion	Abca9:NM_147220:exon2:c.61_62insCCAGAAAGTTCAGGATGAGAAGAGAGACCCTCCTGGTATGGTTCCACATCTGCCTGTTCTCTCTTCTCTGCAAGAACTGGC:p.L21delinsPRKFRMRRETLLVWFHICLFSLLCKNWL	**L21delinsPRKFRMRRETLLVWFHICLFSLLCKNWL**	ENSMUSP00000116142,21,L,PRKFRMRRETLLVWFHICLFSLLCKNWL	11,110165931,A,A	11qE1	.	0.25	.	43	chr11	110165931	.	A	A	.	str_contraction;t_lod_fstar	ECNT=1;HCNT=8;MAX_ED=.;MIN_ED=.;NLOD=8.42;RPA=7,6;RU=CAG;STR;TLOD=4.61	GT:AD:AF:ALT_F1R2:ALT_F2R1:FOXOG:QSS:REF_F1R2:REF_F2R1	0/1:59,2:0.033:0:2:.:1662,57:28:31	0/0:31,0:0.00:0:0:.:872,0:20:11	18	Tolerated	0	Neutral	140	30	1	Tolerated	2.86	158
T32df	chr1	**1**	**12.5**	156641849	156641849	T	G	exonic	**Abl2**	ENSMUSP00000027888	.	nonsynonymous SNV	Abl2:NM_009595:exon12:c.T2682G:p.N894K,Abl2:NM_001136104:exon13:c.T2370G:p.N790K	**N790K**	ENSMUSP00000027888,790,N,K	1,156641849,T,A	1qG3	.	0.25	.	37	chr1	156641849	.	T	A	.	clustered_events;multi_event_alt_allele_in_normal;t_lod_fstar	ECNT=4;HCNT=1;MAX_ED=19;MIN_ED=1;NLOD=7.84;TLOD=5.90	GT:AD:AF:ALT_F1R2:ALT_F2R1:FOXOG:PGT:PID:QSS:REF_F1R2:REF_F2R1	0/1:26,2:0.074:2:0:.:0|1:131129021_T_TTTTA:610,17:14:12	0/0:44,1:0.024:1:0:.:0|1:131129021_T_TTTTA:1068,11:26:18	23	Damaging	-0.65	Neutral	182	30	0.008	Damaging	3.75	82
T32df	chr1	**1**	**12.5**	156641849	156641849	T	G	exonic	**Abl2**	ENSMUSP00000115111	.	nonsynonymous SNV	Abl2:NM_009595:exon12:c.T2682G:p.N894K,Abl2:NM_001136104:exon13:c.T2370G:p.N790K	**N894K**	ENSMUSP00000115111,894,N,K	1,156641849,T,G	1qG3	.	0.25	.	37	chr1	156641849	.	T	G	.	PASS	ECNT=1;HCNT=1;MAX_ED=.;MIN_ED=.;NLOD=18.65;TLOD=14.40	GT:AD:AF:ALT_F1R2:ALT_F2R1:FOXOG:QSS:REF_F1R2:REF_F2R1	0/1:19,7:0.250:3:4:0.571:523,204:8:11	0/0:72,0:0.00:0:0:.:1980,0:36:36	2.5	Damaging	-0.65	Neutral	182	30	0.008	Damaging	3.75	82
T32df	chr9	**1**	**12.5**	104078593	104078593	T	-	exonic	**Acad11**	ENSMUSP00000126181	.	frameshift deletion	Acad11:NM_175324:exon5:c.562delT:p.S188fs	**S188fs**	ENSMUSP00000126181,188,S,.	9,104078593,CT,T	9qF1	.	0.25	.	42	chr9	104078592	.	CT	G	.	alt_allele_in_normal;clustered_events;germline_risk	DB;ECNT=4;HCNT=2;MAX_ED=13;MIN_ED=1;NLOD=3.67;TLOD=18.03	GT:AD:AF:ALT_F1R2:ALT_F2R1:FOXOG:PGT:PID:QSS:REF_F1R2:REF_F2R1	0/1:68,6:0.083:5:1:0.167:0|1:37449667_A_G:1936,160:39:29	0/0:72,3:0.056:0:3:1.00:0|1:37449667_A_G:1976,81:39:33	0.7	Deleterious	-11.71	Deleterious	182	30	NA	NA	NA	NA
T2df	chr19	**1**	**12.5**	56723623	56723625	GAC	-	exonic	**Adrb1**	ENSMUSP00000143827	.	nonframeshift deletion	Adrb1:NM_007419:exon1:c.1252_1254del:p.418_418del	**418_418del**	NA	19,56723623,GGAC,A	19qD2	.	0.25	.	23	chr19	56723622	.	GGAC	A	.	clustered_events	DB;ECNT=2;HCNT=2;MAX_ED=37;MIN_ED=37;NLOD=13.19;TLOD=45.99	GT:AD:AF:ALT_F1R2:ALT_F2R1:FOXOG:QSS:REF_F1R2:REF_F2R1	0/1:35,21:0.375:17:4:0.810:990,588:18:17	0/0:49,0:0.00:0:0:.:1383,0:33:16	2.2	Unknown	.	.	.	.	.	.	.	.
T12df	chr11	**1**	**12.5**	5864043	5864108	AAGGAGAAGCCACCCAAGGCCACCAAGAAGCCCAAGGAGAAACCACCCAAGGCCACCAAGAAGCCC	-	exonic	**Aebp1**	ENSMUSP00000108033	.	nonframeshift deletion	Aebp1:NM_001291857:exon2:c.352_417del:p.118_139del,Aebp1:NM_009636:exon2:c.352_417del:p.118_139del	**118_139del**	NA	11,5864043,AAAGGAGAAGCCACCCAAGGCCACCAAGAAGCCCAAGGAGAAACCACCCAAGGCCACCAAGAAGCCC,A	11qA1	.	0.25	.	43	chr11	5864042	.	AAAGGAGAAGCCACCCAAGGCCACCAAGAAGCCCAAGGAGAAACCACCCAAGGCCACCAAGAAGCCC	A	.	PASS	DB;ECNT=1;HCNT=1;MAX_ED=.;MIN_ED=.;NLOD=10.48;TLOD=64.39	GT:AD:AF:ALT_F1R2:ALT_F2R1:FOXOG:QSS:REF_F1R2:REF_F2R1	0/1:27,26:0.490:14:12:0.538:763,719:20:7	0/0:38,0:0.00:0:0:.:1036,0:16:22	4	Unknown	.	.	.	.	.	.	.	.
T32df	chr5	**1**	**12.5**	35945186	35945186	A	T	splicing	**Afap1**	ENSMUSP00000021726	.	.	.	**NA**	5,35945186,A,G	NA	5qB3	.	0.25	.	41	chr5	35945186	.	A	G	.	PASS	ECNT=1;HCNT=1;MAX_ED=.;MIN_ED=.;NLOD=18.65;TLOD=14.40	GT:AD:AF:ALT_F1R2:ALT_F2R1:FOXOG:QSS:REF_F1R2:REF_F2R1	0/1:19,7:0.250:3:4:0.571:523,204:8:11	0/0:72,0:0.00:0:0:.:1980,0:36:36	2.5	Unkown	.	.	.	.	.	.	.	.
T42df	chr1	**1**	**12.5**	179752690	179752690	C	A	exonic	**Ahctf1**	ENSMUSP00000027768	.	nonsynonymous SNV	Ahctf1:NM_026375:exon33:c.G5946T:p.Q1982H	**Q1982H**	ENSMUSP00000027768,1982,Q,H	NA	1qH4	.	0.25	.	35	chr1	179752690	.	C	TAAAAA	.	clustered_events;multi_event_alt_allele_in_normal	ECNT=7;HCNT=1;MAX_ED=24;MIN_ED=2;NLOD=6.34;TLOD=8.78	GT:AD:AF:ALT_F1R2:ALT_F2R1:FOXOG:PGT:PID:QSS:REF_F1R2:REF_F2R1	0/1:39,3:0.070:0:3:.:0|1:179752666_C_CGGTGGT:1039,69:23:16	0/0:35,1:0.028:0:1:.:0|1:179752666_C_CGGTGGT:980,28:22:13	21	Damaging	-1.15	Neutral	75	30	0.008	Damaging	3.66	41
T42df	chr1	**1**	**12.5**	179752679	179752679	A	T	exonic	**Ahctf1**	ENSMUSP00000027768	.	nonsynonymous SNV	Ahctf1:NM_026375:exon33:c.T5957A:p.V1986D	**V1986D**	ENSMUSP00000027768,1986,V,D	1,179752679,A,G	1qH4	.	0.25	.	37	chr1	179752679	.	A	G	.	clustered_events;multi_event_alt_allele_in_normal	ECNT=7;HCNT=1;MAX_ED=24;MIN_ED=2;NLOD=6.94;TLOD=8.81	GT:AD:AF:ALT_F1R2:ALT_F2R1:FOXOG:PGT:PID:QSS:REF_F1R2:REF_F2R1	0/1:39,3:0.073:0:3:.:0|1:179752666_C_CGGTGGT:935,40:24:14	0/0:37,1:0.028:0:1:.:0|1:179752666_C_CGGTGGT:862,12:22:13	21	Damaging	-0.87	Neutral	75	30	0.018	Damaging	3.67	39
T42df	chr1	**1**	**12.5**	179752689	179752689	-	AAAAA	exonic	**Ahctf1**	ENSMUSP00000027768	.	frameshift insertion	Ahctf1:NM_026375:exon33:c.5946_5947insTTTTT:p.K1983fs	**K1983fs**	ENSMUSP00000027768,1983,K,	1,179752689,T,CGGTGGT	1qH4	.	0.25	.	36	chr1	179752689	.	T	CGGTGGT	.	clustered_events;multi_event_alt_allele_in_normal;t_lod_fstar	ECNT=7;HCNT=1;MAX_ED=24;MIN_ED=2;NLOD=6.33;TLOD=5.55	GT:AD:AF:ALT_F1R2:ALT_F2R1:FOXOG:PGT:PID:QSS:REF_F1R2:REF_F2R1	0/1:36,3:0.050:0:3:.:0|1:179752666_C_CGGTGGT:1019,87:23:13	0/0:37,1:0.028:0:1:.:0|1:179752666_C_CGGTGGT:1039,29:24:13	21	Neutral	-1.49	Neutral	75	30	NA	NA	NA	NA

T42df	chr1	**1**	**12.5**	179752677	179752677	G	T	exonic	**Ahctf1**	ENSMUSP00000067779	.	nonsynonymous SNV	Ahctf1:NM_026375:exon33:c.C5959A:p.P1987T	**P1987T**	ENSMUSP00000067779,1987,P,T	1,179752677,G,A	1qH4	.	0.25	.	37	chr1	179752677	.	G	A	.	clustered_events;multi_event_alt_allele_in_normal	ECNT=7;HCNT=1;MAX_ED=24;MIN_ED=2;NLOD=6.34;TLOD=8.78	GT:AD:AF:ALT_F1R2:ALT_F2R1:FOXOG:PGT:PID:QSS:REF_F1R2:REF_F2R1	0/1:39,3:0.070:0:3:1.00:0|1:179752666_C_CGGTGGT:1081,42:23:16	0/0:34,1:0.028:0:1:1.00:0|1:179752666_C_CGGTGGT:979,13:22:12	21	Tolerated	-0.42	Neutral	75	30	0.511	Tolerated	3.66	41
T42df	chr1	**1**	**12.5**	179752666	179752666	-	GGTGGT	exonic	**Ahctf1**	ENSMUSP00000027768	.	nonframeshift insertion	Ahctf1:NM_026375:exon33:c.5969_5970insACCACC:p.R1990delinsRPP	**R1990delinsRPP**	ENSMUSP00000027768,1990,R,RPP	NA	1qH4	.	0.25	.	38	chr1	179752666	.	C	G	.	t_lod_fstar	ECNT=1;HCNT=1;MAX_ED=.;MIN_ED=.;NLOD=10.18;TLOD=5.56	GT:AD:AF:ALT_F1R2:ALT_F2R1:FOXOG:QSS:REF_F1R2:REF_F2R1	0/1:18,3:0.150:0:3:0.00:520,78:4:14	0/0:37,0:0.00:0:0:.:1028,0:27:10	18	Unknown	-1.73	Neutral	75	30	NA	NA	NA	NA
T42df	chr1	**1**	**12.5**	179752669	179752673	GACAA	-	exonic	**Ahctf1**	ENSMUSP00000067779	.	frameshift deletion	Ahctf1:NM_026375:exon33:c.5963_5967del:p.V1988fs	**V1988fs**	ENSMUSP00000067779,1988,V,.	1,179752669,GGACAA,A	1qH4	.	0.25	.	38	chr1	179752668	.	GGACAA	A	.	clustered_events;multi_event_alt_allele_in_normal	ECNT=7;HCNT=1;MAX_ED=24;MIN_ED=2;NLOD=6.64;TLOD=8.74	GT:AD:AF:ALT_F1R2:ALT_F2R1:FOXOG:PGT:PID:QSS:REF_F1R2:REF_F2R1	0/1:42,3:0.068:0:3:.:0|1:179752666_C_CGGTGGT:1175,45:25:17	0/0:37,1:0.027:0:1:.:0|1:179752666_C_CGGTGGT:1041,22:22:15	21	Unknown	NA	NA	75	30	NA	NA	NA	NA
T42df	chr1	**1**	**12.5**	179752682	179752687	TCTTTC	-	exonic	**Ahctf1**	ENSMUSP00000148414	.	nonframeshift deletion	Ahctf1:NM_026375:exon33:c.5949_5954del:p.1983_1985del	**1983_1985del**	NA	1,179752682,ATCTTTC,A	1qH4	.	0.25	.	38	chr1	179752681	.	ATCTTTC	A	.	str_contraction;t_lod_fstar	ECNT=1;HCNT=1;MAX_ED=.;MIN_ED=.;NLOD=3.80;RPA=2,1;RU=AGCTGCCACCCCCAAAGCCACCACCGCCGT;STR;TLOD=5.69	GT:AD:AF:ALT_F1R2:ALT_F2R1:FOXOG:QSS:REF_F1R2:REF_F2R1	0/1:37,2:0.054:1:1:.:949,55:20:17	0/0:15,0:0.00:0:0:.:403,0:8:7	0.7	Unknown	.	.	.	.	.	.	.	.
T12df	chr18	**1**	**12.5**	36560931	36560933	GCA	-	exonic	**Ankhd1**	ENSMUSP00000112352	.	nonframeshift deletion	Ankhd1:NM_175375:exon1:c.194_196del:p.65_66del	**65_66del**	NA	18,36560931,GGCA,A	18qB2	.	0.25	.	62	chr18	36560930	.	GGCA	A	.	str_contraction;t_lod_fstar	ECNT=1;HCNT=8;MAX_ED=.;MIN_ED=.;NLOD=8.42;RPA=7,6;RU=CAG;STR;TLOD=4.61	GT:AD:AF:ALT_F1R2:ALT_F2R1:FOXOG:QSS:REF_F1R2:REF_F2R1	0/1:59,2:0.033:0:2:.:1662,57:28:31	0/0:31,0:0.00:0:0:.:872,0:20:11	18	Unknown	.	.	.	.	.	.	.	.
T42df	chr4	**1**	**12.5**	47016782	47016782	-	ACGGTTTCTTCCGGCCTTGTCCATCCGGCCATGGCTGTGTCGACGAGGCCAGCGGAACCACCAAAGCTGTGGCTACCTCCTGCTAGAT	exonic	**Anks6**	ENSMUSP00000117110	.	stopgain	Anks6:NM_001024136:exon15:c.2432_2433insATCTAGCAGGAGGTAGCCACAGCTTTGGTGGTTCCGCTGGCCTCGTCGACACAGCCATGGCCGGATGGACAAGGCCGGAAGAAACCGT:p.V811_S812delinsVSSRRX,Anks6:NM_001356426:exon16:c.2636_2637insATCTAGCAGGAGGTAGCCACAGCTTTGGTGGTTCCGCTGGCCTCGTCGACACAGCCATGGCCGGATGGACAAGGCCGGAAGAAACCGT:p.V879_S880delinsVSSRRX	**V811_S812delinsVSSRRX**	ENSMUSP00000117110,811,VS,VSSRRX	NA	4qB1	.	0.25	.	36	chr4	47016782	.	C	C	.	t_lod_fstar	ECNT=1;HCNT=3;MAX_ED=.;MIN_ED=.;NLOD=12.59;TLOD=5.98	GT:AD:AF:ALT_F1R2:ALT_F2R1:FOXOG:QSS:REF_F1R2:REF_F2R1	0/1:30,3:0.091:1:2:0.333:801,89:15:15	0/0:45,0:0.00:0:0:.:1237,0:20:25	0.9	Deletarious	.	.	.	.	.	.	.	.
T42df	chr4	**1**	**12.5**	47016782	47016782	-	ACGGTTTCTTCCGGCCTTGTCCATCCGGCCATGGCTGTGTCGACGAGGCCAGCGGAACCACCAAAGCTGTGGCTACCTCCTGCTAGAT	exonic	**Anks6**	ENSMUSP00000117110	.	stopgain	Anks6:NM_001024136:exon15:c.2432_2433insATCTAGCAGGAGGTAGCCACAGCTTTGGTGGTTCCGCTGGCCTCGTCGACACAGCCATGGCCGGATGGACAAGGCCGGAAGAAACCGT:p.V811_S812delinsVSSRRX,Anks6:NM_001356426:exon16:c.2636_2637insATCTAGCAGGAGGTAGCCACAGCTTTGGTGGTTCCGCTGGCCTCGTCGACACAGCCATGGCCGGATGGACAAGGCCGGAAGAAACCGT:p.V879_S880delinsVSSRRX	**V879_S880delinsVSSRRX**	ENSMUSP00000117110,879,VS,VSSRRX	NA	4qB1	.	0.25	.	36	chr4	47016782	.	C	A	.	t_lod_fstar	ECNT=1;HCNT=2;MAX_ED=.;MIN_ED=.;NLOD=10.23;TLOD=4.07	GT:AD:AF:ALT_F1R2:ALT_F2R1:FOXOG:QSS:REF_F1R2:REF_F2R1	0/1:25,1:0.071:1:0:1.00:717,16:13:12	0/0:38,0:0.00:0:0:.:1055,0:19:19	0.8	Deletarious	.	.	.	.	.	.	.	.
T42df	chr9	**1**	**12.5**	44752229	44752229	C	T	exonic	**Arcn1**	ENSMUSP00000032414	.	nonsynonymous SNV	Arcn1:NM_145985:exon6:c.G865A:p.D289N	**D289N**	ENSMUSP00000032414,289,D,N	9,44752229,C,A	9qA5.2	.	0.25	.	44	chr9	44752229	.	C	A	.	PASS	ECNT=1;HCNT=1;MAX_ED=.;MIN_ED=.;NLOD=8.29;TLOD=7.40	GT:AD:AF:ALT_F1R2:ALT_F2R1:FOXOG:QSS:REF_F1R2:REF_F2R1	0/1:50,4:0.077:0:4:0.00:1383,116:25:25	0/0:34,0:0.00:0:0:.:899,0:17:17	0.8	Deleterious	-8.05	Deleterious	116	30	0	Damaging	2.84	117

T1df	chr9	**1**	**12.5**	44743947	44743947	C	-	exonic	**Arcn1**	ENSMUSP00000032414	.	frameshift deletion	Arcn1:NM_145985:exon10:c.1516delG:p.D506fs	**D506fs**	ENSMUSP00000032414,506,D,.	9,44743947,TC,A	9qA5.2	.	0.25	.	38	chr9	44743946	.	TC	A	.	PASS	DB;ECNT=1;HCNT=1;MAX_ED=.;MIN_ED=.;NLOD=10.13;TLOD=95.88	GT:AD:AF:ALT_F1R2:ALT_F2R1:FOXOG:QSS:REF_F1R2:REF_F2R1	0/1:32,36:0.530:20:16:0.556:881,1021:14:18	0/0:36,0:0.00:0:0:.:906,0:16:20	2.7	Unknown	NA	NA	116	30	NA	NA	NA	NA
T12df	chr3	**1**	**12.5**	88639464	88639464	A	G	exonic	**Arhgef2**	ENSMUSP00000103134	.	nonsynonymous SNV	Arhgef2:NM_001198912:exon13:c.A1609G:p.I537V,Arhgef2:NM_001198913:exon13:c.A1603G:p.I535V,Arhgef2:NM_008487:exon13:c.A1690G:p.I564V,Arhgef2:NM_001198911:exon14:c.A1639G:p.I547V	**I537V**	ENSMUSP00000103134,537,I,V	NA	3qF1	.	0.25	.	46	chr3	88639464	.	A	G	.	PASS	ECNT=1;HCNT=1;MAX_ED=.;MIN_ED=.;NLOD=12.59;TLOD=6.94	GT:AD:AF:ALT_F1R2:ALT_F2R1:FOXOG:QSS:REF_F1R2:REF_F2R1	0/1:47,4:0.083:0:4:1.00:1331,110:24:23	0/0:46,0:0.00:0:0:.:1292,0:25:21	19	Damaging	-0.6	Neutral	134	30	0.037	Damaging	2.84	158
T12df	chr3	**1**	**12.5**	88639464	88639464	A	G	exonic	**Arhgef2**	ENSMUSP00000127843	.	nonsynonymous SNV	Arhgef2:NM_001198912:exon13:c.A1609G:p.I537V,Arhgef2:NM_001198913:exon13:c.A1603G:p.I535V,Arhgef2:NM_008487:exon13:c.A1690G:p.I564V,Arhgef2:NM_001198911:exon14:c.A1639G:p.I547V	**I535V**	ENSMUSP00000127843,535,I,V	NA	3qF1	.	0.25	.	46	chr3	88639464	.	A	G	.	PASS	ECNT=1;HCNT=1;MAX_ED=.;MIN_ED=.;NLOD=12.59;TLOD=6.94	GT:AD:AF:ALT_F1R2:ALT_F2R1:FOXOG:QSS:REF_F1R2:REF_F2R1	0/1:47,4:0.083:0:4:1.00:1331,110:24:23	0/0:46,0:0.00:0:0:.:1292,0:25:21	19	Damaging	-0.6	Neutral	134	30	0.045	Damaging	2.84	154
T12df	chr3	**1**	**12.5**	88639464	88639464	A	G	exonic	**Arhgef2**	ENSMUSP00000081665	.	nonsynonymous SNV	Arhgef2:NM_001198912:exon13:c.A1609G:p.I537V,Arhgef2:NM_001198913:exon13:c.A1603G:p.I535V,Arhgef2:NM_008487:exon13:c.A1690G:p.I564V,Arhgef2:NM_001198911:exon14:c.A1639G:p.I547V	**I547V**	ENSMUSP00000081665,547,I,V	3,88639464,A,T	3qF1	.	0.25	.	46	chr3	88639464	.	A	T	.	clustered_events;homologous_mapping_event;t_lod_fstar	ECNT=8;HCNT=1;MAX_ED=23;MIN_ED=2;NLOD=9.63;TLOD=5.04	GT:AD:AF:ALT_F1R2:ALT_F2R1:FOXOG:PGT:PID:QSS:REF_F1R2:REF_F2R1	0/1:71,2:0.028:0:2:0.00:0|1:45751093_C_CT:1905,32:45:26	0/0:33,0:0.00:0:0:.:0|1:45751093_C_CT:896,0:22:11	0.9	Deleterious	-2.69	Deleterious	179	30	0.006	Damaging	2.82	160
T12df	chr3	**1**	**12.5**	88639464	88639464	A	G	exonic	**Arhgef2**	ENSMUSP00000081665	.	nonsynonymous SNV	Arhgef2:NM_001198912:exon13:c.A1609G:p.I537V,Arhgef2:NM_001198913:exon13:c.A1603G:p.I535V,Arhgef2:NM_008487:exon13:c.A1690G:p.I564V,Arhgef2:NM_001198911:exon14:c.A1639G:p.I547V	**I564V**	ENSMUSP00000081665,564,I,V	3,88639464,A,T	3qF1	.	0.25	.	46	chr3	88639464	.	A	T	.	clustered_events;homologous_mapping_event;t_lod_fstar	ECNT=8;HCNT=1;MAX_ED=23;MIN_ED=2;NLOD=9.33;TLOD=5.04	GT:AD:AF:ALT_F1R2:ALT_F2R1:FOXOG:PGT:PID:QSS:REF_F1R2:REF_F2R1	0/1:71,2:0.028:0:2:0.00:0|1:45751093_C_CT:1891,54:45:26	0/0:33,0:0.00:0:0:.:0|1:45751093_C_CT:916,0:22:11	0.9	Deleterious	-2.69	Deleterious	179	30	0.006	Damaging	2.82	160
T4df	chr7	**1**	**12.5**	99594760	99594793	TGAGTGAGAGCCCGCCCTAAGACCCAGATGCTA	-	splicing	**Arrb1**	ENSMUSP00000072669	.	.	.	**NA**	7,99594760,GTGAGTGAGAGCCCGCCCCTAAGACCCAGATGCTA,T	NA	7qE1	.	0.25	.	21	chr7	99594759	.	GTGAGTGAGAGCCCGCCCCTAAGACCCAGATGCTA	T	.	clustered_events	DB;ECNT=2;HCNT=2;MAX_ED=13;MIN_ED=13;NLOD=12.24;TLOD=52.57	GT:AD:AF:ALT_F1R2:ALT_F2R1:FOXOG:QSS:REF_F1R2:REF_F2R1	0/1:25,21:0.455:12:9:0.571:728,591:11:14	0/0:47,0:0.00:0:0:.:1264,0:14:33	2.8	Unkown	.	.	.	.	.	.	.	.
T2df	chr7	**1**	**12.5**	102110703	102110703	T	A	exonic	**Art1**	ENSMUSP00000032995	.	nonsynonymous SNV	Art1:NM_009710:exon5:c.T880A:p.S294T	**S294T**	ENSMUSP00000032995,294,S,T	7,102110703,T,A	7qE2	.	0.25	.	34	chr7	102110703	.	T	A	.	str_contraction;t_lod_fstar	ECNT=1;HCNT=1;MAX_ED=.;MIN_ED=.;NLOD=5.10;RPA=9,8;RU=GCC;STR;TLOD=4.48	GT:AD:AF:ALT_F1R2:ALT_F2R1:FOXOG:QSS:REF_F1R2:REF_F2R1	0/1:29,2:0.069:1:1:.:819,56:16:13	0/0:21,0:0.00:0:0:.:594,0:15:6	20	Tolerated	-0.1	Neutral	122	30	0.48	Tolerated	3.28	25
T12df	chr2	**1**	**12.5**	73873669	73873669	C	T	splicing	**Atf2**	ENSMUSP00000033300	.	.	.	**NA**	2,73873669,C,G	NA	2qC3	.	0.25	.	56	chr2	73873669	.	C	G	.	PASS	ECNT=1;HCNT=1;MAX_ED=.;MIN_ED=.;NLOD=18.65;TLOD=14.40	GT:AD:AF:ALT_F1R2:ALT_F2R1:FOXOG:QSS:REF_F1R2:REF_F2R1	0/1:19,7:0.250:3:4:0.571:523,204:8:11	0/0:72,0:0.00:0:0:.:1980,0:36:36	2.5	Unkown	.	.	.	.	.	.	.	.
T22df	chr1	**1**	**12.5**	75184496	75184496	-	C	exonic	**Atg9a**	ENSMUSP00000107647	.	frameshift insertion	Atg9a:NM_001003917:exon13:c.2104dupG:p.A702fs,Atg9a:NM_001288612:exon13:c.2104dupG:p.A702fs,Atg9a:NM_001288613:exon13:c.1450dupG:p.A484fs	**A484fs**	ENSMUSP00000107647,484,A,	1,75184496,G,T	1qC4	.	0.25	.	35	chr1	75184496	.	G	T	.	clustered_events;homologous_mapping_event;t_lod_fstar	ECNT=9;HCNT=1;MAX_ED=18;MIN_ED=2;NLOD=10.49;TLOD=5.47	GT:AD:AF:ALT_F1R2:ALT_F2R1:FOXOG:PGT:PID:QSS:REF_F1R2:REF_F2R1	0/1:41,2:0.045:0:2:.:0|1:75184494_A_T:1118,35:22:19	0/0:36,0:0.00:0:0:.:0|1:75184494_A_T:971,0:16:20	0.4	Damaging	-1.93	Neutral	127	30	0.008	Damaging	3.06	83
T22df	chr1	**1**	**12.5**	75184512	75184512	C	A	exonic	**Atg9a**	ENSMUSP00000107648	.	nonsynonymous SNV	Atg9a:NM_001003917:exon13:c.G2089T:p.V697L,Atg9a:NM_001288612:exon13:c.G2089T:p.V697L,Atg9a:NM_001288613:exon13:c.G1435T:p.V479L	**S485T**	ENSMUSP00000107648,485,S,T	NA	1qC4	.	0.25	.	34	chr1	75184512	.	C	A	.	clustered_events;homologous_mapping_event;t_lod_fstar	ECNT=9;HCNT=1;MAX_ED=18;MIN_ED=2;NLOD=10.19;TLOD=5.47	GT:AD:AF:ALT_F1R2:ALT_F2R1:FOXOG:PGT:PID:QSS:REF_F1R2:REF_F2R1	0/1:41,2:0.045:0:2:.:0|1:75184494_A_T:970,24:23:18	0/0:35,0:0.00:0:0:.:0|1:75184494_A_T:891,0:16:19	0.4	Damaging	-0.22	Neutral	235	30	0	Damaging	4.02	49

T22df	chr1	**1**	**12.5**	75184499	75184499	-	C	exonic	**Atg9a**	ENSMUSP00000107647	.	stopgain	Atg9a:NM_001003917:exon13:c.2101_2102insG:p.Y701_A702delinsX,Atg9a:NM_001288612:exon13:c.2101_2102insG:p.Y701_A702delinsX,Atg9a:NM_001288613:exon13:c.1447_1448insG:p.Y483_A484delinsX	**Y483_A484delinsX**	ENSMUSP00000107647,483,YA,X	NA	1qC4	.	0.25	.	35	chr1	75184499	.	T	A	.	clustered_events;homologous_mapping_event;t_lod_fstar	ECNT=9;HCNT=1;MAX_ED=18;MIN_ED=2;NLOD=9.93;TLOD=5.49	GT:AD:AF:ALT_F1R2:ALT_F2R1:FOXOG:PGT:PID:QSS:REF_F1R2:REF_F2R1	0/1:42,2:0.047:0:2:1.00:0|1:75184494_A_T:1010,24:24:18	0/0:34,0:0.00:0:0:.:0|1:75184494_A_T:882,0:16:18	0.4	Deletarious	.	.	.	.	.	.	.	.
T22df	chr1	**1**	**12.5**	75184499	75184499	-	C	exonic	**Atg9a**	ENSMUSP00000107647	.	stopgain	Atg9a:NM_001003917:exon13:c.2101_2102insG:p.Y701_A702delinsX,Atg9a:NM_001288612:exon13:c.2101_2102insG:p.Y701_A702delinsX,Atg9a:NM_001288613:exon13:c.1447_1448insG:p.Y483_A484delinsX	**Y701_A702delinsX**	ENSMUSP00000107647,701,YA,X	NA	1qC4	.	0.25	.	35	chr1	75184499	.	T	T	.	clustered_events;multi_event_alt_allele_in_normal;t_lod_fstar	ECNT=9;HCNT=2;MAX_ED=18;MIN_ED=2;NLOD=8.75;TLOD=5.58	GT:AD:AF:ALT_F1R2:ALT_F2R1:FOXOG:PGT:PID:QSS:REF_F1R2:REF_F2R1	0/1:38,2:0.050:0:2:1.00:0|1:75184494_A_T:922,21:19:19	0/0:34,1:0.030:0:1:1.00:0|1:75184494_A_T:898,12:15:19	0.4	Deletarious	.	.	.	.	.	.	.	.
T22df	chr1	**1**	**12.5**	75184494	75184494	A	T	exonic	**Atg9a**	ENSMUSP00000107647	.	nonsynonymous SNV	Atg9a:NM_001003917:exon13:c.T2107A:p.S703T,Atg9a:NM_001288612:exon13:c.T2107A:p.S703T,Atg9a:NM_001288613:exon13:c.T1453A:p.S485T	**S703T**	ENSMUSP00000107647,703,S,T	1,75184494,A,A	1qC4	.	0.25	.	35	chr1	75184494	.	A	A	.	clustered_events;homologous_mapping_event;t_lod_fstar	ECNT=9;HCNT=1;MAX_ED=18;MIN_ED=2;NLOD=9.93;TLOD=5.49	GT:AD:AF:ALT_F1R2:ALT_F2R1:FOXOG:PGT:PID:QSS:REF_F1R2:REF_F2R1	0/1:42,2:0.047:0:2:1.00:0|1:75184494_A_T:1010,24:24:18	0/0:34,0:0.00:0:0:.:0|1:75184494_A_T:882,0:16:18	0.4	Tolerated	0	Neutral	127	30	1	Tolerated	3.06	83
T22df	chr1	**1**	**12.5**	75184512	75184512	C	A	exonic	**Atg9a**	ENSMUSP00000107647	.	nonsynonymous SNV	Atg9a:NM_001003917:exon13:c.G2089T:p.V697L,Atg9a:NM_001288612:exon13:c.G2089T:p.V697L,Atg9a:NM_001288613:exon13:c.G1435T:p.V479L	**V479L**	ENSMUSP00000107647,479,V,L	1,75184512,C,A	1qC4	.	0.25	.	34	chr1	75184512	.	C	A	.	clustered_events;homologous_mapping_event;t_lod_fstar	ECNT=9;HCNT=1;MAX_ED=18;MIN_ED=2;NLOD=10.19;TLOD=5.47	GT:AD:AF:ALT_F1R2:ALT_F2R1:FOXOG:PGT:PID:QSS:REF_F1R2:REF_F2R1	0/1:41,2:0.045:0:2:.:0|1:75184494_A_T:970,24:23:18	0/0:35,0:0.00:0:0:.:0|1:75184494_A_T:891,0:16:19	0.4	Tolerated	-0.08	Neutral	127	30	0.509	Tolerated	3.08	70
T22df	chr1	**1**	**12.5**	75184512	75184512	C	A	exonic	**Atg9a**	ENSMUSP00000107647	.	nonsynonymous SNV	Atg9a:NM_001003917:exon13:c.G2089T:p.V697L,Atg9a:NM_001288612:exon13:c.G2089T:p.V697L,Atg9a:NM_001288613:exon13:c.G1435T:p.V479L	**V697L**	ENSMUSP00000107647,697,V,L	1,75184512,C,A	1qC4	.	0.25	.	34	chr1	75184512	.	C	A	.	clustered_events;homologous_mapping_event;t_lod_fstar	ECNT=9;HCNT=1;MAX_ED=18;MIN_ED=2;NLOD=9.93;TLOD=5.49	GT:AD:AF:ALT_F1R2:ALT_F2R1:FOXOG:PGT:PID:QSS:REF_F1R2:REF_F2R1	0/1:42,2:0.047:0:2:1.00:0|1:75184494_A_T:1010,24:24:18	0/0:34,0:0.00:0:0:.:0|1:75184494_A_T:882,0:16:18	0.4	Tolerated	-0.08	Neutral	127	30	0.509	Tolerated	3.08	70
T22df	chr1	**1**	**12.5**	75184503	75184505	CGG	-	exonic	**Atg9a**	ENSMUSP00000107647	.	nonframeshift deletion	Atg9a:NM_001003917:exon13:c.2096_2098del:p.699_700del,Atg9a:NM_001288612:exon13:c.2096_2098del:p.699_700del,Atg9a:NM_001288613:exon13:c.1442_1444del:p.481_482del	**481_482del**	NA	1,75184503,TCGG,TC	1qC4	.	0.25	.	36	chr1	75184502	.	TCGG	TC	.	clustered_events;homologous_mapping_event;t_lod_fstar	ECNT=9;HCNT=1;MAX_ED=18;MIN_ED=2;NLOD=9.89;TLOD=5.49	GT:AD:AF:ALT_F1R2:ALT_F2R1:FOXOG:PGT:PID:QSS:REF_F1R2:REF_F2R1	0/1:41,2:0.047:0:2:.:0|1:75184494_A_T:937,56:22:19	0/0:35,0:0.00:0:0:.:0|1:75184494_A_T:851,0:15:20	0.4	Unknown	NA	NA	127	30	NA	NA	NA	NA
T22df	chr1	**1**	**12.5**	75184503	75184505	CGG	-	exonic	**Atg9a**	ENSMUSP00000107647	.	nonframeshift deletion	Atg9a:NM_001003917:exon13:c.2096_2098del:p.699_700del,Atg9a:NM_001288612:exon13:c.2096_2098del:p.699_700del,Atg9a:NM_001288613:exon13:c.1442_1444del:p.481_482del	**699_700del**	NA	1,75184503,TCGG,TC	1qC4	.	0.25	.	36	chr1	75184502	.	TCGG	TC	.	clustered_events;homologous_mapping_event;t_lod_fstar	ECNT=9;HCNT=1;MAX_ED=18;MIN_ED=2;NLOD=9.89;TLOD=5.49	GT:AD:AF:ALT_F1R2:ALT_F2R1:FOXOG:PGT:PID:QSS:REF_F1R2:REF_F2R1	0/1:41,2:0.047:0:2:.:0|1:75184494_A_T:937,56:22:19	0/0:35,0:0.00:0:0:.:0|1:75184494_A_T:851,0:15:20	0.4	Unknown	NA	NA	127	30	NA	NA	NA	NA
T22df	chr1	**1**	**12.5**	75184496	75184496	-	C	exonic	**Atg9a**	ENSMUSP00000107647	.	frameshift insertion	Atg9a:NM_001003917:exon13:c.2104dupG:p.A702fs,Atg9a:NM_001288612:exon13:c.2104dupG:p.A702fs,Atg9a:NM_001288613:exon13:c.1450dupG:p.A484fs	**A702fs**	ENSMUSP00000107647,702,A,	1,75184496,G,GC	1qC4	.	0.25	.	35	chr1	75184496	.	G	GC	.	clustered_events;homologous_mapping_event;t_lod_fstar	ECNT=9;HCNT=1;MAX_ED=18;MIN_ED=2;NLOD=10.19;RPA=1,2;RU=C;STR;TLOD=5.51	GT:AD:AF:ALT_F1R2:ALT_F2R1:FOXOG:PGT:PID:QSS:REF_F1R2:REF_F2R1	0/1:39,2:0.048:0:2:.:0|1:75184494_A_T:954,43:20:19	0/0:35,0:0.00:0:0:.:0|1:75184494_A_T:842,0:15:20	0.4	Unknown	NA	NA	127	30	NA	NA	NA	NA
T22df	chr1	**1**	**12.5**	75184507	75184507	C	-	exonic	**Atg9a**	ENSMUSP00000107647	.	frameshift deletion	Atg9a:NM_001003917:exon13:c.2094delG:p.L698fs,Atg9a:NM_001288612:exon13:c.2094delG:p.L698fs,Atg9a:NM_001288613:exon13:c.1440delG:p.L480fs	**L480fs**	ENSMUSP00000107647,480,L,.	1,75184507,AC,GAA	1qC4	.	0.25	.	35	chr1	75184506	.	AC	GAA	.	clustered_events;homologous_mapping_event;t_lod_fstar	ECNT=9;HCNT=1;MAX_ED=18;MIN_ED=2;NLOD=9.89;TLOD=5.49	GT:AD:AF:ALT_F1R2:ALT_F2R1:FOXOG:PGT:PID:QSS:REF_F1R2:REF_F2R1	0/1:40,2:0.047:0:2:.:0|1:75184494_A_T:773,33:21:19	0/0:35,0:0.00:0:0:.:0|1:75184494_A_T:737,0:15:20	0.4	Unknown	NA	NA	127	30	NA	NA	NA	NA
T22df	chr1	**1**	**12.5**	75184507	75184507	C	-	exonic	**Atg9a**	ENSMUSP00000107647	.	frameshift deletion	Atg9a:NM_001003917:exon13:c.2094delG:p.L698fs,Atg9a:NM_001288612:exon13:c.2094delG:p.L698fs,Atg9a:NM_001288613:exon13:c.1440delG:p.L480fs	**L698fs**	ENSMUSP00000107647,698,L,.	1,75184507,AC,GC	1qC4	.	0.25	.	35	chr1	75184506	.	AC	GC	.	clustered_events;homologous_mapping_event;t_lod_fstar	ECNT=9;HCNT=1;MAX_ED=18;MIN_ED=2;NLOD=10.19;RPA=1,2;RU=C;STR;TLOD=5.51	GT:AD:AF:ALT_F1R2:ALT_F2R1:FOXOG:PGT:PID:QSS:REF_F1R2:REF_F2R1	0/1:39,2:0.048:0:2:.:0|1:75184494_A_T:954,43:20:19	0/0:35,0:0.00:0:0:.:0|1:75184494_A_T:842,0:15:20	0.4	Unknown	.	.	.	.	.	.	.	.

T22df	chr1	**1**	**12.5**	75184498	75184498	-	AA	exonic	**Atg9a**	ENSMUSP00000107647	.	frameshift insertion	Atg9a:NM_001003917:exon13:c.2102_2103insTT:p.Y701fs,Atg9a:NM_001288612:exon13:c.2102_2103insTT:p.Y701fs,Atg9a:NM_001288613:exon13:c.1448_1449insTT:p.Y483fs	**Y483fs**	ENSMUSP00000107647,483,Y,	1,75184498,G,T	1qC4	.	0.25	.	35	chr1	75184498	.	G	T	.	clustered_events;homologous_mapping_event;t_lod_fstar	ECNT=9;HCNT=1;MAX_ED=18;MIN_ED=2;NLOD=10.49;TLOD=5.47	GT:AD:AF:ALT_F1R2:ALT_F2R1:FOXOG:PGT:PID:QSS:REF_F1R2:REF_F2R1	0/1:41,2:0.045:0:2:.:.:0|1:75184494_A_T:1118,35:22:19	0/0:36,0:0.00:0:0:.:.:0|1:75184494_A_T:971,016:20	0.4	Unknown	NA	NA	127	30	NA	NA	NA	NA
T22df	chr1	**1**	**12.5**	75184498	75184498	-	AA	exonic	**Atg9a**	ENSMUSP00000107647	.	frameshift insertion	Atg9a:NM_001003917:exon13:c.2102_2103insTT:p.Y701fs,Atg9a:NM_001288612:exon13:c.2102_2103insTT:p.Y701fs,Atg9a:NM_001288613:exon13:c.1448_1449insTT:p.Y483fs	**Y701fs**	ENSMUSP00000107647,701,Y,	1,75184498,G,GAA	1qC4	.	0.25	.	35	chr1	75184498	.	G	GAA	.	clustered_events;homologous_mapping_event;t_lod_fstar	ECNT=9;HCNT=1;MAX_ED=18;MIN_ED=2;NLOD=9.89;TLOD=5.49	GT:AD:AF:ALT_F1R2:ALT_F2R1:FOXOG:PGT:PID:QSS:REF_F1R2:REF_F2R1	0/1:40,2:0.047:0:2:.:.:0|1:75184494_A_T:773,33:21:19	0/0:35,0:0.00:0:0:.:.:0|1:75184494_A_T:737,015:20	0.4	Unknown	NA	NA	127	30	NA	NA	NA	NA
T3df	chr7	**1**	**12.5**	126494249	126494251	CAG	-	exonic	**Atxn2l**	ENSMUSP00000098606	.	nonframeshift deletion	Atxn2l:NM_001347658:exon18:c.2075_2077del:p.692_693del,Atxn2l:NM_183020:exon18:c.2333_2335del:p.778_779del	**778_779del**	NA	7,126494249,CCAG,A	7qF3	.	0.25	.	36	chr7	126494248	.	CCAG	A	.	PASS	DB;ECNT=1;HCNT=1;MAX_ED=.;MIN_ED=.;NLOD=9.57;TLOD=31.19	GT:AD:AF:ALT_F1R2:ALT_F2R1:FOXOG:QSS:REF_F1R2:REF_F2R1	0/1:19,12:0.400:7:5:0.417:505,342:12:7	0/0:34,0:0.00:0:0:.:854,0:11:23	2.1	Unknown	.	.	.	.	.	.	.	.
T3df	chr7	**1**	**12.5**	126494249	126494251	CAG	-	exonic	**Atxn2l**	ENSMUSP00000151571	.	nonframeshift deletion	Atxn2l:NM_001347658:exon18:c.2075_2077del:p.692_693del,Atxn2l:NM_183020:exon18:c.2333_2335del:p.778_779del	**692_693del**	NA	7,126494249,CCAG,A	7qF3	.	0.25	.	36	chr7	126494248	.	CCAG	A	.	alt_allele_in_normal;germline_risk	ECNT=1;HCNT=4;MAX_ED=.;MIN_ED=.;NLOD=0.932;TLOD=26.93	GT:AD:AF:ALT_F1R2:ALT_F2R1:FOXOG:QSS:REF_F1R2:REF_F2R1	0/1:47,9:0.161:6:3:0.667:1318,243:23:24	0/0:37,4:0.114:2:2:0.500:1054,113:19:18	2.4	Unknown	.	.	.	.	.	.	.	.
T2df	chr7	**1**	**12.5**	6997642	6997642	G	A	exonic	**Aurkc**	ENSMUSP00000125881	.	nonsynonymous SNV	Aurkc:NM_001080965:exon4:c.G461A:p.R154H,Aurkc:NM_001080966:exon4:c.G344A:p.R115H,Aurkc:NM_020572:exon4:c.G344A:p.R115H	**D112A**	ENSMUSP00000125881,112,D,A	7,6997642,G,A	7qA1	.	0.25	.	41	chr7	6997642	.	G	A	.	str_contraction;t_lod_fstar	ECNT=1;HCNT=8;MAX_ED=.;MIN_ED=.;NLOD=8.42;RPA=7,6;RU=CAG;STR;TLOD=4.61	GT:AD:AF:ALT_F1R2:ALT_F2R1:FOXOG:QSS:REF_F1R2:REF_F2R1	0/1:59,2:0.033:0:2:.:1662,57:28:31	0/0:31,0:0.00:0:0:.:872,0:20:11	18	Deleterious	-4.1	Deleterious	227	30	0.027	Damaging	3.04	394
T2df	chr7	**1**	**12.5**	6997642	6997642	G	A	exonic	**Aurkc**	ENSMUSP00000125881	.	nonsynonymous SNV	Aurkc:NM_001080965:exon4:c.G461A:p.R154H,Aurkc:NM_001080966:exon4:c.G344A:p.R115H,Aurkc:NM_020572:exon4:c.G344A:p.R115H	**R115H**	ENSMUSP00000125881,115,R,H	7,6997642,G,A	7qA1	.	0.25	.	41	chr7	6997642	.	G	A	.	str_contraction;t_lod_fstar	ECNT=1;HCNT=8;MAX_ED=.;MIN_ED=.;NLOD=8.42;RPA=7,6;RU=CAG;STR;TLOD=4.61	GT:AD:AF:ALT_F1R2:ALT_F2R1:FOXOG:QSS:REF_F1R2:REF_F2R1	0/1:59,2:0.033:0:2:.:1662,57:28:31	0/0:31,0:0.00:0:0:.:872,0:20:11	18	Deleterious	-4.1	Deleterious	227	30	0.027	Damaging	3.04	394
T2df	chr7	**1**	**12.5**	6997642	6997642	G	A	exonic	**Aurkc**	ENSMUSP00000035415	.	nonsynonymous SNV	Aurkc:NM_001080965:exon4:c.G461A:p.R154H,Aurkc:NM_001080966:exon4:c.G344A:p.R115H,Aurkc:NM_020572:exon4:c.G344A:p.R115H	**R154H**	ENSMUSP00000035415,154,R,H	7,6997642,G,A	7qA1	.	0.25	.	41	chr7	6997642	.	G	A	.	alt_allele_in_normal;clustered_events;germline_risk	ECNT=2;HCNT=22;MAX_ED=9;MIN_ED=9;NLOD=0.627;TLOD=53.26	GT:AD:AF:ALT_F1R2:ALT_F2R1:FOXOG:PGT:PID:QSS:REF_F1R2:REF_F2R1	0/1:54,16:0.239:12:4:0.750:0|1:6997633_A_C:1338,452:25:29	0/0:35,6:0.118:3:3:0.500:0|1:6997633_A_C:890,166:18:17	2.4	Deleterious	-4.1	Deleterious	227	30	0.027	Damaging	3.04	394
T2df	chr7	**1**	**12.5**	6997633	6997633	A	C	exonic	**Aurkc**	ENSMUSP00000132959	.	nonsynonymous SNV	Aurkc:NM_001080965:exon4:c.A452C:p.D151A,Aurkc:NM_001080966:exon4:c.A335C:p.D112A,Aurkc:NM_020572:exon4:c.A335C:p.D112A	**D151A**	ENSMUSP00000132959,151,D,A	NA	7qA1	.	0.25	.	44	chr7	6997633	.	A	A	.	alt_allele_in_normal;clustered_events;germline_risk	ECNT=2;HCNT=22;MAX_ED=9;MIN_ED=9;NLOD=0.627;TLOD=53.26	GT:AD:AF:ALT_F1R2:ALT_F2R1:FOXOG:PGT:PID:QSS:REF_F1R2:REF_F2R1	0/1:54,16:0.239:12:4:0.750:0|1:6997633_A_C:1338,452:25:29	0/0:35,6:0.118:3:3:0.500:0|1:6997633_A_C:890,166:18:17	2.4	Tolerated	-2.37	Neutral	182	30	0.106	Tolerated	2.93	277
T2df	chr2	**1**	**12.5**	152767333	152767371	GGCCTTGAACTTCCGATCCTTCTGTTTCCACCTCCCAAA	-	exonic	**Bcl2l1**	ENSMUSP00000145768	.	nonframeshift deletion	Bcl2l1:NM_001289739:exon3:c.653_691del:p.218_231del	**218_231del**	NA	2,152767333,GGGCCTTGAACTTCCGATCCTTCTGTTTCCACCTCCCAAA,A	2qH1	.	0.25	.	60	chr2	152767332	.	GGGCCTTGAACTTCCGATCCTTCTGTTTCCACCTCCCAAA	A	.	t_lod_fstar	ECNT=2;HCNT=1;MAX_ED=1;MIN_ED=1;NLOD=20.17;TLOD=5.29	GT:AD:AF:ALT_F1R2:ALT_F2R1:FOXOG:PGT:PID:QSS:REF_F1R2:REF_F2R1	0/1:55,2:0.037:2:0:0.00:0|1:121559024_C_A:1517,56:30:25	0/0:71,0:0.00:0:0:.:0|1:121559024_C_A:1893,0:33:38	2	Unkown	.	.	.	.	.	.	.	.
T42df	chr1	**1**	**12.5**	185346674	185346674	G	C	splicing	**Bpnt1**	.	NM_011794:exon5:c.334-1G>C;NM_001347210:exon5:c.334-1G>C;NM_001347212:exon4:c.169-1G>C	.	.	**.**	.	.	1qH5	.	0.25	.	52	chr1	185346674	.	G	C	.	PASS	ECNT=1;HCNT=2;MAX_ED=.;MIN_ED=.;NLOD=11.39;TLOD=18.54	GT:AD:AF:ALT_F1R2:ALT_F2R1:FOXOG:QSS:REF_F1R2:REF_F2R1	0/1:28,9:0.250:1:8:0.111:766,234:15:13	0/0:51,1:0.022:1:0:1.00:1384,18:30:21	21	Unknown	.	.	.	.	.	.	.	.

T1df	chr11	**1**	**12.5**	107055908	107055910	TGC	-	exonic	**Bptf**	ENSMUSP00000027916	.	nonframeshift deletion	Bptf:NM_176850:exon20:c.6710_6712del:p.2237_2238del	**2237_2238del**	NA	11,107055908,TTGC,ATACG	11qE1	.	0.25	.	36	chr11	107055907	.	TTGC	ATACG	.	clustered_events;multi_event_alt_allele_in_normal;t_lod_fstar	ECNT=4;HCNT=1;MAX_ED=19;MIN_ED=1;NLOD=6.94;TLOD=5.90	GT:AD:AF:ALT_F1R2:ALT_F2R1:FOXOG:PGT:PID:QSS:REF_F1R2:REF_F2R1	0/1:26,2:0.074:2:0:.:0|1:131129021_T_TTTTA:676,12:14:12	0/0:41,1:0.026:1:0:.:0|1:131129021_T_TTTTA:1063,13:24:17	23	Unknown	NA	NA	65	30	NA	NA	NA	NA
T2df	chr17	**1**	**12.5**	34116265	34116265	T	C	exonic	**Brd2**	ENSMUSP00000117122	.	nonsynonymous SNV	Brd2:NM_001204973:exon4:c.A401G:p.Y134C,Brd2:NM_010238:exon4:c.A401G:p.Y134C	**Y134C**	ENSMUSP00000117122,134,Y,C	17,34116265,T,A	17qB1	.	0.25	.	45	chr17	34116265	.	T	A	.	t_lod_fstar	ECNT=1;HCNT=2;MAX_ED=.;MIN_ED=.;NLOD=10.23;TLOD=4.07	GT:AD:AF:ALT_F1R2:ALT_F2R1:FOXOG:QSS:REF_F1R2:REF_F2R1	0/1:25,1:0.071:1:0:1.00:717,16:13:12	0/0:38,0:0.00:0:0:.:1055,0:19:19	0.8	Deleterious	-3.8	Deleterious	247	30	0.008	Damaging	2.85	299
T12df	chr17	**1**	**12.5**	32198151	32198153	CTG	-	exonic	**Brd4**	ENSMUSP00000092990	.	nonframeshift deletion	Brd4:NM_001286630:exon19:c.4015_4017del:p.1339_1339del,Brd4:NM_020508:exon19:c.4012_4014del:p.1338_1338del	**1338_1338del**	NA	17,32198151,CCTG,AT	17qB1	.	0.25	.	24	chr17	32198150	.	CCTG	AT	.	t_lod_fstar	ECNT=1;HCNT=1;MAX_ED=.;MIN_ED=.;NLOD=9.03;TLOD=4.11	GT:AD:AF:ALT_F1R2:ALT_F2R1:FOXOG:QSS:REF_F1R2:REF_F2R1	0/1:46,2:0.043:2:0:.:1334,56:29:17	0/0:33,0:0.00:0:0:.:926,0:18:15	0.8	Unknown	NA	NA	260	30	NA	NA	NA	NA
T12df	chr17	**1**	**12.5**	32198151	32198153	CTG	-	exonic	**Brd4**	ENSMUSP00000109880	.	nonframeshift deletion	Brd4:NM_001286630:exon19:c.4015_4017del:p.1339_1339del,Brd4:NM_020508:exon19:c.4012_4014del:p.1338_1338del	**1339_1339del**	NA	17,32198151,CCTG,GA	17qB1	.	0.25	.	24	chr17	32198150	.	CCTG	GA	.	t_lod_fstar	ECNT=1;HCNT=2;MAX_ED=.;MIN_ED=.;NLOD=9.04;TLOD=4.44	GT:AD:AF:ALT_F1R2:ALT_F2R1:FOXOG:QSS:REF_F1R2:REF_F2R1	0/1:39,2:0.050:0:2:.:1137,58:15:24	0/0:37,0:0.00:0:0:.:1079,0:20:17	0.3	Unknown	NA	NA	260	30	NA	NA	NA	NA
T22df	chr16	**1**	**12.5**	96058733	96058733	A	G	exonic	**Brwd1**	ENSMUSP00000115163	.	nonsynonymous SNV	Brwd1:NM_001103179:exon11:c.T1054C:p.F352L,Brwd1:NM_145125:exon11:c.T1054C:p.F352L	**F352L**	ENSMUSP00000115163,352,F,L	16,96058733,A,T	16qC4	.	0.25	.	72	chr16	96058733	.	A	T	.	clustered_events;homologous_mapping_event;t_lod_fstar	ECNT=8;HCNT=2;MAX_ED=15;MIN_ED=2;NLOD=10.23;TLOD=5.24	GT:AD:AF:ALT_F1R2:ALT_F2R1:FOXOG:PGT:PID:QSS:REF_F1R2:REF_F2R1	0/1:56,2:0.035:2:0:1.00:0|1:118057025_T_TA:1524,56:31:25	0/0:37,0:0.00:0:0:.:0|1:118057025_T_TA:974,0:28:9	6	Deleterious	-4.07	Deleterious	180	30	0.038	Damaging	2.85	216
T12df	chr14	**1**	**12.5**	31662296	31662298	GAA	-	exonic	**Btd**	ENSMUSP00000145044	.	nonframeshift deletion	Btd:NM_025295:exon2:c.238_240del:p.80_80del	**80_80del**	NA	14,31662296,TGAA,C	14qB	.	0.25	.	54	chr14	31662295	.	TGAA	C	.	str_contraction;t_lod_fstar	ECNT=1;HCNT=1;MAX_ED=.;MIN_ED=.;NLOD=9.61;RPA=6,5;RU=CAG;STR;TLOD=5.41	GT:AD:AF:ALT_F1R2:ALT_F2R1:FOXOG:QSS:REF_F1R2:REF_F2R1	0/1:47,2:0.043:2:0:.:1303,56:23:24	0/0:36,0:0.00:0:0:.:985,0:20:16	6	Unknown	.	.	.	.	.	.	.	.
T32df	chr11	**1**	**12.5**	49170604	49170604	-	C	exonic	**Btnl9**	ENSMUSP00000109458	.	frameshift insertion	Btnl9:NM_172793:exon9:c.965_966insG:p.Q322fs	**Q322fs**	ENSMUSP00000109458,322,Q,	11,49170604,T,G	11qB1.2	.	0.25	.	49	chr11	49170604	.	T	G	.	clustered_events;t_lod_fstar	ECNT=2;HCNT=2;MAX_ED=7;MIN_ED=7;NLOD=16.25;TLOD=5.65	GT:AD:AF:ALT_F1R2:ALT_F2R1:FOXOG:PGT:PID:QSS:REF_F1R2:REF_F2R1	0/1:35,2:0.056:0:2:.:0|1:27471790_GA_G:919,56:27:8	0/0:62,0:0.00:0:0:.:0|1:27471790_GA_G:1553,0:41:21	0.5	Tolerated	-1.02	Neutral	189	30	0.114	Tolerated	2.78	152
T3df	chr5	**1**	**12.5**	43609137	43609137	-	GAGCTACTCTCCAAGGTACATCTGCAGCATCCCT	exonic	**C1qtnf7**	ENSMUSP00000156709	.	frameshift insertion	C1qtnf7:NM_001135172:exon2:c.98_99insGAGCTACTCTCCAAGGTACATCTGCAGCATCCCTT:p.E33fs,C1qtnf7:NM_175425:exon2:c.77_78insGAGCTACTCTCCAAGGTACATCTGCAGCATCCCTT:p.E26fs	**E26fs**	ENSMUSP00000156709,26,E,	5,43609137,A,A	5qB3	.	0.25	.	21	chr5	43609137	.	A	A	.	PASS	DB;ECNT=1;HCNT=1;MAX_ED=.;MIN_ED=.;NLOD=10.48;TLOD=64.39	GT:AD:AF:ALT_F1R2:ALT_F2R1:FOXOG:QSS:REF_F1R2:REF_F2R1	0/1:27,26:0.490:14:12:0.538:763,719:20:7	0/0:38,0:0.00:0:0:.:1036,0:16:22	4	Tolerated	0	Neutral	235	30	1	Tolerated	2.94	55
T3df	chr5	**1**	**12.5**	43609137	43609137	-	GAGCTACTCTCCAAGGTACATCTGCAGCATCCCT	exonic	**C1qtnf7**	ENSMUSP00000156609	.	frameshift insertion	C1qtnf7:NM_001135172:exon2:c.98_99insGAGCTACTCTCCAAGGTACATCTGCAGCATCCCTT:p.E33fs,C1qtnf7:NM_175425:exon2:c.77_78insGAGCTACTCTCCAAGGTACATCTGCAGCATCCCTT:p.E26fs	**E33fs**	ENSMUSP00000156609,33,E,	5,43609137,A,AGAGCTACTCTCCAAGGTACATCTGCAGCATCCCTT	5qB3	.	0.25	.	21	chr5	43609137	.	A	AGAGCTACTCTCCAAGGTACATCTGCAGCATCCCTT	.	t_lod_fstar	ECNT=1;HCNT=1;MAX_ED=.;MIN_ED=.;NLOD=6.07;TLOD=5.44	GT:AD:AF:ALT_F1R2:ALT_F2R1:FOXOG:QSS:REF_F1R2:REF_F2R1	0/1:41,1:0.048:0:1:.:1136,29:23:18	0/0:21,0:0.00:0:0:.:570,0:5:16	0.4	Unknown	NA	NA	235	30	NA	NA	NA	NA
T12df	chrX	**1**	**12.5**	7613975	7613975	C	T	exonic	**Cacna1f**	ENSP00000321618	.	nonsynonymous SNV	Cacna1f:NM_019582:exon9:c.C1297T:p.R433W	**R433W**	ENSP00000321618,433,R,W	X,7613975,C,T	XqA1.1	.	0.25	.	36	chrX	7613975	.	C	T	.	PASS	ECNT=1;HCNT=1;MAX_ED=.;MIN_ED=.;NLOD=10.17;TLOD=12.22	GT:AD:AF:ALT_F1R2:ALT_F2R1:FOXOG:QSS:REF_F1R2:REF_F2R1	0/1:42,6:0.125:4:2:0.333:1050,172:20:22	0/0:36,0:0.00:0:0:.:871,0:15:21	1.2	Damaging	-1.96	Neutral	311	30	0.022	Damaging	3.16	228
T1df	chr11	**1**	**12.5**	98003232	98003232	G	T	exonic	**Cacnb1**	ENSMUSP00000111390	.	nonsynonymous SNV	Cacnb1:NM_001282977:exon14:c.C1598A:p.A533E,Cacnb1:NM_145121:exon14:c.C1463A:p.A488E	**A533E**	ENSMUSP00000111390,533,A,E	11,98003232,G,A	11qD	.	0.25	.	27	chr11	98003232	.	G	A	.	clustered_events;homologous_mapping_event	ECNT=3;HCNT=2;MAX_ED=3;MIN_ED=1;NLOD=7.52;TLOD=8.46	GT:AD:AF:ALT_F1R2:ALT_F2R1:FOXOG:PGT:PID:QSS:REF_F1R2:REF_F2R1	0/1:52,1:0.056:0:1:1.00:0|1:98003232_G_T:1311,26:27:25	0/0:27,0:0.00:0:0:.:0|1:98003232_G_T:633,0:14:13	9	Damaging	-1.23	Neutral	420	30	0.037	Damaging	4.03	159

T1df	chr11	**1**	**12.5**	98003235	98003235	C	A	exonic	**Cacnb1**	ENSMUSP00000111390	.	nonsynonymous SNV	Cacnb1:NM_001282977:exon14:c.G1595T:p.R532L,Cacnb1:NM_145121:exon14:c.G1460T:p.R487L	**R532L**	ENSMUSP00000111390,532,R,L	11,98003235,C,A	11qD	.	0.25	.	27	chr11	98003235	.	C	A	.	clustered_events;homologous_mapping_event	ECNT=3;HCNT=2;MAX_ED=3;MIN_ED=1;NLOD=7.52;TLOD=8.46	GT:AD:AF:ALT_F1R2:ALT_F2R1:FOXOG:PGT:PID:QSS:REF_F1R2:REF_F2R1	0/1:52,1:0.056:0:1:1.00:0|1:98003232_G_T:1311,26:27:25	0/0:27,0:0.00:0:0:.:0|1:98003232_G_T:633,0:14:13	9	Damaging	-2.46	Neutral	420	30	0.001	Damaging	4.03	159
T42df	chr11	**1**	**12.5**	98003231	98003231	-	TCTTGAGTTCACTTCTTTAGAGTGTCTTCTCAGGGCAGAGGGCATAAAACAGCTGATA	exonic	**Cacnb1**	ENSMUSP00000111390	.	frameshift insertion	Cacnb1:NM_001282977:exon14:c.1598_1599insTATCAGCTGTTTTATGCCCTCTGCCCTGAGAAGACACTCTAAAGAAGTGAACTCAAGA:p.A533fs,Cacnb1:NM_145121:exon14:c.1463_1464insTATCAGCTGTTTTATGCCCTCTGCCCTGAGAAGACACTCTAAAGAAGTGAACTCAAGA:p.A488fs	**A488fs**	ENSMUSP00000111390,488,A,	11,98003231,T,A	11qD	.	0.25	.	27	chr11	98003231	.	T	A	.	clustered_events;homologous_mapping_event	ECNT=3;HCNT=2;MAX_ED=3;MIN_ED=1;NLOD=7.52;TLOD=8.46	GT:AD:AF:ALT_F1R2:ALT_F2R1:FOXOG:PGT:PID:QSS:REF_F1R2:REF_F2R1	0/1:52,1:0.056:0:1:1.00:0|1:98003232_G_T:1311,26:27:25	0/0:27,0:0.00:0:0:.:0|1:98003232_G_T:633,0:14:13	9	Tolerated	0	Neutral	420	30	0.519	Tolerated	4.03	159
T42df	chr11	**1**	**12.5**	98003231	98003231	-	TCTTGAGTTCACTTCTTTAGAGTGTCTTCTCAGGGCAGAGGGCATAAAACAGCTGATA	exonic	**Cacnb1**	ENSMUSP00000111391	.	frameshift insertion	Cacnb1:NM_001282977:exon14:c.1598_1599insTATCAGCTGTTTTATGCCCTCTGCCCTGAGAAGACACTCTAAAGAAGTGAACTCAAGA:p.A533fs,Cacnb1:NM_145121:exon14:c.1463_1464insTATCAGCTGTTTTATGCCCTCTGCCCTGAGAAGACACTCTAAAGAAGTGAACTCAAGA:p.A488fs	**A533fs**	ENSMUSP00000111391,533,A,	11,98003231,T,A	11qD	.	0.25	.	27	chr11	98003231	.	T	A	.	t_lod_fstar	ECNT=1;HCNT=1;MAX_ED=.;MIN_ED=.;NLOD=13.84;TLOD=4.56	GT:AD:AF:ALT_F1R2:ALT_F2R1:FOXOG:QSS:REF_F1R2:REF_F2R1	0/1:80,3:0.037:0:3:0.00:2267,87:38:42	0/0:50,0:0.00:0:0:.:1440,0:19:31	0.5	Tolerated	0	Neutral	420	30	0.519	Tolerated	4.03	159
T1df	chr11	**1**	**12.5**	98003235	98003235	C	A	exonic	**Cacnb1**	ENSMUSP00000111390	.	nonsynonymous SNV	Cacnb1:NM_001282977:exon14:c.G1595T:p.R532L,Cacnb1:NM_145121:exon14:c.G1460T:p.R487L	**R487L**	ENSMUSP00000111390,487,R,L	11,98003235,C,CTTGAGTTCACTTCTTTAGAGTGTCTTCT	11qD	.	0.25	.	27	chr11	98003235	.	C	CTTGAGTTCACTTCTTTAGAGTGTCTTCT	.	clustered_events;homologous_mapping_event	ECNT=3;HCNT=2;MAX_ED=3;MIN_ED=1;NLOD=7.83;TLOD=10.10	GT:AD:AF:ALT_F1R2:ALT_F2R1:FOXOG:PGT:PID:QSS:REF_F1R2:REF_F2R1	0/1:50,3:0.073:1:2:.:0|1:98003232_G_T:1420,87:26:24	0/0:26,0:0.00:0:0:.:0|1:98003232_G_T:706,0:14:12	9	Unknown	NA	NA	420	30	NA	NA	NA	NA
T1df	chr11	**1**	**12.5**	98003235	98003235	C	A	exonic	**Cacnb1**	ENSMUSP00000111390	.	nonsynonymous SNV	Cacnb1:NM_001282977:exon14:c.G1595T:p.R532L,Cacnb1:NM_145121:exon14:c.G1460T:p.R487L	**A488E**	ENSMUSP00000111390,488,A,E	11,98003235,C,CTTGAGTTCACTTCTTTAGAGTGTCTTCT	11qD	.	0.25	.	27	chr11	98003235	.	C	CTTGAGTTCACTTCTTTAGAGTGTCTTCT	.	clustered_events;homologous_mapping_event	ECNT=3;HCNT=2;MAX_ED=3;MIN_ED=1;NLOD=7.83;TLOD=10.10	GT:AD:AF:ALT_F1R2:ALT_F2R1:FOXOG:PGT:PID:QSS:REF_F1R2:REF_F2R1	0/1:50,3:0.073:1:2:.:0|1:98003232_G_T:1420,87:26:24	0/0:26,0:0.00:0:0:.:0|1:98003232_G_T:706,0:14:12	9	Unknown	NA	NA	420	30	NA	NA	NA	NA
T1df	chr11	**1**	**12.5**	98003233	98003233	-	TTGAGTTCACTTCTTAGAGTGTCTTCT	exonic	**Cacnb1**	ENSMUSP00000111390	.	frameshift insertion	Cacnb1:NM_001282977:exon14:c.1596_1597insAGAAGACACTCTAAAGAAGTGAACTCAA:p.A533fs,Cacnb1:NM_145121:exon14:c.1461_1462insAGAAGACACTCTAAAGAAGTGAACTCAA:p.A488fs	**A488fs**	ENSMUSP00000111390,488,A,	11,98003233,C,TTCTTGAGTTCACTTCTTTAGAGTGTCTTCTCAGGGCAGAGGGCATAAAACAGCTGATA	11qD	.	0.25	.	26	chr11	98003233	.	C	TTCTTGAGTTCACTTCTTTAGAGTGTCTTCTCAGGGCAGAGGGCATAAAACAGCTGATA	.	t_lod_fstar	ECNT=1;HCNT=2;MAX_ED=.;MIN_ED=.;NLOD=7.60;TLOD=5.39	GT:AD:AF:ALT_F1R2:ALT_F2R1:FOXOG:QSS:REF_F1R2:REF_F2R1	0/1:46,2:0.043:1:1:.:1319,56:27:19	0/0:27,0:0.00:0:0:.:707,0:14:13	9	Unknown	NA	NA	420	30	NA	NA	NA	NA
T1df	chr11	**1**	**12.5**	98003233	98003233	-	TTGAGTTCACTTCTTAGAGTGTCTTCT	exonic	**Cacnb1**	ENSMUSP00000111390	.	frameshift insertion	Cacnb1:NM_001282977:exon14:c.1596_1597insAGAAGACACTCTAAAGAAGTGAACTCAA:p.A533fs,Cacnb1:NM_145121:exon14:c.1461_1462insAGAAGACACTCTAAAGAAGTGAACTCAA:p.A488fs	**A533fs**	ENSMUSP00000111390,533,A,	11,98003233,C,TTCTTGAGTTCACTTCTTTAGAGTGTCTTCTCAGGGCAGAGGGCATAAAACAGCTGATA	11qD	.	0.25	.	26	chr11	98003233	.	C	TTCTTGAGTTCACTTCTTTAGAGTGTCTTCTCAGGGCAGAGGGCATAAAACAGCTGATA	.	t_lod_fstar	ECNT=1;HCNT=2;MAX_ED=.;MIN_ED=.;NLOD=7.60;TLOD=5.39	GT:AD:AF:ALT_F1R2:ALT_F2R1:FOXOG:QSS:REF_F1R2:REF_F2R1	0/1:46,2:0.043:1:1:.:1319,56:27:19	0/0:27,0:0.00:0:0:.:707,0:14:13	9	Unknown	NA	NA	420	30	NA	NA	NA	NA
T32df	chr9	**1**	**12.5**	47818778	47818780	ACC	-	exonic	**Cadm1**	ENSMUSP00000116051	.	nonframeshift deletion	Cadm1:NM_001310841:exon8:c.1048_1050del:p.350_350del,Cadm1:NM_018770:exon8:c.1048_1050del:p.350_350del,Cadm1:NM_207675:exon8:c.1048_1050del:p.350_350del	**350_350del**	NA	9,47818778,TACC,AGCCAGTTCTTGCAGAGAAGAGAGAACAGGCAGATGTGGAACCATACCAGGAGGGTCTCTCTTCTCATCCTGAACTTTCTGG	9qA5.3	.	0.25	.	24	chr9	47818777	.	TACC	AGCCAGTTCTTGCAGAGAAGAGAGAACAGGCAGATGTGGAACCATACCAGGAGGGTCTCTCTTCTCATCCTGAACTTTCTGG	.	t_lod_fstar	ECNT=1;HCNT=1;MAX_ED=.;MIN_ED=.;NLOD=10.65;TLOD=5.90	GT:AD:AF:ALT_F1R2:ALT_F2R1:FOXOG:QSS:REF_F1R2:REF_F2R1	0/1:41,1:0.071:1:0:.:1142,28:22:19	0/0:43,0:0.027:0:0:.:1230,0:20:23	7	Unknown	.	.	.	.	.	.	.	.
T32df	chr15	**1**	**12.5**	102711387	102711387	-	A	exonic	**Calcoco1**	ENSMUSP00000034581	.	frameshift insertion	Calcoco1:NM_026192:exon8:c.960_961insT:p.L321fs	**L321fs**	ENSMUSP00000034581,321,L,	15,102711387,G,A	15qF3	.	0.25	.	37	chr15	102711387	.	G	A	.	clustered_events;homologous_mapping_event	ECNT=4;HCNT=2;MAX_ED=76;MIN_ED=38;NLOD=8.11;TLOD=9.58	GT:AD:AF:ALT_F1R2:ALT_F2R1:FOXOG:PGT:PID:QSS:REF_F1R2:REF_F2R1	0/1:21,2:0.125:0:2:1.00:0|1:78480110_ACCAACACCAG_A:588,54:11:10	0/0:30,0:0.00:0:0:.:0|1:78480110_ACCAACACCAG_A:837,0:12:18	1.2	Tolerated	0	Neutral	154	30	0.259	Tolerated	2.88	65

T1df	chr16	**1**	**12.5**	93609809	93609809	G	A	exonic	**Cbr1**	ENSMUSP00000107297	.	nonsynonymous SNV	Cbr1:NM_007620:exon3:c.G412A:p.V138M	**V138M**	ENSMUSP00000107297,138,V,M	16,93609809,G,C	16qC4	.	0.25	.	50	chr16	93609809	.	G	C	.	PASS	ECNT=1;HCNT=1;MAX_ED=.;MIN_ED=.;NLOD=9.62;TLOD=20.26	GT:AD:AF:ALT_F1R2:ALT_F2R1:FOXOG:QSS:REF_F1R2:REF_F2R1	0/1:42,10:0.180:5:5:0.500:1186,293:22:20	0/0:36,0:0.00:0:0:.:1006,0:14:22	0.8	Tolerated	-2.42	Neutral	184	30	0.211	Tolerated	2.78	197
T32df	chr11	**1**	**12.5**	119081953	119081953	C	T	exonic	**Cbx4**	ENSP00000269397	.	nonsynonymous SNV	Cbx4:NM_007625:exon5:c.G595A:p.A199T	**A199T**	ENSP00000269397,199,A,T	11,119081953,C,T	11qE2	.	0.25	.	29	chr11	119081953	.	C	T	.	PASS	ECNT=1;HCNT=1;MAX_ED=.;MIN_ED=.;NLOD=8.38;TLOD=22.88	GT:AD:AF:ALT_F1R2:ALT_F2R1:FOXOG:QSS:REF_F1R2:REF_F2R1	0/1:32,10:0.244:1:9:0.900:916,280:18:14	0/0:29,0:0.00:0:0:.:799,0:20:9	7	Tolerated	-1.05	Neutral	150	30	0.219	Tolerated	3.13	40
T2df	chr7	**1**	**12.5**	16996032	16996079	CTGCAGCTGATCCAAGAGCAGAGGCTGCAGCTAGTCCAAGGGCAGAGG	-	exonic	**Ccdc8**	ENSMUSP00000145329	.	nonframeshift deletion	Ccdc8:NM_001101535:exon1:c.1445_1492del:p.482_498del	**482_498del**	NA	7,16996032,ACTGCAGCTGATCCAAGAGCAGAGGCTGCAGCTAGTCCAAGGGCAGAGG,G	7qA2	.	0.25	.	52	chr7	16996031	.	ACTGCAGCTGATCCAAGAGCAGAGGCTGCAGCTAGTCCAAGGGCAGAGG	G	.	PASS	ECNT=1;HCNT=1;MAX_ED=.;MIN_ED=.;NLOD=16.50;TLOD=8.19	GT:AD:AF:ALT_F1R2:ALT_F2R1:FOXOG:QSS:REF_F1R2:REF_F2R1	0/1:65,3:0.045:0:3:.:1792,86:36:29	0/0:60,0:0.00:0:0:.:1656,0:22:38	2.2	Unknown	.	.	.	.	.	.	.	.
T3df	chr1	**1**	**12.5**	171580806	171580806	C	T	exonic	**Cd244**	ENSMUSP00000142896	.	stopgain	Cd244:NM_018729:exon7:c.C1042T:p.Q348X	**Q348X**	ENSMUSP00000142896,348,Q,X	NA	1qH3	.	0.25	.	127	chr1	171580806	.	C	GGCCCCCGAGAA	.	alt_allele_in_normal	ECNT=1;HCNT=2;MAX_ED=.;MIN_ED=.;NLOD=4.00;TLOD=8.34	GT:AD:AF:ALT_F1R2:ALT_F2R1:FOXOG:QSS:REF_F1R2:REF_F2R1	0/1:57,3:0.051:1:2:.:1574,81:28:29	0/0:44,2:0.047:0:2:.:1200,56:24:20	1.6	Deletarious	.	.	.	.	.	.	.	.
T42df	chr1	**1**	**12.5**	171559370	171559370	G	T	exonic	**Cd244**	ENSMUSP00000142896	.	nonsynonymous SNV	Cd244:NM_018729:exon1:c.G46T:p.A16S	**A16S**	ENSMUSP00000142896,16,A,S	1,171559370,G,T	1qH3	.	0.25	.	167	chr1	171559370	.	G	T	.	alt_allele_in_normal;clustered_events	ECNT=7;HCNT=2;MAX_ED=97;MIN_ED=4;NLOD=37.54;TLOD=9.19	GT:AD:AF:ALT_F1R2:ALT_F2R1:FOXOG:PGT:PID:QSS:REF_F1R2:REF_F2R1	0/1:178,7:0.037:4:3:0.571:0|1:171559366_C_T:5042,193:97:81	0/0:164,3:0.019:1:2:0.333:0|1:171559366_C_T:4495,88:95:69	20	Tolerated	-0.18	Neutral	101	30	0.18	Tolerated	3.22	38
T2df	chr1	**1**	**12.5**	171574185	171574185	G	A	exonic	**Cd244**	ENSMUSP00000142896	.	nonsynonymous SNV	Cd244:NM_018729:exon3:c.G478A:p.D160N	**D160N**	ENSMUSP00000142896,160,D,N	1,171574185,G,A	1qH3	rs31537435	0.25	.	180	chr1	171574185	.	G	A	.	alt_allele_in_normal	ECNT=1;HCNT=2;MAX_ED=.;MIN_ED=.;NLOD=29.09;TLOD=14.89	GT:AD:AF:ALT_F1R2:ALT_F2R1:FOXOG:QSS:REF_F1R2:REF_F2R1	0/1:207,13:0.046:5:8:0.385:5966,374:102:105	0/0:173,7:0.041:2:5:0.286:4904,192:94:79	20	Tolerated	-0.91	Neutral	101	30	0.376	Tolerated	2.86	56
T1df	chr8	**1**	**12.5**	102679738	102679738	-	GGGCAAG	exonic	**Cdh11**	ENSMUSP00000043753	.	frameshift insertion	Cdh11:NM_009866:exon3:c.101_102insCTTGCCC:p.S34fs	**S34fs**	ENSMUSP00000043753,34,S,	8,102679738,A,C	8qD1	.	0.25	.	33	chr8	102679738	.	A	C	.	t_lod_fstar	ECNT=1;HCNT=2;MAX_ED=.;MIN_ED=.;NLOD=12.29;TLOD=4.30	GT:AD:AF:ALT_F1R2:ALT_F2R1:FOXOG:QSS:REF_F1R2:REF_F2R1	0/1:25,2:0.077:2:0:0.00:688,45:10:15	0/0:51,0:0.00:0:0:.:1397,0:24:27	0.5	Tolerated	0	Neutral	442	30	1	Tolerated	3.15	223
T2df	chr8	**1**	**12.5**	122862086	122862086	-	TTTTT	exonic	**Cdh15**	ENSMUSP00000129369	.	frameshift insertion	Cdh15:NM_007662:exon7:c.898_899insTTTTT:p.L300fs	**L300fs**	ENSMUSP00000129369,300,L,	8,122862086,C,T	8qE1	.	0.25	.	38	chr8	122862086	.	C	T	.	clustered_events;multi_event_alt_allele_in_normal;t_lod_fstar	ECNT=9;HCNT=1;MAX_ED=27;MIN_ED=3;NLOD=5.74;TLOD=5.22	GT:AD:AF:ALT_F1R2:ALT_F2R1:FOXOG:PGT:PID:QSS:REF_F1R2:REF_F2R1	0/1:55,2:0.034:2:0:1.00:0|1:122862086_C_CTTTTT:1562,58:28:27	0/0:36,1:0.029:1:0:1.00:0|1:122862086_C_CTTTTT:1041,28:16:20	1.1	Damaging	-2.15	Neutral	190	30	0.028	Damaging	2.83	184
T2df	chr8	**1**	**12.5**	122862089	122862089	G	T	exonic	**Cdh15**	ENSMUSP00000129369	.	stopgain	Cdh15:NM_007662:exon7:c.G901T:p.E301X	**E301X**	ENSMUSP00000129369,301,E,X	NA	8qE1	.	0.25	.	37	chr8	122862089	.	G	G	.	clustered_events;homologous_mapping_event;t_lod_fstar	ECNT=3;HCNT=8;MAX_ED=71;MIN_ED=30;NLOD=11.43;TLOD=5.02	GT:AD:AF:ALT_F1R2:ALT_F2R1:FOXOG:QSS:REF_F1R2:REF_F2R1	0/1:36,3:0.081:1:2:0.667:1001,87:17:19	0/0:43,0:0.00:0:0:.:1155,0:20:23	1	Deletarious	.	.	.	.	.	.	.	.
T2df	chr8	**1**	**12.5**	122862110	122862110	T	A	exonic	**Cdh15**	ENSMUSP00000129369	.	nonsynonymous SNV	Cdh15:NM_007662:exon7:c.T922A:p.F308I	**F308I**	ENSMUSP00000129369,308,F,I	8,122862110,T,A	8qE1	.	0.25	.	33	chr8	122862110	.	T	A	.	clustered_events;multi_event_alt_allele_in_normal;t_lod_fstar	ECNT=9;HCNT=1;MAX_ED=27;MIN_ED=3;NLOD=5.74;TLOD=5.22	GT:AD:AF:ALT_F1R2:ALT_F2R1:FOXOG:PGT:PID:QSS:REF_F1R2:REF_F2R1	0/1:57,2:0.034:2:0:.:0|1:122862086_C_CTTTTT:1562,58:29:28	0/0:36,1:0.029:1:0:.:0|1:122862086_C_CTTTTT:1026,28:16:20	1.1	Deleterious	-5.34	Deleterious	190	30	0.001	Damaging	2.81	188
T2df	chr8	**1**	**12.5**	122862111	122862111	T	C	exonic	**Cdh15**	ENSMUSP00000129369	.	nonsynonymous SNV	Cdh15:NM_007662:exon7:c.T923C:p.F308S	**F308S**	ENSMUSP00000129369,308,F,S	8,122862111,T,C	8qE1	.	0.25	.	33	chr8	122862111	.	T	C	.	clustered_events;multi_event_alt_allele_in_normal;t_lod_fstar	ECNT=9;HCNT=1;MAX_ED=27;MIN_ED=3;NLOD=5.74;TLOD=5.27	GT:AD:AF:ALT_F1R2:ALT_F2R1:FOXOG:PGT:PID:QSS:REF_F1R2:REF_F2R1	0/1:53,2:0.036:2:0:.:0|1:122862086_C_CTTTTT:1452,34:26:27	0/0:36,1:0.029:1:0:.:0|1:122862086_C_CTTTTT:999,3:17:19	1.1	Deleterious	-7.04	Deleterious	190	30	0	Damaging	2.81	188

T2df	chr8	**1**	**12.5**	122862098	122862098	C	A	exonic	**Cdh15**	ENSMUSP00000129369	.	nonsynonymous SNV	Cdh15:NM_007662:exon7:c.C910A:p.P304T	**P304T**	ENSMUSP00000129369,304,P,T	8,122862098,C,C	8qE1	.	0.25	.	37	chr8	122862098	.	C	C	.	clustered_events;multi_event_alt_allele_in_normal;t_lod_fstar	ECNT=9;HCNT=1;MAX_ED=27;MIN_ED=3;NLOD=4.53;TLOD=5.16	GT:AD:AF:ALT_F1R2:ALT_F2R1:FOXOG:PGT:PID:QSS:REF_F1R2:REF_F2R1	0/1:56,2:0.032:2:0:1.00:0|1:122862086_C_CTTTTT:1506,35:27:29	0/0:32,1:0.033:1:0:1.00:0|1:122862086_C_CTTTTT:857,8:14:18	1.1	Tolerated	0	Neutral	190	30	1	Tolerated	2.83	177
T2df	chr8	**1**	**12.5**	122862113	122862113	-	CCG	exonic	**Cdh15**	ENSMUSP00000129369	.	nonframeshift insertion	Cdh15:NM_007662:exon7:c.925_926insCCG:p.K309delinsTE	**K309delinsTE**	ENSMUSP00000129369,309,K,TE	8,122862113,A,A	8qE1	.	0.25	.	33	chr8	122862113	.	A	A	.	clustered_events;homologous_mapping_event;t_lod_fstar	ECNT=3;HCNT=8;MAX_ED=71;MIN_ED=30;NLOD=11.13;TLOD=5.36	GT:AD:AF:ALT_F1R2:ALT_F2R1:FOXOG:QSS:REF_F1R2:REF_F2R1	0/1:31,4:0.114:1:3:0.750:857,97:15:16	0/0:41,0:0.00:0:0:.:1124,0:22:19	1	Tolerated	0	Neutral	190	30	0.14	Tolerated	2.81	188
T2df	chr8	**1**	**12.5**	122862092	122862093	GG	-	exonic	**Cdh15**	ENSMUSP00000129369	.	frameshift deletion	Cdh15:NM_007662:exon7:c.904_905del:p.G302fs	**G302fs**	ENSMUSP00000129369,302,G,.	8,122862092,AGG,C	8qE1	.	0.25	.	37	chr8	122862091	.	AGG	C	.	clustered_events;multi_event_alt_allele_in_normal;t_lod_fstar	ECNT=9;HCNT=1;MAX_ED=27;MIN_ED=3;NLOD=4.53;TLOD=5.22	GT:AD:AF:ALT_F1R2:ALT_F2R1:FOXOG:PGT:PID:QSS:REF_F1R2:REF_F2R1	0/1:55,2:0.034:2:0:.:0|1:122862086_C_CTTTTT:1597,26:26:29	0/0:32,1:0.033:1:0:.:0|1:122862086_C_CTTTTT:892,14:14:18	1.1	Unknown	NA	NA	190	30	NA	NA	NA	NA
T2df	chr8	**1**	**12.5**	122862108	122862108	A	-	exonic	**Cdh15**	ENSMUSP00000129369	.	frameshift deletion	Cdh15:NM_007662:exon7:c.920delA:p.Q307fs	**Q307fs**	ENSMUSP00000129369,307,Q,.	8,122862108,CA,ACCG	8qE1	.	0.25	.	33	chr8	122862107	.	CA	ACCG	.	clustered_events;multi_event_alt_allele_in_normal;t_lod_fstar	ECNT=9;HCNT=1;MAX_ED=27;MIN_ED=3;NLOD=4.53;TLOD=5.18	GT:AD:AF:ALT_F1R2:ALT_F2R1:FOXOG:PGT:PID:QSS:REF_F1R2:REF_F2R1	0/1:60,2:0.033:2:0:.:0|1:122862086_C_CTTTTT:1631,29:29:31	0/0:32,1:0.033:1:0:.:0|1:122862086_C_CTTTTT:873,13:14:18	1.1	Unknown	NA	NA	190	30	NA	NA	NA	NA
T2df	chr8	**1**	**12.5**	122862100	122862104	TGATG	-	exonic	**Cdh15**	ENSMUSP00000129369	.	frameshift deletion	Cdh15:NM_007662:exon7:c.912_916del:p.P304fs	**P304fs**	ENSMUSP00000129369,304,P,.	8,122862100,CTGATG,CTTTTT	8qE1	.	0.25	.	37	chr8	122862099	.	CTGATG	CTTTTT	.	alt_allele_in_normal;clustered_events;t_lod_fstar	ECNT=9;HCNT=1;MAX_ED=27;MIN_ED=3;NLOD=4.78;RPA=2,7;RU=T;STR;TLOD=5.27	GT:AD:AF:ALT_F1R2:ALT_F2R1:FOXOG:PGT:PID:QSS:REF_F1R2:REF_F2R1	0/1:53,2:0.036:2:0:.:0|1:122862086_C_CTTTTT:1483,49:27:26	0/0:36,2:0.057:2:0:.:0|1:122862086_C_CTTTTT:1032,56:16:20	1.1	Unknown	.	.	.	.	.	.	.	.
T2df	chr10	**1**	**12.5**	127065952	127065952	G	A	splicing	**Cdk4**	ENSMUSP00000143732	.	.	.	**NA**	10,127065952,G,A	NA	10qD3	.	0.25	.	36	chr10	127065952	.	G	A	.	clustered_events	ECNT=2;HCNT=1;MAX_ED=9;MIN_ED=9;NLOD=8.73;TLOD=69.84	GT:AD:AF:ALT_F1R2:ALT_F2R1:FOXOG:PGT:PID:QSS:REF_F1R2:REF_F2R1	0/1:31,26:0.450:10:11:0.524:0|1:125122132_C_CTCCCTG:616,600:13:11	0/0:31,0:0.00:0:0:.:0|1:125122132_C_CTCCCTG:664,0:14:10	3	Unkown	.	.	.	.	.	.	.	.
T32df	chr5	**1**	**12.5**	3344589	3344589	A	T	exonic	**Cdk6**	ENSMUSP00000004829	.	nonsynonymous SNV	Cdk6:NM_009873:exon2:c.A223T:p.N75Y	**N75Y**	ENSMUSP00000004829,75,N,Y	5,3344589,A,G	5qA1	.	0.25	.	68	chr5	3344589	.	A	G	.	str_contraction;t_lod_fstar	ECNT=1;HCNT=1;MAX_ED=.;MIN_ED=.;NLOD=14.92;RPA=6,5;RU=GCA;STR;TLOD=4.27	GT:AD:AF:ALT_F1R2:ALT_F2R1:FOXOG:QSS:REF_F1R2:REF_F2R1	0/1:40,3:0.050:0:3:.:968,78:20:20	0/0:61,1:0.019:1:0:.:1533,6:30:31	1	Deleterious	-4.41	Deleterious	145	30	0.005	Damaging	2.86	148
T32df	chr5	**1**	**12.5**	3344581	3344581	A	G	exonic	**Cdk6**	ENSMUSP00000004829	.	nonsynonymous SNV	Cdk6:NM_009873:exon2:c.A215G:p.E72G	**E72G**	ENSMUSP00000004829,72,E,G	5,3344581,A,T	5qA1	.	0.25	.	70	chr5	3344581	.	A	T	.	t_lod_fstar	ECNT=1;HCNT=1;MAX_ED=.;MIN_ED=.;NLOD=14.95;TLOD=5.00	GT:AD:AF:ALT_F1R2:ALT_F2R1:FOXOG:QSS:REF_F1R2:REF_F2R1	0/1:62,3:0.051:3:0:1.00:1737,87:29:33	0/0:54,1:0.019:0:1:0.00:1561,14:25:29	1.1	Deleterious	-4.52	Deleterious	145	30	0.011	Damaging	2.93	127
T22df	chr5	**1**	**12.5**	146231815	146231815	A	T	exonic	**Cdk8**	ENSMUSP00000034443	.	nonsynonymous SNV	Cdk8:NM_153599:exon1:c.A123T:p.K41N	**K41N**	ENSMUSP00000034443,41,K,N	5,146231815,A,T	5qG3	.	0.25	.	41	chr5	146231815	.	A	T	.	alt_allele_in_normal;clustered_events	ECNT=4;HCNT=2;MAX_ED=76;MIN_ED=38;NLOD=2.25;TLOD=6.89	GT:AD:AF:ALT_F1R2:ALT_F2R1:FOXOG:QSS:REF_F1R2:REF_F2R1	0/1:29,4:0.125:2:2:0.500:706,116:17:12	0/0:40,3:0.073:2:1:0.333:991,86:21:19	1.2	Deleterious	-3.43	Deleterious	195	30	0.001	Damaging	2.89	223
T32df	chr3	**1**	**12.5**	94488231	94488233	CAG	-	exonic	**Celf3**	ENSMUSP00000037925	.	nonframeshift deletion	Celf3:NM_001289613:exon10:c.1165_1167del:p.389_389del,Celf3:NM_172434:exon10:c.1078_1080del:p.360_360del,Celf3:NM_001289616:exon11:c.850_852del:p.284_284del	**389_389del**	NA	3,94488231,ACAG,A	3qF2.1	rs256919272	0.25	.	31	chr3	94488230	.	ACAG	A	.	str_contraction;t_lod_fstar	ECNT=1;HCNT=8;MAX_ED=.;MIN_ED=.;NLOD=8.42;RPA=7,6;RU=CAG;STR;TLOD=4.61	GT:AD:AF:ALT_F1R2:ALT_F2R1:FOXOG:QSS:REF_F1R2:REF_F2R1	0/1:59,2:0.033:0:2:.:1662,57:28:31	0/0:31,0:0.00:0:0:.:872,0:20:11	18	Unknown	.	.	.	.	.	.	.	.

T32df	chr3	**1**	**12.5**	94488231	94488233	CAG	-	exonic	**Celf3**	ENSMUSP000001229733	.	nonframeshift deletion	Celf3:NM_001289613:exon10:c.1165_1167del:p.389_389del,Celf3:NM_172434:exon10:c.1078_1080del:p.360_360del,Celf3:NM_001289616:exon11:c.850_852del:p.284_284del	**360_360del**	NA	3,94488231,ACAG,A	3qF2.1	rs256919272	0.25	.	31	chr3	94488230	.	ACAG	A	.	t_lod_fstar	ECNT=1;HCNT=1;MAX_ED=.;MIN_ED=.;NLOD=5.37;TLOD=4.52	GT:AD:AF:ALT_F1R2:ALT_F2R1:FOXOG:QSS:REF_F1R2:REF_F2R1	0/1:50,3:0.060:1:2:0.333:1370,80:26:24	0/0:20,0:0.00:0:0:.:557,0:8:12	1.9	Unknown	.	.	.	.	.	.	.	.
T32df	chr3	**1**	**12.5**	94488231	94488233	CAG	-	exonic	**Celf3**	ENSMUSP00000142796	.	nonframeshift deletion	Celf3:NM_001289613:exon10:c.1165_1167del:p.389_389del,Celf3:NM_172434:exon10:c.1078_1080del:p.360_360del,Celf3:NM_001289616:exon11:c.850_852del:p.284_284del	**284_284del**	NA	3,94488231,ACAG,A	3qF2.1	rs256919272	0.25	.	31	chr3	94488230	.	ACAG	A	.	alt_allele_in_normal	ECNT=1;HCNT=2;MAX_ED=.;MIN_ED=.;NLOD=38.26;TLOD=15.17	GT:AD:AF:ALT_F1R2:ALT_F2R1:FOXOG:QSS:REF_F1R2:REF_F2R1	0/1:214,7:0.029:5:2:.:6024,205:114:100	0/0:166,2:0.013:0:2:.:4544,57:85:81	20	Unknown	.	.	.	.	.	.	.	.
T4df	chr19	**1**	**12.5**	5492806	5492806	T	C	splicing	**Cfl1**	ENSMUSP00000142967	.	.	.	**NA**	19,5492806,T,C	NA	19qA	.	0.25	.	46	chr19	5492806	.	T	C	.	alt_allele_in_normal	ECNT=2;HCNT=2;MAX_ED=1;MIN_ED=1;NLOD=3.97;TLOD=11.45	GT:AD:AF:ALT_F1R2:ALT_F2R1:FOXOG:PGT:PID:QSS:REF_F1R2:REF_F2R1	0/1:58,3:0.085:1:2:0.333:0|1:5492805_G_GGCCCC:1681,52:26:32	0/0:44,2:0.047:0:2:0.00:0|1:5492805_G_GGCCCC:1242,43:24:20	1.6	Unkown	.	.	.	.	.	.	.	.
T1df	chr8	**1**	**12.5**	72467903	72467908	TGCTGT	-	exonic	**Cherp**	ENSMUSP00000140645	.	nonframeshift deletion	Cherp:NM_138585:exon8:c.1011_1016del:p.337_339del	**337_339del**	NA	8,72467903,CTGCTGT,A	8qB3.3	rs216556503	0.25	.	26	chr8	72467902	.	CTGCTGT	A	.	PASS	DB;ECNT=1;HCNT=1;MAX_ED=.;MIN_ED=.;NLOD=10.48;TLOD=64.39	GT:AD:AF:ALT_F1R2:ALT_F2R1:FOXOG:QSS:REF_F1R2:REF_F2R1	0/1:27,26:0.490:14:12:0.538:763,719:20:7	0/0:38,0:0.00:0:0:.:1036,0:16:22	4	Unknown	.	.	.	.	.	.	.	.
T2df	chr8	**1**	**12.5**	104309703	104309801	CCGGGTACTGAAGGTCCCTGGCTGGCTGGTGTCCCGGGTACTGAAGGTCCCTGGCTGGCTGGTGTCCCGGGTACTGAAGGTCCCTGGCTGGCTGGTGTC	-	exonic	**Cmtm1**	ENSMUSP00000122745	.	nonframeshift deletion	Cmtm1:NM_181990:exon1:c.195_293del:p.65_98del	**65_98del**	NA	8,104309703,TCCGGGTACTGAAGGTCCCTGGCTGGCTGGTGTCCCGGGTACTGAAGGTCCCTGGCTGGCTGGTGTCCCGGGTACTGAAGGTCCCTGGCTGGCTGGTGTC,G	8qD3	.	0.25	.	42	chr8	104309702	.	TCCGGGTACTGAAGGTCCCTGGCTGGCTGGTGTCCCGGGTACTGAAGGTCCCTGGCTGGCTGGTGTCCCGGGTACTGAAGGTCCCTGGCTGGCTGGTGTC	G	.	clustered_events;homologous_mapping_event	DB;ECNT=4;HCNT=2;MAX_ED=66;MIN_ED=64;NLOD=11.14;TLOD=107.88	GT:AD:AF:ALT_F1R2:ALT_F2R1:FOXOG:PGT:PID:QSS:REF_F1R2:REF_F2R1	0/1:38,29:0.446:19:10:0.345:0|1:129400088_G_A:1061,823:13:25	0/0:42,0:0.00:0:0:.:0|1:129400088_G_A:1191,0:19:23	2.2	Unknown	.	.	.	.	.	.	.	.
T2df	chr12	**1**	**12.5**	51593745	51593747	GCT	-	exonic	**Coch**	ENSMUSP00000107710	.	nonframeshift deletion	Coch:NM_007728:exon2:c.42_44del:p.14_15del,Coch:NM_001198835:exon3:c.42_44del:p.14_15del	**14_15del**	NA	12,51593745,GGCT,A	12qB3	.	0.25	.	22	chr12	51593744	.	GGCT	A	.	clustered_events	DB;ECNT=2;HCNT=1;MAX_ED=5;MIN_ED=5;NLOD=14.40;TLOD=33.81	GT:AD:AF:ALT_F1R2:ALT_F2R1:FOXOG:PGT:PID:QSS:REF_F1R2:REF_F2R1	0/1:15,9:0.391:6:3:0.667:0|1:104804380_T_A:419,261:6:9	0/0:57,0:0.00:0:0:.:0|1:104804380_T_A:1548,0:33:24	2.3	Unknown	.	.	.	.	.	.	.	.
T1df	chr15	**1**	**12.5**	71915797	71915823	GGTCCAGGGGGCCCTGGAACACCGGGT	-	exonic	**Col22a1**	ENSMUSP00000032260	.	nonframeshift deletion	Col22a1:NM_027174:exon24:c.2175_2201del:p.725_734del	**725_734del**	NA	15,71915797,AGGTCCAGGGGGCCCTGGAACACCGGGT,G	15qD3	.	0.25	.	47	chr15	71915796	.	AGGTCCAGGGGGCCCTGGAACACCGGGT	G	.	PASS	DB;ECNT=1;HCNT=1;MAX_ED=.;MIN_ED=.;NLOD=14.09;TLOD=18.12	GT:AD:AF:ALT_F1R2:ALT_F2R1:FOXOG:QSS:REF_F1R2:REF_F2R1	0/1:7,8:0.500:3:5:0.625:193,212:4:3	0/0:49,0:0.00:0:0:.:1356,0:21:28	2.3	Unknown	.	.	.	.	.	.	.	.
T1df	chr12	**1**	**12.5**	28599046	28599046	T	A	exonic	**Colec11**	ENSMUSP00000124656	.	nonsynonymous SNV	Colec11:NM_001313978:exon4:c.A275T:p.K92I,Colec11:NM_027866:exon4:c.A275T:p.K92I	**K92I**	ENSMUSP00000124656,92,K,I	12,28599046,T,G	12qA2	.	0.25	.	37	chr12	28599046	.	T	G	.	PASS	ECNT=1;HCNT=2;MAX_ED=.;MIN_ED=.;NLOD=10.13;TLOD=93.84	GT:AD:AF:ALT_F1R2:ALT_F2R1:FOXOG:QSS:REF_F1R2:REF_F2R1	0/1:12,35:0.744:16:19:0.543:307,978:3:9	0/0:35,0:0.00:0:0:.:832,0:22:13	1.7	Deleterious	-2.72	Deleterious	195	30	0.003	Damaging	2.87	107
T12df	chr5	**1**	**12.5**	113844453	113844453	A	C	exonic	**Coro1c**	ENSMUSP00000124855	.	nonsynonymous SNV	Coro1c:NM_011779:exon11:c.T1321G:p.L441V	**L441V**	ENSMUSP00000124855,441,L,V	5,113844453,A,T	5qF	.	0.25	.	35	chr5	113844453	.	A	T	.	alt_allele_in_normal	ECNT=1;HCNT=1;MAX_ED=.;MIN_ED=.;NLOD=9.62;TLOD=8.16	GT:AD:AF:ALT_F1R2:ALT_F2R1:FOXOG:QSS:REF_F1R2:REF_F2R1	0/1:25,5:0.167:3:2:0.400:678,125:10:15	0/0:54,2:0.043:1:1:0.500:1447,43:27:27	0.4	Damaging	-0.87	Neutral	259	30	0.039	Damaging	2.98	246
T12df	chr5	**1**	**12.5**	113844351	113844351	A	C	exonic	**Coro1c**	ENSMUSP00000086623	.	stoploss	Coro1c:NM_011779:exon11:c.T1423G:p.X475G	**X475G**	NA	NA	5qF	.	0.25	.	41	chr5	113844351	.	A	A	.	PASS	DB;ECNT=1;HCNT=1;MAX_ED=.;MIN_ED=.;NLOD=10.48;TLOD=64.39	GT:AD:AF:ALT_F1R2:ALT_F2R1:FOXOG:QSS:REF_F1R2:REF_F2R1	0/1:27,26:0.490:14:12:0.538:763,719:20:7	0/0:38,0:0.00:0:0:.:1036,0:16:22	4	Deletarious	.	.	.	.	.	.	.	.

T32df	chr1	**1**	**12.5**	10488925	10488925	T	C	exonic	**Cpa6**	ENSMUSP00000124531	.	nonsynonymous SNV	Cpa6:NM_177834:exon3:c.A217G:p.I73V	**I73V**	ENSMUSP00000124531,73,I,V	1,10488925,T,A	1qA2	.	0.25	.	43	chr1	10488925	.	T	A	.	clustered_events;homologous_mapping_event;t_lod_fstar	ECNT=3;HCNT=3;MAX_ED=7;MIN_ED=5;NLOD=9.93;TLOD=5.28	GT:AD:AF:ALT_F1R2:ALT_F2R1:FOXOG:PGT:PID:QSS:REF_F1R2:REF_F2R1	0/1:42,3:0.071:0:3:0.00:0|1:2859904 1_T_G:1164,32:22:20	0/0:37,0:0.00:0:0:.:0|1:28599041_T_G:1000,0:23:14	1.3	Damaging	-1.99	Neutral	180	30	0.032	Damaging	2.79	164
T12df	chr15	**1**	**12.5**	76601604	76601604	T	C	exonic	**Cpsf1**	ENSMUSP00000124270	.	nonsynonymous SNV	Cpsf1:NM_001164173:exon12:c.A1264G:p.K422E,Cpsf1:NM_053193:exon12:c.A1264G:p.K422E	**K422E**	ENSMUSP00000124270,422,K,E	15,76601604,T,T	15qD3	.	0.25	.	41	chr15	76601604	.	T	T	.	alt_allele_in_normal	ECNT=1;HCNT=1;MAX_ED=.;MIN_ED=.;NLOD=9.62;TLOD=8.16	GT:AD:AF:ALT_F1R2:ALT_F2R1:FOXOG:QSS:REF_F1R2:REF_F2R1	0/1:25,5:0.167:3:2:0.400:678,125:10:15	0/0:54,2:0.043:1:1:0.500:1447,43:27:27	0.4	Tolerated	0	Neutral	97	30	1	Tolerated	2.87	80
T4df	chr6	**1**	**12.5**	37362366	37362366	G	A	exonic	**Creb3l2**	ENSMUSP00000040132	.	nonsynonymous SNV	Creb3l2:NM_178661:exon4:c.C575T:p.P192L	**P192L**	ENSMUSP00000040132,192,P,L	6,37362366,G,A	6qB1	.	0.25	.	58	chr6	37362366	.	G	A	.	alt_allele_in_normal;clustered_events	ECNT=3;HCNT=7;MAX_ED=62;MIN_ED=47;NLOD=91.84;TLOD=12.00	GT:AD:AF:ALT_F1R2:ALT_F2R1:FOXOG:QSS:REF_F1R2:REF_F2R1	0/1:433,10:0.024:7:3:0.700:12390,281:217:216	0/0:438,8:0.020:3:5:0.375:12162,224:224:214	0.3	Tolerated	-1.93	Neutral	122	30	0.072	Tolerated	3.08	87
T32df	chr8	**1**	**12.5**	16127191	16127191	G	T	exonic	**Csmd1**	ENSMUSP00000104977	.	nonsynonymous SNV	Csmd1:NM_053171:exon26:c.C4057A:p.L1353I	**L1353I**	ENSMUSP00000104977,1353,L,I	8,16127191,G,C	8qA1.2	.	0.25	.	48	chr8	16127191	.	G	C	.	PASS	ECNT=1;HCNT=1;MAX_ED=.;MIN_ED=.;NLOD=9.58;TLOD=8.42	GT:AD:AF:ALT_F1R2:ALT_F2R1:FOXOG:QSS:REF_F1R2:REF_F2R1	0/1:33,5:0.114:4:1:0.800:927,149:17:16	0/0:39,0:0.00:0:0:.:1097,0:16:23	2.1	Tolerated	-1.81	Neutral	147	30	0.112	Tolerated	2.93	145
T3df	chr8	**1**	**12.5**	16288713	16288713	T	A	exonic	**Csmd1**	ENSMUSP00000104977	.	nonsynonymous SNV	Csmd1:NM_053171:exon11:c.A1379T:p.E460V	**E460V**	ENSMUSP00000104977,460,E,V	8,16288713,T,T	8qA1.2	.	0.25	.	51	chr8	16288713	.	T	T	.	clustered_events;multi_event_alt_allele_in_normal	ECNT=7;HCNT=1;MAX_ED=24;MIN_ED=2;NLOD=6.34;TLOD=8.71	GT:AD:AF:ALT_F1R2:ALT_F2R1:FOXOG:PGT:PID:QSS:REF_F1R2:REF_F2R1	0/1:42,3:0.067:0:3:0.00:0|1:179752666_C_CGGTGGT:1156,49:25:17	0/0:36,1:0.028:0:1:0.00:0|1:179752666_C_CGGTGGT:966,24:22:14	21	Tolerated	0	Neutral	147	30	1	Tolerated	2.92	135
T2df	chr2	**1**	**12.5**	121981235	121981235	A	C	exonic	**Ctdspl2**	ENSMUSP00000138786	.	nonsynonymous SNV	Ctdspl2:NM_001290991:exon5:c.A556C:p.I186L,Ctdspl2:NM_001290992:exon5:c.A217C:p.I73L,Ctdspl2:NM_212450:exon5:c.A559C:p.I187L	**I187L**	ENSMUSP00000138786,187,I,L	2,121981235,A,A	2qE5	.	0.25	.	51	chr2	121981235	.	A	A	.	clustered_events	DB;ECNT=2;HCNT=61;MAX_ED=132;MIN_ED=132;NLOD=12.03;TLOD=34.67	GT:AD:AF:ALT_F1R2:ALT_F2R1:FOXOG:QSS:REF_F1R2:REF_F2R1	0/1:20,16:0.429:4:12:0.750:565,4101:3:7	0/0:41,0:0.00:0:0:.:1165,0:21:20	1.6	Tolerated	0	Neutral	100	30	1	Tolerated	3.36	54
T2df	chr2	**1**	**12.5**	121981235	121981235	A	C	exonic	**Ctdspl2**	ENSMUSP00000138786	.	nonsynonymous SNV	Ctdspl2:NM_001290991:exon5:c.A556C:p.I186L,Ctdspl2:NM_001290992:exon5:c.A217C:p.I73L,Ctdspl2:NM_212450:exon5:c.A559C:p.I187L	**I73L**	ENSMUSP00000138786,73,I,L	2,121981235,A,A	2qE5	.	0.25	.	51	chr2	121981235	.	A	A	.	clustered_events	DB;ECNT=2;HCNT=61;MAX_ED=132;MIN_ED=132;NLOD=12.03;TLOD=34.67	GT:AD:AF:ALT_F1R2:ALT_F2R1:FOXOG:QSS:REF_F1R2:REF_F2R1	0/1:20,16:0.429:4:12:0.750:565,4101:3:7	0/0:41,0:0.00:0:0:.:1165,0:21:20	1.6	Tolerated	0	Neutral	100	30	1	Tolerated	3.36	54
T2df	chr2	**1**	**12.5**	121981235	121981235	A	C	exonic	**Ctdspl2**	ENSMUSP00000044416	.	nonsynonymous SNV	Ctdspl2:NM_001290991:exon5:c.A556C:p.I186L,Ctdspl2:NM_001290992:exon5:c.A217C:p.I73L,Ctdspl2:NM_212450:exon5:c.A559C:p.I187L	**I186L**	ENSMUSP00000044416,186,I,L	2,121981235,A,C	2qE5	.	0.25	.	51	chr2	121981235	.	A	C	.	t_lod_fstar	ECNT=1;HCNT=2;MAX_ED=.;MIN_ED=.;NLOD=12.29;TLOD=4.30	GT:AD:AF:ALT_F1R2:ALT_F2R1:FOXOG:QSS:REF_F1R2:REF_F2R1	0/1:25,2:0.077:2:0:0.00:688,45:10:15	0/0:51,0:0.00:0:0:.:1397,0:24:27	0.5	Tolerated	-0.35	Neutral	100	30	0.16	Tolerated	3.36	54
T1df	chr6	**1**	**12.5**	77605186	77605186	T	G	exonic	**Ctnna2**	ENSMUSP00000040208	.	nonsynonymous SNV	Ctnna2:NM_001355193:exon7:c.A1051C:p.N351H,Ctnna2:NM_009819:exon7:c.A1051C:p.N351H,Ctnna2:NM_001109764:exon8:c.A1051C:p.N351H,Ctnna2:NM_145732:exon8:c.A1051C:p.N351H	**N351H**	ENSMUSP00000040208,351,N,H	6,77605186,T,A	6qC3	.	0.25	.	39	chr6	77605186	.	T	A	.	alt_allele_in_normal;clustered_events	ECNT=4;HCNT=6;MAX_ED=68;MIN_ED=43;NLOD=40.24;TLOD=30.47	GT:AD:AF:ALT_F1R2:ALT_F2R1:FOXOG:QSS:REF_F1R2:REF_F2R1	0/1:248,17:0.065:7:10:0.412:6065,496:129:119	0/0:379,21:0.052:12:9:0.571:9328,600:190:189	0.3	Deleterious	-4.02	Deleterious	231	30	0.016	Damaging	2.9	184
T1df	chr7	**1**	**12.5**	25816443	25816445	CAG	-	exonic	**Cyp2s1**	ENSMUSP00000074809	.	nonframeshift deletion	Cyp2s1:NM_028775:exon1:c.58_60del:p.20_20del	**20_20del**	NA	7,25816443,ACAG,A	7qA3	.	0.25	.	22	chr7	25816442	.	ACAG	A	.	PASS	DB;ECNT=1;HCNT=1;MAX_ED=.;MIN_ED=.;NLOD=10.48;TLOD=64.39	GT:AD:AF:ALT_F1R2:ALT_F2R1:FOXOG:QSS:REF_F1R2:REF_F2R1	0/1:27,26:0.490:14:12:0.538:763,719:20:7	0/0:38,0:0.00:0:0:.:1036,0:16:22	4	Unknown	.	.	.	.	.	.	.	.
T32df	chr4	**1**	**12.5**	115628745	115628745	C	T	exonic	**Cyp4b1**	ENSMUSP00000135036	.	nonsynonymous SNV	Cyp4b1:NM_007823:exon8:c.G994A:p.A332T	**A332T**	ENSMUSP00000135036,332,A,T	4,115628745,C,G	4qD1	.	0.25	.	30	chr4	115628745	.	C	G	.	PASS	ECNT=1;HCNT=1;MAX_ED=.;MIN_ED=.;NLOD=12.59;TLOD=6.94	GT:AD:AF:ALT_F1R2:ALT_F2R1:FOXOG:QSS:REF_F1R2:REF_F2R1	0/1:47,4:0.083:0:4:1.00:1331,110:24:23	0/0:46,0:0.00:0:0:.:1292,0:25:21	19	Deleterious	-4.84	Deleterious	181	30	0.001	Damaging	2.81	227

T1df	chr17	**1**	**12.5**	33912696	33912698	GAG	-	exonic	**Daxx**	ENSMUSP00000135370	.	nonframeshift deletion	Daxx:NM_001199733:exon5:c.1378_1380del:p.460_460del,Daxx:NM_001355704:exon5:c.1378_1380del:p.460_460del,Daxx:NM_007829:exon5:c.1378_1380del:p.460_460del	**460_460del**	NA	17,33912696,TGAG,A	17qB1	.	0.25	.	27	chr17	33912695	.	TGAG	A	.	alt_allele_in_normal;t_lod_fstar	ECNT=1;HCNT=1;MAX_ED=.;MIN_ED=.;NLOD=7.37;TLOD=5.22	GT:AD:AF:ALT_F1R2:ALT_F2R1:FOXOG:QSS:REF_F1R2:REF_F2R1	0/1:30,3:0.094:2:1:0.667:729,84:15:15	0/0:47,2:0.043:1:1:0.500:1174,55:22:25	0.9	Unknown	.	.	.	.	.	.	.	.
T1df	chr9	**1**	**12.5**	99583673	99583675	GAG	-	exonic	**Dbr1**	ENSMUSP00000104032	.	nonframeshift deletion	Dbr1:NM_031403:exon8:c.1303_1305del:p.435_435del	**435_435del**	NA	9,99583673,AGAG,A	9qE3.3	.	0.25	.	16	chr9	99583672	.	AGAG	A	.	t_lod_fstar	ECNT=1;HCNT=4;MAX_ED=.;MIN_ED=.;NLOD=13.84;TLOD=4.86	GT:AD:AF:ALT_F1R2:ALT_F2R1:FOXOG:QSS:REF_F1R2:REF_F2R1	0/1:55,3:0.054:1:2:0.333:1541,85:30:25	0/0:51,0:0.00:0:0:.:1424,0:25:26	5	Unknown	NA	NA	81	30	NA	NA	NA	.
T4df	chr12	**1**	**12.5**	80340262	80340262	G	T	exonic	**Dcaf5**	ENSMUSP00000129291	.	stopgain	Dcaf5:NM_177267:exon9:c.C1089A:p.Y363X	**Y363X**	ENSMUSP00000129291,363,Y,X	NA	12qC3	.	0.25	.	23	chr12	80340262	.	G	A	.	alt_allele_in_normal;t_lod_fstar	ECNT=2;HCNT=2;MAX_ED=2;MIN_ED=2;NLOD=2.44;TLOD=5.41	GT:AD:AF:ALT_F1R2:ALT_F2R1:FOXOG:PGT:PID:QSS:REF_F1R2:REF_F2R1	0/1:46,2:0.043:1:1:0.500:0|1:78346447_T_C:1219,53:24:22	0/0:34,1:0.053:0:1:0.00:0|1:78346447_T_C:847,29:17:17	0.7	Deletarious	.	.	.	.	.	.	.	.
T4df	chr12	**1**	**12.5**	80340269	80340269	-	G	exonic	**Dcaf5**	ENSMUSP00000129291	.	frameshift insertion	Dcaf5:NM_177267:exon9:c.1081_1082insC:p.S361fs	**S361fs**	ENSMUSP00000129291,361,S,	12,80340269,C,A	12qC3	.	0.25	.	24	chr12	80340269	.	C	A	.	clustered_events;multi_event_alt_allele_in_normal	ECNT=7;HCNT=1;MAX_ED=25;MIN_ED=15;NLOD=2.42;TLOD=8.98	GT:AD:AF:ALT_F1R2:ALT_F2R1:FOXOG:PGT:PID:QSS:REF_F1R2:REF_F2R1	0/1:36,3:0.081:3:0:.:0|1:80340247_ACATCCTGGCTGC_A:1040,58:17:19	0/0:21,1:0.043:1:0:.:0|1:80340247_ACATCCTGGCTGC_A:578,20:13:8	0.3	Deleterious	-5.07	Deleterious	83	30	0.023	Damaging	3.05	279
T4df	chr12	**1**	**12.5**	80340272	80340272	-	ACCG	exonic	**Dcaf5**	ENSMUSP00000129291	.	frameshift insertion	Dcaf5:NM_177267:exon9:c.1078_1079insCGGT:p.W360fs	**W360fs**	ENSMUSP00000129291,360,W,	12,80340272,C,TTTA	12qC3	.	0.25	.	25	chr12	80340272	.	C	TTTA	.	clustered_events;multi_event_alt_allele_in_normal	ECNT=7;HCNT=1;MAX_ED=25;MIN_ED=15;NLOD=2.42;TLOD=8.98	GT:AD:AF:ALT_F1R2:ALT_F2R1:FOXOG:PGT:PID:QSS:REF_F1R2:REF_F2R1	0/1:33,3:0.081:3:0:.:0|1:80340247_ACATCCTGGCTGC_A:898,49:15:18	0/0:23,1:0.043:1:0:.:0|1:80340247_ACATCCTGGCTGC_A:614,13:13:10	0.3	Deleterious	-20.74	Deleterious	83	30	NA	NA	NA	NA
T4df	chr12	**1**	**12.5**	80340263	80340263	-	TTA	exonic	**Dcaf5**	ENSMUSP00000129291	.	nonframeshift insertion	Dcaf5:NM_177267:exon9:c.1087_1088insTAA:p.Y363delinsLN	**Y363delinsLN**	ENSMUSP00000129291,363,Y,LN	12,80340263,T,A	12qC3	.	0.25	.	24	chr12	80340263	.	T	A	.	alt_allele_in_normal;clustered_events;germline_risk	DB;ECNT=2;HCNT=1;MAX_ED=10;MIN_ED=10;NLOD=2.37;TLOD=8.66	GT:AD:AF:ALT_F1R2:ALT_F2R1:FOXOG:PGT:PID:QSS:REF_F1R2:REF_F2R1	0/1:63,4:0.063:1:3:0.250:0|1:78349046_T_A:1779,109:34:29	0/0:51,4:0.080:3:1:0.750:0|1:78349046_T_A:1377,88:28:23	0.7	Tolerated	-0.05	Neutral	83	30	0.133	Tolerated	3.06	158
T4df	chr12	**1**	**12.5**	80340270	80340270	T	A	exonic	**Dcaf5**	ENSMUSP00000129291	.	nonsynonymous SNV	Dcaf5:NM_177267:exon9:c.A1081T:p.S361C	**S361C**	ENSMUSP00000129291,361,S,C	12,80340270,T,CG	12qC3	.	0.25	.	24	chr12	80340270	.	T	CG	.	clustered_events;germline_risk;multi_event_alt_allele_in_normal	ECNT=7;HCNT=1;MAX_ED=25;MIN_ED=15;NLOD=2.12;TLOD=9.02	GT:AD:AF:ALT_F1R2:ALT_F2R1:FOXOG:PGT:PID:QSS:REF_F1R2:REF_F2R1	0/1:30,3:0.083:3:0:.:0|1:80340247_ACATCCTGGCTGC_A:828,45:14:16	0/0:23,1:0.045:1:0:.:0|1:80340247_ACATCCTGGCTGC_A:599,3:12:11	0.3	Unknown	NA	NA	83	30	NA	NA	NA	NA
T4df	chr12	**1**	**12.5**	80340266	80340266	-	ATAC	exonic	**Dcaf5**	ENSMUSP00000129291	.	frameshift insertion	Dcaf5:NM_177267:exon9:c.1084_1085insGTAT:p.P362fs	**P362fs**	ENSMUSP00000129291,362,P,	12,80340266,G,CACCG	12qC3	.	0.25	.	24	chr12	80340266	.	G	CACCG	.	clustered_events;germline_risk;multi_event_alt_allele_in_normal	ECNT=7;HCNT=1;MAX_ED=25;MIN_ED=15;NLOD=2.12;TLOD=9.09	GT:AD:AF:ALT_F1R2:ALT_F2R1:FOXOG:PGT:PID:QSS:REF_F1R2:REF_F2R1	0/1:31,3:0.088:3:0:.:0|1:80340247_ACATCCTGGCTGC_A:862,81:14:17	0/0:24,1:0.045:1:0:.:0|1:80340247_ACATCCTGGCTGC_A:644,10:12:12	0.3	Unknown	NA	NA	83	30	NA	NA	NA	NA
T4df	chr12	**1**	**12.5**	80340248	80340259	CATCCTGGCTGC	-	exonic	**Dcaf5**	ENSMUSP00000129291	.	nonframeshift deletion	Dcaf5:NM_177267:exon9:c.1092_1103del:p.364_368del	**364_368del**	NA	12,80340248,ACATCCTGGCTGC,T	12qC3	.	0.25	.	22	chr12	80340247	.	ACATCCTGGCTGC	T	.	alt_allele_in_normal;clustered_events	ECNT=7;HCNT=1;MAX_ED=25;MIN_ED=15;NLOD=2.42;TLOD=8.94	GT:AD:AF:ALT_F1R2:ALT_F2R1:FOXOG:PGT:PID:QSS:REF_F1R2:REF_F2R1	0/1:33,3:0.079:3:0:1.00:0|1:80340247_ACATCCTGGCTGC_A:941,83:15:18	0/0:22,1:0.043:1:0:1.00:0|1:80340247_ACATCCTGGCTGC_A:619,28:13:9	0.3	Unknown	.	.	.	.	.	.	.	.
T32df	chr9	**1**	**12.5**	44634091	44634091	A	T	splicing	**Ddx6**	ENSMUSP00000099768	.	.	.	**NA**	9,44634091,A,T	NA	9qA5.2	.	0.25	.	42	chr9	44634091	.	A	T	.	clustered_events;t_lod_fstar	ECNT=2;HCNT=2;MAX_ED=4;MIN_ED=4;NLOD=11.74;TLOD=5.43	GT:AD:AF:ALT_F1R2:ALT_F2R1:FOXOG:PGT:PID:QSS:REF_F1R2:REF_F2R1	0/1:45,2:0.043:0:2:1.00:0|1:35945182_CT_C:1306,58:27:18	0/0:41,0:0.00:0:0:.:0|1:35945182_CT_C:1174,0:26:15	0.3	Unkown	.	.	.	.	.	.	.	.
T3df	chr7	**1**	**12.5**	109905219	109905219	A	C	exonic	**Dennd5a**	ENSMUSP00000034944	.	nonsynonymous SNV	Dennd5a:NM_001311139:exon14:c.T2614G:p.L872V,Dennd5a:NM_021494:exon15:c.T2686G:p.L896V	**L872V**	ENSMUSP00000034944,872,L,V	7,109905219,A,A	7qE3	.	0.25	.	45	chr7	109905219	.	A	A	.	alt_allele_in_normal;germline_risk	ECNT=1;HCNT=4;MAX_ED=.;MIN_ED=.;NLOD=0.93;TLOD=26.93	GT:AD:AF:ALT_F1R2:ALT_F2R1:FOXOG:QSS:REF_F1R2:REF_F2R1	0/1:47,9:0.161:6:3:0.667:1318,243:23:24	0/0:37,4:0.114:2:2:0.500:1054,113:19:18	2.4	Tolerated	0	Neutral	160	30	1	Tolerated	2.85	136

T3df	chr7	**1**	**12.5**	109905219	109905219	A	C	exonic	**Dennd5a**	ENSMUSP00000034944	.	nonsynonymous SNV	Dennd5a:NM_001311139:exon14:c.T2614G:p.L872V,Dennd5a:NM_021494:exon15:c.T2686G:p.L896V	**L896V**	ENSMUSP00000034944,896,L,V	7,109905219,A,A	7qE3	.	0.25	.	45	chr7	109905219	.	A	A	.	alt_allele_in_normal;germline_risk	ECNT=1;HCNT=4;MAX_ED=.;MIN_ED=.;NLOD=0.932;TLOD=26.93	GT:AD:AF:ALT_F1R2:ALT_F2R1:FOXOG:QSS:REF_F1R2:REF_F2R1	0/1:47,9:0.161:6:3:0.667:1318,243:23:24	0/0:37,4:0.114:2:2:0.500:1054,113:19:18	2.4	Tolerated	0	Neutral	160	30	1	Tolerated	2.85	136
T32df	chr7	**1**	**12.5**	131112069	131112069	T	A	exonic	**Dmbt1**	ENSMUSP00000078390	.	nonsynonymous SNV	Dmbt1:NM_007769:exon44:c.T4832A:p.L1611H,Dmbt1:NM_001347632:exon45:c.T4865A:p.L1622H	**L1611H**	ENSMUSP00000078390,1611,L,H	7,131112069,T,A	7qF3	.	0.25	.	38	chr7	131112069	.	T	A	.	PASS	DB;ECNT=1;HCNT=1;MAX_ED=.;MIN_ED=.;NLOD=9.57;TLOD=31.19	GT:AD:AF:ALT_F1R2:ALT_F2R1:FOXOG:QSS:REF_F1R2:REF_F2R1	0/1:19,12:0.400:7:5:0.417:505,342:12:7	0/0:34,0:0.00:0:0:.:854,0:11:23	2.1	Deleterious	-4.6	Deleterious	66	30	0.001	Damaging	2.91	57
T32df	chr7	**1**	**12.5**	131112069	131112069	T	A	exonic	**Dmbt1**	ENSMUSP00000128504	.	nonsynonymous SNV	Dmbt1:NM_007769:exon44:c.T4832A:p.L1611H,Dmbt1:NM_001347632:exon45:c.T4865A:p.L1622H	**L1622H**	ENSMUSP00000128504,1622,L,H	7,131112069,T,C	7qF3	.	0.25	.	38	chr7	131112069	.	T	C	.	str_contraction;t_lod_fstar	ECNT=1;HCNT=1;MAX_ED=.;MIN_ED=.;NLOD=6.02;RPA=7,6;RU=CTG;STR;TLOD=4.73	GT:AD:AF:ALT_F1R2:ALT_F2R1:FOXOG:QSS:REF_F1R2:REF_F2R1	0/1:54,2:0.038:0:2:.:1525,56:29:25	0/0:24,0:0.00:0:0:.:672,0:13:11	2.8	Deleterious	-4.53	Deleterious	66	30	0.002	Damaging	2.91	57
T2df	chr9	**1**	**12.5**	54387574	54387575	CA	-	exonic	**Dmxl2**	ENSMUSP00000133552	.	frameshift deletion	Dmxl2:NM_172771:exon29:c.7215_7216del:p.S2405fs	**S2405fs**	ENSMUSP00000133552,2405,S,.	9,54387574,TCA,A	9qA5.3	.	0.25	.	50	chr9	54387573	.	TCA	A	.	clustered_events;homologous_mapping_event;t_lod_fstar	ECNT=9;HCNT=1;MAX_ED=18;MIN_ED=2;NLOD=9.93;TLOD=5.49	GT:AD:AF:ALT_F1R2:ALT_F2R1:FOXOG:PGT:PID:QSS:REF_F1R2:REF_F2R1	0/1:42,2:0.047:0:2:1.00:0|1:75184494_A_T:1010,24:24:18	0/0:34,0:0.00:0:0:.:0|1:75184494_A_T:882,0:16:18	0.4	Unknown	NA	NA	143	30	NA	NA	NA	NA
T2df	chr11	**1**	**12.5**	118057025	118057025	-	A	exonic	**Dnah17**	ENSMUSP00000115074	.	frameshift insertion	Dnah17:NM_001167746:exon57:c.8947_8948insT:p.N2983fs	**N2983fs**	ENSMUSP00000115074,2983,N,	11,118057025,T,G	11qE2	.	0.25	.	34	chr11	118057025	.	T	G	.	t_lod_fstar	ECNT=1;HCNT=1;MAX_ED=.;MIN_ED=.;NLOD=10.18;TLOD=5.56	GT:AD:AF:ALT_F1R2:ALT_F2R1:FOXOG:QSS:REF_F1R2:REF_F2R1	0/1:18,3:0.150:0:3:0.00:520,78:4:14	0/0:37,0:0.00:0:0:.:1028,0:27:10	18	Deleterious	-5.36	Deleterious	148	30	0.001	Damaging	2.81	189
T2df	chr11	**1**	**12.5**	118057034	118057034	G	T	exonic	**Dnah17**	ENSMUSP00000114670	.	nonsynonymous SNV	Dnah17:NM_001167746:exon57:c.C8939A:p.T2980N	**T2980N**	ENSMUSP00000114670,2980,T,N	11,118057034,G,G	11qE2	.	0.25	.	37	chr11	118057034	.	G	G	.	clustered_events;homologous_mapping_event;t_lod_fstar	ECNT=8;HCNT=2;MAX_ED=15;MIN_ED=2;NLOD=10.23;TLOD=5.27	GT:AD:AF:ALT_F1R2:ALT_F2R1:FOXOG:PGT:PID:QSS:REF_F1R2:REF_F2R1	0/1:55,2:0.036:2:0:.:0|1:118057025_T_TA:1580,51:31:24	0/0:37,0:0.00:0:0:.:0|1:118057025_T_TA:998,0:27:10	6	Tolerated	0	Neutral	148	30	1	Tolerated	2.81	189
T2df	chr11	**1**	**12.5**	118057036	118057036	G	C	exonic	**Dnah17**	ENSMUSP00000114670	.	nonsynonymous SNV	Dnah17:NM_001167746:exon57:c.C8937G:p.H2979Q	**H2979Q**	ENSMUSP00000114670,2979,H,Q	11,118057036,G,A	11qE2	.	0.25	.	38	chr11	118057036	.	G	A	.	clustered_events;homologous_mapping_event;t_lod_fstar	ECNT=8;HCNT=2;MAX_ED=15;MIN_ED=2;NLOD=10.54;TLOD=5.18	GT:AD:AF:ALT_F1R2:ALT_F2R1:FOXOG:PGT:PID:QSS:REF_F1R2:REF_F2R1	0/1:60,2:0.033:2:0:.:0|1:118057025_T_TA:1705,27:33:27	0/0:40,0:0.00:0:0:.:0|1:118057025_T_TA:1110,0:30:10	6	Tolerated	0	Neutral	148	30	1	Tolerated	2.81	189
T2df	chr11	**1**	**12.5**	118057039	118057039	-	TTGAAAAA	exonic	**Dnah17**	ENSMUSP00000114670	.	frameshift insertion	Dnah17:NM_001167746:exon57:c.8933_8934insTTTTTCAA:p.V2978fs	**V2978fs**	ENSMUSP00000114670,2978,V,	11,118057039,C,A	11qE2	.	0.25	.	38	chr11	118057039	.	C	A	.	clustered_events;homologous_mapping_event;t_lod_fstar	ECNT=9;HCNT=1;MAX_ED=18;MIN_ED=2;NLOD=9.93;TLOD=5.49	GT:AD:AF:ALT_F1R2:ALT_F2R1:FOXOG:PGT:PID:QSS:REF_F1R2:REF_F2R1	0/1:42,2:0.047:0:2:1.00:0|1:75184494_A_T:1010,24:24:18	0/0:34,0:0.00:0:0:.:0|1:75184494_A_T:882,0:16:18	0.4	Tolerated	0	Neutral	148	30	1	Tolerated	2.81	189
T2df	chr11	**1**	**12.5**	118057028	118057028	A	-	exonic	**Dnah17**	ENSMUSP00000114670	.	frameshift deletion	Dnah17:NM_001167746:exon57:c.8945delT:p.V2982fs	**V2982fs**	ENSMUSP00000114670,2982,V,.	11,118057028,GA,TA	11qE2	.	0.25	.	37	chr11	118057027	.	GA	TA	.	clustered_events;homologous_mapping_event;t_lod_fstar	ECNT=8;HCNT=2;MAX_ED=15;MIN_ED=2;NLOD=10.54;TLOD=5.29	GT:AD:AF:ALT_F1R2:ALT_F2R1:FOXOG:PGT:PID:QSS:REF_F1R2:REF_F2R1	0/1:53,2:0.037:2:0:.:0|1:118057025_T_TA:1473,56:30:23	0/0:34,0:0.00:0:0:.:0|1:118057025_T_TA:904,0:25:9	6	Unknown	.	.	.	.	.	.	.	.
T2df	chr11	**1**	**12.5**	118057041	118057048	CGTAGGCC	-	exonic	**Dnah17**	ENSMUSP00000114670	.	frameshift deletion	Dnah17:NM_001167746:exon57:c.8925_8932del:p.M2975fs	**M2975fs**	ENSMUSP00000114670,2975,M,.	11,118057041,ACGTAGGCC,CTTGAAAAA	11qE2	.	0.25	.	40	chr11	118057040	.	ACGTAGGCC	CTTGAAAAA	.	clustered_events;homologous_mapping_event;t_lod_fstar	ECNT=8;HCNT=2;MAX_ED=15;MIN_ED=2;NLOD=9.93;TLOD=5.24	GT:AD:AF:ALT_F1R2:ALT_F2R1:FOXOG:PGT:PID:QSS:REF_F1R2:REF_F2R1	0/1:57,2:0.035:2:0:.:0|1:118057025_T_TA:1676,58:32:25	0/0:38,0:0.00:0:0:.:0|1:118057025_T_TA:1019,0:29:9	6	Unknown	.	.	.	.	.	.	.	.

T1df	chr3	**1**	**12.5**	122276311	122276313	GGT	-	exonic	**Dnttip2**	ENSMUSP00000132108	.	nonframeshift deletion	Dnttip2:NM_153806:exon2:c.1174_1176del:p.392_392del	**392_392del**	NA	3,122276311,CGGT,A	3qG1	.	0.25	.	34	chr3	122276310	.	CGGT	A	.	alt_allele_in_normal;clustered_events;t_lod_fstar	ECNT=21;HCNT=36;MAX_ED=213;MIN_ED=12;NLOD=9.06;TLOD=5.10	GT:AD:AF:ALT_F1R2:ALT_F2R1:FOXOG:PGT:PID:QSS:REF_F1R2:REF_F2R1	0/1:66,2:0.030:0:2:1.00:0|1:37309309_G_A:1846,56:30:36	0/0:61,2:0.033:0:2:1.00:0|1:37309309_G_A:1723,56:29:32	0.7	Unknown	.	.	.	.	.	.	.	.
T42df	chr14	**1**	**12.5**	121559025	121559025	C	A	exonic	**Dock9**	ENSMUSP00000081556	.	nonsynonymous SNV	Dock9:NM_001128308:exon48:c.G5440T:p.G1814C,Dock9:NM_001347596:exon48:c.G5464T:p.G1822C,Dock9:NM_134074:exon48:c.G5374T:p.G1792C,Dock9:NM_001081039:exon49:c.G5539T:p.G1847C,Dock9:NM_001128307:exon50:c.G5479T:p.G1827C	**G1827C**	ENSMUSP00000081556,1827,G,C	14,121559025,C,G	14qE5	.	0.25	.	71	chr14	121559025	.	C	G	.	PASS	ECNT=1;HCNT=1;MAX_ED=.;MIN_ED=.;NLOD=12.59;TLOD=6.94	GT:AD:AF:ALT_F1R2:ALT_F2R1:FOXOG:QSS:REF_F1R2:REF_F2R1	0/1:47,4:0.083:0:4:1.00:1331,110:24:23	0/0:46,0:0.00:0:0:.:1292,0:25:21	19	Deleterious	-6.93	Deleterious	223	30	0	Damaging	2.84	189
T42df	chr14	**1**	**12.5**	121559025	121559025	C	A	exonic	**Dock9**	ENSMUSP00000081556	.	nonsynonymous SNV	Dock9:NM_001128308:exon48:c.G5440T:p.G1814C,Dock9:NM_001347596:exon48:c.G5464T:p.G1822C,Dock9:NM_134074:exon48:c.G5374T:p.G1792C,Dock9:NM_001081039:exon49:c.G5539T:p.G1847C,Dock9:NM_001128307:exon50:c.G5479T:p.G1827C	**G1847C**	ENSMUSP00000081556,1847,G,C	14,121559025,C,G	14qE5	.	0.25	.	71	chr14	121559025	.	C	G	.	PASS	ECNT=1;HCNT=1;MAX_ED=.;MIN_ED=.;NLOD=12.59;TLOD=6.94	GT:AD:AF:ALT_F1R2:ALT_F2R1:FOXOG:QSS:REF_F1R2:REF_F2R1	0/1:47,4:0.083:0:4:1.00:1331,110:24:23	0/0:46,0:0.00:0:0:.:1292,0:25:21	19	Deleterious	-6.93	Deleterious	223	30	0	Damaging	2.84	189
T42df	chr14	**1**	**12.5**	121559024	121559024	C	A	exonic	**Dock9**	ENSMUSP00000081556	.	nonsynonymous SNV	Dock9:NM_001128308:exon48:c.G5441T:p.G1814V,Dock9:NM_001347596:exon48:c.G5465T:p.G1822V,Dock9:NM_134074:exon48:c.G5375T:p.G1792V,Dock9:NM_001081039:exon49:c.G5540T:p.G1847V,Dock9:NM_001128307:exon50:c.G5480T:p.G1827V	**G1792V**	ENSMUSP00000081556,1792,G,V	14,121559024,C,A	14qE5	.	0.25	.	71	chr14	121559024	.	C	A	.	t_lod_fstar	ECNT=2;HCNT=1;MAX_ED=1;MIN_ED=1;NLOD=20.17;TLOD=5.29	GT:AD:AF:ALT_F1R2:ALT_F2R1:FOXOG:PGT:PID:QSS:REF_F1R2:REF_F2R1	0/1:55,2:0.037:2:0:0.00:0|1:121559024_C_A:1517,56:30:25	0/0:71,0:0.00:0:0:.:0|1:121559024_C_A:1893,0:33:38	2	Deleterious	-7.8	Deleterious	223	30	0	Damaging	2.84	189
T42df	chr14	**1**	**12.5**	121559024	121559024	C	A	exonic	**Dock9**	ENSMUSP00000081556	.	nonsynonymous SNV	Dock9:NM_001128308:exon48:c.G5441T:p.G1814V,Dock9:NM_001347596:exon48:c.G5465T:p.G1822V,Dock9:NM_134074:exon48:c.G5375T:p.G1792V,Dock9:NM_001081039:exon49:c.G5540T:p.G1847V,Dock9:NM_001128307:exon50:c.G5480T:p.G1827V	**G1814V**	ENSMUSP00000081556,1814,G,V	14,121559024,C,A	14qE5	.	0.25	.	71	chr14	121559024	.	C	A	.	t_lod_fstar	ECNT=2;HCNT=1;MAX_ED=1;MIN_ED=1;NLOD=20.17;TLOD=5.29	GT:AD:AF:ALT_F1R2:ALT_F2R1:FOXOG:PGT:PID:QSS:REF_F1R2:REF_F2R1	0/1:55,2:0.037:2:0:0.00:0|1:121559024_C_A:1517,56:30:25	0/0:71,0:0.00:0:0:.:0|1:121559024_C_A:1893,0:33:38	2	Deleterious	-7.8	Deleterious	223	30	0	Damaging	2.84	189
T42df	chr14	**1**	**12.5**	121559024	121559024	C	A	exonic	**Dock9**	ENSMUSP00000081556	.	nonsynonymous SNV	Dock9:NM_001128308:exon48:c.G5441T:p.G1814V,Dock9:NM_001347596:exon48:c.G5465T:p.G1822V,Dock9:NM_134074:exon48:c.G5375T:p.G1792V,Dock9:NM_001081039:exon49:c.G5540T:p.G1847V,Dock9:NM_001128307:exon50:c.G5480T:p.G1827V	**G1822V**	ENSMUSP00000081556,1822,G,V	14,121559024,C,A	14qE5	.	0.25	.	71	chr14	121559024	.	C	A	.	t_lod_fstar	ECNT=2;HCNT=1;MAX_ED=1;MIN_ED=1;NLOD=20.17;TLOD=5.29	GT:AD:AF:ALT_F1R2:ALT_F2R1:FOXOG:PGT:PID:QSS:REF_F1R2:REF_F2R1	0/1:55,2:0.037:2:0:0.00:0|1:121559024_C_A:1517,56:30:25	0/0:71,0:0.00:0:0:.:0|1:121559024_C_A:1893,0:33:38	2	Deleterious	-7.8	Deleterious	223	30	0	Damaging	2.84	189
T42df	chr14	**1**	**12.5**	121559024	121559024	C	A	exonic	**Dock9**	ENSMUSP00000081556	.	nonsynonymous SNV	Dock9:NM_001128308:exon48:c.G5441T:p.G1814V,Dock9:NM_001347596:exon48:c.G5465T:p.G1822V,Dock9:NM_134074:exon48:c.G5375T:p.G1792V,Dock9:NM_001081039:exon49:c.G5540T:p.G1847V,Dock9:NM_001128307:exon50:c.G5480T:p.G1827V	**G1827V**	ENSMUSP00000081556,1827,G,V	14,121559024,C,A	14qE5	.	0.25	.	71	chr14	121559024	.	C	A	.	t_lod_fstar	ECNT=2;HCNT=1;MAX_ED=1;MIN_ED=1;NLOD=20.17;TLOD=5.29	GT:AD:AF:ALT_F1R2:ALT_F2R1:FOXOG:PGT:PID:QSS:REF_F1R2:REF_F2R1	0/1:55,2:0.037:2:0:0.00:0|1:121559024_C_A:1519,56:30:25	0/0:71,0:0.00:0:0:.:0|1:121559024_C_A:1970,0:33:38	2	Deleterious	-7.8	Deleterious	223	30	0	Damaging	2.84	189
T42df	chr14	**1**	**12.5**	121559024	121559024	C	A	exonic	**Dock9**	ENSMUSP00000081556	.	nonsynonymous SNV	Dock9:NM_001128308:exon48:c.G5441T:p.G1814V,Dock9:NM_001347596:exon48:c.G5465T:p.G1822V,Dock9:NM_134074:exon48:c.G5375T:p.G1792V,Dock9:NM_001081039:exon49:c.G5540T:p.G1847V,Dock9:NM_001128307:exon50:c.G5480T:p.G1827V	**G1847V**	ENSMUSP00000081556,1847,G,V	14,121559024,C,A	14qE5	.	0.25	.	71	chr14	121559024	.	C	A	.	t_lod_fstar	ECNT=2;HCNT=1;MAX_ED=1;MIN_ED=1;NLOD=20.17;TLOD=5.29	GT:AD:AF:ALT_F1R2:ALT_F2R1:FOXOG:PGT:PID:QSS:REF_F1R2:REF_F2R1	0/1:55,2:0.037:2:0:0.00:0|1:121559024_C_A:1519,56:30:25	0/0:71,0:0.00:0:0:.:0|1:121559024_C_A:1970,0:33:38	2	Deleterious	-7.8	Deleterious	223	30	0	Damaging	2.84	189

T42df	chr14	**1**	**12.5**	121559025	121559025	C	A	exonic	**Dock9**	ENSMUSP00000081556	.	nonsynonymous SNV	Dock9:NM_001128308:exon48:c.G5440T:p.G1814C,Dock9:NM_001347596:exon48:c.G5464T:p.G1822C,Dock9:NM_134074:exon48:c.G5374T:p.G1792C,Dock9:NM_001081039:exon49:c.G5539T:p.G1847C,Dock9:NM_001128307:exon50:c.G5479T:p.G1827C	**G1792C**	ENSMUSP00000081556,1792,G,C	14,121559025,C,A	14qE5	.	0.25	.	71	chr14	121559025	.	C	A	.	t_lod_fstar	ECNT=2;HCNT=1;MAX_ED=1;MIN_ED=1;NLOD=20.17;TLOD=5.29	GT:AD:AF:ALT_F1R2:ALT_F2R1:FOXOG:PGT:PID:QSS:REF_F1R2:REF_F2R1	0/1:55,2:0.037:2:0:0.00:0|1:121559024_C_A:1519,56:30:25	0/0:71,0:0.00:0:0:.:0|1:121559024_C_A:1970,0:33:38	2	Deleterious	-7.8	Deleterious	223	30	0	Damaging	2.84	189
T42df	chr14	**1**	**12.5**	121559025	121559025	C	A	exonic	**Dock9**	ENSMUSP00000081556	.	nonsynonymous SNV	Dock9:NM_001128308:exon48:c.G5440T:p.G1814C,Dock9:NM_001347596:exon48:c.G5464T:p.G1822C,Dock9:NM_134074:exon48:c.G5374T:p.G1792C,Dock9:NM_001081039:exon49:c.G5539T:p.G1847C,Dock9:NM_001128307:exon50:c.G5479T:p.G1827C	**G1814C**	ENSMUSP00000081556,1814,G,C	14,121559025,C,A	14qE5	.	0.25	.	71	chr14	121559025	.	C	A	.	t_lod_fstar	ECNT=2;HCNT=1;MAX_ED=1;MIN_ED=1;NLOD=20.17;TLOD=5.29	GT:AD:AF:ALT_F1R2:ALT_F2R1:FOXOG:PGT:PID:QSS:REF_F1R2:REF_F2R1	0/1:55,2:0.037:2:0:0.00:0|1:121559024_C_A:1517,56:30:25	0/0:71,0:0.00:0:0:.:0|1:121559024_C_A:1893,0:33:38	2	Deleterious	-7.8	Deleterious	223	30	0	Damaging	2.84	189
T42df	chr14	**1**	**12.5**	121559025	121559025	C	A	exonic	**Dock9**	ENSMUSP00000081556	.	nonsynonymous SNV	Dock9:NM_001128308:exon48:c.G5440T:p.G1814C,Dock9:NM_001347596:exon48:c.G5464T:p.G1822C,Dock9:NM_134074:exon48:c.G5374T:p.G1792C,Dock9:NM_001081039:exon49:c.G5539T:p.G1847C,Dock9:NM_001128307:exon50:c.G5479T:p.G1827C	**G1822C**	ENSMUSP00000081556,1822,G,C	14,121559025,C,A	14qE5	.	0.25	.	71	chr14	121559025	.	C	A	.	t_lod_fstar	ECNT=2;HCNT=1;MAX_ED=1;MIN_ED=1;NLOD=20.17;TLOD=5.29	GT:AD:AF:ALT_F1R2:ALT_F2R1:FOXOG:PGT:PID:QSS:REF_F1R2:REF_F2R1	0/1:55,2:0.037:2:0:0.00:0|1:121559024_C_A:1519,56:30:25	0/0:71,0:0.00:0:0:.:0|1:121559024_C_A:1970,0:33:38	2	Deleterious	-7.8	Deleterious	223	30	0	Damaging	2.84	189
T2df	chr16	**1**	**12.5**	48291164	48291165	GA	-	exonic	**Dppa4**	ENSMUSP00000113693	.	frameshift deletion	Dppa4:NM_001018002:exon4:c.355_356del:p.D119fs,Dppa4:NM_028610:exon5:c.511_512del:p.D171fs	**D119fs**	ENSMUSP00000113693,119,D,.	NA	16qB5	.	0.25	.	62	chr16	48291163	.	TGA	T	.	alt_allele_in_normal;clustered_events	ECNT=11;HCNT=16;MAX_ED=75;MIN_ED=11;NLOD=3.66;TLOD=8.37	GT:AD:AF:ALT_F1R2:ALT_F2R1:FOXOG:PGT:PID:QSS:REF_F1R2:REF_F2R1	0/1:55,3:0.052:1:2:.:0|1:48291150_TA_T:1492,84:30:25	0/0:59,3:0.053:2:1:.:0|1:48291150_TA_T:1619,87:24:35	1	Deleterious	-10.59	Deleterious	143	30	NA	NA	NA	NA
T2df	chr16	**1**	**12.5**	48291162	48291162	T	C	exonic	**Dppa4**	ENSMUSP00000113693	.	nonsynonymous SNV	Dppa4:NM_001018002:exon4:c.T353C:p.I118T,Dppa4:NM_028610:exon5:c.T509C:p.I170T	**I118T**	ENSMUSP00000113693,118,I,T	16,48291162,T,C	16qB5	.	0.25	.	61	chr16	48291162	.	T	C	.	alt_allele_in_normal;clustered_events	ECNT=11;HCNT=16;MAX_ED=75;MIN_ED=11;NLOD=3.91;TLOD=8.42	GT:AD:AF:ALT_F1R2:ALT_F2R1:FOXOG:PGT:PID:QSS:REF_F1R2:REF_F2R1	0/1:55,3:0.054:1:2:0.333:0|1:48291150_TA_T:1549,87:29:26	0/0:61,3:0.052:2:1:0.667:0|1:48291150_TA_T:1665,77:26:35	1	Tolerated	0.53	Neutral	73	30	0.646	Tolerated	3.47	38
T2df	chr16	**1**	**12.5**	48291104	48291104	G	C	exonic	**Dppa4**	ENSMUSP00000113693	.	nonsynonymous SNV	Dppa4:NM_001018002:exon4:c.G295C:p.E99Q,Dppa4:NM_028610:exon5:c.G451C:p.E151Q	**E151Q**	ENSMUSP00000113693,151,E,Q	16,48291104,G,C	16qB5	.	0.25	.	47	chr16	48291104	.	G	C	.	alt_allele_in_normal;clustered_events	ECNT=11;HCNT=16;MAX_ED=75;MIN_ED=11;NLOD=3.36;TLOD=8.37	GT:AD:AF:ALT_F1R2:ALT_F2R1:FOXOG:PGT:PID:QSS:REF_F1R2:REF_F2R1	0/1:56,3:0.052:1:2:0.333:0|1:48291150_TA_T:1533,84:31:25	0/0:58,3:0.054:2:1:0.667:0|1:48291150_TA_T:1625,84:24:34	1	Tolerated	-0.43	Neutral	73	30	0.819	Tolerated	3.48	38
T2df	chr16	**1**	**12.5**	48291104	48291104	G	C	exonic	**Dppa4**	ENSMUSP00000113693	.	nonsynonymous SNV	Dppa4:NM_001018002:exon4:c.G295C:p.E99Q,Dppa4:NM_028610:exon5:c.G451C:p.E151Q	**E99Q**	ENSMUSP00000113693,99,E,Q	16,48291104,G,C	16qB5	.	0.25	.	47	chr16	48291104	.	G	C	.	alt_allele_in_normal;clustered_events	ECNT=11;HCNT=16;MAX_ED=75;MIN_ED=11;NLOD=3.36;TLOD=8.37	GT:AD:AF:ALT_F1R2:ALT_F2R1:FOXOG:PGT:PID:QSS:REF_F1R2:REF_F2R1	0/1:56,3:0.052:1:2:0.333:0|1:48291150_TA_T:1533,84:31:25	0/0:58,3:0.054:2:1:0.667:0|1:48291150_TA_T:1625,84:24:34	1	Tolerated	-0.43	Neutral	73	30	0.819	Tolerated	3.48	38
T2df	chr16	**1**	**12.5**	48291153	48291153	C	T	exonic	**Dppa4**	ENSMUSP00000113693	.	nonsynonymous SNV	Dppa4:NM_001018002:exon4:c.C344T:p.P115L,Dppa4:NM_028610:exon5:c.C500T:p.P167L	**P115L**	ENSMUSP00000113693,115,P,L	16,48291153,C,C	16qB5	.	0.25	.	57	chr16	48291153	.	C	C	.	alt_allele_in_normal;clustered_events	ECNT=11;HCNT=16;MAX_ED=75;MIN_ED=11;NLOD=4.21;TLOD=8.42	GT:AD:AF:ALT_F1R2:ALT_F2R1:FOXOG:PGT:PID:QSS:REF_F1R2:REF_F2R1	0/1:54,3:0.054:1:2:0.333:0|1:48291150_TA_T:1538,66:28:26	0/0:61,3:0.051:2:1:0.667:0|1:48291150_TA_T:1713,84:26:35	1	Tolerated	0	Neutral	73	30	1	Tolerated	3.46	40
T2df	chr16	**1**	**12.5**	48291153	48291153	C	T	exonic	**Dppa4**	ENSMUSP00000113693	.	nonsynonymous SNV	Dppa4:NM_001018002:exon4:c.C344T:p.P115L,Dppa4:NM_028610:exon5:c.C500T:p.P167L	**P167L**	ENSMUSP00000113693,167,P,L	16,48291153,C,C	16qB5	.	0.25	.	57	chr16	48291153	.	C	C	.	alt_allele_in_normal;clustered_events	ECNT=11;HCNT=16;MAX_ED=75;MIN_ED=11;NLOD=4.21;TLOD=8.42	GT:AD:AF:ALT_F1R2:ALT_F2R1:FOXOG:PGT:PID:QSS:REF_F1R2:REF_F2R1	0/1:54,3:0.054:1:2:0.333:0|1:48291150_TA_T:1538,66:28:26	0/0:61,3:0.051:2:1:0.667:0|1:48291150_TA_T:1713,84:26:35	1	Tolerated	0	Neutral	73	30	1	Tolerated	3.46	40

T2df	chr16	**1**	**12.5**	48291162	48291162	T	C	exonic	**Dppa4**	ENSMUSP00000113693	.	nonsynonymous SNV	Dppa4:NM_001018002:exon4:c.T353C:p.I118T,Dppa4:NM_028610:exon5:c.T509C:p.I170T	**I170T**	ENSMUSP00000113693,170,I,T	16,48291162,T,C	16qB5	.	0.25	.	61	chr16	48291162	.	T	C	.	alt_allele_in_normal;clustered_events	ECNT=11;HCNT=16;MAX_ED=75;MIN_ED=11;NLOD=3.91;TLOD=8.42	GT:AD:AF:ALT_F1R2:ALT_F2R1:FOXOG:PGT:PID:QSS:REF_F1R2:REF_F2R1	0/1:55,3:0.054:1:2:0.333:0|1:48291150_TA_T:1549,87:29:26	0/0:61,3:0.052:2:1:0.667:0|1:48291150_TA_T:1665,77:26:35	1	Tolerated	0.53	Neutral	73	30	0.646	Tolerated	3.47	38
T2df	chr16	**1**	**12.5**	48291174	48291174	T	C	exonic	**Dppa4**	ENSMUSP00000113693	.	nonsynonymous SNV	Dppa4:NM_001018002:exon4:c.T365C:p.M122T,Dppa4:NM_028610:exon5:c.T521C:p.M174T	**M122T**	ENSMUSP00000113693,122,M,T	16,48291174,T,C	16qB5	.	0.25	.	64	chr16	48291174	.	T	C	.	alt_allele_in_normal;clustered_events;t_lod_fstar	ECNT=11;HCNT=16;MAX_ED=75;MIN_ED=11;NLOD=3.41;TLOD=5.01	GT:AD:AF:ALT_F1R2:ALT_F2R1:FOXOG:QSS:REF_F1R2:REF_F2R1	0/1:49,3:0.060:1:2:0.333:1426,90:25:24	0/0:44,3:0.068:2:1:0.667:1264,84:19:25	1	Tolerated	0.19	Neutral	73	30	0.204	Tolerated	3.48	35
T2df	chr16	**1**	**12.5**	48291174	48291174	T	C	exonic	**Dppa4**	ENSMUSP00000113693	.	nonsynonymous SNV	Dppa4:NM_001018002:exon4:c.T365C:p.M122T,Dppa4:NM_028610:exon5:c.T521C:p.M174T	**M174T**	ENSMUSP00000113693,174,M,T	16,48291174,T,T	16qB5	.	0.25	.	64	chr16	48291174	.	T	T	.	alt_allele_in_normal;clustered_events;germline_risk	ECNT=11;HCNT=16;MAX_ED=75;MIN_ED=11;NLOD=1.25;TLOD=8.37	GT:AD:AF:ALT_F1R2:ALT_F2R1:FOXOG:PGT:PID:QSS:REF_F1R2:REF_F2R1	0/1:55,3:0.052:1:2:.:0|1:48291150_TA_T:1553,85:29:26	0/0:54,3:0.061:2:1:.:0|1:48291150_TA_T:1520,86:23:31	1	Tolerated	0	Neutral	73	30	0.386	Tolerated	3.48	35
T2df	chr16	**1**	**12.5**	48291179	48291179	T	C	exonic	**Dppa4**	ENSMUSP00000113693	.	nonsynonymous SNV	Dppa4:NM_001018002:exon4:c.T370C:p.Y124H,Dppa4:NM_028610:exon5:c.T526C:p.Y176H	**Y124H**	ENSMUSP00000113693,124,Y,H	16,48291179,T,T	16qB5	.	0.25	.	64	chr16	48291179	.	T	T	.	alt_allele_in_normal;clustered_events;germline_risk	ECNT=11;HCNT=16;MAX_ED=75;MIN_ED=11;NLOD=1.85;TLOD=8.35	GT:AD:AF:ALT_F1R2:ALT_F2R1:FOXOG:PGT:PID:QSS:REF_F1R2:REF_F2R1	0/1:56,3:0.051:1:2:0.667:0|1:48291150_TA_T:1559,82:30:26	0/0:54,3:0.059:2:1:0.333:0|1:48291150_TA_T:1485,87:23:31	1	Tolerated	0	Neutral	73	30	1	Tolerated	3.42	36
T2df	chr16	**1**	**12.5**	48291179	48291179	T	C	exonic	**Dppa4**	ENSMUSP00000113693	.	nonsynonymous SNV	Dppa4:NM_001018002:exon4:c.T370C:p.Y124H,Dppa4:NM_028610:exon5:c.T526C:p.Y176H	**Y176H**	ENSMUSP00000113693,176,Y,H	16,48291179,T,T	16qB5	.	0.25	.	64	chr16	48291179	.	T	T	.	alt_allele_in_normal;clustered_events	ECNT=11;HCNT=16;MAX_ED=75;MIN_ED=11;NLOD=3.66;TLOD=8.37	GT:AD:AF:ALT_F1R2:ALT_F2R1:FOXOG:PGT:PID:QSS:REF_F1R2:REF_F2R1	0/1:55,3:0.052:1:2:.:0|1:48291150_TA_T:1492,84:30:25	0/0:59,3:0.053:2:1:.:0|1:48291150_TA_T:1619,87:24:35	1	Tolerated	0	Neutral	73	30	1	Tolerated	3.42	36
T2df	chr16	**1**	**12.5**	48291166	48291166	-	GT	exonic	**Dppa4**	ENSMUSP00000113693	.	frameshift insertion	Dppa4:NM_001018002:exon4:c.357_358insGT:p.D119fs,Dppa4:NM_028610:exon5:c.513_514insGT:p.D171fs	**D171fs**	ENSMUSP00000113693,171,D,	16,48291166,C,T	16qB5	.	0.25	.	60	chr16	48291166	.	C	T	.	alt_allele_in_normal;clustered_events;germline_risk	ECNT=11;HCNT=16;MAX_ED=75;MIN_ED=11;NLOD=1.85;TLOD=8.35	GT:AD:AF:ALT_F1R2:ALT_F2R1:FOXOG:PGT:PID:QSS:REF_F1R2:REF_F2R1	0/1:56,3:0.051:1:2:0.667:0|1:48291150_TA_T:1559,82:30:26	0/0:54,3:0.059:2:1:0.333:0|1:48291150_TA_T:1485,87:23:31	1	Tolerated	0	Neutral	73	30	1	Tolerated	3.48	35
T2df	chr16	**1**	**12.5**	48291115	48291115	-	C	exonic	**Dppa4**	ENSMUSP00000113705	.	frameshift insertion	Dppa4:NM_001018002:exon4:c.307dupC:p.V102fs,Dppa4:NM_028610:exon5:c.463dupC:p.V154fs	**V154fs**	ENSMUSP00000113705,154,V,	16,48291115,T,A	16qB5	.	0.25	.	53	chr16	48291115	.	T	A	.	PASS	ECNT=1;HCNT=1;MAX_ED=.;MIN_ED=.;NLOD=9.53;TLOD=7.02	GT:AD:AF:ALT_F1R2:ALT_F2R1:FOXOG:QSS:REF_F1R2:REF_F2R1	0/1:52,4:0.056:3:1:.:1441,115:24:28	0/0:36,0:0.00:0:0:.:957,0:24:12	1	Tolerated	0	Neutral	73	30	0.928	Tolerated	3.48	39
T2df	chr16	**1**	**12.5**	48291115	48291115	-	C	exonic	**Dppa4**	ENSMUSP00000113693	.	frameshift insertion	Dppa4:NM_001018002:exon4:c.307dupC:p.V102fs,Dppa4:NM_028610:exon5:c.463dupC:p.V154fs	**V102fs**	ENSMUSP00000113693,102,V,	16,48291115,T,TC	16qB5	.	0.25	.	53	chr16	48291115	.	T	TC	.	alt_allele_in_normal;clustered_events;germline_risk	ECNT=11;HCNT=16;MAX_ED=75;MIN_ED=11;NLOD=0.647;RPA=2,3;RU=C;STR;TLOD=8.49	GT:AD:AF:ALT_F1R2:ALT_F2R1:FOXOG:PGT:PID:QSS:REF_F1R2:REF_F2R1	0/1:53,3:0.057:1:2:.:0|1:48291115_T_TC:1475,84:27:26	0/0:50,3:0.064:2:1:.:0|1:48291115_T_TC:1395,82:23:27	1	Unknown	NA	NA	73	30	NA	NA	NA	NA
T2df	chr16	**1**	**12.5**	48291166	48291166	-	GT	exonic	**Dppa4**	ENSMUSP00000113693	.	frameshift insertion	Dppa4:NM_001018002:exon4:c.357_358insGT:p.D119fs,Dppa4:NM_028610:exon5:c.513_514insGT:p.D171fs	**D119fs**	ENSMUSP00000113693,119,D,	16,48291166,C,CGT	16qB5	.	0.25	.	60	chr16	48291166	.	C	CGT	.	alt_allele_in_normal;clustered_events	ECNT=11;HCNT=16;MAX_ED=75;MIN_ED=11;NLOD=3.36;TLOD=8.42	GT:AD:AF:ALT_F1R2:ALT_F2R1:FOXOG:PGT:PID:QSS:REF_F1R2:REF_F2R1	0/1:55,3:0.054:1:2:.:0|1:48291150_TA_T:1513,66:30:25	0/0:57,3:0.054:2:1:.:0|1:48291150_TA_T:1544,86:24:33	1	Unknown	NA	NA	73	30	NA	NA	NA	NA
T2df	chr16	**1**	**12.5**	48291164	48291165	GA	-	exonic	**Dppa4**	ENSMUSP00000113693	.	frameshift deletion	Dppa4:NM_001018002:exon4:c.355_356del:p.D119fs,Dppa4:NM_028610:exon5:c.511_512del:p.D171fs	**D171fs**	ENSMUSP00000113693,171,D,.	16,48291164,TGA,CGT	16qB5	.	0.25	.	62	chr16	48291163	.	TGA	CGT	.	alt_allele_in_normal;clustered_events	ECNT=11;HCNT=16;MAX_ED=75;MIN_ED=11;NLOD=3.36;TLOD=8.42	GT:AD:AF:ALT_F1R2:ALT_F2R1:FOXOG:PGT:PID:QSS:REF_F1R2:REF_F2R1	0/1:55,3:0.054:1:2:.:0|1:48291150_TA_T:1513,66:30:25	0/0:57,3:0.054:2:1:.:0|1:48291150_TA_T:1544,86:24:33	1	Unknown	.	.	.	.	.	.	.	.

T2df	chr16	1	12.5	48291151	48291151	A	-	exonic	**Dppa4**	ENSMUSP00000113693	.	frameshift deletion	Dppa4:NM_001018002:exon4:c.342delA:p.I114fs,Dppa4:NM_028610:exon5:c.498delA:p.I166fs	**I114fs**	ENSMUSP00000113693,114,I,.	16,48291151,TA,T	16qB5	.	0.25	.	57	chr16	48291150	.	TA	T	.	alt_allele_in_normal;clustered_events;germline_risk	ECNT=11;HCNT=16;MAX_ED=75;MIN_ED=11;NLOD=1.25;TLOD=8.37	GT:AD:AF:ALT_F1R2:ALT_F2R1:FOXOG:PGT:PID:QSS:REF_F1R2:REF_F2R1	0/1:55,3:0.052:1:2:.:0|1:48291150_TA_T:1553,85:29:26	0/0:54,3:0.061:2:1:.:0|1:48291150_TA_T:1520,86:23:31	1	Unknown	NA	NA	73	30	NA	NA	NA	NA
T2df	chr16	1	12.5	48291151	48291151	A	-	exonic	**Dppa4**	ENSMUSP00000113693	.	frameshift deletion	Dppa4:NM_001018002:exon4:c.342delA:p.I114fs,Dppa4:NM_028610:exon5:c.498delA:p.I166fs	**I166fs**	ENSMUSP00000113693,166,I,.	16,48291151,TA,TC	16qB5	.	0.25	.	57	chr16	48291150	.	TA	TC	.	alt_allele_in_normal;clustered_events;germline_risk	ECNT=11;HCNT=16;MAX_ED=75;MIN_ED=11;NLOD=0.647;RPA=2,3;RU=C;STR;TLOD=8.49	GT:AD:AF:ALT_F1R2:ALT_F2R1:FOXOG:PGT:PID:QSS:REF_F1R2:REF_F2R1	0/1:53,3:0.057:1:2:.:0|1:48291115_T_TC:1475,84:27:26	0/0:50,3:0.064:2:1:.:0|1:48291115_T_TC:1395,82:23:27	1	Unknown	.	.	.	.	.	.	.	.
T12df	chr7	1	12.5	142082155	142082157	CCA	-	exonic	**Dusp8**	ENSMUSP00000113484	.	nonframeshift deletion	Dusp8:NM_008748:exon7:c.1695_1697del:p.565_566del	**565_566del**	NA	7,142082155,GCCA,A	7qF5	.	0.25	.	9	chr7	142082154	.	GCCA	A	.	PASS	DB;ECNT=1;HCNT=1;MAX_ED=.;MIN_ED=.;NLOD=10.48;TLOD=64.39	GT:AD:AF:ALT_F1R2:ALT_F2R1:FOXOG:QSS:REF_F1R2:REF_F2R1	0/1:27,26:0.490:14:12:0.538:763,719:20:7	0/0:38,0:0.00:0:0:.:1036,0:16:22	4	Unknown	.	.	.	.	.	.	.	.
T3df	chr1	1	12.5	131129021	131129021	-	TTTA	exonic	**Dyrk3**	ENSMUSP00000028295	.	stopgain	Dyrk3:NM_145508:exon3:c.1413_1414insTAAA:p.K472_K473delinsX	**K472_K473delinsX**	ENSMUSP00000028295,472,KK,X	NA	1qE4	.	0.25	.	40	chr1	131129021	.	T	G	.	PASS	ECNT=1;HCNT=1;MAX_ED=.;MIN_ED=.;NLOD=12.59;TLOD=6.94	GT:AD:AF:ALT_F1R2:ALT_F2R1:FOXOG:QSS:REF_F1R2:REF_F2R1	0/1:47,4:0.083:0:4:1.00:1331,110:24:23	0/0:46,0:0.00:0:0:.:1292,0:25:21	19	Deletarious	.	.	.	.	.	.	.	.
T3df	chr1	1	12.5	131129033	131129033	-	TACG	exonic	**Dyrk3**	ENSMUSP00000028295	.	frameshift insertion	Dyrk3:NM_145508:exon3:c.1401_1402insCGTA:p.S468fs	**S468fs**	ENSMUSP00000028295,468,S,	1,131129033,A,G	1qE4	.	0.25	.	42	chr1	131129033	.	A	G	.	PASS	ECNT=1;HCNT=1;MAX_ED=.;MIN_ED=.;NLOD=12.59;TLOD=6.94	GT:AD:AF:ALT_F1R2:ALT_F2R1:FOXOG:QSS:REF_F1R2:REF_F2R1	0/1:47,4:0.083:0:4:1.00:1331,110:24:23	0/0:46,0:0.00:0:0:.:1292,0:25:21	19	Deleterious	-4.84	Deleterious	158	30	0.013	Damaging	2.95	289
T3df	chr1	1	12.5	131129023	131129030	CCCCTGCG	-	exonic	**Dyrk3**	ENSMUSP00000028295	.	frameshift deletion	Dyrk3:NM_145508:exon3:c.1405_1412del:p.R469fs	**R469fs**	ENSMUSP00000028295,469,R,.	1,131129023,ACCCCTGCG,TTTTA	1qE4	.	0.25	.	45	chr1	131129022	.	ACCCCTGCG	TTTTA	.	clustered_events;multi_event_alt_allele_in_normal;t_lod_fstar	ECNT=4;HCNT=1;MAX_ED=19;MIN_ED=1;NLOD=6.64;TLOD=5.94	GT:AD:AF:ALT_F1R2:ALT_F2R1:FOXOG:PGT:PID:QSS:REF_F1R2:REF_F2R1	0/1:26,2:0.077:2:0:.:0|1:131129021_T_TTTTA:710,44:14:12	0/0:39,1:0.027:1:0:.:0|1:131129021_T_TTTTA:1066,24:23:16	23	Unknown	NA	NA	158	30	NA	NA	NA	NA
T4df	chrX	1	12.5	97338876	97338876	-	T	exonic	**Eda2r**	ENSMUSP00000093748	.	frameshift insertion	Eda2r:NM_001161432:exon7:c.843_844insA:p.S282fs,Eda2r:NM_001161433:exon7:c.843_844insA:p.S282fs,Eda2r:NM_175540:exon7:c.843_844insA:p.S282fs	**S282fs**	ENSMUSP00000093748,282,S,	X,97338876,A,G	XqC3	.	0.25	.	33	chrX	97338876	.	A	G	.	t_lod_fstar	ECNT=1;HCNT=1;MAX_ED=.;MIN_ED=.;NLOD=10.30;TLOD=5.24	GT:AD:AF:ALT_F1R2:ALT_F2R1:FOXOG:QSS:REF_F1R2:REF_F2R1	0/1:18,7:0.280:7:0:1.00:506,107:4:14	0/0:39,0:0.00:0:0:.:1011,0:17:22	2.5	Damaging	-0.52	Neutral	164	30	0.005	Damaging	3.86	33
T2df	chr10	1	12.5	81178115	81178115	A	T	exonic	**Eef2**	ENSMUSP00000132786	.	nonsynonymous SNV	Eef2:NM_007907:exon3:c.A281T:p.D94V	**D94V**	ENSMUSP00000132786,94,D,V	10,81178115,A,C	10qC1	.	0.25	.	184	chr10	81178115	.	A	C	.	clustered_events;homologous_mapping_event;t_lod_fstar	ECNT=4;HCNT=4;MAX_ED=108;MIN_ED=6;NLOD=6.66;TLOD=5.35	GT:AD:AF:ALT_F1R2:ALT_F2R1:FOXOG:PGT:PID:QSS:REF_F1R2:REF_F2R1	0/1:50,2:0.040:1:1:0.500:0|1:113844430_G_T:1397,50:29:21	0/0:35,0:0.030:0:0:.:0|1:113844430_G_T:974,0:22:13	0.4	Deleterious	-2.85	Deleterious	706	30	0.022	Damaging	3.61	379
T1df	chr7	1	12.5	27165006	27165008	TGC	-	exonic	**Egln2**	ENSMUSP00000149771	.	nonframeshift deletion	Egln2:NM_053208:exon2:c.491_493del:p.164_165del	**164_165del**	NA	7,27165006,TTGC,C	7qA3	rs231887685	0.25	.	33	chr7	27165005	.	TTGC	C	.	alt_allele_in_normal;germline_risk	ECNT=1;HCNT=1;MAX_ED=.;MIN_ED=.;NLOD=3.02;TLOD=7.43	GT:AD:AF:ALT_F1R2:ALT_F2R1:FOXOG:QSS:REF_F1R2:REF_F2R1	0/1:61,11:0.127:8:3:0.727:1537,182:24:37	0/0:25,2:0.083:0:2:0.00:568,38:14:11	0.5	Unknown	.	.	.	.	.	.	.	.
T4df	chr5	1	12.5	30916945	30916945	T	G	exonic	**Emilin1**	ENSMUSP00000030143	.	nonsynonymous SNV	Emilin1:NM_133918:exon4:c.T529G:p.S177A	**S177A**	ENSMUSP00000030143,177,S,A	5,30916945,T,C	5qB1	.	0.25	.	31	chr5	30916945	.	T	C	.	alt_allele_in_normal;clustered_events	ECNT=5;HCNT=1;MAX_ED=24;MIN_ED=1;NLOD=5.43;TLOD=11.84	GT:AD:AF:ALT_F1R2:ALT_F2R1:FOXOG:PGT:PID:QSS:REF_F1R2:REF_F2R1	0/1:50,4:0.075:4:0:.:0|1:136373432_T_TTTTTAATGATACG:1253,57:21:29	0/0:37,1:0.030:1:0:.:0|1:136373432_T_TTTTTAATGATACG:938,22:13:24	1.4	Damaging	-1.31	Neutral	121	30	0.016	Damaging	2.82	43
T22df	chr2	1	12.5	156008228	156008228	G	T	exonic	**Ergic3**	ENSMUSP00000049414	.	nonsynonymous SNV	Ergic3:NM_001356413:exon1:c.G88T:p.V30L,Ergic3:NM_001356414:exon1:c.G88T:p.V30L,Ergic3:NM_025516:exon1:c.G88T:p.V30L	**V30L**	ENSMUSP00000049414,30,V,L	2,156008228,G,A	2qH1	.	0.25	.	35	chr2	156008228	.	G	A	.	PASS	DB;ECNT=1;HCNT=1;MAX_ED=.;MIN_ED=.;NLOD=8.08;TLOD=66.78	GT:AD:AF:ALT_F1R2:ALT_F2R1:FOXOG:QSS:REF_F1R2:REF_F2R1	0/1:28,27:0.481:12:15:0.444:772,759:13:15	0/0:29,0:0.00:0:0:.:781,0:13:16	2.8	Damaging	-2.32	Neutral	152	30	0.001	Damaging	2.83	147

T2df	chr8	**1**	**12.5**	9770733	9770735	GCT	-	exonic	**Fam155a**	ENSMUSP00000134039	.	nonframeshift deletion	Fam155a:NM_001347127:exon1:c.284_286del:p.95_96del,Fam155a:NM_173446:exon1:c.284_286del:p.95_96del	**95_96del**	NA	8,9770733,CGCT,C	8qA1.1	.	0.25	.	27	chr8	9770732	.	CGCT	C	.	clustered_events;t_lod_fstar	ECNT=2;HCNT=2;MAX_ED=3;MIN_ED=3;NLOD=25.54;TLOD=6.00	GT:AD:AF:ALT_F1R2:ALT_F2R1:FOXOG:PGT:PID:QSS:REF_F1R2:REF_F2R1	0/1:24,2:0.080:2:0:.:0|1:36947077_CA_C:678,56:13:11	0/0:89,0:0.00:0:0:.:0|1:36947077_CA_C:2508,0:42:47	1	Unknown	.	.	.	.	.	.	.	.
T4df	chr14	**1**	**12.5**	27471797	27471797	G	C	exonic	**Fam208a**	ENSMUSP00000041210	.	nonsynonymous SNV	Fam208a:NM_001114879:exon18:c.G2953C:p.V985L,Fam208a:NM_028945:exon18:c.G2953C:p.V985L	**V985L**	ENSMUSP00000041210,985,V,L	14,27471797,G,G	14qA3	.	0.25	.	61	chr14	27471797	.	G	G	.	t_lod_fstar	ECNT=1;HCNT=1;MAX_ED=.;MIN_ED=.;NLOD=7.83;TLOD=5.65	GT:AD:AF:ALT_F1R2:ALT_F2R1:FOXOG:QSS:REF_F1R2:REF_F2R1	0/1:36,2:0.056:0:2:.:1027,56:18:18	0/0:30,0:0.00:0:0:.:859,0:17:13	4	Tolerated	0	Neutral	106	30	1	Tolerated	3.35	54
T4df	chr14	**1**	**12.5**	27471791	27471791	A	-	exonic	**Fam208a**	ENSMUSP00000109463	.	frameshift deletion	Fam208a:NM_001114879:exon18:c.2947delA:p.M983fs,Fam208a:NM_028945:exon18:c.2947delA:p.M983fs	**M983fs**	ENSMUSP00000109463,983,M,.	14,27471791,GA,C	14qA3	.	0.25	.	62	chr14	27471790	.	GA	C	.	str_contraction;t_lod_fstar	ECNT=1;HCNT=1;MAX_ED=.;MIN_ED=.;NLOD=6.02;RPA=7,6;RU=CTG;STR;TLOD=4.73	GT:AD:AF:ALT_F1R2:ALT_F2R1:FOXOG:QSS:REF_F1R2:REF_F2R1	0/1:54,2:0.038:0:2:.:1525,56:29:25	0/0:24,0:0.00:0:0:.:672,0:13:11	2.8	Unknown	NA	NA	106	30	NA	NA	NA	NA
T3df	chr4	**1**	**12.5**	138395208	138395210	TCA	-	exonic	**Fam43b**	ENSMUSP00000121724	.	nonframeshift deletion	Fam43b:NM_001081672:exon1:c.798_800del:p.266_267del	**266_267del**	NA	4,138395208,CTCA,CACGGTTTCTTCCGGCCTTGTCCATCCGGCCATGGCTGTGTCGACGAGGCCAGCGGAACCACCAAAGCTGTGGCTACCTCCTGCTAGAT	4qD3	.	0.25	.	27	chr4	138395207	.	CTCA	CACGGTTTCTTCCGGCCTTGTCCATCCGGCCATGGCTGTGTCGACGAGGCCAGCGGAACCACCAAAGCTGTGGCTACCTCCTGCTAGAT	.	PASS	ECNT=1;HCNT=1;MAX_ED=.;MIN_ED=.;NLOD=10.01;TLOD=7.48	GT:AD:AF:ALT_F1R2:ALT_F2R1:FOXOG:QSS:REF_F1R2:REF_F2R1	0/1:36,1:0.077:1:0:.:1038,28:13:23	0/0:36,0:0.00:0:0:.:1034,0:15:21	0.5	Unknown	NA	NA	102	30	NA	NA	NA	NA
T1df	chr11	**1**	**12.5**	46406919	46406919	C	A	exonic	**Fam71b**	ENSMUSP00000122256	.	nonsynonymous SNV	Fam71b:NM_001013783:exon2:c.C1049A:p.P350H	**P350H**	ENSMUSP00000122256,350,P,H	11,46406919,C,C	11qB1.1	.	0.25	.	32	chr11	46406919	.	C	C	.	clustered_events;homologous_mapping_event;t_lod_fstar	ECNT=4;HCNT=1;MAX_ED=15;MIN_ED=4;NLOD=9.93;TLOD=5.18	GT:AD:AF:ALT_F1R2:ALT_F2R1:FOXOG:PGT:PID:QSS:REF_F1R2:REF_F2R1	0/1:62,2:0.033:0:2:0.00:0|1:46406919_C_A:1740,58:31:31	0/0:32,0:0.00:0:0:.:0|1:46406919_C_A:884,0:14:18	0.9	Tolerated	0	Neutral	81	30	0.304	Tolerated	3.13	13
T1df	chr11	**1**	**12.5**	46406923	46406923	G	A	exonic	**Fam71b**	ENSMUSP00000122256	.	nonsynonymous SNV	Fam71b:NM_001013783:exon2:c.G1053A:p.M351I	**M351I**	ENSMUSP00000122256,351,M,I	11,46406923,G,A	11qB1.1	.	0.25	.	33	chr11	46406923	.	G	A	.	str_contraction;t_lod_fstar	ECNT=1;HCNT=1;MAX_ED=.;MIN_ED=.;NLOD=4.16;RPA=10,9;RU=GAG;STR;TLOD=4.94	GT:AD:AF:ALT_F1R2:ALT_F2R1:FOXOG:QSS:REF_F1R2:REF_F2R1	0/1:33,3:0.086:2:1:.:937,86:17:16	0/0:16,0:0.00:0:0:.:443,0:8:8	1.4	Tolerated	0.08	Neutral	81	30	0.175	Tolerated	3.11	20
T1df	chr11	**1**	**12.5**	46406934	46406934	T	C	exonic	**Fam71b**	ENSMUSP00000122256	.	nonsynonymous SNV	Fam71b:NM_001013783:exon2:c.T1064C:p.V355A	**V355A**	ENSMUSP00000122256,355,V,A	11,46406934,T,T	11qB1.1	.	0.25	.	32	chr11	46406934	.	T	T	.	clustered_events;homologous_mapping_event;t_lod_fstar	ECNT=8;HCNT=1;MAX_ED=23;MIN_ED=2;NLOD=9.33;TLOD=5.04	GT:AD:AF:ALT_F1R2:ALT_F2R1:FOXOG:PGT:PID:QSS:REF_F1R2:REF_F2R1	0/1:71,2:0.028:0:2:0.00:0|1:45751093_C_CT:1891,54:45:26	0/0:33,0:0.00:0:0:.:0|1:45751093_C_CT:916,0:22:11	0.9	Tolerated	0	Neutral	81	30	1	Tolerated	3.15	19
T2df	chr8	**1**	**12.5**	123313320	123313321	AT	-	exonic	**Fanca**	ENSMUSP00000042457	.	frameshift deletion	Fanca:NM_016925:exon4:c.336_337del:p.L112fs	**L112fs**	ENSMUSP00000042457,112,L,.	8,123313320,GAT,A	8qE1	.	0.25	.	30	chr8	123313319	.	GAT	A	.	PASS	DB;ECNT=1;HCNT=1;MAX_ED=.;MIN_ED=.;NLOD=10.13;TLOD=95.88	GT:AD:AF:ALT_F1R2:ALT_F2R1:FOXOG:QSS:REF_F1R2:REF_F2R1	0/1:32,36:0.530:20:16:0.556:881,1021:14:18	0/0:36,0:0.00:0:0:.:906,0:16:20	2.7	Unknown	NA	NA	65	28	NA	NA	NA	NA
T42df	chr9	**1**	**12.5**	55218356	55218356	G	T	exonic	**Fbxo22**	ENSMUSP00000006035	.	stopgain	Fbxo22:NM_028049:exon5:c.G502T:p.E168X	**E168X**	ENSMUSP00000006035,168,E,X	NA	9qB	.	0.25	.	55	chr9	55218356	.	G	T	.	alt_allele_in_normal;t_lod_fstar	ECNT=1;HCNT=8;MAX_ED=.;MIN_ED=.;NLOD=4.18;TLOD=4.81	GT:AD:AF:ALT_F1R2:ALT_F2R1:FOXOG:QSS:REF_F1R2:REF_F2R1	0/1:38,3:0.083:3:0:0.00:1049,81:28:10	0/0:39,2:0.056:1:1:0.500:1099,57:19:20	1.3	Deletarious	.	.	.	.	.	.	.	.

T2df	chr6	**1**	**12.5**	91987223	91987327	CCAGATGAAGAGGCACCAAGTAGAGACAGTGAGGAAGGCATGGTCCACGCTCTGGAAGATGAAGACTGTGATCACGATCCAGAGACGGATGGGACCCAACATCG	-	exonic	**Fgd5**	ENSMUSP00000086025	.	nonframeshift deletion	Fgd5:NM_172731:exon2:c.436_540del:p.146_180del	**146_180del**	NA	6,91987223,TCCAGATGAAGAGGCACCAAGTAGAGACAGTGAGGAAGGCATGGTCCACGCTCTGGAAGATGAAGACTGTGATCACGATCCAGAGACGGATGGGACCCCAACATCG,T	6qD1	.	0.25	.	59	chr6	91987222	.	TCCAGATGAAGAGGCACCAAGTAGAGACAGTGAGGAAGGCATGGTCCACGCTCTGGAAGATGAAGACTGTGATCACGATCCAGAGACGGATGGGACCCCAACATCG	T	.	alt_allele_in_normal;clustered_events	ECNT=10;HCNT=1;MAX_ED=31;MIN_ED=9;NLOD=2.72;TLOD=18.11	GT:AD:AF:ALT_F1R2:ALT_F2R1:FOXOG:PGT:PID:QSS:REF_F1R2:REF_F2R1	0/1:63,6:0.086:0:6:1.00:0|1:80534525_G_T:1643,171:41:22	0/0:26,1:0.042:0:1:1.00:0|1:80534525_G_T:702,28:17:9	0.6	Unknown	.	.	.	.	.	.	.	.
T4df	chr10	**1**	**12.5**	79755286	79755286	-	ATCATTAAAAA	exonic	**Fgf22**	ENSMUSP00000119051	.	nonframeshift insertion	Fgf22:NM_023304:exon1:c.168_169insATCATTAAAAAA:p.R56delinsRIIKK	**R56delinsRIIKK**	ENSMUSP00000119051,56,R,RIIKK	10,79755286,T,T	10qC1	.	0.25	.	26	chr10	79755286	.	T	T	.	clustered_events;multi_event_alt_allele_in_normal	ECNT=7;HCNT=1;MAX_ED=24;MIN_ED=2;NLOD=6.34;TLOD=8.71	GT:AD:AF:ALT_F1R2:ALT_F2R1:FOXOG:PGT:PID:QSS:REF_F1R2:REF_F2R1	0/1:42,3:0.067:0:3:0.00:0|1:179752666_C_CGTGGT:1156,49:25:17	0/0:36,1:0.028:0:1:0.00:0|1:179752666_C_CGTGGT:966,24:22:14	21	Tolerated	0	Neutral	252	30	1	Tolerated	2.86	224
T4df	chr10	**1**	**12.5**	79755270	79755273	GGGT	-	exonic	**Fgf22**	ENSMUSP00000033927	.	frameshift deletion	Fgf22:NM_023304:exon1:c.152_155del:p.R51fs	**R51fs**	ENSMUSP00000033927,51,R,.	10,79755270,CGGGT,TATCATTAAAAA	10qC1	.	0.25	.	32	chr10	79755269	.	CGGGT	TATCATTAAAAA	.	clustered_events;multi_event_alt_allele_in_normal;t_lod_fstar	ECNT=6;HCNT=1;MAX_ED=30;MIN_ED=3;NLOD=3.93;TLOD=5.22	GT:AD:AF:ALT_F1R2:ALT_F2R1:FOXOG:PGT:PID:QSS:REF_F1R2:REF_F2R1	0/1:59,2:0.034:0:2:.:0|1:79755256_G_A:1644,37:29:30	0/0:25,1:0.036:0:1:.:0|1:79755256_G_A:698,12:14:11	0.7	Unknown	NA	NA	252	30	NA	NA	NA	NA
T4df	chr10	**1**	**12.5**	79755276	79755283	AGGGGACG	-	exonic	**Fgf22**	ENSMUSP00000033927	.	frameshift deletion	Fgf22:NM_023304:exon1:c.158_165del:p.Q53fs	**Q53fs**	ENSMUSP00000033927,53,Q,.	10,79755276,CAGGGGACG,T	10qC1	.	0.25	.	31	chr10	79755275	.	CAGGGGACG	T	.	alt_allele_in_normal;t_lod_fstar	ECNT=1;HCNT=8;MAX_ED=.;MIN_ED=.;NLOD=4.18;TLOD=4.81	GT:AD:AF:ALT_F1R2:ALT_F2R1:FOXOG:QSS:REF_F1R2:REF_F2R1	0/1:38,3:0.083:3:0:0.00:1049,81:28:10	0/0:39,2:0.056:1:1:0.500:1099,57:19:20	1.3	Unknown	NA	NA	252	30	NA	NA	NA	NA
T2df	chr18	**1**	**12.5**	25020666	25020668	GAG	-	exonic	**Fhod3**	ENSMUSP00000153693	.	nonframeshift deletion	Fhod3:NM_175276:exon11:c.1204_1206del:p.402_402del	**402_402del**	NA	18,25020666,TGAG,T	18qA2	.	0.25	.	61	chr18	25020665	.	TGAG	T	.	clustered_events;homologous_mapping_event	ECNT=3;HCNT=2;MAX_ED=19;MIN_ED=16;NLOD=8.73;TLOD=9.18	GT:AD:AF:ALT_F1R2:ALT_F2R1:FOXOG:PGT:PID:QSS:REF_F1R2:REF_F2R1	0/1:31,3:0.094:0:3:1.00:0|1:68975120_C_T:817,87:15:16	0/0:36,0:0.00:0:0:.:0|1:68975120_C_T:856,0:20:16	0.3	Unknown	.	.	.	.	.	.	.	.
T32df	chr5	**1**	**12.5**	136965159	136965159	A	G	exonic	**Fis1**	ENSMUSP00000079818	.	nonsynonymous SNV	Fis1:NM_001347504:exon2:c.A95G:p.Y32C,Fis1:NM_001163243:exon3:c.A224G:p.Y75C,Fis1:NM_025562:exon3:c.A245G:p.Y82C	**Y75C**	ENSMUSP00000079818,75,Y,C	5,136965159,A,C	5qG2	.	0.25	.	43	chr5	136965159	.	A	C	.	t_lod_fstar	ECNT=1;HCNT=1;MAX_ED=.;MIN_ED=.;NLOD=8.73;TLOD=5.20	GT:AD:AF:ALT_F1R2:ALT_F2R1:FOXOG:QSS:REF_F1R2:REF_F2R1	0/1:37,2:0.053:0:2:.:1037,55:16:21	0/0:34,0:0.00:0:0:.:932,0:21:13	0.8	Deleterious	-2.55	Deleterious	108	30	0.76	Tolerated	2.81	98
T32df	chr5	**1**	**12.5**	136965159	136965159	A	G	exonic	**Fis1**	ENSMUSP00000079818	.	nonsynonymous SNV	Fis1:NM_001347504:exon2:c.A95G:p.Y32C,Fis1:NM_001163243:exon3:c.A224G:p.Y75C,Fis1:NM_025562:exon3:c.A245G:p.Y82C	**Y82C**	ENSMUSP00000079818,82,Y,C	5,136965159,A,C	5qG2	.	0.25	.	43	chr5	136965159	.	A	C	.	t_lod_fstar	ECNT=1;HCNT=1;MAX_ED=.;MIN_ED=.;NLOD=8.73;TLOD=5.20	GT:AD:AF:ALT_F1R2:ALT_F2R1:FOXOG:QSS:REF_F1R2:REF_F2R1	0/1:37,2:0.053:0:2:.:1037,55:16:21	0/0:34,0:0.00:0:0:.:932,0:21:13	0.8	Deleterious	-2.55	Deleterious	108	30	0.76	Tolerated	2.81	98
T32df	chr5	**1**	**12.5**	136965159	136965159	A	G	exonic	**Fis1**	ENSMUSP00000079818	.	nonsynonymous SNV	Fis1:NM_001347504:exon2:c.A95G:p.Y32C,Fis1:NM_001163243:exon3:c.A224G:p.Y75C,Fis1:NM_025562:exon3:c.A245G:p.Y82C	**Y32C**	ENSMUSP00000079818,32,Y,C	5,136965159,A,G	5qG2	.	0.25	.	43	chr5	136965159	.	A	G	.	PASS	ECNT=1;HCNT=2;MAX_ED=.;MIN_ED=.;NLOD=11.38;TLOD=6.80	GT:AD:AF:ALT_F1R2:ALT_F2R1:FOXOG:QSS:REF_F1R2:REF_F2R1	0/1:56,5:0.069:5:0:0.00:1572,142:23:33	0/0:43,0:0.00:0:0:.:1191,0:23:20	1.1	Deleterious	-3.08	Deleterious	108	30	0.087	Tolerated	2.81	98
T12df	chr14	**1**	**12.5**	7917275	7917275	A	C	exonic	**Flnb**	ENSMUSP00000059798	.	nonsynonymous SNV	Flnb:NM_001081427:exon28:c.A4753C:p.T1585P,Flnb:NM_134080:exon28:c.A4753C:p.T1585P	**T1585P**	ENSMUSP00000059798,1585,T,P	14,7917275,A,G	14qA1	.	0.25	.	64	chr14	7917275	.	A	G	.	clustered_events;homologous_mapping_event	DB;ECNT=8;HCNT=42;MAX_ED=199;MIN_ED=18;NLOD=8.38;TLOD=38.39	GT:AD:AF:ALT_F1R2:ALT_F2R1:FOXOG:QSS:REF_F1R2:REF_F2R1	0/1:21,14:0.424:5:9:0.643:582,392:11:10	0/0:32,0:0.00:0:0:.:900,0:15:17	2	Tolerated	-2.04	Neutral	262	30	0.144	Tolerated	2.93	271
T22df	chr2	**1**	**12.5**	113525668	113525669	CG	-	exonic	**Fmn1**	ENSMUSP00000106596	.	frameshift deletion	Fmn1:NM_001285458:exon4:c.2453_2454del:p.P818fs,Fmn1:NM_001285459:exon4:c.2069_2070del:p.P690fs,Fmn1:NM_010230:exon6:c.2747_2748del:p.P916fs	**P916fs**	ENSMUSP00000106596,916,P,.	2,113525668,CCG,A	2qE4	.	0.25	.	34	chr2	113525667	.	CCG	A	.	PASS	DB;ECNT=1;HCNT=1;MAX_ED=.;MIN_ED=.;NLOD=7.04;TLOD=79.01	GT:AD:AF:ALT_F1R2:ALT_F2R1:FOXOG:QSS:REF_F1R2:REF_F2R1	0/1:37,32:0.462:15:17:0.469:1035,899:25:12	0/0:27,0:0.00:0:0:.:687,0:16:11	3	Unknown	NA	NA	69	30	NA	NA	NA	NA

T22df	chr2	**1**	**12.5**	113525668	113525669	CG	-	exonic	**Fmn1**	ENSMUSP00000106596	.	frameshift deletion	Fmn1:NM_001285458:exon4:c.2453_2454del:p.P818fs,Fmn1:NM_001285459:exon4:c.2069_2070del:p.P690fs,Fmn1:NM_010230:exon6:c.2747_2748del:p.P916fs	**P690fs**	ENSMUSP00000106596,690,P,.	2,113525668,CCG,C	2qE4	.	0.25	.	34	chr2	113525667	.	CCG	C	.	t_lod_fstar	ECNT=1;HCNT=1;MAX_ED=.;MIN_ED=.;NLOD=13.10;TLOD=4.46	GT:AD:AF:ALT_F1R2:ALT_F2R1:FOXOG:QSS:REF_F1R2:REF_F2R1	0/1:71,3:0.042:3:0:1.00:1899,84:27:44	0/0:48,0:0.00:0:0:.:1296,0:28:20	0.5	Unknown	NA	NA	69	30	NA	NA	NA	NA
T22df	chr2	**1**	**12.5**	113525668	113525669	CG	-	exonic	**Fmn1**	ENSMUSP00000106596	.	frameshift deletion	Fmn1:NM_001285458:exon4:c.2453_2454del:p.P818fs,Fmn1:NM_001285459:exon4:c.2069_2070del:p.P690fs,Fmn1:NM_010230:exon6:c.2747_2748del:p.P916fs	**P818fs**	ENSMUSP00000106596,818,P,.	2,113525668,CCG,C	2qE4	.	0.25	.	34	chr2	113525667	.	CCG	C	.	t_lod_fstar	ECNT=1;HCNT=1;MAX_ED=.;MIN_ED=.;NLOD=8.73;TLOD=5.20	GT:AD:AF:ALT_F1R2:ALT_F2R1:FOXOG:QSS:REF_F1R2:REF_F2R1	0/1:37,2:0.053:0:2:.:1037,55:16:21	0/0:34,0:0.00:0:0:.:932,0:21:13	0.8	Unknown	NA	NA	69	30	NA	NA	NA	NA
T1df	chr12	**1**	**12.5**	76875102	76875102	T	C	exonic	**Fntb**	ENSMUSP00000022450	.	nonsynonymous SNV	Fntb:NM_145927:exon4:c.T353C:p.I118T	**I118T**	ENSMUSP00000022450,118,I,T	12,76875102,T,C	12qC3	.	0.25	.	48	chr12	76875102	.	T	C	.	PASS	ECNT=1;HCNT=1;MAX_ED=.;MIN_ED=.;NLOD=11.34;TLOD=6.63	GT:AD:AF:ALT_F1R2:ALT_F2R1:FOXOG:QSS:REF_F1R2:REF_F2R1	0/1:17,3:0.158:1:2:0.333:491,88:6:11	0/0:46,0:0.00:0:0:.:1262,0:23:23	0.6	Deleterious	-2.75	Deleterious	104	30	0.022	Damaging	2.85	138
T22df	chr15	**1**	**12.5**	76669610	76669610	C	G	exonic	**Foxh1**	ENSMUSP00000112745	.	nonsynonymous SNV	Foxh1:NM_007989:exon1:c.G262C:p.A88P	**A88P**	ENSMUSP00000112745,88,A,P	15,76669610,C,A	15qD3	.	0.25	.	46	chr15	76669610	.	C	A	.	PASS	DB;ECNT=1;HCNT=1;MAX_ED=.;MIN_ED=.;NLOD=10.13;TLOD=95.88	GT:AD:AF:ALT_F1R2:ALT_F2R1:FOXOG:QSS:REF_F1R2:REF_F2R1	0/1:32,36:0.530:20:16:0.556:881,1021:14:18	0/0:36,0:0.00:0:0:.:906,0:16:20	2.7	Tolerated	1.32	Neutral	137	30	0.636	Tolerated	3.01	398
T32df	chr5	**1**	**12.5**	70973480	70973480	T	C	exonic	**Gabra2**	ENSMUSP00000113125	.	nonsynonymous SNV	Gabra2:NM_008066:exon9:c.A1004G:p.N335S	**N335S**	ENSMUSP00000113125,335,N,S	5,70973480,T,A	5qC3.1	.	0.25	.	46	chr5	70973480	.	T	A	.	clustered_events;multi_event_alt_allele_in_normal;t_lod_fstar	ECNT=2;HCNT=46;MAX_ED=3;MIN_ED=3;NLOD=10.41;TLOD=4.68	GT:AD:AF:ALT_F1R2:ALT_F2R1:FOXOG:PGT:PID:QSS:REF_F1R2:REF_F2R1	0/1:43,3:0.068:2:1:0.667:0|1:142572539_T_A:1213,84:27:16	0/0:46,1:0.022:0:1:0.00:0|1:142572539_T_A:1277,28:25:21	0.9	Deleterious	-7.18	Deleterious	432	30	0	Damaging	3.23	379
T2df	chrX	**1**	**12.5**	72270377	72270448	GGCTCAGGCTGGGGCTCAGGCTCCGGCTGGGGCTCGGGCTCAGGCTCAGGCTGTGGCTGTGGCTCAGGCTGT	-	exonic	**Gabre**	ENSMUSP00000113336	.	nonframeshift deletion	Gabre:NM_017369:exon2:c.744_815del:p.248_272del	**248_272del**	NA	X,72270377,AGGCTCAGGCTGGGGCTCAGGCTCCGGCTGGGGCTCGGGCTCAGGCTCAGGCTGTGGCTGTGGCTCAGGCTGT,G	XqA7.3	.	0.25	.	44	chrX	72270376	.	AGGCTCAGGCTGGGGCTCAGGCTCCGGCTGGGGCTCGGGCTCAGGCTCAGGCTGTGGCTGTGGCTCAGGCTGT	G	.	str_contraction;t_lod_fstar	ECNT=1;HCNT=1;MAX_ED=.;MIN_ED=.;NLOD=2.67;RPA=7,6;RU=CCA;STR;TLOD=4.97	GT:AD:AF:ALT_F1R2:ALT_F2R1:FOXOG:QSS:REF_F1R2:REF_F2R1	0/1:34,2:0.061:1:1:.:901,51:20:14	0/0:9,0:0.00:0:0:.:200,0:5:4	1	Unknown	.	.	.	.	.	.	.	.
T2df	chr3	**1**	**12.5**	142572539	142572539	T	A	exonic	**Gbp3**	ENSMUSP00000034859	.	nonsynonymous SNV	Gbp3:NM_001289493:exon11:c.T1710A:p.S570R,Gbp3:NM_018734:exon11:c.T1710A:p.S570R,Gbp3:NM_001289492:exon12:c.T1710A:p.S570R,Gbp3:NM_001355403:exon12:c.T1710A:p.S570R	**S570R**	ENSMUSP00000034859,570,S,R	3,142572539,T,T	3qH1	.	0.25	.	47	chr3	142572539	.	T	T	.	str_contraction;t_lod_fstar	ECNT=1;HCNT=2;MAX_ED=.;MIN_ED=.;NLOD=5.98;RPA=9,8;RU=GGA;STR;TLOD=4.23	GT:AD:AF:ALT_F1R2:ALT_F2R1:FOXOG:QSS:REF_F1R2:REF_F2R1	0/1:38,2:0.053:1:1:.:1030,58:29:9	0/0:23,0:0.00:0:0:.:576,0:12:11	1.4	Tolerated	0	Neutral	142	30	0.62	Tolerated	2.84	206
T2df	chr3	**1**	**12.5**	142572542	142572542	C	A	exonic	**Gbp3**	ENSMUSP00000034859	.	nonsynonymous SNV	Gbp3:NM_001289493:exon11:c.C1713A:p.H571Q,Gbp3:NM_018734:exon11:c.C1713A:p.H571Q,Gbp3:NM_001289492:exon12:c.C1713A:p.H571Q,Gbp3:NM_001355403:exon12:c.C1713A:p.H571Q	**H571Q**	ENSMUSP00000034859,571,H,Q	3,142572542,C,G	3qH1	.	0.25	.	48	chr3	142572542	.	C	G	.	alt_allele_in_normal;clustered_events	ECNT=8;HCNT=1;MAX_ED=24;MIN_ED=6;NLOD=4.83;TLOD=21.11	GT:AD:AF:ALT_F1R2:ALT_F2R1:FOXOG:PGT:PID:QSS:REF_F1R2:REF_F2R1	0/1:82,7:0.085:0:7:0.00:0|1:107615748_CCTT_C:2274,47:48:34	0/0:36,1:0.032:0:1:0.00:0|1:107615748_CCTT_C:1013,23:20:16	0.4	Tolerated	1.25	Neutral	142	30	1	Tolerated	2.84	205
T1df	chr8	**1**	**12.5**	116982727	116982727	-	ATCT	splicing	**Gcsh**	ENSMUSP00000020112	NM_026572:exon5:c.416-1->AGAT	.	.	**NA**	8,116982727,C,C	NA	8qE1	.	0.25	.	49	chr8	116982727	.	C	C	.	str_contraction;t_lod_fstar	ECNT=1;HCNT=1;MAX_ED=.;MIN_ED=.;NLOD=9.61;RPA=6,5;RU=CAG;STR;TLOD=5.41	GT:AD:AF:ALT_F1R2:ALT_F2R1:FOXOG:QSS:REF_F1R2:REF_F2R1	0/1:47,2:0.043:2:0:.:1303,56:23:24	0/0:36,0:0.00:0:0:.:985,0:20:16	6	Unkown	.	.	.	.	.	.	.	.
T4df	chr5	**1**	**12.5**	137520938	137520940	GGA	-	exonic	**Gigyf1**	ENSMUSP00000020577	.	nonframeshift deletion	Gigyf1:NM_031408:exon9:c.996_998del:p.332_333del	**332_333del**	NA	5,137520938,TGGA,C	5qG2	rs259442751	0.25	.	23	chr5	137520937	.	TGGA	C	.	alt_allele_in_normal;clustered_events	ECNT=8;HCNT=1;MAX_ED=24;MIN_ED=6;NLOD=5.13;TLOD=21.15	GT:AD:AF:ALT_F1R2:ALT_F2R1:FOXOG:PGT:PID:QSS:REF_F1R2:REF_F2R1	0/1:80,7:0.086:0:7:.:0|1:107615748_CCTT_C:1896,201:48:32	0/0:35,1:0.031:0:1:.:0|1:107615748_CCTT_C:826,29:19:16	0.4	Unknown	.	.	.	.	.	.	.	.

T4df	chr11	**1**	**12.5**	70640677	70640677	T	C	exonic	**Gp1ba**	ENSMUSP00000052020	.	nonsynonymous SNV	Gp1ba:NM_010326:exon2:c.T1268C:p.I423T	**I423T**	ENSMUSP00000052020,423,I,T	11,70640677,T,G	11qB3	.	0.25	.	35	chr11	70640677	.	T	G	.	PASS	ECNT=1;HCNT=1;MAX_ED=.;MIN_ED=.;NLOD=8.68;TLOD=13.11	GT:AD:AF:ALT_F1R2:ALT_F2R1:FOXOG:QSS:REF_F1R2:REF_F2R1	0/1:39,6:0.133:3:3:0.500:1074,179:18:21	0/0:32,0:0.00:0:0:.:886,0:14:18	0.2	Damaging	-0.26	Neutral	123	30	0.029	Damaging	3.46	5
T4df	chr11	**1**	**12.5**	70640673	70640673	A	G	exonic	**Gp1ba**	ENSMUSP00000052020	.	nonsynonymous SNV	Gp1ba:NM_010326:exon2:c.A1264G:p.T422A	**T422A**	ENSMUSP00000052020,422,T,A	11,70640673,A,C	11qB3	.	0.25	.	36	chr11	70640673	.	A	C	.	clustered_events;homologous_mapping_event;t_lod_fstar	DB;ECNT=6;HCNT=1;MAX_ED=22;MIN_ED=4;NLOD=11.14;TLOD=5.53	GT:AD:AF:ALT_F1R2:ALT_F2R1:FOXOG:PGT:PID:QSS:REF_F1R2:REF_F2R1	0/1:40,1:0.049:0:1:1.00:0|1:70640673_A_G:1132,30:19:21	0/0:39,0:0.00:0:0:.:0|1:70640673_A_G:1050,0:14:25	0.3	Tolerated	0.6	Neutral	123	30	0.365	Tolerated	3.38	6
T22df	chr11	**1**	**12.5**	70640785	70640785	T	C	exonic	**Gp1ba**	ENSMUSP00000052020	.	nonsynonymous SNV	Gp1ba:NM_010326:exon2:c.T1376C:p.L459P	**L459P**	ENSMUSP00000052020,459,L,P	11,70640785,T,C	11qB3	.	0.25	.	31	chr11	70640785	.	T	C	.	clustered_events;homologous_mapping_event;t_lod_fstar	ECNT=6;HCNT=1;MAX_ED=22;MIN_ED=4;NLOD=11.14;TLOD=5.43	GT:AD:AF:ALT_F1R2:ALT_F2R1:FOXOG:PGT:PID:QSS:REF_F1R2:REF_F2R1	0/1:42,2:0.043:1:1:0.500:0|1:70640673_A_G:1085,52:17:25	0/0:35,0:0.00:0:0:.:0|1:70640673_A_G:856,0:15:20	0.3	Tolerated	1.96	Neutral	123	30	0.131	Tolerated	3.43	9
T4df	chr11	**1**	**12.5**	70640695	70640695	A	C	exonic	**Gp1ba**	ENSMUSP00000052020	.	nonsynonymous SNV	Gp1ba:NM_010326:exon2:c.A1286C:p.H429P	**H429P**	ENSMUSP00000052020,429,H,P	11,70640695,A,AG	11qB3	.	0.25	.	39	chr11	70640695	.	A	AG	.	PASS	ECNT=1;HCNT=2;MAX_ED=.;MIN_ED=.;NLOD=11.74;TLOD=7.59	GT:AD:AF:ALT_F1R2:ALT_F2R1:FOXOG:QSS:REF_F1R2:REF_F2R1	0/1:23,3:0.125:0:3:.:656,84:9:14	0/0:47,0:0.00:0:0:.:1318,0:24:23	1.4	Unknown	NA	NA	123	30	NA	NA	NA	NA
T4df	chr11	**1**	**12.5**	97334400	97334476	GGTGCTGTCATTTACAGTCCCAAGGGTAGTCAGGGGGAGGGTGCTGTCATTCACAGTCCCAAGGGTAGTCAGGGGGA	-	exonic	**Gpr179**	ENSP00000480024	.	frameshift deletion	Gpr179:NM_001081220:exon11:c.6852_6928del:p.P2284fs	**P2284fs**	NA	11,97334399,GGGTGCTGTCATTTACAGTCCCAAGGGTAGTCAGGGGGAGGGTGCTGTCATTCACAGTCCCAAGGGTAGTCAGGGGGA,G	11qD	.	0.25	.	32	chr11	97334399	.	GGGTGCTGTCATTTACAGTCCCAAGGGTAGTCAGGGGGAGGGTGCTGTCATTCACAGTCCCAAGGGTAGTCAGGGGGA	G	.	t_lod_fstar	ECNT=1;HCNT=1;MAX_ED=.;MIN_ED=.;NLOD=8.20;TLOD=5.64	GT:AD:AF:ALT_F1R2:ALT_F2R1:FOXOG:QSS:REF_F1R2:REF_F2R1	0/1:35,3:0.054:2:1:.:947,86:22:13	0/0:31,1:0.032:1:0:.:780,6:16:15	6	Unknown	NA	NA	NA	NA	NA	NA	NA	NA
T32df	chr9	**1**	**12.5**	66476379	66476379	-	G	exonic	**Herc1**	ENSMUSP00000035498	.	frameshift insertion	Herc1:NM_145617:exon57:c.11124_11125insG:p.T3708fs	**T3708fs**	ENSMUSP00000035498,3708,T,	9,66476379,A,A	9qC	.	0.25	.	47	chr9	66476379	.	A	A	.	clustered_events;homologous_mapping_event;t_lod_fstar	ECNT=11;HCNT=1;MAX_ED=77;MIN_ED=21;NLOD=19.57;TLOD=5.49	GT:AD:AF:ALT_F1R2:ALT_F2R1:FOXOG:PGT:PID:QSS:REF_F1R2:REF_F2R1	0/1:41,2:0.047:1:1:0.500:0|1:149048726_C_A:1173,58:23:18	0/0:93,0:0.00:0:0:.:0|1:149048726_C_A:2656,0:38:55	1.9	Tolerated	0	Neutral	107	30	0.963	Tolerated	3.07	54
T1df	chr6	**1**	**12.5**	57660155	57660155	C	T	exonic	**Herc6**	ENSMUSP00000036591	.	nonsynonymous SNV	Herc6:NM_025992:exon20:c.C2504T:p.T835I	**T835I**	ENSMUSP00000036591,835,T,I	6,57660155,C,T	6qB3	.	0.25	.	48	chr6	57660155	.	C	T	.	clustered_events;homologous_mapping_event;t_lod_fstar	ECNT=11;HCNT=1;MAX_ED=77;MIN_ED=21;NLOD=20.47;TLOD=5.49	GT:AD:AF:ALT_F1R2:ALT_F2R1:FOXOG:PGT:PID:QSS:REF_F1R2:REF_F2R1	0/1:41,2:0.047:1:1:0.500:0|1:149048726_C_A:1177,57:23:18	0/0:97,0:0.00:0:0:.:0|1:149048726_C_A:2701,0:38:58	1.9	Deleterious	-3.69	Deleterious	197	30	0.028	Damaging	2.8	130
T1df	chr5	**1**	**12.5**	149048752	149048752	A	T	exonic	**Hmgb1**	ENSMUSP00000073041	.	nonsynonymous SNV	Hmgb1:NM_001313894:exon5:c.T633A:p.D211E,Hmgb1:NM_010439:exon5:c.T633A:p.D211E	**D211E**	ENSMUSP00000073041,211,D,E	5,149048752,A,C	5qG3	.	0.25	.	97	chr5	149048752	.	A	C	.	clustered_events;homologous_mapping_event;t_lod_fstar	ECNT=11;HCNT=1;MAX_ED=77;MIN_ED=21;NLOD=16.86;TLOD=5.49	GT:AD:AF:ALT_F1R2:ALT_F2R1:FOXOG:PGT:PID:QSS:REF_F1R2:REF_F2R1	0/1:44,2:0.047:1:1:0.500:0|1:149048726_C_A:1226,55:25:19	0/0:82,0:0.00:0:0:.:0|1:149048726_C_A:2253,0:30:52	1.9	Damaging	-0.3	Neutral	388	30	0	Damaging	4.32	23
T1df	chr5	**1**	**12.5**	149048747	149048747	T	A	exonic	**Hmgb1**	ENSMUSP00000000572	.	nonsynonymous SNV	Hmgb1:NM_001313894:exon5:c.A638T:p.D213V,Hmgb1:NM_010439:exon5:c.A638T:p.D213V	**D213V**	ENSMUSP00000000572,213,D,V	5,149048747,T,T	5qG3	.	0.25	.	93	chr5	149048747	.	T	T	.	clustered_events;multi_event_alt_allele_in_normal;t_lod_fstar	ECNT=11;HCNT=3;MAX_ED=77;MIN_ED=21;NLOD=17.38;TLOD=5.51	GT:AD:AF:ALT_F1R2:ALT_F2R1:FOXOG:QSS:REF_F1R2:REF_F2R1	0/1:40,2:0.048:1:1:0.500:1149,54:22:18	0/0:99,1:0.011:0:1:1.00:2801,29:39:60	1.9	Tolerated	0	Neutral	388	30	1	Tolerated	4.32	23
T1df	chr5	**1**	**12.5**	149048764	149048764	A	T	exonic	**Hmgb1**	ENSMUSP00000000572	.	nonsynonymous SNV	Hmgb1:NM_001313894:exon5:c.T621A:p.D207E,Hmgb1:NM_010439:exon5:c.T621A:p.D207E	**D207E**	ENSMUSP00000000572,207,D,E	5,149048764,A,T	5qG3	.	0.25	.	100	chr5	149048764	.	A	T	.	clustered_events;homologous_mapping_event;str_contraction;t_lod_fstar	ECNT=11;HCNT=1;MAX_ED=77;MIN_ED=21;NLOD=17.76;RPA=2,1;RU=TCC;STR;TLOD=5.49	GT:AD:AF:ALT_F1R2:ALT_F2R1:FOXOG:PGT:PID:QSS:REF_F1R2:REF_F2R1	0/1:44,2:0.047:1:1:.:0|1:149048726_C_A:1242,47:26:18	0/0:89,0:0.00:0:0:.:0|1:149048726_C_A:2483,0:33:56	1.9	Tolerated	-1.25	Neutral	388	30	0.301	Tolerated	4.32	25

T1df	chr5	**1**	**12.5**	149048797	149048797	A	C	exonic	**Hmgb1**	ENSMUSP00000000572	.	nonsynonymous SNV	Hmgb1:NM_001313894:exon5:c.T588G:p.D196E,Hmgb1:NM_010439:exon5:c.T588G:p.D196E	**D196E**	ENSMUSP00000000572,196,D,E	5,149048797,A,T	5qG3	.	0.25	.	82	chr5	149048797	.	A	T	.	alt_allele_in_normal;t_lod_fstar	ECNT=1;HCNT=2;MAX_ED=.;MIN_ED=.;NLOD=7.33;TLOD=4.92	GT:AD:AF:ALT_F1R2:ALT_F2R1:FOXOG:QSS:REF_F1R2:REF_F2R1	0/1:31,3:0.088:1:2:0.667:876,79:15:16	0/0:50,2:0.044:2:0:0.00:1452,53:21:29	1.5	Tolerated	-0.96	Neutral	388	30	0.052	Tolerated	3.93	26
T1df	chr5	**1**	**12.5**	149048783	149048785	TCC	-	exonic	**Hmgb1**	ENSMUSP00000000572	.	nonframeshift deletion	Hmgb1:NM_001313894:exon5:c.600_602del:p.200_201del,Hmgb1:NM_010439:exon5:c.600_602del:p.200_201del	**200_201del**	NA	5,149048783,TTCC,G	5qG3	.	0.25	.	89	chr5	149048782	.	TTCC	G	.	str_contraction;t_lod_fstar	ECNT=1;HCNT=1;MAX_ED=.;MIN_ED=.;NLOD=6.92;RPA=7,6;RU=GGA;STR;TLOD=5.00	GT:AD:AF:ALT_F1R2:ALT_F2R1:FOXOG:QSS:REF_F1R2:REF_F2R1	0/1:61,2:0.032:1:1:.:1675,55:21:40	0/0:26,0:0.00:0:0:.:693,0:14:12	0.7	Unknown	.	.	.	.	.	.	.	.
T12df	chr18	**1**	**12.5**	61172186	61172188	CTT	-	exonic	**Hmgxb3**	ENSMUSP00000151698	.	nonframeshift deletion	Hmgxb3:NM_134134:exon2:c.97_99del:p.33_33del,Hmgxb3:NM_178277:exon2:c.97_99del:p.33_33del	**33_33del**	NA	18,61172186,ACTT,G	18qE1	.	0.25	.	60	chr18	61172185	.	ACTT	G	.	alt_allele_in_normal;clustered_events;germline_risk	ECNT=7;HCNT=2;MAX_ED=25;MIN_ED=6;NLOD=2.14;TLOD=11.00	GT:AD:AF:ALT_F1R2:ALT_F2R1:FOXOG:PGT:PID:QSS:REF_F1R2:REF_F2R1	0/1:84,4:0.047:4:0:1.00:0|1:101527856_ACTTG_A:2342,119:45:39	0/0:37,1:0.054:0:1:0.00:0|1:101527856_ACTTG_A:970,29:21:16	0.9	Unknown	.	.	.	.	.	.	.	.
T3df	chr11	**1**	**12.5**	51604404	51604404	C	T	exonic	**Hnrnpab**	ENSMUSP00000029935	.	nonsynonymous SNV	Hnrnpab:NM_001048061:exon5:c.G680A:p.S227N,Hnrnpab:NM_010448:exon5:c.G680A:p.S227N	**S227N**	ENSMUSP00000029935,227,S,N	11,51604404,C,T	11qB1.3	.	0.25	.	52	chr11	51604404	.	C	T	.	alt_allele_in_normal;t_lod_fstar	ECNT=1;HCNT=1;MAX_ED=.;MIN_ED=.;NLOD=4.26;TLOD=5.02	GT:AD:AF:ALT_F1R2:ALT_F2R1:FOXOG:QSS:REF_F1R2:REF_F2R1	0/1:78,2:0.027:1:1:.:2104,55:43:35	0/0:30,1:0.033:0:1:.:792,29:10:20	1.1	Tolerated	-1.5	Neutral	242	30	0.135	Tolerated	2.78	301
T3df	chr17	**1**	**12.5**	80061969	80061971	GGA	-	exonic	**Hnrnpll**	ENSMUSP00000101829	.	nonframeshift deletion	Hnrnpll:NM_144802:exon1:c.22_24del:p.8_8del	**8_8del**	NA	17,80061969,GGGA,T	17qE3	.	0.25	.	26	chr17	80061968	.	GGGA	T	.	alt_allele_in_normal;t_lod_fstar	ECNT=1;HCNT=1;MAX_ED=.;MIN_ED=.;NLOD=4.26;TLOD=5.02	GT:AD:AF:ALT_F1R2:ALT_F2R1:FOXOG:QSS:REF_F1R2:REF_F2R1	0/1:78,2:0.027:1:1:.:2104,55:43:35	0/0:30,1:0.033:0:1:.:792,29:10:20	1.1	Unknown	.	.	.	.	.	.	.	.
T12df	chr17	**1**	**12.5**	33649824	33649862	CGCTCCAGGCCCATGCGCTCAAGACTGTTGGCTCCCATA	-	exonic	**Hnrnpm**	ENSMUSP00000031727	.	nonframeshift deletion	Hnrnpm:NM_029804:exon14:c.1707_1745del:p.569_582del,Hnrnpm:NM_001109913:exon15:c.1590_1628del:p.530_543del	**530_543del**	NA	17,33649824,TCGCTCCAGGCCCATGCGCTCAAGACTGTTGGCTCCCATA,A	17qB1	.	0.25	.	31	chr17	33649823	.	TCGCTCCAGGCCCATGCGCTCAAGACTGTTGGCTCCCATA	A	.	alt_allele_in_normal;t_lod_fstar	ECNT=1;HCNT=1;MAX_ED=.;MIN_ED=.;NLOD=10.97;TLOD=4.87	GT:AD:AF:ALT_F1R2:ALT_F2R1:FOXOG:QSS:REF_F1R2:REF_F2R1	0/1:47,7:0.115:4:3:.:1154,137:23:24	0/0:41,6:0.130:4:2:.:1119,117:17:24	2.7	Unknown	.	.	.	.	.	.	.	.
T12df	chr17	**1**	**12.5**	33649824	33649862	CGCTCCAGGCCCATGCGCTCAAGACTGTTGGCTCCCATA	-	exonic	**Hnrnpm**	ENSMUSP00000031727	.	nonframeshift deletion	Hnrnpm:NM_029804:exon14:c.1707_1745del:p.569_582del,Hnrnpm:NM_001109913:exon15:c.1590_1628del:p.530_543del	**569_582del**	NA	17,33649824,TCGCTCCAGGCCCATGCGCTCAAGACTGTTGGCTCCCATA,G	17qB1	.	0.25	.	31	chr17	33649823	.	TCGCTCCAGGCCCATGCGCTCAAGACTGTTGGCTCCCATA	G	.	str_contraction;t_lod_fstar	ECNT=1;HCNT=1;MAX_ED=.;MIN_ED=.;NLOD=9.31;RPA=6,5;RU=GCT;STR;TLOD=5.38	GT:AD:AF:ALT_F1R2:ALT_F2R1:FOXOG:QSS:REF_F1R2:REF_F2R1	0/1:53,2:0.041:1:1:.:1349,47:33:20	0/0:33,0:0.00:0:0:.:765,0:19:14	2.7	Unknown	.	.	.	.	.	.	.	.
T1df	chr6	**1**	**12.5**	52234493	52234495	GCT	-	exonic	**Hoxa10**	ENSMUSP00000141929	.	nonframeshift deletion	Hoxa10:NM_008263:exon1:c.440_442del:p.147_148del	**147_148del**	NA	6,52234493,GGCT,A	6qB3	.	0.25	.	33	chr6	52234492	.	GGCT	A	.	PASS	DB;ECNT=1;HCNT=1;MAX_ED=.;MIN_ED=.;NLOD=11.69;TLOD=94.92	GT:AD:AF:ALT_F1R2:ALT_F2R1:FOXOG:QSS:REF_F1R2:REF_F2R1	0/1:38,36:0.507:25:11:0.306:1069,1020:23:15	0/0:45,0:0.00:0:0:.:1275,0:22:23	2.7	Unknown	.	.	.	.	.	.	.	.
T32df	chr16	**1**	**12.5**	22961055	22961174	GTCACCATCCCCCCGGTCACCATTCCCATGGTCACCATCCCCACGGTCACCATCCCCACAGTCACCATTCCCACGGTCACCATCCCCCCGGTCACCATCCCCATGGTCACCACCCCCACG	-	exonic	**Hrg**	ENSMUSP00000057563	.	nonframeshift deletion	Hrg:NM_053176:exon7:c.1082_1201del:p.361_401del	**361_401del**	NA	16,22961055,AGTCACCATCCCCCCGGTCACCATTCCCATGGTCACCATCCCCACGGTCACCATCCCCACAGTCACCATTCCCACGGTCACCATCCCCCCGGTCACCATCCCCATGGTCACCACCCCCACG,C	16qB1	.	0.25	.	47	chr16	22961054	.	AGTCACCATCCCCCCGGTCACCATTCCCATGGTCACCATCCCCACGGTCACCATCCCCACAGTCACCATTCCCACGGTCACCATCCCCCCGGTCACCATCCCCATGGTCACCACCCCCACG	C	.	t_lod_fstar	ECNT=1;HCNT=1;MAX_ED=.;MIN_ED=.;NLOD=10.89;TLOD=4.98	GT:AD:AF:ALT_F1R2:ALT_F2R1:FOXOG:QSS:REF_F1R2:REF_F2R1	0/1:37,3:0.079:2:1:0.667:1038,81:16:21	0/0:44,1:0.025:1:0:1.00:1160,10:22:22	0.8	Unknown	.	.	.	.	.	.	.	.
T22df	chr9	**1**	**12.5**	65128855	65128855	G	C	exonic	**Igdcc4**	ENSMUSP00000044801	.	nonsynonymous SNV	Igdcc4:NM_001290315:exon12:c.G2198C:p.G733A,Igdcc4:NM_020043:exon12:c.G2195C:p.G732A	**G732A**	ENSMUSP00000044801,732,G,A	9,65128855,G,A	9qC	.	0.25	.	45	chr9	65128855	.	G	A	.	PASS	DB;ECNT=1;HCNT=6;MAX_ED=.;MIN_ED=.;NLOD=7.82;TLOD=28.96	GT:AD:AF:ALT_F1R2:ALT_F2R1:FOXOG:QSS:REF_F1R2:REF_F2R1	0/1:15,11:0.423:5:6:0.455:390,314:8:7	0/0:30,0:0.00:0:0:.:797,0:14:16	1.7	Tolerated	-1.85	Neutral	136	30	0.065	Tolerated	3.03	53

T22df	chr9	**1**	**12.5**	65128855	65128855	G	C	exonic	**Igdcc4**	ENSMUSP00000044801	.	nonsynonymous SNV	Igdcc4:NM_001290315:exon12:c.G2198C:p.G733A,Igdcc4:NM_020043:exon12:c.G2195C:p.G732A	**G733A**	ENSMUSP00000044801,733,G,A	9,65128855,G,A	9qC	.	0.25	.	45	chr9	65128855	.	G	A	.	alt_allele_in_normal	ECNT=1;HCNT=21;MAX_ED=.;MIN_ED=.;NLOD=2.24;TLOD=12.13	GT:AD:AF:ALT_F1R2:ALT_F2R1:FOXOG:QSS:REF_F1R2:REF_F2R1	0/1:66,7:0.099:2:5:0.714:1863,187:34:32	0/0:90,10:0.102:3:7:0.700:2518,272:37:53	0.8	Tolerated	-1.85	Neutral	136	30	0.065	Tolerated	3.03	53
T3df	chr17	**1**	**12.5**	12701243	12701243	G	A	exonic	**Igf2r**	ENSP00000349437	.	nonsynonymous SNV	Igf2r:NM_010515:exon30:c.C4208T:p.S1403F	**S1403F**	ENSP00000349437,140,S,3F	17,12701243,G,A	17qA1	.	0.25	.	38	chr17	12701243	.	G	A	.	t_lod_fstar	ECNT=1;HCNT=2;MAX_ED=.;MIN_ED=.;NLOD=9.93;TLOD=4.47	GT:AD:AF:ALT_F1R2:ALT_F2R1:FOXOG:QSS:REF_F1R2:REF_F2R1	0/1:64,3:0.046:3:0:1.00:1808,83:39:25	0/0:38,0:0.00:0:0:.:1102,0:20:18	1.2	Deleterious	-4.86	Deleterious	87	30	0.001	Damaging	2.85	66
T3df	chr5	**1**	**12.5**	77407548	77407548	C	A	exonic	**Igfbp7**	ENSMUSP00000031817	.	nonsynonymous SNV	Igfbp7:NM_001159518:exon1:c.G467T:p.C156F,Igfbp7:NM_008048:exon1:c.G467T:p.C156F	**C156F**	ENSMUSP00000031817,156,C,F	5,77407548,C,A	5qC3.3	.	0.25	.	100	chr5	77407548	.	C	A	.	PASS	DB;ECNT=1;HCNT=6;MAX_ED=.;MIN_ED=.;NLOD=7.82;TLOD=28.96	GT:AD:AF:ALT_F1R2:ALT_F2R1:FOXOG:QSS:REF_F1R2:REF_F2R1	0/1:15,11:0.423:5:6:0.455:390,314:8:7	0/0:30,0:0.00:0:0:.:797,0:14:16	1.7	Deleterious	-9.9	Deleterious	132	30	0.001	Damaging	2.83	110
T1df	chr3	**1**	**12.5**	90396404	90396404	G	C	exonic	**Ints3**	ENSMUSP00000074238	.	nonsynonymous SNV	Ints3:NM_145540:exon21:c.C2215G:p.H739D,Ints3:NM_178876:exon21:c.C2215G:p.H739D	**H739D**	ENSMUSP00000074238,739,H,D	3,90396404,G,T	3qF1	.	0.25	.	39	chr3	90396404	.	G	T	.	clustered_events;homologous_mapping_event;t_lod_fstar	ECNT=9;HCNT=2;MAX_ED=27;MIN_ED=4;NLOD=9.63;TLOD=5.10	GT:AD:AF:ALT_F1R2:ALT_F2R1:FOXOG:PGT:PID:QSS:REF_F1R2:REF_F2R1	0/1:70,2:0.030:0:2:.:0|1:90396389_TGGAA_T:2045,58:35:35	0/0:36,0:0.00:0:0:.:0|1:90396389_TGGAA_T:1017,0:22:14	19	Deleterious	-2.78	Deleterious	108	30	0.036	Damaging	2.85	62
T1df	chr3	**1**	**12.5**	90396393	90396393	-	CTC	exonic	**Ints3**	ENSMUSP00000074238	.	nonframeshift insertion	Ints3:NM_145540:exon21:c.2225_2226insGAG:p.P742delinsPS,Ints3:NM_178876:exon21:c.2225_2226insGAG:p.P742delinsPS	**P742delinsPS**	ENSMUSP00000074238,742,P,PS	3,90396393,A,GAAAAAA	3qF1	.	0.25	.	36	chr3	90396393	.	A	GAAAAAA	.	clustered_events;homologous_mapping_event;t_lod_fstar	ECNT=9;HCNT=2;MAX_ED=27;MIN_ED=4;NLOD=9.93;TLOD=5.14	GT:AD:AF:ALT_F1R2:ALT_F2R1:FOXOG:PGT:PID:QSS:REF_F1R2:REF_F2R1	0/1:64,2:0.031:0:2:.:0|1:90396389_TGGAA_T:1704,52:31:33	0/0:36,0:0.00:0:0:.:0|1:90396389_TGGAA_T:904,0:21:15	19	Deleterious	-9.17	Deleterious	108	30	NA	NA	NA	NA
T1df	chr3	**1**	**12.5**	90396406	90396406	-	CGT	exonic	**Ints3**	ENSMUSP00000074238	.	nonframeshift insertion	Ints3:NM_145540:exon21:c.2212_2213insACG:p.C738delinsYG,Ints3:NM_178876:exon21:c.2212_2213insACG:p.C738delinsYG	**C738delinsYG**	ENSMUSP00000074238,738,C,YG	3,90396406,C,A	3qF1	.	0.25	.	38	chr3	90396406	.	C	A	.	clustered_events;multi_event_alt_allele_in_normal	ECNT=2;HCNT=1;MAX_ED=4;MIN_ED=4;NLOD=6.34;TLOD=10.24	GT:AD:AF:ALT_F1R2:ALT_F2R1:FOXOG:PGT:PID:QSS:REF_F1R2:REF_F2R1	0/1:62,4:0.065:1:3:0.750:0|1:8897538_C_A:1729,111:36:26	0/0:40,1:0.028:1:0:0.00:0|1:8897538_C_A:1117,27:19:21	0.9	Deleterious	-2.88	Deleterious	108	30	0.162	Tolerated	2.85	62
T1df	chr3	**1**	**12.5**	90396413	90396413	G	T	exonic	**Ints3**	ENSMUSP00000074238	.	nonsynonymous SNV	Ints3:NM_145540:exon21:c.C2206A:p.L736M,Ints3:NM_178876:exon21:c.C2206A:p.L736M	**L736M**	ENSMUSP00000074238,736,L,M	3,90396413,G,T	3qF1	.	0.25	.	38	chr3	90396413	.	G	T	.	clustered_events;homologous_mapping_event;t_lod_fstar	ECNT=9;HCNT=2;MAX_ED=27;MIN_ED=4;NLOD=10.54;TLOD=5.08	GT:AD:AF:ALT_F1R2:ALT_F2R1:FOXOG:PGT:PID:QSS:REF_F1R2:REF_F2R1	0/1:69,2:0.029:0:2:.:0|1:90396389_TGGAA_T:1932,49:35:34	0/0:40,0:0.00:0:0:.:0|1:90396389_TGGAA_T:1060,0:25:15	19	Tolerated	-0.58	Neutral	108	30	0.457	Tolerated	2.85	62
T1df	chr3	**1**	**12.5**	90396416	90396416	-	AAAAAA	exonic	**Ints3**	ENSMUSP00000074238	.	nonframeshift insertion	Ints3:NM_145540:exon21:c.2202_2203insTTTTTT:p.R735delinsFFR,Ints3:NM_178876:exon21:c.2202_2203insTTTTTT:p.R735delinsFFR	**R735delinsFFR**	ENSMUSP00000074238,735,R,FFR	3,90396416,G,G	3qF1	.	0.25	.	36	chr3	90396416	.	G	G	.	clustered_events;multi_event_alt_allele_in_normal	ECNT=2;HCNT=1;MAX_ED=4;MIN_ED=4;NLOD=6.34;TLOD=10.18	GT:AD:AF:ALT_F1R2:ALT_F2R1:FOXOG:PGT:PID:QSS:REF_F1R2:REF_F2R1	0/1:62,3:0.063:1:2:0.333:0|1:8897538_C_A:1749,81:35:27	0/0:38,1:0.028:1:0:1.00:0|1:8897538_C_A:1051,27:17:21	0.9	Tolerated	0	Neutral	108	30	1	Tolerated	2.85	62
T1df	chr3	**1**	**12.5**	90396390	90396393	GGAA	-	exonic	**Ints3**	ENSMUSP00000074238	.	frameshift deletion	Ints3:NM_145540:exon21:c.2226_2229del:p.P742fs,Ints3:NM_178876:exon21:c.2226_2229del:p.P742fs	**P742fs**	ENSMUSP00000074238,742,P,.	3,90396390,TGGAA,T	3qF1	.	0.25	.	36	chr3	90396389	.	TGGAA	T	.	clustered_events;homologous_mapping_event;t_lod_fstar	ECNT=9;HCNT=2;MAX_ED=27;MIN_ED=4;NLOD=10.23;TLOD=5.10	GT:AD:AF:ALT_F1R2:ALT_F2R1:FOXOG:PGT:PID:QSS:REF_F1R2:REF_F2R1	0/1:64,2:0.030:0:2:.:0|1:90396389_TGGAA_T:1865,48:32:32	0/0:39,0:0.00:0:0:.:0|1:90396389_TGGAA_T:1094,0:23:16	19	Unknown	NA	NA	108	30	NA	NA	NA	NA
T1df	chr3	**1**	**12.5**	90396397	90396400	GTGA	-	exonic	**Ints3**	ENSMUSP00000074238	.	frameshift deletion	Ints3:NM_145540:exon21:c.2219_2222del:p.L740fs,Ints3:NM_178876:exon21:c.2219_2222del:p.L740fs	**L740fs**	ENSMUSP00000074238,740,L,.	3,90396397,TGTGA,T	3qF1	.	0.25	.	40	chr3	90396396	.	TGTGA	T	.	clustered_events;homologous_mapping_event;t_lod_fstar	ECNT=9;HCNT=2;MAX_ED=27;MIN_ED=4;NLOD=10.23;TLOD=5.12	GT:AD:AF:ALT_F1R2:ALT_F2R1:FOXOG:PGT:PID:QSS:REF_F1R2:REF_F2R1	0/1:64,2:0.031:0:2:.:0|1:90396389_TGGAA_T:1803,42:31:33	0/0:37,0:0.00:0:0:.:0|1:90396389_TGGAA_T:1028,0:22:15	19	Unknown	NA	NA	108	30	NA	NA	NA	NA
T1df	chr3	**1**	**12.5**	90396409	90396410	AG	-	exonic	**Ints3**	ENSMUSP00000074238	.	frameshift deletion	Ints3:NM_145540:exon21:c.2209_2210del:p.L737fs,Ints3:NM_178876:exon21:c.2209_2210del:p.L737fs	**L737fs**	ENSMUSP00000074238,737,L,.	3,90396409,TAG,ACTC	3qF1	.	0.25	.	39	chr3	90396408	.	TAG	ACTC	.	clustered_events;homologous_mapping_event;t_lod_fstar	ECNT=9;HCNT=2;MAX_ED=27;MIN_ED=4;NLOD=9.63;TLOD=5.10	GT:AD:AF:ALT_F1R2:ALT_F2R1:FOXOG:PGT:PID:QSS:REF_F1R2:REF_F2R1	0/1:67,2:0.030:0:2:.:0|1:90396389_TGGAA_T:1866,56:34:33	0/0:36,0:0.00:0:0:.:0|1:90396389_TGGAA_T:959,0:22:14	19	Unknown	NA	NA	108	30	NA	NA	NA	NA

T1df	chr3	**1**	**12.5**	90396415	90396416	CG	-	exonic	**Ints3**	ENSMUSP00000074238	.	frameshift deletion	Ints3:NM_145540:exon21:c.2203_2204del:p.R735fs,Ints3:NM_178876:exon21:c.2203_2204del:p.R735fs	**R735fs**	ENSMUSP00000074238,735,R,.	3,90396415,TCG,CCGT	3qF1	.	0.25	.	37	chr3	90396414	.	TCG	CCGT	.	clustered_events;homologous_mapping_event;t_lod_fstar	ECNT=9;HCNT=2;MAX_ED=27;MIN_ED=4;NLOD=10.54;TLOD=5.08	GT:AD:AF:ALT_F1R2:ALT_F2R1:FOXOG:PGT:PID:QSS:REF_F1R2:REF_F2R1	0/1:65,2:0.029:0:2:.:0|1:90396389_TGGAA_T:1863,58:33:32	0/0:38,0:0.00:0:0:.:0|1:90396389_TGGAA_T:1094,0:23:15	19	Unknown	NA	NA	108	30	NA	NA	NA	NA
T32df	chr3	**1**	**12.5**	96658246	96658246	T	-	exonic	**Itga10**	ENSMUSP00000110027	.	frameshift deletion	Itga10:NM_001302471:exon27:c.3185delT:p.V1062fs	**V1062fs**	ENSMUSP00000110027,1062,V,.	3,96658246,GT,A	3qF2.1	.	0.25	.	45	chr3	96658245	.	GT	A	.	alt_allele_in_normal;clustered_events	ECNT=2;HCNT=3;MAX_ED=15;MIN_ED=15;NLOD=9.74;TLOD=9.99	GT:AD:AF:ALT_F1R2:ALT_F2R1:FOXOG:PGT:PID:QSS:REF_F1R2:REF_F2R1	0/1:148,4:0.027:2:2:0.500:0|1:171531090_G_A:3830,112:70:78	0/0:146,8:0.051:2:6:0.250:0|1:171531090_G_A:3635,219:85:61	20	Unknown	NA	NA	176	30	NA	NA	NA	NA
T22df	chr1	**1**	**12.5**	171531181	171531181	G	T	exonic	**Itln1**	ENSMUSP00000116671	.	stopgain	Itln1:NM_010584:exon5:c.C417A:p.Y139X	**Y139X**	ENSMUSP00000116671,139,Y,X	NA	1qH3	.	0.25	.	118	chr1	171531181	.	G	C	.	alt_allele_in_normal	ECNT=1;HCNT=14;MAX_ED=.;MIN_ED=.;NLOD=8.73;TLOD=23.81	GT:AD:AF:ALT_F1R2:ALT_F2R1:FOXOG:QSS:REF_F1R2:REF_F2R1	0/1:215,19:0.082:6:13:0.316:5980,523:101:114	0/0:122,9:0.074:6:3:0.667:3497,242:66:56	20	Deletarious	.	.	.	.	.	.	.	.
T3df	chr1	**1**	**12.5**	171531528	171531528	G	C	exonic	**Itln1**	ENSMUSP00000120115	.	nonsynonymous SNV	Itln1:NM_010584:exon4:c.C383G:p.A128G	**A128G**	ENSMUSP00000120115,128,A,G	1,171531528,G,G	1qH3	rs49646502	0.25	.	131	chr1	171531528	.	G	G	.	clustered_events;multi_event_alt_allele_in_normal	ECNT=9;HCNT=1;MAX_ED=23;MIN_ED=1;NLOD=2.42;TLOD=8.91	GT:AD:AF:ALT_F1R2:ALT_F2R1:FOXOG:PGT:PID:QSS:REF_F1R2:REF_F2R1	0/1:37,3:0.077:0:3:1.00:0|1:146323174_C_G:1007,56:20:17	0/0:26,1:0.043:0:1:1.00:0|1:146323174_C_G:711,15:19:7	2.5	Deleterious	-2.84	Deleterious	168	30	0	Damaging	2.87	259
T3df	chr1	**1**	**12.5**	171518329	171518329	-	C	exonic	**Itln1**	ENSMUSP00000116671	.	frameshift insertion	Itln1:NM_010584:exon8:c.853_854insG:p.S285fs	**S285fs**	ENSMUSP00000116671,285,S,	1,171518329,G,G	1qH3	.	0.25	.	55	chr1	171518329	.	G	G	.	clustered_events;multi_event_alt_allele_in_normal	ECNT=9;HCNT=1;MAX_ED=23;MIN_ED=1;NLOD=2.42;TLOD=8.87	GT:AD:AF:ALT_F1R2:ALT_F2R1:FOXOG:PGT:PID:QSS:REF_F1R2:REF_F2R1	0/1:37,3:0.075:0:3:0.00:0|1:146323174_C_G:1016,54:20:17	0/0:26,1:0.043:0:1:0.00:0|1:146323174_C_G:737,8:19:7	2.5	Tolerated	0	Neutral	144	30	0.493	Tolerated	3.13	146
T2df	chr1	**1**	**12.5**	171518321	171518321	A	C	exonic	**Itln1**	ENSMUSP00000116671	.	nonsynonymous SNV	Itln1:NM_010584:exon8:c.T862G:p.W288G	**W288G**	ENSMUSP00000116671,288,W,G	1,171518321,A,GC	1qH3	.	0.25	.	48	chr1	171518321	.	A	GC	.	alt_allele_in_normal;clustered_events	ECNT=5;HCNT=2;MAX_ED=127;MIN_ED=1;NLOD=6.35;TLOD=13.58	GT:AD:AF:ALT_F1R2:ALT_F2R1:FOXOG:PGT:PID:QSS:REF_F1R2:REF_F2R1	0/1:52,1:0.088:0:1:.:0|1:171518329_G_GC:1456,28:32:20	0/0:54,1:0.039:1:0:.:0|1:171518329_G_GC:1541,29:29:25	20	Unknown	NA	NA	144	30	NA	NA	NA	NA
T22df	chr1	**1**	**12.5**	171531193	171531193	C	T	splicing	**Itln1**	ENSMUSP00000116671	.	NM_010584:exon5:c.406-1G>A	.	**NA**	1,171531193,C,C	NA	1qH3	.	0.25	.	108	chr1	171531193	.	C	C	.	alt_allele_in_normal;clustered_events;germline_risk	ECNT=2;HCNT=4;MAX_ED=120;MIN_ED=120;NLOD=0.648;TLOD=15.03	GT:AD:AF:ALT_F1R2:ALT_F2R1:FOXOG:QSS:REF_F1R2:REF_F2R1	0/1:56,4:0.083:4:0:0.00:1541,117:28:28	0/0:47,1:0.064:1:0:0.00:1265,30:27:20	20	Unkown	.	.	.	.	.	.	.	.
T22df	chr6	**1**	**12.5**	146323174	146323174	C	G	exonic	**Itpr2**	ENSMUSP00000023590	.	nonsynonymous SNV	Itpr2:NM_010586:exon31:c.G4218C:p.K1406N,Itpr2:NM_019923:exon32:c.G4317C:p.K1439N	**K1406N**	ENSMUSP00000023590,1406,K,N	6,146323174,C,G	6qG3	.	0.25	.	27	chr6	146323174	.	C	G	.	clustered_events;multi_event_alt_allele_in_normal	ECNT=9;HCNT=1;MAX_ED=23;MIN_ED=1;NLOD=2.42;TLOD=8.84	GT:AD:AF:ALT_F1R2:ALT_F2R1:FOXOG:PGT:PID:QSS:REF_F1R2:REF_F2R1	0/1:38,3:0.073:0:3:1.00:0|1:146323174_C_G:1066,63:20:18	0/0:26,1:0.043:0:1:1.00:0|1:146323174_C_G:732,10:19:7	2.5	Deleterious	-4.69	Deleterious	198	30	0	Damaging	2.87	227
T22df	chr6	**1**	**12.5**	146323174	146323174	C	G	exonic	**Itpr2**	ENSMUSP00000023590	.	nonsynonymous SNV	Itpr2:NM_010586:exon31:c.G4218C:p.K1406N,Itpr2:NM_019923:exon32:c.G4317C:p.K1439N	**K1439N**	ENSMUSP00000023590,1439,K,N	6,146323174,C,G	6qG3	.	0.25	.	27	chr6	146323174	.	C	G	.	clustered_events;multi_event_alt_allele_in_normal	ECNT=9;HCNT=1;MAX_ED=23;MIN_ED=1;NLOD=2.42;TLOD=8.84	GT:AD:AF:ALT_F1R2:ALT_F2R1:FOXOG:PGT:PID:QSS:REF_F1R2:REF_F2R1	0/1:38,3:0.073:0:3:1.00:0|1:146323174_C_G:1066,63:20:18	0/0:26,1:0.043:0:1:1.00:0|1:146323174_C_G:732,10:19:7	2.5	Deleterious	-4.69	Deleterious	198	30	0	Damaging	2.87	227
T22df	chr6	**1**	**12.5**	146323175	146323175	T	G	exonic	**Itpr2**	ENSMUSP00000023590	.	nonsynonymous SNV	Itpr2:NM_010586:exon31:c.A4217C:p.K1406T,Itpr2:NM_019923:exon32:c.A4316C:p.K1439T	**K1406T**	ENSMUSP00000023590,1406,K,T	6,146323175,T,G	6qG3	.	0.25	.	27	chr6	146323175	.	T	G	.	clustered_events;multi_event_alt_allele_in_normal	ECNT=9;HCNT=1;MAX_ED=23;MIN_ED=1;NLOD=2.72;TLOD=8.81	GT:AD:AF:ALT_F1R2:ALT_F2R1:FOXOG:PGT:PID:QSS:REF_F1R2:REF_F2R1	0/1:38,3:0.071:0:3:1.00:0|1:146323174_C_G:1014,57:20:18	0/0:26,1:0.042:0:1:1.00:0|1:146323174_C_G:731,19:19:7	2.5	Deleterious	-5.63	Deleterious	198	30	0	Damaging	2.87	227

T22df	chr6	**1**	**12.5**	146323175	146323175	T	G	exonic	**Itpr2**	ENSMUSP00000023590	.	nonsynonymous SNV	Itpr2:NM_010586:exon31:c.A4217C:p.K1406T,Itpr2:NM_019923:exon32:c.A4316C:p.K1439T	**K1439T**	ENSMUSP00000023590,1439,K,T	6,146323175,T,G	6qG3	.	0.25	.	27	chr6	146323175	.	T	G	.	clustered_events;multi_event_alt_allele_in_normal	ECNT=9;HCNT=1;MAX_ED=23;MIN_ED=1;NLOD=2.72;TLOD=8.81	GT:AD:AF:ALT_F1R2:ALT_F2R1:FOXOG:PGT:PID:QSS:REF_F1R2:REF_F2R1	0/1:38,3:0.071:0:3:1.00:0|1:146323174_C_G:1014,57:20:18	0/0:26,1:0.042:0:1:1.00:0|1:146323174_C_G:731,19:19:7	2.5	Deleterious	-5.63	Deleterious	198	30	0	Damaging	2.87	227
T22df	chr6	**1**	**12.5**	146323177	146323177	C	G	exonic	**Itpr2**	ENSMUSP00000023590	.	nonsynonymous SNV	Itpr2:NM_010586:exon31:c.G4215C:p.M1405I,Itpr2:NM_019923:exon32:c.G4314C:p.M1438I	**M1405I**	ENSMUSP00000023590,1405,M,I	6,146323177,C,G	6qG3	.	0.25	.	27	chr6	146323177	.	C	G	.	clustered_events;multi_event_alt_allele_in_normal	ECNT=9;HCNT=1;MAX_ED=23;MIN_ED=1;NLOD=2.72;TLOD=8.87	GT:AD:AF:ALT_F1R2:ALT_F2R1:FOXOG:PGT:PID:QSS:REF_F1R2:REF_F2R1	0/1:37,3:0.075:0:3:0.00:0|1:146323174_C_G:1040,38:18:19	0/0:27,1:0.042:0:1:0.00:0|1:146323174_C_G:753,13:20:7	2.5	Deleterious	-3.55	Deleterious	198	30	0.002	Damaging	2.87	227
T22df	chr6	**1**	**12.5**	146323177	146323177	C	G	exonic	**Itpr2**	ENSMUSP00000023590	.	nonsynonymous SNV	Itpr2:NM_010586:exon31:c.G4215C:p.M1405I,Itpr2:NM_019923:exon32:c.G4314C:p.M1438I	**M1438I**	ENSMUSP00000023590,1438,M,I	6,146323177,C,G	6qG3	.	0.25	.	27	chr6	146323177	.	C	G	.	clustered_events;multi_event_alt_allele_in_normal	ECNT=9;HCNT=1;MAX_ED=23;MIN_ED=1;NLOD=2.72;TLOD=8.87	GT:AD:AF:ALT_F1R2:ALT_F2R1:FOXOG:PGT:PID:QSS:REF_F1R2:REF_F2R1	0/1:37,3:0.075:0:3:0.00:0|1:146323174_C_G:1040,38:18:19	0/0:27,1:0.042:0:1:0.00:0|1:146323174_C_G:753,13:20:7	2.5	Deleterious	-3.55	Deleterious	198	30	0.002	Damaging	2.87	227
T22df	chr6	**1**	**12.5**	146323181	146323181	T	G	exonic	**Itpr2**	ENSMUSP00000123580	.	nonsynonymous SNV	Itpr2:NM_010586:exon31:c.A4211C:p.E1404A,Itpr2:NM_019923:exon32:c.A4310C:p.E1437A	**E1437A**	ENSMUSP00000123580,1437,E,A	6,146323181,T,C	6qG3	.	0.25	.	28	chr6	146323181	.	T	C	.	alt_allele_in_normal;clustered_events	ECNT=9;HCNT=1;MAX_ED=23;MIN_ED=1;NLOD=3.03;TLOD=8.87	GT:AD:AF:ALT_F1R2:ALT_F2R1:FOXOG:PGT:PID:QSS:REF_F1R2:REF_F2R1	0/1:39,3:0.075:0:3:.:0|1:146323174_C_G:1114,81:18:21	0/0:26,1:0.040:0:1:.:0|1:146323174_C_G:713,25:19:7	2.5	Deleterious	-6.56	Deleterious	198	30	0	Damaging	2.87	227
T22df	chr6	**1**	**12.5**	146323181	146323181	T	G	exonic	**Itpr2**	ENSMUSP00000023590	.	nonsynonymous SNV	Itpr2:NM_010586:exon31:c.A4211C:p.E1404A,Itpr2:NM_019923:exon32:c.A4310C:p.E1437A	**E1404A**	ENSMUSP00000023590,1404,E,A	6,146323181,T,G	6qG3	.	0.25	.	28	chr6	146323181	.	T	G	.	clustered_events;multi_event_alt_allele_in_normal	ECNT=9;HCNT=1;MAX_ED=23;MIN_ED=1;NLOD=2.42;TLOD=8.91	GT:AD:AF:ALT_F1R2:ALT_F2R1:FOXOG:PGT:PID:QSS:REF_F1R2:REF_F2R1	0/1:37,3:0.077:0:3:1.00:0|1:146323174_C_G:1007,56:20:17	0/0:26,1:0.043:0:1:1.00:0|1:146323174_C_G:711,15:19:7	2.5	Deleterious	-5.63	Deleterious	198	30	0	Damaging	2.87	227
T22df	chr6	**1**	**12.5**	146323194	146323194	-	ATTAAA	exonic	**Itpr2**	ENSMUSP00000123580	.	nonframeshift insertion	Itpr2:NM_010586:exon31:c.4197_4198insTTTAAT:p.D1400delinsFND,Itpr2:NM_019923:exon32:c.4296_4297insTTTAAT:p.D1433delinsFND	**D1433delinsFND**	ENSMUSP00000123580,1433,D,FND	6,146323194,C,G	6qG3	.	0.25	.	26	chr6	146323194	.	C	G	.	alt_allele_in_normal;clustered_events;germline_risk	ECNT=6;HCNT=1;MAX_ED=25;MIN_ED=10;NLOD=1.23;TLOD=8.44	GT:AD:AF:ALT_F1R2:ALT_F2R1:FOXOG:PGT:PID:QSS:REF_F1R2:REF_F2R1	0/1:55,4:0.055:4:0:0.00:0|1:90906153_TGCAGA_T:1544,74:20:35	0/0:35,2:0.059:2:0:0.00:0|1:90906153_TGCAGA_T:944,45:16:19	0.8	Deleterious	-6.56	Deleterious	198	30	0	Damaging	2.87	227
T22df	chr6	**1**	**12.5**	146323194	146323194	-	ATTAAA	exonic	**Itpr2**	ENSMUSP00000123580	.	nonframeshift insertion	Itpr2:NM_010586:exon31:c.4197_4198insTTTAAT:p.D1400delinsFND,Itpr2:NM_019923:exon32:c.4296_4297insTTTAAT:p.D1433delinsFND	**D1400delinsFND**	ENSMUSP00000123580,1400,D,FND	6,146323194,C,T	6qG3	.	0.25	.	26	chr6	146323194	.	C	T	.	alt_allele_in_normal;clustered_events;germline_risk	ECNT=6;HCNT=1;MAX_ED=25;MIN_ED=10;NLOD=0.934;TLOD=10.98	GT:AD:AF:ALT_F1R2:ALT_F2R1:FOXOG:PGT:PID:QSS:REF_F1R2:REF_F2R1	0/1:54,3:0.069:3:0:0.00:0|1:90906153_TGCAGA_T:1462,52:19:35	0/0:36,2:0.061:2:0:0.00:0|1:90906153_TGCAGA_T:980,45:15:21	0.8	Deleterious	-4.69	Deleterious	198	30	0.001	Damaging	2.87	227
T22df	chr6	**1**	**12.5**	146323178	146323178	A	G	exonic	**Itpr2**	ENSMUSP00000023590	.	nonsynonymous SNV	Itpr2:NM_010586:exon31:c.T4214C:p.M1405T,Itpr2:NM_019923:exon32:c.T4313C:p.M1438T	**M1405T**	ENSMUSP00000023590,1405,M,T	6,146323178,A,A	6qG3	.	0.25	.	27	chr6	146323178	.	A	A	.	clustered_events;multi_event_alt_allele_in_normal	ECNT=9;HCNT=1;MAX_ED=23;MIN_ED=1;NLOD=2.72;TLOD=8.84	GT:AD:AF:ALT_F1R2:ALT_F2R1:FOXOG:PGT:PID:QSS:REF_F1R2:REF_F2R1	0/1:39,3:0.073:0:3:1.00:0|1:146323174_C_G:883,28:19:20	0/0:25,1:0.042:0:1:1.00:0|1:146323174_C_G:604,7:18:7	2.5	Tolerated	0	Neutral	198	30	1	Tolerated	2.87	227
T22df	chr6	**1**	**12.5**	146323178	146323178	A	G	exonic	**Itpr2**	ENSMUSP00000023590	.	nonsynonymous SNV	Itpr2:NM_010586:exon31:c.T4214C:p.M1405T,Itpr2:NM_019923:exon32:c.T4313C:p.M1438T	**M1438T**	ENSMUSP00000023590,1438,M,T	6,146323178,A,A	6qG3	.	0.25	.	27	chr6	146323178	.	A	A	.	clustered_events;multi_event_alt_allele_in_normal	ECNT=9;HCNT=1;MAX_ED=23;MIN_ED=1;NLOD=2.72;TLOD=8.84	GT:AD:AF:ALT_F1R2:ALT_F2R1:FOXOG:PGT:PID:QSS:REF_F1R2:REF_F2R1	0/1:39,3:0.073:0:3:1.00:0|1:146323174_C_G:883,28:19:20	0/0:25,1:0.042:0:1:1.00:0|1:146323174_C_G:604,7:18:7	2.5	Tolerated	0	Neutral	198	30	1	Tolerated	2.87	227
T22df	chr6	**1**	**12.5**	146323197	146323197	C	A	exonic	**Itpr2**	ENSMUSP00000123580	.	nonsynonymous SNV	Itpr2:NM_010586:exon31:c.G4195T:p.V1399F,Itpr2:NM_019923:exon32:c.G4294T:p.V1432F	**V1432F**	ENSMUSP00000123580,1432,V,F	6,146323197,C,C	6qG3	.	0.25	.	26	chr6	146323197	.	C	C	.	alt_allele_in_normal;clustered_events	ECNT=9;HCNT=1;MAX_ED=23;MIN_ED=1;NLOD=3.03;TLOD=8.87	GT:AD:AF:ALT_F1R2:ALT_F2R1:FOXOG:PGT:PID:QSS:REF_F1R2:REF_F2R1	0/1:39,3:0.075:0:3:.:0|1:146323174_C_G:1114,81:18:21	0/0:26,1:0.040:0:1:.:0|1:146323174_C_G:713,25:19:7	2.5	Tolerated	0	Neutral	198	30	1	Tolerated	2.87	227

T22df	chr6	**1**	**12.5**	146323197	146323197	C	A	exonic	**Itpr2**	ENSMUSP00000023590	.	nonsynonymous SNV	Itpr2:NM_010586:exon31:c.G4195T:p.V1399F,Itpr2:NM_019923:exon32:c.G4294T:p.V1432F	**V1399F**	ENSMUSP00000023590,1399,V,F	6,146323197,C,G	6qG3	.	0.25	.	26	chr6	146323197	.	C	G	.	clustered_events;multi_event_alt_allele_in_normal	ECNT=9;HCNT=1;MAX_ED=23;MIN_ED=1;NLOD=2.42;TLOD=8.87	GT:AD:AF:ALT_F1R2:ALT_F2R1:FOXOG:PGT:PID:QSS:REF_F1R2:REF_F2R1	0/1:37,3:0.075:0:3:0.00:0|1:146323174_C_G:1016,54:20:17	0/0:26,1:0.043:0:1:0.00:0|1:146323174_C_G:737,8:19:7	2.5	Tolerated	-2.08	Neutral	198	30	0.158	Tolerated	2.87	227
T22df	chr6	**1**	**12.5**	146323184	146323189	ACTTCA	-	exonic	**Itpr2**	ENSMUSP00000123580	.	nonframeshift deletion	Itpr2:NM_010586:exon31:c.4203_4208del:p.1401_1403del,Itpr2:NM_019923:exon32:c.4302_4307del:p.1434_1436del	**1401_1403del**	NA	6,146323184,CACTTCA,CATTAAA	6qG3	.	0.25	.	27	chr6	146323183	.	CACTTCA	CATTAAA	.	alt_allele_in_normal;clustered_events	ECNT=9;HCNT=1;MAX_ED=23;MIN_ED=1;NLOD=2.42;TLOD=8.84	GT:AD:AF:ALT_F1R2:ALT_F2R1:FOXOG:PGT:PID:QSS:REF_F1R2:REF_F2R1	0/1:38,3:0.073:0:3:.:0|1:146323174_C_G:1052,83:19:19	0/0:25,1:0.043:0:1:.:0|1:146323174_C_G:695,29:19:6	2.5	Unknown	.	.	.	.	.	.	.	.
T22df	chr6	**1**	**12.5**	146323184	146323189	ACTTCA	-	exonic	**Itpr2**	ENSMUSP00000123580	.	nonframeshift deletion	Itpr2:NM_010586:exon31:c.4203_4208del:p.1401_1403del,Itpr2:NM_019923:exon32:c.4302_4307del:p.1434_1436del	**1434_1436del**	NA	6,146323184,CACTTCA,CATTAAA	6qG3	.	0.25	.	27	chr6	146323183	.	CACTTCA	CATTAAA	.	alt_allele_in_normal;clustered_events	ECNT=9;HCNT=1;MAX_ED=23;MIN_ED=1;NLOD=2.42;TLOD=8.84	GT:AD:AF:ALT_F1R2:ALT_F2R1:FOXOG:PGT:PID:QSS:REF_F1R2:REF_F2R1	0/1:38,3:0.073:0:3:.:0|1:146323174_C_G:1052,83:19:19	0/0:25,1:0.043:0:1:.:0|1:146323174_C_G:695,29:19:6	2.5	Unknown	.	.	.	.	.	.	.	.
T1df	chr4	**1**	**12.5**	95050653	95050655	GCT	-	exonic	**Jun**	ENSMUSP00000053692	.	nonframeshift deletion	Jun:NM_010591:exon1:c.617_619del:p.206_207del	**206_207del**	NA	4,95050653,GGCT,T	4qC5	.	0.25	.	24	chr4	95050652	.	GGCT	T	.	alt_allele_in_normal;clustered_events;germline_risk	ECNT=2;HCNT=2;MAX_ED=8;MIN_ED=8;NLOD=1.87;TLOD=11.37	GT:AD:AF:ALT_F1R2:ALT_F2R1:FOXOG:PGT:PID:QSS:REF_F1R2:REF_F2R1	0/1:69,4:0.058:2:2:0.500:0|1:3344581_A_G:1931,112:38:31	0/0:64,4:0.061:3:1:0.250:0|1:3344581_A_G:1707,116:36:28	1.9	Unknown	.	.	.	.	.	.	.	.
T32df	chr1	**1**	**12.5**	15312935	15312935	C	T	exonic	**Kcnb2**	ENSMUSP00000045387	.	nonsynonymous SNV	Kcnb2:NM_001098528:exon1:c.C484T:p.R162C	**R162C**	ENSMUSP00000045387,162,R,C	1,15312935,C,A	1qA3	.	0.25	.	39	chr1	15312935	.	C	A	.	str_contraction;t_lod_fstar	ECNT=1;HCNT=1;MAX_ED=.;MIN_ED=.;NLOD=10.81;RPA=5,4;RU=GGC;STR;TLOD=5.39	GT:AD:AF:ALT_F1R2:ALT_F2R1:FOXOG:QSS:REF_F1R2:REF_F2R1	0/1:49,2:0.042:2:0:.:1375,58:28:21	0/0:41,0:0.00:0:0:.:1141,0:26:15	0.4	Deleterious	-4.41	Deleterious	149	30	0.102	Tolerated	2.79	255
T32df	chr1	**1**	**12.5**	15312941	15312941	G	T	exonic	**Kcnb2**	ENSMUSP00000045387	.	nonsynonymous SNV	Kcnb2:NM_001098528:exon1:c.G490T:p.G164C	**G164C**	ENSMUSP00000045387,164,G,C	1,15312941,G,A	1qA3	.	0.25	.	40	chr1	15312941	.	G	A	.	str_contraction;t_lod_fstar	ECNT=1;HCNT=1;MAX_ED=.;MIN_ED=.;NLOD=10.81;RPA=5,4;RU=GGC;STR;TLOD=5.39	GT:AD:AF:ALT_F1R2:ALT_F2R1:FOXOG:QSS:REF_F1R2:REF_F2R1	0/1:49,2:0.042:2:0:.:1375,58:28:21	0/0:41,0:0.00:0:0:.:1141,0:26:15	0.4	Tolerated	-1.89	Neutral	149	30	0.168	Tolerated	2.8	231
T42df	chr15	**1**	**12.5**	99232789	99232791	GCA	-	exonic	**Kcnh3**	ENSMUSP00000109641	.	nonframeshift deletion	Kcnh3:NM_010601:exon8:c.1286_1288del:p.429_430del	**429_430del**	NA	15,99232789,TGCA,C	15qF1	.	0.25	.	29	chr15	99232788	.	TGCA	C	.	str_contraction;t_lod_fstar	ECNT=1;HCNT=1;MAX_ED=.;MIN_ED=.;NLOD=6.02;RPA=7,6;RU=CTG;STR;TLOD=4.73	GT:AD:AF:ALT_F1R2:ALT_F2R1:FOXOG:QSS:REF_F1R2:REF_F2R1	0/1:54,2:0.038:0:2:.:1525,56:29:25	0/0:24,0:0.00:0:0:.:672,0:13:11	2.8	Unknown	.	.	.	.	.	.	.	.
T1df	chr18	**1**	**12.5**	45560418	45560420	GGC	-	exonic	**Kcnn2**	ENSMUSP00000024599	.	nonframeshift deletion	Kcnn2:NM_080465:exon1:c.265_267del:p.89_89del,Kcnn2:NM_001312905:exon4:c.1090_1092del:p.364_364del	**364_364del**	NA	18,45560418,AGGC,CT	18qB3	.	0.25	.	41	chr18	45560417	.	AGGC	CT	.	clustered_events;t_lod_fstar	ECNT=2;HCNT=2;MAX_ED=4;MIN_ED=4;NLOD=8.43;RPA=1,2;RU=T;STR;TLOD=5.12	GT:AD:AF:ALT_F1R2:ALT_F2R1:FOXOG:PGT:PID:QSS:REF_F1R2:REF_F2R1	0/1:64,2:0.031:0:2:.:0|1:4324519_C_CT:1779,56:35:29	0/0:29,0:0.00:0:0:.:0|1:4324519_C_CT:802,0:13:16	6	Unknown	NA	NA	208	30	NA	NA	NA	NA
T1df	chr18	**1**	**12.5**	45560418	45560420	GGC	-	exonic	**Kcnn2**	ENSMUSP00000024599	.	nonframeshift deletion	Kcnn2:NM_080465:exon1:c.265_267del:p.89_89del,Kcnn2:NM_001312905:exon4:c.1090_1092del:p.364_364del	**89_89del**	NA	18,45560418,AGGC,C	18qB3	.	0.25	.	41	chr18	45560417	.	AGGC	C	.	clustered_events;t_lod_fstar	ECNT=2;HCNT=2;MAX_ED=4;MIN_ED=4;NLOD=8.43;TLOD=5.12	GT:AD:AF:ALT_F1R2:ALT_F2R1:FOXOG:PGT:PID:QSS:REF_F1R2:REF_F2R1	0/1:65,2:0.031:0:2:0.00:0|1:4324519_C_CT:1828,54:36:29	0/0:31,0:0.00:0:0:.:0|1:4324519_C_CT:862,0:13:18	6	Unknown	.	.	.	.	.	.	.	.
T3df	chr7	**1**	**12.5**	143227048	143227048	T	G	ncRNA_exonic	**Kcnq1ot1**	NONE	.	.	.	**NA**	7,143227048,T,A	NA	7qF5	.	0.25	.	30	chr7	143227048	.	T	A	.	PASS	ECNT=1;HCNT=1;MAX_ED=.;MIN_ED=.;NLOD=12.24;TLOD=8.82	GT:AD:AF:ALT_F1R2:ALT_F2R1:FOXOG:QSS:REF_F1R2:REF_F2R1	0/1:61,5:0.079:3:2:0.600:1694,140:25:36	0/0:45,0:0.00:0:0:.:1261,0:23:22	0.5	Unkown	.	.	.	.	.	.	.	.

T2df	chr19	**1**	**12.5**	4324523	4324523	T	C	exonic	**Kdm2a**	ENSMUSP00000029542	.	nonsynonymous SNV	Kdm2a:NM_001001984:exon17:c.A2579G:p.E860G	**E860G**	ENSMUSP00000029542,860,E,G	19,4324523,T,A	19qA	.	0.25	.	31	chr19	4324523	.	T	A	.	t_lod_fstar	ECNT=1;HCNT=1;MAX_ED=.;MIN_ED=.;NLOD=18.60;TLOD=4.89	GT:AD:AF:ALT_F1R2:ALT_F2R1:FOXOG:QSS:REF_F1R2:REF_F2R1	0/1:50,3:0.059:2:1:0.667:1404,84:21:29	0/0:69,0:0.00:0:0:.:1918,0:33:36	0.7	Tolerated	0.02	Neutral	140	30	0.219	Tolerated	3.43	60
T2df	chr19	**1**	**12.5**	4324519	4324519	-	T	exonic	**Kdm2a**	ENSMUSP00000029542	.	frameshift insertion	Kdm2a:NM_001001984:exon17:c.2582dupA:p.E861fs	**E861fs**	ENSMUSP00000029542,861,E,	19,4324519,C,C	19qA	.	0.25	.	29	chr19	4324519	.	C	C	.	t_lod_fstar	ECNT=1;HCNT=2;MAX_ED=.;MIN_ED=.;NLOD=11.13;TLOD=4.47	GT:AD:AF:ALT_F1R2:ALT_F2R1:FOXOG:QSS:REF_F1R2:REF_F2R1	0/1:22,3:0.130:0:3:1.00:636,69:9:13	0/0:43,0:0.00:0:0:.:1205,0:19:24	0.2	Tolerated	0	Neutral	140	30	1	Tolerated	3.43	60
T2df	chr18	**1**	**12.5**	34823463	34823463	G	A	exonic	**Kdm3b**	ENSMUSP00000071422	.	nonsynonymous SNV	Kdm3b:NM_001081256:exon14:c.G3470A:p.G1157E	**G1157E**	ENSMUSP00000071422,1157,G,E	18,34823463,G,A	18qB1	.	0.25	.	69	chr18	34823463	.	G	A	.	t_lod_fstar	ECNT=1;HCNT=1;MAX_ED=.;MIN_ED=.;NLOD=13.24;TLOD=4.79	GT:AD:AF:ALT_F1R2:ALT_F2R1:FOXOG:QSS:REF_F1R2:REF_F2R1	0/1:95,2:0.021:2:0:.:2601,57:52:43	0/0:47,0:0.00:0:0:.:1265,0:20:27	0.7	Damaging	-0.12	Neutral	190	30	0.034	Damaging	3.15	171
T4df	chr9	**1**	**12.5**	110490030	110490030	A	C	exonic	**Kif9**	ENSMUSP00000055073	.	nonsynonymous SNV	Kif9:NM_001163569:exon5:c.A561C:p.Q187H,Kif9:NM_010628:exon5:c.A561C:p.Q187H	**Q187H**	ENSMUSP00000055073,187,Q,H	9,110490030,A,G	9qF2	.	0.25	.	43	chr9	110490030	.	A	G	.	clustered_events	DB;ECNT=2;HCNT=2;MAX_ED=32;MIN_ED=32;NLOD=10.23;TLOD=24.06	GT:AD:AF:ALT_F1R2:ALT_F2R1:FOXOG:QSS:REF_F1R2:REF_F2R1	0/1:13,9:0.409:3:6:0.667:353,262:8:5	0/0:38,0:0.00:0:0:.:1053,0:20:18	2.4	Tolerated	0	Neutral	91	30	0.822	Tolerated	2.95	70
T2df	chr4	**1**	**12.5**	156234013	156234056	TCGGGGCTGCAGGCCGGGCCTGCCGGGGCCGAGCTCGCTCTGCC	-	exonic	**Klhl17**	ENSMUSP00000029744	.	frameshift deletion	Klhl17:NM_198305:exon2:c.108_151del:p.E36fs	**E36fs**	ENSMUSP00000029744,36,E,.	4,156234013,ATCGGGGCTGCAGGCCGGGCCTGCCGGGGCCGAGCTCGCTCTGCC,C	4qE2	.	0.25	.	47	chr4	156234012	.	ATCGGGGCTGCAGGCCGGGCCTGCCGGGGCCGAGCTCGCTCTGCC	C	.	clustered_events	DB;ECNT=2;HCNT=2;MAX_ED=32;MIN_ED=32;NLOD=11.69;TLOD=22.53	GT:AD:AF:ALT_F1R2:ALT_F2R1:FOXOG:QSS:REF_F1R2:REF_F2R1	0/1:13,9:0.409:5:4:0.444:369,247:9:4	0/0:43,0:0.00:0:0:.:1206,0:23:20	2.4	Unknown	NA	NA	273	30	NA	NA	NA	NA
T1df	chr5	**1**	**12.5**	25315775	25315777	TGC	-	exonic	**Kmt2c**	ENSMUSP00000047683	.	nonframeshift deletion	Kmt2c:NM_001081383:exon36:c.5334_5336del:p.1778_1779del	**1778_1779del**	NA	5,25315775,TTGC,A	5qA3	.	0.25	.	34	chr5	25315774	.	TTGC	A	.	t_lod_fstar	ECNT=1;HCNT=1;MAX_ED=.;MIN_ED=.;NLOD=12.24;TLOD=4.04	GT:AD:AF:ALT_F1R2:ALT_F2R1:FOXOG:QSS:REF_F1R2:REF_F2R1	0/1:17,2:0.125:0:2:1.00:483,57:12:5	0/0:48,0:0.00:0:0:.:1315,0:23:25	0.9	Unknown	.	.	.	.	.	.	.	.
T1df	chr15	**1**	**12.5**	98845669	98845671	TGC	-	exonic	**Kmt2d**	ENSMUSP00000135418	.	nonframeshift deletion	Kmt2d:NM_001033276:exon40:c.11607_11609del:p.3869_3870del	**3869_3870del**	NA	15,98845669,TTGC,C	15qF1	.	0.25	.	36	chr15	98845668	.	TTGC	C	.	PASS	ECNT=1;HCNT=1;MAX_ED=.;MIN_ED=.;NLOD=9.32;TLOD=7.32	GT:AD:AF:ALT_F1R2:ALT_F2R1:FOXOG:QSS:REF_F1R2:REF_F2R1	0/1:49,6:0.096:4:2:0.333:1348,152:24:25	0/0:36,0:0.00:0:0:.:1000,0:16:20	1.2	Unknown	.	.	.	.	.	.	.	.
T42df	chr15	**1**	**12.5**	101850379	101850408	AGCTGCCACCCCCAAAGCCACCACCGCCGT	-	exonic	**Krt1**	ENSMUSP00000148517	.	nonframeshift deletion	Krt1:NM_008473:exon1:c.320_349del:p.107_117del	**107_117del**	NA	15,101850379,AAGCTGCCACCCCCAAAGCCACCACCGCCGT,A	15qF2	.	0.25	.	15	chr15	101850378	.	AAGCTGCCACCCCCAAAGCCACCACCGCCGT	A	.	t_lod_fstar	ECNT=2;HCNT=1;MAX_ED=1;MIN_ED=1;NLOD=20.17;TLOD=5.29	GT:AD:AF:ALT_F1R2:ALT_F2R1:FOXOG:PGT:PID:QSS:REF_F1R2:REF_F2R1	0/1:55,2:0.037:2:0:0.00:0|1:121559024_C_A:1519,56:30:25	0/0:71,0:0.00:0:0:.:0|1:121559024_C_A:1970,0:33:38	2	Unknown	.	.	.	.	.	.	.	.
T1df	chr11	**1**	**12.5**	99329753	99329753	C	A	exonic	**Krt26**	ENSMUSP00000004470	.	nonsynonymous SNV	Krt26:NM_001033397:exon8:c.G1270T:p.A424S	**A424S**	ENSMUSP00000004470,424,A,S	11,99329753,C,A	11qD	.	0.25	.	49	chr11	99329753	.	C	A	.	alt_allele_in_normal;t_lod_fstar	ECNT=2;HCNT=2;MAX_ED=2;MIN_ED=2;NLOD=2.44;TLOD=5.41	GT:AD:AF:ALT_F1R2:ALT_F2R1:FOXOG:PGT:PID:QSS:REF_F1R2:REF_F2R1	0/1:46,2:0.043:1:1:0.500:0|1:78346447_T_C:1219,53:24:22	0/0:34,1:0.053:0:1:0.00:0|1:78346447_T_C:847,29:17:17	0.7	Damaging	-0.92	Neutral	420	30	0.001	Damaging	3.12	206
T22df	chr15	**1**	**12.5**	101947891	101947891	T	C	exonic	**Krt78**	ENSP00000306261	.	nonsynonymous SNV	Krt78:NM_212487:exon9:c.A1484G:p.E495G	**E495G**	ENSP00000306261,495,E,G	15,101947891,T,C	15qF2	.	0.25	.	27	chr15	101947891	.	T	C	.	t_lod_fstar	ECNT=1;HCNT=1;MAX_ED=.;MIN_ED=.;NLOD=3.74;TLOD=5.03	GT:AD:AF:ALT_F1R2:ALT_F2R1:FOXOG:QSS:REF_F1R2:REF_F2R1	0/1:48,5:0.100:3:2:0.600:1139,104:25:23	0/0:24,3:0.125:3:0:1.00:575,20:12:12	0.5	Damaging	0.79	Neutral	413	30	0.038	Damaging	4.32	6
T12df	chr15	**1**	**12.5**	101947898	101947898	T	C	exonic	**Krt78**	ENSP00000306261	.	nonsynonymous SNV	Krt78:NM_212487:exon9:c.A1477G:p.R493G	**R493G**	ENSP00000306261,493,R,G	15,101947898,T,C	15qF2	.	0.25	.	27	chr15	101947898	.	T	C	.	alt_allele_in_normal;germline_risk	ECNT=1;HCNT=1;MAX_ED=.;MIN_ED=.;NLOD=3.02;TLOD=7.43	GT:AD:AF:ALT_F1R2:ALT_F2R1:FOXOG:QSS:REF_F1R2:REF_F2R1	0/1:61,11:0.127:8:3:0.727:1537,182:24:37	0/0:25,2:0.083:0:2:0.00:568,38:14:11	0.5	Tolerated	1.16	Neutral	413	30	1	Tolerated	4.32	5

T12df	chr15	**1**	**12.5**	101527868	101527868	T	C	exonic	**Krt84**	ENSMUSP00000151748	.	nonsynonymous SNV	Krt84:NM_008474:exon7:c.A1285G:p.N429D	**N429D**	ENSMUSP00000151748,429,N,D	15,101527868,T,C	15qF2	.	0.25	.	40	chr15	101527868	.	T	C	.	alt_allele_in_normal;clustered_events	ECNT=7;HCNT=2;MAX_ED=25;MIN_ED=6;NLOD=2.44;TLOD=11.00	GT:AD:AF:ALT_F1R2:ALT_F2R1:FOXOG:PGT:PID:QSS:REF_F1R2:REF_F2R1	0/1:83,4:0.047:4:0:1.00:0|1:101527856_ACTTG_A:2315,103:45:38	0/0:38,2:0.053:1:1:0.500:0|1:101527856_ACTTG_A:1025,29:22:16	0.9	Damaging	-1.65	Neutral	450	30	0.014	Damaging	3.05	395
T12df	chr15	**1**	**12.5**	101527867	101527867	T	G	exonic	**Krt84**	ENSMUSP00000151748	.	nonsynonymous SNV	Krt84:NM_008474:exon7:c.A1286C:p.N429T	**N429T**	ENSMUSP00000151748,429,N,T	15,101527867,T,G	15qF2	.	0.25	.	38	chr15	101527867	.	T	G	.	alt_allele_in_normal;clustered_events;germline_risk	ECNT=7;HCNT=2;MAX_ED=25;MIN_ED=6;NLOD=2.14;TLOD=11.00	GT:AD:AF:ALT_F1R2:ALT_F2R1:FOXOG:PGT:PID:QSS:REF_F1R2:REF_F2R1	0/1:84,4:0.047:4:0:1.00:0|1:101527856_ACTTG_A:2342,119:45:39	0/0:37,1:0.054:0:1:0.00:0|1:101527856_ACTTG_A:970,29:21:16	0.9	Tolerated	-1.11	Neutral	450	30	0.24	Tolerated	3.05	395
T12df	chr15	**1**	**12.5**	101527862	101527862	-	GGGT	exonic	**Krt84**	ENSMUSP00000151748	.	frameshift insertion	Krt84:NM_008474:exon7:c.1290_1291insACCC:p.A431fs	**A431fs**	ENSMUSP00000151748,431,A,	15,101527862,C,C	15qF2	.	0.25	.	36	chr15	101527862	.	C	C	.	clustered_events;multi_event_alt_allele_in_normal	ECNT=9;HCNT=2;MAX_ED=24;MIN_ED=1;NLOD=3.84;TLOD=8.57	GT:AD:AF:ALT_F1R2:ALT_F2R1:FOXOG:PGT:PID:QSS:REF_F1R2:REF_F2R1	0/1:47,3:0.060:3:0:0.00:0|1:27107648_C_CTTTT:1220,41:21:26	0/0:30,1:0.036:1:0:0.00:0|1:27107648_C_CTTTT:791,17:19:11	1.7	Tolerated	0	Neutral	450	30	1	Tolerated	3.05	395
T12df	chr15	**1**	**12.5**	101527857	101527860	CTTG	-	exonic	**Krt84**	ENSMUSP00000151748	.	frameshift deletion	Krt84:NM_008474:exon7:c.1293_1296del:p.A431fs	**A431fs**	NA	15,101527856,ACTTG,A	15qF2	.	0.25	.	36	chr15	101527856	.	ACTTG	A	.	clustered_events;multi_event_alt_allele_in_normal;t_lod_fstar	ECNT=7;HCNT=2;MAX_ED=25;MIN_ED=6;NLOD=5.73;TLOD=4.93	GT:AD:AF:ALT_F1R2:ALT_F2R1:FOXOG:PGT:PID:QSS:REF_F1R2:REF_F2R1	0/1:82,1:0.025:1:0:.:0|1:101527856_ACTTG_A:2343,31:44:38	0/0:35,1:0.029:0:1:.:0|1:101527856_ACTTG_A:961,30:19:16	0.9	Unknown	NA	NA	450	30	NA	NA	NA	NA
T32df	chrX	**1**	**12.5**	38442069	38442069	C	A	exonic	**Lamp2**	ENSMUSP00000037628	.	stopgain	Lamp2:NM_001017959:exon2:c.G127T:p.E43X,Lamp2:NM_001290485:exon2:c.G127T:p.E43X,Lamp2:NM_010685:exon2:c.G127T:p.E43X	**E43X**	ENSMUSP00000037628,43,E,X	NA	XqA3.3	.	0.25	.	48	chrX	38442069	.	C	C	.	alt_allele_in_normal;clustered_events;germline_risk	ECNT=4;HCNT=12;MAX_ED=31;MIN_ED=10;NLOD=1.23;TLOD=13.15	GT:AD:AF:ALT_F1R2:ALT_F2R1:FOXOG:PGT:PID:QSS:REF_F1R2:REF_F2R1	0/1:22,3:0.154:1:2:0.333:0|1:3908692_T_C:621,84:10:12	0/0:34,2:0.059:1:1:0.500:0|1:3908692_T_C:961,57:18:16	1.9	Deletarious	.	.	.	.	.	.	.	.
T42df	chr11	**1**	**12.5**	34082442	34082442	A	C	exonic	**Lcp2**	ENSMUSP00000057896	.	nonsynonymous SNV	Lcp2:NM_010696:exon16:c.A1019C:p.N340T	**N340T**	ENSMUSP00000057896,340,N,T	11,34082442,A,A	11qA4	.	0.25	.	36	chr11	34082442	.	A	A	.	alt_allele_in_normal;clustered_events;germline_risk	ECNT=4;HCNT=12;MAX_ED=31;MIN_ED=10;NLOD=1.54;TLOD=13.15	GT:AD:AF:ALT_F1R2:ALT_F2R1:FOXOG:PGT:PID:QSS:REF_F1R2:REF_F2R1	0/1:22,4:0.154:1:3:0.250:0|1:3908692_T_C:595,113:10:12	0/0:34,2:0.057:1:1:0.500:0|1:3908692_T_C:910,58:18:16	1.9	Tolerated	0	Neutral	133	30	1	Tolerated	2.87	58
T4df	chr18	**1**	**12.5**	56749741	56749743	GAG	-	exonic	**Lmnb1**	ENSMUSP00000055002	.	nonframeshift deletion	Lmnb1:NM_010721:exon10:c.1657_1659del:p.553_553del	**553_553del**	NA	18,56749741,CGAG,T	18qD3	rs233529689	0.25	.	76	chr18	56749740	.	CGAG	T	.	str_contraction;t_lod_fstar	ECNT=1;HCNT=1;MAX_ED=.;MIN_ED=.;NLOD=5.71;RPA=4,3;RU=TCC;STR;TLOD=5.31	GT:AD:AF:ALT_F1R2:ALT_F2R1:FOXOG:QSS:REF_F1R2:REF_F2R1	0/1:51,2:0.038:1:1:.:1428,56:23:28	0/0:23,0:0.00:0:0:.:625,0:14:9	14	Unknown	.	.	.	.	.	.	.	.
T1df	chr3	**1**	**12.5**	92081879	92081881	GCC	-	exonic	**Lor**	ENSMUSP00000033465	.	nonframeshift deletion	Lor:NM_008508:exon2:c.97_99del:p.33_33del	**33_33del**	NA	3,92081879,AGCC,C	3qF1	.	0.25	.	21	chr3	92081878	.	AGCC	C	.	alt_allele_in_normal;clustered_events;t_lod_fstar	ECNT=6;HCNT=1;MAX_ED=30;MIN_ED=3;NLOD=4.53;TLOD=5.21	GT:AD:AF:ALT_F1R2:ALT_F2R1:FOXOG:PGT:PID:QSS:REF_F1R2:REF_F2R1	0/1:58,2:0.034:0:2:.:0|1:79755256_G_A:1429,59:30:28	0/0:31,1:0.033:0:1:.:0|1:79755256_G_A:764,28:18:13	0.7	Unknown	.	.	.	.	.	.	.	.
T42df	chr8	**1**	**12.5**	69824236	69824236	G	A	exonic	**Lpar2**	ENSMUSP00000101194	.	nonsynonymous SNV	Lpar2:NM_020028:exon2:c.G674A:p.C225Y	**C225Y**	ENSMUSP00000101194,225,C,Y	8,69824236,G,G	8qB3.3	.	0.25	.	35	chr8	69824236	.	G	G	.	PASS	DB;ECNT=1;HCNT=1;MAX_ED=.;MIN_ED=.;NLOD=11.69;TLOD=91.80	GT:AD:AF:ALT_F1R2:ALT_F2R1:FOXOG:QSS:REF_F1R2:REF_F2R1	0/1:31,33:0.524:20:13:0.606:871,950:19:12	0/0:45,0:0.00:0:0:.:1258,0:26:19	4	Tolerated	0	Neutral	447	30	0.343	Tolerated	2.94	313
T1df	chr14	**1**	**12.5**	37072315	37072315	T	G	exonic	**Lrit2**	ENSMUSP00000110393	.	nonsynonymous SNV	Lrit2:NM_173418:exon3:c.T1335G:p.C445W	**C445W**	ENSMUSP00000110393,445,C,W	14,37072315,T,T	14qB	.	0.25	.	59	chr14	37072315	.	T	T	.	clustered_events;homologous_mapping_event	ECNT=3;HCNT=2;MAX_ED=3;MIN_ED=1;NLOD=7.87;TLOD=10.16	GT:AD:AF:ALT_F1R2:ALT_F2R1:FOXOG:PGT:PID:QSS:REF_F1R2:REF_F2R1	0/1:49,4:0.075:1:3:0.250:0|1:98003232_G_T:1404,110:25:24	0/0:27,0:0.00:0:0:.:0|1:98003232_G_T:768,0:14:13	9	Tolerated	0	Neutral	123	30	1	Tolerated	2.82	61

T1df	chr8	**1**	**12.5**	105461133	105461133	G	T	splicing	**Lrrc36**	ENSMUSP00000322270	.	NM_001170788:exon13:c.2048+1G>T;NM_001033371:exon10:c.1679+1G>T;NM_001170789:exon10:c.1685+1G>T	.	**NA**	8,105461133,G,G	NA	8qD3	.	0.25	.	45	chr8	105461133	.	G	G	.	str_contraction;t_lod_fstar	ECNT=1;HCNT=2;MAX_ED=.;MIN_ED=.;NLOD=10.22;RPA=6,5;RU=GT;STR;TLOD=5.15	GT:AD:AF:ALT_F1R2:ALT_F2R1:FOXOG:QSS:REF_F1R2:REF_F2R1	0/1:61,3:0.050:2:1:.:1696,84:35:26	0/0:37,0:0.00:0:0:.:1050,0:15:22	0.4	Unkown	.	.	.	.	.	.	.	.
T3df	chr4	**1**	**12.5**	148144728	148144729	GT	-	splicing	**Mad2l2**	ENSMUSP00000071147	.	.	.	**NA**	4,148144728,GGT,AGCTCCAGCTCCAGCTCCAGCTCCAGCTCCAGCTCCAGCTCCAGCTCC	NA	4qE2	.	0.25	.	37	chr4	148144727	.	GGT	AGCTCCAGCTCCAGCTCCAGCTCCAGCTCCAGCTCCAGCTCCAGCTCC	.	t_lod_fstar	ECNT=1;HCNT=2;MAX_ED=.;MIN_ED=.;NLOD=2.51;TLOD=4.42	GT:AD:AF:ALT_F1R2:ALT_F2R1:FOXOG:QSS:REF_F1R2:REF_F2R1	0/1:17,10:0.267:4:6:.:474,287:7:10	0/0:12,0:0.00:0:0:.:284,0:6:6	1	Unkown	.	.	.	.	.	.	.	.
T32df	chrX	**1**	**12.5**	92120382	92120382	C	G	exonic	**Mageb18**	ENSMUSP00000129830	.	nonsynonymous SNV	Mageb18:NM_173783:exon5:c.G253C:p.D85H	**D85H**	ENSMUSP00000129830,85,D,H	X,92120382,C,AGCTCCAGCTCCAGCTCCAGCTCCAGCTCCAGCTCCAGCTCCAGCTCC	XqC2	.	0.25	.	41	chrX	92120382	.	C	AGCTCCAGCTCCAGCTCCAGCTCCAGCTCCAGCTCCAGCTCCAGCTCC	.	t_lod_fstar	ECNT=1;HCNT=2;MAX_ED=.;MIN_ED=.;NLOD=2.51;TLOD=4.42	GT:AD:AF:ALT_F1R2:ALT_F2R1:FOXOG:QSS:REF_F1R2:REF_F2R1	0/1:17,10:0.267:4:6:.:474,287:7:10	0/0:12,0:0.00:0:0:.:284,0:6:6	1	Unknown	NA	NA	68	30	NA	NA	NA	NA
T22df	chr2	**1**	**12.5**	121306318	121306318	-	GCTCCAGCTCCAGCTCCAGCTCCAGCTCCAGCTCCAGCTCCAGCTCC	exonic	**Map1a**	ENSMUSP00000322252	.	frameshift insertion	Map1a:NM_001173506:exon4:c.6900_6901insGCTCCAGCTCCAGCTCCAGCTCCAGCTCCAGCTCCAGCTCCAGCTCC:p.P2300fs,Map1a:NM_032393:exon5:c.7614_7615insGCTCCAGCTCCAGCTCCAGCTCCAGCTCCAGCTCCAGCTCCAGCTCC:p.P2538fs	**P2300fs**	ENSMUSP00000322252,2300,P,	2,121306318,A,A	2qE5	.	0.25	.	12	chr2	121306318	.	A	A	.	t_lod_fstar	ECNT=1;HCNT=1;MAX_ED=.;MIN_ED=.;NLOD=7.14;TLOD=5.80	GT:AD:AF:ALT_F1R2:ALT_F2R1:FOXOG:QSS:REF_F1R2:REF_F2R1	0/1:32,3:0.063:3:0:.:891,87:18:14	0/0:26,0:0.00:0:0:.:710,0:19:7	1	Tolerated	0	Neutral	117	30	1	Tolerated	3.75	45
T22df	chr2	**1**	**12.5**	121306318	121306318	-	GCTCCAGCTCCAGCTCCAGCTCCAGCTCCAGCTCCAGCTCCAGCTCC	exonic	**Map1a**	ENSMUSP00000322252	.	frameshift insertion	Map1a:NM_001173506:exon4:c.6900_6901insGCTCCAGCTCCAGCTCCAGCTCCAGCTCCAGCTCCAGCTCCAGCTCC:p.P2300fs,Map1a:NM_032393:exon5:c.7614_7615insGCTCCAGCTCCAGCTCCAGCTCCAGCTCCAGCTCCAGCTCCAGCTCC:p.P2538fs	**P2538fs**	ENSMUSP00000322252,2538,P,	2,121306318,A,C	2qE5	.	0.25	.	12	chr2	121306318	.	A	C	.	alt_allele_in_normal	ECNT=1;HCNT=1;MAX_ED=.;MIN_ED=.;NLOD=13.27;TLOD=6.72	GT:AD:AF:ALT_F1R2:ALT_F2R1:FOXOG:QSS:REF_F1R2:REF_F2R1	0/1:26,4:0.143:1:3:0.250:702,99:14:12	0/0:65,2:0.031:1:1:0.500:1803,53:39:26	0.9	Tolerated	0	Neutral	117	30	1	Tolerated	3.75	45
T42df	chrX	**1**	**12.5**	159414707	159414748	GCGGTGGGAACGGCGGCGGCGGCGGTGGCGGCGGCGGCGGCG	-	exonic	**Map7d2**	ENSMUSP00000043874	.	nonframeshift deletion	Map7d2:NM_001081124:exon1:c.11_52del:p.4_18del,Map7d2:NM_001313751:exon1:c.11_52del:p.4_18del	**4_18del**	NA	X,159414707,AGCGGTGGGAACGGCGGCGGCGGCGGTGGCGGCGGCGGCGGCG,ACACTTCTCCTGCAGAGAGCAAGAGGGGTGAGAGCTGGCTCCAGCTCT	XqF4	.	0.25	.	26	chrX	159414706	.	AGCGGTGGGAACGGCGGCGGCGGCGGTGGCGGCGGCGGCGGCG	ACACTTCTCCTGCAGAGAGCAAGAGGGGTGAGAGCTGGCTCCAGCTCT	.	t_lod_fstar	ECNT=1;HCNT=1;MAX_ED=.;MIN_ED=.;NLOD=8.40;TLOD=5.78	GT:AD:AF:ALT_F1R2:ALT_F2R1:FOXOG:QSS:REF_F1R2:REF_F2R1	0/1:27,1:0.069:0:1:.:761,27:14:13	0/0:26,0:0.00:0:0:.:719,0:16:10	0.9	Unknown	NA	NA	153	30	NA	NA	NA	NA
T42df	chr14	**1**	**12.5**	47319411	47319411	G	C	splicing	**Mapk1ip1l**	ENSMUSP00000023741	.	NM_178684:exon4:c.718-1G>C	.	**NA**	14,47319411,G,G	NA	14qC1	.	0.25	.	67	chr14	47319411	.	G	G	.	t_lod_fstar	ECNT=1;HCNT=1;MAX_ED=.;MIN_ED=.;NLOD=20.66;TLOD=4.49	GT:AD:AF:ALT_F1R2:ALT_F2R1:FOXOG:QSS:REF_F1R2:REF_F2R1	0/1:16,3:0.176:3:0:0.00:446,78:7:9	0/0:77,0:0.00:0:0:.:2147,0:36:41	0.4	Unkown	.	.	.	.	.	.	.	.
T22df	chr7	**1**	**12.5**	19447243	19447243	-	CACTTCTCCTGCAGAGAGCAAGAGGGGTGAGAGCTGGCTCCAGCTCT	exonic	**Mark4**	ENSMUSP00000142312	.	frameshift insertion	Mark4:NM_172279:exon6:c.429_430insAGAGCTGGAGCCAGCTCTCACCCCTCTTGCTCTCTGCAGGAGAAGTG:p.F144fs	**F144fs**	ENSMUSP00000142312,144,F,	7,19447243,A,A	7qA3	.	0.25	.	26	chr7	19447243	.	A	A	.	alt_allele_in_normal;clustered_events;t_lod_fstar	ECNT=2;HCNT=4;MAX_ED=47;MIN_ED=47;NLOD=13.93;TLOD=4.55	GT:AD:AF:ALT_F1R2:ALT_F2R1:FOXOG:QSS:REF_F1R2:REF_F2R1	0/1:20,3:0.143:0:3:0.00:552,69:11:9	0/0:70,2:0.031:0:2:0.00:1954,48:31:39	0.4	Tolerated	0	Neutral	308	30	1	Tolerated	3.11	392

T3df	chr18	**1**	**12.5**	35583923	35583923	T	A	splicing	**Matr3**	ENSMUSP00000016678	.	NM_010771:exon11:c.1778+2T>A	.	**NA**	18,35583923,T,G	NA	18qB2	.	0.25	.	72	chr18	35583923	.	T	G	.	alt_allele_in_normal;t_lod_fstar	ECNT=1;HCNT=1;MAX_ED=.;MIN_ED=.;NLOD=9.96;TLOD=5.86	GT:AD:AF:ALT_F1R2:ALT_F2R1:FOXOG:QSS:REF_F1R2:REF_F2R1	0/1:83,6:0.114:3:3:.:2357,164:37:46	0/0:64,2:0.032:1:1:.:1759,58:33:31	1.6	Unkown	.	.	.	.	.	.	.	.
T12df	chr5	**1**	**12.5**	138167438	138167548	ACCTGCAAAGATAAAGAGCAAACTGCTGAAGAAAGGAACCGCTTTCCCCAGGCCTTCCAGAGGCCTTAGTGTTTATCAACCAAACACCAAGCCTCATCCCTGTTCACACTC	-	exonic	**Mcm7**	ENSMUSP00000052584	.	frameshift deletion	Mcm7:NM_001356576:exon11:c.881_882del:p.S294fs,Mcm7:NM_001356577:exon11:c.881_882del:p.S294fs,Mcm7:NM_008568:exon11:c.1202_1203del:p.S401fs	**S294fs**	ENSMUSP00000052584,294,S,.	5,138167438,GACCTGCAAAGATAAAGAGCAAACTGCTGAAGAAAGGAACCGCTTTCCCCAGGCCTTCCAGAGGCCTTAGTGTTTATCAACCAAACACCAAGCCTCATCCCTGTTCACACTC,C	5qG2	.	0.25	.	66	chr5	138167437	.	GACCTGCAAAGATAAAGAGCAAACTGCTGAAGAAAGGAACCGCTTTCCCCAGGCCTTCCAGAGGCCTTAGTGTTTATCAACCAAACACCAAGCCTCATCCCTGTTCACACTC	C	.	PASS	ECNT=1;HCNT=1;MAX_ED=.;MIN_ED=.;NLOD=8.42;TLOD=14.60	GT:AD:AF:ALT_F1R2:ALT_F2R1:FOXOG:QSS:REF_F1R2:REF_F2R1	0/1:43,7:0.143:5:2:0.714:127,200:22:21	0/0:31,0:0.00:0:0:.:877,0:15:16	0.7	Unkown	.	.	.	.	.	.	.	.
T12df	chr5	**1**	**12.5**	138167438	138167548	ACCTGCAAAGATAAAGAGCAAACTGCTGAAGAAAGGAACCGCTTTCCCCAGGCCTTCCAGAGGCCTTAGTGTTTATCAACCAAACACCAAGCCTCATCCCTGTTCACACTC	-	exonic	**Mcm7**	ENSMUSP00000052584	.	frameshift deletion	Mcm7:NM_001356576:exon11:c.881_882del:p.S294fs,Mcm7:NM_001356577:exon11:c.881_882del:p.S294fs,Mcm7:NM_008568:exon11:c.1202_1203del:p.S401fs	**S401fs**	ENSMUSP00000052584,401,S,.	5,138167438,GACCTGCAAAGATAAAGAGCAAACTGCTGAAGAAAGGAACCGCTTTCCCCAGGCCTTCCAGAGGCCTTAGTGTTTATCAACCAAACACCAAGCCTCATCCCTGTTCACACTC,C	5qG2	.	0.25	.	66	chr5	138167437	.	GACCTGCAAAGATAAAGAGCAAACTGCTGAAGAAAGGAACCGCTTTCCCCAGGCCTTCCAGAGGCCTTAGTGTTTATCAACCAAACACCAAGCCTCATCCCTGTTCACACTC	C	.	PASS	ECNT=1;HCNT=1;MAX_ED=.;MIN_ED=.;NLOD=8.42;TLOD=14.60	GT:AD:AF:ALT_F1R2:ALT_F2R1:FOXOG:QSS:REF_F1R2:REF_F2R1	0/1:43,7:0.143:5:2:0.714:127,200:22:21	0/0:31,0:0.00:0:0:.:877,0:15:16	0.7	Unkown	.	.	.	.	.	.	.	.
T4df	chr4	**1**	**12.5**	155656269	155656269	G	A	exonic	**Mib2**	ENSMUSP00000081146	.	stopgain	Mib2:NM_145124:exon15:c.C1897T:p.Q633X,Mib2:NM_001256107:exon16:c.C1993T:p.Q665X,Mib2:NM_001256108:exon16:c.C1897T:p.Q633X	**Q633X**	ENSMUSP00000081146,633,Q,X	NA	4qE2	.	0.25	.	33	chr4	155656269	.	G	A	.	str_contraction	ECNT=1;HCNT=1;MAX_ED=.;MIN_ED=.;NLOD=6.00;RPA=7,6;RU=AGC;STR;TLOD=7.18	GT:AD:AF:ALT_F1R2:ALT_F2R1:FOXOG:QSS:REF_F1R2:REF_F2R1	0/1:74,3:0.039:1:2:.:2069,85:37:37	0/0:23,0:0.00:0:0:.:618,0:10:13	0.2	Deletarious	.	.	.	.	.	.	.	.
T4df	chr4	**1**	**12.5**	155656269	155656269	G	A	exonic	**Mib2**	ENSMUSP00000081146	.	stopgain	Mib2:NM_145124:exon15:c.C1897T:p.Q633X,Mib2:NM_001256107:exon16:c.C1993T:p.Q665X,Mib2:NM_001256108:exon16:c.C1897T:p.Q633X	**Q665X**	ENSMUSP00000081146,665,Q,X	NA	4qE2	.	0.25	.	33	chr4	155656269	.	G	ACAGAGTAGGACTTTAGG	.	t_lod_fstar	ECNT=1;HCNT=1;MAX_ED=.;MIN_ED=.;NLOD=8.13;TLOD=5.12	GT:AD:AF:ALT_F1R2:ALT_F2R1:FOXOG:QSS:REF_F1R2:REF_F2R1	0/1:63,2:0.031:0:2:.:1775,54:33:30	0/0:30,0:0.00:0:0:.:834,0:10:20	1.4	Deletarious	.	.	.	.	.	.	.	.
T1df	chr11	**1**	**12.5**	70607041	70607043	AGC	-	exonic	**Mink1**	ENSMUSP00000046693	.	nonframeshift deletion	Mink1:NM_001045959:exon14:c.1469_1471del:p.490_491del,Mink1:NM_001045964:exon14:c.1469_1471del:p.490_491del,Mink1:NM_016713:exon14:c.1469_1471del:p.490_491del,Mink1:NM_176893:exon14:c.1469_1471del:p.490_491del	**490_491del**	NA	11,70607041,AAGC,TTCTCC	11qB3	.	0.25	.	23	chr11	70607040	.	AAGC	TTCTCC	.	alt_allele_in_normal;clustered_events;germline_risk	ECNT=2;HCNT=1;MAX_ED=3;MIN_ED=3;NLOD=1.85;TLOD=8.78	GT:AD:AF:ALT_F1R2:ALT_F2R1:FOXOG:PGT:PID:QSS:REF_F1R2:REF_F2R1	0/1:43,3:0.070:3:0:.:0|1:17695819_A_AG:1221,83:17:26	0/0:38,2:0.056:1:1:.:0|1:17695819_A_AG:1034,54:19:19	2.2	Unknown	NA	NA	297	30	NA	NA	NA	NA
T4df	chr1	**1**	**12.5**	74900717	74900736	CGGGGCCGGAGCGGAAGACC	-	ncRNA_exonic	**Mir375**	NONE	.	.	.	**NA**	1,74900717,GCGGGGCCGGAGCGGAAGACC,T	NA	1qC4	.	0.25	.	35	chr1	74900716	.	GCGGGGCCGGAGCGGAAGACC	T	.	alt_allele_in_normal;clustered_events;t_lod_fstar	ECNT=2;HCNT=3;MAX_ED=6;MIN_ED=6;NLOD=16.28;TLOD=5.37	GT:AD:AF:ALT_F1R2:ALT_F2R1:FOXOG:PGT:PID:QSS:REF_F1R2:REF_F2R1	0/1:47,2:0.041:0:2:1.00:0|1:59731690_C_T:1317,58:19:28	0/0:120,2:0.017:1:1:0.500:0|1:59731690_C_T:3240,57:65:55	0.5	Unkown	.	.	.	.	.	.	.	.
T42df	chr15	**1**	**12.5**	88966007	88966007	-	CAGAGTAGGACTTTAGG	exonic	**Mlc1**	ENSMUSP00000108090	.	frameshift insertion	Mlc1:NM_133241:exon7:c.546_547insCCTAAAGTCCTACTCTG:p.S183fs	**S183fs**	ENSMUSP00000108090,183,S,	15,88966007,A,AG	15qE3	.	0.25	.	30	chr15	88966007	.	A	AG	.	alt_allele_in_normal;clustered_events;germline_risk	ECNT=2;HCNT=1;MAX_ED=3;MIN_ED=3;NLOD=1.85;RPA=2,3;RU=G;STR;TLOD=8.81	GT:AD:AF:ALT_F1R2:ALT_F2R1:FOXOG:PGT:PID:QSS:REF_F1R2:REF_F2R1	0/1:41,3:0.071:3:0:.:0|1:17695819_A_AG:1177,84:16:25	0/0:38,2:0.056:1:1:.:0|1:17695819_A_AG:1043,57:19:19	2.2	Unknown	NA	NA	64	9	NA	NA	NA	NA
T22df	chr13	**1**	**12.5**	17695819	17695819	-	G	exonic	**Mplkip**	ENSMUSP00000122106	.	frameshift insertion	Mplkip:NM_025479:exon1:c.336dupG:p.Q112fs	**Q112fs**	ENSMUSP00000122106,112,Q,	13,17695819,A,A	13qA2	.	0.25	.	40	chr13	17695819	.	A	A	.	alt_allele_in_normal;germline_risk;t_lod_fstar	ECNT=1;HCNT=2;MAX_ED=.;MIN_ED=.;NLOD=0.72;TLOD=5.21	GT:AD:AF:ALT_F1R2:ALT_F2R1:FOXOG:QSS:REF_F1R2:REF_F2R1	0/1:38,3:0.075:1:2:0.333:977,84:19:19	0/0:43,5:0.093:4:1:0.800:1123,131:23:20	0.7	Tolerated	0	Neutral	33	9	1	Tolerated	3.7	27

T22df	chr13	**1**	**12.5**	17695822	17695822	-	TCTCC	splicing	**Mplkip**	ENSMUSP00000122106	.	NM_025479:exon1:c.336+2->TCTCC	.	**NA**	13,17695822,T,GGATGATGGTAAGGAAGTCAACCACATCACCACACCACTGGT	NA	13qA2	.	0.25	.	40
T32df	chr7	**1**	**12.5**	48021934	48021934	T	C	exonic	**Mrgprx1**	ENSMUSP00000114308	.	nonsynonymous SNV	Mrgprx1:NM_207540:exon2:c.A64G:p.N22D	**N22D**	ENSMUSP00000114308,22,N,D	7,48021934,T,C	7qB4	.	0.25	.	57
T32df	chr7	**1**	**12.5**	48021915	48021915	G	A	exonic	**Mrgprx1**	ENSMUSP00000114308	.	nonsynonymous SNV	Mrgprx1:NM_207540:exon2:c.C83T:p.T28I	**T28I**	ENSMUSP00000114308,28,T,I	7,48021915,G,T	7qB4	.	0.25	.	53
T32df	chr7	**1**	**12.5**	48021916	48021916	T	C	exonic	**Mrgprx1**	ENSMUSP00000114308	.	nonsynonymous SNV	Mrgprx1:NM_207540:exon2:c.A82G:p.T28A	**T28A**	ENSMUSP00000114308,28,T,A	7,48021916,T,C	7qB4	.	0.25	.	53
T32df	chr7	**1**	**12.5**	48021927	48021927	G	T	exonic	**Mrgprx1**	ENSMUSP00000114308	.	nonsynonymous SNV	Mrgprx1:NM_207540:exon2:c.C71A:p.T24N	**T24N**	ENSMUSP00000114308,24,T,N	7,48021927,G,T	7qB4	.	0.25	.	54
T32df	chr1	**1**	**12.5**	88228139	88228139	-	GATGATGGTAAGGAAGTCAACCACATCACCACACCACTGGT	exonic	**Mroh2a**	ENSMUSP00000132875	.	frameshift insertion	Mroh2a:NM_001281466:exon2:c.72_73insGATGATGGTAAGGAAGTCAACCACATCACCACACCACTGGT:p.E24fs	**E24fs**	ENSMUSP00000132875,24,E,	1,88228139,G,A	1qD	.	0.25	.	65
T1df	chr3	**1**	**12.5**	32736641	32736641	C	A	splicing	**Mrpl47**	ENSMUSP00000025178	NM_029017:exon1:c.98+1G>T	.	.	**NA**	3,32736641,C,C	NA	3qA3	.	0.25	.	41
T32df	chr19	**1**	**12.5**	11381353	11381353	C	-	exonic	**Ms4a4a**	ENSMUSP00000098502	.	frameshift deletion	Ms4a4a:NM_001310331:exon3:c.174delC:p.N58fs	**N58fs**	ENSMUSP00000098502,58,N,.	19,11381353,AC,C	19qA	.	0.25	.	42
T12df	chr2	**1**	**12.5**	90865197	90865197	G	A	exonic	**Mtch2**	ENSMUSP00000082862	.	nonsynonymous SNV	Mtch2:NM_001317243:exon12:c.G812A:p.R271Q,Mtch2:NM_001317242:exon13:c.G866A:p.R289Q,Mtch2:NM_001317244:exon13:c.G458A:p.R153Q,Mtch2:NM_019758:exon13:c.G839A:p.R280Q	**R153Q**	ENSMUSP00000082862,153,R,Q	2,90865197,G,A	2qE1	.	0.25	.	48

T22df	chr13	17695822	.	T	GGATGATGGTAAGGAAGTCAACCACATCACCACACCACTGGT	.	t_lod_fstar	ECNT=1;HCNT=1;MAX_ED=.;MIN_ED=.;NLOD=17.39;RPA=1,2;RU=GATGATGGTAAGGAAGTCAACCACATCACCACACCACTGGT;STR;TLOD=5.30	GT:AD:AF:ALT_F1R2:ALT_F2R1:FOXOG:QSS:REF_F1R2:REF_F2R1	0/1:47,1:0.041:1:0:.:1363,30:28:19	0/0:65,0:0.00:0:0:.:1845,0:22:43	0.5	Unkown	.	.	.	.	.	.	.	.
T32df	chr7	48021934	.	T	C	.	alt_allele_in_normal;clustered_events;germline_risk;t_lod_fstar	ECNT=6;HCNT=2;MAX_ED=20;MIN_ED=1;NLOD=1.25;TLOD=5.60	GT:AD:AF:ALT_F1R2:ALT_F2R1:FOXOG:PGT:PID:QSS:REF_F1R2:REF_F2R1	0/1:36,2:0.053:1:1:0.500:0|1:48021914_G_A:1019,58:15:21	0/0:55,2:0.061:0:2:0.00:0|1:48021914_G_A:1513,56:34:21	1.8	Tolerated	-0.22	Neutral	140	30	0.632	Tolerated	2.81	140
T32df	chr7	48021915	.	G	T	.	clustered_events;multi_event_alt_allele_in_normal;t_lod_fstar	ECNT=6;HCNT=2;MAX_ED=20;MIN_ED=1;NLOD=10.55;TLOD=5.60	GT:AD:AF:ALT_F1R2:ALT_F2R1:FOXOG:PGT:PID:QSS:REF_F1R2:REF_F2R1	0/1:37,2:0.053:1:1:0.500:0|1:48021914_G_A:1073,58:16:21	0/0:53,1:0.020:0:1:0.00:0|1:48021914_G_A:1495,28:34:19	1.8	Tolerated	-2.01	Neutral	140	30	0.088	Tolerated	2.82	143
T32df	chr7	48021916	.	T	C	.	alt_allele_in_normal;clustered_events;germline_risk;t_lod_fstar	ECNT=6;HCNT=2;MAX_ED=20;MIN_ED=1;NLOD=1.25;TLOD=5.60	GT:AD:AF:ALT_F1R2:ALT_F2R1:FOXOG:PGT:PID:QSS:REF_F1R2:REF_F2R1	0/1:36,2:0.053:1:1:0.500:0|1:48021914_G_A:1019,58:15:21	0/0:55,2:0.061:0:2:0.00:0|1:48021914_G_A:1513,56:34:21	1.8	Tolerated	-1.05	Neutral	140	30	0.411	Tolerated	2.82	143
T32df	chr7	48021927	.	G	T	.	clustered_events;homologous_mapping_event;t_lod_fstar	ECNT=8;HCNT=2;MAX_ED=15;MIN_ED=2;NLOD=10.23;TLOD=5.24	GT:AD:AF:ALT_F1R2:ALT_F2R1:FOXOG:PGT:PID:QSS:REF_F1R2:REF_F2R1	0/1:56,2:0.035:2:0:1.00:0|1:118057025_T_TA:1524,56:31:25	0/0:37,0:0.00:0:0:.:0|1:118057025_T_TA:974,0:28:9	6	Tolerated	-0.18	Neutral	140	30	0.496	Tolerated	2.82	143
T32df	chr1	88228139	.	G	A	.	alt_allele_in_normal;germline_risk;t_lod_fstar	ECNT=1;HCNT=2;MAX_ED=.;MIN_ED=.;NLOD=0.72;TLOD=5.21	GT:AD:AF:ALT_F1R2:ALT_F2R1:FOXOG:QSS:REF_F1R2:REF_F2R1	0/1:38,3:0.075:1:2:0.333:977,84:19:19	0/0:43,5:0.093:4:1:0.800:1123,131:23:20	0.7	Tolerated	0	Neutral	123	30	1	Tolerated	4.11	26
T1df	chr3	32736641	.	C	C	.	str_contraction;t_lod_fstar	ECNT=1;HCNT=1;MAX_ED=.;MIN_ED=.;NLOD=6.02;RPA=7,6;RU=CTG;STR;TLOD=4.73	GT:AD:AF:ALT_F1R2:ALT_F2R1:FOXOG:QSS:REF_F1R2:REF_F2R1	0/1:54,2:0.038:0:2:.:1525,56:29:25	0/0:24,0:0.00:0:0:.:672,0:13:11	2.8	Unkown	.	.	.	.	.	.	.	.
T32df	chr19	11381352	.	AC	C	.	str_contraction;t_lod_fstar	ECNT=1;HCNT=1;MAX_ED=.;MIN_ED=.;NLOD=9.61;RPA=6,5;RU=CAG;STR;TLOD=5.41	GT:AD:AF:ALT_F1R2:ALT_F2R1:FOXOG:QSS:REF_F1R2:REF_F2R1	0/1:47,2:0.043:2:0:.:1303,56:23:24	0/0:36,0:0.00:0:0:.:985,0:20:16	6	Unkown	.	.	.	.	.	.	.	.
T12df	chr2	90865197	.	G	A	.	alt_allele_in_normal;t_lod_fstar	ECNT=1;HCNT=1;MAX_ED=.;MIN_ED=.;NLOD=5.42;TLOD=4.74	GT:AD:AF:ALT_F1R2:ALT_F2R1:FOXOG:QSS:REF_F1R2:REF_F2R1	0/1:48,3:0.061:2:1:0.667:1254,83:16:32	0/0:42,2:0.050:1:1:0.500:1041,56:16:26	0.5	Deleterious	-3.35	Deleterious	160	30	0	Damaging	2.89	110

T12df	chr2	**1**	**12.5**	90865197	90865197	G	A	exonic	**Mtch2**	ENSMUSP00000082862	.	nonsynonymous SNV	Mtch2:NM_001317243:exon12:c.G812A:p.R271Q,Mtch2:NM_001317242:exon13:c.G866A:p.R289Q,Mtch2:NM_001317244:exon13:c.G458A:p.R153Q,Mtch2:NM_019758:exon13:c.G839A:p.R280Q	**R271Q**	ENSMUSP00000082862,271,R,Q	2,90865197,G,A	2qE1	.	0.25	.	48	chr2	90865197	.	G	A	.	alt_allele_in_normal;germline_risk;t_lod_fstar	ECNT=1;HCNT=2;MAX_ED=.;MIN_ED=.;NLOD=0.722;TLOD=5.21	GT:AD:AF:ALT_F1R2:ALT_F2R1:FOXOG:QSS:REF_F1R2:REF_F2R1	0/1:38,3:0.075:1:2:0.333:977,84:19:19	0/0:43,5:0.093:4:1:0.800:1123,131:23:20	0.7	Deleterious	-3.35	Deleterious	160	30	0	Damaging	2.89	110
T12df	chr2	**1**	**12.5**	90865197	90865197	G	A	exonic	**Mtch2**	ENSMUSP00000082862	.	nonsynonymous SNV	Mtch2:NM_001317243:exon12:c.G812A:p.R271Q,Mtch2:NM_001317242:exon13:c.G866A:p.R289Q,Mtch2:NM_001317244:exon13:c.G458A:p.R153Q,Mtch2:NM_019758:exon13:c.G839A:p.R280Q	**R280Q**	ENSMUSP00000082862,280,R,Q	2,90865197,G,A	2qE1	.	0.25	.	48	chr2	90865197	.	G	A	.	alt_allele_in_normal;t_lod_fstar	ECNT=1;HCNT=1;MAX_ED=.;MIN_ED=.;NLOD=5.42;TLOD=4.74	GT:AD:AF:ALT_F1R2:ALT_F2R1:FOXOG:QSS:REF_F1R2:REF_F2R1	0/1:48,3:0.061:2:1:0.667:1254,83:16:32	0/0:42,2:0.050:1:1:0.500:1041,56:16:26	0.5	Deleterious	-3.35	Deleterious	160	30	0	Damaging	2.89	110
T12df	chr2	**1**	**12.5**	90865197	90865197	G	A	exonic	**Mtch2**	ENSMUSP00000125761	.	nonsynonymous SNV	Mtch2:NM_001317243:exon12:c.G812A:p.R271Q,Mtch2:NM_001317242:exon13:c.G866A:p.R289Q,Mtch2:NM_001317244:exon13:c.G458A:p.R153Q,Mtch2:NM_019758:exon13:c.G839A:p.R280Q	**R289Q**	ENSMUSP00000125761,289,R,Q	2,90865197,G,A	2qE1	.	0.25	.	48	chr2	90865197	.	G	A	.	alt_allele_in_normal;germline_risk;t_lod_fstar	ECNT=1;HCNT=2;MAX_ED=.;MIN_ED=.;NLOD=0.722;TLOD=5.21	GT:AD:AF:ALT_F1R2:ALT_F2R1:FOXOG:QSS:REF_F1R2:REF_F2R1	0/1:38,3:0.075:1:2:0.333:977,84:19:19	0/0:43,5:0.093:4:1:0.800:1123,131:23:20	0.7	Deleterious	-3.35	Deleterious	160	30	0	Damaging	2.89	110
T2df	chr4	**1**	**12.5**	148041745	148041745	G	A	exonic	**Mthfr**	ENSP00000365669	.	nonsynonymous SNV	Mthfr:NM_001161798:exon2:c.G272A:p.R91H,Mthfr:NM_010840:exon2:c.G149A:p.R50H	**R50H**	ENSP00000365669,50,R,H	4,148041745,G,A	4qE2	.	0.25	.	44	chr4	148041745	.	G	A	.	alt_allele_in_normal;t_lod_fstar	ECNT=1;HCNT=1;MAX_ED=.;MIN_ED=.;NLOD=5.42;TLOD=4.74	GT:AD:AF:ALT_F1R2:ALT_F2R1:FOXOG:QSS:REF_F1R2:REF_F2R1	0/1:48,3:0.061:2:1:0.667:1254,83:16:32	0/0:42,2:0.050:1:1:0.500:1041,56:16:26	0.5	Tolerated	-1.4	Neutral	150	30	0.105	Tolerated	2.8	155
T2df	chr4	**1**	**12.5**	148041745	148041745	G	A	exonic	**Mthfr**	ENSP00000365669	.	nonsynonymous SNV	Mthfr:NM_001161798:exon2:c.G272A:p.R91H,Mthfr:NM_010840:exon2:c.G149A:p.R50H	**R91H**	ENSP00000365669,91,R,H	4,148041745,G,A	4qE2	.	0.25	.	44	chr4	148041745	.	G	A	.	alt_allele_in_normal;t_lod_fstar	ECNT=1;HCNT=1;MAX_ED=.;MIN_ED=.;NLOD=5.42;TLOD=4.74	GT:AD:AF:ALT_F1R2:ALT_F2R1:FOXOG:QSS:REF_F1R2:REF_F2R1	0/1:48,3:0.061:2:1:0.667:1254,83:16:32	0/0:42,2:0.050:1:1:0.500:1041,56:16:26	0.5	Tolerated	-1.4	Neutral	150	30	0.105	Tolerated	2.8	155
T32df	chr3	**1**	**12.5**	89230424	89230443	CAGTCCTCAGTGGCACCTCC	-	exonic	**Muc1**	ENSMUSP00000107872	.	frameshift deletion	Muc1:NM_013605:exon2:c.572_591del:p.A191fs	**A191fs**	ENSMUSP00000107872,191,A,.	NA	3qF1	.	0.25	.	40	chr3	89230423	.	GCAGTCCTCAGTGGCACCTCC	A	.	t_lod_fstar	ECNT=1;HCNT=2;MAX_ED=.;MIN_ED=.;NLOD=6.78;TLOD=4.24	GT:AD:AF:ALT_F1R2:ALT_F2R1:FOXOG:QSS:REF_F1R2:REF_F2R1	0/1:45,5:0.065:2:3:0.400:1213,119:22:23	0/0:28,1:0.036:1:0:1.00:781,13:15:13	19	Deleterious	-11.16	Deleterious	108	30	NA	NA	NA	NA
T12df	chr16	**1**	**12.5**	32753806	32753806	A	T	exonic	**Muc4**	ENSMUSP00000059471	.	nonsynonymous SNV	Muc4:NM_080457:exon3:c.A3681T:p.Q1227H	**Q1227H**	ENSMUSP00000059471,1227,Q,H	16,32753806,A,T	16qB3	.	0.25	.	116	chr16	32753806	.	A	T	.	alt_allele_in_normal;clustered_events;germline_risk;t_lod_fstar	ECNT=2;HCNT=2;MAX_ED=35;MIN_ED=35;NLOD=0.632;TLOD=5.94	GT:AD:AF:ALT_F1R2:ALT_F2R1:FOXOG:PGT:PID:QSS:REF_F1R2:REF_F2R1	0/1:24,2:0.077:2:0:0.00:0|1:32754091_G_A:673,51:9:15	0/0:31,2:0.061:1:1:0.500:0|1:32754091_G_A:846,57:12:19	4	Damaging	-0.28	Neutral	56	30	0.006	Damaging	3.54	10
T2df	chr16	**1**	**12.5**	32753871	32753871	C	T	exonic	**Muc4**	ENSMUSP00000059471	.	nonsynonymous SNV	Muc4:NM_080457:exon3:c.C3746T:p.T1249I	**T1249I**	ENSMUSP00000059471,1249,T,I	16,32753871,C,T	16qB3	.	0.25	.	130	chr16	32753871	.	C	T	.	clustered_events;multi_event_alt_allele_in_normal	ECNT=4;HCNT=22;MAX_ED=67;MIN_ED=2;NLOD=11.97;TLOD=13.65	GT:AD:AF:ALT_F1R2:ALT_F2R1:FOXOG:PGT:PID:QSS:REF_F1R2:REF_F2R1	0/1:40,7:0.152:3:4:0.571:0|1:32755932_A_G:1145,197:25:15	0/0:59,1:0.019:0:1:1.00:0|1:32755932_A_G:1669,30:24:35	4	Damaging	-0.55	Neutral	56	30	0.009	Damaging	3.54	11
T1df	chr16	**1**	**12.5**	32754126	32754126	C	T	exonic	**Muc4**	ENSMUSP00000059471	.	nonsynonymous SNV	Muc4:NM_080457:exon3:c.C4001T:p.S1334L	**S1334L**	ENSMUSP00000059471,1334,S,L	16,32754126,C,T	16qB3	.	0.25	.	33	chr16	32754126	.	C	T	.	alt_allele_in_normal;clustered_events	ECNT=10;HCNT=3;MAX_ED=64;MIN_ED=12;NLOD=20.21;TLOD=7.13	GT:AD:AF:ALT_F1R2:ALT_F2R1:FOXOG:PGT:PID:QSS:REF_F1R2:REF_F2R1	0/1:144,3:0.020:0:3:0.00:0|1:32754674_C_T:4041,81:79:65	0/0:119,3:0.027:1:2:0.333:0|1:32754674_C_T:3306,81:67:52	4	Damaging	-0.91	Neutral	56	30	0.006	Damaging	3.59	7
T32df	chr16	**1**	**12.5**	32755940	32755940	C	T	exonic	**Muc4**	ENSMUSP00000059471	.	nonsynonymous SNV	Muc4:NM_080457:exon4:c.C5813T:p.T1938I	**T1938I**	ENSMUSP00000059471,1938,T,I	16,32755940,C,T	16qB3	.	0.25	.	60	chr16	32755940	.	C	T	.	alt_allele_in_normal;clustered_events	ECNT=6;HCNT=2;MAX_ED=56;MIN_ED=4;NLOD=12.40;TLOD=13.84	GT:AD:AF:ALT_F1R2:ALT_F2R1:FOXOG:PGT:PID:QSS:REF_F1R2:REF_F2R1	0/1:98,5:0.049:0:5:0.00:0|1:32754579_C_A:2788,145:53:45	0/0:106,5:0.049:2:3:0.400:0|1:32754579_C_A:2952,142:58:48	4	Damaging	-1.88	Neutral	56	30	0.002	Damaging	3.41	18
T42df	chr16	**1**	**12.5**	32752089	32752089	T	A	exonic	**Muc4**	ENSMUSP00000059471	.	nonsynonymous SNV	Muc4:NM_080457:exon2:c.T1966A:p.L656I	**L656I**	ENSMUSP00000059471,656,L,I	16,32752089,T,G	16qB3	.	0.25	.	52	chr16	32752089	.	T	G	.	alt_allele_in_normal;clustered_events	ECNT=3;HCNT=24;MAX_ED=8;MIN_ED=1;NLOD=5.14;TLOD=12.02	GT:AD:AF:ALT_F1R2:ALT_F2R1:FOXOG:PGT:PID:QSS:REF_F1R2:REF_F2R1	0/1:47,3:0.083:1:2:.:0|1:54944741_T_A:1281,82:22:25	0/0:50,1:0.043:1:0:.:0|1:54944741_T_A:1387,25:22:28	1.5	Tolerated	-0.22	Neutral	56	30	1	Tolerated	3.43	14

T2df	chr16	**1**	**12.5**	32754128	32754128	C	T	exonic	**Muc4**	ENSMUSP00000059471	.	nonsynonymous SNV	Muc4:NM_080457:exon3:c.C4003T:p.P1335S	**P1335S**	ENSMUSP00000059471,1335,P,S	16,32754128,C,A	16qB3	.	0.25	.	23	chr16	32754128	.	C	A	.	t_lod_fstar	ECNT=1;HCNT=7;MAX_ED=.;MIN_ED=.;NLOD=14.15;TLOD=5.65	GT:AD:AF:ALT_F1R2:ALT_F2R1:FOXOG:QSS:REF_F1R2:REF_F2R1	0/1:33,2:0.056:1:1:0.500:824,51:20:13	0/0:52,0:0.00:0:0:.:1345,0:25:27	4	Tolerated	0.74	Neutral	56	30	0.974	Tolerated	3.59	7
T42df	chr16	**1**	**12.5**	32754635	32754635	G	T	exonic	**Muc4**	ENSMUSP00000059471	.	nonsynonymous SNV	Muc4:NM_080457:exon4:c.G4508T:p.R1503M	**R1503M**	ENSMUSP00000059471,1503,R,M	16,32754635,G,A	16qB3	.	0.25	.	111	chr16	32754635	.	G	A	.	alt_allele_in_normal;germline_risk	ECNT=2;HCNT=7;MAX_ED=1;MIN_ED=1;NLOD=1.77;TLOD=10.99	GT:AD:AF:ALT_F1R2:ALT_F2R1:FOXOG:PGT:PID:QSS:REF_F1R2:REF_F2R1	0/1:37,3:0.098:2:1:0.667:0|1:54944741_T_A:1053,84:20:17	0/0:49,2:0.064:2:0:1.00:0|1:54944741_T_A:1380,56:22:27	1.5	Tolerated	0.15	Neutral	56	30	0.081	Tolerated	3.54	12
T12df	chr16	**1**	**12.5**	32753825	32753825	C	A	exonic	**Muc4**	ENSMUSP00000059471	.	nonsynonymous SNV	Muc4:NM_080457:exon3:c.C3700A:p.Q1234K	**Q1234K**	ENSMUSP00000059471,1234,Q,K	16,32753825,C,T	16qB3	.	0.25	.	117	chr16	32753825	.	C	T	.	alt_allele_in_normal;clustered_events	ECNT=10;HCNT=3;MAX_ED=64;MIN_ED=12;NLOD=16.73;TLOD=7.17	GT:AD:AF:ALT_F1R2:ALT_F2R1:FOXOG:PGT:PID:QSS:REF_F1R2:REF_F2R1	0/1:141,3:0.021:0:3:1.00:0|1:32754674_C_T:3974,83:76:65	0/0:121,4:0.035:2:2:0.500:0|1:32754674_C_T:3443,108:67:54	4	Unknown	NA	NA	56	30	NA	NA	NA	NA
T3df	chr14	**1**	**12.5**	54960424	54960424	T	A	exonic	**Myh6**	ENSMUSP00000000505	.	nonsynonymous SNV	Myh6:NM_001164171:exon13:c.A1256T:p.Q419L,Myh6:NM_010856:exon13:c.A1256T:p.Q419L	**Q419L**	ENSMUSP00000000505,419,Q,L	14,54960424,T,A	14qC3	.	0.25	.	62	chr14	54960424	.	T	A	.	t_lod_fstar	ECNT=1;HCNT=1;MAX_ED=.;MIN_ED=.;NLOD=10.13;TLOD=4.01	GT:AD:AF:ALT_F1R2:ALT_F2R1:FOXOG:QSS:REF_F1R2:REF_F2R1	0/1:10,2:0.167:0:2:0.00:258,54:6:4	0/0:37,0:0.00:0:0:.:1005,0:14:23	0.5	Deleterious	-4.57	Deleterious	529	30	0.009	Damaging	3.89	394
T2df	chr5	**1**	**12.5**	112874322	112874426	TGGCTGCCCCGTGGGCCCTGCTGGCTGTCCCGTGGGTCCCGCTGGCTGCCCCGTGGGCCCCGATGGCTGCCCCGTGGACTCTGATAGCTGTCCCGTGGGCCCTGA	-	exonic	**Myo18b**	ENSMUSP00000116169	.	nonframeshift deletion	Myo18b:NM_028901:exon4:c.1099_1203del:p.367_401del	**367_401del**	NA	5,112874322,CTGGCTGCCCCGTGGGCCCTGCTGGCTGTCCCGTGGGTCCCGCTGGCTGCCCCGTGGGCCCCGATGGCTGCCCCGTGGACTCTGATAGCTGTCCCGTGGGCCCTGA,A	5qF	.	0.25	.	51	chr5	112874321	.	CTGGCTGCCCCGTGGGCCCTGCTGGCTGTCCCGTGGGTCCCGCTGGCTGCCCCGTGGGCCCCGATGGCTGCCCCGTGGACTCTGATAGCTGTCCCGTGGGCCCTGA	A	.	PASS	ECNT=1;HCNT=6;MAX_ED=.;MIN_ED=.;NLOD=8.11;TLOD=139.99	GT:AD:AF:ALT_F1R2:ALT_F2R1:FOXOG:QSS:REF_F1R2:REF_F2R1	0/1:11,46:0.807:23:23:0.500:271,1303:4:7	0/0:30,0:0.00:0:0:.:744,0:13:17	2.9	Unknown	.	.	.	.	.	.	.	.
T32df	chr10	**1**	**12.5**	128043020	128043020	T	A	exonic	**Naca**	ENSMUSP00000121344	.	nonsynonymous SNV	Naca:NM_001113199:exon3:c.T3920A:p.I1307N	**I1307N**	ENSMUSP00000121344,1307,I,N	10,128043020,T,A	10qD3	.	0.25	.	37	chr10	128043020	.	T	A	.	PASS	ECNT=1;HCNT=6;MAX_ED=.;MIN_ED=.;NLOD=8.11;TLOD=139.99	GT:AD:AF:ALT_F1R2:ALT_F2R1:FOXOG:QSS:REF_F1R2:REF_F2R1	0/1:11,46:0.807:23:23:0.500:271,1303:4:7	0/0:30,0:0.00:0:0:.:744,0:13:17	2.9	Damaging	-0.51	Neutral	44	30	0.005	Damaging	3.65	4
T1df	chr19	**1**	**12.5**	5826592	5826593	TG	-	ncRNA_exonic	**Neat1**	NONE	.	.	.	**NA**	19,5826592,ATG,A	NA	19qA	.	0.25	.	32	chr19	5826591	.	ATG	A	.	alt_allele_in_normal	ECNT=1;HCNT=1;MAX_ED=.;MIN_ED=.;NLOD=7.51;TLOD=6.70	GT:AD:AF:ALT_F1R2:ALT_F2R1:FOXOG:QSS:REF_F1R2:REF_F2R1	0/1:39,5:0.116:4:1:0.800:1104,139:21:18	0/0:41,2:0.051:0:2:0.00:1095,55:23:18	0.5	Unkown	.	.	.	.	.	.	.	.
T1df	chr19	**1**	**12.5**	5833937	5833938	TT	-	ncRNA_exonic	**Neat1**	NONE	.	.	.	**NA**	19,5833937,GTT,G	NA	19qA	.	0.25	.	45	chr19	5833936	.	GTT	G	.	PASS	ECNT=1;HCNT=1;MAX_ED=.;MIN_ED=.;NLOD=11.43;TLOD=7.70	GT:AD:AF:ALT_F1R2:ALT_F2R1:FOXOG:QSS:REF_F1R2:REF_F2R1	0/1:30,3:0.097:0:3:..:874,69:13:17	0/0:41,0:0.00:0:0:.:1167,0:14:27	3	Unkown	.	.	.	.	.	.	.	.
T1df	chr10	**1**	**12.5**	92700862	92700862	-	C	exonic	**Nedd1**	ENSMUSP00000091954	.	frameshift insertion	Nedd1:NM_008682:exon6:c.576dupG:p.N193fs	**N193fs**	ENSMUSP00000091954,193,N,	10,92700862,T,C	10qC2	.	0.25	.	53	chr10	92700862	.	T	C	.	PASS	ECNT=1;HCNT=2;MAX_ED=.;MIN_ED=.;NLOD=10.39;TLOD=10.60	GT:AD:AF:ALT_F1R2:ALT_F2R1:FOXOG:QSS:REF_F1R2:REF_F2R1	0/1:29,5:0.152:3:2:0.600:790,143:14:15	0/0:40,0:0.00:0:0:.:1025,0:21:19	1.8	Deleterious	-2.89	Deleterious	93	30	0.017	Damaging	2.85	63
T1df	chr11	**1**	**12.5**	4940384	4940455	GACTTGGCCTCACCTGGGGACTTGACTGCAGCTGGAGACTTGGCCTCAGCTGGTGACTTCACTGCAGCTGGA	-	exonic	**Nefh**	ENSMUSP00000138132	.	nonframeshift deletion	Nefh:NM_010904:exon4:c.2163_2234del:p.721_745del	**721_745del**	NA	11,4940384,GGACTTGGCCTCACCTGGGGACTTGACTGCAGCTGGAGACTTGGCCTCAGCTGGTGACTTCACTGCAGCTGGA,C	11qA1	.	0.25	.	52	chr11	4940383	.	GGACTTGGCCTCACCTGGGGACTTGACTGCAGCTGGAGACTTGGCCTCAGCTGGTGACTTCACTGCAGCTGGA	C	.	str_contraction;t_lod_fstar	ECNT=1;HCNT=1;MAX_ED=.;MIN_ED=.;NLOD=9.61;RPA=6,5;RU=CAG;STR;TLOD=5.41	GT:AD:AF:ALT_F1R2:ALT_F2R1:FOXOG:QSS:REF_F1R2:REF_F2R1	0/1:47,2:0.043:2:0:..:1303,56:23:24	0/0:36,0:0.00:0:0:.:985,0:20:16	6	Unknown	.	.	.	.	.	.	.	.

T42df	chr11	**1**	**12.5**	98328077	98328079	TCC	-	exonic	**Neurod2**	ENSMUSP00000057556	.	nonframeshift deletion	Neurod2:NM_010895:exon2:c.258_260del:p.86_87del	**86_87del**	NA	11,98328077,TTCC,G	11qD	.	0.25	.	23	chr11	98328076	.	TTCC	G	.	PASS	ECNT=1;HCNT=1;MAX_ED=.;MIN_ED=.;NLOD=6.92;TLOD=8.92	GT:AD:AF:ALT_F1R2:ALT_F2R1:FOXOG:QSS:REF_F1R2:REF_F2R1	0/1:40,5:0.114:3:2:0.400:1171,133:16:24	0/0:26,0:0.00:0:0:.:755,0:19:7	0.7	Unknown	.	.	.	.	.	.	.	.
T32df	chr8	**1**	**12.5**	107367146	107367146	T	C	exonic	**Nfat5**	ENSMUSP00000036725	.	nonsynonymous SNV	Nfat5:NM_001286260:exon12:c.T1964C:p.L655S,Nfat5:NM_018823:exon13:c.T2018C:p.L673S,Nfat5:NM_133957:exon14:c.T1736C:p.L579S	**L655S**	ENSMUSP00000036725,655,L,S	8,107367146,T,C	8qD3	.	0.25	.	40	chr8	107367146	.	T	C	.	PASS	ECNT=1;HCNT=2;MAX_ED=.;MIN_ED=.;NLOD=10.39;TLOD=10.60	GT:AD:AF:ALT_F1R2:ALT_F2R1:FOXOG:QSS:REF_F1R2:REF_F2R1	0/1:29,5:0.152:3:2:0.600:790,143:14:15	0/0:40,0:0.00:0:0:.:1025,0:21:19	1.8	Damaging	0.14	Neutral	114	30	0	Damaging	3.9	70
T32df	chr8	**1**	**12.5**	107367146	107367146	T	C	exonic	**Nfat5**	ENSMUSP00000036725	.	nonsynonymous SNV	Nfat5:NM_001286260:exon12:c.T1964C:p.L655S,Nfat5:NM_018823:exon13:c.T2018C:p.L673S,Nfat5:NM_133957:exon14:c.T1736C:p.L579S	**L579S**	ENSMUSP00000036725,579,L,S	8,107367146,T,A	8qD3	.	0.25	.	40	chr8	107367146	.	T	A	.	PASS	DB;ECNT=1;HCNT=1;MAX_ED=.;MIN_ED=.;NLOD=9.33;TLOD=64.03	GT:AD:AF:ALT_F1R2:ALT_F2R1:FOXOG:QSS:REF_F1R2:REF_F2R1	0/1:31,28:0.480:12:16:0.429:888,793:12:19	0/0:36,0:0.00:0:0:.:1046,0:19:17	2.8	Unknown	NA	NA	114	30	NA	NA	NA	NA
T32df	chr8	**1**	**12.5**	107367146	107367146	T	C	exonic	**Nfat5**	ENSMUSP00000036725	.	nonsynonymous SNV	Nfat5:NM_001286260:exon12:c.T1964C:p.L655S,Nfat5:NM_018823:exon13:c.T2018C:p.L673S,Nfat5:NM_133957:exon14:c.T1736C:p.L579S	**L673S**	ENSMUSP00000036725,673,L,S	8,107367146,T,A	8qD3	.	0.25	.	40	chr8	107367146	.	T	A	.	PASS	ECNT=1;HCNT=2;MAX_ED=.;MIN_ED=.;NLOD=10.19;TLOD=11.37	GT:AD:AF:ALT_F1R2:ALT_F2R1:FOXOG:QSS:REF_F1R2:REF_F2R1	0/1:66,4:0.058:2:2:.:1875,117:32:34	0/0:39,0:0.00:0:0:.:1067,0:20:19	1.1	Unknown	NA	NA	114	30	NA	NA	NA	NA
T32df	chr8	**1**	**12.5**	94506676	94506705	GTGTCACTTGGACCTGCCACAGCTGACCAT	-	exonic	**Nlrc5**	ENSMUSP00000069774	.	nonframeshift deletion	Nlrc5:NM_001033207:exon30:c.4074_4103del:p.1358_1368del	**1358_1368del**	NA	8,94506676,AGTGTCACTTGGACCTGCCACAGCTGACCAT,G	8qC5	.	0.25	.	39	chr8	94506675	.	AGTGTCACTTGGACCTGCCACAGCTGACCAT	G	.	clustered_events	DB;ECNT=2;HCNT=2;MAX_ED=75;MIN_ED=75;NLOD=8.68;TLOD=56.28	GT:AD:AF:ALT_F1R2:ALT_F2R1:FOXOG:QSS:REF_F1R2:REF_F2R1	0/1:28,22:0.468:10:12:0.545:802,599:15:13	0/0:33,0:0.00:0:0:.:925,0:15:18	2.8	Unknown	.	.	.	.	.	.	.	.
T2df	chr14	**1**	**12.5**	55746403	55746405	GAG	-	exonic	**Nop9**	ENSMUSP00000117068	.	nonframeshift deletion	Nop9:NM_026403:exon2:c.490_492del:p.164_164del	**164_164del**	NA	14,55746403,AGAG,CACGGTTTCTTCGGCCTTGTCCATCCGGCCATGGCTGTGTCGACGAGGCCAGCGGAACCACCAAAGCTGTGGCTACCTCCTGCTAGAT	14qC3	.	0.25	.	19	chr14	55746402	.	AGAG	CACGGTTTCTTCCGGCCTTGTCCATCCGGCCATGGCTGTGTCGACGAGGCCAGCGGAACCACCAAAGCTGTGGCTACCTCCTGCTAGAT	.	PASS	ECNT=1;HCNT=1;MAX_ED=.;MIN_ED=.;NLOD=10.01;TLOD=7.48	GT:AD:AF:ALT_F1R2:ALT_F2R1:FOXOG:QSS:REF_F1R2:REF_F2R1	0/1:36,1:0.077:1:0:.:1038,28:13:23	0/0:36,0:0.00:0:0:.:1034,0:15:21	0.5	Unknown	NA	NA	78	30	NA	NA	NA	NA
T22df	chr8	**1**	**12.5**	72666798	72666798	-	CAGAA	exonic	**Nwd1**	ENSMUSP00000067448	.	frameshift insertion	Nwd1:NM_176940:exon5:c.688_689insCAGAA:p.G230fs	**G230fs**	ENSMUSP00000067448,230,G,	8,72666798,G,T	8qB3.3	.	0.25	.	22	chr8	72666798	.	G	T	.	multi_event_alt_allele_in_normal;t_lod_fstar	ECNT=2;HCNT=1;MAX_ED=1;MIN_ED=1;NLOD=6.04;TLOD=6.09	GT:AD:AF:ALT_F1R2:ALT_F2R1:FOXOG:PGT:PID:QSS:REF_F1R2:REF_F2R1	0/1:22,2:0.091:1:1:0.500:0|1:72666798_C_T:623,56:13:9	0/0:42,1:0.028:1:0:0.00:0|1:72666798_C_T:1151,29:19:23	22	Deleterious	-3.06	Deleterious	85	30	0.016	Damaging	2.88	34
T22df	chr8	**1**	**12.5**	72666808	72666808	T	G	exonic	**Nwd1**	ENSMUSP00000067448	.	nonsynonymous SNV	Nwd1:NM_176940:exon5:c.T698G:p.L233R	**L233R**	ENSMUSP00000067448,233,L,R	8,72666808,T,CG	8qB3.3	.	0.25	.	22	chr8	72666808	.	T	CG	.	clustered_events;homologous_mapping_event;t_lod_fstar	ECNT=4;HCNT=2;MAX_ED=10;MIN_ED=2;NLOD=5.42;RPA=1,2;RU=G;STR;TLOD=5.53	GT:AD:AF:ALT_F1R2:ALT_F2R1:FOXOG:PGT:PID:QSS:REF_F1R2:REF_F2R1	0/1:38,2:0.049:0:2:.:0|1:72666798_G_GCAGAA:961,44:7:31	0/0:23,0:0.00:0:0:.:0|1:72666798_G_GCAGAA:537,0:0:23	1.9	Unknown	NA	NA	85	30	NA	NA	NA	NA
T22df	chr8	**1**	**12.5**	72666801	72666801	-	G	exonic	**Nwd1**	ENSMUSP00000067448	.	frameshift insertion	Nwd1:NM_176940:exon5:c.692dupG:p.R231fs	**R231fs**	ENSMUSP00000067448,231,R,	8,72666801,C,CTGGGGACAAA	8qB3.3	.	0.25	.	23	chr8	72666801	.	C	CTGGGGACAAA	.	PASS	ECNT=1;HCNT=16;MAX_ED=.;MIN_ED=.;NLOD=7.56;TLOD=35.04	GT:AD:AF:ALT_F1R2:ALT_F2R1:FOXOG:QSS:REF_F1R2:REF_F2R1	0/1:37,11:0.229:5:6:.:1059,310:25:12	0/0:29,0:0.00:0:0:.:823,0:13:16	22	Unknown	NA	NA	85	30	NA	NA	NA	NA
T3df	chr11	**1**	**12.5**	59099957	59099957	G	T	exonic	**Obscn**	ENSMUSP00000139309	.	nonsynonymous SNV	Obscn:NM_001171512:exon16:c.C4789A:p.L1597M,Obscn:NM_199152:exon16:c.C4864A:p.L1622M	**L1597M**	ENSMUSP00000139309,1597,L,M	11,59099957,G,C	11qB1.3	.	0.25	.	41	chr11	59099957	.	G	C	.	clustered_events;homologous_mapping_event	ECNT=8;HCNT=16;MAX_ED=69;MIN_ED=4;NLOD=10.84;TLOD=8.00	GT:AD:AF:ALT_F1R2:ALT_F2R1:FOXOG:PGT:PID:QSS:REF_F1R2:REF_F2R1	0/1:75,3:0.039:2:1:0.667:0|1:59099925_G_A:2104,80:35:40	0/0:41,0:0.00:0:0:.:0|1:59099925_G_A:1163,0:18:23	22	Damaging	-1.69	Neutral	199	30	0.003	Damaging	2.98	217

T3df	chr11	**1**	**12.5**	59054369	59054369	C	T	exonic	**Obscn**	ENSMUSP00000016094	.	nonsynonymous SNV	Obscn:NM_199152:exon51:c.G13822A:p.V4608M	**V4608M**	ENSMUSP00000016094,4608,V,M	11,59054369,C,C	11qB1.3	.	0.25	.	41	chr11	59054369	.	C	C	.	clustered_events;homologous_mapping_event	ECNT=8;HCNT=16;MAX_ED=69;MIN_ED=4;NLOD=10.84;TLOD=8.00	GT:AD:AF:ALT_F1R2:ALT_F2R1:FOXOG:PGT:PID:QSS:REF_F1R2:REF_F2R1	0/1:75,3:0.039:2:1:0.667:0|1:59099925_G_A:2104,80:35:40	0/0:41,0:0.00:0:0:.:0|1:59099925_G_A:1163,0:18:23	22	Tolerated	0	Neutral	66	30	0.249	Tolerated	2.77	28
T1df	chr11	**1**	**12.5**	59054855	59054855	A	G	exonic	**Obscn**	ENSMUSP00000016094	.	nonsynonymous SNV	Obscn:NM_001171512:exon46:c.T12529C:p.C4177R,Obscn:NM_199152:exon50:c.T13684C:p.C4562R	**C4562R**	ENSMUSP00000016094,4562,C,R	11,59054855,A,C	11qB1.3	.	0.25	.	49	chr11	59054855	.	A	C	.	clustered_events;homologous_mapping_event	DB;ECNT=8;HCNT=16;MAX_ED=69;MIN_ED=4;NLOD=10.23;TLOD=8.01	GT:AD:AF:ALT_F1R2:ALT_F2R1:FOXOG:PGT:PID:QSS:REF_F1R2:REF_F2R1	0/1:75,3:0.039:2:1:0.667:0|1:59099925_G_A:2107,68:35:40	0/0:37,0:0.00:0:0:.:0|1:59099925_G_A:1043,0:15:22	22	Tolerated	-0.94	Neutral	66	30	0.163	Tolerated	2.77	28
T1df	chr11	**1**	**12.5**	59054855	59054855	A	G	exonic	**Obscn**	ENSMUSP00000016094	.	nonsynonymous SNV	Obscn:NM_001171512:exon46:c.T12529C:p.C4177R,Obscn:NM_199152:exon50:c.T13684C:p.C4562R	**C4177R**	ENSMUSP00000016094,4177,C,R	11,59054855,A,T	11qB1.3	.	0.25	.	49	chr11	59054855	.	A	T	.	alt_allele_in_normal;clustered_events;germline_risk;t_lod_fstar	ECNT=2;HCNT=3;MAX_ED=31;MIN_ED=31;NLOD=2.19;TLOD=5.09	GT:AD:AF:ALT_F1R2:ALT_F2R1:FOXOG:QSS:REF_F1R2:REF_F2R1	0/1:68,2:0.029:0:2:1.00:1863,54:42:26	0/0:39,2:0.054:0:2:1.00:1103,55:15:24	22	Tolerated	0.32	Neutral	66	30	0.164	Tolerated	2.77	28
T3df	chr11	**1**	**12.5**	59099929	59099929	T	C	exonic	**Obscn**	ENSMUSP00000016094	.	nonsynonymous SNV	Obscn:NM_001171512:exon16:c.A4817G:p.H1606R,Obscn:NM_199152:exon16:c.A4892G:p.H1631R	**H1606R**	ENSMUSP00000016094,1606,H,R	11,59099929,T,A	11qB1.3	rs6206347	0.25	.	37	chr11	59099929	rs6206347	T	A	.	clustered_events;homologous_mapping_event	DB;ECNT=8;HCNT=16;MAX_ED=69;MIN_ED=4;NLOD=11.14;TLOD=8.05	GT:AD:AF:ALT_F1R2:ALT_F2R1:FOXOG:PGT:PID:QSS:REF_F1R2:REF_F2R1	0/1:74,3:0.041:2:1:0.667:0|1:59099925_G_A:1844,83:36:38	0/0:41,0:0.00:0:0:.:0|1:59099925_G_A:1031,0:17:24	22	Tolerated	-1.95	Neutral	66	30	0.519	Tolerated	2.83	19
T3df	chr11	**1**	**12.5**	59099929	59099929	T	C	exonic	**Obscn**	ENSMUSP00000016094	.	nonsynonymous SNV	Obscn:NM_001171512:exon16:c.A4817G:p.H1606R,Obscn:NM_199152:exon16:c.A4892G:p.H1631R	**H1631R**	ENSMUSP00000016094,1631,H,R	11,59099929,T,A	11qB1.3	rs6206347	0.25	.	37	chr11	59099929	rs6206347	T	A	.	clustered_events;homologous_mapping_event	DB;ECNT=8;HCNT=16;MAX_ED=69;MIN_ED=4;NLOD=11.14;TLOD=8.05	GT:AD:AF:ALT_F1R2:ALT_F2R1:FOXOG:PGT:PID:QSS:REF_F1R2:REF_F2R1	0/1:74,3:0.041:2:1:0.667:0|1:59099925_G_A:1844,83:36:38	0/0:41,0:0.00:0:0:.:0|1:59099925_G_A:1031,0:17:24	22	Tolerated	-1.95	Neutral	66	30	0.519	Tolerated	2.83	19
T3df	chr11	**1**	**12.5**	59099941	59099941	T	C	exonic	**Obscn**	ENSMUSP00000016094	.	nonsynonymous SNV	Obscn:NM_001171512:exon16:c.A4805G:p.E1602G,Obscn:NM_199152:exon16:c.A4880G:p.E1627G	**E1602G**	ENSMUSP00000016094,1602,E,G	11,59099941,T,T	11qB1.3	.	0.25	.	41	chr11	59099941	.	T	T	.	clustered_events;homologous_mapping_event	ECNT=8;HCNT=16;MAX_ED=69;MIN_ED=4;NLOD=10.84;TLOD=8.05	GT:AD:AF:ALT_F1R2:ALT_F2R1:FOXOG:PGT:PID:QSS:REF_F1R2:REF_F2R1	0/1:72,3:0.041:2:1:0.667:0|1:59099925_G_A:2030,84:33:39	0/0:41,0:0.00:0:0:.:0|1:59099925_G_A:1148,0:18:23	22	Tolerated	0	Neutral	66	30	1	Tolerated	2.77	28
T3df	chr11	**1**	**12.5**	59099941	59099941	T	C	exonic	**Obscn**	ENSMUSP00000016094	.	nonsynonymous SNV	Obscn:NM_001171512:exon16:c.A4805G:p.E1602G,Obscn:NM_199152:exon16:c.A4880G:p.E1627G	**E1627G**	ENSMUSP00000016094,1627,E,G	11,59099941,T,T	11qB1.3	.	0.25	.	41	chr11	59099941	.	T	T	.	clustered_events;homologous_mapping_event	ECNT=8;HCNT=16;MAX_ED=69;MIN_ED=4;NLOD=10.84;TLOD=8.05	GT:AD:AF:ALT_F1R2:ALT_F2R1:FOXOG:PGT:PID:QSS:REF_F1R2:REF_F2R1	0/1:72,3:0.041:2:1:0.667:0|1:59099925_G_A:2030,84:33:39	0/0:41,0:0.00:0:0:.:0|1:59099925_G_A:1148,0:18:23	22	Tolerated	0	Neutral	66	30	1	Tolerated	2.77	28
T3df	chr11	**1**	**12.5**	59099953	59099953	G	A	exonic	**Obscn**	ENSMUSP00000016094	.	nonsynonymous SNV	Obscn:NM_001171512:exon16:c.C4793T:p.A1598V,Obscn:NM_199152:exon16:c.C4868T:p.A1623V	**A1623V**	ENSMUSP00000016094,1623,A,V	11,59099953,G,A	11qB1.3	.	0.25	.	41	chr11	59099953	.	G	A	.	clustered_events	ECNT=2;HCNT=3;MAX_ED=8;MIN_ED=8;NLOD=13.54;TLOD=6.44	GT:AD:AF:ALT_F1R2:ALT_F2R1:FOXOG:QSS:REF_F1R2:REF_F2R1	0/1:13,2:0.133:2:0:0.00:374,56:3:10	0/0:49,0:0.00:0:0:.:1375,0:28:21	0.4	Tolerated	1.65	Neutral	66	30	1	Tolerated	2.8	27
T3df	chr11	**1**	**12.5**	59099953	59099953	G	A	exonic	**Obscn**	ENSMUSP00000016094	.	nonsynonymous SNV	Obscn:NM_001171512:exon16:c.C4793T:p.A1598V,Obscn:NM_199152:exon16:c.C4868T:p.A1623V	**A1598V**	ENSMUSP00000016094,1598,A,V	11,59099953,G,C	11qB1.3	.	0.25	.	41	chr11	59099953	.	G	C	.	clustered_events;homologous_mapping_event	DB;ECNT=8;HCNT=16;MAX_ED=69;MIN_ED=4;NLOD=10.23;TLOD=8.01	GT:AD:AF:ALT_F1R2:ALT_F2R1:FOXOG:PGT:PID:QSS:REF_F1R2:REF_F2R1	0/1:75,3:0.039:2:1:0.667:0|1:59099925_G_A:2107,68:35:40	0/0:37,0:0.00:0:0:.:0|1:59099925_G_A:1043,0:15:22	22	Tolerated	-1.72	Neutral	66	30	0.176	Tolerated	2.8	27
T3df	chr11	**1**	**12.5**	59099957	59099957	G	T	exonic	**Obscn**	ENSMUSP00000016094	.	nonsynonymous SNV	Obscn:NM_001171512:exon16:c.C4789A:p.L1597M,Obscn:NM_199152:exon16:c.C4864A:p.L1622M	**L1622M**	ENSMUSP00000016094,1622,L,M	11,59099957,G,G	11qB1.3	.	0.25	.	41	chr11	59099957	.	G	G	.	clustered_events	ECNT=2;HCNT=2;MAX_ED=8;MIN_ED=8;NLOD=14.14;TLOD=6.44	GT:AD:AF:ALT_F1R2:ALT_F2R1:FOXOG:QSS:REF_F1R2:REF_F2R1	0/1:13,2:0.133:2:0:.:379,58:3:10	0/0:51,0:0.00:0:0:.:1432,0:31:20	0.4	Tolerated	0	Neutral	66	30	0.504	Tolerated	2.83	26
T3df	chr15	**1**	**12.5**	38226494	38226538	TGCGGCCCCTGTGGCCCCTGTGGCCCCTGTGGTCCCTGCGGCCCC	-	exonic	**Odf1**	ENSMUSP000000120118	.	nonframeshift deletion	Odf1:NM_008757:exon2:c.637_681del:p.213_227del	**213_227del**	NA	15,38226494,TTGCGGCCCCTGTGGCCCCTGTGGCCCCTGTGGTCCCTGCGGCCCC,T	15qB3.1	.	0.25	.	44	chr15	38226493	.	TTGCGGCCCCTGTGGCCCCTGTGGCCCCTGTGGTCCCTGCGGCCCC	T	.	t_lod_fstar	ECNT=1;HCNT=1;MAX_ED=.;MIN_ED=.;NLOD=12.33;TLOD=5.25	GT:AD:AF:ALT_F1R2:ALT_F2R1:FOXOG:QSS:REF_F1R2:REF_F2R1	0/1:46,3:0.067:3:0:0.00:1317,86:24:22	0/0:46,0:0.00:0:0:.:1276,0:20:26	1.3	Unknown	.	.	.	.	.	.	.	.

T32df	chr13	**1**	**12.5**	40180668	40180670	GAA	-	exonic	**Ofcc1**	ENSMUSP00000028320	.	nonframeshift deletion	Ofcc1:NM_172143:exon9:c.944_946del:p.315_316del	**315_316del**	NA	13,40180668,GGAA,A	13qA3.3	.	0.25	.	51	chr13	40180667	.	GGAA	A	.	PASS	DB;ECNT=1;HCNT=1;MAX_ED=.;MIN_ED=.;NLOD=8.97;TLOD=27.40	GT:AD:AF:ALT_F1R2:ALT_F2R1:FOXOG:QSS:REF_F1R2:REF_F2R1	0/1:20,11:0.355:8:3:0.727:559,310:16:4	0/0:32,0:0.00:0:0:.:791,0:14:18	.	Unknown	.	.	.	.	.	.	.	.
T32df	chr13	**1**	**12.5**	40180675	40180675	C	A	splicing	**Ofcc1**	ENSMUSP00000028320	.	NM_172143:exon9:c.940-1G>T	.	**NA**	13,40180675,C,T	NA	13qA3.3	.	0.25	.	49	chr13	40180675	.	C	T	.	PASS	DB;ECNT=1;HCNT=2;MAX_ED=.;MIN_ED=.;NLOD=12.23;TLOD=29.41	GT:AD:AF:ALT_F1R2:ALT_F2R1:FOXOG:QSS:REF_F1R2:REF_F2R1	0/1:31,13:0.310:7:6:0.462:749,352:13:18	0/0:48,0:0.00:0:0:.:1156,0:20:28	1.7	Unkown	.	.	.	.	.	.	.	.
T32df	chr2	**1**	**12.5**	119617920	119617920	-	TGGAGAAGGCCAGGGCGCCGAGGGACCGCGACAGGTCCCAGGCGAGGTGCAGGGTGTCA	exonic	**Oip5**	ENSMUSP00000124728	.	frameshift insertion	Oip5:NM_001042653:exon2:c.304_305insTGACACCCTGCACCTCGCCTGGGACCTGTCGCGGTCCCTCGGCGCCCTGGCCTTCTCCA:p.K102fs	**K102fs**	ENSMUSP00000124728,102,K,	2,119617920,T,G	2qE5	.	0.25	.	42	chr2	119617920	.	T	G	.	PASS	ECNT=1;HCNT=2;MAX_ED=.;MIN_ED=.;NLOD=10.13;TLOD=93.84	GT:AD:AF:ALT_F1R2:ALT_F2R1:FOXOG:QSS:REF_F1R2:REF_F2R1	0/1:12,35:0.744:16:19:0.543:307,978:3:9	0/0:35,0:0.00:0:0:.:832,0:22:13	1.7	Deleterious	-2.51	Deleterious	48	19	0.006	Damaging	3.12	39
T32df	chr2	**1**	**12.5**	119617922	119617922	T	C	splicing	**Oip5**	ENSMUSP00000124728	NM_001042653:exon2:c.305-2A>G	.	.	**NA**	2,119617922,T,G	NA	2qE5	.	0.25	.	42	chr2	119617922	.	T	G	.	PASS	ECNT=1;HCNT=2;MAX_ED=.;MIN_ED=.;NLOD=10.13;TLOD=93.84	GT:AD:AF:ALT_F1R2:ALT_F2R1:FOXOG:QSS:REF_F1R2:REF_F2R1	0/1:12,35:0.744:16:19:0.543:307,978:3:9	0/0:35,0:0.00:0:0:.:832,0:22:13	1.7	Unkown	.	.	.	.	.	.	.	.
T12df	chr2	**1**	**12.5**	163407091	163407107	TAAAAGAGAAAAAGACA	-	splicing	**Oser1**	ENSMUSP00000121411	.	.	.	**NA**	2,163407091,CTAAAAGAGAAAAAGACA,T	NA	2qH3	.	0.25	.	28	chr2	163407090	.	CTAAAAGAGAAAAAGACA	T	.	alt_allele_in_normal	ECNT=1;HCNT=3;MAX_ED=.;MIN_ED=.;NLOD=6.28;TLOD=27.25	GT:AD:AF:ALT_F1R2:ALT_F2R1:FOXOG:QSS:REF_F1R2:REF_F2R1	0/1:23,11:0.324:6:5:0.455:626,313:11:12	0/0:46,2:0.047:2:0:0.00:1176,56:25:21	1.4	Unkown	.	.	.	.	.	.	.	.
T1df	chr11	**1**	**12.5**	4239889	4239918	CAAGGGAACCCGCAGAATCCGGGTCCGGCG	-	exonic	**Osm**	ENSMUSP00000113939	.	nonframeshift deletion	Osm:NM_001013365:exon3:c.672_701del:p.224_234del	**224_234del**	NA	11,4239889,ACAAGGGAACCCGCAGAATCCGGGTCCGGCG,T	11qA1	.	0.25	.	36	chr11	4239888	.	ACAAGGGAACCCGCAGAATCCGGGTCCGGCG	T	.	alt_allele_in_normal;clustered_events	ECNT=2;HCNT=4;MAX_ED=12;MIN_ED=12;NLOD=5.20;TLOD=7.42	GT:AD:AF:ALT_F1R2:ALT_F2R1:FOXOG:QSS:REF_F1R2:REF_F2R1	0/1:33,4:0.114:2:2:0.500:789,109:20:13	0/0:56,3:0.061:2:1:0.333:1420,81:35:21	1.8	Unknown	.	.	.	.	.	.	.	.
T1df	chr10	**1**	**12.5**	107777082	107777082	A	T	exonic	**Otogl**	ENSMUSP00000120605	.	nonsynonymous SNV	Otogl:NM_001177567:exon51:c.T6182A:p.V2061E	**V2061E**	ENSMUSP00000120605,2061,V,E	10,107777082,A,C	10qD1	.	0.25	.	50	chr10	107777082	.	A	C	.	clustered_events;homologous_mapping_event	DB;ECNT=4;HCNT=2;MAX_ED=66;MIN_ED=64;NLOD=11.14;TLOD=107.88	GT:AD:AF:ALT_F1R2:ALT_F2R1:FOXOG:PGT:PID:QSS:REF_F1R2:REF_F2R1	0/1:38,29:0.446:19:10:0.655:0|1:129400088_G_A:1053,735:13:25	0/0:42,0:0.00:0:0:.:0|1:129400088_G_A:1180,0:19:23	2.2	Deleterious	-4.51	Deleterious	73	30	0.002	Damaging	3.1	57
T22df	chr15	**1**	**12.5**	83390562	83390562	A	G	exonic	**Pacsin2**	ENSMUSP00000117554	.	nonsynonymous SNV	Pacsin2:NM_001159509:exon4:c.T314C:p.M105T,Pacsin2:NM_001159510:exon4:c.T314C:p.M105T,Pacsin2:NM_011862:exon4:c.T314C:p.M105T	**M105T**	ENSMUSP00000117554,105,M,T	15,83390562,A,CATATTTGGATGATATCGATGATGAGATAGACCCAGAAATTGAAGAAGCTTAT	15qE1	.	0.25	.	41	chr15	83390562	.	A	CATATTTGGATGATATCGATGATGAGATAGACCCAGAAATTGAAGAAGCTTAT	.	t_lod_fstar	ECNT=2;HCNT=2;MAX_ED=2;MIN_ED=2;NLOD=9.28;TLOD=5.98	GT:AD:AF:ALT_F1R2:ALT_F2R1:FOXOG:PGT:PID:QSS:REF_F1R2:REF_F2R1	0/1:24,2:0.083:0:2:.:0|1:119455789_C_CATATTTGGATGATATCGATGATGAGATAGACCCAGAAATTGAAGAAGCTTAT:615,58:14:10	0/0:34,0:0.00:0:0:.:0|1:119455789_C_CATATTTGGATGATATCGATGATGAGATAGACCCAGAAATTGAAGAAGCTTAT:798,0:18:16	2.3	Deleterious	-4.48	Deleterious	76	8	0.001	Damaging	3.69	65
T32df	chr13	**1**	**12.5**	119455789	119455789	-	ATATTTGGATGATATCGATGATGAGATAGACCCAGAAATTGAAGAAGCTTAT	exonic	**Paip1**	ENSMUSP00000118130	.	frameshift insertion	Paip1:NM_001079849:exon10:c.1010_1011insATATTTGGATGATATCGATGATGAGATAGACCCAGAAATTGAAGAAGCTTAT:p.P337fs,Paip1:NM_145457:exon10:c.1109_1110insATATTTGGATGATATCGATGATGAGATAGACCCAGAAATTGAAGAAGCTTAT:p.P370fs	**P370fs**	ENSMUSP00000118130,370,P,	13,119455789,C,A	13qD2.3	.	0.25	.	34	chr13	119455789	.	C	A	.	PASS	ECNT=1;HCNT=1;MAX_ED=.;MIN_ED=.;NLOD=7.47;TLOD=14.95	GT:AD:AF:ALT_F1R2:ALT_F2R1:FOXOG:QSS:REF_F1R2:REF_F2R1	0/1:38,8:0.163:5:3:0.625:1045,230:19:19	0/0:26,0:0.00:0:0:.:675,0:14:12	1.4	Tolerated	-1.02	Neutral	89	30	0.403	Tolerated	3.18	53

T32df	chr13	**1**	**12.5**	119455789	119455789	-	ATATTTGGATGATATCGATGATGAGATAGACCCAGAAATTGAAGAAGCTTAT	exonic	**Paip1**	ENSMUSP00000118130	.	frameshift insertion	Paip1:NM_001079849:exon10:c.1010_1011insATATTTGGATGATATCGATGATGAGATAGACCCAGAAATTGAAGAAGCTTAT:p.P337fs,Paip1:NM_145457:exon10:c.1109_1110insATATTTGGATGATATCGATGATGAGATAGACCCAGAAATTGAAGAAGCTTAT:p.P370fs	**P337fs**	ENSMUSP00000118130,337,P,	13,119455789,C,C	13qD2.3	.	0.25	.	34	chr13	119455789	.	C	C	.	t_lod_fstar	ECNT=1;HCNT=4;MAX_ED=.;MIN_ED=.;NLOD=12.48;TLOD=5.19	GT:AD:AF:ALT_F1R2:ALT_F2R1:FOXOG:QSS:REF_F1R2:REF_F2R1	0/1:59,2:0.033:2:0:.:1680,53:38:21	0/0:50,0:0.00:0:0:.:1316,0:23:27	0.7	Tolerated	0	Neutral	89	30	1	Tolerated	3.18	53
T32df	chr10	**1**	**12.5**	79816852	79816877	TGTCCAGCACCACAGTGCTCCCCCGG	-	exonic	**Palm**	ENSMUSP00000019441	.	frameshift deletion	Palm:NM_023128:exon8:c.560_585del:p.L187fs	**L187fs**	ENSMUSP00000019441,187,L,.	10,79816852,CTGTCCAGCACCACAGTGCTCCCCCGG,GT	10qC1	.	0.25	.	50	chr10	79816851	.	CTGTCCAGCACCACAGTGCTCCCCCGG	GT	.	clustered_events;homologous_mapping_event	ECNT=7;HCNT=1;MAX_ED=22;MIN_ED=3;NLOD=8.73;TLOD=8.09	GT:AD:AF:ALT_F1R2:ALT_F2R1:FOXOG:PGT:PID:QSS:REF_F1R2:REF_F2R1	0/1:44,5:0.102:0:5:.:0|1:93321384_G_GGTGGTC:1182,64:26:18	0/0:29,0:0.00:0:0:.:0|1:93321384_G_GGTGGTC:805,0:13:16	0.3	Unknown	NA	NA	112	30	NA	NA	NA	NA
T12df	chr1	**1**	**12.5**	20935276	20935276	G	A	exonic	**Paqr8**	ENSMUSP00000028288	.	nonsynonymous SNV	Paqr8:NM_001355122:exon3:c.G653A:p.G218E,Paqr8:NM_028829:exon3:c.G653A:p.G218E	**G218E**	ENSMUSP00000028288,218,G,E	1,20935276,G,T	1qA4	.	0.25	.	26	chr1	20935276	.	G	T	.	clustered_events;homologous_mapping_event	ECNT=7;HCNT=1;MAX_ED=22;MIN_ED=3;NLOD=9.63;TLOD=8.27	GT:AD:AF:ALT_F1R2:ALT_F2R1:FOXOG:PGT:PID:QSS:REF_F1R2:REF_F2R1	0/1:40,5:0.111:0:5:1.00:0|1:93321384_G_GGTGGTC:1071,75:24:16	0/0:30,0:0.00:0:0:.:0|1:93321384_G_GGTGGTC:818,0:14:16	0.3	Deleterious	-4	Deleterious	120	30	0.136	Tolerated	2.88	98
T22df	chr1	**1**	**12.5**	93321401	93321401	A	T	exonic	**Pask**	ENSMUSP00000001561	.	nonsynonymous SNV	Pask:NM_080850:exon10:c.T2276A:p.F759Y	**F759Y**	ENSMUSP00000001561,759,F,Y	1,93321401,A,C	1qD	.	0.25	.	30	chr1	93321401	.	A	C	.	clustered_events;multi_event_alt_allele_in_normal	ECNT=7;HCNT=1;MAX_ED=22;MIN_ED=3;NLOD=4.23;TLOD=15.49	GT:AD:AF:ALT_F1R2:ALT_F2R1:FOXOG:PGT:PID:QSS:REF_F1R2:REF_F2R1	0/1:45,5:0.102:0:5:.:0|1:93321384_G_GGTGGTC:1221,69:26:19	0/0:30,1:0.00:0:0:.:0|1:93321384_G_GGTGGTC:862,0:14:16	0.3	Damaging	-0.58	Neutral	89	30	0.045	Damaging	3.48	58
T4df	chr1	**1**	**12.5**	93321397	93321397	-	T	exonic	**Pask**	ENSMUSP00000001561	.	stopgain	Pask:NM_080850:exon10:c.2279_2280insA:p.C760_D761delinsX	**C760_D761delinsX**	ENSMUSP00000001561,760,CD,X	NA	1qD	.	0.25	.	33	chr1	93321397	.	A	A	.	alt_allele_in_normal;t_lod_fstar	ECNT=2;HCNT=6;MAX_ED=1;MIN_ED=1;NLOD=12.07;TLOD=5.78	GT:AD:AF:ALT_F1R2:ALT_F2R1:FOXOG:PGT:PID:QSS:REF_F1R2:REF_F2R1	0/1:31,0:0.065:0:0:.:0|1:37335215_T_C:894,0:16:15	0/0:72,2:0.029:1:1:0.500:0|1:37335215_T_C:2015,56:34:38	0.8	Deletarious	.	.	.	.	.	.	.	.
T22df	chr1	**1**	**12.5**	93321404	93321404	G	A	exonic	**Pask**	ENSMUSP00000001561	.	nonsynonymous SNV	Pask:NM_080850:exon10:c.C2273T:p.S758F	**S758F**	ENSMUSP00000001561,758,S,F	1,93321404,G,T	1qD	.	0.25	.	30	chr1	93321404	.	G	T	.	clustered_events;homologous_mapping_event;str_contraction	ECNT=7;HCNT=1;MAX_ED=22;MIN_ED=3;NLOD=9.03;RPA=2,1;RU=CA;STR;TLOD=8.13	GT:AD:AF:ALT_F1R2:ALT_F2R1:FOXOG:PGT:PID:QSS:REF_F1R2:REF_F2R1	0/1:44,5:0.104:0:5:.:0|1:93321384_G_GGTGGTC:1197,82:26:18	0/0:32,0:0.00:0:0:.:0|1:93321384_G_GGTGGTC:880,0:15:17	0.3	Deleterious	-2.84	Deleterious	89	30	0.002	Damaging	3.45	60
T4df	chr1	**1**	**12.5**	93321398	93321398	C	T	exonic	**Pask**	ENSMUSP00000001561	.	nonsynonymous SNV	Pask:NM_080850:exon10:c.G2279A:p.C760Y	**C760Y**	ENSMUSP00000001561,760,C,Y	1,93321398,C,AT	1qD	.	0.25	.	33	chr1	93321398	.	C	AT	.	alt_allele_in_normal;clustered_events	ECNT=5;HCNT=1;MAX_ED=10;MIN_ED=4;NLOD=4.82;TLOD=9.31	GT:AD:AF:ALT_F1R2:ALT_F2R1:FOXOG:PGT:PID:QSS:REF_F1R2:REF_F2R1	0/1:25,3:0.103:0:3:.:0|1:93321393_G_GT:709,87:11:14	0/0:32,1:0.032:0:1:.:0|1:93321393_G_GT:878,29:15:17	0.3	Unknown	NA	NA	89	30	NA	NA	NA	NA
T22df	chr1	**1**	**12.5**	93321384	93321384	-	GTGGTC	exonic	**Pask**	ENSMUSP00000001561	.	nonframeshift insertion	Pask:NM_080850:exon10:c.2292_2293insGACCAC:p.R765delinsDHR	**R765delinsDHR**	ENSMUSP00000001561,765,R,DHR	1,93321384,G,A	1qD	.	0.25	.	29	chr1	93321384	.	G	A	.	clustered_events;multi_event_alt_allele_in_normal	ECNT=5;HCNT=1;MAX_ED=10;MIN_ED=4;NLOD=5.13;TLOD=9.41	GT:AD:AF:ALT_F1R2:ALT_F2R1:FOXOG:PGT:PID:QSS:REF_F1R2:REF_F2R1	0/1:25,3:0.111:0:3:.:0|1:93321393_G_GT:710,79:12:13	0/0:30,1:0.031:0:1:.:0|1:93321393_G_GT:856,12:13:17	0.3	Unknown	NA	NA	89	30	NA	NA	NA	NA
T22df	chr1	**1**	**12.5**	93321388	93321392	TCCCA	-	exonic	**Pask**	ENSMUSP00000001561	.	frameshift deletion	Pask:NM_080850:exon10:c.2285_2289del:p.L762fs	**L762fs**	ENSMUSP00000001561,762,L,.	1,93321388,CTCCCA,GGTGGTC	1qD	.	0.25	.	31	chr1	93321387	.	CTCCCA	GGTGGTC	.	alt_allele_in_normal;clustered_events	ECNT=7;HCNT=2;MAX_ED=22;MIN_ED=3;NLOD=2.94;TLOD=15.68	GT:AD:AF:ALT_F1R2:ALT_F2R1:FOXOG:PGT:PID:QSS:REF_F1R2:REF_F2R1	0/1:40,5:0.111:0:5:.:0|1:93321384_G_GGTGGTC:1053,115:22:18	0/0:27,2:0.071:0:2:.:0|1:93321384_G_GGTGGTC:691,35:14:13	0.3	Unknown	NA	NA	89	30	NA	NA	NA	NA
T22df	chr1	**1**	**12.5**	93321396	93321397	CA	-	exonic	**Pask**	ENSMUSP00000001561	.	frameshift deletion	Pask:NM_080850:exon10:c.2280_2281del:p.C760fs	**C760fs**	ENSMUSP00000001561,760,C,.	1,93321396,TCA,T	1qD	.	0.25	.	32	chr1	93321395	.	TCA	T	.	alt_allele_in_normal;clustered_events	ECNT=5;HCNT=1;MAX_ED=10;MIN_ED=4;NLOD=4.82;TLOD=9.36	GT:AD:AF:ALT_F1R2:ALT_F2R1:FOXOG:PGT:PID:QSS:REF_F1R2:REF_F2R1	0/1:25,3:0.107:0:3:1.00:0|1:93321393_G_GT:704,80:11:14	0/0:32,1:0.032:0:1:1.00:0|1:93321393_G_GT:918,27:15:17	0.3	Unknown	NA	NA	89	30	NA	NA	NA	NA

T4df	chr1	**1**	**12.5**	93321404	93321406	GAC	-	exonic	**Pask**	ENSMUSP0000001561	.	nonframeshift deletion	Pask:NM_080850:exon10:c.2271_2273del:p.757_758del	**757_758del**	NA	1,93321404,AGAC,G	1qD	.	0.25	.	31	chr1	93321403	.	AGAC	G	.	str_contraction;t_lod_fstar	ECNT=1;HCNT=2;MAX_ED=.;MIN_ED=.;NLOD=8.37;RPA=4,3;RU=GGC;STR;TLOD=5.67	GT:AD:AF:ALT_F1R2:ALT_F2R1:FOXOG:QSS:REF_F1R2:REF_F2R1	0/1:34,2:0.057:2:0:.:871,40:17:17	0/0:32,0:0.00:0:0:.:801,0:17:15	2.2	Unknown	.	.	.	.	.	.	.	.
T4df	chr2	**1**	**12.5**	147365101	147365103	GGC	-	exonic	**Pax1**	ENSMUSP00000122305	.	nonframeshift deletion	Pax1:NM_008780:exon1:c.54_56del:p.18_19del	**18_19del**	NA	2,147365101,GGGC,A	2qG2	.	0.25	.	32	chr2	147365100	.	GGGC	A	.	PASS	ECNT=1;HCNT=1;MAX_ED=.;MIN_ED=.;NLOD=12.92;TLOD=7.77	GT:AD:AF:ALT_F1R2:ALT_F2R1:FOXOG:QSS:REF_F1R2:REF_F2R1	0/1:35,4:0.105:2:2:0.500:853,114:21:14	0/0:45,0:0.00:0:0:.:1118,0:20:25	2	Unknown	.	.	.	.	.	.	.	.
T32df	chr18	**1**	**12.5**	36947078	36947078	A	-	exonic	**Pcdha3**	ENSMUSP00000134266	.	frameshift deletion	Pcdha3:NM_138662:exon1:c.872delA:p.H291fs	**H291fs**	ENSMUSP00000134266,291,H,.	18,36947078,CA,C	18qB2	.	0.25	.	89	chr18	36947077	.	CA	C	.	str_contraction;t_lod_fstar	ECNT=1;HCNT=2;MAX_ED=.;MIN_ED=.;NLOD=5.71;RPA=8,7;RU=CTG;STR;TLOD=4.67	GT:AD:AF:ALT_F1R2:ALT_F2R1:FOXOG:QSS:REF_F1R2:REF_F2R1	0/1:45,2:0.044:0:2:.:1261,57:18:27	0/0:21,0:0.00:0:0:.:580,0:17:4	14	Unkown	.	.	.	.	.	.	.	.
T2df	chr18	**1**	**12.5**	36993241	36993241	T	A	exonic	**Pcdha8**	ENSMUSP00000064333	.	nonsynonymous SNV	Pcdha8:NM_201243:exon1:c.T775A:p.S259T	**S259T**	ENSMUSP00000064333,259,S,T	18,36993241,T,A	18qB2	rs24886811	0.25	.	94	chr18	36993241	rs264484559	T	A	.	PASS	DB;ECNT=1;HCNT=1;MAX_ED=.;MIN_ED=.;NLOD=10.48;TLOD=64.39	GT:AD:AF:ALT_F1R2:ALT_F2R1:FOXOG:QSS:REF_F1R2:REF_F2R1	0/1:27,26:0.490:14:12:0.538:763,719:20:7	0/0:38,0:0.00:0:0:.:1036,0:16:22	4	Unkown	.	.	.	.	.	.	.	.
T4df	chr18	**1**	**12.5**	37475670	37475670	C	G	exonic	**Pcdhb15**	ENSMUSP00000131770	.	nonsynonymous SNV	Pcdhb15:NM_053140:exon1:c.C1954G:p.R652G	**R652G**	ENSMUSP00000131770,652,R,G	18,37475670,C,C	18qB3	.	0.25	.	67	chr18	37475670	.	C	C	.	str_contraction;t_lod_fstar	ECNT=1;HCNT=1;MAX_ED=.;MIN_ED=.;NLOD=9.61;RPA=7,6;RU=GGT;STR;TLOD=4.92	GT:AD:AF:ALT_F1R2:ALT_F2R1:FOXOG:QSS:REF_F1R2:REF_F2R1	0/1:48,2:0.042:2:0:.:1255,58:25:23	0/0:34,0:0.00:0:0:.:848,0:13:21	1.3	Tolerated	0	Neutral	392	30	1	Tolerated	3.33	395
T4df	chr18	**1**	**12.5**	37475671	37475671	G	T	exonic	**Pcdhb15**	ENSMUSP00000131770	.	nonsynonymous SNV	Pcdhb15:NM_053140:exon1:c.G1955T:p.R652L	**R652L**	ENSMUSP00000131770,652,R,L	18,37475671,G,G	18qB3	.	0.25	.	66	chr18	37475671	.	G	G	.	alt_allele_in_normal;clustered_events;t_lod_fstar	ECNT=21;HCNT=37;MAX_ED=213;MIN_ED=12;NLOD=11.47;TLOD=5.08	GT:AD:AF:ALT_F1R2:ALT_F2R1:FOXOG:PGT:PID:QSS:REF_F1R2:REF_F2R1	0/1:71,2:0.029:0:2:1.00:0|1:37309261_C_T:1981,56:35:36	0/0:72,4:0.029:0:4:1.00:0|1:37309261_C_T:1893,114:33:39	0.7	Tolerated	0	Neutral	392	30	1	Tolerated	3.33	395
T42df	chr18	**1**	**12.5**	37309509	37309509	G	A	exonic	**Pcdhb4**	ENSMUSP00000132166	.	nonsynonymous SNV	Pcdhb4:NM_053129:exon1:c.G1871A:p.R624H	**R624H**	ENSMUSP00000132166,624,R,H	18,37309509,G,A	18qB3	.	0.25	.	64	chr18	37309509	.	G	A	.	t_lod_fstar	ECNT=1;HCNT=2;MAX_ED=.;MIN_ED=.;NLOD=13.76;TLOD=4.28	GT:AD:AF:ALT_F1R2:ALT_F2R1:FOXOG:QSS:REF_F1R2:REF_F2R1	0/1:73,3:0.040:1:2:0.333:1917,84:38:35	0/0:63,1:0.018:1:0:1.00:1594,28:27:36	0.7	Deleterious	-3.94	Deleterious	388	30	0.052	Tolerated	3.38	388
T2df	chr18	**1**	**12.5**	37321427	37321427	A	G	exonic	**Pcdhb5**	ENSMUSP00000078589	.	nonsynonymous SNV	Pcdhb5:NM_053130:exon1:c.A859G:p.I287V	**I287V**	ENSMUSP00000078589,287,I,V	18,37321427,A,C	18qB3	.	0.25	.	83	chr18	37321427	.	A	C	.	alt_allele_in_normal;clustered_events	ECNT=2;HCNT=11;MAX_ED=3;MIN_ED=3;NLOD=2.90;TLOD=20.92	GT:AD:AF:ALT_F1R2:ALT_F2R1:FOXOG:PGT:PID:QSS:REF_F1R2:REF_F2R1	0/1:43,7:0.137:3:4:0.429:0|1:37321408_G_C:1175,189:21:22	0/0:90,6:0.066:2:4:0.333:0|1:37321408_G_C:2495,172:47:43	0.7	Damaging	-1.62	Neutral	392	30	0.029	Damaging	3.33	397
T2df	chr18	**1**	**12.5**	37321433	37321433	A	C	exonic	**Pcdhb5**	ENSMUSP00000078589	.	nonsynonymous SNV	Pcdhb5:NM_053130:exon1:c.A865C:p.K289Q	**K289Q**	ENSMUSP00000078589,289,K,Q	18,37321433,A,A	18qB3	.	0.25	.	83	chr18	37321433	.	A	A	.	PASS	ECNT=1;HCNT=8;MAX_ED=.;MIN_ED=.;NLOD=27.92;TLOD=10.35	GT:AD:AF:ALT_F1R2:ALT_F2R1:FOXOG:QSS:REF_F1R2:REF_F2R1	0/1:44,6:0.136:4:2:0.667:1247,154:24:20	0/0:101,0:0.00:0:0:.:2744,0:45:56	0.5	Tolerated	0	Neutral	392	30	1	Tolerated	3.34	398
T32df	chr18	**1**	**12.5**	37335216	37335216	G	A	exonic	**Pcdhb6**	ENSMUSP00000091135	.	nonsynonymous SNV	Pcdhb6:NM_053131:exon1:c.G1189A:p.E397K	**E397K**	ENSMUSP00000091135,397,E,K	18,37335216,G,T	18qB3	.	0.25	.	74	chr18	37335216	.	G	T	.	alt_allele_in_normal	ECNT=1;HCNT=2;MAX_ED=.;MIN_ED=.;NLOD=4.88;TLOD=26.00	GT:AD:AF:ALT_F1R2:ALT_F2R1:FOXOG:QSS:REF_F1R2:REF_F2R1	0/1:47,12:0.214:7:5:0.417:1332,335:24:23	0/0:41,2:0.053:0:2:1.00:1161,56:17:24	0.2	Unknown	NA	NA	388	30	NA	NA	NA	NA

T42df	chr18	**1**	**12.5**	37343414	37343414	A	C	exonic	**Pcdhb7**	ENSMUSP00000117225	.	nonsynonymous SNV	Pcdhb7:NM_053132:exon1:c.A1602C:p.L534F	**L534F**	ENSMUSP00000117225,534,L,F	18,37343414,A,A	18qB3	.	0.25	.	60	chr18	37343414	.	A	A	.	str_contraction;t_lod_fstar	ECNT=1;HCNT=8;MAX_ED=.;MIN_ED=.;NLOD=8.42;RPA=7,6;RU=CAG;STR;TLOD=4.61	GT:AD:AF:ALT_F1R2:ALT_F2R1:FOXOG:QSS:REF_F1R2:REF_F2R1	0/1:59,2:0.033:0:2:.:1662,57:28:31	0/0:31,0:0.00:0:0:.:872,0:20:11	18	Tolerated	0	Neutral	388	30	1	Tolerated	3.32	393
T22df	chr8	**1**	**12.5**	3465913	3465913	-	GGCATTGTTCCACTAGGACCCCACCGATCAGCTGGCAGCAAATT	exonic	**Pex11g**	ENSMUSP00000038264	.	stopgain	Pex11g:NM_026951:exon2:c.118_119insAATTTGCTGCCAGCTGATCGGTGGGGTCCTAGTGGAACAATGCC:p.P40_N41delinsQFAASX	**P40_N41delinsQFAASX**	ENSMUSP00000038264,40,PN,QFAASX	NA	8qA1.1	.	0.25	.	31	chr8	3465913	.	G	C	.	PASS	DB;ECNT=1;HCNT=1;MAX_ED=.;MIN_ED=.;NLOD=10.23;TLOD=47.80	GT:AD:AF:ALT_F1R2:ALT_F2R1:FOXOG:QSS:REF_F1R2:REF_F2R1	0/1:38,21:0.368:8:13:0.381:1056,544:23:15	0/0:37,0:0.00:0:0:.:1027,0:21:16	2	Deletarious	.	.	.	.	.	.	.	.
T1df	chr1	**1**	**12.5**	171356584	171356584	G	A	exonic	**Pfdn2**	ENSMUSP00000120128	.	nonsynonymous SNV	Pfdn2:NM_011070:exon2:c.G130A:p.A44T	**A44T**	ENSMUSP00000120128,44,A,T	1,171356584,G,C	1qH3	.	0.25	.	74	chr1	171356584	.	G	C	.	PASS	DB;ECNT=1;HCNT=1;MAX_ED=.;MIN_ED=.;NLOD=10.23;TLOD=47.80	GT:AD:AF:ALT_F1R2:ALT_F2R1:FOXOG:QSS:REF_F1R2:REF_F2R1	0/1:38,21:0.368:8:13:0.381:1056,544:23:15	0/0:37,0:0.00:0:0:.:1027,0:21:16	2	Deleterious	-2.66	Deleterious	56	30	0.005	Damaging	2.94	42
T2df	chr13	**1**	**12.5**	48804545	48804574	TGGTGGAGGCCGGGGTGGTGGATGCTGGGG	-	exonic	**Phf2**	ENSMUSP00000085172	.	nonframeshift deletion	Phf2:NM_011078:exon21:c.2963_2992del:p.988_998del	**988_998del**	NA	13,48804545,CTGGTGGAGGCCGGGGTGGTGGATGCTGGGG,A	13qA5	.	0.25	.	20	chr13	48804544	.	CTGGTGGAGGCCGGGGTGGTGGATGCTGGGG	A	.	PASS	ECNT=1;HCNT=6;MAX_ED=.;MIN_ED=.;NLOD=8.07;TLOD=13.51	GT:AD:AF:ALT_F1R2:ALT_F2R1:FOXOG:QSS:REF_F1R2:REF_F2R1	0/1:36,6:0.146:4:2:0.667:940,171:15:21	0/0:31,0:0.00:0:0:.:771,0:13:18	1.5	Unknown	.	.	.	.	.	.	.	.
T4df	chr1	**1**	**12.5**	106376176	106376176	T	C	exonic	**Phlpp1**	ENSMUSP00000021692	.	nonsynonymous SNV	Phlpp1:NM_133821:exon13:c.T3208C:p.C1070R	**C1070R**	ENSMUSP00000021692,1070,C,R	1,106376176,T,A	1qE2.1	.	0.25	.	36	chr1	106376176	.	T	A	.	alt_allele_in_normal;clustered_events	ECNT=9;HCNT=1;MAX_ED=25;MIN_ED=1;NLOD=5.43;TLOD=12.82	GT:AD:AF:ALT_F1R2:ALT_F2R1:FOXOG:PGT:PID:QSS:REF_F1R2:REF_F2R1	0/1:30,4:0.129:0:4:0.00:0|1:106376171_T_TC:859,61:16:14	0/0:32,1:0.030:0:1:0.00:0|1:106376171_T_TC:887,23:19:13	1.6	Deleterious	-3.88	Deleterious	123	30	0.554	Tolerated	2.78	236
T4df	chr1	**1**	**12.5**	106376179	106376179	-	TC	exonic	**Phlpp1**	ENSMUSP00000021692	.	frameshift insertion	Phlpp1:NM_133821:exon13:c.3211_3212insTC:p.N1071fs	**N1071fs**	ENSMUSP00000021692,1071,N,	1,106376179,A,T	1qE2.1	.	0.25	.	38	chr1	106376179	.	A	T	.	PASS	ECNT=1;HCNT=2;MAX_ED=.;MIN_ED=.;NLOD=9.88;TLOD=7.47	GT:AD:AF:ALT_F1R2:ALT_F2R1:FOXOG:QSS:REF_F1R2:REF_F2R1	0/1:36,4:0.105:3:1:0.250:1005,111:12:24	0/0:38,0:0.00:0:0:.:1060,0:19:19	2.4	Deleterious	-7.14	Deleterious	123	30	0	Damaging	2.78	236
T4df	chr1	**1**	**12.5**	106376173	106376173	A	C	exonic	**Phlpp1**	ENSMUSP00000021692	.	nonsynonymous SNV	Phlpp1:NM_133821:exon13:c.A3205C:p.S1069R	**S1069R**	ENSMUSP00000021692,1069,S,R	1,106376173,A,A	1qE2.1	.	0.25	.	37	chr1	106376173	.	A	A	.	clustered_events;multi_event_alt_allele_in_normal	ECNT=9;HCNT=1;MAX_ED=25;MIN_ED=1;NLOD=5.73;TLOD=12.88	GT:AD:AF:ALT_F1R2:ALT_F2R1:FOXOG:PGT:PID:QSS:REF_F1R2:REF_F2R1	0/1:28,4:0.133:0:4:0.00:0|1:106376171_T_TC:792,58:14:14	0/0:34,1:0.029:0:1:0.00:0|1:106376171_T_TC:944,24:21:13	1.6	Tolerated	0	Neutral	123	30	1	Tolerated	2.78	236
T4df	chr1	**1**	**12.5**	106376172	106376172	-	GTGGT	exonic	**Phlpp1**	ENSMUSP00000021692	.	frameshift insertion	Phlpp1:NM_133821:exon13:c.3204_3205insGTGGT:p.L1068fs	**L1068fs**	ENSMUSP00000021692,1068,L,	1,106376172,G,T	1qE2.1	.	0.25	.	38	chr1	106376172	.	G	T	.	clustered_events;t_lod_fstar	ECNT=2;HCNT=2;MAX_ED=4;MIN_ED=4;NLOD=11.74;TLOD=5.43	GT:AD:AF:ALT_F1R2:ALT_F2R1:FOXOG:PGT:PID:QSS:REF_F1R2:REF_F2R1	0/1:45,2:0.043:0:2:1.00:0|1:35945182_CT_C:1306,58:27:18	0/0:41,0:0.00:0:0:.:0|1:35945182_CT_C:1174,0:26:15	0.3	Tolerated	0	Neutral	123	30	1	Tolerated	2.78	236
T4df	chr1	**1**	**12.5**	106376191	106376191	G	A	exonic	**Phlpp1**	ENSMUSP00000021692	.	nonsynonymous SNV	Phlpp1:NM_133821:exon13:c.G3223A:p.E1075K	**E1075K**	ENSMUSP00000021692,1075,E,K	1,106376191,G,T	1qE2.1	.	0.25	.	35	chr1	106376191	.	G	T	.	clustered_events;multi_event_alt_allele_in_normal	ECNT=9;HCNT=1;MAX_ED=25;MIN_ED=1;NLOD=7.54;TLOD=12.50	GT:AD:AF:ALT_F1R2:ALT_F2R1:FOXOG:PGT:PID:QSS:REF_F1R2:REF_F2R1	0/1:30,4:0.108:0:4:.:0|1:106376171_T_TC:843,57:15:15	0/0:36,1:0.025:0:1:.:0|1:106376171_T_TC:1009,22:23:13	1.6	Unknown	NA	NA	123	30	NA	NA	NA	NA
T4df	chr1	**1**	**12.5**	106376195	106376195	T	A	exonic	**Phlpp1**	ENSMUSP00000021692	.	nonsynonymous SNV	Phlpp1:NM_133821:exon13:c.T3227A:p.I1076N	**I1076N**	ENSMUSP00000021692,1076,I,N	1,106376195,T,TC	1qE2.1	.	0.25	.	33	chr1	106376195	.	T	TC	.	clustered_events;multi_event_alt_allele_in_normal	ECNT=9;HCNT=1;MAX_ED=25;MIN_ED=1;NLOD=6.04;TLOD=12.71	GT:AD:AF:ALT_F1R2:ALT_F2R1:FOXOG:PGT:PID:QSS:REF_F1R2:REF_F2R1	0/1:29,4:0.121:0:4:.:0|1:106376171_T_TC:855,116:16:13	0/0:37,1:0.029:0:1:.:0|1:106376171_T_TC:858,28:25:12	1.6	Unknown	NA	NA	123	30	NA	NA	NA	NA

T4df	chr1	**1**	**12.5**	106376171	106376171	-	C	exonic	**Phlpp1**	ENSMUSP00000021692	.	frameshift insertion	Phlpp1:NM_133821:exon13:c.3203_3204insC:p.L1068fs	**L1068fs**	ENSMUSP00000021692,1068,L,	1,106376171,T,ATC	1qE2.1	.	0.25	.	38	chr1	106376171	.	T	ATC	.	clustered_events;multi_event_alt_allele_in_normal	ECNT=9;HCNT=1;MAX_ED=25;MIN_ED=1;NLOD=6.04;TLOD=12.71	GT:AD:AF:ALT_F1R2:ALT_F2R1:FOXOG:PGT:PID:QSS:REF_F1R2:REF_F2R1	0/1:30,4:0.121:0:4:.:0|1:106376171_T_TC:796,42:16:14	0/0:37,1:0.029:0:1:.:0|1:106376171_T_TC:1007,12:24:13	1.6	Unknown	NA	NA	123	30	NA	NA	NA	NA
T4df	chr1	**1**	**12.5**	106376182	106376189	GAGCTGAG	-	exonic	**Phlpp1**	ENSMUSP00000021692	.	frameshift deletion	Phlpp1:NM_133821:exon13:c.3214_3221del:p.E1072fs	**E1072fs**	ENSMUSP00000021692,1072,E,.	1,106376182,TGAGCTGAG,GGTGGT	1qE2.1	.	0.25	.	37	chr1	106376181	.	TGAGCTGAG	GGTGGT	.	clustered_events;multi_event_alt_allele_in_normal	ECNT=9;HCNT=1;MAX_ED=25;MIN_ED=1;NLOD=6.04;TLOD=12.71	GT:AD:AF:ALT_F1R2:ALT_F2R1:FOXOG:PGT:PID:QSS:REF_F1R2:REF_F2R1	0/1:29,4:0.121:0:4:.:0|1:106376171_T_TC:846,53:16:13	0/0:37,1:0.029:0:1:.:0|1:106376171_T_TC:1048,12:25:12	1.6	Unknown	.	.	.	.	.	.	.	.
T32df	chr5	**1**	**12.5**	20782398	20782398	C	T	exonic	**Phtf2**	ENSMUSP00000069127	.	nonsynonymous SNV	Phtf2:NM_172992:exon10:c.G1099A:p.V367I	**V367I**	ENSMUSP00000069127,367,V,I	5,20782398,C,T	5qA3	.	0.25	.	42	chr5	20782398	.	C	T	.	t_lod_fstar	ECNT=1;HCNT=1;MAX_ED=.;MIN_ED=.;NLOD=9.27;TLOD=4.32	GT:AD:AF:ALT_F1R2:ALT_F2R1:FOXOG:QSS:REF_F1R2:REF_F2R1	0/1:73,3:0.041:3:0:0.00:1875,83:39:34	0/0:37,0:0.00:0:0:.:904,0:22:15	1.2	Tolerated	-0.25	Neutral	154	30	0.142	Tolerated	2.97	116
T1df	chr8	**1**	**12.5**	122502299	122502299	C	T	exonic	**Piezo1**	ENSMUSP00000027493	.	nonsynonymous SNV	Piezo1:NM_001037298:exon6:c.G475A:p.D159N,Piezo1:NM_001357349:exon6:c.G478A:p.D160N	**D159N**	ENSMUSP00000027493,159,D,N	8,122502299,C,G	8qE1	.	0.25	.	37	chr8	122502299	.	C	G	.	t_lod_fstar	ECNT=2;HCNT=2;MAX_ED=2;MIN_ED=2;NLOD=12.04;TLOD=5.65	GT:AD:AF:ALT_F1R2:ALT_F2R1:FOXOG:PGT:PID:QSS:REF_F1R2:REF_F2R1	0/1:34,2:0.056:0:2:1.00:0|1:133071209_T_TTG:949,58:17:17	0/0:44,0:0.00:0:0:.:0|1:133071209_T_TTG:1177,0:21:23	24	Damaging	-2.35	Neutral	89	30	0.04	Damaging	2.95	71
T1df	chr8	**1**	**12.5**	122502299	122502299	C	T	exonic	**Piezo1**	ENSMUSP00000027493	.	nonsynonymous SNV	Piezo1:NM_001037298:exon6:c.G475A:p.D159N,Piezo1:NM_001357349:exon6:c.G478A:p.D160N	**D160N**	ENSMUSP00000027493,160,D,N	8,122502299,C,TTG	8qE1	.	0.25	.	37	chr8	122502299	.	C	TTG	.	t_lod_fstar	ECNT=2;HCNT=2;MAX_ED=2;MIN_ED=2;NLOD=12.04;TLOD=5.65	GT:AD:AF:ALT_F1R2:ALT_F2R1:FOXOG:PGT:PID:QSS:REF_F1R2:REF_F2R1	0/1:33,2:0.056:0:2:.:0|1:133071209_T_TTG:950,56:17:16	0/0:43,0:0.00:0:0:.:0|1:133071209_T_TTG:1181,0:21:22	24	Unknown	NA	NA	87	30	NA	NA	NA	NA
T32df	chr1	**1**	**12.5**	133071211	133071211	A	G	exonic	**Pik3c2b**	ENSMUSP00000141989	.	nonsynonymous SNV	Pik3c2b:NM_001099276:exon4:c.A1069G:p.T357A	**T357A**	ENSMUSP00000141989,357,T,A	1,133071211,A,G	1qE4	.	0.25	.	44	chr1	133071211	.	A	G	.	t_lod_fstar	ECNT=2;HCNT=2;MAX_ED=2;MIN_ED=2;NLOD=12.04;TLOD=5.65	GT:AD:AF:ALT_F1R2:ALT_F2R1:FOXOG:PGT:PID:QSS:REF_F1R2:REF_F2R1	0/1:34,2:0.056:0:2:1.00:0|1:133071209_T_TTG:949,58:17:17	0/0:44,0:0.00:0:0:.:0|1:133071209_T_TTG:1177,0:21:23	24	Tolerated	-1.28	Neutral	155	30	0.206	Tolerated	2.92	119
T32df	chr1	**1**	**12.5**	133071209	133071209	-	TG	exonic	**Pik3c2b**	ENSMUSP00000141989	.	frameshift insertion	Pik3c2b:NM_001099276:exon4:c.1067_1068insTG:p.L356fs	**L356fs**	ENSMUSP00000141989,356,L,	1,133071209,T,G	1qE4	.	0.25	.	43	chr1	133071209	.	T	G	.	alt_allele_in_normal;germline_risk;t_lod_fstar	ECNT=1;HCNT=2;MAX_ED=.;MIN_ED=.;NLOD=2.08;TLOD=6.15	GT:AD:AF:ALT_F1R2:ALT_F2R1:FOXOG:QSS:REF_F1R2:REF_F2R1	0/1:44,8:0.140:4:4:0.500:1093,158:24:20	0/0:28,6:0.188:2:4:0.333:711,112:12:16	0.7	Tolerated	0.12	Neutral	155	30	0.069	Tolerated	2.94	118
T3df	chr8	**1**	**12.5**	109623617	109623617	T	G	exonic	**Pkd1l3**	ENSMUSP00000054111	.	nonsynonymous SNV	Pkd1l3:NM_001039700:exon5:c.T1093G:p.S365A,Pkd1l3:NM_001286454:exon5:c.T1093G:p.S365A,Pkd1l3:NM_181544:exon5:c.T1093G:p.S365A	**S365A**	ENSMUSP00000054111,365,S,A	8,109623617,T,T	8qD3	.	0.25	.	34	chr8	109623617	.	T	T	.	PASS	ECNT=1;HCNT=2;MAX_ED=.;MIN_ED=.;NLOD=12.28;TLOD=6.78	GT:AD:AF:ALT_F1R2:ALT_F2R1:FOXOG:QSS:REF_F1R2:REF_F2R1	0/1:81,4:0.036:0:4:.:2174,114:48:33	0/0:43,0:0.00:0:0:.:1075,0:18:25	1.4	Tolerated	0	Neutral	63	30	1	Tolerated	4.32	6
T22df	chr1	**1**	**12.5**	135877103	135877103	C	G	exonic	**Pkp1**	ENSMUSP00000077389	.	nonsynonymous SNV	Pkp1:NM_001313701:exon12:c.G2080C:p.A694P,Pkp1:NM_019645:exon12:c.G2080C:p.A694P	**A694P**	ENSMUSP00000077389,694,A,P	NA	1qE4	.	0.25	.	46	chr1	135877103	.	C	G	.	str_contraction;t_lod_fstar	ECNT=1;HCNT=1;MAX_ED=.;MIN_ED=.;NLOD=8.09;RPA=9,8;RU=GAA;STR;TLOD=6.17	GT:AD:AF:ALT_F1R2:ALT_F2R1:FOXOG:QSS:REF_F1R2:REF_F2R1	0/1:25,3:0.107:2:1:.:701,85:13:12	0/0:29,0:0.00:0:0:.:840,0:19:10	0.4	Deleterious	-4.55	Deleterious	392	30	0.001	Damaging	3.34	397
T4df	chr10	**1**	**12.5**	13127900	13127971	TGGAGCCGATGCAGCCTTTGGAGCCAATGCAGCCTTTGGAGCCGATGCAGCCTTTAGAGCCTTTGGAGCCTC	-	exonic	**Plagl1**	ENSMUSP00000030691	.	nonframeshift deletion	Plagl1:NM_009538:exon6:c.911_982del:p.304_328del	**304_328del**	NA	10,13127900,TTGGAGCCGATGCAGCCTTTGGAGCCAATGCAGCCTTTGGAGCCGATGCAGCCTTTAGAGCCTTTGGAGCCTC,T	10qA2	.	0.25	.	43	chr10	13127899	.	TTGGAGCCGATGCAGCCTTTGGAGCCAATGCAGCCTTTGGAGCCGATGCAGCCTTTAGAGCCTTTGGAGCCTC	T	.	t_lod_fstar	ECNT=1;HCNT=2;MAX_ED=.;MIN_ED=.;NLOD=8.07;TLOD=4.49	GT:AD:AF:ALT_F1R2:ALT_F2R1:FOXOG:QSS:REF_F1R2:REF_F2R1	0/1:69,3:0.047:2:1:0.667:1763,83:29:40	0/0:31,0:0.00:0:0:.:762,0:12:19	1.1	Unknown	.	.	.	.	.	.	.	.

T42df	chr1	**1**	**12.5**	74560350	74560352	GAA	-	exonic	**Plcd4**	ENSMUSP00000004686	.	nonframeshift deletion	Plcd4:NM_001081456:exon10:c.1333_1335del:p.445_445del,Plcd4:NM_148937:exon10:c.1333_1335del:p.445_445del	**445_445del**	NA	1,74560350,GGAA,A	1qC3	.	0.25	.	29	chr1	74560349	.	GGAA	A	.	clustered_events;homologous_mapping_event	ECNT=4;HCNT=2;MAX_ED=8;MIN_ED=1;NLOD=10.84;TLOD=9.82	GT:AD:AF:ALT_F1R2:ALT_F2R1:FOXOG:PGT:PID:QSS:REF_F1R2:REF_F2R1	0/1:65,3:0.058:0:3:0.00:0|1:35928837_C_A:1569,79:36:29	0/0:38,0:0.00:0:0:.:0|1:35928837_C_A:947,0:15:23	0.9	Unknown	.	.	.	.	.	.	.	.
T12df	chr17	**1**	**12.5**	12395643	12395643	G	C	exonic	**Plg**	ENSMUSP00000018383	.	nonsynonymous SNV	Plg:NM_008877:exon8:c.G809C:p.S270T	**S270T**	ENSMUSP00000018383,270,S,T	17,12395643,G,C	17qA1	.	0.25	.	20	chr17	12395643	.	G	C	.	str_contraction;t_lod_fstar	ECNT=1;HCNT=1;MAX_ED=.;MIN_ED=.;NLOD=9.61;RPA=6,5;RU=CAG;STR;TLOD=5.41	GT:AD:AF:ALT_F1R2:ALT_F2R1:FOXOG:QSS:REF_F1R2:REF_F2R1	0/1:47,2:0.043:2:0:.:1303,56:23:24	0/0:36,0:0.00:0:0:.:985,0:20:16	6	Tolerated	-0.75	Neutral	154	30	0.57	Tolerated	3.08	74
T12df	chr17	**1**	**12.5**	12395642	12395642	A	C	exonic	**Plg**	ENSMUSP00000018383	.	nonsynonymous SNV	Plg:NM_008877:exon8:c.A808C:p.S270R	**S270R**	ENSMUSP00000018383,270,S,R	17,12395642,A,CATCT	17qA1	.	0.25	.	19	chr17	12395642	.	A	CATCT	.	t_lod_fstar	ECNT=1;HCNT=83;MAX_ED=.;MIN_ED=.;NLOD=10.30;TLOD=5.43	GT:AD:AF:ALT_F1R2:ALT_F2R1:FOXOG:QSS:REF_F1R2:REF_F2R1	0/1:46,2:0.043:0:2:.:1332,57:26:20	0/0:48,1:0.021:0:1:.:1368,21:28:20	1.1	Unknown	NA	NA	154	30	NA	NA	NA	NA
T1df	chr3	**1**	**12.5**	54370258	54370258	G	T	exonic	**Postn**	ENSMUSP00000123911	.	nonsynonymous SNV	Postn:NM_001198765:exon6:c.G673T:p.V225F,Postn:NM_001198766:exon6:c.G673T:p.V225F,Postn:NM_001313898:exon6:c.G673T:p.V225F,Postn:NM_001313899:exon6:c.G673T:p.V225F,Postn:NM_015784:exon6:c.G673T:p.V225F	**V225F**	ENSMUSP00000123911,225,V,F	3,54370258,G,A	3qC	.	0.25	.	31	chr3	54370258	.	G	A	.	clustered_events;homologous_mapping_event;t_lod_fstar	ECNT=4;HCNT=2;MAX_ED=8;MIN_ED=1;NLOD=10.23;TLOD=5.11	GT:AD:AF:ALT_F1R2:ALT_F2R1:FOXOG:PGT:PID:QSS:REF_F1R2:REF_F2R1	0/1:66,2:0.030:0:2:0.00:0|1:35928837_C_A:1856,58:37:29	0/0:37,0:0.00:0:0:.:0|1:35928837_C_A:1017,0:14:23	0.9	Tolerated	-0.63	Neutral	225	30	0.052	Tolerated	2.97	214
T4df	chr17	**1**	**12.5**	35930859	35930859	G	A	exonic	**Ppp1r10**	ENSMUSP00000125030	.	nonsynonymous SNV	Ppp1r10:NM_001163818:exon18:c.G2525A:p.R842Q	**R842Q**	ENSMUSP00000125030,842,R,Q	17,35930859,G,A	17qB1	.	0.25	.	31	chr17	35930859	.	G	A	.	PASS	DB;ECNT=1;HCNT=1;MAX_ED=.;MIN_ED=.;NLOD=5.97;TLOD=45.58	GT:AD:AF:ALT_F1R2:ALT_F2R1:FOXOG:QSS:REF_F1R2:REF_F2R1	0/1:17,19:0.500:6:13:0.316:444,536:7:10	0/0:21,0:0.00:0:0:.:549,0:10:11	1.1	Damaging	-1.11	Neutral	84	30	0	Damaging	3.91	22
T42df	chr17	**1**	**12.5**	35931166	35931166	G	C	exonic	**Ppp1r10**	ENSMUSP00000125030	.	nonsynonymous SNV	Ppp1r10:NM_001163818:exon19:c.G2575C:p.V859L	**V859L**	ENSMUSP00000125030,859,V,L	17,35931166,G,A	17qB1	.	0.25	.	27	chr17	35931166	.	G	A	.	clustered_events	DB;ECNT=2;HCNT=2;MAX_ED=43;MIN_ED=43;NLOD=6.92;TLOD=59.30	GT:AD:AF:ALT_F1R2:ALT_F2R1:FOXOG:QSS:REF_F1R2:REF_F2R1	0/1:34,28:0.436:15:13:0.536:933,755:18:16	0/0:25,0:0.00:0:0:.:698,0:11:14	1.1	Damaging	-0.3	Neutral	84	30	0.019	Damaging	3.67	28
T42df	chr17	**1**	**12.5**	35930825	35930825	C	T	exonic	**Ppp1r10**	ENSMUSP00000125030	.	nonsynonymous SNV	Ppp1r10:NM_001163818:exon18:c.C2491T:p.R831C	**R831C**	ENSMUSP00000125030,831,R,C	17,35930825,C,A	17qB1	.	0.25	.	26	chr17	35930825	.	C	A	.	alt_allele_in_normal;clustered_events	ECNT=3;HCNT=24;MAX_ED=22;MIN_ED=3;NLOD=4.75;TLOD=16.97	GT:AD:AF:ALT_F1R2:ALT_F2R1:FOXOG:PGT:PID:QSS:REF_F1R2:REF_F2R1	0/1:54,7:0.117:4:3:0.571:0|1:35930837_G_A:1387,197:32:22	0/0:29,2:0.067:2:0:1.00:0|1:35930837_G_A:746,45:18:11	0.9	Tolerated	-0.85	Neutral	84	30	0.091	Tolerated	4.32	21
T2df	chr4	**1**	**12.5**	143948805	143948805	C	A	exonic	**Pramef25**	ENSMUSP00000130089	.	nonsynonymous SNV	Pramef25:NM_001126315:exon3:c.G1450T:p.G484C	**G484C**	ENSMUSP00000130089,484,G,C	4,143948805,C,T	4qE1	.	0.25	.	83	chr4	143948805	.	C	T	.	clustered_events;homologous_mapping_event	DB;ECNT=3;HCNT=4;MAX_ED=162;MIN_ED=80;NLOD=12.31;TLOD=51.53	GT:AD:AF:ALT_F1R2:ALT_F2R1:FOXOG:QSS:REF_F1R2:REF_F2R1	0/1:19,20:0.514:12:8:0.400:473,566:11:8	0/0:47,0:0.00:0:0:.:1155,0:17:30	0.9	Tolerated	-0.34	Neutral	203	30	0.24	Tolerated	2.77	121
T42df	chr1	**1**	**12.5**	150455893	150455893	A	T	exonic	**Prg4**	ENSMUSP00000047308	.	nonsynonymous SNV	Prg4:NM_021400:exon7:c.T1028A:p.V343D	**V343D**	ENSMUSP00000047308,343,V,D	1,150455893,A,A	1qG1	.	0.25	.	29	chr1	150455893	.	A	A	.	t_lod_fstar	ECNT=1;HCNT=1;MAX_ED=.;MIN_ED=.;NLOD=6.01;TLOD=5.45	GT:AD:AF:ALT_F1R2:ALT_F2R1:FOXOG:QSS:REF_F1R2:REF_F2R1	0/1:46,2:0.044:0:2:.:1286,58:25:21	0/0:22,0:0.00:0:0:.:566,0:14:8	2.1	Tolerated	0	Neutral	50	30	0.362	Tolerated	3.28	59
T12df	chr17	**1**	**12.5**	35158130	35158132	TGC	-	exonic	**Prrc2a**	ENSMUSP00000038497	.	nonframeshift deletion	Prrc2a:NM_001199044:exon14:c.2001_2003del:p.667_668del,Prrc2a:NM_020027:exon14:c.2001_2003del:p.667_668del	**667_668del**	NA	17,35158130,TTGC,G	17qB1	.	0.25	.	33	chr17	35158129	.	TTGC	G	.	t_lod_fstar	ECNT=1;HCNT=1;MAX_ED=.;MIN_ED=.;NLOD=14.39;TLOD=5.19	GT:AD:AF:ALT_F1R2:ALT_F2R1:FOXOG:QSS:REF_F1R2:REF_F2R1	0/1:53,3:0.059:2:1:0.667:1489,87:25:28	0/0:53,0:0.00:0:0:.:1456,0:25:28	1.8	Unknown	.	.	.	.	.	.	.	.

T1df	chr5	**1**	**12.5**	21799495	21799495	C	A	exonic	**Psmc2**	ENSMUSP00000076157	.	nonsynonymous SNV	Psmc2:NM_011188:exon6:c.C489A:p.F163L	**F163L**	ENSMUSP00000076157,163,F,L	5,21799495,C,A	5qA3	.	0.25	.	38	chr5	21799495	.	C	A	.	PASS	DB;ECNT=1;HCNT=1;MAX_ED=.;MIN_ED=.;NLOD=10.48;TLOD=64.39	GT:AD:AF:ALT_F1R2:ALT_F2R1:FOXOG:QSS:REF_F1R2:REF_F2R1	0/1:27,26:0.490:14:12:0.538:763,719:20:7	0/0:38,0:0.00:0:0:.:1036,0:16:22	4	Deleterious	-5.21	Deleterious	343	30	0.001	Damaging	3.37	356
T1df	chr5	**1**	**12.5**	21799494	21799494	T	G	exonic	**Psmc2**	ENSMUSP00000076157	.	nonsynonymous SNV	Psmc2:NM_011188:exon6:c.T488G:p.F163C	**F163C**	ENSMUSP00000076157,163,F,C	5,21799494,T,A	5qA3	.	0.25	.	39	chr5	21799494	.	T	A	.	PASS	DB;ECNT=1;HCNT=1;MAX_ED=.;MIN_ED=.;NLOD=10.48;TLOD=64.39	GT:AD:AF:ALT_F1R2:ALT_F2R1:FOXOG:QSS:REF_F1R2:REF_F2R1	0/1:27,26:0.490:14:12:0.538:763,719:20:7	0/0:38,0:0.00:0:0:.:1036,0:16:22	4	Tolerated	-1.85	Neutral	343	30	0.21	Tolerated	3.37	356
T4df	chr5	**1**	**12.5**	139824007	139824008	CG	-	exonic	**Psmg3**	ENSMUSP00000124032	.	frameshift deletion	Psmg3:NM_001356963:exon3:c.282_283del:p.A94fs,Psmg3:NM_025604:exon3:c.282_283del:p.A94fs	**A94fs**	ENSMUSP00000124032,94,A,.	5,139824007,ACG,G	5qG2	.	0.25	.	22	chr5	139824006	.	ACG	G	.	t_lod_fstar	ECNT=1;HCNT=1;MAX_ED=.;MIN_ED=.;NLOD=14.39;TLOD=5.19	GT:AD:AF:ALT_F1R2:ALT_F2R1:FOXOG:QSS:REF_F1R2:REF_F2R1	0/1:53,3:0.059:2:1:0.667:1489,87:25:28	0/0:53,0:0.00:0:0:.:1456,0:25:28	1.8	Unknown	NA	NA	77	30	NA	NA	NA	NA
T12df	chr17	**1**	**12.5**	56999521	56999521	-	CAAGGAAGGTCACATCAGCATAGCTGATGTGACCTTCCTTGATGATCAG	exonic	**Pspn**	ENSMUSP00000152962	.	stopgain	Pspn:NM_008954:exon2:c.406_407insCTGATCATCAAGGAAGGTCACATCAGCTATGCTGATGTGACCTTCCTTG:p.D136_D137delinsADHQGRSHQLCX	**D136_D137delinsADHQGRSHQLCX**	ENSMUSP00000152962,136,D,D,ADHQGRSHQLCX	NA	17qD	.	0.25	.	26	chr17	56999521	.	T	CT	.	t_lod_fstar	ECNT=1;HCNT=4;MAX_ED=.;MIN_ED=.;NLOD=14.75;TLOD=4.08	GT:AD:AF:ALT_F1R2:ALT_F2R1:FOXOG:QSS:REF_F1R2:REF_F2R1	0/1:49,2:0.043:0:2:.:1361,48:26:23	0/0:55,0:0.00:0:0:.:1511,0:22:33	2.2	Deletarious	.	.	.	.	.	.	.	.
T42df	chr17	**1**	**12.5**	42502849	42502849	T	G	exonic	**Ptchd4**	ENSMUSP00000124238	.	nonsynonymous SNV	Ptchd4:NM_001355270:exon3:c.T1649G:p.I550S,Ptchd4:NM_028474:exon4:c.T1640G:p.I547S	**I547S**	ENSMUSP00000124238,547,I,S	17,42502849,T,G	17qB3	.	0.25	.	53	chr17	42502849	.	T	G	.	alt_allele_in_normal;clustered_events	ECNT=2;HCNT=3;MAX_ED=31;MIN_ED=31;NLOD=3.82;TLOD=8.41	GT:AD:AF:ALT_F1R2:ALT_F2R1:FOXOG:QSS:REF_F1R2:REF_F2R1	0/1:45,5:0.102:0:5:0.00:1142,130:24:21	0/0:41,3:0.079:0:3:0.00:1122,69:27:14	3	Tolerated	2.42	Neutral	164	30	0.628	Tolerated	2.86	387
T42df	chr17	**1**	**12.5**	42502849	42502849	T	G	exonic	**Ptchd4**	ENSMUSP00000124238	.	nonsynonymous SNV	Ptchd4:NM_001355270:exon3:c.T1649G:p.I550S,Ptchd4:NM_028474:exon4:c.T1640G:p.I547S	**I550S**	ENSMUSP00000124238,550,I,S	17,42502849,T,CTGGACGGCTTTACTAAGTGACCATCAGCTGCATCTCTGGTGAGCTCTCTGCTGAGAAGTCAGCAGCGGGGAGAACAGGTGA	17qB3	.	0.25	.	53	chr17	42502849	.	T	CTGGACGGCTTTACTAAGTGACCATCAGCTGCATCTCTGGTGAGCTCTCTGCTGAGAAGTCAGCAGCGGGGAGAACAGGTGA	.	t_lod_fstar	ECNT=1;HCNT=1;MAX_ED=.;MIN_ED=.;NLOD=11.80;TLOD=6.21	GT:AD:AF:ALT_F1R2:ALT_F2R1:FOXOG:QSS:REF_F1R2:REF_F2R1	0/1:37,1:0.071:0:1:.:1054,29:17:20	0/0:47,0:0.00:0:0:.:1321,0:20:27	4	Unknown	NA	NA	164	30	NA	NA	NA	NA
T42df	chr10	**1**	**12.5**	116317552	116317552	-	TGGACGGCTTTACTAAGTGACCATCAGCTGCATCTCTGGTGAGCTCTCTGCTGAGAAGTCAGCAGCGGGGAGAACAGGTGA	exonic	**Ptprb**	ENSMUSP00000124845	.	stopgain	Ptprb:NM_029928:exon5:c.1052_1053insTGGACGGCTTTACTAAGTGACCATCAGCTGCATCTCTGGTGAGCTCTCTGCTGAGAAGTCAGCAGCGGGGAGAACAGGTGA:p.P351delinsPGRLYX	**P351delinsPGRLYX**	ENSMUSP00000124845,351,P,PGRLYX	NA	10qD2	.	0.25	.	47	chr10	116317552	.	C	TAAA	.	alt_allele_in_normal;clustered_events;t_lod_fstar	ECNT=2;HCNT=2;MAX_ED=31;MIN_ED=31;NLOD=7.77;RPA=1,4;RU=A;STR;TLOD=5.35	GT:AD:AF:ALT_F1R2:ALT_F2R1:FOXOG:QSS:REF_F1R2:REF_F2R1	0/1:42,5:0.106:0:5:.:1128,94:26:16	0/0:44,2:0.050:0:2:.:1194,46:28:16	3	Deletarious	.	.	.	.	.	.	.	.
T2df	chr17	**1**	**12.5**	66698470	66698470	-	AAA	exonic	**Ptprm**	ENSMUSP00000056530	.	nonframeshift insertion	Ptprm:NM_008984:exon22:c.3027_3028insTTT:p.K1010delinsFK	**K1010delinsFK**	ENSMUSP00000056530,1010,K,FK	17,66698470,T,G	17qE1.1	.	0.25	.	46	chr17	66698470	.	T	G	.	t_lod_fstar	ECNT=1;HCNT=2;MAX_ED=.;MIN_ED=.;NLOD=9.33;TLOD=5.36	GT:AD:AF:ALT_F1R2:ALT_F2R1:FOXOG:QSS:REF_F1R2:REF_F2R1	0/1:56,2:0.047:0:2:1.00:1580,46:32:24	0/0:37,0:0.00:0:0:.:1001,0:12:25	1.8	Tolerated	-1.76	Neutral	281	30	0.409	Tolerated	2.9	253
T4df	chr6	**1**	**12.5**	137416841	137416841	C	G	exonic	**Ptpro**	ENSMUSP00000089777	.	nonsynonymous SNV	Ptpro:NM_001164402:exon4:c.C43G:p.L15V,Ptpro:NM_001164403:exon4:c.C43G:p.L15V,Ptpro:NM_001164401:exon15:c.C2506G:p.L836V,Ptpro:NM_011216:exon15:c.C2506G:p.L836V	**L15V**	ENSMUSP00000089777,15,L,V	6,137416841,C,T	6qG1	.	0.25	.	37	chr6	137416841	.	C	T	.	PASS	ECNT=1;HCNT=2;MAX_ED=.;MIN_ED=.;NLOD=14.07;TLOD=39.12	GT:AD:AF:ALT_F1R2:ALT_F2R1:FOXOG:QSS:REF_F1R2:REF_F2R1	0/1:36,16:0.327:12:4:0.250:901,449:17:19	0/0:52,0:0.00:0:0:.:1276,0:30:22	10	Tolerated	-1.47	Neutral	87	30	0.07	Tolerated	3.14	32
T4df	chr6	**1**	**12.5**	137416841	137416841	C	G	exonic	**Ptpro**	ENSMUSP00000089777	.	nonsynonymous SNV	Ptpro:NM_001164402:exon4:c.C43G:p.L15V,Ptpro:NM_001164403:exon4:c.C43G:p.L15V,Ptpro:NM_001164401:exon15:c.C2506G:p.L836V,Ptpro:NM_011216:exon15:c.C2506G:p.L836V	**L836V**	ENSMUSP00000089777,836,L,V	6,137416841,C,T	6qG1	.	0.25	.	37	chr6	137416841	.	C	T	.	PASS	ECNT=1;HCNT=1;MAX_ED=.;MIN_ED=.;NLOD=14.95;TLOD=39.08	GT:AD:AF:ALT_F1R2:ALT_F2R1:FOXOG:QSS:REF_F1R2:REF_F2R1	0/1:30,19:0.370:11:8:0.421:865,513:16:14	0/0:52,0:0.00:0:0:.:1455,0:32:20	10	Tolerated	0	Neutral	176	30	1	Tolerated	3.24	93

T32df	chr15	**1**	**12.5**	62259057	62259057	G	T	ncRNA_exonic	**Pvt1**	NONE	.	.	.	**NA**	15,62259057,G,A	NA	15qD1	.	0.25	.	46	chr15	62259057	.	G	A	.	alt_allele_in_normal;t_lod_fstar	ECNT=1;HCNT=1;MAX_ED=.;MIN_ED=.;NLOD=7.51;TLOD=6.11	GT:AD:AF:ALT_F1R2:ALT_F2R1:FOXOG:QSS:REF_F1R2:REF_F2R1	0/1:52,5:0.089:5:0:1.00:1397,135:24:28	0/0:41,2:0.051:0:2:0.00:1095,55:23:18	0.5	Unkown	.	.	.	.	.	.	.	.
T42df	chr6	**1**	**12.5**	142353550	142353550	-	T	exonic	**Pyroxd1**	ENSMUSP00000153159	.	frameshift insertion	Pyroxd1:NM_183165:exon5:c.431_432insT:p.A144fs	**A144fs**	ENSMUSP00000153159,144,A,	6,142353550,C,A	6qG2	.	0.25	.	55	chr6	142353550	.	C	A	.	PASS	ECNT=1;HCNT=1;MAX_ED=.;MIN_ED=.;NLOD=10.53;TLOD=6.45	GT:AD:AF:ALT_F1R2:ALT_F2R1:FOXOG:QSS:REF_F1R2:REF_F2R1	0/1:74,4:0.053:2:2:0.500:2072,113:41:33	0/0:42,0:0.00:0:0:.:1181,0:26:16	5	Deleterious	-4.63	Deleterious		93	30	0.001	Damaging	2.79	83
T1df	chr2	**1**	**12.5**	37495651	37495652	AG	-	exonic	**Rabgap1**	ENSMUSP00000027667	.	frameshift deletion	Rabgap1:NM_001033960:exon11:c.1428_1429del:p.S476fs,Rabgap1:NM_146121:exon11:c.1428_1429del:p.S476fs	**S476fs**	ENSMUSP00000027667,476,S,.	2,37495651,CAG,AACGGCAGTGTCTCTGCAGGTTCATCTGTTCTCATCG	2qB	.	0.25	.	47	chr2	37495650	.	CAG	AACGGCAGTGTCTCTGCAGGTTCATCTGTTCTCATCG	.	germline_risk	ECNT=1;HCNT=1;MAX_ED=.;MIN_ED=.;NLOD=1.83;RPA=3,4;RU=ACGGCAGTGTCTCTGCAGGTTCATCTGTTCTCATCG;STR;TLOD=32.56	GT:AD:AF:ALT_F1R2:ALT_F2R1:FOXOG:QSS:REF_F1R2:REF_F2R1	0/1:0,2:1.00:1:1:.:0,39:0:0	0/0:0,0:NaN:0:0:.:0,0:0:0	2.2	Unknown	NA	NA	164	30	NA	NA	NA	NA
T1df	chr4	**1**	**12.5**	55385479	55385493	GGTGGAGGAGGTGGT	-	exonic	**Rad23b**	ENSMUSP00000153221	.	nonframeshift deletion	Rad23b:NM_009011:exon9:c.1015_1029del:p.339_343del	**339_343del**	NA	4,55385479,AGGTGGAGGAGGTGGT,GA	4qB3	.	0.25	.	42	chr4	55385478	.	AGGTGGAGGAGGTGGT	GA	.	t_lod_fstar	ECNT=2;HCNT=3;MAX_ED=2;MIN_ED=2;NLOD=9.94;TLOD=5.34	GT:AD:AF:ALT_F1R2:ALT_F2R1:FOXOG:PGT:PID:QSS:REF_F1R2:REF_F2R1	0/1:52,2:0.039:0:2:.:0|1:36757876_G_GA:1470,58:29:23	0/0:37,0:0.00:0:0:.:0|1:36757876_G_GA:1012,0:15:22	0.9	Unknown	NA	NA	166	30	NA	NA	NA	NA
T4df	chr11	**1**	**12.5**	101246546	101246548	TGC	-	exonic	**Ramp2**	ENSMUSP00000014578	.	nonframeshift deletion	Ramp2:NM_019444:exon1:c.95_97del:p.32_33del	**32_33del**	NA	11,101246546,TTGC,GA	11qD	.	0.25	.	31	chr11	101246545	.	TTGC	GA	.	alt_allele_in_normal;clustered_events	ECNT=6;HCNT=1;MAX_ED=23;MIN_ED=3;NLOD=7.26;TLOD=11.58	GT:AD:AF:ALT_F1R2:ALT_F2R1:FOXOG:PGT:PID:QSS:REF_F1R2:REF_F2R1	0/1:59,4:0.066:3:1:.:0|1:129020257_G_A:1589,109:33:26	0/0:57,2:0.037:2:0:.:0|1:129020257_G_A:1499,53:34:23	0.5	Unknown	NA	NA	34	9	NA	NA	NA	NA
T2df	chr5	**1**	**12.5**	129020279	129020279	-	ATCTGCTGAGATGGCCGCCCAGGAAGAGCCGCAGATCCAATTCAAGC	splicing	**Ran**	ENSMUSP00000103619	.	NM_009391:exon1:UTR5	.	**NA**	5,129020279,G,TCTGCTGAGATGGCCGCCCAGGAAGAGCCGCAGATCCAATTCA	NA	5qG1.3	.	0.25	.	60	chr5	129020279	.	G	TCTGCTGAGATGGCCGCCCAGGAAGAGCCGCAGATCCAATTCA	.	clustered_events;homologous_mapping_event	ECNT=6;HCNT=1;MAX_ED=23;MIN_ED=3;NLOD=7.32;TLOD=11.58	GT:AD:AF:ALT_F1R2:ALT_F2R1:FOXOG:PGT:PID:QSS:REF_F1R2:REF_F2R1	0/1:55,0:0.066:0:0:.:0|1:129020257_G_A:1477,0:32:23	0/0:32,0:0.037:0:0:.:0|1:129020257_G_A:857,0:20:12	0.5	Unkown	.	.	.	.	.	.	.	.
T3df	chr5	**1**	**12.5**	129020279	129020279	-	A	splicing	**Ran**	ENSMUSP00000103619	.	NM_009391:exon1:UTR5	.	**NA**	5,129020279,G,C	NA	5qG1.3	.	0.25	.	59	chr5	129020279	.	G	C	.	clustered_events;homologous_mapping_event	ECNT=3;HCNT=3;MAX_ED=12;MIN_ED=1;NLOD=10.54;TLOD=9.36	GT:AD:AF:ALT_F1R2:ALT_F2R1:FOXOG:PGT:PID:QSS:REF_F1R2:REF_F2R1	0/1:25,3:0.107:0:3:1.00:0|1:54619955_G_T:694,82:13:12	0/0:38,0:0.00:0:0:.:0|1:54619955_G_T:1027,0:19:19	0.7	Unkown	.	.	.	.	.	.	.	.
T3df	chr5	**1**	**12.5**	129020280	129020280	-	CTGCTGAGATGGCCGCCCAGGAAGAGCCGCAGATCCAATTCA	splicing	**Ran**	ENSMUSP00000103619	.	NM_009391:exon1:UTR5	.	**NA**	5,129020280,T,A	NA	5qG1.3	.	0.25	.	32	chr5	129020280	.	T	A	.	clustered_events;homologous_mapping_event	ECNT=3;HCNT=3;MAX_ED=12;MIN_ED=1;NLOD=9.63;TLOD=9.31	GT:AD:AF:ALT_F1R2:ALT_F2R1:FOXOG:PGT:PID:QSS:REF_F1R2:REF_F2R1	0/1:26,3:0.103:0:3:0.00:0|1:54619955_G_T:746,84:13:13	0/0:36,0:0.00:0:0:.:0|1:54619955_G_T:1009,0:17:19	0.7	Unkown	.	.	.	.	.	.	.	.
T1df	chr19	**1**	**12.5**	29810296	29810296	T	-	exonic	**Ranbp6**	ENSP00000259569	.	frameshift deletion	Ranbp6:NM_177721:exon1:c.2655delA:p.P885fs	**P885fs**	NA	19,29810295,AT,A	19qC1	.	0.25	.	39	chr19	29810295	.	AT	A	.	t_lod_fstar	ECNT=1;HCNT=1;MAX_ED=.;MIN_ED=.;NLOD=10.83;TLOD=4.20	GT:AD:AF:ALT_F1R2:ALT_F2R1:FOXOG:QSS:REF_F1R2:REF_F2R1	0/1:53,2:0.038:0:2:.:1549,58:23:30	0/0:39,0:0.00:0:0:.:1114,0:16:23	1.8	Unknown	NA	NA	175	30	NA	NA	NA	NA
T32df	chr11	**1**	**12.5**	68975104	68975104	G	A	exonic	**Rangrf**	ENSP00000226105	.	nonsynonymous SNV	Rangrf:NM_001285441:exon1:c.C65T:p.A22V	**A22V**	ENSP00000226105,22,A,V	11,68975104,G,A	11qB3	.	0.25	.	35	chr11	68975104	.	G	A	.	clustered_events;homologous_mapping_event;t_lod_fstar	ECNT=3;HCNT=2;MAX_ED=19;MIN_ED=16;NLOD=8.42;TLOD=5.37	GT:AD:AF:ALT_F1R2:ALT_F2R1:FOXOG:QSS:REF_F1R2:REF_F2R1	0/1:30,3:0.091:0:3:0.00:872,83:15:15	0/0:35,0:0.00:0:0:.:1006,0:18:17	0.3	Unknown	NA	NA	NA	NA	NA	NA	NA	NA

T32df	chr11	**1**	**12.5**	68975120	68975120	C	T	exonic	**Rangrf**	ENSP00000226105	.	nonsynonymous SNV	Rangrf:NM_001285441:exon1:c.G49A:p.G17R	**G17R**	ENSP00000226105,17,G,R	11,68975120,C,T	11qB3	.	0.25	.	36	chr11	68975120	.	C	T	.	clustered_events;homologous_mapping_event	ECNT=3;HCNT=2;MAX_ED=19;MIN_ED=16;NLOD=8.73;TLOD=9.18	GT:AD:AF:ALT_F1R2:ALT_F2R1:FOXOG:PGT:PID:QSS:REF_F1R2:REF_F2R1	0/1:31,3:0.094:0:3:1.00:0|1:68975120_C_T:817,87:15:16	0/0:36,0:0.00:0:0:.:0|1:68975120_C_T:856,0:20:16	0.3	Unknown	NA	NA	NA	NA	NA	NA	NA	NA
T32df	chr11	**1**	**12.5**	68975124	68975124	T	-	exonic	**Rangrf**	ENSP00000226105	.	frameshift deletion	Rangrf:NM_001285441:exon1:c.45delA:p.A15fs	**A15fs**	NA	11,68975123,GT,G	11qB3	.	0.25	.	34	chr11	68975123	.	GT	G	.	clustered_events;homologous_mapping_event	ECNT=3;HCNT=2;MAX_ED=19;MIN_ED=16;NLOD=8.73;TLOD=9.13	GT:AD:AF:ALT_F1R2:ALT_F2R1:FOXOG:PGT:PID:QSS:REF_F1R2:REF_F2R1	0/1:31,3:0.091:0:3:.:0|1:68975120_C_T:874,84:15:16	0/0:34,0:0.00:0:0:.:0|1:68975120_C_T:910,0:18:16	0.3	Unknown	NA	NA	NA	NA	NA	NA	NA	NA
T32df	chr11	**1**	**12.5**	54619956	54619956	A	C	exonic	**Rapgef6**	ENSMUSP00000042173	.	nonsynonymous SNV	Rapgef6:NM_001252497:exon3:c.A257C:p.E86A,Rapgef6:NM_001252498:exon3:c.A257C:p.E86A,Rapgef6:NM_001252494:exon8:c.A698C:p.E233A,Rapgef6:NM_001252496:exon8:c.A698C:p.E233A,Rapgef6:NM_175258:exon8:c.A698C:p.E233A	**E86A**	ENSMUSP00000042173,86,E,A	11,54619956,A,C	11qB1.3	.	0.25	.	38	chr11	54619956	.	A	C	.	clustered_events;homologous_mapping_event	ECNT=3;HCNT=3;MAX_ED=12;MIN_ED=1;NLOD=10.54;TLOD=9.36	GT:AD:AF:ALT_F1R2:ALT_F2R1:FOXOG:PGT:PID:QSS:REF_F1R2:REF_F2R1	0/1:25,3:0.107:0:3:1.00:0|1:54619955_G_T:694,82:13:12	0/0:38,0:0.00:0:0:.:0|1:54619955_G_T:1027,0:19:19	0.7	Damaging	-2.08	Neutral	210	30	0.007	Damaging	3.06	202
T32df	chr11	**1**	**12.5**	54619956	54619956	A	C	exonic	**Rapgef6**	ENSMUSP00000042173	.	nonsynonymous SNV	Rapgef6:NM_001252497:exon3:c.A257C:p.E86A,Rapgef6:NM_001252498:exon3:c.A257C:p.E86A,Rapgef6:NM_001252494:exon8:c.A698C:p.E233A,Rapgef6:NM_001252496:exon8:c.A698C:p.E233A,Rapgef6:NM_175258:exon8:c.A698C:p.E233A	**E233A**	ENSMUSP00000042173,233,E,A	11,54619956,A,T	11qB1.3	.	0.25	.	38	chr11	54619956	.	A	T	.	clustered_events;homologous_mapping_event	ECNT=3;HCNT=3;MAX_ED=12;MIN_ED=1;NLOD=10.54;TLOD=9.36	GT:AD:AF:ALT_F1R2:ALT_F2R1:FOXOG:PGT:PID:QSS:REF_F1R2:REF_F2R1	0/1:25,3:0.107:0:3:0.00:0|1:54619955_G_T:727,83:13:12	0/0:39,0:0.00:0:0:.:0|1:54619955_G_T:1103,0:20:19	0.7	Damaging	-2.44	Neutral	210	30	0.002	Damaging	3.06	202
T32df	chr11	**1**	**12.5**	54619967	54619967	G	A	exonic	**Rapgef6**	ENSMUSP00000042173	.	nonsynonymous SNV	Rapgef6:NM_001252497:exon3:c.G268A:p.E90K,Rapgef6:NM_001252498:exon3:c.G268A:p.E90K,Rapgef6:NM_001252494:exon8:c.G709A:p.E237K,Rapgef6:NM_001252496:exon8:c.G709A:p.E237K,Rapgef6:NM_175258:exon8:c.G709A:p.E237K	**E90K**	ENSMUSP00000042173,90,E,K	11,54619967,G,A	11qB1.3	.	0.25	.	36	chr11	54619967	.	G	A	.	clustered_events;homologous_mapping_event	ECNT=3;HCNT=3;MAX_ED=12;MIN_ED=1;NLOD=9.63;TLOD=9.31	GT:AD:AF:ALT_F1R2:ALT_F2R1:FOXOG:PGT:PID:QSS:REF_F1R2:REF_F2R1	0/1:26,3:0.103:0:3:0.00:0|1:54619955_G_T:746,84:13:13	0/0:36,0:0.00:0:0:.:0|1:54619955_G_T:1009,0:17:19	0.7	Damaging	-0.87	Neutral	210	30	0.019	Damaging	2.96	207
T32df	chr11	**1**	**12.5**	54619955	54619955	G	T	exonic	**Rapgef6**	ENSMUSP00000042173	.	stopgain	Rapgef6:NM_001252497:exon3:c.G256T:p.E86X,Rapgef6:NM_001252498:exon3:c.G256T:p.E86X,Rapgef6:NM_001252494:exon8:c.G697T:p.E233X,Rapgef6:NM_001252496:exon8:c.G697T:p.E233X,Rapgef6:NM_175258:exon8:c.G697T:p.E233X	**E233X**	ENSMUSP00000042173,233,E,X	NA	11qB1.3	.	0.25	.	39	chr11	54619955	.	G	GC	.	t_lod_fstar	ECNT=1;HCNT=1;MAX_ED=.;MIN_ED=.;NLOD=8.73;TLOD=4.17	GT:AD:AF:ALT_F1R2:ALT_F2R1:FOXOG:QSS:REF_F1R2:REF_F2R1	0/1:42,2:0.047:0:2:.:1216,58:25:17	0/0:31,0:0.00:0:0:.:878,0:19:12	0.4	Deleterious	.	.	.	.	.	.	.	.
T32df	chr11	**1**	**12.5**	54619955	54619955	G	T	exonic	**Rapgef6**	ENSMUSP00000042173	.	stopgain	Rapgef6:NM_001252497:exon3:c.G256T:p.E86X,Rapgef6:NM_001252498:exon3:c.G256T:p.E86X,Rapgef6:NM_001252494:exon8:c.G697T:p.E233X,Rapgef6:NM_001252496:exon8:c.G697T:p.E233X,Rapgef6:NM_175258:exon8:c.G697T:p.E233X	**E86X**	ENSMUSP00000042173,86,E,X	NA	11qB1.3	.	0.25	.	39	chr11	54619955	.	G	A	.	str_contraction;t_lod_fstar	ECNT=1;HCNT=1;MAX_ED=.;MIN_ED=.;NLOD=5.48;RPA=6,5;RU=GAG;STR;TLOD=5.47	GT:AD:AF:ALT_F1R2:ALT_F2R1:FOXOG:QSS:REF_F1R2:REF_F2R1	0/1:42,2:0.049:1:1:.:1206,59:23:19	0/0:23,0:0.00:0:0:.:601,0:11:12	0.4	Deleterious	.	.	.	.	.	.	.	.
T32df	chr11	**1**	**12.5**	54619967	54619967	G	A	exonic	**Rapgef6**	ENSMUSP00000042173	.	nonsynonymous SNV	Rapgef6:NM_001252497:exon3:c.G268A:p.E90K,Rapgef6:NM_001252498:exon3:c.G268A:p.E90K,Rapgef6:NM_001252494:exon8:c.G709A:p.E237K,Rapgef6:NM_001252496:exon8:c.G709A:p.E237K,Rapgef6:NM_175258:exon8:c.G709A:p.E237K	**E237K**	ENSMUSP00000042173,237,E,K	11,54619967,G,T	11qB1.3	.	0.25	.	36	chr11	54619967	.	G	T	.	clustered_events;homologous_mapping_event	ECNT=3;HCNT=3;MAX_ED=12;MIN_ED=1;NLOD=10.54;TLOD=9.36	GT:AD:AF:ALT_F1R2:ALT_F2R1:FOXOG:PGT:PID:QSS:REF_F1R2:REF_F2R1	0/1:25,3:0.107:0:3:0.00:0|1:54619955_G_T:727,83:13:12	0/0:39,0:0.00:0:0:.:0|1:54619955_G_T:1103,0:20:19	0.7	Unknown	NA	NA	210	30	NA	NA	NA	NA
T1df	chr7	**1**	**12.5**	45628456	45628458	GCC	-	exonic	**Rasip1**	ENSMUSP00000040057	.	nonframeshift deletion	Rasip1:NM_028544:exon3:c.180_182del:p.60_61del	**60_61del**	NA	7,45628456,TGCC,G	7qB3	.	0.25	.	32	chr7	45628455	.	TGCC	G	.	t_lod_fstar	ECNT=1;HCNT=1;MAX_ED=.;MIN_ED=.;NLOD=10.30;TLOD=5.24	GT:AD:AF:ALT_F1R2:ALT_F2R1:FOXOG:QSS:REF_F1R2:REF_F2R1	0/1:18,7:0.280:7:0:1.00:506,107:4:14	0/0:39,0:0.00:0:0:.:1011,0:17:22	2.5	Unknown	.	.	.	.	.	.	.	.

T32df	chr5	**1**	**12.5**	120130749	120130749	-	C	exonic	**Rbm19**	ENSMUSP00000146109	.	frameshift insertion	Rbm19:NM_028762:exon12:c.1441_1442insC:p.E481fs	**E481fs**	ENSMUSP00000146109,481,E,	5,120130749,G,T	5qF	.	0.25	.	31	chr5	120130749	.	G	T	.	PASS	DB;ECNT=1;HCNT=1;MAX_ED=.;MIN_ED=.;NLOD=10.83;TLOD=97.19	GT:AD:AF:ALT_F1R2:ALT_F2R1:FOXOG:QSS:REF_F1R2:REF_F2R1	0/1:38,39:0.507:19:20:0.487:1043,1081:22:16	0/0:39,0:0.00:0:0:.:1061,0:16:23	1.5	Unknown	NA	NA	80	30	NA	NA	NA	NA
T1df	chr5	**1**	**12.5**	120140281	120140283	GAG	-	exonic	**Rbm19**	ENSMUSP00000146109	.	nonframeshift deletion	Rbm19:NM_028762:exon17:c.2128_2130del:p.710_710del	**710_710del**	NA	5,120140281,AGAG,C	5qF	.	0.25	.	23	chr5	120140280	.	AGAG	C	.	PASS	DB;ECNT=1;HCNT=1;MAX_ED=.;MIN_ED=.;NLOD=10.14;TLOD=33.23	GT:AD:AF:ALT_F1R2:ALT_F2R1:FOXOG:QSS:REF_F1R2:REF_F2R1	0/1:15,13:0.462:7:6:0.538:426,383:7:8	0/0:37,0:0.00:0:0:.:1008,0:15:22	1.5	Unknown	.	.	.	.	.	.	.	.
T22df	chr1	**1**	**12.5**	16128220	16128220	C	A	exonic	**Rdh10**	ENSMUSP00000108521	.	nonsynonymous SNV	Rdh10:NM_133832:exon3:c.C581A:p.T194K	**T194K**	ENSMUSP00000108521,194,T,K	1,16128220,C,C	1qA3	.	0.25	.	35	chr1	16128220	.	C	C	.	clustered_events;t_lod_fstar	ECNT=2;HCNT=2;MAX_ED=7;MIN_ED=7;NLOD=16.56;TLOD=5.62	GT:AD:AF:ALT_F1R2:ALT_F2R1:FOXOG:PGT:PID:QSS:REF_F1R2:REF_F2R1	0/1:37,2:0.054:0:2:0.00:0|1:27471790_GA_G:1053,58:28:9	0/0:61,0:0.00:0:0:.:0|1:27471790_GA_G:1705,0:38:23	0.5	Tolerated	0	Neutral	110	30	1	Tolerated	2.78	101
T42df	chr1	**1**	**12.5**	38088271	38088271	G	T	exonic	**Rev1**	ENSMUSP00000107532	.	nonsynonymous SNV	Rev1:NM_019570:exon6:c.C907A:p.H303N	**H303N**	ENSMUSP00000107532,303,H,N	1,38088271,G,A	1qB	.	0.25	.	34	chr1	38088271	.	G	A	.	clustered_events;homologous_mapping_event;t_lod_fstar	ECNT=6;HCNT=19;MAX_ED=45;MIN_ED=6;NLOD=8.13;TLOD=5.32	GT:AD:AF:ALT_F1R2:ALT_F2R1:FOXOG:PGT:PID:QSS:REF_F1R2:REF_F2R1	0/1:52,2:0.038:1:1:0.500:0|1:38088306_C_T:1480,56:29:23	0/0:29,0:0.00:0:0:.:0|1:38088306_C_T:811,0:18:11	0.5	Damaging	-2.42	Neutral	78	30	0.021	Damaging	3.3	55
T42df	chr1	**1**	**12.5**	38088307	38088307	T	A	exonic	**Rev1**	ENSMUSP00000107532	.	nonsynonymous SNV	Rev1:NM_019570:exon6:c.A871T:p.S291C	**S291C**	ENSMUSP00000107532,291,S,C	1,38088307,T,G	1qB	.	0.25	.	29	chr1	38088307	.	T	G	.	clustered_events;homologous_mapping_event;t_lod_fstar	ECNT=9;HCNT=2;MAX_ED=22;MIN_ED=2;NLOD=10.84;TLOD=5.24	GT:AD:AF:ALT_F1R2:ALT_F2R1:FOXOG:PGT:PID:QSS:REF_F1R2:REF_F2R1	0/1:55,2:0.035:0:2:1.00:0|1:109240861_C_G:1516,55:28:27	0/0:39,0:0.00:0:0:.:0|1:109240861_C_G:1076,0:26:13	8	Damaging	-1.12	Neutral	78	30	0.015	Damaging	3.32	54
T42df	chr1	**1**	**12.5**	38088265	38088265	T	C	exonic	**Rev1**	ENSMUSP00000107532	.	nonsynonymous SNV	Rev1:NM_019570:exon6:c.A913G:p.N305D	**N305D**	ENSMUSP00000107532,305,N,D	1,38088265,T,T	1qB	.	0.25	.	36	chr1	38088265	.	T	T	.	clustered_events;homologous_mapping_event;t_lod_fstar	ECNT=6;HCNT=19;MAX_ED=45;MIN_ED=6;NLOD=8.13;TLOD=5.32	GT:AD:AF:ALT_F1R2:ALT_F2R1:FOXOG:PGT:PID:QSS:REF_F1R2:REF_F2R1	0/1:52,2:0.038:1:1:0.500:0|1:38088306_C_T:1479,58:28:24	0/0:29,0:0.00:0:0:.:0|1:38088306_C_T:828,0:18:11	0.5	Tolerated	0	Neutral	78	30	1	Tolerated	3.3	55
T42df	chr1	**1**	**12.5**	38088306	38088306	C	T	exonic	**Rev1**	ENSMUSP00000107532	.	nonsynonymous SNV	Rev1:NM_019570:exon6:c.G872A:p.S291N	**S291N**	ENSMUSP00000107532,291,S,N	1,38088306,C,C	1qB	.	0.25	.	29	chr1	38088306	.	C	C	.	clustered_events;homologous_mapping_event;t_lod_fstar	ECNT=6;HCNT=19;MAX_ED=45;MIN_ED=6;NLOD=8.13;TLOD=5.32	GT:AD:AF:ALT_F1R2:ALT_F2R1:FOXOG:PGT:PID:QSS:REF_F1R2:REF_F2R1	0/1:53,2:0.038:1:1:0.500:0|1:38088306_C_T:1360,56:28:25	0/0:29,0:0.00:0:0:.:0|1:38088306_C_T:703,0:18:11	0.5	Tolerated	0	Neutral	78	30	0.398	Tolerated	3.32	54
T42df	chr1	**1**	**12.5**	38088310	38088310	G	C	exonic	**Rev1**	ENSMUSP00000107532	.	nonsynonymous SNV	Rev1:NM_019570:exon6:c.C868G:p.R290G	**R290G**	ENSMUSP00000107532,290,R,G	1,38088310,G,G	1qB	.	0.25	.	29	chr1	38088310	.	G	G	.	clustered_events;homologous_mapping_event;t_lod_fstar	ECNT=9;HCNT=2;MAX_ED=22;MIN_ED=2;NLOD=10.84;TLOD=5.22	GT:AD:AF:ALT_F1R2:ALT_F2R1:FOXOG:PGT:PID:QSS:REF_F1R2:REF_F2R1	0/1:55,2:0.034:0:2:0.00:0|1:109240861_C_G:1505,42:28:27	0/0:40,0:0.00:0:0:.:0|1:109240861_C_G:1069,0:27:13	8	Tolerated	0	Neutral	78	30	0.871	Tolerated	3.32	54
T12df	chr11	**1**	**12.5**	109240870	109240870	C	A	exonic	**Rgs9**	ENSMUSP00000025253	.	nonsynonymous SNV	Rgs9:NM_001165934:exon14:c.G1035T:p.E345D,Rgs9:NM_011268:exon14:c.G1035T:p.E345D	**E345D**	ENSMUSP00000025253,345,E,D	11,109240870,C,C	11qE1	.	0.25	.	40	chr11	109240870	.	C	C	.	clustered_events;homologous_mapping_event;t_lod_fstar	ECNT=9;HCNT=2;MAX_ED=22;MIN_ED=2;NLOD=11.14;TLOD=5.24	GT:AD:AF:ALT_F1R2:ALT_F2R1:FOXOG:PGT:PID:QSS:REF_F1R2:REF_F2R1	0/1:56,2:0.035:0:2:.:0|1:109240861_C_G:1590,35:29:27	0/0:40,0:0.00:0:0:.:0|1:109240861_C_G:1137,0:26:14	8	Damaging	-1.39	Neutral	153	30	0.048	Damaging	3.12	151
T12df	chr11	**1**	**12.5**	109240868	109240868	T	G	exonic	**Rgs9**	ENSMUSP00000025253	.	nonsynonymous SNV	Rgs9:NM_001165934:exon14:c.A1037C:p.K346T,Rgs9:NM_011268:exon14:c.A1037C:p.K346T	**K346T**	ENSMUSP00000025253,346,K,T	11,109240868,T,A	11qE1	.	0.25	.	40	chr11	109240868	.	T	A	.	clustered_events;homologous_mapping_event;t_lod_fstar	ECNT=9;HCNT=2;MAX_ED=22;MIN_ED=2;NLOD=10.54;TLOD=5.24	GT:AD:AF:ALT_F1R2:ALT_F2R1:FOXOG:PGT:PID:QSS:REF_F1R2:REF_F2R1	0/1:55,2:0.035:0:2:1.00:0|1:109240861_C_G:1538,47:28:27	0/0:40,0:0.00:0:0:.:0|1:109240861_C_G:1114,0:27:13	8	Deleterious	-4.21	Deleterious	150	30	0.042	Damaging	2.86	352

T12df	chr11	**1**	**12.5**	109240861	109240861	C	G	exonic	**Rgs9**	ENSMU<br>SP0000<br>002525<br>3	.	nonsyno<br>nymous<br>SNV	Rgs9:NM_001165934:e<br>xon14:c.G1044C:p.E348<br>D,Rgs9:NM_011268:ex<br>on14:c.G1044C:p.E348<br>D	**E348D**	ENSMUSP<br>00000025<br>253,348,E,<br>D	11,1092408<br>61,C,A	11qE1	.	0.25	.	39	chr11	109240861	.	C	A	.	clustere<br>d_event<br>s;homol<br>ogous_<br>mappin<br>g_event<br>;t_lod_f<br>star	ECNT=9;<br>HCNT=2;<br>MAX_ED<br>=22;MIN<br>_ED=2;N<br>LOD=10.<br>54;TLOD<br>=5.22	GT:AD:A<br>F:ALT_F1<br>R2:ALT_F<br>2R1:FOX<br>OG:PGT:<br>PID:QSS:<br>REF_F1R<br>2:REF_F2<br>R1	0/1:55,2:<br>0.034:0:2<br>:1.00:0|1<br>:1092408<br>61_C_G:1<br>566,36:28<br>:27	0/0:40,0:<br>0.00:0:0:<br>.:0|1:109<br>240861_<br>C_G:1117<br>,0:27:13	8	Tolerate<br>d	-0.22	Neutral	150	30	0.174	Tolerate<br>d	2.86	352
T12df	chr11	**1**	**12.5**	109240873	109240873	C	A	exonic	**Rgs9**	ENSMU<br>SP0000<br>002525<br>3	.	nonsyno<br>nymous<br>SNV	Rgs9:NM_001165934:e<br>xon14:c.G1032T:p.K344<br>N,Rgs9:NM_011268:ex<br>on14:c.G1032T:p.K344<br>N	**K344N**	ENSMUSP<br>00000025<br>253,344,K,<br>N	11,1092408<br>73,C,A	11qE1	.	0.25	.	40	chr11	109240873	.	C	A	.	clustere<br>d_event<br>s;homol<br>ogous_<br>mappin<br>g_event<br>;t_lod_f<br>star	ECNT=9;<br>HCNT=2;<br>MAX_ED<br>=22;MIN<br>_ED=2;N<br>LOD=10.<br>84;TLOD<br>=5.18	GT:AD:A<br>F:ALT_F1<br>R2:ALT_F<br>2R1:FOX<br>OG:PGT:<br>PID:QSS:<br>REF_F1R<br>2:REF_F2<br>R1	0/1:59,2:<br>0.033:0:2<br>:.:0|1:10<br>9240861_<br>C_G:1649,<br>34:30:29	0/0:38,0:<br>0.00:0:0:<br>.:0|1:109<br>240861_<br>C_G:1007<br>,0:25:13	8	Tolerate<br>d	-1.53	Neutral	150	30	0.121	Tolerate<br>d	2.86	351
T12df	chr11	**1**	**12.5**	109240864	109240864	T	-	exonic	**Rgs9**	ENSMU<br>SP0000<br>002525<br>3	.	framesh<br>ift<br>deletion	Rgs9:NM_001165934:e<br>xon14:c.1041delA:p.A3<br>47fs,Rgs9:NM_011268:<br>exon14:c.1041delA:p.A<br>347fs	**A347fs**	ENSMUSP<br>00000025<br>253,347,A,<br>.	11,1092408<br>64,CT,GAAA	11qE1	.	0.25	.	40	chr11	109240863	.	CT	GAAA	.	clustere<br>d_event<br>s;homol<br>ogous_<br>mappin<br>g_event<br>;t_lod_f<br>star	ECNT=9;<br>HCNT=2;<br>MAX_ED<br>=22;MIN<br>_ED=2;N<br>LOD=9.6<br>3;RPA=1,<br>4;RU=A;S<br>TR;TLOD<br>=5.19	GT:AD:A<br>F:ALT_F1<br>R2:ALT_F<br>2R1:FOX<br>OG:PGT:<br>PID:QSS:<br>REF_F1R<br>2:REF_F2<br>R1	0/1:59,2:<br>0.033:0:2<br>:.:0|1:10<br>9240861_<br>C_G:1631,<br>47:30:29	0/0:35,0:<br>0.00:0:0:<br>.:0|1:109<br>240861_<br>C_G:911,<br>0:23:12	8	Unknow<br>n	NA	NA	150	30	NA	NA	NA	NA
T12df	chr11	**1**	**12.5**	109240878	109240883	CCTTGG	-	exonic	**Rgs9**	ENSMU<br>SP0000<br>002525<br>3	.	nonfram<br>eshift<br>deletion	Rgs9:NM_001165934:e<br>xon14:c.1022_1027del:<br>p.341_343del,Rgs9:NM<br>_011268:exon14:c.102<br>2_1027del:p.341_343d<br>el	**341_34<br>3del**	NA	11,1092408<br>78,ACCTTGG<br>,GAAA	11qE1	.	0.25	.	38	chr11	109240877	.	ACCTTGG	GAAA	.	clustere<br>d_event<br>s;homol<br>ogous_<br>mappin<br>g_event<br>;t_lod_f<br>star	ECNT=9;<br>HCNT=2;<br>MAX_ED<br>=22;MIN<br>_ED=2;N<br>LOD=9.6<br>3;RPA=1,<br>4;RU=A;S<br>TR;TLOD<br>=5.19	GT:AD:A<br>F:ALT_F1<br>R2:ALT_F<br>2R1:FOX<br>OG:PGT:<br>PID:QSS:<br>REF_F1R<br>2:REF_F2<br>R1	0/1:59,2:<br>0.033:0:2<br>:.:0|1:10<br>9240861_<br>C_G:1631,<br>47:30:29	0/0:35,0:<br>0.00:0:0:<br>.:0|1:109<br>240861_<br>C_G:911,<br>0:23:12	8	Unknow<br>n	.	.	.	.	.	.	.	.
T32df	chr6	**1**	**12.5**	128359117	128359117	C	T	exonic	**Rhno1**	ENSMU<br>SP0000<br>013843<br>3	.	nonsyno<br>nymous<br>SNV	Rhno1:NM_001326583:<br>exon2:c.G154A:p.V52I,<br>Rhno1:NM_001326585:<br>exon2:c.G154A:p.V52I,<br>Rhno1:NM_001326586:<br>exon3:c.G154A:p.V52I,<br>Rhno1:NM_001326587:<br>exon3:c.G154A:p.V52I	**V52I**	ENSMUSP<br>00000138<br>433,52,V,I	6,12835911<br>7,C,T	6qF3	.	0.25	.	43	chr6	128359117	.	C	T	.	clustere<br>d_event<br>s;homol<br>ogous_<br>mappin<br>g_event<br>;t_lod_f<br>star	ECNT=3;<br>HCNT=2;<br>MAX_ED<br>=9;MIN_<br>ED=3;NL<br>OD=14.7<br>5;TLOD=<br>5.39	GT:AD:A<br>F:ALT_F1<br>R2:ALT_F<br>2R1:FOX<br>OG:PGT:<br>PID:QSS:<br>REF_F1R<br>2:REF_F2<br>R1	0/1:45,2:<br>0.042:2:0<br>:0.00:0|1<br>:1957524<br>7_C_T:12<br>74,58:22:<br>23	0/0:54,0:<br>0.00:0:0:<br>.:0|1:195<br>75247_C<br>_T:1413,<br>0:21:33	19	Tolerate<br>d	0.22	Neutral	43	14	0.639	Tolerate<br>d	2.85	30
T12df	chr6	**1**	**12.5**	128359192	128359192	G	A	exonic	**Rhno1**	ENSMU<br>SP0000<br>013843<br>3	.	nonsyno<br>nymous<br>SNV	Rhno1:NM_001326583:<br>exon2:c.C79T:p.H27Y,R<br>hno1:NM_001326585:e<br>xon2:c.C79T:p.H27Y,Rh<br>no1:NM_001326586:ex<br>on3:c.C79T:p.H27Y,Rhn<br>o1:NM_001326587:exo<br>n3:c.C79T:p.H27Y	**H27Y**	ENSMUSP<br>00000138<br>433,27,H,Y	6,12835919<br>2,G,AGATGT<br>TAAAGTCTC<br>AGTCAGCTG<br>ACCCTTTTA<br>TTACCCCTG<br>AGATGGAC<br>GCTGGCT	6qF3	.	0.25	.	43	chr6	128359192	.	G	AGATGTT<br>AAAGTCT<br>CAGTCAG<br>CTGACCCT<br>TTTATTAC<br>CCCTGAG<br>ATGGACG<br>CTGGCT	.	t_lod_fs<br>tar	ECNT=1;<br>HCNT=2;<br>MAX_ED<br>=.;MIN_<br>ED=.;NL<br>OD=7.37<br>;TLOD=5.<br>68	GT:AD:A<br>F:ALT_F1<br>R2:ALT_F<br>2R1:FOX<br>OG:QSS:<br>REF_F1R<br>2:REF_F2<br>R1	0/1:29,1:<br>0.069:0:1<br>:.:835,29:<br>16:13	0/0:23,0:<br>0.00:0:0:<br>.:655,0:9:<br>14	0.6	Unknow<br>n	NA	NA	43	14	NA	NA	NA	NA
T2df	chr19	**1**	**12.5**	29600827	29600827	-	GATGTTA<br>AAGTCTC<br>AGTCAGC<br>TGACCCT<br>TTTATTA<br>CCCCTGA<br>GATGGAC<br>GCTGGCT	exonic	**Ric1**	ENSMU<br>SP0000<br>000274<br>0	.	framesh<br>ift<br>insertion	Ric1:NM_001081319:ex<br>on23:c.3431_3432insG<br>ATGTTAAAGTCTCAGTC<br>AGCTGACCCTTTTATTAC<br>CCCTGAGATGGACGCTG<br>GCT:p.Q1144fs	**Q1144fs**	ENSMUSP<br>00000002<br>740,1144,<br>Q,	19,2960082<br>7,A,T	19qC1	.	0.25	.	23	chr19	29600827	.	A	T	.	clustere<br>d_event<br>s;homol<br>ogous_<br>mappin<br>g_event<br>;t_lod_f<br>star	ECNT=3;<br>HCNT=4;<br>MAX_ED<br>=9;MIN_<br>ED=3;NL<br>OD=13.8<br>4;TLOD=<br>5.40	GT:AD:A<br>F:ALT_F1<br>R2:ALT_F<br>2R1:FOX<br>OG:QSS:<br>REF_F1R<br>2:REF_F2<br>R1	0/1:48,2:<br>0.042:2:0<br>:0.00:122<br>1,58:24:2<br>4	0/0:56,0:<br>0.00:0:0:<br>.:1277,0:<br>22:34	19	Tolerate<br>d	-1.32	Neutral	111	30	0.247	Tolerate<br>d	2.88	80
T32df	chr4	**1**	**12.5**	19575256	19575256	C	T	exonic	**Rmdn1**	ENSMU<br>SP0000<br>004764<br>0	.	nonsyno<br>nymous<br>SNV	Rmdn1:NM_025476:ex<br>on1:c.C43T:p.R15C	**R15C**	ENSMUSP<br>00000047<br>640,15,R,C	4,19575256,<br>C,T	4qA3	.	0.25	.	56	chr4	19575256	.	C	T	.	PASS	ECNT=1;<br>HCNT=1;<br>MAX_ED<br>=.;MIN_<br>ED=.;NL<br>OD=12.0<br>2;TLOD=<br>13.47	GT:AD:A<br>F:ALT_F1<br>R2:ALT_F<br>2R1:FOX<br>OG:QSS:<br>REF_F1R<br>2:REF_F2<br>R1	0/1:28,7:<br>0.182:6:1<br>:0.857:72<br>0,203:10:<br>18	0/0:44,0:<br>0.00:0:0:<br>.:1097,0:<br>28:16	0.9	Damagi<br>ng	-1.06	Neutral	91	30	0.009	Damagi<br>ng	4.3	31
T32df	chr4	**1**	**12.5**	19575250	19575250	-	TA	exonic	**Rmdn1**	ENSMU<br>SP0000<br>004764<br>0	.	framesh<br>ift<br>insertion	Rmdn1:NM_025476:ex<br>on1:c.37_38insTA:p.P1<br>3fs	**P13fs**	ENSMUSP<br>00000047<br>640,13,P,	4,19575250,<br>C,T	4qA3	.	0.25	.	55	chr4	19575250	.	C	T	.	PASS	ECNT=1;<br>HCNT=1;<br>MAX_ED<br>=.;MIN_<br>ED=.;NL<br>OD=12.0<br>2;TLOD=<br>13.47	GT:AD:A<br>F:ALT_F1<br>R2:ALT_F<br>2R1:FOX<br>OG:QSS:<br>REF_F1R<br>2:REF_F2<br>R1	0/1:28,7:<br>0.182:6:1<br>:0.857:72<br>0,203:10:<br>18	0/0:44,0:<br>0.00:0:0:<br>.:1097,0:<br>28:16	0.9	Tolerate<br>d	1.15	Neutral	91	30	0.161	Tolerate<br>d	4.32	29
T32df	chr4	**1**	**12.5**	19575247	19575247	C	T	exonic	**Rmdn1**	ENSMU<br>SP0000<br>004764<br>0	.	nonsyno<br>nymous<br>SNV	Rmdn1:NM_025476:ex<br>on1:c.C34T:p.P12S	**P12S**	ENSMUSP<br>00000047<br>640,12,P,S	4,19575247,<br>C,CTA	4qA3	.	0.25	.	54	chr4	19575247	.	C	CTA	.	clustere<br>d_event<br>s;homol<br>ogous_<br>mappin<br>g_event<br>;t_lod_f<br>star	ECNT=3;<br>HCNT=2;<br>MAX_ED<br>=9;MIN_<br>ED=3;NL<br>OD=14.4<br>5;TLOD=<br>5.39	GT:AD:A<br>F:ALT_F1<br>R2:ALT_F<br>2R1:FOX<br>OG:PGT:<br>PID:QSS:<br>REF_F1R<br>2:REF_F2<br>R1	0/1:46,2:<br>0.042:2:0<br>:.:0|1:19<br>575247_C<br>_T:1329,5<br>8:22:24	0/0:55,0:<br>0.00:0:0:<br>.:0|1:195<br>75247_C<br>_T:1500,<br>0:22:33	19	Unknow<br>n	NA	NA	91	30	NA	NA	NA	NA

T32df	chr7	**1**	**12.5**	141164158	141164158	G	T	exonic	**Rnh1**	ENSMUSP00000032216	.	nonsynonymous SNV	Rnh1:NM_001172100:exon4:c.C278A:p.T93K,Rnh1:NM_001172101:exon4:c.C386A:p.T129K,Rnh1:NM_145135:exon4:c.C278A:p.T93K	**T93K**	ENSMUSP00000032216,93,T,K	7,141164158,G,C	7qF5	.	0.25	.	44	chr7	141164158	.	G	C	.	str_contraction	ECNT=1;HCNT=1;MAX_ED=.;MIN_ED=.;NLOD=9.31;RPA=5,4;RU=CCT;STR;TLOD=11.68	GT:AD:AF:ALT_F1R2:ALT_F2R1:FOXOG:QSS:REF_F1R2:REF_F2R1	0/1:55,4:0.069:2:2:.:1537,107:33:22	0/0:37,0:0.00:0:0:.:1004,0:13:24	0.7	Deleterious	-3.55	Deleterious	135	30	0.002	Damaging	2.79	64
T32df	chr7	**1**	**12.5**	141164158	141164158	G	T	exonic	**Rnh1**	ENSMUSP00000032216	.	nonsynonymous SNV	Rnh1:NM_001172100:exon4:c.C278A:p.T93K,Rnh1:NM_001172101:exon4:c.C386A:p.T129K,Rnh1:NM_145135:exon4:c.C278A:p.T93K	**T129K**	ENSMUSP00000032216,129,T,K	7,141164158,G,TTTTC	7qF5	.	0.25	.	44	chr7	141164158	.	G	TTTTC	.	t_lod_fstar	ECNT=1;HCNT=1;MAX_ED=.;MIN_ED=.;NLOD=12.34;TLOD=5.07	GT:AD:AF:ALT_F1R2:ALT_F2R1:FOXOG:QSS:REF_F1R2:REF_F2R1	0/1:68,2:0.029:0:2:.:1927,54:35:33	0/0:48,0:0.00:0:0:.:1305,0:24:24	2.3	Unknown	NA	NA	135	30	NA	NA	NA	NA
T12df	chr3	**1**	**12.5**	93397493	93397493	T	A	exonic	**Rptn**	ENSP00000317895	.	nonsynonymous SNV	Rptn:NM_009100:exon3:c.T2132A:p.L711Q	**L711Q**	ENSP00000317895,711,L,Q	3,93397493,T,A	3qF2.1	.	0.25	.	37	chr3	93397493	.	T	A	.	alt_allele_in_normal	ECNT=1;HCNT=4;MAX_ED=.;MIN_ED=.;NLOD=7.45;TLOD=7.57	GT:AD:AF:ALT_F1R2:ALT_F2R1:FOXOG:QSS:REF_F1R2:REF_F2R1	0/1:48,8:0.143:4:4:0.500:1264,160:18:30	0/0:34,3:0.081:1:2:0.333:890,44:18:16	19	Tolerated	1.8	Neutral	55	30	0.4	Tolerated	2.89	32
T3df	chr3	**1**	**12.5**	93397577	93397577	G	A	exonic	**Rptn**	ENSP00000317895	.	nonsynonymous SNV	Rptn:NM_009100:exon3:c.G2216A:p.R739Q	**R739Q**	ENSP00000317895,739,R,Q	3,93397577,G,A	3qF2.1	.	0.25	.	35	chr3	93397577	.	G	A	.	alt_allele_in_normal;t_lod_fstar	ECNT=1;HCNT=1;MAX_ED=.;MIN_ED=.;NLOD=4.53;TLOD=4.89	GT:AD:AF:ALT_F1R2:ALT_F2R1:FOXOG:QSS:REF_F1R2:REF_F2R1	0/1:49,4:0.077:1:3:0.250:966,98:26:23	0/0:32,3:0.091:1:2:0.333:666,67:14:18	19	Tolerated	0.05	Neutral	55	30	0.296	Tolerated	2.92	32
T32df	chr2	**1**	**12.5**	119038222	119038222	C	G	exonic	**Rpusd2**	ENSP00000323288	.	nonsynonymous SNV	Rpusd2:NM_173450:exon3:c.C1125G:p.C375W	**C375W**	ENSP00000323288,375,C,W	2,119038222,C,G	2qE5	.	0.25	.	31	chr2	119038222	.	C	G	.	PASS	ECNT=1;HCNT=2;MAX_ED=.;MIN_ED=.;NLOD=8.38;TLOD=15.30	GT:AD:AF:ALT_F1R2:ALT_F2R1:FOXOG:QSS:REF_F1R2:REF_F2R1	0/1:39,7:0.156:2:5:0.714:1105,204:22:17	0/0:31,0:0.00:0:0:.:839,0:15:16	0.8	Deleterious	-10.63	Deleterious	94	30	0	Damaging	2.81	105
T2df	chr10	**1**	**12.5**	78411015	78411015	-	TTTC	exonic	**Rrp1**	ENSMUSP00000076364	.	frameshift insertion	Rrp1:NM_010925:exon3:c.224_225insGAAA:p.L75fs	**L75fs**	ENSMUSP00000076364,75,L,	10,78411015,T,G	10qC1	.	0.25	.	48	chr10	78411015	.	T	G	.	PASS	ECNT=2;HCNT=2;MAX_ED=2;MIN_ED=2;NLOD=8.70;TLOD=7.22	GT:AD:AF:ALT_F1R2:ALT_F2R1:FOXOG:PGT:PID:QSS:REF_F1R2:REF_F2R1	0/1:45,3:0.064:1:2:.:0|1:90229853_CT_C:1323,79:24:21	0/0:36,0:0.00:0:0:.:0|1:90229853_CT_C:999,0:14:22	1.3	Neutral	0	Neutral	96	30	NA	NA	NA	NA
T12df	chr11	**1**	**12.5**	115935364	115935364	A	T	splicing	**Sap30bp**	ENSMUSP00000152313	NM_020483:exon2:c.107-2A>T	.	.	**NA**	11,115935364,A,CAT	NA	11qE2	.	0.25	.	33	chr11	115935364	.	A	CAT	.	clustered_events;homologous_mapping_event;t_lod_fstar	ECNT=10;HCNT=2;MAX_ED=25;MIN_ED=2;NLOD=6.92;TLOD=5.32	GT:AD:AF:ALT_F1R2:ALT_F2R1:FOXOG:PGT:PID:QSS:REF_F1R2:REF_F2R1	0/1:53,2:0.038:0:2:.:0|1:106348386_C_T:1407,20:24:29	0/0:27,0:0.00:0:0:.:0|1:106348386_C_T:753,0:17:10	9	Unkown	.	.	.	.	.	.	.	.
T22df	chr16	**1**	**12.5**	90229854	90229854	T	-	exonic	**Scaf4**	ENSMUSP00000127112	.	frameshift deletion	Scaf4:NM_001346799:exon20:c.2996delA:p.Q999fs,Scaf4:NM_178923:exon20:c.3074delA:p.Q1025fs	**Q1025fs**	ENSMUSP00000127112,1025,Q,.	16,90229854,CT,C	16qC3.3	.	0.25	.	35	chr16	90229853	.	CT	C	.	t_lod_fstar	ECNT=1;HCNT=1;MAX_ED=.;MIN_ED=.;NLOD=15.67;TLOD=5.66	GT:AD:AF:ALT_F1R2:ALT_F2R1:FOXOG:QSS:REF_F1R2:REF_F2R1	0/1:43,4:0.045:4:0:.:1157,108:23:20	0/0:57,0:0.00:0:0:.:1531,0:24:33	1.3	Unknown	NA	NA	142	30	NA	NA	NA	NA
T22df	chr16	**1**	**12.5**	90229854	90229854	T	-	exonic	**Scaf4**	ENSMUSP00000127112	.	frameshift deletion	Scaf4:NM_001346799:exon20:c.2996delA:p.Q999fs,Scaf4:NM_178923:exon20:c.3074delA:p.Q1025fs	**Q999fs**	ENSMUSP00000127112,999,Q,.	16,90229854,CT,C	16qC3.3	.	0.25	.	35	chr16	90229853	.	CT	C	.	PASS	ECNT=2;HCNT=2;MAX_ED=2;MIN_ED=2;NLOD=8.40;TLOD=7.31	GT:AD:AF:ALT_F1R2:ALT_F2R1:FOXOG:PGT:PID:QSS:REF_F1R2:REF_F2R1	0/1:42,3:0.068:1:2:.:0|1:90229853_CT_C:1212,76:22:20	0/0:35,0:0.00:0:0:.:0|1:90229853_CT_C:963,0:14:21	1.3	Unknown	NA	NA	142	30	NA	NA	NA	NA
T22df	chr16	**1**	**12.5**	90229856	90229860	CGGCT	-	exonic	**Scaf4**	ENSMUSP00000127112	.	frameshift deletion	Scaf4:NM_001346799:exon20:c.2990_2994del:p.Q997fs,Scaf4:NM_178923:exon20:c.3068_3072del:p.Q1023fs	**Q997fs**	ENSMUSP00000127112,997,Q,.	16,90229856,GCGGCT,G	16qC3.3	.	0.25	.	36	chr16	90229855	.	GCGGCT	G	.	PASS	ECNT=2;HCNT=2;MAX_ED=2;MIN_ED=2;NLOD=8.70;TLOD=7.22	GT:AD:AF:ALT_F1R2:ALT_F2R1:FOXOG:PGT:PID:QSS:REF_F1R2:REF_F2R1	0/1:45,3:0.064:1:2:.:0|1:90229853_CT_C:1323,79:24:21	0/0:36,0:0.00:0:0:.:0|1:90229853_CT_C:999,0:14:22	1.3	Unknown	NA	NA	142	30	NA	NA	NA	NA
T22df	chr16	**1**	**12.5**	90229856	90229860	CGGCT	-	exonic	**Scaf4**	ENSMUSP00000127112	.	frameshift deletion	Scaf4:NM_001346799:exon20:c.2990_2994del:p.Q997fs,Scaf4:NM_178923:exon20:c.3068_3072del:p.Q1023fs	**Q1023fs**	ENSMUSP00000127112,1023,Q,.	16,90229856,GCGGCT,T	16qC3.3	.	0.25	.	36	chr16	90229855	.	GCGGCT	T	.	clustered_events;homologous_mapping_event;t_lod_fstar	ECNT=11;HCNT=1;MAX_ED=26;MIN_ED=1;NLOD=11.14;TLOD=5.34	GT:AD:AF:ALT_F1R2:ALT_F2R1:FOXOG:PGT:PID:QSS:REF_F1R2:REF_F2R1	0/1:48,2:0.039:0:2:0.00:0|1:119550719_G_C:1311,54:24:24	0/0:39,0:0.00:0:0:.:0|1:119550719_G_C:1006,0:18:21	0.9	Unknown	NA	NA	142	30	NA	NA	NA	NA

T22df	chr16	**1**	**12.5**	90250207	90250296	AGGCTGAGCCATCCCCGGCTGTGCCAATCCCGGCTGAGCCATCCCAGGCTGAGGCATCCCTGGCTGAGGCATCCCTGGCTGTGGCATTGT	-	exonic	**Scaf4**	ENSMUSP00000127112	.	nonframeshift deletion	Scaf4:NM_001346799:exon10:c.1192_1281del:p.398_427del,Scaf4:NM_178923:exon10:c.1192_1281del:p.398_427del	**398_427del**	NA	16,90250207,CAGGCTGAGCCATCCCCGGCTGTGCCAATCCCGGCTGAGCCATCCCAGGCTGAGGCATCCCTGGCTGAGGCATCCCTGGCTGTGGCATTGT,A	16qC3.3	.	0.25	.	57	chr16	90250206	.	CAGGCTGAGCCATCCCCGGCTGTGCCAATCCCGGCTGAGCCATCCCAGGCTGAGGCATCCCTGGCTGAGGCATCCCTGGCTGTGGCATTGT	A	.	clustered_events;homologous_mapping_event;t_lod_fstar	ECNT=11;HCNT=1;MAX_ED=26;MIN_ED=1;NLOD=12.04;TLOD=5.43	GT:AD:AF:ALT_F1R2:ALT_F2R1:FOXOG:PGT:PID:QSS:REF_F1R2:REF_F2R1	0/1:46,2:0.043:0:2:1.00:0|1:119550719_G_C:1238,39:24:22	0/0:40,0:0.00:0:0:.:0|1:119550719_G_C:1039,0:17:23	0.9	Unknown	.	.	.	.	.	.	.	.
T2df	chr11	**1**	**12.5**	106348386	106348386	C	T	exonic	**Scn4a**	ENSMUSP00000152710	.	nonsynonymous SNV	Scn4a:NM_133199:exon4:c.G497A:p.G166E	**G166E**	ENSMUSP00000152710,166,G,E	11,106348386,C,T	11qE1	.	0.25	.	29	chr11	106348386	.	C	T	.	clustered_events;homologous_mapping_event;t_lod_fstar	ECNT=10;HCNT=2;MAX_ED=25;MIN_ED=2;NLOD=7.53;TLOD=5.32	GT:AD:AF:ALT_F1R2:ALT_F2R1:FOXOG:PGT:PID:QSS:REF_F1R2:REF_F2R1	0/1:51,2:0.038:0:2:1.00:0|1:106348386_C_T:1343,55:22:29	0/0:29,0:0.00:0:0:.:0|1:106348386_C_T:755,0:17:12	9	Deleterious	-5.82	Deleterious	496	30	0.001	Damaging	3.65	393
T2df	chr11	**1**	**12.5**	106348396	106348396	-	CGCC	exonic	**Scn4a**	ENSMUSP00000152710	.	frameshift insertion	Scn4a:NM_133199:exon4:c.486_487insGGCG:p.T163fs	**T163fs**	ENSMUSP00000152710,163,T,	11,106348396,T,A	11qE1	.	0.25	.	27	chr11	106348396	.	T	A	.	alt_allele_in_normal;clustered_events;germline_risk	DB;ECNT=2;HCNT=1;MAX_ED=10;MIN_ED=10;NLOD=2.37;TLOD=8.66	GT:AD:AF:ALT_F1R2:ALT_F2R1:FOXOG:PGT:PID:QSS:REF_F1R2:REF_F2R1	0/1:63,4:0.063:1:3:0.250:0|1:78349046_T_A:1779,109:34:29	0/0:51,4:0.080:3:1:0.750:0|1:78349046_T_A:1377,88:28:23	0.7	Deleterious	-2.74	Deleterious	496	30	0.001	Damaging	3.65	393
T2df	chr11	**1**	**12.5**	106348391	106348393	GAA	-	exonic	**Scn4a**	ENSMUSP00000152710	.	nonframeshift deletion	Scn4a:NM_133199:exon4:c.490_492del:p.164_164del	**164_164del**	NA	11,106348391,TGAA,A	11qE1	.	0.25	.	28	chr11	106348390	.	TGAA	A	.	alt_allele_in_normal;t_lod_fstar	ECNT=2;HCNT=2;MAX_ED=2;MIN_ED=2;NLOD=2.44;TLOD=5.41	GT:AD:AF:ALT_F1R2:ALT_F2R1:FOXOG:PGT:PID:QSS:REF_F1R2:REF_F2R1	0/1:46,2:0.043:1:1:0.500:0|1:78346447_T_C:1219,53:24:22	0/0:34,1:0.053:0:1:0.00:0|1:78346447_T_C:847,29:17:17	0.7	Unknown	.	.	.	.	.	.	.	.
T2df	chr11	**1**	**12.5**	106348401	106348401	-	AT	splicing	**Scn4a**	ENSMUSP00000152710	NM_133199:exon4:c.483-1->AT	.	.	**NA**	11,106348401,C,T	NA	11qE1	.	0.25	.	27	chr11	106348401	.	C	T	.	clustered_events;homologous_mapping_event;t_lod_fstar	ECNT=10;HCNT=2;MAX_ED=25;MIN_ED=2;NLOD=7.83;TLOD=5.27	GT:AD:AF:ALT_F1R2:ALT_F2R1:FOXOG:PGT:PID:QSS:REF_F1R2:REF_F2R1	0/1:53,2:0.036:0:2:.:0|1:106348386_C_T:1507,58:22:31	0/0:28,0:0.00:0:0:.:0|1:106348386_C_T:782,0:18:10	9	Unkown	.	.	.	.	.	.	.	.
T12df	chr9	**1**	**12.5**	119550725	119550727	GAA	-	exonic	**Scn5a**	ENSMUSP00000036394	.	nonframeshift deletion	Scn5a:NM_001253860:exon4:c.490_492del:p.164_164del,Scn5a:NM_021544:exon5:c.490_492del:p.164_164del	**164_164del**	NA	9,119550724,TGAA,T	9qF3	.	0.25	.	37	chr9	119550724	.	TGAA	T	.	clustered_events;homologous_mapping_event;t_lod_fstar	ECNT=11;HCNT=1;MAX_ED=26;MIN_ED=1;NLOD=11.14;TLOD=5.34	GT:AD:AF:ALT_F1R2:ALT_F2R1:FOXOG:PGT:PID:QSS:REF_F1R2:REF_F2R1	0/1:49,2:0.039:0:2:.:0|1:119550719_G_C:1383,56:24:25	0/0:37,0:0.00:0:0:.:0|1:119550719_G_C:953,0:16:21	0.9	Deleterious	-14.96	Deleterious	535	30	NA	NA	NA	NA
T12df	chr9	**1**	**12.5**	119550733	119550733	-	T	exonic	**Scn5a**	ENSMUSP00000036394	.	frameshift insertion	Scn5a:NM_001253860:exon4:c.483_484insA:p.Y162fs,Scn5a:NM_021544:exon5:c.483_484insA:p.Y162fs	**Y162fs**	ENSMUSP00000036394,162,Y,	9,119550733,A,C	9qF3	.	0.25	.	38	chr9	119550733	.	A	C	.	PASS	DB;ECNT=1;HCNT=1;MAX_ED=.;MIN_ED=.;NLOD=10.78;TLOD=55.27	GT:AD:AF:ALT_F1R2:ALT_F2R1:FOXOG:QSS:REF_F1R2:REF_F2R1	0/1:25,26:0.500:9:17:0.346:698,615:19:6	0/0:41,0:0.00:0:0:.:1117,0:22:19	1.1	Deleterious	-8.56	Deleterious	535	30	0	Damaging	3.54	394
T12df	chr9	**1**	**12.5**	119550730	119550730	-	CGCC	exonic	**Scn5a**	ENSMUSP00000036394	.	frameshift insertion	Scn5a:NM_001253860:exon4:c.486_487insGGCG:p.T163fs,Scn5a:NM_021544:exon5:c.486_487insGGCG:p.T163fs	**T163fs**	ENSMUSP00000036394,163,T,	9,119550730,T,T	9qF3	.	0.25	.	38	chr9	119550730	.	T	T	.	clustered_events;homologous_mapping_event;t_lod_fstar	ECNT=11;HCNT=1;MAX_ED=26;MIN_ED=1;NLOD=11.14;TLOD=5.34	GT:AD:AF:ALT_F1R2:ALT_F2R1:FOXOG:PGT:PID:QSS:REF_F1R2:REF_F2R1	0/1:49,2:0.039:0:2:.:0|1:119550719_G_C:1383,56:24:25	0/0:37,0:0.00:0:0:.:0|1:119550719_G_C:953,0:16:21	0.9	Tolerated	0	Neutral	535	30	1	Tolerated	3.54	395
T12df	chr9	**1**	**12.5**	119550720	119550720	G	T	exonic	**Scn5a**	ENSMUSP00000036394	.	nonsynonymous SNV	Scn5a:NM_001253860:exon4:c.C497A:p.A166D,Scn5a:NM_021544:exon5:c.C497A:p.A166D	**A166D**	ENSMUSP00000036394,166,A,D	9,119550720,G,TCGCC	9qF3	.	0.25	.	39	chr9	119550720	.	G	TCGCC	.	clustered_events;homologous_mapping_event;t_lod_fstar	ECNT=11;HCNT=1;MAX_ED=26;MIN_ED=1;NLOD=10.54;TLOD=5.34	GT:AD:AF:ALT_F1R2:ALT_F2R1:FOXOG:PGT:PID:QSS:REF_F1R2:REF_F2R1	0/1:47,2:0.039:0:2:.:0|1:119550719_G_C:1292,49:23:24	0/0:38,0:0.00:0:0:.:0|1:119550719_G_C:950,0:16:22	0.9	Unknown	NA	NA	535	30	NA	NA	NA	NA
T12df	chr9	**1**	**12.5**	119550735	119550735	C	A	splicing	**Scn5a**	ENSMUSP00000036394	.	NM_021544:exon5:c.483-1G>T;NM_001253860:exon4:c.483-1G>T	.	**NA**	9,119550735,C,AT	NA	9qF3	.	0.25	.	40	chr9	119550735	.	C	AT	.	clustered_events;homologous_mapping_event;t_lod_fstar	ECNT=11;HCNT=1;MAX_ED=26;MIN_ED=1;NLOD=11.44;TLOD=5.41	GT:AD:AF:ALT_F1R2:ALT_F2R1:FOXOG:PGT:PID:QSS:REF_F1R2:REF_F2R1	0/1:46,2:0.043:0:2:.:0|1:119550719_G_C:1272,25:24:22	0/0:38,0:0.00:0:0:.:0|1:119550719_G_C:989,0:16:22	0.9	Unkown	.	.	.	.	.	.	.	.

T4df	chr7	**1**	**12.5**	121746586	121746586	G	T	splicing	**Scnn1g**	ENSMUSP00000068424	NM_011326:exon6:c.932-1G>T	.	.	**NA**	7,121746586,G,C	NA	7qF2	.	0.25	.	24	chr7	121746586	.	G	C	.	t_lod_fstar	ECNT=1;HCNT=2;MAX_ED=.;MIN_ED=.;NLOD=12.29;TLOD=4.30	GT:AD:AF:ALT_F1R2:ALT_F2R1:FOXOG:QSS:REF_F1R2:REF_F2R1	0/1:25,2:0.077:2:0:0.00:688,45:10:15	0/0:51,0:0.00:0:0:.:1397,0:24:27	0.5	Unkown	.	.	.	.	.	.	.	.
T1df	chr9	**1**	**12.5**	50600559	50600559	G	A	exonic	**Sdhd**	ENSMUSP00000112134	.	nonsynonymous SNV	Sdhd:NM_025848:exon2:c.C158T:p.P53L	**P53L**	ENSMUSP00000112134,53,P,L	9,50600559,G,C	9qA5.3	.	0.25	.	50	chr9	50600559	.	G	C	.	PASS	ECNT=1;HCNT=1;MAX_ED=.;MIN_ED=.;NLOD=9.62;TLOD=20.26	GT:AD:AF:ALT_F1R2:ALT_F2R1:FOXOG:QSS:REF_F1R2:REF_F2R1	0/1:42,10:0.180:5:5:0.500:1186,293:22:20	0/0:36,0:0.00:0:0:.:1006,0:14:22	0.8	Damaging	-2.01	Neutral	105	30	0.036	Damaging	2.97	68
T1df	chr11	**1**	**12.5**	4075541	4075541	C	T	exonic	**Sec14l3**	ENSMUSP00000112135	.	nonsynonymous SNV	Sec14l3:NM_001029937:exon11:c.C1064T:p.T355I	**T355I**	ENSMUSP00000112135,355,T,I	11,4075541,C,G	11qA1	.	0.25	.	45	chr11	4075541	.	C	G	.	str_contraction;t_lod_fstar	ECNT=1;HCNT=1;MAX_ED=.;MIN_ED=.;NLOD=14.92;RPA=6,5;RU=GCA;STR;TLOD=4.27	GT:AD:AF:ALT_F1R2:ALT_F2R1:FOXOG:QSS:REF_F1R2:REF_F2R1	0/1:40,3:0.050:0:3:.:968,78:20:20	0/0:61,1:0.019:1:0:.:1533,6:30:31	1	Deleterious	-3.57	Deleterious	170	30	0.159	Tolerated	2.84	170
T3df	chr13	**1**	**12.5**	51673967	51673969	AGC	-	exonic	**Secisbp2**	ENSMUSP00000061624	.	nonframeshift deletion	Secisbp2:NM_001308448:exon10:c.1190_1192del:p.397_398del,Secisbp2:NM_029279:exon10:c.1382_1384del:p.461_462del	**397_398del**	NA	13,51673967,AAGC,A	13qA5	.	0.25	.	30	chr13	51673966	.	AAGC	A	.	PASS	DB;ECNT=1;HCNT=1;MAX_ED=.;MIN_ED=.;NLOD=11.43;TLOD=25.91	GT:AD:AF:ALT_F1R2:ALT_F2R1:FOXOG:QSS:REF_F1R2:REF_F2R1	0/1:29,14:0.306:8:6:0.571:811,387:17:12	0/0:42,0:0.00:0:0:.:1171,0:23:19	2	Unknown	.	.	.	.	.	.	.	.
T3df	chr13	**1**	**12.5**	51673967	51673969	AGC	-	exonic	**Secisbp2**	ENSMUSP00000061624	.	nonframeshift deletion	Secisbp2:NM_001308448:exon10:c.1190_1192del:p.397_398del,Secisbp2:NM_029279:exon10:c.1382_1384del:p.461_462del	**461_462del**	NA	13,51673967,AAGC,A	13qA5	.	0.25	.	30	chr13	51673966	.	AAGC	A	.	PASS	DB;ECNT=1;HCNT=1;MAX_ED=.;MIN_ED=.;NLOD=12.58;TLOD=38.52	GT:AD:AF:ALT_F1R2:ALT_F2R1:FOXOG:QSS:REF_F1R2:REF_F2R1	0/1:23,15:0.395:8:7:0.533:611,424:11:12	0/0:49,0:0.00:0:0:.:1283,0:21:28	2	Unknown	.	.	.	.	.	.	.	.
T22df	chr5	**1**	**12.5**	53213243	53213245	GCT	-	exonic	**Sel1l3**	ENSMUSP00000112862	.	nonframeshift deletion	Sel1l3:NM_172710:exon1:c.62_64del:p.21_22del	**21_22del**	NA	5,53213243,GGCT,C	5qC1	.	0.25	.	34	chr5	53213242	.	GGCT	C	.	str_contraction;t_lod_fstar	ECNT=1;HCNT=2;MAX_ED=.;MIN_ED=.;NLOD=5.71;RPA=8,7;RU=CTG;STR;TLOD=4.67	GT:AD:AF:ALT_F1R2:ALT_F2R1:FOXOG:QSS:REF_F1R2:REF_F2R1	0/1:45,2:0.044:0:2:.:1261,57:18:27	0/0:21,0:0.00:0:0:.:580,0:17:4	14	Unknown	.	.	.	.	.	.	.	.
T32df	chr11	**1**	**12.5**	4177588	4177588	C	A	exonic	**Sf3a1**	ENSP00000215793	.	nonsynonymous SNV	Sf3a1:NM_026175:exon12:c.C1913A:p.A638E	**A638E**	ENSP00000215793,638,A,E	11,4177588,C,A	11qA1	.	0.25	.	47	chr11	4177588	.	C	A	.	t_lod_fstar	ECNT=1;HCNT=1;MAX_ED=.;MIN_ED=.;NLOD=13.09;TLOD=5.33	GT:AD:AF:ALT_F1R2:ALT_F2R1:FOXOG:QSS:REF_F1R2:REF_F2R1	0/1:42,5:0.071:2:3:0.600:1100,145:27:15	0/0:47,0:0.00:0:0:.:1141,0:22:25	0.9	Damaging	-1.56	Neutral	117	30	0.02	Damaging	3.11	66
T3df	chr11	**1**	**12.5**	3164316	3164587	[illegible]GAAAGAGAAAGAAAGAAAGAAAGAAAGAAAGAAAGAAAGAAAGAGAGAAAGGCAAAGCAAAAGCAAAGCCAAAAAGAAATAAATGAGAACCAAAGATGGTGTACTTTTCTTCTAACTTTCTTGTCATCACAATTGCAGTGTAATATCTTCTGTTCCCTCTTCCTGAAGGATCACAGACAGTGGGGAGAGCCATCTGCTCAAGTCTGGCTTGG	-	exonic	**Sfi1**	ENSMUSP00000038485	.	frameshift deletion	Sfi1:NM_030207:exon9:c.820_850del:p.Q274fs	**Q274fs**	ENSMUSP00000038485,274,Q,.	[illegible]AGATAGAAAGAAAGAGAAAGAAAGAAAGAAAGAAAGAAAGAAAGAAAGAAAGAGAGAAAGGCAAAGCAAAAGCAAAGCCAAAAAGAAATAAATGAGAACCAAAGATGGTGTACTTTTCTTCTAACTTTCTTGTCATCACAATTGCAGTGTAATATCTTCTGTTCCCTCTTCCTGAAGGATCACAGACAGTGGGGAGAGCCATCTGCTCAAGTCTGGCTTGGAAGCTTAGGCTT	11qA1	.	0.25	.	3557	chr11	3164315	.	[illegible]AGAAAGAGAAAGAAAGAAAGAAAGAAAGAAAGAAAGAAAGAAAGAGAGAAAGGCAAAGCAAAAGCAAAGCCAAAAAGAAATAAATGAGAACCAAAGATGGTGTACTTTTCTTCTAACTTTCTTGTCATCACAATTGCAGTGTAATATCTTCTGTTCCCTCTTCCTGAAGGATCACAGACAGTGGGGAGAGCCATCTGCTCAAGTCTGGCTTGG	T	.	clustered_events;t_lod_fstar	ECNT=2;HCNT=1;MAX_ED=5;MIN_ED=5;NLOD=11.74;TLOD=5.70	GT:AD:AF:ALT_F1R2:ALT_F2R1:FOXOG:PGT:PID:QSS:REF_F1R2:REF_F2R1	0/1:33,2:0.059:2:0:0.00:0|1:153452331_A_T:931,58:17:16	0/0:44,0:0.00:0:0:.:0|1:153452331_A_T:1239,0:20:24	20	Unkown	.	.	.	.	.	.	.	.

T12df	chr5	**1**	**12.5**	35707391	35707391	T	C	exonic	**Sh3tc1**	ENSMUSP00000027053	.	nonsynonymous SNV	Sh3tc1:NM_194344:exon12:c.A1451G:p.D484G	**D484G**	ENSMUSP00000027053,484,D,G	5,35707391,T,A	5qB3	.	0.25	.	31	chr5	35707391	.	T	A	.	clustered_events;t_lod_fstar	ECNT=2;HCNT=1;MAX_ED=5;MIN_ED=5;NLOD=12.04;TLOD=5.67	GT:AD:AF:ALT_F1R2:ALT_F2R1:FOXOG:PGT:PID:QSS:REF_F1R2:REF_F2R1	0/1:34,2:0.057:2:0:1.00:0|1:153452331_A_T:974,56:17:17	0/0:45,0:0.00:0:0:.:0|1:153452331_A_T:1240,0:21:24	20	Deleterious	-3.83	Deleterious	114	30	0.108	Tolerated	2.79	52
T32df	chr1	**1**	**12.5**	153452331	153452331	A	T	exonic	**Shcbp1l**	ENSMUSP00000027251	.	nonsynonymous SNV	Shcbp1l:NM_001033162:exon10:c.A1787T:p.N596I	**N596I**	ENSMUSP00000027251,596,N,I	1,153452331,A,G	1qG3	.	0.25	.	44	chr1	153452331	.	A	G	.	str_contraction;t_lod_fstar	ECNT=1;HCNT=1;MAX_ED=.;MIN_ED=.;NLOD=8.71;RPA=8,7;RU=CAA;STR;TLOD=4.59	GT:AD:AF:ALT_F1R2:ALT_F2R1:FOXOG:QSS:REF_F1R2:REF_F2R1	0/1:41,2:0.050:1:1:.:1174,56:22:19	0/0:34,0:0.00:0:0:.:940,0:18:16	1.3	Damaging	-1.78	Neutral	100	30	0.01	Damaging	3.39	38
T32df	chr1	**1**	**12.5**	153452336	153452336	G	A	exonic	**Shcbp1l**	ENSMUSP00000027251	.	nonsynonymous SNV	Shcbp1l:NM_001033162:exon10:c.G1792A:p.G598R	**G598R**	ENSMUSP00000027251,598,G,R	1,153452336,G,G	1qG3	.	0.25	.	45	chr1	153452336	.	G	G	.	str_contraction;t_lod_fstar	ECNT=1;HCNT=1;MAX_ED=.;MIN_ED=.;NLOD=8.71;RPA=8,7;RU=CAA;STR;TLOD=4.59	GT:AD:AF:ALT_F1R2:ALT_F2R1:FOXOG:QSS:REF_F1R2:REF_F2R1	0/1:41,2:0.050:1:1:.:1174,56:22:19	0/0:34,0:0.00:0:0:.:940,0:18:16	1.3	Tolerated	0	Neutral	100	30	0.66	Tolerated	3.43	37
T12df	chrX	**1**	**12.5**	6624078	6624080	CAA	-	exonic	**Shroom4**	ENSMUSP00000141379	.	nonframeshift deletion	Shroom4:NM_001313765:exon5:c.3001_3003del:p.1001_1001del,Shroom4:NM_001040459:exon6:c.3349_3351del:p.1117_1117del,Shroom4:NM_001313764:exon6:c.3196_3198del:p.1066_1066del	**1117_1117del**	NA	X,6624078,GCAA,A	XqA1.1	rs236965363	0.25	.	34	chrX	6624077	.	GCAA	A	.	str_contraction;t_lod_fstar	ECNT=1;HCNT=1;MAX_ED=.;MIN_ED=.;NLOD=10.20;RPA=8,7;RU=CAG;STR;TLOD=4.39	GT:AD:AF:ALT_F1R2:ALT_F2R1:FOXOG:QSS:REF_F1R2:REF_F2R1	0/1:47,2:0.044:0:2:.:1331,58:26:21	0/0:39,0:0.00:0:0:.:1121,0:18:21	0.7	Unknown	.	.	.	.	.	.	.	.
T12df	chrX	**1**	**12.5**	6624078	6624080	CAA	-	exonic	**Shroom4**	ENSMUSP00000141379	.	nonframeshift deletion	Shroom4:NM_001313765:exon5:c.3001_3003del:p.1001_1001del,Shroom4:NM_001040459:exon6:c.3349_3351del:p.1117_1117del,Shroom4:NM_001313764:exon6:c.3196_3198del:p.1066_1066del	**1001_1001del**	NA	X,6624078,GCAA,G	XqA1.1	rs236965363	0.25	.	34	chrX	6624077	.	GCAA	G	.	str_contraction;t_lod_fstar	ECNT=1;HCNT=1;MAX_ED=.;MIN_ED=.;NLOD=8.71;RPA=8,7;RU=CAA;STR;TLOD=4.59	GT:AD:AF:ALT_F1R2:ALT_F2R1:FOXOG:QSS:REF_F1R2:REF_F2R1	0/1:41,2:0.050:1:1:.:1174,56:22:19	0/0:34,0:0.00:0:0:.:940,0:18:16	1.3	Unknown	.	.	.	.	.	.	.	.
T12df	chrX	**1**	**12.5**	6624078	6624080	CAA	-	exonic	**Shroom4**	ENSMUSP00000141379	.	nonframeshift deletion	Shroom4:NM_001313765:exon5:c.3001_3003del:p.1001_1001del,Shroom4:NM_001040459:exon6:c.3349_3351del:p.1117_1117del,Shroom4:NM_001313764:exon6:c.3196_3198del:p.1066_1066del	**1066_1066del**	NA	X,6624078,GCAA,T	XqA1.1	rs236965363	0.25	.	34	chrX	6624077	.	GCAA	T	.	str_contraction;t_lod_fstar	ECNT=1;HCNT=1;MAX_ED=.;MIN_ED=.;NLOD=10.48;RPA=8,7;RU=TGC;STR;TLOD=4.46	GT:AD:AF:ALT_F1R2:ALT_F2R1:FOXOG:QSS:REF_F1R2:REF_F2R1	0/1:44,2:0.050:0:2:.:1249,56:18:26	0/0:41,0:0.00:0:0:.:1160,0:21:20	2.5	Unknown	.	.	.	.	.	.	.	.
T12df	chr10	**1**	**12.5**	89606710	89606748	TTGGGGATGCCGCAGCAGCTGCAGTCACAGATGTACCGC	-	exonic	**Slc17a8**	ENSMUSP00000114836	.	nonframeshift deletion	Slc17a8:NM_182959:exon2:c.222_260del:p.74_87del	**74_87del**	NA	10,89606710,TTTGGGGATGCCGCAGCAGCTGCAGTCACAGATGTACCGC,AGCCC	10qC2	.	0.25	.	37	chr10	89606709	.	TTTGGGGATGCCGCAGCAGCTGCAGTCACAGATGTACCGC	AGCCC	.	alt_allele_in_normal;clustered_events	ECNT=2;HCNT=20;MAX_ED=30;MIN_ED=30;NLOD=11.52;RPA=2,3;RU=GCCC;STR;TLOD=21.47	GT:AD:AF:ALT_F1R2:ALT_F2R1:FOXOG:QSS:REF_F1R2:REF_F2R1	0/1:68,7:0.096:4:3:.:1911,197:35:33	0/0:72,3:0.043:1:2:.:2004,86:39:33	0.7	Unknown	NA	NA	162	30	NA	NA	NA	NA
T42df	chrX	**1**	**12.5**	36757876	36757876	-	A	exonic	**Slc25a43**	ENSMUSP00000153241	.	stopgain	Slc25a43:NM_001085497:exon3:c.626_627insA:p.C209_V210delinsX	**C209_V210delinsX**	ENSMUSP00000153241,209,CV,X	NA	XqA3.3	.	0.25	.	37	chrX	36757876	.	G	T	.	str_contraction;t_lod_fstar	ECNT=1;HCNT=1;MAX_ED=.;MIN_ED=.;NLOD=16.20;RPA=5,4;RU=GAG;STR;TLOD=5.22	GT:AD:AF:ALT_F1R2:ALT_F2R1:FOXOG:QSS:REF_F1R2:REF_F2R1	0/1:59,2:0.034:2:0:.:1582,56:31:28	0/0:61,0:0.00:0:0:.:1573,0:38:23	1.3	Deleterious	.	.	.	.	.	.	.	.
T42df	chrX	**1**	**12.5**	36757878	36757878	-	A	exonic	**Slc25a43**	ENSMUSP00000153241	.	frameshift insertion	Slc25a43:NM_001085497:exon3:c.628_629insA:p.V210fs	**V210fs**	ENSMUSP00000153241,210,V,	X,36757878,G,A	XqA3.3	.	0.25	.	37	chrX	36757878	.	G	A	.	t_lod_fstar	ECNT=1;HCNT=1;MAX_ED=.;MIN_ED=.;NLOD=10.83;TLOD=4.20	GT:AD:AF:ALT_F1R2:ALT_F2R1:FOXOG:QSS:REF_F1R2:REF_F2R1	0/1:53,2:0.038:0:2:.:1549,58:23:30	0/0:39,0:0.00:0:0:.:1114,0:16:23	1.8	Tolerated	-0.15	Neutral	126	30	0.057	Tolerated	2.81	91

T4df	chrX	**1**	**12.5**	137015286	137015313	ACATGGCTCCCACAGCAGGTACAGCGGC	-	exonic	**Slc25a53**	ENSMUSP00000043437	.	frameshift deletion	Slc25a53:NM_001082412:exon6:c.33_60del:p.V11fs	**V11fs**	ENSMUSP00000043437,11,V,.	X,137015286,TACATGGCTCCCACAGCAGGTACAGCGGC,G	XqF1	.	0.25	.	45	chrX	137015285	.	TACATGGCTCCCACAGCAGGTACAGCGGC	G	.	clustered_events;multi_event_alt_allele_in_normal	ECNT=8;HCNT=1;MAX_ED=14;MIN_ED=1;NLOD=6.64;TLOD=11.68	GT:AD:AF:ALT_F1R2:ALT_F2R1:FOXOG:PGT:PID:QSS:REF_F1R2:REF_F2R1	0/1:54,4:0.069:4:0:0.00:0|1:76615201_C_T:1411,110:27:27	0/0:38,1:0.027:1:0:0.00:0|1:76615201_C_T:1013,28:20:18	19	Unknown	NA	NA	129	30	NA	NA	NA	NA
T42df	chr15	**1**	**12.5**	76615207	76615207	C	G	exonic	**Slc39a4**	ENSMUSP00000133061	.	nonsynonymous SNV	Slc39a4:NM_028064:exon3:c.G507C:p.E169D	**E169D**	ENSMUSP00000133061,169,E,D	NA	15qD3	.	0.25	.	39	chr15	76615207	.	C	A	.	clustered_events;multi_event_alt_allele_in_normal	ECNT=8;HCNT=1;MAX_ED=14;MIN_ED=1;NLOD=6.64;TLOD=11.71	GT:AD:AF:ALT_F1R2:ALT_F2R1:FOXOG:PGT:PID:QSS:REF_F1R2:REF_F2R1	0/1:54,4:0.070:4:0:1.00:0|1:76615201_C_T:1452,78:27:27	0/0:37,1:0.027:1:0:1.00:0|1:76615201_C_T:1026,28:20:17	19	Damaging	-2.12	Neutral	135	30	0.012	Damaging	2.8	66
T42df	chr15	**1**	**12.5**	76615208	76615208	T	A	exonic	**Slc39a4**	ENSMUSP00000133061	.	nonsynonymous SNV	Slc39a4:NM_028064:exon3:c.A506T:p.E169V	**E169V**	ENSMUSP00000133061,169,E,V	NA	15qD3	.	0.25	.	39	chr15	76615208	.	T	C	.	clustered_events;multi_event_alt_allele_in_normal	ECNT=8;HCNT=1;MAX_ED=14;MIN_ED=1;NLOD=6.04;TLOD=11.71	GT:AD:AF:ALT_F1R2:ALT_F2R1:FOXOG:PGT:PID:QSS:REF_F1R2:REF_F2R1	0/1:54,4:0.070:4:0:.:0|1:76615201_C_T:1400,82:27:27	0/0:37,1:0.029:1:0:.:0|1:76615201_C_T:1010,22:20:17	19	Deleterious	-4.54	Deleterious	135	30	0.001	Damaging	2.8	66
T42df	chr15	**1**	**12.5**	76615211	76615211	T	A	exonic	**Slc39a4**	ENSMUSP00000133061	.	nonsynonymous SNV	Slc39a4:NM_028064:exon3:c.A503T:p.E168V	**E168V**	ENSMUSP00000133061,168,E,V	15,76615211,T,G	15qD3	.	0.25	.	38	chr15	76615211	.	T	G	.	clustered_events;multi_event_alt_allele_in_normal	ECNT=8;HCNT=1;MAX_ED=14;MIN_ED=1;NLOD=6.94;TLOD=11.59	GT:AD:AF:ALT_F1R2:ALT_F2R1:FOXOG:PGT:PID:QSS:REF_F1R2:REF_F2R1	0/1:54,4:0.066:4:0:.:0|1:76615201_C_T:1534,109:26:28	0/0:40,1:0.026:1:0:.:0|1:76615201_C_T:1139,27:21:19	19	Tolerated	-2.06	Neutral	115	30	0.065	Tolerated	2.82	52
T42df	chr15	**1**	**12.5**	76615202	76615202	-	TTTTAATGATA	exonic	**Slc39a4**	ENSMUSP00000133061	.	frameshift insertion	Slc39a4:NM_028064:exon3:c.511_512insTATCATTAAAA:p.E171fs	**E171fs**	ENSMUSP00000133061,171,E,	15,76615202,T,TG	15qD3	.	0.25	.	39	chr15	76615202	.	T	TG	.	t_lod_fstar	ECNT=1;HCNT=1;MAX_ED=.;MIN_ED=.;NLOD=9.33;TLOD=6.22	GT:AD:AF:ALT_F1R2:ALT_F2R1:FOXOG:QSS:REF_F1R2:REF_F2R1	0/1:31,3:0.088:0:3:.:858,87:10:21	0/0:35,0:0.00:0:0:.:953,0:18:17	4	Unknown	NA	NA	115	30	NA	NA	NA	NA
T42df	chr15	**1**	**12.5**	76615214	76615214	A	-	exonic	**Slc39a4**	ENSMUSP00000133061	.	frameshift deletion	Slc39a4:NM_028064:exon3:c.500delT:p.L167fs	**L167fs**	ENSMUSP00000133061,167,L,.	15,76615214,CA,TTTTTAATGATA	15qD3	.	0.25	.	38	chr15	76615213	.	CA	TTTTTAATGATA	.	clustered_events;multi_event_alt_allele_in_normal	ECNT=8;HCNT=1;MAX_ED=14;MIN_ED=1;NLOD=6.94;TLOD=11.65	GT:AD:AF:ALT_F1R2:ALT_F2R1:FOXOG:PGT:PID:QSS:REF_F1R2:REF_F2R1	0/1:57,4:0.068:4:0:.:0|1:76615201_C_T:1609,108:29:28	0/0:38,1:0.026:1:0:.:0|1:76615201_C_T:1084,27:19:19	19	Unknown	NA	NA	115	30	NA	NA	NA	NA
T42df	chr15	**1**	**12.5**	76615216	76615225	CAGTTGGGGA	-	exonic	**Slc39a4**	ENSMUSP00000133061	.	frameshift deletion	Slc39a4:NM_028064:exon3:c.489_498del:p.L163fs	**L163fs**	ENSMUSP00000133061,163,L,.	15,76615216,GCAGTTGGGGA,A	15qD3	.	0.25	.	41	chr15	76615215	.	GCAGTTGGGGA	A	.	alt_allele_in_normal;germline_risk	ECNT=1;HCNT=2;MAX_ED=.;MIN_ED=.;NLOD=2.11;TLOD=8.54	GT:AD:AF:ALT_F1R2:ALT_F2R1:FOXOG:QSS:REF_F1R2:REF_F2R1	0/1:67,5:0.070:2:3:0.400:1921,140:39:28	0/0:44,4:0.093:2:2:0.500:1260,99:22:22	0.7	Unknown	NA	NA	115	30	NA	NA	NA	NA
T32df	chr13	**1**	**12.5**	73668173	73668173	-	G	exonic	**Slc6a18**	ENSMUSP00000021056	.	frameshift insertion	Slc6a18:NM_001040692:exon8:c.1085_1086insC:p.Q362fs,Slc6a18:NM_001136087:exon8:c.1085_1086insC:p.Q362fs,Slc6a18:NM_001168643:exon8:c.1085_1086insC:p.Q362fs,Slc6a18:NM_001168644:exon8:c.1085_1086insC:p.Q362fs,Slc6a18:NM_001168645:exon8:c.1085_1086insC:p.Q362fs,Slc6a18:NM_001168646:exon8:c.1085_1086insC:p.Q362fs	**Q362fs**	ENSMUSP00000021056,362,Q,	13,73668173,T,T	13qC1	.	0.25	.	35	chr13	73668173	.	T	T	.	clustered_events;multi_event_alt_allele_in_normal	ECNT=9;HCNT=1;MAX_ED=32;MIN_ED=2;NLOD=6.94;TLOD=8.37	GT:AD:AF:ALT_F1R2:ALT_F2R1:FOXOG:PGT:PID:QSS:REF_F1R2:REF_F2R1	0/1:57,3:0.052:3:0:1.00:0|1:74166020_G_GATTTTTTA:1534,79:30:27	0/0:44,1:0.026:1:0:1.00:0|1:74166020_G_GATTTTTTA:1161,28:27:17	4	Tolerated	0	Neutral	175	30	0.845	Tolerated	2.83	202

T3df	chr7	**1**	**12.5**	35457091	35457091	-	ACCTGCTTTACAGCCGGCAGGTACCATTAGCAGCATCTTGGGTAGTCCCACTTTTTGTGGCATTTTCAACCATCGGTGCTGCTAATGGC	exonic	**Slc7a9**	ENSMUSP00000000175	.	stopgain	Slc7a9:NM_001199016:exon9:c.957_958insACCTGCTTTACAGCCGGCAGGTACCATTAGCAGCATCTTGGGTAGTCCCACTTTTTGTGGCATTTTCAACCATCGGTGCTGCTAATGGC:p.G319_T320delinsGTCFTAGRYHX,Slc7a9:NM_021291:exon9:c.957_958insACCTGCTTTACAGCCGGCAGGTACCATTAGCAGCATCTTGGGTAGTCCCACTTTTTGTGGCATTTTCAACCATCGGTGCTGCTAATGGC:p.G319_T320delinsGTCFTAGRYHX,Slc7a9:NM_001199015:exon10:c.957_958insACCTGCTTTACAGCCGGCAGGTACCATTAGCAGCATCTTGGGTAGTCCCACTTTTTGTGGCATTTTCAACCATCGGTGCTGCTAATGGC:p.G319_T320delinsGTCFTAGRYHX	**G319_T320delinsGTCFTAGRYHX**	ENSMUSP00000000175,319,GT,GTCFTAGRYHX	NA	7qB2	.	0.25	.	32	chr7	35457091	.	T	A	.	clustered_events;multi_event_alt_allele_in_normal	ECNT=9;HCNT=1;MAX_ED=32;MIN_ED=2;NLOD=7.54;TLOD=8.49	GT:AD:AF:ALT_F1R2:ALT_F2R1:FOXOG:PGT:PID:QSS:REF_F1R2:REF_F2R1	0/1:54,3:0.057:3:0:1.00:0|1:74166020_G_GATTTTTTA:1496,51:29:25	0/0:43,1:0.025:1:0:1.00:0|1:74166020_G_GATTTTTTA:1143,22:24:19	4	Deletarious	.	.	.	.	.	.	.	.
T12df	chr13	**1**	**12.5**	74166024	74166024	-	T	exonic	**Slc9a3**	ENSMUSP00000065084	.	frameshift insertion	Slc9a3:NM_001081060:exon16:c.2450_2451insT:p.E817fs	**E817fs**	ENSMUSP00000065084,817,E,	13,74166024,A,T	13qC1	.	0.25	.	45	chr13	74166024	.	A	T	.	PASS	DB;ECNT=1;HCNT=1;MAX_ED=.;MIN_ED=.;NLOD=13.83;TLOD=45.22	GT:AD:AF:ALT_F1R2:ALT_F2R1:FOXOG:QSS:REF_F1R2:REF_F2R1	0/1:27,18:0.409:12:6:0.667:778,496:13:14	0/0:53,0:0.00:0:0:.:1496,0:22:31	1.1	Damaging	-2.27	Neutral	168	30	0.004	Damaging	3.41	43
T12df	chr13	**1**	**12.5**	74166022	74166022	G	T	exonic	**Slc9a3**	ENSMUSP00000065084	.	nonsynonymous SNV	Slc9a3:NM_001081060:exon16:c.G2448T:p.E816D	**E816D**	ENSMUSP00000065084,816,E,D	13,74166022,G,A	13qC1	.	0.25	.	45	chr13	74166022	.	G	A	.	clustered_events;multi_event_alt_allele_in_normal	ECNT=9;HCNT=1;MAX_ED=32;MIN_ED=2;NLOD=7.54;TLOD=8.47	GT:AD:AF:ALT_F1R2:ALT_F2R1:FOXOG:PGT:PID:QSS:REF_F1R2:REF_F2R1	0/1:54,3:0.056:3:0:0.00:0|1:74166020_G_GATTTTTTA:1486,39:29:25	0/0:44,1:0.025:1:0:0.00:0|1:74166020_G_GATTTTTTA:1212,23:24:20	4	Tolerated	0	Neutral	168	30	1	Tolerated	3.41	43
T12df	chr13	**1**	**12.5**	74166041	74166041	G	A	exonic	**Slc9a3**	ENSMUSP00000065084	.	nonsynonymous SNV	Slc9a3:NM_001081060:exon16:c.G2467A:p.A823T	**A823T**	ENSMUSP00000065084,823,A,T	13,74166041,G,A	13qC1	.	0.25	.	44	chr13	74166041	.	G	A	.	clustered_events;multi_event_alt_allele_in_normal	ECNT=9;HCNT=1;MAX_ED=32;MIN_ED=2;NLOD=8.14;TLOD=8.28	GT:AD:AF:ALT_F1R2:ALT_F2R1:FOXOG:PGT:PID:QSS:REF_F1R2:REF_F2R1	0/1:58,3:0.048:3:0:.:0|1:74166020_G_GATTTTTTA:1565,82:31:27	0/0:47,1:0.024:1:0:.:0|1:74166020_G_GATTTTTTA:1248,27:28:19	4	Tolerated	0.01	Neutral	168	30	0.566	Tolerated	3.47	41
T12df	chr13	**1**	**12.5**	74166049	74166049	-	A	exonic	**Slc9a3**	ENSMUSP00000065084	.	frameshift insertion	Slc9a3:NM_001081060:exon16:c.2475_2476insA:p.E825fs	**E825fs**	ENSMUSP00000065084,825,E,	13,74166049,G,T	13qC1	.	0.25	.	46	chr13	74166049	.	G	T	.	PASS	DB;ECNT=1;HCNT=1;MAX_ED=.;MIN_ED=.;NLOD=13.83;TLOD=45.22	GT:AD:AF:ALT_F1R2:ALT_F2R1:FOXOG:QSS:REF_F1R2:REF_F2R1	0/1:27,18:0.409:12:6:0.667:778,496:13:14	0/0:53,0:0.00:0:0:.:1496,0:22:31	1.1	Tolerated	-1.01	Neutral	168	30	0.126	Tolerated	3.63	41
T12df	chr13	**1**	**12.5**	74166044	74166044	C	A	exonic	**Slc9a3**	ENSMUSP00000065084	.	nonsynonymous SNV	Slc9a3:NM_001081060:exon16:c.C2470A:p.P824T	**P824T**	ENSMUSP00000065084,824,P,T	13,74166044,C,GATTTTTTA	13qC1	.	0.25	.	45	chr13	74166044	.	C	GATTTTTTA	.	clustered_events;multi_event_alt_allele_in_normal	ECNT=9;HCNT=1;MAX_ED=32;MIN_ED=2;NLOD=6.94;TLOD=8.40	GT:AD:AF:ALT_F1R2:ALT_F2R1:FOXOG:PGT:PID:QSS:REF_F1R2:REF_F2R1	0/1:56,3:0.053:3:0:.:0|1:74166020_G_GATTTTTTA:1389,57:29:27	0/0:44,1:0.026:1:0:.:0|1:74166020_G_GATTTTTTA:1071,24:27:17	4	Unknown	NA	NA	168	30	NA	NA	NA	NA
T12df	chr13	**1**	**12.5**	74166020	74166020	-	ATTTTTTA	exonic	**Slc9a3**	ENSMUSP00000065084	.	frameshift insertion	Slc9a3:NM_001081060:exon16:c.2446_2447insATTTTTTA:p.E816fs	**E816fs**	ENSMUSP00000065084,816,E,	13,74166020,G,GA	13qC1	.	0.25	.	45	chr13	74166020	.	G	GA	.	clustered_events;multi_event_alt_allele_in_normal	ECNT=9;HCNT=1;MAX_ED=32;MIN_ED=2;NLOD=7.24;TLOD=8.33	GT:AD:AF:ALT_F1R2:ALT_F2R1:FOXOG:PGT:PID:QSS:REF_F1R2:REF_F2R1	0/1:57,3:0.050:3:0:.:0|1:74166020_G_GATTTTTTA:1513,66:30:27	0/0:45,1:0.026:1:0:.:0|1:74166020_G_GATTTTTTA:1138,28:25:20	4	Unknown	NA	NA	168	30	NA	NA	NA	NA
T12df	chr13	**1**	**12.5**	74166026	74166035	CAGCTCCAAC	-	exonic	**Slc9a3**	ENSMUSP00000065084	.	frameshift deletion	Slc9a3:NM_001081060:exon16:c.2452_2461del:p.Q818fs	**Q818fs**	ENSMUSP00000065084,818,Q,.	13,74166026,ACAGCTCCAAC,AT	13qC1	.	0.25	.	48	chr13	74166025	.	ACAGCTCCAAC	AT	.	clustered_events;multi_event_alt_allele_in_normal	ECNT=9;HCNT=1;MAX_ED=32;MIN_ED=2;NLOD=6.94;TLOD=8.35	GT:AD:AF:ALT_F1R2:ALT_F2R1:FOXOG:PGT:PID:QSS:REF_F1R2:REF_F2R1	0/1:56,3:0.051:3:0:.:0|1:74166020_G_GATTTTTTA:1482,38:30:26	0/0:44,1:0.026:1:0:.:0|1:74166020_G_GATTTTTTA:1147,11:27:17	4	Unknown	.	.	.	.	.	.	.	.

T3df	chr7	**1**	**12.5**	59779721	59779721	G	A	ncRNA_exonic	**Snhg14**	NONE	.	.	.	**NA**	.	.	7qB5	.	0.25	.	43	chr7	59779721	.	G	A	.	alt_allele_in_normal;t_lod_fstar	ECNT=1;HCNT=1;MAX_ED=.;MIN_ED=.;NLOD=7.51;TLOD=6.11	GT:AD:AF:ALT_F1R2:ALT_F2R1:FOXOG:QSS:REF_F1R2:REF_F2R1	0/1:52,5:0.089:5:0:1.00:1397,135:24:28	0/0:41,2:0.051:0:2:0.00:1095,55:23:18	0.5	unknown	.	.	.	.	.	.	.	.
T1df	chr7	**1**	**12.5**	59779721	59779721	G	A	ncRNA_exonic	**Snhg14**	NONE	.	.	.	**NA**	7,59779721,G,A	NA	7qB5	.	0.25	.	43	chr7	59779721	.	G	A	.	alt_allele_in_normal;t_lod_fstar	ECNT=1;HCNT=21;MAX_ED=.;MIN_ED=.;NLOD=9.71;TLOD=4.09	GT:AD:AF:ALT_F1R2:ALT_F2R1:FOXOG:QSS:REF_F1R2:REF_F2R1	0/1:42,4:0.089:2:2:0.500:1195,108:22:20	0/0:109,7:0.064:3:4:0.429:3049,179:60:49	0.5	Unkown	.	.	.	.	.	.	.	.
T22df	chr7	**1**	**12.5**	59731692	59731692	T	A	ncRNA_exonic	**Snhg14**	NONE	.	.	.	**NA**	7,59731692,T,T	NA	7qB5	.	0.25	.	116	chr7	59731692	.	T	T	.	PASS	ECNT=1;HCNT=2;MAX_ED=.;MIN_ED=.;NLOD=12.54;TLOD=20.11	GT:AD:AF:ALT_F1R2:ALT_F2R1:FOXOG:QSS:REF_F1R2:REF_F2R1	0/1:35,9:0.209:5:4:0.556:1004,254:16:19	0/0:46,0:0.00:0:0:.:1262,0:26:20	24	Unkown	.	.	.	.	.	.	.	.
T42df	chr7	**1**	**12.5**	59766995	59766995	C	A	ncRNA_exonic	**Snhg14**	NONE	.	.	.	**NA**	7,59766995,C,T	NA	7qB5	.	0.25	.	277	chr7	59766995	.	C	T	.	alt_allele_in_normal;clustered_events;t_lod_fstar	ECNT=4;HCNT=6;MAX_ED=89;MIN_ED=10;NLOD=42.30;TLOD=5.69	GT:AD:AF:ALT_F1R2:ALT_F2R1:FOXOG:PGT:PID:QSS:REF_F1R2:REF_F2R1	0/1:249,3:0.013:2:1:0.333:0|1:59767078_C_T:7067,86:112:137	0/0:386,11:0.032:5:6:0.545:0|1:59767078_C_T:10773,303:195:191	0.5	Unkown	.	.	.	.	.	.	.	.
T42df	chr7	**1**	**12.5**	59767005	59767005	G	T	ncRNA_exonic	**Snhg14**	NONE	.	.	.	**NA**	7,59767005,G,T	NA	7qB5	.	0.25	.	302	chr7	59767005	.	G	T	.	alt_allele_in_normal;clustered_events;t_lod_fstar	ECNT=4;HCNT=6;MAX_ED=89;MIN_ED=10;NLOD=45.31;TLOD=5.63	GT:AD:AF:ALT_F1R2:ALT_F2R1:FOXOG:PGT:PID:QSS:REF_F1R2:REF_F2R1	0/1:251,3:0.012:2:1:0.333:0|1:59767078_C_T:6947,66:118:133	0/0:404,11:0.031:5:6:0.545:0|1:59767078_C_T:10938,312:204:200	0.5	Unkown	.	.	.	.	.	.	.	.
T42df	chr7	**1**	**12.5**	59767078	59767078	C	T	ncRNA_exonic	**Snhg14**	NONE	.	.	.	**NA**	7,59767078,C,G	NA	7qB5	.	0.25	.	397	chr7	59767078	.	C	G	.	t_lod_fstar	ECNT=1;HCNT=1;MAX_ED=.;MIN_ED=.;NLOD=9.18;TLOD=4.95	GT:AD:AF:ALT_F1R2:ALT_F2R1:FOXOG:QSS:REF_F1R2:REF_F2R1	0/1:39,2:0.049:1:1:.:957,52:20:19	0/0:35,0:0.00:0:0:.:856,0:21:14	0.2	Unkown	.	.	.	.	.	.	.	.
T42df	chr7	**1**	**12.5**	59767084	59767084	C	T	ncRNA_exonic	**Snhg14**	NONE	.	.	.	**NA**	7,59767084,C,T	NA	7qB5	.	0.25	.	415	chr7	59767084	.	C	T	.	alt_allele_in_normal;clustered_events;t_lod_fstar	ECNT=2;HCNT=3;MAX_ED=6;MIN_ED=6;NLOD=16.28;TLOD=5.39	GT:AD:AF:ALT_F1R2:ALT_F2R1:FOXOG:PGT:PID:QSS:REF_F1R2:REF_F2R1	0/1:48,2:0.042:0:2:1.00:0|1:59731690_C_T:1361,56:19:29	0/0:117,2:0.017:1:1:0.500:0|1:59731690_C_T:3192,57:63:54	0.5	Unkown	.	.	.	.	.	.	.	.
T4df	chr7	**1**	**12.5**	59731690	59731690	C	T	ncRNA_exonic	**Snhg14**	NONE	.	.	.	**NA**	7,59731690,C,	NA	7qB5	.	0.25	.	119	chr7	59731690	.	C	.	.	.	.	.	.	.	.	Unkown	.	.	.	.	.	.	.	.
T4df	chr7	**1**	**12.5**	59731696	59731696	C	T	ncRNA_exonic	**Snhg14**	NONE	.	.	.	**NA**	7,59731696,C,	NA	7qB5	.	0.25	.	122	chr7	59731696	.	C	.	.	.	.	.	.	.	.	Unkown	.	.	.	.	.	.	.	.
T1df	chr2	**1**	**12.5**	158355065	158355065	A	-	ncRNA_exonic	**Snhg17**	NONE	.	.	.	**NA**	2,158355065,GA,A	NA	2qH1	.	0.25	.	41	chr2	158355064	.	GA	A	.	t_lod_fstar	ECNT=1;HCNT=1;MAX_ED=.;MIN_ED=.;NLOD=17.04;TLOD=6.17	GT:AD:AF:ALT_F1R2:ALT_F2R1:FOXOG:QSS:REF_F1R2:REF_F2R1	0/1:52,4:0.058:0:4:.:1403,110:28:24	0/0:63,0:0.00:0:0:.:1700,0:34:29	0.5	Unkown	.	.	.	.	.	.	.	.
T1df	chr3	**1**	**12.5**	117837254	117837254	-	TG	exonic	**Snx7**	ENSMUSP00000031090	.	frameshift insertion	Snx7:NM_001190156:exon5:c.703_704insCA:p.R235fs,Snx7:NM_029655:exon5:c.703_704insCA:p.R235fs	**R235fs**	ENSMUSP00000031090,235,R,	3,117837254,C,C	3qG1	.	0.25	.	36	chr3	117837254	.	C	C	.	alt_allele_in_normal;clustered_events	ECNT=15;HCNT=7;MAX_ED=140;MIN_ED=12;NLOD=43.57;TLOD=24.68	GT:AD:AF:ALT_F1R2:ALT_F2R1:FOXOG:PGT:PID:QSS:REF_F1R2:REF_F2R1	0/1:193,9:0.046:5:4:0.556:0|1:85610752_C_T:5488,270:100:93	0/0:433,23:0.044:9:14:0.391:0|1:85610752_C_T:12222,663:208:225	0.2	Tolerated	0	Neutral	140	30	1	Tolerated	2.94	123
T42df	chr1	**1**	**12.5**	85594359	85594359	C	G	exonic	**Sp110**	ENSMUSP00000040248	.	nonsynonymous SNV	Sp110:NM_030194:exon3:c.G222C:p.Q74H,Sp110:NM_175397:exon4:c.G222C:p.Q74H	**Q74H**	ENSMUSP00000040248,74,Q,H	1,85594359,C,A	1qC5	.	0.25	.	293	chr1	85594359	.	C	A	.	t_lod_fstar	ECNT=1;HCNT=1;MAX_ED=.;MIN_ED=.;NLOD=10.73;TLOD=4.10	GT:AD:AF:ALT_F1R2:ALT_F2R1:FOXOG:QSS:REF_F1R2:REF_F2R1	0/1:33,3:0.057:3:0:.:875,87:24:9	0/0:43,0:0.00:0:0:.:1149,0:23:20	1.6	Damaging	-2.01	Neutral	104	30	0.002	Damaging	2.79	110

T2df	chr1	**1**	**12.5**	85620115	85620115	G	A	exonic	**Sp140**	ENSMUSP00000109115	.	nonsynonymous SNV	Sp140:NM_001013817:exon6:c.G562A:p.V188M	**V188M**	ENSMUSP00000109115,188,V,M	1,85620115,G,A	1qC5	.	0.25	.	42	chr1	85620115	.	G	A	.	alt_allele_in_normal;clustered_events;t_lod_fstar	ECNT=10;HCNT=1;MAX_ED=96;MIN_ED=3;NLOD=59.36;TLOD=4.55	GT:AD:AF:ALT_F1R2:ALT_F2R1:FOXOG:PGT:PID:QSS:REF_F1R2:REF_F2R1	0/1:124,2:0.016:1:1:0.500:0|1:85610874_G_A:3368,60:60:64	0/0:297,9:0.032:3:6:0.333:0|1:85610874_G_A:7559,244:128:169	0.2	Damaging	-1.99	Neutral	114	30	0.015	Damaging	3.07	247
T32df	chr1	**1**	**12.5**	85610875	85610875	A	G	exonic	**Sp140**	ENSMUSP00000108782	.	nonsynonymous SNV	Sp140:NM_001013817:exon3:c.A332G:p.D111G	**D111G**	ENSMUSP00000108782,111,D,G	1,85610875,A,C	1qC5	.	0.25	.	302	chr1	85610875	.	A	C	.	alt_allele_in_normal;clustered_events	ECNT=4;HCNT=12;MAX_ED=58;MIN_ED=2;NLOD=14.31;TLOD=8.22	GT:AD:AF:ALT_F1R2:ALT_F2R1:FOXOG:QSS:REF_F1R2:REF_F2R1	0/1:50,5:0.091:5:0:1.00:1435,137:28:22	0/0:256,19:0.070:8:11:0.421:7197,519:147:109	0.2	Deleterious	-5.63	Deleterious	93	30	0.056	Tolerated	2.86	66
T32df	chr1	**1**	**12.5**	85610874	85610874	G	A	exonic	**Sp140**	ENSMUSP00000108782	.	nonsynonymous SNV	Sp140:NM_001013817:exon3:c.G331A:p.D111N	**D111N**	ENSMUSP00000108782,111,D,N	1,85610874,G,A	1qC5	.	0.25	.	306	chr1	85610874	.	G	A	.	alt_allele_in_normal;clustered_events;t_lod_fstar	ECNT=10;HCNT=1;MAX_ED=96;MIN_ED=3;NLOD=53.35;TLOD=4.61	GT:AD:AF:ALT_F1R2:ALT_F2R1:FOXOG:PGT:PID:QSS:REF_F1R2:REF_F2R1	0/1:116,2:0.017:1:1:0.500:0|1:85610874_G_A:3241,59:59:57	0/0:285,9:0.033:3:6:0.333:0|1:85610874_G_A:8008,257:123:162	0.2	Deleterious	-3.46	Deleterious	93	30	0.319	Tolerated	2.86	66
T32df	chr1	**1**	**12.5**	85632585	85632585	C	T	exonic	**Sp140**	ENSMUSP00000108782	.	nonsynonymous SNV	Sp140:NM_001013817:exon10:c.C937T:p.R313C	**R313C**	ENSMUSP00000108782,313,R,C	1,85632585,C,A	1qC5	.	0.25	.	144	chr1	85632585	.	C	A	.	alt_allele_in_normal	ECNT=1;HCNT=5;MAX_ED=.;MIN_ED=.;NLOD=43.98;TLOD=8.99	GT:AD:AF:ALT_F1R2:ALT_F2R1:FOXOG:QSS:REF_F1R2:REF_F2R1	0/1:99,7:0.066:4:3:0.571:2838,204:48:51	0/0:650,47:0.068:27:20:0.574:18685,1303:318:332	0.2	Deleterious	-4.83	Deleterious	93	30	0.002	Damaging	2.79	92
T42df	chr1	**1**	**12.5**	85609716	85609716	G	A	exonic	**Sp140**	ENSMUSP00000108782	.	nonsynonymous SNV	Sp140:NM_001013817:exon2:c.G55A:p.E19K	**E19K**	ENSMUSP00000108782,19,E,K	1,85609716,G,C	1qC5	.	0.25	.	697	chr1	85609716	.	G	C	.	PASS	ECNT=1;HCNT=1;MAX_ED=.;MIN_ED=.;NLOD=9.32;TLOD=13.05	GT:AD:AF:ALT_F1R2:ALT_F2R1:FOXOG:QSS:REF_F1R2:REF_F2R1	0/1:25,6:0.200:2:4:0.333:707,167:11:14	0/0:35,0:0.00:0:0:.:964,0:14:21	0.4	Tolerated	-0.37	Neutral	93	30	0.228	Tolerated	3.05	43
T32df	chr1	**1**	**12.5**	85610901	85610901	T	A	exonic	**Sp140**	ENSMUSP00000108782	.	nonsynonymous SNV	Sp140:NM_001013817:exon3:c.T358A:p.F120I	**F120I**	ENSMUSP00000108782,120,F,I	1,85610901,T,T	1qC5	.	0.25	.	294	chr1	85610901	.	T	T	.	alt_allele_in_normal;clustered_events	ECNT=4;HCNT=9;MAX_ED=58;MIN_ED=2;NLOD=10.80;TLOD=11.06	GT:AD:AF:ALT_F1R2:ALT_F2R1:FOXOG:QSS:REF_F1R2:REF_F2R1	0/1:35,6:0.150:4:2:0.333:964,174:20:14	0/0:130,14:0.082:10:4:0.286:3332,399:64:66	0.2	Tolerated	0	Neutral	93	30	0.419	Tolerated	3.41	12
T32df	chr1	**1**	**12.5**	85632586	85632586	G	A	exonic	**Sp140**	ENSMUSP00000108782	.	nonsynonymous SNV	Sp140:NM_001013817:exon10:c.G938A:p.R313H	**R313H**	ENSMUSP00000108782,313,R,H	1,85632586,G,G	1qC5	.	0.25	.	296	chr1	85632586	.	G	G	.	PASS	ECNT=1;HCNT=1;MAX_ED=.;MIN_ED=.;NLOD=7.22;TLOD=15.12	GT:AD:AF:ALT_F1R2:ALT_F2R1:FOXOG:QSS:REF_F1R2:REF_F2R1	0/1:16,7:0.318:5:2:0.286:464,190:7:9	0/0:26,0:0.00:0:0:.:720,0:9:17	2.7	Tolerated	0	Neutral	93	30	1	Tolerated	2.79	92
T32df	chr1	**1**	**12.5**	85632528	85632528	T	C	exonic	**Sp140**	ENSMUSP00000108782	.	nonsynonymous SNV	Sp140:NM_001013817:exon10:c.T880C:p.S294P	**S294P**	ENSMUSP00000108782,294,S,P	1,85632528,T,A	1qC5	rs108497721	0.25	.	275	chr1	85632528	.	T	A	.	alt_allele_in_normal;clustered_events	ECNT=4;HCNT=9;MAX_ED=58;MIN_ED=2;NLOD=15.72;TLOD=8.19	GT:AD:AF:ALT_F1R2:ALT_F2R1:FOXOG:QSS:REF_F1R2:REF_F2R1	0/1:56,5:0.082:5:0:1.00:1468,149:28:28	0/0:281,15:0.054:3:12:0.200:7470,426:158:123	0.2	Tolerated	0.45	Neutral	93	30	0.211	Tolerated	2.79	92
T1df	chr1	**1**	**12.5**	85610809	85610811	AGA	-	exonic	**Sp140**	ENSMUSP00000108782	.	nonframeshift deletion	Sp140:NM_001013817:exon3:c.266_268del:p.89_90del	**89_90del**	NA	1,85610809,CAGA,T	1qC5	.	0.25	.	366	chr1	85610808	.	CAGA	T	.	alt_allele_in_normal;clustered_events	ECNT=6;HCNT=6;MAX_ED=122;MIN_ED=34;NLOD=47.61;TLOD=15.39	GT:AD:AF:ALT_F1R2:ALT_F2R1:FOXOG:QSS:REF_F1R2:REF_F2R1	0/1:190,6:0.031:2:4:0.333:5369,170:97:93	0/0:289,7:0.026:4:3:0.571:8328,200:138:151	0.2	Unknown	.	.	.	.	.	.	.	.
T32df	chr2	**1**	**12.5**	156065566	156065566	C	G	exonic	**Spag4**	ENSMUSP00000109117	.	nonsynonymous SNV	Spag4:NM_139151:exon1:c.C205G:p.P69A	**P69A**	ENSMUSP00000109117,69,P,A	2,156065566,C,A	2qH1	.	0.25	.	26	chr2	156065566	.	C	A	.	str_contraction	ECNT=1;HCNT=1;MAX_ED=.;MIN_ED=.;NLOD=8.08;RPA=9,8;RU=CTG;STR;TLOD=6.54	GT:AD:AF:ALT_F1R2:ALT_F2R1:FOXOG:QSS:REF_F1R2:REF_F2R1	0/1:42,3:0.068:3:0:.:1173,87:20:22	0/0:33,0:0.00:0:0:.:911,0:17:16	0.6	Damaging	-1.29	Neutral	173	30	0.003	Damaging	3.97	29

T32df	chr11	**1**	**12.5**	72529595	72529595	G	C	exonic	**Spns3**	ENSMUSP00000121309	.	nonsynonymous SNV	Spns3:NM_029932:exon8:c.C1064G:p.A355G	**A355G**	ENSMUSP00000121309,355,A,G	11,72529595,G,A	11qB4	.	0.25	.	35	chr11	72529595	.	G	A	.	alt_allele_in_normal;str_contraction	ECNT=1;HCNT=1;MAX_ED=.;MIN_ED=.;NLOD=4.80;RPA=2,1;RU=GGAGCC;STR;TLOD=9.06	GT:AD:AF:ALT_F1R2:ALT_F2R1:FOXOG:QSS:REF_F1R2:REF_F2R1	0/1:35,3:0.086:1:2:.:981,84:16:19	0/0:28,2:0.071:1:1:.:803,56:15:13	0.2	Tolerated	-1.13	Neutral	163	30	0.426	Tolerated	2.83	137
T2df	chr3	**1**	**12.5**	92437218	92437218	A	G	exonic	**Sprr1b**	ENSMUSP00000106107	.	nonsynonymous SNV	Sprr1b:NM_009265:exon2:c.T350C:p.V117A	**V117A**	ENSMUSP00000106107,117,V,A	3,92437218,A,C	3qF1	.	0.25	.	29	chr3	92437218	.	A	C	.	alt_allele_in_normal;clustered_events	ECNT=5;HCNT=1;MAX_ED=24;MIN_ED=1;NLOD=5.43;TLOD=11.84	GT:AD:AF:ALT_F1R2:ALT_F2R1:FOXOG:PGT:PID:QSS:REF_F1R2:REF_F2R1	0/1:50,4:0.075:4:0:.:0|1:136373432_T_TTTTTAATGATACG:1253,57:21:29	0/0:37,1:0.030:1:0:.:0|1:136373432_T_TTTTTAATGATACG:938,22:13:24	1.4	Deleterious	-4.25	Deleterious	92	30	0.003	Damaging	2.75	77
T12df	chr3	**1**	**12.5**	92317520	92317573	TGAGCCTTGTCCTCCTCCAAAGTGCCCTGAGCCTTGTCCTCCCCCAGTATGCTG	-	exonic	**Sprr2b**	ENSMUSP00000116058	.	nonframeshift deletion	Sprr2b:NM_011469:exon2:c.72_125del:p.24_42del	**24_42del**	NA	3,92317520,CTGAGCCTTGTCCTCCTCCAAAGTGCCCTGAGCCTTGTCCTCCCCCAGTATGCTG,A	3qF1	.	0.25	.	26	chr3	92317519	.	CTGAGCCTTGTCCTCCTCCAAAGTGCCCTGAGCCTTGTCCTCCCCCAGTATGCTG	A	.	str_contraction;t_lod_fstar	ECNT=1;HCNT=8;MAX_ED=.;MIN_ED=.;NLOD=8.42;RPA=7,6;RU=CAG;STR;TLOD=4.61	GT:AD:AF:ALT_F1R2:ALT_F2R1:FOXOG:QSS:REF_F1R2:REF_F2R1	0/1:59,2:0.033:0:2:.:1662,57:28:31	0/0:31,0:0.00:0:0:.:872,0:20:11	18	Unknown	.	.	.	.	.	.	.	.
T42df	chr19	**1**	**12.5**	4739972	4739972	C	A	exonic	**Sptbn2**	ENSMUSP00000153171	.	nonsynonymous SNV	Sptbn2:NM_021287:exon19:c.C3987A:p.N1329K	**N1329K**	ENSMUSP00000153171,1329,N,K	19,4739972,C,A	19qA	.	0.25	.	42	chr19	4739972	.	C	A	.	PASS	ECNT=1;HCNT=1;MAX_ED=.;MIN_ED=.;NLOD=10.53;TLOD=6.45	GT:AD:AF:ALT_F1R2:ALT_F2R1:FOXOG:QSS:REF_F1R2:REF_F2R1	0/1:74,4:0.053:2:2:0.500:2072,113:41:33	0/0:42,0:0.00:0:0:.:1181,0:26:16	5	Deleterious	-4.99	Deleterious	205	30	0.002	Damaging	2.84	230
T1df	chr19	**1**	**12.5**	4731928	4731928	-	A	exonic	**Sptbn2**	ENSMUSP00000153171	.	frameshift insertion	Sptbn2:NM_021287:exon11:c.1194_1195insA:p.A398fs	**A398fs**	ENSMUSP00000153171,398,A,	19,4731928,C,A	19qA	.	0.25	.	36	chr19	4731928	.	C	A	.	t_lod_fstar	ECNT=1;HCNT=1;MAX_ED=.;MIN_ED=.;NLOD=9.93;TLOD=5.71	GT:AD:AF:ALT_F1R2:ALT_F2R1:FOXOG:QSS:REF_F1R2:REF_F2R1	0/1:38,2:0.059:2:0:.:1077,58:26:12	0/0:42,0:0.00:0:0:.:1131,0:20:22	0.4	Tolerated	0	Neutral	205	30	1	Tolerated	2.85	221
T42df	chr5	**1**	**12.5**	116444770	116444772	CTG	-	exonic	**Srrm4**	ENSMUSP00000124590	.	nonframeshift deletion	Srrm4:NM_026886:exon13:c.1623_1625del:p.541_542del	**541_542del**	NA	5,116444770,ACTG,A	5qF	.	0.25	.	33	chr5	116444769	.	ACTG	A	.	alt_allele_in_normal;str_contraction	ECNT=1;HCNT=1;MAX_ED=.;MIN_ED=.;NLOD=4.80;RPA=2,1;RU=GGAGCC;STR;TLOD=9.06	GT:AD:AF:ALT_F1R2:ALT_F2R1:FOXOG:QSS:REF_F1R2:REF_F2R1	0/1:35,3:0.086:1:2:.:981,84:16:19	0/0:28,2:0.071:1:1:.:803,56:15:13	0.2	Unknown	.	.	.	.	.	.	.	.
T1df	chr5	**1**	**12.5**	113946612	113946612	-	CCTGATCCTCTAAGTGGACCAGGCCACCCGTATCATCATGGT	exonic	**Ssh1**	ENSMUSP00000002198	.	stopgain	Ssh1:NM_198109:exon14:c.1565_1566insACCATGATGATACGGGTGGCCTGGTCCACTTAGAGGATCAGG:p.P522delinsPPX	**P522delinsPPX**	ENSMUSP00000002198,522,P,PPX	NA	5qF	.	0.25	.	33	chr5	113946612	.	G	C	.	str_contraction;t_lod_fstar	ECNT=1;HCNT=2;MAX_ED=.;MIN_ED=.;NLOD=11.36;RPA=6,5;RU=CTG;STR;TLOD=5.65	GT:AD:AF:ALT_F1R2:ALT_F2R1:FOXOG:QSS:REF_F1R2:REF_F2R1	0/1:37,2:0.056:0:2:.:1034,56:19:18	0/0:41,0:0.00:0:0:.:1144,0:21:20	3	Deletarious	.	.	.	.	.	.	.	.
T22df	chr11	**1**	**12.5**	116766632	116766632	A	C	exonic	**St6galnac1**	ENSMUSP00000123316	.	nonsynonymous SNV	St6galnac1:NM_011371:exon6:c.T1105G:p.Y369D	**Y369D**	ENSMUSP00000123316,369,Y,D	11,116766632,A,G	11qE2	.	0.25	.	56	chr11	116766632	.	A	G	.	clustered_events;multi_event_alt_allele_in_normal	ECNT=8;HCNT=1;MAX_ED=24;MIN_ED=5;NLOD=11.76;TLOD=7.64	GT:AD:AF:ALT_F1R2:ALT_F2R1:FOXOG:PGT:PID:QSS:REF_F1R2:REF_F2R1	0/1:102,3:0.030:0:3:1.00:0|1:116766632_A_C:2332,60:55:47	0/0:58,1:0.019:0:1:1.00:0|1:116766632_A_C:1331,28:29:29	6	Deleterious	-4.87	Deleterious	83	30	0	Damaging	2.82	88
T22df	chr11	**1**	**12.5**	116766637	116766637	A	T	exonic	**St6galnac1**	ENSMUSP00000123316	.	nonsynonymous SNV	St6galnac1:NM_011371:exon6:c.T1100A:p.V367D	**V367D**	ENSMUSP00000123316,367,V,D	11,116766637,A,C	11qE2	.	0.25	.	59	chr11	116766637	.	A	C	.	clustered_events;multi_event_alt_allele_in_normal	ECNT=8;HCNT=1;MAX_ED=24;MIN_ED=5;NLOD=12.66;TLOD=7.65	GT:AD:AF:ALT_F1R2:ALT_F2R1:FOXOG:PGT:PID:QSS:REF_F1R2:REF_F2R1	0/1:102,3:0.030:0:3:.:0|1:116766632_A_C:2866,85:56:46	0/0:59,1:0.018:0:1:.:0|1:116766632_A_C:1652,30:27:32	6	Deleterious	-6.44	Deleterious	83	30	0.001	Damaging	2.82	89
T22df	chr11	**1**	**12.5**	116766638	116766638	C	G	exonic	**St6galnac1**	ENSMUSP00000123316	.	nonsynonymous SNV	St6galnac1:NM_011371:exon6:c.G1099C:p.V367L	**V367L**	ENSMUSP00000123316,367,V,L	11,116766638,C,A	11qE2	.	0.25	.	59	chr11	116766638	.	C	A	.	str_contraction	ECNT=1;HCNT=1;MAX_ED=.;MIN_ED=.;NLOD=6.59;RPA=8,7;RU=CAG;STR;TLOD=7.63	GT:AD:AF:ALT_F1R2:ALT_F2R1:FOXOG:QSS:REF_F1R2:REF_F2R1	0/1:41,3:0.070:1:2:.:1149,84:16:25	0/0:22,0:0.00:0:0:.:608,0:9:13	0.7	Deleterious	-3.39	Deleterious	83	30	0.003	Damaging	2.82	89

T22df	chr11	**1**	**12.5**	116766642	116766643	CT	-	splicing	**St6galnac1**	ENSMUSP00000123316	.	.	.	**NA**	11,116766642,CCT,C	NA	11qE2	.	0.25	.	60	chr11	116766641	.	CCT	C	.	PASS	ECNT=1;HCNT=1;MAX_ED=.;MIN_ED=.;NLOD=11.13;TLOD=6.58	GT:AD:AF:ALT_F1R2:ALT_F2R1:FOXOG:QSS:REF_F1R2:REF_F2R1	0/1:15,3:0.188:2:1:0.667:431,85:4:11	0/0:43,0:0.00:0:0:.:1222,0:24:19	14	Unkown	.	.	.	.	.	.	.	.
T12df	chr2	**1**	**12.5**	14146281	14146283	CTG	-	exonic	**Stam**	ENSMUSP00000099178	.	nonframeshift deletion	Stam:NM_001356337:exon13:c.1298_1300del:p.433_434del,Stam:NM_011484:exon14:c.1448_1450del:p.483_484del	**433_434del**	NA	2,14146281,CCTG,C	2qA2	.	0.25	.	41	chr2	14146280	.	CCTG	C	.	str_contraction	ECNT=1;HCNT=1;MAX_ED=.;MIN_ED=.;NLOD=10.21;RPA=6,5;RU=GGA;STR;TLOD=11.54	GT:AD:AF:ALT_F1R2:ALT_F2R1:FOXOG:QSS:REF_F1R2:REF_F2R1	0/1:61,4:0.063:2:2:.:1571,99:28:33	0/0:36,0:0.00:0:0:.:904,0:21:15	1.4	Unknown	.	.	.	.	.	.	.	.
T12df	chr2	**1**	**12.5**	14146281	14146283	CTG	-	exonic	**Stam**	ENSMUSP00000099178	.	nonframeshift deletion	Stam:NM_001356337:exon13:c.1298_1300del:p.433_434del,Stam:NM_011484:exon14:c.1448_1450del:p.483_484del	**483_484del**	NA	2,14146281,CCTG,C	2qA2	.	0.25	.	41	chr2	14146280	.	CCTG	C	.	str_contraction	ECNT=1;HCNT=1;MAX_ED=.;MIN_ED=.;NLOD=10.21;RPA=6,5;RU=GGA;STR;TLOD=11.54	GT:AD:AF:ALT_F1R2:ALT_F2R1:FOXOG:QSS:REF_F1R2:REF_F2R1	0/1:61,4:0.063:2:2:.:1571,99:28:33	0/0:36,0:0.00:0:0:.:904,0:21:15	1.4	Unknown	.	.	.	.	.	.	.	.
T3df	chr11	**1**	**12.5**	98380500	98380500	A	C	exonic	**Stard3**	ENSP00000337446	.	nonsynonymous SNV	Stard3:NM_021547:exon15:c.A1279C:p.T427P	**T427P**	ENSP00000337446,427,T,P	11,98380500,A,C	11qD	.	0.25	.	29	chr11	98380500	.	A	C	.	clustered_events;homologous_mapping_event;t_lod_fstar	ECNT=4;HCNT=2;MAX_ED=11;MIN_ED=5;NLOD=8.13;TLOD=5.22	GT:AD:AF:ALT_F1R2:ALT_F2R1:FOXOG:PGT:PID:QSS:REF_F1R2:REF_F2R1	0/1:56,2:0.034:2:0:0.00:0|1:98380500_A_C:1580,51:27:29	0/0:29,0:0.00:0:0:.:0|1:98380500_A_C:824,0:13:16	14	Damaging	-2.13	Neutral	128	30	0.02	Damaging	2.85	71
T3df	chr11	**1**	**12.5**	98380510	98380510	A	C	exonic	**Stard3**	ENSP00000337446	.	nonsynonymous SNV	Stard3:NM_021547:exon15:c.A1289C:p.E430A	**E430A**	ENSP00000337446,430,E,A	11,98380510,A,C	11qD	.	0.25	.	28	chr11	98380510	.	A	C	.	clustered_events;homologous_mapping_event;t_lod_fstar	ECNT=4;HCNT=2;MAX_ED=11;MIN_ED=5;NLOD=8.43;TLOD=5.22	GT:AD:AF:ALT_F1R2:ALT_F2R1:FOXOG:PGT:PID:QSS:REF_F1R2:REF_F2R1	0/1:57,2:0.034:2:0:0.00:0|1:98380500_A_C:1566,44:26:31	0/0:28,0:0.00:0:0:.:0|1:98380500_A_C:754,0:12:16	14	Deleterious	-3.23	Deleterious	128	30	0.011	Damaging	2.85	71
T3df	chr11	**1**	**12.5**	98380511	98380511	A	C	exonic	**Stard3**	ENSP00000337446	.	nonsynonymous SNV	Stard3:NM_021547:exon15:c.A1290C:p.E430D	**E430D**	ENSP00000337446,430,E,D	11,98380511,A,C	11qD	.	0.25	.	29	chr11	98380511	.	A	C	.	clustered_events;homologous_mapping_event;t_lod_fstar	ECNT=4;HCNT=2;MAX_ED=11;MIN_ED=5;NLOD=8.13;TLOD=5.22	GT:AD:AF:ALT_F1R2:ALT_F2R1:FOXOG:PGT:PID:QSS:REF_F1R2:REF_F2R1	0/1:56,2:0.034:2:0:0.00:0|1:98380500_A_C:1539,47:25:31	0/0:29,0:0.00:0:0:.:0|1:98380500_A_C:806,0:13:16	14	Tolerated	0.05	Neutral	128	30	0.559	Tolerated	2.85	71
T3df	chr11	**1**	**12.5**	98380505	98380505	G	T	exonic	**Stard3**	ENSP00000337446	.	nonsynonymous SNV	Stard3:NM_021547:exon15:c.G1284T:p.M428I	**M428I**	ENSP00000337446,428,M,I	11,98380505,G,T	11qD	.	0.25	.	28	chr11	98380505	.	G	T	.	clustered_events;homologous_mapping_event;t_lod_fstar	ECNT=4;HCNT=2;MAX_ED=11;MIN_ED=5;NLOD=8.43;TLOD=5.21	GT:AD:AF:ALT_F1R2:ALT_F2R1:FOXOG:PGT:PID:QSS:REF_F1R2:REF_F2R1	0/1:56,2:0.034:2:0:1.00:0|1:98380500_A_C:1550,29:26:30	0/0:28,0:0.00:0:0:.:0|1:98380500_A_C:792,0:12:16	14	Tolerated	-1.91	Neutral	128	30	0.094	Tolerated	2.85	70
T4df	chr7	**1**	**12.5**	139125272	139125272	A	-	exonic	**Stk32c**	ENSMUSP00000036347	.	frameshift deletion	Stk32c:NM_021302:exon3:c.329delT:p.I110fs	**I110fs**	ENSMUSP00000036347,110,I,.	7,139125272,GA,T	7qF4	.	0.25	.	36	chr7	139125271	.	GA	T	.	t_lod_fstar	ECNT=1;HCNT=1;MAX_ED=.;MIN_ED=.;NLOD=10.84;TLOD=4.10	GT:AD:AF:ALT_F1R2:ALT_F2R1:FOXOG:QSS:REF_F1R2:REF_F2R1	0/1:39,2:0.050:2:0:1.00:1123,37:15:24	0/0:38,0:0.00:0:0:.:1065,0:20:18	0.7	Unknown	NA	NA	166	30	NA	NA	NA	NA
T42df	chrX	**1**	**12.5**	9993073	9993073	G	T	exonic	**Sytl5**	ENSMUSP00000086949	.	nonsynonymous SNV	Sytl5:NM_001290728:exon15:c.G2029T:p.G677C,Sytl5:NM_177704:exon16:c.G2095T:p.G699C	**G677C**	ENSMUSP00000086949,677,G,C	X,9993073,G,C	XqA1.1	.	0.25	.	38	chrX	9993073	.	G	C	.	alt_allele_in_normal;t_lod_fstar	ECNT=1;HCNT=1;MAX_ED=.;MIN_ED=.;NLOD=7.67;TLOD=4.30	GT:AD:AF:ALT_F1R2:ALT_F2R1:FOXOG:QSS:REF_F1R2:REF_F2R1	0/1:43,10:0.189:3:7:.:1060,230:16:27	0/0:45,20:0.333:11:9:.:1114,529:21:24	0.8	Deleterious	-7.46	Deleterious	108	30	0.002	Damaging	2.8	91
T42df	chrX	**1**	**12.5**	9993073	9993073	G	T	exonic	**Sytl5**	ENSMUSP00000086949	.	nonsynonymous SNV	Sytl5:NM_001290728:exon15:c.G2029T:p.G677C,Sytl5:NM_177704:exon16:c.G2095T:p.G699C	**G699C**	ENSMUSP00000086949,699,G,C	X,9993073,G,C	XqA1.1	.	0.25	.	38	chrX	9993073	.	G	C	.	alt_allele_in_normal;t_lod_fstar	ECNT=1;HCNT=1;MAX_ED=.;MIN_ED=.;NLOD=7.67;TLOD=4.30	GT:AD:AF:ALT_F1R2:ALT_F2R1:FOXOG:QSS:REF_F1R2:REF_F2R1	0/1:43,10:0.189:3:7:.:1060,230:16:27	0/0:45,20:0.333:11:9:.:1114,529:21:24	0.8	Deleterious	-7.46	Deleterious	108	30	0.002	Damaging	2.8	91

T42df	chr13	**1**	**12.5**	100655331	100655331	A	G	exonic	**Taf9**	ENSMUSP00000020102	.	nonsynonymous SNV	Taf9:NM_001015889:exon3:c.A281G:p.D94G,Taf9:NM_027139:exon3:c.A281G:p.D94G	**D94G**	ENSMUSP00000020102,94,D,G	13,100655331,A,TGGTCGAGACTGCCACCATA	13qD1	.	0.25	.	38	chr13	100655331	.	A	TGGTCGAGACTGCCACCATA	.	PASS	ECNT=1;HCNT=1;MAX_ED=.;MIN_ED=.;NLOD=6.02;TLOD=8.69	GT:AD:AF:ALT_F1R2:ALT_F2R1:FOXOG:QSS:REF_F1R2:REF_F2R1	0/1:42,3:0.067:1:2:.:1209,81:22:20	0/0:24,0:0.00:0:0:.:685,0:14:10	2	Unknown	NA	NA	151	30	NA	NA	NA	NA
T12df	chr3	**1**	**12.5**	93445695	93445695	T	A	exonic	**Tchh**	ENSMUSP00000107908	.	nonsynonymous SNV	Tchh:NM_001163098:exon2:c.T2441A:p.L814Q	**L814Q**	ENSMUSP00000107908,814,L,Q	3,93445695,T,G	3qF2.1	.	0.25	.	29	chr3	93445695	.	T	G	.	clustered_events;homologous_mapping_event	DB;ECNT=8;HCNT=42;MAX_ED=199;MIN_ED=18;NLOD=8.98;TLOD=36.83	GT:AD:AF:ALT_F1R2:ALT_F2R1:FOXOG:QSS:REF_F1R2:REF_F2R1	0/1:20,14:0.424:7:7:0.500:567,387:10:10	0/0:34,0:0.00:0:0:.:939,0:13:21	2	Tolerated	0.88	Neutral	50	30	0.09	Tolerated	4.32	3
T42df	chr3	**1**	**12.5**	93447318	93447398	AGCAGCGGCGCCGCCAGGAGCGCGAGCAAGAGCAGCAGCGGTGCCTGGAGCGTGAGGAGGAACAATTTCGCTTTGAGGAGC	-	exonic	**Tchh**	ENSMUSP00000107908	.	nonframeshift deletion	Tchh:NM_001163098:exon2:c.4064_4144del:p.1355_1382del	**1355_1382del**	NA	3,93447318,GAGCAGCGGCGCCGCCAGGAGCGCGAGCAAGAGCAGCAGCGGTGCCTGGAGCGTGAGGAGGAACAATTTCGCTTTGAGGAGC,G	3qF2.1	.	0.25	.	46	chr3	93447317	.	GAGCAGCGGCGCCGCCAGGAGCGCGAGCAAGAGCAGCAGCGGTGCCTGGAGCGTGAGGAGGAACAATTTCGCTTTGAGGAGC	G	.	clustered_events;homologous_mapping_event	DB;ECNT=8;HCNT=42;MAX_ED=199;MIN_ED=18;NLOD=8.38;TLOD=38.39	GT:AD:AF:ALT_F1R2:ALT_F2R1:FOXOG:QSS:REF_F1R2:REF_F2R1	0/1:21,14:0.424:5:9:0.643:582,392:11:10	0/0:32,0:0.00:0:0:.:900,0:15:17	2	Unknown	.	.	.	.	.	.	.	.
T42df	chr5	**1**	**12.5**	144200884	144200884	C	T	exonic	**Tecpr1**	ENSMUSP00000120257	.	stopgain	Tecpr1:NM_001356992:exon18:c.G1064A:p.W355X,Tecpr1:NM_001356993:exon18:c.G1049A:p.W350X,Tecpr1:NM_027410:exon18:c.G2636A:p.W879X	**W350X**	ENSMUSP00000120257,350,W,X	NA	5qG2	.	0.25	.	46	chr5	144200884	.	C	A	.	PASS	ECNT=1;HCNT=1;MAX_ED=.;MIN_ED=.;NLOD=8.29;TLOD=7.40	GT:AD:AF:ALT_F1R2:ALT_F2R1:FOXOG:QSS:REF_F1R2:REF_F2R1	0/1:50,4:0.077:0:4:0.00:1383,116:25:25	0/0:34,0:0.00:0:0:.:899,0:17:17	0.8	Deleterious	.	.	.	.	.	.	.	.
T42df	chr5	**1**	**12.5**	144200884	144200884	C	T	exonic	**Tecpr1**	ENSMUSP00000120257	.	stopgain	Tecpr1:NM_001356992:exon18:c.G1064A:p.W355X,Tecpr1:NM_001356993:exon18:c.G1049A:p.W350X,Tecpr1:NM_027410:exon18:c.G2636A:p.W879X	**W355X**	ENSMUSP00000120257,355,W,X	NA	5qG2	.	0.25	.	46	chr5	144200884	.	C	T	.	t_lod_fstar	ECNT=1;HCNT=1;MAX_ED=.;MIN_ED=.;NLOD=12.33;TLOD=5.25	GT:AD:AF:ALT_F1R2:ALT_F2R1:FOXOG:QSS:REF_F1R2:REF_F2R1	0/1:46,3:0.067:3:0:0.00:1317,86:24:22	0/0:46,0:0.00:0:0:.:1276,0:20:26	1.3	Deleterious	.	.	.	.	.	.	.	.
T42df	chr5	**1**	**12.5**	144200884	144200884	C	T	exonic	**Tecpr1**	ENSMUSP00000120257	.	stopgain	Tecpr1:NM_001356992:exon18:c.G1064A:p.W355X,Tecpr1:NM_001356993:exon18:c.G1049A:p.W350X,Tecpr1:NM_027410:exon18:c.G2636A:p.W879X	**W879X**	ENSMUSP00000120257,379,W,X	NA	5qG2	.	0.25	.	46	chr5	144200884	.	C	CACGGTTTCTTCCGGCCTTGTCCATCCGGCCATGGCTGTGTCGACGAGGCCAGCGGAACCACCAAAGCTGTGGCTACCTCCTGCTAGAT	.	PASS	ECNT=1;HCNT=1;MAX_ED=.;MIN_ED=.;NLOD=10.01;TLOD=7.48	GT:AD:AF:ALT_F1R2:ALT_F2R1:FOXOG:QSS:REF_F1R2:REF_F2R1	0/1:36,1:0.077:1:0:.:1038,28:13:23	0/0:36,0:0.00:0:0:.:1034,0:15:21	0.5	Deleterious	.	.	.	.	.	.	.	.
T1df	chr16	**1**	**12.5**	32624389	32624389	T	A	exonic	**Tfrc**	ENSMUSP00000108692	.	nonsynonymous SNV	Tfrc:NM_001357298:exon14:c.T1498A:p.S500T,Tfrc:NM_011638:exon14:c.T1498A:p.S500T	**S500T**	ENSMUSP00000108692,500,S,T	16,32624389,T,G	16qB3	.	0.25	.	58	chr16	32624389	.	T	G	.	t_lod_fstar	ECNT=1;HCNT=1;MAX_ED=.;MIN_ED=.;NLOD=9.33;TLOD=5.91	GT:AD:AF:ALT_F1R2:ALT_F2R1:FOXOG:QSS:REF_F1R2:REF_F2R1	0/1:37,3:0.081:3:0:1.00:1072,90:22:15	0/0:34,0:0.00:0:0:.:965,0:21:13	0.6	Tolerated	-1.43	Neutral	147	30	0.149	Tolerated	2.82	119
T2df	chr14	**1**	**12.5**	118119740	118119740	-	GGTCGAGACTGCCACCATA	splicing	**Tgds**	ENSMUSP00000036081	.	NM_029578:exon6:c.457-2->TATGGTGGCAGTCTCGACC	.	**NA**	14,118119740,T,G	NA	14qE4	.	0.25	.	24	chr14	118119740	.	T	G	.	t_lod_fstar	ECNT=1;HCNT=1;MAX_ED=.;MIN_ED=.;NLOD=9.33;TLOD=5.91	GT:AD:AF:ALT_F1R2:ALT_F2R1:FOXOG:QSS:REF_F1R2:REF_F2R1	0/1:37,3:0.081:3:0:1.00:1072,90:22:15	0/0:34,0:0.00:0:0:.:965,0:21:13	0.6	Unkown	.	.	.	.	.	.	.	.
T12df	chr17	**1**	**12.5**	14685896	14685896	G	T	exonic	**Thbs2**	ENSMUSP00000122747	.	nonsynonymous SNV	Thbs2:NM_011581:exon5:c.C741A:p.S247R	**S247R**	ENSMUSP00000122747,247,S,R	17,14685896,G,C	17qA2	.	0.25	.	43	chr17	14685896	.	G	C	.	clustered_events;homologous_mapping_event	DB;ECNT=4;HCNT=2;MAX_ED=66;MIN_ED=64;NLOD=11.14;TLOD=107.88	GT:AD:AF:ALT_F1R2:ALT_F2R1:FOXOG:PGT:PID:QSS:REF_F1R2:REF_F2R1	0/1:38,29:0.446:19:10:0.655:0|1:129400088_G_A:1053,735:13:25	0/0:42,0:0.00:0:0:.:0|1:129400088_G_A:1180,0:19:23	2.2	Damaging	-1.91	Neutral	245	30	0.003	Damaging	3.52	106
T4df	chr10	**1**	**12.5**	128250371	128250371	T	G	exonic	**Timeless**	ENSMUSP00000073134	.	nonsynonymous SNV	Timeless:NM_001136082:exon24:c.T2937G:p.D979E,Timeless:NM_001164080:exon24:c.T2940G:p.D980E,Timeless:NM_001164081:exon24:c.T2940G:p.D980E,Timeless:NM_011589:exon24:c.T2940G:p.D980E	**D979E**	ENSMUSP00000073134,979,D,E	10,128250371,T,AGTTAGTG	10qD3	.	0.25	.	34	chr10	128250371	.	T	AGTTAGTG	.	t_lod_fstar	ECNT=1;HCNT=1;MAX_ED=.;MIN_ED=.;NLOD=12.37;TLOD=5.90	GT:AD:AF:ALT_F1R2:ALT_F2R1:FOXOG:QSS:REF_F1R2:REF_F2R1	0/1:29,2:0.074:2:0:.:788,58:18:11	0/0:45,0:0.00:0:0:.:1257,0:20:25	0.4	Unknown	NA	NA	94	30	NA	NA	NA	NA

T4df	chr10	**1**	**12.5**	128250371	128250371	T	G	exonic	**Timeless**	ENSMUSP00000073134	.	nonsynonymous SNV	Timeless:NM_001136082:exon24:c.T2937G:p.D979E,Timeless:NM_001164080:exon24:c.T2940G:p.D980E,Timeless:NM_001164081:exon24:c.T2940G:p.D980E,Timeless:NM_011589:exon24:c.T2940G:p.D980E	**D980E**	ENSMUSP00000073134,980,D,E	10,128250371,T,AGTTAGTG	10qD3	.	0.25	.	34	chr10	128250371	.	T	AGTTAGTG	.	t_lod_fstar	ECNT=1;HCNT=1;MAX_ED=.;MIN_ED=.;NLOD=12.37;TLOD=5.90	GT:AD:AF:ALT_F1R2:ALT_F2R1:FOXOG:QSS:REF_F1R2:REF_F2R1	0/1:29,2:0.074:2:0:.:788,58:18:11	0/0:45,0:0.00:0:0:.:1257,0:20:25	0.4	Unknown	NA	NA	94	30	NA	NA	NA	NA
T42df	chr19	**1**	**12.5**	14466593	14466593	-	GTTAGTG	exonic	**Tle4**	ENSMUSP00000143938	.	stopgain	Tle4:NM_001302950:exon10:c.819_820insCACTAAC:p.S274_S275delinsHX,Tle4:NM_001302947:exon11:c.894_895insCACTAAC:p.S299_S300delinsHX,Tle4:NM_001302951:exon11:c.888_889insCACTAAC:p.S297_S298delinsHX,Tle4:NM_011600:exon11:c.891_892insCACTAAC:p.S298_S299delinsHX	**S274_S275delinsHX**	ENSMUSP00000143938,274,S S,HX	NA	19qA	.	0.25	.	45	chr19	14466593	.	A	AGTTAGTG	.	t_lod_fstar	ECNT=1;HCNT=1;MAX_ED=.;MIN_ED=.;NLOD=12.37;TLOD=5.90	GT:AD:AF:ALT_F1R2:ALT_F2R1:FOXOG:QSS:REF_F1R2:REF_F2R1	0/1:29,2:0.074:2:0:.:788,58:18:11	0/0:45,0:0.00:0:0:.:1257,0:20:25	0.4	Deletarious	.	.	.	.	.	.	.	.
T42df	chr19	**1**	**12.5**	14466593	14466593	-	GTTAGTG	exonic	**Tle4**	ENSMUSP00000143938	.	stopgain	Tle4:NM_001302950:exon10:c.819_820insCACTAAC:p.S274_S275delinsHX,Tle4:NM_001302947:exon11:c.894_895insCACTAAC:p.S299_S300delinsHX,Tle4:NM_001302951:exon11:c.888_889insCACTAAC:p.S297_S298delinsHX,Tle4:NM_011600:exon11:c.891_892insCACTAAC:p.S298_S299delinsHX	**S297_S298delinsHX**	ENSMUSP00000143938,297,S S,HX	NA	19qA	.	0.25	.	45	chr19	14466593	.	A	C	.	PASS	ECNT=1;HCNT=1;MAX_ED=.;MIN_ED=.;NLOD=12.93;TLOD=9.58	GT:AD:AF:ALT_F1R2:ALT_F2R1:FOXOG:QSS:REF_F1R2:REF_F2R1	0/1:37,5:0.122:3:2:0.600:1041,138:16:21	0/0:50,0:0.00:0:0:.:1394,0:31:19	0.5	Deletarious	.	.	.	.	.	.	.	.
T42df	chr19	**1**	**12.5**	14466593	14466593	-	GTTAGTG	exonic	**Tle4**	ENSMUSP00000143938	.	stopgain	Tle4:NM_001302950:exon10:c.819_820insCACTAAC:p.S274_S275delinsHX,Tle4:NM_001302947:exon11:c.894_895insCACTAAC:p.S299_S300delinsHX,Tle4:NM_001302951:exon11:c.888_889insCACTAAC:p.S297_S298delinsHX,Tle4:NM_011600:exon11:c.891_892insCACTAAC:p.S298_S299delinsHX	**S298_S299delinsHX**	ENSMUSP00000143938,298,S S,HX	NA	19qA	.	0.25	.	45	chr19	14466593	.	A	C	.	PASS	ECNT=1;HCNT=1;MAX_ED=.;MIN_ED=.;NLOD=12.93;TLOD=6.50	GT:AD:AF:ALT_F1R2:ALT_F2R1:FOXOG:QSS:REF_F1R2:REF_F2R1	0/1:55,4:0.073:2:2:0.500:1570,108:27:28	0/0:50,0:0.00:0:0:.:1394,0:31:19	0.5	Deletarious	.	.	.	.	.	.	.	.
T42df	chr19	**1**	**12.5**	14466593	14466593	-	GTTAGTG	exonic	**Tle4**	ENSMUSP00000143938	.	stopgain	Tle4:NM_001302950:exon10:c.819_820insCACTAAC:p.S274_S275delinsHX,Tle4:NM_001302947:exon11:c.894_895insCACTAAC:p.S299_S300delinsHX,Tle4:NM_001302951:exon11:c.888_889insCACTAAC:p.S297_S298delinsHX,Tle4:NM_011600:exon11:c.891_892insCACTAAC:p.S298_S299delinsHX	**S299_S300delinsHX**	ENSMUSP00000143938,299,S S,HX	NA	19qA	.	0.25	.	45	chr19	14466593	.	A	AGTTAGTG	.	t_lod_fstar	ECNT=1;HCNT=1;MAX_ED=.;MIN_ED=.;NLOD=12.37;TLOD=5.90	GT:AD:AF:ALT_F1R2:ALT_F2R1:FOXOG:QSS:REF_F1R2:REF_F2R1	0/1:29,2:0.074:2:0:.:788,58:18:11	0/0:45,0:0.00:0:0:.:1257,0:20:25	0.4	Deletarious	.	.	.	.	.	.	.	.
T22df	chr1	**1**	**12.5**	74289442	74289442	G	T	exonic	**Tmbim1**	ENSMUSP00000022105	.	nonsynonymous SNV	Tmbim1:NM_027154:exon12:c.C860A:p.A287D	**A287D**	ENSMUSP00000022105,287,A,D	1,74289442,G,A	1qC3	.	0.25	.	29	chr1	74289442	.	G	A	.	clustered_events;multi_event_alt_allele_in_normal;t_lod_fstar	ECNT=5;HCNT=1;MAX_ED=23;MIN_ED=4;NLOD=3.93;TLOD=5.55	GT:AD:AF:ALT_F1R2:ALT_F2R1:FOXOG:PGT:PID:QSS:REF_F1R2:REF_F2R1	0/1:37,2:0.050:0:2:.:0|1:74289438_TA_T:1005,29:21:16	0/0:29,1:0.036:0:1:.:0|1:74289438_TA_T:812,10:16:13	0.4	Deleterious	-3.24	Deleterious	150	30	0.001	Damaging	2.85	135
T22df	chr1	**1**	**12.5**	74289461	74289461	-	ATTAAAAAA	exonic	**Tmbim1**	ENSMUSP00000022105	.	nonframeshift insertion	Tmbim1:NM_027154:exon12:c.840_841insTTTTTAAT:p.E281delinsFFNE	**E281delinsFFNE**	ENSMUSP00000022105,281,E,FFNE	1,74289461,C,C	1qC3	.	0.25	.	26	chr1	74289461	.	C	C	.	PASS	DB;ECNT=1;HCNT=1;MAX_ED=.;MIN_ED=.;NLOD=9.83;TLOD=100.61	GT:AD:AF:ALT_F1R2:ALT_F2R1:FOXOG:QSS:REF_F1R2:REF_F2R1	0/1:28,37:0.574:21:16:0.568:801,1040:17:11	0/0:36,0:0.00:0:0:.:966,0:18:18	1.3	Tolerated	0	Neutral	150	30	1	Tolerated	2.85	135
T22df	chr1	**1**	**12.5**	74289439	74289439	A	-	exonic	**Tmbim1**	ENSMUSP00000022105	.	frameshift deletion	Tmbim1:NM_027154:exon12:c.863delT:p.L288fs	**L288fs**	ENSMUSP00000022105,288,L,.	1,74289439,TA,C	1qC3	.	0.25	.	31	chr1	74289438	.	TA	C	.	alt_allele_in_normal;clustered_events;t_lod_fstar	ECNT=5;HCNT=1;MAX_ED=23;MIN_ED=4;NLOD=4.23;TLOD=5.55	GT:AD:AF:ALT_F1R2:ALT_F2R1:FOXOG:PGT:PID:QSS:REF_F1R2:REF_F2R1	0/1:38,2:0.050:0:2:.:0|1:74289438_TA_T:1029,40:21:17	0/0:30,1:0.034:0:1:.:0|1:74289438_TA_T:830,25:18:12	0.4	Unknown	NA	NA	150	30	NA	NA	NA	NA

T22df	chr1	**1**	**12.5**	74289456	74289459	GTCC	-	exonic	**Tmbim1**	ENSMUSP00000022105	.	frameshift deletion	Tmbim1:NM_027154:exon12:c.843_846del:p.E281fs	**E281fs**	ENSMUSP00000022105,281,E,.	1,74289456,AGTCC,CATTAAAAAA	1qC3	.	0.25	.	30	chr1	74289455	.	AGTCC	CATTAAAAAA	.	alt_allele_in_normal;clustered_events;t_lod_fstar	ECNT=5;HCNT=1;MAX_ED=23;MIN_ED=4;NLOD=3.33;TLOD=5.60	GT:AD:AF:ALT_F1R2:ALT_F2R1:FOXOG:PGT:PID:QSS:REF_F1R2:REF_F2R1	0/1:36,2:0.053:0:2:.:0|1:74289438_TA_T:995,46:20:16	0/0:25,1:0.038:0:1:.:0|1:74289438_TA_T:672,22:14:11	0.4	Unknown	NA	NA	150	30	NA	NA	NA	NA
T22df	chr1	**1**	**12.5**	74289447	74289452	TGTGAT	-	exonic	**Tmbim1**	ENSMUSP00000022105	.	nonframeshift deletion	Tmbim1:NM_027154:exon12:c.850_855del:p.284_285del	**284_285del**	NA	1,74289447,CTGTGAT,C	1qC3	.	0.25	.	31	chr1	74289446	.	CTGTGAT	C	.	PASS	DB;ECNT=1;HCNT=1;MAX_ED=.;MIN_ED=.;NLOD=9.83;TLOD=100.61	GT:AD:AF:ALT_F1R2:ALT_F2R1:FOXOG:QSS:REF_F1R2:REF_F2R1	0/1:28,37:0.574:21:16:0.568:801,1040:17:11	0/0:36,0:0.00:0:0:.:966,0:18:18	1.3	Unknown	.	.	.	.	.	.	.	.
T4df	chr1	**1**	**12.5**	180948832	180948832	C	T	exonic	**Tmem63a**	ENSMUSP00000038142	.	nonsynonymous SNV	Tmem63a:NM_144794:exon3:c.C206T:p.S69F	**S69F**	ENSMUSP00000038142,69,S,F	1,180948832,C,A	1qH4	.	0.25	.	35	chr1	180948832	.	C	A	.	str_contraction;t_lod_fstar	ECNT=1;HCNT=1;MAX_ED=.;MIN_ED=.;NLOD=9.00;RPA=8,7;RU=CTG;STR;TLOD=4.33	GT:AD:AF:ALT_F1R2:ALT_F2R1:FOXOG:QSS:REF_F1R2:REF_F2R1	0/1:46,2:0.042:1:1:.:1322,43:24:22	0/0:35,0:0.00:0:0:.:949,0:13:22	0.6	Deleterious	-4.8	Deleterious	147	30	0	Damaging	2.83	125
T4df	chr1	**1**	**12.5**	180948834	180948834	A	T	exonic	**Tmem63a**	ENSMUSP00000038142	.	nonsynonymous SNV	Tmem63a:NM_144794:exon3:c.A208T:p.I70F	**I70F**	ENSMUSP00000038142,70,I,F	1,180948834,A,T	1qH4	.	0.25	.	36	chr1	180948834	.	A	T	.	clustered_events;t_lod_fstar	ECNT=2;HCNT=1;MAX_ED=3;MIN_ED=3;NLOD=11.14;TLOD=5.04	GT:AD:AF:ALT_F1R2:ALT_F2R1:FOXOG:PGT:PID:QSS:REF_F1R2:REF_F2R1	0/1:72,2:0.028:0:2:1.00:0|1:85038391_A_T:2071,56:39:33	0/0:41,0:0.00:0:0:.:0|1:85038391_A_T:1127,0:16:25	2	Tolerated	-0.21	Neutral	147	30	0.588	Tolerated	2.83	125
T32df	chr4	**1**	**12.5**	152116600	152116602	CTG	-	exonic	**Tnfrsf25**	ENSMUSP00000109750	.	nonframeshift deletion	Tnfrsf25:NM_033042:exon1:c.91_93del:p.31_31del,Tnfrsf25:NM_001291010:exon2:c.52_54del:p.18_18del	**31_31del**	NA	4,152116600,ACTG,C	4qE2	.	0.25	.	35	chr4	152116599	.	ACTG	C	.	clustered_events;t_lod_fstar	ECNT=2;HCNT=1;MAX_ED=3;MIN_ED=3;NLOD=11.14;TLOD=5.04	GT:AD:AF:ALT_F1R2:ALT_F2R1:FOXOG:PGT:PID:QSS:REF_F1R2:REF_F2R1	0/1:72,2:0.028:0:2:0.00:0|1:85038391_A_T:2032,58:39:33	0/0:40,0:0.00:0:0:.:0|1:85038391_A_T:1099,0:15:25	2	Unknown	.	.	.	.	.	.	.	.
T4df	chr8	**1**	**12.5**	85038391	85038391	A	T	exonic	**Tnpo2**	ENSMUSP00000029639	.	nonsynonymous SNV	Tnpo2:NM_001122843:exon2:c.A1T:p.M1L,Tnpo2:NM_145390:exon2:c.A1T:p.M1L	**M1L**	ENSMUSP00000029639,1,M,L	8,85038391,A,A	8qC3	.	0.25	.	41	chr8	85038391	.	A	A	.	t_lod_fstar	ECNT=1;HCNT=1;MAX_ED=.;MIN_ED=.;NLOD=8.10;TLOD=5.11	GT:AD:AF:ALT_F1R2:ALT_F2R1:FOXOG:QSS:REF_F1R2:REF_F2R1	0/1:48,2:0.041:0:2:.:1365,58:30:18	0/0:31,0:0.00:0:0:.:879,0:9:22	6	Damaging	-0.41	Neutral	140	30	0	Damaging	4.32	29
T4df	chr8	**1**	**12.5**	85038394	85038394	G	C	exonic	**Tnpo2**	ENSMUSP00000029639	.	nonsynonymous SNV	Tnpo2:NM_001122843:exon2:c.G4C:p.D2H,Tnpo2:NM_145390:exon2:c.G4C:p.D2H	**D2H**	ENSMUSP00000029639,2,D,H	8,85038394,G,A	8qC3	.	0.25	.	40	chr8	85038394	.	G	A	.	alt_allele_in_normal;str_contraction	ECNT=1;HCNT=2;MAX_ED=.;MIN_ED=.;NLOD=4.95;RPA=7,6;RU=CAG;STR;TLOD=9.65	GT:AD:AF:ALT_F1R2:ALT_F2R1:FOXOG:QSS:REF_F1R2:REF_F2R1	0/1:56,4:0.071:3:1:.:1559,113:25:31	0/0:35,2:0.032:0:2:.:970,57:16:19	0.8	Damaging	-0.22	Neutral	140	30	0.004	Damaging	4.32	29
T2df	chr7	**1**	**12.5**	123162636	123162638	GCC	-	exonic	**Tnrc6a**	ENSMUSP00000079095	.	nonframeshift deletion	Tnrc6a:NM_144925:exon5:c.306_308del:p.102_103del	**102_103del**	NA	7,123162636,AGCC,C	7qF3	.	0.25	.	31	chr7	123162635	.	AGCC	C	.	t_lod_fstar	ECNT=1;HCNT=2;MAX_ED=.;MIN_ED=.;NLOD=15.42;TLOD=4.96	GT:AD:AF:ALT_F1R2:ALT_F2R1:FOXOG:QSS:REF_F1R2:REF_F2R1	0/1:80,3:0.025:2:1:.:2080,83:31:49	0/0:54,0:0.00:0:0:.:1412,0:33:21	19	Unknown	.	.	.	.	.	.	.	.
T3df	chr15	**1**	**12.5**	80902556	80902558	CAG	-	exonic	**Tnrc6b**	ENSMUSP00000036484	.	nonframeshift deletion	Tnrc6b:NM_177124:exon14:c.3787_3789del:p.1263_1263del,Tnrc6b:NM_144812:exon17:c.3895_3897del:p.1299_1299del	**1263_1263del**	NA	15,80902556,ACAG,C	15qE1	.	0.25	.	37	chr15	80902555	.	ACAG	C	.	t_lod_fstar	ECNT=1;HCNT=2;MAX_ED=.;MIN_ED=.;NLOD=7.77;TLOD=5.63	GT:AD:AF:ALT_F1R2:ALT_F2R1:FOXOG:QSS:REF_F1R2:REF_F2R1	0/1:31,3:0.088:3:0:1.00:879,86:15:16	0/0:29,0:0.00:0:0:.:805,0:13:16	2.1	Unknown	.	.	.	.	.	.	.	.
T3df	chr11	**1**	**12.5**	94214378	94214380	CAG	-	exonic	**Tob1**	ENSMUSP00000079557	.	nonframeshift deletion	Tob1:NM_009427:exon2:c.739_741del:p.247_247del	**247_247del**	NA	11,94214378,ACAG,C	11qD	rs215360916	0.25	.	28	chr11	94214377	.	ACAG	C	.	alt_allele_in_normal;clustered_events	ECNT=5;HCNT=1;MAX_ED=24;MIN_ED=1;NLOD=5.43;TLOD=11.84	GT:AD:AF:ALT_F1R2:ALT_F2R1:FOXOG:PGT:PID:QSS:REF_F1R2:REF_F2R1	0/1:50,4:0.075:4:0:.:0|1:136373432_T_TTTTTAATGATACG:1253,57:21:29	0/0:37,1:0.030:1:0:.:0|1:136373432_T_TTTTTAATGATACG:938,22:13:24	1.4	Unknown	.	.	.	.	.	.	.	.

T1df	chr11	1	12.5	94214431	94214433	GCA	-	exonic	**Tob1**	ENSMUSP00000079557	.	nonframeshift deletion	Tob1:NM_009427:exon2:c.792_794del:p.264_265del	**264_265del**	NA	11,94214431,CGCA,G	11qD	.	0.25	.	32	chr11	94214430	.	CGCA	G	.	alt_allele_in_normal;clustered_events	ECNT=5;HCNT=1;MAX_ED=24;MIN_ED=1;NLOD=5.43;RPA=3,1;RU=C;STR;TLOD=11.81	GT:AD:AF:ALT_F1R2:ALT_F2R1:FOXOG:PGT:PID:QSS:REF_F1R2:REF_F2R1	0/1:49,4:0.074:4:0:.:0|1:136373432_T_TTTTTAATGATACG:1370,115:23:26	0/0:38,1:0.030:1:0:.:0|1:136373432_T_TTTTTAATGATACG:1066,27:14:24	1.4	Unknown	.	.	.	.	.	.	.	.
T3df	chr1	1	12.5	156061972	156062034	CAGAGGGAGCAGGTGACAGGGGTGAAGCTGATGAGCACCTGGAGAGCAGCTCTGCAGCCCCCA	-	exonic	**Tor1aip2**	ENSMUSP00000008991	.	nonframeshift deletion	Tor1aip2:NM_172843:exon5:c.452_514del:p.151_172del	**151_172del**	NA	1,156061972,CCAGAGGGAGCAGGTGACAGGGGTGAAGCTGATGAGCACCTGGAGAGCAGCTCTGCAGCCCCCA,G	1qG3	.	0.25	.	54	chr1	156061971	.	CCAGAGGGAGCAGGTGACAGGGGTGAAGCTGATGAGCACCTGGAGAGCAGCTCTGCAGCCCCCA	G	.	t_lod_fstar	ECNT=1;HCNT=1;MAX_ED=.;MIN_ED=.;NLOD=10.09;TLOD=4.92	GT:AD:AF:ALT_F1R2:ALT_F2R1:FOXOG:QSS:REF_F1R2:REF_F2R1	0/1:65,3:0.045:2:1:0.333:1850,88:33:32	0/0:35,0:0.00:0:0:.:917,0:21:14	0.5	Unknown	.	.	.	.	.	.	.	.
T22df	chr1	1	12.5	43954598	43954598	G	C	exonic	**Tpp2**	ENSMUSP00000075488	.	nonsynonymous SNV	Tpp2:NM_001310540:exon6:c.G642C:p.E214D,Tpp2:NM_009418:exon6:c.G642C:p.E214D	**E214D**	ENSMUSP00000075488,214,E,D	1,43954598,G,G	1qC1.1	.	0.25	.	29	chr1	43954598	.	G	G	.	t_lod_fstar	ECNT=1;HCNT=1;MAX_ED=.;MIN_ED=.;NLOD=10.09;TLOD=4.92	GT:AD:AF:ALT_F1R2:ALT_F2R1:FOXOG:QSS:REF_F1R2:REF_F2R1	0/1:65,3:0.045:2:1:0.333:1850,88:33:32	0/0:35,0:0.00:0:0:.:917,0:21:14	0.5	Tolerated	0	Neutral	100	30	1	Tolerated	2.85	91
T42df	chr13	1	12.5	74031249	74031249	G	A	exonic	**Tppp**	ENSMUSP00000106584	.	nonsynonymous SNV	Tppp:NM_182839:exon4:c.G491A:p.R164Q	**R164Q**	ENSMUSP00000106584,164,R,Q	13,74031249,G,G	13qC1	.	0.25	.	33	chr13	74031249	.	G	G	.	clustered_events;homologous_mapping_event	DB;ECNT=6;HCNT=4;MAX_ED=191;MIN_ED=3;NLOD=10.23;TLOD=34.28	GT:AD:AF:ALT_F1R2:ALT_F2R1:FOXOG:QSS:REF_F1R2:REF_F2R1	0/1:8,12:0.600:8:4:0.333:225,344:5:3	0/0:37,0:0.00:0:0:.:1036,0:17:20	0.7	Tolerated	0	Neutral	131	30	1	Tolerated	2.93	133
T32df	chr13	1	12.5	74021319	74021319	A	-	exonic	**Tppp**	ENSMUSP00000106584	.	frameshift deletion	Tppp:NM_182839:exon2:c.177delA:p.V59fs	**V59fs**	ENSMUSP00000106584,59,V,.	13,74021319,TA,A	13qC1	.	0.25	.	30	chr13	74021318	.	TA	A	.	clustered_events;homologous_mapping_event	DB;ECNT=6;HCNT=4;MAX_ED=191;MIN_ED=3;NLOD=6.42;TLOD=49.39	GT:AD:AF:ALT_F1R2:ALT_F2R1:FOXOG:PGT:PID:QSS:REF_F1R2:REF_F2R1	0/1:15,14:0.481:10:4:0.286:0|1:29064857_GA_G:412,387:10:5	0/0:31,0:0.00:0:0:.:0|1:29064857_GAA_G:822,0:16:15	0.7	Unknown	NA	NA	131	30	NA	NA	NA	NA
T42df	chr3	1	12.5	84190720	84190720	A	G	exonic	**Trim2**	ENSMUSP00000009732	.	nonsynonymous SNV	Trim2:NM_001271725:exon6:c.T1300C:p.Y434H,Trim2:NM_001271726:exon6:c.T1222C:p.Y408H,Trim2:NM_001271727:exon6:c.T1222C:p.Y408H,Trim2:NM_001271728:exon6:c.T1222C:p.Y408H,Trim2:NM_030706:exon6:c.T1273C:p.Y425H	**Y434H**	ENSMUSP00000009732,434,Y,H	3,84190720,A,G	3qF1	.	0.25	.	35	chr3	84190720	.	A	G	.	t_lod_fstar	ECNT=1;HCNT=1;MAX_ED=.;MIN_ED=.;NLOD=10.09;TLOD=4.92	GT:AD:AF:ALT_F1R2:ALT_F2R1:FOXOG:QSS:REF_F1R2:REF_F2R1	0/1:65,3:0.045:2:1:0.333:1850,88:33:32	0/0:35,0:0.00:0:0:.:917,0:21:14	0.5	Damaging	-2.03	Neutral	151	30	0.038	Damaging	2.92	109
T42df	chr3	1	12.5	84190720	84190720	A	G	exonic	**Trim2**	ENSMUSP00000009732	.	nonsynonymous SNV	Trim2:NM_001271725:exon6:c.T1300C:p.Y434H,Trim2:NM_001271726:exon6:c.T1222C:p.Y408H,Trim2:NM_001271727:exon6:c.T1222C:p.Y408H,Trim2:NM_001271728:exon6:c.T1222C:p.Y408H,Trim2:NM_030706:exon6:c.T1273C:p.Y425H	**Y408H**	ENSMUSP00000009732,408,Y,H	3,84190720,A,T	3qF1	.	0.25	.	35	chr3	84190720	.	A	T	.	alt_allele_in_normal	ECNT=1;HCNT=1;MAX_ED=.;MIN_ED=.;NLOD=4.49;TLOD=16.63	GT:AD:AF:ALT_F1R2:ALT_F2R1:FOXOG:QSS:REF_F1R2:REF_F2R1	0/1:34,8:0.195:5:3:0.625:932,223:15:19	0/0:45,9:0.146:2:7:0.222:1220,236:19:26	0.2	Deleterious	-3.38	Deleterious	151	30	0.023	Damaging	2.92	109
T42df	chr3	1	12.5	84190720	84190720	A	G	exonic	**Trim2**	ENSMUSP00000009732	.	nonsynonymous SNV	Trim2:NM_001271725:exon6:c.T1300C:p.Y434H,Trim2:NM_001271726:exon6:c.T1222C:p.Y408H,Trim2:NM_001271727:exon6:c.T1222C:p.Y408H,Trim2:NM_001271728:exon6:c.T1222C:p.Y408H,Trim2:NM_030706:exon6:c.T1273C:p.Y425H	**Y425H**	ENSMUSP00000009732,425,Y,H	3,84190720,A,GGACACAGAAAGCCATCTCCAAGACAGAGATGGCT	3qF1	.	0.25	.	35	chr3	84190720	.	A	GGACACAGAAAGCCATCTCCAAGACAGAGATGGCT	.	t_lod_fstar	ECNT=1;HCNT=1;MAX_ED=.;MIN_ED=.;NLOD=11.50;TLOD=5.25	GT:AD:AF:ALT_F1R2:ALT_F2R1:FOXOG:QSS:REF_F1R2:REF_F2R1	0/1:54,1:0.036:1:0:.:1497,28:27:27	0/0:43,0:0.00:0:0:.:1227,0:20:23	1.3	Unknown	NA	NA	151	30	NA	NA	NA	NA
T22df	chr9	1	12.5	89089540	89089540	G	T	exonic	**Trim43b**	ENSMUSP00000100025	.	nonsynonymous SNV	Trim43b:NM_001170884:exon4:c.C562A:p.P188T	**P188T**	ENSMUSP00000100025,188,P,T	9,89089540,G,TG	9qE3.1	rs108062689	0.25	.	54	chr9	89089540	.	G	TG	.	alt_allele_in_normal	ECNT=1;HCNT=1;MAX_ED=.;MIN_ED=.;NLOD=7.73;RPA=7,8;RU=G;STR;TLOD=58.04	GT:AD:AF:ALT_F1R2:ALT_F2R1:FOXOG:QSS:REF_F1R2:REF_F2R1	0/1:30,41:0.582:16:25:.:861,1165:10:20	0/0:31,1:0.034:0:1:.:878,31:15:16	4	Unknown	NA	NA	170	30	NA	NA	NA	NA

T2df	chr11	**1**	**12.5**	62596936	62596936	-	GACACAGAAAGCCATCTCCAAGACAGAGATGGCT	exonic	**Trpv2**	ENSMUSP00000018311	.	frameshift insertion	Trpv2:NM_011706:exon14:c.2001_2002insGACACAGAAAGCCATCTCCAAGACAGAGATGGCT:p.E667fs	**E667fs**	ENSMUSP00000018311,667,E,	11,62596936,G,A	11qB2	.	0.25	.	43	chr11	62596936	.	G	A	.	PASS	DB;ECNT=1;HCNT=1;MAX_ED=.;MIN_ED=.;NLOD=9.78;TLOD=30.49	GT:AD:AF:ALT_F1R2:ALT_F2R1:FOXOG:QSS:REF_F1R2:REF_F2R1	0/1:21,12:0.364:7:5:0.583:485,341:10:11	0/0:36,0:0.00:0:0:.:859,0:16:20	1.7	Tolerated	0	Neutral	220	30	1	Tolerated	2.79	234
T1df	chr15	**1**	**12.5**	89103351	89103351	T	C	exonic	**Tubgcp6**	ENSMUSP00000022135	.	nonsynonymous SNV	Tubgcp6:NM_001163319:exon16:c.A3418G:p.T1140A	**T1140A**	ENSMUSP00000022135,1140,T,A	15,89103351,T,A	15qE3	.	0.25	.	29	chr15	89103351	.	T	A	.	clustered_events	DB;ECNT=2;HCNT=2;MAX_ED=34;MIN_ED=34;NLOD=9.87;TLOD=17.61	GT:AD:AF:ALT_F1R2:ALT_F2R1:FOXOG:QSS:REF_F1R2:REF_F2R1	0/1:36,8:0.190:1:7:0.875:907,227:14:22	0/0:38,0:0.00:0:0:.:957,0:22:16	1.4	Tolerated	-0.32	Neutral	56	30	0.63	Tolerated	3.11	40
T1df	chr4	**1**	**12.5**	41196043	41196043	-	AAACATAGTAGGCCCATGCTGGAAGGCACTGGGCACGCCTGTGTAGTAGGGGAGGCCGGTATAG	exonic	**Ubap2**	ENSMUSP00000047105	.	frameshift insertion	Ubap2:NM_026872:exon25:c.2838_2839insCTATACCGGCCTCCCCTACTACACAGGCGTGCCCAGTGCCTTCCAGCATGGGCCTACTATGTTT:p.V947fs	**V947fs**	ENSMUSP00000047105,947,V,	4,41196043,C,CTAGTCACTGAGCCCTTACTGTGATCCGATGAGTTGGAAGAGA	4qA5	.	0.25	.	41	chr4	41196043	.	C	CTAGTCACTGAGCCCTTACTGTGATCCGATGAGTTGGAAGAGA	.	clustered_events;homologous_mapping_event;t_lod_fstar	ECNT=3;HCNT=2;MAX_ED=6;MIN_ED=2;NLOD=11.51;TLOD=5.13	GT:AD:AF:ALT_F1R2:ALT_F2R1:FOXOG:PGT:PID:QSS:REF_F1R2:REF_F2R1	0/1:63,2:0.031:0:2:.:0|1:41199897_G_GGTTTTTTTA:1762,56:32:31	0/0:40,0:0.00:0:0:.:0|1:41199897_G_GGTTTTTTTA:1089,0:14:26	1.4	Unknown	NA	NA	169	30	NA	NA	NA	NA
T3df	chr4	**1**	**12.5**	41199897	41199897	-	GTTTTTTTTA	exonic	**Ubap2**	ENSMUSP00000047105	.	frameshift insertion	Ubap2:NM_026872:exon20:c.2231_2232insTAAAAAAAAC:p.A744fs	**A744fs**	ENSMUSP00000047105,744,A,	4,41199897,G,TCTCTTTGTGTCTGGCTGCTCCGTGCC	4qA5	.	0.25	.	40	chr4	41199897	.	G	TCTCTTTGTGTCTGGCTGCTCCGTGCC	.	alt_allele_in_normal;t_lod_fstar	ECNT=1;HCNT=2;MAX_ED=.;MIN_ED=.;NLOD=4.35;TLOD=4.20	GT:AD:AF:ALT_F1R2:ALT_F2R1:FOXOG:QSS:REF_F1R2:REF_F2R1	0/1:34,2:0.063:1:1:.:875,46:21:13	0/0:45,2:0.048:1:1:.:1069,42:20:25	0.7	Unknown	NA	NA	169	30	NA	NA	NA	NA
T3df	chr4	**1**	**12.5**	41199900	41199900	A	-	exonic	**Ubap2**	ENSMUSP00000047105	.	frameshift deletion	Ubap2:NM_026872:exon20:c.2229delT:p.H743fs	**H743fs**	ENSMUSP00000047105,743,H,.	4,41199900,CA,CTGAATATGGATTAT	4qA5	.	0.25	.	40	chr4	41199899	.	CA	CTGAATATGGATTAT	.	clustered_events	ECNT=2;HCNT=1;MAX_ED=3;MIN_ED=3;NLOD=9.93;TLOD=17.24	GT:AD:AF:ALT_F1R2:ALT_F2R1:FOXOG:PGT:PID:QSS:REF_F1R2:REF_F2R1	0/1:44,7:0.125:4:3:.:0|1:41196534_C_CTGAATATGGATTAT:1250,188:25:19	0/0:36,0:0.00:0:0:.:0|1:41196534_C_CTGAATATGGATTAT:1016,0:16:20	1.4	Unknown	NA	NA	169	30	NA	NA	NA	NA
T3df	chr4	**1**	**12.5**	41199903	41199903	-	TAGTCACTGAGCCCTTACTGTGATCCGATGAGTTGGAAGAGA	splicing	**Ubap2**	ENSMUSP00000047105	.	NM_026872:exon20:c.2227-1->TCTCTTCCAACTCATCGGATCACAGTAAGGGCTCAGTGACTA	.	**NA**	4,41199903,C,GGTTTTTTTA	NA	4qA5	.	0.25	.	40	chr4	41199903	.	C	GGTTTTTTTTA	.	clustered_events;homologous_mapping_event	ECNT=3;HCNT=2;MAX_ED=6;MIN_ED=2;NLOD=11.44;TLOD=11.35	GT:AD:AF:ALT_F1R2:ALT_F2R1:FOXOG:PGT:PID:QSS:REF_F1R2:REF_F2R1	0/1:66,4:0.061:1:3:.:0|1:41199897_G_GGTTTTTTTA:1823,106:34:32	0/0:40,0:0.00:0:0:.:0|1:41199897_G_GGTTTTTTTA:1098,0:14:26	1.4	Unkown	.	.	.	.	.	.	.	.
T4df	chr4	**1**	**12.5**	41196534	41196534	-	TGAATATGGATTAT	splicing	**Ubap2**	ENSMUSP00000047105	.	NM_026872:exon24:c.2633-1->ATAATCCATATTCA	.	**NA**	4,41196534,C,C	NA	4qA5	.	0.25	.	36	chr4	41196534	.	C	C	.	t_lod_fstar	ECNT=1;HCNT=1;MAX_ED=.;MIN_ED=.;NLOD=7.42;TLOD=4.38	GT:AD:AF:ALT_F1R2:ALT_F2R1:FOXOG:QSS:REF_F1R2:REF_F2R1	0/1:6,2:0.250:0:2:1.00:144,54:5:1	0/0:32,1:0.036:1:0:0.00:831,7:20:12	0.7	Unkown	.	.	.	.	.	.	.	.
T3df	chr14	**1**	**12.5**	18331685	18331685	A	C	splicing	**Ube2e1**	ENSMUSP00000023486	.	NM_009455:exon1:UTR6	.	**NA**	14,18331685,A,T	NA	14qA2	.	0.25	.	33	chr14	18331685	.	A	T	.	clustered_events;multi_event_alt_allele_in_normal;t_lod_fstar	ECNT=10;HCNT=4;MAX_ED=25;MIN_ED=6;NLOD=16.87;TLOD=5.37	GT:AD:AF:ALT_F1R2:ALT_F2R1:FOXOG:PGT:PID:QSS:REF_F1R2:REF_F2R1	0/1:51,2:0.041:2:0:0.00:0|1:89787219_TCCCA_T:1413,36:20:31	0/0:70,1:0.014:1:0:0.00:0|1:89787219_TCCCA_T:1925,27:35:35	0.9	Unkown	.	.	.	.	.	.	.	.
T2df	chr9	**1**	**12.5**	55149756	55149788	CGCCGCCGCCACCACCGCCGGGCAGCTCGCTTT	-	exonic	**Ube2q2**	ENSMUSP00000022727	.	nonframeshift deletion	Ube2q2:NM_001346657:exon1:c.122_154del:p.41_52del,Ube2q2:NM_180600:exon1:c.122_154del:p.41_52del	**41_52del**	NA	9,55149756,CCGCCGCCGCCACCACCGCCGGGCAGCTCGCTTT,T	9qB	.	0.25	.	49	chr9	55149755	.	CCGCCGCCGCCACCACCGCCGGGCAGCTCGCTTT	T	.	clustered_events;multi_event_alt_allele_in_normal;t_lod_fstar	ECNT=10;HCNT=4;MAX_ED=25;MIN_ED=6;NLOD=16.87;TLOD=5.35	GT:AD:AF:ALT_F1R2:ALT_F2R1:FOXOG:PGT:PID:QSS:REF_F1R2:REF_F2R1	0/1:51,2:0.040:2:0:0.00:0|1:89787219_TCCCA_T:1411,36:20:31	0/0:70,1:0.014:1:0:0.00:0|1:89787219_TCCCA_T:1971,25:35:35	0.9	Unknown	.	.	.	.	.	.	.	.
T22df	chr10	**1**	**12.5**	89787225	89787225	C	T	exonic	**Uhrf1bp1l**	ENSMUSP00000025137	.	nonsynonymous SNV	Uhrf1bp1l:NM_029166:exon8:c.C961T:p.H321Y	**H321Y**	ENSMUSP00000025137,321,H,Y	10,89787225,C,A	10qC2	.	0.25	.	71	chr10	89787225	.	C	A	.	clustered_events;multi_event_alt_allele_in_normal;t_lod_fstar	ECNT=10;HCNT=4;MAX_ED=25;MIN_ED=6;NLOD=17.17;TLOD=5.37	GT:AD:AF:ALT_F1R2:ALT_F2R1:FOXOG:PGT:PID:QSS:REF_F1R2:REF_F2R1	0/1:53,2:0.041:2:0:1.00:0|1:89787219_TCCCA_T:1441,36:21:32	0/0:71,1:0.014:1:0:1.00:0|1:89787219_TCCCA_T:1947,27:35:36	0.9	Deleterious	-6.6	Deleterious	150	30	0	Damaging	2.91	89

T22df	chr10	**1**	**12.5**	89787244	89787244	T	G	exonic	**Uhrf1bp1l**	ENSMUSP00000025137	.	nonsynonymous SNV	Uhrf1bp1l:NM_029166:exon8:c.T980G:p.L327R	**L327R**	ENSMUSP00000025137,327,L,R	10,89787244,T,A	10qC2	.	0.25	.	70	chr10	89787244	.	T	A	.	clustered_events;multi_event_alt_allele_in_normal;t_lod_fstar	ECNT=10;HCNT=4;MAX_ED=25;MIN_ED=6;NLOD=18.08;RPA=3,2;RU=T;STR;TLOD=5.41	GT:AD:AF:ALT_F1R2:ALT_F2R1:FOXOG:PGT:PID:QSS:REF_F1R2:REF_F2R1	0/1:54,2:0.043:2:0:.:0|1:89787219_TCCCA_T:1475,54:22:32	0/0:72,1:0.013:1:0:.:0|1:89787219_TCCCA_T:1936,28:35:37	0.9	Deleterious	-5.32	Deleterious	150	30	0	Damaging	2.91	89
T22df	chr10	**1**	**12.5**	89787238	89787238	-	GGCGA	exonic	**Uhrf1bp1l**	ENSMUSP00000025137	.	frameshift insertion	Uhrf1bp1l:NM_029166:exon8:c.974_975insGGCGA:p.S325fs	**S325fs**	ENSMUSP00000025137,325,S,	10,89787238,C,T	10qC2	.	0.25	.	71	chr10	89787238	.	C	T	.	homologous_mapping_event;t_lod_fstar	ECNT=3;HCNT=4;MAX_ED=2;MIN_ED=1;NLOD=10.54;TLOD=5.37	GT:AD:AF:ALT_F1R2:ALT_F2R1:FOXOG:PGT:PID:QSS:REF_F1R2:REF_F2R1	0/1:49,2:0.041:0:2:1.00:0|1:66654494_G_T:1414,56:27:22	0/0:40,0:0.00:0:0:.:0|1:66654494_G_T:1110,0:20:20	0.6	Deleterious	-4.49	Deleterious	150	30	0	Damaging	2.91	89
T22df	chr10	**1**	**12.5**	89787226	89787226	A	T	exonic	**Uhrf1bp1l**	ENSMUSP00000025137	.	nonsynonymous SNV	Uhrf1bp1l:NM_029166:exon8:c.A962T:p.H321L	**H321L**	ENSMUSP00000025137,321,H,L	10,89787226,A,A	10qC2	.	0.25	.	71	chr10	89787226	.	A	A	.	clustered_events;multi_event_alt_allele_in_normal;t_lod_fstar	ECNT=10;HCNT=4;MAX_ED=25;MIN_ED=6;NLOD=17.17;TLOD=5.41	GT:AD:AF:ALT_F1R2:ALT_F2R1:FOXOG:PGT:PID:QSS:REF_F1R2:REF_F2R1	0/1:53,2:0.043:2:0:1.00:0|1:89787219_TCCCA_T:1428,38:22:31	0/0:71,1:0.014:1:0:1.00:0|1:89787219_TCCCA_T:1856,23:35:36	0.9	Tolerated	0	Neutral	150	30	1	Tolerated	2.91	89
T22df	chr10	**1**	**12.5**	89787231	89787231	G	A	exonic	**Uhrf1bp1l**	ENSMUSP00000025137	.	nonsynonymous SNV	Uhrf1bp1l:NM_029166:exon8:c.G967A:p.V323M	**V323M**	ENSMUSP00000025137,323,V,M	10,89787231,G,G	10qC2	.	0.25	.	72	chr10	89787231	.	G	G	.	clustered_events;multi_event_alt_allele_in_normal;t_lod_fstar	ECNT=10;HCNT=4;MAX_ED=25;MIN_ED=6;NLOD=16.22;TLOD=5.41	GT:AD:AF:ALT_F1R2:ALT_F2R1:FOXOG:PGT:PID:QSS:REF_F1R2:REF_F2R1	0/1:46,2:0.043:2:0:1.00:0|1:89787219_TCCCA_T:1247,35:19:27	0/0:69,1:0.014:1:0:1.00:0|1:89787219_TCCCA_T:1852,28:34:35	0.9	Tolerated	0	Neutral	150	30	0.751	Tolerated	2.91	90
T22df	chr10	**1**	**12.5**	89787237	89787237	T	A	exonic	**Uhrf1bp1l**	ENSMUSP00000025137	.	nonsynonymous SNV	Uhrf1bp1l:NM_029166:exon8:c.T973A:p.S325T	**S325T**	ENSMUSP00000025137,325,S,T	10,89787237,T,T	10qC2	.	0.25	.	72	chr10	89787237	.	T	T	.	clustered_events;multi_event_alt_allele_in_normal;t_lod_fstar	ECNT=10;HCNT=4;MAX_ED=25;MIN_ED=6;NLOD=16.87;TLOD=5.29	GT:AD:AF:ALT_F1R2:ALT_F2R1:FOXOG:PGT:PID:QSS:REF_F1R2:REF_F2R1	0/1:51,2:0.037:2:0:.:0|1:89787219_TCCCA_T:1443,45:21:30	0/0:71,1:0.014:1:0:.:0|1:89787219_TCCCA_T:1999,24:36:35	0.9	Tolerated	0	Neutral	150	30	1	Tolerated	2.91	89
T22df	chr10	**1**	**12.5**	89787220	89787223	CCCA	-	exonic	**Uhrf1bp1l**	ENSMUSP00000025137	.	frameshift deletion	Uhrf1bp1l:NM_029166:exon8:c.956_959del:p.S319fs	**S319fs**	ENSMUSP00000025137,319,S,.	10,89787220,TCCCA,CGGCGA	10qC2	.	0.25	.	72	chr10	89787219	.	TCCCA	CGGCGA	.	clustered_events;multi_event_alt_allele_in_normal;t_lod_fstar	ECNT=10;HCNT=4;MAX_ED=25;MIN_ED=6;NLOD=17.78;TLOD=5.35	GT:AD:AF:ALT_F1R2:ALT_F2R1:FOXOG:PGT:PID:QSS:REF_F1R2:REF_F2R1	0/1:49,2:0.040:2:0:.:0|1:89787219_TCCCA_T:1253,27:20:29	0/0:70,1:0.014:1:0:.:0|1:89787219_TCCCA_T:1774,22:34:36	0.9	Unknown	NA	NA	150	30	NA	NA	NA	NA
T22df	chr10	**1**	**12.5**	89787235	89787235	T	-	exonic	**Uhrf1bp1l**	ENSMUSP00000025137	.	frameshift deletion	Uhrf1bp1l:NM_029166:exon8:c.971delT:p.I324fs	**I324fs**	ENSMUSP00000025137,324,I,.	10,89787235,AT,T	10qC2	.	0.25	.	73	chr10	89787234	.	AT	T	.	homologous_mapping_event;t_lod_fstar	ECNT=3;HCNT=4;MAX_ED=2;MIN_ED=1;NLOD=10.54;TLOD=5.37	GT:AD:AF:ALT_F1R2:ALT_F2R1:FOXOG:PGT:PID:QSS:REF_F1R2:REF_F2R1	0/1:48,2:0.041:0:2:0.00:0|1:66654494_G_T:1393,56:26:22	0/0:40,0:0.00:0:0:.:0|1:66654494_G_T:1146,0:20:20	0.6	Unknown	NA	NA	150	30	NA	NA	NA	NA
T1df	chr1	**1**	**12.5**	66654494	66654494	G	T	exonic	**Unc80**	ENSMUSP00000100876	.	nonsynonymous SNV	Unc80:NM_175510:exon51:c.G7703T:p.C2568F	**C2568F**	ENSMUSP00000100876,2568,C,F	1,66654494,G,C	1qC3	.	0.25	.	40	chr1	66654494	.	G	C	.	t_lod_fstar	ECNT=1;HCNT=1;MAX_ED=.;MIN_ED=.;NLOD=13.49;TLOD=5.13	GT:AD:AF:ALT_F1R2:ALT_F2R1:FOXOG:QSS:REF_F1R2:REF_F2R1	0/1:35,3:0.081:2:1:0.667:939,82:21:14	0/0:53,0:0.00:0:0:.:1504,0:21:32	0.9	Deleterious	-3.13	Deleterious	97	30	0.051	Tolerated	2.91	107
T1df	chr1	**1**	**12.5**	66654496	66654496	A	T	exonic	**Unc80**	ENSMUSP00000100876	.	nonsynonymous SNV	Unc80:NM_175510:exon51:c.A7705T:p.T2569S	**T2569S**	ENSMUSP00000100876,2569,T,S	1,66654496,A,G	1qC3	.	0.25	.	40	chr1	66654496	.	A	G	.	t_lod_fstar	ECNT=1;HCNT=1;MAX_ED=.;MIN_ED=.;NLOD=7.83;TLOD=5.42	GT:AD:AF:ALT_F1R2:ALT_F2R1:FOXOG:QSS:REF_F1R2:REF_F2R1	0/1:62,2:0.042:2:0:.:1509,51:30:32	0/0:36,0:0.00:0:0:.:848,0:23:13	1.1	Tolerated	0.22	Neutral	97	30	1	Tolerated	2.91	107
T12df	chrX	**1**	**12.5**	57101894	57101932	TGTCTTTCCCGACAGGCATCTGACTCCAGAGGTCTACCA	-	exonic	**Vgll1**	ENSMUSP00000016309	.	nonframeshift deletion	Vgll1:NM_133251:exon6:c.597_635del:p.199_212del,Vgll1:NM_001313763:exon7:c.597_635del:p.199_212del	**199_212del**	NA	X,57101894,GTGTCTTTCCCGACAGGCATCTGACTCCAGAGGTCTACCA,G	XqA6	.	0.25	.	36	chrX	57101893	.	GTGTCTTTCCCGACAGGCATCTGACTCCAGAGGTCTACCA	G	.	clustered_events;multi_event_alt_allele_in_normal	ECNT=4;HCNT=28;MAX_ED=33;MIN_ED=8;NLOD=6.04;TLOD=21.07	GT:AD:AF:ALT_F1R2:ALT_F2R1:FOXOG:PGT:PID:QSS:REF_F1R2:REF_F2R1	0/1:53,7:0.127:4:3:0.429:0|1:33962974_T_G:1506,188:25:28	0/0:40,1:0.029:0:1:1.00:0|1:33962974_T_G:1099,28:28:12	1	Unknown	.	.	.	.	.	.	.	.

T2df	chr17	**1**	**12.5**	33962919	33962919	G	C	exonic	**Vps52**	ENSMUSP00000121814	.	nonsynonymous SNV	Vps52:NM_001357329:exon16:c.G1255C:p.E419Q,Vps52:NM_001357330:exon16:c.G874C:p.E292Q,Vps52:NM_172620:exon16:c.G1630C:p.E544Q	**E419Q**	ENSMUSP00000121814,419,E,Q	17,33962919,G,C	17qB1	.	0.25	.	37	chr17	33962919	.	G	C	.	clustered_events;homologous_mapping_event;t_lod_fstar	ECNT=5;HCNT=8;MAX_ED=88;MIN_ED=55;NLOD=10.23;TLOD=4.72	GT:AD:AF:ALT_F1R2:ALT_F2R1:FOXOG:QSS:REF_F1R2:REF_F2R1	0/1:51,4:0.073:2:2:0.500:1416,116:32:19	0/0:37,0:0.00:0:0:.:1030,0:22:15	1	Tolerated	-1.93	Neutral	108	30	0.168	Tolerated	2.85	107
T2df	chr17	**1**	**12.5**	33962919	33962919	G	C	exonic	**Vps52**	ENSMUSP00000121814	.	nonsynonymous SNV	Vps52:NM_001357329:exon16:c.G1255C:p.E419Q,Vps52:NM_001357330:exon16:c.G874C:p.E292Q,Vps52:NM_172620:exon16:c.G1630C:p.E544Q	**E292Q**	ENSMUSP00000121814,292,E,Q	17,33962919,G,T	17qB1	.	0.25	.	37	chr17	33962919	.	G	T	.	PASS	DB;ECNT=1;HCNT=1;MAX_ED=.;MIN_ED=.;NLOD=11.41;TLOD=19.49	GT:AD:AF:ALT_F1R2:ALT_F2R1:FOXOG:QSS:REF_F1R2:REF_F2R1	0/1:20,9:0.296:5:4:0.444:509,255:13:7	0/0:40,0:0.00:0:0:.:974,0:19:21	3	Unknown	NA	NA	108	30	NA	NA	NA	NA
T2df	chr17	**1**	**12.5**	33962919	33962919	G	C	exonic	**Vps52**	ENSMUSP00000121814	.	nonsynonymous SNV	Vps52:NM_001357329:exon16:c.G1255C:p.E419Q,Vps52:NM_001357330:exon16:c.G874C:p.E292Q,Vps52:NM_172620:exon16:c.G1630C:p.E544Q	**E544Q**	ENSMUSP00000121814,544,E,Q	17,33962919,G,T	17qB1	.	0.25	.	37	chr17	33962919	.	G	T	.	clustered_events;homologous_mapping_event	DB;ECNT=3;HCNT=4;MAX_ED=119;MIN_ED=57;NLOD=11.72;TLOD=69.63	GT:AD:AF:ALT_F1R2:ALT_F2R1:FOXOG:QSS:REF_F1R2:REF_F2R1	0/1:23,26:0.543:10:16:0.385:568,748:14:9	0/0:45,0:0.00:0:0:.:1128,0:22:23	3	Unknown	NA	NA	108	30	NA	NA	NA	NA
T1df	chr14	**1**	**12.5**	33099105	33099105	-	GGATCAACT	exonic	**Wdfy4**	ENSMUSP00000027800	.	nonframeshift insertion	Wdfy4:NM_001146022:exon23:c.4165_4166insAGTTGATCC:p.L1389delinsQLIL	**L1389delinsQLIL**	ENSMUSP00000027800,1389,L,QLIL	14,33099105,A,A	14qB	.	0.25	.	78	chr14	33099105	.	A	A	.	clustered_events;multi_event_alt_allele_in_normal	ECNT=5;HCNT=1;MAX_ED=26;MIN_ED=5;NLOD=6.64;TLOD=14.68	GT:AD:AF:ALT_F1R2:ALT_F2R1:FOXOG:PGT:PID:QSS:REF_F1R2:REF_F2R1	0/1:67,5:0.071:0:5:.:0|1:75452968_G_GTGGTCGCC:1726,129:33:34	0/0:38,1:0.027:0:1:.:0|1:75452968_G_GTGGTCGCC:1028,25:19:19	0.7	Tolerated	0	Neutral	105	30	0.443	Tolerated	2.83	94
T12df	chr11	**1**	**12.5**	75452989	75452989	-	TAAAAA	exonic	**Wdr81**	ENSMUSP00000124860	.	nonframeshift insertion	Wdr81:NM_138950:exon1:c.1450_1451insTTTTTA:p.W484delinsFFR	**W484delinsFFR**	ENSMUSP00000124860,484,W,FFR	11,75452989,C,G	11qB5	.	0.25	.	41	chr11	75452989	.	C	G	.	PASS	ECNT=1;HCNT=1;MAX_ED=.;MIN_ED=.;NLOD=8.42;TLOD=20.44	GT:AD:AF:ALT_F1R2:ALT_F2R1:FOXOG:QSS:REF_F1R2:REF_F2R1	0/1:33,9:0.220:4:5:0.556:918,255:14:19	0/0:31,0:0.00:0:0:.:866,0:9:22	7	Deleterious	-12.84	Deleterious	83	30	0	Damaging	2.89	33
T12df	chr11	**1**	**12.5**	75452968	75452968	-	TGGTCGCC	exonic	**Wdr81**	ENSMUSP00000124860	.	frameshift insertion	Wdr81:NM_138950:exon1:c.1471_1472insGGCGACCA:p.P491fs	**P491fs**	ENSMUSP00000124860,491,P,	11,75452968,G,CTAAAAA	11qB5	.	0.25	.	38	chr11	75452968	.	G	CTAAAAA	.	clustered_events;multi_event_alt_allele_in_normal	ECNT=5;HCNT=1;MAX_ED=26;MIN_ED=5;NLOD=6.94;TLOD=14.74	GT:AD:AF:ALT_F1R2:ALT_F2R1:FOXOG:PGT:PID:QSS:REF_F1R2:REF_F2R1	0/1:64,5:0.074:0:5:.:0|1:75452968_G_GTGGTCGCC:1713,59:31:33	0/0:40,1:0.026:0:1:.:0|1:75452968_G_GTGGTCGCC:1073,10:19:21	0.7	Deleterious	-17.1	Deleterious	83	30	NA	NA	NA	NA
T12df	chr11	**1**	**12.5**	75452963	75452963	A	T	exonic	**Wdr81**	ENSMUSP00000124860	.	nonsynonymous SNV	Wdr81:NM_138950:exon1:c.T1477A:p.F493I	**F493I**	ENSMUSP00000124860,493,F,I	11,75452963,A,GTGGTCGCC	11qB5	.	0.25	.	35	chr11	75452963	.	A	GTGGTCGCC	.	clustered_events;multi_event_alt_allele_in_normal	ECNT=5;HCNT=1;MAX_ED=26;MIN_ED=5;NLOD=6.94;TLOD=14.61	GT:AD:AF:ALT_F1R2:ALT_F2R1:FOXOG:PGT:PID:QSS:REF_F1R2:REF_F2R1	0/1:66,5:0.072:0:5:.:0|1:75452968_G_GTGGTCGCC:1764,136:31:34	0/0:37,1:0.029:0:1:.:0|1:75452968_G_GTGGTCGCC:993,27:20:17	0.7	Unknown	NA	NA	83	30	NA	NA	NA	NA
T12df	chr11	**1**	**12.5**	75452972	75452976	TGCAC	-	exonic	**Wdr81**	ENSMUSP00000124860	.	frameshift deletion	Wdr81:NM_138950:exon1:c.1464_1468del:p.E488fs	**E488fs**	ENSMUSP00000124860,488,E,.	11,75452972,ATGCAC,T	11qB5	.	0.25	.	39	chr11	75452971	.	ATGCAC	T	.	clustered_events;multi_event_alt_allele_in_normal	ECNT=5;HCNT=1;MAX_ED=26;MIN_ED=5;NLOD=7.54;TLOD=14.74	GT:AD:AF:ALT_F1R2:ALT_F2R1:FOXOG:PGT:PID:QSS:REF_F1R2:REF_F2R1	0/1:66,5:0.074:0:5:.:0|1:75452968_G_GTGGTCGCC:1883,68:31:35	0/0:42,1:0.025:0:1:.:0|1:75452968_G_GTGGTCGCC:1158,10:21:21	0.7	Unknown	NA	NA	83	30	NA	NA	NA	NA
T12df	chr11	**1**	**12.5**	75452981	75452989	CCGGTGTCC	-	exonic	**Wdr81**	ENSMUSP00000124860	.	nonframeshift deletion	Wdr81:NM_138950:exon1:c.1451_1459del:p.484_487del	**484_487del**	NA	11,75452981,TCCGGTGTCC,G	11qB5	.	0.25	.	43	chr11	75452980	.	TCCGGTGTCC	G	.	PASS	ECNT=1;HCNT=1;MAX_ED=.;MIN_ED=.;NLOD=8.42;TLOD=20.44	GT:AD:AF:ALT_F1R2:ALT_F2R1:FOXOG:QSS:REF_F1R2:REF_F2R1	0/1:33,9:0.220:4:5:0.556:918,255:14:19	0/0:31,0:0.00:0:0:.:866,0:9:22	7	Unknown	.	.	.	.	.	.	.	.
T2df	chrX	**1**	**12.5**	103467422	103467481	ATAGACACACAAAGCAAGGAAGAATAGATGCAACAACGAATTAGACAACACAAGGAGAC	-	ncRNA_exonic	**Xist**	NONE	.	.	.	**NA**	X,103467422,AATAGACACACAAAGCAAGGAAGAAATAGATGCAACAACGAATTAGACAACACAAGGAGAC,G	NA	XqD	.	0.25	.	63	chrX	103467421	.	AATAGACACACAAAGCAAGGAAGAAATAGATGCAACAACGAATTAGACAACACAAGGAGAC	G	.	PASS	ECNT=1;HCNT=1;MAX_ED=.;MIN_ED=.;NLOD=9.02;TLOD=8.12	GT:AD:AF:ALT_F1R2:ALT_F2R1:FOXOG:QSS:REF_F1R2:REF_F2R1	0/1:3,3:0.500:3:0:1.00:83,85:2:1	0/0:30,0:0.00:0:0:.:835,0:20:10	0.8	Unkown	.	.	.	.	.	.	.	.

T3df	chrX	**1**	**12.5**	103474026	103474026	G	A	ncRNA_exonic	**Xist**	NONE	.	.	.	**NA**	X,103474026,G,T	NA	XqD	.	0.25	.	45	chrX	103474026	.	G	T	.	alt_allele_in_normal;clustered_events	ECNT=4;HCNT=8;MAX_ED=89;MIN_ED=10;NLOD=6.06;TLOD=11.11	GT:AD:AF:ALT_F1R2:ALT_F2R1:FOXOG:PGT:PID:QSS:REF_F1R2:REF_F2R1	0/1:137,5:0.035:3:2:0.600:0|1:59766995_C_A:3984,145:66:71	0/0:293,9:0.034:7:2:0.778:0|1:59766995_C_A:8305,256:155:138	0.5	Unkown	.	.	.	.	.	.	.	.
T42df	chr4	**1**	**12.5**	119279200	119279200	-	TTCGGAATCG	splicing	**Ybx1**	ENSMUSP00000133076	.	NM_011732:exon7:c.735-1->CGATTCCGAA	.	**NA**	4,119279200,C,C	NA	4qD2.1	.	0.25	.	45	chr4	119279200	.	C	C	.	str_contraction;t_lod_fstar	ECNT=1;HCNT=2;MAX_ED=.;MIN_ED=.;NLOD=8.38;RPA=7,6;RU=CAG;STR;TLOD=5.55	GT:AD:AF:ALT_F1R2:ALT_F2R1:FOXOG:QSS:REF_F1R2:REF_F2R1	0/1:31,2:0.065:2:0:.:877,59:15:16	0/0:34,0:0.00:0:0:.:941,0:12:22	0.5	Tolerated	0	Neutral	202	30	1	Tolerated	2.85	70
T12df	chr4	**1**	**12.5**	119282446	119282446	-	ACCTTTTCT	exonic	**Ybx1**	ENSMUSP00000133076	.	nonframeshift insertion	Ybx1:NM_011732:exon5:c.350_351insAGAAAAGGT:p.G117delinsGEKV	**G117delinsGEKV**	ENSMUSP00000133076,117,G,GEKV	4,119282446,A,TTTTCTCCTTCA	4qD2.1	.	0.25	.	40	chr4	119282446	.	A	TTTTCTCCTTCA	.	alt_allele_in_normal;clustered_events	ECNT=5;HCNT=8;MAX_ED=35;MIN_ED=24;NLOD=3.04;TLOD=8.98	GT:AD:AF:ALT_F1R2:ALT_F2R1:FOXOG:PGT:PID:QSS:REF_F1R2:REF_F2R1	0/1:33,3:0.081:2:1:.:0|1:119282444_G_A:931,80:13:20	0/0:39,1:0.050:1:0:.:0|1:119282444_G_A:1110,27:18:21	1.9	Unknown	NA	NA	213	30	NA	NA	NA	NA
T12df	chr4	**1**	**12.5**	119282449	119282449	C	T	splicing	**Ybx1**	ENSMUSP00000133076	.	NM_011732:exon5:c.349-1G>A	.	**NA**	4,119282449,C,A	NA	4qD2.1	.	0.25	.	38	chr4	119282449	.	C	A	.	alt_allele_in_normal;clustered_events	ECNT=5;HCNT=8;MAX_ED=35;MIN_ED=24;NLOD=2.74;TLOD=9.05	GT:AD:AF:ALT_F1R2:ALT_F2R1:FOXOG:PGT:PID:QSS:REF_F1R2:REF_F2R1	0/1:33,3:0.086:2:1:0.333:0|1:119282444_G_A:909,84:13:20	0/0:40,2:0.051:1:1:0.500:0|1:119282444_G_A:1084,55:18:22	1.9	Unkown	.	.	.	.	.	.	.	.
T1df	chr6	**1**	**12.5**	131376158	131376160	TCC	-	exonic	**Ybx3**	ENSMUSP00000091595	.	nonframeshift deletion	Ybx3:NM_139117:exon6:c.570_572del:p.190_191del	**190_191del**	NA	6,131376158,TTCC,G	6qF3	.	0.25	.	30	chr6	131376157	.	TTCC	G	.	PASS	ECNT=1;HCNT=1;MAX_ED=.;MIN_ED=.;NLOD=12.63;TLOD=11.80	GT:AD:AF:ALT_F1R2:ALT_F2R1:FOXOG:QSS:REF_F1R2:REF_F2R1	0/1:15,5:0.250:4:1:0.800:430,143:6:9	0/0:51,0:0.00:0:0:.:1463,0:19:32	0.4	Unknown	.	.	.	.	.	.	.	.
T32df	chr12	**1**	**12.5**	108793632	108793634	CAC	-	exonic	**Yy1**	ENSMUSP00000119570	.	nonframeshift deletion	Yy1:NM_009537:exon1:c.220_222del:p.74_74del	**74_74del**	NA	12,108793632,TCAC,T	12qF1	.	0.25	.	19	chr12	108793631	.	TCAC	T	.	t_lod_fstar	ECNT=1;HCNT=1;MAX_ED=.;MIN_ED=.;NLOD=6.10;RPA=4,1;RU=CCGGGTACTGAAGGTCCCTGGCTGGCTGGTGTC;STR;TLOD=5.39	GT:AD:AF:ALT_F1R2:ALT_F2R1:FOXOG:QSS:REF_F1R2:REF_F2R1	0/1:60,2:0.033:2:0:.:1479,56:27:33	0/0:41,1:0.025:0:1:.:1000,28:22:19	0.7	Unknown	.	.	.	.	.	.	.	.
T1df	chr10	**1**	**12.5**	127742420	127742420	-	TTGGGCAGATGGCCTGTTTGAGCAATAGCTACCACTCCAGAGATCCAAGCA	exonic	**Zbtb39**	ENSMUSP00000121495	.	nonframeshift insertion	Zbtb39:NM_198035:exon2:c.862_863insTTGGGCAGATGGCCTGTTTGAGCAATAGCTACCACTCCAGAGATCCAAGCA:p.F288delinsFGQMACLSNSYHSRDPSI	**F288delinsFGQMACLSNSYHSRDPSI**	ENSMUSP00000121495,288,F,FGQMACLSNSYHSRDPSI	10,127742420,T,CACGGTTTCTTCCGGCCTTGTCCATCCGGCCATGGCTGTGTCGACGAGGCCAGCGGAACCACCAAAGCTGTGGCTACCTCCTGCTAGAT	10qD3	.	0.25	.	52	chr10	127742420	.	T	CACGGTTTCTTCCGGCCTTGTCCATCCGGCCATGGCTGTGTCGACGAGGCCAGCGGAACCACCAAAGCTGTGGCTACCTCCTGCTAGAT	.	PASS	ECNT=1;HCNT=1;MAX_ED=.;MIN_ED=.;NLOD=10.01;TLOD=7.48	GT:AD:AF:ALT_F1R2:ALT_F2R1:FOXOG:QSS:REF_F1R2:REF_F2R1	0/1:36,1:0.077:1:0:.:1038,28:13:23	0/0:36,0:0.00:0:0:.:1034,0:15:21	0.5	Unknown	NA	NA	280	30	NA	NA	NA	NA
T4df	chr11	**1**	**12.5**	53332518	53332520	CAG	-	exonic	**Zcchc10**	ENSMUSP00000145912	.	nonframeshift deletion	Zcchc10:NM_026479:exon4:c.462_464del:p.154_155del	**154_155del**	NA	11,53332518,CCAG,G	11qB1.3	rs214585133	0.25	.	34	chr11	53332517	.	CCAG	G	.	PASS	DB;ECNT=1;HCNT=1;MAX_ED=.;MIN_ED=.;NLOD=14.09;TLOD=39.87	GT:AD:AF:ALT_F1R2:ALT_F2R1:FOXOG:QSS:REF_F1R2:REF_F2R1	0/1:21,16:0.455:9:7:0.438:600,456:5:16	0/0:54,0:0.00:0:0:.:1511,0:33:21	1	Unknown	.	.	.	.	.	.	.	.
T32df	chr19	**1**	**12.5**	55302564	55302564	T	G	exonic	**Zdhhc6**	ENSMUSP00000036039	.	nonsynonymous SNV	Zdhhc6:NM_001033573:exon10:c.A1132C:p.I378L,Zdhhc6:NM_025883:exon10:c.A1132C:p.I378L	**I378L**	ENSMUSP00000036039,378,I,L	19,55302564,T,G	19qD2	.	0.25	.	51	chr19	55302564	.	T	G	.	PASS	DB;ECNT=1;HCNT=1;MAX_ED=.;MIN_ED=.;NLOD=14.09;TLOD=39.87	GT:AD:AF:ALT_F1R2:ALT_F2R1:FOXOG:QSS:REF_F1R2:REF_F2R1	0/1:21,16:0.455:9:7:0.438:600,456:5:16	0/0:54,0:0.00:0:0:.:1511,0:33:21	1	Tolerated	-0.25	Neutral	105	30	0.33	Tolerated	2.85	78

T3df	chr13	**1**	**12.5**	21520444	21520527	TCTCCCCACTGTGGATTCTCTGGTGCAAAATAAGGCCTGCACTCTGACTAAAGGCCTTCCCACACTGATTACATTGATAGGGCT	-	exonic	**Zkscan8**	ENSMUSP00000049902	.	nonframeshift deletion	Zkscan8:NM_001251833:exon4:c.1025_1108del:p.342_370del,Zkscan8:NM_139141:exon6:c.1241_1324del:p.414_442del	**414_442del**	NA	13,21520444,CTCTCCCCACTGTGGATTCTCTGGTGCAAAATAAGGCCTGCACTCTGACTAAAGGCCTTCCCACACTGATTACATTGATAGGGCT,C	13qA3.1	.	0.25	.	56	chr13	21520443	.	CTCTCCCCACTGTGGATTCTCTGGTGCAAAATAAGGCCTGCACTCTGACTAAAGGCCTTCCCACACTGATTACATTGATAGGGCT	C	.	t_lod_fstar	ECNT=1;HCNT=1;MAX_ED=.;MIN_ED=.;NLOD=10.23;TLOD=4.42	GT:AD:AF:ALT_F1R2:ALT_F2R1:FOXOG:QSS:REF_F1R2:REF_F2R1	0/1:34,4:0.111:4:0:1.00:956,88:14:20	0/0:38,0:0.00:0:0:.:1053,0:19:19	1.1	Unknown	.	.	.	.	.	.	.	.
T3df	chr13	**1**	**12.5**	21520444	21520527	TCTCCCCACTGTGGATTCTCTGGTGCAAAATAAGGCCTGCACTCTGACTAAAGGCCTTCCCACACTGATTACATTGATAGGGCT	-	exonic	**Zkscan8**	ENSMUSP00000049902	.	nonframeshift deletion	Zkscan8:NM_001251833:exon4:c.1025_1108del:p.342_370del,Zkscan8:NM_139141:exon6:c.1241_1324del:p.414_442del	**342_370del**	NA	13,21520444,CTCTCCCCACTGTGGATTCTCTGGTGCAAAATAAGGCCTGCACTCTGACTAAAGGCCTTCCCACACTGATTACATTGATAGGGCT,T	13qA3.1	.	0.25	.	56	chr13	21520443	.	CTCTCCCCACTGTGGATTCTCTGGTGCAAAATAAGGCCTGCACTCTGACTAAAGGCCTTCCCACACTGATTACATTGATAGGGCT	T	.	t_lod_fstar	ECNT=1;HCNT=1;MAX_ED=.;MIN_ED=.;NLOD=9.88;TLOD=6.03	GT:AD:AF:ALT_F1R2:ALT_F2R1:FOXOG:QSS:REF_F1R2:REF_F2R1	0/1:17,3:0.158:3:0:0.00:485,82:11:6	0/0:37,0:0.00:0:0:.:1065,0:17:20	2.5	Unknown	.	.	.	.	.	.	.	.
T22df	chr6	**1**	**12.5**	54884797	54884797	C	T	exonic	**Znrf2**	ENSMUSP00000103321	.	nonsynonymous SNV	Znrf2:NM_199143:exon4:c.C692T:p.S231F	**S231F**	ENSMUSP00000103321,231,S,F	6,54884797,C,T	6qB3	.	0.25	.	37	chr6	54884797	.	C	T	.	alt_allele_in_normal;clustered_events	ECNT=9;HCNT=1;MAX_ED=30;MIN_ED=2;NLOD=4.24;TLOD=11.59	GT:AD:AF:ALT_F1R2:ALT_F2R1:FOXOG:PGT:PID:QSS:REF_F1R2:REF_F2R1	0/1:62,4:0.066:4:0:0.00:0|1:138125496_C_T:1764,46:30:32	0/0:45,2:0.045:2:0:0.00:0|1:138125496_C_T:1262,37:24:21	1.4	Deleterious	-5.71	Deleterious	106	30	0	Damaging	2.82	93
T42df	chrX	**1**	**12.5**	163954412	163954412	T	C	exonic	**Zrsr2**	ENSMUSP00000118888	.	nonsynonymous SNV	Zrsr2:NM_009453:exon4:c.A286G:p.K96E,Zrsr2:NM_178794:exon4:c.A286G:p.K96E	**K96E**	ENSMUSP00000118888,96,K,E	X,163954412,T,A	XqF5	.	0.25	.	38	chrX	163954412	.	T	A	.	alt_allele_in_normal;clustered_events	ECNT=9;HCNT=1;MAX_ED=30;MIN_ED=2;NLOD=3.94;TLOD=11.56	GT:AD:AF:ALT_F1R2:ALT_F2R1:FOXOG:PGT:PID:QSS:REF_F1R2:REF_F2R1	0/1:62,4:0.065:4:0:1.00:0|1:138125496_C_T:1739,71:31:31	0/0:43,2:0.047:2:0:1.00:0|1:138125496_C_T:1186,35:23:20	1.4	Unknown	NA	NA	68	17	NA	NA	NA	NA

Supplementary Table S2. The Profiles of CNAs and CNA-affected Genes in the Mouse PtenI/Tp53I Mammary Tumors

PeakID (cmd=annotatedPeaks.pl GainRegions.bed mm10	Chr	Start	End	Size (end-Start)	Strand	Peak Score	Freq (%)	Focus Ratio/ Region Size	Annotation	Detailed Annotation	Distance to TSS	Nearest PromoterID	Entrez ID	Nearest Unigene	Nearest Refseq	Nearest Ensembl	Gene Name	Gene Alias	Gene Description	CNV Type	Gene Type
1318_T12,T1,T22,T2,T32,T3,T42,T4	chr17	9.3E+07	9.3E+07	6999	+	**8**	**100**	NA	Intergenic	L1_Mur3|LINE|L1	-269017	NM_001315503	11516	Mm.3407	NM_009625	ENSMUSG00000024256	**Adcyap1**	PACAP	adenylate cyclase activating polypeptide 1	Amp	protein-coding
1324_T12,T1,T22,T2,T32,T3,T42,T4	chr17	9.4E+07	9.4E+07	3999	+	**8**	**100**	NA	Intergenic	Intergenic	439332	NM_009625	11516	Mm.3407	NM_009625	ENSMUSG00000024256	**Adcyap1**	PACAP	adenylate cyclase activating polypeptide 1	Amp	protein-coding
3534_T12,T1,T22,T2,T32,T3,T42,T4	chr8	5.3E+07	5.3E+07	5999	+	**8**	**100**	NA	Intergenic	Lx8b|LINE|L1	-780202	NM_001205054	11593	Mm.334535	NM_001005847	ENSMUSG00000031521	**Aga**	AW060726	aspartylglucosaminidase	Amp	protein-coding
3538_T12,T1,T22,T2,T32,T3,T42,T4	chr8	5.3E+07	5.3E+07	8999	+	**8**	**100**	NA	Intergenic	Intergenic	-340702	NM_001205054	11593	Mm.334535	NM_001005847	ENSMUSG00000031521	**Aga**	AW060726	aspartylglucosaminidase	Amp	protein-coding
3570_T12,T1,T22,T2,T32,T3,T42,T4	chr8	6.3E+07	6.3E+07	4999	+	**8**	**100**	NA	Intergenic	Intergenic	-404807	NM_011922	26359	Mm.42179	NM_011922	ENSMUSG00000031635	**Anxa10**	-	annexin A10	Amp	protein-coding
3371_T12,T1,T22,T2,T32,T3,T42,T4	chr7	6.4E+07	6.4E+07	28999	+	**8**	**100**	NA	Intergenic	Intergenic	-20706	NM_007461	11784	Mm.4657	NM_007461	ENSMUSG00000030519	**Apba2**	X11-like|X11L|XllL|mXllL	amyloid beta (A4) precursor protein-binding, family A, member 2	Amp	protein-coding
2606_T12,T1,T22,T2,T32,T3,T42,T4	chr4	6.9E+07	6.9E+07	4999	+	**8**	**100**	NA	Intergenic	Intergenic	218397	NM_019967	56710	Mm.248788	NM_019967	ENSMUSG00000028351	**Brinp1**	BRINP|Dbc1|Dbccr1|Fam5a	bone morphogenic protein/retinoic acid inducible neural specific 1	Amp	protein-coding
2612_T12,T1,T22,T2,T32,T3,T42,T4	chr4	6.9E+07	6.9E+07	4999	+	**8**	**100**	NA	Intergenic	Intergenic	-426603	NM_019967	56710	Mm.248788	NM_019967	ENSMUSG00000028351	**Brinp1**	BRINP|Dbc1|Dbccr1|Fam5a	bone morphogenic protein/retinoic acid inducible neural specific 1	Amp	protein-coding
182_T12,T1,T22,T2,T32,T3,T42,T4	chr1	1.5E+08	1.5E+08	7999	+	**8**	**100**	NA	intron (NM_001145807, intron 4 of 7)	intron (NM_001145807, intron 4 of 7)	212813	NM_001145807	215378	Mm.441817	NM_153539	ENSMUSG00000035131	**Brinp3**	B830045N13Rik|Fam5c	bone morphogenetic protein/retinoic acid inducible neural specific 3	Amp	protein-coding
183_T12,T1,T22,T2,T32,T3,T42,T4	chr1	1.5E+08	1.5E+08	5999	+	**8**	**100**	NA	intron (NM_001145807, intron 7 of 7)	intron (NM_001145807, intron 7 of 7)	347813	NM_001145807	215378	Mm.441817	NM_153539	ENSMUSG00000035131	**Brinp3**	B830045N13Rik|Fam5c	bone morphogenetic protein/retinoic acid inducible neural specific 3	Amp	protein-coding
2936_T12,T1,T22,T2,T32,T3,T42,T4	chr6	1.8E+07	1.8E+07	999	+	**8**	**100**	NA	Intergenic	MTA_Mm|LTR|ERVL-MaLR	-12098	NM_007604	12343	Mm.392504	NM_007604	ENSMUSG00000015733	**Capza2**	1110053K06Rik|AW208808|Cappa2	capping protein (actin filament) muscle Z-line, alpha 2	Amp	protein-coding

2938_T12,T1,T22,T2,T32,T3,T42,T4	chr6	1.8E+07	1.8E+07	4999	+	**8**	**100**	NA	Intergenic	Intergenic	-8098	NM_007604	12343	Mm.392504	NM_007604	ENSMUSG00000015733	**Capza2**	1110053K06Rik|AW208808|Cappa2	capping protein (actin filament) muscle Z-line, alpha 2	Amp	protein-coding
2940_T12,T1,T22,T2,T32,T3,T42,T4	chr6	1.8E+07	1.8E+07	53999	+	**8**	**100**	NA	intron (NM_007604, intron 6 of 9)	intron (NM_007604, intron 6 of 9)	22402	NM_007604	12343	Mm.392504	NM_007604	ENSMUSG00000015733	**Capza2**	1110053K06Rik|AW208808|Cappa2	capping protein (actin filament) muscle Z-line, alpha 2	Amp	protein-coding
2933_T12,T1,T22,T2,T32,T3,T42,T4	chr6	1.7E+07	1.8E+07	546999	+	**8**	**100**	NA	Intergenic	B1_Mus1|SINE|Alu	41360	NM_001243064	12389	Mm.28278	NM_007616	ENSMUSG00000007655	**Cav1**	Cav|Cav-1	caveolin 1, caveolae protein	Amp	protein-coding
560_T12,T1,T22,T2,T32,T3,T42,T4	chr13	8.5E+07	8.5E+07	5999	+	**8**	**100**	NA	intron (NM_145452, intron 15 of 24)	intron (NM_145452, intron 15 of 24)	38023	NM_023243	66671	Mm.18474	NM_023243	ENSMUSG00000021548	**Ccnh**	6330408H09Rik|AI661354|AV102684|AW538719	cyclin H	Amp	protein-coding
411_T12,T1,T22,T2,T32,T3,T42,T4	chr11	1.1E+08	1.1E+08	5999	+	**8**	**100**	NA	Intergenic	ERVB4_1B-I_MM-int|LTR|ERVK	13386	NM_145437	217305	Mm.275986	NM_145437	ENSMUSG00000034641	**Cd300ld**	4732429D16Rik|CLM5|Cd300ld1|LMIR4|MAIR-IV|clm-5	CD300 molecule like family member d	Amp	protein-coding
928_T12,T1,T22,T2,T32,T3,T42,T4	chr15	1.9E+07	1.9E+07	2999	+	**8**	**100**	NA	Intergenic	Intergenic	363671	NM_009865	320873	Mm.117794	NM_009865	ENSMUSG00000022321	**Cdh10**	A830016G23Rik|C030003B10Rik|C030011H18Rik	cadherin 10	Amp	protein-coding
3649_T12,T1,T22,T2,T32,T3,T42,T4	chr8	1E+08	1E+08	4999	+	**8**	**100**	NA	Intergenic	Intergenic	1302111	NM_009866	12552	Mm.1571	NM_009866	ENSMUSG00000031673	**Cdh11**	Cad11|OSF-4	cadherin 11	Amp	protein-coding
3653_T12,T1,T22,T2,T32,T3,T42,T4	chr8	1E+08	1E+08	7999	+	**8**	**100**	NA	Intergenic	Intergenic	1262611	NM_009866	12552	Mm.1571	NM_009866	ENSMUSG00000031673	**Cdh11**	Cad11|OSF-4	cadherin 11	Amp	protein-coding
949_T12,T1,T22,T2,T32,T3,T42,T4	chr15	2.1E+07	2.1E+07	5999	+	**8**	**100**	NA	intron (NM_001008420, intron 2 of 11)	intron (NM_001008420, intron 2 of 11)	142048	NM_001008420	215654	Mm.300909	NM_001008420	ENSMUSG00000040452	**Cdh12**	Cdhb	cadherin 12	Amp	protein-coding
118_T12,T1,T22,T2,T32,T3,T42,T4	chr1	1.1E+08	1.1E+08	4999	+	**8**	**100**	NA	Intergenic	Intergenic	-682900	NM_001316743	241201	Mm.213407	NM_172853	ENSMUSG00000026312	**Cdh7**	9330156F07Rik|CDH7L1	cadherin 7, type 2	Amp	protein-coding
120_T12,T1,T22,T2,T32,T3,T42,T4	chr1	1.1E+08	1.1E+08	5999	+	**8**	**100**	NA	Intergenic	Intergenic	-465400	NM_001316743	241201	Mm.213407	NM_172853	ENSMUSG00000026312	**Cdh7**	9330156F07Rik|CDH7L1	cadherin 7, type 2	Amp	protein-coding
902_T12,T1,T22,T2,T32,T3,T42,T4	chr15	1.6E+07	1.6E+07	3999	+	**8**	**100**	NA	Intergenic	Lx9|LINE|L1	-799601	NM_009869	12565	Mm.439758	NM_009869	ENSMUSG00000025370	**Cdh9**	-	cadherin 9	Amp	protein-coding
1263_T12,T1,T22,T2,T32,T3,T42,T4	chr17	2.9E+07	2.9E+07	5999	+	**8**	**100**	NA	Intergenic	Intergenic	11731	NM_007669	12575	Mm.195663	NM_007669	ENSMUSG00000023067	**Cdkn1a**	CAP20|CDKI|CIP1|Cdkn1|P21|SDI1|Waf1|mda6|p21Cip1|p21WAF	cyclin-dependent kinase inhibitor 1A (P21)	Amp	protein-coding
2943_T12,T1,T22,T2,T32,T3,T42,T4	chr6	1.8E+07	1.8E+07	18999	+	**8**	**100**	NA	Intergenic	L1MC3|LINE|L1	-19687	NM_021050	12638	Mm.15621	NM_021050	ENSMUSG00000041301	**Cftr**	AW495489|Abcc7	cystic fibrosis transmembrane conductance regulator	Amp	protein-coding

<table>
<tr><td>2945_T12,T1,T22,T2,T32,T3,T42,T4</td><td>chr6</td><td>1.8E+07</td><td>1.8E+07</td><td>184999</td><td>+</td><td>8</td><td>100</td><td>NA</td><td>intron (NM_021050, intron 13 of 26)</td><td>Lx3B|LINE|L1</td><td>91313</td><td>NM_021050</td><td>12638</td><td>Mm.15621</td><td>NM_021050</td><td>ENSMUSG00000041301</td><td>Cftr</td><td>AW495489|Abcc7</td><td>cystic fibrosis transmembrane conductance regulator</td><td>Amp</td><td>protein-coding</td></tr>
<tr><td>3725_T12,T1,T22,T2,T32,T3,T42,T4</td><td>chr9</td><td>1.1E+07</td><td>1.1E+07</td><td>4999</td><td>+</td><td>8</td><td>100</td><td>NA</td><td>Intergenic</td><td>Intergenic</td><td>-458225</td><td>NM_001170787</td><td>244682</td><td>Mm.187021</td><td>NM_001033359</td><td>ENSMUSG00000039488</td><td>Cntn5</td><td>6720426O10Rik|A830025P08Rik|Gm507|NB-2</td><td>contactin 5</td><td>Amp</td><td>protein-coding</td></tr>
<tr><td>3051_T12,T1,T22,T2,T32,T3,T42,T4</td><td>chr6</td><td>4.5E+07</td><td>4.5E+07</td><td>6999</td><td>+</td><td>8</td><td>100</td><td>NA</td><td>intron (NM_001004357, intron 1 of 23)</td><td>intron (NM_001004357, intron 1 of 23)</td><td>56939</td><td>NM_001004357</td><td>66797</td><td>Mm.440084</td><td>NM_025771</td><td>ENSMUSG00000039419</td><td>Cntnap2</td><td>5430425M22Rik|Caspr2|mKIAA0868</td><td>contactin associated protein-like 2</td><td>Amp</td><td>protein-coding</td></tr>
<tr><td>2214_T12,T1,T22,T2,T32,T3,T42,T4</td><td>chr3</td><td>5.3E+07</td><td>5.3E+07</td><td>180999</td><td>+</td><td>8</td><td>100</td><td>NA</td><td>promoter-TSS (NM_026225)</td><td>promoter-TSS (NM_026225)</td><td>-777</td><td>NM_026225</td><td>67542</td><td>Mm.249548</td><td>NM_026225</td><td>ENSMUSG00000027742</td><td>Cog6</td><td>4933405E16Rik|AU018618|mKIAA1134</td><td>component of oligomeric golgi complex 6</td><td>Amp</td><td>protein-coding</td></tr>
<tr><td>5_T12,T1,T22,T2,T32,T3,T42,T4</td><td>chr1</td><td>2.5E+07</td><td>2.5E+07</td><td>4999</td><td>+</td><td>8</td><td>100</td><td>NA</td><td>Intergenic</td><td>Intergenic</td><td>-26563</td><td>NM_007733</td><td>12823</td><td>Mm.329196</td><td>NM_007733</td><td>ENSMUSG00000026141</td><td>Col19a1</td><td>-</td><td>collagen, type XIX, alpha 1</td><td>Amp</td><td>protein-coding</td></tr>
<tr><td>24_T12,T1,T22,T2,T32,T3,T42,T4</td><td>chr1</td><td>4.5E+07</td><td>4.5E+07</td><td>4999</td><td>+</td><td>8</td><td>100</td><td>NA</td><td>Intergenic</td><td>Intergenic</td><td>-130538</td><td>NM_009930</td><td>12825</td><td>Mm.249555</td><td>NM_009930</td><td>ENSMUSG00000026043</td><td>Col3a1</td><td>AW550625|Col3a-1|Tsk-2|Tsk2</td><td>collagen, type III, alpha 1</td><td>Amp</td><td>protein-coding</td></tr>
<tr><td>572_T12,T1,T22,T2,T32,T3,T42,T4</td><td>chr13</td><td>8.7E+07</td><td>8.7E+07</td><td>4999</td><td>+</td><td>8</td><td>100</td><td>NA</td><td>Intergenic</td><td>RLTR15|LTR|ERVK</td><td>-1E+06</td><td>NM_007749</td><td>12867</td><td>Mm.378898</td><td>NM_007749</td><td>ENSMUSG00000017778</td><td>Cox7c</td><td>COXVIIc|Cox7c1</td><td>cytochrome c oxidase subunit VIIc</td><td>Amp</td><td>protein-coding</td></tr>
<tr><td>1030_T12,T1,T22,T2,T32,T3,T42,T4</td><td>chr15</td><td>4.9E+07</td><td>4.9E+07</td><td>5999</td><td>+</td><td>8</td><td>100</td><td>NA</td><td>Intergenic</td><td>L1_Mur1|LINE|L1</td><td>-350511</td><td>NM_001081391</td><td>239420</td><td>Mm.309363</td><td>NM_001081391</td><td>ENSMUSG00000022311</td><td>Csmd3</td><td>4930500N14Rik|mKIAA1894</td><td>CUB and Sushi multiple domains 3</td><td>Amp</td><td>protein-coding</td></tr>
<tr><td>1033_T12,T1,T22,T2,T32,T3,T42,T4</td><td>chr15</td><td>4.9E+07</td><td>4.9E+07</td><td>3999</td><td>+</td><td>8</td><td>100</td><td>NA</td><td>Intergenic</td><td>RCHARR1|DNA|hAT-Charlie</td><td>-412511</td><td>NM_001081391</td><td>239420</td><td>Mm.309363</td><td>NM_001081391</td><td>ENSMUSG00000022311</td><td>Csmd3</td><td>4930500N14Rik|mKIAA1894</td><td>CUB and Sushi multiple domains 3</td><td>Amp</td><td>protein-coding</td></tr>
<tr><td>1036_T12,T1,T22,T2,T32,T3,T42,T4</td><td>chr15</td><td>5E+07</td><td>5E+07</td><td>2999</td><td>+</td><td>8</td><td>100</td><td>NA</td><td>Intergenic</td><td>Intergenic</td><td>-751011</td><td>NM_001081391</td><td>239420</td><td>Mm.309363</td><td>NM_001081391</td><td>ENSMUSG00000022311</td><td>Csmd3</td><td>4930500N14Rik|mKIAA1894</td><td>CUB and Sushi multiple domains 3</td><td>Amp</td><td>protein-coding</td></tr>
<tr><td>1616_T12,T1,T22,T2,T32,T3,T42,T4</td><td>chr2</td><td>1.5E+08</td><td>1.5E+08</td><td>5999</td><td>+</td><td>8</td><td>100</td><td>NA</td><td>Intergenic</td><td>(TG)n|Simple_repeat|Simple_repeat</td><td>-122988</td><td>NM_009976</td><td>13010</td><td>Mm.4263</td><td>NM_009976</td><td>ENSMUSG00000027447</td><td>Cst3</td><td>CysC</td><td>cystatin C</td><td>Amp</td><td>protein-coding</td></tr>
<tr><td>1621_T12,T1,T22,T2,T32,T3,T42,T4</td><td>chr2</td><td>1.5E+08</td><td>1.5E+08</td><td>6999</td><td>+</td><td>8</td><td>100</td><td>NA</td><td>Intergenic</td><td>Intergenic</td><td>-213488</td><td>NM_009976</td><td>13010</td><td>Mm.4263</td><td>NM_009976</td><td>ENSMUSG00000027447</td><td>Cst3</td><td>CysC</td><td>cystatin C</td><td>Amp</td><td>protein-coding</td></tr>
<tr><td>2952_T12,T1,T22,T2,T32,T3,T42,T4</td><td>chr6</td><td>1.9E+07</td><td>1.9E+07</td><td>52999</td><td>+</td><td>8</td><td>100</td><td>NA</td><td>Intergenic</td><td>Intergenic</td><td>-37175</td><td>NM_080285</td><td>30785</td><td>Mm.224189</td><td>NM_080285</td><td>ENSMUSG00000000416</td><td>Cttnbp2</td><td>3010022N24Rik|4732477G22Rik|6430526E05|9130022E09Rik|AU040881|Cortbp2|ORF4|mKIAA1758</td><td>cortactin binding protein 2</td><td>Amp</td><td>protein-coding</td></tr>
</table>

2957_T12,T1,T22,T2,T32,T3,T42,T4	chr6	1.9E+07	1.9E+07	21999	+	**8**	**100**	NA	Intergenic	RSINE1|SINE|B4	-84675	NM_080285	30785	Mm.224189	NM_080285	ENSMUSG00000000416	**Cttnbp2**	3010022N24Rik|4732477G22Rik|6430526E05|9130022E09Rik|AU040881|Cortbp2|ORF4|mKIAA1758	cortactin binding protein 2	Amp	protein-coding
2959_T12,T1,T22,T2,T32,T3,T42,T4	chr6	1.9E+07	1.9E+07	17999	+	**8**	**100**	NA	Intergenic	Intergenic	-105675	NM_080285	30785	Mm.224189	NM_080285	ENSMUSG00000000416	**Cttnbp2**	3010022N24Rik|4732477G22Rik|6430526E05|9130022E09Rik|AU040881|Cortbp2|ORF4|mKIAA1758	cortactin binding protein 2	Amp	protein-coding
2961_T12,T1,T22,T2,T32,T3,T42,T4	chr6	1.9E+07	1.9E+07	3999	+	**8**	**100**	NA	Intergenic	Intergenic	-117675	NM_080285	30785	Mm.224189	NM_080285	ENSMUSG00000000416	**Cttnbp2**	3010022N24Rik|4732477G22Rik|6430526E05|9130022E09Rik|AU040881|Cortbp2|ORF4|mKIAA1758	cortactin binding protein 2	Amp	protein-coding
2948_T12,T1,T22,T2,T32,T3,T42,T4	chr6	1.9E+07	1.9E+07	20999	+	**8**	**100**	NA	intron (NM_080285, intron 1 of 22)	intron (NM_080285, intron 1 of 22)	2825	NM_080285	30785	Mm.224189	NM_080285	ENSMUSG00000000416	**Cttnbp2**	3010022N24Rik|4732477G22Rik|6430526E05|9130022E09Rik|AU040881|Cortbp2|ORF4|mKIAA1758	cortactin binding protein 2	Amp	protein-coding
781_T12,T1,T22,T2,T32,T3,T42,T4	chr14	9.8E+07	9.8E+07	8999	+	**8**	**100**	NA	Intergenic	Intergenic	635765	NM_001038610	13134	Mm.320593	NM_007826	ENSMUSG00000055639	**Dach1**	Dac|Dach	dachshund 1 (Drosophila)	Amp	protein-coding
2234_T12,T1,T22,T2,T32,T3,T42,T4	chr3	5.5E+07	5.6E+07	701999	+	**8**	**100**	NA	Intergenic	Intergenic	-16026	NM_019978	13175	Mm.393242	NM_019978	ENSMUSG00000027797	**Dclk1**	1700113D08Rik|2810480F11Rik|AI836758|Click-I|Cpg16|Dcamkl1|Dcl|Dclk|mKIAA0369	doublecortin-like kinase 1	Amp	protein-coding
2236_T12,T1,T22,T2,T32,T3,T42,T4	chr3	5.6E+07	5.6E+07	14999	+	**8**	**100**	NA	Intergenic	Intergenic	132242	NM_001195540	13175	Mm.393242	NM_019978	ENSMUSG00000027797	**Dclk1**	1700113D08Rik|2810480F11Rik|AI836758|Click-I|Cpg16|Dcamkl1|Dcl|Dclk|mKIAA0369	doublecortin-like kinase 1	Amp	protein-coding
369_T12,T1,T22,T2,T32,T3,T42,T4	chr11	3.5E+07	3.5E+07	139999	+	**8**	**100**	NA	intron (NM_033374, intron 22 of 51)	intron (NM_033374, intron 22 of 51)	170405	NM_033374	94176	Mm.380679	NM_033374	ENSMUSG00000020143	**Dock2**	AI662014|AW122239|CED-5|Hch|MBC	dedicator of cyto-kinesis 2	Amp	protein-coding
131_T12,T1,T22,T2,T32,T3,T42,T4	chr1	1.1E+08	1.1E+08	5999	+	**8**	**100**	NA	Intergenic	Intergenic	-1E+06	NM_001081316	319901	Mm.103468	NM_001081316	ENSMUSG00000038702	**Dsel**	9330132E09Rik|DS-epi2	dermatan sulfate epimerase-like	Amp	protein-coding

<table>
<tr><td>135_T12,T1,T22,T2,T32,T3,T42,T4</td><td>chr1</td><td>1.1E+08</td><td>1.1E+08</td><td>6999</td><td>+</td><td>8</td><td>100</td><td>NA</td><td>Intergenic</td><td>B4A|SINE|B4</td><td>-1E+06</td><td>NM_001081316</td><td>319901</td><td>Mm.103468</td><td>NM_001081316</td><td>ENSMUSG00000038702</td><td>Dsel</td><td>9330132E09Rik|DS-epi2</td><td>dermatan sulfate epimerase-like</td><td>Amp</td><td>protein-coding</td></tr>
<tr><td>138_T12,T1,T22,T2,T32,T3,T42,T4</td><td>chr1</td><td>1.1E+08</td><td>1.1E+08</td><td>5999</td><td>+</td><td>8</td><td>100</td><td>NA</td><td>Intergenic</td><td>B4A|SINE|B4</td><td>-1E+06</td><td>NM_001081316</td><td>319901</td><td>Mm.103468</td><td>NM_001081316</td><td>ENSMUSG00000038702</td><td>Dsel</td><td>9330132E09Rik|DS-epi2</td><td>dermatan sulfate epimerase-like</td><td>Amp</td><td>protein-coding</td></tr>
<tr><td>1162_T12,T1,T22,T2,T32,T3,T42,T4</td><td>chr16</td><td>4.9E+07</td><td>4.9E+07</td><td>4999</td><td>+</td><td>8</td><td>100</td><td>NA</td><td>intron (NM_027341, intron 19 of 30)</td><td>intron (NM_027341, intron 19 of 30)</td><td>51112</td><td>NM_001110017</td><td>224170</td><td>Mm.275138</td><td>NM_027341</td><td>ENSMUSG00000064061</td><td>Dzip3</td><td>2310047C04Rik|2A-HUB|6430549P11Rik|A230104G20</td><td>DAZ interacting protein 3, zinc finger</td><td>Amp</td><td>protein-coding</td></tr>
<tr><td>1289_T12,T1,T22,T2,T32,T3,T42,T4</td><td>chr17</td><td>6.1E+07</td><td>6.1E+07</td><td>3999</td><td>+</td><td>8</td><td>100</td><td>NA</td><td>Intergenic</td><td>Intergenic</td><td>1664817</td><td>NM_207654</td><td>13640</td><td>Mm.7978</td><td>NM_010109</td><td>ENSMUSG00000048915</td><td>Efna5</td><td>AL-1|AV158822|EFL-5|Ephrin-A5|Epl7|LERK-7|RAGS</td><td>ephrin A5</td><td>Amp</td><td>protein-coding</td></tr>
<tr><td>1294_T12,T1,T22,T2,T32,T3,T42,T4</td><td>chr17</td><td>6.2E+07</td><td>6.2E+07</td><td>4999</td><td>+</td><td>8</td><td>100</td><td>NA</td><td>Intergenic</td><td>Intergenic</td><td>1032317</td><td>NM_207654</td><td>13640</td><td>Mm.7978</td><td>NM_010109</td><td>ENSMUSG00000048915</td><td>Efna5</td><td>AL-1|AV158822|EFL-5|Ephrin-A5|Epl7|LERK-7|RAGS</td><td>ephrin A5</td><td>Amp</td><td>protein-coding</td></tr>
<tr><td>1111_T12,T1,T22,T2,T32,T3,T42,T4</td><td>chr15</td><td>6.5E+07</td><td>6.5E+07</td><td>3999</td><td>+</td><td>8</td><td>100</td><td>NA</td><td>Intergenic</td><td>Intergenic</td><td>-368541</td><td>NM_133766</td><td>76740</td><td>Mm.260647</td><td>NM_133766</td><td>ENSMUSG00000015002</td><td>Efr3a</td><td>A130089M23Rik|BB071175|C76891|C920006C10Rik|D030063F01Rik|mKIAA0143</td><td>EFR3 homolog A</td><td>Amp</td><td>protein-coding</td></tr>
<tr><td>2178_T12,T1,T22,T2,T32,T3,T42,T4</td><td>chr3</td><td>5.1E+07</td><td>5.1E+07</td><td>145999</td><td>+</td><td>8</td><td>100</td><td>NA</td><td>intron (NM_001291059, intron 3 of 9)</td><td>L1Md_F3|LINE|L1</td><td>22144</td><td>NM_001291059</td><td>69257</td><td>Mm.131038</td><td>NM_023502</td><td>ENSMUSG00000037174</td><td>Elf2</td><td>2610036A20Rik|A230104O07Rik|AW111824|BB183398|EU32|NERF|NERF-1A|NERF-1B|NERF-2</td><td>E74-like factor 2</td><td>Amp</td><td>protein-coding</td></tr>
<tr><td>2870_T12,T1,T22,T2,T32,T3,T42,T4</td><td>chr5</td><td>8.5E+07</td><td>8.5E+07</td><td>4999</td><td>+</td><td>8</td><td>100</td><td>NA</td><td>Intergenic</td><td>Lx3_Mus|LINE|L1</td><td>-180618</td><td>NM_007937</td><td>13839</td><td>Mm.137991</td><td>NM_007937</td><td>ENSMUSG00000029245</td><td>Epha5</td><td>AI854630|AW125296|Cek7|Ehk1|Els1|Hek7|Rek7|bsk</td><td>Eph receptor A5</td><td>Amp</td><td>protein-coding</td></tr>
<tr><td>45_T12,T1,T22,T2,T32,T3,T42,T4</td><td>chr1</td><td>6.9E+07</td><td>6.9E+07</td><td>3999</td><td>+</td><td>8</td><td>100</td><td>NA</td><td>intron (NM_010154, intron 2 of 26)</td><td>intron (NM_010154, intron 2 of 26)</td><td>425559</td><td>NM_010154</td><td>13869</td><td>Mm.442420</td><td>NM_010154</td><td>ENSMUSG00000062209</td><td>Erbb4</td><td>Her4|c-erbB-4</td><td>erb-b2 receptor tyrosine kinase 4</td><td>Amp</td><td>protein-coding</td></tr>
<tr><td>866_T12,T1,T22,T2,T32,T3,T42,T4</td><td>chr14</td><td>1.2E+08</td><td>1.2E+08</td><td>4999</td><td>+</td><td>8</td><td>100</td><td>NA</td><td>intron (NM_207667, intron 1 of 4)</td><td>intron (NM_207667, intron 1 of 4)</td><td>-109454</td><td>NM_010201</td><td>14169</td><td>Mm.206362</td><td>NM_010201</td><td>ENSMUSG00000025551</td><td>Fgf14</td><td>FHF-4|Fhf4|mFHF-4(1B)</td><td>fibroblast growth factor 14</td><td>Amp</td><td>protein-coding</td></tr>
<tr><td>2196_T12,T1,T22,T2,T32,T3,T42,T4</td><td>chr3</td><td>5.2E+07</td><td>5.2E+07</td><td>14999</td><td>+</td><td>8</td><td>100</td><td>NA</td><td>Intergenic</td><td>MMERGLN-int|LTR|ERV1</td><td>-27337</td><td>NM_019739</td><td>56458</td><td>Mm.29891</td><td>NM_019739</td><td>ENSMUSG00000044167</td><td>Foxo1</td><td>AI876417|Afxh|FKHR|Fkhr1|Foxo1a</td><td>forkhead box O1</td><td>Amp</td><td>protein-coding</td></tr>
</table>

2203_T12,T1,T22,T2,T32,T3,T42,T4	chr3	5.2E+07	5.2E+07	96999	+	**8**	**100**	NA	Intergenic	Intergenic	110663	NM_019739	56458	Mm.29891	NM_019739	ENSMUSG00000044167	**Foxo1**	AI876417|Afxh|FKHR|Fkhr1|Foxo1a	forkhead box O1	Amp	protein-coding
2199_T12,T1,T22,T2,T32,T3,T42,T4	chr3	5.2E+07	5.2E+07	33999	+	**8**	**100**	NA	intron (NM_019739, intron 1 of 2)	intron (NM_019739, intron 1 of 2)	2163	NM_019739	56458	Mm.29891	NM_019739	ENSMUSG00000044167	**Foxo1**	AI876417|Afxh|FKHR|Fkhr1|Foxo1a	forkhead box O1	Amp	protein-coding
2201_T12,T1,T22,T2,T32,T3,T42,T4	chr3	5.2E+07	5.2E+07	37999	+	**8**	**100**	NA	intron (NM_019739, intron 1 of 2)	intron (NM_019739, intron 1 of 2)	39163	NM_019739	56458	Mm.29891	NM_019739	ENSMUSG00000044167	**Foxo1**	AI876417|Afxh|FKHR|Fkhr1|Foxo1a	forkhead box O1	Amp	protein-coding
2400_T12,T1,T22,T2,T32,T3,T42,T4	chr3	7.7E+07	7.7E+07	3999	+	**8**	**100**	NA	intron (NM_178673, intron 13 of 15)	intron (NM_178673, intron 13 of 15)	572917	NM_001253719	213262	Mm.379337	NM_178673	ENSMUSG00000034098	**Fstl5**	9130207J01Rik	follistatin-like 5	Amp	protein-coding
2553_T12,T1,T22,T2,T32,T3,T42,T4	chr4	2.6E+07	2.6E+07	7999	+	**8**	**100**	NA	3' UTR (NM_010243, exon 3 of 3)	3' UTR (NM_010243, exon 3 of 3)	186503	NM_010243	14348	Mm.39101	NM_010243	ENSMUSG00000055373	**Fut9**	AI746471|AU067636|mFUT9|mFuc-TIX	fucosyltransferase 9	Amp	protein-coding
1490_T12,T1,T22,T2,T32,T3,T42,T4	chr2	5.5E+07	5.5E+07	3999	+	**8**	**100**	NA	intron (NM_173030, intron 4 of 12)	MIRb|SINE|MIR	318113	NM_173030	271786	Mm.330227	NM_173030	ENSMUSG00000060988	**Galnt13**	A230002A12|A230020F20|BB182356	UDP-N-acetyl-alpha-D-galactosamine:polypeptide N-acetylgalactosaminyltransferase 13	Amp	protein-coding
3559_T12,T1,T22,T2,T32,T3,T42,T4	chr8	5.9E+07	5.9E+07	5999	+	**8**	**100**	NA	intron (NM_175032, intron 1 of 11)	intron (NM_175032, intron 1 of 11)	75127	NM_175032	270049	Mm.236368	NM_175032	ENSMUSG00000096914	**Galntl6**	1700021K10Rik|4930431L04Rik|A830023L05	UDP-N-acetyl-alpha-D-galactosamine:polypeptide N-acetylgalactosaminyltransferase-like 6	Amp	protein-coding
3067_T12,T1,T22,T2,T32,T3,T42,T4	chr6	6.4E+07	6.4E+07	9999	+	**8**	**100**	NA	intron (NM_008167, intron 2 of 15)	intron (NM_008167, intron 2 of 15)	254643	NM_008167	14804	Mm.439651	NM_008167	ENSMUSG00000071424	**Grid2**	B230104L07Rik|GluD2|GluRdelta2|Lc|Lc<J>|MMS10-AC|Ms10ac|cpr|ho|nmf408|tpr	glutamate receptor, ionotropic, delta 2	Amp	protein-coding
3070_T12,T1,T22,T2,T32,T3,T42,T4	chr6	6.4E+07	6.4E+07	6999	+	**8**	**100**	NA	intron (NM_008167, intron 2 of 15)	Lx8|LINE|L1	349143	NM_008167	14804	Mm.439651	NM_008167	ENSMUSG00000071424	**Grid2**	B230104L07Rik|GluD2|GluRdelta2|Lc|Lc<J>|MMS10-AC|Ms10ac|cpr|ho|nmf408|tpr	glutamate receptor, ionotropic, delta 2	Amp	protein-coding
273_T12,T1,T22,T2,T32,T3,T42,T4	chr10	5E+07	5E+07	4999	+	**8**	**100**	NA	intron (NM_010349, intron 2 of 17)	intron (NM_010349, intron 2 of 17)	108754	NM_010349	14806	Mm.332838	NM_010349	ENSMUSG00000056073	**Grik2**	AW124492|GluK2|Glur-6|Glur6|Glurbeta2	glutamate receptor, ionotropic, kainate 2 (beta 2)	Amp	protein-coding
2698_T12,T1,T22,T2,T32,T3,T42,T4	chr5	1E+07	1E+07	5999	+	**8**	**100**	NA	Intergenic	Intergenic	-289148	NM_181850	108069	Mm.318966	NM_181850	ENSMUSG00000003974	**Grm3**	0710001G23Rik|Gprc1c|mGlu3|mGluR3	glutamate receptor, metabotropic 3	Amp	protein-coding

3679_T12,T1,T22,T2,T32,T3,T42,T4	chr9	3896501	3906500	9999	+	**8**	**100**	NA	3' UTR (NM_001033322, exon 8 of 8)	3' UTR (NM_001033322, exon 8 of 8)	369151	NM_001033322	234889	Mm.91240	NM_001033322	ENSMUSG00000041624	**Gucy1a2**	6330407I18Rik|A230060L24Rik	guanylate cyclase 1, soluble, alpha 2	Amp	protein-coding
1633_T12,T1,T22,T2,T32,T3,T42,T4	chr3	3000501	3016500	15999	+	**8**	**100**	NA	Intergenic	Intergenic	-499530	NM_013920	30942	Mm.330897	NM_013920	ENSMUSG00000017688	**Hnf4g**	NR2A2	hepatocyte nuclear factor 4, gamma	Amp	protein-coding
1634_T12,T1,T22,T2,T32,T3,T42,T4	chr3	3021501	3033500	11999	+	**8**	**100**	NA	Intergenic	(CACAG)n|Simple_repeat|Simple_repeat	-480530	NM_013920	30942	Mm.330897	NM_013920	ENSMUSG00000017688	**Hnf4g**	NR2A2	hepatocyte nuclear factor 4, gamma	Amp	protein-coding
1642_T12,T1,T22,T2,T32,T3,T42,T4	chr3	3983501	3990500	6999	+	**8**	**100**	NA	Intergenic	Intergenic	478970	NM_013920	30942	Mm.330897	NM_013920	ENSMUSG00000017688	**Hnf4g**	NR2A2	hepatocyte nuclear factor 4, gamma	Amp	protein-coding
1636_T12,T1,T22,T2,T32,T3,T42,T4	chr3	3608501	3613500	4999	+	**8**	**100**	NA	intron (NM_013920, intron 1 of 9)	intron (NM_013920, intron 1 of 9)	102970	NM_013920	30942	Mm.330897	NM_013920	ENSMUSG00000017688	**Hnf4g**	NR2A2	hepatocyte nuclear factor 4, gamma	Amp	protein-coding
240_T12,T1,T22,T2,T32,T3,T42,T4	chr10	3.6E+07	3.6E+07	5999	+	**8**	**100**	NA	Intergenic	Intergenic	-719307	NM_001253355	319415	Mm.332442	NM_001081208	ENSMUSG00000044499	**Hs3st5**	D930005L05Rik|Gm1151|Hs3ost5	heparan sulfate (glucosamine) 3-O-sulfotransferase 5	Amp	protein-coding
242_T12,T1,T22,T2,T32,T3,T42,T4	chr10	3.6E+07	3.6E+07	7999	+	**8**	**100**	NA	Intergenic	RMER6C|LTR|ERVK	-705307	NM_001253355	319415	Mm.332442	NM_001081208	ENSMUSG00000044499	**Hs3st5**	D930005L05Rik|Gm1151|Hs3ost5	heparan sulfate (glucosamine) 3-O-sulfotransferase 5	Amp	protein-coding
2758_T12,T1,T22,T2,T32,T3,T42,T4	chr5	4.9E+07	4.9E+07	5999	+	**8**	**100**	NA	intron (NM_001199244, intron 1 of 7)	intron (NM_001199244, intron 1 of 7)	30057	NM_030265	80334	Mm.160172	NM_030265	ENSMUSG00000029088	**Kcnip4**	AV032399|Calp|Calp250|KChIP4|KChIP4a	Kv channel interacting protein 4	Amp	protein-coding
3393_T12,T1,T22,T2,T32,T3,T42,T4	chr7	1.4E+08	1.4E+08	4999	+	**8**	**100**	NA	non-coding (NR_001461, exon 1 of 1)	non-coding (NR_001461, exon 1 of 1)	50547	NR_001461	63830	Mm.356096	NR_001461	ENSMUSG00000101609	**Kcnq1ot1**	Kvlqt1-as|Lit1|Tssc8	KCNQ1 overlapping transcript 1	Amp	ncRNA
405_T12,T1,T22,T2,T32,T3,T42,T4	chr11	9.2E+07	9.2E+07	4999	+	**8**	**100**	NA	Intergenic	L1_Mur2|LINE|L1	-506445	NM_028547	73470	Mm.67677	NM_028547	ENSMUSG00000046755	**Kif2b**	1700063D03Rik	kinesin family member 2B	Amp	protein-coding
766_T12,T1,T22,T2,T32,T3,T42,T4	chr14	9.6E+07	9.6E+07	6999	+	**8**	**100**	NA	intron (NM_053105, intron 1 of 10)	intron (NM_053105, intron 1 of 10)	130034	NM_053105	93688	Mm.308735	NM_053105	ENSMUSG00000022076	**Klhl1**	mKIAA1490	kelch-like 1	Amp	protein-coding
1467_T12,T1,T22,T2,T32,T3,T42,T4	chr2	4.3E+07	4.3E+07	3999	+	**8**	**100**	NA	Intergenic	Intergenic	-257902	NM_053011	94217	Mm.441398	NM_053011	ENSMUSG00000049252	**Lrp1b**	9630004P12Rik|LRP-DIT	low density lipoprotein-related protein 1B (deleted in tumors)	Amp	protein-coding
1560_T12,T1,T22,T2,T32,T3,T42,T4	chr2	9.8E+07	9.8E+07	4999	+	**8**	**100**	NA	Intergenic	Intergenic	169343	NM_178725	241568	Mm.241682	NM_178725	ENSMUSG00000050587	**Lrrc4c**	6430556C10Rik|NGL-1	leucine rich repeat containing 4C	Amp	protein-coding

1565_T12,T1,T22,T2,T32,T3,T42,T4	chr2	9.9E+07	9.9E+07	4999	+	**8**	**100**	NA	Intergenic	SYNREP_MM|Satellite|Satellite	1197343	NM_178725	241568	Mm.241682	NM_178725	ENSMUSG00000050587	**Lrrc4c**	6430556C10Rik|NGL-1	leucine rich repeat containing 4C	Amp	protein-coding
1570_T12,T1,T22,T2,T32,T3,T42,T4	chr2	9.9E+07	9.9E+07	5999	+	**8**	**100**	NA	Intergenic	Intergenic	1672843	NM_178725	241568	Mm.241682	NM_178725	ENSMUSG00000050587	**Lrrc4c**	6430556C10Rik|NGL-1	leucine rich repeat containing 4C	Amp	protein-coding
1540_T12,T1,T22,T2,T32,T3,T42,T4	chr2	9.7E+07	9.7E+07	6999	+	**8**	**100**	NA	intron (NM_001289742, intron 1 of 7)	intron (NM_001289742, intron 1 of 7)	192831	NM_001289743	241568	Mm.241682	NM_178725	ENSMUSG00000050587	**Lrrc4c**	6430556C10Rik|NGL-1	leucine rich repeat containing 4C	Amp	protein-coding
1556_T12,T1,T22,T2,T32,T3,T42,T4	chr2	9.8E+07	9.8E+07	7999	+	**8**	**100**	NA	intron (NM_001289742, intron 7 of 7)	intron (NM_001289742, intron 7 of 7)	144843	NM_178725	241568	Mm.241682	NM_178725	ENSMUSG00000050587	**Lrrc4c**	6430556C10Rik|NGL-1	leucine rich repeat containing 4C	Amp	protein-coding
2963_T12,T1,T22,T2,T32,T3,T42,T4	chr6	1.9E+07	1.9E+07	121999	+	**8**	**100**	NA	Intergenic	Intergenic	-152135	NM_133939	76522	Mm.275158	NM_133939	ENSMUSG00000044155	**Lsm8**	2010003I05Rik|AW214405|Naa38	LSM8 homolog, U6 small nuclear RNA associated	Amp	protein-coding
2966_T12,T1,T22,T2,T32,T3,T42,T4	chr6	1.9E+07	1.9E+07	15999	+	**8**	**100**	NA	Intergenic	Intergenic	-77135	NM_133939	76522	Mm.275158	NM_133939	ENSMUSG00000044155	**Lsm8**	2010003I05Rik|AW214405|Naa38	LSM8 homolog, U6 small nuclear RNA associated	Amp	protein-coding
2969_T12,T1,T22,T2,T32,T3,T42,T4	chr6	1.9E+07	1.9E+07	2999	+	**8**	**100**	NA	Intergenic	L1Md_T|LINE|L1	-62635	NM_133939	76522	Mm.275158	NM_133939	ENSMUSG00000044155	**Lsm8**	2010003I05Rik|AW214405|Naa38	LSM8 homolog, U6 small nuclear RNA associated	Amp	protein-coding
2971_T12,T1,T22,T2,T32,T3,T42,T4	chr6	1.9E+07	1.9E+07	34999	+	**8**	**100**	NA	Intergenic	Intergenic	-42635	NM_133939	76522	Mm.275158	NM_133939	ENSMUSG00000044155	**Lsm8**	2010003I05Rik|AW214405|Naa38	LSM8 homolog, U6 small nuclear RNA associated	Amp	protein-coding
1262_T12,T1,T22,T2,T32,T3,T42,T4	chr17	2.6E+07	2.6E+07	3999	+	**8**	**100**	NA	Intergenic	Intergenic	67590	NR_037905	66978	Mm.440216	NM_025881	ENSMUSG00000024188	**Luc7l**	1810045C04Rik|2410018D03Rik	Luc7-like	Amp	protein-coding
2243_T12,T1,T22,T2,T32,T3,T42,T4	chr3	5.6E+07	5.6E+07	16999	+	**8**	**100**	NA	intron (NM_030595, intron 41 of 57)	intron (NM_030595, intron 41 of 57)	-4510	NM_010750	17116	Mm.440209	NM_010750	ENSMUSG00000056947	**Mab21l1**	AW047968	mab-21-like 1 (C. elegans)	Amp	protein-coding
2241_T12,T1,T22,T2,T32,T3,T42,T4	chr3	5.6E+07	5.6E+07	156999	+	**8**	**100**	NA	intron (NM_030595, intron 47 of 57)	Lx8|LINE|L1	-92510	NM_010750	17116	Mm.440209	NM_010750	ENSMUSG00000056947	**Mab21l1**	AW047968	mab-21-like 1 (C. elegans)	Amp	protein-coding
2728_T12,T1,T22,T2,T32,T3,T42,T4	chr5	2E+07	2E+07	3999	+	**8**	**100**	NA	intron (NM_001170746, intron 1 of 22)	MLT1B|LTR|ERVL-MaLR	333454	NM_001170746	50791	Mm.332231	NM_015823	ENSMUSG00000040003	**Magi2**	AIP-1|Acvri1|Acvrinp1|Acvrip1|Magi-2|S-SCAM|mKIAA0705	membrane associated guanylate kinase, WW and PDZ domain containing 2	Amp	protein-coding
2194_T12,T1,T22,T2,T32,T3,T42,T4	chr3	5.2E+07	5.2E+07	290999	+	**8**	**100**	NA	intron (NM_001004176, intron 1 of 4)	intron (NM_001004176, intron 1 of 4)	21006	NM_001004176	433586	Mm.234972	NM_001004176	ENSMUSG00000061143	**Maml3**	AV234550|BC049812|Mam-2|mKIAA1816	mastermind like 3 (Drosophila)	Amp	protein-coding

2556_T12,T1,T22,T2,T32,T3,T42,T4	chr4	2.6E+07	2.6E+07	6999	+	**8**	**100**	NA	Intergenic	Intergenic	244652	NM_172865	242362	Mm.245602	NM_172865	ENSMUSG00000040520	**Manea**	4932703L02Rik	mannosidase, endo-alpha	Amp	protein-coding
2189_T12,T1,T22,T2,T32,T3,T42,T4	chr3	5.2E+07	5.2E+07	299999	+	**8**	**100**	NA	intron (NM_001004176, intron 2 of 4)	intron (NM_001004176, intron 2 of 4)	113321	NM_001310482	211666	Mm.24679	NM_174995	ENSMUSG00000074604	**Mgst2**	GST2|MGST-II	microsomal glutathione S-transferase 2	Amp	protein-coding
2533_T12,T1,T22,T2,T32,T3,T42,T4	chr4	1.8E+07	1.8E+07	6999	+	**8**	**100**	NA	Intergenic	Intergenic	-211482	NM_019724	17389	Mm.187315	NM_019724	ENSMUSG00000028226	**Mmp16**	MT-MMP 3|MT3-MMP|Mt3mmp	matrix metallopeptidase 16	Amp	protein-coding
3483_T12,T1,T22,T2,T32,T3,T42,T4	chr8	4E+07	4E+07	4999	+	**8**	**100**	NA	TTS (NM_001113326)	TTS (NM_001113326)	61678	NM_031195	20288	Mm.239291	NM_031195	ENSMUSG00000025044	**Msr1**	MRS-A|MSR|MSR-A|SR-AI|SR-AII|Scara1|Scvr	macrophage scavenger receptor 1	Amp	protein-coding
2180_T12,T1,T22,T2,T32,T3,T42,T4	chr3	5.1E+07	5.1E+07	19999	+	**8**	**100**	NA	Intergenic	Intergenic	-2516	NM_053089	74838	Mm.275281	NM_053089	ENSMUSG00000063273	**Naa15**	5730450D16Rik|6330400I15|ASTBDN|Narg1|Tbdn-1|mNAT1	N(alpha)-acetyltransferase 15, NatA auxiliary subunit	Amp	protein-coding
2183_T12,T1,T22,T2,T32,T3,T42,T4	chr3	5.1E+07	5.1E+07	13999	+	**8**	**100**	NA	intron (NM_053089, intron 6 of 19)	intron (NM_053089, intron 6 of 19)	28484	NM_053089	74838	Mm.275281	NM_053089	ENSMUSG00000063273	**Naa15**	5730450D16Rik|6330400I15|ASTBDN|Narg1|Tbdn-1|mNAT1	N(alpha)-acetyltransferase 15, NatA auxiliary subunit	Amp	protein-coding
2264_T12,T1,T22,T2,T32,T3,T42,T4	chr3	5.6E+07	5.6E+07	29999	+	**8**	**100**	NA	Intergenic	MT2B1|LTR|ERVL	-47799	NM_030595	26422	Mm.384353	NM_030595	ENSMUSG00000027799	**Nbea**	Lyst2|mKIAA1544	neurobeachin	Amp	protein-coding
2266_T12,T1,T22,T2,T32,T3,T42,T4	chr3	5.6E+07	5.6E+07	116999	+	**8**	**100**	NA	Intergenic	MER31B|LTR|ERV1	-128299	NM_030595	26422	Mm.384353	NM_030595	ENSMUSG00000027799	**Nbea**	Lyst2|mKIAA1544	neurobeachin	Amp	protein-coding
2271_T12,T1,T22,T2,T32,T3,T42,T4	chr3	5.6E+07	5.6E+07	999	+	**8**	**100**	NA	Intergenic	L1_Mus3|LINE|L1	-199299	NM_030595	26422	Mm.384353	NM_030595	ENSMUSG00000027799	**Nbea**	Lyst2|mKIAA1544	neurobeachin	Amp	protein-coding
2274_T12,T1,T22,T2,T32,T3,T42,T4	chr3	5.6E+07	5.6E+07	4999	+	**8**	**100**	NA	Intergenic	Lx5|LINE|L1	-204299	NM_030595	26422	Mm.384353	NM_030595	ENSMUSG00000027799	**Nbea**	Lyst2|mKIAA1544	neurobeachin	Amp	protein-coding
2276_T12,T1,T22,T2,T32,T3,T42,T4	chr3	5.6E+07	5.7E+07	120999	+	**8**	**100**	NA	Intergenic	Intergenic	-268299	NM_030595	26422	Mm.384353	NM_030595	ENSMUSG00000027799	**Nbea**	Lyst2|mKIAA1544	neurobeachin	Amp	protein-coding
2280_T12,T1,T22,T2,T32,T3,T42,T4	chr3	5.7E+07	5.7E+07	16999	+	**8**	**100**	NA	Intergenic	ORR1B2|LTR|ERVL-MaLR	-346299	NM_030595	26422	Mm.384353	NM_030595	ENSMUSG00000027799	**Nbea**	Lyst2|mKIAA1544	neurobeachin	Amp	protein-coding
2282_T12,T1,T22,T2,T32,T3,T42,T4	chr3	5.7E+07	5.7E+07	17999	+	**8**	**100**	NA	Intergenic	Intergenic	-370799	NM_030595	26422	Mm.384353	NM_030595	ENSMUSG00000027799	**Nbea**	Lyst2|mKIAA1544	neurobeachin	Amp	protein-coding
2284_T12,T1,T22,T2,T32,T3,T42,T4	chr3	5.7E+07	5.7E+07	3999	+	**8**	**100**	NA	Intergenic	Lx2|LINE|L1	-382799	NM_030595	26422	Mm.384353	NM_030595	ENSMUSG00000027799	**Nbea**	Lyst2|mKIAA1544	neurobeachin	Amp	protein-coding
2286_T12,T1,T22,T2,T32,T3,T42,T4	chr3	5.7E+07	5.7E+07	26999	+	**8**	**100**	NA	Intergenic	Intergenic	-399299	NM_030595	26422	Mm.384353	NM_030595	ENSMUSG00000027799	**Nbea**	Lyst2|mKIAA1544	neurobeachin	Amp	protein-coding

2258_T12,T1,T22,T2,T32,T3,T42,T4	chr3	5.6E+07	5.6E+07	12999	+	**8**	**100**	NA	intron (NM_030595, intron 1 of 57)	intron (NM_030595, intron 1 of 57)	54701	NM_030595	26422	Mm.384353	NM_030595	ENSMUSG00000027799	**Nbea**	Lyst2|mKIAA1544	neurobeachin	Amp	protein-coding
2260_T12,T1,T22,T2,T32,T3,T42,T4	chr3	5.6E+07	5.6E+07	64999	+	**8**	**100**	NA	intron (NM_030595, intron 1 of 57)	intron (NM_030595, intron 1 of 57)	7701	NM_030595	26422	Mm.384353	NM_030595	ENSMUSG00000027799	**Nbea**	Lyst2|mKIAA1544	neurobeachin	Amp	protein-coding
2256_T12,T1,T22,T2,T32,T3,T42,T4	chr3	5.6E+07	5.6E+07	91999	+	**8**	**100**	NA	intron (NM_030595, intron 8 of 57)	L1Md_T|LINE|L1	111201	NM_030595	26422	Mm.384353	NM_030595	ENSMUSG00000027799	**Nbea**	Lyst2|mKIAA1544	neurobeachin	Amp	protein-coding
2224_T12,T1,T22,T2,T32,T3,T42,T4	chr3	5.3E+07	5.3E+07	80999	+	**8**	**100**	NA	Intergenic	Intergenic	139258	NM_172501	212114	Mm.85744	NM_172501	ENSMUSG00000042997	**Nhlrc3**	4833441N19|8030451K01Rik|mKIAA4083	NHL repeat containing 3	Amp	protein-coding
1723_T12,T1,T22,T2,T32,T3,T42,T4	chr3	2.4E+07	2.4E+07	6999	+	**8**	**100**	NA	Intergenic	Intergenic	1870307	NM_001163387	192167	Mm.316080	NM_138666	ENSMUSG00000063887	**Nlgn1**	6330415N05Rik|BB179718|NL1|Nlg1|mKIAA1070	neuroligin 1	Amp	protein-coding
2172_T12,T1,T22,T2,T32,T3,T42,T4	chr3	5.1E+07	5.1E+07	124999	+	**8**	**100**	NA	Intergenic	Intergenic	-191447	NM_009834	12457	Mm.86541	NM_009834	ENSMUSG00000023087	**Noct**	AU043840|Ccr4|Ccrn4l	nocturnin	Amp	protein-coding
2174_T12,T1,T22,T2,T32,T3,T42,T4	chr3	5.1E+07	5.1E+07	62999	+	**8**	**100**	NA	Intergenic	Intergenic	-87447	NM_009834	12457	Mm.86541	NM_009834	ENSMUSG00000023087	**Noct**	AU043840|Ccr4|Ccrn4l	nocturnin	Amp	protein-coding
2176_T12,T1,T22,T2,T32,T3,T42,T4	chr3	5.1E+07	5.1E+07	69999	+	**8**	**100**	NA	Intergenic	B1_Mur4|SINE|Alu	-19947	NM_009834	12457	Mm.86541	NM_009834	ENSMUSG00000023087	**Noct**	AU043840|Ccr4|Ccrn4l	nocturnin	Amp	protein-coding
532_T12,T1,T22,T2,T32,T3,T42,T4	chr13	7.9E+07	7.9E+07	4999	+	**8**	**100**	NA	Intergenic	Intergenic	-875018	NM_010151	13865	Mm.439653	NM_010151	ENSMUSG00000069171	**Nr2f1**	COUP-TF1|COUP-TFI|COUPTFA|EAR-3|EAR3|Erbal3|SVP44|Tcfcoup1	nuclear receptor subfamily 2, group F, member 1	Amp	protein-coding
534_T12,T1,T22,T2,T32,T3,T42,T4	chr13	7.9E+07	7.9E+07	5999	+	**8**	**100**	NA	Intergenic	Intergenic	-923518	NM_010151	13865	Mm.439653	NM_010151	ENSMUSG00000069171	**Nr2f1**	COUP-TF1|COUP-TFI|COUPTFA|EAR-3|EAR3|Erbal3|SVP44|Tcfcoup1	nuclear receptor subfamily 2, group F, member 1	Amp	protein-coding
540_T12,T1,T22,T2,T32,T3,T42,T4	chr13	7.9E+07	7.9E+07	3999	+	**8**	**100**	NA	Intergenic	Intergenic	-973518	NM_010151	13865	Mm.439653	NM_010151	ENSMUSG00000069171	**Nr2f1**	COUP-TF1|COUP-TFI|COUPTFA|EAR-3|EAR3|Erbal3|SVP44|Tcfcoup1	nuclear receptor subfamily 2, group F, member 1	Amp	protein-coding
458_T12,T1,T22,T2,T32,T3,T42,T4	chr12	8.9E+07	8.9E+07	5999	+	**8**	**100**	NA	intron (NM_172544, intron 8 of 19)	intron (NM_172544, intron 8 of 19)	-408983	NM_001252074	18191	Mm.425766	NM_172544	ENSMUSG00000066392	**Nrxn3**	-	neurexin III	Amp	protein-coding

1277_T12,T1,T22,T2,T32,T3,T42,T4	chr17	5.9E+07	5.9E+07	3999	+	**8**	**100**	NA	Intergenic	Intergenic	-391178	NM_026497	67993	Mm.36507	NM_026497	ENSMUSG00000024228	**Nudt12**	0610016O18Rik	nudix (nucleoside diphosphate linked moiety X)-type motif 12	Amp	protein-coding
1284_T12,T1,T22,T2,T32,T3,T42,T4	chr17	6E+07	6E+07	6999	+	**8**	**100**	NA	Intergenic	Intergenic	-1E+06	NM_026497	67993	Mm.36507	NM_026497	ENSMUSG00000024228	**Nudt12**	0610016O18Rik	nudix (nucleoside diphosphate linked moiety X)-type motif 12	Amp	protein-coding
2884_T12,T1,T22,T2,T32,T3,T42,T4	chr6	9373501	9377500	3999	+	**8**	**100**	NA	Intergenic	Intergenic	425481	NM_008751	18231	Mm.46954	NM_008751	ENSMUSG00000046178	**Nxph1**	C130005L03Rik	neurexophilin 1	Amp	protein-coding
2885_T12,T1,T22,T2,T32,T3,T42,T4	chr6	9413501	9419500	5999	+	**8**	**100**	NA	Intergenic	(TTTG)n|Simple_repeat|Simple_repeat	466481	NM_008751	18231	Mm.46954	NM_008751	ENSMUSG00000046178	**Nxph1**	C130005L03Rik	neurexophilin 1	Amp	protein-coding
2473_T12,T1,T22,T2,T32,T3,T42,T4	chr3	1.2E+08	1.2E+08	3999	+	**8**	**100**	NA	Intergenic	Intergenic	57535	NM_153157	229759	Mm.54183	NM_153157	ENSMUSG00000027965	**Olfm3**	B230206G02Rik	olfactomedin 3	Amp	protein-coding
627_T12,T1,T22,T2,T32,T3,T42,T4	chr14	8.1E+07	8.1E+07	3999	+	**8**	**100**	NA	Intergenic	Intergenic	526198	NM_001030294	380924	Mm.26456	NM_001030294	ENSMUSG00000022026	**Olfm4**	GC1|GW112|Gm296|Gm913|OlfD|pPD4	olfactomedin 4	Amp	protein-coding
1904_T12,T1,T22,T2,T32,T3,T42,T4	chr3	4.6E+07	4.6E+07	90999	+	**8**	**100**	NA	Intergenic	L1_Mus3|LINE|L1	497941	NM_001101479	241989	Mm.119234	NM_001101479	ENSMUSG00000090919	**Pabpc4l**	C330050A14Rik|EG241989	poly(A) binding protein, cytoplasmic 4-like	Amp	protein-coding
1906_T12,T1,T22,T2,T32,T3,T42,T4	chr3	4.6E+07	4.6E+07	2999	+	**8**	**100**	NA	Intergenic	RMER6C|LTR|ERVK	449941	NM_001101479	241989	Mm.119234	NM_001101479	ENSMUSG00000090919	**Pabpc4l**	C330050A14Rik|EG241989	poly(A) binding protein, cytoplasmic 4-like	Amp	protein-coding
1910_T12,T1,T22,T2,T32,T3,T42,T4	chr3	4.6E+07	4.6E+07	3999	+	**8**	**100**	NA	Intergenic	Intergenic	443441	NM_001101479	241989	Mm.119234	NM_001101479	ENSMUSG00000090919	**Pabpc4l**	C330050A14Rik|EG241989	poly(A) binding protein, cytoplasmic 4-like	Amp	protein-coding
1913_T12,T1,T22,T2,T32,T3,T42,T4	chr3	4.6E+07	4.6E+07	26999	+	**8**	**100**	NA	Intergenic	Intergenic	425941	NM_001101479	241989	Mm.119234	NM_001101479	ENSMUSG00000090919	**Pabpc4l**	C330050A14Rik|EG241989	poly(A) binding protein, cytoplasmic 4-like	Amp	protein-coding
1916_T12,T1,T22,T2,T32,T3,T42,T4	chr3	4.6E+07	4.6E+07	3999	+	**8**	**100**	NA	Intergenic	AT_rich|Low_complexity|Low_complexity	401441	NM_001101479	241989	Mm.119234	NM_001101479	ENSMUSG00000090919	**Pabpc4l**	C330050A14Rik|EG241989	poly(A) binding protein, cytoplasmic 4-like	Amp	protein-coding
1918_T12,T1,T22,T2,T32,T3,T42,T4	chr3	4.6E+07	4.6E+07	5999	+	**8**	**100**	NA	Intergenic	Intergenic	395441	NM_001101479	241989	Mm.119234	NM_001101479	ENSMUSG00000090919	**Pabpc4l**	C330050A14Rik|EG241989	poly(A) binding protein, cytoplasmic 4-like	Amp	protein-coding
1923_T12,T1,T22,T2,T32,T3,T42,T4	chr3	4.6E+07	4.6E+07	14999	+	**8**	**100**	NA	Intergenic	Intergenic	377941	NM_001101479	241989	Mm.119234	NM_001101479	ENSMUSG00000090919	**Pabpc4l**	C330050A14Rik|EG241989	poly(A) binding protein, cytoplasmic 4-like	Amp	protein-coding
1925_T12,T1,T22,T2,T32,T3,T42,T4	chr3	4.6E+07	4.6E+07	12999	+	**8**	**100**	NA	Intergenic	Lx2|LINE|L1	359941	NM_001101479	241989	Mm.119234	NM_001101479	ENSMUSG00000090919	**Pabpc4l**	C330050A14Rik|EG241989	poly(A) binding protein, cytoplasmic 4-like	Amp	protein-coding
1927_T12,T1,T22,T2,T32,T3,T42,T4	chr3	4.6E+07	4.6E+07	26999	+	**8**	**100**	NA	Intergenic	Intergenic	323941	NM_001101479	241989	Mm.119234	NM_001101479	ENSMUSG00000090919	**Pabpc4l**	C330050A14Rik|EG241989	poly(A) binding protein, cytoplasmic 4-like	Amp	protein-coding

1932_T12,T1,T22,T2,T32,T3,T42,T4	chr3	4.6E+07	4.6E+07	32999	+	**8**	**100**	NA	Intergenic	L1Md_F2|LINE|L1	281941	NM_001101479	241989	Mm.119234	NM_001101479	ENSMUSG00000090919	**Pabpc4l**	C330050A14Rik|EG241989	poly(A) binding protein, cytoplasmic 4-like	Amp	protein-coding
1936_T12,T1,T22,T2,T32,T3,T42,T4	chr3	4.6E+07	4.6E+07	4999	+	**8**	**100**	NA	Intergenic	MTE-int|LTR|ERVL-MaLR	259941	NM_001101479	241989	Mm.119234	NM_001101479	ENSMUSG00000090919	**Pabpc4l**	C330050A14Rik|EG241989	poly(A) binding protein, cytoplasmic 4-like	Amp	protein-coding
1941_T12,T1,T22,T2,T32,T3,T42,T4	chr3	4.6E+07	4.6E+07	46999	+	**8**	**100**	NA	Intergenic	Intergenic	214941	NM_001101479	241989	Mm.119234	NM_001101479	ENSMUSG00000090919	**Pabpc4l**	C330050A14Rik|EG241989	poly(A) binding protein, cytoplasmic 4-like	Amp	protein-coding
1943_T12,T1,T22,T2,T32,T3,T42,T4	chr3	4.6E+07	4.7E+07	319999	+	**8**	**100**	NA	Intergenic	MTC|LTR|ERVL-MaLR	18441	NM_001101479	241989	Mm.119234	NM_001101479	ENSMUSG00000090919	**Pabpc4l**	C330050A14Rik|EG241989	poly(A) binding protein, cytoplasmic 4-like	Amp	protein-coding
1946_T12,T1,T22,T2,T32,T3,T42,T4	chr3	4.7E+07	4.7E+07	4999	+	**8**	**100**	NA	Intergenic	Intergenic	-146059	NM_001101479	241989	Mm.119234	NM_001101479	ENSMUSG00000090919	**Pabpc4l**	C330050A14Rik|EG241989	poly(A) binding protein, cytoplasmic 4-like	Amp	protein-coding
1948_T12,T1,T22,T2,T32,T3,T42,T4	chr3	4.7E+07	4.7E+07	34999	+	**8**	**100**	NA	Intergenic	Intergenic	-167059	NM_001101479	241989	Mm.119234	NM_001101479	ENSMUSG00000090919	**Pabpc4l**	C330050A14Rik|EG241989	poly(A) binding protein, cytoplasmic 4-like	Amp	protein-coding
1956_T12,T1,T22,T2,T32,T3,T42,T4	chr3	4.7E+07	4.7E+07	3999	+	**8**	**100**	NA	Intergenic	Intergenic	-213559	NM_001101479	241989	Mm.119234	NM_001101479	ENSMUSG00000090919	**Pabpc4l**	C330050A14Rik|EG241989	poly(A) binding protein, cytoplasmic 4-like	Amp	protein-coding
1958_T12,T1,T22,T2,T32,T3,T42,T4	chr3	4.7E+07	4.7E+07	999	+	**8**	**100**	NA	Intergenic	Intergenic	-217059	NM_001101479	241989	Mm.119234	NM_001101479	ENSMUSG00000090919	**Pabpc4l**	C330050A14Rik|EG241989	poly(A) binding protein, cytoplasmic 4-like	Amp	protein-coding
1871_T12,T1,T22,T2,T32,T3,T42,T4	chr3	4.5E+07	4.5E+07	4999	+	**8**	**100**	NA	TTS (NM_001098172)	TTS (NM_001098172)	21602	NM_001098171	18526	Mm.242644	NM_011043	ENSMUSG00000049100	**Pcdh10**	6430521D13Rik|6430703F07Rik|OL-pc|mKIAA1400	protocadherin 10	Amp	protein-coding
1873_T12,T1,T22,T2,T32,T3,T42,T4	chr3	4.5E+07	4.5E+07	7999	+	**8**	**100**	NA	intron (NM_001098170, intron 4 of 4)	intron (NM_001098170, intron 4 of 4)	29102	NM_001098171	18526	Mm.242644	NM_011043	ENSMUSG00000049100	**Pcdh10**	6430521D13Rik|6430703F07Rik|OL-pc|mKIAA1400	protocadherin 10	Amp	protein-coding
641_T12,T1,T22,T2,T32,T3,T42,T4	chr14	8.2E+07	8.2E+07	4999	+	**8**	**100**	NA	Intergenic	Intergenic	-2E+06	NM_001013753	219228	Mm.153643	NM_001013753	ENSMUSG00000035566	**Pcdh17**	C030033F14Rik|Gm78	protocadherin 17	Amp	protein-coding
644_T12,T1,T22,T2,T32,T3,T42,T4	chr14	8.3E+07	8.3E+07	4999	+	**8**	**100**	NA	Intergenic	Intergenic	-1E+06	NM_001013753	219228	Mm.153643	NM_001013753	ENSMUSG00000035566	**Pcdh17**	C030033F14Rik|Gm78	protocadherin 17	Amp	protein-coding
663_T12,T1,T22,T2,T32,T3,T42,T4	chr14	8.4E+07	8.4E+07	6999	+	**8**	**100**	NA	intron (NM_001013753, intron 1 of 3)	intron (NM_001013753, intron 1 of 3)	11437	NM_001013753	219228	Mm.153643	NM_001013753	ENSMUSG00000035566	**Pcdh17**	C030033F14Rik|Gm78	protocadherin 17	Amp	protein-coding
2113_T12,T1,T22,T2,T32,T3,T42,T4	chr3	4.9E+07	4.9E+07	100999	+	**8**	**100**	NA	Intergenic	L1Md_F2|LINE|L1	541316	NM_130448	73173	Mm.87246	NM_130448	ENSMUSG00000037892	**Pcdh18**	3110038E07Rik|BB095589|PCDH68L	protocadherin 18	Amp	protein-coding
2115_T12,T1,T22,T2,T32,T3,T42,T4	chr3	4.9E+07	4.9E+07	17999	+	**8**	**100**	NA	Intergenic	Intergenic	474816	NM_130448	73173	Mm.87246	NM_130448	ENSMUSG00000037892	**Pcdh18**	3110038E07Rik|BB095589|PCDH68L	protocadherin 18	Amp	protein-coding

2118_T12,T1,T22,T2,T32,T3,T42,T4	chr3	4.9E+07	4.9E+07	145999	+	**8**	**100**	NA	Intergenic	L1Md_F2|LINE|L1	387816	NM_130448	73173	Mm.87246	NM_130448	ENSMUSG00000037892	**Pcdh18**	3110038E07Rik|BB095589|PCDH68L	protocadherin 18	Amp	protein-coding
2126_T12,T1,T22,T2,T32,T3,T42,T4	chr3	4.9E+07	5E+07	188999	+	**8**	**100**	NA	Intergenic	Intergenic	179316	NM_130448	73173	Mm.87246	NM_130448	ENSMUSG00000037892	**Pcdh18**	3110038E07Rik|BB095589|PCDH68L	protocadherin 18	Amp	protein-coding
2129_T12,T1,T22,T2,T32,T3,T42,T4	chr3	5E+07	5E+07	3999	+	**8**	**100**	NA	Intergenic	Intergenic	80816	NM_130448	73173	Mm.87246	NM_130448	ENSMUSG00000037892	**Pcdh18**	3110038E07Rik|BB095589|PCDH68L	protocadherin 18	Amp	protein-coding
2132_T12,T1,T22,T2,T32,T3,T42,T4	chr3	5E+07	5E+07	45999	+	**8**	**100**	NA	Intergenic	MLT1D|LTR|ERVL-MaLR	53816	NM_130448	73173	Mm.87246	NM_130448	ENSMUSG00000037892	**Pcdh18**	3110038E07Rik|BB095589|PCDH68L	protocadherin 18	Amp	protein-coding
2134_T12,T1,T22,T2,T32,T3,T42,T4	chr3	5E+07	5E+07	61999	+	**8**	**100**	NA	Intergenic	B4A|SINE|B4	-8184	NM_130448	73173	Mm.87246	NM_130448	ENSMUSG00000037892	**Pcdh18**	3110038E07Rik|BB095589|PCDH68L	protocadherin 18	Amp	protein-coding
2137_T12,T1,T22,T2,T32,T3,T42,T4	chr3	5E+07	5E+07	11999	+	**8**	**100**	NA	Intergenic	Intergenic	-53184	NM_130448	73173	Mm.87246	NM_130448	ENSMUSG00000037892	**Pcdh18**	3110038E07Rik|BB095589|PCDH68L	protocadherin 18	Amp	protein-coding
2144_T12,T1,T22,T2,T32,T3,T42,T4	chr3	5E+07	5E+07	221999	+	**8**	**100**	NA	Intergenic	Intergenic	-184184	NM_130448	73173	Mm.87246	NM_130448	ENSMUSG00000037892	**Pcdh18**	3110038E07Rik|BB095589|PCDH68L	protocadherin 18	Amp	protein-coding
2148_T12,T1,T22,T2,T32,T3,T42,T4	chr3	5E+07	5E+07	27999	+	**8**	**100**	NA	Intergenic	MT2C_Mm|LTR|ERVL	-318184	NM_130448	73173	Mm.87246	NM_130448	ENSMUSG00000037892	**Pcdh18**	3110038E07Rik|BB095589|PCDH68L	protocadherin 18	Amp	protein-coding
726_T12,T1,T22,T2,T32,T3,T42,T4	chr14	9.2E+07	9.2E+07	5999	+	**8**	**100**	NA	Intergenic	Intergenic	1588388	NM_001271800	211712	Mm.37126	NM_001081377	ENSMUSG00000055421	**Pcdh9**	-	protocadherin 9	Amp	protein-coding
748_T12,T1,T22,T2,T32,T3,T42,T4	chr14	9.4E+07	9.4E+07	4999	+	**8**	**100**	NA	intron (NM_001081377, intron 1 of 3)	intron (NM_001081377, intron 1 of 3)	17888	NM_001271800	211712	Mm.37126	NM_001081377	ENSMUSG00000055421	**Pcdh9**	-	protocadherin 9	Amp	protein-coding
2327_T12,T1,T22,T2,T32,T3,T42,T4	chr3	5.8E+07	5.8E+07	238999	+	**8**	**100**	NA	intron (NM_001304454, intron 6 of 9)	intron (NM_001304454, intron 6 of 9)	40757	NM_019410	18645	Mm.271744	NM_019410	ENSMUSG00000027805	**Pfn2**	Pfn	profilin 2	Amp	protein-coding
980_T12,T1,T22,T2,T32,T3,T42,T4	chr15	4.4E+07	4.5E+07	5999	+	**8**	**100**	NA	intron (NM_138674, intron 19 of 76)	intron (NM_138674, intron 19 of 76)	44947	NM_138674	192190	Mm.211494	NM_138674	ENSMUSG00000038725	**Pkhd1l1**	AB055648|PKHDL1	polycystic kidney and hepatic disease 1-like 1	Amp	protein-coding
192_T12,T1,T22,T2,T32,T3,T42,T4	chr1	1.5E+08	1.5E+08	6999	+	**8**	**100**	NA	Intergenic	Intergenic	1017439	NM_001305632	18783	Mm.4186	NM_008869	ENSMUSG00000056220	**Pla2g4a**	Pla2g4|cPLA2|cPLA2-alpha|cPLA2alpha	phospholipase A2, group IVA (cytosolic, calcium-dependent)	Amp	protein-coding
2230_T12,T1,T22,T2,T32,T3,T42,T4	chr3	5.4E+07	5.5E+07	1115999	+	**8**	**100**	NA	intron (NM_016984, intron 5 of 10)	intron (NM_016984, intron 5 of 10)	-70607	NM_001198766	50706	Mm.236067	NM_015784	ENSMUSG00000027750	**Postn**	A630052E07Rik|AI747096|OSF-2|Osf2|PLF|PN	periostin, osteoblast specific factor	Amp	protein-coding
2459_T12,T1,T22,T2,T32,T3,T42,T4	chr3	1.1E+08	1.1E+08	3999	+	**8**	**100**	NA	Intergenic	Intergenic	-500501	NR_024139	99890	Mm.36115	NM_178891	ENSMUSG00000049300	**Prmt6**	AW124876|BB233495|Hrmt1l6	protein arginine N-methyltransferase 6	Amp	protein-coding

168_T12,T1,T22,T2,T32,T3,T42,T4	chr1	1.5E+08	1.5E+08	2999	+	**8**	**100**	NA	Intergenic	Intergenic	-368579	NM_022881	64214	Mm.253927	NM_022881	ENSMUSG00000026357	**Rgs18**	-	regulator of G-protein signaling 18	Amp	protein-coding
176_T12,T1,T22,T2,T32,T3,T42,T4	chr1	1.5E+08	1.5E+08	5999	+	**8**	**100**	NA	Intergenic	ORR1B1|LTR|ERVL-MaLR	-728079	NM_022881	64214	Mm.253927	NM_022881	ENSMUSG00000026357	**Rgs18**	-	regulator of G-protein signaling 18	Amp	protein-coding
1510_T12,T1,T22,T2,T32,T3,T42,T4	chr2	6.7E+07	6.7E+07	3999	+	**8**	**100**	NA	Intergenic	L1MB5|LINE|L1	-33538	NM_001290675	20274	Mm.440889	NM_018852	ENSMUSG00000075316	**Scn9a**	Nav1.7|PN1|mKIAA4197	sodium channel, voltage-gated, type IX, alpha	Amp	protein-coding
969_T12,T1,T22,T2,T32,T3,T42,T4	chr15	3.3E+07	3.3E+07	3999	+	**8**	**100**	NA	Intergenic	Intergenic	-27223	NM_008304	15529	Mm.234266	NM_008304	ENSMUSG00000022261	**Sdc2**	4833414L08Rik|AA960457|Hspg1|Synd2|syndecan-2	syndecan 2	Amp	protein-coding
2350_T12,T1,T22,T2,T32,T3,T42,T4	chr3	5.8E+07	5.9E+07	267999	+	**8**	**100**	NA	Intergenic	L1_Mur3|LINE|L1	-4158	NM_001040396	69227	Mm.270950	NM_001040396	ENSMUSG00000075700	**Selenot**	2810407C02Rik|5730408P04Rik|Selt	selenoprotein T	Amp	protein-coding
2707_T12,T1,T22,T2,T32,T3,T42,T4	chr5	1.2E+07	1.2E+07	7999	+	**8**	**100**	NA	intron (NM_028882, intron 3 of 17)	intron (NM_028882, intron 3 of 17)	81334	NM_028882	108151	Mm.89313	NM_028882	ENSMUSG00000040254	**Sema3d**	4631426B19Rik	sema domain, immunoglobulin domain (Ig), short basic domain, secreted, (semaphorin) 3D	Amp	protein-coding
2231_T12,T1,T22,T2,T32,T3,T42,T4	chr3	5.5E+07	5.5E+07	14999	+	**8**	**100**	NA	Intergenic	Lx5b|LINE|L1	51887	NM_177854	329641	Mm.51187	NM_177854	ENSMUSG00000056306	**Sertm1**	6030405A18Rik	serine rich and transmembrane domain containing 1	Amp	protein-coding
3478_T12,T1,T22,T2,T32,T3,T42,T4	chr8	3.9E+07	3.9E+07	3999	+	**8**	**100**	NA	intron (NM_145841, intron 1 of 7)	intron (NM_145841, intron 1 of 7)	105008	NM_145841	244431	Mm.210388	NM_145841	ENSMUSG00000039539	**Sgcz**	C230085N17Rik	sarcoglycan zeta	Amp	protein-coding
3403_T12,T1,T22,T2,T32,T3,T42,T4	chr8	6390501	6396500	5999	+	**8**	**100**	NA	Intergenic	Intergenic	-1E+06	NM_011388	20494	Mm.3500	NM_011388	ENSMUSG00000023073	**Slc10a2**	9130221J18Rik|AI605518|ASBT|IBAT|ISBT	solute carrier family 10, member 2	Amp	protein-coding
3409_T12,T1,T22,T2,T32,T3,T42,T4	chr8	6522501	6528500	5999	+	**8**	**100**	NA	Intergenic	Intergenic	-1E+06	NM_011388	20494	Mm.3500	NM_011388	ENSMUSG00000023073	**Slc10a2**	9130221J18Rik|AI605518|ASBT|IBAT|ISBT	solute carrier family 10, member 2	Amp	protein-coding
3414_T12,T1,T22,T2,T32,T3,T42,T4	chr8	6647501	6653500	5999	+	**8**	**100**	NA	Intergenic	B4A|SINE|B4	-2E+06	NM_011388	20494	Mm.3500	NM_011388	ENSMUSG00000023073	**Slc10a2**	9130221J18Rik|AI605518|ASBT|IBAT|ISBT	solute carrier family 10, member 2	Amp	protein-coding
2151_T12,T1,T22,T2,T32,T3,T42,T4	chr3	5E+07	5E+07	92999	+	**8**	**100**	NA	Intergenic	MER45A|DNA|hAT-Tip100	303613	NM_011990	26570	Mm.260988	NM_011990	ENSMUSG00000027737	**Slc7a11**	9930009M05Rik|AI451155|sut|xCT	solute carrier family 7 (cationic amino acid transporter, y+ system), member 11	Amp	protein-coding
2153_T12,T1,T22,T2,T32,T3,T42,T4	chr3	5E+07	5E+07	3999	+	**8**	**100**	NA	Intergenic	L1_Mur3|LINE|L1	254113	NM_011990	26570	Mm.260988	NM_011990	ENSMUSG00000027737	**Slc7a11**	9930009M05Rik|AI451155|sut|xCT	solute carrier family 7 (cationic amino acid transporter, y+ system), member 11	Amp	protein-coding

2155_T12,T1,T22,T2,T32,T3,T42,T4	chr3	5E+07	5E+07	271999	+	**8**	**100**	NA	Intergenic	B3|SINE|B2	115113	NM_011990	26570	Mm.260988	NM_011990	ENSMUSG00000027737	**Slc7a11**	9930009M05Rik|AI451155|sut|xCT	solute carrier family 7 (cationic amino acid transporter, y+ system), member 11	Amp	protein-coding
2159_T12,T1,T22,T2,T32,T3,T42,T4	chr3	5E+07	5.1E+07	59999	+	**8**	**100**	NA	Intergenic	AT_rich|Low_complexity|Low_complexity	-66887	NM_011990	26570	Mm.260988	NM_011990	ENSMUSG00000027737	**Slc7a11**	9930009M05Rik|AI451155|sut|xCT	solute carrier family 7 (cationic amino acid transporter, y+ system), member 11	Amp	protein-coding
2161_T12,T1,T22,T2,T32,T3,T42,T4	chr3	5.1E+07	5.1E+07	118999	+	**8**	**100**	NA	Intergenic	Intergenic	-163387	NM_011990	26570	Mm.260988	NM_011990	ENSMUSG00000027737	**Slc7a11**	9930009M05Rik|AI451155|sut|xCT	solute carrier family 7 (cationic amino acid transporter, y+ system), member 11	Amp	protein-coding
2164_T12,T1,T22,T2,T32,T3,T42,T4	chr3	5.1E+07	5.1E+07	20999	+	**8**	**100**	NA	Intergenic	Intergenic	-241387	NM_011990	26570	Mm.260988	NM_011990	ENSMUSG00000027737	**Slc7a11**	9930009M05Rik|AI451155|sut|xCT	solute carrier family 7 (cationic amino acid transporter, y+ system), member 11	Amp	protein-coding
2169_T12,T1,T22,T2,T32,T3,T42,T4	chr3	5.1E+07	5.1E+07	251999	+	**8**	**100**	NA	Intergenic	Intergenic	-386887	NM_011990	26570	Mm.260988	NM_011990	ENSMUSG00000027737	**Slc7a11**	9930009M05Rik|AI451155|sut|xCT	solute carrier family 7 (cationic amino acid transporter, y+ system), member 11	Amp	protein-coding
2747_T12,T1,T22,T2,T32,T3,T42,T4	chr5	4.7E+07	4.7E+07	3999	+	**8**	**100**	NA	Intergenic	Intergenic	-769655	NR_111900	20563	Mm.289739	NM_178804	ENSMUSG00000031558	**Slit2**	Drad-1|E030015M03Rik|E130320P19Rik|Slil3|mKIAA4141|slit-2	slit homolog 2 (Drosophila)	Amp	protein-coding
2749_T12,T1,T22,T2,T32,T3,T42,T4	chr5	4.7E+07	4.7E+07	4999	+	**8**	**100**	NA	Intergenic	Intergenic	-655155	NR_111900	20563	Mm.289739	NM_178804	ENSMUSG00000031558	**Slit2**	Drad-1|E030015M03Rik|E130320P19Rik|Slil3|mKIAA4141|slit-2	slit homolog 2 (Drosophila)	Amp	protein-coding
2752_T12,T1,T22,T2,T32,T3,T42,T4	chr5	4.7E+07	4.7E+07	5999	+	**8**	**100**	NA	Intergenic	Intergenic	-523655	NR_111900	20563	Mm.289739	NM_178804	ENSMUSG00000031558	**Slit2**	Drad-1|E030015M03Rik|E130320P19Rik|Slil3|mKIAA4141|slit-2	slit homolog 2 (Drosophila)	Amp	protein-coding
816_T12,T1,T22,T2,T32,T3,T42,T4	chr14	1.1E+08	1.1E+08	2999	+	**8**	**100**	NA	Intergenic	(CATA)n|Simple_repeat|Simple_repeat	22239	NM_199065	76965	Mm.257268	NM_199065	ENSMUSG00000075478	**Slitrk1**	3200001I04Rik	SLIT and NTRK-like family, member 1	Amp	protein-coding

820_T12,T1,T22,T2,T32,T3,T42,T4	chr14	1.1E+08	1.1E+08	4999	+	**8**	**100**	NA	Intergenic	AT_rich|Low_complexity|Low_complexity	9239	NM_199065	76965	Mm.257268	NM_199065	ENSMUSG00000075478	**Slitrk1**	3200001I04Rik	SLIT and NTRK-like family, member 1	Amp	protein-coding
2376_T12,T1,T22,T2,T32,T3,T42,T4	chr3	7.3E+07	7.3E+07	7999	+	**8**	**100**	NA	Intergenic	Intergenic	20443	NM_198864	386750	Mm.331076	NM_198864	ENSMUSG00000048304	**Slitrk3**	-	SLIT and NTRK-like family, member 3	Amp	protein-coding
844_T12,T1,T22,T2,T32,T3,T42,T4	chr14	1.1E+08	1.1E+08	3999	+	**8**	**100**	NA	Intergenic	Intergenic	136649	NM_175499	239250	Mm.49728	NM_175499	ENSMUSG00000045871	**Slitrk6**	4832410J21Rik|Sltk6	SLIT and NTRK-like family, member 6	Amp	protein-coding
256_T12,T1,T22,T2,T32,T3,T42,T4	chr10	4.7E+07	4.7E+07	4999	+	**8**	**100**	NA	Intergenic	Intergenic	1003251	NR_028276	108637		NR_028276	ENSMUSG00000096263	**Snord14c**	Gm23107|Rnu14|U14	small nucleolar RNA, C/D box 14C	Amp	snoRNA
1360_T12,T1,T22,T2,T32,T3,T42,T4	chr19	5.1E+07	5.1E+07	8999	+	**8**	**100**	NA	Intergenic	Intergenic	-275354	NM_001290356	58178	Mm.313672	NM_021377	ENSMUSG00000043531	**Sorcs1**	Sorcs|mSorCS	sortilin-related VPS10 domain containing receptor 1	Amp	protein-coding
449_T12,T1,T22,T2,T32,T3,T42,T4	chr12	6.2E+07	6.2E+07	4999	+	**8**	**100**	NA	Intergenic	L1_Mus3|LINE|L1	-222787	NR_131949	73309	Mm.276558	NR_131949		**Spanxn4**	1700047L15Rik	SPANX family, member N4	Amp	ncRNA
3574_T12,T1,T22,T2,T32,T3,T42,T4	chr8	6.3E+07	6.3E+07	3999	+	**8**	**100**	NA	intron (NM_023689, intron 7 of 11)	(TG)n|Simple_repeat|Simple_repeat	369155	NM_023689	72902	Mm.334552	NM_023689	ENSMUSG00000054162	**Spock3**	2900045C01Rik|AI428471|mKIAA4039	sparc/osteonectin, cwcv and kazal-like domains proteoglycan 3	Amp	protein-coding
2226_T12,T1,T22,T2,T32,T3,T42,T4	chr3	5.3E+07	5.4E+07	341999	+	**8**	**100**	NA	intron (NM_172862, intron 12 of 23)	intron (NM_172862, intron 12 of 23)	50707	NM_153156	229277	Mm.222810	NM_153156	ENSMUSG00000027744	**Stoml3**	Epb7.2l|SLP3|sro	stomatin (Epb7.2)-like 3	Amp	protein-coding
1627_T12,T1,T22,T2,T32,T3,T42,T4	chr2	1.7E+08	1.7E+08	6999	+	**8**	**100**	NA	Intergenic	(TCTA)n|Simple_repeat|Simple_repeat	-122051	NM_172675	228960	Mm.277977	NM_172675	ENSMUSG00000027522	**Stx16**	4930401D03|5430410K23Rik|6330500A18Rik|AI648908|AW553605|Syn16	syntaxin 16	Amp	protein-coding
2927_T12,T1,T22,T2,T32,T3,T42,T4	chr6	1.7E+07	1.7E+07	82999	+	**8**	**100**	NA	Intergenic	Intergenic	-79149	NM_207176	21753	Mm.436548	NM_011570	ENSMUSG00000029552	**Tes**	D6Ertd352e|TESS|Tes1|Tes2|testin2	testis derived transcript	Amp	protein-coding
2929_T12,T1,T22,T2,T32,T3,T42,T4	chr6	1.7E+07	1.7E+07	6999	+	**8**	**100**	NA	Intergenic	RLTR19-int|LTR|ERVK	-33149	NM_207176	21753	Mm.436548	NM_011570	ENSMUSG00000029552	**Tes**	D6Ertd352e|TESS|Tes1|Tes2|testin2	testis derived transcript	Amp	protein-coding
2930_T12,T1,T22,T2,T32,T3,T42,T4	chr6	1.7E+07	1.7E+07	30999	+	**8**	**100**	NA	Intergenic	HAL1M8|LINE|L1	-7149	NM_207176	21753	Mm.436548	NM_011570	ENSMUSG00000029552	**Tes**	D6Ertd352e|TESS|Tes1|Tes2|testin2	testis derived transcript	Amp	protein-coding
2923_T12,T1,T22,T2,T32,T3,T42,T4	chr6	1.7E+07	1.7E+07	14999	+	**8**	**100**	NA	Intergenic	Lx5|LINE|L1	-2559	NM_031198	21426	Mm.36217	NM_031198	ENSMUSG00000029553	**Tfec**	BB107417|Tcfec|bHLHe34	transcription factor EC	Amp	protein-coding
2925_T12,T1,T22,T2,T32,T3,T42,T4	chr6	1.7E+07	1.7E+07	33999	+	**8**	**100**	NA	Intergenic	Intergenic	-28059	NM_031198	21426	Mm.36217	NM_031198	ENSMUSG00000029553	**Tfec**	BB107417|Tcfec|bHLHe34	transcription factor EC	Amp	protein-coding

2917_T12,T1,T22,T2,T32,T3,T42,T4	chr6	1.7E+07	1.7E+07	85999	+	**8**	**100**	NA	intron (NM_031198, intron 4 of 6)	intron (NM_031198, intron 4 of 6)	54941	NM_031198	21426	Mm.36217	NM_031198	ENSMUSG00000029553	**Tfec**	BB107417|Tcfec|bHLHe34	transcription factor EC	Amp	protein-coding
2287_T12,T1,T22,T2,T32,T3,T42,T4	chr3	5.7E+07	5.7E+07	295999	+	**8**	**100**	NA	Intergenic	Intergenic	549419	NM_008536	17112	Mm.856	NM_008536	ENSMUSG00000027800	**Tm4sf1**	L6|M3s1	transmembrane 4 superfamily member 1	Amp	protein-coding
2290_T12,T1,T22,T2,T32,T3,T42,T4	chr3	5.7E+07	5.7E+07	29999	+	**8**	**100**	NA	Intergenic	Intergenic	375419	NM_008536	17112	Mm.856	NM_008536	ENSMUSG00000027800	**Tm4sf1**	L6|M3s1	transmembrane 4 superfamily member 1	Amp	protein-coding
2292_T12,T1,T22,T2,T32,T3,T42,T4	chr3	5.7E+07	5.7E+07	22999	+	**8**	**100**	NA	Intergenic	Lx8b|LINE|L1	347919	NM_008536	17112	Mm.856	NM_008536	ENSMUSG00000027800	**Tm4sf1**	L6|M3s1	transmembrane 4 superfamily member 1	Amp	protein-coding
2296_T12,T1,T22,T2,T32,T3,T42,T4	chr3	5.7E+07	5.7E+07	69999	+	**8**	**100**	NA	Intergenic	Lx8|LINE|L1	285419	NM_008536	17112	Mm.856	NM_008536	ENSMUSG00000027800	**Tm4sf1**	L6|M3s1	transmembrane 4 superfamily member 1	Amp	protein-coding
2298_T12,T1,T22,T2,T32,T3,T42,T4	chr3	5.7E+07	5.7E+07	4999	+	**8**	**100**	NA	Intergenic	RMER1B|Other|Other	246919	NM_008536	17112	Mm.856	NM_008536	ENSMUSG00000027800	**Tm4sf1**	L6|M3s1	transmembrane 4 superfamily member 1	Amp	protein-coding
2302_T12,T1,T22,T2,T32,T3,T42,T4	chr3	5.7E+07	5.7E+07	11999	+	**8**	**100**	NA	Intergenic	Intergenic	224419	NM_008536	17112	Mm.856	NM_008536	ENSMUSG00000027800	**Tm4sf1**	L6|M3s1	transmembrane 4 superfamily member 1	Amp	protein-coding
2304_T12,T1,T22,T2,T32,T3,T42,T4	chr3	5.7E+07	5.7E+07	99999	+	**8**	**100**	NA	Intergenic	Intergenic	163419	NM_008536	17112	Mm.856	NM_008536	ENSMUSG00000027800	**Tm4sf1**	L6|M3s1	transmembrane 4 superfamily member 1	Amp	protein-coding
2306_T12,T1,T22,T2,T32,T3,T42,T4	chr3	5.7E+07	5.7E+07	22999	+	**8**	**100**	NA	Intergenic	L1_Mus4|LINE|L1	100919	NM_008536	17112	Mm.856	NM_008536	ENSMUSG00000027800	**Tm4sf1**	L6|M3s1	transmembrane 4 superfamily member 1	Amp	protein-coding
2308_T12,T1,T22,T2,T32,T3,T42,T4	chr3	5.7E+07	5.7E+07	22999	+	**8**	**100**	NA	Intergenic	Intergenic	76919	NM_008536	17112	Mm.856	NM_008536	ENSMUSG00000027800	**Tm4sf1**	L6|M3s1	transmembrane 4 superfamily member 1	Amp	protein-coding
2310_T12,T1,T22,T2,T32,T3,T42,T4	chr3	5.7E+07	5.7E+07	13999	+	**8**	**100**	NA	Intergenic	ORR1D1-int|LTR|ERVL-MaLR	54419	NM_008536	17112	Mm.856	NM_008536	ENSMUSG00000027800	**Tm4sf1**	L6|M3s1	transmembrane 4 superfamily member 1	Amp	protein-coding
2315_T12,T1,T22,T2,T32,T3,T42,T4	chr3	5.7E+07	5.7E+07	26999	+	**8**	**100**	NA	Intergenic	Intergenic	-40081	NM_008536	17112	Mm.856	NM_008536	ENSMUSG00000027800	**Tm4sf1**	L6|M3s1	transmembrane 4 superfamily member 1	Amp	protein-coding
2312_T12,T1,T22,T2,T32,T3,T42,T4	chr3	5.7E+07	5.7E+07	62999	+	**8**	**100**	NA	intron (NM_008536, intron 6 of 6)	intron (NM_008536, intron 6 of 6)	12919	NM_008536	17112	Mm.856	NM_008536	ENSMUSG00000027800	**Tm4sf1**	L6|M3s1	transmembrane 4 superfamily member 1	Amp	protein-coding
2318_T12,T1,T22,T2,T32,T3,T42,T4	chr3	5.7E+07	5.7E+07	8999	+	**8**	**100**	NA	Intergenic	Intergenic	-60410	NM_145539	229302	Mm.26618	NM_145539	ENSMUSG00000027801	**Tm4sf4**	Iltmp	transmembrane 4 superfamily member 4	Amp	protein-coding
29_T12,T1,T22,T2,T32,T3,T42,T4	chr1	5E+07	5E+07	3999	+	**8**	**100**	NA	Intergenic	Intergenic	-857023	NM_019790	56363	Mm.245154	NM_019790	ENSMUSG00000026109	**Tmeff2**	4832418D20Rik|7630402F16Rik|TR-2	transmembrane protein with EGF-like and two follistatin-like domains 2	Amp	protein-coding

483_T12,T1, T22,T2,T32, T3,T42,T4	chr12	1.2E+08	1.2E+08	4999	+	**8**	**100**	NA	intron (NM_001294146, intron 1 of 3)	intron (NM_001294146, intron 1 of 3)	63038	NM_001160385	217951	Mm.387395	NM_001160385	ENSMUSG00000048004	**Tmem196**	Gm528	transmembrane protein 196	Amp	protein-coding
977_T12,T1, T22,T2,T32, T3,T42,T4	chr15	4.4E+07	4.4E+07	3999	+	**8**	**100**	NA	Intergenic	Intergenic	19529	NM_175502	239408	Mm.41219	NM_175502	ENSMUSG00000054409	**Tmem74**	AA549547|B230382K22Rik	transmembrane protein 74	Amp	protein-coding
612_T12,T1, T22,T2,T32, T3,T42,T4	chr14	6.1E+07	6.1E+07	4999	+	**8**	**100**	NA	Intergenic	Intergenic	104987	NM_001164155	29820	Mm.281356	NM_013869	ENSMUSG00000060548	**Tnfrsf19**	AL023044|AW123854|TAJ|TAJ-ALPHA|TRADE|Troy	tumor necrosis factor receptor superfamily, member 19	Amp	protein-coding
614_T12,T1, T22,T2,T32, T3,T42,T4	chr14	6.1E+07	6.1E+07	6999	+	**8**	**100**	NA	Intergenic	B1_Mus1|SINE|Alu	93987	NM_001164155	29820	Mm.281356	NM_013869	ENSMUSG00000060548	**Tnfrsf19**	AL023044|AW123854|TAJ|TAJ-ALPHA|TRADE|Troy	tumor necrosis factor receptor superfamily, member 19	Amp	protein-coding
3369_T12,T1,T22,T2,T32,T3,T42,T4	chr7	6.4E+07	6.4E+07	8999	+	**8**	**100**	NA	Intergenic	(CTGTG)n|Simple_repeat|Simple_repeat	-67835	NM_001039104	17364	Mm.38875	NM_018752	ENSMUSG00000030523	**Trpm1**	4732499L03Rik|AI606771|Ltrpc1|Mlsn1	transient receptor potential cation channel, subfamily M, member 1	Amp	protein-coding
1047_T12,T1,T22,T2,T32,T3,T42,T4	chr15	5E+07	5E+07	6999	+	**8**	**100**	NA	Intergenic	Intergenic	669049	NM_001310481	83925	Mm.30466	NM_032000	ENSMUSG00000038679	**Trps1**	AI115454|AI447310|D15Ertd586e	trichorhinophalangeal syndrome I (human)	Amp	protein-coding
1049_T12,T1,T22,T2,T32,T3,T42,T4	chr15	5E+07	5E+07	2999	+	**8**	**100**	NA	Intergenic	Intergenic	549049	NM_001310481	83925	Mm.30466	NM_032000	ENSMUSG00000038679	**Trps1**	AI115454|AI447310|D15Ertd586e	trichorhinophalangeal syndrome I (human)	Amp	protein-coding
2338_T12,T1,T22,T2,T32,T3,T42,T4	chr3	5.8E+07	5.8E+07	999	+	**8**	**100**	NA	exon (NM_001081229, exon 1 of 4)	exon (NM_001081229, exon 1 of 4)	1311	NM_001081229	72033	Mm.218409	NM_001081229	ENSMUSG00000027806	**Tsc22d2**	1810043J12Rik|5530402M19Rik	TSC22 domain family, member 2	Amp	protein-coding
2334_T12,T1,T22,T2,T32,T3,T42,T4	chr3	5.8E+07	5.8E+07	121999	+	**8**	**100**	NA	Intergenic	Intergenic	-73189	NM_001081229	72033	Mm.218409	NM_001081229	ENSMUSG00000027806	**Tsc22d2**	1810043J12Rik|5530402M19Rik	TSC22 domain family, member 2	Amp	protein-coding
2341_T12,T1,T22,T2,T32,T3,T42,T4	chr3	5.8E+07	5.8E+07	2999	+	**8**	**100**	NA	intron (NM_001081229, intron 1 of 3)	intron (NM_001081229, intron 1 of 3)	5311	NM_001081229	72033	Mm.218409	NM_001081229	ENSMUSG00000027806	**Tsc22d2**	1810043J12Rik|5530402M19Rik	TSC22 domain family, member 2	Amp	protein-coding
2343_T12,T1,T22,T2,T32,T3,T42,T4	chr3	5.8E+07	5.8E+07	2999	+	**8**	**100**	NA	intron (NM_001081229, intron 1 of 3)	intron (NM_001081229, intron 1 of 3)	9311	NM_001081229	72033	Mm.218409	NM_001081229	ENSMUSG00000027806	**Tsc22d2**	1810043J12Rik|5530402M19Rik	TSC22 domain family, member 2	Amp	protein-coding
2345_T12,T1,T22,T2,T32,T3,T42,T4	chr3	5.8E+07	5.8E+07	4999	+	**8**	**100**	NA	intron (NM_001081229, intron 2 of 3)	B4A|SINE|B4	14311	NM_001081229	72033	Mm.218409	NM_001081229	ENSMUSG00000027806	**Tsc22d2**	1810043J12Rik|5530402M19Rik	TSC22 domain family, member 2	Amp	protein-coding

2348_T12,T1,T22,T2,T32,T3,T42,T4	chr3	5.8E+07	5.8E+07	2999	+	**8**	**100**	NA	intron (NM_001081229, intron 2 of 3)	intron (NM_001081229, intron 2 of 3)	20311	NM_001081229	72033	Mm.218409	NM_001081229	ENSMUSG00000027806	**Tsc22d2**	1810043J12Rik|5530402M19Rik	TSC22 domain family, member 2	Amp	protein-coding
606_T12,T1,T22,T2,T32,T3,T42,T4	chr14	1.9E+07	1.9E+07	7999	+	**8**	**100**	NA	Intergenic	Intergenic	-160873	NM_144839	218793	Mm.23551	NM_144839	ENSMUSG00000058317	**Ube2e2**	BC016265	ubiquitin-conjugating enzyme E2E 2	Amp	protein-coding
2942_T12,T1,T22,T2,T32,T3,T42,T4	chr6	1.8E+07	1.8E+07	449999	+	**8**	**100**	NA	intron (NM_001289625, intron 12 of 15)	intron (NM_001289625, intron 12 of 15)	117945	NM_023653	22413	Mm.33653	NM_023653	ENSMUSG00000010797	**Wnt2**	2610510E18Rik|Int1l1|Irp|Mirp|Wnt-2|Wnt2a	wingless-type MMTV integration site family, member 2	Amp	protein-coding
2323_T12,T1,T22,T2,T32,T3,T42,T4	chr3	5.7E+07	5.8E+07	292999	+	**8**	**100**	NA	intron (NM_001168281, intron 2 of 6)	intron (NM_001168281, intron 2 of 6)	41876	NM_133784	97064	Mm.405029	NM_133784	ENSMUSG00000027803	**Wwtr1**	2310058J06Rik|2610021I22Rik|C78399|Taz	WW domain containing transcription regulator 1	Amp	protein-coding
1704_T12,T1,T22,T2,T32,T3,T42,T4	chr3	1.7E+07	1.7E+07	4999	+	**8**	**100**	NA	Intergenic	L1MA9|LINE|L1	412817	NM_001145919	229096	Mm.23834	NM_172677	ENSMUSG00000047213	**Ythdf3**	9130022A11Rik	YTH domain family 3	Amp	protein-coding
1698_T12,T1,T22,T2,T32,T3,T42,T4	chr3	1.6E+07	1.6E+07	7999	+	**8**	**100**	NA	intron (NR_027375, intron 5 of 5)	intron (NR_027375, intron 5 of 5)	29317	NM_001145919	229096	Mm.23834	NM_172677	ENSMUSG00000047213	**Ythdf3**	9130022A11Rik	YTH domain family 3	Amp	protein-coding
1325_T12,T1,T22,T32,T3,T42,T4	chr17	9.4E+07	9.4E+07	999	+	**7**	**87.5**	NA	Intergenic	L2a|LINE|L2	441832	NM_009625	11516	Mm.3407	NM_009625	ENSMUSG00000024256	**Adcyap1**	PACAP	adenylate cyclase activating polypeptide 1	Amp	protein-coding
3526_T12,T1,T22,T2,T32,T42,T4	chr8	5.2E+07	5.2E+07	3999	+	**7**	**87.5**	NA	Intergenic	Intergenic	-1E+06	NM_001205054	11593	Mm.334535	NM_001005847	ENSMUSG00000031521	**Aga**	AW060726	aspartylglucosaminidase	Amp	protein-coding
423_T12,T1,T2,T32,T3,T42,T4	chr12	3.7E+07	3.7E+07	5999	+	**7**	**87.5**	NA	intron (NM_178767, intron 12 of 12)	Lx6|LINE|L1	241861	NM_178767	319660	Mm.485540	NM_178767	ENSMUSG00000050103	**Agmo**	A530016O06Rik|AI790538|Tmem195	alkylglycerol monooxygenase	Amp	protein-coding
2981_T12,T1,T22,T2,T3,T42,T4	chr6	1.9E+07	1.9E+07	5999	+	**7**	**87.5**	NA	Intergenic	L1Md_T|LINE|L1	37182	NM_001167757	75196	Mm.67665	NM_029202	ENSMUSG00000029517	**Ankrd7**	4930532L20Rik|AV278427	ankyrin repeat domain 7	Amp	protein-coding
2983_T12,T1,T22,T2,T3,T42,T4	chr6	1.9E+07	1.9E+07	18999	+	**7**	**87.5**	NA	Intergenic	Intergenic	50682	NM_001167757	75196	Mm.67665	NM_029202	ENSMUSG00000029517	**Ankrd7**	4930532L20Rik|AV278427	ankyrin repeat domain 7	Amp	protein-coding
2985_T12,T1,T22,T2,T3,T42,T4	chr6	1.9E+07	1.9E+07	20999	+	**7**	**87.5**	NA	Intergenic	Lx7|LINE|L1	71682	NM_001167757	75196	Mm.67665	NM_029202	ENSMUSG00000029517	**Ankrd7**	4930532L20Rik|AV278427	ankyrin repeat domain 7	Amp	protein-coding
2987_T12,T1,T22,T2,T3,T42,T4	chr6	1.9E+07	1.9E+07	31999	+	**7**	**87.5**	NA	Intergenic	MER34A1|LTR|ERV1	99182	NM_001167757	75196	Mm.67665	NM_029202	ENSMUSG00000029517	**Ankrd7**	4930532L20Rik|AV278427	ankyrin repeat domain 7	Amp	protein-coding
2990_T12,T1,T22,T2,T3,T42,T4	chr6	1.9E+07	1.9E+07	3999	+	**7**	**87.5**	NA	Intergenic	L1Md_T|LINE|L1	119182	NM_001167757	75196	Mm.67665	NM_029202	ENSMUSG00000029517	**Ankrd7**	4930532L20Rik|AV278427	ankyrin repeat domain 7	Amp	protein-coding

2994_T12,T1,T22,T2,T3,T42,T4	chr6	1.9E+07	1.9E+07	35999	+	**7**	**87.5**	NA	Intergenic	L1Md_F2|LINE|L1	142182	NM_001167757	75196	Mm.67665	NM_029202	ENSMUSG00000029517	**Ankrd7**	4930532L20Rik|AV278427	ankyrin repeat domain 7	Amp	protein-coding
2997_T12,T1,T22,T2,T3,T42,T4	chr6	1.9E+07	1.9E+07	12999	+	**7**	**87.5**	NA	Intergenic	L1MB4|LINE|L1	170682	NM_001167757	75196	Mm.67665	NM_029202	ENSMUSG00000029517	**Ankrd7**	4930532L20Rik|AV278427	ankyrin repeat domain 7	Amp	protein-coding
2998_T12,T1,T22,T2,T3,T42,T4	chr6	1.9E+07	1.9E+07	12999	+	**7**	**87.5**	NA	Intergenic	(TAA)n|Simple_repeat|Simple_repeat	184682	NM_001167757	75196	Mm.67665	NM_029202	ENSMUSG00000029517	**Ankrd7**	4930532L20Rik|AV278427	ankyrin repeat domain 7	Amp	protein-coding
3001_T12,T1,T22,T2,T3,T42,T4	chr6	1.9E+07	1.9E+07	8999	+	**7**	**87.5**	NA	Intergenic	Intergenic	197682	NM_001167757	75196	Mm.67665	NM_029202	ENSMUSG00000029517	**Ankrd7**	4930532L20Rik|AV278427	ankyrin repeat domain 7	Amp	protein-coding
3004_T12,T1,T22,T2,T3,T42,T4	chr6	1.9E+07	1.9E+07	3999	+	**7**	**87.5**	NA	Intergenic	L1Md_T|LINE|L1	206182	NM_001167757	75196	Mm.67665	NM_029202	ENSMUSG00000029517	**Ankrd7**	4930532L20Rik|AV278427	ankyrin repeat domain 7	Amp	protein-coding
2979_T12,T1,T22,T2,T3,T42,T4	chr6	1.9E+07	1.9E+07	50999	+	**7**	**87.5**	NA	intron (NM_001167757, intron 5 of 5)	Lx5c|LINE|L1	7682	NM_001167757	75196	Mm.67665	NM_029202	ENSMUSG00000029517	**Ankrd7**	4930532L20Rik|AV278427	ankyrin repeat domain 7	Amp	protein-coding
2326_T12,T1,T22,T2,T3,T42,T4	chr3	5.8E+07	5.8E+07	1999	+	**7**	**87.5**	NA	intron (NM_001033349, intron 2 of 3)	MMETn-int|LTR|ERVK	6037	NM_001033349	242037	Mm.291091	NM_001033349	ENSMUSG00000074591	**Ankub1**	Gm410	ankrin repeat and ubiquitin domain containing 1	Amp	protein-coding
2934_T12,T1,T22,T2,T32,T3,T4	chr6	1.8E+07	1.8E+07	999	+	**7**	**87.5**	NA	Intergenic	Intergenic	-14098	NM_007604	12343	Mm.392504	NM_007604	ENSMUSG00000015733	**Capza2**	1110053K06Rik|AW208808|Cappa2	capping protein (actin filament) muscle Z-line, alpha 2	Amp	protein-coding
2937_T12,T1,T22,T32,T3,T42,T4	chr6	1.8E+07	1.8E+07	999	+	**7**	**87.5**	NA	Intergenic	PB1D10|SINE|Alu	-11098	NM_007604	12343	Mm.392504	NM_007604	ENSMUSG00000015733	**Capza2**	1110053K06Rik|AW208808|Cappa2	capping protein (actin filament) muscle Z-line, alpha 2	Amp	protein-coding
561_T1,T22,T2,T32,T3,T42,T4	chr13	8.5E+07	8.5E+07	999	+	**7**	**87.5**	NA	intron (NM_145452, intron 11 of 24)	intron (NM_145452, intron 11 of 24)	41523	NM_023243	66671	Mm.18474	NM_023243	ENSMUSG00000021548	**Ccnh**	6330408H09Rik|AI661354|AV102684|AW538719	cyclin H	Amp	protein-coding
559_T12,T1,T22,T2,T3,T42,T4	chr13	8.5E+07	8.5E+07	999	+	**7**	**87.5**	NA	intron (NM_145452, intron 19 of 24)	PB1D10|SINE|Alu	34523	NM_023243	66671	Mm.18474	NM_023243	ENSMUSG00000021548	**Ccnh**	6330408H09Rik|AI661354|AV102684|AW538719	cyclin H	Amp	protein-coding
927_T12,T22,T2,T32,T3,T42,T4	chr15	1.9E+07	1.9E+07	999	+	**7**	**87.5**	NA	Intergenic	Intergenic	361671	NM_009865	320873	Mm.117794	NM_009865	ENSMUSG00000022321	**Cdh10**	A830016G23Rik|C030003B10Rik|C030011H18Rik	cadherin 10	Amp	protein-coding
929_T12,T1,T22,T2,T32,T3,T4	chr15	1.9E+07	1.9E+07	999	+	**7**	**87.5**	NA	Intergenic	Intergenic	365671	NM_009865	320873	Mm.117794	NM_009865	ENSMUSG00000022321	**Cdh10**	A830016G23Rik|C030003B10Rik|C030011H18Rik	cadherin 10	Amp	protein-coding
3650_T12,T1,T22,T2,T32,T3,T42	chr8	1E+08	1E+08	999	+	**7**	**87.5**	NA	Intergenic	MTE2a|LTR|ERVL-MaLR	1299111	NM_009866	12552	Mm.1571	NM_009866	ENSMUSG00000031673	**Cdh11**	Cad11|OSF-4	cadherin 11	Amp	protein-coding

3654_T12,T22,T2,T32,T3,T42,T4	chr8	1E+08	1E+08	999	+	**7**	**87.5**	NA	Intergenic	Intergenic	1258111	NM_009866	12552	Mm.1571	NM_009866	ENSMUSG00000031673	**Cdh11**	Cad11|OSF-4	cadherin 11	Amp	protein-coding
958_T12,T1,T22,T2,T32,T42,T4	chr15	2.4E+07	2.4E+07	3999	+	**7**	**87.5**	NA	Intergenic	Intergenic	834037	NM_001081299	320865	Mm.241965	NM_001081299	ENSMUSG00000040420	**Cdh18**	B230220E17Rik|Cdh14l	cadherin 18	Amp	protein-coding
103_T12,T1,T22,T2,T32,T3,T4	chr1	1E+08	1E+08	3999	+	**7**	**87.5**	NA	Intergenic	Intergenic	-1E+06	NM_011800	23836	Mm.103640	NM_011800	ENSMUSG00000050840	**Cdh20**	Cdh7	cadherin 20	Amp	protein-coding
886_T1,T22,T2,T32,T3,T42,T4	chr15	1.5E+07	1.5E+07	3999	+	**7**	**87.5**	NA	Intergenic	Intergenic	-2E+06	NM_007666	12563	Mm.57048	NM_007666	ENSMUSG00000039385	**Cdh6**	cad6	cadherin 6	Amp	protein-coding
119_T12,T1,T22,T2,T32,T42,T4	chr1	1.1E+08	1.1E+08	999	+	**7**	**87.5**	NA	Intergenic	Intergenic	-679900	NM_001316743	241201	Mm.213407	NM_172853	ENSMUSG00000026312	**Cdh7**	9330156F07Rik|CDH7L1	cadherin 7, type 2	Amp	protein-coding
121_T12,T1,T22,T2,T3,T42,T4	chr1	1.1E+08	1.1E+08	999	+	**7**	**87.5**	NA	Intergenic	Intergenic	-461900	NM_001316743	241201	Mm.213407	NM_172853	ENSMUSG00000026312	**Cdh7**	9330156F07Rik|CDH7L1	cadherin 7, type 2	Amp	protein-coding
3623_T12,T1,T22,T2,T32,T3,T4	chr8	9.9E+07	9.9E+07	3999	+	**7**	**87.5**	NA	intron (NR_133570, intron 3 of 10)	intron (NR_133570, intron 3 of 10)	139971	NM_001285913	12564	Mm.441131	NM_007667	ENSMUSG00000036510	**Cdh8**	AI851472|cad8	cadherin 8	Amp	protein-coding
903_T12,T1,T22,T2,T3,T42,T4	chr15	1.6E+07	1.6E+07	999	+	**7**	**87.5**	NA	Intergenic	Intergenic	-797101	NM_009869	12565	Mm.439758	NM_009869	ENSMUSG00000025370	**Cdh9**	-	cadherin 9	Amp	protein-coding
2944_T1,T22,T2,T32,T3,T42,T4	chr6	1.8E+07	1.8E+07	999	+	**7**	**87.5**	NA	Intergenic	Intergenic	-9687	NM_021050	12638	Mm.15621	NM_021050	ENSMUSG00000041301	**Cftr**	AW495489|Abcc7	cystic fibrosis transmembrane conductance regulator	Amp	protein-coding
3729_T12,T1,T22,T2,T3,T42,T4	chr9	1.8E+07	1.8E+07	3999	+	**7**	**87.5**	NA	Intergenic	Intergenic	-607767	NM_025844	66917	Mm.103534	NM_025844	ENSMUSG00000001774	**Chordc1**	1110001O09Rik|AA409036|Chp-1|morgana	cysteine and histidine-rich domain (CHORD)-containing, zinc-binding protein 1	Amp	protein-coding
198_T12,T22,T2,T32,T3,T42,T4	chr1	1.8E+08	1.8E+08	3999	+	**7**	**87.5**	NA	Intergenic	Intergenic	6347	NM_025511	66359	Mm.489720	NM_025511	ENSMUSG00000026500	**Cox20**	2310005N03Rik|Fam36a	COX20 Cox2 chaperone	Amp	protein-coding
1311_T12,T1,T22,T2,T32,T42,T4	chr17	7.7E+07	7.7E+07	4999	+	**7**	**87.5**	NA	Intergenic	RMER15|LTR|ERVL	-981248	NM_015800	50766	Mm.311912	NM_015800	ENSMUSG00000024074	**Crim1**	AU015004	cysteine rich transmembrane BMP regulator 1 (chordin like)	Amp	protein-coding
1029_T12,T1,T2,T32,T3,T42,T4	chr15	4.9E+07	4.9E+07	999	+	**7**	**87.5**	NA	Intergenic	Intergenic	-347011	NM_001081391	239420	Mm.309363	NM_001081391	ENSMUSG00000022311	**Csmd3**	4930500N14Rik|mKIAA1894	CUB and Sushi multiple domains 3	Amp	protein-coding
1032_T12,T1,T2,T32,T3,T42,T4	chr15	4.9E+07	4.9E+07	1999	+	**7**	**87.5**	NA	Intergenic	Intergenic	-409511	NM_001081391	239420	Mm.309363	NM_001081391	ENSMUSG00000022311	**Csmd3**	4930500N14Rik|mKIAA1894	CUB and Sushi multiple domains 3	Amp	protein-coding
1034_T1,T22,T2,T32,T3,T42,T4	chr15	4.9E+07	4.9E+07	999	+	**7**	**87.5**	NA	Intergenic	Intergenic	-415011	NM_001081391	239420	Mm.309363	NM_001081391	ENSMUSG00000022311	**Csmd3**	4930500N14Rik|mKIAA1894	CUB and Sushi multiple domains 3	Amp	protein-coding

1037_T12,T1,T2,T32,T3,T42,T4	chr15	5E+07	5E+07	999	+	**7**	**87.5**	NA	Intergenic	L1_Mur3|LINE|L1	-753011	NM_001081391	239420	Mm.309363	NM_001081391	ENSMUSG00000022311	**Csmd3**	4930500N14Rik|mKIAA1894	CUB and Sushi multiple domains 3	Amp	protein-coding
1025_T12,T1,T22,T2,T32,T3,T42	chr15	4.9E+07	4.9E+07	3999	+	**7**	**87.5**	NA	intron (NM_001081391, intron 1 of 70)	intron (NM_001081391, intron 1 of 70)	34489	NM_001081391	239420	Mm.309363	NM_001081391	ENSMUSG00000022311	**Csmd3**	4930500N14Rik|mKIAA1894	CUB and Sushi multiple domains 3	Amp	protein-coding
1017_T12,T1,T22,T2,T32,T3,T42	chr15	4.8E+07	4.8E+07	3999	+	**7**	**87.5**	NA	intron (NM_001081391, intron 13 of 70)	intron (NM_001081391, intron 13 of 70)	645489	NM_001081391	239420	Mm.309363	NM_001081391	ENSMUSG00000022311	**Csmd3**	4930500N14Rik|mKIAA1894	CUB and Sushi multiple domains 3	Amp	protein-coding
2426_T12,T1,T22,T2,T32,T3,T4	chr3	8.2E+07	8.2E+07	3999	+	**7**	**87.5**	NA	Intergenic	Intergenic	-324116	NM_177662	229445	Mm.254642	NM_177662	ENSMUSG00000028015	**Ctso**	A330105D01Rik|AI118514	cathepsin O	Amp	protein-coding
2951_T1,T22,T2,T32,T3,T42,T4	chr6	1.9E+07	1.9E+07	999	+	**7**	**87.5**	NA	Intergenic	MTC|LTR|ERVL-MaLR	-10175	NM_080285	30785	Mm.224189	NM_080285	ENSMUSG00000000416	**Cttnbp2**	3010022N24Rik|4732477G22Rik|6430526E05|9130022E09Rik|AU040881|Cortbp2|ORF4|mKIAA1758	cortactin binding protein 2	Amp	protein-coding
2955_T1,T22,T2,T32,T3,T42,T4	chr6	1.9E+07	1.9E+07	5999	+	**7**	**87.5**	NA	Intergenic	L1Md_T|LINE|L1	-68675	NM_080285	30785	Mm.224189	NM_080285	ENSMUSG00000000416	**Cttnbp2**	3010022N24Rik|4732477G22Rik|6430526E05|9130022E09Rik|AU040881|Cortbp2|ORF4|mKIAA1758	cortactin binding protein 2	Amp	protein-coding
2956_T1,T22,T2,T32,T3,T42,T4	chr6	1.9E+07	1.9E+07	999	+	**7**	**87.5**	NA	Intergenic	Intergenic	-73175	NM_080285	30785	Mm.224189	NM_080285	ENSMUSG00000000416	**Cttnbp2**	3010022N24Rik|4732477G22Rik|6430526E05|9130022E09Rik|AU040881|Cortbp2|ORF4|mKIAA1758	cortactin binding protein 2	Amp	protein-coding
2960_T12,T1,T22,T2,T32,T42,T4	chr6	1.9E+07	1.9E+07	999	+	**7**	**87.5**	NA	Intergenic	L1MEg|LINE|L1	-115175	NM_080285	30785	Mm.224189	NM_080285	ENSMUSG00000000416	**Cttnbp2**	3010022N24Rik|4732477G22Rik|6430526E05|9130022E09Rik|AU040881|Cortbp2|ORF4|mKIAA1758	cortactin binding protein 2	Amp	protein-coding
2946_T12,T1,T22,T2,T3,T42,T4	chr6	1.8E+07	1.8E+07	60999	+	**7**	**87.5**	NA	intron (NM_080285, intron 16 of 22)	B4|SINE|B4	129825	NM_080285	30785	Mm.224189	NM_080285	ENSMUSG00000000416	**Cttnbp2**	3010022N24Rik|4732477G22Rik|6430526E05|9130022E09Rik|AU040881|Cortbp2|ORF4|mKIAA1758	cortactin binding protein 2	Amp	protein-coding

2947_T12,T1,T22,T2,T3,T42,T4	chr6	1.8E+07	1.9E+07	84999	+	**7**	**87.5**	NA	intron (NM_080285, intron 2 of 22)	intron (NM_080285, intron 2 of 22)	55825	NM_080285	30785	Mm.224189	NM_080285	ENSMUSG00000000416	**Cttnbp2**	3010022N24Rik|4732477G22Rik|6430526E05|9130022E09Rik|AU040881|Cortbp2|ORF4|mKIAA1758	cortactin binding protein 2	Amp	protein-coding
876_T12,T1,T2,T32,T3,T42,T4	chr15	5978501	5982500	3999	+	**7**	**87.5**	NA	Intergenic	Intergenic	-319289	NM_001008702	13132	Mm.240830	NM_023118	ENSMUSG00000022150	**Dab2**	5730435J12Rik|AA960054|AI957090|D15Wsu122e|D630005B22Rik|Doc-2|Doc2|p96	disabled 2, mitogen-responsive phosphoprotein	Amp	protein-coding
879_T12,T1,T22,T2,T32,T3,T42	chr15	6128501	6132500	3999	+	**7**	**87.5**	NA	Intergenic	MTE2b|LTR|ERVL-MaLR	-169289	NM_001008702	13132	Mm.240830	NM_023118	ENSMUSG00000022150	**Dab2**	5730435J12Rik|AA960054|AI957090|D15Wsu122e|D630005B22Rik|Doc-2|Doc2|p96	disabled 2, mitogen-responsive phosphoprotein	Amp	protein-coding
780_T1,T22,T2,T32,T3,T42,T4	chr14	9.8E+07	9.8E+07	999	+	**7**	**87.5**	NA	Intergenic	Intergenic	640765	NM_001038610	13134	Mm.320593	NM_007826	ENSMUSG00000055639	**Dach1**	Dac|Dach	dachshund 1 (Drosophila)	Amp	protein-coding
2237_T12,T1,T22,T2,T32,T42,T4	chr3	5.6E+07	5.6E+07	999	+	**7**	**87.5**	NA	Intergenic	Intergenic	140242	NM_001195540	13175	Mm.393242	NM_019978	ENSMUSG00000027797	**Dclk1**	1700113D08Rik|2810480F11Rik|AI836758|Click-I|Cpg16|Dcamkl1|Dcl|Dclk|mKIAA0369	doublecortin-like kinase 1	Amp	protein-coding
132_T12,T1,T22,T32,T3,T42,T4	chr1	1.1E+08	1.1E+08	999	+	**7**	**87.5**	NA	Intergenic	MTC|LTR|ERVL-MaLR	-1E+06	NM_001081316	319901	Mm.103468	NM_001081316	ENSMUSG00000038702	**Dsel**	9330132E09Rik|DS-epi2	dermatan sulfate epimerase-like	Amp	protein-coding
1293_T12,T22,T2,T32,T3,T42,T4	chr17	6.2E+07	6.2E+07	999	+	**7**	**87.5**	NA	Intergenic	Intergenic	1035317	NM_207654	13640	Mm.7978	NM_010109	ENSMUSG00000048915	**Efna5**	AL-1|AV158822|EFL-5|Ephrin-A5|Epl7|LERK-7|RAGS	ephrin A5	Amp	protein-coding
1112_T12,T1,T2,T32,T3,T42,T4	chr15	6.5E+07	6.5E+07	999	+	**7**	**87.5**	NA	Intergenic	(TTTG)n|Simple_repeat|Simple_repeat	-366041	NM_133766	76740	Mm.260647	NM_133766	ENSMUSG00000015002	**Efr3a**	A130089M23Rik|BB071175|C76891|C920006C10Rik|D030063F01Rik|mKIAA0143	EFR3 homolog A	Amp	protein-coding
2873_T12,T1,T22,T2,T32,T3,T4	chr5	8.5E+07	8.5E+07	3999	+	**7**	**87.5**	NA	Intergenic	Intergenic	-259118	NM_007937	13839	Mm.137991	NM_007937	ENSMUSG00000029245	**Epha5**	AI854630|AW125296|Cek7|Ehk1|Els1|Hek7|Rek7|bsk	Eph receptor A5	Amp	protein-coding
1165_T12,T22,T2,T32,T3,T42,T4	chr16	6.1E+07	6.1E+07	5999	+	**7**	**87.5**	NA	Intergenic	Intergenic	-666969	NM_007938	13840	Mm.455790	NM_007938	ENSMUSG00000055540	**Epha6**	Ehk2|Hek12|m-ehk2	Eph receptor A6	Amp	protein-coding

2559_T1,T22,T2,T32,T3,T42,T4	chr4	2.9E+07	2.9E+07	5999	+	**7**	**87.5**	NA	intron (NM_010141, intron 5 of 16)	intron (NM_010141, intron 5 of 16)	68355	NM_001122889	13841	Mm.257266	NM_010141	ENSMUSG00000028289	**Epha7**	Cek11|Ebk|Ehk3|Hek11|Mdk1	Eph receptor A7	Amp	protein-coding
46_T12,T1,T22,T2,T32,T3,T4	chr1	6.9E+07	6.9E+07	1999	+	**7**	**87.5**	NA	intron (NM_010154, intron 2 of 26)	A-rich|Low_complexity|Low_complexity	422559	NM_010154	13869	Mm.442420	NM_010154	ENSMUSG00000062209	**Erbb4**	Her4|c-erbB-4	erb-b2 receptor tyrosine kinase 4	Amp	protein-coding
2663_T12,T1,T22,T2,T32,T3,T42	chr4	1.1E+08	1.1E+08	4999	+	**7**	**87.5**	NA	intron (NM_007983, intron 6 of 18)	MIRb|SINE|MIR	69373	NM_007983	14084	Mm.318259	NM_007983	ENSMUSG00000010517	**Faf1**	AA408698|Dffrx|Fam	Fas-associated factor 1	Amp	protein-coding
3762_T12,T1,T22,T2,T32,T42,T4	chr9	7.7E+07	7.7E+07	3999	+	**7**	**87.5**	NA	Intergenic	Intergenic	-91696	NM_001045518	208994	Mm.256929	NM_001045518	ENSMUSG00000032358	**Fam83b**	C530008M07Rik|Gm516	family with sequence similarity 83, member B	Amp	protein-coding
1342_T12,T1,T22,T2,T3,T42,T4	chr19	2.5E+07	2.5E+07	3999	+	**7**	**87.5**	NA	Intergenic	Intergenic	-14191	NM_008022	14237	Mm.377080	NM_008022	ENSMUSG00000051490	**Foxd4**	FREAC5|Fkh2	forkhead box D4	Amp	protein-coding
474_T12,T1,T2,T32,T3,T42,T4	chr12	1E+08	1E+08	4999	+	**7**	**87.5**	NA	Intergenic	Intergenic	-56926	NM_183186	71375	Mm.341972	NM_183186	ENSMUSG00000033713	**Foxn3**	5430426H20Rik|AA589593|AW556347|Ches1|Ches1l|HTLFL1	forkhead box N3	Amp	protein-coding
2204_T1,T22,T2,T32,T3,T42,T4	chr3	5.2E+07	5.2E+07	999	+	**7**	**87.5**	NA	Intergenic	Intergenic	159663	NM_019739	56458	Mm.29891	NM_019739	ENSMUSG00000044167	**Foxo1**	AI876417|Afxh|FKHR|Fkhr1|Foxo1a	forkhead box O1	Amp	protein-coding
2200_T1,T22,T2,T32,T3,T42,T4	chr3	5.2E+07	5.2E+07	999	+	**7**	**87.5**	NA	intron (NM_019739, intron 1 of 2)	intron (NM_019739, intron 1 of 2)	19663	NM_019739	56458	Mm.29891	NM_019739	ENSMUSG00000044167	**Foxo1**	AI876417|Afxh|FKHR|Fkhr1|Foxo1a	forkhead box O1	Amp	protein-coding
2227_T12,T1,T2,T32,T3,T42,T4	chr3	5.4E+07	5.4E+07	3999	+	**7**	**87.5**	NA	Intergenic	L1Md_F2|LINE|L1	-55145	NM_172862	242022	Mm.38378	NM_172862	ENSMUSG00000037016	**Frem2**	6030440P17Rik|8430406N05Rik|Gm409|b2b1562Clo|my|ne|nv1	Fras1 related extracellular matrix protein 2	Amp	protein-coding
2229_T12,T1,T2,T32,T3,T42,T4	chr3	5.4E+07	5.4E+07	9999	+	**7**	**87.5**	NA	Intergenic	Intergenic	-70145	NM_172862	242022	Mm.38378	NM_172862	ENSMUSG00000037016	**Frem2**	6030440P17Rik|8430406N05Rik|Gm409|b2b1562Clo|my|ne|nv1	Fras1 related extracellular matrix protein 2	Amp	protein-coding
2399_T12,T1,T2,T32,T3,T42,T4	chr3	7.7E+07	7.7E+07	999	+	**7**	**87.5**	NA	intron (NM_178673, intron 12 of 15)	L1MD|LINE|L1	570417	NM_001253719	213262	Mm.379337	NM_178673	ENSMUSG00000034098	**Fstl5**	9130207J01Rik	follistatin-like 5	Amp	protein-coding
384_T12,T1,T22,T2,T3,T42,T4	chr11	4.2E+07	4.2E+07	3999	+	**7**	**87.5**	NA	intron (NM_177408, intron 1 of 8)	intron (NM_177408, intron 1 of 8)	6214	NM_177408	14406	Mm.5309	NM_008073	ENSMUSG00000020436	**Gabrg2**	GABAA-R|Gabrg-2|gamma2	gamma-aminobutyric acid (GABA) A receptor, subunit gamma 2	Amp	protein-coding

581_T1,T22,T2,T32,T3,T42,T4	chr13	9.7E+07	9.7E+07	4999	+	**7**	**87.5**	NA	Intergenic	L1_Mus3|LINE|L1	-84689	NM_001166065	218476	Mm.483490	NM_001166065	ENSMUSG00000091387	**Gcnt4**	C2gnt3|Gm279|Gm73	glucosaminyl (N-acetyl) transferase 4, core 2 (beta-1,6-N-acetylglucosaminyl transferase)	Amp	protein-coding
274_T12,T1,T2,T32,T3,T42,T4	chr10	5E+07	5E+07	999	+	**7**	**87.5**	NA	intron (NM_010349, intron 2 of 17)	Lx7|LINE|L1	105754	NM_010349	14806	Mm.332838	NM_010349	ENSMUSG00000056073	**Grik2**	AW124492|GluK2|Glur-6|Glur6|Glurbeta2	glutamate receptor, ionotropic, kainate 2 (beta 2)	Amp	protein-coding
2699_T12,T1,T22,T2,T3,T42,T4	chr5	1E+07	1E+07	999	+	**7**	**87.5**	NA	Intergenic	Lx8b|LINE|L1	-292648	NM_181850	108069	Mm.318966	NM_181850	ENSMUSG00000003974	**Grm3**	0710001G23Rik|Gprc1c|mGlu3|mGluR3	glutamate receptor, metabotropic 3	Amp	protein-coding
1599_T12,T1,T22,T2,T3,T42,T4	chr2	1.3E+08	1.3E+08	4999	+	**7**	**87.5**	NA	Intergenic	Intergenic	197352	NM_010403	15112	Mm.26634	NM_010403	ENSMUSG00000027261	**Hao1**	GOX|Gox1|Hao-1	hydroxyacid oxidase 1, liver	Amp	protein-coding
1072_T12,T1,T22,T2,T3,T42,T4	chr15	5.7E+07	5.7E+07	4999	+	**7**	**87.5**	NA	Intergenic	Intergenic	-282454	NM_008216	15117	Mm.5148	NM_008216	ENSMUSG00000022367	**Has2**	-	hyaluronan synthase 2	Amp	protein-coding
1075_T12,T1,T22,T2,T32,T42,T4	chr15	5.7E+07	5.7E+07	5999	+	**7**	**87.5**	NA	Intergenic	Intergenic	-379954	NM_008216	15117	Mm.5148	NM_008216	ENSMUSG00000022367	**Has2**	-	hyaluronan synthase 2	Amp	protein-coding
1624_T12,T1,T22,T2,T3,T42,T4	chr2	1.5E+08	1.5E+08	3999	+	**7**	**87.5**	NA	Intergenic	Intergenic	-11968	NM_001172117	15162	Mm.715	NM_010407	ENSMUSG00000003283	**Hck**	AI849071|Bmk	hemopoietic cell kinase	Amp	protein-coding
1644_T12,T1,T22,T2,T32,T42,T4	chr3	4010501	4014500	3999	+	**7**	**87.5**	NA	Intergenic	Intergenic	504470	NM_013920	30942	Mm.330897	NM_013920	ENSMUSG00000017688	**Hnf4g**	NR2A2	hepatocyte nuclear factor 4, gamma	Amp	protein-coding
2445_T1,T22,T2,T32,T3,T42,T4	chr3	9.3E+07	9.3E+07	10999	+	**7**	**87.5**	NA	Intergenic	Intergenic	-33749	NM_133698	68723	Mm.208047	NM_133698	ENSMUSG00000041991	**Hrnr**	1110033K19Rik|A530063N20Rik|S100a18	hornerin	Amp	protein-coding
2737_T1,T22,T2,T32,T3,T42,T4	chr5	4E+07	4E+07	4999	+	**7**	**87.5**	NA	Intergenic	Intergenic	-744369	NM_010474	15476	Mm.12559	NM_010474	ENSMUSG00000051022	**Hs3st1**	3-Ost|D5Wsu110e|Hsg3ost	heparan sulfate (glucosamine) 3-O-sulfotransferase 1	Amp	protein-coding
239_T12,T1,T2,T32,T3,T42,T4	chr10	3.6E+07	3.6E+07	1999	+	**7**	**87.5**	NA	Intergenic	L1_Mus3|LINE|L1	-723307	NM_001253355	319415	Mm.332442	NM_001081208	ENSMUSG00000044499	**Hs3st5**	D930005L05Rik|Gm1151|Hs3ost5	heparan sulfate (glucosamine) 3-O-sulfotransferase 5	Amp	protein-coding
241_T12,T1,T22,T2,T3,T42,T4	chr10	3.6E+07	3.6E+07	6999	+	**7**	**87.5**	NA	Intergenic	ORR1B1-int|LTR|ERVL-MaLR	-712807	NM_001253355	319415	Mm.332442	NM_001081208	ENSMUSG00000044499	**Hs3st5**	D930005L05Rik|Gm1151|Hs3ost5	heparan sulfate (glucosamine) 3-O-sulfotransferase 5	Amp	protein-coding
588_T12,T1,T22,T2,T32,T3,T4	chr13	1.1E+08	1.1E+08	4999	+	**7**	**87.5**	NA	Intergenic	Intergenic	241307	NM_008308	15550	Mm.4716	NM_008308	ENSMUSG00000021721	**Htr1a**	Gpcr18	5-hydroxytryptamine (serotonin) receptor 1A	Amp	protein-coding
596_T12,T22,T2,T32,T3,T42,T4	chr13	1.2E+08	1.2E+08	3999	+	**7**	**87.5**	NA	Intergenic	Intergenic	394188	NM_021459	16392	Mm.42242	NM_021459	ENSMUSG00000042258	**Isl1**	-	ISL1 transcription factor, LIM/homeodomain	Amp	protein-coding

1581_T1,T22,T2,T32,T3,T42,T4	chr2	1.1E+08	1.1E+08	4999	+	**7**	**87.5**	NA	Intergenic	Lx8b|LINE|L1	851411	NM_021275	16492	Mm.142718	NM_021275	ENSMUSG00000042604	**Kcna4**	Kv1.4	potassium voltage-gated channel, shaker-related subfamily, member 4	Amp	protein-coding
3034_T12,T1,T22,T2,T3,T42,T4	chr6	2E+07	2.1E+07	210999	+	**7**	**87.5**	NA	Intergenic	RLTR33|LTR|ERVK	-790109	NM_019697	16508	Mm.425316	NM_019697	ENSMUSG00000060882	**Kcnd2**	AI839615|AW555701|Kv4.2|R75121|mKIAA1044	potassium voltage-gated channel, Shal-related family, member 2	Amp	protein-coding
1496_T1,T22,T2,T32,T3,T42,T4	chr2	5.5E+07	5.5E+07	3999	+	**7**	**87.5**	NA	intron (NM_008426, intron 3 of 3)	intron (NM_008426, intron 3 of 3)	24530	NM_008426	16519	Mm.5127	NM_008426	ENSMUSG00000026824	**Kcnj3**	GIRK-1|GIRK1|Kcnf3|Kir3.1	potassium inwardly-rectifying channel, subfamily J, member 3	Amp	protein-coding
3392_T12,T1,T22,T32,T3,T42,T4	chr7	1.4E+08	1.4E+08	3999	+	**7**	**87.5**	NA	non-coding (NR_001461, exon 1 of 1)	non-coding (NR_001461, exon 1 of 1)	55047	NR_001461	63830	Mm.356096	NR_001461	ENSMUSG00000101609	**Kcnq1ot1**	Kvlqt1-as|Lit1|Tssc8	KCNQ1 overlapping transcript 1	Amp	ncRNA
991_T1,T22,T2,T32,T3,T42,T4	chr15	4.6E+07	4.6E+07	3999	+	**7**	**87.5**	NA	Intergenic	(TA)n|Simple_repeat|Simple_repeat	-389566	NM_026200	67498	Mm.300079	NM_026200	ENSMUSG00000022342	**Kcnv1**	2700023A03Rik|vibe	potassium channel, subfamily V, member 1	Amp	protein-coding
401_T12,T1,T22,T2,T3,T42,T4	chr11	9.2E+07	9.2E+07	5999	+	**7**	**87.5**	NA	Intergenic	Intergenic	-399945	NM_028547	73470	Mm.67677	NM_028547	ENSMUSG00000046755	**Kif2b**	1700063D03Rik	kinesin family member 2B	Amp	protein-coding
404_T12,T1,T22,T2,T3,T42,T4	chr11	9.2E+07	9.2E+07	999	+	**7**	**87.5**	NA	Intergenic	Intergenic	-503445	NM_028547	73470	Mm.67677	NM_028547	ENSMUSG00000046755	**Kif2b**	1700063D03Rik	kinesin family member 2B	Amp	protein-coding
406_T1,T22,T2,T32,T3,T42,T4	chr11	9.2E+07	9.2E+07	1999	+	**7**	**87.5**	NA	Intergenic	Intergenic	-509945	NM_028547	73470	Mm.67677	NM_028547	ENSMUSG00000046755	**Kif2b**	1700063D03Rik	kinesin family member 2B	Amp	protein-coding
777_T12,T1,T22,T2,T3,T42,T4	chr14	9.7E+07	9.7E+07	5999	+	**7**	**87.5**	NA	Intergenic	Intergenic	-294466	NM_053105	93688	Mm.308735	NM_053105	ENSMUSG00000022076	**Klhl1**	mKIAA1490	kelch-like 1	Amp	protein-coding
1466_T1,T22,T2,T32,T3,T42,T4	chr2	4.3E+07	4.3E+07	999	+	**7**	**87.5**	NA	Intergenic	Intergenic	-255402	NM_053011	94217	Mm.441398	NM_053011	ENSMUSG00000049252	**Lrp1b**	9630004P12Rik|LRP-DIT	low density lipoprotein-related protein 1B (deleted in tumors)	Amp	protein-coding
1468_T12,T1,T2,T32,T3,T42,T4	chr2	4.3E+07	4.3E+07	999	+	**7**	**87.5**	NA	Intergenic	Intergenic	-260402	NM_053011	94217	Mm.441398	NM_053011	ENSMUSG00000049252	**Lrp1b**	9630004P12Rik|LRP-DIT	low density lipoprotein-related protein 1B (deleted in tumors)	Amp	protein-coding
1561_T12,T1,T22,T32,T3,T42,T4	chr2	9.8E+07	9.8E+07	999	+	**7**	**87.5**	NA	Intergenic	Intergenic	172343	NM_178725	241568	Mm.241682	NM_178725	ENSMUSG00000050587	**Lrrc4c**	6430556C10Rik|NGL-1	leucine rich repeat containing 4C	Amp	protein-coding
1543_T12,T1,T22,T2,T3,T42,T4	chr2	9.7E+07	9.7E+07	4999	+	**7**	**87.5**	NA	intron (NM_001289742, intron 3 of 7)	MamRep38|DNA|hAT	-389657	NM_001289744	241568	Mm.241682	NM_178725	ENSMUSG00000050587	**Lrrc4c**	6430556C10Rik|NGL-1	leucine rich repeat containing 4C	Amp	protein-coding

1555_T12,T1,T22,T2,T32,T3,T42	chr2	9.8E+07	9.8E+07	999	+	**7**	**87.5**	NA	intron (NM_001289742, intron 7 of 7)	Lx8|LINE|L1	140343	NM_178725	241568	Mm.241682	NM_178725	ENSMUSG00000050587	**Lrrc4c**	6430556C10Rik|NGL-1	leucine rich repeat containing 4C	Amp	protein-coding
2965_T12,T1,T22,T2,T3,T42,T4	chr6	1.9E+07	1.9E+07	999	+	**7**	**87.5**	NA	Intergenic	Intergenic	-85635	NM_133939	76522	Mm.275158	NM_133939	ENSMUSG00000044155	**Lsm8**	2010003I05Rik|AW214405|Naa38	LSM8 homolog, U6 small nuclear RNA associated	Amp	protein-coding
2972_T12,T1,T22,T2,T3,T42,T4	chr6	1.9E+07	1.9E+07	16999	+	**7**	**87.5**	NA	Intergenic	Intergenic	-16635	NM_133939	76522	Mm.275158	NM_133939	ENSMUSG00000044155	**Lsm8**	2010003I05Rik|AW214405|Naa38	LSM8 homolog, U6 small nuclear RNA associated	Amp	protein-coding
2976_T12,T1,T22,T2,T3,T42,T4	chr6	1.9E+07	1.9E+07	2999	+	**7**	**87.5**	NA	Intergenic	MMVL30-int|LTR|ERV1	-3635	NM_133939	76522	Mm.275158	NM_133939	ENSMUSG00000044155	**Lsm8**	2010003I05Rik|AW214405|Naa38	LSM8 homolog, U6 small nuclear RNA associated	Amp	protein-coding
2245_T12,T1,T22,T32,T3,T42,T4	chr3	5.6E+07	5.6E+07	4999	+	**7**	**87.5**	NA	intron (NM_030595, intron 40 of 57)	MER90a|LTR|ERV1	7490	NM_010750	17116	Mm.440209	NM_010750	ENSMUSG00000056947	**Mab21l1**	AW047968	mab-21-like 1 (C. elegans)	Amp	protein-coding
2729_T12,T1,T22,T2,T3,T42,T4	chr5	2E+07	2E+07	999	+	**7**	**87.5**	NA	intron (NM_001170746, intron 1 of 22)	intron (NM_001170746, intron 1 of 22)	335954	NM_001170746	50791	Mm.332231	NM_015823	ENSMUSG00000040003	**Magi2**	AIP-1|Acvri1|Acvrinp1|Acvrip1|Magi-2|S-SCAM|mKIAA0705	membrane associated guanylate kinase, WW and PDZ domain containing 2	Amp	protein-coding
2557_T12,T1,T2,T32,T3,T42,T4	chr4	2.6E+07	2.6E+07	999	+	**7**	**87.5**	NA	Intergenic	Intergenic	240652	NM_172865	242362	Mm.245602	NM_172865	ENSMUSG00000040520	**Manea**	4932703L02Rik	mannosidase, endo-alpha	Amp	protein-coding
3763_T12,T1,T22,T2,T32,T3,T4	chr9	8.2E+07	8.2E+07	4999	+	**7**	**87.5**	NA	intron (NM_175213, intron 3 of 4)	intron (NM_175213, intron 3 of 4)	111256	NM_175213	75033	Mm.335329	NM_175213	ENSMUSG00000043289	**Mei4**	4930486G11Rik	meiotic double-stranded break formation protein 4	Amp	protein-coding
2179_T12,T1,T2,T32,T3,T42,T4	chr3	5.1E+07	5.1E+07	11999	+	**7**	**87.5**	NA	promoter-TSS (NR_028121)	promoter-TSS (NR_028121)	-953	NR_028121	67749	Mm.273339	NM_026358	ENSMUSG00000037161	**Mgarp**	4930583H14Rik|AI195347|CESP-1|HUMMR|Osap|Qsap	mitochondria localized glutamic acid rich protein	Amp	protein-coding
3466_T12,T1,T22,T2,T32,T3,T42	chr8	3.8E+07	3.8E+07	2999	+	**7**	**87.5**	NA	intron (NM_145841, intron 1 of 7)	intron (NM_145841, intron 1 of 7)	75202	NR_029884	723860		NR_029884	ENSMUSG00000065457	**Mir383**	Mirn383|mir-383|mmu-mir-383	microRNA 383	Amp	ncRNA
2578_T12,T1,T22,T32,T3,T42,T4	chr4	3.6E+07	3.6E+07	4999	+	**7**	**87.5**	NA	intron (NM_001166000, intron 1 of 8)	intron (NM_001166000, intron 1 of 8)	181453	NR_030545	1E+08		NR_030545	ENSMUSG00000077851	**Mir876**	Mirn876|mir-876|mmu-mir-876	microRNA 876	Amp	ncRNA
2582_T12,T1,T22,T32,T3,T42,T4	chr4	3.7E+07	3.7E+07	4999	+	**7**	**87.5**	NA	intron (NM_001166000, intron 1 of 8)	B1F|SINE|Alu	86453	NR_030545	1E+08		NR_030545	ENSMUSG00000077851	**Mir876**	Mirn876|mir-876|mmu-mir-876	microRNA 876	Amp	ncRNA

3484_T1,T22,T2,T32,T3,T42,T4	chr8	4E+07	4E+07	999	+	**7**	**87.5**	NA	intron (NM_001113326, intron 9 of 9)	intron (NM_001113326, intron 9 of 9)	58678	NM_031195	20288	Mm.239291	NM_031195	ENSMUSG00000025044	**Msr1**	MRS-A|MSR|MSR-A|SR-AI|SR-AII|Scara1|Scvr	macrophage scavenger receptor 1	Amp	protein-coding
2182_T12,T1,T22,T32,T3,T42,T4	chr3	5.1E+07	5.1E+07	999	+	**7**	**87.5**	NA	intron (NM_053089, intron 2 of 19)	intron (NM_053089, intron 2 of 19)	20984	NM_053089	74838	Mm.275281	NM_053089	ENSMUSG00000063273	**Naa15**	5730450D16Rik|6330400I15|ASTBDN|Narg1|Tbdn-1|mNAT1	N(alpha)-acetyltransferase 15, NatA auxiliary subunit	Amp	protein-coding
2262_T12,T1,T2,T32,T3,T42,T4	chr3	5.6E+07	5.6E+07	5999	+	**7**	**87.5**	NA	Intergenic	L1Md_T|LINE|L1	-28799	NM_030595	26422	Mm.384353	NM_030595	ENSMUSG00000027799	**Nbea**	Lyst2|mKIAA1544	neurobeachin	Amp	protein-coding
2265_T12,T1,T2,T32,T3,T42,T4	chr3	5.6E+07	5.6E+07	6999	+	**7**	**87.5**	NA	Intergenic	L1Md_A|LINE|L1	-66299	NM_030595	26422	Mm.384353	NM_030595	ENSMUSG00000027799	**Nbea**	Lyst2|mKIAA1544	neurobeachin	Amp	protein-coding
2270_T12,T1,T22,T2,T3,T42,T4	chr3	5.6E+07	5.6E+07	2999	+	**7**	**87.5**	NA	Intergenic	Intergenic	-197299	NM_030595	26422	Mm.384353	NM_030595	ENSMUSG00000027799	**Nbea**	Lyst2|mKIAA1544	neurobeachin	Amp	protein-coding
2272_T12,T1,T22,T2,T32,T42,T4	chr3	5.6E+07	5.6E+07	999	+	**7**	**87.5**	NA	Intergenic	L1_Mus3|LINE|L1	-200299	NM_030595	26422	Mm.384353	NM_030595	ENSMUSG00000027799	**Nbea**	Lyst2|mKIAA1544	neurobeachin	Amp	protein-coding
2273_T12,T22,T2,T32,T3,T42,T4	chr3	5.6E+07	5.6E+07	999	+	**7**	**87.5**	NA	Intergenic	L1_Mus3|LINE|L1	-201299	NM_030595	26422	Mm.384353	NM_030595	ENSMUSG00000027799	**Nbea**	Lyst2|mKIAA1544	neurobeachin	Amp	protein-coding
2283_T12,T1,T22,T2,T32,T3,T4	chr3	5.7E+07	5.7E+07	999	+	**7**	**87.5**	NA	Intergenic	Lx2|LINE|L1	-380299	NM_030595	26422	Mm.384353	NM_030595	ENSMUSG00000027799	**Nbea**	Lyst2|mKIAA1544	neurobeachin	Amp	protein-coding
2285_T12,T1,T22,T2,T32,T3,T4	chr3	5.7E+07	5.7E+07	999	+	**7**	**87.5**	NA	Intergenic	Lx2|LINE|L1	-385299	NM_030595	26422	Mm.384353	NM_030595	ENSMUSG00000027799	**Nbea**	Lyst2|mKIAA1544	neurobeachin	Amp	protein-coding
2257_T12,T1,T22,T2,T3,T42,T4	chr3	5.6E+07	5.6E+07	3999	+	**7**	**87.5**	NA	intron (NM_030595, intron 1 of 57)	L1Md_T|LINE|L1	63201	NM_030595	26422	Mm.384353	NM_030595	ENSMUSG00000027799	**Nbea**	Lyst2|mKIAA1544	neurobeachin	Amp	protein-coding
2223_T12,T1,T22,T2,T32,T42,T4	chr3	5.3E+07	5.3E+07	9999	+	**7**	**87.5**	NA	Intergenic	Intergenic	184758	NM_172501	212114	Mm.85744	NM_172501	ENSMUSG00000042997	**Nhlrc3**	4833441N19|8030451K01Rik|mKIAA4083	NHL repeat containing 3	Amp	protein-coding
2225_T12,T1,T22,T2,T32,T42,T4	chr3	5.3E+07	5.3E+07	3999	+	**7**	**87.5**	NA	Intergenic	MuRRS-int|LTR|ERV1	96758	NM_172501	212114	Mm.85744	NM_172501	ENSMUSG00000042997	**Nhlrc3**	4833441N19|8030451K01Rik|mKIAA4083	NHL repeat containing 3	Amp	protein-coding
2171_T12,T1,T22,T2,T32,T3,T4	chr3	5.1E+07	5.1E+07	5999	+	**7**	**87.5**	NA	Intergenic	Intergenic	-256947	NM_009834	12457	Mm.86541	NM_009834	ENSMUSG00000023087	**Noct**	AU043840|Ccr4|Ccrn4l	nocturnin	Amp	protein-coding
2175_T1,T22,T2,T32,T3,T42,T4	chr3	5.1E+07	5.1E+07	999	+	**7**	**87.5**	NA	Intergenic	B3|SINE|B2	-55447	NM_009834	12457	Mm.86541	NM_009834	ENSMUSG00000023087	**Noct**	AU043840|Ccr4|Ccrn4l	nocturnin	Amp	protein-coding
541_T12,T1,T22,T2,T32,T3,T4	chr13	7.9E+07	7.9E+07	999	+	**7**	**87.5**	NA	Intergenic	Intergenic	-976018	NM_010151	13865	Mm.439653	NM_010151	ENSMUSG00000069171	**Nr2f1**	COUP-TF1|COUP-TFI|COUPTFA|EAR-3|EAR3|Erbal3|SVP44|Tcfcoup1	nuclear receptor subfamily 2, group F, member 1	Amp	protein-coding

459_T12,T1,T22,T32,T3,T42,T4	chr12	8.9E+07	8.9E+07	999	+	**7**	**87.5**	NA	intron (NM_172544, intron 8 of 19)	intron (NM_172544, intron 8 of 19)	-405483	NM_001252074	18191	Mm.425766	NM_172544	ENSMUSG00000066392	**Nrxn3**	-	neurexin III	Amp	protein-coding
2883_T12,T1,T22,T2,T32,T3,T42	chr6	9372501	9373500	999	+	**7**	**87.5**	NA	Intergenic	Intergenic	422981	NM_008751	18231	Mm.46954	NM_008751	ENSMUSG00000046178	**Nxph1**	C130005L03Rik	neurexophilin 1	Amp	protein-coding
2888_T12,T1,T22,T2,T32,T3,T4	chr6	9901501	9905500	3999	+	**7**	**87.5**	NA	Intergenic	MERVL_2A-int|LTR|ERVL	953481	NM_008751	18231	Mm.46954	NM_008751	ENSMUSG00000046178	**Nxph1**	C130005L03Rik	neurexophilin 1	Amp	protein-coding
626_T12,T1,T22,T2,T3,T42,T4	chr14	8.1E+07	8.1E+07	999	+	**7**	**87.5**	NA	Intergenic	Intergenic	523698	NM_001030294	380924	Mm.26456	NM_001030294	ENSMUSG00000022026	**Olfm4**	GC1|GW112|Gm296|Gm913|OlfD|pPD4	olfactomedin 4	Amp	protein-coding
628_T12,T1,T22,T2,T32,T3,T42	chr14	8.1E+07	8.1E+07	999	+	**7**	**87.5**	NA	Intergenic	Intergenic	528698	NM_001030294	380924	Mm.26456	NM_001030294	ENSMUSG00000022026	**Olfm4**	GC1|GW112|Gm296|Gm913|OlfD|pPD4	olfactomedin 4	Amp	protein-coding
1905_T12,T22,T2,T32,T3,T42,T4	chr3	4.6E+07	4.6E+07	999	+	**7**	**87.5**	NA	Intergenic	Intergenic	451941	NM_001101479	241989	Mm.119234	NM_001101479	ENSMUSG00000090919	**Pabpc4l**	C330050A14Rik|EG241989	poly(A) binding protein, cytoplasmic 4-like	Amp	protein-coding
1908_T12,T1,T22,T2,T32,T3,T42	chr3	4.6E+07	4.6E+07	999	+	**7**	**87.5**	NA	Intergenic	L1_Mur3|LINE|L1	446941	NM_001101479	241989	Mm.119234	NM_001101479	ENSMUSG00000090919	**Pabpc4l**	C330050A14Rik|EG241989	poly(A) binding protein, cytoplasmic 4-like	Amp	protein-coding
1909_T12,T1,T22,T2,T32,T3,T4	chr3	4.6E+07	4.6E+07	999	+	**7**	**87.5**	NA	Intergenic	L1_Mur3|LINE|L1	445941	NM_001101479	241989	Mm.119234	NM_001101479	ENSMUSG00000090919	**Pabpc4l**	C330050A14Rik|EG241989	poly(A) binding protein, cytoplasmic 4-like	Amp	protein-coding
1915_T12,T1,T22,T2,T3,T42,T4	chr3	4.6E+07	4.6E+07	7999	+	**7**	**87.5**	NA	Intergenic	Intergenic	407441	NM_001101479	241989	Mm.119234	NM_001101479	ENSMUSG00000090919	**Pabpc4l**	C330050A14Rik|EG241989	poly(A) binding protein, cytoplasmic 4-like	Amp	protein-coding
1917_T12,T1,T22,T2,T32,T3,T42	chr3	4.6E+07	4.6E+07	999	+	**7**	**87.5**	NA	Intergenic	Intergenic	398941	NM_001101479	241989	Mm.119234	NM_001101479	ENSMUSG00000090919	**Pabpc4l**	C330050A14Rik|EG241989	poly(A) binding protein, cytoplasmic 4-like	Amp	protein-coding
1919_T1,T22,T2,T32,T3,T42,T4	chr3	4.6E+07	4.6E+07	999	+	**7**	**87.5**	NA	Intergenic	Lx6|LINE|L1	391941	NM_001101479	241989	Mm.119234	NM_001101479	ENSMUSG00000090919	**Pabpc4l**	C330050A14Rik|EG241989	poly(A) binding protein, cytoplasmic 4-like	Amp	protein-coding
1920_T12,T1,T22,T2,T32,T3,T42	chr3	4.6E+07	4.6E+07	999	+	**7**	**87.5**	NA	Intergenic	Lx7|LINE|L1	390941	NM_001101479	241989	Mm.119234	NM_001101479	ENSMUSG00000090919	**Pabpc4l**	C330050A14Rik|EG241989	poly(A) binding protein, cytoplasmic 4-like	Amp	protein-coding
1921_T12,T1,T22,T2,T3,T42,T4	chr3	4.6E+07	4.6E+07	3999	+	**7**	**87.5**	NA	Intergenic	L1_Mus3|LINE|L1	388441	NM_001101479	241989	Mm.119234	NM_001101479	ENSMUSG00000090919	**Pabpc4l**	C330050A14Rik|EG241989	poly(A) binding protein, cytoplasmic 4-like	Amp	protein-coding
1924_T12,T1,T22,T2,T32,T3,T42	chr3	4.6E+07	4.6E+07	3999	+	**7**	**87.5**	NA	Intergenic	Intergenic	368441	NM_001101479	241989	Mm.119234	NM_001101479	ENSMUSG00000090919	**Pabpc4l**	C330050A14Rik|EG241989	poly(A) binding protein, cytoplasmic 4-like	Amp	protein-coding
1926_T12,T1,T22,T2,T32,T3,T42	chr3	4.6E+07	4.6E+07	15999	+	**7**	**87.5**	NA	Intergenic	Lx9|LINE|L1	345441	NM_001101479	241989	Mm.119234	NM_001101479	ENSMUSG00000090919	**Pabpc4l**	C330050A14Rik|EG241989	poly(A) binding protein, cytoplasmic 4-like	Amp	protein-coding
1928_T12,T1,T22,T2,T32,T3,T42	chr3	4.6E+07	4.6E+07	999	+	**7**	**87.5**	NA	Intergenic	Intergenic	309941	NM_001101479	241989	Mm.119234	NM_001101479	ENSMUSG00000090919	**Pabpc4l**	C330050A14Rik|EG241989	poly(A) binding protein, cytoplasmic 4-like	Amp	protein-coding

1934_T12,T1,T22,T2,T3,T42,T4	chr3	4.6E+07	4.6E+07	999	+	**7**	**87.5**	NA	Intergenic	Intergenic	263941	NM_001101479	241989	Mm.119234	NM_001101479	ENSMUSG00000090919	**Pabpc4l**	C330050A14Rik|EG241989	poly(A) binding protein, cytoplasmic 4-like	Amp	protein-coding
1940_T12,T1,T22,T2,T32,T42,T4	chr3	4.6E+07	4.6E+07	2999	+	**7**	**87.5**	NA	Intergenic	Lx|LINE|L1	239941	NM_001101479	241989	Mm.119234	NM_001101479	ENSMUSG00000090919	**Pabpc4l**	C330050A14Rik|EG241989	poly(A) binding protein, cytoplasmic 4-like	Amp	protein-coding
1942_T12,T1,T22,T32,T3,T42,T4	chr3	4.6E+07	4.6E+07	12999	+	**7**	**87.5**	NA	Intergenic	RSINE1|SINE|B4	184941	NM_001101479	241989	Mm.119234	NM_001101479	ENSMUSG00000090919	**Pabpc4l**	C330050A14Rik|EG241989	poly(A) binding protein, cytoplasmic 4-like	Amp	protein-coding
1944_T12,T1,T22,T2,T3,T42,T4	chr3	4.7E+07	4.7E+07	999	+	**7**	**87.5**	NA	Intergenic	Intergenic	-142059	NM_001101479	241989	Mm.119234	NM_001101479	ENSMUSG00000090919	**Pabpc4l**	C330050A14Rik|EG241989	poly(A) binding protein, cytoplasmic 4-like	Amp	protein-coding
1949_T12,T22,T2,T32,T3,T42,T4	chr3	4.7E+07	4.7E+07	8999	+	**7**	**87.5**	NA	Intergenic	RLTR17|LTR|ERVK	-189059	NM_001101479	241989	Mm.119234	NM_001101479	ENSMUSG00000090919	**Pabpc4l**	C330050A14Rik|EG241989	poly(A) binding protein, cytoplasmic 4-like	Amp	protein-coding
1957_T12,T22,T2,T32,T3,T42,T4	chr3	4.7E+07	4.7E+07	999	+	**7**	**87.5**	NA	Intergenic	Intergenic	-216059	NM_001101479	241989	Mm.119234	NM_001101479	ENSMUSG00000090919	**Pabpc4l**	C330050A14Rik|EG241989	poly(A) binding protein, cytoplasmic 4-like	Amp	protein-coding
664_T12,T1,T22,T2,T32,T42,T4	chr14	8.4E+07	8.4E+07	1999	+	**7**	**87.5**	NA	non-coding (NR_015539, exon 6 of 6)	non-coding (NR_015539, exon 6 of 6)	15937	NM_001013753	219228	Mm.153643	NM_001013753	ENSMUSG00000035566	**Pcdh17**	C030033F14Rik|Gm78	protocadherin 17	Amp	protein-coding
643_T12,T1,T22,T2,T32,T42,T4	chr14	8.3E+07	8.3E+07	1999	+	**7**	**87.5**	NA	Intergenic	Intergenic	-1E+06	NM_001013753	219228	Mm.153643	NM_001013753	ENSMUSG00000035566	**Pcdh17**	C030033F14Rik|Gm78	protocadherin 17	Amp	protein-coding
645_T12,T1,T22,T2,T3,T42,T4	chr14	8.3E+07	8.3E+07	999	+	**7**	**87.5**	NA	Intergenic	Intergenic	-1E+06	NM_001013753	219228	Mm.153643	NM_001013753	ENSMUSG00000035566	**Pcdh17**	C030033F14Rik|Gm78	protocadherin 17	Amp	protein-coding
659_T12,T1,T22,T2,T32,T3,T4	chr14	8.4E+07	8.4E+07	4999	+	**7**	**87.5**	NA	Intergenic	L1_Mur2|LINE|L1	-700563	NM_001013753	219228	Mm.153643	NM_001013753	ENSMUSG00000035566	**Pcdh17**	C030033F14Rik|Gm78	protocadherin 17	Amp	protein-coding
2122_T12,T1,T2,T32,T3,T42,T4	chr3	4.9E+07	4.9E+07	6999	+	**7**	**87.5**	NA	Intergenic	RLTR20A3_MM|LTR|ERVK	303316	NM_130448	73173	Mm.87246	NM_130448	ENSMUSG00000037892	**Pcdh18**	3110038E07Rik|BB095589|PCDH68L	protocadherin 18	Amp	protein-coding
2124_T12,T1,T2,T32,T3,T42,T4	chr3	4.9E+07	4.9E+07	8999	+	**7**	**87.5**	NA	Intergenic	Lx6|LINE|L1	287316	NM_130448	73173	Mm.87246	NM_130448	ENSMUSG00000037892	**Pcdh18**	3110038E07Rik|BB095589|PCDH68L	protocadherin 18	Amp	protein-coding
2127_T1,T22,T2,T32,T3,T42,T4	chr3	5E+07	5E+07	999	+	**7**	**87.5**	NA	Intergenic	ERV3-16A3_I-int|LTR|ERVL	84316	NM_130448	73173	Mm.87246	NM_130448	ENSMUSG00000037892	**Pcdh18**	3110038E07Rik|BB095589|PCDH68L	protocadherin 18	Amp	protein-coding
2130_T12,T1,T22,T2,T32,T3,T4	chr3	5E+07	5E+07	999	+	**7**	**87.5**	NA	Intergenic	Intergenic	78316	NM_130448	73173	Mm.87246	NM_130448	ENSMUSG00000037892	**Pcdh18**	3110038E07Rik|BB095589|PCDH68L	protocadherin 18	Amp	protein-coding
2145_T1,T22,T2,T32,T3,T42,T4	chr3	5E+07	5E+07	999	+	**7**	**87.5**	NA	Intergenic	Lx5c|LINE|L1	-295684	NM_130448	73173	Mm.87246	NM_130448	ENSMUSG00000037892	**Pcdh18**	3110038E07Rik|BB095589|PCDH68L	protocadherin 18	Amp	protein-coding
727_T12,T1,T22,T2,T32,T3,T4	chr14	9.2E+07	9.2E+07	999	+	**7**	**87.5**	NA	Intergenic	Intergenic	1584888	NM_001271800	211712	Mm.37126	NM_001081377	ENSMUSG00000055421	**Pcdh9**	-	protocadherin 9	Amp	protein-coding

2389_T12,T1,T22,T2,T32,T3,T4	chr3	7.5E+07	7.5E+07	3999	+	**7**	**87.5**	NA	Intergenic	Intergenic	126352	NM_019745	56426	Mm.316473	NM_019745	ENSMUSG00000027835	**Pdcd10**	2410003B13Rik|Ccm3|Tfa15|Tfar15	programmed cell death 10	Amp	protein-coding
2328_T1,T22,T2,T32,T3,T42,T4	chr3	5.8E+07	5.8E+07	2999	+	**7**	**87.5**	NA	Intergenic	L1M2|LINE|L1	-80243	NM_019410	18645	Mm.271744	NM_019410	ENSMUSG00000027805	**Pfn2**	Pfn	profilin 2	Amp	protein-coding
981_T12,T1,T22,T32,T3,T42,T4	chr15	4.5E+07	4.5E+07	999	+	**7**	**87.5**	NA	intron (NM_138674, intron 23 of 76)	RSINE1|SINE|B4	48447	NM_138674	192190	Mm.211494	NM_138674	ENSMUSG00000038725	**Pkhd1l1**	AB055648|PKHDL1	polycystic kidney and hepatic disease 1-like 1	Amp	protein-coding
1441_T12,T1,T22,T2,T3,T42,T4	chr2	4.1E+07	4.1E+07	3999	+	**7**	**87.5**	NA	exon (NM_053011, exon 83 of 91)	exon (NM_053011, exon 83 of 91)	-1E+06	NM_024209	67857	Mm.389242	NM_024209	ENSMUSG00000026753	**Ppp6c**	2310003C10Rik	protein phosphatase 6, catalytic subunit	Amp	protein-coding
2184_T1,T22,T2,T32,T3,T42,T4	chr3	5.1E+07	5.1E+07	999	+	**7**	**87.5**	NA	intron (NM_053089, intron 10 of 19)	PB1D11|SINE|Alu	-31966	NM_016858	19338	Mm.1664	NM_016858	ENSMUSG00000027739	**Rab33b**	-	RAB33B, member RAS oncogene family	Amp	protein-coding
167_T12,T1,T22,T2,T3,T42,T4	chr1	1.5E+08	1.5E+08	999	+	**7**	**87.5**	NA	Intergenic	Intergenic	-366579	NM_022881	64214	Mm.253927	NM_022881	ENSMUSG00000026357	**Rgs18**	-	regulator of G-protein signaling 18	Amp	protein-coding
170_T12,T1,T2,T32,T3,T42,T4	chr1	1.5E+08	1.5E+08	3999	+	**7**	**87.5**	NA	Intergenic	Intergenic	-551079	NM_022881	64214	Mm.253927	NM_022881	ENSMUSG00000026357	**Rgs18**	-	regulator of G-protein signaling 18	Amp	protein-coding
173_T12,T1,T22,T2,T32,T3,T4	chr1	1.5E+08	1.5E+08	4999	+	**7**	**87.5**	NA	Intergenic	Intergenic	-588579	NM_022881	64214	Mm.253927	NM_022881	ENSMUSG00000026357	**Rgs18**	-	regulator of G-protein signaling 18	Amp	protein-coding
224_T12,T1,T22,T2,T32,T3,T4	chr10	3.2E+07	3.2E+07	2999	+	**7**	**87.5**	NA	intron (NM_001013411, intron 3 of 6)	L1MC1|LINE|L1	-354275	NM_001146349	268291	Mm.295212	NM_001146349	ENSMUSG00000063760	**Rnf217**	AU016819|Ibrdc1	ring finger protein 217	Amp	protein-coding
1509_T12,T1,T22,T32,T3,T42,T4	chr2	6.7E+07	6.7E+07	999	+	**7**	**87.5**	NA	Intergenic	Intergenic	-31038	NM_001290675	20274	Mm.440889	NM_018852	ENSMUSG00000075316	**Scn9a**	Nav1.7|PN1|mKIAA4197	sodium channel, voltage-gated, type IX, alpha	Amp	protein-coding
968_T12,T1,T22,T2,T3,T42,T4	chr15	3.3E+07	3.3E+07	999	+	**7**	**87.5**	NA	Intergenic	Intergenic	-29723	NM_008304	15529	Mm.234266	NM_008304	ENSMUSG00000022261	**Sdc2**	4833414L08Rik|AA960457|Hspg1|Synd2|syndecan-2	syndecan 2	Amp	protein-coding
2706_T1,T22,T2,T32,T3,T42,T4	chr5	1.2E+07	1.2E+07	999	+	**7**	**87.5**	NA	intron (NM_028882, intron 2 of 17)	intron (NM_028882, intron 2 of 17)	76834	NM_028882	108151	Mm.89313	NM_028882	ENSMUSG00000040254	**Sema3d**	4631426B19Rik	sema domain, immunoglobulin domain (Ig), short basic domain, secreted, (semaphorin) 3D	Amp	protein-coding
2233_T12,T1,T2,T32,T3,T42,T4	chr3	5.5E+07	5.5E+07	999	+	**7**	**87.5**	NA	Intergenic	Intergenic	40887	NM_177854	329641	Mm.51187	NM_177854	ENSMUSG00000056306	**Sertm1**	6030405A18Rik	serine rich and transmembrane domain containing 1	Amp	protein-coding

3477_T12,T1,T22,T2,T32,T42,T4	chr8	3.9E+07	3.9E+07	999	+	**7**	**87.5**	NA	intron (NM_145841, intron 1 of 7)	intron (NM_145841, intron 1 of 7)	107508	NM_145841	244431	Mm.210388	NM_145841	ENSMUSG00000039539	**Sgcz**	C230085N17Rik	sarcoglycan zeta	Amp	protein-coding
3479_T12,T1,T22,T2,T32,T3,T4	chr8	3.9E+07	3.9E+07	999	+	**7**	**87.5**	NA	intron (NM_145841, intron 1 of 7)	MER2|DNA|TcMar-Tigger	102508	NM_145841	244431	Mm.210388	NM_145841	ENSMUSG00000039539	**Sgcz**	C230085N17Rik	sarcoglycan zeta	Amp	protein-coding
3404_T12,T1,T22,T2,T3,T42,T4	chr8	6396501	6397500	999	+	**7**	**87.5**	NA	Intergenic	Intergenic	-1E+06	NM_011388	20494	Mm.3500	NM_011388	ENSMUSG00000023073	**Slc10a2**	9130221J18Rik|AI605518|ASBT|IBAT|ISBT	solute carrier family 10, member 2	Amp	protein-coding
3408_T12,T1,T2,T32,T3,T42,T4	chr8	6520501	6522500	1999	+	**7**	**87.5**	NA	Intergenic	Intergenic	-1E+06	NM_011388	20494	Mm.3500	NM_011388	ENSMUSG00000023073	**Slc10a2**	9130221J18Rik|AI605518|ASBT|IBAT|ISBT	solute carrier family 10, member 2	Amp	protein-coding
3413_T12,T22,T2,T32,T3,T42,T4	chr8	6646501	6647500	999	+	**7**	**87.5**	NA	Intergenic	RMER6D|LTR|ERVK	-2E+06	NM_011388	20494	Mm.3500	NM_011388	ENSMUSG00000023073	**Slc10a2**	9130221J18Rik|AI605518|ASBT|IBAT|ISBT	solute carrier family 10, member 2	Amp	protein-coding
3416_T12,T1,T22,T2,T32,T3,T42	chr8	6831501	6835500	3999	+	**7**	**87.5**	NA	Intergenic	Intergenic	-2E+06	NM_011388	20494	Mm.3500	NM_011388	ENSMUSG00000023073	**Slc10a2**	9130221J18Rik|AI605518|ASBT|IBAT|ISBT	solute carrier family 10, member 2	Amp	protein-coding
2152_T12,T1,T22,T2,T32,T3,T4	chr3	5E+07	5E+07	999	+	**7**	**87.5**	NA	Intergenic	(TTTC)n|Simple_repeat|Simple_repeat	256613	NM_011990	26570	Mm.260988	NM_011990	ENSMUSG00000027737	**Slc7a11**	9930009M05Rik|AI451155|sut|xCT	solute carrier family 7 (cationic amino acid transporter, y+ system), member 11	Amp	protein-coding
2154_T12,T1,T22,T2,T32,T3,T4	chr3	5E+07	5E+07	999	+	**7**	**87.5**	NA	Intergenic	L1_Mus3|LINE|L1	251613	NM_011990	26570	Mm.260988	NM_011990	ENSMUSG00000027737	**Slc7a11**	9930009M05Rik|AI451155|sut|xCT	solute carrier family 7 (cationic amino acid transporter, y+ system), member 11	Amp	protein-coding
2158_T12,T1,T22,T2,T32,T3,T4	chr3	5E+07	5E+07	5999	+	**7**	**87.5**	NA	Intergenic	Intergenic	-33887	NM_011990	26570	Mm.260988	NM_011990	ENSMUSG00000027737	**Slc7a11**	9930009M05Rik|AI451155|sut|xCT	solute carrier family 7 (cationic amino acid transporter, y+ system), member 11	Amp	protein-coding
2168_T12,T1,T22,T2,T32,T3,T42	chr3	5.1E+07	5.1E+07	999	+	**7**	**87.5**	NA	Intergenic	Intergenic	-260387	NM_011990	26570	Mm.260988	NM_011990	ENSMUSG00000027737	**Slc7a11**	9930009M05Rik|AI451155|sut|xCT	solute carrier family 7 (cationic amino acid transporter, y+ system), member 11	Amp	protein-coding
2755_T12,T1,T22,T2,T3,T42,T4	chr5	4.8E+07	4.8E+07	3999	+	**7**	**87.5**	NA	Intergenic	RMER6C|LTR|ERVK	-385655	NR_111900	20563	Mm.289739	NM_178804	ENSMUSG00000031558	**Slit2**	Drad-1|E030015M03Rik|E130320P19Rik|Slil3|mKIAA4141|slit-2	slit homolog 2 (Drosophila)	Amp	protein-coding
815_T12,T1,T22,T2,T32,T3,T4	chr14	1.1E+08	1.1E+08	999	+	**7**	**87.5**	NA	Intergenic	Intergenic	24239	NM_199065	76965	Mm.257268	NM_199065	ENSMUSG00000075478	**Slitrk1**	3200001I04Rik	SLIT and NTRK-like family, member 1	Amp	protein-coding

817_T12,T1,T22,T2,T32,T3,T42	chr14	1.1E+08	1.1E+08	1999	+	**7**	**87.5**	NA	Intergenic	Intergenic	19739	NM_199065	76965	Mm.257268	NM_199065	ENSMUSG00000075478	**Slitrk1**	3200001I04Rik	SLIT and NTRK-like family, member 1	Amp	protein-coding
1359_T12,T1,T2,T32,T3,T42,T4	chr19	5.1E+07	5.1E+07	999	+	**7**	**87.5**	NA	Intergenic	Intergenic	-270354	NM_001290356	58178	Mm.313672	NM_021377	ENSMUSG00000043531	**Sorcs1**	Sorcs|mSorCS	sortilin-related VPS10 domain containing receptor 1	Amp	protein-coding
454_T12,T1,T22,T2,T32,T42,T4	chr12	6.3E+07	6.3E+07	4999	+	**7**	**87.5**	NA	Intergenic	Intergenic	408213	NR_131949	73309	Mm.276558	NR_131949		**Spanxn4**	1700047L15Rik	SPANX family, member N4	Amp	ncRNA
3573_T12,T1,T2,T32,T3,T42,T4	chr8	6.3E+07	6.3E+07	3999	+	**7**	**87.5**	NA	intron (NM_023689, intron 3 of 11)	intron (NM_023689, intron 3 of 11)	146155	NM_023689	72902	Mm.334552	NM_023689	ENSMUSG00000054162	**Spock3**	2900045C01Rik|AI428471|mKIAA4039	sparc/osteonectin, cwcv and kazal-like domains proteoglycan 3	Amp	protein-coding
3783_T12,T1,T22,T2,T32,T3,T4	chr9	1.1E+08	1.1E+08	5999	+	**7**	**87.5**	NA	Intergenic	Intergenic	119146	NM_001101510	382111	Mm.376140	NM_001101510	ENSMUSG00000086596	**Susd5**	Gm1126	sushi domain containing 5	Amp	protein-coding
2932_T1,T22,T2,T32,T3,T42,T4	chr6	1.7E+07	1.7E+07	999	+	**7**	**87.5**	NA	intron (NM_207176, intron 1 of 6)	intron (NM_207176, intron 1 of 6)	9851	NM_207176	21753	Mm.436548	NM_011570	ENSMUSG00000029552	**Tes**	D6Ertd352e|TESS|Tes1|Tes2|testin2	testis derived transcript	Amp	protein-coding
2924_T12,T1,T22,T2,T32,T3,T42	chr6	1.7E+07	1.7E+07	999	+	**7**	**87.5**	NA	Intergenic	Intergenic	-10559	NM_031198	21426	Mm.36217	NM_031198	ENSMUSG00000029553	**Tfec**	BB107417|Tcfec|bHLHe34	transcription factor EC	Amp	protein-coding
2920_T12,T1,T22,T32,T3,T42,T4	chr6	1.7E+07	1.7E+07	2999	+	**7**	**87.5**	NA	intron (NM_031198, intron 1 of 6)	intron (NM_031198, intron 1 of 6)	8441	NM_031198	21426	Mm.36217	NM_031198	ENSMUSG00000029553	**Tfec**	BB107417|Tcfec|bHLHe34	transcription factor EC	Amp	protein-coding
2922_T12,T1,T22,T2,T3,T42,T4	chr6	1.7E+07	1.7E+07	999	+	**7**	**87.5**	NA	intron (NM_031198, intron 1 of 6)	L1Md_F2|LINE|L1	5441	NM_031198	21426	Mm.36217	NM_031198	ENSMUSG00000029553	**Tfec**	BB107417|Tcfec|bHLHe34	transcription factor EC	Amp	protein-coding
2291_T12,T22,T2,T32,T3,T42,T4	chr3	5.7E+07	5.7E+07	999	+	**7**	**87.5**	NA	Intergenic	Intergenic	359919	NM_008536	17112	Mm.856	NM_008536	ENSMUSG00000027800	**Tm4sf1**	L6|M3s1	transmembrane 4 superfamily member 1	Amp	protein-coding
2295_T12,T1,T2,T32,T3,T42,T4	chr3	5.7E+07	5.7E+07	2999	+	**7**	**87.5**	NA	Intergenic	Lx7|LINE|L1	321919	NM_008536	17112	Mm.856	NM_008536	ENSMUSG00000027800	**Tm4sf1**	L6|M3s1	transmembrane 4 superfamily member 1	Amp	protein-coding
2297_T12,T22,T2,T32,T3,T42,T4	chr3	5.7E+07	5.7E+07	999	+	**7**	**87.5**	NA	Intergenic	RMER17A|LTR|ERVK	249919	NM_008536	17112	Mm.856	NM_008536	ENSMUSG00000027800	**Tm4sf1**	L6|M3s1	transmembrane 4 superfamily member 1	Amp	protein-coding
2299_T12,T22,T2,T32,T3,T42,T4	chr3	5.7E+07	5.7E+07	1999	+	**7**	**87.5**	NA	Intergenic	ORR1D2|LTR|ERVL-MaLR	243419	NM_008536	17112	Mm.856	NM_008536	ENSMUSG00000027800	**Tm4sf1**	L6|M3s1	transmembrane 4 superfamily member 1	Amp	protein-coding
2301_T12,T1,T2,T32,T3,T42,T4	chr3	5.7E+07	5.7E+07	999	+	**7**	**87.5**	NA	Intergenic	Intergenic	230919	NM_008536	17112	Mm.856	NM_008536	ENSMUSG00000027800	**Tm4sf1**	L6|M3s1	transmembrane 4 superfamily member 1	Amp	protein-coding
2303_T12,T22,T2,T32,T3,T42,T4	chr3	5.7E+07	5.7E+07	4999	+	**7**	**87.5**	NA	Intergenic	Intergenic	215919	NM_008536	17112	Mm.856	NM_008536	ENSMUSG00000027800	**Tm4sf1**	L6|M3s1	transmembrane 4 superfamily member 1	Amp	protein-coding

2305_T12,T1,T22,T2,T3,T42,T4	chr3	5.7E+07	5.7E+07	999	+	**7**	**87.5**	NA	Intergenic	RLTR17B_Mm|LTR|ERVK	112919	NM_008536	17112	Mm.856	NM_008536	ENSMUSG00000027800	**Tm4sf1**	L6|M3s1	transmembrane 4 superfamily member 1	Amp	protein-coding
2307_T12,T1,T22,T2,T3,T42,T4	chr3	5.7E+07	5.7E+07	999	+	**7**	**87.5**	NA	Intergenic	L1_Mus2|LINE|L1	88919	NM_008536	17112	Mm.856	NM_008536	ENSMUSG00000027800	**Tm4sf1**	L6|M3s1	transmembrane 4 superfamily member 1	Amp	protein-coding
2309_T12,T1,T22,T2,T3,T42,T4	chr3	5.7E+07	5.7E+07	3999	+	**7**	**87.5**	NA	Intergenic	IAPEz-int|LTR|ERVK	63419	NM_008536	17112	Mm.856	NM_008536	ENSMUSG00000027800	**Tm4sf1**	L6|M3s1	transmembrane 4 superfamily member 1	Amp	protein-coding
2313_T1,T22,T2,T32,T3,T42,T4	chr3	5.7E+07	5.7E+07	999	+	**7**	**87.5**	NA	Intergenic	Intergenic	-19081	NM_008536	17112	Mm.856	NM_008536	ENSMUSG00000027800	**Tm4sf1**	L6|M3s1	transmembrane 4 superfamily member 1	Amp	protein-coding
2319_T12,T22,T2,T32,T3,T42,T4	chr3	5.7E+07	5.7E+07	1999	+	**7**	**87.5**	NA	Intergenic	B1_Mus2|SINE|Alu	-54910	NM_145539	229302	Mm.26618	NM_145539	ENSMUSG00000027801	**Tm4sf4**	Iltmp	transmembrane 4 superfamily member 4	Amp	protein-coding
2322_T12,T22,T2,T32,T3,T42,T4	chr3	5.7E+07	5.7E+07	1999	+	**7**	**87.5**	NA	Intergenic	Intergenic	-38910	NM_145539	229302	Mm.26618	NM_145539	ENSMUSG00000027801	**Tm4sf4**	Iltmp	transmembrane 4 superfamily member 4	Amp	protein-coding
482_T12,T1,T22,T2,T3,T42,T4	chr12	1.2E+08	1.2E+08	999	+	**7**	**87.5**	NA	intron (NM_001294146, intron 1 of 3)	intron (NM_001294146, intron 1 of 3)	60038	NM_001160385	217951	Mm.387395	NM_001160385	ENSMUSG00000048004	**Tmem196**	Gm528	transmembrane protein 196	Amp	protein-coding
613_T12,T1,T2,T32,T3,T42,T4	chr14	6.1E+07	6.1E+07	4999	+	**7**	**87.5**	NA	Intergenic	Intergenic	99987	NM_001164155	29820	Mm.281356	NM_013869	ENSMUSG00000060548	**Tnfrsf19**	AL023044|AW123854|TAJ|TAJ-ALPHA|TRADE|Troy	tumor necrosis factor receptor superfamily, member 19	Amp	protein-coding
1045_T12,T22,T2,T32,T3,T42,T4	chr15	5E+07	5E+07	3999	+	**7**	**87.5**	NA	Intergenic	Intergenic	718549	NM_001310481	83925	Mm.30466	NM_032000	ENSMUSG00000038679	**Trps1**	AI115454|AI447310|D15Ertd586e	trichorhinophalangeal syndrome I (human)	Amp	protein-coding
1048_T12,T1,T22,T2,T3,T42,T4	chr15	5E+07	5E+07	999	+	**7**	**87.5**	NA	Intergenic	L1MD3|LINE|L1	551049	NM_001310481	83925	Mm.30466	NM_032000	ENSMUSG00000038679	**Trps1**	AI115454|AI447310|D15Ertd586e	trichorhinophalangeal syndrome I (human)	Amp	protein-coding
1050_T12,T1,T2,T32,T3,T42,T4	chr15	5E+07	5E+07	999	+	**7**	**87.5**	NA	Intergenic	Lx7|LINE|L1	547049	NM_001310481	83925	Mm.30466	NM_032000	ENSMUSG00000038679	**Trps1**	AI115454|AI447310|D15Ertd586e	trichorhinophalangeal syndrome I (human)	Amp	protein-coding
1056_T12,T1,T2,T32,T3,T42,T4	chr15	5.1E+07	5.1E+07	3999	+	**7**	**87.5**	NA	intron (NM_001310481, intron 4 of 5)	intron (NM_001310481, intron 4 of 5)	162549	NM_001310481	83925	Mm.30466	NM_032000	ENSMUSG00000038679	**Trps1**	AI115454|AI447310|D15Ertd586e	trichorhinophalangeal syndrome I (human)	Amp	protein-coding
1061_T12,T1,T22,T2,T32,T3,T4	chr15	5.1E+07	5.1E+07	3999	+	**7**	**87.5**	NA	intron (NM_001310485, intron 5 of 7)	intron (NM_001310485, intron 5 of 7)	77549	NM_001310481	83925	Mm.30466	NM_032000	ENSMUSG00000038679	**Trps1**	AI115454|AI447310|D15Ertd586e	trichorhinophalangeal syndrome I (human)	Amp	protein-coding
2337_T12,T1,T22,T2,T32,T42,T4	chr3	5.8E+07	5.8E+07	999	+	**7**	**87.5**	NA	exon (NM_001081229, exon 1 of 4)	exon (NM_001081229, exon 1 of 4)	311	NM_001081229	72033	Mm.218409	NM_001081229	ENSMUSG00000027806	**Tsc22d2**	1810043J12Rik|5530402M19Rik	TSC22 domain family, member 2	Amp	protein-coding

2335_T12,T1,T2,T32,T3,T42,T4	chr3	5.8E+07	5.8E+07	10999	+	**7**	**87.5**	NA	Intergenic	MTEa|LTR|ERVL-MaLR	-6689	NM_001081229	72033	Mm.218409	NM_001081229	ENSMUSG00000027806	**Tsc22d2**	1810043J12Rik|5530402M19Rik	TSC22 domain family, member 2	Amp	protein-coding
2339_T12,T1,T22,T2,T32,T3,T42	chr3	5.8E+07	5.8E+07	999	+	**7**	**87.5**	NA	intron (NM_001081229, intron 1 of 3)	intron (NM_001081229, intron 1 of 3)	2311	NM_001081229	72033	Mm.218409	NM_001081229	ENSMUSG00000027806	**Tsc22d2**	1810043J12Rik|5530402M19Rik	TSC22 domain family, member 2	Amp	protein-coding
2340_T12,T1,T22,T32,T3,T42,T4	chr3	5.8E+07	5.8E+07	999	+	**7**	**87.5**	NA	intron (NM_001081229, intron 1 of 3)	intron (NM_001081229, intron 1 of 3)	3311	NM_001081229	72033	Mm.218409	NM_001081229	ENSMUSG00000027806	**Tsc22d2**	1810043J12Rik|5530402M19Rik	TSC22 domain family, member 2	Amp	protein-coding
2344_T12,T1,T22,T2,T32,T3,T42	chr3	5.8E+07	5.8E+07	999	+	**7**	**87.5**	NA	intron (NM_001081229, intron 1 of 3)	B3|SINE|B2	11311	NM_001081229	72033	Mm.218409	NM_001081229	ENSMUSG00000027806	**Tsc22d2**	1810043J12Rik|5530402M19Rik	TSC22 domain family, member 2	Amp	protein-coding
605_T12,T1,T22,T2,T3,T42,T4	chr14	1.9E+07	1.9E+07	999	+	**7**	**87.5**	NA	Intergenic	Intergenic	-156373	NM_144839	218793	Mm.23551	NM_144839	ENSMUSG00000058317	**Ube2e2**	BC016265	ubiquitin-conjugating enzyme E2E 2	Amp	protein-coding
3435_T12,T1,T22,T2,T3,T42,T4	chr8	3E+07	3E+07	3999	+	**7**	**87.5**	NA	Intergenic	Intergenic	-481864	NM_153135	210801	Mm.248433	NM_153135	ENSMUSG00000063626	**Unc5d**	D930029E11Rik|Unc5h4|mKIAA1777	unc-5 netrin receptor D	Amp	protein-coding
3440_T12,T1,T22,T2,T3,T42,T4	chr8	3E+07	3E+07	3999	+	**7**	**87.5**	NA	Intergenic	RMER19B|LTR|ERVK	-519864	NM_153135	210801	Mm.248433	NM_153135	ENSMUSG00000063626	**Unc5d**	D930029E11Rik|Unc5h4|mKIAA1777	unc-5 netrin receptor D	Amp	protein-coding
1703_T12,T1,T22,T2,T32,T42,T4	chr3	1.7E+07	1.7E+07	999	+	**7**	**87.5**	NA	Intergenic	Lx5|LINE|L1	409817	NM_001145919	229096	Mm.23834	NM_172677	ENSMUSG00000047213	**Ythdf3**	9130022A11Rik	YTH domain family 3	Amp	protein-coding
1661_T12,T1,T22,T32,T3,T42,T4	chr3	7535501	7539500	3999	+	**7**	**87.5**	NA	intron (NM_173181, intron 7 of 8)	intron (NM_173181, intron 7 of 8)	34074	NM_173181	67306	Mm.332366	NM_173181	ENSMUSG00000043542	**Zc2hc1a**	3110050N22Rik|AI790358|AU023959|Fam164a	zinc finger, C2HC-type containing 1A	Amp	protein-coding
584_T12,T1,T32,T3,T42,T4	chr13	1E+08	1E+08	4999	+	**6**	**75**	NA	intron (NM_001081020, intron 14 of 24)	intron (NM_001081020, intron 14 of 24)	116127	NM_001081020	108154	Mm.208125	NM_175496	ENSMUSG00000046169	**Adamts6**	A930019D11Rik|ADAM-TS6|b2b1879.1Clo|b2b2029Clo|b2b2182Clo|b2b2187.1Clo|b2b2228Clo	a disintegrin-like and metallopeptidase (reprolysin type) with thrombospondin type 1 motif, 6	Amp	protein-coding
1322_T12,T22,T2,T32,T42,T4	chr17	9.4E+07	9.4E+07	3999	+	**6**	**75**	NA	Intergenic	Intergenic	388332	NM_009625	11516	Mm.3407	NM_009625	ENSMUSG00000024256	**Adcyap1**	PACAP	adenylate cyclase activating polypeptide 1	Amp	protein-coding
1327_T12,T22,T32,T3,T42,T4	chr17	9.4E+07	9.4E+07	6999	+	**6**	**75**	NA	Intergenic	Intergenic	580832	NM_009625	11516	Mm.3407	NM_009625	ENSMUSG00000024256	**Adcyap1**	PACAP	adenylate cyclase activating polypeptide 1	Amp	protein-coding
2766_T12,T1,T22,T32,T3,T4	chr5	5E+07	5E+07	3999	+	**6**	**75**	NA	Intergenic	Intergenic	379496	NM_133911	70693	Mm.272974	NM_133911	ENSMUSG00000029090	**Adgra3**	3830613O22Rik|AU044632|Gpr125|Tem5-like	adhesion G protein-coupled receptor A3	Amp	protein-coding

2864_T1,T22,T2,T32,T3,T42	chr5	8.2E+07	8.2E+07	3999	+	**6**	**75**	NA	Intergenic	Intergenic	863907	NM_198702	319387	Mm.273631	NM_198702	ENSMUSG00000037605	**Adgrl3**	5430402I23Rik|CIRL-3|D130075K09Rik|Gm1379|LEC3|Lphn3|mKIAA0768	adhesion G protein-coupled receptor L3	Amp	protein-coding
2984_T12,T22,T2,T3,T42,T4	chr6	1.9E+07	1.9E+07	999	+	**6**	**75**	NA	Intergenic	Intergenic	60682	NM_001167757	75196	Mm.67665	NM_029202	ENSMUSG00000029517	**Ankrd7**	4930532L20Rik|AV278427	ankyrin repeat domain 7	Amp	protein-coding
2986_T12,T22,T2,T3,T42,T4	chr6	1.9E+07	1.9E+07	999	+	**6**	**75**	NA	Intergenic	ORR1B2|LTR|ERVL-MaLR	82682	NM_001167757	75196	Mm.67665	NM_029202	ENSMUSG00000029517	**Ankrd7**	4930532L20Rik|AV278427	ankyrin repeat domain 7	Amp	protein-coding
2999_T12,T1,T2,T3,T42,T4	chr6	1.9E+07	1.9E+07	999	+	**6**	**75**	NA	Intergenic	Lx5c|LINE|L1	191682	NM_001167757	75196	Mm.67665	NM_029202	ENSMUSG00000029517	**Ankrd7**	4930532L20Rik|AV278427	ankyrin repeat domain 7	Amp	protein-coding
2324_T12,T1,T32,T3,T42,T4	chr3	5.8E+07	5.8E+07	999	+	**6**	**75**	NA	intron (NM_001033349, intron 2 of 3)	Lx6|LINE|L1	11537	NM_001033349	242037	Mm.291091	NM_001033349	ENSMUSG00000074591	**Ankub1**	Gm410	ankrin repeat and ubiquitin domain containing 1	Amp	protein-coding
2825_T12,T22,T2,T32,T3,T42	chr5	6.2E+07	6.2E+07	3999	+	**6**	**75**	NA	Intergenic	Intergenic	415677	NM_178407	212285	Mm.244403	NM_178407	ENSMUSG00000037999	**Arap2**	Centd1|Gm148|mKIAA0580	ArfGAP with RhoGAP domain, ankyrin repeat and PH domain 2	Amp	protein-coding
442_T12,T1,T32,T3,T42,T4	chr12	5.3E+07	5.3E+07	3999	+	**6**	**75**	NA	exon (NM_009706, exon 1 of 6)	exon (NM_009706, exon 1 of 6)	2423	NM_009706	11855	Mm.35059	NM_009706	ENSMUSG00000035133	**Arhgap5**	p190-B|p190B	Rho GTPase activating protein 5	Amp	protein-coding
546_T1,T22,T2,T3,T42,T4	chr13	8E+07	8E+07	4999	+	**6**	**75**	NA	Intergenic	Intergenic	-478422	NM_001042591	105171	Mm.423137	NM_001042591	ENSMUSG00000074794	**Arrdc3**	AI450344|mKIAA1376	arrestin domain containing 3	Amp	protein-coding
160_T12,T22,T2,T3,T42,T4	chr1	1.4E+08	1.4E+08	4999	+	**6**	**75**	NA	Intergenic	RMER5|LTR|ERV1	-1E+06	NM_020025	26878	Mm.285580	NM_020025	ENSMUSG00000033849	**B3galt2**	-	UDP-Gal:betaGlcNAc beta 1,3-galactosyltransferase, polypeptide 2	Amp	protein-coding
162_T12,T1,T2,T3,T42,T4	chr1	1.4E+08	1.4E+08	3999	+	**6**	**75**	NA	Intergenic	Intergenic	-1E+06	NM_020025	26878	Mm.285580	NM_020025	ENSMUSG00000033849	**B3galt2**	-	UDP-Gal:betaGlcNAc beta 1,3-galactosyltransferase, polypeptide 2	Amp	protein-coding
188_T1,T22,T2,T3,T42,T4	chr1	1.5E+08	1.5E+08	3999	+	**6**	**75**	NA	Intergenic	Intergenic	619813	NM_001145807	215378	Mm.441817	NM_153539	ENSMUSG00000035131	**Brinp3**	B830045N13Rik|Fam5c	bone morphogenetic protein/retinoic acid inducible neural specific 3	Amp	protein-coding
184_T12,T1,T22,T2,T32,T3	chr1	1.5E+08	1.5E+08	999	+	**6**	**75**	NA	intron (NM_001145807, intron 7 of 7)	intron (NM_001145807, intron 7 of 7)	351313	NM_001145807	215378	Mm.441817	NM_153539	ENSMUSG00000035131	**Brinp3**	B830045N13Rik|Fam5c	bone morphogenetic protein/retinoic acid inducible neural specific 3	Amp	protein-coding

2935_T12,T22,T2,T32,T3,T42	chr6	1.8E+07	1.8E+07	999	+	**6**	**75**	NA	Intergenic	ORR1A2-int|LTR|ERVL-MaLR	-13098	NM_007604	12343	Mm.392504	NM_007604	ENSMUSG00000015733	**Capza2**	1110053K06Rik|AW208808|Capp a2	capping protein (actin filament) muscle Z-line, alpha 2	Amp	protein-coding
2939_T1,T22,T2,T32,T42,T4	chr6	1.8E+07	1.8E+07	999	+	**6**	**75**	NA	Intergenic	Intergenic	-5098	NM_007604	12343	Mm.392504	NM_007604	ENSMUSG00000015733	**Capza2**	1110053K06Rik|AW208808|Capp a2	capping protein (actin filament) muscle Z-line, alpha 2	Amp	protein-coding
959_T12,T1,T22,T2,T32,T42	chr15	2.4E+07	2.4E+07	1999	+	**6**	**75**	NA	Intergenic	RMER12|LTR|ERVK	837037	NM_001081299	320865	Mm.241965	NM_001081299	ENSMUSG00000040420	**Cdh18**	B230220E17Rik|Cdh14l	cadherin 18	Amp	protein-coding
104_T1,T22,T2,T32,T3,T4	chr1	1E+08	1E+08	999	+	**6**	**75**	NA	Intergenic	Intergenic	-1E+06	NM_011800	23836	Mm.103640	NM_011800	ENSMUSG00000050840	**Cdh20**	Cdh7	cadherin 20	Amp	protein-coding
889_T1,T22,T2,T32,T3,T42	chr15	1.5E+07	1.5E+07	3999	+	**6**	**75**	NA	Intergenic	Intergenic	-2E+06	NM_007666	12563	Mm.57048	NM_007666	ENSMUSG00000039385	**Cdh6**	cad6	cadherin 6	Amp	protein-coding
3052_T12,T1,T32,T3,T42,T4	chr6	4.5E+07	4.5E+07	999	+	**6**	**75**	NA	intron (NM_001004357, intron 1 of 23)	intron (NM_001004357, intron 1 of 23)	60939	NM_001004357	66797	Mm.440084	NM_025771	ENSMUSG00000039419	**Cntnap2**	5430425M22Rik|Caspr2|mKIAA0868	contactin associated protein-like 2	Amp	protein-coding
1031_T12,T1,T2,T32,T42,T4	chr15	4.9E+07	4.9E+07	999	+	**6**	**75**	NA	Intergenic	Intergenic	-354011	NM_001081391	239420	Mm.309363	NM_001081391	ENSMUSG00000022311	**Csmd3**	4930500N14Rik|mKIAA1894	CUB and Sushi multiple domains 3	Amp	protein-coding
1011_T1,T22,T32,T3,T42,T4	chr15	4.8E+07	4.8E+07	3999	+	**6**	**75**	NA	intron (NM_001081391, intron 32 of 70)	intron (NM_001081391, intron 32 of 70)	1028489	NM_001081391	239420	Mm.309363	NM_001081391	ENSMUSG00000022311	**Csmd3**	4930500N14Rik|mKIAA1894	CUB and Sushi multiple domains 3	Amp	protein-coding
2427_T12,T1,T22,T2,T3,T4	chr3	8.2E+07	8.2E+07	1999	+	**6**	**75**	NA	Intergenic	Intergenic	-321116	NM_177662	229445	Mm.254642	NM_177662	ENSMUSG00000028015	**Ctso**	A330105D01Rik|AI118514	cathepsin O	Amp	protein-coding
2949_T12,T1,T2,T32,T42,T4	chr6	1.9E+07	1.9E+07	999	+	**6**	**75**	NA	Intergenic	Intergenic	-8175	NM_080285	30785	Mm.224189	NM_080285	ENSMUSG00000000416	**Cttnbp2**	3010022N24Rik|4732477G22Rik|6430526E05|9130022E09Rik|AU040881|Cortbp2|ORF4|mKIAA1758	cortactin binding protein 2	Amp	protein-coding
2953_T1,T22,T2,T32,T3,T4	chr6	1.9E+07	1.9E+07	999	+	**6**	**75**	NA	Intergenic	Intergenic	-64175	NM_080285	30785	Mm.224189	NM_080285	ENSMUSG00000000416	**Cttnbp2**	3010022N24Rik|4732477G22Rik|6430526E05|9130022E09Rik|AU040881|Cortbp2|ORF4|mKIAA1758	cortactin binding protein 2	Amp	protein-coding

1296_T12,T1,T22,T2,T32,T3	chr17	7E+07	7E+07	3999	+	**6**	**75**	NA	Intergenic	RSINE1|SINE|B4	-434292	NM_177639	224997	Mm.311840	NM_027712	ENSMUSG00000003279	**Dlgap1**	4933422O14Rik|9630002F18|AI845682|AI848168|BB075781|D17Bwg0511e|GKAP/SAPAP|GKPA/SAPAP|Gkap|Sapap1|mKIAA4162	discs, large (Drosophila) homolog-associated protein 1	Amp	protein-coding
127_T12,T1,T22,T2,T42,T4	chr1	1.1E+08	1.1E+08	3999	+	**6**	**75**	NA	Intergenic	Intergenic	-946582	NM_001081316	319901	Mm.103468	NM_001081316	ENSMUSG00000038702	**Dsel**	9330132E09Rik|DS-epi2	dermatan sulfate epimerase-like	Amp	protein-coding
578_T12,T1,T22,T32,T3,T4	chr13	8.9E+07	8.9E+07	4999	+	**6**	**75**	NA	intron (NM_010103, intron 1 of 9)	intron (NM_010103, intron 1 of 9)	103528	NM_001037987	13612	Mm.125580	NM_010103	ENSMUSG00000034488	**Edil3**	Del-1|Del1	EGF-like repeats and discoidin I-like domains 3	Amp	protein-coding
579_T1,T22,T2,T32,T3,T4	chr13	8.9E+07	8.9E+07	3999	+	**6**	**75**	NA	intron (NM_010103, intron 3 of 9)	intron (NM_010103, intron 3 of 9)	258028	NM_001037987	13612	Mm.125580	NM_010103	ENSMUSG00000034488	**Edil3**	Del-1|Del1	EGF-like repeats and discoidin I-like domains 3	Amp	protein-coding
1295_T12,T22,T2,T3,T42,T4	chr17	6.2E+07	6.2E+07	1999	+	**6**	**75**	NA	Intergenic	Lx6|LINE|L1	1028817	NM_207654	13640	Mm.7978	NM_010109	ENSMUSG00000048915	**Efna5**	AL-1|AV158822|EFL-5|Ephrin-A5|Epl7|LERK-7|RAGS	ephrin A5	Amp	protein-coding
146_T12,T1,T22,T2,T3,T4	chr1	1.2E+08	1.2E+08	4999	+	**6**	**75**	NA	intron (NM_019933, intron 11 of 26)	intron (NM_019933, intron 11 of 26)	-74000	NM_145506	226352	Mm.253156	NM_145506	ENSMUSG00000026383	**Epb41l5**	1700030C16Rik|AL022914|BE37|E230025E14Rik|Epb4.1l5|Lulu1|NBL5	erythrocyte membrane protein band 4.1 like 5	Amp	protein-coding
2874_T12,T1,T22,T32,T3,T4	chr5	8.5E+07	8.5E+07	4999	+	**6**	**75**	NA	Intergenic	RMER6A|LTR|ERVK	-263618	NM_007937	13839	Mm.137991	NM_007937	ENSMUSG00000029245	**Epha5**	AI854630|AW125296|Cek7|Ehk1|Els1|Hek7|Rek7|bsk	Eph receptor A5	Amp	protein-coding
1164_T12,T22,T2,T32,T3,T4	chr16	6.1E+07	6.1E+07	999	+	**6**	**75**	NA	Intergenic	Intergenic	-663469	NM_007938	13840	Mm.455790	NM_007938	ENSMUSG00000055540	**Epha6**	Ehk2|Hek12|m-ehk2	Eph receptor A6	Amp	protein-coding
47_T12,T1,T22,T2,T32,T4	chr1	6.9E+07	6.9E+07	999	+	**6**	**75**	NA	intron (NM_010154, intron 2 of 26)	intron (NM_010154, intron 2 of 26)	421059	NM_010154	13869	Mm.442420	NM_010154	ENSMUSG00000062209	**Erbb4**	Her4|c-erbB-4	erb-b2 receptor tyrosine kinase 4	Amp	protein-coding
425_T12,T1,T22,T32,T3,T42	chr12	3.9E+07	3.9E+07	5999	+	**6**	**75**	NA	Intergenic	Intergenic	326789	NM_001163154	14009	Mm.4866	NM_007960	ENSMUSG00000004151	**Etv1**	ER81|Etsrp81	ets variant 1	Amp	protein-coding
2197_T12,T1,T2,T3,T42,T4	chr3	5.2E+07	5.2E+07	999	+	**6**	**75**	NA	Intergenic	RLTR19B|LTR|ERVK	-19337	NM_019739	56458	Mm.29891	NM_019739	ENSMUSG00000044167	**Foxo1**	AI876417|Afxh|FKHR|Fkhr1|Foxo1a	forkhead box O1	Amp	protein-coding
2207_T1,T2,T32,T3,T42,T4	chr3	5.2E+07	5.2E+07	999	+	**6**	**75**	NA	Intergenic	Lx8|LINE|L1	169663	NM_019739	56458	Mm.29891	NM_019739	ENSMUSG00000044167	**Foxo1**	AI876417|Afxh|FKHR|Fkhr1|Foxo1a	forkhead box O1	Amp	protein-coding

383_T12,T1,T22,T2,T3,T42	chr11	4.2E+07	4.2E+07	999	+	**6**	**75**	NA	intron (NM_177408, intron 1 of 8)	intron (NM_177408, intron 1 of 8)	8714	NM_177408	14406	Mm.5309	NM_008073	ENSMUSG00000020436	**Gabrg2**	GABAA-R|Gabrg-2|gamma2	gamma-aminobutyric acid (GABA) A receptor, subunit gamma 2	Amp	protein-coding
1491_T12,T2,T32,T3,T42,T4	chr2	5.5E+07	5.5E+07	999	+	**6**	**75**	NA	intron (NM_173030, intron 4 of 12)	intron (NM_173030, intron 4 of 12)	320613	NM_173030	271786	Mm.330227	NM_173030	ENSMUSG00000060988	**Galnt13**	A230002A12|A230020F20|BB182356	UDP-N-acetyl-alpha-D-galactosamine:polypeptide N-acetylgalactosaminyltransferase 13	Amp	protein-coding
2733_T12,T1,T22,T2,T32,T3	chr5	3E+07	3E+07	4999	+	**6**	**75**	NA	Intergenic	Intergenic	-5196	NM_001167879	242915	Mm.332287	NM_001167879	ENSMUSG00000044576	**Garem2**	Fam59b|Gareml|Gm444	GRB2 associated regulator of MAPK1 subtype 2	Amp	protein-coding
582_T1,T22,T2,T3,T42,T4	chr13	9.7E+07	9.7E+07	999	+	**6**	**75**	NA	Intergenic	L1Md_F|LINE|L1	-81689	NM_001166065	218476	Mm.483490	NM_001166065	ENSMUSG00000091387	**Gcnt4**	C2gnt3|Gm279|Gm73	glucosaminyl (N-acetyl) transferase 4, core 2 (beta-1,6-N-acetylglucosaminyl transferase)	Amp	protein-coding
2700_T12,T1,T2,T3,T42,T4	chr5	1E+07	1E+07	999	+	**6**	**75**	NA	Intergenic	Intergenic	-293648	NM_181850	108069	Mm.318966	NM_181850	ENSMUSG00000003974	**Grm3**	0710001G23Rik|Gprc1c|mGlu3|mGluR3	glutamate receptor, metabotropic 3	Amp	protein-coding
1598_T12,T1,T2,T3,T42,T4	chr2	1.3E+08	1.3E+08	999	+	**6**	**75**	NA	Intergenic	B4A|SINE|B4	200352	NM_010403	15112	Mm.26634	NM_010403	ENSMUSG00000027261	**Hao1**	GOX|Gox1|Hao-1	hydroxyacid oxidase 1, liver	Amp	protein-coding
1076_T12,T1,T22,T2,T32,T42	chr15	5.7E+07	5.7E+07	999	+	**6**	**75**	NA	Intergenic	Intergenic	-383454	NM_008216	15117	Mm.5148	NM_008216	ENSMUSG00000022367	**Has2**	-	hyaluronan synthase 2	Amp	protein-coding
1643_T12,T1,T22,T2,T42,T4	chr3	4009501	4010500	999	+	**6**	**75**	NA	Intergenic	Intergenic	501970	NM_013920	30942	Mm.330897	NM_013920	ENSMUSG00000017688	**Hnf4g**	NR2A2	hepatocyte nuclear factor 4, gamma	Amp	protein-coding
1582_T1,T22,T2,T32,T3,T42	chr2	1.1E+08	1.1E+08	999	+	**6**	**75**	NA	Intergenic	Intergenic	854411	NM_021275	16492	Mm.142718	NM_021275	ENSMUSG00000042604	**Kcna4**	Kv1.4	potassium voltage-gated channel, shaker-related subfamily, member 4	Amp	protein-coding
1497_T1,T22,T32,T3,T42,T4	chr2	5.5E+07	5.5E+07	999	+	**6**	**75**	NA	intron (NM_008426, intron 3 of 3)	intron (NM_008426, intron 3 of 3)	27030	NM_008426	16519	Mm.5127	NM_008426	ENSMUSG00000026824	**Kcnj3**	GIRK-1|GIRK1|Kcnf3|Kir3.1	potassium inwardly-rectifying channel, subfamily J, member 3	Amp	protein-coding
2829_T12,T1,T22,T2,T32,T4	chr5	6.9E+07	6.9E+07	3999	+	**6**	**75**	NA	intron (NM_175519, intron 1 of 1)	intron (NM_175519, intron 1 of 1)	89209	NM_175519	243043	Mm.234821	NM_175519	ENSMUSG00000037653	**Kctd8**	A730087N02Rik	potassium channel tetramerisation domain containing 8	Amp	protein-coding
771_T12,T1,T32,T3,T42,T4	chr14	9.6E+07	9.6E+07	4999	+	**6**	**75**	NA	intron (NM_053105, intron 1 of 10)	intron (NM_053105, intron 1 of 10)	94034	NM_053105	93688	Mm.308735	NM_053105	ENSMUSG00000022076	**Klhl1**	mKIAA1490	kelch-like 1	Amp	protein-coding

446_T12,T2,T32,T3,T42,T4	chr12	6.2E+07	6.2E+07	3999	+	**6**	**75**	NA	intron (NM_001310586, intron 1 of 5)	Lx8|LINE|L1	94333	NM_001310586	238205	Mm.28802	NM_178714	ENSMUSG00000035653	**Lrfn5**	AI427653|AI604817|C130061B21|mKIAA4208	leucine rich repeat and fibronectin type III domain containing 5	Amp	protein-coding
2450_T1,T22,T2,T32,T3,T4	chr3	1.1E+08	1.1E+08	3999	+	**6**	**75**	NA	exon (NM_001039488, exon 2 of 4)	exon (NM_001039488, exon 2 of 4)	10818	NM_001286685	321000	Mm.259638	NM_028081	ENSMUSG00000056260	**Lrif1**	2010012G17Rik|4933421E11Rik|AI450568	ligand dependent nuclear receptor interacting factor 1	Amp	protein-coding
1465_T1,T2,T32,T3,T42,T4	chr2	4.3E+07	4.3E+07	999	+	**6**	**75**	NA	Intergenic	Intergenic	-254402	NM_053011	94217	Mm.441398	NM_053011	ENSMUSG00000049252	**Lrp1b**	9630004P12Rik|LRP-DIT	low density lipoprotein-related protein 1B (deleted in tumors)	Amp	protein-coding
1541_T12,T1,T22,T2,T3,T4	chr2	9.7E+07	9.7E+07	3999	+	**6**	**75**	NA	intron (NM_001289742, intron 3 of 7)	intron (NM_001289742, intron 3 of 7)	528331	NM_001289743	241568	Mm.241682	NM_178725	ENSMUSG00000050587	**Lrrc4c**	6430556C10Rik|NGL-1	leucine rich repeat containing 4C	Amp	protein-coding
2968_T12,T1,T2,T32,T3,T42	chr6	1.9E+07	1.9E+07	3999	+	**6**	**75**	NA	Intergenic	L1Md_T|LINE|L1	-66135	NM_133939	76522	Mm.275158	NM_133939	ENSMUSG00000044155	**Lsm8**	2010003I05Rik|AW214405|Naa38	LSM8 homolog, U6 small nuclear RNA associated	Amp	protein-coding
2970_T12,T22,T2,T32,T42,T4	chr6	1.9E+07	1.9E+07	999	+	**6**	**75**	NA	Intergenic	L1_Mus1|LINE|L1	-60635	NM_133939	76522	Mm.275158	NM_133939	ENSMUSG00000044155	**Lsm8**	2010003I05Rik|AW214405|Naa38	LSM8 homolog, U6 small nuclear RNA associated	Amp	protein-coding
2973_T1,T22,T2,T3,T42,T4	chr6	1.9E+07	1.9E+07	999	+	**6**	**75**	NA	Intergenic	Intergenic	-7635	NM_133939	76522	Mm.275158	NM_133939	ENSMUSG00000044155	**Lsm8**	2010003I05Rik|AW214405|Naa38	LSM8 homolog, U6 small nuclear RNA associated	Amp	protein-coding
2974_T12,T22,T2,T3,T42,T4	chr6	1.9E+07	1.9E+07	999	+	**6**	**75**	NA	Intergenic	Intergenic	-6635	NM_133939	76522	Mm.275158	NM_133939	ENSMUSG00000044155	**Lsm8**	2010003I05Rik|AW214405|Naa38	LSM8 homolog, U6 small nuclear RNA associated	Amp	protein-coding
2977_T12,T22,T2,T3,T42,T4	chr6	1.9E+07	1.9E+07	999	+	**6**	**75**	NA	Intergenic	RLTR6-int|LTR|ERV1	-1635	NM_133939	76522	Mm.275158	NM_133939	ENSMUSG00000044155	**Lsm8**	2010003I05Rik|AW214405|Naa38	LSM8 homolog, U6 small nuclear RNA associated	Amp	protein-coding
2244_T12,T1,T32,T3,T42,T4	chr3	5.6E+07	5.6E+07	999	+	**6**	**75**	NA	intron (NM_030595, intron 40 of 57)	intron (NM_030595, intron 40 of 57)	4490	NM_010750	17116	Mm.440209	NM_010750	ENSMUSG00000056947	**Mab21l1**	AW047968	mab-21-like 1 (C. elegans)	Amp	protein-coding
2190_T12,T1,T2,T32,T3,T42	chr3	5.2E+07	5.2E+07	999	+	**6**	**75**	NA	intron (NM_001004176, intron 1 of 4)	intron (NM_001004176, intron 1 of 4)	180006	NM_001004176	433586	Mm.234972	NM_001004176	ENSMUSG00000061143	**Maml3**	AV234550|BC049812|Mam-2|mKIAA1816	mastermind like 3 (Drosophila)	Amp	protein-coding
2193_T1,T22,T2,T32,T3,T42	chr3	5.2E+07	5.2E+07	999	+	**6**	**75**	NA	intron (NM_001004176, intron 1 of 4)	intron (NM_001004176, intron 1 of 4)	167006	NM_001004176	433586	Mm.234972	NM_001004176	ENSMUSG00000061143	**Maml3**	AV234550|BC049812|Mam-2|mKIAA1816	mastermind like 3 (Drosophila)	Amp	protein-coding
554_T1,T22,T2,T3,T42,T4	chr13	8.3E+07	8.3E+07	6999	+	**6**	**75**	NA	Intergenic	L1VL1|LINE|L1	-278034	NM_001170537	17260	Mm.24001	NM_025282	ENSMUSG00000005583	**Mef2c**	5430401D19Rik|9930028G15Rik|AV011172|Mef2	myocyte enhancer factor 2C	Amp	protein-coding

2188_T12,T1,T22,T2,T3,T4	chr3	5.2E+07	5.2E+07	999	+	**6**	**75**	NA	Intergenic	B4|SINE|B4	-37179	NM_174995	211666	Mm.24679	NM_174995	ENSMUSG00000074604	**Mgst2**	GST2|MGST-II	microsomal glutathione S-transferase 2	Amp	protein-coding
3465_T12,T22,T2,T32,T3,T42	chr8	3.8E+07	3.8E+07	999	+	**6**	**75**	NA	intron (NM_145841, intron 1 of 7)	intron (NM_145841, intron 1 of 7)	77202	NR_029884	723860		NR_029884	ENSMUSG00000065457	**Mir383**	Mirn383|mir-383|mmu-mir-383	microRNA 383	Amp	ncRNA
3467_T12,T1,T2,T32,T3,T42	chr8	3.8E+07	3.8E+07	1999	+	**6**	**75**	NA	intron (NM_145841, intron 1 of 7)	intron (NM_145841, intron 1 of 7)	72702	NR_029884	723860		NR_029884	ENSMUSG00000065457	**Mir383**	Mirn383|mir-383|mmu-mir-383	microRNA 383	Amp	ncRNA
3471_T12,T1,T22,T2,T32,T42	chr8	3.8E+07	3.8E+07	2999	+	**6**	**75**	NA	intron (NM_145841, intron 1 of 7)	intron (NM_145841, intron 1 of 7)	-100798	NR_029884	723860		NR_029884	ENSMUSG00000065457	**Mir383**	Mirn383|mir-383|mmu-mir-383	microRNA 383	Amp	ncRNA
3456_T12,T22,T2,T32,T3,T4	chr8	3.8E+07	3.8E+07	3999	+	**6**	**75**	NA	intron (NM_145841, intron 2 of 7)	intron (NM_145841, intron 2 of 7)	344702	NR_029884	723860		NR_029884	ENSMUSG00000065457	**Mir383**	Mirn383|mir-383|mmu-mir-383	microRNA 383	Amp	ncRNA
3460_T22,T2,T32,T3,T42,T4	chr8	3.8E+07	3.8E+07	5999	+	**6**	**75**	NA	intron (NM_145841, intron 2 of 7)	intron (NM_145841, intron 2 of 7)	329702	NR_029884	723860		NR_029884	ENSMUSG00000065457	**Mir383**	Mirn383|mir-383|mmu-mir-383	microRNA 383	Amp	ncRNA
2581_T12,T1,T22,T3,T42,T4	chr4	3.7E+07	3.7E+07	999	+	**6**	**75**	NA	intron (NM_001166000, intron 1 of 8)	intron (NM_001166000, intron 1 of 8)	89453	NR_030545	1E+08		NR_030545	ENSMUSG00000077851	**Mir876**	Mirn876|mir-876|mmu-mir-876	microRNA 876	Amp	ncRNA
2181_T12,T1,T32,T3,T42,T4	chr3	5.1E+07	5.1E+07	12999	+	**6**	**75**	NA	intron (NM_053089, intron 1 of 19)	(TA)n|Simple_repeat|Simple_repeat	13984	NM_053089	74838	Mm.275281	NM_053089	ENSMUSG00000063273	**Naa15**	5730450D16Rik|6330400I15|ASTBDN|Narg1|Tbdn-1|mNAT1	N(alpha)-acetyltransferase 15, NatA auxiliary subunit	Amp	protein-coding
2261_T12,T1,T2,T3,T42,T4	chr3	5.6E+07	5.6E+07	999	+	**6**	**75**	NA	Intergenic	Intergenic	-25299	NM_030595	26422	Mm.384353	NM_030595	ENSMUSG00000027799	**Nbea**	Lyst2|mKIAA1544	neurobeachin	Amp	protein-coding
2263_T12,T1,T2,T3,T42,T4	chr3	5.6E+07	5.6E+07	999	+	**6**	**75**	NA	Intergenic	ORR1E|LTR|ERVL-MaLR	-32299	NM_030595	26422	Mm.384353	NM_030595	ENSMUSG00000027799	**Nbea**	Lyst2|mKIAA1544	neurobeachin	Amp	protein-coding
2259_T12,T1,T2,T3,T42,T4	chr3	5.6E+07	5.6E+07	7999	+	**6**	**75**	NA	intron (NM_030595, intron 1 of 57)	L1Md_T|LINE|L1	44201	NM_030595	26422	Mm.384353	NM_030595	ENSMUSG00000027799	**Nbea**	Lyst2|mKIAA1544	neurobeachin	Amp	protein-coding
1726_T1,T22,T32,T3,T42,T4	chr3	2.5E+07	2.5E+07	3999	+	**6**	**75**	NA	Intergenic	Lx5c|LINE|L1	1626807	NM_001163387	192167	Mm.316080	NM_138666	ENSMUSG00000063887	**Nlgn1**	6330415N05Rik|BB179718|NL1|Nlg1|mKIAA1070	neuroligin 1	Amp	protein-coding
1735_T1,T22,T32,T3,T42,T4	chr3	2.6E+07	2.6E+07	3999	+	**6**	**75**	NA	intron (NM_138666, intron 5 of 7)	intron (NM_138666, intron 5 of 7)	634807	NM_001163387	192167	Mm.316080	NM_138666	ENSMUSG00000063887	**Nlgn1**	6330415N05Rik|BB179718|NL1|Nlg1|mKIAA1070	neuroligin 1	Amp	protein-coding

535_T12,T1,T2,T32,T42,T4	chr13	7.9E+07	7.9E+07	999	+	**6**	**75**	NA	Intergenic	L1Md_T|LINE|L1	-927018	NM_010151	13865	Mm.439653	NM_010151	ENSMUSG00000069171	**Nr2f1**	COUP-TF1|COUP-TFI|COUPTFA|EAR-3|EAR3|Erbal3|SVP44|Tcfcoup1	nuclear receptor subfamily 2, group F, member 1	Amp	protein-coding
539_T1,T22,T2,T32,T3,T4	chr13	7.9E+07	7.9E+07	1999	+	**6**	**75**	NA	Intergenic	MLT1D|LTR|ERVL-MaLR	-970518	NM_010151	13865	Mm.439653	NM_010151	ENSMUSG00000069171	**Nr2f1**	COUP-TF1|COUP-TFI|COUPTFA|EAR-3|EAR3|Erbal3|SVP44|Tcfcoup1	nuclear receptor subfamily 2, group F, member 1	Amp	protein-coding
1280_T12,T22,T2,T32,T3,T4	chr17	6E+07	6E+07	3999	+	**6**	**75**	NA	Intergenic	Intergenic	-628178	NM_026497	67993	Mm.36507	NM_026497	ENSMUSG00000024228	**Nudt12**	0610016O18Rik	nudix (nucleoside diphosphate linked moiety X)-type motif 12	Amp	protein-coding
2889_T12,T1,T2,T32,T3,T4	chr6	9905501	9906500	999	+	**6**	**75**	NA	Intergenic	Intergenic	955981	NM_008751	18231	Mm.46954	NM_008751	ENSMUSG00000046178	**Nxph1**	C130005L03Rik	neurexophilin 1	Amp	protein-coding
631_T1,T22,T2,T32,T3,T4	chr14	8.1E+07	8.1E+07	9999	+	**6**	**75**	NA	Intergenic	Intergenic	942198	NM_001030294	380924	Mm.26456	NM_001030294	ENSMUSG00000022026	**Olfm4**	GC1|GW112|Gm296|Gm913|OlfD|pPD4	olfactomedin 4	Amp	protein-coding
1914_T12,T1,T22,T2,T3,T42	chr3	4.6E+07	4.6E+07	999	+	**6**	**75**	NA	Intergenic	Intergenic	411941	NM_001101479	241989	Mm.119234	NM_001101479	ENSMUSG00000090919	**Pabpc4l**	C330050A14Rik|EG241989	poly(A) binding protein, cytoplasmic 4-like	Amp	protein-coding
1929_T12,T1,T22,T32,T3,T42	chr3	4.6E+07	4.6E+07	999	+	**6**	**75**	NA	Intergenic	L3|LINE|CR1	308941	NM_001101479	241989	Mm.119234	NM_001101479	ENSMUSG00000090919	**Pabpc4l**	C330050A14Rik|EG241989	poly(A) binding protein, cytoplasmic 4-like	Amp	protein-coding
1935_T12,T2,T32,T3,T42,T4	chr3	4.6E+07	4.6E+07	999	+	**6**	**75**	NA	Intergenic	Intergenic	262941	NM_001101479	241989	Mm.119234	NM_001101479	ENSMUSG00000090919	**Pabpc4l**	C330050A14Rik|EG241989	poly(A) binding protein, cytoplasmic 4-like	Amp	protein-coding
1870_T12,T22,T2,T32,T3,T4	chr3	4.5E+07	4.5E+07	999	+	**6**	**75**	NA	intron (NM_001098170, intron 4 of 4)	(TTTC)n|Simple_repeat|Simple_repeat	18602	NM_001098171	18526	Mm.242644	NM_011043	ENSMUSG00000049100	**Pcdh10**	6430521D13Rik|6430703F07Rik|OL-pc|mKIAA1400	protocadherin 10	Amp	protein-coding
1872_T12,T22,T2,T32,T3,T4	chr3	4.5E+07	4.5E+07	999	+	**6**	**75**	NA	intron (NM_001098170, intron 4 of 4)	B2_Mm2|SINE|B2	24602	NM_001098171	18526	Mm.242644	NM_011043	ENSMUSG00000049100	**Pcdh10**	6430521D13Rik|6430703F07Rik|OL-pc|mKIAA1400	protocadherin 10	Amp	protein-coding
646_T12,T1,T22,T2,T3,T42	chr14	8.3E+07	8.3E+07	999	+	**6**	**75**	NA	Intergenic	L1_Mus1|LINE|L1	-1E+06	NM_001013753	219228	Mm.153643	NM_001013753	ENSMUSG00000035566	**Pcdh17**	C030033F14Rik|Gm78	protocadherin 17	Amp	protein-coding
654_T12,T1,T22,T32,T3,T4	chr14	8.3E+07	8.3E+07	5999	+	**6**	**75**	NA	Intergenic	Intergenic	-957063	NM_001013753	219228	Mm.153643	NM_001013753	ENSMUSG00000035566	**Pcdh17**	C030033F14Rik|Gm78	protocadherin 17	Amp	protein-coding
2116_T12,T1,T22,T32,T3,T4	chr3	4.9E+07	4.9E+07	999	+	**6**	**75**	NA	Intergenic	(TAGA)n|Simple_repeat|Simple_repeat	465316	NM_130448	73173	Mm.87246	NM_130448	ENSMUSG00000037892	**Pcdh18**	3110038E07Rik|BB095589|PCDH68L	protocadherin 18	Amp	protein-coding

2138_T12,T1,T22,T32,T3,T42	chr3	5E+07	5E+07	999	+	**6**	**75**	NA	Intergenic	L1Md_T|LINE|L1	-59684	NM_130448	73173	Mm.87246	NM_130448	ENSMUSG00000037892	**Pcdh18**	3110038E07Rik|BB095589|PCDH68L	protocadherin 18	Amp	protein-coding
2150_T12,T22,T2,T32,T3,T4	chr3	5E+07	5E+07	999	+	**6**	**75**	NA	Intergenic	Intergenic	-335684	NM_130448	73173	Mm.87246	NM_130448	ENSMUSG00000037892	**Pcdh18**	3110038E07Rik|BB095589|PCDH68L	protocadherin 18	Amp	protein-coding
690_T1,T22,T32,T3,T42,T4	chr14	8.8E+07	8.8E+07	5999	+	**6**	**75**	NA	Intergenic	Intergenic	106896	NM_178685	219257	Mm.128861	NM_178685	ENSMUSG00000050505	**Pcdh20**	C630015B17Rik|Pcdh13	protocadherin 20	Amp	protein-coding
725_T12,T2,T32,T3,T42,T4	chr14	9.2E+07	9.2E+07	999	+	**6**	**75**	NA	Intergenic	Intergenic	1591888	NM_001271800	211712	Mm.37126	NM_001081377	ENSMUSG00000055421	**Pcdh9**	-	protocadherin 9	Amp	protein-coding
728_T12,T1,T22,T2,T32,T4	chr14	9.2E+07	9.2E+07	999	+	**6**	**75**	NA	Intergenic	Intergenic	1583888	NM_001271800	211712	Mm.37126	NM_001081377	ENSMUSG00000055421	**Pcdh9**	-	protocadherin 9	Amp	protein-coding
741_T12,T1,T22,T32,T3,T42	chr14	9.4E+07	9.4E+07	4999	+	**6**	**75**	NA	intron (NM_001081377, intron 1 of 3)	intron (NM_001081377, intron 1 of 3)	217888	NM_001271800	211712	Mm.37126	NM_001081377	ENSMUSG00000055421	**Pcdh9**	-	protocadherin 9	Amp	protein-coding
2388_T12,T1,T2,T32,T3,T4	chr3	7.5E+07	7.5E+07	999	+	**6**	**75**	NA	Intergenic	Intergenic	128852	NM_019745	56426	Mm.316473	NM_019745	ENSMUSG00000027835	**Pdcd10**	2410003B13Rik|Ccm3|Tfa15|Tfar15	programmed cell death 10	Amp	protein-coding
2390_T12,T1,T2,T32,T3,T4	chr3	7.6E+07	7.6E+07	3999	+	**6**	**75**	NA	intron (NM_019745, intron 2 of 7)	L1_Mus3|LINE|L1	19352	NM_019745	56426	Mm.316473	NM_019745	ENSMUSG00000027835	**Pdcd10**	2410003B13Rik|Ccm3|Tfa15|Tfar15	programmed cell death 10	Amp	protein-coding
1440_T12,T1,T22,T2,T42,T4	chr2	4.1E+07	4.1E+07	999	+	**6**	**75**	NA	intron (NM_053011, intron 83 of 90)	L1_Mur2|LINE|L1	-1E+06	NM_024209	67857	Mm.389242	NM_024209	ENSMUSG00000026753	**Ppp6c**	2310003C10Rik	protein phosphatase 6, catalytic subunit	Amp	protein-coding
2625_T1,T22,T2,T32,T3,T4	chr4	7.8E+07	7.8E+07	3999	+	**6**	**75**	NA	intron (NM_011211, intron 1 of 39)	intron (NM_011211, intron 1 of 39)	183395	NM_011211	19266	Mm.184021	NM_011211	ENSMUSG00000028399	**Ptprd**	1110002J03Rik|3000002J10Rik|B230219D21Rik|R-PTP-delta	protein tyrosine phosphatase, receptor type, D	Amp	protein-coding
206_T12,T1,T22,T2,T42,T4	chr10	6313501	6319500	5999	+	**6**	**75**	NA	Intergenic	RMER4B|LTR|ERVK	-394100	NM_019958	56533	Mm.44606	NM_019958	ENSMUSG00000019775	**Rgs17**	6430507P11Rik|Rgsz2	regulator of G-protein signaling 17	Amp	protein-coding
169_T1,T22,T2,T32,T3,T4	chr1	1.5E+08	1.5E+08	999	+	**6**	**75**	NA	Intergenic	Intergenic	-370579	NM_022881	64214	Mm.253927	NM_022881	ENSMUSG00000026357	**Rgs18**	-	regulator of G-protein signaling 18	Amp	protein-coding
174_T12,T1,T2,T32,T3,T4	chr1	1.5E+08	1.5E+08	999	+	**6**	**75**	NA	Intergenic	Lx7|LINE|L1	-591579	NM_022881	64214	Mm.253927	NM_022881	ENSMUSG00000026357	**Rgs18**	-	regulator of G-protein signaling 18	Amp	protein-coding
970_T12,T1,T22,T2,T3,T42	chr15	3.3E+07	3.3E+07	1999	+	**6**	**75**	NA	Intergenic	Lx5c|LINE|L1	-24223	NM_008304	15529	Mm.234266	NM_008304	ENSMUSG00000022261	**Sdc2**	4833414L08Rik|AA960457|Hspg1|Synd2|syndecan-2	syndecan 2	Amp	protein-coding

2713_T1,T22,T2,T32,T3,T42	chr5	1.3E+07	1.3E+07	3999	+	**6**	**75**	NA	Intergenic	MER58A|DNA|hAT-Charlie	-289284	NM_001243072	20346	Mm.372039	NM_009152	ENSMUSG00000028883	**Sema3a**	Hsema-I|SEMA1|SemD|Semad|coll-1	sema domain, immunoglobulin domain (Ig), short basic domain, secreted, (semaphorin) 3A	Amp	protein-coding
2708_T12,T1,T22,T2,T3,T4	chr5	1.2E+07	1.2E+07	999	+	**6**	**75**	NA	intron (NM_028882, intron 3 of 17)	IMPB_01|Satellite|Satellite	85834	NM_028882	108151	Mm.89313	NM_028882	ENSMUSG00000040254	**Sema3d**	4631426B19Rik	sema domain, immunoglobulin domain (Ig), short basic domain, secreted, (semaphorin) 3D	Amp	protein-coding
3480_T12,T22,T2,T32,T3,T4	chr8	3.9E+07	3.9E+07	999	+	**6**	**75**	NA	intron (NM_145841, intron 1 of 7)	intron (NM_145841, intron 1 of 7)	101508	NM_145841	244431	Mm.210388	NM_145841	ENSMUSG00000039539	**Sgcz**	C230085N17Rik	sarcoglycan zeta	Amp	protein-coding
3397_T12,T1,T2,T32,T3,T42	chr8	5021501	5025500	3999	+	**6**	**75**	NA	Intergenic	Intergenic	81732	NM_011388	20494	Mm.3500	NM_011388	ENSMUSG00000023073	**Slc10a2**	9130221J18Rik|AI605518|ASBT|IBAT|ISBT	solute carrier family 10, member 2	Amp	protein-coding
3405_T1,T22,T2,T3,T42,T4	chr8	6397501	6398500	999	+	**6**	**75**	NA	Intergenic	Intergenic	-1E+06	NM_011388	20494	Mm.3500	NM_011388	ENSMUSG00000023073	**Slc10a2**	9130221J18Rik|AI605518|ASBT|IBAT|ISBT	solute carrier family 10, member 2	Amp	protein-coding
3410_T1,T22,T32,T3,T42,T4	chr8	6528501	6530500	1999	+	**6**	**75**	NA	Intergenic	Intergenic	-1E+06	NM_011388	20494	Mm.3500	NM_011388	ENSMUSG00000023073	**Slc10a2**	9130221J18Rik|AI605518|ASBT|IBAT|ISBT	solute carrier family 10, member 2	Amp	protein-coding
1079_T1,T22,T2,T32,T3,T42	chr15	5.7E+07	5.7E+07	3999	+	**6**	**75**	NA	intron (NM_172378, intron 2 of 11)	intron (NM_172378, intron 2 of 11)	206125	NM_172378	210463	Mm.26555	NM_172378	ENSMUSG00000022366	**Slc22a22**	oat-pg	solute carrier family 22 (organic cation transporter), member 22	Amp	protein-coding
2157_T12,T1,T22,T2,T3,T4	chr3	5E+07	5E+07	1999	+	**6**	**75**	NA	Intergenic	MTA_Mm-int|LTR|ERVL-MaLR	-29887	NM_011990	26570	Mm.260988	NM_011990	ENSMUSG00000027737	**Slc7a11**	9930009M05Rik|AI451155|sut|xCT	solute carrier family 7 (cationic amino acid transporter, y+ system), member 11	Amp	protein-coding
2751_T12,T1,T22,T32,T3,T42	chr5	4.7E+07	4.7E+07	999	+	**6**	**75**	NA	Intergenic	L2|LINE|L2	-527155	NR_111900	20563	Mm.289739	NM_178804	ENSMUSG00000031558	**Slit2**	Drad-1|E030015M03Rik|E130320P19Rik|Slil3|mKIAA4141|slit-2	slit homolog 2 (Drosophila)	Amp	protein-coding
2754_T12,T1,T22,T2,T3,T42	chr5	4.8E+07	4.8E+07	999	+	**6**	**75**	NA	Intergenic	Lx9|LINE|L1	-388155	NR_111900	20563	Mm.289739	NM_178804	ENSMUSG00000031558	**Slit2**	Drad-1|E030015M03Rik|E130320P19Rik|Slil3|mKIAA4141|slit-2	slit homolog 2 (Drosophila)	Amp	protein-coding
804_T12,T1,T22,T2,T32,T3	chr14	1.1E+08	1.1E+08	2999	+	**6**	**75**	NA	Intergenic	Intergenic	831239	NM_199065	76965	Mm.257268	NM_199065	ENSMUSG00000075478	**Slitrk1**	3200001I04Rik	SLIT and NTRK-like family, member 1	Amp	protein-coding
818_T12,T1,T22,T2,T32,T3	chr14	1.1E+08	1.1E+08	5999	+	**6**	**75**	NA	Intergenic	Intergenic	15739	NM_199065	76965	Mm.257268	NM_199065	ENSMUSG00000075478	**Slitrk1**	3200001I04Rik	SLIT and NTRK-like family, member 1	Amp	protein-coding

826_T12,T1,T22,T2,T3,T42	chr14	1.1E+08	1.1E+08	4999	+	**6**	**75**	NA	Intergenic	Intergenic	-246761	NM_199065	76965	Mm.257268	NM_199065	ENSMUSG00000075478	**Slitrk1**	3200001I04Rik	SLIT and NTRK-like family, member 1	Amp	protein-coding
845_T12,T22,T2,T32,T3,T42	chr14	1.1E+08	1.1E+08	999	+	**6**	**75**	NA	Intergenic	Intergenic	134149	NM_175499	239250	Mm.49728	NM_175499	ENSMUSG00000045871	**Slitrk6**	4832410J21Rik|Sltk6	SLIT and NTRK-like family, member 6	Amp	protein-coding
3364_T12,T1,T22,T2,T3,T42	chr7	6E+07	6E+07	3999	+	**6**	**75**	NA	Intergenic	Intergenic	6931	NR_015456	52480	Mm.482346	NR_015456	ENSMUSG00000100826	**Snhg14**	AU018661|D7Ertd715e|Lncat	small nucleolar RNA host gene 14	Amp	ncRNA
255_T12,T1,T2,T3,T42,T4	chr10	4.7E+07	4.7E+07	999	+	**6**	**75**	NA	Intergenic	L1_Mus2|LINE|L1	1000251	NR_028276	108637		NR_028276	ENSMUSG00000096263	**Snord14c**	Gm23107|Rnu14|U14	small nucleolar RNA, C/D box 14C	Amp	snoRNA
3575_T12,T1,T22,T2,T3,T42	chr8	6.3E+07	6.3E+07	999	+	**6**	**75**	NA	intron (NM_023689, intron 7 of 11)	RLTR42-int|LTR|ERVK	371655	NM_023689	72902	Mm.334552	NM_023689	ENSMUSG00000054162	**Spock3**	2900045C01Rik|AI428471|mKIAA4039	sparc/osteonectin, cwcv and kazal-like domains proteoglycan 3	Amp	protein-coding
2941_T12,T1,T2,T3,T42,T4	chr6	1.8E+07	1.8E+07	999	+	**6**	**75**	NA	Intergenic	Intergenic	-6994	NM_001289624	64213	Mm.12051	NM_022332	ENSMUSG00000029534	**St7**	9430001H04Rik|Fam4a2|HELG|RAY1|SEN4|TSG7	suppression of tumorigenicity 7	Amp	protein-coding
3603_T12,T1,T32,T3,T42,T4	chr8	8.3E+07	8.3E+07	3999	+	**6**	**75**	NA	Intergenic	L1Md_A|LINE|L1	-41852	NM_001111304	71310	Mm.24031	NM_027758	ENSMUSG00000031709	**Tbc1d9**	4933431N12Rik|AI847101|AW490653|C76116	TBC1 domain family, member 9	Amp	protein-coding
2919_T12,T1,T22,T3,T42,T4	chr6	1.7E+07	1.7E+07	999	+	**6**	**75**	NA	intron (NM_031198, intron 1 of 6)	intron (NM_031198, intron 1 of 6)	10441	NM_031198	21426	Mm.36217	NM_031198	ENSMUSG00000029553	**Tfec**	BB107417|Tcfec|bHLHe34	transcription factor EC	Amp	protein-coding
2616_T12,T1,T2,T32,T3,T4	chr4	7.2E+07	7.2E+07	4999	+	**6**	**75**	NA	Intergenic	MER77|LTR|ERVL	543893	NM_011599	21885	Mm.278444	NM_011599	ENSMUSG00000008305	**Tle1**	C230057C06Rik|Estm14|Grg-1|Grg1|Tle4l	transducin-like enhancer of split 1	Amp	protein-coding
2321_T12,T22,T32,T3,T42,T4	chr3	5.7E+07	5.7E+07	5999	+	**6**	**75**	NA	Intergenic	RLTR25B|LTR|ERVK	-42910	NM_145539	229302	Mm.26618	NM_145539	ENSMUSG00000027801	**Tm4sf4**	Iltmp	transmembrane 4 superfamily member 4	Amp	protein-coding
28_T12,T1,T22,T3,T42,T4	chr1	5E+07	5E+07	999	+	**6**	**75**	NA	Intergenic	Intergenic	-859523	NM_019790	56363	Mm.245154	NM_019790	ENSMUSG00000026109	**Tmeff2**	4832418D20Rik|7630402F16Rik|TR-2	transmembrane protein with EGF-like and two follistatin-like domains 2	Amp	protein-coding
30_T12,T22,T32,T3,T42,T4	chr1	5E+07	5E+07	1999	+	**6**	**75**	NA	Intergenic	Intergenic	-854023	NM_019790	56363	Mm.245154	NM_019790	ENSMUSG00000026109	**Tmeff2**	4832418D20Rik|7630402F16Rik|TR-2	transmembrane protein with EGF-like and two follistatin-like domains 2	Amp	protein-coding
31_T1,T22,T2,T32,T3,T4	chr1	5E+07	5E+07	3999	+	**6**	**75**	NA	Intergenic	Intergenic	-653023	NM_019790	56363	Mm.245154	NM_019790	ENSMUSG00000026109	**Tmeff2**	4832418D20Rik|7630402F16Rik|TR-2	transmembrane protein with EGF-like and two follistatin-like domains 2	Amp	protein-coding
3368_T12,T1,T22,T2,T3,T42	chr7	6.4E+07	6.4E+07	6999	+	**6**	**75**	NA	Intergenic	Intergenic	-76835	NM_001039104	17364	Mm.38875	NM_018752	ENSMUSG00000030523	**Trpm1**	4732499L03Rik|AI606771|Ltrpc1|Mlsn1	transient receptor potential cation channel, subfamily M, member 1	Amp	protein-coding

1044_T12,T2,T32,T3,T42,T4	chr15	5E+07	5E+07	999	+	**6**	**75**	NA	Intergenic	Intergenic	721049	NM_001310481	83925	Mm.30466	NM_032000	ENSMUSG00000038679	**Trps1**	AI115454|AI447310|D15Ertd586e	trichorhinophalangeal syndrome I (human)	Amp	protein-coding
1055_T12,T1,T2,T32,T3,T4	chr15	5.1E+07	5.1E+07	999	+	**6**	**75**	NA	intron (NM_001310481, intron 4 of 5)	intron (NM_001310481, intron 4 of 5)	165049	NM_001310481	83925	Mm.30466	NM_032000	ENSMUSG00000038679	**Trps1**	AI115454|AI447310|D15Ertd586e	trichorhinophalangeal syndrome I (human)	Amp	protein-coding
1057_T12,T1,T32,T3,T42,T4	chr15	5.1E+07	5.1E+07	999	+	**6**	**75**	NA	intron (NM_001310481, intron 4 of 5)	(CATA)n|Simple_repeat|Simple_repeat	160049	NM_001310481	83925	Mm.30466	NM_032000	ENSMUSG00000038679	**Trps1**	AI115454|AI447310|D15Ertd586e	trichorhinophalangeal syndrome I (human)	Amp	protein-coding
1060_T12,T1,T22,T2,T32,T3	chr15	5.1E+07	5.1E+07	999	+	**6**	**75**	NA	intron (NM_001310485, intron 5 of 7)	Lx7|LINE|L1	80049	NM_001310481	83925	Mm.30466	NM_032000	ENSMUSG00000038679	**Trps1**	AI115454|AI447310|D15Ertd586e	trichorhinophalangeal syndrome I (human)	Amp	protein-coding
2342_T22,T2,T32,T3,T42,T4	chr3	5.8E+07	5.8E+07	999	+	**6**	**75**	NA	intron (NM_001081229, intron 1 of 3)	intron (NM_001081229, intron 1 of 3)	7311	NM_001081229	72033	Mm.218409	NM_001081229	ENSMUSG00000027806	**Tsc22d2**	1810043J12Rik|5530402M19Rik	TSC22 domain family, member 2	Amp	protein-coding
2347_T12,T1,T22,T2,T32,T3	chr3	5.8E+07	5.8E+07	999	+	**6**	**75**	NA	intron (NM_001081229, intron 2 of 3)	intron (NM_001081229, intron 2 of 3)	18311	NM_001081229	72033	Mm.218409	NM_001081229	ENSMUSG00000027806	**Tsc22d2**	1810043J12Rik|5530402M19Rik	TSC22 domain family, member 2	Amp	protein-coding
2655_T22,T2,T32,T3,T42,T4	chr4	9.4E+07	9.4E+07	5999	+	**6**	**75**	NA	Intergenic	Intergenic	-480989	NM_026954	69136	Mm.171558	NM_026954	ENSMUSG00000054000	**Tusc1**	2200001D17Rik|TSG-9	tumor suppressor candidate 1	Amp	protein-coding
3355_T1,T22,T2,T32,T3,T4	chr7	5.9E+07	5.9E+07	3999	+	**6**	**75**	NA	intron (NM_001033962, intron 3 of 10)	intron (NM_001033962, intron 3 of 10)	46748	NM_011668	22215	Mm.9002	NM_011668	ENSMUSG00000025326	**Ube3a**	4732496B02|5830462N02Rik|A130086L21Rik|Hpve6a|mKIAA4216	ubiquitin protein ligase E3A	Amp	protein-coding
3439_T12,T1,T22,T2,T3,T42	chr8	3E+07	3E+07	999	+	**6**	**75**	NA	Intergenic	Intergenic	-517364	NM_153135	210801	Mm.248433	NM_153135	ENSMUSG00000063626	**Unc5d**	D930029E11Rik|Unc5h4|mKIAA1777	unc-5 netrin receptor D	Amp	protein-coding
1697_T12,T22,T2,T32,T3,T42	chr3	1.6E+07	1.6E+07	999	+	**6**	**75**	NA	intron (NR_027375, intron 5 of 5)	intron (NR_027375, intron 5 of 5)	24817	NM_001145919	229096	Mm.23834	NM_172677	ENSMUSG00000047213	**Ythdf3**	9130022A11Rik	YTH domain family 3	Amp	protein-coding
1382_T22,T2,T3,T42,T4	chr19	5.7E+07	5.7E+07	173999	+	**5**	**62.5**	NA	intron (NM_001290813, intron 6 of 20)	intron (NM_001290813, intron 6 of 20)	34524	NM_001103178	226251	Mm.217161	NM_178688	ENSMUSG00000025085	**Ablim1**	2210411C18Rik|2610209L21Rik|4833406P10Rik|9330196J19Rik|AV079770|AW060987|AW215784|Limab1|mKIAA0059	actin-binding LIM protein 1	Amp	protein-coding

1321_T12,T22,T32,T42,T4	chr17	9.4E+07	9.4E+07	999	+	**5**	**62.5**	NA	Intergenic	Intergenic	385832	NM_009625	11516	Mm.3407	NM_009625	ENSMUSG00000024256	**Adcyap1**	PACAP	adenylate cyclase activating polypeptide 1	Amp	protein-coding
1323_T12,T1,T22,T2,T42	chr17	9.4E+07	9.4E+07	999	+	**5**	**62.5**	NA	Intergenic	Intergenic	436832	NM_009625	11516	Mm.3407	NM_009625	ENSMUSG00000024256	**Adcyap1**	PACAP	adenylate cyclase activating polypeptide 1	Amp	protein-coding
2765_T12,T1,T22,T3,T4	chr5	5E+07	5E+07	999	+	**5**	**62.5**	NA	Intergenic	Intergenic	381996	NM_133911	70693	Mm.272974	NM_133911	ENSMUSG00000029090	**Adgra3**	3830613O22Rik|AU044632|Gpr125|Tem5-like	adhesion G protein-coupled receptor A3	Amp	protein-coding
2858_T12,T1,T32,T42,T4	chr5	8.1E+07	8.1E+07	4999	+	**5**	**62.5**	NA	Intergenic	Lx9|LINE|L1	-406593	NM_198702	319387	Mm.273631	NM_198702	ENSMUSG00000037605	**Adgrl3**	5430402I23Rik|CIRL-3|D130075K09Rik|Gm1379|LEC3|Lphn3|mKIAA0768	adhesion G protein-coupled receptor L3	Amp	protein-coding
3525_T12,T1,T22,T2,T4	chr8	5.2E+07	5.2E+07	999	+	**5**	**62.5**	NA	Intergenic	Intergenic	-1E+06	NM_001205054	11593	Mm.334535	NM_001005847	ENSMUSG00000031521	**Aga**	AW060726	aspartylglucosaminidase	Amp	protein-coding
3533_T2,T32,T3,T42,T4	chr8	5.3E+07	5.3E+07	999	+	**5**	**62.5**	NA	Intergenic	Intergenic	-783702	NM_001205054	11593	Mm.334535	NM_001005847	ENSMUSG00000031521	**Aga**	AW060726	aspartylglucosaminidase	Amp	protein-coding
3539_T1,T22,T2,T32,T42	chr8	5.3E+07	5.3E+07	999	+	**5**	**62.5**	NA	Intergenic	MLTR11A|LTR|ERVK	-335702	NM_001205054	11593	Mm.334535	NM_001005847	ENSMUSG00000031521	**Aga**	AW060726	aspartylglucosaminidase	Amp	protein-coding
395_T12,T1,T2,T32,T42	chr11	6.1E+07	6.1E+07	37999	+	**5**	**62.5**	NA	Intergenic	B2_Mm2|SINE|B2	73817	NM_172943	268420	Mm.262056	NM_172943	ENSMUSG00000042650	**Alkbh5**	AW050020|AW215868|Abh5|E130207K11|Ofoxd	alkB homolog 5, RNA demethylase	Amp	protein-coding
2991_T12,T1,T22,T2,T42	chr6	1.9E+07	1.9E+07	999	+	**5**	**62.5**	NA	Intergenic	L1Md_T|LINE|L1	121682	NM_001167757	75196	Mm.67665	NM_029202	ENSMUSG00000029517	**Ankrd7**	4930532L20Rik|AV278427	ankyrin repeat domain 7	Amp	protein-coding
2993_T22,T2,T3,T42,T4	chr6	1.9E+07	1.9E+07	999	+	**5**	**62.5**	NA	Intergenic	L1Md_F3|LINE|L1	123682	NM_001167757	75196	Mm.67665	NM_029202	ENSMUSG00000029517	**Ankrd7**	4930532L20Rik|AV278427	ankyrin repeat domain 7	Amp	protein-coding
544_T1,T22,T2,T3,T42	chr13	8E+07	8E+07	4999	+	**5**	**62.5**	NA	Intergenic	L1MB1|LINE|L1	-697422	NM_001042591	105171	Mm.423137	NM_001042591	ENSMUSG00000074794	**Arrdc3**	AI450344|mKIAA1376	arrestin domain containing 3	Amp	protein-coding
1389_T22,T2,T3,T42,T4	chr19	5.8E+07	5.8E+07	305999	+	**5**	**62.5**	NA	intron (NM_181415, intron 10 of 28)	intron (NM_181415, intron 10 of 28)	62466	NM_181415	226255	Mm.245340	NM_181415	ENSMUSG00000054843	**Atrnl1**	AI504415|AW555641|Alp|Atrnl|Atrnlp	attractin like 1	Amp	protein-coding
163_T12,T1,T2,T42,T4	chr1	1.4E+08	1.4E+08	999	+	**5**	**62.5**	NA	Intergenic	B1_Mur3|SINE|Alu	-1E+06	NM_020025	26878	Mm.285580	NM_020025	ENSMUSG00000033849	**B3galt2**	-	UDP-Gal:betaGlcNAc beta 1,3-galactosyltransferase, polypeptide 2	Amp	protein-coding
189_T1,T22,T2,T42,T4	chr1	1.5E+08	1.5E+08	999	+	**5**	**62.5**	NA	Intergenic	Intergenic	622313	NM_001145807	215378	Mm.441817	NM_153539	ENSMUSG00000035131	**Brinp3**	B830045N13Rik|Fam5c	bone morphogenetic protein/retinoic acid inducible neural specific 3	Amp	protein-coding

181_T12,T1, T2,T32,T42	chr1	1.5E+08	1.5E+08	999	+	**5**	**62.5**	NA	intron (NM_001145807, intron 4 of 7)	intron (NM_001145807, intron 4 of 7)	208313	NM_001145807	215378	Mm.441817	NM_153539	ENSMUSG00000035131	**Brinp3**	B830045N13Rik|Fam5c	bone morphogenetic protein/retinoic acid inducible neural specific 3	Amp	protein-coding
1418_T22,T2,T3,T42,T4	chr19	6.1E+07	6.1E+07	121999	+	**5**	**62.5**	NA	promoter-TSS (NM_001172096)	promoter-TSS (NM_001172096)	-477	NM_001172096	78832	Mm.259026	NM_030197	ENSMUSG00000033417	**Cacul1**	2700078E11Rik|2810417M16Rik|9830127L17Rik|AI450346|D130033C15Rik	CDK2 associated, cullin domain 1	Amp	protein-coding
1374_T22,T2,T3,T42,T4	chr19	5.6E+07	5.6E+07	4999	+	**5**	**62.5**	NA	intron (NM_007611, intron 2 of 6)	L1Md_T|LINE|L1	15871	NM_007611	12369	Mm.35687	NM_007611	ENSMUSG00000025076	**Casp7**	AI314680|CMH-1|ICE-IAP3|Mch3|caspase-7|mCASP-7	caspase 7	Amp	protein-coding
1393_T22,T2,T3,T42,T4	chr19	5.8E+07	5.9E+07	948999	+	**5**	**62.5**	NA	Intergenic	Lx5c|LINE|L1	-22002	NM_029372	75645	Mm.159542	NM_029372	ENSMUSG00000025090	**Ccdc172**	1700011F14Rik	coiled-coil domain containing 172	Amp	protein-coding
925_T1,T22, T32,T3,T4	chr15	1.9E+07	1.9E+07	4999	+	**5**	**62.5**	NA	Intergenic	Intergenic	-76947	NM_001316758	320873	Mm.117794	NM_009865	ENSMUSG00000022321	**Cdh10**	A830016G23Rik|C030003B10Rik|C030011H18Rik	cadherin 10	Amp	protein-coding
3646_T1,T22,T2,T32,T4	chr8	1E+08	1E+08	11999	+	**5**	**62.5**	NA	Intergenic	MurERV4_19-int|LTR|ERVK	1593611	NM_009866	12552	Mm.1571	NM_009866	ENSMUSG00000031673	**Cdh11**	Cad11|OSF-4	cadherin 11	Amp	protein-coding
3651_T1,T22,T32,T3,T42	chr8	1E+08	1E+08	2999	+	**5**	**62.5**	NA	Intergenic	Intergenic	1297111	NM_009866	12552	Mm.1571	NM_009866	ENSMUSG00000031673	**Cdh11**	Cad11|OSF-4	cadherin 11	Amp	protein-coding
3652_T1,T22,T2,T3,T4	chr8	1E+08	1E+08	999	+	**5**	**62.5**	NA	Intergenic	Intergenic	1267111	NM_009866	12552	Mm.1571	NM_009866	ENSMUSG00000031673	**Cdh11**	Cad11|OSF-4	cadherin 11	Amp	protein-coding
3661_T1,T22,T2,T42,T4	chr8	1E+08	1E+08	11999	+	**5**	**62.5**	NA	Intergenic	Intergenic	762611	NM_009866	12552	Mm.1571	NM_009866	ENSMUSG00000031673	**Cdh11**	Cad11|OSF-4	cadherin 11	Amp	protein-coding
3669_T12,T1,T2,T32,T4	chr8	1E+08	1E+08	3999	+	**5**	**62.5**	NA	Intergenic	Intergenic	160611	NM_009866	12552	Mm.1571	NM_009866	ENSMUSG00000031673	**Cdh11**	Cad11|OSF-4	cadherin 11	Amp	protein-coding
102_T12,T1, T22,T2,T4	chr1	1E+08	1E+08	999	+	**5**	**62.5**	NA	Intergenic	Intergenic	-1E+06	NM_011800	23836	Mm.103640	NM_011800	ENSMUSG00000050840	**Cdh20**	Cdh7	cadherin 20	Amp	protein-coding
883_T22,T2, T32,T3,T4	chr15	1.4E+07	1.4E+07	3999	+	**5**	**62.5**	NA	Intergenic	Intergenic	-791825	NM_007666	12563	Mm.57048	NM_007666	ENSMUSG00000039385	**Cdh6**	cad6	cadherin 6	Amp	protein-coding
893_T12,T1, T22,T42,T4	chr15	1.5E+07	1.5E+07	3999	+	**5**	**62.5**	NA	Intergenic	Intergenic	-1E+06	NM_009869	12565	Mm.439758	NM_009869	ENSMUSG00000025370	**Cdh9**	-	cadherin 9	Amp	protein-coding
901_T1,T22, T32,T3,T42	chr15	1.6E+07	1.6E+07	999	+	**5**	**62.5**	NA	Intergenic	Intergenic	-802101	NM_009869	12565	Mm.439758	NM_009869	ENSMUSG00000025370	**Cdh9**	-	cadherin 9	Amp	protein-coding

3101_T12,T1,T2,T32,T4	chr6	1E+08	1E+08	3999	+	**5**	**62.5**	NA	intron (NM_017383, intron 7 of 22)	intron (NM_017383, intron 7 of 22)	237456	NM_017383	53870	Mm.321671	NM_017383	ENSMUSG00000030092	**Cntn6**	NB-3	contactin 6	Amp	protein-coding
2469_T12,T1,T2,T32,T4	chr3	1.1E+08	1.1E+08	4999	+	**5**	**62.5**	NA	intron (NM_007729, intron 4 of 66)	B1_Mur3|SINE|Alu	40460	NM_007729	12814	Mm.209715	NM_007729	ENSMUSG00000027966	**Col11a1**	C530001D20Rik|cho	collagen, type XI, alpha 1	Amp	protein-coding
1312_T12,T1,T32,T42,T4	chr17	7.7E+07	7.7E+07	999	+	**5**	**62.5**	NA	Intergenic	Intergenic	-978248	NM_015800	50766	Mm.311912	NM_015800	ENSMUSG00000024074	**Crim1**	AU015004	cysteine rich transmembrane BMP regulator 1 (chordin like)	Amp	protein-coding
1028_T1,T2,T3,T42,T4	chr15	4.9E+07	4.9E+07	999	+	**5**	**62.5**	NA	Intergenic	Intergenic	-346011	NM_001081391	239420	Mm.309363	NM_001081391	ENSMUSG00000022311	**Csmd3**	4930500N14Rik|mKIAA1894	CUB and Sushi multiple domains 3	Amp	protein-coding
1026_T12,T1,T22,T32,T42	chr15	4.9E+07	4.9E+07	1999	+	**5**	**62.5**	NA	intron (NM_001081391, intron 1 of 70)	intron (NM_001081391, intron 1 of 70)	31489	NM_001081391	239420	Mm.309363	NM_001081391	ENSMUSG00000022311	**Csmd3**	4930500N14Rik|mKIAA1894	CUB and Sushi multiple domains 3	Amp	protein-coding
1016_T12,T2,T32,T3,T42	chr15	4.8E+07	4.8E+07	999	+	**5**	**62.5**	NA	intron (NM_001081391, intron 13 of 70)	GA-rich|Low_complexity|Low_complexity	647989	NM_001081391	239420	Mm.309363	NM_001081391	ENSMUSG00000022311	**Csmd3**	4930500N14Rik|mKIAA1894	CUB and Sushi multiple domains 3	Amp	protein-coding
1018_T12,T1,T22,T32,T3	chr15	4.8E+07	4.8E+07	999	+	**5**	**62.5**	NA	intron (NM_001081391, intron 13 of 70)	intron (NM_001081391, intron 13 of 70)	642989	NM_001081391	239420	Mm.309363	NM_001081391	ENSMUSG00000022311	**Csmd3**	4930500N14Rik|mKIAA1894	CUB and Sushi multiple domains 3	Amp	protein-coding
2962_T1,T32,T3,T42,T4	chr6	1.9E+07	1.9E+07	999	+	**5**	**62.5**	NA	Intergenic	Intergenic	-120175	NM_080285	30785	Mm.224189	NM_080285	ENSMUSG00000000416	**Cttnbp2**	3010022N24Rik|4732477G22Rik|6430526E05|9130022E09Rik|AU040881|Cortbp2|ORF4|mKIAA1758	cortactin binding protein 2	Amp	protein-coding
878_T1,T22,T2,T3,T42	chr15	6127501	6128500	999	+	**5**	**62.5**	NA	Intergenic	Intergenic	-171789	NM_001008702	13132	Mm.240830	NM_023118	ENSMUSG00000022150	**Dab2**	5730435J12Rik|AA960054|AI957090|D15Wsu122e|D630005B22Rik|Doc-2|Doc2|p96	disabled 2, mitogen-responsive phosphoprotein	Amp	protein-coding
2238_T12,T1,T2,T42,T4	chr3	5.6E+07	5.6E+07	2999	+	**5**	**62.5**	NA	Intergenic	L1Md_T|LINE|L1	142242	NM_001195540	13175	Mm.393242	NM_019978	ENSMUSG00000027797	**Dclk1**	1700113D08Rik|2810480F11Rik|AI836758|Click-I|Cpg16|Dcamkl1|Dcl|Dclk|mKIAA0369	doublecortin-like kinase 1	Amp	protein-coding
126_T12,T22,T2,T42,T4	chr1	1.1E+08	1.1E+08	999	+	**5**	**62.5**	NA	Intergenic	Intergenic	-944082	NM_001081316	319901	Mm.103468	NM_001081316	ENSMUSG00000038702	**Dsel**	9330132E09Rik|DS-epi2	dermatan sulfate epimerase-like	Amp	protein-coding

<table>
<tr><td>128_T12,T1,T22,T2,T4</td><td>chr1</td><td>1.1E+08</td><td>1.1E+08</td><td>999</td><td>+</td><td>5</td><td>62.5</td><td>NA</td><td>Intergenic</td><td>MLTR14|LTR|ERV1</td><td>-949082</td><td>NM_001081316</td><td>319901</td><td>Mm.103468</td><td>NM_001081316</td><td>ENSMUSG00000038702</td><td>Dsel</td><td>9330132E09Rik|DS-epi2</td><td>dermatan sulfate epimerase-like</td><td>Amp</td><td>protein-coding</td></tr>
<tr><td>137_T1,T32,T3,T42,T4</td><td>chr1</td><td>1.1E+08</td><td>1.1E+08</td><td>999</td><td>+</td><td>5</td><td>62.5</td><td>NA</td><td>Intergenic</td><td>Intergenic</td><td>-1E+06</td><td>NM_001081316</td><td>319901</td><td>Mm.103468</td><td>NM_001081316</td><td>ENSMUSG00000038702</td><td>Dsel</td><td>9330132E09Rik|DS-epi2</td><td>dermatan sulfate epimerase-like</td><td>Amp</td><td>protein-coding</td></tr>
<tr><td>574_T1,T2,T3,T42,T4</td><td>chr13</td><td>8.8E+07</td><td>8.8E+07</td><td>4999</td><td>+</td><td>5</td><td>62.5</td><td>NA</td><td>Intergenic</td><td>Lx4A|LINE|L1</td><td>-574472</td><td>NM_010103</td><td>13612</td><td>Mm.125580</td><td>NM_010103</td><td>ENSMUSG00000034488</td><td>Edil3</td><td>Del-1|Del1</td><td>EGF-like repeats and discoidin I-like domains 3</td><td>Amp</td><td>protein-coding</td></tr>
<tr><td>1292_T12,T2,T32,T42,T4</td><td>chr17</td><td>6.2E+07</td><td>6.2E+07</td><td>999</td><td>+</td><td>5</td><td>62.5</td><td>NA</td><td>Intergenic</td><td>Intergenic</td><td>1036317</td><td>NM_207654</td><td>13640</td><td>Mm.7978</td><td>NM_010109</td><td>ENSMUSG00000048915</td><td>Efna5</td><td>AL-1|AV158822|EFL-5|Ephrin-A5|Epl7|LERK-7|RAGS</td><td>ephrin A5</td><td>Amp</td><td>protein-coding</td></tr>
<tr><td>1398_T22,T2,T3,T42,T4</td><td>chr19</td><td>5.9E+07</td><td>6E+07</td><td>377999</td><td>+</td><td>5</td><td>62.5</td><td>NA</td><td>Intergenic</td><td>Intergenic</td><td>44135</td><td>NR_002863</td><td>329078</td><td>Mm.80382</td><td>NR_002863</td><td>ENSMUSG00000087095</td><td>Emx2os</td><td>-</td><td>Emx2 opposite strand/antisense transcript (non-protein coding)</td><td>Amp</td><td>ncRNA</td></tr>
<tr><td>1394_T22,T2,T3,T42,T4</td><td>chr19</td><td>5.9E+07</td><td>5.9E+07</td><td>26999</td><td>+</td><td>5</td><td>62.5</td><td>NA</td><td>intron (NM_175172, intron 14 of 14)</td><td>(CA)n|Simple_repeat|Simple_repeat</td><td>38575</td><td>NM_178689</td><td>226265</td><td>Mm.103154</td><td>NM_178689</td><td>ENSMUSG00000048029</td><td>Eno4</td><td>6430537H07Rik</td><td>enolase 4</td><td>Amp</td><td>protein-coding</td></tr>
<tr><td>2869_T1,T22,T2,T32,T42</td><td>chr5</td><td>8.5E+07</td><td>8.5E+07</td><td>999</td><td>+</td><td>5</td><td>62.5</td><td>NA</td><td>Intergenic</td><td>MTD|LTR|ERVL-MaLR</td><td>-177618</td><td>NM_007937</td><td>13839</td><td>Mm.137991</td><td>NM_007937</td><td>ENSMUSG00000029245</td><td>Epha5</td><td>AI854630|AW125296|Cek7|Ehk1|Els1|Hek7|Rek7|bsk</td><td>Eph receptor A5</td><td>Amp</td><td>protein-coding</td></tr>
<tr><td>1166_T12,T22,T32,T42,T4</td><td>chr16</td><td>6.1E+07</td><td>6.1E+07</td><td>999</td><td>+</td><td>5</td><td>62.5</td><td>NA</td><td>Intergenic</td><td>L1Md_F2|LINE|L1</td><td>-670469</td><td>NM_007938</td><td>13840</td><td>Mm.455790</td><td>NM_007938</td><td>ENSMUSG00000055540</td><td>Epha6</td><td>Ehk2|Hek12|m-ehk2</td><td>Eph receptor A6</td><td>Amp</td><td>protein-coding</td></tr>
<tr><td>42_T12,T22,T2,T32,T4</td><td>chr1</td><td>6.8E+07</td><td>6.8E+07</td><td>3999</td><td>+</td><td>5</td><td>62.5</td><td>NA</td><td>intron (NM_010154, intron 3 of 26)</td><td>intron (NM_010154, intron 3 of 26)</td><td>711559</td><td>NM_010154</td><td>13869</td><td>Mm.442420</td><td>NM_010154</td><td>ENSMUSG00000062209</td><td>Erbb4</td><td>Her4|c-erbB-4</td><td>erb-b2 receptor tyrosine kinase 4</td><td>Amp</td><td>protein-coding</td></tr>
<tr><td>426_T1,T22,T32,T3,T42</td><td>chr12</td><td>3.9E+07</td><td>3.9E+07</td><td>999</td><td>+</td><td>5</td><td>62.5</td><td>NA</td><td>Intergenic</td><td>MTC|LTR|ERVL-MaLR</td><td>330289</td><td>NM_001163154</td><td>14009</td><td>Mm.4866</td><td>NM_007960</td><td>ENSMUSG00000004151</td><td>Etv1</td><td>ER81|Etsrp81</td><td>ets variant 1</td><td>Amp</td><td>protein-coding</td></tr>
<tr><td>1412_T22,T2,T3,T42,T4</td><td>chr19</td><td>6E+07</td><td>6E+07</td><td>258999</td><td>+</td><td>5</td><td>62.5</td><td>NA</td><td>intron (NM_029648, intron 5 of 7)</td><td>intron (NM_029648, intron 5 of 7)</td><td>8697</td><td>NM_029648</td><td>76539</td><td>Mm.290183</td><td>NM_029648</td><td>ENSMUSG00000057858</td><td>Fam204a</td><td>2310065H12Rik|2610015K05Rik|D19Ertd737e</td><td>family with sequence similarity 204, member A</td><td>Amp</td><td>protein-coding</td></tr>
<tr><td>2205_T1,T32,T3,T42,T4</td><td>chr3</td><td>5.2E+07</td><td>5.2E+07</td><td>999</td><td>+</td><td>5</td><td>62.5</td><td>NA</td><td>Intergenic</td><td>Intergenic</td><td>160663</td><td>NM_019739</td><td>56458</td><td>Mm.29891</td><td>NM_019739</td><td>ENSMUSG00000044167</td><td>Foxo1</td><td>AI876417|Afxh|FKHR|Fkhr1|Foxo1a</td><td>forkhead box O1</td><td>Amp</td><td>protein-coding</td></tr>
<tr><td>237_T12,T1,T2,T32,T3</td><td>chr10</td><td>3.5E+07</td><td>3.5E+07</td><td>3999</td><td>+</td><td>5</td><td>62.5</td><td>NA</td><td>Intergenic</td><td>Intergenic</td><td>367100</td><td>NM_010237</td><td>14302</td><td>Mm.332432</td><td>NM_010237</td><td>ENSMUSG00000019779</td><td>Frk</td><td>BSK|BSK/IYK|C85044|GTK|RAK</td><td>fyn-related kinase</td><td>Amp</td><td>protein-coding</td></tr>
<tr><td>2414_T1,T22,T32,T3,T4</td><td>chr3</td><td>7.7E+07</td><td>7.7E+07</td><td>3999</td><td>+</td><td>5</td><td>62.5</td><td>NA</td><td>Intergenic</td><td>Intergenic</td><td>1414917</td><td>NM_001253719</td><td>213262</td><td>Mm.379337</td><td>NM_178673</td><td>ENSMUSG00000034098</td><td>Fstl5</td><td>9130207J01Rik</td><td>follistatin-like 5</td><td>Amp</td><td>protein-coding</td></tr>
</table>

2393_T12,T22,T32,T3,T4	chr3	7.6E+07	7.6E+07	3999	+	**5**	**62.5**	NA	intron (NM_178673, intron 3 of 15)	intron (NM_178673, intron 3 of 15)	197917	NM_001253719	213262	Mm.379337	NM_178673	ENSMUSG00000034098	**Fstl5**	9130207J01Rik	follistatin-like 5	Amp	protein-coding
870_T12,T22,T3,T42,T4	chr15	3570501	3574500	3999	+	**5**	**62.5**	NA	intron (NM_010284, intron 1 of 10)	intron (NM_010284, intron 1 of 10)	9342	NM_001286370	14600	Mm.3986	NM_010284	ENSMUSG00000055737	**Ghr**	GHBP|GHR/BP	growth hormone receptor	Amp	protein-coding
316_T12,T1,T22,T32,T42	chr10	7.5E+07	7.5E+07	5999	+	**5**	**62.5**	NA	Intergenic	Intergenic	-27731	NM_010311	14687	Mm.32595	NM_010311	ENSMUSG00000040009	**Gnaz**	AI847979|Gz	guanine nucleotide binding protein, alpha z subunit	Amp	protein-coding
2697_T12,T22,T2,T32,T42	chr5	1E+07	1E+07	999	+	**5**	**62.5**	NA	Intergenic	Intergenic	-285648	NM_181850	108069	Mm.318966	NM_181850	ENSMUSG00000003974	**Grm3**	0710001G23Rik|Gprc1c|mGlu3|mGluR3	glutamate receptor, metabotropic 3	Amp	protein-coding
1370_T22,T2,T3,T42,T4	chr19	5.6E+07	5.6E+07	90999	+	**5**	**62.5**	NA	intron (NM_001286552, intron 36 of 39)	intron (NM_001286552, intron 36 of 39)	37062	NM_146101	226243	Mm.25791	NM_146101	ENSMUSG00000025075	**Habp2**	AI035669|FSAP|HABP|HGFAL|PHBP	hyaluronic acid binding protein 2	Amp	protein-coding
580_T1,T22,T2,T3,T4	chr13	8.9E+07	8.9E+07	5999	+	**5**	**62.5**	NA	intron (NM_010103, intron 9 of 9)	intron (NM_010103, intron 9 of 9)	-229136	NM_013500	12950	Mm.266790	NM_013500	ENSMUSG00000021613	**Hapln1**	BB099155|CLP|Crtl1|Crtl1l|LP|LP-1	hyaluronan and proteoglycan link protein 1	Amp	protein-coding
1071_T1,T22,T2,T42,T4	chr15	5.7E+07	5.7E+07	999	+	**5**	**62.5**	NA	Intergenic	Lx6|LINE|L1	-279454	NM_008216	15117	Mm.5148	NM_008216	ENSMUSG00000022367	**Has2**	-	hyaluronan synthase 2	Amp	protein-coding
2446_T1,T22,T32,T3,T42	chr3	9.3E+07	9.3E+07	999	+	**5**	**62.5**	NA	Intergenic	Intergenic	-27749	NM_133698	68723	Mm.208047	NM_133698	ENSMUSG00000041991	**Hrnr**	1110033K19Rik|A530063N20Rik|S100a18	hornerin	Amp	protein-coding
597_T22,T32,T3,T42,T4	chr13	1.2E+08	1.2E+08	999	+	**5**	**62.5**	NA	Intergenic	HAL1|LINE|L1	391688	NM_021459	16392	Mm.42242	NM_021459	ENSMUSG00000042258	**Isl1**	-	ISL1 transcription factor, LIM/homeodomain	Amp	protein-coding
1580_T1,T22,T2,T3,T42	chr2	1.1E+08	1.1E+08	999	+	**5**	**62.5**	NA	Intergenic	ID_B1|SINE|B4	848411	NM_021275	16492	Mm.142718	NM_021275	ENSMUSG00000042604	**Kcna4**	Kv1.4	potassium voltage-gated channel, shaker-related subfamily, member 4	Amp	protein-coding
3035_T22,T2,T3,T42,T4	chr6	2.1E+07	2.1E+07	999	+	**5**	**62.5**	NA	Intergenic	L1Md_T|LINE|L1	-684109	NM_019697	16508	Mm.425316	NM_019697	ENSMUSG00000060882	**Kcnd2**	AI839615|AW555701|Kv4.2|R75121|mKIAA1044	potassium voltage-gated channel, Shal-related family, member 2	Amp	protein-coding
1492_T22,T2,T32,T3,T4	chr2	5.5E+07	5.5E+07	5999	+	**5**	**62.5**	NA	3' UTR (NM_173030, exon 13 of 13)	3' UTR (NM_173030, exon 13 of 13)	-318470	NM_001304810	16519	Mm.5127	NM_008426	ENSMUSG00000026824	**Kcnj3**	GIRK-1|GIRK1|Kcnf3|Kir3.1	potassium inwardly-rectifying channel, subfamily J, member 3	Amp	protein-coding
986_T12,T22,T32,T42,T4	chr15	4.5E+07	4.5E+07	3999	+	**5**	**62.5**	NA	Intergenic	Intergenic	-125566	NM_026200	67498	Mm.300079	NM_026200	ENSMUSG00000022342	**Kcnv1**	2700023A03Rik|vibe	potassium channel, subfamily V, member 1	Amp	protein-coding

2828_T12,T1,T22,T32,T4	chr5	6.9E+07	6.9E+07	999	+	**5**	**62.5**	NA	intron (NM_175519, intron 1 of 1)	B4A|SINE|B4	91709	NM_175519	243043	Mm.234821	NM_175519	ENSMUSG00000037653	**Kctd8**	A730087N02Rik	potassium channel tetramerisation domain containing 8	Amp	protein-coding
2830_T12,T1,T22,T32,T4	chr5	6.9E+07	6.9E+07	999	+	**5**	**62.5**	NA	intron (NM_175519, intron 1 of 1)	intron (NM_175519, intron 1 of 1)	86709	NM_175519	243043	Mm.234821	NM_175519	ENSMUSG00000037653	**Kctd8**	A730087N02Rik	potassium channel tetramerisation domain containing 8	Amp	protein-coding
397_T12,T1,T2,T32,T42	chr11	6.1E+07	6.1E+07	11999	+	**5**	**62.5**	NA	Intergenic	Intergenic	-55190	NM_001159405	16897	Mm.285453	NM_008502	ENSMUSG00000020536	**Llgl1**	AI325176|Lgl1|Llglh|Mgl1	lethal giant larvae homolog 1 (Drosophila)	Amp	protein-coding
1559_T1,T2,T32,T42,T4	chr2	9.8E+07	9.8E+07	1999	+	**5**	**62.5**	NA	Intergenic	Intergenic	165843	NM_178725	241568	Mm.241682	NM_178725	ENSMUSG00000050587	**Lrrc4c**	6430556C10Rik|NGL-1	leucine rich repeat containing 4C	Amp	protein-coding
2512_T1,T22,T32,T42,T4	chr3	1.6E+08	1.6E+08	3999	+	**5**	**62.5**	NA	intron (NM_001081358, intron 3 of 27)	L1MA9|LINE|L1	177721	NM_001291453	242274	Mm.132162	NM_001081358	ENSMUSG00000028176	**Lrrc7**	AK122498|densin|mKIAA1365	leucine rich repeat containing 7	Amp	protein-coding
3086_T12,T1,T2,T32,T4	chr6	8.1E+07	8.1E+07	5999	+	**5**	**62.5**	NA	intron (NM_001134743, intron 2 of 2)	intron (NM_001134743, intron 2 of 2)	720857	NM_178731	243499	Mm.483425	NM_178731	ENSMUSG00000052581	**Lrrtm4**	7530419J18Rik|A230052N11	leucine rich repeat transmembrane neuronal 4	Amp	protein-coding
2493_T1,T22,T2,T3,T4	chr3	1.4E+08	1.4E+08	2999	+	**5**	**62.5**	NA	3' UTR (NM_025356, exon 8 of 8)	3' UTR (NM_025356, exon 8 of 8)	-18611	NM_027288	110173	Mm.280536	NM_027288	ENSMUSG00000028164	**Manba**	2410030O07Rik|B930014J03Rik|Bmn	mannosidase, beta A, lysosomal	Amp	protein-coding
3764_T12,T1,T22,T2,T3	chr9	8.2E+07	8.2E+07	999	+	**5**	**62.5**	NA	intron (NM_175213, intron 3 of 4)	intron (NM_175213, intron 3 of 4)	114256	NM_175213	75033	Mm.335329	NM_175213	ENSMUSG00000043289	**Mei4**	4930486G11Rik	meiotic double-stranded break formation protein 4	Amp	protein-coding
3462_T12,T1,T22,T2,T3	chr8	3.8E+07	3.8E+07	3999	+	**5**	**62.5**	NA	intron (NM_145841, intron 1 of 7)	intron (NM_145841, intron 1 of 7)	247702	NR_029884	723860		NR_029884	ENSMUSG00000065457	**Mir383**	Mirn383|mir-383|mmu-mir-383	microRNA 383	Amp	ncRNA
3470_T12,T1,T22,T2,T42	chr8	3.8E+07	3.8E+07	999	+	**5**	**62.5**	NA	intron (NM_145841, intron 1 of 7)	ORR1D1|LTR|ERVL-MaLR	-98798	NR_029884	723860		NR_029884	ENSMUSG00000065457	**Mir383**	Mirn383|mir-383|mmu-mir-383	microRNA 383	Amp	ncRNA
3472_T12,T1,T2,T32,T42	chr8	3.8E+07	3.8E+07	999	+	**5**	**62.5**	NA	intron (NM_145841, intron 1 of 7)	intron (NM_145841, intron 1 of 7)	-102798	NR_029884	723860		NR_029884	ENSMUSG00000065457	**Mir383**	Mirn383|mir-383|mmu-mir-383	microRNA 383	Amp	ncRNA
3452_T22,T2,T32,T3,T42	chr8	3.8E+07	3.8E+07	5999	+	**5**	**62.5**	NA	intron (NM_145841, intron 2 of 7)	intron (NM_145841, intron 2 of 7)	453702	NR_029884	723860		NR_029884	ENSMUSG00000065457	**Mir383**	Mirn383|mir-383|mmu-mir-383	microRNA 383	Amp	ncRNA
3455_T22,T2,T32,T3,T4	chr8	3.8E+07	3.8E+07	999	+	**5**	**62.5**	NA	intron (NM_145841, intron 2 of 7)	Lx8|LINE|L1	347202	NR_029884	723860		NR_029884	ENSMUSG00000065457	**Mir383**	Mirn383|mir-383|mmu-mir-383	microRNA 383	Amp	ncRNA

3457_T22,T2,T32,T3,T4	chr8	3.8E+07	3.8E+07	3999	+	**5**	**62.5**	NA	intron (NM_145841, intron 2 of 7)	RLTR17B_Mm|LTR|ERVK	340702	NR_029884	723860		NR_029884	ENSMUSG00000065457	**Mir383**	Mirn383|mir-383|mmu-mir-383	microRNA 383	Amp	ncRNA
1422_T22,T2,T3,T42,T4	chr19	6.1E+07	6.1E+07	130999	+	**5**	**62.5**	NA	Intergenic	MTEa|LTR|ERVL-MaLR	-32987	NM_178421	332397	Mm.186390	NM_178421	ENSMUSG00000072437	**Nanos1**	NOS-1	nanos homolog 1 (Drosophila)	Amp	protein-coding
2269_T12,T22,T2,T42,T4	chr3	5.6E+07	5.6E+07	999	+	**5**	**62.5**	NA	Intergenic	Intergenic	-195299	NM_030595	26422	Mm.384353	NM_030595	ENSMUSG00000027799	**Nbea**	Lyst2|mKIAA1544	neurobeachin	Amp	protein-coding
2275_T12,T22,T2,T42,T4	chr3	5.6E+07	5.6E+07	999	+	**5**	**62.5**	NA	Intergenic	Lx5|LINE|L1	-207299	NM_030595	26422	Mm.384353	NM_030595	ENSMUSG00000027799	**Nbea**	Lyst2|mKIAA1544	neurobeachin	Amp	protein-coding
1376_T22,T2,T3,T42,T4	chr19	5.6E+07	5.7E+07	417999	+	**5**	**62.5**	NA	Intergenic	MLT1C|LTR|ERVL-MaLR	77239	NM_025811	66866	Mm.46346	NM_025811	ENSMUSG00000025078	**Nhlrc2**	1200003G01Rik|AI835049|AV002846|AW496455	NHL repeat containing 2	Amp	protein-coding
1737_T1,T22,T32,T3,T4	chr3	2.6E+07	2.6E+07	3999	+	**5**	**62.5**	NA	intron (NM_138666, intron 3 of 7)	intron (NM_138666, intron 3 of 7)	114807	NM_001163387	192167	Mm.316080	NM_138666	ENSMUSG00000063887	**Nlgn1**	6330415N05Rik|BB179718|NL1|Nlg1|mKIAA1070	neuroligin 1	Amp	protein-coding
538_T22,T2,T32,T3,T4	chr13	7.9E+07	7.9E+07	999	+	**5**	**62.5**	NA	Intergenic	Lx6|LINE|L1	-969018	NM_010151	13865	Mm.439653	NM_010151	ENSMUSG00000069171	**Nr2f1**	COUP-TF1|COUP-TFI|COUPTFA|EAR-3|EAR3|Erbal3|SVP44|Tcfcoup1	nuclear receptor subfamily 2, group F, member 1	Amp	protein-coding
1372_T22,T2,T3,T42,T4	chr19	5.6E+07	5.6E+07	37999	+	**5**	**62.5**	NA	promoter-TSS (NM_198059)	promoter-TSS (NM_198059)	-462	NM_001286552	18175	Mm.386769	NM_008733	ENSMUSG00000049134	**Nrap**	-	nebulin-related anchoring protein	Amp	protein-coding
1082_T12,T1,T22,T2,T42	chr15	5.9E+07	5.9E+07	3999	+	**5**	**62.5**	NA	intron (NM_026746, intron 3 of 6)	intron (NM_026746, intron 3 of 6)	59302	NM_001164604	68501	Mm.27762	NM_026746	ENSMUSG00000059586	**Nsmce2**	1110014D18Rik|AI661537	NSE2/MMS21 homolog, SMC5-SMC6 complex SUMO ligase	Amp	protein-coding
2887_T1,T2,T32,T3,T4	chr6	9900501	9901500	999	+	**5**	**62.5**	NA	Intergenic	MERVL_2A-int|LTR|ERVL	950981	NM_008751	18231	Mm.46954	NM_008751	ENSMUSG00000046178	**Nxph1**	C130005L03Rik	neurexophilin 1	Amp	protein-coding
2474_T12,T1,T22,T3,T42	chr3	1.2E+08	1.2E+08	999	+	**5**	**62.5**	NA	Intergenic	Intergenic	60035	NM_153157	229759	Mm.54183	NM_153157	ENSMUSG00000027965	**Olfm3**	B230206G02Rik	olfactomedin 3	Amp	protein-coding
625_T12,T22,T2,T42,T4	chr14	8.1E+07	8.1E+07	999	+	**5**	**62.5**	NA	Intergenic	Intergenic	522698	NM_001030294	380924	Mm.26456	NM_001030294	ENSMUSG00000022026	**Olfm4**	GC1|GW112|Gm296|Gm913|OlfD|pPD4	olfactomedin 4	Amp	protein-coding
629_T12,T1,T22,T32,T3	chr14	8.1E+07	8.1E+07	999	+	**5**	**62.5**	NA	Intergenic	Intergenic	529698	NM_001030294	380924	Mm.26456	NM_001030294	ENSMUSG00000022026	**Olfm4**	GC1|GW112|Gm296|Gm913|OlfD|pPD4	olfactomedin 4	Amp	protein-coding
1922_T1,T22,T2,T3,T42	chr3	4.6E+07	4.6E+07	999	+	**5**	**62.5**	NA	Intergenic	L1_Mus3|LINE|L1	385941	NM_001101479	241989	Mm.119234	NM_001101479	ENSMUSG00000090919	**Pabpc4l**	C330050A14Rik|EG241989	poly(A) binding protein, cytoplasmic 4-like	Amp	protein-coding

1960_T1,T22,T3,T42,T4	chr3	4.7E+07	4.7E+07	999	+	**5**	**62.5**	NA	Intergenic	URR1B|DNA|hAT-Charlie	-219059	NM_001101479	241989	Mm.119234	NM_001101479	ENSMUSG00000090919	**Pabpc4l**	C330050A14Rik|EG241989	poly(A) binding protein, cytoplasmic 4-like	Amp	protein-coding
311_T1,T2,T3,T42,T4	chr10	7.4E+07	7.4E+07	3999	+	**5**	**62.5**	NA	intron (NM_001142735, intron 1 of 32)	intron (NM_001142735, intron 1 of 32)	15633	NM_001142735	11994	Mm.338933	NM_023115	ENSMUSG00000052613	**Pcdh15**	BB078305|ENSMUSG00000046980|Gm9815|Ush1f|av|nmf19|roda	protocadherin 15	Amp	protein-coding
2114_T1,T2,T3,T42,T4	chr3	4.9E+07	4.9E+07	6999	+	**5**	**62.5**	NA	Intergenic	L1Md_A|LINE|L1	487316	NM_130448	73173	Mm.87246	NM_130448	ENSMUSG00000037892	**Pcdh18**	3110038E07Rik|BB095589|PCDH68L	protocadherin 18	Amp	protein-coding
2136_T1,T22,T2,T3,T4	chr3	5E+07	5E+07	999	+	**5**	**62.5**	NA	Intergenic	Intergenic	-46684	NM_130448	73173	Mm.87246	NM_130448	ENSMUSG00000037892	**Pcdh18**	3110038E07Rik|BB095589|PCDH68L	protocadherin 18	Amp	protein-coding
2139_T1,T22,T32,T3,T42	chr3	5E+07	5E+07	999	+	**5**	**62.5**	NA	Intergenic	L1Md_T|LINE|L1	-60684	NM_130448	73173	Mm.87246	NM_130448	ENSMUSG00000037892	**Pcdh18**	3110038E07Rik|BB095589|PCDH68L	protocadherin 18	Amp	protein-coding
2147_T1,T22,T32,T3,T42	chr3	5E+07	5E+07	999	+	**5**	**62.5**	NA	Intergenic	Lx2|LINE|L1	-303684	NM_130448	73173	Mm.87246	NM_130448	ENSMUSG00000037892	**Pcdh18**	3110038E07Rik|BB095589|PCDH68L	protocadherin 18	Amp	protein-coding
689_T1,T32,T3,T42,T4	chr14	8.8E+07	8.8E+07	4999	+	**5**	**62.5**	NA	Intergenic	Intergenic	213396	NM_178685	219257	Mm.128861	NM_178685	ENSMUSG00000050505	**Pcdh20**	C630015B17Rik|Pcdh13	protocadherin 20	Amp	protein-coding
740_T1,T22,T32,T3,T42	chr14	9.4E+07	9.4E+07	1999	+	**5**	**62.5**	NA	intron (NM_001081377, intron 1 of 3)	intron (NM_001081377, intron 1 of 3)	221388	NM_001271800	211712	Mm.37126	NM_001081377	ENSMUSG00000055421	**Pcdh9**	-	protocadherin 9	Amp	protein-coding
745_T12,T1,T22,T3,T4	chr14	9.4E+07	9.4E+07	2999	+	**5**	**62.5**	NA	intron (NM_001081377, intron 1 of 3)	intron (NM_001081377, intron 1 of 3)	29888	NM_001271800	211712	Mm.37126	NM_001081377	ENSMUSG00000055421	**Pcdh9**	-	protocadherin 9	Amp	protein-coding
2391_T1,T2,T32,T3,T4	chr3	7.6E+07	7.6E+07	999	+	**5**	**62.5**	NA	intron (NM_019745, intron 2 of 7)	intron (NM_019745, intron 2 of 7)	16852	NM_019745	56426	Mm.316473	NM_019745	ENSMUSG00000027835	**Pdcd10**	2410003B13Rik|Ccm3|Tfa15|Tfar15	programmed cell death 10	Amp	protein-coding
2424_T2,T32,T3,T42,T4	chr3	8.1E+07	8.1E+07	4999	+	**5**	**62.5**	NA	Intergenic	Intergenic	376584	NM_019971	54635	Mm.331089	NM_019971	ENSMUSG00000028019	**Pdgfc**	1110064L01Rik|AI647969|PDGF-C	platelet-derived growth factor, C polypeptide	Amp	protein-coding
3683_T12,T1,T22,T2,T42	chr9	5946501	5950500	3999	+	**5**	**62.5**	NA	Intergenic	Intergenic	-220112	NM_027924	71785	Mm.390122	NM_027924	ENSMUSG00000032006	**Pdgfd**	1110003I09Rik	platelet-derived growth factor, D polypeptide	Amp	protein-coding
2437_T12,T22,T32,T42,T4	chr3	9.2E+07	9.2E+07	4999	+	**5**	**62.5**	NA	Intergenic	Intergenic	-198583	NM_207247	242100	Mm.297406	NM_207247	ENSMUSG00000042244	**Pglyrp3**	Gm420	peptidoglycan recognition protein 3	Amp	protein-coding
3340_T1,T32,T3,T42,T4	chr7	3.8E+07	3.8E+07	5999	+	**5**	**62.5**	NA	Intergenic	L1_Mm|LINE|L1	-75152	NM_025390	66161	Mm.22284	NM_025390	ENSMUSG00000030423	**Pop4**	1110023P21Rik|Rpp29	processing of precursor 4, ribonuclease P/MRP family, (S. cerevisiae)	Amp	protein-coding

1414_T22,T2,T3,T42,T4	chr19	6E+07	6E+07	20999	+	**5**	**62.5**	NA	Intergenic	Intergenic	103304	NM_201615	226278	Mm.377241	NM_201615	ENSMUSG00000045052	**Prlhr**	Gm339|Gpr10|Gr3|Prrpr	prolactin releasing hormone receptor	Amp	protein-coding
1416_T22,T2,T3,T42,T4	chr19	6E+07	6.1E+07	142999	+	**5**	**62.5**	NA	Intergenic	B2_Mm2|SINE|B2	20304	NM_201615	226278	Mm.377241	NM_201615	ENSMUSG00000045052	**Prlhr**	Gm339|Gpr10|Gr3|Prrpr	prolactin releasing hormone receptor	Amp	protein-coding
2453_T22,T2,T32,T3,T4	chr3	1.1E+08	1.1E+08	3999	+	**5**	**62.5**	NA	Intergenic	Intergenic	-252501	NR_024139	99890	Mm.36115	NM_178891	ENSMUSG00000049300	**Prmt6**	AW124876|BB233495|Hrmt1l6	protein arginine N-methyltransferase 6	Amp	protein-coding
2460_T12,T1,T22,T2,T4	chr3	1.1E+08	1.1E+08	999	+	**5**	**62.5**	NA	Intergenic	Intergenic	-503001	NR_024139	99890	Mm.36115	NM_178891	ENSMUSG00000049300	**Prmt6**	AW124876|BB233495|Hrmt1l6	protein arginine N-methyltransferase 6	Amp	protein-coding
2462_T1,T32,T3,T42,T4	chr3	1.1E+08	1.1E+08	4999	+	**5**	**62.5**	NA	Intergenic	Intergenic	-549001	NR_024139	99890	Mm.36115	NM_178891	ENSMUSG00000049300	**Prmt6**	AW124876|BB233495|Hrmt1l6	protein arginine N-methyltransferase 6	Amp	protein-coding
2624_T22,T2,T32,T3,T4	chr4	7.8E+07	7.8E+07	999	+	**5**	**62.5**	NA	intron (NM_011211, intron 1 of 39)	B4A|SINE|B4	185895	NM_011211	19266	Mm.184021	NM_011211	ENSMUSG00000028399	**Ptprd**	1110002J03Rik|3000002J10Rik|B230219D21Rik|R-PTP-delta	protein tyrosine phosphatase, receptor type, D	Amp	protein-coding
1405_T22,T2,T3,T42,T4	chr19	6E+07	6E+07	32999	+	**5**	**62.5**	NA	Intergenic	L1_Mus1|LINE|L1	146364	NM_001033172	74998	Mm.24167	NM_001033172	ENSMUSG00000040022	**Rab11fip2**	4930470G04Rik|A830046J09Rik|AW558126|Nrip11	RAB11 family interacting protein 2 (class I)	Amp	protein-coding
1407_T22,T2,T3,T42,T4	chr19	6E+07	6E+07	197999	+	**5**	**62.5**	NA	intron (NM_001033172, intron 3 of 5)	intron (NM_001033172, intron 3 of 5)	23864	NM_001033172	74998	Mm.24167	NM_001033172	ENSMUSG00000040022	**Rab11fip2**	4930470G04Rik|A830046J09Rik|AW558126|Nrip11	RAB11 family interacting protein 2 (class I)	Amp	protein-coding
1678_T1,T22,T32,T3,T42	chr3	1.3E+07	1.3E+07	3999	+	**5**	**62.5**	NA	Intergenic	Intergenic	-371155	NM_178631	76897	Mm.422625	NM_178631	ENSMUSG00000039717	**Ralyl**	0710005M24Rik	RALY RNA binding protein-like	Amp	protein-coding
1328_T1,T22,T2,T3,T4	chr18	1.2E+07	1.2E+07	3999	+	**5**	**62.5**	NA	intron (NR_045527, intron 1 of 17)	MMVL30-int|LTR|ERV1	-11849	NR_045526	225182	Mm.154275	NM_175458	ENSMUSG00000041238	**Rbbp8**	9930104E21Rik|CtIP|RBBP-8|RIM|SAE2	retinoblastoma binding protein 8	Amp	protein-coding
205_T12,T1,T22,T2,T42	chr10	6312501	6313500	999	+	**5**	**62.5**	NA	Intergenic	RMER6D|LTR|ERVK	-390600	NM_019958	56533	Mm.44606	NM_019958	ENSMUSG00000019775	**Rgs17**	6430507P11Rik|Rgsz2	regulator of G-protein signaling 17	Amp	protein-coding
3674_T1,T2,T32,T3,T4	chr8	1.2E+08	1.2E+08	999	+	**5**	**62.5**	NA	Intergenic	Intergenic	-29929	NM_133955	69581	Mm.168257	NM_133955	ENSMUSG00000039960	**Rhou**	2310026M05Rik|AI182090|Arhu|CDC42L1|G28K|WRCH-1|WRCH1|mG28K	ras homolog family member U	Amp	protein-coding
223_T12,T1,T32,T3,T4	chr10	3.2E+07	3.2E+07	999	+	**5**	**62.5**	NA	intron (NM_001013411, intron 3 of 6)	L1_Mus3|LINE|L1	-352275	NM_001146349	268291	Mm.295212	NM_001146349	ENSMUSG00000063760	**Rnf217**	AU016819|Ibrdc1	ring finger protein 217	Amp	protein-coding

225_T12,T22,T2,T3,T4	chr10	3.2E+07	3.2E+07	999	+	**5**	**62.5**	NA	intron (NM_001013411, intron 3 of 6)	MER67D|LTR|ERV1	-356275	NM_001146349	268291	Mm.295212	NM_001146349	ENSMUSG00000063760	**Rnf217**	AU016819|Ibrdc1	ring finger protein 217	Amp	protein-coding
1511_T12,T22,T2,T32,T42	chr2	6.7E+07	6.7E+07	1999	+	**5**	**62.5**	NA	Intergenic	Intergenic	-36538	NM_001290675	20274	Mm.440889	NM_018852	ENSMUSG00000075316	**Scn9a**	Nav1.7|PN1|mKIAA4197	sodium channel, voltage-gated, type IX, alpha	Amp	protein-coding
1396_T22,T2,T3,T42,T4	chr19	5.9E+07	5.9E+07	223999	+	**5**	**62.5**	NA	Intergenic	L1MD1|LINE|L1	-32431	NM_001114312	71653	Mm.37338	NM_175172	ENSMUSG00000041362	**Shtn1**	4930506M07Rik|Kiaa1598|Shootin1|mKIAA1598	shootin 1	Amp	protein-coding
3398_T12,T1,T2,T32,T42	chr8	5025501	5027500	1999	+	**5**	**62.5**	NA	Intergenic	L1_Rod|LINE|L1	78732	NM_011388	20494	Mm.3500	NM_011388	ENSMUSG00000023073	**Slc10a2**	9130221J18Rik|AI605518|ASBT|IBAT|ISBT	solute carrier family 10, member 2	Amp	protein-coding
3411_T1,T32,T3,T42,T4	chr8	6530501	6531500	999	+	**5**	**62.5**	NA	Intergenic	Intergenic	-1E+06	NM_011388	20494	Mm.3500	NM_011388	ENSMUSG00000023073	**Slc10a2**	9130221J18Rik|AI605518|ASBT|IBAT|ISBT	solute carrier family 10, member 2	Amp	protein-coding
2750_T12,T1,T22,T32,T3	chr5	4.7E+07	4.7E+07	999	+	**5**	**62.5**	NA	Intergenic	Intergenic	-528155	NR_111900	20563	Mm.289739	NM_178804	ENSMUSG00000031558	**Slit2**	Drad-1|E030015M03Rik|E130320P19Rik|Slil3|mKIAA4141|slit-2	slit homolog 2 (Drosophila)	Amp	protein-coding
803_T12,T1,T22,T2,T3	chr14	1.1E+08	1.1E+08	999	+	**5**	**62.5**	NA	Intergenic	RMER20A|LTR|ERVK	833239	NM_199065	76965	Mm.257268	NM_199065	ENSMUSG00000075478	**Slitrk1**	3200001I04Rik	SLIT and NTRK-like family, member 1	Amp	protein-coding
819_T12,T22,T2,T32,T3	chr14	1.1E+08	1.1E+08	999	+	**5**	**62.5**	NA	Intergenic	Intergenic	12239	NM_199065	76965	Mm.257268	NM_199065	ENSMUSG00000075478	**Slitrk1**	3200001I04Rik	SLIT and NTRK-like family, member 1	Amp	protein-coding
823_T1,T32,T3,T42,T4	chr14	1.1E+08	1.1E+08	3999	+	**5**	**62.5**	NA	Intergenic	Intergenic	-172261	NM_199065	76965	Mm.257268	NM_199065	ENSMUSG00000075478	**Slitrk1**	3200001I04Rik	SLIT and NTRK-like family, member 1	Amp	protein-coding
825_T12,T22,T2,T3,T42	chr14	1.1E+08	1.1E+08	999	+	**5**	**62.5**	NA	Intergenic	Intergenic	-243761	NM_199065	76965	Mm.257268	NM_199065	ENSMUSG00000075478	**Slitrk1**	3200001I04Rik	SLIT and NTRK-like family, member 1	Amp	protein-coding
827_T12,T22,T2,T3,T42	chr14	1.1E+08	1.1E+08	999	+	**5**	**62.5**	NA	Intergenic	Intergenic	-249761	NM_199065	76965	Mm.257268	NM_199065	ENSMUSG00000075478	**Slitrk1**	3200001I04Rik	SLIT and NTRK-like family, member 1	Amp	protein-coding
252_T12,T1,T22,T2,T42	chr10	4.7E+07	4.7E+07	3999	+	**5**	**62.5**	NA	Intergenic	Intergenic	914751	NR_028276	108637		NR_028276	ENSMUSG00000096263	**Snord14c**	Gm23107|Rnu14|U14	small nucleolar RNA, C/D box 14C	Amp	snoRNA
1354_T22,T2,T32,T3,T4	chr19	4.9E+07	4.9E+07	3999	+	**5**	**62.5**	NA	Intergenic	Intergenic	966475	NM_025696	66673	Mm.70980	NM_025696	ENSMUSG00000063434	**Sorcs3**	6330404A12Rik|AW045579	sortilin-related VPS10 domain containing receptor 3	Amp	protein-coding
58_T2,T32,T3,T42,T4	chr1	9.6E+07	9.6E+07	4999	+	**5**	**62.5**	NA	intron (NM_001159745, intron 2 of 3)	intron (NM_001159745, intron 2 of 3)	7594	NM_009183	20452	Mm.306228	NM_009183	ENSMUSG00000040710	**St8sia4**	PST|PST-1|SIAT8-D|ST8SiaIV|Siat8d	ST8 alpha-N-acetyl-neuraminide alpha-2,8-sialyltransferase 4	Amp	protein-coding

2918_T12,T1,T2,T32,T4	chr6	1.7E+07	1.7E+07	999	+	**5**	**62.5**	NA	intron (NM_031198, intron 1 of 6)	intron (NM_031198, intron 1 of 6)	11441	NM_031198	21426	Mm.36217	NM_031198	ENSMUSG00000029553	**Tfec**	BB107417|Tcfec|bHLHe34	transcription factor EC	Amp	protein-coding
3584_T1,T22,T2,T3,T42	chr8	6.7E+07	6.7E+07	4999	+	**5**	**62.5**	NA	Intergenic	B1_Mur1|SINE|Alu	52260	NM_028927	74419	Mm.159193	NM_028927	ENSMUSG00000025519	**Tktl2**	4933401I19Rik	transketolase-like 2	Amp	protein-coding
2288_T12,T2,T32,T3,T42	chr3	5.7E+07	5.7E+07	2999	+	**5**	**62.5**	NA	Intergenic	L1MC4|LINE|L1	399919	NM_008536	17112	Mm.856	NM_008536	ENSMUSG00000027800	**Tm4sf1**	L6|M3s1	transmembrane 4 superfamily member 1	Amp	protein-coding
2294_T12,T1,T2,T32,T42	chr3	5.7E+07	5.7E+07	11999	+	**5**	**62.5**	NA	Intergenic	(TATATG)n|Simple_repeat|Simple_repeat	329419	NM_008536	17112	Mm.856	NM_008536	ENSMUSG00000027800	**Tm4sf1**	L6|M3s1	transmembrane 4 superfamily member 1	Amp	protein-coding
32_T1,T22,T2,T3,T4	chr1	5E+07	5E+07	999	+	**5**	**62.5**	NA	Intergenic	L1_Mur1|LINE|L1	-650523	NM_019790	56363	Mm.245154	NM_019790	ENSMUSG00000026109	**Tmeff2**	4832418D20Rik|7630402F16Rik|TR-2	transmembrane protein with EGF-like and two follistatin-like domains 2	Amp	protein-coding
616_T12,T1,T22,T2,T3	chr14	6.1E+07	6.1E+07	4999	+	**5**	**62.5**	NA	Intergenic	Intergenic	81987	NM_001164155	29820	Mm.281356	NM_013869	ENSMUSG00000060548	**Tnfrsf19**	AL023044|AW123854|TAJ|TAJ-ALPHA|TRADE|Troy	tumor necrosis factor receptor superfamily, member 19	Amp	protein-coding
1046_T12,T22,T3,T42,T4	chr15	5E+07	5E+07	999	+	**5**	**62.5**	NA	Intergenic	Intergenic	673049	NM_001310481	83925	Mm.30466	NM_032000	ENSMUSG00000038679	**Trps1**	AI115454|AI447310|D15Ertd586e	trichorhinophalangeal syndrome I (human)	Amp	protein-coding
1051_T12,T1,T2,T3,T42	chr15	5E+07	5E+07	999	+	**5**	**62.5**	NA	Intergenic	Lx7|LINE|L1	546049	NM_001310481	83925	Mm.30466	NM_032000	ENSMUSG00000038679	**Trps1**	AI115454|AI447310|D15Ertd586e	trichorhinophalangeal syndrome I (human)	Amp	protein-coding
1059_T12,T1,T22,T2,T32	chr15	5.1E+07	5.1E+07	999	+	**5**	**62.5**	NA	intron (NM_001310485, intron 5 of 7)	intron (NM_001310485, intron 5 of 7)	81049	NM_001310481	83925	Mm.30466	NM_032000	ENSMUSG00000038679	**Trps1**	AI115454|AI447310|D15Ertd586e	trichorhinophalangeal syndrome I (human)	Amp	protein-coding
2349_T12,T32,T3,T42,T4	chr3	5.8E+07	5.8E+07	999	+	**5**	**62.5**	NA	intron (NM_001081229, intron 2 of 3)	intron (NM_001081229, intron 2 of 3)	22311	NM_001081229	72033	Mm.218409	NM_001081229	ENSMUSG00000027806	**Tsc22d2**	1810043J12Rik|5530402M19Rik	TSC22 domain family, member 2	Amp	protein-coding
1507_T12,T22,T2,T32,T3	chr2	6.6E+07	6.6E+07	3999	+	**5**	**62.5**	NA	intron (NM_001313997, intron 23 of 26)	PB1D10|SINE|Alu	-29883	NM_001290669	73668	Mm.250868	NM_001047604	ENSMUSG00000034848	**Ttc21b**	2410066K11Rik|Thm1|aln|mKIAA1992	tetratricopeptide repeat domain 21B	Amp	protein-coding
2656_T22,T2,T3,T42,T4	chr4	9.4E+07	9.4E+07	999	+	**5**	**62.5**	NA	Intergenic	Intergenic	-484489	NM_026954	69136	Mm.171558	NM_026954	ENSMUSG00000054000	**Tusc1**	2200001D17Rik|TSG-9	tumor suppressor candidate 1	Amp	protein-coding
3438_T12,T1,T22,T2,T3	chr8	3E+07	3E+07	999	+	**5**	**62.5**	NA	Intergenic	L1_Mur3|LINE|L1	-516364	NM_153135	210801	Mm.248433	NM_153135	ENSMUSG00000063626	**Unc5d**	D930029E11Rik|Unc5h4|mKIAA1777	unc-5 netrin receptor D	Amp	protein-coding

3545_T12,T2,T3,T42,T4	chr8	5.4E+07	5.4E+07	3999	+	**5**	**62.5**	NA	intron (NM_009506, intron 3 of 6)	L1MA7|LINE|L1	89968	NM_009506	22341	Mm.1402	NM_009506	ENSMUSG00000031520	**Vegfc**	AW228853|VEGF-C	vascular endothelial growth factor C	Amp	protein-coding
1380_T22,T2,T3,T42,T4	chr19	5.7E+07	5.7E+07	158999	+	**5**	**62.5**	NA	intron (NM_001177796, intron 14 of 19)	intron (NM_001177796, intron 14 of 19)	42584	NM_172840	240675	Mm.129534	NM_172840	ENSMUSG00000025082	**Vwa2**	Amaco	von Willebrand factor A domain containing 2	Amp	protein-coding
1699_T12,T22,T2,T3,T42	chr3	1.6E+07	1.6E+07	999	+	**5**	**62.5**	NA	TTS (NM_001145919)	TTS (NM_001145919)	33817	NM_001145919	229096	Mm.23834	NM_172677	ENSMUSG00000047213	**Ythdf3**	9130022A11Rik	YTH domain family 3	Amp	protein-coding
1705_T12,T1,T32,T3,T4	chr3	1.7E+07	1.7E+07	999	+	**5**	**62.5**	NA	Intergenic	Intergenic	415817	NM_001145919	229096	Mm.23834	NM_172677	ENSMUSG00000047213	**Ythdf3**	9130022A11Rik	YTH domain family 3	Amp	protein-coding
1694_T12,T1,T22,T3,T4	chr3	1.6E+07	1.6E+07	3999	+	**5**	**62.5**	NA	intron (NR_027375, intron 3 of 5)	intron (NR_027375, intron 3 of 5)	5317	NM_001145919	229096	Mm.23834	NM_172677	ENSMUSG00000047213	**Ythdf3**	9130022A11Rik	YTH domain family 3	Amp	protein-coding
1383_T22,T2,T3,T4	chr19	5.7E+07	5.7E+07	999	+	**4**	**50**	NA	intron (NM_001290813, intron 2 of 20)	RLTR17B_Mm|LTR|ERVK	25631	NM_001103177	226251	Mm.217161	NM_178688	ENSMUSG00000025085	**Ablim1**	2210411C18Rik|2610209L21Rik|4833406P10Rik|9330196J19Rik|AV079770|AW060987|AW215784|Limab1|mKIAA0059	actin-binding LIM protein 1	Amp	protein-coding
337_T12,T1,T2,T3	chr10	1.1E+08	1.1E+08	4999	+	**4**	**50**	NA	intron (NM_198636, intron 1 of 10)	intron (NM_198636, intron 1 of 10)	12664	NM_001142804	380660	Mm.336072	NM_198636	ENSMUSG00000035948	**Acss3**	8430416H19Rik|Gm874	acyl-CoA synthetase short-chain family member 3	Amp	protein-coding
585_T1,T32,T3,T4	chr13	1E+08	1E+08	999	+	**4**	**50**	NA	intron (NM_001081020, intron 14 of 24)	intron (NM_001081020, intron 14 of 24)	119127	NM_001081020	108154	Mm.208125	NM_175496	ENSMUSG00000046169	**Adamts6**	A930019D11Rik|ADAM-TS6|b2b1879.1Clo|b2b2029Clo|b2b2182Clo|b2b2187.1Clo|b2b2228Clo	a disintegrin-like and metallopeptidase (reprolysin type) with thrombospondin type 1 motif, 6	Amp	protein-coding
1381_T22,T2,T3,T4	chr19	5.7E+07	5.7E+07	999	+	**4**	**50**	NA	intron (NM_146102, intron 1 of 20)	intron (NM_146102, intron 1 of 20)	11575	NM_146102	226250	Mm.226284	NM_146102	ENSMUSG00000025083	**Afap1l2**	AU041783|C86904|mKIAA1914	actin filament associated protein 1-like 2	Amp	protein-coding
3527_T12,T22,T42,T4	chr8	5.2E+07	5.2E+07	999	+	**4**	**50**	NA	Intergenic	MTE2b|LTR|ERVL-MaLR	-1E+06	NM_001205054	11593	Mm.334535	NM_001005847	ENSMUSG00000031521	**Aga**	AW060726	aspartylglucosaminidase	Amp	protein-coding
3529_T1,T22,T2,T4	chr8	5.3E+07	5.3E+07	3999	+	**4**	**50**	NA	Intergenic	Intergenic	-838202	NM_001205054	11593	Mm.334535	NM_001005847	ENSMUSG00000031521	**Aga**	AW060726	aspartylglucosaminidase	Amp	protein-coding
394_T1,T2,T32,T42	chr11	6.1E+07	6.1E+07	1999	+	**4**	**50**	NA	Intergenic	RSINE1|SINE|B4	53817	NM_172943	268420	Mm.262056	NM_172943	ENSMUSG00000042650	**Alkbh5**	AW050020|AW215868|Abh5|E130207K11|Ofoxd	alkB homolog 5, RNA demethylase	Amp	protein-coding

973_T22,T2,T32,T3	chr15	4.2E+07	4.2E+07	3999	+	**4**	**50**	NA	intron (NM_001286062, intron 4 of 8)	intron (NM_001286062, intron 4 of 8)	189477	NM_001286062	11600	Mm.309336	NM_009640	ENSMUSG00000022309	**Angpt1**	1110046O21Rik|Ang-1|Ang1	angiopoietin 1	Amp	protein-coding
2989_T12,T1,T42,T4	chr6	1.9E+07	1.9E+07	999	+	**4**	**50**	NA	Intergenic	L1Md_T|LINE|L1	116682	NM_001167757	75196	Mm.67665	NM_029202	ENSMUSG00000029517	**Ankrd7**	4930532L20Rik|AV278427	ankyrin repeat domain 7	Amp	protein-coding
2992_T12,T1,T3,T4	chr6	1.9E+07	1.9E+07	999	+	**4**	**50**	NA	Intergenic	L1Md_T|LINE|L1	122682	NM_001167757	75196	Mm.67665	NM_029202	ENSMUSG00000029517	**Ankrd7**	4930532L20Rik|AV278427	ankyrin repeat domain 7	Amp	protein-coding
2996_T12,T2,T3,T42	chr6	1.9E+07	1.9E+07	2999	+	**4**	**50**	NA	Intergenic	Intergenic	162682	NM_001167757	75196	Mm.67665	NM_029202	ENSMUSG00000029517	**Ankrd7**	4930532L20Rik|AV278427	ankyrin repeat domain 7	Amp	protein-coding
3000_T12,T1,T22,T4	chr6	1.9E+07	1.9E+07	999	+	**4**	**50**	NA	Intergenic	Lx5c|LINE|L1	192682	NM_001167757	75196	Mm.67665	NM_029202	ENSMUSG00000029517	**Ankrd7**	4930532L20Rik|AV278427	ankyrin repeat domain 7	Amp	protein-coding
3002_T12,T1,T22,T4	chr6	1.9E+07	1.9E+07	999	+	**4**	**50**	NA	Intergenic	L1Md_T|LINE|L1	202682	NM_001167757	75196	Mm.67665	NM_029202	ENSMUSG00000029517	**Ankrd7**	4930532L20Rik|AV278427	ankyrin repeat domain 7	Amp	protein-coding
3003_T12,T2,T3,T42	chr6	1.9E+07	1.9E+07	999	+	**4**	**50**	NA	Intergenic	L1Md_T|LINE|L1	203682	NM_001167757	75196	Mm.67665	NM_029202	ENSMUSG00000029517	**Ankrd7**	4930532L20Rik|AV278427	ankyrin repeat domain 7	Amp	protein-coding
2325_T12,T3,T42,T4	chr3	5.8E+07	5.8E+07	3999	+	**4**	**50**	NA	intron (NM_001033349, intron 2 of 3)	MMETn-int|LTR|ERVK	9037	NM_001033349	242037	Mm.291091	NM_001033349	ENSMUSG00000074591	**Ankub1**	Gm410	ankrin repeat and ubiquitin domain containing 1	Amp	protein-coding
443_T12,T1,T32,T3	chr12	5.3E+07	5.3E+07	999	+	**4**	**50**	NA	intron (NM_009706, intron 1 of 5)	intron (NM_009706, intron 1 of 5)	4923	NM_009706	11855	Mm.35059	NM_009706	ENSMUSG00000035133	**Arhgap5**	p190-B|p190B	Rho GTPase activating protein 5	Amp	protein-coding
611_T12,T1,T3,T4	chr14	6E+07	6E+07	3999	+	**4**	**50**	NA	intron (NM_015803, intron 23 of 35)	intron (NM_015803, intron 23 of 35)	93334	NM_015803	50769	Mm.319599	NM_015803	ENSMUSG00000021983	**Atp8a2**	AI415030|Atpc1b|agil|wl	ATPase, aminophospholipid transporter-like, class I, type 8A, member 2	Amp	protein-coding
159_T12,T2,T42,T4	chr1	1.4E+08	1.4E+08	999	+	**4**	**50**	NA	Intergenic	Intergenic	-1E+06	NM_020025	26878	Mm.285580	NM_020025	ENSMUSG00000033849	**B3galt2**	-	UDP-Gal:betaGlcNAc beta 1,3-galactosyltransferase, polypeptide 2	Amp	protein-coding
1589_T12,T22,T42,T4	chr2	1.1E+08	1.1E+08	2999	+	**4**	**50**	NA	Intergenic	ID_B1|SINE|B4	-151700	NM_007540	12064	Mm.1442	NM_007540	ENSMUSG00000048482	**Bdnf**	-	brain derived neurotrophic factor	Amp	protein-coding
2602_T12,T1,T3,T42	chr4	6.8E+07	6.8E+07	3999	+	**4**	**50**	NA	Intergenic	MT2B1|LTR|ERVL	792897	NM_019967	56710	Mm.248788	NM_019967	ENSMUSG00000028351	**Brinp1**	BRINP|Dbc1|Dbccr1|Fam5a	bone morphogenic protein/retinoic acid inducible neural specific 1	Amp	protein-coding

2607_T12,T2,T32,T3	chr4	6.9E+07	6.9E+07	999	+	**4**	**50**	NA	Intergenic	L1MC2|LINE|L1	215397	NM_019967	56710	Mm.248788	NM_019967	ENSMUSG00000028351	**Brinp1**	BRINP|Dbc1|Dbccr1|Fam5a	bone morphogenic protein/retinoic acid inducible neural specific 1	Amp	protein-coding
2611_T12,T2,T32,T3	chr4	6.9E+07	6.9E+07	999	+	**4**	**50**	NA	Intergenic	L1MA4|LINE|L1	-423603	NM_019967	56710	Mm.248788	NM_019967	ENSMUSG00000028351	**Brinp1**	BRINP|Dbc1|Dbccr1|Fam5a	bone morphogenic protein/retinoic acid inducible neural specific 1	Amp	protein-coding
187_T1,T22,T2,T4	chr1	1.5E+08	1.5E+08	1999	+	**4**	**50**	NA	Intergenic	Intergenic	616813	NM_001145807	215378	Mm.441817	NM_153539	ENSMUSG00000035131	**Brinp3**	B830045N13Rik|Fam5c	bone morphogenetic protein/retinoic acid inducible neural specific 3	Amp	protein-coding
1419_T22,T2,T42,T4	chr19	6.1E+07	6.1E+07	999	+	**4**	**50**	NA	Intergenic	Lx8|LINE|L1	-61977	NM_001172096	78832	Mm.259026	NM_030197	ENSMUSG00000033417	**Cacul1**	2700078E11Rik|2810417M16Rik|9830127L17Rik|AI450346|D130033C15Rik	CDK2 associated, cullin domain 1	Amp	protein-coding
1421_T22,T2,T42,T4	chr19	6.1E+07	6.1E+07	6999	+	**4**	**50**	NA	Intergenic	Intergenic	-72977	NM_001172096	78832	Mm.259026	NM_030197	ENSMUSG00000033417	**Cacul1**	2700078E11Rik|2810417M16Rik|9830127L17Rik|AI450346|D130033C15Rik	CDK2 associated, cullin domain 1	Amp	protein-coding
379_T12,T22,T32,T42	chr11	4.1E+07	4.1E+07	3999	+	**4**	**50**	NA	Intergenic	Lx8b|LINE|L1	-550214	NM_009831	12450	Mm.2103	NM_009831	ENSMUSG00000020326	**Ccng1**	AI314029	cyclin G1	Amp	protein-coding
930_T12,T22,T3,T4	chr15	1.9E+07	1.9E+07	999	+	**4**	**50**	NA	Intergenic	Lx5c|LINE|L1	366671	NM_009865	320873	Mm.117794	NM_009865	ENSMUSG00000022321	**Cdh10**	A830016G23Rik|C030003B10Rik|C030011H18Rik	cadherin 10	Amp	protein-coding
932_T1,T22,T42,T4	chr15	1.9E+07	1.9E+07	3999	+	**4**	**50**	NA	Intergenic	ERVL-int|LTR|ERVL	588171	NM_009865	320873	Mm.117794	NM_009865	ENSMUSG00000022321	**Cdh10**	A830016G23Rik|C030003B10Rik|C030011H18Rik	cadherin 10	Amp	protein-coding
3655_T22,T32,T3,T4	chr8	1E+08	1E+08	999	+	**4**	**50**	NA	Intergenic	Intergenic	1257111	NM_009866	12552	Mm.1571	NM_009866	ENSMUSG00000031673	**Cdh11**	Cad11|OSF-4	cadherin 11	Amp	protein-coding
3658_T1,T2,T32,T4	chr8	1E+08	1E+08	3999	+	**4**	**50**	NA	Intergenic	L1MA5|LINE|L1	1141611	NM_009866	12552	Mm.1571	NM_009866	ENSMUSG00000031673	**Cdh11**	Cad11|OSF-4	cadherin 11	Amp	protein-coding
953_T1,T2,T32,T3	chr15	2.2E+07	2.2E+07	3999	+	**4**	**50**	NA	intron (NM_001008420, intron 9 of 11)	intron (NM_001008420, intron 9 of 11)	466048	NM_001008420	215654	Mm.300909	NM_001008420	ENSMUSG00000040452	**Cdh12**	Cdhb	cadherin 12	Amp	protein-coding
107_T1,T22,T2,T3	chr1	1E+08	1E+08	4999	+	**4**	**50**	NA	Intergenic	Intergenic	-506819	NM_011800	23836	Mm.103640	NM_011800	ENSMUSG00000050840	**Cdh20**	Cdh7	cadherin 20	Amp	protein-coding
123_T1,T22,T2,T42	chr1	1.1E+08	1.1E+08	4999	+	**4**	**50**	NA	Intergenic	Intergenic	158277	NM_172853	241201	Mm.213407	NM_172853	ENSMUSG00000026312	**Cdh7**	9330156F07Rik|CDH7L1	cadherin 7, type 2	Amp	protein-coding

3625_T12,T1,T32,T4	chr8	9.9E+07	9.9E+07	3999	+	**4**	**50**	NA	intron (NR_133570, intron 2 of 10)	intron (NR_133570, intron 2 of 10)	101971	NM_001285913	12564	Mm.441131	NM_007667	ENSMUSG00000036510	**Cdh8**	AI851472|cad8	cadherin 8	Amp	protein-coding
3624_T12,T1,T22,T4	chr8	9.9E+07	9.9E+07	999	+	**4**	**50**	NA	intron (NR_133570, intron 3 of 10)	intron (NR_133570, intron 3 of 10)	137471	NM_001285913	12564	Mm.441131	NM_007667	ENSMUSG00000036510	**Cdh8**	AI851472|cad8	cadherin 8	Amp	protein-coding
897_T12,T2,T3,T4	chr15	1.6E+07	1.6E+07	3999	+	**4**	**50**	NA	Intergenic	RMER6A|LTR|ERVK	-1E+06	NM_009869	12565	Mm.439758	NM_009869	ENSMUSG00000025370	**Cdh9**	-	cadherin 9	Amp	protein-coding
904_T12,T22,T42,T4	chr15	1.6E+07	1.6E+07	999	+	**4**	**50**	NA	Intergenic	Lx9|LINE|L1	-796101	NM_009869	12565	Mm.439758	NM_009869	ENSMUSG00000025370	**Cdh9**	-	cadherin 9	Amp	protein-coding
907_T12,T1,T3,T4	chr15	1.7E+07	1.7E+07	4999	+	**4**	**50**	NA	intron (NM_009869, intron 1 of 10)	Lx7|LINE|L1	21899	NM_009869	12565	Mm.439758	NM_009869	ENSMUSG00000025370	**Cdh9**	-	cadherin 9	Amp	protein-coding
3097_T2,T32,T42,T4	chr6	1E+08	1E+08	3999	+	**4**	**50**	NA	Intergenic	L1Md_A|LINE|L1	-101376	NM_007697	12661	Mm.251288	NM_007697	ENSMUSG00000030077	**Chl1**	A530023M13Rik|AI465420|CALL|LICAM2	cell adhesion molecule L1-like	Amp	protein-coding
3367_T1,T32,T3,T4	chr7	6.3E+07	6.3E+07	4999	+	**4**	**50**	NA	Intergenic	Intergenic	-54474	NM_007390	11441	Mm.113464	NM_007390	ENSMUSG00000030525	**Chrna7**	Acra7|alpha7|nAchR	cholinergic receptor, nicotinic, alpha polypeptide 7	Amp	protein-coding
199_T12,T22,T2,T4	chr1	1.8E+08	1.8E+08	999	+	**4**	**50**	NA	TTS (NM_016805)	TTS (NM_016805)	8847	NM_025511	66359	Mm.489720	NM_025511	ENSMUSG00000026500	**Cox20**	2310005N03Rik|Fam36a	COX20 Cox2 chaperone	Amp	protein-coding
1010_T1,T22,T42,T4	chr15	4.8E+07	4.8E+07	4999	+	**4**	**50**	NA	intron (NM_001081391, intron 38 of 70)	intron (NM_001081391, intron 38 of 70)	1063989	NM_001081391	239420	Mm.309363	NM_001081391	ENSMUSG00000022311	**Csmd3**	4930500N14Rik|mKIAA1894	CUB and Sushi multiple domains 3	Amp	protein-coding
1613_T1,T2,T3,T4	chr2	1.5E+08	1.5E+08	3999	+	**4**	**50**	NA	Intergenic	Intergenic	-70988	NM_009976	13010	Mm.4263	NM_009976	ENSMUSG00000027447	**Cst3**	CysC	cystatin C	Amp	protein-coding
1620_T12,T1,T2,T4	chr2	1.5E+08	1.5E+08	999	+	**4**	**50**	NA	Intergenic	Intergenic	-209488	NM_009976	13010	Mm.4263	NM_009976	ENSMUSG00000027447	**Cst3**	CysC	cystatin C	Amp	protein-coding
966_T2,T32,T3,T4	chr15	3E+07	3E+07	4999	+	**4**	**50**	NA	Intergenic	RMER15|LTR|ERVL	-533593	NM_008729	18163	Mm.321648	NM_008729	ENSMUSG00000022240	**Ctnnd2**	Catnd2|Nprap|neurojugin	catenin (cadherin associated protein), delta 2	Amp	protein-coding
2950_T12,T22,T32,T3	chr6	1.9E+07	1.9E+07	999	+	**4**	**50**	NA	Intergenic	Intergenic	-9175	NM_080285	30785	Mm.224189	NM_080285	ENSMUSG00000000416	**Cttnbp2**	3010022N24Rik|4732477G22Rik|6430526E05|9130022E09Rik|AU040881|Cortbp2|ORF4|mKIAA1758	cortactin binding protein 2	Amp	protein-coding

2958_T1,T32,T42,T4	chr6	1.9E+07	1.9E+07	999	+	**4**	**50**	NA	Intergenic	Intergenic	-96175	NM_080285	30785	Mm.224189	NM_080285	ENSMUSG00000000416	**Cttnbp2**	3010022N24Rik|4732477G22Rik|6430526E05|9130022E09Rik|AU040881|Cortbp2|ORF4|mKIAA1758	cortactin binding protein 2	Amp	protein-coding
789_T1,T22,T2,T42	chr14	9.8E+07	9.8E+07	3999	+	**4**	**50**	NA	Intergenic	Intergenic	-36735	NM_007826	13134	Mm.320593	NM_007826	ENSMUSG00000055639	**Dach1**	Dac|Dach	dachshund 1 (Drosophila)	Amp	protein-coding
783_T1,T22,T42,T4	chr14	9.8E+07	9.8E+07	5999	+	**4**	**50**	NA	intron (NM_007826, intron 2 of 11)	Lx9|LINE|L1	197265	NM_001038610	13134	Mm.320593	NM_007826	ENSMUSG00000055639	**Dach1**	Dac|Dach	dachshund 1 (Drosophila)	Amp	protein-coding
2239_T12,T2,T42,T4	chr3	5.6E+07	5.6E+07	999	+	**4**	**50**	NA	Intergenic	L1Md_T|LINE|L1	144242	NM_001195540	13175	Mm.393242	NM_019978	ENSMUSG00000027797	**Dclk1**	1700113D08Rik|2810480F11Rik|AI836758|Click-I|Cpg16|Dcamkl1|Dcl|Dclk|mKIAA0369	doublecortin-like kinase 1	Amp	protein-coding
1503_T22,T2,T3,T42	chr2	6.2E+07	6.2E+07	3999	+	**4**	**50**	NA	intron (NM_001242381, intron 6 of 25)	intron (NM_001242381, intron 6 of 25)	172731	NM_010074	13482	Mm.1151	NM_010074	ENSMUSG00000035000	**Dpp4**	Cd26|Dpp-4|THAM	dipeptidylpeptidase 4	Amp	protein-coding
2476_T2,T32,T3,T4	chr3	1.2E+08	1.2E+08	3999	+	**4**	**50**	NA	intron (NM_170778, intron 5 of 22)	intron (NM_170778, intron 5 of 22)	222322	NM_170778	99586	Mm.27907	NM_170778	ENSMUSG00000033308	**Dpyd**	AI315208|DPD|E330028L06Rik	dihydropyrimidine dehydrogenase	Amp	protein-coding
983_T1,T22,T32,T4	chr15	4.5E+07	4.5E+07	3999	+	**4**	**50**	NA	exon (NM_138674, exon 56 of 77)	exon (NM_138674, exon 56 of 77)	-59141	NM_019480	55960	Mm.287896	NM_019480	ENSMUSG00000022339	**Ebag9**	AI835379|Rcas1	estrogen receptor-binding fragment-associated gene 9	Amp	protein-coding
575_T1,T2,T42,T4	chr13	8.8E+07	8.8E+07	999	+	**4**	**50**	NA	Intergenic	Intergenic	-571472	NM_010103	13612	Mm.125580	NM_010103	ENSMUSG00000034488	**Edil3**	Del-1|Del1	EGF-like repeats and discoidin I-like domains 3	Amp	protein-coding
3418_T22,T2,T32,T42	chr8	7909501	7913500	3999	+	**4**	**50**	NA	Intergenic	Intergenic	749273	NM_010111	13642	Mm.209813	NM_010111	ENSMUSG00000001300	**Efnb2**	ELF-2|Epl5|Eplg5|Htk-L|LERK-5|Lerk5|NLERK-1	ephrin B2	Amp	protein-coding
3421_T22,T2,T3,T42	chr8	7932501	7936500	3999	+	**4**	**50**	NA	Intergenic	Intergenic	726273	NM_010111	13642	Mm.209813	NM_010111	ENSMUSG00000001300	**Efnb2**	ELF-2|Epl5|Eplg5|Htk-L|LERK-5|Lerk5|NLERK-1	ephrin B2	Amp	protein-coding
3424_T2,T3,T42,T4	chr8	7979501	7982500	2999	+	**4**	**50**	NA	Intergenic	MER2|DNA|TcMar-Tigger	679773	NM_010111	13642	Mm.209813	NM_010111	ENSMUSG00000001300	**Efnb2**	ELF-2|Epl5|Eplg5|Htk-L|LERK-5|Lerk5|NLERK-1	ephrin B2	Amp	protein-coding

3427_T1,T22,T2,T32	chr8	8199501	8203500	3999	+	**4**	**50**	NA	Intergenic	Intergenic	459273	NM_010111	13642	Mm.209813	NM_010111	ENSMUSG00000001300	**Efnb2**	ELF-2|Epl5|Eplg5|Htk-L|LERK-5|Lerk5|NLERK-1	ephrin B2	Amp	protein-coding
1395_T22,T2,T3,T4	chr19	5.9E+07	5.9E+07	999	+	**4**	**50**	NA	intron (NM_001114312, intron 14 of 16)	intron (NM_001114312, intron 14 of 16)	52575	NM_178689	226265	Mm.103154	NM_178689	ENSMUSG00000048029	**Eno4**	6430537H07Rik	enolase 4	Amp	protein-coding
2872_T12,T1,T22,T4	chr5	8.5E+07	8.5E+07	999	+	**4**	**50**	NA	Intergenic	Intergenic	-256618	NM_007937	13839	Mm.137991	NM_007937	ENSMUSG00000029245	**Epha5**	AI854630|AW125296|Cek7|Ehk1|Els1|Hek7|Rek7|bsk	Eph receptor A5	Amp	protein-coding
2442_T22,T2,T32,T3	chr3	9.3E+07	9.3E+07	2999	+	**4**	**50**	NA	Intergenic	Intergenic	-20273	NM_001013804	229574	Mm.10755	NM_001013804		**Flg2**	EG229574	filaggrin family member 2	Amp	protein-coding
1611_T22,T2,T3,T42	chr2	1.4E+08	1.4E+08	3999	+	**4**	**50**	NA	intron (NM_001013802, intron 5 of 18)	intron (NM_001013802, intron 5 of 18)	-753024	NM_178382	71436	Mm.426781	NM_178382	ENSMUSG00000051379	**Flrt3**	5530600M07Rik|C430047I10Rik|mKIAA1469	fibronectin leucine rich transmembrane protein 3	Amp	protein-coding
2202_T2,T3,T42,T4	chr3	5.2E+07	5.2E+07	3999	+	**4**	**50**	NA	intron (NM_019739, intron 1 of 2)	intron (NM_019739, intron 1 of 2)	60163	NM_019739	56458	Mm.29891	NM_019739	ENSMUSG00000044167	**Foxo1**	AI876417|Afxh|FKHR|Fkhr1|Foxo1a	forkhead box O1	Amp	protein-coding
2228_T12,T1,T42,T4	chr3	5.4E+07	5.4E+07	7999	+	**4**	**50**	NA	Intergenic	L1Md_T|LINE|L1	-61145	NM_172862	242022	Mm.38378	NM_172862	ENSMUSG00000037016	**Frem2**	6030440P17Rik|8430406N05Rik|Gm409|b2b1562Clo|my|ne|nv1	Fras1 related extracellular matrix protein 2	Amp	protein-coding
593_T12,T32,T3,T42	chr13	1.1E+08	1.1E+08	7999	+	**4**	**50**	NA	Intergenic	Intergenic	-51549	NM_008046	14313	Mm.4913	NM_008046	ENSMUSG00000021765	**Fst**	AL033346|FS	follistatin	Amp	protein-coding
2408_T1,T32,T3,T4	chr3	7.7E+07	7.7E+07	4999	+	**4**	**50**	NA	Intergenic	ORR1B2|LTR|ERVL-MaLR	1073417	NM_001253719	213262	Mm.379337	NM_178673	ENSMUSG00000034098	**Fstl5**	9130207J01Rik	follistatin-like 5	Amp	protein-coding
2413_T1,T22,T32,T3	chr3	7.7E+07	7.7E+07	2999	+	**4**	**50**	NA	Intergenic	Intergenic	1411417	NM_001253719	213262	Mm.379337	NM_178673	ENSMUSG00000034098	**Fstl5**	9130207J01Rik	follistatin-like 5	Amp	protein-coding
2418_T1,T22,T32,T3	chr3	7.8E+07	7.8E+07	5999	+	**4**	**50**	NA	Intergenic	Intergenic	1441917	NM_001253719	213262	Mm.379337	NM_178673	ENSMUSG00000034098	**Fstl5**	9130207J01Rik	follistatin-like 5	Amp	protein-coding
2392_T12,T22,T32,T3	chr3	7.6E+07	7.6E+07	999	+	**4**	**50**	NA	intron (NM_178673, intron 3 of 15)	intron (NM_178673, intron 3 of 15)	195417	NM_001253719	213262	Mm.379337	NM_178673	ENSMUSG00000034098	**Fstl5**	9130207J01Rik	follistatin-like 5	Amp	protein-coding
388_T22,T2,T3,T42	chr11	4.2E+07	4.2E+07	3999	+	**4**	**50**	NA	intron (NM_010250, intron 3 of 9)	intron (NM_010250, intron 3 of 9)	15430	NM_010250	14394	Mm.439668	NM_010250	ENSMUSG00000010803	**Gabra1**	Gabra-1	gamma-aminobutyric acid (GABA) A receptor, subunit alpha 1	Amp	protein-coding

387_T1,T32,T42,T4	chr11	4.2E+07	4.2E+07	5999	+	**4**	**50**	NA	intron (NM_010250, intron 6 of 9)	intron (NM_010250, intron 6 of 9)	35430	NM_010250	14394	Mm.439668	NM_010250	ENSMUSG00000010803	**Gabra1**	Gabra-1	gamma-aminobutyric acid (GABA) A receptor, subunit alpha 1	Amp	protein-coding
392_T22,T2,T3,T4	chr11	4.3E+07	4.3E+07	3999	+	**4**	**50**	NA	intron (NM_008070, intron 6 of 9)	intron (NM_008070, intron 6 of 9)	127743	NM_008070	14401	Mm.338723	NM_008070	ENSMUSG00000007653	**Gabrb2**	AI834970|C030002O17Rik|C030021G16Rik|Gabrab2|Gabrb-2	gamma-aminobutyric acid (GABA) A receptor, subunit beta 2	Amp	protein-coding
385_T12,T2,T3,T42	chr11	4.2E+07	4.2E+07	999	+	**4**	**50**	NA	intron (NM_177408, intron 1 of 8)	intron (NM_177408, intron 1 of 8)	3714	NM_177408	14406	Mm.5309	NM_008073	ENSMUSG00000020436	**Gabrg2**	GABAA-R|Gabrg-2|gamma2	gamma-aminobutyric acid (GABA) A receptor, subunit gamma 2	Amp	protein-coding
3560_T1,T22,T2,T4	chr8	5.9E+07	5.9E+07	999	+	**4**	**50**	NA	intron (NM_175032, intron 1 of 11)	Lx8|LINE|L1	71627	NM_175032	270049	Mm.236368	NM_175032	ENSMUSG00000096914	**Galntl6**	1700021K10Rik|4930431L04Rik|A830023L05	UDP-N-acetyl-alpha-D-galactosamine:polypeptide N-acetylgalactosaminyltransferase-like 6	Amp	protein-coding
2734_T1,T22,T2,T3	chr5	3E+07	3E+07	999	+	**4**	**50**	NA	TTS (NM_001256263)	TTS (NM_001256263)	-2196	NM_001167879	242915	Mm.332287	NM_001167879	ENSMUSG00000044576	**Garem2**	Fam59b|Gareml|Gm444	GRB2 associated regulator of MAPK1 subtype 2	Amp	protein-coding
3068_T1,T22,T2,T3	chr6	6.4E+07	6.4E+07	999	+	**4**	**50**	NA	intron (NM_008167, intron 2 of 15)	intron (NM_008167, intron 2 of 15)	260143	NM_008167	14804	Mm.439651	NM_008167	ENSMUSG00000071424	**Grid2**	B230104L07Rik|GluD2|GluRdelta2|Lc|Lc<J>|MMS10-AC|Ms10ac|cpr|ho|nmf408|tpr	glutamate receptor, ionotropic, delta 2	Amp	protein-coding
3071_T12,T1,T22,T2	chr6	6.4E+07	6.4E+07	999	+	**4**	**50**	NA	intron (NM_008167, intron 2 of 15)	intron (NM_008167, intron 2 of 15)	353143	NM_008167	14804	Mm.439651	NM_008167	ENSMUSG00000071424	**Grid2**	B230104L07Rik|GluD2|GluRdelta2|Lc|Lc<J>|MMS10-AC|Ms10ac|cpr|ho|nmf408|tpr	glutamate receptor, ionotropic, delta 2	Amp	protein-coding
2444_T1,T3,T42,T4	chr3	9.3E+07	9.3E+07	999	+	**4**	**50**	NA	Intergenic	Intergenic	-39749	NM_133698	68723	Mm.208047	NM_133698	ENSMUSG00000041991	**Hrnr**	1110033K19Rik|A530063N20Rik|S100a18	hornerin	Amp	protein-coding
2740_T12,T1,T2,T3	chr5	4.1E+07	4.1E+07	2999	+	**4**	**50**	NA	Intergenic	Intergenic	-985369	NM_010474	15476	Mm.12559	NM_010474	ENSMUSG00000051022	**Hs3st1**	3-Ost|D5Wsu110e|Hsg3ost	heparan sulfate (glucosamine) 3-O-sulfotransferase 1	Amp	protein-coding
3598_T1,T2,T32,T4	chr8	8.2E+07	8.2E+07	5999	+	**4**	**50**	NA	Intergenic	L1MA5|LINE|L1	245086	NM_001254747	16168	Mm.4392	NM_008357	ENSMUSG00000031712	**Il15**	AI503618|IL-15	interleukin 15	Amp	protein-coding
1120_T12,T1,T22,T32	chr15	6.9E+07	6.9E+07	3999	+	**4**	**50**	NA	intron (NM_010158, intron 7 of 8)	Lx7|LINE|L1	126080	NM_010158	13992	Mm.17964	NM_010158	ENSMUSG00000022332	**Khdrbs3**	Etle|SLM-2|Salp|Slm2|T-STAR	KH domain containing, RNA binding, signal transduction associated 3	Amp	protein-coding
767_T1,T2,T32,T42	chr14	9.6E+07	9.6E+07	6999	+	**4**	**50**	NA	intron (NM_053105, intron 1 of 10)	L1Md_F2|LINE|L1	123034	NM_053105	93688	Mm.308735	NM_053105	ENSMUSG00000022076	**Klhl1**	mKIAA1490	kelch-like 1	Amp	protein-coding

447_T12,T2,T3,T4	chr12	6.2E+07	6.2E+07	999	+	**4**	**50**	NA	intron (NM_001310586, intron 1 of 5)	intron (NM_001310586, intron 1 of 5)	96833	NM_001310586	238205	Mm.28802	NM_178714	ENSMUSG00000035653	**Lrfn5**	AI427653|AI604817|C130061B21|mKIAA4208	leucine rich repeat and fibronectin type III domain containing 5	Amp	protein-coding
2449_T1,T22,T2,T3	chr3	1.1E+08	1.1E+08	999	+	**4**	**50**	NA	intron (NM_028081, intron 1 of 2)	MER58B|DNA|hAT-Charlie	8318	NM_001286685	321000	Mm.259638	NM_028081	ENSMUSG00000056260	**Lrif1**	2010012G17Rik|4933421E11Rik|AI450568	ligand dependent nuclear receptor interacting factor 1	Amp	protein-coding
1469_T1,T2,T32,T42	chr2	4.3E+07	4.3E+07	999	+	**4**	**50**	NA	Intergenic	L1Md_F2|LINE|L1	-261402	NM_053011	94217	Mm.441398	NM_053011	ENSMUSG00000049252	**Lrp1b**	9630004P12Rik|LRP-DIT	low density lipoprotein-related protein 1B (deleted in tumors)	Amp	protein-coding
1566_T22,T2,T3,T42	chr2	9.9E+07	9.9E+07	3999	+	**4**	**50**	NA	Intergenic	Intergenic	1245843	NM_178725	241568	Mm.241682	NM_178725	ENSMUSG00000050587	**Lrrc4c**	6430556C10Rik|NGL-1	leucine rich repeat containing 4C	Amp	protein-coding
1542_T12,T2,T3,T4	chr2	9.7E+07	9.7E+07	999	+	**4**	**50**	NA	intron (NM_001289742, intron 3 of 7)	intron (NM_001289742, intron 3 of 7)	530831	NM_001289743	241568	Mm.241682	NM_178725	ENSMUSG00000050587	**Lrrc4c**	6430556C10Rik|NGL-1	leucine rich repeat containing 4C	Amp	protein-coding
1557_T12,T22,T32,T42	chr2	9.8E+07	9.8E+07	999	+	**4**	**50**	NA	intron (NM_001289742, intron 7 of 7)	intron (NM_001289742, intron 7 of 7)	149343	NM_178725	241568	Mm.241682	NM_178725	ENSMUSG00000050587	**Lrrc4c**	6430556C10Rik|NGL-1	leucine rich repeat containing 4C	Amp	protein-coding
1550_T12,T2,T42,T4	chr2	9.8E+07	9.8E+07	3999	+	**4**	**50**	NA	intron (NM_001289744, intron 1 of 1)	RLTR40|LTR|ERVK	45843	NM_178725	241568	Mm.241682	NM_178725	ENSMUSG00000050587	**Lrrc4c**	6430556C10Rik|NGL-1	leucine rich repeat containing 4C	Amp	protein-coding
2513_T1,T22,T32,T42	chr3	1.6E+08	1.6E+08	999	+	**4**	**50**	NA	intron (NM_001081358, intron 3 of 27)	RLTR22_Mur|LTR|ERVK	175221	NM_001291453	242274	Mm.132162	NM_001081358	ENSMUSG00000028176	**Lrrc7**	AK122498|densin|mKIAA1365	leucine rich repeat containing 7	Amp	protein-coding
3087_T12,T1,T32,T4	chr6	8.1E+07	8.1E+07	999	+	**4**	**50**	NA	intron (NM_001134743, intron 2 of 2)	intron (NM_001134743, intron 2 of 2)	724357	NM_178731	243499	Mm.483425	NM_178731	ENSMUSG00000052581	**Lrrtm4**	7530419J18Rik|A230052N11	leucine rich repeat transmembrane neuronal 4	Amp	protein-coding
2967_T12,T2,T32,T42	chr6	1.9E+07	1.9E+07	999	+	**4**	**50**	NA	Intergenic	L1_Mus1|LINE|L1	-68635	NM_133939	76522	Mm.275158	NM_133939	ENSMUSG00000044155	**Lsm8**	2010003I05Rik|AW214405|Naa38	LSM8 homolog, U6 small nuclear RNA associated	Amp	protein-coding
2492_T1,T22,T2,T3	chr3	1.4E+08	1.4E+08	999	+	**4**	**50**	NA	intron (NM_025356, intron 7 of 7)	intron (NM_025356, intron 7 of 7)	-20611	NM_027288	110173	Mm.280536	NM_027288	ENSMUSG00000028164	**Manba**	2410030O07Rik|B930014J03Rik|Bmn	mannosidase, beta A, lysosomal	Amp	protein-coding
555_T2,T3,T42,T4	chr13	8.3E+07	8.3E+07	999	+	**4**	**50**	NA	Intergenic	L1Md_F2|LINE|L1	-274034	NM_001170537	17260	Mm.24001	NM_025282	ENSMUSG00000005583	**Mef2c**	5430401D19Rik|9930028G15Rik|AV011172|Mef2	myocyte enhancer factor 2C	Amp	protein-coding

2613_T22,T2,T32,T4	chr4	7.1E+07	7.1E+07	3999	+	**4**	**50**	NA	Intergenic	Intergenic	-508572	NM_172694	230316	Mm.251188	NM_172694	ENSMUSG00000039270	**Megf9**	4933405H16Rik|9630025C07|Egfl5|mKIAA0818	multiple EGF-like-domains 9	Amp	protein-coding
2187_T12,T1,T2,T3	chr3	5.2E+07	5.2E+07	999	+	**4**	**50**	NA	Intergenic	IAPEz-int|LTR|ERVK	-38179	NM_174995	211666	Mm.24679	NM_174995	ENSMUSG00000074604	**Mgst2**	GST2|MGST-II	microsomal glutathione S-transferase 2	Amp	protein-coding
3453_T22,T2,T32,T42	chr8	3.8E+07	3.8E+07	999	+	**4**	**50**	NA	intron (NM_145841, intron 2 of 7)	AT_rich|Low_complexity|Low_complexity	450202	NR_029884	723860		NR_029884	ENSMUSG00000065457	**Mir383**	Mirn383|mir-383|mmu-mir-383	microRNA 383	Amp	ncRNA
3459_T22,T2,T3,T4	chr8	3.8E+07	3.8E+07	999	+	**4**	**50**	NA	intron (NM_145841, intron 2 of 7)	intron (NM_145841, intron 2 of 7)	333202	NR_029884	723860		NR_029884	ENSMUSG00000065457	**Mir383**	Mirn383|mir-383|mmu-mir-383	microRNA 383	Amp	ncRNA
2577_T12,T22,T3,T42	chr4	3.6E+07	3.6E+07	999	+	**4**	**50**	NA	intron (NM_001166000, intron 1 of 8)	intron (NM_001166000, intron 1 of 8)	184453	NR_030545	1E+08		NR_030545	ENSMUSG00000077851	**Mir876**	Mirn876|mir-876|mmu-mir-876	microRNA 876	Amp	ncRNA
2541_T2,T32,T3,T4	chr4	1.8E+07	1.8E+07	2999	+	**4**	**50**	NA	Intergenic	B1_Mus2|SINE|Alu	472518	NM_019724	17389	Mm.187315	NM_019724	ENSMUSG00000028226	**Mmp16**	MT-MMP3|MT3-MMP|Mt3mmp	matrix metallopeptidase 16	Amp	protein-coding
2268_T22,T2,T42,T4	chr3	5.6E+07	5.6E+07	4999	+	**4**	**50**	NA	Intergenic	L1Md_A|LINE|L1	-192299	NM_030595	26422	Mm.384353	NM_030595	ENSMUSG00000027799	**Nbea**	Lyst2|mKIAA1544	neurobeachin	Amp	protein-coding
2279_T1,T2,T32,T42	chr3	5.7E+07	5.7E+07	999	+	**4**	**50**	NA	Intergenic	Lx8|LINE|L1	-337299	NM_030595	26422	Mm.384353	NM_030595	ENSMUSG00000027799	**Nbea**	Lyst2|mKIAA1544	neurobeachin	Amp	protein-coding
1736_T1,T22,T3,T4	chr3	2.6E+07	2.6E+07	999	+	**4**	**50**	NA	intron (NM_138666, intron 3 of 7)	RLTR12A|LTR|ERVK	117307	NM_001163387	192167	Mm.316080	NM_138666	ENSMUSG00000063887	**Nlgn1**	6330415N05Rik|BB179718|NL1|Nlg1|mKIAA1070	neuroligin 1	Amp	protein-coding
1738_T1,T22,T3,T4	chr3	2.6E+07	2.6E+07	1999	+	**4**	**50**	NA	intron (NM_138666, intron 3 of 7)	intron (NM_138666, intron 3 of 7)	111807	NM_001163387	192167	Mm.316080	NM_138666	ENSMUSG00000063887	**Nlgn1**	6330415N05Rik|BB179718|NL1|Nlg1|mKIAA1070	neuroligin 1	Amp	protein-coding
533_T12,T22,T2,T42	chr13	7.9E+07	7.9E+07	999	+	**4**	**50**	NA	Intergenic	Intergenic	-878018	NM_010151	13865	Mm.439653	NM_010151	ENSMUSG00000069171	**Nr2f1**	COUP-TF1|COUP-TFI|COUPTFA|EAR-3|EAR3|Erbal3|SVP44|Tcfcoup1	nuclear receptor subfamily 2, group F, member 1	Amp	protein-coding
536_T12,T1,T2,T42	chr13	7.9E+07	7.9E+07	999	+	**4**	**50**	NA	Intergenic	L1Md_T|LINE|L1	-928018	NM_010151	13865	Mm.439653	NM_010151	ENSMUSG00000069171	**Nr2f1**	COUP-TF1|COUP-TFI|COUPTFA|EAR-3|EAR3|Erbal3|SVP44|Tcfcoup1	nuclear receptor subfamily 2, group F, member 1	Amp	protein-coding

1279_T12,T2,T32,T3	chr17	6E+07	6E+07	999	+	**4**	**50**	NA	Intergenic	L1_Mur2|LINE|L1	-625678	NM_026497	67993	Mm.36507	NM_026497	ENSMUSG00000024228	**Nudt12**	0610016O18Rik	nudix (nucleoside diphosphate linked moiety X)-type motif 12	Amp	protein-coding
632_T1,T22,T2,T32	chr14	8.1E+07	8.1E+07	999	+	**4**	**50**	NA	Intergenic	(TG)n|Simple_repeat|Simple_repeat	947698	NM_001030294	380924	Mm.26456	NM_001030294	ENSMUSG00000022026	**Olfm4**	GC1|GW112|Gm296|Gm913|OlfD|pPD4	olfactomedin 4	Amp	protein-coding
1911_T1,T22,T32,T3	chr3	4.6E+07	4.6E+07	999	+	**4**	**50**	NA	Intergenic	Intergenic	440941	NM_001101479	241989	Mm.119234	NM_001101479	ENSMUSG00000090919	**Pabpc4l**	C330050A14Rik|EG241989	poly(A) binding protein, cytoplasmic 4-like	Amp	protein-coding
1912_T12,T2,T42,T4	chr3	4.6E+07	4.6E+07	999	+	**4**	**50**	NA	Intergenic	L1Md_F2|LINE|L1	439941	NM_001101479	241989	Mm.119234	NM_001101479	ENSMUSG00000090919	**Pabpc4l**	C330050A14Rik|EG241989	poly(A) binding protein, cytoplasmic 4-like	Amp	protein-coding
1930_T1,T22,T3,T42	chr3	4.6E+07	4.6E+07	999	+	**4**	**50**	NA	Intergenic	Intergenic	307941	NM_001101479	241989	Mm.119234	NM_001101479	ENSMUSG00000090919	**Pabpc4l**	C330050A14Rik|EG241989	poly(A) binding protein, cytoplasmic 4-like	Amp	protein-coding
1939_T12,T22,T2,T4	chr3	4.6E+07	4.6E+07	999	+	**4**	**50**	NA	Intergenic	Intergenic	241941	NM_001101479	241989	Mm.119234	NM_001101479	ENSMUSG00000090919	**Pabpc4l**	C330050A14Rik|EG241989	poly(A) binding protein, cytoplasmic 4-like	Amp	protein-coding
1945_T12,T32,T3,T4	chr3	4.7E+07	4.7E+07	999	+	**4**	**50**	NA	Intergenic	Intergenic	-143059	NM_001101479	241989	Mm.119234	NM_001101479	ENSMUSG00000090919	**Pabpc4l**	C330050A14Rik|EG241989	poly(A) binding protein, cytoplasmic 4-like	Amp	protein-coding
1950_T12,T2,T32,T3	chr3	4.7E+07	4.7E+07	999	+	**4**	**50**	NA	Intergenic	L1_Mus1|LINE|L1	-194059	NM_001101479	241989	Mm.119234	NM_001101479	ENSMUSG00000090919	**Pabpc4l**	C330050A14Rik|EG241989	poly(A) binding protein, cytoplasmic 4-like	Amp	protein-coding
1959_T12,T1,T2,T32	chr3	4.7E+07	4.7E+07	999	+	**4**	**50**	NA	Intergenic	Intergenic	-218059	NM_001101479	241989	Mm.119234	NM_001101479	ENSMUSG00000090919	**Pabpc4l**	C330050A14Rik|EG241989	poly(A) binding protein, cytoplasmic 4-like	Amp	protein-coding
651_T22,T32,T3,T4	chr14	8.3E+07	8.3E+07	8999	+	**4**	**50**	NA	Intergenic	Intergenic	-1E+06	NM_001013753	219228	Mm.153643	NM_001013753	ENSMUSG00000035566	**Pcdh17**	C030033F14Rik|Gm78	protocadherin 17	Amp	protein-coding
660_T22,T32,T3,T4	chr14	8.4E+07	8.4E+07	999	+	**4**	**50**	NA	Intergenic	L1Md_F2|LINE|L1	-697563	NM_001013753	219228	Mm.153643	NM_001013753	ENSMUSG00000035566	**Pcdh17**	C030033F14Rik|Gm78	protocadherin 17	Amp	protein-coding
2117_T1,T32,T3,T4	chr3	4.9E+07	4.9E+07	3999	+	**4**	**50**	NA	Intergenic	IAPEY3-int|LTR|ERVK	462816	NM_130448	73173	Mm.87246	NM_130448	ENSMUSG00000037892	**Pcdh18**	3110038E07Rik|BB095589|PCDH68L	protocadherin 18	Amp	protein-coding
2119_T2,T3,T42,T4	chr3	4.9E+07	4.9E+07	999	+	**4**	**50**	NA	Intergenic	MT2B|LTR|ERVL	314316	NM_130448	73173	Mm.87246	NM_130448	ENSMUSG00000037892	**Pcdh18**	3110038E07Rik|BB095589|PCDH68L	protocadherin 18	Amp	protein-coding
2121_T2,T3,T42,T4	chr3	4.9E+07	4.9E+07	999	+	**4**	**50**	NA	Intergenic	Intergenic	307316	NM_130448	73173	Mm.87246	NM_130448	ENSMUSG00000037892	**Pcdh18**	3110038E07Rik|BB095589|PCDH68L	protocadherin 18	Amp	protein-coding
2140_T1,T32,T3,T42	chr3	5E+07	5E+07	1999	+	**4**	**50**	NA	Intergenic	L1Md_T|LINE|L1	-62184	NM_130448	73173	Mm.87246	NM_130448	ENSMUSG00000037892	**Pcdh18**	3110038E07Rik|BB095589|PCDH68L	protocadherin 18	Amp	protein-coding
2149_T12,T22,T3,T4	chr3	5E+07	5E+07	2999	+	**4**	**50**	NA	Intergenic	L1Md_T|LINE|L1	-333684	NM_130448	73173	Mm.87246	NM_130448	ENSMUSG00000037892	**Pcdh18**	3110038E07Rik|BB095589|PCDH68L	protocadherin 18	Amp	protein-coding

688_T32,T3,T42,T4	chr14	8.8E+07	8.8E+07	3999	+	**4**	**50**	NA	Intergenic	Intergenic	217896	NM_178685	219257	Mm.128861	NM_178685	ENSMUSG00000050505	**Pcdh20**	C630015B17Rik|Pcdh13	protocadherin 20	Amp	protein-coding
746_T12,T1,T3,T4	chr14	9.4E+07	9.4E+07	6999	+	**4**	**50**	NA	intron (NM_001081377, intron 1 of 3)	intron (NM_001081377, intron 1 of 3)	24888	NM_001271800	211712	Mm.37126	NM_001081377	ENSMUSG00000055421	**Pcdh9**	-	protocadherin 9	Amp	protein-coding
2715_T12,T1,T22,T32	chr5	1.4E+07	1.5E+07	3999	+	**4**	**50**	NA	Intergenic	Intergenic	-15418	NM_011995	26875	Mm.146275	NM_011995	ENSMUSG00000061601	**Pclo**	Acz|Pico	piccolo (presynaptic cytomatrix protein)	Amp	protein-coding
54_T1,T2,T3,T42	chr1	9.4E+07	9.4E+07	4999	+	**4**	**50**	NA	Intergenic	Lx7|LINE|L1	-371447	NM_008798	18566	Mm.5024	NM_008798	ENSMUSG00000026285	**Pdcd1**	Ly101|PD-1|Pdc1	programmed cell death 1	Amp	protein-coding
2425_T2,T32,T3,T4	chr3	8.1E+07	8.1E+07	999	+	**4**	**50**	NA	Intergenic	(TAGA)n|Simple_repeat|Simple_repeat	379584	NM_019971	54635	Mm.331089	NM_019971	ENSMUSG00000028019	**Pdgfc**	1110064L01Rik|AI647969|PDGF-C	platelet-derived growth factor, C polypeptide	Amp	protein-coding
3684_T12,T1,T22,T2	chr9	5950501	5951500	999	+	**4**	**50**	NA	Intergenic	Intergenic	-217612	NM_027924	71785	Mm.390122	NM_027924	ENSMUSG00000032006	**Pdgfd**	1110003I09Rik	platelet-derived growth factor, D polypeptide	Amp	protein-coding
2436_T12,T22,T32,T42	chr3	9.2E+07	9.2E+07	999	+	**4**	**50**	NA	Intergenic	L1Md_F2|LINE|L1	-201583	NM_207247	242100	Mm.297406	NM_207247	ENSMUSG00000042244	**Pglyrp3**	Gm420	peptidoglycan recognition protein 3	Amp	protein-coding
3341_T1,T32,T42,T4	chr7	3.8E+07	3.8E+07	2999	+	**4**	**50**	NA	Intergenic	L1Md_F2|LINE|L1	-79652	NM_025390	66161	Mm.22284	NM_025390	ENSMUSG00000030423	**Pop4**	1110023P21Rik|Rpp29	processing of precursor 4, ribonuclease P/MRP family, (S. cerevisiae)	Amp	protein-coding
1442_T22,T2,T3,T4	chr2	4.1E+07	4.1E+07	999	+	**4**	**50**	NA	intron (NM_053011, intron 82 of 90)	intron (NM_053011, intron 82 of 90)	-1E+06	NM_024209	67857	Mm.389242	NM_024209	ENSMUSG00000026753	**Ppp6c**	2310003C10Rik	protein phosphatase 6, catalytic subunit	Amp	protein-coding
1415_T22,T2,T3,T4	chr19	6E+07	6E+07	999	+	**4**	**50**	NA	Intergenic	MTE2a|LTR|ERVL-MaLR	92304	NM_201615	226278	Mm.377241	NM_201615	ENSMUSG00000045052	**Prlhr**	Gm339|Gpr10|Gr3|Prrpr	prolactin releasing hormone receptor	Amp	protein-coding
1417_T22,T2,T3,T4	chr19	6.1E+07	6.1E+07	999	+	**4**	**50**	NA	Intergenic	Lx3B|LINE|L1	-51696	NM_201615	226278	Mm.377241	NM_201615	ENSMUSG00000045052	**Prlhr**	Gm339|Gpr10|Gr3|Prrpr	prolactin releasing hormone receptor	Amp	protein-coding
2452_T22,T32,T3,T4	chr3	1.1E+08	1.1E+08	999	+	**4**	**50**	NA	Intergenic	Lx8|LINE|L1	-250001	NR_024139	99890	Mm.36115	NM_178891	ENSMUSG00000049300	**Prmt6**	AW124876|BB233495|Hrmt1l6	protein arginine N-methyltransferase 6	Amp	protein-coding
2458_T22,T2,T42,T4	chr3	1.1E+08	1.1E+08	999	+	**4**	**50**	NA	Intergenic	Intergenic	-498001	NR_024139	99890	Mm.36115	NM_178891	ENSMUSG00000049300	**Prmt6**	AW124876|BB233495|Hrmt1l6	protein arginine N-methyltransferase 6	Amp	protein-coding
1338_T1,T3,T42,T4	chr19	1.9E+07	1.9E+07	3999	+	**4**	**50**	NA	Intergenic	L1_Mus3|LINE|L1	-281304	NM_001043354	225998	Mm.234641	NM_146095	ENSMUSG00000036192	**Rorb**	Nr1f2|RZR-beta|RZRB|Rorbeta|hstp	RAR-related orphan receptor beta	Amp	protein-coding
3474_T22,T2,T3,T4	chr8	3.8E+07	3.8E+07	3999	+	**4**	**50**	NA	intron (NM_145841, intron 1 of 7)	intron (NM_145841, intron 1 of 7)	188008	NM_145841	244431	Mm.210388	NM_145841	ENSMUSG00000039539	**Sgcz**	C230085N17Rik	sarcoglycan zeta	Amp	protein-coding

3396_T12,T1,T32,T3	chr8	5020501	5021500	999	+	**4**	**50**	NA	Intergenic	Intergenic	84232	NM_011388	20494	Mm.3500	NM_011388	ENSMUSG00000023073	**Slc10a2**	9130221J18Rik|AI605518|ASBT|IBAT|ISBT	solute carrier family 10, member 2	Amp	protein-coding
3402_T22,T2,T3,T42	chr8	6389501	6390500	999	+	**4**	**50**	NA	Intergenic	Intergenic	-1E+06	NM_011388	20494	Mm.3500	NM_011388	ENSMUSG00000023073	**Slc10a2**	9130221J18Rik|AI605518|ASBT|IBAT|ISBT	solute carrier family 10, member 2	Amp	protein-coding
3406_T1,T22,T3,T4	chr8	6460501	6465500	4999	+	**4**	**50**	NA	Intergenic	(TAAA)n|Simple_repeat|Simple_repeat	-1E+06	NM_011388	20494	Mm.3500	NM_011388	ENSMUSG00000023073	**Slc10a2**	9130221J18Rik|AI605518|ASBT|IBAT|ISBT	solute carrier family 10, member 2	Amp	protein-coding
3417_T1,T22,T32,T3	chr8	6835501	6836500	999	+	**4**	**50**	NA	Intergenic	Intergenic	-2E+06	NM_011388	20494	Mm.3500	NM_011388	ENSMUSG00000023073	**Slc10a2**	9130221J18Rik|AI605518|ASBT|IBAT|ISBT	solute carrier family 10, member 2	Amp	protein-coding
1080_T22,T32,T3,T42	chr15	5.7E+07	5.7E+07	999	+	**4**	**50**	NA	intron (NM_172378, intron 2 of 11)	intron (NM_172378, intron 2 of 11)	203625	NM_172378	210463	Mm.26555	NM_172378	ENSMUSG00000022366	**Slc22a22**	oat-pg	solute carrier family 22 (organic cation transporter), member 22	Amp	protein-coding
2748_T1,T32,T42,T4	chr5	4.7E+07	4.7E+07	999	+	**4**	**50**	NA	Intergenic	Lx8|LINE|L1	-658155	NR_111900	20563	Mm.289739	NM_178804	ENSMUSG00000031558	**Slit2**	Drad-1|E030015M03Rik|E130320P19Rik|Slil3|mKIAA4141|slit-2	slit homolog 2 (Drosophila)	Amp	protein-coding
2756_T12,T2,T3,T4	chr5	4.8E+07	4.8E+07	999	+	**4**	**50**	NA	Intergenic	Lx7|LINE|L1	-383155	NR_111900	20563	Mm.289739	NM_178804	ENSMUSG00000031558	**Slit2**	Drad-1|E030015M03Rik|E130320P19Rik|Slil3|mKIAA4141|slit-2	slit homolog 2 (Drosophila)	Amp	protein-coding
810_T12,T22,T2,T3	chr14	1.1E+08	1.1E+08	3999	+	**4**	**50**	NA	Intergenic	Intergenic	101739	NM_199065	76965	Mm.257268	NM_199065	ENSMUSG00000075478	**Slitrk1**	3200001I04Rik	SLIT and NTRK-like family, member 1	Amp	protein-coding
813_T12,T1,T22,T2	chr14	1.1E+08	1.1E+08	4999	+	**4**	**50**	NA	Intergenic	Intergenic	63239	NM_199065	76965	Mm.257268	NM_199065	ENSMUSG00000075478	**Slitrk1**	3200001I04Rik	SLIT and NTRK-like family, member 1	Amp	protein-coding
847_T1,T22,T42,T4	chr14	1.1E+08	1.1E+08	2999	+	**4**	**50**	NA	Intergenic	RMER1A|Other|Other	-200851	NM_175499	239250	Mm.49728	NM_175499	ENSMUSG00000045871	**Slitrk6**	4832410J21Rik|Sltk6	SLIT and NTRK-like family, member 6	Amp	protein-coding
3363_T1,T22,T3,T42	chr7	6E+07	6E+07	999	+	**4**	**50**	NA	Intergenic	Lx7|LINE|L1	9431	NR_015456	52480	Mm.482346	NR_015456	ENSMUSG00000100826	**Snhg14**	AU018661|D7Ertd715e|Lncat	small nucleolar RNA host gene 14	Amp	ncRNA
1358_T2,T32,T3,T4	chr19	5.1E+07	5.1E+07	999	+	**4**	**50**	NA	Intergenic	Intergenic	-269354	NM_001290356	58178	Mm.313672	NM_021377	ENSMUSG00000043531	**Sorcs1**	Sorcs|mSorCS	sortilin-related VPS10 domain containing receptor 1	Amp	protein-coding
1353_T22,T2,T3,T4	chr19	4.9E+07	4.9E+07	999	+	**4**	**50**	NA	Intergenic	Intergenic	963975	NM_025696	66673	Mm.70980	NM_025696	ENSMUSG00000063434	**Sorcs3**	6330404A12Rik|AW045579	sortilin-related VPS10 domain containing receptor 3	Amp	protein-coding
450_T1,T2,T3,T4	chr12	6.2E+07	6.2E+07	999	+	**4**	**50**	NA	Intergenic	Lx5c|LINE|L1	-219787	NR_131949	73309	Mm.276558	NR_131949		**Spanxn4**	1700047L15Rik	SPANX family, member N4	Amp	ncRNA
453_T22,T2,T42,T4	chr12	6.3E+07	6.3E+07	999	+	**4**	**50**	NA	Intergenic	Lx9|LINE|L1	405213	NR_131949	73309	Mm.276558	NR_131949		**Spanxn4**	1700047L15Rik	SPANX family, member N4	Amp	ncRNA

3572_T1,T32,T42,T4	chr8	6.3E+07	6.3E+07	999	+	**4**	**50**	NA	intron (NM_023689, intron 3 of 11)	intron (NM_023689, intron 3 of 11)	143655	NM_023689	72902	Mm.334552	NM_023689	ENSMUSG00000054162	**Spock3**	2900045C01Rik|AI428471|mKIAA4039	sparc/osteonectin, cwcv and kazal-like domains proteoglycan 3	Amp	protein-coding
2694_T12,T1,T22,T32	chr5	6384501	6388500	3999	+	**4**	**50**	NA	Intergenic	Intergenic	-637183	NM_027399	70358	Mm.85429	NM_027399	ENSMUSG00000015652	**Steap1**	2410007B19Rik|Prss24|Steap	six transmembrane epithelial antigen of the prostate 1	Amp	protein-coding
432_T12,T3,T42,T4	chr12	4.6E+07	4.6E+07	4999	+	**4**	**50**	NA	Intergenic	Intergenic	-799517	NM_144552	217517	Mm.285400	NM_144552	ENSMUSG00000046314	**Stxbp6**	BC024598|C85317	syntaxin binding protein 6 (amisyn)	Amp	protein-coding
499_T1,T22,T3,T42	chr13	1.7E+07	1.7E+07	3999	+	**4**	**50**	NA	intron (NM_138654, intron 11 of 13)	L1_Mus3|LINE|L1	419265	NM_138654	192136	Mm.364180	NM_138654	ENSMUSG00000055137	**Sugct**	5033411D12Rik|D17907	succinyl-CoA glutarate-CoA transferase	Amp	protein-coding
1185_T12,T22,T32,T42	chr17	7908501	7912500	3999	+	**4**	**50**	NA	Intergenic	CT-rich|Low_complexity|Low_complexity	-15500	NM_145968	72536	Mm.119680	NM_145968	ENSMUSG00000033450	**Tagap**	2610315E15Rik	T cell activation Rho GTPase activating protein	Amp	protein-coding
1377_T22,T2,T3,T42	chr19	5.7E+07	5.7E+07	999	+	**4**	**50**	NA	intron (NM_001002238, intron 3 of 25)	intron (NM_001002238, intron 3 of 25)	4288	NM_001002241	83561	Mm.247541	NM_031387	ENSMUSG00000025081	**Tdrd1**	MTR-1	tudor domain containing 1	Amp	protein-coding
1379_T22,T2,T3,T4	chr19	5.7E+07	5.7E+07	999	+	**4**	**50**	NA	intron (NM_001002238, intron 3 of 25)	L1MD1|LINE|L1	6288	NM_001002241	83561	Mm.247541	NM_031387	ENSMUSG00000025081	**Tdrd1**	MTR-1	tudor domain containing 1	Amp	protein-coding
2931_T12,T22,T3,T4	chr6	1.7E+07	1.7E+07	999	+	**4**	**50**	NA	intron (NM_207176, intron 1 of 6)	intron (NM_207176, intron 1 of 6)	8851	NM_207176	21753	Mm.436548	NM_011570	ENSMUSG00000029552	**Tes**	D6Ertd352e|TESS|Tes1|Tes2|testin2	testis derived transcript	Amp	protein-coding
2921_T12,T1,T32,T4	chr6	1.7E+07	1.7E+07	999	+	**4**	**50**	NA	intron (NM_031198, intron 1 of 6)	intron (NM_031198, intron 1 of 6)	6441	NM_031198	21426	Mm.36217	NM_031198	ENSMUSG00000029553	**Tfec**	BB107417|Tcfec|bHLHe34	transcription factor EC	Amp	protein-coding
2599_T22,T2,T32,T4	chr4	6.7E+07	6.7E+07	5999	+	**4**	**50**	NA	Intergenic	Intergenic	555949	NM_021297	21898	Mm.38049	NM_021297	ENSMUSG00000039005	**Tlr4**	Lps|Ly87|Ran/M1|Rasl2-8	toll-like receptor 4	Amp	protein-coding
2293_T12,T1,T32,T42	chr3	5.7E+07	5.7E+07	999	+	**4**	**50**	NA	Intergenic	B3|SINE|B2	335919	NM_008536	17112	Mm.856	NM_008536	ENSMUSG00000027800	**Tm4sf1**	L6|M3s1	transmembrane 4 superfamily member 1	Amp	protein-coding
2320_T12,T22,T42,T4	chr3	5.7E+07	5.7E+07	7999	+	**4**	**50**	NA	Intergenic	MERVL-int|LTR|ERVL	-49910	NM_145539	229302	Mm.26618	NM_145539	ENSMUSG00000027801	**Tm4sf4**	Iltmp	transmembrane 4 superfamily member 4	Amp	protein-coding
36_T1,T2,T42,T4	chr1	5.1E+07	5.1E+07	3999	+	**4**	**50**	NA	Intergenic	Intergenic	-282023	NM_019790	56363	Mm.245154	NM_019790	ENSMUSG00000026109	**Tmeff2**	4832418D20Rik|7630402F16Rik|TR-2	transmembrane protein with EGF-like and two follistatin-like domains 2	Amp	protein-coding

484_T22,T2,T32,T3	chr12	1.2E+08	1.2E+08	999	+	**4**	**50**	NA	intron (NM_001294154, intron 2 of 4)	intron (NM_001294154, intron 2 of 4)	66038	NM_001160385	217951	Mm.387395	NM_001160385	ENSMUSG00000048004	**Tmem196**	Gm528	transmembrane protein 196	Amp	protein-coding
617_T12,T1,T22,T3	chr14	6.1E+07	6.1E+07	999	+	**4**	**50**	NA	Intergenic	Intergenic	78987	NM_001164155	29820	Mm.281356	NM_013869	ENSMUSG00000060548	**Tnfrsf19**	AL023044|AW123854|TAJ|TAJ-ALPHA|TRADE|Troy	tumor necrosis factor receptor superfamily, member 19	Amp	protein-coding
1053_T1,T22,T3,T4	chr15	5E+07	5E+07	4999	+	**4**	**50**	NA	Intergenic	Intergenic	413049	NM_001310481	83925	Mm.30466	NM_032000	ENSMUSG00000038679	**Trps1**	AI115454|AI447310|D15Ertd586e	trichorhinophalangeal syndrome I (human)	Amp	protein-coding
2336_T12,T2,T3,T4	chr3	5.8E+07	5.8E+07	999	+	**4**	**50**	NA	promoter-TSS (NM_001081229)	promoter-TSS (NM_001081229)	-689	NM_001081229	72033	Mm.218409	NM_001081229	ENSMUSG00000027806	**Tsc22d2**	1810043J12Rik|5530402M19Rik	TSC22 domain family, member 2	Amp	protein-coding
2346_T22,T2,T42,T4	chr3	5.8E+07	5.8E+07	999	+	**4**	**50**	NA	intron (NM_001081229, intron 2 of 3)	intron (NM_001081229, intron 2 of 3)	17311	NM_001081229	72033	Mm.218409	NM_001081229	ENSMUSG00000027806	**Tsc22d2**	1810043J12Rik|5530402M19Rik	TSC22 domain family, member 2	Amp	protein-coding
1506_T22,T2,T32,T3	chr2	6.6E+07	6.6E+07	999	+	**4**	**50**	NA	intron (NM_001313997, intron 24 of 26)	L1_Mur1|LINE|L1	-27383	NM_001290669	73668	Mm.250868	NM_001047604	ENSMUSG00000034848	**Ttc21b**	2410066K11Rik|Thm1|aln|mKIAA1992	tetratricopeptide repeat domain 21B	Amp	protein-coding
2651_T1,T22,T2,T32	chr4	9.3E+07	9.3E+07	3999	+	**4**	**50**	NA	Intergenic	Intergenic	21011	NM_026954	69136	Mm.171558	NM_026954	ENSMUSG00000054000	**Tusc1**	2200001D17Rik|TSG-9	tumor suppressor candidate 1	Amp	protein-coding
2657_T22,T2,T42,T4	chr4	9.4E+07	9.4E+07	1999	+	**4**	**50**	NA	Intergenic	Intergenic	-485989	NM_026954	69136	Mm.171558	NM_026954	ENSMUSG00000054000	**Tusc1**	2200001D17Rik|TSG-9	tumor suppressor candidate 1	Amp	protein-coding
3441_T1,T2,T3,T4	chr8	3E+07	3E+07	999	+	**4**	**50**	NA	Intergenic	Intergenic	-522364	NM_153135	210801	Mm.248433	NM_153135	ENSMUSG00000063626	**Unc5d**	D930029E11Rik|Unc5h4|mKIAA1777	unc-5 netrin receptor D	Amp	protein-coding
360_T12,T1,T22,T3	chr11	1.6E+07	1.6E+07	2999	+	**4**	**50**	NA	Intergenic	ERVB5_2-LTR_MM|LTR|ERVK	-686724	NM_001290539	211739	Mm.284015	NM_145967	ENSMUSG00000048834	**Vstm2a**	Vstm2	V-set and transmembrane domain containing 2A	Amp	protein-coding
1700_T12,T22,T2,T3	chr3	1.6E+07	1.6E+07	999	+	**4**	**50**	NA	TTS (NM_001145919)	TTS (NM_001145919)	34817	NM_001145919	229096	Mm.23834	NM_172677	ENSMUSG00000047213	**Ythdf3**	9130022A11Rik	YTH domain family 3	Amp	protein-coding
1695_T12,T22,T3,T4	chr3	1.6E+07	1.6E+07	999	+	**4**	**50**	NA	intron (NR_027375, intron 4 of 5)	intron (NR_027375, intron 4 of 5)	7817	NM_001145919	229096	Mm.23834	NM_172677	ENSMUSG00000047213	**Ythdf3**	9130022A11Rik	YTH domain family 3	Amp	protein-coding
1662_T12,T1,T32,T4	chr3	7539501	7540500	999	+	**4**	**50**	NA	intron (NM_173181, intron 8 of 8)	intron (NM_173181, intron 8 of 8)	36574	NM_173181	67306	Mm.332366	NM_173181	ENSMUSG00000043542	**Zc2hc1a**	3110050N22Rik|AI790358|AU023959|Fam164a	zinc finger, C2HC-type containing 1A	Amp	protein-coding

336_T12,T1,T2	chr10	1.1E+08	1.1E+08	999	+	**3**	**37.5**	NA	intron (NM_198636, intron 1 of 10)	intron (NM_198636, intron 1 of 10)	15664	NM_001142804	380660	Mm.336072	NM_198636	ENSMUSG00000035948	**Acss3**	8430416H19Rik|Gm874	acyl-CoA synthetase short-chain family member 3	Amp	protein-coding
621_T12,T1,T3	chr14	6.9E+07	6.9E+07	5999	+	**3**	**37.5**	NA	Intergenic	L1M3c|LINE|L1	-126658	NM_183366	13522	Mm.117450	NM_010082	ENSMUSG00000014725	**Adam28**	C130072N01Rik|D430033C21Rik|Dtgn1|MDC-L|MDCL|TECADAM|eMDCII	a disintegrin and metallopeptidase domain 28	Amp	protein-coding
2764_T12,T1,T4	chr5	5E+07	5E+07	999	+	**3**	**37.5**	NA	Intergenic	Intergenic	382996	NM_133911	70693	Mm.272974	NM_133911	ENSMUSG00000029090	**Adgra3**	3830613O22Rik|AU044632|Gpr125|Tem5-like	adhesion G protein-coupled receptor A3	Amp	protein-coding
3530_T1,T22,T4	chr8	5.3E+07	5.3E+07	999	+	**3**	**37.5**	NA	Intergenic	Intergenic	-835702	NM_001205054	11593	Mm.334535	NM_001005847	ENSMUSG00000031521	**Aga**	AW060726	aspartylglucosaminidase	Amp	protein-coding
3535_T2,T32,T4	chr8	5.3E+07	5.3E+07	999	+	**3**	**37.5**	NA	Intergenic	Intergenic	-776702	NM_001205054	11593	Mm.334535	NM_001005847	ENSMUSG00000031521	**Aga**	AW060726	aspartylglucosaminidase	Amp	protein-coding
972_T22,T32,T3	chr15	4.2E+07	4.2E+07	3999	+	**3**	**37.5**	NA	intron (NM_001286062, intron 4 of 8)	intron (NM_001286062, intron 4 of 8)	193477	NM_001286062	11600	Mm.309336	NM_009640	ENSMUSG00000022309	**Angpt1**	1110046O21Rik|Ang-1|Ang1	angiopoietin 1	Amp	protein-coding
2988_T22,T2,T3	chr6	1.9E+07	1.9E+07	999	+	**3**	**37.5**	NA	Intergenic	RLTR11A|LTR|ERVK	115682	NM_001167757	75196	Mm.67665	NM_029202	ENSMUSG00000029517	**Ankrd7**	4930532L20Rik|AV278427	ankyrin repeat domain 7	Amp	protein-coding
2824_T12,T22,T32	chr5	6.2E+07	6.2E+07	999	+	**3**	**37.5**	NA	Intergenic	Lx7|LINE|L1	418177	NM_178407	212285	Mm.244403	NM_178407	ENSMUSG00000037999	**Arap2**	Centd1|Gm148|mKIAA0580	ArfGAP with RhoGAP domain, ankyrin repeat and PH domain 2	Amp	protein-coding
444_T12,T32,T3	chr12	5.3E+07	5.3E+07	999	+	**3**	**37.5**	NA	intron (NM_009706, intron 1 of 5)	intron (NM_009706, intron 1 of 5)	5923	NM_009706	11855	Mm.35059	NM_009706	ENSMUSG00000035133	**Arhgap5**	p190-B|p190B	Rho GTPase activating protein 5	Amp	protein-coding
547_T1,T22,T3	chr13	8E+07	8E+07	4999	+	**3**	**37.5**	NA	Intergenic	Intergenic	-443422	NM_001042591	105171	Mm.423137	NM_001042591	ENSMUSG00000074794	**Arrdc3**	AI450344|mKIAA1376	arrestin domain containing 3	Amp	protein-coding
1590_T22,T42,T4	chr2	1.1E+08	1.1E+08	999	+	**3**	**37.5**	NA	Intergenic	Intergenic	-149700	NM_007540	12064	Mm.1442	NM_007540	ENSMUSG00000048482	**Bdnf**	-	brain derived neurotrophic factor	Amp	protein-coding
2609_T22,T2,T32	chr4	6.9E+07	6.9E+07	3999	+	**3**	**37.5**	NA	Intergenic	Intergenic	-393103	NM_019967	56710	Mm.248788	NM_019967	ENSMUSG00000028351	**Brinp1**	BRINP|Dbc1|Dbccr1|Fam5a	bone morphogenic protein/retinoic acid inducible neural specific 1	Amp	protein-coding
178_T22,T2,T3	chr1	1.5E+08	1.5E+08	4999	+	**3**	**37.5**	NA	Intergenic	Intergenic	-627666	NM_153539	215378	Mm.441817	NM_153539	ENSMUSG00000035131	**Brinp3**	B830045N13Rik|Fam5c	bone morphogenetic protein/retinoic acid inducible neural specific 3	Amp	protein-coding

1373_T3,T42,T4	chr19	5.6E+07	5.6E+07	999	+	**3**	**37.5**	NA	intron (NM_007611, intron 2 of 6)	RLTR31B2|LTR|ERVK	12871	NM_007611	12369	Mm.35687	NM_007611	ENSMUSG00000025076	**Casp7**	AI314680|CMH-1|ICE-IAP3|Mch3|caspase-7|mCASP-7	caspase 7	Amp	protein-coding
1375_T3,T42,T4	chr19	5.6E+07	5.6E+07	999	+	**3**	**37.5**	NA	intron (NM_007611, intron 2 of 6)	L1Md_T|LINE|L1	18871	NM_007611	12369	Mm.35687	NM_007611	ENSMUSG00000025076	**Casp7**	AI314680|CMH-1|ICE-IAP3|Mch3|caspase-7|mCASP-7	caspase 7	Amp	protein-coding
378_T12,T32,T42	chr11	4.1E+07	4.1E+07	999	+	**3**	**37.5**	NA	Intergenic	ORR1E|LTR|ERVL-MaLR	-547714	NM_009831	12450	Mm.2103	NM_009831	ENSMUSG00000020326	**Ccng1**	AI314029	cyclin G1	Amp	protein-coding
412_T12,T22,T4	chr11	1.1E+08	1.1E+08	999	+	**3**	**37.5**	NA	Intergenic	RLTR44-int|LTR|ERVK	9886	NM_145437	217305	Mm.275986	NM_145437	ENSMUSG00000034641	**Cd300ld**	4732429D16Rik|CLM5|Cd300ld1|LMIR4|MAIR-IV|clm-5	CD300 molecule like family member d	Amp	protein-coding
924_T1,T22,T3	chr15	1.9E+07	1.9E+07	999	+	**3**	**37.5**	NA	Intergenic	Intergenic	-79947	NM_001316758	320873	Mm.117794	NM_009865	ENSMUSG00000022321	**Cdh10**	A830016G23Rik|C030003B10Rik|C030011H18Rik	cadherin 10	Amp	protein-coding
3647_T1,T32,T4	chr8	1E+08	1E+08	999	+	**3**	**37.5**	NA	Intergenic	Intergenic	1587111	NM_009866	12552	Mm.1571	NM_009866	ENSMUSG00000031673	**Cdh11**	Cad11|OSF-4	cadherin 11	Amp	protein-coding
3657_T2,T32,T4	chr8	1E+08	1E+08	2999	+	**3**	**37.5**	NA	Intergenic	Intergenic	1145111	NM_009866	12552	Mm.1571	NM_009866	ENSMUSG00000031673	**Cdh11**	Cad11|OSF-4	cadherin 11	Amp	protein-coding
3660_T22,T42,T4	chr8	1E+08	1E+08	4999	+	**3**	**37.5**	NA	Intergenic	RLTR17B_Mm|LTR|ERVK	1047111	NM_009866	12552	Mm.1571	NM_009866	ENSMUSG00000031673	**Cdh11**	Cad11|OSF-4	cadherin 11	Amp	protein-coding
3662_T1,T22,T2	chr8	1E+08	1E+08	999	+	**3**	**37.5**	NA	Intergenic	Intergenic	756111	NM_009866	12552	Mm.1571	NM_009866	ENSMUSG00000031673	**Cdh11**	Cad11|OSF-4	cadherin 11	Amp	protein-coding
3667_T1,T22,T2	chr8	1E+08	1E+08	12999	+	**3**	**37.5**	NA	Intergenic	Intergenic	363111	NM_009866	12552	Mm.1571	NM_009866	ENSMUSG00000031673	**Cdh11**	Cad11|OSF-4	cadherin 11	Amp	protein-coding
952_T1,T2,T3	chr15	2.2E+07	2.2E+07	999	+	**3**	**37.5**	NA	intron (NM_001008420, intron 8 of 11)	intron (NM_001008420, intron 8 of 11)	463548	NM_001008420	215654	Mm.300909	NM_001008420	ENSMUSG00000040452	**Cdh12**	Cdhb	cadherin 12	Amp	protein-coding
960_T12,T1,T42	chr15	2.4E+07	2.4E+07	999	+	**3**	**37.5**	NA	Intergenic	Intergenic	838537	NM_001081299	320865	Mm.241965	NM_001081299	ENSMUSG00000040420	**Cdh18**	B230220E17Rik|Cdh14l	cadherin 18	Amp	protein-coding
961_T2,T32,T42	chr15	2.4E+07	2.4E+07	5999	+	**3**	**37.5**	NA	Intergenic	Intergenic	981037	NM_001081299	320865	Mm.241965	NM_001081299	ENSMUSG00000040420	**Cdh18**	B230220E17Rik|Cdh14l	cadherin 18	Amp	protein-coding
106_T22,T2,T3	chr1	1E+08	1E+08	1999	+	**3**	**37.5**	NA	Intergenic	Lx|LINE|L1	-510319	NM_011800	23836	Mm.103640	NM_011800	ENSMUSG00000050840	**Cdh20**	Cdh7	cadherin 20	Amp	protein-coding

3622_T1,T32,T4	chr8	9.9E+07	9.9E+07	999	+	**3**	**37.5**	NA	intron (NR_133570, intron 3 of 10)	intron (NR_133570, intron 3 of 10)	142471	NM_001285913	12564	Mm.441131	NM_007667	ENSMUSG00000036510	**Cdh8**	AI851472|cad8	cadherin 8	Amp	protein-coding
892_T12,T1,T4	chr15	1.5E+07	1.5E+07	999	+	**3**	**37.5**	NA	Intergenic	IMPB_01|Satellite|Satellite	-1E+06	NM_009869	12565	Mm.439758	NM_009869	ENSMUSG00000025370	**Cdh9**	-	cadherin 9	Amp	protein-coding
898_T2,T3,T4	chr15	1.6E+07	1.6E+07	1999	+	**3**	**37.5**	NA	Intergenic	Intergenic	-1E+06	NM_009869	12565	Mm.439758	NM_009869	ENSMUSG00000025370	**Cdh9**	-	cadherin 9	Amp	protein-coding
3098_T2,T42,T4	chr6	1E+08	1E+08	999	+	**3**	**37.5**	NA	Intergenic	L1Md_A|LINE|L1	-98876	NM_007697	12661	Mm.251288	NM_007697	ENSMUSG00000030077	**Chl1**	A530023M13Rik|AI465420|CALL|LICAM2	cell adhesion molecule L1-like	Amp	protein-coding
2545_T2,T32,T3	chr4	1.9E+07	1.9E+07	3999	+	**3**	**37.5**	NA	Intergenic	(TAGA)n|Simple_repeat|Simple_repeat	-28350	NM_013927	30952	Mm.445778	NM_013927	ENSMUSG00000056494	**Cngb3**	CCNC2|CNG6|Cngbeta2	cyclic nucleotide gated channel beta 3	Amp	protein-coding
3100_T12,T32,T4	chr6	1E+08	1E+08	999	+	**3**	**37.5**	NA	intron (NM_017383, intron 6 of 22)	intron (NM_017383, intron 6 of 22)	234956	NM_017383	53870	Mm.321671	NM_017383	ENSMUSG00000030092	**Cntn6**	NB-3	contactin 6	Amp	protein-coding
3102_T1,T32,T4	chr6	1E+08	1E+08	999	+	**3**	**37.5**	NA	intron (NM_017383, intron 7 of 22)	L1MEg|LINE|L1	239956	NM_017383	53870	Mm.321671	NM_017383	ENSMUSG00000030092	**Cntn6**	NB-3	contactin 6	Amp	protein-coding
3050_T32,T3,T42	chr6	4.5E+07	4.5E+07	999	+	**3**	**37.5**	NA	intron (NM_001004357, intron 1 of 23)	intron (NM_001004357, intron 1 of 23)	52939	NM_001004357	66797	Mm.440084	NM_025771	ENSMUSG00000039419	**Cntnap2**	5430425M22Rik|Caspr2|mKIAA0868	contactin associated protein-like 2	Amp	protein-coding
3055_T12,T1,T32	chr6	4.5E+07	4.5E+07	4999	+	**3**	**37.5**	NA	intron (NM_001004357, intron 1 of 23)	intron (NM_001004357, intron 1 of 23)	229939	NM_001004357	66797	Mm.440084	NM_025771	ENSMUSG00000039419	**Cntnap2**	5430425M22Rik|Caspr2|mKIAA0868	contactin associated protein-like 2	Amp	protein-coding
23_T32,T42,T4	chr1	4.5E+07	4.5E+07	999	+	**3**	**37.5**	NA	Intergenic	L1_Mur1|LINE|L1	-133538	NM_009930	12825	Mm.249555	NM_009930	ENSMUSG00000026043	**Col3a1**	AW550625|Col3a-1|Tsk-2|Tsk2	collagen, type III, alpha 1	Amp	protein-coding
565_T2,T32,T3	chr13	8.6E+07	8.6E+07	4999	+	**3**	**37.5**	NA	Intergenic	Intergenic	-266205	NM_007749	12867	Mm.378898	NM_007749	ENSMUSG00000017778	**Cox7c**	COXVIIc|Cox7c1	cytochrome c oxidase subunit VIIc	Amp	protein-coding
567_T32,T3,T42	chr13	8.6E+07	8.6E+07	4999	+	**3**	**37.5**	NA	Intergenic	Lx6|LINE|L1	-286205	NM_007749	12867	Mm.378898	NM_007749	ENSMUSG00000017778	**Cox7c**	COXVIIc|Cox7c1	cytochrome c oxidase subunit VIIc	Amp	protein-coding
571_T22,T42,T4	chr13	8.6E+07	8.6E+07	3999	+	**3**	**37.5**	NA	Intergenic	MYSERV6-int|LTR|ERVK	-442705	NM_007749	12867	Mm.378898	NM_007749	ENSMUSG00000017778	**Cox7c**	COXVIIc|Cox7c1	cytochrome c oxidase subunit VIIc	Amp	protein-coding
1019_T12,T22,T3	chr15	4.8E+07	4.8E+07	999	+	**3**	**37.5**	NA	intron (NM_001081391, intron 13 of 70)	intron (NM_001081391, intron 13 of 70)	641989	NM_001081391	239420	Mm.309363	NM_001081391	ENSMUSG00000022311	**Csmd3**	4930500N14Rik|mKIAA1894	CUB and Sushi multiple domains 3	Amp	protein-coding

1023_T1,T22,T3	chr15	4.9E+07	4.9E+07	3999	+	**3**	**37.5**	NA	intron (NM_001081391, intron 2 of 70)	intron (NM_001081391, intron 2 of 70)	158489	NM_001081391	239420	Mm.309363	NM_001081391	ENSMUSG00000022311	**Csmd3**	4930500N14Rik|mKIAA1894	CUB and Sushi multiple domains 3	Amp	protein-coding
1012_T22,T32,T42	chr15	4.8E+07	4.8E+07	6999	+	**3**	**37.5**	NA	intron (NM_001081391, intron 32 of 70)	intron (NM_001081391, intron 32 of 70)	1022989	NM_001081391	239420	Mm.309363	NM_001081391	ENSMUSG00000022311	**Csmd3**	4930500N14Rik|mKIAA1894	CUB and Sushi multiple domains 3	Amp	protein-coding
1622_T2,T32,T3	chr2	1.5E+08	1.5E+08	999	+	**3**	**37.5**	NA	Intergenic	L1_Rod|LINE|L1	-217488	NM_009976	13010	Mm.4263	NM_009976	ENSMUSG00000027447	**Cst3**	CysC	cystatin C	Amp	protein-coding
790_T22,T2,T42	chr14	9.8E+07	9.8E+07	999	+	**3**	**37.5**	NA	Intergenic	Intergenic	-39235	NM_007826	13134	Mm.320593	NM_007826	ENSMUSG00000055639	**Dach1**	Dac|Dach	dachshund 1 (Drosophila)	Amp	protein-coding
2240_T12,T2,T4	chr3	5.6E+07	5.6E+07	999	+	**3**	**37.5**	NA	Intergenic	L1Md_T|LINE|L1	145242	NM_001195540	13175	Mm.393242	NM_019978	ENSMUSG00000027797	**Dclk1**	1700113D08Rik|2810480F11Rik|AI836758|Click-I|Cpg16|Dcamkl1|Dcl|Dclk|mKIAA0369	doublecortin-like kinase 1	Amp	protein-coding
429_T2,T3,T4	chr12	4.3E+07	4.3E+07	6999	+	**3**	**37.5**	NA	Intergenic	RLTR14-int|LTR|ERV1	1106068	NM_013760	27362	Mm.27432	NM_013760	ENSMUSG00000014905	**Dnajb9**	AA408011|AA673251|AA673481|AW556981|ERdj4|Mdg1|mDj7	DnaJ heat shock protein family (Hsp40) member B9	Amp	protein-coding
149_T2,T32,T3	chr1	1.2E+08	1.2E+08	4999	+	**3**	**37.5**	NA	intron (NM_199021, intron 2 of 25)	B4A|SINE|B4	152559	NM_199021	269109	Mm.417716	NM_199021	ENSMUSG00000036815	**Dpp10**	6430601K09Rik|DPP X|Dprp3	dipeptidylpeptidase 10	Amp	protein-coding
2475_T2,T32,T3	chr3	1.2E+08	1.2E+08	999	+	**3**	**37.5**	NA	intron (NM_170778, intron 5 of 22)	intron (NM_170778, intron 5 of 22)	219822	NM_170778	99586	Mm.27907	NM_170778	ENSMUSG00000033308	**Dpyd**	AI315208|DPD|E330028L06Rik	dihydropyrimidine dehydrogenase	Amp	protein-coding
2477_T2,T32,T3	chr3	1.2E+08	1.2E+08	999	+	**3**	**37.5**	NA	intron (NM_170778, intron 5 of 22)	L1_Mur1|LINE|L1	224822	NM_170778	99586	Mm.27907	NM_170778	ENSMUSG00000033308	**Dpyd**	AI315208|DPD|E330028L06Rik	dihydropyrimidine dehydrogenase	Amp	protein-coding
1369_T22,T32,T42	chr19	5.4E+07	5.4E+07	3999	+	**3**	**37.5**	NA	Intergenic	Intergenic	-4818	NM_001085390	240672	Mm.482424	NM_001085390	ENSMUSG00000034765	**Dusp5**	Gm337	dual specificity phosphatase 5	Amp	protein-coding
3419_T2,T32,T42	chr8	7913501	7914500	999	+	**3**	**37.5**	NA	Intergenic	Intergenic	746773	NM_010111	13642	Mm.209813	NM_010111	ENSMUSG00000001300	**Efnb2**	ELF-2|Epl5|Eplg5|Htk-L|LERK-5|Lerk5|NLERK-1	ephrin B2	Amp	protein-coding

1113_T12,T2,T32	chr15	6.5E+07	6.5E+07	999	+	**3**	**37.5**	NA	Intergenic	Lx4B|LINE|L1	-365041	NM_133766	76740	Mm.260647	NM_133766	ENSMUSG00000015002	**Efr3a**	A130089M23Rik|BB071175|C76891|C920006C10Rik|D030063F01Rik|mKIAA0143	EFR3 homolog A	Amp	protein-coding
2691_T3,T42,T4	chr4	1.4E+08	1.4E+08	1230999	+	**3**	**37.5**	NA	intron (NM_172703, intron 2 of 34)	(CA)n|Simple_repeat|Simple_repeat	9544	NM_172703	230861	Mm.268903	NM_172703	ENSMUSG00000028760	**Eif4g3**	1500002J22Rik|4930523M17Rik|G1-419-52|eIF4GII|repro8	eukaryotic translation initiation factor 4 gamma, 3	Amp	protein-coding
48_T1,T2,T4	chr1	6.9E+07	6.9E+07	999	+	**3**	**37.5**	NA	intron (NM_010154, intron 2 of 26)	intron (NM_010154, intron 2 of 26)	420059	NM_010154	13869	Mm.442420	NM_010154	ENSMUSG00000062209	**Erbb4**	Her4|c-erbB-4	erb-b2 receptor tyrosine kinase 4	Amp	protein-coding
2662_T1,T2,T32	chr4	1.1E+08	1.1E+08	999	+	**3**	**37.5**	NA	intron (NM_007983, intron 6 of 18)	intron (NM_007983, intron 6 of 18)	66373	NM_007983	14084	Mm.318259	NM_007983	ENSMUSG00000010517	**Faf1**	AA408698|Dffrx|Fam	Fas-associated factor 1	Amp	protein-coding
1154_T2,T42,T4	chr15	7.1E+07	7.1E+07	3999	+	**3**	**37.5**	NA	Intergenic	ORR1D2|LTR|ERVL-MaLR	665338	NM_177819	70363	Mm.126450	NM_177819	ENSMUSG00000036800	**Fam135b**	1700010C24Rik|A830008O07Rik	family with sequence similarity 135, member B	Amp	protein-coding
1156_T22,T2,T4	chr15	7.2E+07	7.2E+07	5999	+	**3**	**37.5**	NA	intron (NM_177819, intron 2 of 19)	intron (NM_177819, intron 2 of 19)	133338	NM_177819	70363	Mm.126450	NM_177819	ENSMUSG00000036800	**Fam135b**	1700010C24Rik|A830008O07Rik	family with sequence similarity 135, member B	Amp	protein-coding
3770_T12,T22,T32	chr9	8.6E+07	8.6E+07	3999	+	**3**	**37.5**	NA	Intergenic	Intergenic	-196350	NM_001160379	212943	Mm.74610	NM_001160378	ENSMUSG00000032265	**Fam46a**	BAP014|D930050G01Rik	family with sequence similarity 46, member A	Amp	protein-coding
864_T12,T3,T4	chr14	1.2E+08	1.2E+08	999	+	**3**	**37.5**	NA	intron (NM_207667, intron 1 of 4)	MLT1A0|LTR|ERVL-MaLR	-105454	NM_010201	14169	Mm.206362	NM_010201	ENSMUSG00000025551	**Fgf14**	FHF-4|Fhf4|mFHF-4(1B)	fibroblast growth factor 14	Amp	protein-coding
2443_T22,T2,T3	chr3	9.3E+07	9.3E+07	999	+	**3**	**37.5**	NA	Intergenic	MLT1D|LTR|ERVL-MaLR	-18273	NM_001013804	229574	Mm.10755	NM_001013804		**Flg2**	EG229574	filaggrin family member 2	Amp	protein-coding
1610_T22,T3,T42	chr2	1.4E+08	1.4E+08	1999	+	**3**	**37.5**	NA	intron (NM_001013802, intron 5 of 18)	intron (NM_001013802, intron 5 of 18)	-750024	NM_178382	71436	Mm.426781	NM_178382	ENSMUSG00000051379	**Flrt3**	5530600M07Rik|C430047I10Rik|mKIAA1469	fibronectin leucine rich transmembrane protein 3	Amp	protein-coding
1343_T12,T22,T42	chr19	2.5E+07	2.5E+07	999	+	**3**	**37.5**	NA	Intergenic	Intergenic	-16691	NM_008022	14237	Mm.377080	NM_008022	ENSMUSG00000051490	**Foxd4**	FREAC5|Fkh2	forkhead box D4	Amp	protein-coding
2198_T2,T3,T42	chr3	5.2E+07	5.2E+07	3999	+	**3**	**37.5**	NA	Intergenic	LTR28B|LTR|ERV1	-16837	NM_019739	56458	Mm.29891	NM_019739	ENSMUSG00000044167	**Foxo1**	AI876417|Afxh|FKHR|Fkhr1|Foxo1a	forkhead box O1	Amp	protein-coding

236_T1,T22,T2	chr10	3.5E+07	3.5E+07	3999	+	**3**	**37.5**	NA	intron (NM_010237, intron 2 of 8)	intron (NM_010237, intron 2 of 8)	24100	NM_010237	14302	Mm.332432	NM_010237	ENSMUSG00000019779	**Frk**	BSK|BSK/IYK|C85044|GTK|RAK	fyn-related kinase	Amp	protein-coding
592_T12,T32,T42	chr13	1.1E+08	1.1E+08	999	+	**3**	**37.5**	NA	Intergenic	ID_B1|SINE|B4	-47049	NM_008046	14313	Mm.4913	NM_008046	ENSMUSG00000021765	**Fst**	AL033346|FS	follistatin	Amp	protein-coding
2405_T3,T42,T4	chr3	7.7E+07	7.7E+07	3999	+	**3**	**37.5**	NA	Intergenic	Intergenic	866917	NM_001253719	213262	Mm.379337	NM_178673	ENSMUSG00000034098	**Fstl5**	9130207J01Rik	follistatin-like 5	Amp	protein-coding
2417_T1,T22,T32	chr3	7.8E+07	7.8E+07	999	+	**3**	**37.5**	NA	Intergenic	Intergenic	1438417	NM_001253719	213262	Mm.379337	NM_178673	ENSMUSG00000034098	**Fstl5**	9130207J01Rik	follistatin-like 5	Amp	protein-coding
871_T22,T42,T4	chr15	3574501	3575500	999	+	**3**	**37.5**	NA	intron (NM_010284, intron 1 of 10)	MLT1A|LTR|ERVL-MaLR	6842	NM_001286370	14600	Mm.3986	NM_010284	ENSMUSG00000055737	**Ghr**	GHBP|GHR/BP	growth hormone receptor	Amp	protein-coding
315_T12,T1,T32	chr10	7.5E+07	7.5E+07	999	+	**3**	**37.5**	NA	Intergenic	Intergenic	-31231	NM_010311	14687	Mm.32595	NM_010311	ENSMUSG00000040009	**Gnaz**	AI847979|Gz	guanine nucleotide binding protein, alpha z subunit	Amp	protein-coding
3066_T22,T2,T3	chr6	6.4E+07	6.4E+07	999	+	**3**	**37.5**	NA	intron (NM_008167, intron 2 of 15)	intron (NM_008167, intron 2 of 15)	249143	NM_008167	14804	Mm.439651	NM_008167	ENSMUSG00000071424	**Grid2**	B230104L07Rik|GluD2|GluRdelta2|Lc|Lc<J>|MMS10-AC|Ms10ac|cpr|ho|nmf408|tpr	glutamate receptor, ionotropic, delta 2	Amp	protein-coding
3069_T1,T2,T32	chr6	6.4E+07	6.4E+07	999	+	**3**	**37.5**	NA	intron (NM_008167, intron 2 of 15)	intron (NM_008167, intron 2 of 15)	345143	NM_008167	14804	Mm.439651	NM_008167	ENSMUSG00000071424	**Grid2**	B230104L07Rik|GluD2|GluRdelta2|Lc|Lc<J>|MMS10-AC|Ms10ac|cpr|ho|nmf408|tpr	glutamate receptor, ionotropic, delta 2	Amp	protein-coding
277_T12,T2,T4	chr10	5E+07	5E+07	4999	+	**3**	**37.5**	NA	intron (NM_010349, intron 2 of 17)	intron (NM_010349, intron 2 of 17)	67754	NM_010349	14806	Mm.332838	NM_010349	ENSMUSG00000056073	**Grik2**	AW124492|GluK2|Glur-6|Glur6|Glurbeta2	glutamate receptor, ionotropic, kainate 2 (beta 2)	Amp	protein-coding
2826_T22,T2,T3	chr5	6.8E+07	6.8E+07	3999	+	**3**	**37.5**	NA	Intergenic	Intergenic	214665	NM_001018019	433899	Mm.332422	NM_001018019	ENSMUSG00000068082	**Grxcr1**	Tg(Eno2-Gabrb3)0370Brll|pi|tde	glutaredoxin, cysteine rich 1	Amp	protein-coding
1073_T12,T1,T4	chr15	5.7E+07	5.7E+07	999	+	**3**	**37.5**	NA	Intergenic	Intergenic	-285454	NM_008216	15117	Mm.5148	NM_008216	ENSMUSG00000022367	**Has2**	-	hyaluronan synthase 2	Amp	protein-coding
1074_T22,T32,T42	chr15	5.7E+07	5.7E+07	999	+	**3**	**37.5**	NA	Intergenic	Intergenic	-376454	NM_008216	15117	Mm.5148	NM_008216	ENSMUSG00000022367	**Has2**	-	hyaluronan synthase 2	Amp	protein-coding
1638_T12,T32,T4	chr3	3885501	3890500	4999	+	**3**	**37.5**	NA	Intergenic	L1Md_F2|LINE|L1	379970	NM_013920	30942	Mm.330897	NM_013920	ENSMUSG00000017688	**Hnf4g**	NR2A2	hepatocyte nuclear factor 4, gamma	Amp	protein-coding
2736_T32,T42,T4	chr5	4E+07	4E+07	1999	+	**3**	**37.5**	NA	Intergenic	L1_Mus3|LINE|L1	-740869	NM_010474	15476	Mm.12559	NM_010474	ENSMUSG00000051022	**Hs3st1**	3-Ost|D5Wsu110e|Hsg3ost	heparan sulfate (glucosamine) 3-O-sulfotransferase 1	Amp	protein-coding

2739_T1,T2,T3	chr5	4.1E+07	4.1E+07	999	+	**3**	**37.5**	NA	Intergenic	Intergenic	-983369	NM_010474	15476	Mm.12559	NM_010474	ENSMUSG00000051022	**Hs3st1**	3-Ost|D5Wsu110e|Hsg3ost	heparan sulfate (glucosamine) 3-O-sulfotransferase 1	Amp	protein-coding
3599_T1,T2,T4	chr8	8.2E+07	8.2E+07	1999	+	**3**	**37.5**	NA	Intergenic	L1_Mus4|LINE|L1	241086	NM_001254747	16168	Mm.4392	NM_008357	ENSMUSG00000031712	**Il15**	AI503618|IL-15	interleukin 15	Amp	protein-coding
498_T22,T32,T42	chr13	1.6E+07	1.6E+07	3999	+	**3**	**37.5**	NA	Intergenic	Intergenic	448025	NM_008380	16323	Mm.8042	NM_008380	ENSMUSG00000041324	**Inhba**	-	inhibin beta-A	Amp	protein-coding
1493_T22,T2,T3	chr2	5.5E+07	5.5E+07	999	+	**3**	**37.5**	NA	Intergenic	Intergenic	-314970	NM_001304810	16519	Mm.5127	NM_008426	ENSMUSG00000026824	**Kcnj3**	GIRK-1|GIRK1|Kcnf3|Kir3.1	potassium inwardly-rectifying channel, subfamily J, member 3	Amp	protein-coding
1397_T22,T2,T3	chr19	5.9E+07	5.9E+07	999	+	**3**	**37.5**	NA	intron (NM_207261, intron 1 of 2)	intron (NM_207261, intron 1 of 2)	1352	NM_207261	332396	Mm.329947	NM_207261	ENSMUSG00000040901	**Kcnk18**	Gm781|Tresk|Tresk-2|Trik	potassium channel, subfamily K, member 18	Amp	protein-coding
3391_T1,T3,T42	chr7	1.4E+08	1.4E+08	999	+	**3**	**37.5**	NA	non-coding (NR_001461, exon 1 of 1)	non-coding (NR_001461, exon 1 of 1)	57547	NR_001461	63830	Mm.356096	NR_001461	ENSMUSG00000101609	**Kcnq1ot1**	Kvlqt1-as|Lit1|Tssc8	KCNQ1 overlapping transcript 1	Amp	ncRNA
987_T1,T42,T4	chr15	4.5E+07	4.5E+07	4999	+	**3**	**37.5**	NA	Intergenic	L1Md_F2|LINE|L1	-143066	NM_026200	67498	Mm.300079	NM_026200	ENSMUSG00000022342	**Kcnv1**	2700023A03Rik|vibe	potassium channel, subfamily V, member 1	Amp	protein-coding
993_T22,T3,T4	chr15	4.6E+07	4.6E+07	3999	+	**3**	**37.5**	NA	Intergenic	Intergenic	-408566	NM_026200	67498	Mm.300079	NM_026200	ENSMUSG00000022342	**Kcnv1**	2700023A03Rik|vibe	potassium channel, subfamily V, member 1	Amp	protein-coding
402_T12,T3,T42	chr11	9.2E+07	9.2E+07	999	+	**3**	**37.5**	NA	Intergenic	Lx|LINE|L1	-403445	NM_028547	73470	Mm.67677	NM_028547	ENSMUSG00000046755	**Kif2b**	1700063D03Rik	kinesin family member 2B	Amp	protein-coding
773_T12,T1,T32	chr14	9.7E+07	9.7E+07	3999	+	**3**	**37.5**	NA	Intergenic	Lx10|LINE|L1	-56466	NM_053105	93688	Mm.308735	NM_053105	ENSMUSG00000022076	**Klhl1**	mKIAA1490	kelch-like 1	Amp	protein-coding
1174_T32,T3,T4	chr16	7.5E+07	7.5E+07	3999	+	**3**	**37.5**	NA	Intergenic	Intergenic	439561	NM_001252513	320355	Mm.99517	NM_177142	ENSMUSG00000032948	**Lipi**	D930038D03Rik|lpd1	lipase, member I	Amp	protein-coding
398_T1,T2,T42	chr11	6.1E+07	6.1E+07	1999	+	**3**	**37.5**	NA	Intergenic	MT2_Mm|LTR|ERVL	-48190	NM_001159405	16897	Mm.285453	NM_008502	ENSMUSG00000020536	**Llgl1**	AI325176|Lgl1|Llglh|Mgl1	lethal giant larvae homolog 1 (Drosophila)	Amp	protein-coding
1563_T12,T3,T4	chr2	9.8E+07	9.8E+07	5999	+	**3**	**37.5**	NA	Intergenic	Intergenic	652843	NM_178725	241568	Mm.241682	NM_178725	ENSMUSG00000050587	**Lrrc4c**	6430556C10Rik|NGL-1	leucine rich repeat containing 4C	Amp	protein-coding
1567_T22,T2,T42	chr2	9.9E+07	9.9E+07	1999	+	**3**	**37.5**	NA	Intergenic	Intergenic	1248843	NM_178725	241568	Mm.241682	NM_178725	ENSMUSG00000050587	**Lrrc4c**	6430556C10Rik|NGL-1	leucine rich repeat containing 4C	Amp	protein-coding
1547_T22,T2,T32	chr2	9.7E+07	9.7E+07	2999	+	**3**	**37.5**	NA	intron (NM_001289742, intron 5 of 7)	intron (NM_001289742, intron 5 of 7)	-129657	NM_001289744	241568	Mm.241682	NM_178725	ENSMUSG00000050587	**Lrrc4c**	6430556C10Rik|NGL-1	leucine rich repeat containing 4C	Amp	protein-coding

1554_T12,T22,T3	chr2	9.8E+07	9.8E+07	999	+	**3**	**37.5**	NA	intron (NM_001289742, intron 7 of 7)	intron (NM_001289742, intron 7 of 7)	139343	NM_178725	241568	Mm.241682	NM_178725	ENSMUSG00000050587	**Lrrc4c**	6430556C10Rik|NGL-1	leucine rich repeat containing 4C	Amp	protein-coding
1549_T12,T2,T42	chr2	9.8E+07	9.8E+07	999	+	**3**	**37.5**	NA	intron (NM_001289744, intron 1 of 1)	intron (NM_001289744, intron 1 of 1)	43343	NM_178725	241568	Mm.241682	NM_178725	ENSMUSG00000050587	**Lrrc4c**	6430556C10Rik|NGL-1	leucine rich repeat containing 4C	Amp	protein-coding
1551_T12,T2,T4	chr2	9.8E+07	9.8E+07	999	+	**3**	**37.5**	NA	intron (NM_001289744, intron 1 of 1)	intron (NM_001289744, intron 1 of 1)	48343	NM_178725	241568	Mm.241682	NM_178725	ENSMUSG00000050587	**Lrrc4c**	6430556C10Rik|NGL-1	leucine rich repeat containing 4C	Amp	protein-coding
2731_T2,T32,T42	chr5	2E+07	2E+07	2999	+	**3**	**37.5**	NA	intron (NM_001170746, intron 1 of 22)	intron (NM_001170746, intron 1 of 22)	-294518	NM_001170745	50791	Mm.332231	NM_015823	ENSMUSG00000040003	**Magi2**	AIP-1|Acvri1|Acvrinp1|Acvrip1|Magi-2|S-SCAM|mKIAA0705	membrane associated guanylate kinase, WW and PDZ domain containing 2	Amp	protein-coding
2494_T1,T2,T4	chr3	1.4E+08	1.4E+08	999	+	**3**	**37.5**	NA	Intergenic	Intergenic	-16611	NM_027288	110173	Mm.280536	NM_027288	ENSMUSG00000028164	**Manba**	2410030O07Rik|B930014J03Rik|Bmn	mannosidase, beta A, lysosomal	Amp	protein-coding
328_T1,T2,T42	chr10	1E+08	1E+08	17999	+	**3**	**37.5**	NA	Intergenic	Lx10|LINE|L1	-528987	NM_001205098	67569	Mm.25252	NM_026243	ENSMUSG00000019888	**Mgat4c**	9130411I17Rik|GntIVh	MGAT4 family, member C	Amp	protein-coding
3463_T1,T22,T2	chr8	3.8E+07	3.8E+07	999	+	**3**	**37.5**	NA	intron (NM_145841, intron 1 of 7)	intron (NM_145841, intron 1 of 7)	245202	NR_029884	723860		NR_029884	ENSMUSG00000065457	**Mir383**	Mirn383|mir-383|mmu-mir-383	microRNA 383	Amp	ncRNA
3469_T2,T32,T4	chr8	3.8E+07	3.8E+07	3999	+	**3**	**37.5**	NA	intron (NM_145841, intron 1 of 7)	intron (NM_145841, intron 1 of 7)	-13298	NR_029884	723860		NR_029884	ENSMUSG00000065457	**Mir383**	Mirn383|mir-383|mmu-mir-383	microRNA 383	Amp	ncRNA
2532_T2,T42,T4	chr4	1.8E+07	1.8E+07	999	+	**3**	**37.5**	NA	Intergenic	Intergenic	-215482	NM_019724	17389	Mm.187315	NM_019724	ENSMUSG00000028226	**Mmp16**	MT-MMP 3|MT3-MMP|Mt3mmp	matrix metallopeptidase 16	Amp	protein-coding
2539_T2,T32,T4	chr4	1.8E+07	1.8E+07	4999	+	**3**	**37.5**	NA	Intergenic	L1_Mur1|LINE|L1	447518	NM_019724	17389	Mm.187315	NM_019724	ENSMUSG00000028226	**Mmp16**	MT-MMP 3|MT3-MMP|Mt3mmp	matrix metallopeptidase 16	Amp	protein-coding
2540_T2,T32,T3	chr4	1.8E+07	1.8E+07	999	+	**3**	**37.5**	NA	Intergenic	MLT1D|LTR|ERVL-MaLR	470518	NM_019724	17389	Mm.187315	NM_019724	ENSMUSG00000028226	**Mmp16**	MT-MMP 3|MT3-MMP|Mt3mmp	matrix metallopeptidase 16	Amp	protein-coding
2542_T2,T3,T4	chr4	1.8E+07	1.8E+07	999	+	**3**	**37.5**	NA	Intergenic	MLT1J|LTR|ERVL-MaLR	474518	NM_019724	17389	Mm.187315	NM_019724	ENSMUSG00000028226	**Mmp16**	MT-MMP 3|MT3-MMP|Mt3mmp	matrix metallopeptidase 16	Amp	protein-coding
2537_T22,T2,T32	chr4	1.8E+07	1.8E+07	3999	+	**3**	**37.5**	NA	intron (NM_019724, intron 4 of 9)	intron (NM_019724, intron 4 of 9)	163018	NM_019724	17389	Mm.187315	NM_019724	ENSMUSG00000028226	**Mmp16**	MT-MMP 3|MT3-MMP|Mt3mmp	matrix metallopeptidase 16	Amp	protein-coding

2551_T22,T32,T4	chr4	2.4E+07	2.4E+07	3999	+	**3**	**37.5**	NA	Intergenic	Intergenic	-273962	NM_199467	212377	Mm.120919	NM_199467	ENSMUSG00000045751	**Mms22l**	F730047E07Rik|Gm134	MMS22-like, DNA repair protein	Amp	protein-coding
2267_T2,T42,T4	chr3	5.6E+07	5.6E+07	2999	+	**3**	**37.5**	NA	Intergenic	L1Md_A|LINE|L1	-188299	NM_030595	26422	Mm.384353	NM_030595	ENSMUSG00000027799	**Nbea**	Lyst2|mKIAA1544	neurobeachin	Amp	protein-coding
2277_T1,T2,T42	chr3	5.7E+07	5.7E+07	999	+	**3**	**37.5**	NA	Intergenic	Intergenic	-329299	NM_030595	26422	Mm.384353	NM_030595	ENSMUSG00000027799	**Nbea**	Lyst2|mKIAA1544	neurobeachin	Amp	protein-coding
1725_T22,T32,T4	chr3	2.5E+07	2.5E+07	999	+	**3**	**37.5**	NA	Intergenic	Intergenic	1629307	NM_001163387	192167	Mm.316080	NM_138666	ENSMUSG00000063887	**Nlgn1**	6330415N05Rik|BB179718|NL1|Nlg1|mKIAA1070	neuroligin 1	Amp	protein-coding
1732_T22,T3,T4	chr3	2.5E+07	2.5E+07	3999	+	**3**	**37.5**	NA	intron (NM_138666, intron 5 of 7)	intron (NM_138666, intron 5 of 7)	694807	NM_001163387	192167	Mm.316080	NM_138666	ENSMUSG00000063887	**Nlgn1**	6330415N05Rik|BB179718|NL1|Nlg1|mKIAA1070	neuroligin 1	Amp	protein-coding
1734_T1,T22,T4	chr3	2.6E+07	2.6E+07	999	+	**3**	**37.5**	NA	intron (NM_138666, intron 5 of 7)	intron (NM_138666, intron 5 of 7)	637307	NM_001163387	192167	Mm.316080	NM_138666	ENSMUSG00000063887	**Nlgn1**	6330415N05Rik|BB179718|NL1|Nlg1|mKIAA1070	neuroligin 1	Amp	protein-coding
434_T12,T2,T32	chr12	4.6E+07	4.6E+07	3999	+	**3**	**37.5**	NA	Intergenic	Intergenic	710275	NM_021361	664883	Mm.247195	NM_021361	ENSMUSG00000021047	**Nova1**	9430099M15Rik|G630039L02|Nova-1	neuro-oncological ventral antigen 1	Amp	protein-coding
456_T32,T3,T42	chr12	8.9E+07	8.9E+07	3999	+	**3**	**37.5**	NA	intron (NM_172544, intron 2 of 19)	MER103C|DNA|hAT-Charlie	102110	NM_172544	18191	Mm.425766	NM_172544	ENSMUSG00000066392	**Nrxn3**	-	neurexin III	Amp	protein-coding
460_T12,T1,T3	chr12	8.9E+07	8.9E+07	999	+	**3**	**37.5**	NA	intron (NM_172544, intron 8 of 19)	MIRb|SINE|MIR	-404483	NM_001252074	18191	Mm.425766	NM_172544	ENSMUSG00000066392	**Nrxn3**	-	neurexin III	Amp	protein-coding
413_T12,T32,T3	chr12	1E+07	1E+07	3999	+	**3**	**37.5**	NA	Intergenic	Intergenic	-268471	NM_027588	70881	Mm.272843	NM_027588	ENSMUSG00000020622	**Nt5c1b**	4921514H13Rik|AIRP|CN-IB|cN1B	5'-nucleotidase, cytosolic IB	Amp	protein-coding
1281_T12,T22,T2	chr17	6E+07	6E+07	999	+	**3**	**37.5**	NA	Intergenic	Intergenic	-630678	NM_026497	67993	Mm.36507	NM_026497	ENSMUSG00000024228	**Nudt12**	0610016O18Rik	nudix (nucleoside diphosphate linked moiety X)-type motif 12	Amp	protein-coding
1283_T12,T22,T32	chr17	6E+07	6E+07	999	+	**3**	**37.5**	NA	Intergenic	L1Md_F3|LINE|L1	-1E+06	NM_026497	67993	Mm.36507	NM_026497	ENSMUSG00000024228	**Nudt12**	0610016O18Rik	nudix (nucleoside diphosphate linked moiety X)-type motif 12	Amp	protein-coding
1286_T12,T3,T4	chr17	6E+07	6E+07	3999	+	**3**	**37.5**	NA	Intergenic	Intergenic	-1E+06	NM_026497	67993	Mm.36507	NM_026497	ENSMUSG00000024228	**Nudt12**	0610016O18Rik	nudix (nucleoside diphosphate linked moiety X)-type motif 12	Amp	protein-coding
2886_T1,T42,T4	chr6	9419501	9420500	999	+	**3**	**37.5**	NA	Intergenic	Lx6|LINE|L1	469981	NM_008751	18231	Mm.46954	NM_008751	ENSMUSG00000046178	**Nxph1**	C130005L03Rik	neurexophilin 1	Amp	protein-coding

51_T1,T22,T2	chr1	8.1E+07	8.1E+07	2999	+	**3**	**37.5**	NA	Intergenic	Intergenic	-118317	NM_172849	241134	Mm.313904	NM_172849	ENSMUSG00000054976	**Nyap2**	9430031J16Rik|Jr6|Kiaa1486	neuronal tyrosine-phophorylated phosphoinositide 3-kinase adaptor 2	Amp	protein-coding
638_T2,T3,T4	chr14	8.2E+07	8.2E+07	3999	+	**3**	**37.5**	NA	Intergenic	Intergenic	1833198	NM_001030294	380924	Mm.26456	NM_001030294	ENSMUSG00000022026	**Olfm4**	GC1|GW112|Gm296|Gm913|OlfD|pPD4	olfactomedin 4	Amp	protein-coding
1907_T1,T42,T4	chr3	4.6E+07	4.6E+07	999	+	**3**	**37.5**	NA	Intergenic	Lx5c|LINE|L1	447941	NM_001101479	241989	Mm.119234	NM_001101479	ENSMUSG00000090919	**Pabpc4l**	C330050A14Rik|EG241989	poly(A) binding protein, cytoplasmic 4-like	Amp	protein-coding
1931_T1,T22,T42	chr3	4.6E+07	4.6E+07	8999	+	**3**	**37.5**	NA	Intergenic	Lx|LINE|L1	302941	NM_001101479	241989	Mm.119234	NM_001101479	ENSMUSG00000090919	**Pabpc4l**	C330050A14Rik|EG241989	poly(A) binding protein, cytoplasmic 4-like	Amp	protein-coding
1933_T1,T22,T32	chr3	4.6E+07	4.6E+07	999	+	**3**	**37.5**	NA	Intergenic	Intergenic	264941	NM_001101479	241989	Mm.119234	NM_001101479	ENSMUSG00000090919	**Pabpc4l**	C330050A14Rik|EG241989	poly(A) binding protein, cytoplasmic 4-like	Amp	protein-coding
1947_T12,T3,T4	chr3	4.7E+07	4.7E+07	999	+	**3**	**37.5**	NA	Intergenic	L1_Mus2|LINE|L1	-149059	NM_001101479	241989	Mm.119234	NM_001101479	ENSMUSG00000090919	**Pabpc4l**	C330050A14Rik|EG241989	poly(A) binding protein, cytoplasmic 4-like	Amp	protein-coding
1952_T12,T32,T3	chr3	4.7E+07	4.7E+07	1999	+	**3**	**37.5**	NA	Intergenic	Intergenic	-196559	NM_001101479	241989	Mm.119234	NM_001101479	ENSMUSG00000090919	**Pabpc4l**	C330050A14Rik|EG241989	poly(A) binding protein, cytoplasmic 4-like	Amp	protein-coding
655_T1,T3,T4	chr14	8.3E+07	8.3E+07	999	+	**3**	**37.5**	NA	Intergenic	Lx8|LINE|L1	-953563	NM_001013753	219228	Mm.153643	NM_001013753	ENSMUSG00000035566	**Pcdh17**	C030033F14Rik|Gm78	protocadherin 17	Amp	protein-coding
662_T2,T32,T42	chr14	8.4E+07	8.4E+07	999	+	**3**	**37.5**	NA	intron (NM_001013753, intron 1 of 3)	intron (NM_001013753, intron 1 of 3)	7437	NM_001013753	219228	Mm.153643	NM_001013753	ENSMUSG00000035566	**Pcdh17**	C030033F14Rik|Gm78	protocadherin 17	Amp	protein-coding
2120_T3,T42,T4	chr3	4.9E+07	4.9E+07	5999	+	**3**	**37.5**	NA	Intergenic	L1Md_T|LINE|L1	310816	NM_130448	73173	Mm.87246	NM_130448	ENSMUSG00000037892	**Pcdh18**	3110038E07Rik|BB095589|PCDH68L	protocadherin 18	Amp	protein-coding
2123_T3,T42,T4	chr3	4.9E+07	4.9E+07	7999	+	**3**	**37.5**	NA	Intergenic	L1Md_A|LINE|L1	295816	NM_130448	73173	Mm.87246	NM_130448	ENSMUSG00000037892	**Pcdh18**	3110038E07Rik|BB095589|PCDH68L	protocadherin 18	Amp	protein-coding
2125_T3,T42,T4	chr3	4.9E+07	4.9E+07	8999	+	**3**	**37.5**	NA	Intergenic	L1_Mus2|LINE|L1	278316	NM_130448	73173	Mm.87246	NM_130448	ENSMUSG00000037892	**Pcdh18**	3110038E07Rik|BB095589|PCDH68L	protocadherin 18	Amp	protein-coding
2128_T12,T32,T3	chr3	5E+07	5E+07	999	+	**3**	**37.5**	NA	Intergenic	Intergenic	83316	NM_130448	73173	Mm.87246	NM_130448	ENSMUSG00000037892	**Pcdh18**	3110038E07Rik|BB095589|PCDH68L	protocadherin 18	Amp	protein-coding
2131_T32,T3,T42	chr3	5E+07	5E+07	999	+	**3**	**37.5**	NA	Intergenic	Intergenic	77316	NM_130448	73173	Mm.87246	NM_130448	ENSMUSG00000037892	**Pcdh18**	3110038E07Rik|BB095589|PCDH68L	protocadherin 18	Amp	protein-coding
2141_T1,T32,T3	chr3	5E+07	5E+07	999	+	**3**	**37.5**	NA	Intergenic	L1Md_T|LINE|L1	-63684	NM_130448	73173	Mm.87246	NM_130448	ENSMUSG00000037892	**Pcdh18**	3110038E07Rik|BB095589|PCDH68L	protocadherin 18	Amp	protein-coding
2146_T22,T3,T42	chr3	5E+07	5E+07	6999	+	**3**	**37.5**	NA	Intergenic	L1Md_T|LINE|L1	-299684	NM_130448	73173	Mm.87246	NM_130448	ENSMUSG00000037892	**Pcdh18**	3110038E07Rik|BB095589|PCDH68L	protocadherin 18	Amp	protein-coding

686_T1,T32,T4	chr14	8.8E+07	8.8E+07	5999	+	**3**	**37.5**	NA	Intergenic	L1_Mus3|LINE|L1	406896	NM_178685	219257	Mm.128861	NM_178685	ENSMUSG00000050505	**Pcdh20**	C630015B17Rik|Pcdh13	protocadherin 20	Amp	protein-coding
691_T32,T42,T4	chr14	8.8E+07	8.8E+07	999	+	**3**	**37.5**	NA	Intergenic	Intergenic	103396	NM_178685	219257	Mm.128861	NM_178685	ENSMUSG00000050505	**Pcdh20**	C630015B17Rik|Pcdh13	protocadherin 20	Amp	protein-coding
2785_T12,T2,T4	chr5	5.8E+07	5.8E+07	3999	+	**3**	**37.5**	NA	intron (NM_001310608, intron 1 of 3)	intron (NM_001310608, intron 1 of 3)	176419	NM_018764	54216	Mm.332387	NM_018764	ENSMUSG00000029108	**Pcdh7**	-	protocadherin 7	Amp	protein-coding
719_T22,T2,T3	chr14	9.2E+07	9.2E+07	2999	+	**3**	**37.5**	NA	Intergenic	Intergenic	1779888	NM_001271800	211712	Mm.37126	NM_001081377	ENSMUSG00000055421	**Pcdh9**	-	protocadherin 9	Amp	protein-coding
722_T22,T2,T3	chr14	9.2E+07	9.2E+07	9999	+	**3**	**37.5**	NA	Intergenic	Intergenic	1727388	NM_001271800	211712	Mm.37126	NM_001081377	ENSMUSG00000055421	**Pcdh9**	-	protocadherin 9	Amp	protein-coding
737_T12,T32,T3	chr14	9.4E+07	9.4E+07	3999	+	**3**	**37.5**	NA	intron (NM_001081377, intron 1 of 3)	intron (NM_001081377, intron 1 of 3)	282388	NM_001271800	211712	Mm.37126	NM_001081377	ENSMUSG00000055421	**Pcdh9**	-	protocadherin 9	Amp	protein-coding
736_T12,T3,T42	chr14	9.3E+07	9.3E+07	4999	+	**3**	**37.5**	NA	intron (NM_001271798, intron 2 of 3)	intron (NM_001271798, intron 2 of 3)	466888	NM_001271800	211712	Mm.37126	NM_001081377	ENSMUSG00000055421	**Pcdh9**	-	protocadherin 9	Amp	protein-coding
731_T22,T3,T4	chr14	9.3E+07	9.3E+07	3999	+	**3**	**37.5**	NA	intron (NM_001271800, intron 2 of 2)	intron (NM_001271800, intron 2 of 2)	765388	NM_001271800	211712	Mm.37126	NM_001081377	ENSMUSG00000055421	**Pcdh9**	-	protocadherin 9	Amp	protein-coding
2507_T1,T2,T4	chr3	1.4E+08	1.4E+08	3999	+	**3**	**37.5**	NA	Intergenic	Intergenic	24855	NM_008811	18598	Mm.4223	NM_008811	ENSMUSG00000047674	**Pdha2**	Pdhal	pyruvate dehydrogenase E1 alpha 2	Amp	protein-coding
21_T2,T3,T4	chr1	3E+07	3E+07	5999	+	**3**	**37.5**	NA	Intergenic	Intergenic	418756	NM_001081080	213109	Mm.194486	NM_025871	ENSMUSG00000048874	**Phf3**	2310061N19Rik|mKIAA0244	PHD finger protein 3	Amp	protein-coding
982_T1,T32,T4	chr15	4.5E+07	4.5E+07	3999	+	**3**	**37.5**	NA	intron (NM_138674, intron 30 of 76)	B4|SINE|B4	64947	NM_138674	192190	Mm.211494	NM_138674	ENSMUSG00000038725	**Pkhd1l1**	AB055648|PKHDL1	polycystic kidney and hepatic disease 1-like 1	Amp	protein-coding
190_T1,T2,T3	chr1	1.5E+08	1.5E+08	3999	+	**3**	**37.5**	NA	Intergenic	Intergenic	1602939	NM_001305632	18783	Mm.4186	NM_008869	ENSMUSG00000056220	**Pla2g4a**	Pla2g4|cPLA2|cPLA2-alpha|cPLA2alpha	phospholipase A2, group IVA (cytosolic, calcium-dependent)	Amp	protein-coding
3342_T1,T32,T42	chr7	3.8E+07	3.8E+07	999	+	**3**	**37.5**	NA	Intergenic	L1Md_F2|LINE|L1	-81652	NM_025390	66161	Mm.22284	NM_025390	ENSMUSG00000030423	**Pop4**	1110023P21Rik|Rpp29	processing of precursor 4, ribonuclease P/MRP family, (S. cerevisiae)	Amp	protein-coding

<table>
<tr><td>1437_T22,T32,T4</td><td>chr2</td><td>4E+07</td><td>4E+07</td><td>2999</td><td>+</td><td>3</td><td>37.5</td><td>NA</td><td>Intergenic</td><td>L1_Mus3|LINE|L1</td><td>-1E+06</td><td>NM_024209</td><td>67857</td><td>Mm.389242</td><td>NM_024209</td><td>ENSMUSG00000026753</td><td>Ppp6c</td><td>2310003C10Rik</td><td>protein phosphatase 6, catalytic subunit</td><td>Amp</td><td>protein-coding</td></tr>
<tr><td>2454_T1,T22,T32</td><td>chr3</td><td>1.1E+08</td><td>1.1E+08</td><td>3999</td><td>+</td><td>3</td><td>37.5</td><td>NA</td><td>Intergenic</td><td>Intergenic</td><td>-319501</td><td>NR_024139</td><td>99890</td><td>Mm.36115</td><td>NM_178891</td><td>ENSMUSG00000049300</td><td>Prmt6</td><td>AW124876|BB233495|Hrmt1l6</td><td>protein arginine N-methyltransferase 6</td><td>Amp</td><td>protein-coding</td></tr>
<tr><td>2457_T1,T22,T2</td><td>chr3</td><td>1.1E+08</td><td>1.1E+08</td><td>6999</td><td>+</td><td>3</td><td>37.5</td><td>NA</td><td>Intergenic</td><td>Intergenic</td><td>-445001</td><td>NR_024139</td><td>99890</td><td>Mm.36115</td><td>NM_178891</td><td>ENSMUSG00000049300</td><td>Prmt6</td><td>AW124876|BB233495|Hrmt1l6</td><td>protein arginine N-methyltransferase 6</td><td>Amp</td><td>protein-coding</td></tr>
<tr><td>2623_T12,T32,T4</td><td>chr4</td><td>7.8E+07</td><td>7.8E+07</td><td>3999</td><td>+</td><td>3</td><td>37.5</td><td>NA</td><td>intron (NM_011211, intron 2 of 39)</td><td>RSINE1|SINE|B4</td><td>516395</td><td>NM_011211</td><td>19266</td><td>Mm.184021</td><td>NM_011211</td><td>ENSMUSG00000028399</td><td>Ptprd</td><td>1110002J03Rik|3000002J10Rik|B230219D21Rik|R-PTP-delta</td><td>protein tyrosine phosphatase, receptor type, D</td><td>Amp</td><td>protein-coding</td></tr>
<tr><td>1409_T2,T42,T4</td><td>chr19</td><td>6E+07</td><td>6E+07</td><td>999</td><td>+</td><td>3</td><td>37.5</td><td>NA</td><td>Intergenic</td><td>Intergenic</td><td>-81346</td><td>NM_001164367</td><td>74998</td><td>Mm.24167</td><td>NM_001033172</td><td>ENSMUSG00000040022</td><td>Rab11fip2</td><td>4930470G04Rik|A830046J09Rik|AW558126|Nrip11</td><td>RAB11 family interacting protein 2 (class I)</td><td>Amp</td><td>protein-coding</td></tr>
<tr><td>1577_T1,T32,T3</td><td>chr2</td><td>1E+08</td><td>1E+08</td><td>3999</td><td>+</td><td>3</td><td>37.5</td><td>NA</td><td>Intergenic</td><td>Intergenic</td><td>-990248</td><td>NM_009020</td><td>19374</td><td>Mm.4988</td><td>NM_009020</td><td>ENSMUSG00000032864</td><td>Rag2</td><td>Rag-2</td><td>recombination activating gene 2</td><td>Amp</td><td>protein-coding</td></tr>
<tr><td>1677_T1,T32,T3</td><td>chr3</td><td>1.3E+07</td><td>1.3E+07</td><td>999</td><td>+</td><td>3</td><td>37.5</td><td>NA</td><td>Intergenic</td><td>Intergenic</td><td>-373655</td><td>NM_178631</td><td>76897</td><td>Mm.422625</td><td>NM_178631</td><td>ENSMUSG00000039717</td><td>Ralyl</td><td>0710005M24Rik</td><td>RALY RNA binding protein-like</td><td>Amp</td><td>protein-coding</td></tr>
<tr><td>1188_T12,T2,T3</td><td>chr17</td><td>1.6E+07</td><td>1.6E+07</td><td>3999</td><td>+</td><td>3</td><td>37.5</td><td>NA</td><td>Intergenic</td><td>L1MA6|LINE|L1</td><td>-489914</td><td>NM_178615</td><td>68799</td><td>Mm.293466</td><td>NM_178615</td><td>ENSMUSG00000048027</td><td>Rgmb</td><td>1110059F19Rik|DRAGON</td><td>repulsive guidance molecule family member B</td><td>Amp</td><td>protein-coding</td></tr>
<tr><td>171_T12,T3,T4</td><td>chr1</td><td>1.5E+08</td><td>1.5E+08</td><td>999</td><td>+</td><td>3</td><td>37.5</td><td>NA</td><td>Intergenic</td><td>Intergenic</td><td>-553579</td><td>NM_022881</td><td>64214</td><td>Mm.253927</td><td>NM_022881</td><td>ENSMUSG00000026357</td><td>Rgs18</td><td>-</td><td>regulator of G-protein signaling 18</td><td>Amp</td><td>protein-coding</td></tr>
<tr><td>2546_T12,T22,T42</td><td>chr4</td><td>2E+07</td><td>2E+07</td><td>3999</td><td>+</td><td>3</td><td>37.5</td><td>NA</td><td>intron (NM_001276292, intron 23 of 23)</td><td>L1MA4A|LINE|L1</td><td>39434</td><td>NM_025476</td><td>66302</td><td>Mm.440686</td><td>NM_025476</td><td>ENSMUSG00000028229</td><td>Rmdn1</td><td>Fam82b|RMD-1</td><td>regulator of microtubule dynamics 1</td><td>Amp</td><td>protein-coding</td></tr>
<tr><td>222_T12,T1,T4</td><td>chr10</td><td>3.2E+07</td><td>3.2E+07</td><td>999</td><td>+</td><td>3</td><td>37.5</td><td>NA</td><td>intron (NM_001013411, intron 3 of 6)</td><td>L1_Mus3|LINE|L1</td><td>-351275</td><td>NM_001146349</td><td>268291</td><td>Mm.295212</td><td>NM_001146349</td><td>ENSMUSG00000063760</td><td>Rnf217</td><td>AU016819|Ibrdc1</td><td>ring finger protein 217</td><td>Amp</td><td>protein-coding</td></tr>
<tr><td>228_T12,T42,T4</td><td>chr10</td><td>3.2E+07</td><td>3.2E+07</td><td>4999</td><td>+</td><td>3</td><td>37.5</td><td>NA</td><td>intron (NM_001013411, intron 3 of 6)</td><td>intron (NM_001013411, intron 3 of 6)</td><td>-592275</td><td>NM_001146349</td><td>268291</td><td>Mm.295212</td><td>NM_001146349</td><td>ENSMUSG00000063760</td><td>Rnf217</td><td>AU016819|Ibrdc1</td><td>ring finger protein 217</td><td>Amp</td><td>protein-coding</td></tr>
<tr><td>967_T2,T3,T42</td><td>chr15</td><td>3.3E+07</td><td>3.3E+07</td><td>999</td><td>+</td><td>3</td><td>37.5</td><td>NA</td><td>Intergenic</td><td>Intergenic</td><td>-30723</td><td>NM_008304</td><td>15529</td><td>Mm.234266</td><td>NM_008304</td><td>ENSMUSG00000022261</td><td>Sdc2</td><td>4833414L08Rik|AA960457|Hspg1|Synd2|syndecan-2</td><td>syndecan 2</td><td>Amp</td><td>protein-coding</td></tr>
</table>

2712_T1,T2 2,T2	chr5	1.3E+07	1.3E+07	999	+	**3**	**37.5**	NA	Intergenic	L1Md_F2|LINE|L1	-291784	NM_001 243072	20346	Mm.372 039	NM_009 152	ENSMUS G000000 28883	**Sema3a**	Hsema-I|SEMA1|SemD|Semad|coll-1	sema domain, immunoglobulin domain (Ig), short basic domain, secreted, (semaphorin) 3A	Amp	protein-coding
3475_T22,T 2,T3	chr8	3.8E+07	3.8E+07	999	+	**3**	**37.5**	NA	intron (NM_1458 41, intron 1 of 7)	intron (NM_1458 41, intron 1 of 7)	185508	NM_145 841	244431	Mm.210 388	NM_145 841	ENSMUS G000000 39539	**Sgcz**	C230085N17Rik	sarcoglycan zeta	Amp	protein-coding
3412_T32,T 3,T4	chr8	6531501	6532500	999	+	**3**	**37.5**	NA	Intergenic	MTD|LTR|ERVL-MaLR	-1E+06	NM_011 388	20494	Mm.350 0	NM_011 388	ENSMUS G000000 23073	**Slc10a2**	9130221J18Rik|AI605518|ASBT|IBAT|ISBT	solute carrier family 10, member 2	Amp	protein-coding
2156_T22,T 3,T4	chr3	5E+07	5E+07	7999	+	**3**	**37.5**	NA	Intergenic	L1Md_A|LINE|L1	-24887	NM_011 990	26570	Mm.260 988	NM_011 990	ENSMUS G000000 27737	**Slc7a11**	9930009M05Rik|AI451155|sut|xCT	solute carrier family 7 (cationic amino acid transporter, y+ system), member 11	Amp	protein-coding
2163_T32,T 3,T4	chr3	5.1E+07	5.1E+07	999	+	**3**	**37.5**	NA	Intergenic	L1Md_F2|LINE|L1	-230387	NM_011 990	26570	Mm.260 988	NM_011 990	ENSMUS G000000 27737	**Slc7a11**	9930009M05Rik|AI451155|sut|xCT	solute carrier family 7 (cationic amino acid transporter, y+ system), member 11	Amp	protein-coding
2165_T22,T 3,T4	chr3	5.1E+07	5.1E+07	999	+	**3**	**37.5**	NA	Intergenic	ID4|SINE|ID	-252387	NM_011 990	26570	Mm.260 988	NM_011 990	ENSMUS G000000 27737	**Slc7a11**	9930009M05Rik|AI451155|sut|xCT	solute carrier family 7 (cationic amino acid transporter, y+ system), member 11	Amp	protein-coding
811_T12,T2 2,T2	chr14	1.1E+08	1.1E+08	999	+	**3**	**37.5**	NA	Intergenic	Intergenic	99239	NM_199 065	76965	Mm.257 268	NM_199 065	ENSMUS G000000 75478	**Slitrk1**	3200001I04Rik	SLIT and NTRK-like family, member 1	Amp	protein-coding
2375_T12,T 22,T2	chr3	7.3E+07	7.3E+07	999	+	**3**	**37.5**	NA	Intergenic	Intergenic	24943	NM_198 864	386750	Mm.331 076	NM_198 864	ENSMUS G000000 48304	**Slitrk3**	-	SLIT and NTRK-like family, member 3	Amp	protein-coding
843_T2,T32, T42	chr14	1.1E+08	1.1E+08	999	+	**3**	**37.5**	NA	Intergenic	Intergenic	139149	NM_175 499	239250	Mm.497 28	NM_175 499	ENSMUS G000000 45871	**Slitrk6**	4832410J21Rik|Sltk6	SLIT and NTRK-like family, member 6	Amp	protein-coding
846_T1,T42, T4	chr14	1.1E+08	1.1E+08	999	+	**3**	**37.5**	NA	Intergenic	L1Md_F2|LINE|L1	-198851	NM_175 499	239250	Mm.497 28	NM_175 499	ENSMUS G000000 45871	**Slitrk6**	4832410J21Rik|Sltk6	SLIT and NTRK-like family, member 6	Amp	protein-coding
848_T22,T4 2,T4	chr14	1.1E+08	1.1E+08	999	+	**3**	**37.5**	NA	Intergenic	Intergenic	-202851	NM_175 499	239250	Mm.497 28	NM_175 499	ENSMUS G000000 45871	**Slitrk6**	4832410J21Rik|Sltk6	SLIT and NTRK-like family, member 6	Amp	protein-coding
1357_T2,T3 2,T4	chr19	5.1E+07	5.1E+07	999	+	**3**	**37.5**	NA	Intergenic	Intergenic	-268354	NM_001 290356	58178	Mm.313 672	NM_021 377	ENSMUS G000000 43531	**Sorcs1**	Sorcs|mSorCS	sortilin-related VPS10 domain containing receptor 1	Amp	protein-coding

1361_T12,T 1,T22	chr19	5.1E+07	5.1E+07	999	+	**3**	**37.5**	NA	Intergenic	Intergenic	-280354	NM_001 290356	58178	Mm.313 672	NM_021 377	ENSMUS G000000 43531	**Sorcs1**	Sorcs|mSorCS	sortilin-related VPS10 domain containing receptor 1	Amp	protein-coding
1352_T22,T 2,T3	chr19	4.9E+07	4.9E+07	999	+	**3**	**37.5**	NA	Intergenic	Lx9|LINE|L 1	962975	NM_025 696	66673	Mm.709 80	NM_025 696	ENSMUS G000000 63434	**Sorcs3**	6330404A12Rik| AW045579	sortilin-related VPS10 domain containing receptor 3	Amp	protein-coding
1741_T1,T3, T4	chr3	3.7E+07	3.7E+07	3999	+	**3**	**37.5**	NA	intron (NM_0013 10473, intron 14 of 16)	intron (NM_0013 10473, intron 14 of 16)	73550	NM_021 343	57815	Mm.172 679	NM_021 343	ENSMUS G000000 27722	**Spata5**	2510048F20Rik| C78064|Spaf	spermatogenesis associated 5	Amp	protein-coding
1433_T12,T 32,T42	chr2	2.4E+07	2.4E+07	3999	+	**3**	**37.5**	NA	Intergenic	Lx3A|LINE |L1	-218396	NM_001 165997	76857	Mm.276 415	NM_029 773	ENSMUS G000000 26771	**Spopl**	4921517N04Rik| AU014935|BB23 3739|E430033K0 4Rik	speckle-type POZ protein-like	Amp	protein-coding
57_T32,T42, T4	chr1	9.6E+07	9.6E+07	999	+	**3**	**37.5**	NA	intron (NM_0011 59745, intron 2 of 3)	intron (NM_0011 59745, intron 2 of 3)	10594	NM_009 183	20452	Mm.306 228	NM_009 183	ENSMUS G000000 40710	**St8sia4**	PST|PST- 1|SIAT8- D|ST8SiaIV|Siat 8d	ST8 alpha-N-acetyl-neuraminide alpha-2,8-sialyltransferase 4	Amp	protein-coding
2362_T1,T3 2,T4	chr3	6E+07	6E+07	4999	+	**3**	**37.5**	NA	Intergenic	Intergenic	128131	NM_032 400	84112	Mm.125 110	NM_032 400	ENSMUS G000000 27762	**Sucnr1**	Gpr91	succinate receptor 1	Amp	protein-coding
3326_T1,T3 2,T42	chr7	1.4E+07	1.4E+07	6999	+	**3**	**37.5**	NA	Intergenic	Intergenic	-8590	NM_001 111296	20859	Mm.260 026	NM_001 111296	ENSMUS G000000 78798	**Sult2a1**	ST2A1|Std|Sth1 |mSTa1	sulfotransferase family 2A, dehydroepiandrost erone (DHEA)-preferring, member 1	Amp	protein-coding
3782_T12,T 22,T2	chr9	1.1E+08	1.1E+08	999	+	**3**	**37.5**	NA	Intergenic	Intergenic	115646	NM_001 101510	382111	Mm.376 140	NM_001 101510	ENSMUS G000000 86596	**Susd5**	Gm1126	sushi domain containing 5	Amp	protein-coding
2491_T22,T 3,T4	chr3	1.3E+08	1.3E+08	3999	+	**3**	**37.5**	NA	Intergenic	Intergenic	122493	NM_021 382	21338	Mm.103 810	NM_021 382	ENSMUS G000000 28172	**Tacr3**	Nk3r|Nkr|Tac3r	tachykinin receptor 3	Amp	protein-coding
1378_T3,T4 2,T4	chr19	5.7E+07	5.7E+07	999	+	**3**	**37.5**	NA	intron (NM_0010 02238, intron 3 of 25)	L1MD1|LI NE|L1	5288	NM_001 002241	83561	Mm.247 541	NM_031 387	ENSMUS G000000 25081	**Tdrd1**	MTR-1	tudor domain containing 1	Amp	protein-coding
3498_T22,T 32,T3	chr8	4.9E+07	4.9E+07	4999	+	**3**	**37.5**	NA	Intergenic	ID4|SINE| ID	-456310	NM_011 857	23965	Mm.421 91	NM_011 857	ENSMUS G000000 31561	**Tenm3**	2610100B16Rik| Odz1|Odz3|Ten-m3|mKIAA1455	teneurin transmembrane protein 3	Amp	protein-coding
3446_T12,T 22,T3	chr8	3.4E+07	3.4E+07	3999	+	**3**	**37.5**	NA	exon (NM_0313 74, exon 1 of 4)	exon (NM_0313 74, exon 1 of 4)	1956	NM_031 374	104271	Mm.280 624	NM_031 374	ENSMUS G000000 09628	**Tex15**	2210014E14Rik| AL022622|AU02 2940	testis expressed gene 15	Amp	protein-coding
3585_T1,T2 2,T2	chr8	6.7E+07	6.7E+07	999	+	**3**	**37.5**	NA	Intergenic	Intergenic	55260	NM_028 927	74419	Mm.159 193	NM_028 927	ENSMUS G000000 25519	**Tktl2**	4933401I19Rik	transketolase-like 2	Amp	protein-coding

2311_T22,T3,T42	chr3	5.7E+07	5.7E+07	2999	+	**3**	**37.5**	NA	Intergenic	RLTR10-int|LTR|ERVK	45919	NM_008536	17112	Mm.856	NM_008536	ENSMUSG00000027800	**Tm4sf1**	L6|M3s1	transmembrane 4 superfamily member 1	Amp	protein-coding
34_T22,T32,T4	chr1	5.1E+07	5.1E+07	3999	+	**3**	**37.5**	NA	Intergenic	Intergenic	-306023	NM_019790	56363	Mm.245154	NM_019790	ENSMUSG00000026109	**Tmeff2**	4832418D20Rik|7630402F16Rik|TR-2	transmembrane protein with EGF-like and two follistatin-like domains 2	Amp	protein-coding
615_T1,T2,T3	chr14	6.1E+07	6.1E+07	5999	+	**3**	**37.5**	NA	Intergenic	L1_Mur3|LINE|L1	87487	NM_001164155	29820	Mm.281356	NM_013869	ENSMUSG00000060548	**Tnfrsf19**	AL023044|AW123854|TAJ|TAJ-ALPHA|TRADE|Troy	tumor necrosis factor receptor superfamily, member 19	Amp	protein-coding
1054_T1,T2,T4	chr15	5.1E+07	5.1E+07	999	+	**3**	**37.5**	NA	intron (NM_001310481, intron 4 of 5)	intron (NM_001310481, intron 4 of 5)	166049	NM_001310481	83925	Mm.30466	NM_032000	ENSMUSG00000038679	**Trps1**	AI115454|AI447310|D15Ertd586e	trichorhinophalangeal syndrome I (human)	Amp	protein-coding
2646_T2,T42,T4	chr4	9.3E+07	9.3E+07	3999	+	**3**	**37.5**	NA	Intergenic	Intergenic	490011	NM_026954	69136	Mm.171558	NM_026954	ENSMUSG00000054000	**Tusc1**	2200001D17Rik|TSG-9	tumor suppressor candidate 1	Amp	protein-coding
2647_T2,T32,T4	chr4	9.3E+07	9.3E+07	3999	+	**3**	**37.5**	NA	Intergenic	Intergenic	356011	NM_026954	69136	Mm.171558	NM_026954	ENSMUSG00000054000	**Tusc1**	2200001D17Rik|TSG-9	tumor suppressor candidate 1	Amp	protein-coding
2648_T1,T22,T2	chr4	9.3E+07	9.3E+07	6999	+	**3**	**37.5**	NA	Intergenic	Intergenic	198511	NM_026954	69136	Mm.171558	NM_026954	ENSMUSG00000054000	**Tusc1**	2200001D17Rik|TSG-9	tumor suppressor candidate 1	Amp	protein-coding
2653_T22,T2,T4	chr4	9.4E+07	9.4E+07	3999	+	**3**	**37.5**	NA	Intergenic	Intergenic	-430989	NM_026954	69136	Mm.171558	NM_026954	ENSMUSG00000054000	**Tusc1**	2200001D17Rik|TSG-9	tumor suppressor candidate 1	Amp	protein-coding
2654_T22,T2,T4	chr4	9.4E+07	9.4E+07	999	+	**3**	**37.5**	NA	Intergenic	Lx10|LINE|L1	-477489	NM_026954	69136	Mm.171558	NM_026954	ENSMUSG00000054000	**Tusc1**	2200001D17Rik|TSG-9	tumor suppressor candidate 1	Amp	protein-coding
2633_T22,T2,T42	chr4	8E+07	8E+07	4999	+	**3**	**37.5**	NA	Intergenic	RMER15-int|LTR|ERVL	-414213	NM_031202	22178	Mm.30438	NM_031202	ENSMUSG00000005994	**Tyrp1**	Oca3|TRP-1|TRP1|Tyrp|b|brown|isa	tyrosinase-related protein 1	Amp	protein-coding
3434_T1,T3,T42	chr8	3E+07	3E+07	999	+	**3**	**37.5**	NA	Intergenic	RLTR17B_Mm|LTR|ERVK	-479364	NM_153135	210801	Mm.248433	NM_153135	ENSMUSG00000063626	**Unc5d**	D930029E11Rik|Unc5h4|mKIAA1777	unc-5 netrin receptor D	Amp	protein-coding
3437_T12,T1,T2	chr8	3E+07	3E+07	999	+	**3**	**37.5**	NA	Intergenic	L1_Mur3|LINE|L1	-515364	NM_153135	210801	Mm.248433	NM_153135	ENSMUSG00000063626	**Unc5d**	D930029E11Rik|Unc5h4|mKIAA1777	unc-5 netrin receptor D	Amp	protein-coding
3541_T2,T3,T42	chr8	5.4E+07	5.4E+07	2999	+	**3**	**37.5**	NA	Intergenic	Intergenic	-96532	NM_009506	22341	Mm.1402	NM_009506	ENSMUSG00000031520	**Vegfc**	AW228853|VEGF-C	vascular endothelial growth factor C	Amp	protein-coding
3546_T2,T3,T4	chr8	5.4E+07	5.4E+07	999	+	**3**	**37.5**	NA	intron (NM_009506, intron 4 of 6)	intron (NM_009506, intron 4 of 6)	92468	NM_009506	22341	Mm.1402	NM_009506	ENSMUSG00000031520	**Vegfc**	AW228853|VEGF-C	vascular endothelial growth factor C	Amp	protein-coding
359_T12,T1,T3	chr11	1.6E+07	1.6E+07	999	+	**3**	**37.5**	NA	Intergenic	Lx7|LINE|L1	-688724	NM_001290539	211739	Mm.284015	NM_145967	ENSMUSG00000048834	**Vstm2a**	Vstm2	V-set and transmembrane domain containing 2A	Amp	protein-coding

365_T12,T1,T3	chr11	1.6E+07	1.6E+07	4999	+	**3**	**37.5**	NA	Intergenic	Intergenic	-127724	NM_001290539	211739	Mm.284015	NM_145967	ENSMUSG00000048834	**Vstm2a**	Vstm2	V-set and transmembrane domain containing 2A	Amp	protein-coding
350_T1,T22,T2	chr11	1.1E+07	1.1E+07	3999	+	**3**	**37.5**	NA	Intergenic	Intergenic	-278516	NM_177033	319922	Mm.337851	NM_177033	ENSMUSG00000050830	**Vwc2**	A930041G11Rik|G11|PSST739|UNQ739|cradin	von Willebrand factor C domain containing 2	Amp	protein-coding
1693_T12,T22,T3	chr3	1.6E+07	1.6E+07	999	+	**3**	**37.5**	NA	intron (NM_172677, intron 2 of 5)	intron (NM_172677, intron 2 of 5)	2817	NM_001145919	229096	Mm.23834	NM_172677	ENSMUSG00000047213	**Ythdf3**	9130022A11Rik	YTH domain family 3	Amp	protein-coding
2689_T3,T42,T4	chr4	1.4E+08	1.4E+08	722999	+	**3**	**37.5**	NA	intron (NM_198248, intron 1 of 18)	MIRb|SINE|MIR	29695	NM_198248	230848	Mm.31068	NM_198248	ENSMUSG00000060862	**Zbtb40**	BC059177|C230087D24|Gm571|mKIAA0478	zinc finger and BTB domain containing 40	Amp	protein-coding
1660_T22,T32,T4	chr3	7534501	7535500	999	+	**3**	**37.5**	NA	intron (NM_173181, intron 7 of 8)	RLTR17B_Mm|LTR|ERVK	31574	NM_173181	67306	Mm.332366	NM_173181	ENSMUSG00000043542	**Zc2hc1a**	3110050N22Rik|AI790358|AU023959|Fam164a	zinc finger, C2HC-type containing 1A	Amp	protein-coding
335_T32,T42	chr10	1.1E+08	1.1E+08	4999	+	**2**	**25**	NA	intron (NM_001205341, intron 21 of 31)	MTE2b|LTR|ERVL-MaLR	251664	NM_001142804	380660	Mm.336072	NM_198636	ENSMUSG00000035948	**Acss3**	8430416H19Rik|Gm874	acyl-CoA synthetase short-chain family member 3	Amp	protein-coding
3554_T2,T4	chr8	5.6E+07	5.6E+07	4999	+	**2**	**25**	NA	Intergenic	Intergenic	104964	NM_175939	244486	Mm.67684	NM_175939	ENSMUSG00000046258	**Adam29**	-	a disintegrin and metallopeptidase domain 29	Amp	protein-coding
3555_T2,T4	chr8	5.6E+07	5.6E+07	3999	+	**2**	**25**	NA	Intergenic	Intergenic	89464	NM_175939	244486	Mm.67684	NM_175939	ENSMUSG00000046258	**Adam29**	-	a disintegrin and metallopeptidase domain 29	Amp	protein-coding
586_T32,T3	chr13	1E+08	1E+08	999	+	**2**	**25**	NA	intron (NM_001081020, intron 14 of 24)	intron (NM_001081020, intron 14 of 24)	120127	NM_001081020	108154	Mm.208125	NM_175496	ENSMUSG00000046169	**Adamts6**	A930019D11Rik|ADAM-TS6|b2b1879.1Clo|b2b2029Clo|b2b2182Clo|b2b2187.1Clo|b2b2228Clo	a disintegrin-like and metallopeptidase (reprolysin type) with thrombospondin type 1 motif, 6	Amp	protein-coding
1316_T2,T32	chr17	9.2E+07	9.2E+07	4999	+	**2**	**25**	NA	Intergenic	(CA)n|Simple_repeat|Simple_repeat	-981017	NM_001315503	11516	Mm.3407	NM_009625	ENSMUSG00000024256	**Adcyap1**	PACAP	adenylate cyclase activating polypeptide 1	Amp	protein-coding
1317_T32,T42	chr17	9.3E+07	9.3E+07	999	+	**2**	**25**	NA	Intergenic	L1Md_A|LINE|L1	-273017	NM_001315503	11516	Mm.3407	NM_009625	ENSMUSG00000024256	**Adcyap1**	PACAP	adenylate cyclase activating polypeptide 1	Amp	protein-coding
1319_T22,T2	chr17	9.3E+07	9.3E+07	999	+	**2**	**25**	NA	Intergenic	Intergenic	-265017	NM_001315503	11516	Mm.3407	NM_009625	ENSMUSG00000024256	**Adcyap1**	PACAP	adenylate cyclase activating polypeptide 1	Amp	protein-coding

2859_T1,T32	chr5	8.1E+07	8.1E+07	999	+	**2**	**25**	NA	Intergenic	Intergenic	-403593	NM_198702	319387	Mm.273631	NM_198702	ENSMUSG00000037605	**Adgrl3**	5430402I23Rik|CIRL-3|D130075K09Rik|Gm1379|LEC3|Lphn3|mKIAA0768	adhesion G protein-coupled receptor L3	Amp	protein-coding
2862_T1,T32	chr5	8.2E+07	8.2E+07	5999	+	**2**	**25**	NA	Intergenic	Intergenic	781907	NM_198702	319387	Mm.273631	NM_198702	ENSMUSG00000037605	**Adgrl3**	5430402I23Rik|CIRL-3|D130075K09Rik|Gm1379|LEC3|Lphn3|mKIAA0768	adhesion G protein-coupled receptor L3	Amp	protein-coding
3531_T22,T4	chr8	5.3E+07	5.3E+07	999	+	**2**	**25**	NA	Intergenic	Intergenic	-834702	NM_001205054	11593	Mm.334535	NM_001005847	ENSMUSG00000031521	**Aga**	AW060726	aspartylglucosaminidase	Amp	protein-coding
3536_T2,T4	chr8	5.3E+07	5.3E+07	999	+	**2**	**25**	NA	Intergenic	Intergenic	-775702	NM_001205054	11593	Mm.334535	NM_001005847	ENSMUSG00000031521	**Aga**	AW060726	aspartylglucosaminidase	Amp	protein-coding
3537_T1,T2	chr8	5.3E+07	5.3E+07	3999	+	**2**	**25**	NA	Intergenic	RSINE1|SINE|B4	-496202	NM_001205054	11593	Mm.334535	NM_001005847	ENSMUSG00000031521	**Aga**	AW060726	aspartylglucosaminidase	Amp	protein-coding
974_T22,T32	chr15	4.3E+07	4.3E+07	4999	+	**2**	**25**	NA	intron (NM_009640, intron 1 of 8)	L1Md_T|LINE|L1	100977	NM_001286062	11600	Mm.309336	NM_009640	ENSMUSG00000022309	**Angpt1**	1110046O21Rik|Ang-1|Ang1	angiopoietin 1	Amp	protein-coding
3569_T2,T42	chr8	6.3E+07	6.3E+07	999	+	**2**	**25**	NA	Intergenic	Lx8|LINE|L1	-401807	NM_011922	26359	Mm.42179	NM_011922	ENSMUSG00000031635	**Anxa10**	-	annexin A10	Amp	protein-coding
441_T12,T1	chr12	5.3E+07	5.3E+07	999	+	**2**	**25**	NA	promoter-TSS (NM_009706)	promoter-TSS (NM_009706)	-77	NM_009706	11855	Mm.35059	NM_009706	ENSMUSG00000035133	**Arhgap5**	p190-B|p190B	Rho GTPase activating protein 5	Amp	protein-coding
545_T22,T2	chr13	8E+07	8E+07	999	+	**2**	**25**	NA	Intergenic	Intergenic	-481422	NM_001042591	105171	Mm.423137	NM_001042591	ENSMUSG00000074794	**Arrdc3**	AI450344|mKIAA1376	arrestin domain containing 3	Amp	protein-coding
165_T2,T4	chr1	1.4E+08	1.4E+08	3999	+	**2**	**25**	NA	Intergenic	Intergenic	-600197	NM_020025	26878	Mm.285580	NM_020025	ENSMUSG00000033849	**B3galt2**	-	UDP-Gal:betaGlcNAc beta 1,3-galactosyltransferase, polypeptide 2	Amp	protein-coding
2496_T3,T4	chr3	1.4E+08	1.4E+08	3999	+	**2**	**25**	NA	intron (NM_001310749, intron 6 of 15)	intron (NM_001310749, intron 6 of 15)	133566	NM_001310749	242248	Mm.30832	NM_001033350	ENSMUSG00000037922	**Bank1**	A530094C12Rik|AI451642|AVIEF|BANK	B cell scaffold protein with ankyrin repeats 1	Amp	protein-coding
2603_T22,T2	chr4	6.8E+07	6.8E+07	4999	+	**2**	**25**	NA	Intergenic	Intergenic	472397	NM_019967	56710	Mm.248788	NM_019967	ENSMUSG00000028351	**Brinp1**	BRINP|Dbc1|Dbccr1|Fam5a	bone morphogenic protein/retinoic acid inducible neural specific 1	Amp	protein-coding

2605_T2,T4	chr4	6.9E+07	6.9E+07	2999	+	**2**	**25**	NA	Intergenic	Intergenic	222397	NM_019967	56710	Mm.248788	NM_019967	ENSMUSG00000028351	**Brinp1**	BRINP|Dbc1|Dbccr1|Fam5a	bone morphogenic protein/retinoic acid inducible neural specific 1	Amp	protein-coding
2608_T22,T2	chr4	6.9E+07	6.9E+07	4999	+	**2**	**25**	NA	Intergenic	Intergenic	-245603	NM_019967	56710	Mm.248788	NM_019967	ENSMUSG00000028351	**Brinp1**	BRINP|Dbc1|Dbccr1|Fam5a	bone morphogenic protein/retinoic acid inducible neural specific 1	Amp	protein-coding
179_T22,T3	chr1	1.5E+08	1.5E+08	1999	+	**2**	**25**	NA	Intergenic	AT_rich|Low_complexity|Low_complexity	-624166	NM_153539	215378	Mm.441817	NM_153539	ENSMUSG00000035131	**Brinp3**	B830045N13Rik|Fam5c	bone morphogenetic protein/retinoic acid inducible neural specific 3	Amp	protein-coding
186_T1,T4	chr1	1.5E+08	1.5E+08	999	+	**2**	**25**	NA	Intergenic	L1Md_F2|LINE|L1	615313	NM_001145807	215378	Mm.441817	NM_153539	ENSMUSG00000035131	**Brinp3**	B830045N13Rik|Fam5c	bone morphogenetic protein/retinoic acid inducible neural specific 3	Amp	protein-coding
185_T12,T32	chr1	1.5E+08	1.5E+08	999	+	**2**	**25**	NA	intron (NM_001145807, intron 7 of 7)	intron (NM_001145807, intron 7 of 7)	352313	NM_001145807	215378	Mm.441817	NM_153539	ENSMUSG00000035131	**Brinp3**	B830045N13Rik|Fam5c	bone morphogenetic protein/retinoic acid inducible neural specific 3	Amp	protein-coding
1603_T12,T3	chr2	1.4E+08	1.4E+08	3999	+	**2**	**25**	NA	Intergenic	MIR|SINE|MIR	-471084	NM_001025431	228662	Mm.153405	NM_145534	ENSMUSG00000062098	**Btbd3**	mKIAA0952	BTB (POZ) domain containing 3	Amp	protein-coding
1420_T42,T4	chr19	6.1E+07	6.1E+07	6999	+	**2**	**25**	NA	Intergenic	L1Md_T|LINE|L1	-65977	NM_001172096	78832	Mm.259026	NM_030197	ENSMUSG00000033417	**Cacul1**	2700078E11Rik|2810417M16Rik|9830127L17Rik|AI450346|D130033C15Rik	CDK2 associated, cullin domain 1	Amp	protein-coding
3681_T2,T3	chr9	5435501	5439500	3999	+	**2**	**25**	NA	Intergenic	Intergenic	92024	NM_009808	12364	Mm.42163	NM_009808	ENSMUSG00000025887	**Casp12**	-	caspase 12	Amp	protein-coding
562_T22,T2	chr13	8.5E+07	8.5E+07	999	+	**2**	**25**	NA	intron (NM_145452, intron 11 of 24)	L1MEg|LINE|L1	42523	NM_023243	66671	Mm.18474	NM_023243	ENSMUSG00000021548	**Ccnh**	6330408H09Rik|AI661354|AV102684|AW538719	cyclin H	Amp	protein-coding
931_T3,T4	chr15	1.9E+07	1.9E+07	999	+	**2**	**25**	NA	Intergenic	Lx10|LINE|L1	367671	NM_009865	320873	Mm.117794	NM_009865	ENSMUSG00000022321	**Cdh10**	A830016G23Rik|C030003B10Rik|C030011H18Rik	cadherin 10	Amp	protein-coding
935_T12,T4	chr15	2E+07	2E+07	3999	+	**2**	**25**	NA	Intergenic	Intergenic	795171	NM_009865	320873	Mm.117794	NM_009865	ENSMUSG00000022321	**Cdh10**	A830016G23Rik|C030003B10Rik|C030011H18Rik	cadherin 10	Amp	protein-coding
3644_T22,T4	chr8	1E+08	1E+08	2999	+	**2**	**25**	NA	Intergenic	Intergenic	1625111	NM_009866	12552	Mm.1571	NM_009866	ENSMUSG00000031673	**Cdh11**	Cad11|OSF-4	cadherin 11	Amp	protein-coding
3656_T2,T4	chr8	1E+08	1E+08	999	+	**2**	**25**	NA	Intergenic	L2a|LINE|L2	1147111	NM_009866	12552	Mm.1571	NM_009866	ENSMUSG00000031673	**Cdh11**	Cad11|OSF-4	cadherin 11	Amp	protein-coding

3663_T1,T2	chr8	1E+08	1E+08	999	+	**2**	**25**	NA	Intergenic	L1M4|LINE|L1	755111	NM_009866	12552	Mm.1571	NM_009866	ENSMUSG00000031673	**Cdh11**	Cad11|OSF-4	cadherin 11	Amp	protein-coding
3668_T1,T32	chr8	1E+08	1E+08	3999	+	**2**	**25**	NA	Intergenic	Intergenic	337611	NM_009866	12552	Mm.1571	NM_009866	ENSMUSG00000031673	**Cdh11**	Cad11|OSF-4	cadherin 11	Amp	protein-coding
882_T12,T3	chr15	1.4E+07	1.4E+07	3999	+	**2**	**25**	NA	Intergenic	L1ME1|LINE|L1	-622825	NM_007666	12563	Mm.57048	NM_007666	ENSMUSG00000039385	**Cdh6**	cad6	cadherin 6	Amp	protein-coding
885_T22,T4	chr15	1.5E+07	1.5E+07	999	+	**2**	**25**	NA	Intergenic	Intergenic	-2E+06	NM_007666	12563	Mm.57048	NM_007666	ENSMUSG00000039385	**Cdh6**	cad6	cadherin 6	Amp	protein-coding
887_T1,T3	chr15	1.5E+07	1.5E+07	4999	+	**2**	**25**	NA	Intergenic	Intergenic	-2E+06	NM_007666	12563	Mm.57048	NM_007666	ENSMUSG00000039385	**Cdh6**	cad6	cadherin 6	Amp	protein-coding
117_T22,T3	chr1	1.1E+08	1.1E+08	999	+	**2**	**25**	NA	Intergenic	Intergenic	-685900	NM_001316743	241201	Mm.213407	NM_172853	ENSMUSG00000026312	**Cdh7**	9330156F07Rik|CDH7L1	cadherin 7, type 2	Amp	protein-coding
124_T1,T2	chr1	1.1E+08	1.1E+08	999	+	**2**	**25**	NA	Intergenic	Intergenic	161277	NM_172853	241201	Mm.213407	NM_172853	ENSMUSG00000026312	**Cdh7**	9330156F07Rik|CDH7L1	cadherin 7, type 2	Amp	protein-coding
3621_T32,T4	chr8	9.9E+07	9.9E+07	3999	+	**2**	**25**	NA	intron (NR_133570, intron 3 of 10)	Lx9|LINE|L1	167971	NM_001285913	12564	Mm.441131	NM_007667	ENSMUSG00000036510	**Cdh8**	AI851472|cad8	cadherin 8	Amp	protein-coding
899_T2,T4	chr15	1.6E+07	1.6E+07	999	+	**2**	**25**	NA	Intergenic	Lx|LINE|L1	-1E+06	NM_009869	12565	Mm.439758	NM_009869	ENSMUSG00000025370	**Cdh9**	-	cadherin 9	Amp	protein-coding
905_T22,T32	chr15	1.6E+07	1.6E+07	3999	+	**2**	**25**	NA	Intergenic	B1_Mur2|SINE|Alu	-507601	NM_009869	12565	Mm.439758	NM_009869	ENSMUSG00000025370	**Cdh9**	-	cadherin 9	Amp	protein-coding
910_T1,T4	chr15	1.7E+07	1.7E+07	3999	+	**2**	**25**	NA	Intergenic	Intergenic	293399	NM_009869	12565	Mm.439758	NM_009869	ENSMUSG00000025370	**Cdh9**	-	cadherin 9	Amp	protein-coding
908_T12,T1	chr15	1.7E+07	1.7E+07	999	+	**2**	**25**	NA	intron (NM_009869, intron 1 of 10)	intron (NM_009869, intron 1 of 10)	24899	NM_009869	12565	Mm.439758	NM_009869	ENSMUSG00000025370	**Cdh9**	-	cadherin 9	Amp	protein-coding
549_T1,T4	chr13	8.2E+07	8.2E+07	3999	+	**2**	**25**	NA	Intergenic	L1_Mus3|LINE|L1	62208	NM_007684	12626	Mm.12481	NM_007684	ENSMUSG00000021537	**Cetn3**	MmCEN3	centrin 3	Amp	protein-coding
150_T1,T4	chr1	1.4E+08	1.4E+08	3999	+	**2**	**25**	NA	intron (NM_009888, intron 21 of 21)	intron (NM_009888, intron 21 of 21)	94911	NM_009888	12628	Mm.8655	NM_009888	ENSMUSG00000026365	**Cfh**	Mud-1|NOM|Sas-1|Sas1	complement component factor h	Amp	protein-coding
3728_T1,T22	chr9	1.7E+07	1.7E+07	3999	+	**2**	**25**	NA	Intergenic	Intergenic	-860767	NM_025844	66917	Mm.103534	NM_025844	ENSMUSG00000001774	**Chordc1**	1110001O09Rik|AA409036|Chp-1|morgana	cysteine and histidine-rich domain (CHORD)-containing, zinc-binding protein 1	Amp	protein-coding

2543_T22,T2	chr4	1.9E+07	1.9E+07	3999	+	**2**	**25**	NA	Intergenic	Intergenic	-594350	NM_013927	30952	Mm.445778	NM_013927	ENSMUSG00000056494	**Cngb3**	CCNC2|CNG6|Cngbeta2	cyclic nucleotide gated channel beta 3	Amp	protein-coding
2544_T32,T3	chr4	1.9E+07	1.9E+07	999	+	**2**	**25**	NA	Intergenic	(CA)n|Simple_repeat|Simple_repeat	-30850	NM_013927	30952	Mm.445778	NM_013927	ENSMUSG00000056494	**Cngb3**	CCNC2|CNG6|Cngbeta2	cyclic nucleotide gated channel beta 3	Amp	protein-coding
3049_T12,T1	chr6	4.4E+07	4.4E+07	4999	+	**2**	**25**	NA	Intergenic	Intergenic	-576061	NM_001004357	66797	Mm.440084	NM_025771	ENSMUSG00000039419	**Cntnap2**	5430425M22Rik|Caspr2|mKIAA0868	contactin associated protein-like 2	Amp	protein-coding
3053_T12,T1	chr6	4.5E+07	4.5E+07	999	+	**2**	**25**	NA	intron (NM_001004357, intron 1 of 23)	intron (NM_001004357, intron 1 of 23)	61939	NM_001004357	66797	Mm.440084	NM_025771	ENSMUSG00000039419	**Cntnap2**	5430425M22Rik|Caspr2|mKIAA0868	contactin associated protein-like 2	Amp	protein-coding
2470_T12,T2	chr3	1.1E+08	1.1E+08	999	+	**2**	**25**	NA	intron (NM_007729, intron 4 of 66)	intron (NM_007729, intron 4 of 66)	43460	NM_007729	12814	Mm.209715	NM_007729	ENSMUSG00000027966	**Col11a1**	C530001D20Rik|cho	collagen, type XI, alpha 1	Amp	protein-coding
197_T12,T4	chr1	1.8E+08	1.8E+08	1999	+	**2**	**25**	NA	exon (NM_025511, exon 4 of 4)	exon (NM_025511, exon 4 of 4)	3347	NM_025511	66359	Mm.489720	NM_025511	ENSMUSG00000026500	**Cox20**	2310005N03Rik|Fam36a	COX20 Cox2 chaperone	Amp	protein-coding
564_T22,T32	chr13	8.6E+07	8.6E+07	3999	+	**2**	**25**	NA	Intergenic	L1_Mus4|LINE|L1	245295	NM_007749	12867	Mm.378898	NM_007749	ENSMUSG00000017778	**Cox7c**	COXVIIc|Cox7c1	cytochrome c oxidase subunit VIIc	Amp	protein-coding
568_T32,T3	chr13	8.6E+07	8.6E+07	999	+	**2**	**25**	NA	Intergenic	Lx5|LINE|L1	-289205	NM_007749	12867	Mm.378898	NM_007749	ENSMUSG00000017778	**Cox7c**	COXVIIc|Cox7c1	cytochrome c oxidase subunit VIIc	Amp	protein-coding
570_T42,T4	chr13	8.6E+07	8.6E+07	8999	+	**2**	**25**	NA	Intergenic	RLTR51A_Mm|LTR|ERVK?	-436205	NM_007749	12867	Mm.378898	NM_007749	ENSMUSG00000017778	**Cox7c**	COXVIIc|Cox7c1	cytochrome c oxidase subunit VIIc	Amp	protein-coding
3109_T12,T32	chr6	1.1E+08	1.1E+08	2999	+	**2**	**25**	NA	Intergenic	L1Md_F2|LINE|L1	-81913	NM_175357	58799	Mm.290085	NM_021449	ENSMUSG00000005362	**Crbn**	2610203G15Rik|2900045O07Rik|AF229032|AW108261|piL	cereblon	Amp	protein-coding
1310_T32,T4	chr17	7.7E+07	7.7E+07	999	+	**2**	**25**	NA	Intergenic	L1_Mus3|LINE|L1	-984248	NM_015800	50766	Mm.311912	NM_015800	ENSMUSG00000024074	**Crim1**	AU015004	cysteine rich transmembrane BMP regulator 1 (chordin like)	Amp	protein-coding
1014_T22,T2	chr15	4.8E+07	4.8E+07	2999	+	**2**	**25**	NA	intron (NM_001081391, intron 16 of 70)	ORR1D1|LTR|ERVL-MaLR	807989	NM_001081391	239420	Mm.309363	NM_001081391	ENSMUSG00000022311	**Csmd3**	4930500N14Rik|mKIAA1894	CUB and Sushi multiple domains 3	Amp	protein-coding
1021_T1,T32	chr15	4.8E+07	4.8E+07	9999	+	**2**	**25**	NA	intron (NM_001081391, intron 7 of 70)	intron (NM_001081391, intron 7 of 70)	497489	NM_001081391	239420	Mm.309363	NM_001081391	ENSMUSG00000022311	**Csmd3**	4930500N14Rik|mKIAA1894	CUB and Sushi multiple domains 3	Amp	protein-coding

1612_T3,T4	chr2	1.5E+08	1.5E+08	999	+	**2**	**25**	NA	Intergenic	MTC|LTR|ERVL-MaLR	-68488	NM_009976	13010	Mm.4263	NM_009976	ENSMUSG00000027447	**Cst3**	CysC	cystatin C	Amp	protein-coding
1615_T32,T4	chr2	1.5E+08	1.5E+08	999	+	**2**	**25**	NA	Intergenic	RLTR17D_Mm|LTR|ERVK	-119488	NM_009976	13010	Mm.4263	NM_009976	ENSMUSG00000027447	**Cst3**	CysC	cystatin C	Amp	protein-coding
1617_T12,T4	chr2	1.5E+08	1.5E+08	3999	+	**2**	**25**	NA	Intergenic	Intergenic	-168988	NM_009976	13010	Mm.4263	NM_009976	ENSMUSG00000027447	**Cst3**	CysC	cystatin C	Amp	protein-coding
1619_T1,T2	chr2	1.5E+08	1.5E+08	999	+	**2**	**25**	NA	Intergenic	Intergenic	-208488	NM_009976	13010	Mm.4263	NM_009976	ENSMUSG00000027447	**Cst3**	CysC	cystatin C	Amp	protein-coding
965_T2,T32	chr15	3E+07	3E+07	999	+	**2**	**25**	NA	Intergenic	Intergenic	-536593	NM_008729	18163	Mm.321648	NM_008729	ENSMUSG00000022240	**Ctnnd2**	Catnd2|Nprap|neurojugin	catenin (cadherin associated protein), delta 2	Amp	protein-coding
2430_T32,T3	chr3	8.2E+07	8.2E+07	4999	+	**2**	**25**	NA	Intergenic	Intergenic	-34616	NM_177662	229445	Mm.254642	NM_177662	ENSMUSG00000028015	**Ctso**	A330105D01Rik|AI118514	cathepsin O	Amp	protein-coding
49_T12,T22	chr1	8E+07	8E+07	3999	+	**2**	**25**	NA	intron (NM_016716, intron 2 of 15)	intron (NM_016716, intron 2 of 15)	19190	NM_001313728	26554	Mm.12665	NM_016716	ENSMUSG00000004364	**Cul3**	KIAA0617	cullin 3	Amp	protein-coding
788_T1,T22	chr14	9.8E+07	9.8E+07	1999	+	**2**	**25**	NA	Intergenic	CT-rich|Low_complexity|Low_complexity	-33735	NM_007826	13134	Mm.320593	NM_007826	ENSMUSG00000055639	**Dach1**	Dac|Dach	dachshund 1 (Drosophila)	Amp	protein-coding
786_T3,T4	chr14	9.8E+07	9.8E+07	10999	+	**2**	**25**	NA	intron (NM_007826, intron 1 of 11)	L1Md_F3|LINE|L1	107765	NM_001038610	13134	Mm.320593	NM_007826	ENSMUSG00000055639	**Dach1**	Dac|Dach	dachshund 1 (Drosophila)	Amp	protein-coding
962_T1,T2	chr15	2.9E+07	2.9E+07	3999	+	**2**	**25**	NA	Intergenic	Intergenic	616734	NM_133365	110082	Mm.248464	NM_133365	ENSMUSG00000022262	**Dnah5**	AU022615|Dnahc5|Mdnah5|b2b1134Clo|b2b1154Clo|b2b1537Clo|b2b1565Clo|b2b601Clo|mKIAA1603	dynein, axonemal, heavy chain 5	Amp	protein-coding
963_T1,T2	chr15	2.9E+07	2.9E+07	3999	+	**2**	**25**	NA	Intergenic	ORR1B1|LTR|ERVL-MaLR	715734	NM_133365	110082	Mm.248464	NM_133365	ENSMUSG00000022262	**Dnah5**	AU022615|Dnahc5|Mdnah5|b2b1134Clo|b2b1154Clo|b2b1537Clo|b2b1565Clo|b2b601Clo|mKIAA1603	dynein, axonemal, heavy chain 5	Amp	protein-coding
428_T3,T4	chr12	4.3E+07	4.3E+07	999	+	**2**	**25**	NA	Intergenic	Intergenic	1110068	NM_013760	27362	Mm.27432	NM_013760	ENSMUSG00000014905	**Dnajb9**	AA408011|AA673251|AA673481|AW556981|ERdj4|Mdg1|mDj7	DnaJ heat shock protein family (Hsp40) member B9	Amp	protein-coding

430_T3,T4	chr12	4.3E+07	4.3E+07	999	+	**2**	**25**	NA	Intergenic	Intergenic	1102068	NM_013760	27362	Mm.27432	NM_013760	ENSMUSG00000014905	**Dnajb9**	AA408011|AA673251|AA673481|AW556981|ERdj4|Mdg1|mDj7	DnaJ heat shock protein family (Hsp40) member B9	Amp	protein-coding
1504_T3,T42	chr2	6.2E+07	6.2E+07	999	+	**2**	**25**	NA	intron (NM_001242381, intron 6 of 25)	intron (NM_001242381, intron 6 of 25)	170231	NM_010074	13482	Mm.1151	NM_010074	ENSMUSG00000035000	**Dpp4**	Cd26|Dpp-4|THAM	dipeptidylpeptidase 4	Amp	protein-coding
125_T12,T4	chr1	1.1E+08	1.1E+08	3999	+	**2**	**25**	NA	Intergenic	Intergenic	7418	NM_001081316	319901	Mm.103468	NM_001081316	ENSMUSG00000038702	**Dsel**	9330132E09Rik|DS-epi2	dermatan sulfate epimerase-like	Amp	protein-coding
136_T3,T42	chr1	1.1E+08	1.1E+08	999	+	**2**	**25**	NA	Intergenic	Intergenic	-1E+06	NM_001081316	319901	Mm.103468	NM_001081316	ENSMUSG00000038702	**Dsel**	9330132E09Rik|DS-epi2	dermatan sulfate epimerase-like	Amp	protein-coding
1161_T3,T42	chr16	4.9E+07	4.9E+07	999	+	**2**	**25**	NA	intron (NM_027341, intron 21 of 30)	intron (NM_027341, intron 21 of 30)	54112	NM_001110017	224170	Mm.275138	NM_027341	ENSMUSG00000064061	**Dzip3**	2310047C04Rik|2A-HUB|6430549P11Rik|A230104G20	DAZ interacting protein 3, zinc finger	Amp	protein-coding
577_T12,T22	chr13	8.9E+07	8.9E+07	999	+	**2**	**25**	NA	intron (NM_010103, intron 1 of 9)	intron (NM_010103, intron 1 of 9)	100528	NM_001037987	13612	Mm.125580	NM_010103	ENSMUSG00000034488	**Edil3**	Del-1|Del1	EGF-like repeats and discoidin I-like domains 3	Amp	protein-coding
1290_T12,T32	chr17	6.1E+07	6.1E+07	999	+	**2**	**25**	NA	Intergenic	Intergenic	1662317	NM_207654	13640	Mm.7978	NM_010109	ENSMUSG00000048915	**Efna5**	AL-1|AV158822|EFL-5|Ephrin-A5|Epl7|LERK-7|RAGS	ephrin A5	Amp	protein-coding
3420_T2,T32	chr8	7914501	7917500	2999	+	**2**	**25**	NA	Intergenic	L1MA9|LINE|L1	744773	NM_010111	13642	Mm.209813	NM_010111	ENSMUSG00000001300	**Efnb2**	ELF-2|Epl5|Eplg5|Htk-L|LERK-5|Lerk5|NLERK-1	ephrin B2	Amp	protein-coding
3423_T2,T4	chr8	7974501	7979500	4999	+	**2**	**25**	NA	Intergenic	Intergenic	683773	NM_010111	13642	Mm.209813	NM_010111	ENSMUSG00000001300	**Efnb2**	ELF-2|Epl5|Eplg5|Htk-L|LERK-5|Lerk5|NLERK-1	ephrin B2	Amp	protein-coding
3425_T3,T42	chr8	7982501	7983500	999	+	**2**	**25**	NA	Intergenic	Intergenic	677773	NM_010111	13642	Mm.209813	NM_010111	ENSMUSG00000001300	**Efnb2**	ELF-2|Epl5|Eplg5|Htk-L|LERK-5|Lerk5|NLERK-1	ephrin B2	Amp	protein-coding
3428_T22,T2	chr8	8203501	8205500	1999	+	**2**	**25**	NA	Intergenic	L1Md_F2|LINE|L1	456273	NM_010111	13642	Mm.209813	NM_010111	ENSMUSG00000001300	**Efnb2**	ELF-2|Epl5|Eplg5|Htk-L|LERK-5|Lerk5|NLERK-1	ephrin B2	Amp	protein-coding

2871_T12,T4	chr5	8.5E+07	8.5E+07	999	+	**2**	**25**	NA	Intergenic	AT_rich|Low_complexity|Low_complexity	-255618	NM_007937	13839	Mm.137991	NM_007937	ENSMUSG00000029245	**Epha5**	AI854630|AW125296|Cek7|Ehk1|Els1|Hek7|Rek7|bsk	Eph receptor A5	Amp	protein-coding
1163_T2,T4	chr16	6.1E+07	6.1E+07	999	+	**2**	**25**	NA	Intergenic	Intergenic	-662469	NM_007938	13840	Mm.455790	NM_007938	ENSMUSG00000055540	**Epha6**	Ehk2|Hek12|m-ehk2	Eph receptor A6	Amp	protein-coding
2560_T22,T32	chr4	2.9E+07	2.9E+07	999	+	**2**	**25**	NA	intron (NM_010141, intron 5 of 16)	intron (NM_010141, intron 5 of 16)	71855	NM_001122889	13841	Mm.257266	NM_010141	ENSMUSG00000028289	**Epha7**	Cek11|Ebk|Ehk3|Hek11|Mdk1	Eph receptor A7	Amp	protein-coding
43_T12,T2	chr1	6.8E+07	6.8E+07	1999	+	**2**	**25**	NA	intron (NM_010154, intron 3 of 26)	intron (NM_010154, intron 3 of 26)	708559	NM_010154	13869	Mm.442420	NM_010154	ENSMUSG00000062209	**Erbb4**	Her4|c-erbB-4	erb-b2 receptor tyrosine kinase 4	Amp	protein-coding
41_T22,T4	chr1	6.8E+07	6.8E+07	999	+	**2**	**25**	NA	intron (NM_010154, intron 4 of 26)	intron (NM_010154, intron 4 of 26)	714059	NM_010154	13869	Mm.442420	NM_010154	ENSMUSG00000062209	**Erbb4**	Her4|c-erbB-4	erb-b2 receptor tyrosine kinase 4	Amp	protein-coding
1179_T32,T42	chr16	9.6E+07	9.6E+07	3999	+	**2**	**25**	NA	Intergenic	L1Md_T|LINE|L1	83093	NM_011809	23872	Mm.290207	NM_011809	ENSMUSG00000022895	**Ets2**	AU022856|Ets-2	E26 avian leukemia oncogene 2, 3' domain	Amp	protein-coding
865_T3,T4	chr14	1.2E+08	1.2E+08	999	+	**2**	**25**	NA	intron (NM_207667, intron 1 of 4)	(TATATG)n|Simple_repeat|Simple_repeat	-106454	NM_010201	14169	Mm.206362	NM_010201	ENSMUSG00000025551	**Fgf14**	FHF-4|Fhf4|mFHF-4(1B)	fibroblast growth factor 14	Amp	protein-coding
867_T12,T22	chr14	1.2E+08	1.2E+08	1999	+	**2**	**25**	NA	intron (NM_207667, intron 1 of 4)	intron (NM_207667, intron 1 of 4)	-112954	NM_010201	14169	Mm.206362	NM_010201	ENSMUSG00000025551	**Fgf14**	FHF-4|Fhf4|mFHF-4(1B)	fibroblast growth factor 14	Amp	protein-coding
2206_T3,T42	chr3	5.2E+07	5.2E+07	7999	+	**2**	**25**	NA	Intergenic	L1Md_T|LINE|L1	165163	NM_019739	56458	Mm.29891	NM_019739	ENSMUSG00000044167	**Foxo1**	AI876417|Afxh|FKHR|Fkhr1|Foxo1a	forkhead box O1	Amp	protein-coding
2897_T1,T32	chr6	1.5E+07	1.5E+07	3999	+	**2**	**25**	NA	intron (NM_053242, intron 5 of 18)	AT_rich|Low_complexity|Low_complexity	104994	NM_001286607	114142	Mm.332919	NM_053242	ENSMUSG00000029563	**Foxp2**	2810043D05Rik|AI449000|CAG-16|D0Kist7	forkhead box P2	Amp	protein-coding
2901_T12,T32	chr6	1.5E+07	1.5E+07	5999	+	**2**	**25**	NA	intron (NM_053242, intron 5 of 18)	intron (NM_053242, intron 5 of 18)	143994	NM_001286607	114142	Mm.332919	NM_053242	ENSMUSG00000029563	**Foxp2**	2810043D05Rik|AI449000|CAG-16|D0Kist7	forkhead box P2	Amp	protein-coding
594_T32,T42	chr13	1.1E+08	1.1E+08	5999	+	**2**	**25**	NA	Intergenic	Intergenic	-58549	NM_008046	14313	Mm.4913	NM_008046	ENSMUSG00000021765	**Fst**	AL033346|FS	follistatin	Amp	protein-coding
2402_T1,T2	chr3	7.7E+07	7.7E+07	3999	+	**2**	**25**	NA	Intergenic	RLTR11D|LTR|ERVK	648917	NM_001253719	213262	Mm.379337	NM_178673	ENSMUSG00000034098	**Fstl5**	9130207J01Rik	follistatin-like 5	Amp	protein-coding

2404_T42,T4	chr3	7.7E+07	7.7E+07	999	+	**2**	**25**	NA	Intergenic	Intergenic	864417	NM_001253719	213262	Mm.379337	NM_178673	ENSMUSG00000034098	**Fstl5**	9130207J01Rik	follistatin-like 5	Amp	protein-coding
2409_T32,T3	chr3	7.7E+07	7.7E+07	999	+	**2**	**25**	NA	Intergenic	Intergenic	1076417	NM_001253719	213262	Mm.379337	NM_178673	ENSMUSG00000034098	**Fstl5**	9130207J01Rik	follistatin-like 5	Amp	protein-coding
2415_T1,T32	chr3	7.7E+07	7.7E+07	999	+	**2**	**25**	NA	Intergenic	(TAGA)n|Simple_repeat|Simple_repeat	1417417	NM_001253719	213262	Mm.379337	NM_178673	ENSMUSG00000034098	**Fstl5**	9130207J01Rik	follistatin-like 5	Amp	protein-coding
2401_T12,T32	chr3	7.7E+07	7.7E+07	999	+	**2**	**25**	NA	intron (NM_178673, intron 13 of 15)	intron (NM_178673, intron 13 of 15)	575417	NM_001253719	213262	Mm.379337	NM_178673	ENSMUSG00000034098	**Fstl5**	9130207J01Rik	follistatin-like 5	Amp	protein-coding
2394_T32,T3	chr3	7.6E+07	7.6E+07	999	+	**2**	**25**	NA	intron (NM_178673, intron 3 of 15)	intron (NM_178673, intron 3 of 15)	200417	NM_001253719	213262	Mm.379337	NM_178673	ENSMUSG00000034098	**Fstl5**	9130207J01Rik	follistatin-like 5	Amp	protein-coding
2396_T32,T4	chr3	7.6E+07	7.6E+07	3999	+	**2**	**25**	NA	intron (NM_178673, intron 7 of 15)	intron (NM_178673, intron 7 of 15)	417917	NM_001253719	213262	Mm.379337	NM_178673	ENSMUSG00000034098	**Fstl5**	9130207J01Rik	follistatin-like 5	Amp	protein-coding
2554_T32,T3	chr4	2.6E+07	2.6E+07	3999	+	**2**	**25**	NA	Intergenic	Lx8b|LINE|L1	-82497	NM_010243	14348	Mm.39101	NM_010243	ENSMUSG00000055373	**Fut9**	AI746471|AU067636|mFUT9|mFuc-TIX	fucosyltransferase 9	Amp	protein-coding
389_T22,T2	chr11	4.2E+07	4.2E+07	999	+	**2**	**25**	NA	intron (NM_010250, intron 3 of 9)	intron (NM_010250, intron 3 of 9)	12930	NM_010250	14394	Mm.439668	NM_010250	ENSMUSG00000010803	**Gabra1**	Gabra-1	gamma-aminobutyric acid (GABA) A receptor, subunit alpha 1	Amp	protein-coding
386_T1,T4	chr11	4.2E+07	4.2E+07	999	+	**2**	**25**	NA	intron (NM_010250, intron 7 of 9)	intron (NM_010250, intron 7 of 9)	38930	NM_010250	14394	Mm.439668	NM_010250	ENSMUSG00000010803	**Gabra1**	Gabra-1	gamma-aminobutyric acid (GABA) A receptor, subunit alpha 1	Amp	protein-coding
391_T22,T3	chr11	4.3E+07	4.3E+07	999	+	**2**	**25**	NA	intron (NM_008070, intron 6 of 9)	intron (NM_008070, intron 6 of 9)	125243	NM_008070	14401	Mm.338723	NM_008070	ENSMUSG00000007653	**Gabrb2**	AI834970|C030002O17Rik|C030021G16Rik|Gabrab2|Gabrb-2	gamma-aminobutyric acid (GABA) A receptor, subunit beta 2	Amp	protein-coding
2832_T2,T32	chr5	7.1E+07	7.1E+07	3999	+	**2**	**25**	NA	Intergenic	Intergenic	317117	NM_010252	14405	Mm.255292	NM_010252	ENSMUSG00000001260	**Gabrg1**	GabaA|GabaA/BZ	gamma-aminobutyric acid (GABA) A receptor, subunit gamma 1	Amp	protein-coding
314_T1,T32	chr10	7.5E+07	7.5E+07	999	+	**2**	**25**	NA	Intergenic	Intergenic	-32231	NM_010311	14687	Mm.32595	NM_010311	ENSMUSG00000040009	**Gnaz**	AI847979|Gz	guanine nucleotide binding protein, alpha z subunit	Amp	protein-coding
3549_T2,T4	chr8	5.5E+07	5.5E+07	4999	+	**2**	**25**	NA	intron (NM_001253754, intron 1 of 6)	intron (NM_001253754, intron 1 of 6)	10566	NM_001253754	234267	Mm.241700	NM_153581	ENSMUSG00000031517	**Gpm6a**	Gpm6|M6A	glycoprotein m6a	Amp	protein-coding

1429_T12,T32	chr2	2.2E+07	2.2E+07	4999	+	**2**	**25**	NA	intron (NM_001004761, intron 6 of 10)	Lx|LINE|L1	392433	NM_001004761	241263	Mm.166647	NM_175706	ENSMUSG00000045967	**Gpr158**	5330427M13Rik|mKIAA1136	G protein-coupled receptor 158	Amp	protein-coding
195_T3,T4	chr1	1.6E+08	1.6E+08	3999	+	**2**	**25**	NA	intron (NM_013862, intron 13 of 20)	intron (NM_013862, intron 13 of 20)	-10747	NM_001146330	620246	Mm.478780	NM_001146330		**Gpr52**	EG620246	G protein-coupled receptor 52	Amp	protein-coding
3065_T22,T3	chr6	6.4E+07	6.4E+07	999	+	**2**	**25**	NA	intron (NM_008167, intron 2 of 15)	intron (NM_008167, intron 2 of 15)	248143	NM_008167	14804	Mm.439651	NM_008167	ENSMUSG00000071424	**Grid2**	B230104L07Rik|GluD2|GluRdelta2|Lc|Lc<J>|MMS10-AC|Ms10ac|cpr|ho|nmf408|tpr	glutamate receptor, ionotropic, delta 2	Amp	protein-coding
272_T12,T2	chr10	5E+07	5E+07	4999	+	**2**	**25**	NA	intron (NM_010349, intron 7 of 17)	intron (NM_010349, intron 7 of 17)	269754	NM_010349	14806	Mm.332838	NM_010349	ENSMUSG00000056073	**Grik2**	AW124492|GluK2|Glur-6|Glur6|Glurbeta2	glutamate receptor, ionotropic, kainate 2 (beta 2)	Amp	protein-coding
3112_T32,T3	chr6	1.1E+08	1.1E+08	7999	+	**2**	**25**	NA	Intergenic	Intergenic	-849098	NM_177328	108073	Mm.240881	NM_177328	ENSMUSG00000056755	**Grm7**	6330570A01Rik|BB176677|C030018L03|E130018M02Rik|Gpr1g|Gprc1g|SMN2|Tg(SMN2)89Ahmb|mGluR7	glutamate receptor, metabotropic 7	Amp	protein-coding
3678_T12,T42	chr9	3895501	3896500	999	+	**2**	**25**	NA	3' UTR (NM_001033322, exon 8 of 8)	3' UTR (NM_001033322, exon 8 of 8)	363651	NM_001033322	234889	Mm.91240	NM_001033322	ENSMUSG00000041624	**Gucy1a2**	6330407I18Rik|A230060L24Rik	guanylate cyclase 1, soluble, alpha 2	Amp	protein-coding
1625_T1,T4	chr2	1.5E+08	1.5E+08	999	+	**2**	**25**	NA	Intergenic	Intergenic	-9468	NM_001172117	15162	Mm.715	NM_010407	ENSMUSG00000003283	**Hck**	AI849071|Bmk	hemopoietic cell kinase	Amp	protein-coding
1639_T12,T4	chr3	3890501	3891500	999	+	**2**	**25**	NA	Intergenic	Lx7|LINE|L1	382970	NM_013920	30942	Mm.330897	NM_013920	ENSMUSG00000017688	**Hnf4g**	NR2A2	hepatocyte nuclear factor 4, gamma	Amp	protein-coding
2738_T32,T4	chr5	4E+07	4E+07	999	+	**2**	**25**	NA	Intergenic	Intergenic	-747369	NM_010474	15476	Mm.12559	NM_010474	ENSMUSG00000051022	**Hs3st1**	3-Ost|D5Wsu110e|Hsg3ost	heparan sulfate (glucosamine) 3-O-sulfotransferase 1	Amp	protein-coding
2741_T12,T2	chr5	4.1E+07	4.1E+07	999	+	**2**	**25**	NA	Intergenic	Intergenic	-987369	NM_010474	15476	Mm.12559	NM_010474	ENSMUSG00000051022	**Hs3st1**	3-Ost|D5Wsu110e|Hsg3ost	heparan sulfate (glucosamine) 3-O-sulfotransferase 1	Amp	protein-coding
589_T22,T3	chr13	1.1E+08	1.1E+08	999	+	**2**	**25**	NA	Intergenic	Intergenic	244307	NM_008308	15550	Mm.4716	NM_008308	ENSMUSG00000021721	**Htr1a**	Gpcr18	5-hydroxytryptamine (serotonin) receptor 1A	Amp	protein-coding
590_T2,T3	chr13	1.1E+08	1.1E+08	4999	+	**2**	**25**	NA	Intergenic	Intergenic	562307	NM_008308	15550	Mm.4716	NM_008308	ENSMUSG00000021721	**Htr1a**	Gpcr18	5-hydroxytryptamine (serotonin) receptor 1A	Amp	protein-coding

2357_T32,T4	chr3	5.9E+07	5.9E+07	6999	+	**2**	**25**	NA	Intergenic	Intergenic	-37744	NM_001162884	242050	Mm.228086	NM_001162884	ENSMUSG00000036334	**Igsf10**	6530405F15Rik|9030224D03|AA409708|AA536958|AI414626|Adlican2|CMF608	immunoglobulin superfamily, member 10	Amp	protein-coding
3600_T1,T4	chr8	8.2E+07	8.2E+07	999	+	**2**	**25**	NA	Intergenic	L1_Mus4|LINE|L1	239586	NM_001254747	16168	Mm.4392	NM_008357	ENSMUSG00000031712	**Il15**	AI503618|IL-15	interleukin 15	Amp	protein-coding
497_T32,T42	chr13	1.6E+07	1.6E+07	999	+	**2**	**25**	NA	Intergenic	Lx8|LINE|L1	445525	NM_008380	16323	Mm.8042	NM_008380	ENSMUSG00000041324	**Inhba**	-	inhibin beta-A	Amp	protein-coding
3096_T12,T32	chr6	9.5E+07	9.5E+07	4999	+	**2**	**25**	NA	Intergenic	L1Md_F2|LINE|L1	-84880	NM_001102670	243574	Mm.333275	NM_001008785	ENSMUSG00000030031	**Kbtbd8**	AU040152|SSEC-51|SSEC51|Takrp|mKIAA1842	kelch repeat and BTB (POZ) domain containing 8	Amp	protein-coding
1584_T1,T4	chr2	1.1E+08	1.1E+08	2999	+	**2**	**25**	NA	Intergenic	Intergenic	869411	NM_021275	16492	Mm.142718	NM_021275	ENSMUSG00000042604	**Kcna4**	Kv1.4	potassium voltage-gated channel, shaker-related subfamily, member 4	Amp	protein-coding
2762_T12,T22	chr5	4.9E+07	4.9E+07	3999	+	**2**	**25**	NA	Intergenic	Intergenic	-105841	NM_001199244	80334	Mm.160172	NM_030265	ENSMUSG00000029088	**Kcnip4**	AV032399|Calp|Calp250|KChIP4|KChIP4a	Kv channel interacting protein 4	Amp	protein-coding
2759_T2,T32	chr5	4.9E+07	4.9E+07	4999	+	**2**	**25**	NA	intron (NM_001199244, intron 1 of 7)	intron (NM_001199244, intron 1 of 7)	5557	NM_030265	80334	Mm.160172	NM_030265	ENSMUSG00000029088	**Kcnip4**	AV032399|Calp|Calp250|KChIP4|KChIP4a	Kv channel interacting protein 4	Amp	protein-coding
1495_T1,T32	chr2	5.5E+07	5.5E+07	999	+	**2**	**25**	NA	intron (NM_008426, intron 3 of 3)	intron (NM_008426, intron 3 of 3)	22030	NM_008426	16519	Mm.5127	NM_008426	ENSMUSG00000026824	**Kcnj3**	GIRK-1|GIRK1|Kcnf3|Kir3.1	potassium inwardly-rectifying channel, subfamily J, member 3	Amp	protein-coding
1157_T2,T3	chr15	7.3E+07	7.3E+07	3999	+	**2**	**25**	NA	intron (NM_001033876, intron 1 of 1)	intron (NM_001033876, intron 1 of 1)	4779	NM_001033876	223604	Mm.386856	NM_001033876	ENSMUSG00000036760	**Kcnk9**	Task3	potassium channel, subfamily K, member 9	Amp	protein-coding
985_T32,T4	chr15	4.5E+07	4.5E+07	999	+	**2**	**25**	NA	Intergenic	Intergenic	-123066	NM_026200	67498	Mm.300079	NM_026200	ENSMUSG00000022342	**Kcnv1**	2700023A03Rik|vibe	potassium channel, subfamily V, member 1	Amp	protein-coding
990_T1,T2	chr15	4.6E+07	4.6E+07	999	+	**2**	**25**	NA	Intergenic	RMER5|LTR|ERV1	-387066	NM_026200	67498	Mm.300079	NM_026200	ENSMUSG00000022342	**Kcnv1**	2700023A03Rik|vibe	potassium channel, subfamily V, member 1	Amp	protein-coding
2827_T22,T2	chr5	6.9E+07	6.9E+07	3999	+	**2**	**25**	NA	intron (NM_175519, intron 1 of 1)	intron (NM_175519, intron 1 of 1)	144209	NM_175519	243043	Mm.234821	NM_175519	ENSMUSG00000037653	**Kctd8**	A730087N02Rik	potassium channel tetramerisation domain containing 8	Amp	protein-coding
403_T22,T4	chr11	9.2E+07	9.2E+07	999	+	**2**	**25**	NA	Intergenic	Intergenic	-502445	NM_028547	73470	Mm.67677	NM_028547	ENSMUSG00000046755	**Kif2b**	1700063D03Rik	kinesin family member 2B	Amp	protein-coding
776_T3,T42	chr14	9.7E+07	9.7E+07	999	+	**2**	**25**	NA	Intergenic	L1_Mur1|LINE|L1	-290966	NM_053105	93688	Mm.308735	NM_053105	ENSMUSG00000022076	**Klhl1**	mKIAA1490	kelch-like 1	Amp	protein-coding

778_T22,T3	chr14	9.7E+07	9.7E+07	999	+	**2**	**25**	NA	Intergenic	Lx7|LINE|L1	-297966	NM_053105	93688	Mm.308735	NM_053105	ENSMUSG00000022076	**Klhl1**	mKIAA1490	kelch-like 1	Amp	protein-coding
765_T1,T2	chr14	9.6E+07	9.6E+07	999	+	**2**	**25**	NA	intron (NM_053105, intron 1 of 10)	intron (NM_053105, intron 1 of 10)	134034	NM_053105	93688	Mm.308735	NM_053105	ENSMUSG00000022076	**Klhl1**	mKIAA1490	kelch-like 1	Amp	protein-coding
768_T1,T2	chr14	9.6E+07	9.6E+07	4999	+	**2**	**25**	NA	intron (NM_053105, intron 1 of 10)	L1Md_F2|LINE|L1	117034	NM_053105	93688	Mm.308735	NM_053105	ENSMUSG00000022076	**Klhl1**	mKIAA1490	kelch-like 1	Amp	protein-coding
772_T12,T32	chr14	9.6E+07	9.6E+07	999	+	**2**	**25**	NA	intron (NM_053105, intron 1 of 10)	intron (NM_053105, intron 1 of 10)	91034	NM_053105	93688	Mm.308735	NM_053105	ENSMUSG00000022076	**Klhl1**	mKIAA1490	kelch-like 1	Amp	protein-coding
3596_T32,T4	chr8	7.4E+07	7.4E+07	39999	+	**2**	**25**	NA	Intergenic	Intergenic	-664944	NM_010687	16795	Mm.324371	NM_010687	ENSMUSG00000004383	**Large1**	BPFD#36|Gyltl1a|Large|Mbp-1|Mbp1|enr|fg|froggy|myd	LARGE xylosyl- and glucuronyltransferase 1	Amp	protein-coding
2584_T3,T42	chr4	3.7E+07	3.7E+07	3999	+	**2**	**25**	NA	Intergenic	Intergenic	-114756	NM_001165999	242384	Mm.132507	NM_175516	ENSMUSG00000045083	**Lingo2**	B230217C06Rik|Lern3|Lrrn6c	leucine rich repeat and Ig domain containing 2	Amp	protein-coding
396_T32,T42	chr11	6.1E+07	6.1E+07	7999	+	**2**	**25**	NA	Intergenic	MLTR31D_MM|LTR|ERVK	-65190	NM_001159405	16897	Mm.285453	NM_008502	ENSMUSG00000020536	**Llgl1**	AI325176|Lgl1|Llglh|Mgl1	lethal giant larvae homolog 1 (Drosophila)	Amp	protein-coding
399_T1,T42	chr11	6.1E+07	6.1E+07	1999	+	**2**	**25**	NA	Intergenic	MERVL-int|LTR|ERVL	-46190	NM_001159405	16897	Mm.285453	NM_008502	ENSMUSG00000020536	**Llgl1**	AI325176|Lgl1|Llglh|Mgl1	lethal giant larvae homolog 1 (Drosophila)	Amp	protein-coding
2451_T22,T2	chr3	1.1E+08	1.1E+08	999	+	**2**	**25**	NA	intron (NM_028081, intron 2 of 2)	intron (NM_028081, intron 2 of 2)	13318	NM_001286685	321000	Mm.259638	NM_028081	ENSMUSG00000056260	**Lrif1**	2010012G17Rik|4933421E11Rik|AI450568	ligand dependent nuclear receptor interacting factor 1	Amp	protein-coding
1539_T3,T4	chr2	9.6E+07	9.6E+07	3999	+	**2**	**25**	NA	Intergenic	Intergenic	-372669	NM_001289742	241568	Mm.241682	NM_178725	ENSMUSG00000050587	**Lrrc4c**	6430556C10Rik|NGL-1	leucine rich repeat containing 4C	Amp	protein-coding
1562_T3,T4	chr2	9.8E+07	9.8E+07	3999	+	**2**	**25**	NA	Intergenic	Intergenic	318843	NM_178725	241568	Mm.241682	NM_178725	ENSMUSG00000050587	**Lrrc4c**	6430556C10Rik|NGL-1	leucine rich repeat containing 4C	Amp	protein-coding
1564_T3,T4	chr2	9.8E+07	9.8E+07	999	+	**2**	**25**	NA	Intergenic	Intergenic	656343	NM_178725	241568	Mm.241682	NM_178725	ENSMUSG00000050587	**Lrrc4c**	6430556C10Rik|NGL-1	leucine rich repeat containing 4C	Amp	protein-coding
1544_T42,T4	chr2	9.7E+07	9.7E+07	999	+	**2**	**25**	NA	intron (NM_001289742, intron 3 of 7)	intron (NM_001289742, intron 3 of 7)	-386657	NM_001289744	241568	Mm.241682	NM_178725	ENSMUSG00000050587	**Lrrc4c**	6430556C10Rik|NGL-1	leucine rich repeat containing 4C	Amp	protein-coding
1546_T2,T32	chr2	9.7E+07	9.7E+07	999	+	**2**	**25**	NA	intron (NM_001289742, intron 5 of 7)	intron (NM_001289742, intron 5 of 7)	-131657	NM_001289744	241568	Mm.241682	NM_178725	ENSMUSG00000050587	**Lrrc4c**	6430556C10Rik|NGL-1	leucine rich repeat containing 4C	Amp	protein-coding

1548_T22,T32	chr2	9.7E+07	9.7E+07	999	+	**2**	**25**	NA	intron (NM_001289742, intron 5 of 7)	intron (NM_001289742, intron 5 of 7)	-127657	NM_001289744	241568	Mm.241682	NM_178725	ENSMUSG00000050587	**Lrrc4c**	6430556C10Rik|NGL-1	leucine rich repeat containing 4C	Amp	protein-coding
1553_T12,T3	chr2	9.8E+07	9.8E+07	999	+	**2**	**25**	NA	intron (NM_001289742, intron 7 of 7)	intron (NM_001289742, intron 7 of 7)	138343	NM_178725	241568	Mm.241682	NM_178725	ENSMUSG00000050587	**Lrrc4c**	6430556C10Rik|NGL-1	leucine rich repeat containing 4C	Amp	protein-coding
1558_T12,T32	chr2	9.8E+07	9.8E+07	999	+	**2**	**25**	NA	intron (NM_001289742, intron 7 of 7)	intron (NM_001289742, intron 7 of 7)	150343	NM_178725	241568	Mm.241682	NM_178725	ENSMUSG00000050587	**Lrrc4c**	6430556C10Rik|NGL-1	leucine rich repeat containing 4C	Amp	protein-coding
427_T12,T22	chr12	4.3E+07	4.3E+07	4999	+	**2**	**25**	NA	Intergenic	Lx9|LINE|L1	-1E+06	NM_010733	16981	Mm.404786	NM_010733	ENSMUSG00000036295	**Lrrn3**	NLRR-3	leucine rich repeat protein 3, neuronal	Amp	protein-coding
3085_T12,T32	chr6	8.1E+07	8.1E+07	1999	+	**2**	**25**	NA	intron (NM_001134743, intron 2 of 2)	intron (NM_001134743, intron 2 of 2)	716857	NM_178731	243499	Mm.483425	NM_178731	ENSMUSG00000052581	**Lrrtm4**	7530419J18Rik|A230052N11	leucine rich repeat transmembrane neuronal 4	Amp	protein-coding
2978_T12,T1	chr6	1.9E+07	1.9E+07	999	+	**2**	**25**	NA	promoter-TSS (NM_133939)	promoter-TSS (NM_133939)	-635	NM_133939	76522	Mm.275158	NM_133939	ENSMUSG00000044155	**Lsm8**	2010003I05Rik|AW214405|Naa38	LSM8 homolog, U6 small nuclear RNA associated	Amp	protein-coding
2975_T12,T1	chr6	1.9E+07	1.9E+07	999	+	**2**	**25**	NA	Intergenic	RLTR6_Mm|LTR|ERV1	-5635	NM_133939	76522	Mm.275158	NM_133939	ENSMUSG00000044155	**Lsm8**	2010003I05Rik|AW214405|Naa38	LSM8 homolog, U6 small nuclear RNA associated	Amp	protein-coding
2242_T22,T2	chr3	5.6E+07	5.6E+07	999	+	**2**	**25**	NA	intron (NM_030595, intron 41 of 57)	intron (NM_030595, intron 41 of 57)	-13510	NM_010750	17116	Mm.440209	NM_010750	ENSMUSG00000056947	**Mab21l1**	AW047968	mab-21-like 1 (C. elegans)	Amp	protein-coding
1607_T12,T3	chr2	1.4E+08	1.4E+08	2999	+	**2**	**25**	NA	intron (NM_001013802, intron 2 of 18)	L1MB4|LINE|L1	16565	NM_028387	72899	Mm.229466	NM_028387	ENSMUSG00000068205	**Macrod2**	1110033L15Rik|2610107G07Rik|2900006F19Rik	MACRO domain containing 2	Amp	protein-coding
2727_T12,T1	chr5	2E+07	2E+07	999	+	**2**	**25**	NA	intron (NM_001170746, intron 1 of 22)	intron (NM_001170746, intron 1 of 22)	330954	NM_001170746	50791	Mm.332231	NM_015823	ENSMUSG00000040003	**Magi2**	AIP-1|Acvri1|Acvrinp1|Acvrip1|Magi-2|S-SCAM|mKIAA0705	membrane associated guanylate kinase, WW and PDZ domain containing 2	Amp	protein-coding
2730_T2,T32	chr5	2E+07	2E+07	999	+	**2**	**25**	NA	intron (NM_001170746, intron 1 of 22)	intron (NM_001170746, intron 1 of 22)	-296518	NM_001170745	50791	Mm.332231	NM_015823	ENSMUSG00000040003	**Magi2**	AIP-1|Acvri1|Acvrinp1|Acvrip1|Magi-2|S-SCAM|mKIAA0705	membrane associated guanylate kinase, WW and PDZ domain containing 2	Amp	protein-coding

2732_T2,T42	chr5	2E+07	2E+07	999	+	**2**	**25**	NA	intron (NM_001170746, intron 1 of 22)	intron (NM_001170746, intron 1 of 22)	-292518	NM_001170745	50791	Mm.332231	NM_015823	ENSMUSG00000040003	**Magi2**	AIP-1|Acvri1|Acvrip1|Acvrip1|Magi-2|S-SCAM|mKIAA0705	membrane associated guanylate kinase, WW and PDZ domain containing 2	Amp	protein-coding
2192_T1,T3	chr3	5.2E+07	5.2E+07	1999	+	**2**	**25**	NA	intron (NM_001004176, intron 1 of 4)	intron (NM_001004176, intron 1 of 4)	168506	NM_001004176	433586	Mm.234972	NM_001004176	ENSMUSG00000061143	**Maml3**	AV234550|BC049812|Mam-2|mKIAA1816	mastermind like 3 (Drosophila)	Amp	protein-coding
3586_T2,T32	chr8	7.1E+07	7.1E+07	7999	+	**2**	**25**	NA	intron (NM_173013, intron 5 of 6)	PB1D7|SINE|Alu	9526	NM_173013	270058	Mm.248559	NM_173013	ENSMUSG00000019261	**Map1s**	6430517J16Rik|Bpy2ip1|Map8|Mtap1s|VCY2IP1	microtubule-associated protein 1S	Amp	protein-coding
2365_T1,T32	chr3	6.1E+07	6.1E+07	7999	+	**2**	**25**	NA	Intergenic	Intergenic	149321	NM_001253708	56758	Mm.255723	NM_020007	ENSMUSG00000027763	**Mbnl1**	Mbnl|mKIAA0428	muscleblind-like 1 (Drosophila)	Amp	protein-coding
2614_T32,T3	chr4	7.1E+07	7.1E+07	4999	+	**2**	**25**	NA	Intergenic	RCHARR1|DNA|hAT-Charlie	-820072	NM_172694	230316	Mm.251188	NM_172694	ENSMUSG00000039270	**Megf9**	4933405H16Rik|9630025C07|Egfl5|mKIAA0818	multiple EGF-like-domains 9	Amp	protein-coding
1586_T2,T42	chr2	1.1E+08	1.1E+08	3999	+	**2**	**25**	NA	Intergenic	Intergenic	209790	NM_029790	76894	Mm.330451	NM_029790	ENSMUSG00000057234	**Mettl15**	0610027B03Rik|AI303697|AW049952|Mett5d1	methyltransferase like 15	Amp	protein-coding
329_T2,T42	chr10	1E+08	1E+08	2999	+	**2**	**25**	NA	Intergenic	Intergenic	-518487	NM_001205098	67569	Mm.25252	NM_026243	ENSMUSG00000019888	**Mgat4c**	9130411I17Rik|GntIVh	MGAT4 family, member C	Amp	protein-coding
3458_T22,T4	chr8	3.8E+07	3.8E+07	4999	+	**2**	**25**	NA	intron (NM_145841, intron 2 of 7)	intron (NM_145841, intron 2 of 7)	336202	NR_029884	723860		NR_029884	ENSMUSG00000065457	**Mir383**	Mirn383|mir-383|mmu-mir-383	microRNA 383	Amp	ncRNA
2579_T42,T4	chr4	3.6E+07	3.6E+07	1999	+	**2**	**25**	NA	intron (NM_001166000, intron 1 of 8)	intron (NM_001166000, intron 1 of 8)	177953	NR_030545	1E+08		NR_030545	ENSMUSG00000077851	**Mir876**	Mirn876|mir-876|mmu-mir-876	microRNA 876	Amp	ncRNA
2368_T3,T4	chr3	6.7E+07	6.7E+07	4999	+	**2**	**25**	NA	intron (NM_025822, intron 5 of 9)	intron (NM_025822, intron 5 of 9)	-184097	NM_010801	17349	Mm.10414	NM_010801	ENSMUSG00000048416	**Mlf1**	HLS7	myeloid leukemia factor 1	Amp	protein-coding
2531_T2,T42	chr4	1.8E+07	1.8E+07	999	+	**2**	**25**	NA	Intergenic	B3A|SINE|B2	-216482	NM_019724	17389	Mm.187315	NM_019724	ENSMUSG00000028226	**Mmp16**	MT-MMP 3|MT3-MMP|Mt3mmp	matrix metallopeptidase 16	Amp	protein-coding
3704_T12,T22	chr9	7431501	7435500	3999	+	**2**	**25**	NA	Intergenic	L1_Mus1|LINE|L1	-12322	NM_010809	17392	Mm.4993	NM_010809	ENSMUSG00000043613	**Mmp3**	EMS-2|MMP-3|SL-1|SLN-1|SLN1|STR-1|Stmy1|Str1	matrix metallopeptidase 3	Amp	protein-coding
3041_T12,T1	chr6	3.6E+07	3.6E+07	4999	+	**2**	**25**	NA	intron (NM_008098, intron 1 of 3)	intron (NM_008098, intron 1 of 3)	14888	NM_008098	14489	Mm.182746	NM_008098	ENSMUSG00000029840	**Mtpn**	5033418D15Rik|Gcdp|V1	myotrophin	Amp	protein-coding

1092_T2,T3	chr15	6.2E+07	6.2E+07	267999	+	**2**	**25**	NA	Intergenic	Lx7|LINE|L1	-11841	NM_001177354	17869	Mm.2444	NM_010849	ENSMUSG00000022346	**Myc**	AU016757|Myc2|Niard|Nird|bHLHe39	myelocytomatosis oncogene	Amp	protein-coding
2278_T2,T42	chr3	5.7E+07	5.7E+07	6999	+	**2**	**25**	NA	Intergenic	L1Md_T|LINE|L1	-333299	NM_030595	26422	Mm.384353	NM_030595	ENSMUSG00000027799	**Nbea**	Lyst2|mKIAA1544	neurobeachin	Amp	protein-coding
2255_T2,T32	chr3	5.6E+07	5.6E+07	6999	+	**2**	**25**	NA	intron (NM_030595, intron 18 of 57)	intron (NM_030595, intron 18 of 57)	166701	NM_030595	26422	Mm.384353	NM_030595	ENSMUSG00000027799	**Nbea**	Lyst2|mKIAA1544	neurobeachin	Amp	protein-coding
2483_T32,T4	chr3	1.3E+08	1.3E+08	7999	+	**2**	**25**	NA	Intergenic	Intergenic	-232591	NM_022565	64580	Mm.444289	NM_022565	ENSMUSG00000027971	**Ndst4**	4930439H17Rik	N-deacetylase/N-sulfotransferase (heparin glucosaminyl) 4	Amp	protein-coding
2485_T2,T32	chr3	1.3E+08	1.3E+08	3999	+	**2**	**25**	NA	Intergenic	Intergenic	-67591	NM_022565	64580	Mm.444289	NM_022565	ENSMUSG00000027971	**Ndst4**	4930439H17Rik	N-deacetylase/N-sulfotransferase (heparin glucosaminyl) 4	Amp	protein-coding
2548_T32,T3	chr4	2.1E+07	2.1E+07	4999	+	**2**	**25**	NA	intron (NM_172987, intron 1 of 4)	MERVL_2A-int|LTR|ERVL	87668	NM_001290410	269513	Mm.317473	NM_172987	ENSMUSG00000055761	**Nkain3**	C530048M05|E130310K16Rik	Na+/K+ transporting ATPase interacting 3	Amp	protein-coding
1727_T3,T4	chr3	2.5E+07	2.5E+07	999	+	**2**	**25**	NA	Intergenic	Intergenic	1624307	NM_001163387	192167	Mm.316080	NM_138666	ENSMUSG00000063887	**Nlgn1**	6330415N05Rik|BB179718|NL1|Nlg1|mKIAA1070	neuroligin 1	Amp	protein-coding
2173_T2,T3	chr3	5.1E+07	5.1E+07	9999	+	**2**	**25**	NA	Intergenic	Intergenic	-123947	NM_009834	12457	Mm.86541	NM_009834	ENSMUSG00000023087	**Noct**	AU043840|Ccr4|Ccrn4l	nocturnin	Amp	protein-coding
437_T2,T32	chr12	4.8E+07	4.8E+07	999	+	**2**	**25**	NA	Intergenic	MLTR14|LTR|ERV1	-739225	NM_021361	664883	Mm.247195	NM_021361	ENSMUSG00000021047	**Nova1**	9430099M15Rik|G630039L02|Nova-1	neuro-oncological ventral antigen 1	Amp	protein-coding
1371_T22,T3	chr19	5.6E+07	5.6E+07	999	+	**2**	**25**	NA	intron (NM_001286552, intron 12 of 39)	intron (NM_001286552, intron 12 of 39)	19038	NM_198059	18175	Mm.386769	NM_008733	ENSMUSG00000049134	**Nrap**	-	nebulin-related anchoring protein	Amp	protein-coding
1278_T32,T42	chr17	5.9E+07	5.9E+07	1999	+	**2**	**25**	NA	Intergenic	L1MA6|LINE|L1	-394178	NM_026497	67993	Mm.36507	NM_026497	ENSMUSG00000024228	**Nudt12**	0610016O18Rik	nudix (nucleoside diphosphate linked moiety X)-type motif 12	Amp	protein-coding
1282_T12,T4	chr17	6E+07	6E+07	3999	+	**2**	**25**	NA	Intergenic	Intergenic	-821178	NM_026497	67993	Mm.36507	NM_026497	ENSMUSG00000024228	**Nudt12**	0610016O18Rik	nudix (nucleoside diphosphate linked moiety X)-type motif 12	Amp	protein-coding
1285_T22,T32	chr17	6E+07	6E+07	1999	+	**2**	**25**	NA	Intergenic	Intergenic	-1E+06	NM_026497	67993	Mm.36507	NM_026497	ENSMUSG00000024228	**Nudt12**	0610016O18Rik	nudix (nucleoside diphosphate linked moiety X)-type motif 12	Amp	protein-coding

2890_T2,T32	chr6	9906501	9908500	1999	+	**2**	**25**	NA	Intergenic	T-rich|Low_complexity|Low_complexity	957481	NM_008751	18231	Mm.46954	NM_008751	ENSMUSG00000046178	**Nxph1**	C130005L03Rik	neurexophilin 1	Amp	protein-coding
50_T1,T22	chr1	8.1E+07	8.1E+07	999	+	**2**	**25**	NA	Intergenic	Intergenic	-120317	NM_172849	241134	Mm.313904	NM_172849	ENSMUSG00000054976	**Nyap2**	9430031J16Rik|Jr6|Kiaa1486	neuronal tyrosine-phophorylated phosphoinositide 3-kinase adaptor 2	Amp	protein-coding
630_T3,T4	chr14	8.1E+07	8.1E+07	999	+	**2**	**25**	NA	Intergenic	Lx9|LINE|L1	936698	NM_001030294	380924	Mm.26456	NM_001030294	ENSMUSG00000022026	**Olfm4**	GC1|GW112|Gm296|Gm913|OlfD|pPD4	olfactomedin 4	Amp	protein-coding
637_T2,T3	chr14	8.2E+07	8.2E+07	999	+	**2**	**25**	NA	Intergenic	Intergenic	1830698	NM_001030294	380924	Mm.26456	NM_001030294	ENSMUSG00000022026	**Olfm4**	GC1|GW112|Gm296|Gm913|OlfD|pPD4	olfactomedin 4	Amp	protein-coding
639_T2,T3	chr14	8.2E+07	8.2E+07	999	+	**2**	**25**	NA	Intergenic	Lx5c|LINE|L1	1835698	NM_001030294	380924	Mm.26456	NM_001030294	ENSMUSG00000022026	**Olfm4**	GC1|GW112|Gm296|Gm913|OlfD|pPD4	olfactomedin 4	Amp	protein-coding
2370_T3,T4	chr3	7E+07	7E+07	3999	+	**2**	**25**	NA	Intergenic	MER5A1|DNA|hAT-Charlie	264887	NM_001018031	229389	Mm.331071	NM_001018031	ENSMUSG00000027788	**Otol1**	Gm414	otolin 1	Amp	protein-coding
1951_T12,T3	chr3	4.7E+07	4.7E+07	999	+	**2**	**25**	NA	Intergenic	Intergenic	-195059	NM_001101479	241989	Mm.119234	NM_001101479	ENSMUSG00000090919	**Pabpc4l**	C330050A14Rik|EG241989	poly(A) binding protein, cytoplasmic 4-like	Amp	protein-coding
1953_T12,T32	chr3	4.7E+07	4.7E+07	1999	+	**2**	**25**	NA	Intergenic	Intergenic	-198559	NM_001101479	241989	Mm.119234	NM_001101479	ENSMUSG00000090919	**Pabpc4l**	C330050A14Rik|EG241989	poly(A) binding protein, cytoplasmic 4-like	Amp	protein-coding
310_T1,T2	chr10	7.4E+07	7.4E+07	999	+	**2**	**25**	NA	intron (NM_001142735, intron 1 of 32)	intron (NM_001142735, intron 1 of 32)	13133	NM_001142735	11994	Mm.338933	NM_023115	ENSMUSG00000052613	**Pcdh15**	BB078305|ENSMUSG00000046980|Gm9815|Ush1f|av|nmf19|roda	protocadherin 15	Amp	protein-coding
312_T2,T3	chr10	7.4E+07	7.4E+07	999	+	**2**	**25**	NA	intron (NM_001142735, intron 1 of 32)	intron (NM_001142735, intron 1 of 32)	18133	NM_001142735	11994	Mm.338933	NM_023115	ENSMUSG00000052613	**Pcdh15**	BB078305|ENSMUSG00000046980|Gm9815|Ush1f|av|nmf19|roda	protocadherin 15	Amp	protein-coding
653_T3,T4	chr14	8.3E+07	8.3E+07	3999	+	**2**	**25**	NA	Intergenic	Lx5|LINE|L1	-962063	NM_001013753	219228	Mm.153643	NM_001013753	ENSMUSG00000035566	**Pcdh17**	C030033F14Rik|Gm78	protocadherin 17	Amp	protein-coding
656_T3,T4	chr14	8.3E+07	8.3E+07	999	+	**2**	**25**	NA	Intergenic	Lx6|LINE|L1	-952563	NM_001013753	219228	Mm.153643	NM_001013753	ENSMUSG00000035566	**Pcdh17**	C030033F14Rik|Gm78	protocadherin 17	Amp	protein-coding
658_T12,T32	chr14	8.4E+07	8.4E+07	999	+	**2**	**25**	NA	Intergenic	(TA)n|Simple_repeat|Simple_repeat	-703563	NM_001013753	219228	Mm.153643	NM_001013753	ENSMUSG00000035566	**Pcdh17**	C030033F14Rik|Gm78	protocadherin 17	Amp	protein-coding
2142_T32,T3	chr3	5E+07	5E+07	999	+	**2**	**25**	NA	Intergenic	L1Md_T|LINE|L1	-64684	NM_130448	73173	Mm.87246	NM_130448	ENSMUSG00000037892	**Pcdh18**	3110038E07Rik|BB095589|PCDH68L	protocadherin 18	Amp	protein-coding

718_T2,T3	chr14	9.2E+07	9.2E+07	999	+	**2**	**25**	NA	Intergenic	Intergenic	1781888	NM_001271800	211712	Mm.37126	NM_001081377	ENSMUSG00000055421	**Pcdh9**	-	protocadherin 9	Amp	protein-coding
720_T22,T3	chr14	9.2E+07	9.2E+07	1999	+	**2**	**25**	NA	Intergenic	Intergenic	1777388	NM_001271800	211712	Mm.37126	NM_001081377	ENSMUSG00000055421	**Pcdh9**	-	protocadherin 9	Amp	protein-coding
721_T22,T2	chr14	9.2E+07	9.2E+07	1999	+	**2**	**25**	NA	Intergenic	L1_Mur3|LINE|L1	1733388	NM_001271800	211712	Mm.37126	NM_001081377	ENSMUSG00000055421	**Pcdh9**	-	protocadherin 9	Amp	protein-coding
723_T22,T3	chr14	9.2E+07	9.2E+07	4999	+	**2**	**25**	NA	Intergenic	Intergenic	1682888	NM_001271800	211712	Mm.37126	NM_001081377	ENSMUSG00000055421	**Pcdh9**	-	protocadherin 9	Amp	protein-coding
750_T22,T3	chr14	9.4E+07	9.4E+07	3999	+	**2**	**25**	NA	Intergenic	Intergenic	-81831	NM_001271798	211712	Mm.37126	NM_001081377	ENSMUSG00000055421	**Pcdh9**	-	protocadherin 9	Amp	protein-coding
752_T1,T22	chr14	9.4E+07	9.4E+07	3999	+	**2**	**25**	NA	Intergenic	Intergenic	-182831	NM_001271798	211712	Mm.37126	NM_001081377	ENSMUSG00000055421	**Pcdh9**	-	protocadherin 9	Amp	protein-coding
754_T1,T22	chr14	9.4E+07	9.4E+07	3999	+	**2**	**25**	NA	Intergenic	Intergenic	-220831	NM_001271798	211712	Mm.37126	NM_001081377	ENSMUSG00000055421	**Pcdh9**	-	protocadherin 9	Amp	protein-coding
755_T32,T3	chr14	9.4E+07	9.4E+07	4999	+	**2**	**25**	NA	Intergenic	Intergenic	-281331	NM_001271798	211712	Mm.37126	NM_001081377	ENSMUSG00000055421	**Pcdh9**	-	protocadherin 9	Amp	protein-coding
738_T32,T3	chr14	9.4E+07	9.4E+07	999	+	**2**	**25**	NA	intron (NM_001081377, intron 1 of 3)	intron (NM_001081377, intron 1 of 3)	279888	NM_001271800	211712	Mm.37126	NM_001081377	ENSMUSG00000055421	**Pcdh9**	-	protocadherin 9	Amp	protein-coding
739_T22,T32	chr14	9.4E+07	9.4E+07	999	+	**2**	**25**	NA	intron (NM_001081377, intron 1 of 3)	ORR1C2|LTR|ERVL-MaLR	222888	NM_001271800	211712	Mm.37126	NM_001081377	ENSMUSG00000055421	**Pcdh9**	-	protocadherin 9	Amp	protein-coding
744_T1,T22	chr14	9.4E+07	9.4E+07	1999	+	**2**	**25**	NA	intron (NM_001081377, intron 1 of 3)	intron (NM_001081377, intron 1 of 3)	32388	NM_001271800	211712	Mm.37126	NM_001081377	ENSMUSG00000055421	**Pcdh9**	-	protocadherin 9	Amp	protein-coding
749_T12,T4	chr14	9.4E+07	9.4E+07	1999	+	**2**	**25**	NA	intron (NM_001081377, intron 1 of 3)	intron (NM_001081377, intron 1 of 3)	14388	NM_001271800	211712	Mm.37126	NM_001081377	ENSMUSG00000055421	**Pcdh9**	-	protocadherin 9	Amp	protein-coding
735_T3,T42	chr14	9.3E+07	9.3E+07	999	+	**2**	**25**	NA	intron (NM_001271798, intron 2 of 3)	(CA)n|Simple_repeat|Simple_repeat	469888	NM_001271800	211712	Mm.37126	NM_001081377	ENSMUSG00000055421	**Pcdh9**	-	protocadherin 9	Amp	protein-coding

730_T3,T4	chr14	9.3E+07	9.3E+07	999	+	**2**	**25**	NA	intron (NM_001271800, intron 2 of 2)	intron (NM_001271800, intron 2 of 2)	767888	NM_001271800	211712	Mm.37126	NM_001081377	ENSMUSG00000055421	**Pcdh9**	-	protocadherin 9	Amp	protein-coding
732_T3,T4	chr14	9.3E+07	9.3E+07	11999	+	**2**	**25**	NA	intron (NM_001271800, intron 2 of 2)	L1Md_F2|LINE|L1	757388	NM_001271800	211712	Mm.37126	NM_001081377	ENSMUSG00000055421	**Pcdh9**	-	protocadherin 9	Amp	protein-coding
2716_T12,T32	chr5	1.5E+07	1.5E+07	999	+	**2**	**25**	NA	Intergenic	Intergenic	-12918	NM_011995	26875	Mm.146275	NM_011995	ENSMUSG00000061601	**Pclo**	Acz|Pico	piccolo (presynaptic cytomatrix protein)	Amp	protein-coding
2505_T2,T3	chr3	1.4E+08	1.4E+08	7999	+	**2**	**25**	NA	Intergenic	Intergenic	105855	NM_008811	18598	Mm.4223	NM_008811	ENSMUSG00000047674	**Pdha2**	Pdhal	pyruvate dehydrogenase E1 alpha 2	Amp	protein-coding
2508_T1,T4	chr3	1.4E+08	1.4E+08	1999	+	**2**	**25**	NA	Intergenic	Intergenic	21855	NM_008811	18598	Mm.4223	NM_008811	ENSMUSG00000047674	**Pdha2**	Pdhal	pyruvate dehydrogenase E1 alpha 2	Amp	protein-coding
1652_T22,T3	chr3	5791501	5796500	4999	+	**2**	**25**	NA	Intergenic	Intergenic	-217752	NM_001163305	19302	Mm.132336	NM_008994	ENSMUSG00000040374	**Pex2**	D3Ertd138e|PAF-1|PMP35|Pxmp3	peroxisomal biogenesis factor 2	Amp	protein-coding
2438_T42,T4	chr3	9.2E+07	9.2E+07	999	+	**2**	**25**	NA	Intergenic	L1Md_F2|LINE|L1	-195583	NM_207247	242100	Mm.297406	NM_207247	ENSMUSG00000042244	**Pglyrp3**	Gm420	peptidoglycan recognition protein 3	Amp	protein-coding
979_T12,T3	chr15	4.4E+07	4.4E+07	999	+	**2**	**25**	NA	intron (NM_138674, intron 16 of 76)	intron (NM_138674, intron 16 of 76)	41447	NM_138674	192190	Mm.211494	NM_138674	ENSMUSG00000038725	**Pkhd1l1**	AB055648|PKHDL1	polycystic kidney and hepatic disease 1-like 1	Amp	protein-coding
193_T2,T3	chr1	1.5E+08	1.5E+08	6999	+	**2**	**25**	NA	Intergenic	Intergenic	512439	NM_001305632	18783	Mm.4186	NM_008869	ENSMUSG00000056220	**Pla2g4a**	Pla2g4|cPLA2|cPLA2-alpha|cPLA2alpha	phospholipase A2, group IVA (cytosolic, calcium-dependent)	Amp	protein-coding
3343_T1,T32	chr7	3.8E+07	3.8E+07	2999	+	**2**	**25**	NA	Intergenic	L1_Mm|LINE|L1	-83652	NM_025390	66161	Mm.22284	NM_025390	ENSMUSG00000030423	**Pop4**	1110023P21Rik|Rpp29	processing of precursor 4, ribonuclease P/MRP family, (S. cerevisiae)	Amp	protein-coding
1436_T22,T4	chr2	4E+07	4E+07	999	+	**2**	**25**	NA	Intergenic	Intergenic	-1E+06	NM_024209	67857	Mm.389242	NM_024209	ENSMUSG00000026753	**Ppp6c**	2310003C10Rik	protein phosphatase 6, catalytic subunit	Amp	protein-coding
1438_T32,T4	chr2	4E+07	4E+07	999	+	**2**	**25**	NA	Intergenic	L1_Mus3|LINE|L1	-1E+06	NM_024209	67857	Mm.389242	NM_024209	ENSMUSG00000026753	**Ppp6c**	2310003C10Rik	protein phosphatase 6, catalytic subunit	Amp	protein-coding
3215_T1,T22	chr6	1.3E+08	1.3E+08	3999	+	**2**	**25**	NA	Intergenic	Intergenic	-79342	NM_011174	19131	Mm.425348	NM_011174	ENSMUSG00000059934	**Prh1**	MP2|Prp	proline rich protein HaeIII subfamily 1	Amp	protein-coding
2455_T1,T32	chr3	1.1E+08	1.1E+08	2999	+	**2**	**25**	NA	Intergenic	Lx8|LINE|L1	-323001	NR_024139	99890	Mm.36115	NM_178891	ENSMUSG00000049300	**Prmt6**	AW124876|BB233495|Hrmt1l6	protein arginine N-methyltransferase 6	Amp	protein-coding

2461_T12,T22	chr3	1.1E+08	1.1E+08	999	+	**2**	**25**	NA	Intergenic	ID_B1|SINE|B4	-504001	NR_024139	99890	Mm.36115	NM_178891	ENSMUSG00000049300	**Prmt6**	AW124876|BB233495|Hrmt1l6	protein arginine N-methyltransferase 6	Amp	protein-coding
2463_T1,T42	chr3	1.1E+08	1.1E+08	3999	+	**2**	**25**	NA	Intergenic	Intergenic	-589501	NR_024139	99890	Mm.36115	NM_178891	ENSMUSG00000049300	**Prmt6**	AW124876|BB233495|Hrmt1l6	protein arginine N-methyltransferase 6	Amp	protein-coding
2628_T2,T3	chr4	7.8E+07	7.8E+07	4999	+	**2**	**25**	NA	intron (NM_011211, intron 1 of 39)	intron (NM_011211, intron 1 of 39)	23895	NM_011211	19266	Mm.184021	NM_011211	ENSMUSG00000028399	**Ptprd**	1110002J03Rik|3000002J10Rik|B230219D21Rik|R-PTP-delta	protein tyrosine phosphatase, receptor type, D	Amp	protein-coding
1404_T42,T4	chr19	6E+07	6E+07	6999	+	**2**	**25**	NA	Intergenic	L1Md_A|LINE|L1	166364	NM_001033172	74998	Mm.24167	NM_001033172	ENSMUSG00000040022	**Rab11fip2**	4930470G04Rik|A830046J09Rik|AW558126|Nrip11	RAB11 family interacting protein 2 (class I)	Amp	protein-coding
1406_T42,T4	chr19	6E+07	6E+07	6999	+	**2**	**25**	NA	Intergenic	L1Md_A|LINE|L1	126364	NM_001033172	74998	Mm.24167	NM_001033172	ENSMUSG00000040022	**Rab11fip2**	4930470G04Rik|A830046J09Rik|AW558126|Nrip11	RAB11 family interacting protein 2 (class I)	Amp	protein-coding
1408_T42,T4	chr19	6E+07	6E+07	5999	+	**2**	**25**	NA	Intergenic	L1Md_T|LINE|L1	-77846	NM_001164367	74998	Mm.24167	NM_001033172	ENSMUSG00000040022	**Rab11fip2**	4930470G04Rik|A830046J09Rik|AW558126|Nrip11	RAB11 family interacting protein 2 (class I)	Amp	protein-coding
1576_T32,T3	chr2	1E+08	1E+08	1999	+	**2**	**25**	NA	Intergenic	Intergenic	-993248	NM_009020	19374	Mm.4988	NM_009020	ENSMUSG00000032864	**Rag2**	Rag-2	recombination activating gene 2	Amp	protein-coding
204_T12,T42	chr10	6311501	6312500	999	+	**2**	**25**	NA	Intergenic	(CA)n|Simple_repeat|Simple_repeat	-389600	NM_019958	56533	Mm.44606	NM_019958	ENSMUSG00000019775	**Rgs17**	6430507P11Rik|Rgsz2	regulator of G-protein signaling 17	Amp	protein-coding
207_T12,T22	chr10	6319501	6320500	999	+	**2**	**25**	NA	Intergenic	Lx8b|LINE|L1	-397600	NM_019958	56533	Mm.44606	NM_019958	ENSMUSG00000019775	**Rgs17**	6430507P11Rik|Rgsz2	regulator of G-protein signaling 17	Amp	protein-coding
177_T42,T4	chr1	1.5E+08	1.5E+08	999	+	**2**	**25**	NA	Intergenic	Intergenic	-731579	NM_022881	64214	Mm.253927	NM_022881	ENSMUSG00000026357	**Rgs18**	-	regulator of G-protein signaling 18	Amp	protein-coding
166_T1,T42	chr1	1.4E+08	1.4E+08	3999	+	**2**	**25**	NA	intron (NM_022881, intron 3 of 4)	intron (NM_022881, intron 3 of 4)	12921	NM_022881	64214	Mm.253927	NM_022881	ENSMUSG00000026357	**Rgs18**	-	regulator of G-protein signaling 18	Amp	protein-coding
221_T12,T4	chr10	3.2E+07	3.2E+07	999	+	**2**	**25**	NA	intron (NM_001013411, intron 3 of 6)	L1_Mus3|LINE|L1	-350275	NM_001146349	268291	Mm.295212	NM_001146349	ENSMUSG00000063760	**Rnf217**	AU016819|Ibrdc1	ring finger protein 217	Amp	protein-coding
227_T1,T32	chr10	3.2E+07	3.2E+07	4999	+	**2**	**25**	NA	intron (NM_001013411, intron 3 of 6)	intron (NM_001013411, intron 3 of 6)	-413275	NM_001146349	268291	Mm.295212	NM_001146349	ENSMUSG00000063760	**Rnf217**	AU016819|Ibrdc1	ring finger protein 217	Amp	protein-coding
2468_T1,T32	chr3	1.1E+08	1.1E+08	4999	+	**2**	**25**	NA	Intergenic	L2|LINE|L2	-192851	NM_001038696	67225	Mm.316928	NM_026043	ENSMUSG00000027981	**Rnpc3**	2810441O16Rik|AI447568|C030014B17Rik	RNA-binding region (RNP1, RRM) containing 3	Amp	protein-coding

1340_T1,T42	chr19	1.9E+07	1.9E+07	4999	+	**2**	**25**	NA	Intergenic	L1_Mur1|LINE|L1	-338804	NM_001043354	225998	Mm.234641	NM_146095	ENSMUSG00000036192	**Rorb**	Nr1f2|RZR-beta|RZRB|Rorbeta|hstp	RAR-related orphan receptor beta	Amp	protein-coding
2714_T1,T32	chr5	1.3E+07	1.3E+07	999	+	**2**	**25**	NA	Intergenic	Intergenic	-286784	NM_001243072	20346	Mm.372039	NM_009152	ENSMUSG00000028883	**Sema3a**	Hsema-I|SEMA1|SemD|Semad|coll-1	sema domain, immunoglobulin domain (Ig), short basic domain, secreted, (semaphorin) 3A	Amp	protein-coding
3481_T22,T4	chr8	3.9E+07	3.9E+07	999	+	**2**	**25**	NA	intron (NM_145841, intron 1 of 7)	intron (NM_145841, intron 1 of 7)	100508	NM_145841	244431	Mm.210388	NM_145841	ENSMUSG00000039539	**Sgcz**	C230085N17Rik	sarcoglycan zeta	Amp	protein-coding
3400_T2,T4	chr8	5522501	5526500	3999	+	**2**	**25**	NA	Intergenic	Intergenic	-419268	NM_011388	20494	Mm.3500	NM_011388	ENSMUSG00000023073	**Slc10a2**	9130221J18Rik|AI605518|ASBT|IBAT|ISBT	solute carrier family 10, member 2	Amp	protein-coding
3415_T2,T3	chr8	6830501	6831500	999	+	**2**	**25**	NA	Intergenic	Intergenic	-2E+06	NM_011388	20494	Mm.3500	NM_011388	ENSMUSG00000023073	**Slc10a2**	9130221J18Rik|AI605518|ASBT|IBAT|ISBT	solute carrier family 10, member 2	Amp	protein-coding
1501_T22,T42	chr2	6.2E+07	6.2E+07	3999	+	**2**	**25**	NA	intron (NM_033552, intron 2 of 25)	intron (NM_033552, intron 2 of 25)	111985	NM_033552	94229	Mm.314497	NM_033552	ENSMUSG00000026904	**Slc4a10**	NCBE|mKIAA4136	solute carrier family 4, sodium bicarbonate cotransporter-like, member 10	Amp	protein-coding
331_T32,T4	chr10	1E+08	1E+08	3999	+	**2**	**25**	NA	Intergenic	Intergenic	332692	NM_175328	103098	Mm.458408	NM_175328	ENSMUSG00000019894	**Slc6a15**	AA536730|AI326450|AI326451|v7-3	solute carrier family 6 (neurotransmitter transporter), member 15	Amp	protein-coding
2167_T22,T3	chr3	5.1E+07	5.1E+07	999	+	**2**	**25**	NA	Intergenic	ORR1E|LTR|ERVL-MaLR	-259387	NM_011990	26570	Mm.260988	NM_011990	ENSMUSG00000027737	**Slc7a11**	9930009M05Rik|AI451155|sut|xCT	solute carrier family 7 (cationic amino acid transporter, y+ system), member 11	Amp	protein-coding
807_T1,T32	chr14	1.1E+08	1.1E+08	10999	+	**2**	**25**	NA	Intergenic	L1VL4|LINE|L1	429239	NM_199065	76965	Mm.257268	NM_199065	ENSMUSG00000075478	**Slitrk1**	3200001I04Rik	SLIT and NTRK-like family, member 1	Amp	protein-coding
809_T22,T3	chr14	1.1E+08	1.1E+08	3999	+	**2**	**25**	NA	Intergenic	(CCAA)n|Simple_repeat|Simple_repeat	105739	NM_199065	76965	Mm.257268	NM_199065	ENSMUSG00000075478	**Slitrk1**	3200001I04Rik	SLIT and NTRK-like family, member 1	Amp	protein-coding
824_T3,T42	chr14	1.1E+08	1.1E+08	999	+	**2**	**25**	NA	Intergenic	Intergenic	-174761	NM_199065	76965	Mm.257268	NM_199065	ENSMUSG00000075478	**Slitrk1**	3200001I04Rik	SLIT and NTRK-like family, member 1	Amp	protein-coding
852_T22,T4	chr14	1.1E+08	1.1E+08	9999	+	**2**	**25**	NA	Intergenic	Intergenic	15385	NM_198865	75409	Mm.235097	NM_029273	ENSMUSG00000033214	**Slitrk5**	2610019D03Rik	SLIT and NTRK-like family, member 5	Amp	protein-coding
849_T42,T4	chr14	1.1E+08	1.1E+08	999	+	**2**	**25**	NA	Intergenic	Intergenic	-203851	NM_175499	239250	Mm.49728	NM_175499	ENSMUSG00000045871	**Slitrk6**	4832410J21Rik|Sltk6	SLIT and NTRK-like family, member 6	Amp	protein-coding

3362_T3,T42	chr7	6E+07	6E+07	1999	+	**2**	**25**	NA	Intergenic	L1_Mur1|LINE|L1	10931	NR_015456	52480	Mm.482346	NR_015456	ENSMUSG00000100826	**Snhg14**	AU018661|D7Ertd715e|Lncat	small nucleolar RNA host gene 14	Amp	ncRNA
248_T3,T42	chr10	4.6E+07	4.6E+07	3999	+	**2**	**25**	NA	Intergenic	Intergenic	165751	NR_028276	108637		NR_028276	ENSMUSG00000096263	**Snord14c**	Gm23107|Rnu14|U14	small nucleolar RNA, C/D box 14C	Amp	snoRNA
250_T22,T2	chr10	4.7E+07	4.7E+07	4999	+	**2**	**25**	NA	Intergenic	RMER15-int|LTR|ERVL	401251	NR_028276	108637		NR_028276	ENSMUSG00000096263	**Snord14c**	Gm23107|Rnu14|U14	small nucleolar RNA, C/D box 14C	Amp	snoRNA
253_T1,T42	chr10	4.7E+07	4.7E+07	999	+	**2**	**25**	NA	Intergenic	RMER15|LTR|ERVL	917251	NR_028276	108637		NR_028276	ENSMUSG00000096263	**Snord14c**	Gm23107|Rnu14|U14	small nucleolar RNA, C/D box 14C	Amp	snoRNA
448_T32,T4	chr12	6.2E+07	6.2E+07	999	+	**2**	**25**	NA	Intergenic	Intergenic	-225787	NR_131949	73309	Mm.276558	NR_131949		**Spanxn4**	1700047L15Rik	SPANX family, member N4	Amp	ncRNA
452_T22,T3	chr12	6.3E+07	6.3E+07	4999	+	**2**	**25**	NA	Intergenic	Intergenic	34213	NR_131949	73309	Mm.276558	NR_131949		**Spanxn4**	1700047L15Rik	SPANX family, member N4	Amp	ncRNA
1742_T1,T3	chr3	3.7E+07	3.7E+07	999	+	**2**	**25**	NA	intron (NM_001310473, intron 14 of 16)	intron (NM_001310473, intron 14 of 16)	76050	NM_021343	57815	Mm.172679	NM_021343	ENSMUSG00000027722	**Spata5**	2510048F20Rik|C78064|Spaf	spermatogenesis associated 5	Amp	protein-coding
1434_T12,T32	chr2	2.4E+07	2.4E+07	6999	+	**2**	**25**	NA	Intergenic	L1_Mus2|LINE|L1	-223896	NM_001165997	76857	Mm.276415	NM_029773	ENSMUSG00000026771	**Spopl**	4921517N04Rik|AU014935|BB233739|E430033K04Rik	speckle-type POZ protein-like	Amp	protein-coding
56_T32,T4	chr1	9.6E+07	9.6E+07	999	+	**2**	**25**	NA	intron (NM_001159745, intron 2 of 3)	intron (NM_001159745, intron 2 of 3)	11594	NM_009183	20452	Mm.306228	NM_009183	ENSMUSG00000040710	**St8sia4**	PST|PST-1|SIAT8-D|ST8SiaIV|Siat8d	ST8 alpha-N-acetyl-neuraminide alpha-2,8-sialyltransferase 4	Amp	protein-coding
1628_T22,T4	chr2	1.7E+08	1.7E+08	999	+	**2**	**25**	NA	Intergenic	(CATA)n|Simple_repeat|Simple_repeat	-118051	NM_172675	228960	Mm.277977	NM_172675	ENSMUSG00000027522	**Stx16**	4930401D03|5430410K23Rik|6330500A18Rik|AI648908|AW553605|Syn16	syntaxin 16	Amp	protein-coding
431_T12,T32	chr12	4.6E+07	4.6E+07	5999	+	**2**	**25**	NA	Intergenic	Intergenic	-451017	NM_144552	217517	Mm.285400	NM_144552	ENSMUSG00000046314	**Stxbp6**	BC024598|C85317	syntaxin binding protein 6 (amisyn)	Amp	protein-coding
500_T22,T42	chr13	1.7E+07	1.7E+07	1999	+	**2**	**25**	NA	intron (NM_138654, intron 11 of 13)	L1_Mm|LINE|L1	416265	NM_138654	192136	Mm.364180	NM_138654	ENSMUSG00000055137	**Sugct**	5033411D12Rik|D17907	succinyl-CoA glutarate-CoA transferase	Amp	protein-coding
3327_T1,T42	chr7	1.4E+07	1.4E+07	999	+	**2**	**25**	NA	Intergenic	Lx7|LINE|L1	-12590	NM_001111296	20859	Mm.260026	NM_001111296	ENSMUSG00000078798	**Sult2a1**	ST2A1|Std|Sth1|mSTa1	sulfotransferase family 2A, dehydroepiandrosterone (DHEA)-preferring, member 1	Amp	protein-coding
3602_T32,T3	chr8	8.3E+07	8.3E+07	999	+	**2**	**25**	NA	Intergenic	L1Md_A|LINE|L1	-44352	NM_001111304	71310	Mm.24031	NM_027758	ENSMUSG00000031709	**Tbc1d9**	4933431N12Rik|AI847101|AW490653|C76116	TBC1 domain family, member 9	Amp	protein-coding

3499_T32,T3	chr8	4.9E+07	4.9E+07	1999	+	**2**	**25**	NA	Intergenic	Intergenic	-459810	NM_011857	23965	Mm.42191	NM_011857	ENSMUSG00000031561	**Tenm3**	2610100B16Rik|Odz1|Odz3|Ten-m3|mKIAA1455	teneurin transmembrane protein 3	Amp	protein-coding
2928_T12,T32	chr6	1.7E+07	1.7E+07	999	+	**2**	**25**	NA	Intergenic	Intergenic	-37149	NM_207176	21753	Mm.436548	NM_011570	ENSMUSG00000029552	**Tes**	D6Ertd352e|TESS|Tes1|Tes2|testin2	testis derived transcript	Amp	protein-coding
3447_T22,T3	chr8	3.4E+07	3.4E+07	999	+	**2**	**25**	NA	exon (NM_031374, exon 1 of 4)	exon (NM_031374, exon 1 of 4)	4456	NM_031374	104271	Mm.280624	NM_031374	ENSMUSG00000009628	**Tex15**	2210014E14Rik|AL022622|AU022940	testis expressed gene 15	Amp	protein-coding
2300_T12,T42	chr3	5.7E+07	5.7E+07	10999	+	**2**	**25**	NA	Intergenic	L1Md_A|LINE|L1	236919	NM_008536	17112	Mm.856	NM_008536	ENSMUSG00000027800	**Tm4sf1**	L6|M3s1	transmembrane 4 superfamily member 1	Amp	protein-coding
33_T22,T4	chr1	5.1E+07	5.1E+07	999	+	**2**	**25**	NA	Intergenic	L1M4|LINE|L1	-308523	NM_019790	56363	Mm.245154	NM_019790	ENSMUSG00000026109	**Tmeff2**	4832418D20Rik|7630402F16Rik|TR-2	transmembrane protein with EGF-like and two follistatin-like domains 2	Amp	protein-coding
558_T1,T2	chr13	8.5E+07	8.5E+07	3999	+	**2**	**25**	NA	Intergenic	Intergenic	298204	NM_175187	72745	Mm.295063	NM_175187	ENSMUSG00000035762	**Tmem161b**	2810446P07Rik|AI843389	transmembrane protein 161B	Amp	protein-coding
978_T32,T3	chr15	4.4E+07	4.4E+07	999	+	**2**	**25**	NA	Intergenic	L1MA8|LINE|L1	17029	NM_175502	239408	Mm.41219	NM_175502	ENSMUSG00000054409	**Tmem74**	AA549547|B230382K22Rik	transmembrane protein 74	Amp	protein-coding
2594_T2,T32	chr4	6.4E+07	6.4E+07	3999	+	**2**	**25**	NA	Intergenic	Kanga11a|DNA|TcMar-Tc2	-352485	NM_011607	21923	Mm.980	NM_011607	ENSMUSG00000028364	**Tnc**	AI528729|C130033P17Rik|Hxb|TN|TN-C|Ten|cytotactin|tenascin-C	tenascin C	Amp	protein-coding
1041_T12,T32	chr15	5E+07	5E+07	3999	+	**2**	**25**	NA	Intergenic	Intergenic	952549	NM_001310481	83925	Mm.30466	NM_032000	ENSMUSG00000038679	**Trps1**	AI115454|AI447310|D15Ertd586e	trichorhinophalangeal syndrome I (human)	Amp	protein-coding
1043_T12,T4	chr15	5E+07	5E+07	999	+	**2**	**25**	NA	Intergenic	Intergenic	722049	NM_001310481	83925	Mm.30466	NM_032000	ENSMUSG00000038679	**Trps1**	AI115454|AI447310|D15Ertd586e	trichorhinophalangeal syndrome I (human)	Amp	protein-coding
1508_T12,T3	chr2	6.6E+07	6.6E+07	999	+	**2**	**25**	NA	exon (NM_018733, exon 22 of 27)	exon (NM_018733, exon 22 of 27)	-32383	NM_001290669	73668	Mm.250868	NM_001047604	ENSMUSG00000034848	**Ttc21b**	2410066K11Rik|Thm1|aln|mKIAA1992	tetratricopeptide repeat domain 21B	Amp	protein-coding
2692_T3,T4	chr4	1.5E+08	1.5E+08	8999	+	**2**	**25**	NA	Intergenic	Intergenic	-9200	NM_172878	242800	Mm.72944	NM_172878	ENSMUSG00000046637	**Ttc34**	4932418L02|B230396O12Rik	tetratricopeptide repeat domain 34	Amp	protein-coding
2645_T2,T4	chr4	9.3E+07	9.3E+07	999	+	**2**	**25**	NA	Intergenic	Lx5c|LINE|L1	492511	NM_026954	69136	Mm.171558	NM_026954	ENSMUSG00000054000	**Tusc1**	2200001D17Rik|TSG-9	tumor suppressor candidate 1	Amp	protein-coding
2652_T22,T2	chr4	9.4E+07	9.4E+07	999	+	**2**	**25**	NA	Intergenic	Intergenic	-428489	NM_026954	69136	Mm.171558	NM_026954	ENSMUSG00000054000	**Tusc1**	2200001D17Rik|TSG-9	tumor suppressor candidate 1	Amp	protein-coding

3784_T22,T2	chr9	1.2E+08	1.2E+08	9999	+	**2**	**25**	NA	intron (NM_177589, intron 17 of 36)	GA-rich|Low_complexity|Low_complexity	45672	NM_177589	209012	Mm.371804	NM_177589	ENSMUSG00000040936	**Ulk4**	4932415A06Rik|A730098P15	unc-51-like kinase 4	Amp	protein-coding
3430_T1,T2	chr8	2.9E+07	2.9E+07	3999	+	**2**	**25**	NA	Intergenic	Intergenic	-180864	NM_153135	210801	Mm.248433	NM_153135	ENSMUSG00000063626	**Unc5d**	D930029E11Rik|Unc5h4|mKIAA1777	unc-5 netrin receptor D	Amp	protein-coding
3433_T1,T2	chr8	2.9E+07	2.9E+07	3999	+	**2**	**25**	NA	Intergenic	Intergenic	-238864	NM_153135	210801	Mm.248433	NM_153135	ENSMUSG00000063626	**Unc5d**	D930029E11Rik|Unc5h4|mKIAA1777	unc-5 netrin receptor D	Amp	protein-coding
3436_T22,T42	chr8	3E+07	3E+07	999	+	**2**	**25**	NA	Intergenic	Intergenic	-484364	NM_153135	210801	Mm.248433	NM_153135	ENSMUSG00000063626	**Unc5d**	D930029E11Rik|Unc5h4|mKIAA1777	unc-5 netrin receptor D	Amp	protein-coding
3542_T2,T42	chr8	5.4E+07	5.4E+07	999	+	**2**	**25**	NA	Intergenic	Intergenic	-94532	NM_009506	22341	Mm.1402	NM_009506	ENSMUSG00000031520	**Vegfc**	AW228853|VEGF-C	vascular endothelial growth factor C	Amp	protein-coding
3544_T3,T4	chr8	5.4E+07	5.4E+07	999	+	**2**	**25**	NA	intron (NM_009506, intron 3 of 6)	intron (NM_009506, intron 3 of 6)	87468	NM_009506	22341	Mm.1402	NM_009506	ENSMUSG00000031520	**Vegfc**	AW228853|VEGF-C	vascular endothelial growth factor C	Amp	protein-coding
358_T12,T1	chr11	1.6E+07	1.6E+07	999	+	**2**	**25**	NA	Intergenic	IMPB_01|Satellite|Satellite	-689724	NM_001290539	211739	Mm.284015	NM_145967	ENSMUSG00000048834	**Vstm2a**	Vstm2	V-set and transmembrane domain containing 2A	Amp	protein-coding
361_T1,T22	chr11	1.6E+07	1.6E+07	999	+	**2**	**25**	NA	Intergenic	Intergenic	-684724	NM_001290539	211739	Mm.284015	NM_145967	ENSMUSG00000048834	**Vstm2a**	Vstm2	V-set and transmembrane domain containing 2A	Amp	protein-coding
362_T12,T1	chr11	1.6E+07	1.6E+07	3999	+	**2**	**25**	NA	Intergenic	Intergenic	-619224	NM_001290539	211739	Mm.284015	NM_145967	ENSMUSG00000048834	**Vstm2a**	Vstm2	V-set and transmembrane domain containing 2A	Amp	protein-coding
363_T2,T4	chr11	1.6E+07	1.6E+07	3999	+	**2**	**25**	NA	Intergenic	MTE2a|LTR|ERVL-MaLR	-598224	NM_001290539	211739	Mm.284015	NM_145967	ENSMUSG00000048834	**Vstm2a**	Vstm2	V-set and transmembrane domain containing 2A	Amp	protein-coding
348_T12,T3	chr11	1E+07	1E+07	3999	+	**2**	**25**	NA	Intergenic	Intergenic	-799516	NM_177033	319922	Mm.337851	NM_177033	ENSMUSG00000050830	**Vwc2**	A930041G11Rik|G11|PSST739|UNQ739|cradin	von Willebrand factor C domain containing 2	Amp	protein-coding
349_T22,T2	chr11	1.1E+07	1.1E+07	999	+	**2**	**25**	NA	Intergenic	Intergenic	-281016	NM_177033	319922	Mm.337851	NM_177033	ENSMUSG00000050830	**Vwc2**	A930041G11Rik|G11|PSST739|UNQ739|cradin	von Willebrand factor C domain containing 2	Amp	protein-coding
1701_T32,T4	chr3	1.7E+07	1.7E+07	10999	+	**2**	**25**	NA	Intergenic	Lx6|LINE|L1	378817	NM_001145919	229096	Mm.23834	NM_172677	ENSMUSG00000047213	**Ythdf3**	9130022A11Rik	YTH domain family 3	Amp	protein-coding
1706_T2,T3	chr3	1.7E+07	1.7E+07	3999	+	**2**	**25**	NA	Intergenic	Intergenic	474317	NM_001145919	229096	Mm.23834	NM_172677	ENSMUSG00000047213	**Ythdf3**	9130022A11Rik	YTH domain family 3	Amp	protein-coding
1489_T3	chr2	4.8E+07	4.8E+07	4999	+	**1**	**12.5**	NA	Intergenic	Intergenic	-1E+06	NM_007396	11480	Mm.314338	NM_007396	ENSMUSG00000052155	**Acvr2a**	ActrIIa|Acvr2|TactrII	activin receptor IIA	Amp	protein-coding

620_T3	chr14	6.9E+07	6.9E+07	999	+	**1**	**12.5**	NA	Intergenic	Intergenic	-123158	NM_183366	13522	Mm.117450	NM_010082	ENSMUSG00000014725	**Adam28**	C130072N01Rik|D430033C21Rik|Dtgn1|MDC-L|MDCL|TECADAM|eMDCII	a disintegrin and metallopeptidase domain 28	Amp	protein-coding
619_T2	chr14	6.9E+07	6.9E+07	17999	+	**1**	**12.5**	NA	intron (NR_102399, intron 12 of 21)	intron (NR_102399, intron 12 of 21)	25342	NM_010082	13522	Mm.117450	NM_010082	ENSMUSG00000014725	**Adam28**	C130072N01Rik|D430033C21Rik|Dtgn1|MDC-L|MDCL|TECADAM|eMDCII	a disintegrin and metallopeptidase domain 28	Amp	protein-coding
3553_T2	chr8	5.6E+07	5.6E+07	3999	+	**1**	**12.5**	NA	Intergenic	T-rich|Low_complexity|Low_complexity	122464	NM_175939	244486	Mm.67684	NM_175939	ENSMUSG00000046258	**Adam29**	-	a disintegrin and metallopeptidase domain 29	Amp	protein-coding
3556_T2	chr8	5.6E+07	5.6E+07	999	+	**1**	**12.5**	NA	Intergenic	Intergenic	86964	NM_175939	244486	Mm.67684	NM_175939	ENSMUSG00000046258	**Adam29**	-	a disintegrin and metallopeptidase domain 29	Amp	protein-coding
587_T1	chr13	1E+08	1E+08	3999	+	**1**	**12.5**	NA	intron (NM_001081020, intron 20 of 24)	intron (NM_001081020, intron 20 of 24)	162627	NM_001081020	108154	Mm.208125	NM_175496	ENSMUSG00000046169	**Adamts6**	A930019D11Rik|ADAM-TS6|b2b1879.1Clo|b2b2029Clo|b2b2182Clo|b2b2187.1Clo|b2b2228Clo	a disintegrin-like and metallopeptidase (reprolysin type) with thrombospondin type 1 motif, 6	Amp	protein-coding
1110_T2	chr15	6.5E+07	6.5E+07	3999	+	**1**	**12.5**	NA	Intergenic	Lx8b|LINE|L1	-205204	NM_009623	11514	Mm.1425	NM_009623	ENSMUSG00000022376	**Adcy8**	AC8|AW060868	adenylate cyclase 8	Amp	protein-coding
1320_T42	chr17	9.4E+07	9.4E+07	999	+	**1**	**12.5**	NA	Intergenic	L1ME2|LINE|L1	384832	NM_009625	11516	Mm.3407	NM_009625	ENSMUSG00000024256	**Adcyap1**	PACAP	adenylate cyclase activating polypeptide 1	Amp	protein-coding
1326_T4	chr17	9.4E+07	9.4E+07	3999	+	**1**	**12.5**	NA	Intergenic	ORR1D1|LTR|ERVL-MaLR	575332	NM_009625	11516	Mm.3407	NM_009625	ENSMUSG00000024256	**Adcyap1**	PACAP	adenylate cyclase activating polypeptide 1	Amp	protein-coding
2763_T12	chr5	5E+07	5E+07	999	+	**1**	**12.5**	NA	Intergenic	Intergenic	383996	NM_133911	70693	Mm.272974	NM_133911	ENSMUSG00000029090	**Adgra3**	3830613O22Rik|AU044632|Gpr125|Tem5-like	adhesion G protein-coupled receptor A3	Amp	protein-coding
2856_T4	chr5	8E+07	8E+07	4999	+	**1**	**12.5**	NA	Intergenic	Intergenic	-581593	NM_198702	319387	Mm.273631	NM_198702	ENSMUSG00000037605	**Adgrl3**	5430402I23Rik|CIRL-3|D130075K09Rik|Gm1379|LEC3|Lphn3|mKIAA0768	adhesion G protein-coupled receptor L3	Amp	protein-coding
2857_T1	chr5	8.1E+07	8.1E+07	999	+	**1**	**12.5**	NA	Intergenic	Intergenic	-409593	NM_198702	319387	Mm.273631	NM_198702	ENSMUSG00000037605	**Adgrl3**	5430402I23Rik|CIRL-3|D130075K09Rik|Gm1379|LEC3|Lphn3|mKIAA0768	adhesion G protein-coupled receptor L3	Amp	protein-coding

2860_T2	chr5	8.1E+07	8.1E+07	3999	+	**1**	**12.5**	NA	Intergenic	Lx7|LINE|L1	-181093	NM_198702	319387	Mm.273631	NM_198702	ENSMUSG00000037605	**Adgrl3**	5430402I23Rik|CIRL-3|D130075K09Rik|Gm1379|LEC3|Lphn3|mKIAA0768	adhesion G protein-coupled receptor L3	Amp	protein-coding
2861_T32	chr5	8.2E+07	8.2E+07	1999	+	**1**	**12.5**	NA	Intergenic	Intergenic	777907	NM_198702	319387	Mm.273631	NM_198702	ENSMUSG00000037605	**Adgrl3**	5430402I23Rik|CIRL-3|D130075K09Rik|Gm1379|LEC3|Lphn3|mKIAA0768	adhesion G protein-coupled receptor L3	Amp	protein-coding
2863_T32	chr5	8.2E+07	8.2E+07	999	+	**1**	**12.5**	NA	Intergenic	Lx8|LINE|L1	861407	NM_198702	319387	Mm.273631	NM_198702	ENSMUSG00000037605	**Adgrl3**	5430402I23Rik|CIRL-3|D130075K09Rik|Gm1379|LEC3|Lphn3|mKIAA0768	adhesion G protein-coupled receptor L3	Amp	protein-coding
3524_T32	chr8	5.2E+07	5.2E+07	14999	+	**1**	**12.5**	NA	Intergenic	Intergenic	-2E+06	NM_001205054	11593	Mm.334535	NM_001005847	ENSMUSG00000031521	**Aga**	AW060726	aspartylglucosaminidase	Amp	protein-coding
3528_T2	chr8	5.2E+07	5.2E+07	4999	+	**1**	**12.5**	NA	Intergenic	L1_Mur3|LINE|L1	-1E+06	NM_001205054	11593	Mm.334535	NM_001005847	ENSMUSG00000031521	**Aga**	AW060726	aspartylglucosaminidase	Amp	protein-coding
3532_T4	chr8	5.3E+07	5.3E+07	999	+	**1**	**12.5**	NA	Intergenic	AT_rich|Low_complexity|Low_complexity	-784702	NM_001205054	11593	Mm.334535	NM_001005847	ENSMUSG00000031521	**Aga**	AW060726	aspartylglucosaminidase	Amp	protein-coding
975_T32	chr15	4.3E+07	4.3E+07	999	+	**1**	**12.5**	NA	intron (NM_009640, intron 1 of 8)	L1_Mm|LINE|L1	97977	NM_001286062	11600	Mm.309336	NM_009640	ENSMUSG00000022309	**Angpt1**	1110046O21Rik|Ang-1|Ang1	angiopoietin 1	Amp	protein-coding
2980_T12	chr6	1.9E+07	1.9E+07	999	+	**1**	**12.5**	NA	Intergenic	L1Md_T|LINE|L1	33682	NM_001167757	75196	Mm.67665	NM_029202	ENSMUSG00000029517	**Ankrd7**	4930532L20Rik|AV278427	ankyrin repeat domain 7	Amp	protein-coding
2982_T12	chr6	1.9E+07	1.9E+07	999	+	**1**	**12.5**	NA	Intergenic	B4A|SINE|B4	40682	NM_001167757	75196	Mm.67665	NM_029202	ENSMUSG00000029517	**Ankrd7**	4930532L20Rik|AV278427	ankyrin repeat domain 7	Amp	protein-coding
2995_T12	chr6	1.9E+07	1.9E+07	999	+	**1**	**12.5**	NA	Intergenic	Intergenic	160682	NM_001167757	75196	Mm.67665	NM_029202	ENSMUSG00000029517	**Ankrd7**	4930532L20Rik|AV278427	ankyrin repeat domain 7	Amp	protein-coding
3571_T2	chr8	6.3E+07	6.3E+07	999	+	**1**	**12.5**	NA	Intergenic	Intergenic	-407807	NM_011922	26359	Mm.42179	NM_011922	ENSMUSG00000031635	**Anxa10**	-	annexin A10	Amp	protein-coding
3370_T2	chr7	6.4E+07	6.4E+07	999	+	**1**	**12.5**	NA	Intergenic	Intergenic	-35706	NM_007461	11784	Mm.4657	NM_007461	ENSMUSG00000030519	**Apba2**	X11-like|X11L|XllL|mXllL	amyloid beta (A4) precursor protein-binding, family A, member 2	Amp	protein-coding
1538_T42	chr2	9.4E+07	9.4E+07	4999	+	**1**	**12.5**	NA	Intergenic	L1Md_F3|LINE|L1	-12814	NM_001305258	11800	Mm.181824	NM_007466	ENSMUSG00000027193	**Api5**	AAC-11|AI196452|API-5	apoptosis inhibitor 5	Amp	protein-coding

548_T42	chr13	8.1E+07	8.1E+07	5999	+	**1**	**12.5**	NA	Intergenic	LTR82B|LTR|ERVL	-246922	NM_001042591	105171	Mm.423137	NM_001042591	ENSMUSG00000074794	**Arrdc3**	AI450344|mKIAA1376	arrestin domain containing 3	Amp	protein-coding
161_T2	chr1	1.4E+08	1.4E+08	999	+	**1**	**12.5**	NA	Intergenic	Intergenic	-1E+06	NM_020025	26878	Mm.285580	NM_020025	ENSMUSG00000033849	**B3galt2**	-	UDP-Gal:betaGlcNAc beta 1,3-galactosyltransferase, polypeptide 2	Amp	protein-coding
164_T4	chr1	1.4E+08	1.4E+08	1999	+	**1**	**12.5**	NA	Intergenic	L1Md_T|LINE|L1	-603197	NM_020025	26878	Mm.285580	NM_020025	ENSMUSG00000033849	**B3galt2**	-	UDP-Gal:betaGlcNAc beta 1,3-galactosyltransferase, polypeptide 2	Amp	protein-coding
2495_T4	chr3	1.4E+08	1.4E+08	1999	+	**1**	**12.5**	NA	intron (NM_001310749, intron 6 of 15)	intron (NM_001310749, intron 6 of 15)	136566	NM_001310749	242248	Mm.30832	NM_001033350	ENSMUSG00000037922	**Bank1**	A530094C12Rik|AI451642|AVIEF|BANK	B cell scaffold protein with ankyrin repeats 1	Amp	protein-coding
2497_T3	chr3	1.4E+08	1.4E+08	999	+	**1**	**12.5**	NA	intron (NM_001310749, intron 6 of 15)	intron (NM_001310749, intron 6 of 15)	131066	NM_001310749	242248	Mm.30832	NM_001033350	ENSMUSG00000037922	**Bank1**	A530094C12Rik|AI451642|AVIEF|BANK	B cell scaffold protein with ankyrin repeats 1	Amp	protein-coding
1588_T12	chr2	1.1E+08	1.1E+08	999	+	**1**	**12.5**	NA	Intergenic	Intergenic	-153700	NM_007540	12064	Mm.1442	NM_007540	ENSMUSG00000048482	**Bdnf**	-	brain derived neurotrophic factor	Amp	protein-coding
2664_T2	chr4	1.1E+08	1.1E+08	3999	+	**1**	**12.5**	NA	intron (NM_030231, intron 3 of 12)	intron (NM_030231, intron 3 of 12)	-474506	NR_033793	67621	Mm.45533	NM_026279	ENSMUSG00000028545	**Bend5**	2310026E23Rik	BEN domain containing 5	Amp	protein-coding
2665_T42	chr4	1.1E+08	1.1E+08	3999	+	**1**	**12.5**	NA	intron (NM_030231, intron 4 of 12)	intron (NM_030231, intron 4 of 12)	-413506	NR_033793	67621	Mm.45533	NM_026279	ENSMUSG00000028545	**Bend5**	2310026E23Rik	BEN domain containing 5	Amp	protein-coding
3710_T12	chr9	7805501	7848500	42999	+	**1**	**12.5**	NA	exon (NM_007465, exon 3 of 8)	exon (NM_007465, exon 3 of 8)	9806	NM_001291503	11797	Mm.335659	NM_007465	ENSMUSG00000057367	**Birc2**	AW146227|Api1|Api2|Birc3|C-IAP1|C330006D17Rik|HIAP1|HIAP2|IAP1|IAP2|MIAP1|MIAP2|MIHB|MIHC|RNF48|cIAP1|cIAP2|mcIAP1	baculoviral IAP repeat-containing 2	Amp	protein-coding
2601_T1	chr4	6.8E+07	6.8E+07	999	+	**1**	**12.5**	NA	Intergenic	Intergenic	795397	NM_019967	56710	Mm.248788	NM_019967	ENSMUSG00000028351	**Brinp1**	BRINP|Dbc1|Dbccr1|Fam5a	bone morphogenic protein/retinoic acid inducible neural specific 1	Amp	protein-coding
2604_T2	chr4	6.8E+07	6.8E+07	999	+	**1**	**12.5**	NA	Intergenic	Intergenic	469397	NM_019967	56710	Mm.248788	NM_019967	ENSMUSG00000028351	**Brinp1**	BRINP|Dbc1|Dbccr1|Fam5a	bone morphogenic protein/retinoic acid inducible neural specific 1	Amp	protein-coding

2610_T32	chr4	6.9E+07	6.9E+07	999	+	**1**	**12.5**	NA	Intergenic	RSINE1|SINE|B4	-422603	NM_019967	56710	Mm.248788	NM_019967	ENSMUSG00000028351	**Brinp1**	BRINP|Dbc1|Dbccr1|Fam5a	bone morphogenic protein/retinoic acid inducible neural specific 1	Amp	protein-coding
180_T22	chr1	1.5E+08	1.5E+08	3999	+	**1**	**12.5**	NA	intron (NM_001145807, intron 1 of 7)	intron (NM_001145807, intron 1 of 7)	4813	NM_001145807	215378	Mm.441817	NM_153539	ENSMUSG00000035131	**Brinp3**	B830045N13Rik|Fam5c	bone morphogenetic protein/retinoic acid inducible neural specific 3	Amp	protein-coding
3390_T42	chr7	1.2E+08	1.2E+08	3999	+	**1**	**12.5**	NA	intron (NM_001316672, intron 8 of 16)	L1Md_A|LINE|L1	-133244	NM_019430	54376	Mm.444212	NM_019430	ENSMUSG00000066189	**Cacng3**	-	calcium channel, voltage-dependent, gamma subunit 3	Amp	protein-coding
1168_T2	chr16	6.7E+07	6.7E+07	4999	+	**1**	**12.5**	NA	intron (NM_178721, intron 1 of 9)	intron (NM_178721, intron 1 of 9)	263908	NM_001145977	239857	Mm.394300	NM_178721	ENSMUSG00000064115	**Cadm2**	2900078E11Rik|9330131D06|A830029E02Rik|Igdf4d|Igsf4d|NECL3|SynCAM2|SynCAM2	cell adhesion molecule 2	Amp	protein-coding
1169_T3	chr16	6.8E+07	6.8E+07	6999	+	**1**	**12.5**	NA	intron (NM_178721, intron 1 of 9)	intron (NM_178721, intron 1 of 9)	103908	NM_001145977	239857	Mm.394300	NM_178721	ENSMUSG00000064115	**Cadm2**	2900078E11Rik|9330131D06|A830029E02Rik|Igdf4d|Igsf4d|NECL3|SynCAM2|SynCAM2	cell adhesion molecule 2	Amp	protein-coding
563_T2	chr13	8.5E+07	8.5E+07	999	+	**1**	**12.5**	NA	intron (NM_145452, intron 11 of 24)	intron (NM_145452, intron 11 of 24)	43523	NM_023243	66671	Mm.18474	NM_023243	ENSMUSG00000021548	**Ccnh**	6330408H09Rik|AI661354|AV102684|AW538719	cyclin H	Amp	protein-coding
583_T22	chr13	1E+08	1E+08	5999	+	**1**	**12.5**	NA	Intergenic	Intergenic	-212058	NM_008533	17079	Mm.373974	NM_008533	ENSMUSG00000021624	**Cd180**	F630107B15|Ly78|RP105	CD180 antigen	Amp	protein-coding
1160_T12	chr16	4.5E+07	4.5E+07	4999	+	**1**	**12.5**	NA	intron (NM_010818, intron 5 of 5)	intron (NM_010818, intron 5 of 5)	21053	NM_010818	17470	Mm.245851	NM_010818	ENSMUSG00000022661	**Cd200**	Mox2|OX2	CD200 antigen	Amp	protein-coding
2690_T3	chr4	1.4E+08	1.4E+08	999	+	**1**	**12.5**	NA	Intergenic	Intergenic	-23241	NM_001243769	12540	Mm.1022	NM_009861	ENSMUSG00000006699	**Cdc42**	AI747189|AU018915	cell division cycle 42	Amp	protein-coding
922_T42	chr15	1.9E+07	1.9E+07	3999	+	**1**	**12.5**	NA	Intergenic	URR1A|DNA|hAT-Charlie	-131447	NM_001316758	320873	Mm.117794	NM_009865	ENSMUSG00000022321	**Cdh10**	A830016G23Rik|C030003B10Rik|C030011H18Rik	cadherin 10	Amp	protein-coding
923_T3	chr15	1.9E+07	1.9E+07	2999	+	**1**	**12.5**	NA	Intergenic	Intergenic	-81947	NM_001316758	320873	Mm.117794	NM_009865	ENSMUSG00000022321	**Cdh10**	A830016G23Rik|C030003B10Rik|C030011H18Rik	cadherin 10	Amp	protein-coding
926_T4	chr15	1.9E+07	1.9E+07	10999	+	**1**	**12.5**	NA	Intergenic	Intergenic	-36947	NM_001316758	320873	Mm.117794	NM_009865	ENSMUSG00000022321	**Cdh10**	A830016G23Rik|C030003B10Rik|C030011H18Rik	cadherin 10	Amp	protein-coding
933_T4	chr15	1.9E+07	1.9E+07	999	+	**1**	**12.5**	NA	Intergenic	Intergenic	590671	NM_009865	320873	Mm.117794	NM_009865	ENSMUSG00000022321	**Cdh10**	A830016G23Rik|C030003B10Rik|C030011H18Rik	cadherin 10	Amp	protein-coding

934_T12	chr15	1.9E+07	1.9E+07	3999	+	**1**	**12.5**	NA	Intergenic	ORR1E-int|LTR|ERVL-MaLR	649171	NM_009865	320873	Mm.117794	NM_009865	ENSMUSG00000022321	**Cdh10**	A830016G23Rik|C030003B10Rik|C030011H18Rik	cadherin 10	Amp	protein-coding
3643_T22	chr8	1E+08	1E+08	999	+	**1**	**12.5**	NA	Intergenic	Intergenic	1627111	NM_009866	12552	Mm.1571	NM_009866	ENSMUSG00000031673	**Cdh11**	Cad11|OSF-4	cadherin 11	Amp	protein-coding
3645_T4	chr8	1E+08	1E+08	999	+	**1**	**12.5**	NA	Intergenic	Intergenic	1623111	NM_009866	12552	Mm.1571	NM_009866	ENSMUSG00000031673	**Cdh11**	Cad11|OSF-4	cadherin 11	Amp	protein-coding
3648_T22	chr8	1E+08	1E+08	4999	+	**1**	**12.5**	NA	Intergenic	Intergenic	1562111	NM_009866	12552	Mm.1571	NM_009866	ENSMUSG00000031673	**Cdh11**	Cad11|OSF-4	cadherin 11	Amp	protein-coding
3659_T4	chr8	1E+08	1E+08	3999	+	**1**	**12.5**	NA	Intergenic	Intergenic	1092611	NM_009866	12552	Mm.1571	NM_009866	ENSMUSG00000031673	**Cdh11**	Cad11|OSF-4	cadherin 11	Amp	protein-coding
3664_T1	chr8	1E+08	1E+08	6999	+	**1**	**12.5**	NA	Intergenic	L1_Mus1|LINE|L1	751111	NM_009866	12552	Mm.1571	NM_009866	ENSMUSG00000031673	**Cdh11**	Cad11|OSF-4	cadherin 11	Amp	protein-coding
3665_T4	chr8	1E+08	1E+08	3999	+	**1**	**12.5**	NA	Intergenic	Intergenic	618611	NM_009866	12552	Mm.1571	NM_009866	ENSMUSG00000031673	**Cdh11**	Cad11|OSF-4	cadherin 11	Amp	protein-coding
3666_T2	chr8	1E+08	1E+08	3999	+	**1**	**12.5**	NA	Intergenic	Intergenic	555611	NM_009866	12552	Mm.1571	NM_009866	ENSMUSG00000031673	**Cdh11**	Cad11|OSF-4	cadherin 11	Amp	protein-coding
948_T22	chr15	2.1E+07	2.1E+07	4999	+	**1**	**12.5**	NA	Intergenic	Intergenic	-79452	NM_001008420	215654	Mm.300909	NM_001008420	ENSMUSG00000040452	**Cdh12**	Cdhb	cadherin 12	Amp	protein-coding
950_T2	chr15	2.1E+07	2.1E+07	999	+	**1**	**12.5**	NA	intron (NM_001008420, intron 2 of 11)	intron (NM_001008420, intron 2 of 11)	145548	NM_001008420	215654	Mm.300909	NM_001008420	ENSMUSG00000040452	**Cdh12**	Cdhb	cadherin 12	Amp	protein-coding
951_T3	chr15	2.2E+07	2.2E+07	3999	+	**1**	**12.5**	NA	intron (NM_001008420, intron 8 of 11)	MTE2b|LTR|ERVL-MaLR	448048	NM_001008420	215654	Mm.300909	NM_001008420	ENSMUSG00000040452	**Cdh12**	Cdhb	cadherin 12	Amp	protein-coding
105_T4	chr1	1E+08	1E+08	999	+	**1**	**12.5**	NA	Intergenic	Intergenic	-1E+06	NM_011800	23836	Mm.103640	NM_011800	ENSMUSG00000050840	**Cdh20**	Cdh7	cadherin 20	Amp	protein-coding
108_T2	chr1	1E+08	1E+08	4999	+	**1**	**12.5**	NA	Intergenic	AT_rich|Low_complexity|Low_complexity	-482819	NM_011800	23836	Mm.103640	NM_011800	ENSMUSG00000050840	**Cdh20**	Cdh7	cadherin 20	Amp	protein-coding
884_T2	chr15	1.4E+07	1.4E+07	3999	+	**1**	**12.5**	NA	Intergenic	Intergenic	-1E+06	NM_007666	12563	Mm.57048	NM_007666	ENSMUSG00000039385	**Cdh6**	cad6	cadherin 6	Amp	protein-coding
888_T1	chr15	1.5E+07	1.5E+07	999	+	**1**	**12.5**	NA	Intergenic	ORR1A1|LTR|ERVL-MaLR	-2E+06	NM_007666	12563	Mm.57048	NM_007666	ENSMUSG00000039385	**Cdh6**	cad6	cadherin 6	Amp	protein-coding

890_T32	chr15	1.5E+07	1.5E+07	999	+	**1**	**12.5**	NA	Intergenic	Intergenic	-2E+06	NM_007666	12563	Mm.57048	NM_007666	ENSMUSG00000039385	**Cdh6**	cad6	cadherin 6	Amp	protein-coding
115_T22	chr1	1.1E+08	1.1E+08	5999	+	**1**	**12.5**	NA	Intergenic	Intergenic	-903400	NM_001316743	241201	Mm.213407	NM_172853	ENSMUSG00000026312	**Cdh7**	9330156F07Rik|CDH7L1	cadherin 7, type 2	Amp	protein-coding
116_T3	chr1	1.1E+08	1.1E+08	999	+	**1**	**12.5**	NA	Intergenic	Intergenic	-686900	NM_001316743	241201	Mm.213407	NM_172853	ENSMUSG00000026312	**Cdh7**	9330156F07Rik|CDH7L1	cadherin 7, type 2	Amp	protein-coding
122_T3	chr1	1.1E+08	1.1E+08	2999	+	**1**	**12.5**	NA	Intergenic	Intergenic	-459900	NM_001316743	241201	Mm.213407	NM_172853	ENSMUSG00000026312	**Cdh7**	9330156F07Rik|CDH7L1	cadherin 7, type 2	Amp	protein-coding
3626_T4	chr8	9.9E+07	9.9E+07	999	+	**1**	**12.5**	NA	intron (NR_133570, intron 2 of 10)	L1_Mur2|LINE|L1	99471	NM_001285913	12564	Mm.441131	NM_007667	ENSMUSG00000036510	**Cdh8**	AI851472|cad8	cadherin 8	Amp	protein-coding
3620_T1	chr8	9.9E+07	9.9E+07	3999	+	**1**	**12.5**	NA	intron (NR_133570, intron 4 of 10)	MT2A|LTR|ERVL	188971	NM_001285913	12564	Mm.441131	NM_007667	ENSMUSG00000036510	**Cdh8**	AI851472|cad8	cadherin 8	Amp	protein-coding
891_T32	chr15	1.5E+07	1.5E+07	4999	+	**1**	**12.5**	NA	Intergenic	Intergenic	-2E+06	NM_009869	12565	Mm.439758	NM_009869	ENSMUSG00000025370	**Cdh9**	-	cadherin 9	Amp	protein-coding
894_T12	chr15	1.5E+07	1.5E+07	999	+	**1**	**12.5**	NA	Intergenic	Intergenic	-1E+06	NM_009869	12565	Mm.439758	NM_009869	ENSMUSG00000025370	**Cdh9**	-	cadherin 9	Amp	protein-coding
895_T22	chr15	1.6E+07	1.6E+07	3999	+	**1**	**12.5**	NA	Intergenic	L1_Rod|LINE|L1	-1E+06	NM_009869	12565	Mm.439758	NM_009869	ENSMUSG00000025370	**Cdh9**	-	cadherin 9	Amp	protein-coding
896_T22	chr15	1.6E+07	1.6E+07	4999	+	**1**	**12.5**	NA	Intergenic	Intergenic	-1E+06	NM_009869	12565	Mm.439758	NM_009869	ENSMUSG00000025370	**Cdh9**	-	cadherin 9	Amp	protein-coding
900_T2	chr15	1.6E+07	1.6E+07	4999	+	**1**	**12.5**	NA	Intergenic	L1_Mur2|LINE|L1	-1E+06	NM_009869	12565	Mm.439758	NM_009869	ENSMUSG00000025370	**Cdh9**	-	cadherin 9	Amp	protein-coding
906_T1	chr15	1.6E+07	1.6E+07	3999	+	**1**	**12.5**	NA	Intergenic	Lx5c|LINE|L1	-437601	NM_009869	12565	Mm.439758	NM_009869	ENSMUSG00000025370	**Cdh9**	-	cadherin 9	Amp	protein-coding
909_T4	chr15	1.7E+07	1.7E+07	4999	+	**1**	**12.5**	NA	Intergenic	RMER19C|LTR|ERVK	154899	NM_009869	12565	Mm.439758	NM_009869	ENSMUSG00000025370	**Cdh9**	-	cadherin 9	Amp	protein-coding
911_T1	chr15	1.7E+07	1.7E+07	999	+	**1**	**12.5**	NA	Intergenic	Lx7|LINE|L1	295899	NM_009869	12565	Mm.439758	NM_009869	ENSMUSG00000025370	**Cdh9**	-	cadherin 9	Amp	protein-coding
912_T1	chr15	1.7E+07	1.7E+07	3999	+	**1**	**12.5**	NA	Intergenic	AT_rich|Low_complexity|Low_complexity	342399	NM_009869	12565	Mm.439758	NM_009869	ENSMUSG00000025370	**Cdh9**	-	cadherin 9	Amp	protein-coding
913_T4	chr15	1.7E+07	1.7E+07	3999	+	**1**	**12.5**	NA	Intergenic	Intergenic	443399	NM_009869	12565	Mm.439758	NM_009869	ENSMUSG00000025370	**Cdh9**	-	cadherin 9	Amp	protein-coding

914_T4	chr15	1.7E+07	1.7E+07	13999	+	**1**	**12.5**	NA	Intergenic	Intergenic	520399	NM_009869	12565	Mm.439758	NM_009869	ENSMUSG00000025370	**Cdh9**	-	cadherin 9	Amp	protein-coding
3713_T12	chr9	8054501	8276500	221999	+	**1**	**12.5**	NA	Intergenic	L1_Mus1|LINE|L1	-31206	NM_001045524	234915	Mm.101341	NM_001045524	ENSMUSG00000040729	**Cep126**	Kiaa1377|mKIAA1377	centrosomal protein 126	Amp	protein-coding
3095_T42	chr6	9E+07	9E+07	3999	+	**1**	**12.5**	NA	Intergenic	Intergenic	-13020	NM_173775	243538	Mm.386876	NM_173775	ENSMUSG00000048794	**Cfap100**	AW048498|C030041G11Rik|C230069K22Rik|Ccdc37	cilia and flagella associated protein 100	Amp	protein-coding
151_T4	chr1	1.4E+08	1.4E+08	999	+	**1**	**12.5**	NA	intron (NM_009888, intron 20 of 21)	L1_Mus3|LINE|L1	92411	NM_009888	12628	Mm.8655	NM_009888	ENSMUSG00000026365	**Cfh**	Mud-1|NOM|Sas-1|Sas1	complement component factor h	Amp	protein-coding
3727_T1	chr9	1.7E+07	1.7E+07	1999	+	**1**	**12.5**	NA	Intergenic	Intergenic	-863767	NM_025844	66917	Mm.103534	NM_025844	ENSMUSG00000001774	**Chordc1**	1110001O09Rik|AA409036|Chp-1|morgana	cysteine and histidine-rich domain (CHORD)-containing, zinc-binding protein 1	Amp	protein-coding
3366_T3	chr7	6.3E+07	6.3E+07	999	+	**1**	**12.5**	NA	Intergenic	Lx9|LINE|L1	-51474	NM_007390	11441	Mm.113464	NM_007390	ENSMUSG00000030525	**Chrna7**	Acra7|alpha7|nAchR	cholinergic receptor, nicotinic, alpha polypeptide 7	Amp	protein-coding
3107_T12	chr6	1.1E+08	1.1E+08	6999	+	**1**	**12.5**	NA	intron (NM_173004, intron 3 of 19)	MLT1B|LTR|ERVL-MaLR	-137765	NM_001109751	269784	Mm.321683	NM_173004	ENSMUSG00000064293	**Cntn4**	9630050B05|Axcam|BIG-2	contactin 4	Amp	protein-coding
3726_T4	chr9	1.1E+07	1.1E+07	999	+	**1**	**12.5**	NA	Intergenic	Intergenic	-461225	NM_001170787	244682	Mm.187021	NM_001033359	ENSMUSG00000039488	**Cntn5**	6720426O10Rik|A830025P08Rik|Gm507|NB-2	contactin 5	Amp	protein-coding
3724_T22	chr9	1.1E+07	1.1E+07	3999	+	**1**	**12.5**	NA	intron (NM_001170787, intron 1 of 24)	intron (NM_001170787, intron 1 of 24)	83226	NM_001033359	244682	Mm.187021	NM_001033359	ENSMUSG00000039488	**Cntn5**	6720426O10Rik|A830025P08Rik|Gm507|NB-2	contactin 5	Amp	protein-coding
3099_T32	chr6	1E+08	1E+08	999	+	**1**	**12.5**	NA	intron (NM_017383, intron 6 of 22)	intron (NM_017383, intron 6 of 22)	233956	NM_017383	53870	Mm.321671	NM_017383	ENSMUSG00000030092	**Cntn6**	NB-3	contactin 6	Amp	protein-coding
3048_T12	chr6	4.4E+07	4.4E+07	999	+	**1**	**12.5**	NA	Intergenic	Intergenic	-579061	NM_001004357	66797	Mm.440084	NM_025771	ENSMUSG00000039419	**Cntnap2**	5430425M22Rik|Caspr2|mKIAA0868	contactin associated protein-like 2	Amp	protein-coding
3054_T1	chr6	4.5E+07	4.5E+07	999	+	**1**	**12.5**	NA	intron (NM_001004357, intron 1 of 23)	intron (NM_001004357, intron 1 of 23)	62939	NM_001004357	66797	Mm.440084	NM_025771	ENSMUSG00000039419	**Cntnap2**	5430425M22Rik|Caspr2|mKIAA0868	contactin associated protein-like 2	Amp	protein-coding
3056_T12	chr6	4.6E+07	4.6E+07	6999	+	**1**	**12.5**	NA	intron (NM_001004357, intron 3 of 23)	intron (NM_001004357, intron 3 of 23)	752939	NM_001004357	66797	Mm.440084	NM_025771	ENSMUSG00000039419	**Cntnap2**	5430425M22Rik|Caspr2|mKIAA0868	contactin associated protein-like 2	Amp	protein-coding

566_T3	chr13	8.6E+07	8.6E+07	999	+	**1**	**12.5**	NA	Intergenic	Intergenic	-283205	NM_007749	12867	Mm.378898	NM_007749	ENSMUSG00000017778	**Cox7c**	COXVIIc|Cox7c1	cytochrome c oxidase subunit VIIc	Amp	protein-coding
569_T4	chr13	8.6E+07	8.6E+07	999	+	**1**	**12.5**	NA	Intergenic	L1_Mus2|LINE|L1	-431205	NM_007749	12867	Mm.378898	NM_007749	ENSMUSG00000017778	**Cox7c**	COXVIIc|Cox7c1	cytochrome c oxidase subunit VIIc	Amp	protein-coding
3108_T32	chr6	1.1E+08	1.1E+08	999	+	**1**	**12.5**	NA	Intergenic	L1Md_F2|LINE|L1	-79913	NM_175357	58799	Mm.290085	NM_021449	ENSMUSG00000005362	**Crbn**	2610203G15Rik|2900045O07Rik|AF229032|AW108261|piL	cereblon	Amp	protein-coding
3110_T12	chr6	1.1E+08	1.1E+08	999	+	**1**	**12.5**	NA	Intergenic	L1Md_T|LINE|L1	-83913	NM_175357	58799	Mm.290085	NM_021449	ENSMUSG00000005362	**Crbn**	2610203G15Rik|2900045O07Rik|AF229032|AW108261|piL	cereblon	Amp	protein-coding
1309_T4	chr17	7.7E+07	7.7E+07	999	+	**1**	**12.5**	NA	Intergenic	L1_Mus3|LINE|L1	-985248	NM_015800	50766	Mm.311912	NM_015800	ENSMUSG00000024074	**Crim1**	AU015004	cysteine rich transmembrane BMP regulator 1 (chordin like)	Amp	protein-coding
196_T42	chr1	1.7E+08	1.7E+08	3999	+	**1**	**12.5**	NA	Intergenic	L1Md_A|LINE|L1	37444	NM_007768	12944	Mm.28767	NM_007768	ENSMUSG00000037942	**Crp**	AI255847	C-reactive protein, pentraxin-related	Amp	protein-coding
1013_T22	chr15	4.8E+07	4.8E+07	999	+	**1**	**12.5**	NA	exon (NM_001081391, exon 17 of 71)	exon (NM_001081391, exon 17 of 71)	809989	NM_001081391	239420	Mm.309363	NM_001081391	ENSMUSG00000022311	**Csmd3**	4930500N14Rik|mKIAA1894	CUB and Sushi multiple domains 3	Amp	protein-coding
1035_T22	chr15	5E+07	5E+07	999	+	**1**	**12.5**	NA	Intergenic	Intergenic	-749011	NM_001081391	239420	Mm.309363	NM_001081391	ENSMUSG00000022311	**Csmd3**	4930500N14Rik|mKIAA1894	CUB and Sushi multiple domains 3	Amp	protein-coding
1038_T2	chr15	5E+07	5E+07	999	+	**1**	**12.5**	NA	Intergenic	Intergenic	-754011	NM_001081391	239420	Mm.309363	NM_001081391	ENSMUSG00000022311	**Csmd3**	4930500N14Rik|mKIAA1894	CUB and Sushi multiple domains 3	Amp	protein-coding
1039_T2	chr15	5E+07	5E+07	3999	+	**1**	**12.5**	NA	Intergenic	Intergenic	-928511	NM_001081391	239420	Mm.309363	NM_001081391	ENSMUSG00000022311	**Csmd3**	4930500N14Rik|mKIAA1894	CUB and Sushi multiple domains 3	Amp	protein-coding
1024_T1	chr15	4.9E+07	4.9E+07	3999	+	**1**	**12.5**	NA	intron (NM_001081391, intron 1 of 70)	Lx7|LINE|L1	59489	NM_001081391	239420	Mm.309363	NM_001081391	ENSMUSG00000022311	**Csmd3**	4930500N14Rik|mKIAA1894	CUB and Sushi multiple domains 3	Amp	protein-coding
1027_T42	chr15	4.9E+07	4.9E+07	999	+	**1**	**12.5**	NA	intron (NM_001081391, intron 1 of 70)	B4A|SINE|B4	29989	NM_001081391	239420	Mm.309363	NM_001081391	ENSMUSG00000022311	**Csmd3**	4930500N14Rik|mKIAA1894	CUB and Sushi multiple domains 3	Amp	protein-coding
1020_T12	chr15	4.8E+07	4.8E+07	999	+	**1**	**12.5**	NA	intron (NM_001081391, intron 13 of 70)	intron (NM_001081391, intron 13 of 70)	640989	NM_001081391	239420	Mm.309363	NM_001081391	ENSMUSG00000022311	**Csmd3**	4930500N14Rik|mKIAA1894	CUB and Sushi multiple domains 3	Amp	protein-coding

1015_T2	chr15	4.8E+07	4.8E+07	1999	+	**1**	**12.5**	NA	intron (NM_001081391, intron 16 of 70)	ID_B1|SINE|B4	805489	NM_001081391	239420	Mm.309363	NM_001081391	ENSMUSG00000022311	**Csmd3**	4930500N14Rik|mKIAA1894	CUB and Sushi multiple domains 3	Amp	protein-coding
1022_T1	chr15	4.8E+07	4.8E+07	3999	+	**1**	**12.5**	NA	intron (NM_001081391, intron 4 of 70)	intron (NM_001081391, intron 4 of 70)	330489	NM_001081391	239420	Mm.309363	NM_001081391	ENSMUSG00000022311	**Csmd3**	4930500N14Rik|mKIAA1894	CUB and Sushi multiple domains 3	Amp	protein-coding
1614_T32	chr2	1.5E+08	1.5E+08	1999	+	**1**	**12.5**	NA	Intergenic	L1Md_F2|LINE|L1	-117988	NM_009976	13010	Mm.4263	NM_009976	ENSMUSG00000027447	**Cst3**	CysC	cystatin C	Amp	protein-coding
1618_T1	chr2	1.5E+08	1.5E+08	999	+	**1**	**12.5**	NA	Intergenic	ORR1A4-int|LTR|ERVL-MaLR	-207488	NM_009976	13010	Mm.4263	NM_009976	ENSMUSG00000027447	**Cst3**	CysC	cystatin C	Amp	protein-coding
2428_T2	chr3	8.2E+07	8.2E+07	999	+	**1**	**12.5**	NA	Intergenic	Intergenic	-319616	NM_177662	229445	Mm.254642	NM_177662	ENSMUSG00000028015	**Ctso**	A330105D01Rik|AI118514	cathepsin O	Amp	protein-coding
2429_T32	chr3	8.2E+07	8.2E+07	1999	+	**1**	**12.5**	NA	Intergenic	Intergenic	-38116	NM_177662	229445	Mm.254642	NM_177662	ENSMUSG00000028015	**Ctso**	A330105D01Rik|AI118514	cathepsin O	Amp	protein-coding
2431_T32	chr3	8.2E+07	8.2E+07	16999	+	**1**	**12.5**	NA	Intergenic	Intergenic	-23616	NM_177662	229445	Mm.254642	NM_177662	ENSMUSG00000028015	**Ctso**	A330105D01Rik|AI118514	cathepsin O	Amp	protein-coding
2954_T42	chr6	1.9E+07	1.9E+07	999	+	**1**	**12.5**	NA	Intergenic	L1Md_T|LINE|L1	-65175	NM_080285	30785	Mm.224189	NM_080285	ENSMUSG00000000416	**Cttnbp2**	3010022N24Rik|4732477G22Rik|6430526E05|9130022E09Rik|AU040881|Cortbp2|ORF4|mKIAA1758	cortactin binding protein 2	Amp	protein-coding
2589_T12	chr4	5.1E+07	5.1E+07	3999	+	**1**	**12.5**	NA	Intergenic	Intergenic	-614178	NM_001162865	74914	Mm.159368	NM_001162865	ENSMUSG00000039555	**Cylc2**	4930488P18Rik	cylicin, basic protein of sperm head cytoskeleton 2	Amp	protein-coding
875_T42	chr15	5977501	5978500	999	+	**1**	**12.5**	NA	Intergenic	Intergenic	-321789	NM_001008702	13132	Mm.240830	NM_023118	ENSMUSG00000022150	**Dab2**	5730435J12Rik|AA960054|AI957090|D15Wsu122e|D630005B22Rik|Doc-2|Doc2|p96	disabled 2, mitogen-responsive phosphoprotein	Amp	protein-coding
877_T42	chr15	5982501	5984500	1999	+	**1**	**12.5**	NA	Intergenic	L1_Mm|LINE|L1	-316289	NM_001008702	13132	Mm.240830	NM_023118	ENSMUSG00000022150	**Dab2**	5730435J12Rik|AA960054|AI957090|D15Wsu122e|D630005B22Rik|Doc-2|Doc2|p96	disabled 2, mitogen-responsive phosphoprotein	Amp	protein-coding
782_T1	chr14	9.8E+07	9.8E+07	18999	+	**1**	**12.5**	NA	Intergenic	Intergenic	508765	NM_001038610	13134	Mm.320593	NM_007826	ENSMUSG00000055639	**Dach1**	Dac|Dach	dachshund 1 (Drosophila)	Amp	protein-coding

785_T4	chr14	9.8E+07	9.8E+07	4999	+	**1**	**12.5**	NA	intron (NM_007826, intron 1 of 11)	intron (NM_007826, intron 1 of 11)	141765	NM_001038610	13134	Mm.320593	NM_007826	ENSMUSG00000055639	**Dach1**	Dac|Dach	dachshund 1 (Drosophila)	Amp	protein-coding
787_T3	chr14	9.8E+07	9.8E+07	3999	+	**1**	**12.5**	NA	intron (NM_007826, intron 1 of 11)	intron (NM_007826, intron 1 of 11)	8265	NM_001038610	13134	Mm.320593	NM_007826	ENSMUSG00000055639	**Dach1**	Dac|Dach	dachshund 1 (Drosophila)	Amp	protein-coding
784_T4	chr14	9.8E+07	9.8E+07	1999	+	**1**	**12.5**	NA	intron (NM_007826, intron 2 of 11)	intron (NM_007826, intron 2 of 11)	193265	NM_001038610	13134	Mm.320593	NM_007826	ENSMUSG00000055639	**Dach1**	Dac|Dach	dachshund 1 (Drosophila)	Amp	protein-coding
2235_T22	chr3	5.6E+07	5.6E+07	999	+	**1**	**12.5**	NA	Intergenic	MMERGLN_LTR|LTR|ERV1	124242	NM_001195540	13175	Mm.393242	NM_019978	ENSMUSG00000027797	**Dclk1**	1700113D08Rik|2810480F11Rik|AI836758|Click-I|Cpg16|DcamkI1|Dcl|Dclk|mKIAA0369	doublecortin-like kinase 1	Amp	protein-coding
3700_T12	chr9	7186501	7245500	58999	+	**1**	**12.5**	NA	Intergenic	Lx|LINE|L1	31434	NM_029775	76863	Mm.27293	NM_029775	ENSMUSG00000032002	**Dcun1d5**	3110001A18Rik|4833420K19Rik|AW060460|D430047L21Rik	DCN1, defective in cullin neddylation 1, domain containing 5 (S. cerevisiae)	Amp	protein-coding
3689_T12	chr9	6439501	6475500	35999	+	**1**	**12.5**	NA	Intergenic	Intergenic	-190953	NM_027942	71829	Mm.141452	NM_027942	ENSMUSG00000047619	**Ddi1**	1700011N24Rik	DNA-damage inducible 1	Amp	protein-coding
3690_T12	chr9	6476501	6631500	154999	+	**1**	**12.5**	NA	Intergenic	Intergenic	-287453	NM_027942	71829	Mm.141452	NM_027942	ENSMUSG00000047619	**Ddi1**	1700011N24Rik	DNA-damage inducible 1	Amp	protein-coding
3691_T12	chr9	6635501	6677500	41999	+	**1**	**12.5**	NA	Intergenic	ORR1C1|LTR|ERVL-MaLR	-389953	NM_027942	71829	Mm.141452	NM_027942	ENSMUSG00000047619	**Ddi1**	1700011N24Rik	DNA-damage inducible 1	Amp	protein-coding
3692_T12	chr9	6678501	6691500	12999	+	**1**	**12.5**	NA	Intergenic	Lx3A|LINE|L1	-418453	NM_027942	71829	Mm.141452	NM_027942	ENSMUSG00000047619	**Ddi1**	1700011N24Rik	DNA-damage inducible 1	Amp	protein-coding
3693_T12	chr9	6692501	6750500	57999	+	**1**	**12.5**	NA	Intergenic	L1MC3|LINE|L1	-454953	NM_027942	71829	Mm.141452	NM_027942	ENSMUSG00000047619	**Ddi1**	1700011N24Rik	DNA-damage inducible 1	Amp	protein-coding
3688_T12	chr9	6141501	6438500	296999	+	**1**	**12.5**	NA	intron (NM_027924, intron 2 of 6)	intron (NM_027924, intron 2 of 6)	-23453	NM_027942	71829	Mm.141452	NM_027942	ENSMUSG00000047619	**Ddi1**	1700011N24Rik	DNA-damage inducible 1	Amp	protein-coding
964_T1	chr15	2.9E+07	2.9E+07	999	+	**1**	**12.5**	NA	Intergenic	Intergenic	718234	NM_133365	110082	Mm.248464	NM_133365	ENSMUSG00000022262	**Dnah5**	AU022615|Dnahc5|Mdnah5|b2b1134Clo|b2b1154Clo|b2b1537Clo|b2b1565Clo|b2b601Clo|mKIAA1603	dynein, axonemal, heavy chain 5	Amp	protein-coding
1626_T42	chr2	1.7E+08	1.7E+08	4999	+	**1**	**12.5**	NA	Intergenic	L1Md_T|LINE|L1	355193	NM_001163686	76829	Mm.41633	NM_029761	ENSMUSG00000027560	**Dok5**	2700055C10Rik	docking protein 5	Amp	protein-coding

148_T3	chr1	1.2E+08	1.2E+08	5999	+	**1**	**12.5**	NA	intron (NM_199021, intron 5 of 25)	intron (NM_199021, intron 5 of 25)	535059	NM_199021	269109	Mm.417716	NM_199021	ENSMUSG00000036815	**Dpp10**	6430601K09Rik|DPP X|Dprp3	dipeptidylpeptidase 10	Amp	protein-coding
147_T42	chr1	1.2E+08	1.2E+08	3999	+	**1**	**12.5**	NA	intron (NM_199021, intron 8 of 25)	intron (NM_199021, intron 8 of 25)	605059	NM_199021	269109	Mm.417716	NM_199021	ENSMUSG00000036815	**Dpp10**	6430601K09Rik|DPP X|Dprp3	dipeptidylpeptidase 10	Amp	protein-coding
2478_T4	chr3	1.2E+08	1.2E+08	3999	+	**1**	**12.5**	NA	intron (NM_170778, intron 13 of 22)	intron (NM_170778, intron 13 of 22)	456322	NM_170778	99586	Mm.27907	NM_170778	ENSMUSG00000033308	**Dpyd**	AI315208|DPD|E330028L06Rik	dihydropyrimidine dehydrogenase	Amp	protein-coding
129_T1	chr1	1.1E+08	1.1E+08	999	+	**1**	**12.5**	NA	Intergenic	Intergenic	-950082	NM_001081316	319901	Mm.103468	NM_001081316	ENSMUSG00000038702	**Dsel**	9330132E09Rik|DS-epi2	dermatan sulfate epimerase-like	Amp	protein-coding
130_T22	chr1	1.1E+08	1.1E+08	999	+	**1**	**12.5**	NA	Intergenic	Intergenic	-1E+06	NM_001081316	319901	Mm.103468	NM_001081316	ENSMUSG00000038702	**Dsel**	9330132E09Rik|DS-epi2	dermatan sulfate epimerase-like	Amp	protein-coding
133_T22	chr1	1.1E+08	1.1E+08	999	+	**1**	**12.5**	NA	Intergenic	Intergenic	-1E+06	NM_001081316	319901	Mm.103468	NM_001081316	ENSMUSG00000038702	**Dsel**	9330132E09Rik|DS-epi2	dermatan sulfate epimerase-like	Amp	protein-coding
134_T22	chr1	1.1E+08	1.1E+08	999	+	**1**	**12.5**	NA	Intergenic	Intergenic	-1E+06	NM_001081316	319901	Mm.103468	NM_001081316	ENSMUSG00000038702	**Dsel**	9330132E09Rik|DS-epi2	dermatan sulfate epimerase-like	Amp	protein-coding
139_T32	chr1	1.1E+08	1.1E+08	999	+	**1**	**12.5**	NA	Intergenic	(CATA)n|Simple_repeat|Simple_repeat	-1E+06	NM_001081316	319901	Mm.103468	NM_001081316	ENSMUSG00000038702	**Dsel**	9330132E09Rik|DS-epi2	dermatan sulfate epimerase-like	Amp	protein-coding
3699_T12	chr9	7078501	7185500	106999	+	**1**	**12.5**	NA	exon (NM_029851, exon 32 of 89)	exon (NM_029851, exon 32 of 89)	45046	NM_029851	110350	Mm.237596	NM_029851	ENSMUSG00000047193	**Dync2h1**	4432416O06Rik|AI448217|D030010H02Rik|D330044F14Rik|DHC1b|DHC2|Dnchc2|b2b414Clo|mKIAA1997	dynein cytoplasmic 2 heavy chain 1	Amp	protein-coding
3694_T12	chr9	6751501	6779500	27999	+	**1**	**12.5**	NA	Intergenic	Intergenic	411546	NM_029851	110350	Mm.237596	NM_029851	ENSMUSG00000047193	**Dync2h1**	4432416O06Rik|AI448217|D030010H02Rik|D330044F14Rik|DHC1b|DHC2|Dnchc2|b2b414Clo|mKIAA1997	dynein cytoplasmic 2 heavy chain 1	Amp	protein-coding
3695_T12	chr9	6785501	6804500	18999	+	**1**	**12.5**	NA	Intergenic	L1_Mus1|LINE|L1	382046	NM_029851	110350	Mm.237596	NM_029851	ENSMUSG00000047193	**Dync2h1**	4432416O06Rik|AI448217|D030010H02Rik|D330044F14Rik|DHC1b|DHC2|Dnchc2|b2b414Clo|mKIAA1997	dynein cytoplasmic 2 heavy chain 1	Amp	protein-coding

3696_T12	chr9	6805501	6816500	10999	+	**1**	**12.5**	NA	Intergenic	RLTR6-int|LTR|ERV1	366046	NM_029851	110350	Mm.237596	NM_029851	ENSMUSG00000047193	**Dync2h1**	4432416O06Rik|AI448217|D030010H02Rik|D330044F14Rik|DHC1b|DHC2|Dnchc2|b2b414Clo|mKIAA1997	dynein cytoplasmic 2 heavy chain 1	Amp	protein-coding
3698_T12	chr9	7072501	7077500	4999	+	**1**	**12.5**	NA	intron (NM_029851, intron 57 of 88)	MTE-int|LTR|ERVL-MaLR	102046	NM_029851	110350	Mm.237596	NM_029851	ENSMUSG00000047193	**Dync2h1**	4432416O06Rik|AI448217|D030010H02Rik|D330044F14Rik|DHC1b|DHC2|Dnchc2|b2b414Clo|mKIAA1997	dynein cytoplasmic 2 heavy chain 1	Amp	protein-coding
3697_T12	chr9	6817501	7063500	245999	+	**1**	**12.5**	NA	intron (NM_029851, intron 87 of 88)	intron (NM_029851, intron 87 of 88)	236546	NM_029851	110350	Mm.237596	NM_029851	ENSMUSG00000047193	**Dync2h1**	4432416O06Rik|AI448217|D030010H02Rik|D330044F14Rik|DHC1b|DHC2|Dnchc2|b2b414Clo|mKIAA1997	dynein cytoplasmic 2 heavy chain 1	Amp	protein-coding
573_T3	chr13	8.8E+07	8.8E+07	4999	+	**1**	**12.5**	NA	Intergenic	RMER6D|LTR|ERVK	-605472	NM_010103	13612	Mm.125580	NM_010103	ENSMUSG00000034488	**Edil3**	Del-1|Del1	EGF-like repeats and discoidin I-like domains 3	Amp	protein-coding
576_T4	chr13	8.9E+07	8.9E+07	4999	+	**1**	**12.5**	NA	Intergenic	Intergenic	-259472	NM_010103	13612	Mm.125580	NM_010103	ENSMUSG00000034488	**Edil3**	Del-1|Del1	EGF-like repeats and discoidin I-like domains 3	Amp	protein-coding
3747_T1	chr9	2.6E+07	2.6E+07	3999	+	**1**	**12.5**	NA	Intergenic	Intergenic	202903	NM_026189	67484	Mm.112977	NM_026189	ENSMUSG00000036611	**Eepd1**	2310005P05Rik|AI481005	endonuclease/exonuclease/phosphatase family domain containing 1	Amp	protein-coding
1291_T4	chr17	6.2E+07	6.2E+07	3999	+	**1**	**12.5**	NA	Intergenic	Intergenic	1371817	NM_207654	13640	Mm.7978	NM_010109	ENSMUSG00000048915	**Efna5**	AL-1|AV158822|EFL-5|Ephrin-A5|Epl7|LERK-7|RAGS	ephrin A5	Amp	protein-coding
3422_T2	chr8	7973501	7974500	999	+	**1**	**12.5**	NA	Intergenic	Intergenic	686773	NM_010111	13642	Mm.209813	NM_010111	ENSMUSG00000001300	**Efnb2**	ELF-2|Epl5|Eplg5|Htk-L|LERK-5|Lerk5|NLERK-1	ephrin B2	Amp	protein-coding
3426_T32	chr8	8198501	8199500	999	+	**1**	**12.5**	NA	Intergenic	Intergenic	461773	NM_010111	13642	Mm.209813	NM_010111	ENSMUSG00000001300	**Efnb2**	ELF-2|Epl5|Eplg5|Htk-L|LERK-5|Lerk5|NLERK-1	ephrin B2	Amp	protein-coding

1114_T2	chr15	6.5E+07	6.5E+07	2999	+	**1**	**12.5**	NA	Intergenic	FordPrefect|DNA|hAT-Tip100	-363041	NM_133766	76740	Mm.260647	NM_133766	ENSMUSG00000015002	**Efr3a**	A130089M23Rik|BB071175|C76891|C920006C10Rik|D030063F01Rik|mKIAA0143	EFR3 homolog A	Amp	protein-coding
1423_T22	chr19	6.1E+07	6.1E+07	999	+	**1**	**12.5**	NA	intron (NM_010123, intron 1 of 21)	intron (NM_010123, intron 1 of 21)	1693	NM_010123	13669	Mm.2238	NM_010123	ENSMUSG00000024991	**Eif3a**	A830012B05Rik|Csma|Eif3|Eif3s10|mKIAA0139	eukaryotic translation initiation factor 3, subunit A	Amp	protein-coding
1167_T32	chr16	6.1E+07	6.1E+07	5999	+	**1**	**12.5**	NA	Intergenic	Lx8|LINE|L1	-689969	NM_007938	13840	Mm.455790	NM_007938	ENSMUSG00000055540	**Epha6**	Ehk2|Hek12|m-ehk2	Eph receptor A6	Amp	protein-coding
2558_T3	chr4	2.9E+07	2.9E+07	999	+	**1**	**12.5**	NA	intron (NM_010141, intron 5 of 16)	intron (NM_010141, intron 5 of 16)	64855	NM_001122889	13841	Mm.257266	NM_010141	ENSMUSG00000028289	**Epha7**	Cek11|Ebk|Ehk3|Hek11|Mdk1	Eph receptor A7	Amp	protein-coding
319_T1	chr10	9.8E+07	9.8E+07	3999	+	**1**	**12.5**	NA	intron (NM_007884, intron 2 of 7)	intron (NM_007884, intron 2 of 7)	5432	NM_007884	13516	Mm.148886	NM_007884	ENSMUSG00000019936	**Epyc**	Dspg3|PG-Lb|SLRR3B	epiphycan	Amp	protein-coding
44_T2	chr1	6.9E+07	6.9E+07	999	+	**1**	**12.5**	NA	intron (NM_010154, intron 2 of 26)	intron (NM_010154, intron 2 of 26)	428059	NM_010154	13869	Mm.442420	NM_010154	ENSMUSG00000062209	**Erbb4**	Her4|c-erbB-4	erb-b2 receptor tyrosine kinase 4	Amp	protein-coding
1180_T42	chr16	9.6E+07	9.6E+07	999	+	**1**	**12.5**	NA	Intergenic	L1Md_T|LINE|L1	85593	NM_011809	23872	Mm.290207	NM_011809	ENSMUSG00000022895	**Ets2**	AU022856|Ets-2	E26 avian leukemia oncogene 2, 3' domain	Amp	protein-coding
424_T3	chr12	3.9E+07	3.9E+07	999	+	**1**	**12.5**	NA	Intergenic	Intergenic	323289	NM_001163154	14009	Mm.4866	NM_007960	ENSMUSG00000004151	**Etv1**	ER81|Etsrp81	ets variant 1	Amp	protein-coding
1153_T42	chr15	7.1E+07	7.1E+07	999	+	**1**	**12.5**	NA	Intergenic	MTEa|LTR|ERVL-MaLR	667838	NM_177819	70363	Mm.126450	NM_177819	ENSMUSG00000036800	**Fam135b**	1700010C24Rik|A830008O07Rik	family with sequence similarity 135, member B	Amp	protein-coding
1155_T3	chr15	7.1E+07	7.1E+07	3999	+	**1**	**12.5**	NA	Intergenic	MLTR11A|LTR|ERVK	406338	NM_177819	70363	Mm.126450	NM_177819	ENSMUSG00000036800	**Fam135b**	1700010C24Rik|A830008O07Rik	family with sequence similarity 135, member B	Amp	protein-coding
529_T1	chr13	7.7E+07	7.7E+07	3999	+	**1**	**12.5**	NA	intron (NM_001145676, intron 19 of 19)	intron (NM_001145676, intron 19 of 19)	-236190	NM_001163420	68675	Mm.343005	NM_138312	ENSMUSG00000064138	**Fam172a**	1110033M05Rik|2610318O14Rik|53-E6|9430037D06Rik|AF064782|pEN87	family with sequence similarity 172, member A	Amp	protein-coding
3769_T12	chr9	8.6E+07	8.6E+07	999	+	**1**	**12.5**	NA	Intergenic	L1MC4|LINE|L1	-193850	NM_001160379	212943	Mm.74610	NM_001160378	ENSMUSG00000032265	**Fam46a**	BAP014|D930050G01Rik	family with sequence similarity 46, member A	Amp	protein-coding

3771_T22	chr9	8.6E+07	8.6E+07	1999	+	**1**	**12.5**	NA	Intergenic	(GAAA)n|Simple_repeat|Simple_repeat	-199350	NM_001160379	212943	Mm.74610	NM_001160378	ENSMUSG00000032265	**Fam46a**	BAP014|D930050G01Rik	family with sequence similarity 46, member A	Amp	protein-coding
3557_T2	chr8	5.7E+07	5.7E+07	3999	+	**1**	**12.5**	NA	Intergenic	Intergenic	228366	NM_015791	50753	Mm.251174	NM_015791	ENSMUSG00000038206	**Fbxo8**	Fbx8	F-box protein 8	Amp	protein-coding
2432_T32	chr3	8.7E+07	8.7E+07	4999	+	**1**	**12.5**	NA	intron (NM_001113238, intron 11 of 11)	L1Md_F3|LINE|L1	37218	NM_001113238	329693	Mm.331122	NM_183222	ENSMUSG00000048031	**Fcrl5**	Fcrh3|mBXMH2	Fc receptor-like 5	Amp	protein-coding
603_T4	chr13	1.2E+08	1.2E+08	6999	+	**1**	**12.5**	NA	Intergenic	L1Md_A|LINE|L1	282301	NM_008002	14165	Mm.317323	NM_008002	ENSMUSG00000021732	**Fgf10**	AEY17|BB213776|Fgf-10|Gsfaey17	fibroblast growth factor 10	Amp	protein-coding
1159_T32	chr16	2.8E+07	2.8E+07	4999	+	**1**	**12.5**	NA	3' UTR (NM_001276419, exon 5 of 5)	3' UTR (NM_001276419, exon 5 of 5)	288075	NM_183064	14167	Mm.7996	NM_010199	ENSMUSG00000022523	**Fgf12**	AV114868|B230343J05Rik|FGF-12|FHF-1|Fhf1	fibroblast growth factor 12	Amp	protein-coding
863_T12	chr14	1.2E+08	1.2E+08	4999	+	**1**	**12.5**	NA	intron (NM_207667, intron 1 of 4)	intron (NM_207667, intron 1 of 4)	-102454	NM_010201	14169	Mm.206362	NM_010201	ENSMUSG00000025551	**Fgf14**	FHF-4|Fhf4|mFHF-4(1B)	fibroblast growth factor 14	Amp	protein-coding
868_T22	chr14	1.2E+08	1.2E+08	999	+	**1**	**12.5**	NA	intron (NM_207667, intron 1 of 4)	intron (NM_207667, intron 1 of 4)	-114454	NM_010201	14169	Mm.206362	NM_010201	ENSMUSG00000025551	**Fgf14**	FHF-4|Fhf4|mFHF-4(1B)	fibroblast growth factor 14	Amp	protein-coding
2441_T32	chr3	9.3E+07	9.3E+07	999	+	**1**	**12.5**	NA	Intergenic	(TG)n|Simple_repeat|Simple_repeat	-22273	NM_001013804	229574	Mm.10755	NM_001013804		**Flg2**	EG229574	filaggrin family member 2	Amp	protein-coding
1609_T32	chr2	1.4E+08	1.4E+08	3999	+	**1**	**12.5**	NA	intron (NM_001013802, intron 3 of 18)	intron (NM_001013802, intron 3 of 18)	54976	NM_001172160	71436	Mm.426781	NM_178382	ENSMUSG00000051379	**Flrt3**	5530600M07Rik|C430047I10Rik|mKIAA1469	fibronectin leucine rich transmembrane protein 3	Amp	protein-coding
622_T2	chr14	7.3E+07	7.3E+07	4999	+	**1**	**12.5**	NA	intron (NM_207636, intron 2 of 25)	intron (NM_207636, intron 2 of 25)	45003	NM_207636	319448	Mm.205421	NM_207636	ENSMUSG00000033487	**Fndc3a**	1700094E19Rik|D14Ertd453e|F730017H24Rik|Fndc3|sys	fibronectin type III domain containing 3A	Amp	protein-coding
2195_T3	chr3	5.2E+07	5.2E+07	3999	+	**1**	**12.5**	NA	Intergenic	IAPEz-int|LTR|ERVK	-36837	NM_019739	56458	Mm.29891	NM_019739	ENSMUSG00000044167	**Foxo1**	AI876417|Afxh|FKHR|Fkhr1|Foxo1a	forkhead box O1	Amp	protein-coding
2893_T32	chr6	1.5E+07	1.5E+07	94999	+	**1**	**12.5**	NA	intron (NM_053242, intron 1 of 18)	B3|SINE|B2	10651	NM_053242	114142	Mm.332919	NM_053242	ENSMUSG00000029563	**Foxp2**	2810043D05Rik|AI449000|CAG-16|D0Kist7	forkhead box P2	Amp	protein-coding
2894_T32	chr6	1.5E+07	1.5E+07	5999	+	**1**	**12.5**	NA	intron (NM_053242, intron 1 of 18)	L1Md_T|LINE|L1	62151	NM_053242	114142	Mm.332919	NM_053242	ENSMUSG00000029563	**Foxp2**	2810043D05Rik|AI449000|CAG-16|D0Kist7	forkhead box P2	Amp	protein-coding

2895_T32	chr6	1.5E+07	1.5E+07	71999	+	**1**	**12.5**	NA	intron (NM_053242, intron 2 of 18)	intron (NM_053242, intron 2 of 18)	102151	NM_053242	114142	Mm.332919	NM_053242	ENSMUSG00000029563	**Foxp2**	2810043D05Rik|AI449000|CAG-16|D0Kist7	forkhead box P2	Amp	protein-coding
2896_T32	chr6	1.5E+07	1.5E+07	101999	+	**1**	**12.5**	NA	intron (NM_053242, intron 4 of 18)	intron (NM_053242, intron 4 of 18)	50994	NM_001286607	114142	Mm.332919	NM_053242	ENSMUSG00000029563	**Foxp2**	2810043D05Rik|AI449000|CAG-16|D0Kist7	forkhead box P2	Amp	protein-coding
2898_T1	chr6	1.5E+07	1.5E+07	999	+	**1**	**12.5**	NA	intron (NM_053242, intron 5 of 18)	intron (NM_053242, intron 5 of 18)	107494	NM_001286607	114142	Mm.332919	NM_053242	ENSMUSG00000029563	**Foxp2**	2810043D05Rik|AI449000|CAG-16|D0Kist7	forkhead box P2	Amp	protein-coding
2899_T32	chr6	1.5E+07	1.5E+07	31999	+	**1**	**12.5**	NA	intron (NM_053242, intron 5 of 18)	intron (NM_053242, intron 5 of 18)	123994	NM_001286607	114142	Mm.332919	NM_053242	ENSMUSG00000029563	**Foxp2**	2810043D05Rik|AI449000|CAG-16|D0Kist7	forkhead box P2	Amp	protein-coding
2900_T12	chr6	1.5E+07	1.5E+07	999	+	**1**	**12.5**	NA	intron (NM_053242, intron 5 of 18)	L1_Mm|LINE|L1	140494	NM_001286607	114142	Mm.332919	NM_053242	ENSMUSG00000029563	**Foxp2**	2810043D05Rik|AI449000|CAG-16|D0Kist7	forkhead box P2	Amp	protein-coding
2902_T12	chr6	1.5E+07	1.5E+07	999	+	**1**	**12.5**	NA	intron (NM_053242, intron 5 of 18)	intron (NM_053242, intron 5 of 18)	147494	NM_001286607	114142	Mm.332919	NM_053242	ENSMUSG00000029563	**Foxp2**	2810043D05Rik|AI449000|CAG-16|D0Kist7	forkhead box P2	Amp	protein-coding
2903_T32	chr6	1.5E+07	1.5E+07	118999	+	**1**	**12.5**	NA	intron (NM_053242, intron 8 of 18)	L1Md_F3|LINE|L1	207494	NM_001286607	114142	Mm.332919	NM_053242	ENSMUSG00000029563	**Foxp2**	2810043D05Rik|AI449000|CAG-16|D0Kist7	forkhead box P2	Amp	protein-coding
238_T1	chr10	3.5E+07	3.5E+07	3999	+	**1**	**12.5**	NA	Intergenic	Intergenic	379100	NM_010237	14302	Mm.332432	NM_010237	ENSMUSG00000019779	**Frk**	BSK|BSK/IYK|C85044|GTK|RAK	fyn-related kinase	Amp	protein-coding
235_T2	chr10	3.5E+07	3.5E+07	999	+	**1**	**12.5**	NA	intron (NM_010237, intron 2 of 8)	intron (NM_010237, intron 2 of 8)	21600	NM_010237	14302	Mm.332432	NM_010237	ENSMUSG00000019779	**Frk**	BSK|BSK/IYK|C85044|GTK|RAK	fyn-related kinase	Amp	protein-coding
2403_T32	chr3	7.7E+07	7.7E+07	8999	+	**1**	**12.5**	NA	Intergenic	B1_Mur3|SINE|Alu	851417	NM_001253719	213262	Mm.379337	NM_178673	ENSMUSG00000034098	**Fstl5**	9130207J01Rik	follistatin-like 5	Amp	protein-coding
2406_T32	chr3	7.7E+07	7.7E+07	8999	+	**1**	**12.5**	NA	Intergenic	MTD|LTR|ERVL-MaLR	1037417	NM_001253719	213262	Mm.379337	NM_178673	ENSMUSG00000034098	**Fstl5**	9130207J01Rik	follistatin-like 5	Amp	protein-coding
2407_T32	chr3	7.7E+07	7.7E+07	2999	+	**1**	**12.5**	NA	Intergenic	L1Md_A|LINE|L1	1069417	NM_001253719	213262	Mm.379337	NM_178673	ENSMUSG00000034098	**Fstl5**	9130207J01Rik	follistatin-like 5	Amp	protein-coding
2410_T32	chr3	7.7E+07	7.7E+07	14999	+	**1**	**12.5**	NA	Intergenic	Intergenic	1084417	NM_001253719	213262	Mm.379337	NM_178673	ENSMUSG00000034098	**Fstl5**	9130207J01Rik	follistatin-like 5	Amp	protein-coding
2411_T2	chr3	7.7E+07	7.7E+07	6999	+	**1**	**12.5**	NA	Intergenic	Intergenic	1205417	NM_001253719	213262	Mm.379337	NM_178673	ENSMUSG00000034098	**Fstl5**	9130207J01Rik	follistatin-like 5	Amp	protein-coding

2412_T32	chr3	7.7E+07	7.7E+07	9999	+	**1**	**12.5**	NA	Intergenic	(GA)n|Simple_repeat|Simple_repeat	1404917	NM_001253719	213262	Mm.379337	NM_178673	ENSMUSG00000034098	**Fstl5**	9130207J01Rik	follistatin-like 5	Amp	protein-coding
2416_T32	chr3	7.7E+07	7.8E+07	19999	+	**1**	**12.5**	NA	Intergenic	L1Md_F2|LINE|L1	1427917	NM_001253719	213262	Mm.379337	NM_178673	ENSMUSG00000034098	**Fstl5**	9130207J01Rik	follistatin-like 5	Amp	protein-coding
2419_T32	chr3	7.8E+07	7.8E+07	999	+	**1**	**12.5**	NA	Intergenic	Lx|LINE|L1	1445417	NM_001253719	213262	Mm.379337	NM_178673	ENSMUSG00000034098	**Fstl5**	9130207J01Rik	follistatin-like 5	Amp	protein-coding
2398_T12	chr3	7.7E+07	7.7E+07	1999	+	**1**	**12.5**	NA	intron (NM_178673, intron 12 of 15)	intron (NM_178673, intron 12 of 15)	568917	NM_001253719	213262	Mm.379337	NM_178673	ENSMUSG00000034098	**Fstl5**	9130207J01Rik	follistatin-like 5	Amp	protein-coding
2395_T32	chr3	7.6E+07	7.6E+07	6999	+	**1**	**12.5**	NA	intron (NM_178673, intron 7 of 15)	intron (NM_178673, intron 7 of 15)	412417	NM_001253719	213262	Mm.379337	NM_178673	ENSMUSG00000034098	**Fstl5**	9130207J01Rik	follistatin-like 5	Amp	protein-coding
2397_T32	chr3	7.6E+07	7.6E+07	1999	+	**1**	**12.5**	NA	intron (NM_178673, intron 7 of 15)	intron (NM_178673, intron 7 of 15)	420917	NM_001253719	213262	Mm.379337	NM_178673	ENSMUSG00000034098	**Fstl5**	9130207J01Rik	follistatin-like 5	Amp	protein-coding
2833_T2	chr5	7.2E+07	7.2E+07	3999	+	**1**	**12.5**	NA	intron (NM_008069, intron 4 of 8)	MLT1D|LTR|ERVL-MaLR	213484	NM_008069	14400	Mm.38567	NM_008069	ENSMUSG00000029212	**Gabrb1**	AW061132|B230208N19Rik|Gabrb-1	gamma-aminobutyric acid (GABA) A receptor, subunit beta 1	Amp	protein-coding
390_T3	chr11	4.3E+07	4.3E+07	999	+	**1**	**12.5**	NA	intron (NM_008070, intron 6 of 9)	intron (NM_008070, intron 6 of 9)	124243	NM_008070	14401	Mm.338723	NM_008070	ENSMUSG00000007653	**Gabrb2**	AI834970|C030002O17Rik|C030021G16Rik|Gabrab2|Gabrb-2	gamma-aminobutyric acid (GABA) A receptor, subunit beta 2	Amp	protein-coding
380_T12	chr11	4.1E+07	4.1E+07	4999	+	**1**	**12.5**	NA	Intergenic	Intergenic	581714	NM_177408	14406	Mm.5309	NM_008073	ENSMUSG00000020436	**Gabrg2**	GABAA-R|Gabrg-2|gamma2	gamma-aminobutyric acid (GABA) A receptor, subunit gamma 2	Amp	protein-coding
381_T2	chr11	4.1E+07	4.1E+07	3999	+	**1**	**12.5**	NA	Intergenic	Intergenic	566214	NM_177408	14406	Mm.5309	NM_008073	ENSMUSG00000020436	**Gabrg2**	GABAA-R|Gabrg-2|gamma2	gamma-aminobutyric acid (GABA) A receptor, subunit gamma 2	Amp	protein-coding
382_T1	chr11	4.2E+07	4.2E+07	3999	+	**1**	**12.5**	NA	intron (NM_177408, intron 4 of 8)	intron (NM_177408, intron 4 of 8)	31214	NM_177408	14406	Mm.5309	NM_008073	ENSMUSG00000020436	**Gabrg2**	GABAA-R|Gabrg-2|gamma2	gamma-aminobutyric acid (GABA) A receptor, subunit gamma 2	Amp	protein-coding
869_T4	chr15	3569501	3570500	999	+	**1**	**12.5**	NA	intron (NM_010284, intron 1 of 10)	MLT1F1|LTR|ERVL-MaLR	11842	NM_001286370	14600	Mm.3986	NM_010284	ENSMUSG00000055737	**Ghr**	GHBP|GHR/BP	growth hormone receptor	Amp	protein-coding
304_T2	chr10	5.7E+07	5.7E+07	3999	+	**1**	**12.5**	NA	Intergenic	Intergenic	318200	NM_010288	14609	Mm.378921	NM_010288	ENSMUSG00000050953	**Gja1**	AU042049|AW546267|Cnx43|Cx43|Cx43alpha1|Gja-1|Npm1|connexin43	gap junction protein, alpha 1	Amp	protein-coding

313_T32	chr10	7.5E+07	7.5E+07	999	+	**1**	**12.5**	NA	Intergenic	Intergenic	-33231	NM_010311	14687	Mm.32595	NM_010311	ENSMUSG00000040009	**Gnaz**	AI847979|Gz	guanine nucleotide binding protein, alpha z subunit	Amp	protein-coding
2831_T2	chr5	7E+07	7E+07	4999	+	**1**	**12.5**	NA	Intergenic	Intergenic	-257715	NM_001038015	67980	Mm.33830	NM_027681	ENSMUSG00000029209	**Gnpda2**	4921523I18Rik|4933412A11Rik|BB189630|GNPDA|Gnp2|Sb52|mKIAA4008	glucosamine-6-phosphate deaminase 2	Amp	protein-coding
3548_T4	chr8	5.5E+07	5.5E+07	1999	+	**1**	**12.5**	NA	intron (NM_001253754, intron 1 of 6)	intron (NM_001253754, intron 1 of 6)	7066	NM_001253754	234267	Mm.241700	NM_153581	ENSMUSG00000031517	**Gpm6a**	Gpm6|M6A	glycoprotein m6a	Amp	protein-coding
3550_T2	chr8	5.5E+07	5.5E+07	8999	+	**1**	**12.5**	NA	intron (NM_001253754, intron 1 of 6)	intron (NM_001253754, intron 1 of 6)	39566	NM_001253754	234267	Mm.241700	NM_153581	ENSMUSG00000031517	**Gpm6a**	Gpm6|M6A	glycoprotein m6a	Amp	protein-coding
3551_T2	chr8	5.5E+07	5.5E+07	3999	+	**1**	**12.5**	NA	intron (NM_001253756, intron 1 of 5)	intron (NM_001253756, intron 1 of 5)	6772	NM_001253756	234267	Mm.241700	NM_153581	ENSMUSG00000031517	**Gpm6a**	Gpm6|M6A	glycoprotein m6a	Amp	protein-coding
3552_T2	chr8	5.5E+07	5.5E+07	7999	+	**1**	**12.5**	NA	intron (NM_001253756, intron 1 of 5)	intron (NM_001253756, intron 1 of 5)	49772	NM_001253756	234267	Mm.241700	NM_153581	ENSMUSG00000031517	**Gpm6a**	Gpm6|M6A	glycoprotein m6a	Amp	protein-coding
3064_T42	chr6	6E+07	6E+07	3999	+	**1**	**12.5**	NA	Intergenic	L1Md_T|LINE|L1	-208210	NM_183183	243385	Mm.138080	NM_183183	ENSMUSG00000045441	**Gprin3**	C030038J10Rik|C730021L23	GPRIN family member 3	Amp	protein-coding
2423_T4	chr3	8.1E+07	8.1E+07	3999	+	**1**	**12.5**	NA	intron (NM_001039195, intron 11 of 12)	intron (NM_001039195, intron 11 of 12)	96291	NM_013540	14800	Mm.220224	NM_013540	ENSMUSG00000033981	**Gria2**	GluA2|GluR-B|Glur-2|Glur2	glutamate receptor, ionotropic, AMPA2 (alpha 2)	Amp	protein-coding
3680_T1	chr9	4713501	4717500	3999	+	**1**	**12.5**	NA	intron (NM_001113180, intron 3 of 16)	(TTTC)n|Simple_repeat|Simple_repeat	80734	NM_001113180	14802	Mm.209263	NM_019691	ENSMUSG00000025892	**Gria4**	GluA4|GluR-D|Glur-4|Glur4|Gluralpha4|spkw1	glutamate receptor, ionotropic, AMPA4 (alpha 4)	Amp	protein-coding
275_T3	chr10	5E+07	5E+07	999	+	**1**	**12.5**	NA	intron (NM_010349, intron 2 of 17)	RMER4B|LTR|ERVK	104754	NM_010349	14806	Mm.332838	NM_010349	ENSMUSG00000056073	**Grik2**	AW124492|GluK2|Glur-6|Glur6|Glurbeta2	glutamate receptor, ionotropic, kainate 2 (beta 2)	Amp	protein-coding
276_T2	chr10	5E+07	5E+07	999	+	**1**	**12.5**	NA	intron (NM_010349, intron 2 of 17)	intron (NM_010349, intron 2 of 17)	70754	NM_010349	14806	Mm.332838	NM_010349	ENSMUSG00000056073	**Grik2**	AW124492|GluK2|Glur-6|Glur6|Glurbeta2	glutamate receptor, ionotropic, kainate 2 (beta 2)	Amp	protein-coding

278_T2	chr10	5E+07	5E+07	999	+	**1**	**12.5**	NA	intron (NM_010349, intron 2 of 17)	MLT1D|LTR|ERVL-MaLR	64754	NM_010349	14806	Mm.332838	NM_010349	ENSMUSG00000056073	**Grik2**	AW124492|GluK2|Glur-6|Glur6|Glurbeta2	glutamate receptor, ionotropic, kainate 2 (beta 2)	Amp	protein-coding
279_T2	chr10	5E+07	5E+07	3999	+	**1**	**12.5**	NA	intron (NM_010349, intron 2 of 17)	intron (NM_010349, intron 2 of 17)	13254	NM_010349	14806	Mm.332838	NM_010349	ENSMUSG00000056073	**Grik2**	AW124492|GluK2|Glur-6|Glur6|Glurbeta2	glutamate receptor, ionotropic, kainate 2 (beta 2)	Amp	protein-coding
3383_T1	chr7	8.8E+07	8.8E+07	3999	+	**1**	**12.5**	NA	intron (NM_001143834, intron 3 of 10)	Lx5b|LINE|L1	25332	NM_001143834	108071	Mm.235018	NM_001033224	ENSMUSG00000049583	**Grm5**	6430542K11Rik|AI850523|Glu5R|Gprc1e|mGluR5|mGluR5b	glutamate receptor, metabotropic 5	Amp	protein-coding
3111_T32	chr6	1.1E+08	1.1E+08	999	+	**1**	**12.5**	NA	Intergenic	AT_rich|Low_complexity|Low_complexity	-853598	NM_177328	108073	Mm.240881	NM_177328	ENSMUSG00000056755	**Grm7**	6330570A01Rik|BB176677|C030018L03|E130018M02Rik|Gpr1g|Gprc1g|SMN2|Tg(SMN2)89Ahmb|mGluR7	glutamate receptor, metabotropic 7	Amp	protein-coding
3113_T12	chr6	1.1E+08	1.1E+08	5999	+	**1**	**12.5**	NA	Intergenic	Intergenic	-755098	NM_177328	108073	Mm.240881	NM_177328	ENSMUSG00000056755	**Grm7**	6330570A01Rik|BB176677|C030018L03|E130018M02Rik|Gpr1g|Gprc1g|SMN2|Tg(SMN2)89Ahmb|mGluR7	glutamate receptor, metabotropic 7	Amp	protein-coding
246_T32	chr10	4.6E+07	4.6E+07	3999	+	**1**	**12.5**	NA	intron (NM_172473, intron 6 of 23)	(TG)n|Simple_repeat|Simple_repeat	48671	NM_172473	209462	Mm.458633	NM_172473	ENSMUSG00000038822	**Hace1**	1700042J16Rik|A730034A22Rik|BC025474	HECT domain and ankyrin repeat containing, E3 ubiquitin protein ligase 1	Amp	protein-coding
1077_T32	chr15	5.7E+07	5.7E+07	999	+	**1**	**12.5**	NA	Intergenic	(TG)n|Simple_repeat|Simple_repeat	-384454	NM_008216	15117	Mm.5148	NM_008216	ENSMUSG00000022367	**Has2**	-	hyaluronan synthase 2	Amp	protein-coding
1623_T42	chr2	1.5E+08	1.5E+08	999	+	**1**	**12.5**	NA	Intergenic	Intergenic	-14468	NM_001172117	15162	Mm.715	NM_010407	ENSMUSG00000003283	**Hck**	AI849071|Bmk	hemopoietic cell kinase	Amp	protein-coding
2718_T1	chr5	1.6E+07	1.6E+07	4999	+	**1**	**12.5**	NA	Intergenic	Intergenic	-70495	NM_001289460	15234	Mm.267078	NM_010427	ENSMUSG00000028864	**Hgf**	C230052L06Rik|HGF/SF|NK1|NK2|SF|SF/HGF	hepatocyte growth factor	Amp	protein-coding
1635_T4	chr3	3155501	3160500	4999	+	**1**	**12.5**	NA	Intergenic	RLTR21|LTR|ERVK	-350030	NM_013920	30942	Mm.330897	NM_013920	ENSMUSG00000017688	**Hnf4g**	NR2A2	hepatocyte nuclear factor 4, gamma	Amp	protein-coding
1637_T4	chr3	3883501	3885500	1999	+	**1**	**12.5**	NA	Intergenic	L1Md_F2|LINE|L1	376470	NM_013920	30942	Mm.330897	NM_013920	ENSMUSG00000017688	**Hnf4g**	NR2A2	hepatocyte nuclear factor 4, gamma	Amp	protein-coding
1640_T3	chr3	3948501	3952500	3999	+	**1**	**12.5**	NA	Intergenic	Intergenic	442470	NM_013920	30942	Mm.330897	NM_013920	ENSMUSG00000017688	**Hnf4g**	NR2A2	hepatocyte nuclear factor 4, gamma	Amp	protein-coding

1641_T1	chr3	3982501	3983500	999	+	**1**	**12.5**	NA	Intergenic	Lx6|LINE|L1	474970	NM_013920	30942	Mm.330897	NM_013920	ENSMUSG00000017688	**Hnf4g**	NR2A2	hepatocyte nuclear factor 4, gamma	Amp	protein-coding
243_T42	chr10	3.6E+07	3.6E+07	3999	+	**1**	**12.5**	NA	Intergenic	Intergenic	-590307	NM_001253355	319415	Mm.332442	NM_001081208	ENSMUSG00000044499	**Hs3st5**	D930005L05Rik|Gm1151|Hs3ost5	heparan sulfate (glucosamine) 3-O-sulfotransferase 5	Amp	protein-coding
244_T1	chr10	3.7E+07	3.7E+07	7999	+	**1**	**12.5**	NA	intron (NM_001081208, intron 2 of 3)	RMER19C|LTR|ERVK	178693	NM_001253356	319415	Mm.332442	NM_001081208	ENSMUSG00000044499	**Hs3st5**	D930005L05Rik|Gm1151|Hs3ost5	heparan sulfate (glucosamine) 3-O-sulfotransferase 5	Amp	protein-coding
591_T2	chr13	1.1E+08	1.1E+08	999	+	**1**	**12.5**	NA	Intergenic	Intergenic	565307	NM_008308	15550	Mm.4716	NM_008308	ENSMUSG00000021721	**Htr1a**	Gpcr18	5-hydroxytryptamine (serotonin) receptor 1A	Amp	protein-coding
2358_T32	chr3	5.9E+07	5.9E+07	6999	+	**1**	**12.5**	NA	Intergenic	Lx7|LINE|L1	-69744	NM_001162884	242050	Mm.228086	NM_001162884	ENSMUSG00000036334	**Igsf10**	6530405F15Rik|9030224D03|AA409708|AA536958|AI414626|Adlican2|CMF608	immunoglobulin superfamily, member 10	Amp	protein-coding
2359_T32	chr3	5.9E+07	5.9E+07	7999	+	**1**	**12.5**	NA	Intergenic	Intergenic	-78244	NM_001162884	242050	Mm.228086	NM_001162884	ENSMUSG00000036334	**Igsf10**	6530405F15Rik|9030224D03|AA409708|AA536958|AI414626|Adlican2|CMF608	immunoglobulin superfamily, member 10	Amp	protein-coding
2360_T32	chr3	5.9E+07	5.9E+07	15999	+	**1**	**12.5**	NA	Intergenic	L1_Mus2|LINE|L1	-91244	NM_001162884	242050	Mm.228086	NM_001162884	ENSMUSG00000036334	**Igsf10**	6530405F15Rik|9030224D03|AA409708|AA536958|AI414626|Adlican2|CMF608	immunoglobulin superfamily, member 10	Amp	protein-coding
595_T22	chr13	1.2E+08	1.2E+08	999	+	**1**	**12.5**	NA	Intergenic	Intergenic	396688	NM_021459	16392	Mm.42242	NM_021459	ENSMUSG00000042258	**Isl1**	-	ISL1 transcription factor, LIM/homeodomain	Amp	protein-coding
2643_T2	chr4	9.2E+07	9.2E+07	3999	+	**1**	**12.5**	NA	Intergenic	Intergenic	-249279	NM_027034	69314	Mm.55891	NM_027034	ENSMUSG00000028533	**Izumo3**	1700011H22Rik	IZUMO family member 3	Amp	protein-coding
1602_T42	chr2	1.4E+08	1.4E+08	3999	+	**1**	**12.5**	NA	Intergenic	Intergenic	-381980	NM_013822	16449	Mm.22398	NM_013822	ENSMUSG00000027276	**Jag1**	ABE2|Gsfabe2|Htu|Ozz|Ser-1	jagged 1	Amp	protein-coding
1579_T2	chr2	1.1E+08	1.1E+08	3999	+	**1**	**12.5**	NA	Intergenic	Intergenic	128911	NM_021275	16492	Mm.142718	NM_021275	ENSMUSG00000042604	**Kcna4**	Kv1.4	potassium voltage-gated channel, shaker-related subfamily, member 4	Amp	protein-coding
1583_T4	chr2	1.1E+08	1.1E+08	999	+	**1**	**12.5**	NA	Intergenic	MER5B|DNA|hAT-Charlie	867411	NM_021275	16492	Mm.142718	NM_021275	ENSMUSG00000042604	**Kcna4**	Kv1.4	potassium voltage-gated channel, shaker-related subfamily, member 4	Amp	protein-coding

1585_T1	chr2	1.1E+08	1.1E+08	999	+	**1**	**12.5**	NA	Intergenic	Intergenic	871411	NM_021275	16492	Mm.142718	NM_021275	ENSMUSG00000042604	**Kcna4**	Kv1.4	potassium voltage-gated channel, shaker-related subfamily, member 4	Amp	protein-coding
2761_T22	chr5	4.9E+07	4.9E+07	999	+	**1**	**12.5**	NA	Intergenic	MLT2B2|LTR|ERVL	-103341	NM_001199244	80334	Mm.160172	NM_030265	ENSMUSG00000029088	**Kcnip4**	AV032399|Calp|Calp250|KChIP4|KChIP4a	Kv channel interacting protein 4	Amp	protein-coding
2757_T32	chr5	4.9E+07	4.9E+07	999	+	**1**	**12.5**	NA	intron (NM_001199244, intron 1 of 7)	intron (NM_001199244, intron 1 of 7)	33557	NM_030265	80334	Mm.160172	NM_030265	ENSMUSG00000029088	**Kcnip4**	AV032399|Calp|Calp250|KChIP4|KChIP4a	Kv channel interacting protein 4	Amp	protein-coding
2760_T2	chr5	4.9E+07	4.9E+07	1999	+	**1**	**12.5**	NA	intron (NM_001199244, intron 1 of 7)	intron (NM_001199244, intron 1 of 7)	2057	NM_030265	80334	Mm.160172	NM_030265	ENSMUSG00000029088	**Kcnip4**	AV032399|Calp|Calp250|KChIP4|KChIP4a	Kv channel interacting protein 4	Amp	protein-coding
1494_T2	chr2	5.5E+07	5.5E+07	999	+	**1**	**12.5**	NA	Intergenic	Intergenic	-313970	NM_001304810	16519	Mm.5127	NM_008426	ENSMUSG00000026824	**Kcnj3**	GIRK-1|GIRK1|Kcnf3|Kir3.1	potassium inwardly-rectifying channel, subfamily J, member 3	Amp	protein-coding
1498_T32	chr2	5.5E+07	5.5E+07	999	+	**1**	**12.5**	NA	intron (NM_008426, intron 3 of 3)	intron (NM_008426, intron 3 of 3)	28030	NM_008426	16519	Mm.5127	NM_008426	ENSMUSG00000026824	**Kcnj3**	GIRK-1|GIRK1|Kcnf3|Kir3.1	potassium inwardly-rectifying channel, subfamily J, member 3	Amp	protein-coding
3394_T2	chr7	1.4E+08	1.4E+08	999	+	**1**	**12.5**	NA	non-coding (NR_001461, exon 1 of 1)	non-coding (NR_001461, exon 1 of 1)	47547	NR_001461	63830	Mm.356096	NR_001461	ENSMUSG00000101609	**Kcnq1ot1**	Kvlqt1-as|Lit1|Tssc8	KCNQ1 overlapping transcript 1	Amp	ncRNA
988_T42	chr15	4.5E+07	4.5E+07	999	+	**1**	**12.5**	NA	Intergenic	L1_Mus3|LINE|L1	-146066	NM_026200	67498	Mm.300079	NM_026200	ENSMUSG00000022342	**Kcnv1**	2700023A03Rik|vibe	potassium channel, subfamily V, member 1	Amp	protein-coding
989_T2	chr15	4.6E+07	4.6E+07	999	+	**1**	**12.5**	NA	Intergenic	Intergenic	-386066	NM_026200	67498	Mm.300079	NM_026200	ENSMUSG00000022342	**Kcnv1**	2700023A03Rik|vibe	potassium channel, subfamily V, member 1	Amp	protein-coding
992_T22	chr15	4.6E+07	4.6E+07	999	+	**1**	**12.5**	NA	Intergenic	Intergenic	-392066	NM_026200	67498	Mm.300079	NM_026200	ENSMUSG00000022342	**Kcnv1**	2700023A03Rik|vibe	potassium channel, subfamily V, member 1	Amp	protein-coding
407_T2	chr11	9.2E+07	9.2E+07	999	+	**1**	**12.5**	NA	Intergenic	Intergenic	-511445	NM_028547	73470	Mm.67677	NM_028547	ENSMUSG00000046755	**Kif2b**	1700063D03Rik	kinesin family member 2B	Amp	protein-coding
791_T1	chr14	9.9E+07	9.9E+07	15999	+	**1**	**12.5**	NA	Intergenic	Intergenic	172809	NM_009769	12224	Mm.30262	NM_009769	ENSMUSG00000005148	**Klf5**	4930520J07Rik|Bteb2|CKLF|IKLF	Kruppel-like factor 5	Amp	protein-coding
774_T1	chr14	9.7E+07	9.7E+07	41999	+	**1**	**12.5**	NA	Intergenic	L1Md_F2|LINE|L1	-184466	NM_053105	93688	Mm.308735	NM_053105	ENSMUSG00000022076	**Klhl1**	mKIAA1490	kelch-like 1	Amp	protein-coding
775_T3	chr14	9.7E+07	9.7E+07	1999	+	**1**	**12.5**	NA	Intergenic	Intergenic	-289466	NM_053105	93688	Mm.308735	NM_053105	ENSMUSG00000022076	**Klhl1**	mKIAA1490	kelch-like 1	Amp	protein-coding

779_T3	chr14	9.7E+07	9.7E+07	3999	+	**1**	**12.5**	NA	Intergenic	Intergenic	-300466	NM_053105	93688	Mm.308735	NM_053105	ENSMUSG00000022076	**Klhl1**	mKIAA1490	kelch-like 1	Amp	protein-coding
764_T2	chr14	9.6E+07	9.6E+07	1999	+	**1**	**12.5**	NA	intron (NM_053105, intron 1 of 10)	L1_Mur2|LINE|L1	135534	NM_053105	93688	Mm.308735	NM_053105	ENSMUSG00000022076	**Klhl1**	mKIAA1490	kelch-like 1	Amp	protein-coding
769_T1	chr14	9.6E+07	9.6E+07	999	+	**1**	**12.5**	NA	intron (NM_053105, intron 1 of 10)	intron (NM_053105, intron 1 of 10)	114034	NM_053105	93688	Mm.308735	NM_053105	ENSMUSG00000022076	**Klhl1**	mKIAA1490	kelch-like 1	Amp	protein-coding
770_T32	chr14	9.6E+07	9.6E+07	999	+	**1**	**12.5**	NA	intron (NM_053105, intron 1 of 10)	intron (NM_053105, intron 1 of 10)	97034	NM_053105	93688	Mm.308735	NM_053105	ENSMUSG00000022076	**Klhl1**	mKIAA1490	kelch-like 1	Amp	protein-coding
3595_T4	chr8	7.4E+07	7.4E+07	999	+	**1**	**12.5**	NA	Intergenic	L1_Mur2|LINE|L1	-644444	NM_010687	16795	Mm.324371	NM_010687	ENSMUSG00000004383	**Large1**	BPFD#36|Gyltl1a|Large|Mbp-1|Mbp1|enr|fg|froggy|myd	LARGE xylosyl- and glucuronyltransferase 1	Amp	protein-coding
3597_T4	chr8	7.4E+07	7.4E+07	8999	+	**1**	**12.5**	NA	Intergenic	MTD|LTR|ERVL-MaLR	-758444	NM_010687	16795	Mm.324371	NM_010687	ENSMUSG00000004383	**Large1**	BPFD#36|Gyltl1a|Large|Mbp-1|Mbp1|enr|fg|froggy|myd	LARGE xylosyl- and glucuronyltransferase 1	Amp	protein-coding
2585_T42	chr4	3.7E+07	3.7E+07	999	+	**1**	**12.5**	NA	Intergenic	MTEa-int|LTR|ERVL-MaLR	-117256	NM_001165999	242384	Mm.132507	NM_175516	ENSMUSG00000045083	**Lingo2**	B230217C06Rik|Lern3|Lrrn6c	leucine rich repeat and Ig domain containing 2	Amp	protein-coding
2583_T32	chr4	3.7E+07	3.7E+07	3999	+	**1**	**12.5**	NA	intron (NM_001165999, intron 1 of 7)	intron (NM_001165999, intron 1 of 7)	-99537	NM_001166000	242384	Mm.132507	NM_175516	ENSMUSG00000045083	**Lingo2**	B230217C06Rik|Lern3|Lrrn6c	leucine rich repeat and Ig domain containing 2	Amp	protein-coding
2575_T32	chr4	3.6E+07	3.6E+07	4999	+	**1**	**12.5**	NA	intron (NM_001166000, intron 4 of 8)	intron (NM_001166000, intron 4 of 8)	-398796	NM_175516	242384	Mm.132507	NM_175516	ENSMUSG00000045083	**Lingo2**	B230217C06Rik|Lern3|Lrrn6c	leucine rich repeat and Ig domain containing 2	Amp	protein-coding
2574_T22	chr4	3.6E+07	3.6E+07	5999	+	**1**	**12.5**	NA	intron (NM_175516, intron 1 of 2)	intron (NM_175516, intron 1 of 2)	4704	NM_001166001	242384	Mm.132507	NM_175516	ENSMUSG00000045083	**Lingo2**	B230217C06Rik|Lern3|Lrrn6c	leucine rich repeat and Ig domain containing 2	Amp	protein-coding
1345_T22	chr19	3.4E+07	3.4E+07	3999	+	**1**	**12.5**	NA	Intergenic	Intergenic	-182748	NM_026334	67717	Mm.329816	NM_026334	ENSMUSG00000024768	**Lipf**	2310051B21Rik|AV082900	lipase, gastric	Amp	protein-coding
445_T32	chr12	6.2E+07	6.2E+07	999	+	**1**	**12.5**	NA	intron (NM_001310586, intron 1 of 5)	Lx|LINE|L1	91833	NM_001310586	238205	Mm.28802	NM_178714	ENSMUSG00000035653	**Lrfn5**	AI427653|AI604817|C130061B21|mKIAA4208	leucine rich repeat and fibronectin type III domain containing 5	Amp	protein-coding
1464_T22	chr2	4.3E+07	4.3E+07	3999	+	**1**	**12.5**	NA	Intergenic	Intergenic	-227902	NM_053011	94217	Mm.441398	NM_053011	ENSMUSG00000049252	**Lrp1b**	9630004P12Rik|LRP-DIT	low density lipoprotein-related protein 1B (deleted in tumors)	Amp	protein-coding

1470_T42	chr2	4.3E+07	4.3E+07	3999	+	**1**	**12.5**	NA	Intergenic	L1_Mur1|LINE|L1	-318902	NM_053011	94217	Mm.441398	NM_053011	ENSMUSG00000049252	**Lrp1b**	9630004P12Rik|LRP-DIT	low density lipoprotein-related protein 1B (deleted in tumors)	Amp	protein-coding
1568_T4	chr2	9.9E+07	9.9E+07	6999	+	**1**	**12.5**	NA	Intergenic	Intergenic	1276343	NM_178725	241568	Mm.241682	NM_178725	ENSMUSG00000050587	**Lrrc4c**	6430556C10Rik|NGL-1	leucine rich repeat containing 4C	Amp	protein-coding
1569_T42	chr2	9.9E+07	9.9E+07	999	+	**1**	**12.5**	NA	Intergenic	Intergenic	1669343	NM_178725	241568	Mm.241682	NM_178725	ENSMUSG00000050587	**Lrrc4c**	6430556C10Rik|NGL-1	leucine rich repeat containing 4C	Amp	protein-coding
1571_T42	chr2	9.9E+07	9.9E+07	1999	+	**1**	**12.5**	NA	Intergenic	Intergenic	1676843	NM_178725	241568	Mm.241682	NM_178725	ENSMUSG00000050587	**Lrrc4c**	6430556C10Rik|NGL-1	leucine rich repeat containing 4C	Amp	protein-coding
1572_T4	chr2	9.9E+07	9.9E+07	4999	+	**1**	**12.5**	NA	Intergenic	RMER19B2|LTR|ERVK	1844343	NM_178725	241568	Mm.241682	NM_178725	ENSMUSG00000050587	**Lrrc4c**	6430556C10Rik|NGL-1	leucine rich repeat containing 4C	Amp	protein-coding
1545_T4	chr2	9.7E+07	9.7E+07	999	+	**1**	**12.5**	NA	intron (NM_001289742, intron 3 of 7)	BC1_Mm|scRNA|scRNA	-385657	NM_001289744	241568	Mm.241682	NM_178725	ENSMUSG00000050587	**Lrrc4c**	6430556C10Rik|NGL-1	leucine rich repeat containing 4C	Amp	protein-coding
1552_T3	chr2	9.8E+07	9.8E+07	999	+	**1**	**12.5**	NA	intron (NM_001289742, intron 7 of 7)	intron (NM_001289742, intron 7 of 7)	137343	NM_178725	241568	Mm.241682	NM_178725	ENSMUSG00000050587	**Lrrc4c**	6430556C10Rik|NGL-1	leucine rich repeat containing 4C	Amp	protein-coding
2514_T32	chr3	1.6E+08	1.6E+08	3999	+	**1**	**12.5**	NA	Intergenic	L1_Mur3|LINE|L1	-17279	NM_001081358	242274	Mm.132162	NM_001081358	ENSMUSG00000028176	**Lrrc7**	AK122498|densin|mKIAA1365	leucine rich repeat containing 7	Amp	protein-coding
2511_T1	chr3	1.6E+08	1.6E+08	1999	+	**1**	**12.5**	NA	intron (NM_001081358, intron 3 of 27)	L1MA9|LINE|L1	180721	NM_001291453	242274	Mm.132162	NM_001081358	ENSMUSG00000028176	**Lrrc7**	AK122498|densin|mKIAA1365	leucine rich repeat containing 7	Amp	protein-coding
3082_T32	chr6	7.5E+07	7.5E+07	3999	+	**1**	**12.5**	NA	Intergenic	Intergenic	-2E+06	NM_028880	74342	Mm.292568	NM_028880	ENSMUSG00000060780	**Lrrtm1**	4632401D06Rik|AW125451	leucine rich repeat transmembrane neuronal 1	Amp	protein-coding
3083_T12	chr6	7.7E+07	7.7E+07	3999	+	**1**	**12.5**	NA	Intergenic	Intergenic	-420217	NM_028880	74342	Mm.292568	NM_028880	ENSMUSG00000060780	**Lrrtm1**	4632401D06Rik|AW125451	leucine rich repeat transmembrane neuronal 1	Amp	protein-coding
2964_T32	chr6	1.9E+07	1.9E+07	3999	+	**1**	**12.5**	NA	Intergenic	IAPEz-int|LTR|ERVK	-88135	NM_133939	76522	Mm.275158	NM_133939	ENSMUSG00000044155	**Lsm8**	2010003I05Rik|AW214405|Naa38	LSM8 homolog, U6 small nuclear RNA associated	Amp	protein-coding
2634_T2	chr4	8.1E+07	8.1E+07	3999	+	**1**	**12.5**	NA	Intergenic	ORR1E-int|LTR|ERVL-MaLR	79814	NM_026821	52829	Mm.273986	NM_026821	ENSMUSG00000048706	**Lurap1l**	1110029A09Rik|AV077978|AV175137|D4Bwg0951e|WI-13707|bM350F23.1	leucine rich adaptor protein 1-like	Amp	protein-coding

1606_T3	chr2	1.4E+08	1.4E+08	999	+	**1**	**12.5**	NA	intron (NM_001013802, intron 2 of 18)	intron (NM_001013802, intron 2 of 18)	14565	NM_028387	72899	Mm.229466	NM_028387	ENSMUSG00000068205	**Macrod2**	1110033L15Rik|2610107G07Rik|2900006F19Rik	MACRO domain containing 2	Amp	protein-coding
1608_T12	chr2	1.4E+08	1.4E+08	999	+	**1**	**12.5**	NA	intron (NM_001013802, intron 2 of 18)	intron (NM_001013802, intron 2 of 18)	18565	NM_028387	72899	Mm.229466	NM_028387	ENSMUSG00000068205	**Macrod2**	1110033L15Rik|2610107G07Rik|2900006F19Rik	MACRO domain containing 2	Amp	protein-coding
2191_T3	chr3	5.2E+07	5.2E+07	9999	+	**1**	**12.5**	NA	intron (NM_001004176, intron 1 of 4)	IAPEz-int|LTR|ERVK	174506	NM_001004176	433586	Mm.234972	NM_001004176	ENSMUSG00000061143	**Maml3**	AV234550|BC049812|Mam-2|mKIAA1816	mastermind like 3 (Drosophila)	Amp	protein-coding
2555_T4	chr4	2.6E+07	2.6E+07	999	+	**1**	**12.5**	NA	Intergenic	L1Md_F2|LINE|L1	248652	NM_172865	242362	Mm.245602	NM_172865	ENSMUSG00000040520	**Manea**	4932703L02Rik	mannosidase, endo-alpha	Amp	protein-coding
1330_T42	chr18	2.4E+07	2.4E+07	3999	+	**1**	**12.5**	NA	intron (NM_001162942, intron 2 of 7)	L1Md_T|LINE|L1	-12470	NM_153058	212307	Mm.132237	NM_153058	ENSMUSG00000024277	**Mapre2**	AI314113|C820009F03Rik|D18Ab b1e|EB1|EB2|RP1	microtubule-associated protein, RP/EB family, member 2	Amp	protein-coding
2364_T1	chr3	6.1E+07	6.1E+07	999	+	**1**	**12.5**	NA	Intergenic	Intergenic	144821	NM_001253708	56758	Mm.255723	NM_020007	ENSMUSG00000027763	**Mbnl1**	Mbnl|mKIAA0428	muscleblind-like 1 (Drosophila)	Amp	protein-coding
2904_T32	chr6	1.5E+07	1.5E+07	3999	+	**1**	**12.5**	NA	Intergenic	L1Md_A|LINE|L1	-265161	NM_175088	16543	Mm.1314	NM_175088	ENSMUSG00000041390	**Mdfic**	Kdt1|Mdfid	MyoD family inhibitor domain containing	Amp	protein-coding
2905_T32	chr6	1.5E+07	1.6E+07	58999	+	**1**	**12.5**	NA	Intergenic	Intergenic	-232661	NM_175088	16543	Mm.1314	NM_175088	ENSMUSG00000041390	**Mdfic**	Kdt1|Mdfid	MyoD family inhibitor domain containing	Amp	protein-coding
2906_T32	chr6	1.6E+07	1.6E+07	3999	+	**1**	**12.5**	NA	Intergenic	L1Md_A|LINE|L1	-200161	NM_175088	16543	Mm.1314	NM_175088	ENSMUSG00000041390	**Mdfic**	Kdt1|Mdfid	MyoD family inhibitor domain containing	Amp	protein-coding
2907_T32	chr6	1.6E+07	1.6E+07	139999	+	**1**	**12.5**	NA	Intergenic	L1_Mus3|LINE|L1	-127161	NM_175088	16543	Mm.1314	NM_175088	ENSMUSG00000041390	**Mdfic**	Kdt1|Mdfid	MyoD family inhibitor domain containing	Amp	protein-coding
2909_T32	chr6	1.6E+07	1.6E+07	6999	+	**1**	**12.5**	NA	Intergenic	L1Md_T|LINE|L1	581339	NM_175088	16543	Mm.1314	NM_175088	ENSMUSG00000041390	**Mdfic**	Kdt1|Mdfid	MyoD family inhibitor domain containing	Amp	protein-coding
2908_T32	chr6	1.6E+07	1.6E+07	114999	+	**1**	**12.5**	NA	intron (NM_175088, intron 2 of 4)	MER20|DNA|hAT-Charlie	4339	NM_175088	16543	Mm.1314	NM_175088	ENSMUSG00000041390	**Mdfic**	Kdt1|Mdfid	MyoD family inhibitor domain containing	Amp	protein-coding
1070_T1	chr15	5.3E+07	5.3E+07	3999	+	**1**	**12.5**	NA	Intergenic	Lx8b|LINE|L1	-23945	NM_027212	69790	Mm.195562	NM_027212	ENSMUSG00000038622	**Med30**	1810038N03Rik|2510044J04Rik|TRAP25|Thrap6	mediator complex subunit 30	Amp	protein-coding
556_T2	chr13	8.3E+07	8.3E+07	999	+	**1**	**12.5**	NA	Intergenic	L1Md_F2|LINE|L1	-273034	NM_001170537	17260	Mm.24001	NM_025282	ENSMUSG00000005583	**Mef2c**	5430401D19Rik|9930028G15Rik|AV011172|Mef2	myocyte enhancer factor 2C	Amp	protein-coding

3765_T12	chr9	8.2E+07	8.2E+07	3999	+	**1**	**12.5**	NA	Intergenic	Lx9|LINE|L1	315756	NM_175213	75033	Mm.335329	NM_175213	ENSMUSG00000043289	**Mei4**	4930486G11Rik	meiotic double-stranded break formation protein 4	Amp	protein-coding
1587_T42	chr2	1.1E+08	1.1E+08	999	+	**1**	**12.5**	NA	Intergenic	Lx7|LINE|L1	207290	NM_029790	76894	Mm.330451	NM_029790	ENSMUSG00000057234	**Mettl15**	0610027B03Rik|AI303697|AW049952|Mett5d1	methyltransferase like 15	Amp	protein-coding
327_T42	chr10	1E+08	1E+08	9999	+	**1**	**12.5**	NA	Intergenic	(GA)n|Simple_repeat|Simple_repeat	-542987	NM_001205098	67569	Mm.25252	NM_026243	ENSMUSG00000019888	**Mgat4c**	9130411I17Rik|GntIVh	MGAT4 family, member C	Amp	protein-coding
330_T42	chr10	1E+08	1E+08	30999	+	**1**	**12.5**	NA	Intergenic	L1_Mus2|LINE|L1	-501487	NM_001205098	67569	Mm.25252	NM_026243	ENSMUSG00000019888	**Mgat4c**	9130411I17Rik|GntIVh	MGAT4 family, member C	Amp	protein-coding
2186_T3	chr3	5.2E+07	5.2E+07	6999	+	**1**	**12.5**	NA	Intergenic	IAPEz-int|LTR|ERVK	-42179	NM_174995	211666	Mm.24679	NM_174995	ENSMUSG00000074604	**Mgst2**	GST2|MGST-II	microsomal glutathione S-transferase 2	Amp	protein-coding
3464_T2	chr8	3.8E+07	3.8E+07	5999	+	**1**	**12.5**	NA	intron (NM_145841, intron 1 of 7)	L1Md_F2|LINE|L1	241702	NR_029884	723860		NR_029884	ENSMUSG00000065457	**Mir383**	Mirn383|mir-383|mmu-mir-383	microRNA 383	Amp	ncRNA
3468_T32	chr8	3.8E+07	3.8E+07	999	+	**1**	**12.5**	NA	intron (NM_145841, intron 1 of 7)	intron (NM_145841, intron 1 of 7)	-10798	NR_029884	723860		NR_029884	ENSMUSG00000065457	**Mir383**	Mirn383|mir-383|mmu-mir-383	microRNA 383	Amp	ncRNA
3473_T1	chr8	3.8E+07	3.8E+07	4999	+	**1**	**12.5**	NA	intron (NM_145841, intron 1 of 7)	intron (NM_145841, intron 1 of 7)	-120798	NR_029884	723860		NR_029884	ENSMUSG00000065457	**Mir383**	Mirn383|mir-383|mmu-mir-383	microRNA 383	Amp	ncRNA
3454_T2	chr8	3.8E+07	3.8E+07	999	+	**1**	**12.5**	NA	intron (NM_145841, intron 2 of 7)	B2_Mm2|SINE|B2	348202	NR_029884	723860		NR_029884	ENSMUSG00000065457	**Mir383**	Mirn383|mir-383|mmu-mir-383	microRNA 383	Amp	ncRNA
3461_T22	chr8	3.8E+07	3.8E+07	4999	+	**1**	**12.5**	NA	intron (NM_145841, intron 2 of 7)	intron (NM_145841, intron 2 of 7)	324202	NR_029884	723860		NR_029884	ENSMUSG00000065457	**Mir383**	Mirn383|mir-383|mmu-mir-383	microRNA 383	Amp	ncRNA
2576_T42	chr4	3.6E+07	3.6E+07	999	+	**1**	**12.5**	NA	intron (NM_001166000, intron 1 of 8)	intron (NM_001166000, intron 1 of 8)	185453	NR_030545	1E+08		NR_030545	ENSMUSG00000077851	**Mir876**	Mirn876|mir-876|mmu-mir-876	microRNA 876	Amp	ncRNA
2580_T42	chr4	3.7E+07	3.7E+07	999	+	**1**	**12.5**	NA	intron (NM_001166000, intron 1 of 8)	intron (NM_001166000, intron 1 of 8)	90453	NR_030545	1E+08		NR_030545	ENSMUSG00000077851	**Mir876**	Mirn876|mir-876|mmu-mir-876	microRNA 876	Amp	ncRNA
2369_T3	chr3	6.7E+07	6.7E+07	999	+	**1**	**12.5**	NA	intron (NM_025822, intron 5 of 9)	intron (NM_025822, intron 5 of 9)	-181097	NM_010801	17349	Mm.10414	NM_010801	ENSMUSG00000048416	**Mlf1**	HLS7	myeloid leukemia factor 1	Amp	protein-coding

3707_T12	chr9	7483501	7498500	14999	+	**1**	**12.5**	NA	Intergenic	MuRRS-int|LTR|ERV1	-11342	NM_019471	17384	Mm.14126	NM_019471	ENSMUSG00000047562	**Mmp10**	AV377895|MMP-10|SL-2	matrix metallopeptidase 10	Amp	protein-coding
3701_T12	chr9	7248501	7371500	122999	+	**1**	**12.5**	NA	Intergenic	RMER16-int|LTR|ERVK	-37374	NM_008605	17381	Mm.2055	NM_008605	ENSMUSG00000049723	**Mmp12**	AV378681|MME|Mmel	matrix metallopeptidase 12	Amp	protein-coding
2534_T3	chr4	1.8E+07	1.8E+07	999	+	**1**	**12.5**	NA	Intergenic	Intergenic	-207482	NM_019724	17389	Mm.187315	NM_019724	ENSMUSG00000028226	**Mmp16**	MT-MMP3|MT3-MMP|Mt3mmp	matrix metallopeptidase 16	Amp	protein-coding
2535_T2	chr4	1.8E+07	1.8E+07	5999	+	**1**	**12.5**	NA	intron (NM_019724, intron 1 of 9)	intron (NM_019724, intron 1 of 9)	123018	NM_019724	17389	Mm.187315	NM_019724	ENSMUSG00000028226	**Mmp16**	MT-MMP3|MT3-MMP|Mt3mmp	matrix metallopeptidase 16	Amp	protein-coding
2536_T2	chr4	1.8E+07	1.8E+07	999	+	**1**	**12.5**	NA	intron (NM_019724, intron 4 of 9)	intron (NM_019724, intron 4 of 9)	160518	NM_019724	17389	Mm.187315	NM_019724	ENSMUSG00000028226	**Mmp16**	MT-MMP3|MT3-MMP|Mt3mmp	matrix metallopeptidase 16	Amp	protein-coding
2538_T22	chr4	1.8E+07	1.8E+07	1999	+	**1**	**12.5**	NA	intron (NM_019724, intron 4 of 9)	intron (NM_019724, intron 4 of 9)	166018	NM_019724	17389	Mm.187315	NM_019724	ENSMUSG00000028226	**Mmp16**	MT-MMP3|MT3-MMP|Mt3mmp	matrix metallopeptidase 16	Amp	protein-coding
3703_T12	chr9	7414501	7430500	15999	+	**1**	**12.5**	NA	Intergenic	Intergenic	-23322	NM_010809	17392	Mm.4993	NM_010809	ENSMUSG00000043613	**Mmp3**	EMS-2|MMP-3|SL-1|SLN-1|SLN1|STR-1|Stmy1|Str1	matrix metallopeptidase 3	Amp	protein-coding
3705_T12	chr9	7435501	7436500	999	+	**1**	**12.5**	NA	Intergenic	L1_Mus1|LINE|L1	-9822	NM_010809	17392	Mm.4993	NM_010809	ENSMUSG00000043613	**Mmp3**	EMS-2|MMP-3|SL-1|SLN-1|SLN1|STR-1|Stmy1|Str1	matrix metallopeptidase 3	Amp	protein-coding
3708_T12	chr9	7499501	7609500	109999	+	**1**	**12.5**	NA	Intergenic	GA-rich|Low_complexity|Low_complexity	-3929	NM_008611	17394	Mm.16415	NM_008611	ENSMUSG00000005800	**Mmp8**	BB138268	matrix metallopeptidase 8	Amp	protein-coding
2550_T3	chr4	2.4E+07	2.4E+07	3999	+	**1**	**12.5**	NA	Intergenic	Lx7|LINE|L1	-795962	NM_199467	212377	Mm.120919	NM_199467	ENSMUSG00000045751	**Mms22l**	F730047E07Rik|Gm134	MMS22-like, DNA repair protein	Amp	protein-coding
3040_T42	chr6	3.5E+07	3.5E+07	3999	+	**1**	**12.5**	NA	Intergenic	L1Md_A|LINE|L1	103388	NM_008098	14489	Mm.182746	NM_008098	ENSMUSG00000029840	**Mtpn**	5033418D15Rik|Gcdp|V1	myotrophin	Amp	protein-coding
1430_T32	chr2	2.2E+07	2.2E+07	3999	+	**1**	**12.5**	NA	Intergenic	L1Md_A|LINE|L1	-175003	NM_148413	667663	Mm.330033	NM_148413	ENSMUSG00000025716	**Myo3a**	9030416P08Rik	myosin IIIA	Amp	protein-coding
1431_T32	chr2	2.2E+07	2.2E+07	3999	+	**1**	**12.5**	NA	Intergenic	L1Md_T|LINE|L1	-139003	NM_148413	667663	Mm.330033	NM_148413	ENSMUSG00000025716	**Myo3a**	9030416P08Rik	myosin IIIA	Amp	protein-coding
2281_T3	chr3	5.7E+07	5.7E+07	999	+	**1**	**12.5**	NA	Intergenic	L1Md_T|LINE|L1	-361299	NM_030595	26422	Mm.384353	NM_030595	ENSMUSG00000027799	**Nbea**	Lyst2|mKIAA1544	neurobeachin	Amp	protein-coding
1176_T3	chr16	8.1E+07	8.1E+07	3999	+	**1**	**12.5**	NA	Intergenic	Intergenic	-294197	NM_001113208	17968	Mm.433941	NM_010954	ENSMUSG00000022762	**Ncam2**	Ncam-2|Ocam|RNCAM	neural cell adhesion molecule 2	Amp	protein-coding

1177_T42	chr16	8.2E+07	8.2E+07	4999	+	**1**	**12.5**	NA	Intergenic	Intergenic	703303	NM_010954	17968	Mm.433941	NM_010954	ENSMUSG00000022762	**Ncam2**	Ncam-2|Ocam|RNCAM	neural cell adhesion molecule 2	Amp	protein-coding
1178_T22	chr16	8.2E+07	8.2E+07	6999	+	**1**	**12.5**	NA	Intergenic	Intergenic	816303	NM_010954	17968	Mm.433941	NM_010954	ENSMUSG00000022762	**Ncam2**	Ncam-2|Ocam|RNCAM	neural cell adhesion molecule 2	Amp	protein-coding
2484_T2	chr3	1.3E+08	1.3E+08	999	+	**1**	**12.5**	NA	Intergenic	Lx7|LINE|L1	-70091	NM_022565	64580	Mm.444289	NM_022565	ENSMUSG00000027971	**Ndst4**	4930439H17Rik	N-deacetylase/N-sulfotransferase (heparin glucosaminyl) 4	Amp	protein-coding
2486_T32	chr3	1.3E+08	1.3E+08	3999	+	**1**	**12.5**	NA	Intergenic	Intergenic	-3591	NM_022565	64580	Mm.444289	NM_022565	ENSMUSG00000027971	**Ndst4**	4930439H17Rik	N-deacetylase/N-sulfotransferase (heparin glucosaminyl) 4	Amp	protein-coding
2487_T2	chr3	1.3E+08	1.3E+08	3999	+	**1**	**12.5**	NA	intron (NM_022565, intron 2 of 13)	L1M2|LINE|L1	92409	NM_022565	64580	Mm.444289	NM_022565	ENSMUSG00000027971	**Ndst4**	4930439H17Rik	N-deacetylase/N-sulfotransferase (heparin glucosaminyl) 4	Amp	protein-coding
2659_T22	chr4	9.7E+07	9.7E+07	4999	+	**1**	**12.5**	NA	Intergenic	RMER6A|LTR|ERVK	-250943	NM_001122952	18027	Mm.31274	NM_010905	ENSMUSG00000028565	**Nfia**	1110047K16Rik|9430022M17Rik|CTF|NF1-A|NF1A	nuclear factor I/A	Amp	protein-coding
2660_T32	chr4	9.7E+07	9.7E+07	4999	+	**1**	**12.5**	NA	Intergenic	L1Md_F2|LINE|L1	-188943	NM_001122952	18027	Mm.31274	NM_010905	ENSMUSG00000028565	**Nfia**	1110047K16Rik|9430022M17Rik|CTF|NF1-A|NF1A	nuclear factor I/A	Amp	protein-coding
2661_T3	chr4	9.7E+07	9.7E+07	3999	+	**1**	**12.5**	NA	Intergenic	MIRb|SINE|MIR	-147443	NM_001122952	18027	Mm.31274	NM_010905	ENSMUSG00000028565	**Nfia**	1110047K16Rik|9430022M17Rik|CTF|NF1-A|NF1A	nuclear factor I/A	Amp	protein-coding
229_T3	chr10	3.2E+07	3.2E+07	4999	+	**1**	**12.5**	NA	intron (NM_001013411, intron 1 of 6)	intron (NM_001013411, intron 1 of 6)	409915	NM_001013411	432450	Mm.49665	NM_001013411	ENSMUSG00000069670	**Nkain2**	6330571D19Rik|AW455467|Tcba1	Na+/K+ transporting ATPase interacting 2	Amp	protein-coding
2549_T3	chr4	2.1E+07	2.1E+07	2999	+	**1**	**12.5**	NA	intron (NM_172987, intron 1 of 4)	intron (NM_172987, intron 1 of 4)	83668	NM_001290410	269513	Mm.317473	NM_172987	ENSMUSG00000055761	**Nkain3**	C530048M05|E130310K16Rik	Na+/K+ transporting ATPase interacting 3	Amp	protein-coding
1724_T32	chr3	2.5E+07	2.5E+07	999	+	**1**	**12.5**	NA	Intergenic	Intergenic	1630307	NM_001163387	192167	Mm.316080	NM_138666	ENSMUSG00000063887	**Nlgn1**	6330415N05Rik|BB179718|NL1|Nlg1|mKIAA1070	neuroligin 1	Amp	protein-coding
1728_T32	chr3	2.5E+07	2.5E+07	3999	+	**1**	**12.5**	NA	Intergenic	Intergenic	1515807	NM_001163387	192167	Mm.316080	NM_138666	ENSMUSG00000063887	**Nlgn1**	6330415N05Rik|BB179718|NL1|Nlg1|mKIAA1070	neuroligin 1	Amp	protein-coding
1729_T22	chr3	2.5E+07	2.5E+07	3999	+	**1**	**12.5**	NA	Intergenic	Intergenic	1312807	NM_001163387	192167	Mm.316080	NM_138666	ENSMUSG00000063887	**Nlgn1**	6330415N05Rik|BB179718|NL1|Nlg1|mKIAA1070	neuroligin 1	Amp	protein-coding

1730_T3	chr3	2.5E+07	2.5E+07	4999	+	**1**	**12.5**	NA	Intergenic	MT2B1|LTR|ERVL	794307	NM_001163387	192167	Mm.316080	NM_138666	ENSMUSG00000063887	**Nlgn1**	6330415N05Rik|BB179718|NL1|Nlg1|mKIAA1070	neuroligin 1	Amp	protein-coding
1731_T22	chr3	2.5E+07	2.5E+07	4999	+	**1**	**12.5**	NA	Intergenic	Intergenic	723307	NM_001163387	192167	Mm.316080	NM_138666	ENSMUSG00000063887	**Nlgn1**	6330415N05Rik|BB179718|NL1|Nlg1|mKIAA1070	neuroligin 1	Amp	protein-coding
1739_T4	chr3	2.6E+07	2.6E+07	2999	+	**1**	**12.5**	NA	intron (NM_138666, intron 3 of 7)	intron (NM_138666, intron 3 of 7)	109307	NM_001163387	192167	Mm.316080	NM_138666	ENSMUSG00000063887	**Nlgn1**	6330415N05Rik|BB179718|NL1|Nlg1|mKIAA1070	neuroligin 1	Amp	protein-coding
1733_T1	chr3	2.6E+07	2.6E+07	999	+	**1**	**12.5**	NA	intron (NM_138666, intron 5 of 7)	intron (NM_138666, intron 5 of 7)	638307	NM_001163387	192167	Mm.316080	NM_138666	ENSMUSG00000063887	**Nlgn1**	6330415N05Rik|BB179718|NL1|Nlg1|mKIAA1070	neuroligin 1	Amp	protein-coding
2170_T3	chr3	5.1E+07	5.1E+07	7999	+	**1**	**12.5**	NA	Intergenic	L1Md_T|LINE|L1	-263947	NM_009834	12457	Mm.86541	NM_009834	ENSMUSG00000023087	**Noct**	AU043840|Ccr4|Ccrn4l	nocturnin	Amp	protein-coding
2177_T3	chr3	5.1E+07	5.1E+07	5999	+	**1**	**12.5**	NA	intron (NM_009834, intron 1 of 2)	IAPEz-int|LTR|ERVK	18053	NM_009834	12457	Mm.86541	NM_009834	ENSMUSG00000023087	**Noct**	AU043840|Ccr4|Ccrn4l	nocturnin	Amp	protein-coding
1329_T42	chr18	2.3E+07	2.3E+07	3999	+	**1**	**12.5**	NA	intron (NM_199024, intron 1 of 9)	L1Md_T|LINE|L1	72163	NM_001161483	319211	Mm.209896	NM_199024	ENSMUSG00000041923	**Nol4**	1700013J13Rik|4930568N03Rik|Gm1262	nucleolar protein 4	Amp	protein-coding
435_T32	chr12	4.6E+07	4.6E+07	999	+	**1**	**12.5**	NA	Intergenic	G-rich|Low_complexity|Low_complexity	707775	NM_021361	664883	Mm.247195	NM_021361	ENSMUSG00000021047	**Nova1**	9430099M15Rik|G630039L02|Nova-1	neuro-oncological ventral antigen 1	Amp	protein-coding
436_T32	chr12	4.8E+07	4.8E+07	999	+	**1**	**12.5**	NA	Intergenic	Intergenic	-738225	NM_021361	664883	Mm.247195	NM_021361	ENSMUSG00000021047	**Nova1**	9430099M15Rik|G630039L02|Nova-1	neuro-oncological ventral antigen 1	Amp	protein-coding
3761_T1	chr9	6E+07	6E+07	4999	+	**1**	**12.5**	NA	intron (NM_173018, intron 16 of 43)	L1_Mus2|LINE|L1	97079	NM_013708	23958	Mm.103641	NM_013708	ENSMUSG00000032292	**Nr2e3**	A930035N01Rik|PNR|RNR|rd7	nuclear receptor subfamily 2, group E, member 3	Amp	protein-coding
530_T1	chr13	7.9E+07	7.9E+07	3999	+	**1**	**12.5**	NA	Intergenic	MLT1B|LTR|ERVL-MaLR	-421518	NM_010151	13865	Mm.439653	NM_010151	ENSMUSG00000069171	**Nr2f1**	COUP-TF1|COUP-TFI|COUPTFA|EAR-3|EAR3|Erbal3|SVP44|Tcfcoup1	nuclear receptor subfamily 2, group F, member 1	Amp	protein-coding
531_T42	chr13	7.9E+07	7.9E+07	999	+	**1**	**12.5**	NA	Intergenic	Lx|LINE|L1	-872018	NM_010151	13865	Mm.439653	NM_010151	ENSMUSG00000069171	**Nr2f1**	COUP-TF1|COUP-TFI|COUPTFA|EAR-3|EAR3|Erbal3|SVP44|Tcfcoup1	nuclear receptor subfamily 2, group F, member 1	Amp	protein-coding

537_T2	chr13	7.9E+07	7.9E+07	3999	+	**1**	**12.5**	NA	Intergenic	Intergenic	-944518	NM_010151	13865	Mm.439653	NM_010151	ENSMUSG00000069171	**Nr2f1**	COUP-TF1|COUP-TFI|COUPTFA|EAR-3|EAR3|Erbal3|SVP44|Tcfcoup1	nuclear receptor subfamily 2, group F, member 1	Amp	protein-coding
542_T1	chr13	7.9E+07	7.9E+07	999	+	**1**	**12.5**	NA	Intergenic	Intergenic	-977018	NM_010151	13865	Mm.439653	NM_010151	ENSMUSG00000069171	**Nr2f1**	COUP-TF1|COUP-TFI|COUPTFA|EAR-3|EAR3|Erbal3|SVP44|Tcfcoup1	nuclear receptor subfamily 2, group F, member 1	Amp	protein-coding
543_T3	chr13	7.9E+07	7.9E+07	3999	+	**1**	**12.5**	NA	Intergenic	Intergenic	-1E+06	NM_010151	13865	Mm.439653	NM_010151	ENSMUSG00000069171	**Nr2f1**	COUP-TF1|COUP-TFI|COUPTFA|EAR-3|EAR3|Erbal3|SVP44|Tcfcoup1	nuclear receptor subfamily 2, group F, member 1	Amp	protein-coding
457_T3	chr12	8.9E+07	8.9E+07	1999	+	**1**	**12.5**	NA	intron (NM_172544, intron 2 of 19)	RSINE1|SINE|B4	105110	NM_172544	18191	Mm.425766	NM_172544	ENSMUSG00000066392	**Nrxn3**	-	neurexin III	Amp	protein-coding
3772_T42	chr9	8.8E+07	8.8E+07	3999	+	**1**	**12.5**	NA	Intergenic	L1Md_T|LINE|L1	-195109	NM_011851	23959	Mm.244235	NM_011851	ENSMUSG00000032420	**Nt5e**	2210401F01Rik|5'-NT|AI447961|CD73|NT|Nt5|eNT	5' nucleotidase, ecto	Amp	protein-coding
1276_T32	chr17	5.9E+07	5.9E+07	1999	+	**1**	**12.5**	NA	Intergenic	AT_rich|Low_complexity|Low_complexity	-388178	NM_026497	67993	Mm.36507	NM_026497	ENSMUSG00000024228	**Nudt12**	0610016O18Rik	nudix (nucleoside diphosphate linked moiety X)-type motif 12	Amp	protein-coding
1287_T3	chr17	6E+07	6E+07	999	+	**1**	**12.5**	NA	Intergenic	Intergenic	-1E+06	NM_026497	67993	Mm.36507	NM_026497	ENSMUSG00000024228	**Nudt12**	0610016O18Rik	nudix (nucleoside diphosphate linked moiety X)-type motif 12	Amp	protein-coding
1288_T12	chr17	6.1E+07	6.1E+07	4999	+	**1**	**12.5**	NA	Intergenic	Intergenic	-2E+06	NM_026497	67993	Mm.36507	NM_026497	ENSMUSG00000024228	**Nudt12**	0610016O18Rik	nudix (nucleoside diphosphate linked moiety X)-type motif 12	Amp	protein-coding
1514_T4	chr2	8.1E+07	8.1E+07	4999	+	**1**	**12.5**	NA	Intergenic	Intergenic	473736	NM_001190179	69482	Mm.29200	NM_027091	ENSMUSG00000026999	**Nup35**	2310006I24Rik|35kDa|5330402E05Rik|MP44|NO44	nucleoporin 35	Amp	protein-coding
2891_T32	chr6	9908501	9909500	999	+	**1**	**12.5**	NA	Intergenic	Lx9|LINE|L1	958981	NM_008751	18231	Mm.46954	NM_008751	ENSMUSG00000046178	**Nxph1**	C130005L03Rik	neurexophilin 1	Amp	protein-coding
2882_T12	chr6	9201501	9206500	4999	+	**1**	**12.5**	NA	intron (NM_008751, intron 1 of 1)	intron (NM_008751, intron 1 of 1)	253981	NM_008751	18231	Mm.46954	NM_008751	ENSMUSG00000046178	**Nxph1**	C130005L03Rik	neurexophilin 1	Amp	protein-coding

52_T2	chr1	8.1E+07	8.1E+07	999	+	**1**	**12.5**	NA	Intergenic	Intergenic	-116317	NM_172849	241134	Mm.313904	NM_172849	ENSMUSG00000054976	**Nyap2**	9430031J16Rik|Jr6|Kiaa1486	neuronal tyrosine-phophorylated phosphoinositide 3-kinase adaptor 2	Amp	protein-coding
2472_T4	chr3	1.2E+08	1.2E+08	1999	+	**1**	**12.5**	NA	Intergenic	Intergenic	54535	NM_153157	229759	Mm.54183	NM_153157	ENSMUSG00000027965	**Olfm3**	B230206G02Rik	olfactomedin 3	Amp	protein-coding
633_T2	chr14	8.1E+07	8.1E+07	999	+	**1**	**12.5**	NA	Intergenic	Intergenic	948698	NM_001030294	380924	Mm.26456	NM_001030294	ENSMUSG00000022026	**Olfm4**	GC1|GW112|Gm296|Gm913|OlfD|pPD4	olfactomedin 4	Amp	protein-coding
634_T3	chr14	8.1E+07	8.1E+07	3999	+	**1**	**12.5**	NA	Intergenic	Intergenic	1100198	NM_001030294	380924	Mm.26456	NM_001030294	ENSMUSG00000022026	**Olfm4**	GC1|GW112|Gm296|Gm913|OlfD|pPD4	olfactomedin 4	Amp	protein-coding
635_T1	chr14	8.2E+07	8.2E+07	4999	+	**1**	**12.5**	NA	Intergenic	MRLTR33|LTR|ERVK	1606698	NM_001030294	380924	Mm.26456	NM_001030294	ENSMUSG00000022026	**Olfm4**	GC1|GW112|Gm296|Gm913|OlfD|pPD4	olfactomedin 4	Amp	protein-coding
636_T2	chr14	8.2E+07	8.2E+07	999	+	**1**	**12.5**	NA	Intergenic	(TC)n|Simple_repeat|Simple_repeat	1829698	NM_001030294	380924	Mm.26456	NM_001030294	ENSMUSG00000022026	**Olfm4**	GC1|GW112|Gm296|Gm913|OlfD|pPD4	olfactomedin 4	Amp	protein-coding
640_T2	chr14	8.2E+07	8.2E+07	8999	+	**1**	**12.5**	NA	Intergenic	Intergenic	2114698	NM_001030294	380924	Mm.26456	NM_001030294	ENSMUSG00000022026	**Olfm4**	GC1|GW112|Gm296|Gm913|OlfD|pPD4	olfactomedin 4	Amp	protein-coding
1337_T1	chr19	1.8E+07	1.8E+07	3999	+	**1**	**12.5**	NA	Intergenic	Lx6|LINE|L1	230313	NM_017375	20409	Mm.172222	NM_017375	ENSMUSG00000024725	**Ostf1**	C78236|SH3P2|Sh3d3	osteoclast stimulating factor 1	Amp	protein-coding
1937_T12	chr3	4.6E+07	4.6E+07	999	+	**1**	**12.5**	NA	Intergenic	Intergenic	256941	NM_001101479	241989	Mm.119234	NM_001101479	ENSMUSG00000090919	**Pabpc4l**	C330050A14Rik|EG241989	poly(A) binding protein, cytoplasmic 4-like	Amp	protein-coding
1938_T32	chr3	4.6E+07	4.6E+07	13999	+	**1**	**12.5**	NA	Intergenic	Lx2|LINE|L1	249441	NM_001101479	241989	Mm.119234	NM_001101479	ENSMUSG00000090919	**Pabpc4l**	C330050A14Rik|EG241989	poly(A) binding protein, cytoplasmic 4-like	Amp	protein-coding
1954_T32	chr3	4.7E+07	4.7E+07	9999	+	**1**	**12.5**	NA	Intergenic	IAPEz-int|LTR|ERVK	-204559	NM_001101479	241989	Mm.119234	NM_001101479	ENSMUSG00000090919	**Pabpc4l**	C330050A14Rik|EG241989	poly(A) binding protein, cytoplasmic 4-like	Amp	protein-coding
1955_T32	chr3	4.7E+07	4.7E+07	999	+	**1**	**12.5**	NA	Intergenic	B4A|SINE|B4	-211059	NM_001101479	241989	Mm.119234	NM_001101479	ENSMUSG00000090919	**Pabpc4l**	C330050A14Rik|EG241989	poly(A) binding protein, cytoplasmic 4-like	Amp	protein-coding
642_T4	chr14	8.3E+07	8.3E+07	6999	+	**1**	**12.5**	NA	Intergenic	Intergenic	-1E+06	NM_001013753	219228	Mm.153643	NM_001013753	ENSMUSG00000035566	**Pcdh17**	C030033F14Rik|Gm78	protocadherin 17	Amp	protein-coding
647_T3	chr14	8.3E+07	8.3E+07	5999	+	**1**	**12.5**	NA	Intergenic	Intergenic	-1E+06	NM_001013753	219228	Mm.153643	NM_001013753	ENSMUSG00000035566	**Pcdh17**	C030033F14Rik|Gm78	protocadherin 17	Amp	protein-coding
648_T3	chr14	8.3E+07	8.3E+07	5999	+	**1**	**12.5**	NA	Intergenic	Intergenic	-1E+06	NM_001013753	219228	Mm.153643	NM_001013753	ENSMUSG00000035566	**Pcdh17**	C030033F14Rik|Gm78	protocadherin 17	Amp	protein-coding
649_T4	chr14	8.3E+07	8.3E+07	9999	+	**1**	**12.5**	NA	Intergenic	RSINE1|SINE|B4	-1E+06	NM_001013753	219228	Mm.153643	NM_001013753	ENSMUSG00000035566	**Pcdh17**	C030033F14Rik|Gm78	protocadherin 17	Amp	protein-coding

650_T32	chr14	8.3E+07	8.3E+07	2999	+	**1**	**12.5**	NA	Intergenic	Intergenic	-1E+06	NM_001013753	219228	Mm.153643	NM_001013753	ENSMUSG00000035566	**Pcdh17**	C030033F14Rik|Gm78	protocadherin 17	Amp	protein-coding
652_T22	chr14	8.3E+07	8.3E+07	4999	+	**1**	**12.5**	NA	Intergenic	Intergenic	-1E+06	NM_001013753	219228	Mm.153643	NM_001013753	ENSMUSG00000035566	**Pcdh17**	C030033F14Rik|Gm78	protocadherin 17	Amp	protein-coding
657_T4	chr14	8.3E+07	8.3E+07	999	+	**1**	**12.5**	NA	Intergenic	Lx6|LINE|L1	-951563	NM_001013753	219228	Mm.153643	NM_001013753	ENSMUSG00000035566	**Pcdh17**	C030033F14Rik|Gm78	protocadherin 17	Amp	protein-coding
661_T1	chr14	8.4E+07	8.4E+07	7999	+	**1**	**12.5**	NA	Intergenic	L1Md_F3|LINE|L1	-626063	NM_001013753	219228	Mm.153643	NM_001013753	ENSMUSG00000035566	**Pcdh17**	C030033F14Rik|Gm78	protocadherin 17	Amp	protein-coding
2133_T3	chr3	5E+07	5E+07	7999	+	**1**	**12.5**	NA	Intergenic	MMERVK10C-int|LTR|ERVK	26816	NM_130448	73173	Mm.87246	NM_130448	ENSMUSG00000037892	**Pcdh18**	3110038E07Rik|BB095589|PCDH68L	protocadherin 18	Amp	protein-coding
2135_T3	chr3	5E+07	5E+07	6999	+	**1**	**12.5**	NA	Intergenic	L1Md_T|LINE|L1	-42684	NM_130448	73173	Mm.87246	NM_130448	ENSMUSG00000037892	**Pcdh18**	3110038E07Rik|BB095589|PCDH68L	protocadherin 18	Amp	protein-coding
2143_T3	chr3	5E+07	5E+07	7999	+	**1**	**12.5**	NA	Intergenic	L1Md_A|LINE|L1	-69184	NM_130448	73173	Mm.87246	NM_130448	ENSMUSG00000037892	**Pcdh18**	3110038E07Rik|BB095589|PCDH68L	protocadherin 18	Amp	protein-coding
685_T4	chr14	8.8E+07	8.8E+07	999	+	**1**	**12.5**	NA	Intergenic	Intergenic	410396	NM_178685	219257	Mm.128861	NM_178685	ENSMUSG00000050505	**Pcdh20**	C630015B17Rik|Pcdh13	protocadherin 20	Amp	protein-coding
687_T3	chr14	8.8E+07	8.8E+07	999	+	**1**	**12.5**	NA	Intergenic	Intergenic	220396	NM_178685	219257	Mm.128861	NM_178685	ENSMUSG00000050505	**Pcdh20**	C630015B17Rik|Pcdh13	protocadherin 20	Amp	protein-coding
692_T42	chr14	8.8E+07	8.8E+07	7999	+	**1**	**12.5**	NA	Intergenic	L1Md_T|LINE|L1	98896	NM_178685	219257	Mm.128861	NM_178685	ENSMUSG00000050505	**Pcdh20**	C630015B17Rik|Pcdh13	protocadherin 20	Amp	protein-coding
2786_T2	chr5	5.8E+07	5.8E+07	999	+	**1**	**12.5**	NA	intron (NM_001310608, intron 1 of 3)	intron (NM_001310608, intron 1 of 3)	178919	NM_018764	54216	Mm.332387	NM_018764	ENSMUSG00000029108	**Pcdh7**	-	protocadherin 7	Amp	protein-coding
724_T2	chr14	9.2E+07	9.2E+07	8999	+	**1**	**12.5**	NA	Intergenic	Intergenic	1617888	NM_001271800	211712	Mm.37126	NM_001081377	ENSMUSG00000055421	**Pcdh9**	-	protocadherin 9	Amp	protein-coding
729_T22	chr14	9.3E+07	9.3E+07	3999	+	**1**	**12.5**	NA	Intergenic	Lx6|LINE|L1	1022388	NM_001271800	211712	Mm.37126	NM_001081377	ENSMUSG00000055421	**Pcdh9**	-	protocadherin 9	Amp	protein-coding
751_T1	chr14	9.4E+07	9.4E+07	1999	+	**1**	**12.5**	NA	Intergenic	Intergenic	-179831	NM_001271798	211712	Mm.37126	NM_001081377	ENSMUSG00000055421	**Pcdh9**	-	protocadherin 9	Amp	protein-coding
753_T22	chr14	9.4E+07	9.4E+07	999	+	**1**	**12.5**	NA	Intergenic	MLT1C|LTR|ERVL-MaLR	-218331	NM_001271798	211712	Mm.37126	NM_001081377	ENSMUSG00000055421	**Pcdh9**	-	protocadherin 9	Amp	protein-coding
756_T32	chr14	9.4E+07	9.4E+07	6999	+	**1**	**12.5**	NA	Intergenic	L1Md_F2|LINE|L1	-353331	NM_001271798	211712	Mm.37126	NM_001081377	ENSMUSG00000055421	**Pcdh9**	-	protocadherin 9	Amp	protein-coding

757_T32	chr14	9.4E+07	9.4E+07	4999	+	**1**	**12.5**	NA	Intergenic	Intergenic	-402331	NM_001271798	211712	Mm.37126	NM_001081377	ENSMUSG00000055421	**Pcdh9**	-	protocadherin 9	Amp	protein-coding
758_T1	chr14	9.4E+07	9.4E+07	3999	+	**1**	**12.5**	NA	Intergenic	Intergenic	-454831	NM_001271798	211712	Mm.37126	NM_001081377	ENSMUSG00000055421	**Pcdh9**	-	protocadherin 9	Amp	protein-coding
759_T32	chr14	9.4E+07	9.4E+07	4999	+	**1**	**12.5**	NA	Intergenic	L1_Mus3|LINE|L1	-514331	NM_001271798	211712	Mm.37126	NM_001081377	ENSMUSG00000055421	**Pcdh9**	-	protocadherin 9	Amp	protein-coding
742_T22	chr14	9.4E+07	9.4E+07	999	+	**1**	**12.5**	NA	intron (NM_001081377, intron 1 of 3)	intron (NM_001081377, intron 1 of 3)	214888	NM_001271800	211712	Mm.37126	NM_001081377	ENSMUSG00000055421	**Pcdh9**	-	protocadherin 9	Amp	protein-coding
743_T1	chr14	9.4E+07	9.4E+07	999	+	**1**	**12.5**	NA	intron (NM_001081377, intron 1 of 3)	B1F|SINE|Alu	33888	NM_001271800	211712	Mm.37126	NM_001081377	ENSMUSG00000055421	**Pcdh9**	-	protocadherin 9	Amp	protein-coding
747_T4	chr14	9.4E+07	9.4E+07	999	+	**1**	**12.5**	NA	intron (NM_001081377, intron 1 of 3)	intron (NM_001081377, intron 1 of 3)	20888	NM_001271800	211712	Mm.37126	NM_001081377	ENSMUSG00000055421	**Pcdh9**	-	protocadherin 9	Amp	protein-coding
734_T12	chr14	9.3E+07	9.3E+07	5999	+	**1**	**12.5**	NA	intron (NM_001271798, intron 2 of 3)	intron (NM_001271798, intron 2 of 3)	553388	NM_001271800	211712	Mm.37126	NM_001081377	ENSMUSG00000055421	**Pcdh9**	-	protocadherin 9	Amp	protein-coding
733_T4	chr14	9.3E+07	9.3E+07	999	+	**1**	**12.5**	NA	intron (NM_001271800, intron 2 of 2)	intron (NM_001271800, intron 2 of 2)	750888	NM_001271800	211712	Mm.37126	NM_001081377	ENSMUSG00000055421	**Pcdh9**	-	protocadherin 9	Amp	protein-coding
2717_T1	chr5	1.5E+07	1.5E+07	3999	+	**1**	**12.5**	NA	intron (NM_001110796, intron 3 of 19)	Lx7|LINE|L1	112582	NM_001110796	26875	Mm.146275	NM_011995	ENSMUSG00000061601	**Pclo**	Acz|Pico	piccolo (presynaptic cytomatrix protein)	Amp	protein-coding
1336_T22	chr19	1.8E+07	1.8E+07	4999	+	**1**	**12.5**	NA	Intergenic	(CACAC)n|Simple_repeat|Simple_repeat	-153368	NM_001190483	18552	Mm.3401	NM_001163144	ENSMUSG00000024713	**Pcsk5**	PC5|PC6|SPC6|b2b1549Clo|b2b585Clo	proprotein convertase subtilisin/kexin type 5	Amp	protein-coding
194_T42	chr1	1.5E+08	1.5E+08	3999	+	**1**	**12.5**	NA	Intergenic	Intergenic	-42917	NM_024458	20028	Mm.440883	NM_024458	ENSMUSG00000006007	**Pdc**	Rpr-1|Rpr1	phosducin	Amp	protein-coding
2387_T3	chr3	7.5E+07	7.5E+07	999	+	**1**	**12.5**	NA	Intergenic	Intergenic	129852	NM_019745	56426	Mm.316473	NM_019745	ENSMUSG00000027835	**Pdcd10**	2410003B13Rik|Ccm3|Tfa15|Tfar15	programmed cell death 10	Amp	protein-coding
608_T12	chr14	2.7E+07	2.7E+07	17999	+	**1**	**12.5**	NA	Intergenic	Intergenic	-78654	NM_178668	211948	Mm.290758	NM_178668	ENSMUSG00000043702	**Pde12**	2'-PDE|E430028B21Rik	phosphodiesterase 12	Amp	protein-coding

3682_T12	chr9	5920501	5945500	24999	+	**1**	**12.5**	NA	Intergenic	L1_Mus1|LINE|L1	-235612	NM_027924	71785	Mm.390122	NM_027924	ENSMUSG00000032006	**Pdgfd**	1110003I09Rik	platelet-derived growth factor, D polypeptide	Amp	protein-coding
3685_T12	chr9	5952501	6034500	81999	+	**1**	**12.5**	NA	Intergenic	Intergenic	-175112	NM_027924	71785	Mm.390122	NM_027924	ENSMUSG00000032006	**Pdgfd**	1110003I09Rik	platelet-derived growth factor, D polypeptide	Amp	protein-coding
3686_T12	chr9	6035501	6084500	48999	+	**1**	**12.5**	NA	Intergenic	ERVB7_4-LTR_MM|LTR|ERVK	-108612	NM_027924	71785	Mm.390122	NM_027924	ENSMUSG00000032006	**Pdgfd**	1110003I09Rik	platelet-derived growth factor, D polypeptide	Amp	protein-coding
3687_T12	chr9	6085501	6140500	54999	+	**1**	**12.5**	NA	Intergenic	ORR1A0|LTR|ERVL-MaLR	-55612	NM_027924	71785	Mm.390122	NM_027924	ENSMUSG00000032006	**Pdgfd**	1110003I09Rik	platelet-derived growth factor, D polypeptide	Amp	protein-coding
2506_T3	chr3	1.4E+08	1.4E+08	999	+	**1**	**12.5**	NA	Intergenic	Intergenic	101355	NM_008811	18598	Mm.4223	NM_008811	ENSMUSG00000047674	**Pdha2**	Pdhal	pyruvate dehydrogenase E1 alpha 2	Amp	protein-coding
2447_T12	chr3	9.7E+07	9.7E+07	3999	+	**1**	**12.5**	NA	intron (NM_001146001, intron 1 of 8)	(TG)n|Simple_repeat|Simple_repeat	9826	NM_001146001	59020	Mm.489677	NM_021517	ENSMUSG00000038298	**Pdzk1**	1700023D20Rik|2610507N21Rik|4921513F16Rik|AI267131|AI314638|AL022680|D3Ertd537e|Pdzd1|mPDZK1	PDZ domain containing 1	Amp	protein-coding
1653_T2	chr3	5957501	5961500	3999	+	**1**	**12.5**	NA	Intergenic	Intergenic	-383252	NM_001163305	19302	Mm.132336	NM_008994	ENSMUSG00000040374	**Pex2**	D3Ertd138e|PAF-1|PMP35|Pxmp3	peroxisomal biogenesis factor 2	Amp	protein-coding
2439_T42	chr3	9.2E+07	9.2E+07	999	+	**1**	**12.5**	NA	Intergenic	L1Md_F2|LINE|L1	-194583	NM_207247	242100	Mm.297406	NM_207247	ENSMUSG00000042244	**Pglyrp3**	Gm420	peptidoglycan recognition protein 3	Amp	protein-coding
2433_T32	chr3	9.1E+07	9.1E+07	3999	+	**1**	**12.5**	NA	Intergenic	Intergenic	18594	NM_207263	384997	Mm.316644	NM_207263	ENSMUSG00000042250	**Pglyrp4**	Gm1478|Gm413	peptidoglycan recognition protein 4	Amp	protein-coding
2434_T32	chr3	9.1E+07	9.1E+07	3999	+	**1**	**12.5**	NA	Intergenic	L1M2|LINE|L1	89594	NM_207263	384997	Mm.316644	NM_207263	ENSMUSG00000042250	**Pglyrp4**	Gm1478|Gm413	peptidoglycan recognition protein 4	Amp	protein-coding
20_T4	chr1	3E+07	3E+07	5999	+	**1**	**12.5**	NA	Intergenic	Intergenic	720756	NM_001081080	213109	Mm.194486	NM_025871	ENSMUSG00000048874	**Phf3**	2310061N19Rik|mKIAA0244	PHD finger protein 3	Amp	protein-coding
3604_T42	chr8	8.6E+07	8.6E+07	3999	+	**1**	**12.5**	NA	intron (NM_199446, intron 7 of 31)	L1Md_A|LINE|L1	76498	NM_199446	102093	Mm.237296	NM_199446	ENSMUSG00000036879	**Phkb**	AI463271	phosphorylase kinase beta	Amp	protein-coding
1331_T42	chr18	6.4E+07	6.4E+07	3999	+	**1**	**12.5**	NA	Intergenic	L1Md_A|LINE|L1	-150317	NM_001039485	667742	Mm.158720	NM_172629	ENSMUSG00000041482	**Piezo2**	5930434P17|9030411M15Rik|9430028L06Rik|Fam38b|Fam38b2	piezo-type mechanosensitive ion channel component 2	Amp	protein-coding
191_T2	chr1	1.5E+08	1.5E+08	999	+	**1**	**12.5**	NA	Intergenic	Intergenic	1021439	NM_001305632	18783	Mm.4186	NM_008869	ENSMUSG00000056220	**Pla2g4a**	Pla2g4|cPLA2|cPLA2-alpha|cPLA2alpha	phospholipase A2, group IVA (cytosolic, calcium-dependent)	Amp	protein-coding

368_T32	chr11	2.9E+07	2.9E+07	3999	+	**1**	**12.5**	NA	Intergenic	L1Md_F2|LINE|L1	-86251	NM_027869	71701	Mm.211131	NM_027869	ENSMUSG00000020464	**Pnpt1**	1200003F12Rik|Old35|PNPase|Pnptl1	polyribonucleotide nucleotidyltransferase 1	Amp	protein-coding
3429_T1	chr8	2.8E+07	2.8E+07	3999	+	**1**	**12.5**	NA	Intergenic	Intergenic	314830	NM_027618	70952	Mm.158160	NM_026256	ENSMUSG00000063932	**Poteg**	4921537P18Rik|4930456F22Rik|Gsarp1|Gsarp2	POTE ankyrin domain family, member G	Amp	protein-coding
2767_T22	chr5	5.1E+07	5.1E+07	4999	+	**1**	**12.5**	NA	Intergenic	Intergenic	379921	NR_027710	19017	Mm.259072	NM_008904	ENSMUSG00000029167	**Ppargc1a**	A830037N07Rik|ENSMUSG00000079510|Gm11133|PGC-1|PPARGC-1-alpha|Pgc-1alpha|Pgc1|Pgco1|Ppargc1	peroxisome proliferative activated receptor, gamma, coactivator 1 alpha	Amp	protein-coding
1439_T42	chr2	4.1E+07	4.1E+07	999	+	**1**	**12.5**	NA	intron (NM_053011, intron 83 of 90)	L1_Mur3|LINE|L1	-1E+06	NM_024209	67857	Mm.389242	NM_024209	ENSMUSG00000026753	**Ppp6c**	2310003C10Rik	protein phosphatase 6, catalytic subunit	Amp	protein-coding
1413_T4	chr19	6E+07	6E+07	6999	+	**1**	**12.5**	NA	Intergenic	L1Md_A|LINE|L1	117304	NM_201615	226278	Mm.377241	NM_201615	ENSMUSG00000045052	**Prlhr**	Gm339|Gpr10|Gr3|Prrpr	prolactin releasing hormone receptor	Amp	protein-coding
2456_T32	chr3	1.1E+08	1.1E+08	5999	+	**1**	**12.5**	NA	Intergenic	Intergenic	-327501	NR_024139	99890	Mm.36115	NM_178891	ENSMUSG00000049300	**Prmt6**	AW124876|BB233495|Hrmt1l6	protein arginine N-methyltransferase 6	Amp	protein-coding
792_T1	chr14	1E+08	1E+08	3999	+	**1**	**12.5**	NA	Intergenic	L1M1|LINE|L1	67569	NM_029680	76627	Mm.31415	NM_029680	ENSMUSG00000042888	**Prr30**	1700110M21Rik	proline rich 30	Amp	protein-coding
793_T12	chr14	1E+08	1E+08	3999	+	**1**	**12.5**	NA	Intergenic	Intergenic	11569	NM_029680	76627	Mm.31415	NM_029680	ENSMUSG00000042888	**Prr30**	1700110M21Rik	proline rich 30	Amp	protein-coding
2626_T1	chr4	7.8E+07	7.8E+07	999	+	**1**	**12.5**	NA	intron (NM_011211, intron 1 of 39)	MTE-int|LTR|ERVL-MaLR	180895	NM_011211	19266	Mm.184021	NM_011211	ENSMUSG00000028399	**Ptprd**	1110002J03Rik|3000002J10Rik|B230219D21Rik|R-PTP-delta	protein tyrosine phosphatase, receptor type, D	Amp	protein-coding
2627_T2	chr4	7.8E+07	7.8E+07	999	+	**1**	**12.5**	NA	intron (NM_011211, intron 1 of 39)	intron (NM_011211, intron 1 of 39)	26895	NM_011211	19266	Mm.184021	NM_011211	ENSMUSG00000028399	**Ptprd**	1110002J03Rik|3000002J10Rik|B230219D21Rik|R-PTP-delta	protein tyrosine phosphatase, receptor type, D	Amp	protein-coding
2629_T3	chr4	7.8E+07	7.8E+07	999	+	**1**	**12.5**	NA	intron (NM_011211, intron 1 of 39)	intron (NM_011211, intron 1 of 39)	20895	NM_011211	19266	Mm.184021	NM_011211	ENSMUSG00000028399	**Ptprd**	1110002J03Rik|3000002J10Rik|B230219D21Rik|R-PTP-delta	protein tyrosine phosphatase, receptor type, D	Amp	protein-coding
1573_T4	chr2	1E+08	1E+08	6999	+	**1**	**12.5**	NA	Intergenic	RMER12|LTR|ERVK	-2E+06	NM_009020	19374	Mm.4988	NM_009020	ENSMUSG00000032864	**Rag2**	Rag-2	recombination activating gene 2	Amp	protein-coding
1574_T42	chr2	1E+08	1E+08	3999	+	**1**	**12.5**	NA	Intergenic	L1Md_T|LINE|L1	-2E+06	NM_009020	19374	Mm.4988	NM_009020	ENSMUSG00000032864	**Rag2**	Rag-2	recombination activating gene 2	Amp	protein-coding
1575_T4	chr2	1E+08	1E+08	3999	+	**1**	**12.5**	NA	Intergenic	Intergenic	-1E+06	NM_009020	19374	Mm.4988	NM_009020	ENSMUSG00000032864	**Rag2**	Rag-2	recombination activating gene 2	Amp	protein-coding

1578_T32	chr2	1E+08	1E+08	999	+	**1**	**12.5**	NA	Intergenic	Intergenic	-987748	NM_009020	19374	Mm.4988	NM_009020	ENSMUSG00000032864	**Rag2**	Rag-2	recombination activating gene 2	Amp	protein-coding
1676_T3	chr3	1.3E+07	1.3E+07	5999	+	**1**	**12.5**	NA	Intergenic	Lx2|LINE|L1	-460155	NM_178631	76897	Mm.422625	NM_178631	ENSMUSG00000039717	**Ralyl**	0710005M24Rik	RALY RNA binding protein-like	Amp	protein-coding
1679_T3	chr3	1.3E+07	1.3E+07	999	+	**1**	**12.5**	NA	Intergenic	MERVL-int|LTR|ERVL	-368655	NM_178631	76897	Mm.422625	NM_178631	ENSMUSG00000039717	**Ralyl**	0710005M24Rik	RALY RNA binding protein-like	Amp	protein-coding
1680_T42	chr3	1.3E+07	1.3E+07	5999	+	**1**	**12.5**	NA	Intergenic	L1Md_F2|LINE|L1	-282155	NM_178631	76897	Mm.422625	NM_178631	ENSMUSG00000039717	**Ralyl**	0710005M24Rik	RALY RNA binding protein-like	Amp	protein-coding
2420_T32	chr3	7.8E+07	7.8E+07	33999	+	**1**	**12.5**	NA	Intergenic	Lx3A|LINE|L1	1503387	NM_001310536	76089	Mm.31220	NM_001099624	ENSMUSG00000062232	**Rapgef2**	5830453M24Rik|CNRasGEF|Pdzgef1|RA-GEF-1|mKIAA0313|nRapGEP	Rap guanine nucleotide exchange factor (GEF) 2	Amp	protein-coding
2421_T32	chr3	7.8E+07	7.8E+07	4999	+	**1**	**12.5**	NA	Intergenic	Intergenic	1408887	NM_001310536	76089	Mm.31220	NM_001099624	ENSMUSG00000062232	**Rapgef2**	5830453M24Rik|CNRasGEF|Pdzgef1|RA-GEF-1|mKIAA0313|nRapGEP	Rap guanine nucleotide exchange factor (GEF) 2	Amp	protein-coding
2422_T32	chr3	7.8E+07	7.8E+07	3999	+	**1**	**12.5**	NA	Intergenic	Intergenic	1242387	NM_001310536	76089	Mm.31220	NM_001099624	ENSMUSG00000062232	**Rapgef2**	5830453M24Rik|CNRasGEF|Pdzgef1|RA-GEF-1|mKIAA0313|nRapGEP	Rap guanine nucleotide exchange factor (GEF) 2	Amp	protein-coding
604_T12	chr14	1.7E+07	1.7E+07	3999	+	**1**	**12.5**	NA	intron (NM_001289760, intron 1 of 9)	intron (NM_001289760, intron 1 of 9)	227831	NM_001289760	218772	Mm.259318	NM_011243	ENSMUSG00000017491	**Rarb**	A830025K23|Hap|Nr1b2	retinoic acid receptor, beta	Amp	protein-coding
203_T12	chr10	6309501	6311500	1999	+	**1**	**12.5**	NA	Intergenic	Intergenic	-388100	NM_019958	56533	Mm.44606	NM_019958	ENSMUSG00000019775	**Rgs17**	6430507P11Rik|Rgsz2	regulator of G-protein signaling 17	Amp	protein-coding
172_T12	chr1	1.5E+08	1.5E+08	999	+	**1**	**12.5**	NA	Intergenic	Intergenic	-554579	NM_022881	64214	Mm.253927	NM_022881	ENSMUSG00000026357	**Rgs18**	-	regulator of G-protein signaling 18	Amp	protein-coding
175_T2	chr1	1.5E+08	1.5E+08	999	+	**1**	**12.5**	NA	Intergenic	Intergenic	-592579	NM_022881	64214	Mm.253927	NM_022881	ENSMUSG00000026357	**Rgs18**	-	regulator of G-protein signaling 18	Amp	protein-coding
2547_T22	chr4	2E+07	2E+07	999	+	**1**	**12.5**	NA	intron (NM_001276292, intron 23 of 23)	L1MA4A|LINE|L1	41934	NM_025476	66302	Mm.440686	NM_025476	ENSMUSG00000028229	**Rmdn1**	Fam82b|RMD-1	regulator of microtubule dynamics 1	Amp	protein-coding
226_T1	chr10	3.2E+07	3.2E+07	999	+	**1**	**12.5**	NA	intron (NM_001013411, intron 3 of 6)	intron (NM_001013411, intron 3 of 6)	-410275	NM_001146349	268291	Mm.295212	NM_001146349	ENSMUSG00000063760	**Rnf217**	AU016819|Ibrdc1	ring finger protein 217	Amp	protein-coding

2467_T32	chr3	1.1E+08	1.1E+08	999	+	**1**	**12.5**	NA	Intergenic	Intergenic	-189851	NM_001038696	67225	Mm.316928	NM_026043	ENSMUSG00000027981	**Rnpc3**	2810441O16Rik|AI447568|C030014B17Rik	RNA-binding region (RNP1, RRM) containing 3	Amp	protein-coding
1339_T1	chr19	1.9E+07	1.9E+07	999	+	**1**	**12.5**	NA	Intergenic	Intergenic	-335804	NM_001043354	225998	Mm.234641	NM_146095	ENSMUSG00000036192	**Rorb**	Nr1f2|RZR-beta|RZRB|Rorbeta|hstp	RAR-related orphan receptor beta	Amp	protein-coding
976_T2	chr15	4.3E+07	4.3E+07	5999	+	**1**	**12.5**	NA	Intergenic	L2|LINE|L2	153318	NM_172815	239405	Mm.193274	NM_172815	ENSMUSG00000051920	**Rspo2**	2610028F08Rik|AA673245|D430027K22|ftls	R-spondin 2	Amp	protein-coding
2367_T4	chr3	6.7E+07	6.7E+07	3999	+	**1**	**12.5**	NA	intron (NM_025822, intron 4 of 9)	Lx|LINE|L1	144828	NM_025822	66880	Mm.316418	NM_025822	ENSMUSG00000034544	**Rsrc1**	1200013F24Rik|SRrp53	arginine/serine-rich coiled-coil 1	Amp	protein-coding
1512_T42	chr2	6.7E+07	6.7E+07	999	+	**1**	**12.5**	NA	TTS (NM_009135)	TTS (NM_009135)	-38038	NM_001290675	20274	Mm.440889	NM_018852	ENSMUSG00000075316	**Scn9a**	Nav1.7|PN1|mKIAA4197	sodium channel, voltage-gated, type IX, alpha	Amp	protein-coding
2710_T4	chr5	1.3E+07	1.3E+07	3999	+	**1**	**12.5**	NA	Intergenic	Intergenic	-397284	NM_001243072	20346	Mm.372039	NM_009152	ENSMUSG00000028883	**Sema3a**	Hsema-I|SEMA1|SemD|Semad|coll-1	sema domain, immunoglobulin domain (Ig), short basic domain, secreted, (semaphorin) 3A	Amp	protein-coding
2711_T4	chr5	1.3E+07	1.3E+07	8999	+	**1**	**12.5**	NA	Intergenic	Intergenic	-383784	NM_001243072	20346	Mm.372039	NM_009152	ENSMUSG00000028883	**Sema3a**	Hsema-I|SEMA1|SemD|Semad|coll-1	sema domain, immunoglobulin domain (Ig), short basic domain, secreted, (semaphorin) 3A	Amp	protein-coding
2705_T2	chr5	1.2E+07	1.2E+07	4999	+	**1**	**12.5**	NA	Intergenic	Lx9|LINE|L1	-109166	NM_028882	108151	Mm.89313	NM_028882	ENSMUSG00000040254	**Sema3d**	4631426B19Rik	sema domain, immunoglobulin domain (Ig), short basic domain, secreted, (semaphorin) 3D	Amp	protein-coding
2709_T4	chr5	1.2E+07	1.2E+07	999	+	**1**	**12.5**	NA	intron (NM_028882, intron 3 of 17)	(TTTG)n|Simple_repeat|Simple_repeat	86834	NM_028882	108151	Mm.89313	NM_028882	ENSMUSG00000040254	**Sema3d**	4631426B19Rik	sema domain, immunoglobulin domain (Ig), short basic domain, secreted, (semaphorin) 3D	Amp	protein-coding
2385_T22	chr3	7.5E+07	7.5E+07	5999	+	**1**	**12.5**	NA	Intergenic	Intergenic	60578	NM_026460	67931	Mm.259446	NM_026460	ENSMUSG00000034139	**Serpini2**	Spi14	serine (or cysteine) peptidase inhibitor, clade I, member 2	Amp	protein-coding
2386_T1	chr3	7.5E+07	7.5E+07	4999	+	**1**	**12.5**	NA	Intergenic	Intergenic	-1922	NM_026460	67931	Mm.259446	NM_026460	ENSMUSG00000034139	**Serpini2**	Spi14	serine (or cysteine) peptidase inhibitor, clade I, member 2	Amp	protein-coding
2232_T4	chr3	5.5E+07	5.5E+07	2999	+	**1**	**12.5**	NA	Intergenic	IAPEz-int|LTR|ERVK	42887	NM_177854	329641	Mm.51187	NM_177854	ENSMUSG00000056306	**Sertm1**	6030405A18Rik	serine rich and transmembrane domain containing 1	Amp	protein-coding

3476_T22	chr8	3.8E+07	3.8E+07	999	+	**1**	**12.5**	NA	intron (NM_145841, intron 1 of 7)	L1M3e|LINE|L1	184508	NM_145841	244431	Mm.210388	NM_145841	ENSMUSG00000039539	**Sgcz**	C230085N17Rik	sarcoglycan zeta	Amp	protein-coding
3395_T12	chr8	5019501	5020500	999	+	**1**	**12.5**	NA	Intergenic	Intergenic	85232	NM_011388	20494	Mm.3500	NM_011388	ENSMUSG00000023073	**Slc10a2**	9130221J18Rik|AI605518|ASBT|IBAT|ISBT	solute carrier family 10, member 2	Amp	protein-coding
3399_T2	chr8	5521501	5522500	999	+	**1**	**12.5**	NA	Intergenic	MLT1J2|LTR|ERVL-MaLR	-416768	NM_011388	20494	Mm.3500	NM_011388	ENSMUSG00000023073	**Slc10a2**	9130221J18Rik|AI605518|ASBT|IBAT|ISBT	solute carrier family 10, member 2	Amp	protein-coding
3401_T3	chr8	5901501	5905500	3999	+	**1**	**12.5**	NA	Intergenic	Intergenic	-798268	NM_011388	20494	Mm.3500	NM_011388	ENSMUSG00000023073	**Slc10a2**	9130221J18Rik|AI605518|ASBT|IBAT|ISBT	solute carrier family 10, member 2	Amp	protein-coding
3407_T1	chr8	6465501	6467500	1999	+	**1**	**12.5**	NA	Intergenic	Intergenic	-1E+06	NM_011388	20494	Mm.3500	NM_011388	ENSMUSG00000023073	**Slc10a2**	9130221J18Rik|AI605518|ASBT|IBAT|ISBT	solute carrier family 10, member 2	Amp	protein-coding
1186_T12	chr17	1.3E+07	1.3E+07	3999	+	**1**	**12.5**	NA	intron (NM_013667, intron 10 of 10)	(TTG)n|Simple_repeat|Simple_repeat	40311	NM_013667	20518	Mm.17322	NM_013667	ENSMUSG00000040966	**Slc22a2**	Oct2|Orct2	solute carrier family 22 (organic cation transporter), member 2	Amp	protein-coding
1078_T42	chr15	5.7E+07	5.7E+07	999	+	**1**	**12.5**	NA	intron (NM_172378, intron 2 of 11)	intron (NM_172378, intron 2 of 11)	208625	NM_172378	210463	Mm.26555	NM_172378	ENSMUSG00000022366	**Slc22a22**	oat-pg	solute carrier family 22 (organic cation transporter), member 22	Amp	protein-coding
1081_T32	chr15	5.7E+07	5.7E+07	999	+	**1**	**12.5**	NA	intron (NM_172378, intron 2 of 11)	RMER17A|LTR|ERVK	202625	NM_172378	210463	Mm.26555	NM_172378	ENSMUSG00000022366	**Slc22a22**	oat-pg	solute carrier family 22 (organic cation transporter), member 22	Amp	protein-coding
1333_T42	chr19	8501501	8507500	5999	+	**1**	**12.5**	NA	Intergenic	L1Md_F2|LINE|L1	-86754	NM_001164634	19879	Mm.285294	NM_031194	ENSMUSG00000063796	**Slc22a8**	Oat3|Roct	solute carrier family 22 (organic anion transporter), member 8	Amp	protein-coding
1502_T42	chr2	6.2E+07	6.2E+07	999	+	**1**	**12.5**	NA	intron (NM_033552, intron 2 of 25)	intron (NM_033552, intron 2 of 25)	114485	NM_033552	94229	Mm.314497	NM_033552	ENSMUSG00000026904	**Slc4a10**	NCBE|mKIAA4136	solute carrier family 4, sodium bicarbonate cotransporter-like, member 10	Amp	protein-coding
2160_T3	chr3	5.1E+07	5.1E+07	6999	+	**1**	**12.5**	NA	Intergenic	L1Md_A|LINE|L1	-100387	NM_011990	26570	Mm.260988	NM_011990	ENSMUSG00000027737	**Slc7a11**	9930009M05Rik|AI451155|sut|xCT	solute carrier family 7 (cationic amino acid transporter, y+ system), member 11	Amp	protein-coding
2162_T3	chr3	5.1E+07	5.1E+07	6999	+	**1**	**12.5**	NA	Intergenic	L1Md_A|LINE|L1	-226387	NM_011990	26570	Mm.260988	NM_011990	ENSMUSG00000027737	**Slc7a11**	9930009M05Rik|AI451155|sut|xCT	solute carrier family 7 (cationic amino acid transporter, y+ system), member 11	Amp	protein-coding

2166_T3	chr3	5.1E+07	5.1E+07	5999	+	**1**	**12.5**	NA	Intergenic	L1Md_A|LINE|L1	-255887	NM_011990	26570	Mm.260988	NM_011990	ENSMUSG00000027737	**Slc7a11**	9930009M05Rik|AI451155|sut|xCT	solute carrier family 7 (cationic amino acid transporter, y+ system), member 11	Amp	protein-coding
1740_T4	chr3	3.2E+07	3.2E+07	4999	+	**1**	**12.5**	NA	Intergenic	L1Md_F2|LINE|L1	-240681	NM_172861	241919	Mm.285366	NM_172861	ENSMUSG00000069072	**Slc7a14**	A930013N06|BC061928	solute carrier family 7 (cationic amino acid transporter, y+ system), member 14	Amp	protein-coding
2753_T1	chr5	4.8E+07	4.8E+07	3999	+	**1**	**12.5**	NA	Intergenic	B3A|SINE|B2	-478655	NR_111900	20563	Mm.289739	NM_178804	ENSMUSG00000031558	**Slit2**	Drad-1|E030015M03Rik|E130320P19Rik|Slil3|mKIAA4141|slit-2	slit homolog 2 (Drosophila)	Amp	protein-coding
805_T32	chr14	1.1E+08	1.1E+08	999	+	**1**	**12.5**	NA	Intergenic	Intergenic	829239	NM_199065	76965	Mm.257268	NM_199065	ENSMUSG00000075478	**Slitrk1**	3200001I04Rik	SLIT and NTRK-like family, member 1	Amp	protein-coding
806_T1	chr14	1.1E+08	1.1E+08	5999	+	**1**	**12.5**	NA	Intergenic	L1Md_F2|LINE|L1	437739	NM_199065	76965	Mm.257268	NM_199065	ENSMUSG00000075478	**Slitrk1**	3200001I04Rik	SLIT and NTRK-like family, member 1	Amp	protein-coding
808_T3	chr14	1.1E+08	1.1E+08	1999	+	**1**	**12.5**	NA	Intergenic	Intergenic	108739	NM_199065	76965	Mm.257268	NM_199065	ENSMUSG00000075478	**Slitrk1**	3200001I04Rik	SLIT and NTRK-like family, member 1	Amp	protein-coding
812_T2	chr14	1.1E+08	1.1E+08	999	+	**1**	**12.5**	NA	Intergenic	Intergenic	66239	NM_199065	76965	Mm.257268	NM_199065	ENSMUSG00000075478	**Slitrk1**	3200001I04Rik	SLIT and NTRK-like family, member 1	Amp	protein-coding
814_T22	chr14	1.1E+08	1.1E+08	999	+	**1**	**12.5**	NA	Intergenic	Intergenic	60239	NM_199065	76965	Mm.257268	NM_199065	ENSMUSG00000075478	**Slitrk1**	3200001I04Rik	SLIT and NTRK-like family, member 1	Amp	protein-coding
821_T12	chr14	1.1E+08	1.1E+08	999	+	**1**	**12.5**	NA	Intergenic	Intergenic	6239	NM_199065	76965	Mm.257268	NM_199065	ENSMUSG00000075478	**Slitrk1**	3200001I04Rik	SLIT and NTRK-like family, member 1	Amp	protein-coding
822_T32	chr14	1.1E+08	1.1E+08	3999	+	**1**	**12.5**	NA	Intergenic	Intergenic	-93261	NM_199065	76965	Mm.257268	NM_199065	ENSMUSG00000075478	**Slitrk1**	3200001I04Rik	SLIT and NTRK-like family, member 1	Amp	protein-coding
850_T22	chr14	1.1E+08	1.1E+08	3999	+	**1**	**12.5**	NA	Intergenic	ORR1A3-int|LTR|ERVL-MaLR	-207615	NM_198865	75409	Mm.235097	NM_029273	ENSMUSG00000033214	**Slitrk5**	2610019D03Rik	SLIT and NTRK-like family, member 5	Amp	protein-coding
851_T22	chr14	1.1E+08	1.1E+08	999	+	**1**	**12.5**	NA	Intergenic	Intergenic	9885	NM_198865	75409	Mm.235097	NM_029273	ENSMUSG00000033214	**Slitrk5**	2610019D03Rik	SLIT and NTRK-like family, member 5	Amp	protein-coding
853_T22	chr14	1.1E+08	1.1E+08	3999	+	**1**	**12.5**	NA	Intergenic	Lx7|LINE|L1	22385	NM_198865	75409	Mm.235097	NM_029273	ENSMUSG00000033214	**Slitrk5**	2610019D03Rik	SLIT and NTRK-like family, member 5	Amp	protein-coding
854_T4	chr14	1.1E+08	1.1E+08	3999	+	**1**	**12.5**	NA	Intergenic	Intergenic	82385	NM_198865	75409	Mm.235097	NM_029273	ENSMUSG00000033214	**Slitrk5**	2610019D03Rik	SLIT and NTRK-like family, member 5	Amp	protein-coding

842_T22	chr14	1.1E+08	1.1E+08	4999	+	**1**	**12.5**	NA	Intergenic	Intergenic	586149	NM_175499	239250	Mm.49728	NM_175499	ENSMUSG00000045871	**Slitrk6**	4832410J21Rik|Sltk6	SLIT and NTRK-like family, member 6	Amp	protein-coding
1332_T42	chr18	7.4E+07	7.4E+07	3999	+	**1**	**12.5**	NA	intron (NM_008540, intron 1 of 11)	L1Md_T|LINE|L1	19241	NM_008540	17128	Mm.100399	NM_008540	ENSMUSG00000024515	**Smad4**	AW743858|D18Wsu70e|DPC4|Madh4	SMAD family member 4	Amp	protein-coding
247_T3	chr10	4.6E+07	4.6E+07	999	+	**1**	**12.5**	NA	Intergenic	Intergenic	163251	NR_028276	108637		NR_028276	ENSMUSG00000096263	**Snord14c**	Gm23107|Rnu14|U14	small nucleolar RNA, C/D box 14C	Amp	snoRNA
249_T42	chr10	4.6E+07	4.6E+07	3999	+	**1**	**12.5**	NA	Intergenic	MIR|SINE|MIR	175751	NR_028276	108637		NR_028276	ENSMUSG00000096263	**Snord14c**	Gm23107|Rnu14|U14	small nucleolar RNA, C/D box 14C	Amp	snoRNA
251_T1	chr10	4.7E+07	4.7E+07	999	+	**1**	**12.5**	NA	Intergenic	Intergenic	912251	NR_028276	108637		NR_028276	ENSMUSG00000096263	**Snord14c**	Gm23107|Rnu14|U14	small nucleolar RNA, C/D box 14C	Amp	snoRNA
254_T42	chr10	4.7E+07	4.7E+07	999	+	**1**	**12.5**	NA	Intergenic	L1_Mus2|LINE|L1	999251	NR_028276	108637		NR_028276	ENSMUSG00000096263	**Snord14c**	Gm23107|Rnu14|U14	small nucleolar RNA, C/D box 14C	Amp	snoRNA
257_T42	chr10	4.7E+07	4.7E+07	4999	+	**1**	**12.5**	NA	Intergenic	Intergenic	1015251	NR_028276	108637		NR_028276	ENSMUSG00000096263	**Snord14c**	Gm23107|Rnu14|U14	small nucleolar RNA, C/D box 14C	Amp	snoRNA
3365_T32	chr7	6E+07	6.1E+07	340999	+	**1**	**12.5**	NA	Intergenic	L1Md_F2|LINE|L1	-434781	NM_001082962	20646	Mm.274995	NM_013670	ENSMUSG00000102252	**Snrpn**	2410045I01Rik|HCERN3|Peg4|Pwcr1|SMN	small nuclear ribonucleoprotein N	Amp	protein-coding
1_T1	chr1	9266501	9274500	7999	+	**1**	**12.5**	NA	intron (NM_001290390, intron 1 of 20)	intron (NM_001290390, intron 1 of 20)	29377	NM_001290390	71096	Mm.336395	NM_027671	ENSMUSG00000025909	**Sntg1**	4933426D16Rik|G1SYN|SYN4	syntrophin, gamma 1	Amp	protein-coding
3773_T3	chr9	8.8E+07	8.8E+07	3999	+	**1**	**12.5**	NA	3' UTR (NM_001284328, exon 12 of 12)	3' UTR (NM_001284328, exon 12 of 12)	-11549	NM_172926	244962	Mm.439873	NM_172926	ENSMUSG00000032422	**Snx14**	B830022K16|C330035N22Rik|YR-14	sorting nexin 14	Amp	protein-coding
1183_T12	chr17	5880501	5885500	4999	+	**1**	**12.5**	NA	intron (NM_025664, intron 1 of 17)	intron (NM_025664, intron 1 of 17)	41620	NM_025664	66616	Mm.89515	NM_025664	ENSMUSG00000002365	**Snx9**	2700073N08Rik|SDP1|SH3PX1	sorting nexin 9	Amp	protein-coding
1355_T22	chr19	4.9E+07	4.9E+07	3999	+	**1**	**12.5**	NA	Intergenic	RMER15-int|LTR|ERVL	1184146	NM_001252501	58178	Mm.313672	NM_021377	ENSMUSG00000043531	**Sorcs1**	Sorcs|mSorCS	sortilin-related VPS10 domain containing receptor 1	Amp	protein-coding
1356_T4	chr19	5.1E+07	5.1E+07	3999	+	**1**	**12.5**	NA	Intergenic	L1Md_A|LINE|L1	-186854	NM_001290356	58178	Mm.313672	NM_021377	ENSMUSG00000043531	**Sorcs1**	Sorcs|mSorCS	sortilin-related VPS10 domain containing receptor 1	Amp	protein-coding
1362_T2	chr19	5.1E+07	5.1E+07	5999	+	**1**	**12.5**	NA	Intergenic	Intergenic	-377854	NM_001290356	58178	Mm.313672	NM_021377	ENSMUSG00000043531	**Sorcs1**	Sorcs|mSorCS	sortilin-related VPS10 domain containing receptor 1	Amp	protein-coding

1351_T22	chr19	4.9E+07	4.9E+07	999	+	**1**	**12.5**	NA	Intergenic	Intergenic	961975	NM_025696	66673	Mm.70980	NM_025696	ENSMUSG00000063434	**Sorcs3**	6330404A12Rik|AW045579	sortilin-related VPS10 domain containing receptor 3	Amp	protein-coding
451_T3	chr12	6.3E+07	6.3E+07	1999	+	**1**	**12.5**	NA	Intergenic	Intergenic	30713	NR_131949	73309	Mm.276558	NR_131949		**Spanxn4**	1700047L15Rik	SPANX family, member N4	Amp	ncRNA
455_T12	chr12	6.3E+07	6.3E+07	3999	+	**1**	**12.5**	NA	Intergenic	Intergenic	554713	NR_131949	73309	Mm.276558	NR_131949		**Spanxn4**	1700047L15Rik	SPANX family, member N4	Amp	ncRNA
3576_T1	chr8	6.3E+07	6.3E+07	2999	+	**1**	**12.5**	NA	intron (NM_023689, intron 7 of 11)	RLTR42-int|LTR|ERVK	373655	NM_023689	72902	Mm.334552	NM_023689	ENSMUSG00000054162	**Spock3**	2900045C01Rik|AI428471|mKIAA4039	sparc/osteonectin, cwcv and kazal-like domains proteoglycan 3	Amp	protein-coding
1432_T42	chr2	2.4E+07	2.4E+07	5999	+	**1**	**12.5**	NA	Intergenic	RLTR10|LTR|ERVK	-213396	NM_001165997	76857	Mm.276415	NM_029773	ENSMUSG00000026771	**Spopl**	4921517N04Rik|AU014935|BB233739|E430033K04Rik	speckle-type POZ protein-like	Amp	protein-coding
1435_T32	chr2	2.4E+07	2.4E+07	999	+	**1**	**12.5**	NA	Intergenic	L1Md_F2|LINE|L1	-227896	NM_001165997	76857	Mm.276415	NM_029773	ENSMUSG00000026771	**Spopl**	4921517N04Rik|AU014935|BB233739|E430033K04Rik	speckle-type POZ protein-like	Amp	protein-coding
2440_T3	chr3	9.2E+07	9.2E+07	4999	+	**1**	**12.5**	NA	Intergenic	Intergenic	-10705	NM_011469	20756	Mm.445310	NM_011469	ENSMUSG00000050092	**Sprr2b**	-	small proline-rich protein 2B	Amp	protein-coding
3372_T32	chr7	7.4E+07	7.4E+07	5999	+	**1**	**12.5**	NA	Intergenic	Lx8|LINE|L1	-192818	NM_009181	20450	Mm.4954	NM_009181	ENSMUSG00000025789	**St8sia2**	AI323367|ST8SiaII|STX|Siat8b	ST8 alpha-N-acetyl-neuraminide alpha-2,8-sialyltransferase 2	Amp	protein-coding
60_T4	chr1	9.6E+07	9.6E+07	3999	+	**1**	**12.5**	NA	Intergenic	Intergenic	-17906	NM_001159745	20452	Mm.306228	NM_009183	ENSMUSG00000040710	**St8sia4**	PST|PST-1|SIAT8-D|ST8SiaIV|Siat8d	ST8 alpha-N-acetyl-neuraminide alpha-2,8-sialyltransferase 4	Amp	protein-coding
59_T2	chr1	9.6E+07	9.6E+07	999	+	**1**	**12.5**	NA	intron (NM_001159745, intron 1 of 3)	intron (NM_001159745, intron 1 of 3)	4594	NM_009183	20452	Mm.306228	NM_009183	ENSMUSG00000040710	**St8sia4**	PST|PST-1|SIAT8-D|ST8SiaIV|Siat8d	ST8 alpha-N-acetyl-neuraminide alpha-2,8-sialyltransferase 4	Amp	protein-coding
2693_T22	chr5	6383501	6384500	999	+	**1**	**12.5**	NA	Intergenic	Intergenic	-634683	NM_027399	70358	Mm.85429	NM_027399	ENSMUSG00000015652	**Steap1**	2410007B19Rik|Prss24|Steap	six transmembrane epithelial antigen of the prostate 1	Amp	protein-coding
2696_T4	chr5	7789501	7794500	4999	+	**1**	**12.5**	NA	Intergenic	Intergenic	-168472	NM_054098	117167	Mm.31403	NM_054098	ENSMUSG00000012428	**Steap4**	1110021O17Rik|AI481214|Tiarp|Tnfaip9	STEAP family member 4	Amp	protein-coding
433_T12	chr12	4.6E+07	4.6E+07	999	+	**1**	**12.5**	NA	Intergenic	Intergenic	-802517	NM_144552	217517	Mm.285400	NM_144552	ENSMUSG00000046314	**Stxbp6**	BC024598|C85317	syntaxin binding protein 6 (amisyn)	Amp	protein-coding
2361_T32	chr3	6E+07	6E+07	999	+	**1**	**12.5**	NA	Intergenic	Intergenic	125131	NM_032400	84112	Mm.125110	NM_032400	ENSMUSG00000027762	**Sucnr1**	Gpr91	succinate receptor 1	Amp	protein-coding
2363_T32	chr3	6E+07	6E+07	9999	+	**1**	**12.5**	NA	Intergenic	Intergenic	170631	NM_032400	84112	Mm.125110	NM_032400	ENSMUSG00000027762	**Sucnr1**	Gpr91	succinate receptor 1	Amp	protein-coding

501_T42	chr13	1.7E+07	1.7E+07	999	+	**1**	**12.5**	NA	intron (NM_138654, intron 11 of 13)	L1_Mm|LINE|L1	414765	NM_138654	192136	Mm.364180	NM_138654	ENSMUSG00000055137	**Sugct**	5033411D12Rik|D17907	succinyl-CoA glutarate-CoA transferase	Amp	protein-coding
3325_T32	chr7	1.4E+07	1.4E+07	999	+	**1**	**12.5**	NA	Intergenic	L1_Mus1|LINE|L1	-4590	NM_001111296	20859	Mm.260026	NM_001111296	ENSMUSG00000078798	**Sult2a1**	ST2A1|Std|Sth1|mSTa1	sulfotransferase family 2A, dehydroepiandrosterone (DHEA)-preferring, member 1	Amp	protein-coding
3781_T12	chr9	1.1E+08	1.1E+08	999	+	**1**	**12.5**	NA	Intergenic	Intergenic	114646	NM_001101510	382111	Mm.376140	NM_001101510	ENSMUSG00000086596	**Susd5**	Gm1126	sushi domain containing 5	Amp	protein-coding
1184_T12	chr17	7907501	7908500	999	+	**1**	**12.5**	NA	Intergenic	(TCC)n|Simple_repeat|Simple_repeat	-18000	NM_145968	72536	Mm.119680	NM_145968	ENSMUSG00000033450	**Tagap**	2610315E15Rik	T cell activation Rho GTPase activating protein	Amp	protein-coding
3601_T32	chr8	8.3E+07	8.3E+07	4999	+	**1**	**12.5**	NA	Intergenic	L1Md_A|LINE|L1	-47352	NM_001111304	71310	Mm.24031	NM_027758	ENSMUSG00000031709	**Tbc1d9**	4933431N12Rik|AI847101|AW490653|C76116	TBC1 domain family, member 9	Amp	protein-coding
684_T22	chr14	8.8E+07	8.8E+07	4999	+	**1**	**12.5**	NA	Intergenic	Lx7|LINE|L1	364417	NM_001253755	219249	Mm.35168	NM_172605	ENSMUSG00000022019	**Tdrd3**	4732418C03Rik|6720468N18	tudor domain containing 3	Amp	protein-coding
2868_T2	chr5	8.3E+07	8.3E+07	4999	+	**1**	**12.5**	NA	Intergenic	Intergenic	108195	NM_153801	243078	Mm.126783	NM_153801	ENSMUSG00000049537	**Tecrl**	D330017N19Rik|Srd5a2l2	trans-2,3-enoyl-CoA reductase-like	Amp	protein-coding
3500_T32	chr8	4.9E+07	4.9E+07	999	+	**1**	**12.5**	NA	Intergenic	Intergenic	-461310	NM_011857	23965	Mm.42191	NM_011857	ENSMUSG00000031561	**Tenm3**	2610100B16Rik|Odz1|Odz3|Ten-m3|mKIAA1455	teneurin transmembrane protein 3	Amp	protein-coding
2910_T32	chr6	1.6E+07	1.6E+07	83999	+	**1**	**12.5**	NA	Intergenic	Intergenic	549941	NM_031198	21426	Mm.36217	NM_031198	ENSMUSG00000029553	**Tfec**	BB107417|Tcfec|bHLHe34	transcription factor EC	Amp	protein-coding
2911_T32	chr6	1.6E+07	1.6E+07	5999	+	**1**	**12.5**	NA	Intergenic	L1Md_T|LINE|L1	503941	NM_031198	21426	Mm.36217	NM_031198	ENSMUSG00000029553	**Tfec**	BB107417|Tcfec|bHLHe34	transcription factor EC	Amp	protein-coding
2912_T32	chr6	1.6E+07	1.7E+07	156999	+	**1**	**12.5**	NA	Intergenic	Lx8|LINE|L1	421441	NM_031198	21426	Mm.36217	NM_031198	ENSMUSG00000029553	**Tfec**	BB107417|Tcfec|bHLHe34	transcription factor EC	Amp	protein-coding
2913_T32	chr6	1.7E+07	1.7E+07	7999	+	**1**	**12.5**	NA	Intergenic	Lx2|LINE|L1	337941	NM_031198	21426	Mm.36217	NM_031198	ENSMUSG00000029553	**Tfec**	BB107417|Tcfec|bHLHe34	transcription factor EC	Amp	protein-coding
2914_T32	chr6	1.7E+07	1.7E+07	29999	+	**1**	**12.5**	NA	Intergenic	Intergenic	317941	NM_031198	21426	Mm.36217	NM_031198	ENSMUSG00000029553	**Tfec**	BB107417|Tcfec|bHLHe34	transcription factor EC	Amp	protein-coding
2915_T32	chr6	1.7E+07	1.7E+07	4999	+	**1**	**12.5**	NA	Intergenic	L1Md_T|LINE|L1	299441	NM_031198	21426	Mm.36217	NM_031198	ENSMUSG00000029553	**Tfec**	BB107417|Tcfec|bHLHe34	transcription factor EC	Amp	protein-coding
2916_T32	chr6	1.7E+07	1.7E+07	196999	+	**1**	**12.5**	NA	Intergenic	Intergenic	197441	NM_031198	21426	Mm.36217	NM_031198	ENSMUSG00000029553	**Tfec**	BB107417|Tcfec|bHLHe34	transcription factor EC	Amp	protein-coding

2926_T32	chr6	1.7E+07	1.7E+07	999	+	**1**	**12.5**	NA	Intergenic	Intergenic	-45559	NM_031198	21426	Mm.36217	NM_031198	ENSMUSG00000029553	**Tfec**	BB107417|Tcfec|bHLHe34	transcription factor EC	Amp	protein-coding
1428_T32	chr2	2.1E+07	2.1E+07	4999	+	**1**	**12.5**	NA	Intergenic	L1Md_A|LINE|L1	44276	NM_177588	208967	Mm.268841	NM_177588	ENSMUSG00000048550	**Thnsl1**	AW413632	threonine synthase-like 1 (bacterial)	Amp	protein-coding
3583_T22	chr8	6.7E+07	6.7E+07	2999	+	**1**	**12.5**	NA	Intergenic	Intergenic	48260	NM_028927	74419	Mm.159193	NM_028927	ENSMUSG00000025519	**Tktl2**	4933401I19Rik	transketolase-like 2	Amp	protein-coding
2615_T2	chr4	7.2E+07	7.2E+07	3999	+	**1**	**12.5**	NA	Intergenic	Lx8|LINE|L1	628393	NM_011599	21885	Mm.278444	NM_011599	ENSMUSG00000008305	**Tle1**	C230057C06Rik|Estm14|Grg-1|Grg1|Tle4l	transducin-like enhancer of split 1	Amp	protein-coding
2597_T22	chr4	6.7E+07	6.7E+07	5999	+	**1**	**12.5**	NA	Intergenic	Intergenic	286949	NM_021297	21898	Mm.38049	NM_021297	ENSMUSG00000039005	**Tlr4**	Lps|Ly87|Ran/M1|Rasl2-8	toll-like receptor 4	Amp	protein-coding
2598_T32	chr4	6.7E+07	6.7E+07	999	+	**1**	**12.5**	NA	Intergenic	Intergenic	552449	NM_021297	21898	Mm.38049	NM_021297	ENSMUSG00000039005	**Tlr4**	Lps|Ly87|Ran/M1|Rasl2-8	toll-like receptor 4	Amp	protein-coding
2600_T22	chr4	6.7E+07	6.7E+07	999	+	**1**	**12.5**	NA	Intergenic	Intergenic	559449	NM_021297	21898	Mm.38049	NM_021297	ENSMUSG00000039005	**Tlr4**	Lps|Ly87|Ran/M1|Rasl2-8	toll-like receptor 4	Amp	protein-coding
2289_T42	chr3	5.7E+07	5.7E+07	7999	+	**1**	**12.5**	NA	Intergenic	L1Md_T|LINE|L1	394419	NM_008536	17112	Mm.856	NM_008536	ENSMUSG00000027800	**Tm4sf1**	L6|M3s1	transmembrane 4 superfamily member 1	Amp	protein-coding
2314_T42	chr3	5.7E+07	5.7E+07	6999	+	**1**	**12.5**	NA	Intergenic	L1Md_A|LINE|L1	-23081	NM_008536	17112	Mm.856	NM_008536	ENSMUSG00000027800	**Tm4sf1**	L6|M3s1	transmembrane 4 superfamily member 1	Amp	protein-coding
2316_T2	chr3	5.7E+07	5.7E+07	999	+	**1**	**12.5**	NA	Intergenic	L1Md_F2|LINE|L1	-54081	NM_008536	17112	Mm.856	NM_008536	ENSMUSG00000027800	**Tm4sf1**	L6|M3s1	transmembrane 4 superfamily member 1	Amp	protein-coding
2317_T1	chr3	5.7E+07	5.7E+07	999	+	**1**	**12.5**	NA	Intergenic	L1Md_F2|LINE|L1	-58081	NM_008536	17112	Mm.856	NM_008536	ENSMUSG00000027800	**Tm4sf1**	L6|M3s1	transmembrane 4 superfamily member 1	Amp	protein-coding
26_T3	chr1	4.9E+07	4.9E+07	5999	+	**1**	**12.5**	NA	Intergenic	Intergenic	-1E+06	NM_019790	56363	Mm.245154	NM_019790	ENSMUSG00000026109	**Tmeff2**	4832418D20Rik|7630402F16Rik|TR-2	transmembrane protein with EGF-like and two follistatin-like domains 2	Amp	protein-coding
27_T2	chr1	5E+07	5E+07	3999	+	**1**	**12.5**	NA	Intergenic	Intergenic	-1E+06	NM_019790	56363	Mm.245154	NM_019790	ENSMUSG00000026109	**Tmeff2**	4832418D20Rik|7630402F16Rik|TR-2	transmembrane protein with EGF-like and two follistatin-like domains 2	Amp	protein-coding
35_T42	chr1	5.1E+07	5.1E+07	999	+	**1**	**12.5**	NA	Intergenic	Intergenic	-284523	NM_019790	56363	Mm.245154	NM_019790	ENSMUSG00000026109	**Tmeff2**	4832418D20Rik|7630402F16Rik|TR-2	transmembrane protein with EGF-like and two follistatin-like domains 2	Amp	protein-coding
3384_T42	chr7	9.1E+07	9.1E+07	3999	+	**1**	**12.5**	NA	Intergenic	L1Md_T|LINE|L1	-54505	NM_026734	68472	Mm.289791	NM_026734	ENSMUSG00000030614	**Tmem126b**	1110001A23Rik	transmembrane protein 126B	Amp	protein-coding

557_T2	chr13	8.5E+07	8.5E+07	999	+	**1**	**12.5**	NA	Intergenic	L1_Mur3|LINE|L1	295704	NM_175187	72745	Mm.295063	NM_175187	ENSMUSG00000035762	**Tmem161b**	2810446P07Rik|AI843389	transmembrane protein 161B	Amp	protein-coding
367_T2	chr11	2.3E+07	2.3E+07	3999	+	**1**	**12.5**	NA	Intergenic	B2_Mm1a|SINE|B2	152217	NM_153596	103765	Mm.221274	NM_153596	ENSMUSG00000049904	**Tmem17**	AI503894|AW049890	transmembrane protein 17	Amp	protein-coding
481_T4	chr12	1.2E+08	1.2E+08	999	+	**1**	**12.5**	NA	intron (NM_001294146, intron 1 of 3)	intron (NM_001294146, intron 1 of 3)	59038	NM_001160385	217951	Mm.387395	NM_001160385	ENSMUSG00000048004	**Tmem196**	Gm528	transmembrane protein 196	Amp	protein-coding
2595_T2	chr4	6.4E+07	6.4E+07	1999	+	**1**	**12.5**	NA	Intergenic	Intergenic	-355485	NM_011607	21923	Mm.980	NM_011607	ENSMUSG00000028364	**Tnc**	AI528729|C130033P17Rik|Hxb|TN|TN-C|Ten|cytotactin|tenascin-C	tenascin C	Amp	protein-coding
618_T12	chr14	6.1E+07	6.1E+07	2999	+	**1**	**12.5**	NA	Intergenic	MTA_Mm-int|LTR|ERVL-MaLR	76987	NM_001164155	29820	Mm.281356	NM_013869	ENSMUSG00000060548	**Tnfrsf19**	AL023044|AW123854|TAJ|TAJ-ALPHA|TRADE|Troy	tumor necrosis factor receptor superfamily, member 19	Amp	protein-coding
2596_T3	chr4	6.6E+07	6.6E+07	4999	+	**1**	**12.5**	NA	intron (NM_207109, intron 13 of 22)	intron (NM_207109, intron 13 of 22)	92014	NM_001161782	69807	Mm.22786	NM_053084	ENSMUSG00000051675	**Trim32**	1810045E12Rik|3f3|BBS11|Zfp117	tripartite motif-containing 32	Amp	protein-coding
3494_T42	chr8	4.3E+07	4.3E+07	3999	+	**1**	**12.5**	NA	Intergenic	L1Md_A|LINE|L1	-12014	NM_177742	244448	Mm.64542	NM_177742	ENSMUSG00000031651	**Triml1**	4933403D14|BC050188	tripartite motif family-like 1	Amp	protein-coding
3716_T12	chr9	8385501	8444500	58999	+	**1**	**12.5**	NA	Intergenic	Intergenic	-129142	NM_013838	22068	Mm.325086	NM_013838	ENSMUSG00000031997	**Trpc6**	AV025995|LLHWJM002|LLHWJM003|LLHWJM004|TRP-6|Trrp6|mtrp6	transient receptor potential cation channel, subfamily C, member 6	Amp	protein-coding
3717_T12	chr9	8445501	8458500	12999	+	**1**	**12.5**	NA	Intergenic	Lx5c|LINE|L1	-92142	NM_013838	22068	Mm.325086	NM_013838	ENSMUSG00000031997	**Trpc6**	AV025995|LLHWJM002|LLHWJM003|LLHWJM004|TRP-6|Trrp6|mtrp6	transient receptor potential cation channel, subfamily C, member 6	Amp	protein-coding
3718_T12	chr9	8459501	8466500	6999	+	**1**	**12.5**	NA	Intergenic	Intergenic	-81142	NM_013838	22068	Mm.325086	NM_013838	ENSMUSG00000031997	**Trpc6**	AV025995|LLHWJM002|LLHWJM003|LLHWJM004|TRP-6|Trrp6|mtrp6	transient receptor potential cation channel, subfamily C, member 6	Amp	protein-coding
3719_T12	chr9	8467501	8474500	6999	+	**1**	**12.5**	NA	Intergenic	L1Md_T|LINE|L1	-73142	NM_013838	22068	Mm.325086	NM_013838	ENSMUSG00000031997	**Trpc6**	AV025995|LLHWJM002|LLHWJM003|LLHWJM004|TRP-6|Trrp6|mtrp6	transient receptor potential cation channel, subfamily C, member 6	Amp	protein-coding
3720_T12	chr9	8475501	8586500	110999	+	**1**	**12.5**	NA	Intergenic	L1VL4|LINE|L1	-13142	NM_013838	22068	Mm.325086	NM_013838	ENSMUSG00000031997	**Trpc6**	AV025995|LLHWJM002|LLHWJM003|LLHWJM004|TRP-6|Trrp6|mtrp6	transient receptor potential cation channel, subfamily C, member 6	Amp	protein-coding

3721_T12	chr9	8587501	8626500	38999	+	**1**	**12.5**	NA	intron (NM_013838, intron 1 of 12)	intron (NM_013838, intron 1 of 12)	62858	NM_001282087	22068	Mm.325086	NM_013838	ENSMUSG00000031997	**Trpc6**	AV025995|LLHWJM002|LLHWJM003|LLHWJM004|TRP-6|Trrp6|mtrp6	transient receptor potential cation channel, subfamily C, member 6	Amp	protein-coding
1040_T12	chr15	5E+07	5E+07	999	+	**1**	**12.5**	NA	Intergenic	Lx7|LINE|L1	955049	NM_001310481	83925	Mm.30466	NM_032000	ENSMUSG00000038679	**Trps1**	AI115454|AI447310|D15Ertd586e	trichorhinophalangeal syndrome I (human)	Amp	protein-coding
1042_T32	chr15	5E+07	5E+07	3999	+	**1**	**12.5**	NA	Intergenic	Intergenic	805549	NM_001310481	83925	Mm.30466	NM_032000	ENSMUSG00000038679	**Trps1**	AI115454|AI447310|D15Ertd586e	trichorhinophalangeal syndrome I (human)	Amp	protein-coding
1052_T22	chr15	5E+07	5E+07	999	+	**1**	**12.5**	NA	Intergenic	Intergenic	416049	NM_001310481	83925	Mm.30466	NM_032000	ENSMUSG00000038679	**Trps1**	AI115454|AI447310|D15Ertd586e	trichorhinophalangeal syndrome I (human)	Amp	protein-coding
1058_T4	chr15	5.1E+07	5.1E+07	999	+	**1**	**12.5**	NA	intron (NM_001310481, intron 4 of 5)	intron (NM_001310481, intron 4 of 5)	159049	NM_001310481	83925	Mm.30466	NM_032000	ENSMUSG00000038679	**Trps1**	AI115454|AI447310|D15Ertd586e	trichorhinophalangeal syndrome I (human)	Amp	protein-coding
1062_T1	chr15	5.1E+07	5.1E+07	999	+	**1**	**12.5**	NA	intron (NM_001310485, intron 5 of 7)	(GA)n|Simple_repeat|Simple_repeat	75049	NM_001310481	83925	Mm.30466	NM_032000	ENSMUSG00000038679	**Trps1**	AI115454|AI447310|D15Ertd586e	trichorhinophalangeal syndrome I (human)	Amp	protein-coding
1505_T32	chr2	6.6E+07	6.6E+07	999	+	**1**	**12.5**	NA	intron (NM_001313997, intron 24 of 26)	L1_Mur1|LINE|L1	-26383	NM_001290669	73668	Mm.250868	NM_001047604	ENSMUSG00000034848	**Ttc21b**	2410066K11Rik|Thm1|aln|mKIAA1992	tetratricopeptide repeat domain 21B	Amp	protein-coding
2509_T1	chr3	1.5E+08	1.5E+08	3999	+	**1**	**12.5**	NA	Intergenic	Intergenic	644133	NM_001302958	70892	Mm.187793	NM_027594	ENSMUSG00000036745	**Ttll7**	1110049N09Rik|4921517B04Rik|C630030B20|mTTLL7	tubulin tyrosine ligase-like family, member 7	Amp	protein-coding
2644_T4	chr4	9.3E+07	9.3E+07	999	+	**1**	**12.5**	NA	Intergenic	Lx5c|LINE|L1	493511	NM_026954	69136	Mm.171558	NM_026954	ENSMUSG00000054000	**Tusc1**	2200001D17Rik|TSG-9	tumor suppressor candidate 1	Amp	protein-coding
2649_T2	chr4	9.3E+07	9.3E+07	999	+	**1**	**12.5**	NA	Intergenic	B1_Mur1|SINE|Alu	194511	NM_026954	69136	Mm.171558	NM_026954	ENSMUSG00000054000	**Tusc1**	2200001D17Rik|TSG-9	tumor suppressor candidate 1	Amp	protein-coding
2650_T22	chr4	9.3E+07	9.3E+07	999	+	**1**	**12.5**	NA	Intergenic	MTC|LTR|ERVL-MaLR	23511	NM_026954	69136	Mm.171558	NM_026954	ENSMUSG00000054000	**Tusc1**	2200001D17Rik|TSG-9	tumor suppressor candidate 1	Amp	protein-coding
3353_T32	chr7	5.9E+07	5.9E+07	4999	+	**1**	**12.5**	NA	5' UTR (NM_011668, exon 2 of 13)	5' UTR (NM_011668, exon 2 of 13)	12248	NM_011668	22215	Mm.9002	NM_011668	ENSMUSG00000025326	**Ube3a**	4732496B02|5830462N02Rik|A130086L21Rik|Hpve6a|mKIAA4216	ubiquitin protein ligase E3A	Amp	protein-coding
3354_T32	chr7	5.9E+07	5.9E+07	999	+	**1**	**12.5**	NA	intron (NM_001033962, intron 3 of 10)	intron (NM_001033962, intron 3 of 10)	44248	NM_011668	22215	Mm.9002	NM_011668	ENSMUSG00000025326	**Ube3a**	4732496B02|5830462N02Rik|A130086L21Rik|Hpve6a|mKIAA4216	ubiquitin protein ligase E3A	Amp	protein-coding

2552_T1	chr4	2.5E+07	2.5E+07	9999	+	**1**	**12.5**	NA	Intergenic	L1_Mur3|LINE|L1	-11679	NM_026194	67490	Mm.226295	NM_026194	ENSMUSG00000040359	**Ufl1**	1810074P20Rik|AI429228|Kiaa0776|Maxer|Rcad|mKIAA0776	UFM1 specific ligase 1	Amp	protein-coding
3785_T22	chr9	1.2E+08	1.2E+08	999	+	**1**	**12.5**	NA	intron (NM_177589, intron 17 of 36)	GA-rich|Low_complexity|Low_complexity	40172	NM_177589	209012	Mm.371804	NM_177589	ENSMUSG00000040936	**Ulk4**	4932415A06Rik|A730098P15	unc-51-like kinase 4	Amp	protein-coding
3431_T1	chr8	2.9E+07	2.9E+07	999	+	**1**	**12.5**	NA	Intergenic	Intergenic	-183364	NM_153135	210801	Mm.248433	NM_153135	ENSMUSG00000063626	**Unc5d**	D930029E11Rik|Unc5h4|mKIAA1777	unc-5 netrin receptor D	Amp	protein-coding
3432_T2	chr8	2.9E+07	2.9E+07	3999	+	**1**	**12.5**	NA	Intergenic	Intergenic	-228864	NM_153135	210801	Mm.248433	NM_153135	ENSMUSG00000063626	**Unc5d**	D930029E11Rik|Unc5h4|mKIAA1777	unc-5 netrin receptor D	Amp	protein-coding
3540_T3	chr8	5.4E+07	5.4E+07	999	+	**1**	**12.5**	NA	Intergenic	L1_Mm|LINE|L1	-98532	NM_009506	22341	Mm.1402	NM_009506	ENSMUSG00000031520	**Vegfc**	AW228853|VEGF-C	vascular endothelial growth factor C	Amp	protein-coding
3543_T42	chr8	5.4E+07	5.4E+07	999	+	**1**	**12.5**	NA	Intergenic	Intergenic	-93532	NM_009506	22341	Mm.1402	NM_009506	ENSMUSG00000031520	**Vegfc**	AW228853|VEGF-C	vascular endothelial growth factor C	Amp	protein-coding
3547_T4	chr8	5.4E+07	5.4E+07	999	+	**1**	**12.5**	NA	intron (NM_009506, intron 4 of 6)	intron (NM_009506, intron 4 of 6)	93468	NM_009506	22341	Mm.1402	NM_009506	ENSMUSG00000031520	**Vegfc**	AW228853|VEGF-C	vascular endothelial growth factor C	Amp	protein-coding
357_T4	chr11	1.5E+07	1.5E+07	4999	+	**1**	**12.5**	NA	Intergenic	MTE-int|LTR|ERVL-MaLR	-764724	NM_001290539	211739	Mm.284015	NM_145967	ENSMUSG00000048834	**Vstm2a**	Vstm2	V-set and transmembrane domain containing 2A	Amp	protein-coding
364_T2	chr11	1.6E+07	1.6E+07	999	+	**1**	**12.5**	NA	Intergenic	MLT1A0|LTR|ERVL-MaLR	-595724	NM_001290539	211739	Mm.284015	NM_145967	ENSMUSG00000048834	**Vstm2a**	Vstm2	V-set and transmembrane domain containing 2A	Amp	protein-coding
366_T12	chr11	1.6E+07	1.6E+07	999	+	**1**	**12.5**	NA	Intergenic	ORR1C2-int|LTR|ERVL-MaLR	-124724	NM_001290539	211739	Mm.284015	NM_145967	ENSMUSG00000048834	**Vstm2a**	Vstm2	V-set and transmembrane domain containing 2A	Amp	protein-coding
3711_T12	chr9	7849501	8048500	198999	+	**1**	**12.5**	NA	intron (NM_009534, intron 5 of 7)	intron (NM_009534, intron 5 of 7)	55596	NM_001171147	22601	Mm.221992	NM_009534	ENSMUSG00000053110	**Yap1**	AI325207|Yap|Yap65|Yki|Yorkie	yes-associated protein 1	Amp	protein-coding
1702_T32	chr3	1.7E+07	1.7E+07	999	+	**1**	**12.5**	NA	Intergenic	L1Md_F2|LINE|L1	384817	NM_001145919	229096	Mm.23834	NM_172677	ENSMUSG00000047213	**Ythdf3**	9130022A11Rik	YTH domain family 3	Amp	protein-coding
1692_T12	chr3	1.6E+07	1.6E+07	999	+	**1**	**12.5**	NA	intron (NM_172677, intron 2 of 5)	intron (NM_172677, intron 2 of 5)	1817	NM_001145919	229096	Mm.23834	NM_172677	ENSMUSG00000047213	**Ythdf3**	9130022A11Rik	YTH domain family 3	Amp	protein-coding
1696_T22	chr3	1.6E+07	1.6E+07	999	+	**1**	**12.5**	NA	intron (NR_027375, intron 4 of 5)	intron (NR_027375, intron 4 of 5)	8817	NM_001145919	229096	Mm.23834	NM_172677	ENSMUSG00000047213	**Ythdf3**	9130022A11Rik	YTH domain family 3	Amp	protein-coding

2384_T22	chr3	7.5E+07	7.5E+07	4999	+	**1**	**12.5**	NA	intron (NM_172515, intron 6 of 16)	L1Md_F2|LINE|L1	42772	NM_001310529	213234	Mm.80075	NM_172515	ENSMUSG00000034151	**Zbbx**	4931432L23	zinc finger, B-box domain containing	Amp	protein-coding
1663_T4	chr3	7540501	7545500	4999	+	**1**	**12.5**	NA	intron (NM_173181, intron 8 of 8)	L1Md_F2|LINE|L1	39574	NM_173181	67306	Mm.332366	NM_173181	ENSMUSG00000043542	**Zc2hc1a**	3110050N22Rik|AI790358|AU023959|Fam164a	zinc finger, C2HC-type containing 1A	Amp	protein-coding
3774_T1	chr9	9.1E+07	9.1E+07	3999	+	**1**	**12.5**	NA	Intergenic	L1_Rod|LINE|L1	452299	NM_009573	22771	Mm.335350	NM_009573	ENSMUSG00000032368	**Zic1**	ZIC|ZNF201	zinc finger protein of the cerebellum 1	Amp	protein-coding
1275_T12	chr17	5.7E+07	5.7E+07	3999	+	**1**	**12.5**	NA	Intergenic	Intergenic	-20017	NM_011483	20834	Mm.76862	NM_011483	ENSMUSG00000044526	**Znrf4**	1700094M01Rik|Ssrzf1|spzn	zinc and ring finger 4	Amp	protein-coding

Supplementary Table S3. The Profiles of CNDs and CND-affected Genes in the Mouse PtenI/Tp53I Mammary Tumors

PeakID (cmd=annotatePeaks.pl LossRegions.bed mm10)	Chr	Start	End	Size(End-Start)	Strand	**Peak Score**	**Freq (%)**	Focus Ratio/Region Size	Annotation	Detailed Annotation	Distance to TSS	Nearest Promoter ID	Entrez ID	Nearest Unigene	Nearest Refseq	Nearest Ensembl	**Gene Name**	Gene Alias	Gene Description	CNV Type	Gene Type
1673_T12,T1,T22,T2,T32,T3,T42,T4	chr18	5.9E+07	6E+07	43999	+	**8**	**100**	NA	Intergenic	Intergenic	-4264	NM_175506	240322	Mm.71963	NM_175506	ENSMUSG00000053441	**Adamts19**	4831407I23Rik|AU015154|D230034E10Rik	a disintegrin-like and metallopeptidase (reprolysin type) with thrombospondin type 1 motif, 19	LOSS	protein-coding
1147_T12,T1,T22,T2,T32,T3,T42,T4	chr18	3.2E+07	3E+07	10999	+	**8**	**100**	NA	Intergenic	Intergenic	-34824	NM_153515	225339	Mm.290804	NM_153515	ENSMUSG00000041915	**Ammecr1l**	5430429D03Rik|AU022236|AU040755|AW111353|E230022H04Rik	AMME chromosomal region gene 1-like	LOSS	protein-coding
1220_T12,T1,T22,T2,T32,T3,T42,T4	chr18	3.7E+07	4E+07	43999	+	**8**	**100**	NA	intron (NM_175375, intron 5 of 33)	intron (NM_175375, intron 5 of 33)	25897	NM_175375	108857	Mm.482287	NM_175375	ENSMUSG00000024483	**Ankhd1**	1110004O12Rik|4933432B13Rik|9130019P20Rik|A530027J04Rik|A630021B20Rik|AA571404|Mask|mFLJ00246|mKIAA1085	ankyrin repeat and KH domain containing 1	LOSS	protein-coding
931_T12,T1,T22,T2,T32,T3,T42,T4	chr18	2.1E+07	2E+07	152999	+	**8**	**100**	NA	Intergenic	RLTR30D2_MM|LTR|ERV1	-47596	NM_019737	56386	Mm.393827	NM_019737	ENSMUSG00000056124	**B4galt6**	AA536803|AU022389	UDP-Gal:betaGlcNAc beta 1,4-galactosyltransferase, polypeptide 6	LOSS	protein-coding
1812_T12,T1,T22,T2,T32,T3,T42,T4	chr18	6.8E+07	7E+07	31999	+	**8**	**100**	NA	intron (NM_027556, intron 10 of 45)	intron (NM_027556, intron 10 of 45)	17393	NM_027556	70799	Mm.30857	NM_027556	ENSMUSG00000024542	**Cep192**	4631422C13Rik|D430014P18Rik	centrosomal protein 192	LOSS	protein-coding
1194_T12,T1,T22,T2,T32,T3,T42,T4	chr18	3.5E+07	4E+07	35999	+	**8**	**100**	NA	intron (NM_009818, intron 2 of 17)	intron (NM_009818, intron 2 of 17)	34588	NM_009818	12385	Mm.18962	NM_009818	ENSMUSG00000037815	**Ctnna1**	2010010M04Rik|AA517462|AI988031|Catna1	catenin (cadherin associated protein), alpha 1	LOSS	protein-coding
1923_T12,T1,T22,T2,T32,T3,T42,T4	chr18	7.3E+07	7E+07	37999	+	**8**	**100**	NA	Intergenic	L1Md_F2|LINE|L1	-251431	NM_007831	13176	Mm.167882	NM_007831	ENSMUSG00000060534	**Dcc**	C030036D22Rik|Igdcc1	deleted in colorectal carcinoma	LOSS	protein-coding
2016_T12,T1,T22,T2,T32,T3,T42,T4	chr18	7.8E+07	8E+07	1999	+	**8**	**100**	NA	intron (NM_013831, intron 14 of 14)	intron (NM_013831, intron 14 of 14)	-57967	NM_001195633	1E+08	Mm.297782	NM_001195633	ENSMUSG00000039840	**Epg5**	4732475F16|5430411K18Rik|AI661957|AW456499|mKIAA1632	ectopic P-granules autophagy protein 5 homolog (C. elegans)	LOSS	protein-coding
19_T12,T1,T22,T2,T32,T3,T42,T4	chr10	2.8E+07	3E+07	4999	+	**8**	**100**	NA	Intergenic	L1Md_A|LINE|L1	-205058	NM_008481	16773	Mm.256087	NM_008481	ENSMUSG00000019899	**Lama2**	5830440B04|dy|mKIAA4087|mer|merosin	laminin, alpha 2	LOSS	protein-coding

1799_T12,T1,T22,T2,T32,T3,T42,T4	chr18	6.7E+07	7E+07	120999	+	**8**	**100**	NA	Intergenic	Intergenic	39487	NM_016977	17202	Mm.229447	NM_016977	ENSMUSG00000047259	**Mc4r**	Mc4-r	melanocortin 4 receptor	LOSS	protein-coding
940_T12,T1,T22,T2,T32,T3,T42,T4	chr18	2.1E+07	2E+07	71999	+	**8**	**100**	NA	Intergenic	Intergenic	-12844	NM_008586	17288	Mm.2682	NM_008586	ENSMUSG00000024313	**Mep1b**	Mep-1b	meprin 1 beta	LOSS	protein-coding
2301_T12,T1,T22,T2,T32,T3,T42,T4	chr2	8.1E+07	8E+07	2999	+	**8**	**100**	NA	Intergenic	L1Md_A|LINE|L1	175736	NM_001190179	69482	Mm.29200	NM_027091	ENSMUSG00000026999	**Nup35**	2310006I24Rik|35kDa|5330402E05Rik|MP44|NO44	nucleoporin 35	LOSS	protein-coding
1992_T12,T1,T22,T2,T32,T3,T42,T4	chr18	7.7E+07	8E+07	999	+	**8**	**100**	NA	intron (NM_001164170, intron 1 of 12)	intron (NM_001164170, intron 1 of 12)	13320	NM_001164168	17344	Mm.6370	NM_008602	ENSMUSG00000025423	**Pias2**	6330408K17Rik|AI462206|ARIP3|AU018068|Dib|Miz1|PIASxalpha|PIASxb|PIASxbeta|SIZ2	protein inhibitor of activated STAT 2	LOSS	protein-coding
1994_T12,T1,T22,T2,T32,T3,T42,T4	chr18	7.7E+07	8E+07	6999	+	**8**	**100**	NA	intron (NM_001164170, intron 1 of 12)	intron (NM_001164170, intron 1 of 12)	18320	NM_001164168	17344	Mm.6370	NM_008602	ENSMUSG00000025423	**Pias2**	6330408K17Rik|AI462206|ARIP3|AU018068|Dib|Miz1|PIASxalpha|PIASxb|PIASxbeta|SIZ2	protein inhibitor of activated STAT 2	LOSS	protein-coding
1337_T12,T1,T22,T2,T32,T3,T42,T4	chr18	4.2E+07	4E+07	313999	+	**8**	**100**	NA	Intergenic	Intergenic	4903	NM_138945	18998	Mm.488204	NM_138945	ENSMUSG00000024497	**Pou4f3**	Brn3.1|Brn3c|ddl|dreidel	POU domain, class 4, transcription factor 3	LOSS	protein-coding
744_T12,T1,T22,T2,T32,T3,T42,T4	chr18	1.2E+07	1E+07	9999	+	**8**	**100**	NA	intron (NR_045527, intron 1 of 17)	intron (NR_045527, intron 1 of 17)	3224	NR_045527	225182	Mm.154275	NM_175458	ENSMUSG00000041238	**Rbbp8**	9930104E21Rik|CtIP|RBBP-8|RIM|SAE2	retinoblastoma binding protein 8	LOSS	protein-coding
936_T12,T1,T22,T2,T32,T3,T42,T4	chr18	2.1E+07	2E+07	79999	+	**8**	**100**	NA	intron (NM_026301, intron 1 of 5)	MLTR25A|LTR|ERVK	5875	NM_026301	67664	Mm.45980	NM_026301	ENSMUSG00000033107	**Rnf125**	4930553F04Rik	ring finger protein 125	LOSS	protein-coding
938_T12,T1,T22,T2,T32,T3,T42,T4	chr18	2.1E+07	2E+07	30999	+	**8**	**100**	NA	intron (NM_207623, intron 1 of 6)	MTE2a|LTR|ERVL-MaLR	5043	NM_207623	56515	Mm.253542	NM_019706	ENSMUSG00000024317	**Rnf138**	2410015A17Rik|2810480D20Rik|STRIN|Trif|Trif-d	ring finger protein 138	LOSS	protein-coding
2226_T12,T1,T22,T2,T32,T3,T42,T4	chr18	8.9E+07	9E+07	30999	+	**8**	**100**	NA	Intergenic	Intergenic	-9790	NM_175542	246102	Mm.772	NM_175542	ENSMUSG00000023066	**Rttn**	4921538A15Rik|AI666264|C530033I08Rik|D230040K24	rotatin	LOSS	protein-coding
2203_T12,T1,T22,T2,T32,T3,T42,T4	chr18	8.8E+07	9E+07	44999	+	**8**	**100**	NA	Intergenic	Intergenic	1040207	NM_018821	54607	Mm.91920	NM_018821	ENSMUSG00000056153	**Socs6**	1500012M23Rik|5830401B18Rik|AI447482|Cis4|Cish4|HSPC060|SOCS-4|SOCS-6|SSI4|STAI4|STATI4|Socs4	suppressor of cytokine signaling 6	LOSS	protein-coding

1643_T12,T1,T22,T2,T32,T3,T42,T4	chr18	5.7E+07	6E+07	157999	+	**8**	**100**	NA	Intergenic	B1_Mm|SINE|Alu	40152	NM_026099	67343	Mm.87057	NM_026099	ENSMUSG00000032900	**Tex43**	1700065I17Rik|Tseg7	testis expressed 43	LOSS	protein-coding
2146_T12,T1,T22,T2,T32,T3,T42,T4	chr18	8.5E+07	9E+07	5999	+	**8**	**100**	NA	Intergenic	Intergenic	-422976	NM_025969	67105	Mm.250617	NM_025969	ENSMUSG00000024645	**Timm21**	1700034H14Rik|2700002I20Rik	tranlocase of inner mitochondrial membrane 21	LOSS	protein-coding
933_T12,T1,T22,T2,T32,T3,T42,T4	chr18	2.1E+07	2E+07	19999	+	**8**	**100**	NA	intron (NM_177038, intron 2 of 28)	B1_Mur1|SINE|Alu	14578	NM_177038	75964	Mm.273769	NM_029491	ENSMUSG00000033382	**Trappc8**	5033403J15Rik|AI845423|D030074E01Rik|Trs85|mKIAA1012	trafficking protein particle complex 8	LOSS	protein-coding
1409_T12,T1,T22,T2,T32,T3,T42,T4	chr18	4.6E+07	5E+07	214999	+	**8**	**100**	NA	intron (NM_178872, intron 1 of 9)	B1_Mus2|SINE|Alu	2607	NM_178872	28105	Mm.277382	NM_178872	ENSMUSG00000033949	**Trim36**	D18Wsu100e|haprin	tripartite motif-containing 36	LOSS	protein-coding
30_T12,T1,T2,T32,T4	chr11	7E+07	7E+07	999	+	**8**	**100**	NA	intron (NM_001127233, intron 1 of 10)	intron (NM_001127233, intron 1 of 10)	3641	NM_001127233	22059	Mm.222	NM_011640	ENSMUSG00000059552	**Trp53**	Tp53|bbl|bfy|bhy|p44|p53	transformation related protein 53	LOSS	protein-coding
31_T12,T1,T22,T2,T32,T4	chr11	7E+07	7E+07	4999	+	**8**	**100**	NA	intron (NM_001127233, intron 2 of 10)	intron (NM_001127233, intron 2 of 10)	6641	NM_001127233	22059	Mm.222	NM_011640	ENSMUSG00000059552	**Trp53**	Tp53|bbl|bfy|bhy|p44|p53	transformation related protein 53	LOSS	protein-coding
32_T12,T1,T22,T2,T32	chr11	7E+07	7E+07	999	+	**8**	**100**	NA	intron (NM_001127233, intron 9 of 10)	intron (NM_001127233, intron 9 of 10)	9641	NM_001127233	22059	Mm.222	NM_011640	ENSMUSG00000059552	**Trp53**	Tp53|bbl|bfy|bhy|p44|p53	transformation related protein 53	LOSS	protein-coding
1761_T12,T1,T22,T2,T32,T3,T42,T4	chr18	6.4E+07	6E+07	12999	+	**8**	**100**	NA	Intergenic	RLTR1C|LTR|ERV1	-2641	NM_016792	53382	Mm.19169	NM_016792	ENSMUSG00000024583	**Txnl1**	32kDa|TRP32|Txnl	thioredoxin-like 1	LOSS	protein-coding
1759_T12,T1,T22,T2,T32,T3,T42,T4	chr18	6.4E+07	6E+07	2999	+	**8**	**100**	NA	intron (NM_016792, intron 1 of 7)	B2_Mm1t|SINE|B2	6359	NM_016792	53382	Mm.19169	NM_016792	ENSMUSG00000024583	**Txnl1**	32kDa|TRP32|Txnl	thioredoxin-like 1	LOSS	protein-coding
1756_T12,T1,T22,T2,T32,T3,T42,T4	chr18	6.4E+07	6E+07	14999	+	**8**	**100**	NA	intron (NM_016792, intron 6 of 7)	intron (NM_016792, intron 6 of 7)	20359	NM_016792	53382	Mm.19169	NM_016792	ENSMUSG00000024583	**Txnl1**	32kDa|TRP32|Txnl	thioredoxin-like 1	LOSS	protein-coding
322_T12,T1,T22,T2,T32,T3,T42,T4	chr14	3.5E+07	3E+07	18999	+	**8**	**100**	NA	intron (NM_001004436, intron 4 of 18)	B2_Mm2|SINE|B2	44072	NM_001301330	218914	Mm.27957	NM_001004436	ENSMUSG00000041408	**Wapl**	A530089A20Rik|BC037674|DIF-2|FOE|Wapal	WAPL cohesin release factor	LOSS	protein-coding

1763_T12,T1,T22,T2,T32,T3,T42,T4	chr18	6.4E+07	6E+07	153999	+	**8**	**100**	NA	intron (NM_001014981, intron 17 of 27)	intron (NM_001014981, intron 17 of 27)	83805	NM_001014981	104082	Mm.30850	NM_001014981	ENSMUSG00000040560	**Wdr7**	AI462727|TRAG|mKIAA0541	WD repeat domain 7	LOSS	protein-coding
1376_T12,T1,T22,T2,T32,T3,T42,T4	chr18	4.5E+07	4E+07	72999	+	**8**	**100**	NA	exon (NM_001163013, exon 23 of 30)	exon (NM_001163013, exon 23 of 30)	36335	NM_001163013	240255	Mm.244482	NM_001163013	ENSMUSG00000034653	**Ythdc2**	3010002F02Rik|BC037178	YTH domain containing 2	LOSS	protein-coding
1806_T12,T1,T22,T2,T32,T3,T42	chr18	6.7E+07	7E+07	715999	+	**7**	**87.5**	NA	intron (NM_027130, intron 10 of 16)	intron (NM_027130, intron 10 of 16)	26636	NM_027130	69597	Mm.426052	NM_027130	ENSMUSG00000024527	**Afg3l2**	2310036I02Rik|AW260507|Emv66|par	AFG3-like AAA ATPase 2	LOSS	protein-coding
248_T1,T22,T2,T32,T3,T42,T4	chr14	2.7E+07	3E+07	337999	+	**7**	**87.5**	NA	intron (NM_145221, intron 16 of 21)	intron (NM_145221, intron 16 of 21)	34896	NM_080856	142687	Mm.138259	NM_080856	ENSMUSG00000021898	**Asb14**	Asb-14	ankyrin repeat and SOCS box-containing 14	LOSS	protein-coding
958_T12,T1,T22,T2,T32,T3,T42	chr18	2.2E+07	2E+07	75999	+	**7**	**87.5**	NA	Intergenic	Intergenic	-2589	NM_001167777	211961	Mm.392310	NM_001167777	ENSMUSG00000045215	**Asxl3**	C230079D11Rik|D430002O22Rik|D930044O18Rik|Gm329|Gm945	additional sex combs like 3 (Drosophila)	LOSS	protein-coding
1423_T12,T1,T22,T2,T32,T3,T42	chr18	4.7E+07	5E+07	23999	+	**7**	**87.5**	NA	intron (NM_026217, intron 2 of 3)	(GA)n|Simple_repeat|Simple_repeat	6079	NM_026217	67526	Mm.9852	NM_026217	ENSMUSG00000032905	**Atg12**	4931423H11Rik|A330058M13Rik|Apg12l|Atg12l	autophagy related 12	LOSS	protein-coding
152_T1,T22,T2,T32,T3,T42,T4	chr14	1.4E+07	1E+07	5999	+	**7**	**87.5**	NA	Intergenic	Intergenic	-1991	NM_139227	246103	Mm.133625	NM_139227	ENSMUSG00000021738	**Atxn7**	A430107N12Rik|AI627028|Sca7|ataxin-7	ataxin 7	LOSS	protein-coding
1410_T12,T1,T22,T2,T32,T3,T42	chr18	4.6E+07	5E+07	120999	+	**7**	**87.5**	NA	Intergenic	Intergenic	-66072	NM_001160399	240261	Mm.329416	NM_001160399	ENSMUSG00000071855	**Ccdc112**	8430438M01Rik|AW108467	coiled-coil domain containing 112	LOSS	protein-coding
1842_T12,T1,T22,T2,T32,T3,T42	chr18	7E+07	7E+07	3999	+	**7**	**87.5**	NA	promoter-TSS (NM_201362)	promoter-TSS (NM_201362)	-59	NM_201362	381175	Mm.266831	NM_201362	ENSMUSG00000038903	**Ccdc68**	BC046343	coiled-coil domain containing 68	LOSS	protein-coding
2229_T12,T1,T22,T32,T3,T42,T4	chr18	8.9E+07	9E+07	33999	+	**7**	**87.5**	NA	intron (NM_175542, intron 45 of 48)	intron (NM_175542, intron 45 of 48)	-76927	NM_178687	225825	Mm.139293	NM_178687	ENSMUSG00000034028	**Cd226**	BC051526|DNAM-1|DNAM1|Pta1|TLiSA1	CD226 antigen	LOSS	protein-coding
1596_T12,T1,T22,T2,T32,T3,T42	chr18	5.4E+07	5E+07	16999	+	**7**	**87.5**	NA	Intergenic	L1MA4A|LINE|L1	-42453	NM_178686	225523	Mm.193678	NM_178686	ENSMUSG00000048799	**Cep120**	A230075C01|AU016693|Ccdc100	centrosomal protein 120	LOSS	protein-coding
1811_T12,T1,T22,T2,T32,T3,T42	chr18	6.8E+07	7E+07	999	+	**7**	**87.5**	NA	intron (NM_027556, intron 2 of 45)	intron (NM_027556, intron 2 of 45)	893	NM_027556	70799	Mm.30857	NM_027556	ENSMUSG00000024542	**Cep192**	4631422C13Rik|D430014P18Rik	centrosomal protein 192	LOSS	protein-coding

1193_T12,T1,T22,T2,T32,T3,T4	chr18	3.5E+07	4E+07	18999	+	**7**	**87.5**	NA	intron (NM_009818, intron 1 of 17)	(TG)n|Simple_repeat|Simple_repeat	7088	NM_009818	12385	Mm.18962	NM_009818	ENSMUSG00000037815	**Ctnna1**	2010010M04Rik|AA517462|AI988031|Catna1	catenin (cadherin associated protein), alpha 1	LOSS	protein-coding
1924_T12,T1,T22,T2,T32,T42,T4	chr18	7.3E+07	7E+07	999	+	**7**	**87.5**	NA	Intergenic	Intergenic	-270931	NM_007831	13176	Mm.167882	NM_007831	ENSMUSG00000060534	**Dcc**	C030036D22Rik|Igdcc1	deleted in colorectal carcinoma	LOSS	protein-coding
1901_T12,T1,T22,T2,T32,T3,T42	chr18	7.2E+07	7E+07	52999	+	**7**	**87.5**	NA	intron (NM_007831, intron 5 of 28)	intron (NM_007831, intron 5 of 28)	646069	NM_007831	13176	Mm.167882	NM_007831	ENSMUSG00000060534	**Dcc**	C030036D22Rik|Igdcc1	deleted in colorectal carcinoma	LOSS	protein-coding
911_T12,T1,T22,T2,T32,T42,T4	chr18	2E+07	2E+07	12999	+	**7**	**87.5**	NA	Intergenic	Lx3C|LINE|L1	396097	NM_007882	13507	Mm.89935	NM_007882	ENSMUSG00000059898	**Dsc3**	5430426I24Rik	desmocollin 3	LOSS	protein-coding
1224_T12,T1,T22,T2,T32,T3,T4	chr18	3.7E+07	4E+07	18999	+	**7**	**87.5**	NA	intron (NM_175375, intron 14 of 33)	intron (NM_175375, intron 14 of 33)	-43060	NM_201256	108112	Mm.24790	NM_201256	ENSMUSG00000090264	**Eif4ebp3**	4e-bp3	eukaryotic translation initiation factor 4E binding protein 3	LOSS	protein-coding
2015_T12,T22,T2,T32,T3,T42,T4	chr18	7.8E+07	8E+07	999	+	**7**	**87.5**	NA	intron (NM_013831, intron 13 of 14)	intron (NM_013831, intron 13 of 14)	-59467	NM_001195633	1E+08	Mm.297782	NM_001195633	ENSMUSG00000039840	**Epg5**	4732475F16|5430411K18Rik|AI661957|AW456499|mKIAA1632	ectopic P-granules autophagy protein 5 homolog (C. elegans)	LOSS	protein-coding
722_T12,T1,T22,T2,T32,T3,T42	chr18	1E+07	1E+07	625999	+	**7**	**87.5**	NA	intron (NM_001083628, intron 27 of 32)	L1M4|LINE|L1	60852	NM_001081222	77805	Mm.210996	NM_144542	ENSMUSG00000024293	**Esco1**	A930014I12Rik	establishment of sister chromatid cohesion N-acetyltransferase 1	LOSS	protein-coding
1657_T12,T1,T22,T2,T32,T3,T42	chr18	5.8E+07	6E+07	370999	+	**7**	**87.5**	NA	intron (NM_010181, intron 31 of 64)	intron (NM_010181, intron 31 of 64)	141926	NM_010181	14119	Mm.20271	NM_010181	ENSMUSG00000024598	**Fbn2**	BC063774|Fib-2|mKIAA4226|sne|sy	fibrillin 2	LOSS	protein-coding
1413_T12,T1,T22,T2,T32,T3,T42	chr18	4.7E+07	5E+07	23999	+	**7**	**87.5**	NA	intron (NM_173423, intron 2 of 2)	RMER4B|LTR|ERVK	3471	NM_173423	240263	Mm.38249	NM_173423	ENSMUSG00000033319	**Fem1c**	2610312A07Rik|3632443A22Rik|AI646567	fem-1 homolog c (C.elegans)	LOSS	protein-coding
1804_T12,T1,T22,T2,T32,T3,T42	chr18	6.7E+07	7E+07	16999	+	**7**	**87.5**	NA	Intergenic	Intergenic	-33298	NM_177137	14680	Mm.440746	NM_010307	ENSMUSG00000024524	**Gnal**	2610011C15Rik|9630020G10Rik|Galphaolf|Gna10|Golf	guanine nucleotide binding protein, alpha stimulating, olfactory type	LOSS	protein-coding
1338_T12,T1,T22,T2,T32,T3,T4	chr18	4.3E+07	4E+07	36999	+	**7**	**87.5**	NA	non-coding (NR_110344, exon 23 of 23)	non-coding (NR_110344, exon 23 of 23)	4652	NM_181543	240239	Mm.186779	NM_181543	ENSMUSG00000042816	**Gpr151**	C130082O03Rik|GalRL|PGR7|nGPCR-2037	G protein-coupled receptor 151	LOSS	protein-coding
1989_T12,T1,T22,T2,T32,T3,T42	chr18	7.7E+07	8E+07	221999	+	**7**	**87.5**	NA	promoter-TSS (NM_001039202)	promoter-TSS (NM_001039202)	-615	NM_029826	76987	Mm.236127	NM_029826	ENSMUSG00000025421	**Hdhd2**	0610039H12Rik|3110052N05Rik	haloacid dehalogenase-like hydrolase domain containing 2	LOSS	protein-coding

552_T12,T22,T2,T32,T3,T42,T4	chr14	5.2E+07	5E+07	14999	+	**7**	**87.5**	NA	intron (NM_001170983, intron 1 of 8)	intron (NM_001170983, intron 1 of 8)	1028	NM_001170982	15381	Mm.427321	NM_016884	ENSMUSG00000060373	**Hnrnpc**	AL022939|D14Wsu171e|Hnrpc|Hnrpc1|Hnrpc2|hnRNPC1|hnRNPC2|hnrnp-C	heterogeneous nuclear ribonucleoprotein C	LOSS	protein-coding
1668_T12,T1,T22,T2,T32,T3,T42	chr18	5.9E+07	6E+07	121999	+	**7**	**87.5**	NA	Intergenic	Intergenic	79018	NM_025478	66307	Mm.182574	NM_025478	ENSMUSG00000024601	**Isoc1**	2610034N03Rik	isochorismatase domain containing 1	LOSS	protein-coding
1260_T12,T1,T22,T2,T32,T3,T42	chr18	4E+07	4E+07	3999	+	**7**	**87.5**	NA	intron (NM_026135, intron 1 of 1)	intron (NM_026135, intron 1 of 1)	10139	NM_026135	383348	Mm.76366	NM_026135	ENSMUSG00000051401	**Kctd16**	4930434H12Rik|Gm1267	potassium channel tetramerisation domain containing 16	LOSS	protein-coding
1262_T12,T1,T22,T2,T32,T3,T42	chr18	4E+07	4E+07	95999	+	**7**	**87.5**	NA	intron (NM_026135, intron 1 of 1)	intron (NM_026135, intron 1 of 1)	66139	NM_026135	383348	Mm.76366	NM_026135	ENSMUSG00000051401	**Kctd16**	4930434H12Rik|Gm1267	potassium channel tetramerisation domain containing 16	LOSS	protein-coding
1264_T12,T1,T22,T2,T32,T3,T42	chr18	4E+07	4E+07	41999	+	**7**	**87.5**	NA	intron (NM_026135, intron 1 of 1)	intron (NM_026135, intron 1 of 1)	140139	NM_026135	383348	Mm.76366	NM_026135	ENSMUSG00000051401	**Kctd16**	4930434H12Rik|Gm1267	potassium channel tetramerisation domain containing 16	LOSS	protein-coding
1814_T12,T1,T22,T2,T32,T3,T42	chr18	6.8E+07	7E+07	148999	+	**7**	**87.5**	NA	Intergenic	Intergenic	-24257	NM_172631	52662	Mm.440183	NM_172631	ENSMUSG00000024544	**Ldlrad4**	8230401C20Rik|A430083H02|A430108L08Rik|C18orf1|D18Ertd653e|D330030L18Rik	low density lipoprotein receptor class A domain containing 4	LOSS	protein-coding
1816_T12,T1,T22,T2,T32,T3,T42	chr18	6.8E+07	7E+07	87999	+	**7**	**87.5**	NA	intron (NM_172631, intron 1 of 5)	intron (NM_172631, intron 1 of 5)	97243	NM_172631	52662	Mm.440183	NM_172631	ENSMUSG00000024544	**Ldlrad4**	8230401C20Rik|A430083H02|A430108L08Rik|C18orf1|D18Ertd653e|D330030L18Rik	low density lipoprotein receptor class A domain containing 4	LOSS	protein-coding
1195_T12,T1,T22,T2,T32,T3,T4	chr18	3.5E+07	4E+07	3999	+	**7**	**87.5**	NA	intron (NM_009818, intron 3 of 17)	B4|SINE|B4	41524	NM_178005	107065	Mm.39900	NM_178005	ENSMUSG00000071862	**Lrrtm2**	AI851755|BB129880|C630011A14Rik|mKIAA0416	leucine rich repeat transmembrane neuronal 2	LOSS	protein-coding
1777_T12,T1,T22,T2,T32,T3,T4	chr18	6.5E+07	7E+07	150999	+	**7**	**87.5**	NA	intron (NR_105742, intron 3 of 6)	ORR1D1|LTR|ERVL-MaLR	50003	NM_172833	240354	Mm.132613	NM_172833	ENSMUSG00000032688	**Malt1**	A630046N12|D430033E09Rik|Pcasp1	MALT1 paracaspase	LOSS	protein-coding
1798_T12,T1,T22,T2,T32,T3,T42	chr18	6.7E+07	7E+07	3999	+	**7**	**87.5**	NA	Intergenic	L1MDa|LINE|L1	101987	NM_016977	17202	Mm.229447	NM_016977	ENSMUSG00000047259	**Mc4r**	Mc4-r	melanocortin 4 receptor	LOSS	protein-coding
941_T12,T1,T22,T2,T32,T3,T42	chr18	2.1E+07	2E+07	71999	+	**7**	**87.5**	NA	intron (NM_001033445, intron 4 of 5)	intron (NM_001033445, intron 4 of 5)	59156	NM_008586	17288	Mm.2682	NM_008586	ENSMUSG00000024313	**Mep1b**	Mep-1b	meprin 1 beta	LOSS	protein-coding
548_T12,T22,T2,T32,T3,T42,T4	chr14	5.2E+07	5E+07	7999	+	**7**	**87.5**	NA	Intergenic	PB1D9|SINE|Alu	-4342	NM_001029990	52535	Mm.5750	NM_001029990	ENSMUSG00000004561	**Mettl17**	2310032K15Rik|D14Ertd209e|Mettl1d1	methyltransferase like 17	LOSS	protein-coding

971_T12,T1,T22,T2,T32,T3,T42	chr18	2.3E+07	2E+07	19999	+	**7**	**87.5**	NA	intron (NM_199024, intron 1 of 9)	B3A|SINE|B2	59163	NM_001161483	319211	Mm.209896	NM_199024	ENSMUSG00000041923	**Nol4**	1700013J13Rik|4930568N03Rik|Gm1262	nucleolar protein 4	LOSS	protein-coding
2053_T12,T1,T22,T2,T32,T3,T42	chr18	8E+07	8E+07	57999	+	**7**	**87.5**	NA	Intergenic	Intergenic	-372395	NM_053117	93737	Mm.24678	NM_053117	ENSMUSG00000056214	**Pard6g**	2410049N21Rik|Par6a	par-6 family cell polarity regulator gamma	LOSS	protein-coding
1998_T12,T1,T2,T32,T3,T42,T4	chr18	7.7E+07	8E+07	37999	+	**7**	**87.5**	NA	intron (NM_001164167, intron 3 of 13)	Lx|LINE|L1	47820	NM_001164168	17344	Mm.6370	NM_008602	ENSMUSG00000025423	**Pias2**	6330408K17Rik|AI462206|ARIP3|AU018068|Dib|Miz1|PIASxalpha|PIASxb|PIASxbeta|SIZ2	protein inhibitor of activated STAT 2	LOSS	protein-coding
1991_T12,T1,T22,T2,T32,T3,T42	chr18	7.7E+07	8E+07	999	+	**7**	**87.5**	NA	intron (NM_001164170, intron 1 of 12)	intron (NM_001164170, intron 1 of 12)	12320	NM_001164168	17344	Mm.6370	NM_008602	ENSMUSG00000025423	**Pias2**	6330408K17Rik|AI462206|ARIP3|AU018068|Dib|Miz1|PIASxalpha|PIASxb|PIASxbeta|SIZ2	protein inhibitor of activated STAT 2	LOSS	protein-coding
1790_T1,T22,T2,T32,T3,T42,T4	chr18	6.6E+07	7E+07	999	+	**7**	**87.5**	NA	Intergenic	Intergenic	-14604	NM_021451	58801	Mm.271878	NM_021451	ENSMUSG00000024521	**Pmaip1**	Noxa	phorbol-12-myristate-13-acetate-induced protein 1	LOSS	protein-coding
1582_T12,T1,T22,T2,T32,T3,T42	chr18	5.3E+07	5E+07	46999	+	**7**	**87.5**	NA	Intergenic	Intergenic	-8993	NM_008908	19038	Mm.4587	NM_008908	ENSMUSG00000024538	**Ppic**	CyP-20c	peptidylprolyl isomerase C	LOSS	protein-coding
1584_T12,T1,T22,T2,T32,T3,T42	chr18	5.3E+07	5E+07	108999	+	**7**	**87.5**	NA	intron (NM_001033281, intron 2 of 6)	B1_Mur1|SINE|Alu	41454	NM_001033281	225518	Mm.297645	NM_001033281	ENSMUSG00000069378	**Prdm6**	Gm92|PRISM	PR domain containing 6	LOSS	protein-coding
746_T12,T1,T22,T2,T32,T3,T4	chr18	1.2E+07	1E+07	162999	+	**7**	**87.5**	NA	intron (NR_045526, intron 13 of 17)	L1Md_F2|LINE|L1	72651	NM_001081223	225182	Mm.154275	NM_175458	ENSMUSG00000041238	**Rbbp8**	9930104E21Rik|CtIP|RBBP-8|RIM|SAE2	retinoblastoma binding protein 8	LOSS	protein-coding
745_T12,T1,T22,T2,T32,T3,T4	chr18	1.2E+07	1E+07	999	+	**7**	**87.5**	NA	intron (NR_045527, intron 1 of 17)	intron (NR_045527, intron 1 of 17)	8724	NR_045527	225182	Mm.154275	NM_175458	ENSMUSG00000041238	**Rbbp8**	9930104E21Rik|CtIP|RBBP-8|RIM|SAE2	retinoblastoma binding protein 8	LOSS	protein-coding
937_T12,T1,T22,T32,T3,T42,T4	chr18	2.1E+07	2E+07	999	+	**7**	**87.5**	NA	Intergenic	MTEa|LTR|ERVL-MaLR	-10300	NM_019706	56515	Mm.253542	NM_019706	ENSMUSG00000024317	**Rnf138**	2410015A17Rik|2810480D20Rik|STRIN|Trif|Trif-d	ring finger protein 138	LOSS	protein-coding
939_T12,T1,T22,T32,T3,T42,T4	chr18	2.1E+07	2E+07	999	+	**7**	**87.5**	NA	intron (NM_001303005, intron 6 of 6)	B2_Mm2|SINE|B2	21043	NM_207623	56515	Mm.253542	NM_019706	ENSMUSG00000024317	**Rnf138**	2410015A17Rik|2810480D20Rik|STRIN|Trif|Trif-d	ring finger protein 138	LOSS	protein-coding
2011_T12,T1,T2,T32,T3,T42,T4	chr18	7.8E+07	8E+07	5999	+	**7**	**87.5**	NA	Intergenic	Intergenic	-65364	NM_001164504	225743	Mm.23293	NM_001164504	ENSMUSG00000025427	**Rnf165**	2900024M11Rik|AI427432|Akd2|Ark2c|G630064H08Rik|Gm96	ring finger protein 165	LOSS	protein-coding
2047_T12,T1,T22,T2,T32,T42,T4	chr18	7.9E+07	8E+07	3999	+	**7**	**87.5**	NA	Intergenic	L1M4b|LINE|L1	-290109	NM_053099	240427	Mm.312871	NM_053099	ENSMUSG00000024548	**Setbp1**	C130092E12|Seb|mKIAA0437	SET binding protein 1	LOSS	protein-coding

1649_T12,T1,T22,T2,T32,T3,T42	chr18	5.8E+07	6E+07	10999	+	**7**	**87.5**	NA	Intergenic	Intergenic	-58678	NM_009194	20496	Mm.399997	NM_009194	ENSMUSG00000024597	**Slc12a2**	9330166H04Rik|Nkcc1|mBSC2|sy-ns	solute carrier family 12, member 2	LOSS	protein-coding
1651_T12,T1,T22,T2,T32,T3,T42	chr18	5.8E+07	6E+07	999	+	**7**	**87.5**	NA	Intergenic	Intergenic	-51678	NM_009194	20496	Mm.399997	NM_009194	ENSMUSG00000024597	**Slc12a2**	9330166H04Rik|Nkcc1|mBSC2|sy-ns	solute carrier family 12, member 2	LOSS	protein-coding
1653_T12,T1,T22,T2,T32,T3,T42	chr18	5.8E+07	6E+07	44999	+	**7**	**87.5**	NA	Intergenic	Intergenic	-27678	NM_009194	20496	Mm.399997	NM_009194	ENSMUSG00000024597	**Slc12a2**	9330166H04Rik|Nkcc1|mBSC2|sy-ns	solute carrier family 12, member 2	LOSS	protein-coding
1655_T12,T1,T22,T2,T32,T3,T42	chr18	5.8E+07	6E+07	6999	+	**7**	**87.5**	NA	promoter-TSS (NM_009194)	promoter-TSS (NM_009194)	-678	NM_009194	20496	Mm.399997	NM_009194	ENSMUSG00000024597	**Slc12a2**	9330166H04Rik|Nkcc1|mBSC2|sy-ns	solute carrier family 12, member 2	LOSS	protein-coding
1666_T12,T1,T22,T2,T32,T3,T42	chr18	5.8E+07	6E+07	301999	+	**7**	**87.5**	NA	Intergenic	Intergenic	-32740	NM_001081072	225579	Mm.258517	NM_001081072	ENSMUSG00000024600	**Slc27a6**	4732438L20Rik|FACVL2|FATP6|VLCS-H1	solute carrier family 27 (fatty acid transporter), member 6	LOSS	protein-coding
554_T12,T22,T2,T32,T3,T42,T4	chr14	5.2E+07	5E+07	219999	+	**7**	**87.5**	NA	intron (NM_201637, intron 10 of 36)	L1MC5|LINE|L1	-11616	NR_028542	1E+08		NR_028542	ENSMUSG00000093044	**Snord8**	MBII-28	small nucleolar RNA, C/D box 8	LOSS	snoRNA
1571_T12,T1,T22,T2,T32,T3,T42	chr18	5.3E+07	5E+07	95999	+	**7**	**87.5**	NA	Intergenic	B1_Mur4|SINE|Alu	-62865	NM_026386	67804	Mm.252171	NM_026386	ENSMUSG00000034484	**Snx2**	0610030A03Rik	sorting nexin 2	LOSS	protein-coding
1573_T12,T1,T22,T2,T32,T3,T42	chr18	5.3E+07	5E+07	4999	+	**7**	**87.5**	NA	Intergenic	Intergenic	-11365	NM_026386	67804	Mm.252171	NM_026386	ENSMUSG00000034484	**Snx2**	0610030A03Rik	sorting nexin 2	LOSS	protein-coding
1575_T12,T1,T22,T2,T32,T3,T42	chr18	5.3E+07	5E+07	11999	+	**7**	**87.5**	NA	Intergenic	Intergenic	-1865	NM_026386	67804	Mm.252171	NM_026386	ENSMUSG00000034484	**Snx2**	0610030A03Rik	sorting nexin 2	LOSS	protein-coding
1578_T12,T1,T22,T2,T32,T3,T42	chr18	5.3E+07	5E+07	67999	+	**7**	**87.5**	NA	intron (NM_026386, intron 14 of 14)	intron (NM_026386, intron 14 of 14)	-27162	NM_029394	69226	Mm.32414	NM_029394	ENSMUSG00000024535	**Snx24**	2810011K15Rik|5730433I16Rik|AI451341	sorting nexing 24	LOSS	protein-coding
1580_T12,T1,T22,T2,T32,T3,T42	chr18	5.3E+07	5E+07	7999	+	**7**	**87.5**	NA	intron (NM_029394, intron 1 of 6)	intron (NM_029394, intron 1 of 6)	11838	NM_029394	69226	Mm.32414	NM_029394	ENSMUSG00000024535	**Snx24**	2810011K15Rik|5730433I16Rik|AI451341	sorting nexing 24	LOSS	protein-coding
2202_T12,T1,T22,T2,T32,T3,T4	chr18	8.8E+07	9E+07	5999	+	**7**	**87.5**	NA	Intergenic	Intergenic	1065707	NM_018821	54607	Mm.91920	NM_018821	ENSMUSG00000056153	**Socs6**	1500012M23Rik|5830401B18Rik|AI447482|Cis4|Cish4|HSPC060|SOCS-4|SOCS-6|SSI4|STAI4|STATI4|Socs4	suppressor of cytokine signaling 6	LOSS	protein-coding

2204_T12,T1,T22,T2,T32,T42,T4	chr18	8.8E+07	9E+07	999	+	**7**	**87.5**	NA	Intergenic	Intergenic	1017207	NM_018821	54607	Mm.91920	NM_018821	ENSMUSG00000056153	**Socs6**	1500012M23Rik|5830401B18Rik|AI447482|Cis4|Cish4|HSPC060|SOCS-4|SOCS-6|SSI4|STAI4|STATI4|Socs4	suppressor of cytokine signaling 6	LOSS	protein-coding
307_T12,T22,T2,T32,T3,T42,T4	chr14	3.4E+07	3E+07	66999	+	**7**	**87.5**	NA	intron (NM_029389, intron 4 of 10)	intron (NM_029389, intron 4 of 10)	43954	NM_181529	319508	Mm.228006	NM_176931	ENSMUSG00000041479	**Syt15**	CHR10SYT|E230025K04Rik|sytXV	synaptotagmin XV	LOSS	protein-coding
1415_T12,T1,T22,T2,T32,T3,T42	chr18	4.7E+07	5E+07	58999	+	**7**	**87.5**	NA	intron (NM_173394, intron 2 of 2)	intron (NM_173394, intron 2 of 2)	9533	NM_173394	225471	Mm.149280	NM_173394	ENSMUSG00000056130	**Ticam2**	B430113A10|TICAM-2|TRAM|Tirp|Trif	toll-like receptor adaptor molecule 2	LOSS	protein-coding
2287_T12,T1,T22,T2,T32,T3,T42	chr18	9E+07	9E+07	122999	+	**7**	**87.5**	NA	3' UTR (NM_198295, exon 16 of 16)	3' UTR (NM_198295, exon 16 of 16)	30846	NM_198295	67988	Mm.268041	NM_198295	ENSMUSG00000024614	**Tmx3**	6430411B10Rik|A730024F05Rik|AV259382|Txndc10|mKIAA1830	thioredoxin-related transmembrane protein 3	LOSS	protein-coding
932_T12,T1,T22,T2,T32,T3,T4	chr18	2.1E+07	2E+07	999	+	**7**	**87.5**	NA	intron (NM_177038, intron 4 of 28)	RSINE1|SINE|B4	25078	NM_177038	75964	Mm.273769	NM_029491	ENSMUSG00000033382	**Trappc8**	5033403J15Rik|AI845423|D030074E01Rik|Trs85|mKIAA1012	trafficking protein particle complex 8	LOSS	protein-coding
934_T12,T1,T22,T2,T32,T3,T4	chr18	2.1E+07	2E+07	9999	+	**7**	**87.5**	NA	promoter-TSS (NM_029491)	promoter-TSS (NM_029491)	-422	NM_029491	75964	Mm.273769	NM_029491	ENSMUSG00000033382	**Trappc8**	5033403J15Rik|AI845423|D030074E01Rik|Trs85|mKIAA1012	trafficking protein particle complex 8	LOSS	protein-coding
930_T12,T1,T22,T2,T32,T3,T42	chr18	2.1E+07	2E+07	188999	+	**7**	**87.5**	NA	Intergenic	L1M3e|LINE|L1	-42250	NM_013697	22139	Mm.2108	NM_013697	ENSMUSG00000061808	**Ttr**	AA408768|AI787086|D17860|prealbumin	transthyretin	LOSS	protein-coding
1755_T12,T1,T22,T2,T32,T3,T42	chr18	6.4E+07	6E+07	999	+	**7**	**87.5**	NA	3' UTR (NM_016792, exon 8 of 8)	3' UTR (NM_016792, exon 8 of 8)	28359	NM_016792	53382	Mm.19169	NM_016792	ENSMUSG00000024583	**Txnl1**	32kDa|TRP32|Txnl	thioredoxin-like 1	LOSS	protein-coding
1758_T12,T1,T22,T2,T32,T3,T4	chr18	6.4E+07	6E+07	3999	+	**7**	**87.5**	NA	intron (NM_016792, intron 1 of 7)	intron (NM_016792, intron 1 of 7)	9859	NM_016792	53382	Mm.19169	NM_016792	ENSMUSG00000024583	**Txnl1**	32kDa|TRP32|Txnl	thioredoxin-like 1	LOSS	protein-coding
720_T12,T1,T22,T2,T32,T3,T42	chr18	9952501	1E+07	277999	+	**7**	**87.5**	NA	intron (NM_009071, intron 22 of 32)	intron (NM_009071, intron 22 of 32)	-61351	NM_001038589	59025	Mm.329277	NM_021522	ENSMUSG00000047879	**Usp14**	2610005K12Rik|2610037B11Rik|AW107924|C78769|ax|nmf375	ubiquitin specific peptidase 14	LOSS	protein-coding
684_T12,T1,T22,T2,T32,T3,T4	chr18	7852501	8E+06	3999	+	**7**	**87.5**	NA	Intergenic	B2_Mm2|SINE|B2	-14332	NM_001282093	225131	Mm.204524	NM_153085	ENSMUSG00000024283	**Wac**	1110067P07Rik|A230035H12Rik|AI256735|AI256776|Wwp4	WW domain containing adaptor with coiled-coil	LOSS	protein-coding

687_T12,T1,T22,T2,T32,T3,T4	chr18	7949501	8E+06	61999	+	**7**	**87.5**	NA	Intergenic	Intergenic	111303	NM_153085	225131	Mm.204524	NM_153085	ENSMUSG00000024283	**Wac**	1110067P07Rik|A230035H12Rik|AI256735|AI256776|Wwp4	WW domain containing adaptor with coiled-coil	LOSS	protein-coding
321_T12,T22,T2,T32,T3,T42,T4	chr14	3.5E+07	3E+07	23999	+	**7**	**87.5**	NA	intron (NM_001004436, intron 2 of 18)	B1_Mm|SINE|Alu	15572	NM_001301330	218914	Mm.27957	NM_001004436	ENSMUSG00000041408	**Wapl**	A530089A20Rik|BC037674|DIF-2|FOE|Wapal	WAPL cohesin release factor	LOSS	protein-coding
1148_T12,T1,T22,T2,T32,T3,T42	chr18	3.2E+07	3E+07	208999	+	**7**	**87.5**	NA	3' UTR (NM_001170966, exon 6 of 6)	3' UTR (NM_001170966, exon 6 of 6)	30943	NM_001170966	74320	Mm.277705	NM_028866	ENSMUSG00000024400	**Wdr33**	1110001N06Rik|2310011G05Rik|2810021O11Rik|8430413N20Rik|WDC146	WD repeat domain 33	LOSS	protein-coding
1764_T12,T1,T2,T32,T3,T42,T4	chr18	6.4E+07	6E+07	40999	+	**7**	**87.5**	NA	intron (NM_001014981, intron 21 of 27)	B3A|SINE|B2	181305	NM_001014981	104082	Mm.30850	NM_001014981	ENSMUSG00000040560	**Wdr7**	AI462727|TRAG|mKIAA0541	WD repeat domain 7	LOSS	protein-coding
1762_T12,T22,T2,T32,T3,T42,T4	chr18	6.4E+07	6E+07	13999	+	**7**	**87.5**	NA	promoter-TSS (NM_001014981)	promoter-TSS (NM_001014981)	-195	NM_001014981	104082	Mm.30850	NM_001014981	ENSMUSG00000040560	**Wdr7**	AI462727|TRAG|mKIAA0541	WD repeat domain 7	LOSS	protein-coding
265_T1,T22,T2,T32,T3,T42,T4	chr14	2.9E+07	3E+07	7999	+	**7**	**87.5**	NA	intron (NM_001256224, intron 3 of 4)	intron (NM_001256224, intron 3 of 4)	4095	NM_001256224	22418	Mm.287544	NM_009524	ENSMUSG00000021994	**Wnt5a**	8030457G12Rik|Wnt-5a	wingless-type MMTV integration site family, member 5A	LOSS	protein-coding
1258_T12,T1,T22,T2,T32,T3,T42	chr18	4E+07	4E+07	89999	+	**7**	**87.5**	NA	intron (NM_023311, intron 2 of 5)	Charlie1a|DNA|hAT-Charlie	3899	NM_023311	67180	Mm.270382	NM_023311	ENSMUSG00000024487	**Yipf5**	2610311I19Rik|AA408236|Yip1a	Yip1 domain family, member 5	LOSS	protein-coding
1377_T12,T1,T22,T2,T32,T3,T42	chr18	4.5E+07	4E+07	3999	+	**7**	**87.5**	NA	Intergenic	L1_Mur2|LINE|L1	74835	NM_001163013	240255	Mm.244482	NM_001163013	ENSMUSG00000034653	**Ythdc2**	3010002F02Rik|BC037178	YTH domain containing 2	LOSS	protein-coding
1379_T12,T1,T22,T2,T32,T3,T42	chr18	4.5E+07	4E+07	73999	+	**7**	**87.5**	NA	Intergenic	CT-rich|Low_complexity|Low_complexity	114835	NM_001163013	240255	Mm.244482	NM_001163013	ENSMUSG00000034653	**Ythdc2**	3010002F02Rik|BC037178	YTH domain containing 2	LOSS	protein-coding
1382_T12,T1,T22,T2,T32,T3,T4	chr18	4.5E+07	5E+07	28999	+	**7**	**87.5**	NA	Intergenic	Intergenic	173335	NM_001163013	240255	Mm.244482	NM_001163013	ENSMUSG00000034653	**Ythdc2**	3010002F02Rik|BC037178	YTH domain containing 2	LOSS	protein-coding
1669_T12,T1,T22,T32,T3,T42	chr18	5.9E+07	6E+07	4999	+	**6**	**75**	NA	Intergenic	Intergenic	-34764	NM_175506	240322	Mm.71963	NM_175506	ENSMUSG00000053441	**Adamts19**	4831407I23Rik|AU015154|D230034E10Rik	a disintegrin-like and metallopeptidase (reprolysin type) with thrombospondin type 1 motif, 19	LOSS	protein-coding
1676_T12,T1,T22,T2,T32,T3	chr18	5.9E+07	6E+07	56999	+	**6**	**75**	NA	intron (NM_175506, intron 2 of 22)	intron (NM_175506, intron 2 of 22)	51236	NM_175506	240322	Mm.71963	NM_175506	ENSMUSG00000053441	**Adamts19**	4831407I23Rik|AU015154|D230034E10Rik	a disintegrin-like and metallopeptidase (reprolysin type) with thrombospondin type 1 motif, 19	LOSS	protein-coding

214_T22,T2,T32,T3,T42,T4	chr14	2.1E+07	2E+07	274999	+	**6**	**75**	NA	intron (NM_134079, intron 4 of 10)	intron (NM_134079, intron 4 of 10)	104848	NM_001243041	11534	Mm.188734	NM_134079	ENSMUSG00000039197	**Adk**	2310026J05Rik|5033405D03Rik|AI255373|AI987814|Ak	adenosine kinase	LOSS	protein-coding
1221_T12,T22,T2,T32,T3,T4	chr18	3.7E+07	4E+07	999	+	**6**	**75**	NA	intron (NM_175375, intron 10 of 33)	B1F|SINE|Alu	48397	NM_175375	108857	Mm.482287	NM_175375	ENSMUSG00000024483	**Ankhd1**	1110004O12Rik|4933432B13Rik|9130019P20Rik|A530027J04Rik|A630021B20Rik|AA571404|Mask|mFLJ00246|mKIAA1085	ankyrin repeat and KH domain containing 1	LOSS	protein-coding
1223_T12,T1,T22,T2,T32,T4	chr18	3.7E+07	4E+07	999	+	**6**	**75**	NA	intron (NM_175375, intron 10 of 33)	intron (NM_175375, intron 10 of 33)	50397	NM_175375	108857	Mm.482287	NM_175375	ENSMUSG00000024483	**Ankhd1**	1110004O12Rik|4933432B13Rik|9130019P20Rik|A530027J04Rik|A630021B20Rik|AA571404|Mask|mFLJ00246|mKIAA1085	ankyrin repeat and KH domain containing 1	LOSS	protein-coding
1247_T12,T1,T22,T2,T32,T3	chr18	3.8E+07	4E+07	1217999	+	**6**	**75**	NA	Intergenic	RLTR28B|LTR|ERVL	-3645	NM_175164	71302	Mm.329396	NM_175164	ENSMUSG00000036452	**Arhgap26**	1810044B20Rik|2610010G17Rik|4933432P15Rik|AI853435|mKIAA0621	Rho GTPase activating protein 26	LOSS	protein-coding
249_T1,T22,T2,T32,T3,T42	chr14	2.7E+07	3E+07	1999	+	**6**	**75**	NA	exon (NM_134437, exon 12 of 13)	exon (NM_134437, exon 12 of 13)	-44493	NM_001289686	71704	Mm.248606	NM_027871	ENSMUSG00000021895	**Arhgef3**	1200004I24Rik|9830169H03Rik|C76747|Xpln	Rho guanine nucleotide exchange factor (GEF) 3	LOSS	protein-coding
257_T22,T2,T32,T3,T42,T4	chr14	2.7E+07	3E+07	13999	+	**6**	**75**	NA	intron (NM_001289688, intron 2 of 9)	intron (NM_001289688, intron 2 of 9)	4111	NM_001289688	71704	Mm.248606	NM_027871	ENSMUSG00000021895	**Arhgef3**	1200004I24Rik|9830169H03Rik|C76747|Xpln	Rho guanine nucleotide exchange factor (GEF) 3	LOSS	protein-coding
1720_T12,T1,T2,T32,T3,T42	chr18	6.2E+07	6E+07	21999	+	**6**	**75**	NA	intron (NM_177828, intron 1 of 12)	Lx7|LINE|L1	6036	NM_177828	328967	Mm.488098	NM_177828	ENSMUSG00000045094	**Arhgef37**	4933429F08Rik	Rho guanine nucleotide exchange factor (GEF) 37	LOSS	protein-coding
957_T12,T1,T2,T32,T3,T42	chr18	2.2E+07	2E+07	4999	+	**6**	**75**	NA	Intergenic	Intergenic	-50089	NM_001167777	211961	Mm.392310	NM_001167777	ENSMUSG00000045215	**Asxl3**	C230079D11Rik|D430002O22Rik|D930044O18Rik|Gm329|Gm945	additional sex combs like 3 (Drosophila)	LOSS	protein-coding
2082_T12,T1,T2,T32,T3,T42	chr18	8.1E+07	8E+07	17999	+	**6**	**75**	NA	intron (NM_001201569, intron 1 of 29)	intron (NM_001201569, intron 1 of 29)	9558	NM_001201569	50771	Mm.247138	NM_015805	ENSMUSG00000024566	**Atp9b**	AA934181|Atpc2b|IIb|MMR	ATPase, class II, type 9B	LOSS	protein-coding
2078_T12,T1,T2,T32,T3,T42	chr18	8.1E+07	8E+07	999	+	**6**	**75**	NA	intron (NM_001201569, intron 4 of 29)	intron (NM_001201569, intron 4 of 29)	34058	NM_001201569	50771	Mm.247138	NM_015805	ENSMUSG00000024566	**Atp9b**	AA934181|Atpc2b|IIb|MMR	ATPase, class II, type 9B	LOSS	protein-coding

2080_T12,T1,T2,T32,T3,T42	chr18	8.1E+07	8E+07	12999	+	**6**	**75**	NA	intron (NM_001201569, intron 4 of 29)	L2a|LINE|L2	26058	NM_001201569	50771	Mm.247138	NM_015805	ENSMUSG00000024566	**Atp9b**	AA934181|Atpc2b|IIb|MMR	ATPase, class II, type 9B	LOSS	protein-coding
151_T1,T22,T2,T32,T3,T4	chr14	1.4E+07	1E+07	999	+	**6**	**75**	NA	Intergenic	B2_Mm2|SINE|B2	-5491	NM_139227	246103	Mm.133625	NM_139227	ENSMUSG00000021738	**Atxn7**	A430107N12Rik|AI627028|Sca7|ataxin-7	ataxin 7	LOSS	protein-coding
313_T12,T22,T2,T3,T42,T4	chr14	3.4E+07	3E+07	83999	+	**6**	**75**	NA	intron (NM_009758, intron 1 of 12)	L1MCa|LINE|L1	27046	NM_009758	12166	Mm.237825	NM_009758	ENSMUSG00000021796	**Bmpr1a**	1110037I22Rik|ALK3|AU045487|BMPR-IA|Bmpr	bone morphogenetic protein receptor, type 1A	LOSS	protein-coding
2192_T12,T1,T22,T2,T32,T3	chr18	8.7E+07	9E+07	270999	+	**6**	**75**	NA	Intergenic	Intergenic	774935	NM_172633	12405	Mm.70775	NM_172633	ENSMUSG00000024647	**Cbln2**	6330593N19Rik|A730004O05	cerebellin 2 precursor protein	LOSS	protein-coding
955_T12,T1,T2,T32,T3,T42	chr18	2.2E+07	2E+07	139999	+	**6**	**75**	NA	Intergenic	Intergenic	-50104	NM_027616	70950	Mm.235716	NM_027616	ENSMUSG00000024306	**Ccdc178**	4921528I01Rik	coiled coil domain containing 178	LOSS	protein-coding
1841_T12,T1,T22,T2,T32,T3	chr18	6.9E+07	7E+07	574999	+	**6**	**75**	NA	intron (NM_001083967, intron 9 of 19)	intron (NM_001083967, intron 9 of 19)	-289559	NM_201362	381175	Mm.266831	NM_201362	ENSMUSG00000038903	**Ccdc68**	BC046343	coiled-coil domain containing 68	LOSS	protein-coding
1843_T12,T1,T22,T2,T32,T3	chr18	7E+07	7E+07	46999	+	**6**	**75**	NA	intron (NM_201362, intron 6 of 10)	intron (NM_201362, intron 6 of 10)	25441	NM_201362	381175	Mm.266831	NM_201362	ENSMUSG00000038903	**Ccdc68**	BC046343	coiled-coil domain containing 68	LOSS	protein-coding
350_T12,T22,T2,T3,T42,T4	chr14	3.7E+07	4E+07	50999	+	**6**	**75**	NA	Intergenic	Intergenic	364568	NM_028407	72972	Mm.27621	NM_027045	ENSMUSG00000058690	**Ccser2**	1700012P13Rik|2900054P12Rik|AI035535|AW108503|Fam190b|Gcap14	coiled-coil serine rich 2	LOSS	protein-coding
1420_T12,T1,T22,T2,T32,T3	chr18	4.7E+07	5E+07	22999	+	**6**	**75**	NA	Intergenic	(CA)n|Simple_repeat|Simple_repeat	24342	NM_033037	12583	Mm.241056	NM_033037	ENSMUSG00000033022	**Cdo1**	1300002L19Rik|Cdo|D18Ucla3	cysteine dioxygenase 1, cytosolic	LOSS	protein-coding
1422_T12,T1,T22,T2,T32,T3	chr18	4.7E+07	5E+07	6999	+	**6**	**75**	NA	intron (NM_033037, intron 3 of 4)	intron (NM_033037, intron 3 of 4)	8342	NM_033037	12583	Mm.241056	NM_033037	ENSMUSG00000033022	**Cdo1**	1300002L19Rik|Cdo|D18Ucla3	cysteine dioxygenase 1, cytosolic	LOSS	protein-coding
1595_T12,T1,T22,T2,T32,T3	chr18	5.4E+07	5E+07	26999	+	**6**	**75**	NA	Intergenic	MTB|LTR|ERVL-MaLR	-20453	NM_178686	225523	Mm.193678	NM_178686	ENSMUSG00000048799	**Cep120**	A230075C01|AU016693|Ccdc100	centrosomal protein 120	LOSS	protein-coding
803_T12,T1,T22,T2,T32,T3	chr18	1.6E+07	2E+07	19999	+	**6**	**75**	NA	Intergenic	Intergenic	-3454	NM_199055	71367	Mm.329304	NM_199055	ENSMUSG00000047161	**Chst9**	5430438D01Rik	carbohydrate (N-acetylgalactosamine 4-0) sulfotransferase 9	LOSS	protein-coding
1425_T12,T1,T22,T2,T32,T3	chr18	4.7E+07	5E+07	433999	+	**6**	**75**	NA	intron (NM_178377, intron 3 of 6)	ORR1E-int|LTR|ERVL-MaLR	6624	NM_178377	69456	Mm.210734	NM_178377	ENSMUSG00000042705	**Commd10**	2310003A05Rik|AU019438|DRWMS2	COMM domain containing 10	LOSS	protein-coding

1722_T12,T1,T2,T32,T3,T42	chr18	6.2E+07	6E+07	17999	+	**6**	**75**	NA	Intergenic	(TTTG)n|Simple_repeat|Simple_repeat	-4082	NM_146087	93687	Mm.26908	NM_146087	ENSMUSG00000024576	**Csnk1a1**	2610208K14Rik|4632404G05Rik|5430427P18Rik|CK1a|Csnk1a	casein kinase 1, alpha 1	LOSS	protein-coding
1599_T12,T1,T22,T2,T32,T3	chr18	5.4E+07	5E+07	25999	+	**6**	**75**	NA	Intergenic	ORR1A2|LTR|ERVL-MaLR	-8613	NM_152809	70425	Mm.368668	NM_152809	ENSMUSG00000073563	**Csnk1g3**	3300002K07Rik|C330049O21Rik	casein kinase 1, gamma 3	LOSS	protein-coding
1606_T12,T1,T22,T2,T32,T3	chr18	5.4E+07	5E+07	82999	+	**6**	**75**	NA	Intergenic	MTE2b|LTR|ERVL-MaLR	104887	NM_152809	70425	Mm.368668	NM_152809	ENSMUSG00000073563	**Csnk1g3**	3300002K07Rik|C330049O21Rik	casein kinase 1, gamma 3	LOSS	protein-coding
1610_T12,T1,T22,T2,T32,T3	chr18	5.4E+07	5E+07	7999	+	**6**	**75**	NA	Intergenic	Intergenic	193387	NM_152809	70425	Mm.368668	NM_152809	ENSMUSG00000073563	**Csnk1g3**	3300002K07Rik|C330049O21Rik	casein kinase 1, gamma 3	LOSS	protein-coding
1612_T12,T1,T22,T2,T32,T3	chr18	5.4E+07	5E+07	125999	+	**6**	**75**	NA	Intergenic	Intergenic	266387	NM_152809	70425	Mm.368668	NM_152809	ENSMUSG00000073563	**Csnk1g3**	3300002K07Rik|C330049O21Rik	casein kinase 1, gamma 3	LOSS	protein-coding
1602_T12,T1,T22,T2,T32,T3	chr18	5.4E+07	5E+07	11999	+	**6**	**75**	NA	intron (NM_152809, intron 1 of 12)	intron (NM_152809, intron 1 of 12)	20387	NM_152809	70425	Mm.368668	NM_152809	ENSMUSG00000073563	**Csnk1g3**	3300002K07Rik|C330049O21Rik	casein kinase 1, gamma 3	LOSS	protein-coding
1604_T12,T1,T22,T2,T32,T3	chr18	5.4E+07	5E+07	34999	+	**6**	**75**	NA	intron (NM_152809, intron 4 of 12)	RSINE1|SINE|B4	44887	NM_152809	70425	Mm.368668	NM_152809	ENSMUSG00000073563	**Csnk1g3**	3300002K07Rik|C330049O21Rik	casein kinase 1, gamma 3	LOSS	protein-coding
1203_T12,T1,T22,T2,T32,T3	chr18	3.6E+07	4E+07	126999	+	**6**	**75**	NA	intron (NM_001081365, intron 1 of 2)	B4A|SINE|B4	13376	NM_001081365	66060	Mm.272527	NM_001081365	ENSMUSG00000046727	**Cystm1**	0610010O12Rik	cysteine-rich transmembrane module containing 1	LOSS	protein-coding
1905_T12,T1,T22,T2,T32,T42	chr18	7.2E+07	7E+07	26999	+	**6**	**75**	NA	intron (NM_007831, intron 5 of 28)	intron (NM_007831, intron 5 of 28)	600069	NM_007831	13176	Mm.167882	NM_007831	ENSMUSG00000060534	**Dcc**	C030036D22Rik|Igdcc1	deleted in colorectal carcinoma	LOSS	protein-coding
1907_T12,T1,T22,T2,T32,T42	chr18	7.2E+07	7E+07	22999	+	**6**	**75**	NA	intron (NM_007831, intron 5 of 28)	(TATATG)n|Simple_repeat|Simple_repeat	571069	NM_007831	13176	Mm.167882	NM_007831	ENSMUSG00000060534	**Dcc**	C030036D22Rik|Igdcc1	deleted in colorectal carcinoma	LOSS	protein-coding
1900_T1,T22,T2,T32,T3,T42	chr18	7.2E+07	7E+07	999	+	**6**	**75**	NA	intron (NM_007831, intron 7 of 28)	intron (NM_007831, intron 7 of 28)	673069	NM_007831	13176	Mm.167882	NM_007831	ENSMUSG00000060534	**Dcc**	C030036D22Rik|Igdcc1	deleted in colorectal carcinoma	LOSS	protein-coding
473_T12,T22,T2,T3,T42,T4	chr14	4.6E+07	5E+07	16999	+	**6**	**75**	NA	Intergenic	Intergenic	-81857	NM_001042719	114874	Mm.121918	NM_176845	ENSMUSG00000037697	**Ddhd1**	Mir5131|PA-PLA1	DDHD domain containing 1	LOSS	protein-coding
2259_T12,T1,T22,T2,T32,T3	chr18	9E+07	9E+07	6999	+	**6**	**75**	NA	Intergenic	RMER3D-int|LTR|ERVK	-227864	NM_001039173	623279	Mm.447164	NM_001039173	ENSMUSG00000073514	**Dok6**	Dok-6	docking protein 6	LOSS	protein-coding

2261_T12,T1,T22,T2,T32,T3	chr18	9E+07	9E+07	16999	+	**6**	**75**	NA	Intergenic	L1Md_F2|LINE|L1	-240864	NM_001039173	623279	Mm.447164	NM_001039173	ENSMUSG00000073514	**Dok6**	Dok-6	docking protein 6	LOSS	protein-coding
2263_T12,T1,T22,T2,T32,T3	chr18	9E+07	9E+07	32999	+	**6**	**75**	NA	Intergenic	L1Md_F2|LINE|L1	-266864	NM_001039173	623279	Mm.447164	NM_001039173	ENSMUSG00000073514	**Dok6**	Dok-6	docking protein 6	LOSS	protein-coding
893_T12,T1,T22,T2,T32,T3	chr18	1.9E+07	2E+07	54999	+	**6**	**75**	NA	Intergenic	L1_Mus3|LINE|L1	685097	NM_007882	13507	Mm.89935	NM_007882	ENSMUSG00000059898	**Dsc3**	5430426I24Rik	desmocollin 3	LOSS	protein-coding
910_T12,T1,T2,T32,T42,T4	chr18	2E+07	2E+07	1999	+	**6**	**75**	NA	Intergenic	Intergenic	403597	NM_007882	13507	Mm.89935	NM_007882	ENSMUSG00000059898	**Dsc3**	5430426I24Rik	desmocollin 3	LOSS	protein-coding
929_T12,T1,T22,T2,T32,T42	chr18	2.1E+07	2E+07	15999	+	**6**	**75**	NA	exon (NM_030596, exon 3 of 16)	exon (NM_030596, exon 3 of 16)	10196	NM_030596	13512	Mm.106811	NM_030596	ENSMUSG00000056632	**Dsg3**	bal	desmoglein 3	LOSS	protein-coding
2018_T12,T1,T2,T32,T3,T42	chr18	7.8E+07	8E+07	104999	+	**6**	**75**	NA	Intergenic	(CA)n|Simple_repeat|Simple_repeat	-3467	NM_001195633	1E+08	Mm.297782	NM_001195633	ENSMUSG00000039840	**Epg5**	4732475F16|5430411K18Rik|AI661957|AW456499|mKIAA1632	ectopic P-granules autophagy protein 5 homolog (C. elegans)	LOSS	protein-coding
2020_T12,T1,T2,T32,T3,T42	chr18	7.8E+07	8E+07	3999	+	**6**	**75**	NA	intron (NM_001195633, intron 24 of 43)	intron (NM_001195633, intron 24 of 43)	52033	NM_001195633	1E+08	Mm.297782	NM_001195633	ENSMUSG00000039840	**Epg5**	4732475F16|5430411K18Rik|AI661957|AW456499|mKIAA1632	ectopic P-granules autophagy protein 5 homolog (C. elegans)	LOSS	protein-coding
1158_T12,T1,T22,T2,T32,T3	chr18	3.2E+07	3E+07	13999	+	**6**	**75**	NA	intron (NM_011946, intron 5 of 16)	intron (NM_011946, intron 5 of 16)	-37831	NM_133658	13872	Mm.282335	NM_133658	ENSMUSG00000024382	**Ercc3**	BTF2 p89|Ercc-3|XPB	excision repair cross-complementing rodent repair deficiency, complementation group 3	LOSS	protein-coding
1160_T12,T1,T22,T2,T32,T3	chr18	3.2E+07	3E+07	82999	+	**6**	**75**	NA	intron (NM_133658, intron 8 of 14)	L1ME3|LINE|L1	11669	NM_133658	13872	Mm.282335	NM_133658	ENSMUSG00000024382	**Ercc3**	BTF2 p89|Ercc-3|XPB	excision repair cross-complementing rodent repair deficiency, complementation group 3	LOSS	protein-coding
1497_T12,T1,T22,T2,T32,T3	chr18	5E+07	5E+07	26999	+	**6**	**75**	NA	Intergenic	Lx7|LINE|L1	-38369	NM_001004061	225497	Mm.329435	NM_001004061	ENSMUSG00000035420	**Fam170a**	Gm93|Znfd	family with sequence similarity 170, member A	LOSS	protein-coding
259_T22,T2,T32,T3,T42,T4	chr14	2.7E+07	3E+07	146999	+	**6**	**75**	NA	intron (NM_001114879, intron 7 of 23)	intron (NM_001114879, intron 7 of 23)	18153	NM_001114879	218850	Mm.247767	NM_028945	ENSMUSG00000040651	**Fam208a**	4732418E12|4933409E02Rik|D14Abb1e|MommeD6	family with sequence similarity 208, member A	LOSS	protein-coding
308_T12,T22,T2,T3,T42,T4	chr14	3.4E+07	3E+07	999	+	**6**	**75**	NA	intron (NM_029389, intron 2 of 10)	intron (NM_029389, intron 2 of 10)	12503	NM_029389	75698	Mm.45406	NM_029389	ENSMUSG00000041471	**Fam35a**	3110001K24Rik	family with sequence similarity 35, member A	LOSS	protein-coding
1658_T12,T1,T22,T2,T3,T42	chr18	5.8E+07	6E+07	999	+	**6**	**75**	NA	Intergenic	L1M2|LINE|L1	-44074	NM_010181	14119	Mm.20271	NM_010181	ENSMUSG00000024598	**Fbn2**	BC063774|Fib-2|mKIAA4226|sne|sy	fibrillin 2	LOSS	protein-coding

1661_T12,T1,T22,T2,T3,T4	chr18	5.8E+07	6E+07	17999	+	**6**	**75**	NA	Intergenic	Intergenic	-59574	NM_010181	14119	Mm.20271	NM_010181	ENSMUSG00000024598	**Fbn2**	BC063774|Fib-2|mKIAA4226|sne|sy	fibrillin 2	LOSS	protein-coding
1414_T12,T1,T2,T32,T3,T42	chr18	4.7E+07	5E+07	999	+	**6**	**75**	NA	Intergenic	Intergenic	-9029	NM_173423	240263	Mm.38249	NM_173423	ENSMUSG00000033319	**Fem1c**	2610312A07Rik|3632443A22Rik|AI646567	fem-1 homolog c (C.elegans)	LOSS	protein-coding
1412_T12,T1,T2,T32,T3,T42	chr18	4.7E+07	5E+07	999	+	**6**	**75**	NA	intron (NM_173423, intron 2 of 2)	intron (NM_173423, intron 2 of 2)	15971	NM_173423	240263	Mm.38249	NM_173423	ENSMUSG00000033319	**Fem1c**	2610312A07Rik|3632443A22Rik|AI646567	fem-1 homolog c (C.elegans)	LOSS	protein-coding
1803_T1,T22,T2,T32,T3,T42	chr18	6.7E+07	7E+07	999	+	**6**	**75**	NA	Intergenic	Intergenic	-42298	NM_177137	14680	Mm.440746	NM_010307	ENSMUSG00000024524	**Gnal**	2610011C15Rik|9630020G10Rik|Galphaolf|Gna10|Golf	guanine nucleotide binding protein, alpha stimulating, olfactory type	LOSS	protein-coding
1805_T1,T22,T2,T32,T3,T42	chr18	6.7E+07	7E+07	999	+	**6**	**75**	NA	Intergenic	Intergenic	-24298	NM_177137	14680	Mm.440746	NM_010307	ENSMUSG00000024524	**Gnal**	2610011C15Rik|9630020G10Rik|Galphaolf|Gna10|Golf	guanine nucleotide binding protein, alpha stimulating, olfactory type	LOSS	protein-coding
466_T12,T22,T2,T3,T42,T4	chr14	4.5E+07	5E+07	154999	+	**6**	**75**	NA	Intergenic	Intergenic	-62204	NM_019425	54342	Mm.312945	NM_019425	ENSMUSG00000037722	**Gnpnat1**	AU017428|AU040593|EMeg32|Gpnat1|Gsnpat	glucosamine-phosphate N-acetyltransferase 1	LOSS	protein-coding
451_T12,T22,T2,T3,T42,T4	chr14	4.5E+07	5E+07	20999	+	**6**	**75**	NA	intron (NM_027518, intron 1 of 6)	intron (NM_027518, intron 1 of 6)	4283	NM_027518	70713	Mm.343793	NM_027518	ENSMUSG00000049092	**Gpr137c**	6330416L11Rik|Gm908|TM7SF1L2	G protein-coupled receptor 137C	LOSS	protein-coding
551_T12,T22,T2,T32,T3,T4	chr14	5.2E+07	5E+07	999	+	**6**	**75**	NA	intron (NM_001170982, intron 1 of 7)	B2_Mm1t|SINE|B2	9028	NM_001170982	15381	Mm.427321	NM_016884	ENSMUSG00000060373	**Hnrnpc**	AL022939|D14Wsu171e|Hnrpc|Hnrpc1|Hnrpc2|hnRNPC1|hnRNPC2|hnrnp-C	heterogeneous nuclear ribonucleoprotein C	LOSS	protein-coding
1667_T12,T1,T2,T32,T3,T42	chr18	5.9E+07	6E+07	2999	+	**6**	**75**	NA	intron (NM_025478, intron 4 of 4)	intron (NM_025478, intron 4 of 4)	16518	NM_025478	66307	Mm.182574	NM_025478	ENSMUSG00000024601	**Isoc1**	2610034N03Rik	isochorismatase domain containing 1	LOSS	protein-coding
591_T12,T22,T2,T3,T42,T4	chr14	5.5E+07	6E+07	40999	+	**6**	**75**	NA	Intergenic	Intergenic	-24065	NM_177049	319984	Mm.19047	NM_177049	ENSMUSG00000022208	**Jph4**	9330157P13Rik|JP-4|JPHL1	junctophilin 4	LOSS	protein-coding
1386_T12,T1,T22,T2,T32,T3	chr18	4.5E+07	5E+07	12999	+	**6**	**75**	NA	intron (NM_001312905, intron 1 of 10)	intron (NM_001312905, intron 1 of 10)	34140	NM_001312905	140492	Mm.411614	NM_080465	ENSMUSG00000054477	**Kcnn2**	KCa2.2|SK2|SKCA2|bc|fri	potassium intermediate/small conductance calcium-activated channel, subfamily N, member 2	LOSS	protein-coding
1390_T12,T1,T22,T2,T32,T3	chr18	4.5E+07	5E+07	142999	+	**6**	**75**	NA	intron (NM_001312905, intron 2 of 10)	MT2C_Mm|LTR|ERVL	-125154	NM_080465	140492	Mm.411614	NM_080465	ENSMUSG00000054477	**Kcnn2**	KCa2.2|SK2|SKCA2|bc|fri	potassium intermediate/small conductance calcium-activated channel, subfamily N, member 2	LOSS	protein-coding

1280_T12,T1,T22,T2,T3,T42	chr18	4.1E+07	4E+07	3999	+	**6**	**75**	NA	Intergenic	L1ME1|LINE|L1	341139	NM_026135	383348	Mm.76366	NM_026135	ENSMUSG00000051401	**Kctd16**	4930434H12Rik|Gm1267	potassium channel tetramerisation domain containing 16	LOSS	protein-coding
1259_T12,T1,T22,T2,T32,T42	chr18	4E+07	4E+07	5999	+	**6**	**75**	NA	intron (NM_026135, intron 1 of 1)	intron (NM_026135, intron 1 of 1)	5139	NM_026135	383348	Mm.76366	NM_026135	ENSMUSG00000051401	**Kctd16**	4930434H12Rik|Gm1267	potassium channel tetramerisation domain containing 16	LOSS	protein-coding
1261_T12,T1,T22,T2,T32,T42	chr18	4E+07	4E+07	5999	+	**6**	**75**	NA	intron (NM_026135, intron 1 of 1)	intron (NM_026135, intron 1 of 1)	15139	NM_026135	383348	Mm.76366	NM_026135	ENSMUSG00000051401	**Kctd16**	4930434H12Rik|Gm1267	potassium channel tetramerisation domain containing 16	LOSS	protein-coding
1263_T1,T22,T2,T32,T3,T42	chr18	4E+07	4E+07	4999	+	**6**	**75**	NA	intron (NM_026135, intron 1 of 1)	MTE2a|LTR|ERVL-MaLR	116639	NM_026135	383348	Mm.76366	NM_026135	ENSMUSG00000051401	**Kctd16**	4930434H12Rik|Gm1267	potassium channel tetramerisation domain containing 16	LOSS	protein-coding
1265_T12,T1,T2,T32,T3,T42	chr18	4E+07	4E+07	999	+	**6**	**75**	NA	intron (NM_026135, intron 1 of 1)	intron (NM_026135, intron 1 of 1)	161639	NM_026135	383348	Mm.76366	NM_026135	ENSMUSG00000051401	**Kctd16**	4930434H12Rik|Gm1267	potassium channel tetramerisation domain containing 16	LOSS	protein-coding
487_T12,T22,T2,T3,T42,T4	chr14	4.8E+07	5E+07	104999	+	**6**	**75**	NA	promoter-TSS (NM_008477)	promoter-TSS (NM_008477)	-756	NM_008477	16709	Mm.3110	NM_008477	ENSMUSG00000021843	**Ktn1**	-	kinectin 1	LOSS	protein-coding
1333_T12,T1,T22,T2,T32,T4	chr18	4.2E+07	4E+07	999	+	**6**	**75**	NA	intron (NM_134137, intron 10 of 31)	B1_Mur2|SINE|Alu	23071	NM_134137	107045	Mm.312170	NM_134137	ENSMUSG00000024493	**Lars**	2310045K21Rik|3110009L02Rik|AW536573|mKIAA1352	leucyl-tRNA synthetase	LOSS	protein-coding
1335_T12,T1,T22,T2,T32,T4	chr18	4.2E+07	4E+07	999	+	**6**	**75**	NA	intron (NM_134137, intron 10 of 31)	ID_B1|SINE|B4	21071	NM_134137	107045	Mm.312170	NM_134137	ENSMUSG00000024493	**Lars**	2310045K21Rik|3110009L02Rik|AW536573|mKIAA1352	leucyl-tRNA synthetase	LOSS	protein-coding
1331_T12,T1,T22,T2,T32,T4	chr18	4.2E+07	4E+07	6999	+	**6**	**75**	NA	intron (NM_134137, intron 14 of 31)	intron (NM_134137, intron 14 of 31)	28071	NM_134137	107045	Mm.312170	NM_134137	ENSMUSG00000024493	**Lars**	2310045K21Rik|3110009L02Rik|AW536573|mKIAA1352	leucyl-tRNA synthetase	LOSS	protein-coding
1815_T12,T1,T22,T32,T3,T42	chr18	6.8E+07	7E+07	2999	+	**6**	**75**	NA	intron (NM_172631, intron 1 of 5)	intron (NM_172631, intron 1 of 5)	51743	NM_172631	52662	Mm.440183	NM_172631	ENSMUSG00000024544	**Ldlrad4**	8230401C20Rik|A430083H02|A430108L08Rik|C18orf1|D18Ertd653e|D330030L18Rik	low density lipoprotein receptor class A domain containing 4	LOSS	protein-coding
1149_T12,T1,T2,T32,T3,T42	chr18	3.2E+07	3E+07	999	+	**6**	**75**	NA	intron (NM_144862, intron 1 of 9)	intron (NM_144862, intron 1 of 9)	8493	NM_144862	225341	Mm.24551	NM_144862	ENSMUSG00000024395	**Lims2**	PINCH2	LIM and senescent cell antigen like domains 2	LOSS	protein-coding

1196_T12,T1,T22,T2,T32,T3	chr18	3.5E+07	4E+07	68999	+	**6**	**75**	NA	3' UTR (NM_178005, exon 2 of 2)	3' UTR (NM_178005, exon 2 of 2)	5024	NM_178005	107065	Mm.39900	NM_178005	ENSMUSG00000071862	**Lrrtm2**	AI851755|BB129880|C630011A14Rik|mKIAA0416	leucine rich repeat transmembrane neuronal 2	LOSS	protein-coding
1823_T12,T1,T2,T32,T3,T42	chr18	6.8E+07	7E+07	22999	+	**6**	**75**	NA	Intergenic	RLTR34D_MM|LTR|ERVK	28840	NM_001271717	17200	Mm.426053	NM_008560	ENSMUSG00000045569	**Mc2r**	ACTH-R|ACTHR|MC2-R	melanocortin 2 receptor	LOSS	protein-coding
1820_T12,T22,T2,T32,T3,T42	chr18	6.8E+07	7E+07	3999	+	**6**	**75**	NA	Intergenic	Intergenic	40897	NM_013596	17203	Mm.8003	NM_013596	ENSMUSG00000007480	**Mc5r**	-	melanocortin 5 receptor	LOSS	protein-coding
1186_T12,T1,T2,T32,T3,T4	chr18	3.3E+07	3E+07	999	+	**6**	**75**	NA	intron (NM_001267717, intron 3 of 4)	intron (NM_001267717, intron 3 of 4)	2634	NM_001267717	27528	Mm.407415	NM_053078	ENSMUSG00000042834	**Nrep**	AI325076|D0H4S114|Harp|P311|PTZ17|SEZ17	neuronal regeneration related protein	LOSS	protein-coding
2300_T12,T22,T2,T32,T3,T42	chr2	8.1E+07	8E+07	999	+	**6**	**75**	NA	Intergenic	L1Md_A|LINE|L1	173736	NM_001190179	69482	Mm.29200	NM_027091	ENSMUSG00000026999	**Nup35**	2310006I24Rik|35kDa|5330402E05Rik|MP44|NO44	nucleoporin 35	LOSS	protein-coding
494_T12,T22,T2,T3,T42,T4	chr14	4.9E+07	5E+07	48999	+	**6**	**75**	NA	3' UTR (NM_001286483, exon 4 of 4)	3' UTR (NM_001286483, exon 4 of 4)	4872	NM_144841	18424	Mm.134516	NM_144841	ENSMUSG00000021848	**Otx2**	E130306E05Rik	orthodenticle homeobox 2	LOSS	protein-coding
2052_T12,T1,T22,T2,T32,T3	chr18	8E+07	8E+07	130999	+	**6**	**75**	NA	Intergenic	Intergenic	-466895	NM_053117	93737	Mm.24678	NM_053117	ENSMUSG00000056214	**Pard6g**	2410049N21Rik|Par6a	par-6 family cell polarity regulator gamma	LOSS	protein-coding
2059_T1,T22,T2,T32,T3,T42	chr18	8E+07	8E+07	13999	+	**6**	**75**	NA	Intergenic	Intergenic	-328395	NM_053117	93737	Mm.24678	NM_053117	ENSMUSG00000056214	**Pard6g**	2410049N21Rik|Par6a	par-6 family cell polarity regulator gamma	LOSS	protein-coding
596_T12,T22,T2,T3,T42,T4	chr14	5.6E+07	6E+07	48999	+	**6**	**75**	NA	intron (NM_028994, intron 2 of 9)	intron (NM_028994, intron 2 of 9)	2734	NM_028994	74551	Mm.29856	NM_028994	ENSMUSG00000040618	**Pck2**	1810010O14Rik|9130022B02Rik|PEPCK-M	phosphoenolpyruvate carboxykinase 2 (mitochondrial)	LOSS	protein-coding
247_T1,T2,T32,T3,T42,T4	chr14	2.7E+07	3E+07	1999	+	**6**	**75**	NA	Intergenic	ERVB4_1-I_MM-int|LTR|ERVK	-89654	NM_178668	211948	Mm.290758	NM_178668	ENSMUSG00000043702	**Pde12**	2'-PDE|E430028B21Rik	phosphodiesterase 12	LOSS	protein-coding
1210_T12,T1,T22,T2,T32,T3	chr18	3.6E+07	4E+07	36999	+	**6**	**75**	NA	intron (NM_026027, intron 1 of 3)	intron (NM_026027, intron 1 of 3)	495	NM_026027	67199	Mm.30184	NM_026027	ENSMUSG00000024346	**Pfdn1**	2700086I23Rik|AA408327|AU044714	prefoldin 1	LOSS	protein-coding
1208_T12,T1,T22,T2,T32,T3	chr18	3.6E+07	4E+07	1999	+	**6**	**75**	NA	intron (NM_026027, intron 2 of 3)	intron (NM_026027, intron 2 of 3)	20995	NM_026027	67199	Mm.30184	NM_026027	ENSMUSG00000024346	**Pfdn1**	2700086I23Rik|AA408327|AU044714	prefoldin 1	LOSS	protein-coding
1206_T12,T1,T22,T2,T32,T3	chr18	3.6E+07	4E+07	3999	+	**6**	**75**	NA	intron (NM_026027, intron 3 of 3)	B1_Mur3|SINE|Alu	24995	NM_026027	67199	Mm.30184	NM_026027	ENSMUSG00000024346	**Pfdn1**	2700086I23Rik|AA408327|AU044714	prefoldin 1	LOSS	protein-coding

1990_T12,T1,T2,T32,T3,T42	chr18	7.7E+07	8E+07	22999	+	**6**	**75**	NA	intron (NM_001164170, intron 1 of 12)	intron (NM_001164170, intron 1 of 12)	320	NM_001164168	17344	Mm.6370	NM_008602	ENSMUSG00000025423	**Pias2**	6330408K17Rik|AI462206|ARIP3|AU018068|Dib|Miz1|PIASxalpha|PIASxb|PIASxbeta|SIZ2	protein inhibitor of activated STAT 2	LOSS	protein-coding
1993_T1,T22,T32,T3,T42,T4	chr18	7.7E+07	8E+07	999	+	**6**	**75**	NA	intron (NM_001164170, intron 1 of 12)	intron (NM_001164170, intron 1 of 12)	14320	NM_001164168	17344	Mm.6370	NM_008602	ENSMUSG00000025423	**Pias2**	6330408K17Rik|AI462206|ARIP3|AU018068|Dib|Miz1|PIASxalpha|PIASxb|PIASxbeta|SIZ2	protein inhibitor of activated STAT 2	LOSS	protein-coding
1997_T12,T1,T2,T32,T3,T4	chr18	7.7E+07	8E+07	999	+	**6**	**75**	NA	intron (NM_001164170, intron 1 of 12)	B3|SINE|B2	28320	NM_001164168	17344	Mm.6370	NM_008602	ENSMUSG00000025423	**Pias2**	6330408K17Rik|AI462206|ARIP3|AU018068|Dib|Miz1|PIASxalpha|PIASxb|PIASxbeta|SIZ2	protein inhibitor of activated STAT 2	LOSS	protein-coding
1192_T12,T1,T22,T2,T32,T3	chr18	3.4E+07	4E+07	1410999	+	**6**	**75**	NA	intron (NM_001163004, intron 2 of 14)	RSINE1|SINE|B4	1577	NM_016927	53871	Mm.445792	NM_016927	ENSMUSG00000014503	**Pkd2l2**	TRPP5	polycystic kidney disease 2-like 2	LOSS	protein-coding
1787_T12,T1,T22,T2,T32,T3	chr18	6.6E+07	7E+07	4999	+	**6**	**75**	NA	Intergenic	URR1B|DNA|hAT-Charlie	-37604	NM_021451	58801	Mm.271878	NM_021451	ENSMUSG00000024521	**Pmaip1**	Noxa	phorbol-12-myristate-13-acetate-induced protein 1	LOSS	protein-coding
1789_T12,T1,T22,T2,T32,T3	chr18	6.6E+07	7E+07	18999	+	**6**	**75**	NA	Intergenic	B1_Mus1|SINE|Alu	-24604	NM_021451	58801	Mm.271878	NM_021451	ENSMUSG00000024521	**Pmaip1**	Noxa	phorbol-12-myristate-13-acetate-induced protein 1	LOSS	protein-coding
1581_T12,T1,T22,T2,T32,T3	chr18	5.3E+07	5E+07	141999	+	**6**	**75**	NA	intron (NM_029394, intron 2 of 6)	MLT1D|LTR|ERVL-MaLR	85507	NM_008908	19038	Mm.4587	NM_008908	ENSMUSG00000024538	**Ppic**	CyP-20c	peptidylprolyl isomerase C	LOSS	protein-coding
206_T22,T2,T32,T3,T42,T4	chr14	2.1E+07	2E+07	37999	+	**6**	**75**	NA	intron (NM_030180, intron 20 of 20)	MER20|DNA|hAT-Charlie	-4927	NM_008914	19056	Mm.274432	NM_008914	ENSMUSG00000021816	**Ppp3cb**	1110063J16Rik|Calnb|CnAbeta|Cnab	protein phosphatase 3, catalytic subunit, beta isoform	LOSS	protein-coding
1583_T12,T22,T2,T32,T3,T42	chr18	5.3E+07	5E+07	999	+	**6**	**75**	NA	Intergenic	Intergenic	-13546	NM_001033281	225518	Mm.297645	NM_001033281	ENSMUSG00000069378	**Prdm6**	Gm92|PRISM	PR domain containing 6	LOSS	protein-coding
1294_T12,T1,T22,T2,T3	chr18	4.1E+07	4E+07	118999	+	**6**	**75**	NA	Intergenic	Intergenic	720194	NM_029942	77619	Mm.30649	NM_029942	ENSMUSG00000056671	**Prelid2**	1700003A01Rik|C330008K14Rik	PRELI domain containing 2	LOSS	protein-coding
1306_T12,T1,T22,T2,T32,T42	chr18	4.1E+07	4E+07	6999	+	**6**	**75**	NA	Intergenic	Intergenic	559194	NM_029942	77619	Mm.30649	NM_029942	ENSMUSG00000056671	**Prelid2**	1700003A01Rik|C330008K14Rik	PRELI domain containing 2	LOSS	protein-coding
1308_T12,T1,T22,T2,T32,T42	chr18	4.1E+07	4E+07	4999	+	**6**	**75**	NA	Intergenic	(TG)n|Simple_repeat|Simple_repeat	488194	NM_029942	77619	Mm.30649	NM_029942	ENSMUSG00000056671	**Prelid2**	1700003A01Rik|C330008K14Rik	PRELI domain containing 2	LOSS	protein-coding

1323_T12,T1,T22,T2,T32,T42	chr18	4.2E+07	4E+07	4999	+	**6**	**75**	NA	Intergenic	Intergenic	-20806	NM_029942	77619	Mm.30649	NM_029942	ENSMUSG00000056671	**Prelid2**	1700003A01Rik|C330008K14Rik	PRELI domain containing 2	LOSS	protein-coding
1320_T12,T1,T22,T2,T32,T3	chr18	4.2E+07	4E+07	11999	+	**6**	**75**	NA	intron (NM_029942, intron 5 of 6)	intron (NM_029942, intron 5 of 6)	60694	NM_029942	77619	Mm.30649	NM_029942	ENSMUSG00000056671	**Prelid2**	1700003A01Rik|C330008K14Rik	PRELI domain containing 2	LOSS	protein-coding
1156_T12,T1,T22,T2,T32,T3	chr18	3.2E+07	3E+07	143999	+	**6**	**75**	NA	TTS (NM_008934)	TTS (NM_008934)	15705	NM_008934	19123	Mm.2786	NM_008934	ENSMUSG00000024386	**Proc**	PC	protein C	LOSS	protein-coding
1527_T12,T1,T22,T2,T32,T42	chr18	5.1E+07	5E+07	7999	+	**6**	**75**	NA	Intergenic	L1M2|LINE|L1	265602	NM_001081224	71373	Mm.63546	NM_001081224	ENSMUSG00000073565	**Prr16**	5430406M13Rik|AI607429	proline rich 16	LOSS	protein-coding
1529_T12,T1,T22,T2,T32,T42	chr18	5.1E+07	5E+07	5999	+	**6**	**75**	NA	Intergenic	Intergenic	273602	NM_001081224	71373	Mm.63546	NM_001081224	ENSMUSG00000073565	**Prr16**	5430406M13Rik|AI607429	proline rich 16	LOSS	protein-coding
1532_T12,T1,T22,T2,T32,T42	chr18	5.1E+07	5E+07	53999	+	**6**	**75**	NA	Intergenic	L1_Mur2|LINE|L1	309602	NM_001081224	71373	Mm.63546	NM_001081224	ENSMUSG00000073565	**Prr16**	5430406M13Rik|AI607429	proline rich 16	LOSS	protein-coding
1535_T12,T1,T22,T2,T32,T42	chr18	5.1E+07	5E+07	100999	+	**6**	**75**	NA	Intergenic	MTE-int|LTR|ERVL-MaLR	397102	NM_001081224	71373	Mm.63546	NM_001081224	ENSMUSG00000073565	**Prr16**	5430406M13Rik|AI607429	proline rich 16	LOSS	protein-coding
1537_T12,T1,T22,T2,T32,T42	chr18	5.2E+07	5E+07	999	+	**6**	**75**	NA	Intergenic	Lx5c|LINE|L1	449102	NM_001081224	71373	Mm.63546	NM_001081224	ENSMUSG00000073565	**Prr16**	5430406M13Rik|AI607429	proline rich 16	LOSS	protein-coding
1849_T12,T1,T22,T2,T32,T42	chr18	7E+07	7E+07	16999	+	**6**	**75**	NA	intron (NM_001082553, intron 2 of 6)	intron (NM_001082553, intron 2 of 6)	31588	NM_001302798	80718	Mm.246753	NM_030554	ENSMUSG00000024511	**Rab27b**	2310021G14Rik|B130064M09Rik	RAB27B, member RAS oncogene family	LOSS	protein-coding
1116_T12,T1,T22,T2,T32,T3	chr18	3.1E+07	3E+07	4999	+	**6**	**75**	NA	Intergenic	Intergenic	506128	NM_009065	19762	Mm.5163	NM_009065	ENSMUSG00000057455	**Rit2**	RIBA|Rin|Roc2	Ras-like without CAAX 2	LOSS	protein-coding
1120_T12,T1,T22,T2,T32,T3	chr18	3.1E+07	3E+07	6999	+	**6**	**75**	NA	Intergenic	L1_Mus3|LINE|L1	450128	NM_009065	19762	Mm.5163	NM_009065	ENSMUSG00000057455	**Rit2**	RIBA|Rin|Roc2	Ras-like without CAAX 2	LOSS	protein-coding
1131_T12,T1,T22,T2,T32,T4	chr18	3.1E+07	3E+07	3999	+	**6**	**75**	NA	intron (NM_009065, intron 4 of 4)	intron (NM_009065, intron 4 of 4)	290628	NM_009065	19762	Mm.5163	NM_009065	ENSMUSG00000057455	**Rit2**	RIBA|Rin|Roc2	Ras-like without CAAX 2	LOSS	protein-coding
2012_T12,T1,T32,T3,T42,T4	chr18	7.8E+07	8E+07	999	+	**6**	**75**	NA	3' UTR (NM_178670, exon 5 of 5)	3' UTR (NM_178670, exon 5 of 5)	-68864	NM_001164504	225743	Mm.23293	NM_001164504	ENSMUSG00000025427	**Rnf165**	2900024M11Rik|AI427432|Akd2|Ark2c|G630064H08Rik|Gm96	ring finger protein 165	LOSS	protein-coding
2009_T12,T1,T2,T32,T3,T4	chr18	7.8E+07	8E+07	2999	+	**6**	**75**	NA	Intergenic	Intergenic	-59864	NM_001164504	225743	Mm.23293	NM_001164504	ENSMUSG00000025427	**Rnf165**	2900024M11Rik|AI427432|Akd2|Ark2c|G630064H08Rik|Gm96	ring finger protein 165	LOSS	protein-coding

2010_T12,T2,T32,T3,T42,T4	chr18	7.8E+07	8E+07	999	+	**6**	**75**	NA	Intergenic	Intergenic	-61864	NM_001164504	225743	Mm.23293	NM_001164504	ENSMUSG00000025427	**Rnf165**	2900024M11Rik|AI427432|Akd2|Ark2c|G630064H08Rik|Gm96	ring finger protein 165	LOSS	protein-coding
553_T12,T22,T2,T32,T3,T4	chr14	5.2E+07	5E+07	999	+	**6**	**75**	NA	promoter-TSS (NM_001168515)	promoter-TSS (NM_001168515)	97	NM_023879	77945	Mm.21662	NM_023879	ENSMUSG00000057132	**Rpgrip1**	0610005A07Rik|4930401L23Rik|4930505G06Rik|A930002K18Rik|AA415034|nmf247	retinitis pigmentosa GTPase regulator interacting protein 1	LOSS	protein-coding
2084_T12,T1,T2,T32,T3,T42	chr18	8.1E+07	8E+07	16999	+	**6**	**75**	NA	intron (NM_178280, intron 1 of 3)	intron (NM_178280, intron 1 of 3)	1578	NM_178280	20689	Mm.215917	NM_178280	ENSMUSG00000024565	**Sall3**	B130022O04Rik|Msal|Msal-1|Sal|Salt|Spalt	sal-like 3 (Drosophila)	LOSS	protein-coding
1808_T12,T1,T2,T32,T3,T42	chr18	6.8E+07	7E+07	999	+	**6**	**75**	NA	intron (NM_028112, intron 2 of 7)	B2_Mm1t|SINE|B2	7124	NM_001039088	72124	Mm.307315	NM_028112	ENSMUSG00000079614	**Seh1l**	2610007A16Rik|AW540070|SEC13L|SEH1A|SEH1B|Seh1	SEH1-like (S. cerevisiae	LOSS	protein-coding
2046_T12,T1,T22,T2,T32,T42	chr18	7.9E+07	8E+07	3999	+	**6**	**75**	NA	Intergenic	MYSERV6-int|LTR|ERVK	-286109	NM_053099	240427	Mm.312871	NM_053099	ENSMUSG00000024548	**Setbp1**	C130092E12|Seb|mKIAA0437	SET binding protein 1	LOSS	protein-coding
2048_T12,T1,T22,T2,T32,T42	chr18	7.9E+07	8E+07	999	+	**6**	**75**	NA	Intergenic	Lx7|LINE|L1	-292609	NM_053099	240427	Mm.312871	NM_053099	ENSMUSG00000024548	**Setbp1**	C130092E12|Seb|mKIAA0437	SET binding protein 1	LOSS	protein-coding
2033_T12,T1,T22,T2,T32,T42	chr18	7.9E+07	8E+07	16999	+	**6**	**75**	NA	intron (NM_053099, intron 3 of 5)	intron (NM_053099, intron 3 of 5)	209391	NM_053099	240427	Mm.312871	NM_053099	ENSMUSG00000024548	**Setbp1**	C130092E12|Seb|mKIAA0437	SET binding protein 1	LOSS	protein-coding
2035_T12,T1,T22,T2,T32,T42	chr18	7.9E+07	8E+07	9999	+	**6**	**75**	NA	intron (NM_053099, intron 3 of 5)	(TGGA)n|Simple_repeat|Simple_repeat	194891	NM_053099	240427	Mm.312871	NM_053099	ENSMUSG00000024548	**Setbp1**	C130092E12|Seb|mKIAA0437	SET binding protein 1	LOSS	protein-coding
1648_T12,T1,T22,T2,T3,T42	chr18	5.8E+07	6E+07	14999	+	**6**	**75**	NA	Intergenic	Intergenic	-71678	NM_009194	20496	Mm.399997	NM_009194	ENSMUSG00000024597	**Slc12a2**	9330166H04Rik|Nkcc1|mBSC2|sy-ns	solute carrier family 12, member 2	LOSS	protein-coding
1650_T12,T1,T22,T32,T3,T42	chr18	5.8E+07	6E+07	999	+	**6**	**75**	NA	Intergenic	Intergenic	-52678	NM_009194	20496	Mm.399997	NM_009194	ENSMUSG00000024597	**Slc12a2**	9330166H04Rik|Nkcc1|mBSC2|sy-ns	solute carrier family 12, member 2	LOSS	protein-coding
1652_T12,T22,T2,T32,T3,T42	chr18	5.8E+07	6E+07	999	+	**6**	**75**	NA	Intergenic	Intergenic	-50678	NM_009194	20496	Mm.399997	NM_009194	ENSMUSG00000024597	**Slc12a2**	9330166H04Rik|Nkcc1|mBSC2|sy-ns	solute carrier family 12, member 2	LOSS	protein-coding
1654_T12,T1,T22,T32,T3,T42	chr18	5.8E+07	6E+07	999	+	**6**	**75**	NA	Intergenic	Lx7|LINE|L1	-4678	NM_009194	20496	Mm.399997	NM_009194	ENSMUSG00000024597	**Slc12a2**	9330166H04Rik|Nkcc1|mBSC2|sy-ns	solute carrier family 12, member 2	LOSS	protein-coding
1656_T12,T22,T2,T32,T3,T42	chr18	5.8E+07	6E+07	999	+	**6**	**75**	NA	intron (NM_009194, intron 1 of 26)	intron (NM_009194, intron 1 of 26)	3322	NM_009194	20496	Mm.399997	NM_009194	ENSMUSG00000024597	**Slc12a2**	9330166H04Rik|Nkcc1|mBSC2|sy-ns	solute carrier family 12, member 2	LOSS	protein-coding

<table>
<tr><td>589_T12,T22,T2,T3,T42,T4</td><td>chr14</td><td>5.5E+07</td><td>6E+07</td><td>430999</td><td>+</td><td>6</td><td>75</td><td>NA</td><td>Intergenic</td><td>Intergenic</td><td>9132</td><td>NM_021551</td><td>59049</td><td>Mm.27435</td><td>NM_021551</td><td>ENSMUSG00000022199</td><td>Slc22a17</td><td>1700094C23Rik|24p3R|AU041908|AW555662|BOIT|Boct</td><td>solute carrier family 22 (organic cation transporter), member 17</td><td>LOSS</td><td>protein-coding</td></tr>
<tr><td>245_T22,T2,T32,T3,T42,T4</td><td>chr14</td><td>2.6E+07</td><td>3E+07</td><td>290999</td><td>+</td><td>6</td><td>75</td><td>NA</td><td>Intergenic</td><td>ID_B1|SINE|B4</td><td>-6376</td><td>NM_001310445</td><td>83997</td><td>Mm.36769</td><td>NM_032008</td><td>ENSMUSG00000021870</td><td>Slmap</td><td>D330001L02Rik|Miranda|Slap|mKIAA1601</td><td>sarcolemma associated protein</td><td>LOSS</td><td>protein-coding</td></tr>
<tr><td>1971_T12,T1,T22,T2,T32,T3</td><td>chr18</td><td>7.6E+07</td><td>8E+07</td><td>55999</td><td>+</td><td>6</td><td>75</td><td>NA</td><td>Intergenic</td><td>MER58B|DNA|hAT-Charlie</td><td>-29201</td><td>NM_010754</td><td>17126</td><td>Mm.152699</td><td>NM_010754</td><td>ENSMUSG00000024563</td><td>Smad2</td><td>7120426M23Rik|Madh2|Madr2|Smad-2|mMad2</td><td>SMAD family member 2</td><td>LOSS</td><td>protein-coding</td></tr>
<tr><td>1977_T12,T1,T22,T2,T32,T3</td><td>chr18</td><td>7.6E+07</td><td>8E+07</td><td>63999</td><td>+</td><td>6</td><td>75</td><td>NA</td><td>Intergenic</td><td>Intergenic</td><td>63055</td><td>NM_001311070</td><td>17126</td><td>Mm.152699</td><td>NM_010754</td><td>ENSMUSG00000024563</td><td>Smad2</td><td>7120426M23Rik|Madh2|Madr2|Smad-2|mMad2</td><td>SMAD family member 2</td><td>LOSS</td><td>protein-coding</td></tr>
<tr><td>1973_T12,T1,T22,T2,T32,T3</td><td>chr18</td><td>7.6E+07</td><td>8E+07</td><td>43999</td><td>+</td><td>6</td><td>75</td><td>NA</td><td>intron (NM_001311070, intron 1 of 8)</td><td>Lx8|LINE|L1</td><td>3055</td><td>NM_001311070</td><td>17126</td><td>Mm.152699</td><td>NM_010754</td><td>ENSMUSG00000024563</td><td>Smad2</td><td>7120426M23Rik|Madh2|Madr2|Smad-2|mMad2</td><td>SMAD family member 2</td><td>LOSS</td><td>protein-coding</td></tr>
<tr><td>1975_T12,T1,T22,T2,T32,T3</td><td>chr18</td><td>7.6E+07</td><td>8E+07</td><td>3999</td><td>+</td><td>6</td><td>75</td><td>NA</td><td>intron (NM_010754, intron 5 of 10)</td><td>intron (NM_010754, intron 5 of 10)</td><td>28055</td><td>NM_001311070</td><td>17126</td><td>Mm.152699</td><td>NM_010754</td><td>ENSMUSG00000024563</td><td>Smad2</td><td>7120426M23Rik|Madh2|Madr2|Smad-2|mMad2</td><td>SMAD family member 2</td><td>LOSS</td><td>protein-coding</td></tr>
<tr><td>1572_T12,T1,T22,T2,T32,T42</td><td>chr18</td><td>5.3E+07</td><td>5E+07</td><td>999</td><td>+</td><td>6</td><td>75</td><td>NA</td><td>Intergenic</td><td>Intergenic</td><td>-14365</td><td>NM_026386</td><td>67804</td><td>Mm.252171</td><td>NM_026386</td><td>ENSMUSG00000034484</td><td>Snx2</td><td>0610030A03Rik</td><td>sorting nexin 2</td><td>LOSS</td><td>protein-coding</td></tr>
<tr><td>1574_T1,T22,T2,T32,T3,T42</td><td>chr18</td><td>5.3E+07</td><td>5E+07</td><td>999</td><td>+</td><td>6</td><td>75</td><td>NA</td><td>Intergenic</td><td>L1Md_F3|LINE|L1</td><td>-8365</td><td>NM_026386</td><td>67804</td><td>Mm.252171</td><td>NM_026386</td><td>ENSMUSG00000034484</td><td>Snx2</td><td>0610030A03Rik</td><td>sorting nexin 2</td><td>LOSS</td><td>protein-coding</td></tr>
<tr><td>1576_T12,T1,T22,T2,T3,T42</td><td>chr18</td><td>5.3E+07</td><td>5E+07</td><td>2999</td><td>+</td><td>6</td><td>75</td><td>NA</td><td>intron (NM_026386, intron 1 of 14)</td><td>L1M2|LINE|L1</td><td>5635</td><td>NM_026386</td><td>67804</td><td>Mm.252171</td><td>NM_026386</td><td>ENSMUSG00000034484</td><td>Snx2</td><td>0610030A03Rik</td><td>sorting nexin 2</td><td>LOSS</td><td>protein-coding</td></tr>
<tr><td>1579_T12,T22,T2,T32,T3,T42</td><td>chr18</td><td>5.3E+07</td><td>5E+07</td><td>999</td><td>+</td><td>6</td><td>75</td><td>NA</td><td>intron (NM_029394, intron 1 of 6)</td><td>intron (NM_029394, intron 1 of 6)</td><td>7338</td><td>NM_029394</td><td>69226</td><td>Mm.32414</td><td>NM_029394</td><td>ENSMUSG00000024535</td><td>Snx24</td><td>2810011K15Rik|5730433I16Rik|AI451341</td><td>sorting nexing 24</td><td>LOSS</td><td>protein-coding</td></tr>
<tr><td>2201_T12,T1,T22,T2,T3,T4</td><td>chr18</td><td>8.8E+07</td><td>9E+07</td><td>999</td><td>+</td><td>6</td><td>75</td><td>NA</td><td>Intergenic</td><td>Intergenic</td><td>1069207</td><td>NM_018821</td><td>54607</td><td>Mm.91920</td><td>NM_018821</td><td>ENSMUSG00000056153</td><td>Socs6</td><td>1500012M23Rik|5830401B18Rik|AI447482|Cis4|Cish4|HSPC060|SOCS-4|SOCS-6|SSI4|STAI4|STATI4|Socs4</td><td>suppressor of cytokine signaling 6</td><td>LOSS</td><td>protein-coding</td></tr>
<tr><td>2391_T1,T22,T32,T3,T42,T4</td><td>chr6</td><td>1.2E+08</td><td>1E+08</td><td>3999</td><td>+</td><td>6</td><td>75</td><td>NA</td><td>Intergenic</td><td>MMETn-int|LTR|ERVK</td><td>-9161</td><td>NM_001306051</td><td>14794</td><td>Mm.261906</td><td>NM_013539</td><td>ENSMUSG00000038451</td><td>Spsb2</td><td>AI461677|C9|Grcc9|SSB2</td><td>splA/ryanodine receptor domain and SOCS box containing 2</td><td>LOSS</td><td>protein-coding</td></tr>
</table>

463_T12,T22,T2,T3,T42,T4	chr14	4.5E+07	5E+07	21999	+	**6**	**75**	NA	intron (NM_019637, intron 4 of 10)	L1_Mus3|LINE|L1	9314	NM_019637	56291	Mm.202561	NM_019637	ENSMUSG00000053205	**Styx**	0610039A20Rik|hStyxb	serine/threonine/tyrosine interaction protein	LOSS	protein-coding
461_T12,T22,T2,T3,T42,T4	chr14	4.5E+07	5E+07	7999	+	**6**	**75**	NA	intron (NM_025959, intron 12 of 13)	MER58A|DNA|hAT-Charlie	-6686	NM_019637	56291	Mm.202561	NM_019637	ENSMUSG00000053205	**Styx**	0610039A20Rik|hStyxb	serine/threonine/tyrosine interaction protein	LOSS	protein-coding
1839_T12,T1,T22,T2,T32,T3	chr18	6.9E+07	7E+07	21999	+	**6**	**75**	NA	Intergenic	Intergenic	-9992	NM_013685	21413	Mm.4269	NM_013685	ENSMUSG00000053477	**Tcf4**	5730422P05Rik|ASP-I2|E2-2|E2.2|ITF-2|ITF-2b|ITF2|ME2|MITF-2A|MITF-2B|SEF-2|SEF2|SEF2-1|TFE|Tcf-4|bHLHb19	transcription factor 4	LOSS	protein-coding
718_T12,T1,T22,T2,T3,T42	chr18	9950501	1E+07	999	+	**6**	**75**	NA	Intergenic	Tigger1|DNA|TcMar-Tigger	-7180	NM_153552	225160	Mm.219648	NM_153552	ENSMUSG00000024287	**Thoc1**	3110002N20Rik|AW107452|NMP-84	THO complex 1	LOSS	protein-coding
719_T12,T1,T22,T32,T3,T42	chr18	9951501	1E+07	999	+	**6**	**75**	NA	Intergenic	Intergenic	-6180	NM_153552	225160	Mm.219648	NM_153552	ENSMUSG00000024287	**Thoc1**	3110002N20Rik|AW107452|NMP-84	THO complex 1	LOSS	protein-coding
148_T1,T22,T2,T3,T42,T4	chr14	1.4E+07	1E+07	96999	+	**6**	**75**	NA	intron (NM_025435, intron 1 of 7)	intron (NM_025435, intron 1 of 7)	5313	NM_001285780	66231	Mm.295875	NM_025435	ENSMUSG00000053453	**Thoc7**	Nif3l1bp1	THO complex 7	LOSS	protein-coding
2144_T12,T1,T22,T2,T32,T3	chr18	8.5E+07	9E+07	44999	+	**6**	**75**	NA	Intergenic	Lx7|LINE|L1	-396476	NM_025969	67105	Mm.250617	NM_025969	ENSMUSG00000024645	**Timm21**	1700034H14Rik|2700002I20Rik	tranlocase of inner mitochondrial membrane 21	LOSS	protein-coding
2145_T1,T22,T2,T32,T3,T42	chr18	8.5E+07	9E+07	999	+	**6**	**75**	NA	Intergenic	Intergenic	-419476	NM_025969	67105	Mm.250617	NM_025969	ENSMUSG00000024645	**Timm21**	1700034H14Rik|2700002I20Rik	tranlocase of inner mitochondrial membrane 21	LOSS	protein-coding
1225_T12,T1,T22,T2,T32,T3	chr18	3.7E+07	4E+07	217999	+	**6**	**75**	NA	intron (NM_028036, intron 9 of 11)	intron (NM_028036, intron 9 of 11)	4430	NM_028036	71983	Mm.135188	NM_028036	ENSMUSG00000006850	**Tmco6**	2410015B03Rik	transmembrane and coiled-coil domains 6	LOSS	protein-coding
1416_T12,T1,T22,T2,T3,T42	chr18	4.7E+07	5E+07	999	+	**6**	**75**	NA	intron (NM_025698, intron 1 of 2)	intron (NM_025698, intron 1 of 2)	2535	NM_025698	66676	Mm.296043	NM_025698	ENSMUSG00000033184	**Tmed7**	3930401E15Rik|5830493P14Rik|AU044611|AW545765|TRAM	transmembrane p24 trafficking protein 7	LOSS	protein-coding
492_T12,T22,T2,T3,T42,T4	chr14	4.8E+07	5E+07	162999	+	**6**	**75**	NA	Intergenic	B3|SINE|B2	104648	NR_132324	218989	Mm.85211	NM_172600	ENSMUSG00000036339	**Tmem260**	5930435P15|6720456H20Rik	transmembrane protein 260	LOSS	protein-coding
2274_T12,T1,T22,T2,T32,T3	chr18	9E+07	9E+07	4999	+	**6**	**75**	NA	Intergenic	L1_Mus2|LINE|L1	-336154	NM_198295	67988	Mm.268041	NM_198295	ENSMUSG00000024614	**Tmx3**	6430411B10Rik|A730024F05Rik|AV259382|Txndc10|mKIAA1830	thioredoxin-related transmembrane protein 3	LOSS	protein-coding

2276_T12,T1,T22,T2,T32,T3	chr18	9E+07	9E+07	3999	+	**6**	**75**	NA	Intergenic	L1_Mus2|LINE|L1	-330654	NM_198295	67988	Mm.268041	NM_198295	ENSMUSG00000024614	**Tmx3**	6430411B10Rik|A730024F05Rik|AV259382|Txndc10|mKIAA1830	thioredoxin-related transmembrane protein 3	LOSS	protein-coding
2278_T12,T1,T22,T2,T32,T3	chr18	9E+07	9E+07	73999	+	**6**	**75**	NA	Intergenic	Lx2A1|LINE|L1	-276654	NM_198295	67988	Mm.268041	NM_198295	ENSMUSG00000024614	**Tmx3**	6430411B10Rik|A730024F05Rik|AV259382|Txndc10|mKIAA1830	thioredoxin-related transmembrane protein 3	LOSS	protein-coding
2283_T12,T1,T22,T2,T32,T3	chr18	9E+07	9E+07	19999	+	**6**	**75**	NA	Intergenic	B3A|SINE|B2	-58654	NM_198295	67988	Mm.268041	NM_198295	ENSMUSG00000024614	**Tmx3**	6430411B10Rik|A730024F05Rik|AV259382|Txndc10|mKIAA1830	thioredoxin-related transmembrane protein 3	LOSS	protein-coding
2286_T12,T1,T22,T2,T3,T42	chr18	9E+07	9E+07	8999	+	**6**	**75**	NA	Intergenic	L1Md_F3|LINE|L1	-35154	NM_198295	67988	Mm.268041	NM_198295	ENSMUSG00000024614	**Tmx3**	6430411B10Rik|A730024F05Rik|AV259382|Txndc10|mKIAA1830	thioredoxin-related transmembrane protein 3	LOSS	protein-coding
1491_T12,T1,T22,T2,T32,T3	chr18	5E+07	5E+07	516999	+	**6**	**75**	NA	intron (NM_001081371, intron 38 of 42)	intron (NM_001081371, intron 38 of 42)	-26427	NM_001177760	106869	Mm.27740	NM_134131	ENSMUSG00000062210	**Tnfaip8**	AA987150|E130304C20Rik|ENSMUSG00000073567|Gg2-1|Gm10539|Nded|Ssc-2|Tipe	tumor necrosis factor, alpha-induced protein 8	LOSS	protein-coding
549_T12,T22,T2,T3,T42,T4	chr14	5.2E+07	5E+07	69999	+	**6**	**75**	NA	intron (NM_001128634, intron 2 of 2)	intron (NM_001128634, intron 2 of 2)	739	NM_001128634	219038	Mm.296425	NM_001128634	ENSMUSG00000008813	**Tppp2**	Gm1790|Gm77	tubulin polymerization-promoting protein family member 2	LOSS	protein-coding
1760_T12,T1,T2,T32,T42,T4	chr18	6.4E+07	6E+07	999	+	**6**	**75**	NA	intron (NM_016792, intron 1 of 7)	L1_Mus1|LINE|L1	4359	NM_016792	53382	Mm.19169	NM_016792	ENSMUSG00000024583	**Txnl1**	32kDa|TRP32|Txnl	thioredoxin-like 1	LOSS	protein-coding
1754_T12,T1,T22,T32,T3,T42	chr18	6.4E+07	6E+07	1999	+	**6**	**75**	NA	TTS (NM_016792)	TTS (NM_016792)	29859	NM_016792	53382	Mm.19169	NM_016792	ENSMUSG00000024583	**Txnl1**	32kDa|TRP32|Txnl	thioredoxin-like 1	LOSS	protein-coding
208_T22,T2,T32,T3,T42,T4	chr14	2.1E+07	2E+07	69999	+	**6**	**75**	NA	intron (NM_030180, intron 2 of 20)	B3|SINE|B2	11854	NM_030180	78787	Mm.301173	NM_030180	ENSMUSG00000034235	**Usp54**	4930429G18Rik|AI115571|C030002J06Rik	ubiquitin specific peptidase 54	LOSS	protein-coding
681_T12,T1,T22,T2,T32,T3	chr18	7790501	8E+06	59999	+	**6**	**75**	NA	Intergenic	L1_Mus3|LINE|L1	-48332	NM_001282093	225131	Mm.204524	NM_153085	ENSMUSG00000024283	**Wac**	1110067P07Rik|A230035H12Rik|AI256735|AI256776|Wwp4	WW domain containing adaptor with coiled-coil	LOSS	protein-coding
686_T12,T1,T22,T2,T32,T3	chr18	7857501	8E+06	91999	+	**6**	**75**	NA	intron (NM_153085, intron 4 of 13)	B1F2|SINE|Alu	34303	NM_153085	225131	Mm.204524	NM_153085	ENSMUSG00000024283	**Wac**	1110067P07Rik|A230035H12Rik|AI256735|AI256776|Wwp4	WW domain containing adaptor with coiled-coil	LOSS	protein-coding
320_T12,T22,T2,T32,T42,T4	chr14	3.5E+07	3E+07	4999	+	**6**	**75**	NA	intron (NM_001004436, intron 1 of 18)	intron (NM_001004436, intron 1 of 18)	1072	NM_001301330	218914	Mm.27957	NM_001004436	ENSMUSG00000041408	**Wapl**	A530089A20Rik|BC037674|DIF-2|FOE|Wapal	WAPL cohesin release factor	LOSS	protein-coding

323_T12,T22,T2,T32,T3,T4	chr14	3.5E+07	3E+07	1999	+	**6**	**75**	NA	intron (NM_001004436, intron 8 of 18)	intron (NM_001004436, intron 8 of 18)	54572	NM_001301330	218914	Mm.27957	NM_001004436	ENSMUSG00000041408	**Wapl**	A530089A20Rik|BC037674|DIF-2|FOE|Wapal	WAPL cohesin release factor	LOSS	protein-coding
266_T1,T22,T2,T32,T42,T4	chr14	2.9E+07	3E+07	999	+	**6**	**75**	NA	intron (NM_001256224, intron 4 of 4)	MIR3|SINE|MIR	8595	NM_001256224	22418	Mm.287544	NM_009524	ENSMUSG00000021994	**Wnt5a**	8030457G12Rik|Wnt-5a	wingless-type MMTV integration site family, member 5A	LOSS	protein-coding
264_T1,T22,T2,T3,T42,T4	chr14	2.9E+07	3E+07	1999	+	**6**	**75**	NA	promoter-TSS (NM_001256224)	promoter-TSS (NM_001256224)	-905	NM_001256224	22418	Mm.287544	NM_009524	ENSMUSG00000021994	**Wnt5a**	8030457G12Rik|Wnt-5a	wingless-type MMTV integration site family, member 5A	LOSS	protein-coding
1374_T12,T1,T22,T2,T32,T42	chr18	4.5E+07	4E+07	999	+	**6**	**75**	NA	Intergenic	CT-rich|Low_complexity|Low_complexity	-1665	NM_001163013	240255	Mm.244482	NM_001163013	ENSMUSG00000034653	**Ythdc2**	3010002F02Rik|BC037178	YTH domain containing 2	LOSS	protein-coding
1381_T12,T1,T22,T32,T3,T4	chr18	4.5E+07	4E+07	999	+	**6**	**75**	NA	Intergenic	ORR1A2|LTR|ERVL-MaLR	158335	NM_001163013	240255	Mm.244482	NM_001163013	ENSMUSG00000034653	**Ythdc2**	3010002F02Rik|BC037178	YTH domain containing 2	LOSS	protein-coding
1375_T12,T1,T22,T32,T3,T42	chr18	4.5E+07	4E+07	999	+	**6**	**75**	NA	promoter-TSS (NM_001163013)	promoter-TSS (NM_001163013)	-665	NM_001163013	240255	Mm.244482	NM_001163013	ENSMUSG00000034653	**Ythdc2**	3010002F02Rik|BC037178	YTH domain containing 2	LOSS	protein-coding
580_T12,T22,T2,T3,T4	chr14	5.4E+07	5E+07	51999	+	**5**	**62.5**	NA	intron (NM_134076, intron 5 of 6)	intron (NM_134076, intron 5 of 6)	7268	NM_134076	105501	Mm.28771	NM_134076	ENSMUSG00000040997	**Abhd4**	1110035H23Rik|AI429574|Abh4	abhydrolase domain containing 4	LOSS	protein-coding
1961_T12,T1,T2,T32,T3	chr18	7.5E+07	7E+07	3999	+	**5**	**62.5**	NA	intron (NM_177470, intron 1 of 9)	intron (NM_177470, intron 1 of 9)	288	NM_177470	52538	Mm.245724	NM_177470	ENSMUSG00000036880	**Acaa2**	0610011L04Rik|AI255831|AI265397|D18Ertd240e	acetyl-Coenzyme A acyltransferase 2 (mitochondrial 3-oxoacyl-Coenzyme A thiolase)	LOSS	protein-coding
588_T12,T22,T2,T42,T4	chr14	5.5E+07	5E+07	999	+	**5**	**62.5**	NA	intron (NM_021437, intron 1 of 1)	intron (NM_021437, intron 1 of 1)	-1069	NM_023190	56215	Mm.297078	NM_019567	ENSMUSG00000022185	**Acin1**	2610036I19Rik|2610510L13Rik|Acinus|Acn|C79325|acinusL|acinusS|mKIAA0670	apoptotic chromatin condensation inducer 1	LOSS	protein-coding
1672_T12,T1,T22,T3,T42	chr18	5.9E+07	6E+07	999	+	**5**	**62.5**	NA	Intergenic	L1Md_T|LINE|L1	-26764	NM_175506	240322	Mm.71963	NM_175506	ENSMUSG00000053441	**Adamts19**	4831407I23Rik|AU015154|D230034E10Rik	a disintegrin-like and metallopeptidase (reprolysin type) with thrombospondin type 1 motif, 19	LOSS	protein-coding
1675_T12,T22,T2,T32,T3	chr18	5.9E+07	6E+07	999	+	**5**	**62.5**	NA	intron (NM_175506, intron 2 of 22)	L1Md_A|LINE|L1	22236	NM_175506	240322	Mm.71963	NM_175506	ENSMUSG00000053441	**Adamts19**	4831407I23Rik|AU015154|D230034E10Rik	a disintegrin-like and metallopeptidase (reprolysin type) with thrombospondin type 1 motif, 19	LOSS	protein-coding

1677_T12,T1,T2,T32,T3	chr18	5.9E+07	6E+07	999	+	**5**	**62.5**	NA	intron (NM_175506, intron 6 of 22)	L1Md_T|LINE|L1	86236	NM_175506	240322	Mm.71963	NM_175506	ENSMUSG00000053441	**Adamts19**	4831407I23Rik|AU015154|D230034E10Rik	a disintegrin-like and metallopeptidase (reprolysin type) with thrombospondin type 1 motif, 19	LOSS	protein-coding
1219_T12,T22,T32,T3,T4	chr18	3.7E+07	4E+07	999	+	**5**	**62.5**	NA	intron (NM_175375, intron 1 of 33)	intron (NM_175375, intron 1 of 33)	3397	NM_175375	108857	Mm.482287	NM_175375	ENSMUSG00000024483	**Ankhd1**	1110004O12Rik|4933432B13Rik|9130019P20Rik|A530027J04Rik|A630021B20Rik|AA571404|Mask|mFLJ00246|mKIAA1085	ankyrin repeat and KH domain containing 1	LOSS	protein-coding
288_T22,T2,T3,T42,T4	chr14	3.2E+07	3E+07	16999	+	**5**	**62.5**	NA	intron (NM_001024604, intron 1 of 27)	intron (NM_001024604, intron 1 of 27)	12415	NM_001024604	105522	Mm.37660	NM_001024604	ENSMUSG00000014496	**Ankrd28**	AI465466|AI604979|E430019N21Rik|mKIAA0379	ankyrin repeat domain 28	LOSS	protein-coding
286_T22,T2,T3,T42,T4	chr14	3.2E+07	3E+07	61999	+	**5**	**62.5**	NA	intron (NM_001024604, intron 2 of 27)	intron (NM_001024604, intron 2 of 27)	52915	NM_001024604	105522	Mm.37660	NM_001024604	ENSMUSG00000014496	**Ankrd28**	AI465466|AI604979|E430019N21Rik|mKIAA0379	ankyrin repeat domain 28	LOSS	protein-coding
284_T22,T2,T3,T42,T4	chr14	3.2E+07	3E+07	14999	+	**5**	**62.5**	NA	intron (NM_001024604, intron 8 of 27)	RSINE1|SINE|B4	92415	NM_001024604	105522	Mm.37660	NM_001024604	ENSMUSG00000014496	**Ankrd28**	AI465466|AI604979|E430019N21Rik|mKIAA0379	ankyrin repeat domain 28	LOSS	protein-coding
213_T22,T2,T32,T3,T42	chr14	2.1E+07	2E+07	3999	+	**5**	**62.5**	NA	intron (NM_018829, intron 3 of 8)	intron (NM_018829, intron 3 of 8)	10942	NM_018829	55946	Mm.376269	NM_018829	ENSMUSG00000021824	**Ap3m1**	1200013D09Rik|C78982|R75378	adaptor-related protein complex 3, mu 1 subunit	LOSS	protein-coding
256_T2,T32,T3,T42,T4	chr14	2.7E+07	3E+07	77999	+	**5**	**62.5**	NA	intron (NM_001289686, intron 4 of 12)	intron (NM_001289686, intron 4 of 12)	-16537	NM_001289687	71704	Mm.248606	NM_027871	ENSMUSG00000021895	**Arhgef3**	1200004I24Rik|9830169H03Rik|C76747|Xpln	Rho guanine nucleotide exchange factor (GEF) 3	LOSS	protein-coding
258_T22,T32,T3,T42,T4	chr14	2.7E+07	3E+07	999	+	**5**	**62.5**	NA	intron (NM_001289688, intron 2 of 9)	ORR1E|LTR|ERVL-MaLR	11611	NM_001289688	71704	Mm.248606	NM_027871	ENSMUSG00000021895	**Arhgef3**	1200004I24Rik|9830169H03Rik|C76747|Xpln	Rho guanine nucleotide exchange factor (GEF) 3	LOSS	protein-coding
1721_T12,T1,T2,T3,T42	chr18	6.2E+07	6E+07	999	+	**5**	**62.5**	NA	Intergenic	ERVL-B4-int|LTR|ERVL	-5464	NM_177828	328967	Mm.488098	NM_177828	ENSMUSG00000045094	**Arhgef37**	4933429F08Rik	Rho guanine nucleotide exchange factor (GEF) 37	LOSS	protein-coding
1718_T12,T1,T2,T32,T3	chr18	6.2E+07	6E+07	5999	+	**5**	**62.5**	NA	intron (NM_177828, intron 4 of 12)	PB1D10|SINE|Alu	21036	NM_177828	328967	Mm.488098	NM_177828	ENSMUSG00000045094	**Arhgef37**	4933429F08Rik	Rho guanine nucleotide exchange factor (GEF) 37	LOSS	protein-coding
956_T1,T2,T32,T3,T42	chr18	2.2E+07	2E+07	999	+	**5**	**62.5**	NA	Intergenic	Intergenic	-53089	NM_001167777	211961	Mm.392310	NM_001167777	ENSMUSG00000045215	**Asxl3**	C230079D11Rik|D430002O22Rik|D930044O18Rik|Gm329|Gm945	additional sex combs like 3 (Drosophila)	LOSS	protein-coding

959_T12,T1,T2,T32,T3	chr18	2.2E+07	2E+07	999	+	**5**	**62.5**	NA	intron (NM_001167777, intron 2 of 12)	intron (NM_001167777, intron 2 of 12)	35911	NM_001167777	211961	Mm.392310	NM_001167777	ENSMUSG00000045215	**Asxl3**	C230079D11Rik|D430002O22Rik|D930044O18Rik|Gm329|Gm945	additional sex combs like 3 (Drosophila)	LOSS	protein-coding
2083_T12,T1,T2,T32,T42	chr18	8.1E+07	8E+07	42999	+	**5**	**62.5**	NA	Intergenic	Intergenic	-20942	NM_015805	50771	Mm.247138	NM_015805	ENSMUSG00000024566	**Atp9b**	AA934181|Atpc2b|IIb|MMR	ATPase, class II, type 9B	LOSS	protein-coding
2081_T12,T1,T2,T3,T42	chr18	8.1E+07	8E+07	999	+	**5**	**62.5**	NA	intron (NM_001201569, intron 2 of 29)	intron (NM_001201569, intron 2 of 29)	19058	NM_001201569	50771	Mm.247138	NM_015805	ENSMUSG00000024566	**Atp9b**	AA934181|Atpc2b|IIb|MMR	ATPase, class II, type 9B	LOSS	protein-coding
2077_T12,T1,T2,T32,T42	chr18	8.1E+07	8E+07	3999	+	**5**	**62.5**	NA	intron (NM_001201569, intron 4 of 29)	intron (NM_001201569, intron 4 of 29)	36558	NM_001201569	50771	Mm.247138	NM_015805	ENSMUSG00000024566	**Atp9b**	AA934181|Atpc2b|IIb|MMR	ATPase, class II, type 9B	LOSS	protein-coding
2079_T12,T1,T2,T3,T42	chr18	8.1E+07	8E+07	999	+	**5**	**62.5**	NA	intron (NM_001201569, intron 4 of 29)	Lx5|LINE|L1	33058	NM_001201569	50771	Mm.247138	NM_015805	ENSMUSG00000024566	**Atp9b**	AA934181|Atpc2b|IIb|MMR	ATPase, class II, type 9B	LOSS	protein-coding
149_T1,T22,T2,T42,T4	chr14	1.4E+07	1E+07	999	+	**5**	**62.5**	NA	Intergenic	Intergenic	-7491	NM_139227	246103	Mm.133625	NM_139227	ENSMUSG00000021738	**Atxn7**	A430107N12Rik|AI627028|Sca7|ataxin-7	ataxin 7	LOSS	protein-coding
153_T22,T2,T3,T42,T4	chr14	1.4E+07	1E+07	999	+	**5**	**62.5**	NA	intron (NM_139227, intron 3 of 11)	intron (NM_139227, intron 3 of 11)	1509	NM_139227	246103	Mm.133625	NM_139227	ENSMUSG00000021738	**Atxn7**	A430107N12Rik|AI627028|Sca7|ataxin-7	ataxin 7	LOSS	protein-coding
954_T12,T1,T2,T3,T42	chr18	2.2E+07	2E+07	999	+	**5**	**62.5**	NA	intron (NM_027616, intron 2 of 22)	intron (NM_027616, intron 2 of 22)	20396	NM_027616	70950	Mm.235716	NM_027616	ENSMUSG00000024306	**Ccdc178**	4921528I01Rik	coiled coil domain containing 178	LOSS	protein-coding
1844_T12,T1,T22,T32,T3	chr18	7E+07	7E+07	3999	+	**5**	**62.5**	NA	Intergenic	L1ME1|LINE|L1	50941	NM_201362	381175	Mm.266831	NM_201362	ENSMUSG00000038903	**Ccdc68**	BC046343	coiled-coil domain containing 68	LOSS	protein-coding
1594_T12,T1,T22,T2,T3	chr18	5.4E+07	5E+07	53999	+	**5**	**62.5**	NA	exon (NM_178686, exon 8 of 21)	exon (NM_178686, exon 8 of 21)	20047	NM_178686	225523	Mm.193678	NM_178686	ENSMUSG00000048799	**Cep120**	A230075C01|AU016693|Ccdc100	centrosomal protein 120	LOSS	protein-coding
805_T12,T1,T2,T32,T3	chr18	1.6E+07	2E+07	113999	+	**5**	**62.5**	NA	Intergenic	MLT1A0|LTR|ERVL-MaLR	-131454	NM_199055	71367	Mm.329304	NM_199055	ENSMUSG00000047161	**Chst9**	5430438D01Rik	carbohydrate (N-acetylgalactosamine 4-0) sulfotransferase 9	LOSS	protein-coding
796_T12,T1,T2,T32,T4	chr18	1.6E+07	2E+07	4999	+	**5**	**62.5**	NA	intron (NM_199055, intron 1 of 3)	intron (NM_199055, intron 1 of 3)	66046	NM_199055	71367	Mm.329304	NM_199055	ENSMUSG00000047161	**Chst9**	5430438D01Rik	carbohydrate (N-acetylgalactosamine 4-0) sulfotransferase 9	LOSS	protein-coding

1723_T12,T1,T32,T3,T42	chr18	6.2E+07	6E+07	999	+	**5**	**62.5**	NA	intron (NM_146087, intron 2 of 9)	intron (NM_146087, intron 2 of 9)	5418	NM_146087	93687	Mm.26908	NM_146087	ENSMUSG00000024576	**Csnk1a1**	2610208K14Rik|4632404G05Rik|5430427P18Rik|CK1a|Csnk1a	casein kinase 1, alpha 1	LOSS	protein-coding
1724_T12,T1,T2,T3,T42	chr18	6.2E+07	6E+07	999	+	**5**	**62.5**	NA	intron (NM_146087, intron 2 of 9)	intron (NM_146087, intron 2 of 9)	6418	NM_146087	93687	Mm.26908	NM_146087	ENSMUSG00000024576	**Csnk1a1**	2610208K14Rik|4632404G05Rik|5430427P18Rik|CK1a|Csnk1a	casein kinase 1, alpha 1	LOSS	protein-coding
1725_T12,T1,T2,T32,T3	chr18	6.2E+07	6E+07	1999	+	**5**	**62.5**	NA	intron (NM_146087, intron 2 of 9)	PB1D10|SINE|Alu	7918	NM_146087	93687	Mm.26908	NM_146087	ENSMUSG00000024576	**Csnk1a1**	2610208K14Rik|4632404G05Rik|5430427P18Rik|CK1a|Csnk1a	casein kinase 1, alpha 1	LOSS	protein-coding
1597_T12,T1,T22,T2,T3	chr18	5.4E+07	5E+07	36999	+	**5**	**62.5**	NA	Intergenic	Intergenic	-48113	NM_152809	70425	Mm.368668	NM_152809	ENSMUSG00000073563	**Csnk1g3**	3300002K07Rik|C330049O21Rik	casein kinase 1, gamma 3	LOSS	protein-coding
1607_T12,T22,T2,T32,T3	chr18	5.4E+07	5E+07	2999	+	**5**	**62.5**	NA	Intergenic	Intergenic	147887	NM_152809	70425	Mm.368668	NM_152809	ENSMUSG00000073563	**Csnk1g3**	3300002K07Rik|C330049O21Rik	casein kinase 1, gamma 3	LOSS	protein-coding
1609_T12,T22,T2,T32,T3	chr18	5.4E+07	5E+07	8999	+	**5**	**62.5**	NA	Intergenic	Lx9|LINE|L1	184887	NM_152809	70425	Mm.368668	NM_152809	ENSMUSG00000073563	**Csnk1g3**	3300002K07Rik|C330049O21Rik	casein kinase 1, gamma 3	LOSS	protein-coding
1611_T12,T22,T2,T32,T3	chr18	5.4E+07	5E+07	5999	+	**5**	**62.5**	NA	Intergenic	Intergenic	200387	NM_152809	70425	Mm.368668	NM_152809	ENSMUSG00000073563	**Csnk1g3**	3300002K07Rik|C330049O21Rik	casein kinase 1, gamma 3	LOSS	protein-coding
1600_T12,T1,T22,T32,T3	chr18	5.4E+07	5E+07	1999	+	**5**	**62.5**	NA	intron (NM_152809, intron 1 of 12)	intron (NM_152809, intron 1 of 12)	5387	NM_152809	70425	Mm.368668	NM_152809	ENSMUSG00000073563	**Csnk1g3**	3300002K07Rik|C330049O21Rik	casein kinase 1, gamma 3	LOSS	protein-coding
578_T12,T22,T2,T3,T4	chr14	5.4E+07	5E+07	82999	+	**5**	**62.5**	NA	Intergenic	Intergenic	58929	NM_001113358	13135	Mm.319038	NM_010015	ENSMUSG00000022174	**Dad1**	AI323713	defender against cell death 1	LOSS	protein-coding
1920_T12,T1,T2,T32,T42	chr18	7.2E+07	7E+07	16999	+	**5**	**62.5**	NA	Intergenic	MusHAL1|LINE|L1	-69931	NM_007831	13176	Mm.167882	NM_007831	ENSMUSG00000060534	**Dcc**	C030036D22Rik|Igdcc1	deleted in colorectal carcinoma	LOSS	protein-coding
1918_T12,T1,T2,T32,T42	chr18	7.2E+07	7E+07	33999	+	**5**	**62.5**	NA	intron (NM_007831, intron 1 of 28)	L1MC3|LINE|L1	17569	NM_007831	13176	Mm.167882	NM_007831	ENSMUSG00000060534	**Dcc**	C030036D22Rik|Igdcc1	deleted in colorectal carcinoma	LOSS	protein-coding
1888_T12,T1,T2,T32,T42	chr18	7.1E+07	7E+07	28999	+	**5**	**62.5**	NA	intron (NM_007831, intron 12 of 28)	MLT1H2|LTR|ERVL-MaLR	837069	NM_007831	13176	Mm.167882	NM_007831	ENSMUSG00000060534	**Dcc**	C030036D22Rik|Igdcc1	deleted in colorectal carcinoma	LOSS	protein-coding
1911_T12,T1,T2,T32,T3	chr18	7.2E+07	7E+07	139999	+	**5**	**62.5**	NA	intron (NM_007831, intron 2 of 28)	intron (NM_007831, intron 2 of 28)	479569	NM_007831	13176	Mm.167882	NM_007831	ENSMUSG00000060534	**Dcc**	C030036D22Rik|Igdcc1	deleted in colorectal carcinoma	LOSS	protein-coding

1903_T1,T22,T2,T32,T42	chr18	7.2E+07	7E+07	3999	+	**5**	**62.5**	NA	intron (NM_007831, intron 5 of 28)	L1_Mus3|LINE|L1	616569	NM_007831	13176	Mm.167882	NM_007831	ENSMUSG00000060534	**Dcc**	C030036D22Rik|Igdcc1	deleted in colorectal carcinoma	LOSS	protein-coding
1904_T12,T1,T22,T32,T42	chr18	7.2E+07	7E+07	999	+	**5**	**62.5**	NA	intron (NM_007831, intron 5 of 28)	intron (NM_007831, intron 5 of 28)	614069	NM_007831	13176	Mm.167882	NM_007831	ENSMUSG00000060534	**Dcc**	C030036D22Rik|Igdcc1	deleted in colorectal carcinoma	LOSS	protein-coding
1906_T1,T22,T2,T32,T42	chr18	7.2E+07	7E+07	3999	+	**5**	**62.5**	NA	intron (NM_007831, intron 5 of 28)	intron (NM_007831, intron 5 of 28)	584569	NM_007831	13176	Mm.167882	NM_007831	ENSMUSG00000060534	**Dcc**	C030036D22Rik|Igdcc1	deleted in colorectal carcinoma	LOSS	protein-coding
1896_T12,T1,T22,T2,T3	chr18	7.2E+07	7E+07	28999	+	**5**	**62.5**	NA	intron (NM_007831, intron 7 of 28)	intron (NM_007831, intron 7 of 28)	698069	NM_007831	13176	Mm.167882	NM_007831	ENSMUSG00000060534	**Dcc**	C030036D22Rik|Igdcc1	deleted in colorectal carcinoma	LOSS	protein-coding
1899_T1,T22,T32,T3,T42	chr18	7.2E+07	7E+07	999	+	**5**	**62.5**	NA	intron (NM_007831, intron 7 of 28)	intron (NM_007831, intron 7 of 28)	674069	NM_007831	13176	Mm.167882	NM_007831	ENSMUSG00000060534	**Dcc**	C030036D22Rik|Igdcc1	deleted in colorectal carcinoma	LOSS	protein-coding
592_T12,T22,T2,T42,T4	chr14	5.5E+07	6E+07	64999	+	**5**	**62.5**	NA	Intergenic	Intergenic	-28007	NM_027790	71412	Mm.85484	NM_027790	ENSMUSG00000022209	**Dhrs2**	5430405K24Rik|Hep27	dehydrogenase/reductase member 2	LOSS	protein-coding
595_T12,T22,T2,T42,T4	chr14	5.5E+07	6E+07	198999	+	**5**	**62.5**	NA	Intergenic	Lx5c|LINE|L1	-59758	NM_001037938	28200	Mm.27427	NM_030686	ENSMUSG00000022210	**Dhrs4**	AI043103|AI790593|CR|D14Ucla2|NDRD|PHCR|PSCD|RRD|mouNRDR	dehydrogenase/reductase (SDR family) member 4	LOSS	protein-coding
2246_T12,T1,T22,T2,T32	chr18	9E+07	9E+07	20999	+	**5**	**62.5**	NA	Intergenic	Intergenic	-54864	NM_001039173	623279	Mm.447164	NM_001039173	ENSMUSG00000073514	**Dok6**	Dok-6	docking protein 6	LOSS	protein-coding
2250_T12,T1,T22,T2,T32	chr18	9E+07	9E+07	11999	+	**5**	**62.5**	NA	Intergenic	L1Md_A|LINE|L1	-89364	NM_001039173	623279	Mm.447164	NM_001039173	ENSMUSG00000073514	**Dok6**	Dok-6	docking protein 6	LOSS	protein-coding
2255_T12,T1,T22,T2,T32	chr18	9E+07	9E+07	22999	+	**5**	**62.5**	NA	Intergenic	Intergenic	-129864	NM_001039173	623279	Mm.447164	NM_001039173	ENSMUSG00000073514	**Dok6**	Dok-6	docking protein 6	LOSS	protein-coding
2260_T12,T22,T2,T32,T3	chr18	9E+07	9E+07	999	+	**5**	**62.5**	NA	Intergenic	Intergenic	-231864	NM_001039173	623279	Mm.447164	NM_001039173	ENSMUSG00000073514	**Dok6**	Dok-6	docking protein 6	LOSS	protein-coding
2262_T12,T22,T32,T3	chr18	9E+07	9E+07	999	+	**5**	**62.5**	NA	Intergenic	Intergenic	-249864	NM_001039173	623279	Mm.447164	NM_001039173	ENSMUSG00000073514	**Dok6**	Dok-6	docking protein 6	LOSS	protein-coding
2264_T12,T22,T2,T32,T3	chr18	9E+07	9E+07	999	+	**5**	**62.5**	NA	Intergenic	L1_Mus3|LINE|L1	-283864	NM_001039173	623279	Mm.447164	NM_001039173	ENSMUSG00000073514	**Dok6**	Dok-6	docking protein 6	LOSS	protein-coding

2244_T12,T1,T22,T2,T32	chr18	9E+07	9E+07	95999	+	**5**	**62.5**	NA	intron (NM_001039173, intron 1 of 7)	ORR1D1-int|LTR|ERVL-MaLR	9636	NM_001039173	623279	Mm.447164	NM_001039173	ENSMUSG00000073514	**Dok6**	Dok-6	docking protein 6	LOSS	protein-coding
877_T12,T1,T2,T32,T3	chr18	1.9E+07	2E+07	34999	+	**5**	**62.5**	NA	Intergenic	L1MEc|LINE|L1	1161097	NM_007882	13507	Mm.89935	NM_007882	ENSMUSG00000059898	**Dsc3**	5430426I24Rik	desmocollin 3	LOSS	protein-coding
881_T12,T1,T2,T32,T3	chr18	1.9E+07	2E+07	99999	+	**5**	**62.5**	NA	Intergenic	L1_Mus2|LINE|L1	1075597	NM_007882	13507	Mm.89935	NM_007882	ENSMUSG00000059898	**Dsc3**	5430426I24Rik	desmocollin 3	LOSS	protein-coding
883_T12,T1,T22,T2,T3	chr18	1.9E+07	2E+07	131999	+	**5**	**62.5**	NA	Intergenic	L1_Mus3|LINE|L1	898597	NM_007882	13507	Mm.89935	NM_007882	ENSMUSG00000059898	**Dsc3**	5430426I24Rik	desmocollin 3	LOSS	protein-coding
889_T12,T1,T2,T32,T3	chr18	1.9E+07	2E+07	4999	+	**5**	**62.5**	NA	Intergenic	Intergenic	776097	NM_007882	13507	Mm.89935	NM_007882	ENSMUSG00000059898	**Dsc3**	5430426I24Rik	desmocollin 3	LOSS	protein-coding
891_T12,T1,T2,T32,T3	chr18	1.9E+07	2E+07	52999	+	**5**	**62.5**	NA	Intergenic	Intergenic	746097	NM_007882	13507	Mm.89935	NM_007882	ENSMUSG00000059898	**Dsc3**	5430426I24Rik	desmocollin 3	LOSS	protein-coding
894_T12,T1,T22,T2,T3	chr18	1.9E+07	2E+07	999	+	**5**	**62.5**	NA	Intergenic	L1Md_F3|LINE|L1	657097	NM_007882	13507	Mm.89935	NM_007882	ENSMUSG00000059898	**Dsc3**	5430426I24Rik	desmocollin 3	LOSS	protein-coding
896_T12,T1,T22,T2,T32	chr18	1.9E+07	2E+07	75999	+	**5**	**62.5**	NA	Intergenic	L1_Mus2|LINE|L1	610597	NM_007882	13507	Mm.89935	NM_007882	ENSMUSG00000059898	**Dsc3**	5430426I24Rik	desmocollin 3	LOSS	protein-coding
908_T12,T1,T2,T32,T42	chr18	2E+07	2E+07	9999	+	**5**	**62.5**	NA	Intergenic	Lx|LINE|L1	416597	NM_007882	13507	Mm.89935	NM_007882	ENSMUSG00000059898	**Dsc3**	5430426I24Rik	desmocollin 3	LOSS	protein-coding
919_T12,T1,T2,T32,T42	chr18	2E+07	2E+07	8999	+	**5**	**62.5**	NA	Intergenic	B4|SINE|B4	120097	NM_007882	13507	Mm.89935	NM_007882	ENSMUSG00000059898	**Dsc3**	5430426I24Rik	desmocollin 3	LOSS	protein-coding
928_T12,T1,T2,T32,T42	chr18	2.1E+07	2E+07	1999	+	**5**	**62.5**	NA	intron (NM_030596, intron 1 of 15)	intron (NM_030596, intron 1 of 15)	1196	NM_030596	13512	Mm.106811	NM_030596	ENSMUSG00000056632	**Dsg3**	bal	desmoglein 3	LOSS	protein-coding
1963_T12,T1,T2,T32,T3	chr18	7.5E+07	8E+07	598999	+	**5**	**62.5**	NA	intron (NM_027727, intron 7 of 16)	intron (NM_027727, intron 7 of 16)	63228	NM_027727	69190	Mm.173826	NM_027727	ENSMUSG00000035765	**Dym**	1810041M12Rik|4933427L07Rik|C030019K18Rik	dymeclin	LOSS	protein-coding
2019_T12,T1,T32,T3,T42	chr18	7.8E+07	8E+07	999	+	**5**	**62.5**	NA	intron (NM_001195633, intron 23 of 43)	intron (NM_001195633, intron 23 of 43)	49533	NM_001195633	1E+08	Mm.297782	NM_001195633	ENSMUSG00000039840	**Epg5**	4732475F16|5430411K18Rik|AI661957|AW456499|mKIAA1632	ectopic P-granules autophagy protein 5 homolog (C. elegans)	LOSS	protein-coding
1159_T12,T1,T22,T32,T3	chr18	3.2E+07	3E+07	999	+	**5**	**62.5**	NA	exon (NM_011946, exon 11 of 17)	exon (NM_011946, exon 11 of 17)	-30331	NM_133658	13872	Mm.282335	NM_133658	ENSMUSG00000024382	**Ercc3**	BTF2 p89|Ercc-3|XPB	excision repair cross-complementing rodent repair deficiency, complementation group 3	LOSS	protein-coding

1161_T12,T1,T22,T2,T32	chr18	3.2E+07	3E+07	999	+	**5**	**62.5**	NA	Intergenic	MLTR14|LTR|ERV1	53669	NM_133658	13872	Mm.282335	NM_133658	ENSMUSG00000024382	**Ercc3**	BTF2 p89|Ercc-3|XPB	excision repair cross-complementing rodent repair deficiency, complementation group 3	LOSS	protein-coding
1496_T1,T22,T2,T32,T3	chr18	5E+07	5E+07	999	+	**5**	**62.5**	NA	Intergenic	MTD|LTR|ERVL-MaLR	-52369	NM_001004061	225497	Mm.329435	NM_001004061	ENSMUSG00000035420	**Fam170a**	Gm93|Znfd	family with sequence similarity 170, member A	LOSS	protein-coding
309_T12,T22,T2,T42,T4	chr14	3.4E+07	3E+07	20999	+	**5**	**62.5**	NA	intron (NM_029389, intron 2 of 10)	intron (NM_029389, intron 2 of 10)	1503	NM_029389	75698	Mm.45406	NM_029389	ENSMUSG00000041471	**Fam35a**	3110001K24Rik	family with sequence similarity 35, member A	LOSS	protein-coding
2135_T12,T1,T22,T2,T32	chr18	8.5E+07	8E+07	219999	+	**5**	**62.5**	NA	intron (NM_173770, intron 1 of 4)	intron (NM_173770, intron 1 of 4)	258	NM_173770	240479	Mm.472378	NM_173770	ENSMUSG00000047992	**Fam69c**	B230399E16Rik	family with sequence similarity 69, member C	LOSS	protein-coding
1660_T12,T1,T22,T2,T3	chr18	5.8E+07	6E+07	999	+	**5**	**62.5**	NA	Intergenic	L1Md_F3|LINE|L1	-50074	NM_010181	14119	Mm.20271	NM_010181	ENSMUSG00000024598	**Fbn2**	BC063774|Fib-2|mKIAA4226|sne|sy	fibrillin 2	LOSS	protein-coding
1662_T12,T1,T2,T3,T4	chr18	5.8E+07	6E+07	3999	+	**5**	**62.5**	NA	Intergenic	(CCCCCT)n|Simple_repeat|Simple_repeat	-70574	NM_010181	14119	Mm.20271	NM_010181	ENSMUSG00000024598	**Fbn2**	BC063774|Fib-2|mKIAA4226|sne|sy	fibrillin 2	LOSS	protein-coding
2137_T12,T1,T22,T2,T32	chr18	8.5E+07	8E+07	134999	+	**5**	**62.5**	NA	Intergenic	RMER15|LTR|ERVL	-34025	NM_015798	50764	Mm.28369	NM_015798	ENSMUSG00000034391	**Fbxo15**	AU019763|Fbx15|ecat3	F-box protein 15	LOSS	protein-coding
133_T22,T2,T3,T42,T4	chr14	1E+07	1E+07	999	+	**5**	**62.5**	NA	intron (NM_001308286, intron 2 of 6)	ORR1E-int|LTR|ERVL-MaLR	276458	NM_001308286	14198	Mm.397619	NM_010210	ENSMUSG00000060579	**Fhit**	AW045638|Fra14A2	fragile histidine triad gene	LOSS	protein-coding
945_T12,T1,T2,T32,T42	chr18	2.1E+07	2E+07	63999	+	**5**	**62.5**	NA	intron (NM_001033445, intron 2 of 5)	intron (NM_001033445, intron 2 of 5)	70639	NM_001033445	381126	Mm.312276	NM_001033445	ENSMUSG00000042680	**Garem1**	C86169|Fam59a|Garem|Gm944|mKIAA4238	GRB2 associated regulator of MAPK1 subtype 1	LOSS	protein-coding
730_T12,T1,T22,T2,T4	chr18	1.1E+07	1E+07	7999	+	**5**	**62.5**	NA	Intergenic	Lx7|LINE|L1	-27010	NM_010258	14465	Mm.329287	NM_010258	ENSMUSG00000005836	**Gata6**	AA410133|GATA-6	GATA binding protein 6	LOSS	protein-coding
732_T12,T1,T22,T2,T4	chr18	1.1E+07	1E+07	10999	+	**5**	**62.5**	NA	Intergenic	(CA)n|Simple_repeat|Simple_repeat	-16510	NM_010258	14465	Mm.329287	NM_010258	ENSMUSG00000005836	**Gata6**	AA410133|GATA-6	GATA binding protein 6	LOSS	protein-coding
355_T12,T2,T3,T42,T4	chr14	3.7E+07	4E+07	30999	+	**5**	**62.5**	NA	Intergenic	Intergenic	-77678	NM_078478	66092	Mm.182912	NM_078478	ENSMUSG00000041028	**Ghitm**	1010001P14Rik|C77840|MICS1|PTD010|Tmbim5	growth hormone inducible transmembrane protein	LOSS	protein-coding

465_T12,T22,T2,T42,T4	chr14	4.5E+07	5E+07	999	+	**5**	**62.5**	NA	intron (NM_019637, intron 10 of 10)	B2_Mm1t|SINE|B2	15796	NM_019425	54342	Mm.312945	NM_019425	ENSMUSG00000037722	**Gnpnat1**	AU017428|AU040593|EMeg32|Gpnat1|Gsnpat	glucosamine-phosphate N-acetyltransferase 1	LOSS	protein-coding
452_T12,T22,T2,T42,T4	chr14	4.5E+07	5E+07	2999	+	**5**	**62.5**	NA	intron (NM_027518, intron 1 of 6)	intron (NM_027518, intron 1 of 6)	16283	NM_027518	70713	Mm.343793	NM_027518	ENSMUSG00000049092	**Gpr137c**	6330416L11Rik|Gm908|TM7SF1L2	G protein-coupled receptor 137C	LOSS	protein-coding
454_T12,T22,T2,T3,T4	chr14	4.5E+07	5E+07	44999	+	**5**	**62.5**	NA	intron (NM_027518, intron 3 of 6)	B4|SINE|B4	42283	NM_027518	70713	Mm.343793	NM_027518	ENSMUSG00000049092	**Gpr137c**	6330416L11Rik|Gm908|TM7SF1L2	G protein-coupled receptor 137C	LOSS	protein-coding
1324_T12,T1,T22,T2,T42	chr18	4.2E+07	4E+07	3999	+	**5**	**62.5**	NA	Intergenic	Intergenic	22549	NM_001033426	332309	Mm.240520	NM_001033426	ENSMUSG00000073574	**Grxcr2**	Gm851	glutaredoxin, cysteine rich 2	LOSS	protein-coding
584_T12,T22,T2,T42,T4	chr14	5.5E+07	5E+07	2999	+	**5**	**62.5**	NA	Intergenic	B2_Mm1t|SINE|B2	14361	NM_145462	219072	Mm.202152	NM_145462	ENSMUSG00000022177	**Haus4**	9430093H08Rik|AU016300|D14Ertd500e	HAUS augmin-like complex, subunit 4	LOSS	protein-coding
590_T12,T22,T2,T42,T4	chr14	5.5E+07	6E+07	999	+	**5**	**62.5**	NA	Intergenic	B3|SINE|B2	-3065	NM_177049	319984	Mm.19047	NM_177049	ENSMUSG00000022208	**Jph4**	9330157P13Rik|JP-4|JPHL1	junctophilin 4	LOSS	protein-coding
222_T22,T2,T3,T42,T4	chr14	2.1E+07	2E+07	218999	+	**5**	**62.5**	NA	Intergenic	B3A|SINE|B2	-22770	NM_001205241	54169	Mm.248967	NM_017479	ENSMUSG00000021767	**Kat6b**	AI507552|B130044K16Rik|MYST-4|Morf|Myst4|mKIAA0383|qkf|querkopf	K(lysine) acetyltransferase 6B	LOSS	protein-coding
215_T22,T2,T3,T42,T4	chr14	2.1E+07	2E+07	24999	+	**5**	**62.5**	NA	intron (NM_134079, intron 7 of 10)	intron (NM_134079, intron 7 of 10)	-168770	NM_001205241	54169	Mm.248967	NM_017479	ENSMUSG00000021767	**Kat6b**	AI507552|B130044K16Rik|MYST-4|Morf|Myst4|mKIAA0383|qkf|querkopf	K(lysine) acetyltransferase 6B	LOSS	protein-coding
217_T22,T2,T3,T42,T4	chr14	2.1E+07	2E+07	7999	+	**5**	**62.5**	NA	intron (NM_134079, intron 7 of 10)	intron (NM_134079, intron 7 of 10)	-151270	NM_001205241	54169	Mm.248967	NM_017479	ENSMUSG00000021767	**Kat6b**	AI507552|B130044K16Rik|MYST-4|Morf|Myst4|mKIAA0383|qkf|querkopf	K(lysine) acetyltransferase 6B	LOSS	protein-coding
1389_T12,T1,T2,T32,T3	chr18	4.5E+07	5E+07	42999	+	**5**	**62.5**	NA	intron (NM_001312905, intron 1 of 10)	FordPrefect|DNA|hAT-Tip100	68140	NM_001312905	140492	Mm.411614	NM_080465	ENSMUSG00000054477	**Kcnn2**	KCa2.2|SK2|SKCA2|bc|fri	potassium intermediate/small conductance calcium-activated channel, subfamily N, member 2	LOSS	protein-coding
782_T12,T1,T22,T2,T32	chr18	1.5E+07	2E+07	264999	+	**5**	**62.5**	NA	TTS (NR_045372)	TTS (NR_045372)	15582	NM_001142731	106931	Mm.329299	NM_134112	ENSMUSG00000036225	**Kctd1**	4933402K10Rik|AI661543|AW553000	potassium channel tetramerisation domain containing 1	LOSS	protein-coding
1278_T12,T1,T22,T2,T3	chr18	4.1E+07	4E+07	999	+	**5**	**62.5**	NA	Intergenic	Lx5b|LINE|L1	337639	NM_026135	383348	Mm.76366	NM_026135	ENSMUSG00000051401	**Kctd16**	4930434H12Rik|Gm1267	potassium channel tetramerisation domain containing 16	LOSS	protein-coding

1267_T12,T1,T2,T32,T42	chr18	4E+07	4E+07	3999	+	**5**	**62.5**	NA	intron (NM_026135, intron 1 of 1)	Lx4A|LINE|L1	165139	NM_026135	383348	Mm.76366	NM_026135	ENSMUSG00000051401	**Kctd16**	4930434H12Rik|Gm1267	potassium channel tetramerisation domain containing 16	LOSS	protein-coding
488_T12,T22,T2,T3,T4	chr14	4.8E+07	5E+07	7999	+	**5**	**62.5**	NA	intron (NM_008477, intron 31 of 41)	intron (NM_008477, intron 31 of 41)	55744	NM_008477	16709	Mm.3110	NM_008477	ENSMUSG00000021843	**Ktn1**	-	kinectin 1	LOSS	protein-coding
1332_T1,T22,T2,T32,T4	chr18	4.2E+07	4E+07	999	+	**5**	**62.5**	NA	intron (NM_134137, intron 10 of 31)	B1_Mur4|SINE|Alu	24071	NM_134137	107045	Mm.312170	NM_134137	ENSMUSG00000024493	**Lars**	2310045K21Rik|3110009L02Rik|AW536573|mKIAA1352	leucyl-tRNA synthetase	LOSS	protein-coding
1334_T12,T1,T22,T2,T4	chr18	4.2E+07	4E+07	999	+	**5**	**62.5**	NA	intron (NM_134137, intron 10 of 31)	B2_Mm2|SINE|B2	22071	NM_134137	107045	Mm.312170	NM_134137	ENSMUSG00000024493	**Lars**	2310045K21Rik|3110009L02Rik|AW536573|mKIAA1352	leucyl-tRNA synthetase	LOSS	protein-coding
1818_T12,T22,T32,T3,T42	chr18	6.8E+07	7E+07	999	+	**5**	**62.5**	NA	intron (NM_172631, intron 2 of 5)	intron (NM_172631, intron 2 of 5)	153743	NM_172631	52662	Mm.440183	NM_172631	ENSMUSG00000024544	**Ldlrad4**	8230401C20Rik|A430083H02|A430108L08Rik|C18orf1|D18Ertd653e|D330030L18Rik	low density lipoprotein receptor class A domain containing 4	LOSS	protein-coding
1197_T12,T1,T2,T32,T3	chr18	3.5E+07	4E+07	30999	+	**5**	**62.5**	NA	Intergenic	L1MD|LINE|L1	-44976	NM_178005	107065	Mm.39900	NM_178005	ENSMUSG00000071862	**Lrrtm2**	AI851755|BB129880|C630011A14Rik|mKIAA0416	leucine rich repeat transmembrane neuronal 2	LOSS	protein-coding
1157_T12,T1,T22,T32,T3	chr18	3.2E+07	3E+07	999	+	**5**	**62.5**	NA	intron (NM_011946, intron 2 of 16)	intron (NM_011946, intron 2 of 16)	31911	NM_011946	26405	Mm.211762	NM_011946	ENSMUSG00000024383	**Map3k2**	9630061B06Rik|AI585793|Mekk2|Mekk2b	mitogen-activated protein kinase kinase kinase 2	LOSS	protein-coding
1856_T12,T1,T2,T32,T42	chr18	7.1E+07	7E+07	92999	+	**5**	**62.5**	NA	Intergenic	Intergenic	-5292	NM_001311071	17191	Mm.322	NM_010773	ENSMUSG00000024513	**Mbd2**	MBD2a	methyl-CpG binding domain protein 2	LOSS	protein-coding
1882_T12,T1,T2,T32,T42	chr18	7.1E+07	7E+07	55999	+	**5**	**62.5**	NA	intron (NM_007831, intron 15 of 28)	L1M3|LINE|L1	828208	NM_010773	17191	Mm.322	NM_010773	ENSMUSG00000024513	**Mbd2**	MBD2a	methyl-CpG binding domain protein 2	LOSS	protein-coding
1824_T12,T1,T2,T32,T3	chr18	6.8E+07	7E+07	999	+	**5**	**62.5**	NA	intron (NM_008560, intron 1 of 2)	Lx8b|LINE|L1	16840	NM_001271717	17200	Mm.426053	NM_008560	ENSMUSG00000045569	**Mc2r**	ACTH-R|ACTHR|MC2-R	melanocortin 2 receptor	LOSS	protein-coding
1797_T12,T1,T22,T2,T3	chr18	6.7E+07	7E+07	49999	+	**5**	**62.5**	NA	Intergenic	ORR1C1|LTR|ERVL-MaLR	135987	NM_016977	17202	Mm.229447	NM_016977	ENSMUSG00000047259	**Mc4r**	Mc4-r	melanocortin 4 receptor	LOSS	protein-coding
1821_T12,T2,T32,T3,T42	chr18	6.8E+07	7E+07	999	+	**5**	**62.5**	NA	Intergenic	Intergenic	43397	NM_013596	17203	Mm.8003	NM_013596	ENSMUSG00000007480	**Mc5r**	-	melanocortin 5 receptor	LOSS	protein-coding

<table>
<tr><td>943_T12,T1,T2,T32,T42</td><td>chr18</td><td>2.1E+07</td><td>2E+07</td><td>27999</td><td>+</td><td>5</td><td>62.5</td><td>NA</td><td>intron (NM_001033445, intron 2 of 5)</td><td>intron (NM_001033445, intron 2 of 5)</td><td>110156</td><td>NM_008586</td><td>17288</td><td>Mm.2682</td><td>NM_008586</td><td>ENSMUSG00000024313</td><td>Mep1b</td><td>Mep-1b</td><td>meprin 1 beta</td><td>LOSS</td><td>protein-coding</td></tr>
<tr><td>547_T12,T2,T3,T42,T4</td><td>chr14</td><td>5.2E+07</td><td>5E+07</td><td>1999</td><td>+</td><td>5</td><td>62.5</td><td>NA</td><td>Intergenic</td><td>Intergenic</td><td>-9342</td><td>NM_001029990</td><td>52535</td><td>Mm.5750</td><td>NM_001029990</td><td>ENSMUSG00000004561</td><td>Mettl17</td><td>2310032K15Rik|D14Ertd209e|Mett11d1</td><td>methyltransferase like 17</td><td>LOSS</td><td>protein-coding</td></tr>
<tr><td>979_T12,T1,T22,T2,T32</td><td>chr18</td><td>2.4E+07</td><td>2E+07</td><td>322999</td><td>+</td><td>5</td><td>62.5</td><td>NA</td><td>Intergenic</td><td>Intergenic</td><td>60170</td><td>NR_029573</td><td>387182</td><td></td><td>NR_029573</td><td>ENSMUSG00000065532</td><td>Mir187</td><td>Mirn187|mir-187|mmu-mir-187</td><td>microRNA 187</td><td>LOSS</td><td>ncRNA</td></tr>
<tr><td>312_T12,T22,T2,T42,T4</td><td>chr14</td><td>3.4E+07</td><td>3E+07</td><td>5999</td><td>+</td><td>5</td><td>62.5</td><td>NA</td><td>intron (NM_009758, intron 7 of 12)</td><td>intron (NM_009758, intron 7 of 12)</td><td>54996</td><td>NM_153127</td><td>105450</td><td>Mm.272673</td><td>NM_153127</td><td>ENSMUSG00000041445</td><td>Mmrn2</td><td>AA986839|ENDOGLYX1|Emilin3|EndoGlyx-1</td><td>multimerin 2</td><td>LOSS</td><td>protein-coding</td></tr>
<tr><td>203_T22,T2,T3,T42,T4</td><td>chr14</td><td>2E+07</td><td>2E+07</td><td>219999</td><td>+</td><td>5</td><td>62.5</td><td>NA</td><td>intron (NM_029698, intron 25 of 28)</td><td>B3|SINE|B2</td><td>-5945</td><td>NM_025440</td><td>66242</td><td>Mm.23747</td><td>NM_025440</td><td>ENSMUSG00000049960</td><td>Mrps16</td><td>1500011E11Rik|Mrps16</td><td>mitochondrial ribosomal protein S16</td><td>LOSS</td><td>protein-coding</td></tr>
<tr><td>1369_T12,T1,T22,T2,T32</td><td>chr18</td><td>4.4E+07</td><td>4E+07</td><td>54999</td><td>+</td><td>5</td><td>62.5</td><td>NA</td><td>Intergenic</td><td>Intergenic</td><td>-11074</td><td>NM_001033621</td><td>58916</td><td>Mm.143804</td><td>NM_001033621</td><td>ENSMUSG00000024471</td><td>Myot</td><td>5530402I04Rik|Ttid</td><td>myotilin</td><td>LOSS</td><td>protein-coding</td></tr>
<tr><td>209_T22,T2,T32,T3,T42</td><td>chr14</td><td>2.1E+07</td><td>2E+07</td><td>1999</td><td>+</td><td>5</td><td>62.5</td><td>NA</td><td>Intergenic</td><td>Intergenic</td><td>14040</td><td>NM_021508</td><td>59011</td><td>Mm.439911</td><td>NM_021508</td><td>ENSMUSG00000068697</td><td>Myoz1</td><td>2310001N11Rik|AV090278|FATZ|Myoz</td><td>myozenin 1</td><td>LOSS</td><td>protein-coding</td></tr>
<tr><td>1246_T12,T1,T22,T32,T3</td><td>chr18</td><td>3.8E+07</td><td>4E+07</td><td>2999</td><td>+</td><td>5</td><td>62.5</td><td>NA</td><td>Intergenic</td><td>Intergenic</td><td>-39975</td><td>NM_022996</td><td>65113</td><td>Mm.102496</td><td>NM_022996</td><td>ENSMUSG00000024425</td><td>Ndfip1</td><td>0610010M22Rik|N4wbp5</td><td>Nedd4 family interacting protein 1</td><td>LOSS</td><td>protein-coding</td></tr>
<tr><td>2166_T12,T1,T22,T2,T32</td><td>chr18</td><td>8.6E+07</td><td>9E+07</td><td>352999</td><td>+</td><td>5</td><td>62.5</td><td>NA</td><td>Intergenic</td><td>Intergenic</td><td>-10952</td><td>NM_144946</td><td>246317</td><td>Mm.329578</td><td>NM_144946</td><td>ENSMUSG00000050321</td><td>Neto1</td><td>AI851453|Btcl1|C130005O10Rik</td><td>neuropilin (NRP) and tolloid (TLL)-like 1</td><td>LOSS</td><td>protein-coding</td></tr>
<tr><td>1187_T22,T2,T32,T3,T4</td><td>chr18</td><td>3.3E+07</td><td>3E+07</td><td>999</td><td>+</td><td>5</td><td>62.5</td><td>NA</td><td>intron (NM_001267717, intron 3 of 4)</td><td>intron (NM_001267717, intron 3 of 4)</td><td>1634</td><td>NM_001267717</td><td>27528</td><td>Mm.407415</td><td>NM_053078</td><td>ENSMUSG00000042834</td><td>Nrep</td><td>AI325076|D0H4S114|Harp|P311|PTZ17|SEZ17</td><td>neuronal regeneration related protein</td><td>LOSS</td><td>protein-coding</td></tr>
<tr><td>2302_T1,T2,T32,T42,T4</td><td>chr2</td><td>8.1E+07</td><td>8E+07</td><td>999</td><td>+</td><td>5</td><td>62.5</td><td>NA</td><td>Intergenic</td><td>L1Md_A|LINE|L1</td><td>177736</td><td>NM_001190179</td><td>69482</td><td>Mm.29200</td><td>NM_027091</td><td>ENSMUSG00000026999</td><td>Nup35</td><td>2310006I24Rik|35kDa|5330402E05Rik|MP44|NO44</td><td>nucleoporin 35</td><td>LOSS</td><td>protein-coding</td></tr>
<tr><td>493_T12,T2,T3,T42,T4</td><td>chr14</td><td>4.9E+07</td><td>5E+07</td><td>999</td><td>+</td><td>5</td><td>62.5</td><td>NA</td><td>Intergenic</td><td>Intergenic</td><td>29872</td><td>NM_144841</td><td>18424</td><td>Mm.134516</td><td>NM_144841</td><td>ENSMUSG00000021848</td><td>Otx2</td><td>E130306E05Rik</td><td>orthodenticle homeobox 2</td><td>LOSS</td><td>protein-coding</td></tr>
<tr><td>2058_T1,T22,T2,T32,T42</td><td>chr18</td><td>8E+07</td><td>8E+07</td><td>999</td><td>+</td><td>5</td><td>62.5</td><td>NA</td><td>Intergenic</td><td>IAPLTR1a_Mm|LTR|ERVK</td><td>-335895</td><td>NM_053117</td><td>93737</td><td>Mm.24678</td><td>NM_053117</td><td>ENSMUSG00000056214</td><td>Pard6g</td><td>2410049N21Rik|Par6a</td><td>par-6 family cell polarity regulator gamma</td><td>LOSS</td><td>protein-coding</td></tr>
<tr><td>2061_T1,T22,T2,T32,T42</td><td>chr18</td><td>8E+07</td><td>8E+07</td><td>10999</td><td>+</td><td>5</td><td>62.5</td><td>NA</td><td>Intergenic</td><td>Lx2B2|LINE|L1</td><td>-308895</td><td>NM_053117</td><td>93737</td><td>Mm.24678</td><td>NM_053117</td><td>ENSMUSG00000056214</td><td>Pard6g</td><td>2410049N21Rik|Par6a</td><td>par-6 family cell polarity regulator gamma</td><td>LOSS</td><td>protein-coding</td></tr>
<tr><td>2064_T12,T1,T22,T2,T32</td><td>chr18</td><td>8E+07</td><td>8E+07</td><td>261999</td><td>+</td><td>5</td><td>62.5</td><td>NA</td><td>Intergenic</td><td>B1F2|SINE|Alu</td><td>-165395</td><td>NM_053117</td><td>93737</td><td>Mm.24678</td><td>NM_053117</td><td>ENSMUSG00000056214</td><td>Pard6g</td><td>2410049N21Rik|Par6a</td><td>par-6 family cell polarity regulator gamma</td><td>LOSS</td><td>protein-coding</td></tr>
</table>

276_T22,T2,T3,T42,T4	chr14	3.1E+07	3E+07	109999	+	**5**	**62.5**	NA	intron (NM_001081251, intron 19 of 32)	intron (NM_001081251, intron 19 of 32)	50362	NM_001081251	66923	Mm.27913	NM_025847	ENSMUSG00000042323	**Pbrm1**	2610016F04Rik|AI507524|BAF180|Pb1	polybromo 1	LOSS	protein-coding
1244_T1,T22,T2,T32,T3	chr18	3.8E+07	4E+07	2999	+	**5**	**62.5**	NA	Intergenic	(CA)n|Simple_repeat|Simple_repeat	73762	NM_029357	75599	Mm.36560	NM_029357	ENSMUSG00000051375	**Pcdh1**	2010005A06Rik|AI585920	protocadherin 1	LOSS	protein-coding
1238_T12,T1,T2,T32,T3	chr18	3.7E+07	4E+07	243999	+	**5**	**62.5**	NA	intron (NM_001301259, intron 1 of 3)	MMERVK10D3_I-int|LTR|ERVK	-23498	NM_053126	93872	Mm.196703	NM_053126	ENSMUSG00000051663	**Pcdhb1**	PcdhbA	protocadherin beta 1	LOSS	protein-coding
1209_T12,T1,T2,T32,T3	chr18	3.6E+07	4E+07	999	+	**5**	**62.5**	NA	intron (NM_026027, intron 2 of 3)	intron (NM_026027, intron 2 of 3)	19495	NM_026027	67199	Mm.30184	NM_026027	ENSMUSG00000024346	**Pfdn1**	2700086I23Rik|AA408327|AU044714	prefoldin 1	LOSS	protein-coding
1204_T12,T22,T2,T32,T3	chr18	3.6E+07	4E+07	999	+	**5**	**62.5**	NA	intron (NM_026027, intron 3 of 3)	B2_Mm1a|SINE|B2	28495	NM_026027	67199	Mm.30184	NM_026027	ENSMUSG00000024346	**Pfdn1**	2700086I23Rik|AA408327|AU044714	prefoldin 1	LOSS	protein-coding
1205_T12,T1,T2,T32,T3	chr18	3.6E+07	4E+07	999	+	**5**	**62.5**	NA	intron (NM_026027, intron 3 of 3)	RSINE1|SINE|B4	27495	NM_026027	67199	Mm.30184	NM_026027	ENSMUSG00000024346	**Pfdn1**	2700086I23Rik|AA408327|AU044714	prefoldin 1	LOSS	protein-coding
1207_T12,T22,T2,T32,T3	chr18	3.6E+07	4E+07	999	+	**5**	**62.5**	NA	intron (NM_026027, intron 3 of 3)	B1_Mur4|SINE|Alu	22495	NM_026027	67199	Mm.30184	NM_026027	ENSMUSG00000024346	**Pfdn1**	2700086I23Rik|AA408327|AU044714	prefoldin 1	LOSS	protein-coding
1111_T12,T1,T2,T32,T3	chr18	3.1E+07	3E+07	51999	+	**5**	**62.5**	NA	Intergenic	MTD|LTR|ERVL-MaLR	488604	NM_181414	225326	Mm.194127	NM_181414	ENSMUSG00000033628	**Pik3c3**	5330434F23Rik|Vps34	phosphoinositide-3-kinase, class 3	LOSS	protein-coding
1329_T12,T1,T22,T2,T32	chr18	4.2E+07	4E+07	38999	+	**5**	**62.5**	NA	intron (NM_134137, intron 31 of 31)	intron (NM_134137, intron 31 of 31)	-13291	NM_027072	69401	Mm.34841	NM_027072	ENSMUSG00000059455	**Plac8l1**	1700009N18Rik|1700015M15Rik	PLAC8-like 1	LOSS	protein-coding
1788_T12,T22,T2,T32,T3	chr18	6.6E+07	7E+07	999	+	**5**	**62.5**	NA	Intergenic	B1_Mus2|SINE|Alu	-34604	NM_021451	58801	Mm.271878	NM_021451	ENSMUSG00000024521	**Pmaip1**	Noxa	phorbol-12-myristate-13-acetate-induced protein 1	LOSS	protein-coding
207_T22,T2,T32,T3,T42	chr14	2.1E+07	2E+07	999	+	**5**	**62.5**	NA	TTS (NR_033571)	TTS (NR_033571)	-24427	NM_008914	19056	Mm.274432	NM_008914	ENSMUSG00000021816	**Ppp3cb**	1110063J16Rik|Calnb|CnAbeta|Cnab	protein phosphatase 3, catalytic subunit, beta isoform	LOSS	protein-coding
1295_T12,T1,T22,T2,T32	chr18	4.1E+07	4E+07	999	+	**5**	**62.5**	NA	Intergenic	RLTR40|LTR|ERVK	660194	NM_029942	77619	Mm.30649	NM_029942	ENSMUSG00000056671	**Prelid2**	1700003A01Rik|C330008K14Rik	PRELI domain containing 2	LOSS	protein-coding
1298_T12,T1,T22,T2,T32	chr18	4.1E+07	4E+07	12999	+	**5**	**62.5**	NA	Intergenic	Intergenic	634194	NM_029942	77619	Mm.30649	NM_029942	ENSMUSG00000056671	**Prelid2**	1700003A01Rik|C330008K14Rik	PRELI domain containing 2	LOSS	protein-coding

1303_T12,T1,T22,T2,T32	chr18	4.1E+07	4E+07	18999	+	**5**	**62.5**	NA	Intergenic	Intergenic	579194	NM_029942	77619	Mm.30649	NM_029942	ENSMUSG00000056671	**Prelid2**	1700003A01Rik|C330008K14Rik	PRELI domain containing 2	LOSS	protein-coding
1307_T12,T1,T22,T2,T32	chr18	4.1E+07	4E+07	64999	+	**5**	**62.5**	NA	Intergenic	Intergenic	523194	NM_029942	77619	Mm.30649	NM_029942	ENSMUSG00000056671	**Prelid2**	1700003A01Rik|C330008K14Rik	PRELI domain containing 2	LOSS	protein-coding
1311_T12,T1,T2,T32,T3	chr18	4.1E+07	4E+07	37999	+	**5**	**62.5**	NA	Intergenic	L1MA8|LINE|L1	459694	NM_029942	77619	Mm.30649	NM_029942	ENSMUSG00000056671	**Prelid2**	1700003A01Rik|C330008K14Rik	PRELI domain containing 2	LOSS	protein-coding
1318_T12,T1,T22,T2,T32	chr18	4.2E+07	4E+07	39999	+	**5**	**62.5**	NA	Intergenic	ORR1D1|LTR|ERVL-MaLR	102694	NM_029942	77619	Mm.30649	NM_029942	ENSMUSG00000056671	**Prelid2**	1700003A01Rik|C330008K14Rik	PRELI domain containing 2	LOSS	protein-coding
1322_T12,T22,T2,T32,T42	chr18	4.2E+07	4E+07	2999	+	**5**	**62.5**	NA	Intergenic	Intergenic	-16806	NM_029942	77619	Mm.30649	NM_029942	ENSMUSG00000056671	**Prelid2**	1700003A01Rik|C330008K14Rik	PRELI domain containing 2	LOSS	protein-coding
1321_T12,T1,T22,T2,T32	chr18	4.2E+07	4E+07	62999	+	**5**	**62.5**	NA	intron (NM_029942, intron 4 of 6)	(TTTG)n|Simple_repeat|Simple_repeat	23194	NM_029942	77619	Mm.30649	NM_029942	ENSMUSG00000056671	**Prelid2**	1700003A01Rik|C330008K14Rik	PRELI domain containing 2	LOSS	protein-coding
1319_T12,T1,T22,T2,T32	chr18	4.2E+07	4E+07	9999	+	**5**	**62.5**	NA	intron (NM_029942, intron 6 of 6)	intron (NM_029942, intron 6 of 6)	71694	NM_029942	77619	Mm.30649	NM_029942	ENSMUSG00000056671	**Prelid2**	1700003A01Rik|C330008K14Rik	PRELI domain containing 2	LOSS	protein-coding
1528_T12,T1,T22,T2,T32	chr18	5.1E+07	5E+07	999	+	**5**	**62.5**	NA	Intergenic	AT_rich|Low_complexity|Low_complexity	270102	NM_001081224	71373	Mm.63546	NM_001081224	ENSMUSG00000073565	**Prr16**	5430406M13Rik|AI607429	proline rich 16	LOSS	protein-coding
1531_T12,T1,T22,T2,T42	chr18	5.1E+07	5E+07	999	+	**5**	**62.5**	NA	Intergenic	L1Md_A|LINE|L1	282102	NM_001081224	71373	Mm.63546	NM_001081224	ENSMUSG00000073565	**Prr16**	5430406M13Rik|AI607429	proline rich 16	LOSS	protein-coding
1534_T12,T1,T22,T32,T42	chr18	5.1E+07	5E+07	999	+	**5**	**62.5**	NA	Intergenic	MLT1A|LTR|ERVL-MaLR	346102	NM_001081224	71373	Mm.63546	NM_001081224	ENSMUSG00000073565	**Prr16**	5430406M13Rik|AI607429	proline rich 16	LOSS	protein-coding
1536_T12,T1,T22,T32,T42	chr18	5.2E+07	5E+07	999	+	**5**	**62.5**	NA	Intergenic	Intergenic	448102	NM_001081224	71373	Mm.63546	NM_001081224	ENSMUSG00000073565	**Prr16**	5430406M13Rik|AI607429	proline rich 16	LOSS	protein-coding
1538_T12,T1,T22,T2,T32	chr18	5.2E+07	5E+07	83999	+	**5**	**62.5**	NA	Intergenic	CT-rich|Low_complexity|Low_complexity	491602	NM_001081224	71373	Mm.63546	NM_001081224	ENSMUSG00000073565	**Prr16**	5430406M13Rik|AI607429	proline rich 16	LOSS	protein-coding
777_T12,T1,T2,T32,T42	chr18	1.5E+07	1E+07	7999	+	**5**	**62.5**	NA	Intergenic	ID_B1|SINE|B4	-2651	NM_001163609	73677	Mm.87277	NM_001163609	ENSMUSG00000036743	**Psma8**	2410072D24Rik	proteasome (prosome, macropain) subunit, alpha type, 8	LOSS	protein-coding
779_T12,T1,T2,T32,T42	chr18	1.5E+07	1E+07	23999	+	**5**	**62.5**	NA	intron (NM_001163609, intron 1 of 6)	MTE2b|LTR|ERVL-MaLR	14349	NM_001163609	73677	Mm.87277	NM_001163609	ENSMUSG00000036743	**Psma8**	2410072D24Rik	proteasome (prosome, macropain) subunit, alpha type, 8	LOSS	protein-coding

460_T12,T22,T2,T42,T4	chr14	4.5E+07	5E+07	999	+	**5**	**62.5**	NA	intron (NM_025959, intron 7 of 13)	L1MA7|LINE|L1	10176	NM_025959	67089	Mm.18472	NM_025959	ENSMUSG00000021832	**Psmc6**	2300001E01Rik|AI451058	proteasome (prosome, macropain) 26S subunit, ATPase, 6	LOSS	protein-coding
1199_T12,T1,T2,T32,T3	chr18	3.6E+07	4E+07	999	+	**5**	**62.5**	NA	Intergenic	Intergenic	8838	NM_008989	19290	Mm.231802	NM_008989	ENSMUSG00000043991	**Pura**	CAGER-1|Pur-alpha|ssCRE-BP	purine rich element binding protein A	LOSS	protein-coding
1113_T12,T1,T2,T32,T3	chr18	3.1E+07	3E+07	8999	+	**5**	**62.5**	NA	Intergenic	L1MA9|LINE|L1	519128	NM_009065	19762	Mm.5163	NM_009065	ENSMUSG00000057455	**Rit2**	RIBA|Rin|Roc2	Ras-like without CAAX 2	LOSS	protein-coding
1117_T12,T1,T2,T32,T3	chr18	3.1E+07	3E+07	11999	+	**5**	**62.5**	NA	Intergenic	Intergenic	497628	NM_009065	19762	Mm.5163	NM_009065	ENSMUSG00000057455	**Rit2**	RIBA|Rin|Roc2	Ras-like without CAAX 2	LOSS	protein-coding
1119_T12,T1,T2,T32,T3	chr18	3.1E+07	3E+07	30999	+	**5**	**62.5**	NA	Intergenic	Lx6|LINE|L1	469128	NM_009065	19762	Mm.5163	NM_009065	ENSMUSG00000057455	**Rit2**	RIBA|Rin|Roc2	Ras-like without CAAX 2	LOSS	protein-coding
1121_T12,T1,T2,T32,T3	chr18	3.1E+07	3E+07	999	+	**5**	**62.5**	NA	Intergenic	L1Md_F2|LINE|L1	446128	NM_009065	19762	Mm.5163	NM_009065	ENSMUSG00000057455	**Rit2**	RIBA|Rin|Roc2	Ras-like without CAAX 2	LOSS	protein-coding
1140_T12,T1,T2,T32,T42	chr18	3.1E+07	3E+07	30999	+	**5**	**62.5**	NA	intron (NM_009065, intron 1 of 4)	intron (NM_009065, intron 1 of 4)	52128	NM_009065	19762	Mm.5163	NM_009065	ENSMUSG00000057455	**Rit2**	RIBA|Rin|Roc2	Ras-like without CAAX 2	LOSS	protein-coding
1138_T12,T1,T2,T32,T42	chr18	3.1E+07	3E+07	8999	+	**5**	**62.5**	NA	intron (NM_009065, intron 2 of 4)	intron (NM_009065, intron 2 of 4)	76128	NM_009065	19762	Mm.5163	NM_009065	ENSMUSG00000057455	**Rit2**	RIBA|Rin|Roc2	Ras-like without CAAX 2	LOSS	protein-coding
1136_T12,T1,T2,T32,T42	chr18	3.1E+07	3E+07	37999	+	**5**	**62.5**	NA	intron (NM_009065, intron 3 of 4)	L1_Mus2|LINE|L1	111628	NM_009065	19762	Mm.5163	NM_009065	ENSMUSG00000057455	**Rit2**	RIBA|Rin|Roc2	Ras-like without CAAX 2	LOSS	protein-coding
1130_T12,T1,T22,T2,T32	chr18	3.1E+07	3E+07	15999	+	**5**	**62.5**	NA	intron (NM_009065, intron 4 of 4)	intron (NM_009065, intron 4 of 4)	300628	NM_009065	19762	Mm.5163	NM_009065	ENSMUSG00000057455	**Rit2**	RIBA|Rin|Roc2	Ras-like without CAAX 2	LOSS	protein-coding
1132_T12,T1,T22,T2,T32	chr18	3.1E+07	3E+07	149999	+	**5**	**62.5**	NA	intron (NM_009065, intron 4 of 4)	Lx2|LINE|L1	213628	NM_009065	19762	Mm.5163	NM_009065	ENSMUSG00000057455	**Rit2**	RIBA|Rin|Roc2	Ras-like without CAAX 2	LOSS	protein-coding
721_T12,T1,T22,T3,T42	chr18	1E+07	1E+07	5999	+	**5**	**62.5**	NA	Intergenic	L1Md_A|LINE|L1	-51708	NM_009071	19877	Mm.6710	NM_009071	ENSMUSG00000024290	**Rock1**	1110055K06Rik|Rock-I	Rho-associated coiled-coil containing protein kinase 1	LOSS	protein-coding
1146_T12,T1,T22,T2,T32	chr18	3.2E+07	3E+07	133999	+	**5**	**62.5**	NA	intron (NM_172965, intron 4 of 20)	intron (NM_172965, intron 4 of 20)	14117	NM_172965	269003	Mm.194538	NM_172965	ENSMUSG00000024260	**Sap130**	2610304F09Rik|6720406D06	Sin3A associated protein	LOSS	protein-coding

1807_T22,T2,T32,T3,T42	chr18	6.8E+07	7E+07	999	+	**5**	**62.5**	NA	intron (NM_028112, intron 2 of 7)	B2_Mm2|SINE|B2	6124	NM_001039088	72124	Mm.307315	NM_028112	ENSMUSG00000079614	**Seh1l**	2610007A16Rik|AW540070|SEC13L|SEH1A|SEH1B|Seh1	SEH1-like (S. cerevisiae	LOSS	protein-coding
2038_T12,T1,T22,T2,T32	chr18	7.9E+07	8E+07	123999	+	**5**	**62.5**	NA	Intergenic	Intergenic	-64109	NM_053099	240427	Mm.312871	NM_053099	ENSMUSG00000024548	**Setbp1**	C130092E12|Seb|mKIAA0437	SET binding protein 1	LOSS	protein-coding
2040_T12,T1,T22,T2,T32	chr18	7.9E+07	8E+07	85999	+	**5**	**62.5**	NA	Intergenic	Intergenic	-178109	NM_053099	240427	Mm.312871	NM_053099	ENSMUSG00000024548	**Setbp1**	C130092E12|Seb|mKIAA0437	SET binding protein 1	LOSS	protein-coding
2042_T12,T1,T22,T2,T32	chr18	7.9E+07	8E+07	14999	+	**5**	**62.5**	NA	Intergenic	Intergenic	-232609	NM_053099	240427	Mm.312871	NM_053099	ENSMUSG00000024548	**Setbp1**	C130092E12|Seb|mKIAA0437	SET binding protein 1	LOSS	protein-coding
2045_T12,T1,T2,T32,T42	chr18	7.9E+07	8E+07	27999	+	**5**	**62.5**	NA	Intergenic	L1MCa|LINE|L1	-270109	NM_053099	240427	Mm.312871	NM_053099	ENSMUSG00000024548	**Setbp1**	C130092E12|Seb|mKIAA0437	SET binding protein 1	LOSS	protein-coding
2049_T12,T1,T2,T32,T42	chr18	7.9E+07	8E+07	17999	+	**5**	**62.5**	NA	Intergenic	B2_Mm1a|SINE|B2	-302109	NM_053099	240427	Mm.312871	NM_053099	ENSMUSG00000024548	**Setbp1**	C130092E12|Seb|mKIAA0437	SET binding protein 1	LOSS	protein-coding
2036_T12,T1,T22,T2,T32	chr18	7.9E+07	8E+07	187999	+	**5**	**62.5**	NA	intron (NM_053099, intron 2 of 5)	B1F2|SINE|Alu	95891	NM_053099	240427	Mm.312871	NM_053099	ENSMUSG00000024548	**Setbp1**	C130092E12|Seb|mKIAA0437	SET binding protein 1	LOSS	protein-coding
2032_T12,T1,T22,T2,T32	chr18	7.9E+07	8E+07	999	+	**5**	**62.5**	NA	intron (NM_053099, intron 3 of 5)	L1_Mur3|LINE|L1	218391	NM_053099	240427	Mm.312871	NM_053099	ENSMUSG00000024548	**Setbp1**	C130092E12|Seb|mKIAA0437	SET binding protein 1	LOSS	protein-coding
2034_T12,T1,T22,T2,T42	chr18	7.9E+07	8E+07	999	+	**5**	**62.5**	NA	intron (NM_053099, intron 3 of 5)	intron (NM_053099, intron 3 of 5)	200391	NM_053099	240427	Mm.312871	NM_053099	ENSMUSG00000024548	**Setbp1**	C130092E12|Seb|mKIAA0437	SET binding protein 1	LOSS	protein-coding
397_T12,T22,T2,T3,T42	chr14	4E+07	4E+07	8999	+	**5**	**62.5**	NA	Intergenic	Intergenic	597965	NM_177816	328381	Mm.235694	NM_177816	ENSMUSG00000037833	**Sh2d4b**	A430109M18Rik|D030001E08	SH2 domain containing 4B	LOSS	protein-coding
1325_T12,T1,T22,T2,T32	chr18	4.2E+07	4E+07	158999	+	**5**	**62.5**	NA	intron (NM_172966, intron 1 of 7)	intron (NM_172966, intron 1 of 7)	4290	NM_001146299	269016	Mm.45472	NM_172966	ENSMUSG00000057719	**Sh3rf2**	2310046K19|9130023G24Rik|AI452083|Ppp1r39|RNF158	SH3 domain containing ring finger 2	LOSS	protein-coding
1986_T12,T1,T2,T32,T42	chr18	7.7E+07	8E+07	1999	+	**5**	**62.5**	NA	Intergenic	Intergenic	-54905	NM_001109743	664805	Mm.329543	NM_001109743	ENSMUSG00000091519	**Skor2**	Corl2|EG664805|Fussel18|Gm7348	SKI family transcriptional corepressor 2	LOSS	protein-coding
1988_T12,T1,T2,T32,T42	chr18	7.7E+07	8E+07	28999	+	**5**	**62.5**	NA	Intergenic	Intergenic	-38405	NM_001109743	664805	Mm.329543	NM_001109743	ENSMUSG00000091519	**Skor2**	Corl2|EG664805|Fussel18|Gm7348	SKI family transcriptional corepressor 2	LOSS	protein-coding
2030_T12,T1,T22,T2,T32	chr18	7.9E+07	8E+07	156999	+	**5**	**62.5**	NA	intron (NM_053099, intron 5 of 5)	ID_B1|SINE|B4	-186050	NM_030683	27411	Mm.44158	NM_030683	ENSMUSG00000024552	**Slc14a2**	UT-A1|UT-A3	solute carrier family 14 (urea transporter), member 2	LOSS	protein-coding

582_T12,T22,T2,T3,T4	chr14	5.4E+07	5E+07	233999	+	**5**	**62.5**	NA	intron (NM_001253679, intron 2 of 10)	intron (NM_001253679, intron 2 of 10)	-4028	NM_001253680	20540	Mm.142455	NM_011405	ENSMUSG00000000958	**Slc7a7**	AI790233|my+lat1	solute carrier family 7 (cationic amino acid transporter, y+ system), member 7	LOSS	protein-coding
1974_T1,T22,T2,T32,T3	chr18	7.6E+07	8E+07	999	+	**5**	**62.5**	NA	exon (NM_001311070, exon 2 of 9)	exon (NM_001311070, exon 2 of 9)	25555	NM_001311070	17126	Mm.152699	NM_010754	ENSMUSG00000024563	**Smad2**	7120426M23Rik|Madh2|Madr2|Smad-2|mMad2	SMAD family member 2	LOSS	protein-coding
1972_T12,T22,T2,T32,T3	chr18	7.6E+07	8E+07	2999	+	**5**	**62.5**	NA	intron (NM_010754, intron 1 of 10)	CpG	299	NM_010754	17126	Mm.152699	NM_010754	ENSMUSG00000024563	**Smad2**	7120426M23Rik|Madh2|Madr2|Smad-2|mMad2	SMAD family member 2	LOSS	protein-coding
1976_T1,T22,T2,T32,T3	chr18	7.6E+07	8E+07	999	+	**5**	**62.5**	NA	intron (NM_010754, intron 6 of 10)	intron (NM_010754, intron 6 of 10)	30555	NM_001311070	17126	Mm.152699	NM_010754	ENSMUSG00000024563	**Smad2**	7120426M23Rik|Madh2|Madr2|Smad-2|mMad2	SMAD family member 2	LOSS	protein-coding
1967_T12,T1,T2,T32,T3	chr18	7.5E+07	8E+07	78999	+	**5**	**62.5**	NA	Intergenic	MLT1A0|LTR|ERVL-MaLR	60635	NM_001042660	17131	Mm.34407	NM_008543	ENSMUSG00000025880	**Smad7**	Madh7	SMAD family member 7	LOSS	protein-coding
1965_T12,T1,T2,T32,T3	chr18	7.5E+07	8E+07	4999	+	**5**	**62.5**	NA	intron (NM_001042660, intron 3 of 3)	intron (NM_001042660, intron 3 of 3)	17635	NM_001042660	17131	Mm.34407	NM_008543	ENSMUSG00000025880	**Smad7**	Madh7	SMAD family member 7	LOSS	protein-coding
1577_T1,T22,T2,T32,T42	chr18	5.3E+07	5E+07	999	+	**5**	**62.5**	NA	intron (NM_026386, intron 1 of 14)	L1_Mur3|LINE|L1	7635	NM_026386	67804	Mm.252171	NM_026386	ENSMUSG00000034484	**Snx2**	0610030A03Rik	sorting nexin 2	LOSS	protein-coding
485_T12,T22,T2,T3,T42	chr14	4.7E+07	5E+07	641999	+	**5**	**62.5**	NA	intron (NM_080843, intron 1 of 1)	intron (NM_080843, intron 1 of 1)	9357	NM_080843	67296	Mm.23957	NM_080843	ENSMUSG00000048379	**Socs4**	3110032M18Rik|A730004F22Rik|AI427843|AU015727|AU015859|Socs7	suppressor of cytokine signaling 4	LOSS	protein-coding
2205_T12,T1,T22,T2,T42	chr18	8.8E+07	9E+07	1999	+	**5**	**62.5**	NA	Intergenic	Lx3B|LINE|L1	1015707	NM_018821	54607	Mm.91920	NM_018821	ENSMUSG00000056153	**Socs6**	1500012M23Rik|5830401B18Rik|AI447482|Cis4|Cish4|HSPC060|SOCS-4|SOCS-6|SSI4|STAI4|STATI4|Socs4	suppressor of cytokine signaling 6	LOSS	protein-coding
775_T12,T1,T2,T32,T42	chr18	1.5E+07	1E+07	100999	+	**5**	**62.5**	NA	intron (NM_001161371, intron 4 of 8)	intron (NM_001161371, intron 4 of 8)	34914	NM_001161369	268996	Mm.31761	NM_009280	ENSMUSG00000037013	**Ss18**	D130059H17|Ssxt|Syt	SS18, nBAF chromatin remodeling complex subunit	LOSS	protein-coding
1766_T12,T1,T2,T32,T42	chr18	6.4E+07	6E+07	6999	+	**5**	**62.5**	NA	Intergenic	Intergenic	-242359	NM_009182	20451	Mm.252110	NM_009182	ENSMUSG00000056812	**St8sia3**	AI847333|ST8SiaIII|Siat8c	ST8 alpha-N-acetyl-neuraminide alpha-2,8-sialyltransferase 3	LOSS	protein-coding

2000_T12,T1,T2,T32,T4	chr18	7.7E+07	8E+07	7999	+	**5**	**62.5**	NA	intron (NM_001164167, intron 11 of 13)	intron (NM_001164167, intron 11 of 13)	-48347	NM_153124	225742	Mm.365178	NM_013666	ENSMUSG00000025425	**St8sia5**	ST8SiaV|Siat8e	ST8 alpha-N-acetyl-neuraminide alpha-2,8-sialyltransferase 5	LOSS	protein-coding
1176_T12,T1,T22,T2,T32	chr18	3.3E+07	3E+07	190999	+	**5**	**62.5**	NA	intron (NM_009793, intron 5 of 10)	intron (NM_009793, intron 5 of 10)	67816	NM_133774	170459	Mm.127058	NM_133774	ENSMUSG00000024378	**Stard4**	4632419C16Rik|9030213J02Rik	StAR-related lipid transfer (START) domain containing 4	LOSS	protein-coding
462_T12,T22,T2,T3,T4	chr14	4.5E+07	5E+07	999	+	**5**	**62.5**	NA	TTS (NM_025959)	TTS (NM_025959)	-2186	NM_019637	56291	Mm.202561	NM_019637	ENSMUSG00000053205	**Styx**	0610039A20Rik|hStyxb	serine/threonine/tyrosine interaction protein	LOSS	protein-coding
1142_T12,T1,T22,T2,T32	chr18	3.1E+07	3E+07	98999	+	**5**	**62.5**	NA	Intergenic	MTE2b-int|LTR|ERVL-MaLR	36415	NM_009308	20983	Mm.233846	NM_009308	ENSMUSG00000024261	**Syt4**	SytIV	synaptotagmin IV	LOSS	protein-coding
780_T12,T1,T22,T2,T32	chr18	1.5E+07	1E+07	174999	+	**5**	**62.5**	NA	intron (NM_001100449, intron 9 of 14)	intron (NM_001100449, intron 9 of 14)	41755	NM_001100449	72504	Mm.312233	NM_001100449	ENSMUSG00000054321	**Taf4b**	105kDa|2610524B04Rik|4932409F03Rik|AW987595|TAFII-105|TAFII105|Taf2c2	TATA-box binding protein associated factor 4b	LOSS	protein-coding
1835_T12,T1,T2,T32,T3	chr18	6.9E+07	7E+07	999	+	**5**	**62.5**	NA	Intergenic	MTD|LTR|ERVL-MaLR	-116492	NM_013685	21413	Mm.4269	NM_013685	ENSMUSG00000053477	**Tcf4**	5730422P05Rik|ASP-I2|E2-2|E2.2|ITF-2|ITF-2b|ITF2|ME2|MITF-2A|MITF-2B|SEF-2|SEF2|SEF2-1|TFE|Tcf-4|bHLHb19	transcription factor 4	LOSS	protein-coding
1838_T12,T1,T22,T2,T32	chr18	6.9E+07	7E+07	81999	+	**5**	**62.5**	NA	Intergenic	Intergenic	-61992	NM_013685	21413	Mm.4269	NM_013685	ENSMUSG00000053477	**Tcf4**	5730422P05Rik|ASP-I2|E2-2|E2.2|ITF-2|ITF-2b|ITF2|ME2|MITF-2A|MITF-2B|SEF-2|SEF2|SEF2-1|TFE|Tcf-4|bHLHb19	transcription factor 4	LOSS	protein-coding
717_T12,T1,T22,T2,T3	chr18	9826501	1E+07	123999	+	**5**	**62.5**	NA	Intergenic	ORR1A1|LTR|ERVL-MaLR	-69680	NM_153552	225160	Mm.219648	NM_153552	ENSMUSG00000024287	**Thoc1**	3110002N20Rik|AW107452|NMP-84	THO complex 1	LOSS	protein-coding
147_T1,T2,T3,T42,T4	chr14	1.4E+07	1E+07	12999	+	**5**	**62.5**	NA	Intergenic	LTR73|LTR|ERV1	60313	NM_001285780	66231	Mm.295875	NM_025435	ENSMUSG00000053453	**Thoc7**	Nif3l1bp1	THO complex 7	LOSS	protein-coding
755_T12,T1,T2,T3,T4	chr18	1.2E+07	1E+07	31999	+	**5**	**62.5**	NA	intron (NM_001289666, intron 2 of 14)	intron (NM_001289666, intron 2 of 14)	8037	NR_110356	338363	Mm.271567	NM_178801	ENSMUSG00000049411	**Tmem241**	6030446N20Rik	transmembrane protein 241	LOSS	protein-coding

491_T12,T22,T2,T3,T4	chr14	4.8E+07	5E+07	17999	+	**5**	**62.5**	NA	intron (NM_001310584, intron 4 of 16)	PB1D11|SINE|Alu	14148	NR_132324	218989	Mm.85211	NM_172600	ENSMUSG00000036339	**Tmem260**	5930435P15|6720456H20Rik	transmembrane protein 260	LOSS	protein-coding
2273_T12,T1,T2,T32,T3	chr18	9E+07	9E+07	5999	+	**5**	**62.5**	NA	Intergenic	Intergenic	-341654	NM_198295	67988	Mm.268041	NM_198295	ENSMUSG00000024614	**Tmx3**	6430411B10Rik|A730024F05Rik|AV259382|Txndc10|mKIAA1830	thioredoxin-related transmembrane protein 3	LOSS	protein-coding
2275_T12,T1,T2,T32,T3	chr18	9E+07	9E+07	999	+	**5**	**62.5**	NA	Intergenic	L1_Mus2|LINE|L1	-333154	NM_198295	67988	Mm.268041	NM_198295	ENSMUSG00000024614	**Tmx3**	6430411B10Rik|A730024F05Rik|AV259382|Txndc10|mKIAA1830	thioredoxin-related transmembrane protein 3	LOSS	protein-coding
2277_T12,T1,T2,T32,T3	chr18	9E+07	9E+07	14999	+	**5**	**62.5**	NA	Intergenic	MusHAL1|LINE|L1	-321154	NM_198295	67988	Mm.268041	NM_198295	ENSMUSG00000024614	**Tmx3**	6430411B10Rik|A730024F05Rik|AV259382|Txndc10|mKIAA1830	thioredoxin-related transmembrane protein 3	LOSS	protein-coding
2282_T12,T1,T22,T2,T3	chr18	9E+07	9E+07	10999	+	**5**	**62.5**	NA	Intergenic	L1Md_A|LINE|L1	-74154	NM_198295	67988	Mm.268041	NM_198295	ENSMUSG00000024614	**Tmx3**	6430411B10Rik|A730024F05Rik|AV259382|Txndc10|mKIAA1830	thioredoxin-related transmembrane protein 3	LOSS	protein-coding
2284_T12,T1,T22,T2,T3	chr18	9E+07	9E+07	1999	+	**5**	**62.5**	NA	Intergenic	Intergenic	-47654	NM_198295	67988	Mm.268041	NM_198295	ENSMUSG00000024614	**Tmx3**	6430411B10Rik|A730024F05Rik|AV259382|Txndc10|mKIAA1830	thioredoxin-related transmembrane protein 3	LOSS	protein-coding
450_T12,T22,T2,T3,T4	chr14	4.5E+07	5E+07	179999	+	**5**	**62.5**	NA	Intergenic	L1_Mur3|LINE|L1	95925	NM_172597	70561	Mm.28129	NM_172597	ENSMUSG00000021830	**Txndc16**	5730420B22Rik|C77647	thioredoxin domain containing 16	LOSS	protein-coding
1757_T12,T1,T2,T32,T4	chr18	6.4E+07	6E+07	999	+	**5**	**62.5**	NA	intron (NM_016792, intron 2 of 7)	intron (NM_016792, intron 2 of 7)	12359	NM_016792	53382	Mm.19169	NM_016792	ENSMUSG00000024583	**Txnl1**	32kDa|TRP32|Txnl	thioredoxin-like 1	LOSS	protein-coding
682_T12,T22,T2,T32,T3	chr18	7850501	8E+06	999	+	**5**	**62.5**	NA	Intergenic	Lx3A|LINE|L1	-17832	NM_001282093	225131	Mm.204524	NM_153085	ENSMUSG00000024283	**Wac**	1110067P07Rik|A230035H12Rik|AI256735|AI256776|Wwp4	WW domain containing adaptor with coiled-coil	LOSS	protein-coding
325_T12,T22,T2,T32,T4	chr14	3.5E+07	3E+07	999	+	**5**	**62.5**	NA	intron (NM_001004436, intron 10 of 18)	B1_Mm|SINE|Alu	57072	NM_001301330	218914	Mm.27957	NM_001004436	ENSMUSG00000041408	**Wapl**	A530089A20Rik|BC037674|DIF-2|FOE|Wapal	WAPL cohesin release factor	LOSS	protein-coding
267_T1,T2,T32,T42,T4	chr14	2.9E+07	3E+07	3999	+	**5**	**62.5**	NA	exon (NM_009524, exon 5 of 5)	exon (NM_009524, exon 5 of 5)	11095	NM_001256224	22418	Mm.287544	NM_009524	ENSMUSG00000021994	**Wnt5a**	8030457G12Rik|Wnt-5a	wingless-type MMTV integration site family, member 5A	LOSS	protein-coding
1373_T1,T22,T2,T32,T42	chr18	4.5E+07	4E+07	999	+	**5**	**62.5**	NA	Intergenic	MTEa-int|LTR|ERVL-MaLR	-2665	NM_001163013	240255	Mm.244482	NM_001163013	ENSMUSG00000034653	**Ythdc2**	3010002F02Rik|BC037178	YTH domain containing 2	LOSS	protein-coding

1378_T1,T22,T2,T32,T42	chr18	4.5E+07	4E+07	999	+	**5**	**62.5**	NA	Intergenic	(TTTTC)n|Simple_repeat|Simple_repeat	77335	NM_001163013	240255	Mm.244482	NM_001163013	ENSMUSG00000034653	**Ythdc2**	3010002F02Rik|BC037178	YTH domain containing 2	LOSS	protein-coding
1962_T12,T1,T2,T3	chr18	7.5E+07	7E+07	999	+	**4**	**50**	NA	intron (NM_177470, intron 1 of 9)	L1MC2|LINE|L1	2788	NM_177470	52538	Mm.245724	NM_177470	ENSMUSG00000036880	**Acaa2**	0610011L04Rik|AI255831|AI265397|D18Ertd240e	acetyl-Coenzyme A acyltransferase 2 (mitochondrial 3-oxoacyl-Coenzyme A thiolase)	LOSS	protein-coding
1670_T12,T1,T22,T3	chr18	5.9E+07	6E+07	999	+	**4**	**50**	NA	Intergenic	L1Md_T|LINE|L1	-31764	NM_175506	240322	Mm.71963	NM_175506	ENSMUSG00000053441	**Adamts19**	4831407I23Rik|AU015154|D230034E10Rik	a disintegrin-like and metallopeptidase (reprolysin type) with thrombospondin type 1 motif, 19	LOSS	protein-coding
1674_T12,T22,T2,T3	chr18	5.9E+07	6E+07	3999	+	**4**	**50**	NA	intron (NM_175506, intron 2 of 22)	L1Md_A|LINE|L1	19736	NM_175506	240322	Mm.71963	NM_175506	ENSMUSG00000053441	**Adamts19**	4831407I23Rik|AU015154|D230034E10Rik	a disintegrin-like and metallopeptidase (reprolysin type) with thrombospondin type 1 motif, 19	LOSS	protein-coding
2066_T12,T1,T22,T2	chr18	8E+07	8E+07	290999	+	**4**	**50**	NA	Intergenic	(TG)n|Simple_repeat|Simple_repeat	-9518	NM_175028	240442	Mm.26594	NM_175028	ENSMUSG00000053950	**Adnp2**	8430420L05Rik|BC024969|Kiaa0863|Zfp508|mKIAA0863	ADNP homeobox 2	LOSS	protein-coding
1736_T12,T1,T2,T4	chr18	6.2E+07	6E+07	6999	+	**4**	**50**	NA	Intergenic	L1_Rod|LINE|L1	-72019	NM_007420	11555	Mm.5598	NM_007420	ENSMUSG00000045730	**Adrb2**	Adrb-2|Badm|Gpcr7	adrenergic receptor, beta 2	LOSS	protein-coding
1218_T12,T1,T2,T32	chr18	3.7E+07	4E+07	4999	+	**4**	**50**	NA	exon (NM_175375, exon 1 of 34)	exon (NM_175375, exon 1 of 34)	397	NM_175375	108857	Mm.482287	NM_175375	ENSMUSG00000024483	**Ankhd1**	1110004O12Rik|4933432B13Rik|9130019P20Rik|A530027J04Rik|A630021B20Rik|AA571404|Mask|mFLJ00246|mKIAA1085	ankyrin repeat and KH domain containing 1	LOSS	protein-coding
289_T22,T2,T3,T4	chr14	3.2E+07	3E+07	17999	+	**4**	**50**	NA	Intergenic	Intergenic	-5085	NM_001024604	105522	Mm.37660	NM_001024604	ENSMUSG00000014496	**Ankrd28**	AI465466|AI604979|E430019N21Rik|mKIAA0379	ankyrin repeat domain 28	LOSS	protein-coding
287_T22,T2,T42,T4	chr14	3.2E+07	3E+07	999	+	**4**	**50**	NA	intron (NM_001024604, intron 1 of 27)	intron (NM_001024604, intron 1 of 27)	21415	NM_001024604	105522	Mm.37660	NM_001024604	ENSMUSG00000014496	**Ankrd28**	AI465466|AI604979|E430019N21Rik|mKIAA0379	ankyrin repeat domain 28	LOSS	protein-coding
285_T22,T2,T42,T4	chr14	3.2E+07	3E+07	999	+	**4**	**50**	NA	intron (NM_001024604, intron 6 of 27)	intron (NM_001024604, intron 6 of 27)	84415	NM_001024604	105522	Mm.37660	NM_001024604	ENSMUSG00000014496	**Ankrd28**	AI465466|AI604979|E430019N21Rik|mKIAA0379	ankyrin repeat domain 28	LOSS	protein-coding
1424_T12,T22,T32,T3	chr18	4.7E+07	5E+07	999	+	**4**	**50**	NA	intron (NM_001303015, intron 1 of 6)	intron (NM_001303015, intron 1 of 6)	6124	NM_001303015	11777	Mm.312536	NM_009681	ENSMUSG00000024480	**Ap3s1**	-	adaptor-related protein complex 3, sigma 1 subunit	LOSS	protein-coding

1742_T12,T1,T2,T32	chr18	6.3E+07	6E+07	277999	+	**4**	**50**	NA	Intergenic	Intergenic	-27827	NM_133237	494504	Mm.391102	NM_133237	ENSMUSG00000071847	**Apcdd1**	AB023957|AU041258|Drapc1|EIG180	adenomatosis polyposis coli down-regulated 1	LOSS	protein-coding
787_T12,T1,T2,T32	chr18	1.5E+07	2E+07	130999	+	**4**	**50**	NA	Intergenic	L1_Mus4|LINE|L1	104016	NM_001308647	11829	Mm.250786	NM_009700	ENSMUSG00000024411	**Aqp4**	WCH4	aquaporin 4	LOSS	protein-coding
793_T12,T1,T2,T32	chr18	1.6E+07	2E+07	65999	+	**4**	**50**	NA	intron (NM_199055, intron 2 of 3)	intron (NM_199055, intron 2 of 3)	-153518	NM_001308646	11829	Mm.250786	NM_009700	ENSMUSG00000024411	**Aqp4**	WCH4	aquaporin 4	LOSS	protein-coding
791_T12,T1,T2,T4	chr18	1.5E+07	2E+07	5999	+	**4**	**50**	NA	intron (NM_199055, intron 3 of 3)	intron (NM_199055, intron 3 of 3)	-69518	NM_001308646	11829	Mm.250786	NM_009700	ENSMUSG00000024411	**Aqp4**	WCH4	aquaporin 4	LOSS	protein-coding
251_T22,T2,T32,T3	chr14	2.7E+07	3E+07	1999	+	**4**	**50**	NA	3' UTR (NM_134437, exon 13 of 13)	3' UTR (NM_134437, exon 13 of 13)	-41493	NM_001289686	71704	Mm.248606	NM_027871	ENSMUSG00000021895	**Arhgef3**	1200004I24Rik|9830169H03Rik|C76747|Xpln	Rho guanine nucleotide exchange factor (GEF) 3	LOSS	protein-coding
255_T2,T32,T3,T4	chr14	2.7E+07	3E+07	17999	+	**4**	**50**	NA	intron (NM_001289686, intron 4 of 12)	intron (NM_001289686, intron 4 of 12)	33490	NM_027871	71704	Mm.248606	NM_027871	ENSMUSG00000021895	**Arhgef3**	1200004I24Rik|9830169H03Rik|C76747|Xpln	Rho guanine nucleotide exchange factor (GEF) 3	LOSS	protein-coding
250_T22,T32,T3,T42	chr14	2.7E+07	3E+07	999	+	**4**	**50**	NA	intron (NM_134437, intron 12 of 12)	intron (NM_134437, intron 12 of 12)	-42993	NM_001289686	71704	Mm.248606	NM_027871	ENSMUSG00000021895	**Arhgef3**	1200004I24Rik|9830169H03Rik|C76747|Xpln	Rho guanine nucleotide exchange factor (GEF) 3	LOSS	protein-coding
1719_T12,T1,T32,T3	chr18	6.2E+07	6E+07	999	+	**4**	**50**	NA	intron (NM_177828, intron 3 of 12)	B3A|SINE|B2	17536	NM_177828	328967	Mm.488098	NM_177828	ENSMUSG00000045094	**Arhgef37**	4933429F08Rik	Rho guanine nucleotide exchange factor (GEF) 37	LOSS	protein-coding
671_T12,T1,T2,T32	chr18	7094501	8E+06	431999	+	**4**	**50**	NA	Intergenic	B1_Mur4|SINE|Alu	-12599	NM_001081393	74934	Mm.107918	NM_001081393	ENSMUSG00000061802	**Armc4**	4930463I21Rik|b2b227.1Clo|b2b643Clo	armadillo repeat containing 4	LOSS	protein-coding
964_T12,T1,T2,T32	chr18	2.2E+07	2E+07	106999	+	**4**	**50**	NA	intron (NM_001167777, intron 9 of 12)	intron (NM_001167777, intron 9 of 12)	155911	NM_001167777	211961	Mm.392310	NM_001167777	ENSMUSG00000045215	**Asxl3**	C230079D11Rik|D430002O22Rik|D930044O18Rik|Gm329|Gm945	additional sex combs like 3 (Drosophila)	LOSS	protein-coding
2076_T12,T1,T2,T32	chr18	8.1E+07	8E+07	8999	+	**4**	**50**	NA	intron (NM_001201569, intron 5 of 29)	L1MB7|LINE|L1	43058	NM_001201569	50771	Mm.247138	NM_015805	ENSMUSG00000024566	**Atp9b**	AA934181|Atpc2b|IIb|MMR	ATPase, class II, type 9B	LOSS	protein-coding
150_T22,T3,T42,T4	chr14	1.4E+07	1E+07	999	+	**4**	**50**	NA	Intergenic	Intergenic	-6491	NM_139227	246103	Mm.133625	NM_139227	ENSMUSG00000021738	**Atxn7**	A430107N12Rik|AI627028|Sca7|ataxin-7	ataxin 7	LOSS	protein-coding

283_T22,T2,T3,T4	chr14	3.2E+07	3E+07	999	+	**4**	**50**	NA	intron (NM_001024604, intron 12 of 27)	intron (NM_001024604, intron 12 of 27)	88943	NM_025295	26363	Mm.282679	NM_025295	ENSMUSG00000021900	**Btd**	-	biotinidase	LOSS	protein-coding
2170_T12,T1,T2,T42	chr18	8.7E+07	9E+07	7999	+	**4**	**50**	NA	Intergenic	MT2A|LTR|ERVL	-120610	NM_001302356	12405	Mm.70775	NM_172633	ENSMUSG00000024647	**Cbln2**	6330593N19Rik|A730004O05	cerebellin 2 precursor protein	LOSS	protein-coding
2180_T12,T1,T22,T2	chr18	8.7E+07	9E+07	13999	+	**4**	**50**	NA	Intergenic	Lx3A|LINE|L1	219435	NM_172633	12405	Mm.70775	NM_172633	ENSMUSG00000024647	**Cbln2**	6330593N19Rik|A730004O05	cerebellin 2 precursor protein	LOSS	protein-coding
2184_T12,T1,T2,T32	chr18	8.7E+07	9E+07	174999	+	**4**	**50**	NA	Intergenic	Lx6|LINE|L1	354935	NM_172633	12405	Mm.70775	NM_172633	ENSMUSG00000024647	**Cbln2**	6330593N19Rik|A730004O05	cerebellin 2 precursor protein	LOSS	protein-coding
2186_T12,T1,T2,T32	chr18	8.7E+07	9E+07	26999	+	**4**	**50**	NA	Intergenic	Lx2|LINE|L1	461935	NM_172633	12405	Mm.70775	NM_172633	ENSMUSG00000024647	**Cbln2**	6330593N19Rik|A730004O05	cerebellin 2 precursor protein	LOSS	protein-coding
2189_T12,T1,T2,T32	chr18	8.7E+07	9E+07	151999	+	**4**	**50**	NA	Intergenic	B2_Mm2|SINE|B2	557435	NM_172633	12405	Mm.70775	NM_172633	ENSMUSG00000024647	**Cbln2**	6330593N19Rik|A730004O05	cerebellin 2 precursor protein	LOSS	protein-coding
2172_T12,T1,T22,T2	chr18	8.7E+07	9E+07	95999	+	**4**	**50**	NA	intron (NM_001302356, intron 4 of 4)	intron (NM_001302356, intron 4 of 4)	4435	NM_172633	12405	Mm.70775	NM_172633	ENSMUSG00000024647	**Cbln2**	6330593N19Rik|A730004O05	cerebellin 2 precursor protein	LOSS	protein-coding
260_T22,T2,T3,T42	chr14	2.8E+07	3E+07	3999	+	**4**	**50**	NA	Intergenic	Intergenic	-14040	NM_177111	320234	Mm.247767	NM_177111	ENSMUSG00000046753	**Ccdc66**	E230015L20Rik	coiled-coil domain containing 66	LOSS	protein-coding
1846_T12,T1,T22,T32	chr18	7E+07	7E+07	3999	+	**4**	**50**	NA	3' UTR (NM_030554, exon 6 of 6)	3' UTR (NM_030554, exon 6 of 6)	55941	NM_201362	381175	Mm.266831	NM_201362	ENSMUSG00000038903	**Ccdc68**	BC046343	coiled-coil domain containing 68	LOSS	protein-coding
710_T12,T1,T2,T42	chr18	9531501	1E+07	4999	+	**4**	**50**	NA	Intergenic	L1M2|LINE|L1	-83850	NM_026484	67974	Mm.86523	NM_026484	ENSMUSG00000024286	**Ccny**	1700025H17Rik|3110050L10Rik|4631402G10Rik|5730405I09Rik	cyclin Y	LOSS	protein-coding
2235_T12,T1,T2,T32	chr18	8.9E+07	9E+07	30999	+	**4**	**50**	NA	intron (NM_001039173, intron 7 of 7)	Lx9|LINE|L1	195573	NM_001039149	225825	Mm.139293	NM_178687	ENSMUSG00000034028	**Cd226**	BC051526|DNAM-1|DNAM1|Pta1|TLiSA1	CD226 antigen	LOSS	protein-coding
1714_T12,T1,T2,T32	chr18	6.1E+07	6E+07	19999	+	**4**	**50**	NA	Intergenic	Intergenic	-11349	NM_001042605	16149	Mm.439737	NM_010545	ENSMUSG00000024610	**Cd74**	CLIP|DHLAG|HLADG|Ia-GAMMA|Ii	CD74 antigen (invariant polypeptide of major histocompatibility complex, class II antigen-associated)	LOSS	protein-coding
813_T12,T1,T2,T32	chr18	1.6E+07	2E+07	199999	+	**4**	**50**	NA	Intergenic	Intergenic	419746	NM_007664	12558	Mm.257437	NM_007664	ENSMUSG00000024304	**Cdh2**	CDHN|N-CAD|Ncad	cadherin 2	LOSS	protein-coding

815_T12,T1,T2,T32	chr18	1.6E+07	2E+07	354999	+	**4**	**50**	NA	intron (NM_007664, intron 2 of 15)	intron (NM_007664, intron 2 of 15)	136246	NM_007664	12558	Mm.257437	NM_007664	ENSMUSG00000024304	**Cdh2**	CDHN|N-CAD|Ncad	cadherin 2	LOSS	protein-coding
1421_T12,T22,T32,T3	chr18	4.7E+07	5E+07	999	+	**4**	**50**	NA	intron (NM_033037, intron 3 of 4)	intron (NM_033037, intron 3 of 4)	12342	NM_033037	12583	Mm.241056	NM_033037	ENSMUSG00000033022	**Cdo1**	1300002L19Rik|Cdo|D18Ucla3	cysteine dioxygenase 1, cytosolic	LOSS	protein-coding
1028_T12,T1,T2,T32	chr18	2.7E+07	3E+07	8999	+	**4**	**50**	NA	Intergenic	Intergenic	-1E+06	NM_001174074	108013	Mm.266435	NM_133195	ENSMUSG00000024268	**Celf4**	A230070D14Rik|BRUNOL-4|Brul4|Brunol4|C130060B05Rik	CUGBP, Elav-like family member 4	LOSS	protein-coding
1033_T12,T1,T2,T32	chr18	2.7E+07	3E+07	64999	+	**4**	**50**	NA	Intergenic	Lx9|LINE|L1	-1E+06	NM_001174074	108013	Mm.266435	NM_133195	ENSMUSG00000024268	**Celf4**	A230070D14Rik|BRUNOL-4|Brul4|Brunol4|C130060B05Rik	CUGBP, Elav-like family member 4	LOSS	protein-coding
1010_T12,T1,T2,T32	chr18	2.5E+07	3E+07	4999	+	**4**	**50**	NA	intron (NM_001146292, intron 12 of 12)	intron (NM_001146292, intron 12 of 12)	273983	NM_133195	108013	Mm.266435	NM_133195	ENSMUSG00000024268	**Celf4**	A230070D14Rik|BRUNOL-4|Brul4|Brunol4|C130060B05Rik	CUGBP, Elav-like family member 4	LOSS	protein-coding
1012_T12,T1,T2,T32	chr18	2.5E+07	3E+07	275999	+	**4**	**50**	NA	intron (NM_001146292, intron 2 of 12)	intron (NM_001146292, intron 2 of 12)	132483	NM_133195	108013	Mm.266435	NM_133195	ENSMUSG00000024268	**Celf4**	A230070D14Rik|BRUNOL-4|Brul4|Brunol4|C130060B05Rik	CUGBP, Elav-like family member 4	LOSS	protein-coding
804_T12,T1,T2,T32	chr18	1.6E+07	2E+07	54999	+	**4**	**50**	NA	Intergenic	Intergenic	-46954	NM_199055	71367	Mm.329304	NM_199055	ENSMUSG00000047161	**Chst9**	5430438D01Rik	carbohydrate (N-acetylgalactosamine 4-0) sulfotransferase 9	LOSS	protein-coding
806_T12,T1,T32,T3	chr18	1.6E+07	2E+07	2999	+	**4**	**50**	NA	Intergenic	Lx6|LINE|L1	-189954	NM_199055	71367	Mm.329304	NM_199055	ENSMUSG00000047161	**Chst9**	5430438D01Rik	carbohydrate (N-acetylgalactosamine 4-0) sulfotransferase 9	LOSS	protein-coding
812_T12,T1,T2,T32	chr18	1.6E+07	2E+07	293999	+	**4**	**50**	NA	Intergenic	RSINE1|SINE|B4	-417454	NM_199055	71367	Mm.329304	NM_199055	ENSMUSG00000047161	**Chst9**	5430438D01Rik	carbohydrate (N-acetylgalactosamine 4-0) sulfotransferase 9	LOSS	protein-coding
795_T12,T1,T2,T32	chr18	1.6E+07	2E+07	1999	+	**4**	**50**	NA	intron (NM_199055, intron 1 of 3)	intron (NM_199055, intron 1 of 3)	69546	NM_199055	71367	Mm.329304	NM_199055	ENSMUSG00000047161	**Chst9**	5430438D01Rik	carbohydrate (N-acetylgalactosamine 4-0) sulfotransferase 9	LOSS	protein-coding
798_T12,T1,T2,T32	chr18	1.6E+07	2E+07	30999	+	**4**	**50**	NA	intron (NM_199055, intron 1 of 3)	intron (NM_199055, intron 1 of 3)	47046	NM_199055	71367	Mm.329304	NM_199055	ENSMUSG00000047161	**Chst9**	5430438D01Rik	carbohydrate (N-acetylgalactosamine 4-0) sulfotransferase 9	LOSS	protein-coding

1680_T12,T1,T2,T32	chr18	5.9E+07	6E+07	349999	+	**4**	**50**	NA	intron (NM_001081328, intron 2 of 2)	L1Md_A|LINE|L1	122160	NM_001081328	78923	Mm.84007	NM_001081328	ENSMUSG00000058152	**Chsy3**	4833446K15Rik|AI662215	chondroitin sulfate synthase 3	LOSS	protein-coding
716_T12,T22,T2,T3	chr18	9825501	1E+07	999	+	**4**	**50**	NA	intron (NM_130449, intron 2 of 9)	L1_Mus3|LINE|L1	118352	NM_130449	140792	Mm.218571	NM_130449	ENSMUSG00000036103	**Colec12**	CL-P1|SRCL|Scara4	collectin sub-family member 12	LOSS	protein-coding
1726_T12,T1,T2,T32	chr18	6.2E+07	6E+07	37999	+	**4**	**50**	NA	intron (NM_146087, intron 9 of 9)	intron (NM_146087, intron 9 of 9)	27918	NM_146087	93687	Mm.26908	NM_146087	ENSMUSG00000024576	**Csnk1a1**	2610208K14Rik|4632404G05Rik|5430427P18Rik|CK1a|Csnk1a	casein kinase 1, alpha 1	LOSS	protein-coding
1608_T12,T22,T2,T3	chr18	5.4E+07	5E+07	30999	+	**4**	**50**	NA	Intergenic	L1Md_F2|LINE|L1	164887	NM_152809	70425	Mm.368668	NM_152809	ENSMUSG00000073563	**Csnk1g3**	3300002K07Rik|C330049O21Rik	casein kinase 1, gamma 3	LOSS	protein-coding
1601_T12,T22,T32,T3	chr18	5.4E+07	5E+07	7999	+	**4**	**50**	NA	intron (NM_152809, intron 1 of 12)	MMERVK10C-int|LTR|ERVK	10387	NM_152809	70425	Mm.368668	NM_152809	ENSMUSG00000073563	**Csnk1g3**	3300002K07Rik|C330049O21Rik	casein kinase 1, gamma 3	LOSS	protein-coding
1603_T12,T22,T32,T3	chr18	5.4E+07	5E+07	999	+	**4**	**50**	NA	intron (NM_152809, intron 1 of 12)	intron (NM_152809, intron 1 of 12)	26887	NM_152809	70425	Mm.368668	NM_152809	ENSMUSG00000073563	**Csnk1g3**	3300002K07Rik|C330049O21Rik	casein kinase 1, gamma 3	LOSS	protein-coding
1605_T12,T22,T32,T3	chr18	5.4E+07	5E+07	999	+	**4**	**50**	NA	intron (NM_152809, intron 6 of 12)	intron (NM_152809, intron 6 of 12)	62887	NM_152809	70425	Mm.368668	NM_152809	ENSMUSG00000073563	**Csnk1g3**	3300002K07Rik|C330049O21Rik	casein kinase 1, gamma 3	LOSS	protein-coding
1968_T12,T1,T2,T32	chr18	7.5E+07	8E+07	416999	+	**4**	**50**	NA	intron (NM_201354, intron 1 of 11)	intron (NM_201354, intron 1 of 11)	21696	NM_201354	269037	Mm.36745	NM_201354	ENSMUSG00000052928	**Ctif**	Gm672	CBP80/20-dependent translation initiation factor	LOSS	protein-coding
577_T12,T22,T2,T3	chr14	5.4E+07	5E+07	16999	+	**4**	**50**	NA	Intergenic	Intergenic	108929	NM_001113358	13135	Mm.319038	NM_010015	ENSMUSG00000022174	**Dad1**	AI323713	defender against cell death 1	LOSS	protein-coding
579_T12,T22,T2,T3	chr14	5.4E+07	5E+07	3999	+	**4**	**50**	NA	intron (NM_001113358, intron 2 of 2)	PB1D10|SINE|Alu	15429	NM_001113358	13135	Mm.319038	NM_010015	ENSMUSG00000022174	**Dad1**	AI323713	defender against cell death 1	LOSS	protein-coding
1919_T12,T1,T2,T32	chr18	7.2E+07	7E+07	61999	+	**4**	**50**	NA	Intergenic	Intergenic	-30431	NM_007831	13176	Mm.167882	NM_007831	ENSMUSG00000060534	**Dcc**	C030036D22Rik|Igdcc1	deleted in colorectal carcinoma	LOSS	protein-coding
1930_T12,T1,T22,T2	chr18	7.3E+07	7E+07	18999	+	**4**	**50**	NA	Intergenic	Intergenic	-434931	NM_007831	13176	Mm.167882	NM_007831	ENSMUSG00000060534	**Dcc**	C030036D22Rik|Igdcc1	deleted in colorectal carcinoma	LOSS	protein-coding

1887_T12,T1,T2,T32	chr18	7.1E+07	7E+07	21999	+	**4**	**50**	NA	intron (NM_007831, intron 12 of 28)	intron (NM_007831, intron 12 of 28)	862569	NM_007831	13176	Mm.167882	NM_007831	ENSMUSG00000060534	**Dcc**	C030036D22Rik|Igdcc1	deleted in colorectal carcinoma	LOSS	protein-coding
1889_T12,T1,T2,T32	chr18	7.2E+07	7E+07	9999	+	**4**	**50**	NA	intron (NM_007831, intron 12 of 28)	L1Md_F2|LINE|L1	817569	NM_007831	13176	Mm.167882	NM_007831	ENSMUSG00000060534	**Dcc**	C030036D22Rik|Igdcc1	deleted in colorectal carcinoma	LOSS	protein-coding
1912_T12,T1,T2,T32	chr18	7.2E+07	7E+07	2999	+	**4**	**50**	NA	intron (NM_007831, intron 2 of 28)	intron (NM_007831, intron 2 of 28)	408069	NM_007831	13176	Mm.167882	NM_007831	ENSMUSG00000060534	**Dcc**	C030036D22Rik|Igdcc1	deleted in colorectal carcinoma	LOSS	protein-coding
1902_T1,T22,T32,T42	chr18	7.2E+07	7E+07	999	+	**4**	**50**	NA	intron (NM_007831, intron 5 of 28)	(CAAAA)n|Simple_repeat|Simple_repeat	619069	NM_007831	13176	Mm.167882	NM_007831	ENSMUSG00000060534	**Dcc**	C030036D22Rik|Igdcc1	deleted in colorectal carcinoma	LOSS	protein-coding
1892_T12,T1,T2,T32	chr18	7.2E+07	7E+07	52999	+	**4**	**50**	NA	intron (NM_007831, intron 8 of 28)	intron (NM_007831, intron 8 of 28)	775069	NM_007831	13176	Mm.167882	NM_007831	ENSMUSG00000060534	**Dcc**	C030036D22Rik|Igdcc1	deleted in colorectal carcinoma	LOSS	protein-coding
1370_T12,T1,T22,T2	chr18	4.4E+07	4E+07	24999	+	**4**	**50**	NA	Intergenic	Intergenic	-17500	NM_027490	70640	Mm.87629	NM_027490	ENSMUSG00000024472	**Dcp2**	2410015D23Rik|5730537H01Rik|AL118268	decapping mRNA 2	LOSS	protein-coding
471_T12,T22,T2,T4	chr14	4.6E+07	5E+07	2999	+	**4**	**50**	NA	Intergenic	MERVL_2A-int|LTR|ERVL	-67857	NM_001042719	114874	Mm.121918	NM_176845	ENSMUSG00000037697	**Ddhd1**	Mir5131|PA-PLA1	DDHD domain containing 1	LOSS	protein-coding
594_T12,T22,T2,T4	chr14	5.5E+07	6E+07	88999	+	**4**	**50**	NA	Intergenic	Intergenic	52993	NM_027790	71412	Mm.85484	NM_027790	ENSMUSG00000022209	**Dhrs2**	5430405K24Rik|Hep27	dehydrogenase/reductase member 2	LOSS	protein-coding
2245_T12,T1,T22,T32	chr18	9E+07	9E+07	5999	+	**4**	**50**	NA	Intergenic	Intergenic	-41364	NM_001039173	623279	Mm.447164	NM_001039173	ENSMUSG00000073514	**Dok6**	Dok-6	docking protein 6	LOSS	protein-coding
2247_T12,T1,T2,T32	chr18	9E+07	9E+07	999	+	**4**	**50**	NA	Intergenic	ORR1A2-int|LTR|ERVL-MaLR	-65864	NM_001039173	623279	Mm.447164	NM_001039173	ENSMUSG00000073514	**Dok6**	Dok-6	docking protein 6	LOSS	protein-coding
2249_T1,T22,T2,T32	chr18	9E+07	9E+07	999	+	**4**	**50**	NA	Intergenic	Lx2|LINE|L1	-82864	NM_001039173	623279	Mm.447164	NM_001039173	ENSMUSG00000073514	**Dok6**	Dok-6	docking protein 6	LOSS	protein-coding
2251_T12,T1,T22,T2	chr18	9E+07	9E+07	4999	+	**4**	**50**	NA	Intergenic	L1Md_F2|LINE|L1	-97864	NM_001039173	623279	Mm.447164	NM_001039173	ENSMUSG00000073514	**Dok6**	Dok-6	docking protein 6	LOSS	protein-coding
2254_T12,T1,T22,T2	chr18	9E+07	9E+07	11999	+	**4**	**50**	NA	Intergenic	Intergenic	-112364	NM_001039173	623279	Mm.447164	NM_001039173	ENSMUSG00000073514	**Dok6**	Dok-6	docking protein 6	LOSS	protein-coding
2258_T12,T2,T32,T3	chr18	9E+07	9E+07	3999	+	**4**	**50**	NA	Intergenic	Lx9|LINE|L1	-222364	NM_001039173	623279	Mm.447164	NM_001039173	ENSMUSG00000073514	**Dok6**	Dok-6	docking protein 6	LOSS	protein-coding

2265_T12,T22,T2,T3	chr18	9E+07	9E+07	999	+	**4**	**50**	NA	Intergenic	Lx7|LINE|L1	-284864	NM_001039173	623279	Mm.447164	NM_001039173	ENSMUSG00000073514	**Dok6**	Dok-6	docking protein 6	LOSS	protein-coding
2269_T12,T1,T2,T32	chr18	9E+07	9E+07	19999	+	**4**	**50**	NA	Intergenic	Lx7|LINE|L1	-333364	NM_001039173	623279	Mm.447164	NM_001039173	ENSMUSG00000073514	**Dok6**	Dok-6	docking protein 6	LOSS	protein-coding
2271_T12,T1,T2,T32	chr18	9E+07	9E+07	40999	+	**4**	**50**	NA	Intergenic	Intergenic	-369864	NM_001039173	623279	Mm.447164	NM_001039173	ENSMUSG00000073514	**Dok6**	Dok-6	docking protein 6	LOSS	protein-coding
1344_T12,T1,T2,T32	chr18	4.3E+07	4E+07	132999	+	**4**	**50**	NA	intron (NM_001291455, intron 1 of 13)	MLT1A0|LTR|ERVL-MaLR	-11623	NM_009468	22240	Mm.8180	NM_009468	ENSMUSG00000024501	**Dpysl3**	CRMP-4|DRP-3|TUC4|ULIP-1|Ulip|Ulip1	dihydropyrimidinase-like 3	LOSS	protein-coding
921_T12,T1,T2,T32	chr18	2E+07	2E+07	475999	+	**4**	**50**	NA	Intergenic	Intergenic	-10727	NM_013504	13505	Mm.33740	NM_013504	ENSMUSG00000044322	**Dsc1**	1110020A10Rik|AI507491	desmocollin 1	LOSS	protein-coding
878_T1,T2,T32,T3	chr18	1.9E+07	2E+07	999	+	**4**	**50**	NA	Intergenic	MTA_Mm-int|LTR|ERVL-MaLR	1143097	NM_007882	13507	Mm.89935	NM_007882	ENSMUSG00000059898	**Dsc3**	5430426I24Rik	desmocollin 3	LOSS	protein-coding
880_T1,T2,T32,T3	chr18	1.9E+07	2E+07	999	+	**4**	**50**	NA	Intergenic	Intergenic	1126097	NM_007882	13507	Mm.89935	NM_007882	ENSMUSG00000059898	**Dsc3**	5430426I24Rik	desmocollin 3	LOSS	protein-coding
882_T12,T1,T2,T3	chr18	1.9E+07	2E+07	44999	+	**4**	**50**	NA	Intergenic	Intergenic	987097	NM_007882	13507	Mm.89935	NM_007882	ENSMUSG00000059898	**Dsc3**	5430426I24Rik	desmocollin 3	LOSS	protein-coding
890_T1,T2,T32,T3	chr18	1.9E+07	2E+07	999	+	**4**	**50**	NA	Intergenic	Intergenic	773097	NM_007882	13507	Mm.89935	NM_007882	ENSMUSG00000059898	**Dsc3**	5430426I24Rik	desmocollin 3	LOSS	protein-coding
892_T12,T1,T2,T3	chr18	1.9E+07	2E+07	999	+	**4**	**50**	NA	Intergenic	L1Md_A|LINE|L1	719097	NM_007882	13507	Mm.89935	NM_007882	ENSMUSG00000059898	**Dsc3**	5430426I24Rik	desmocollin 3	LOSS	protein-coding
895_T12,T1,T22,T2	chr18	1.9E+07	2E+07	999	+	**4**	**50**	NA	Intergenic	L1Md_A|LINE|L1	649097	NM_007882	13507	Mm.89935	NM_007882	ENSMUSG00000059898	**Dsc3**	5430426I24Rik	desmocollin 3	LOSS	protein-coding
897_T12,T1,T22,T2	chr18	1.9E+07	2E+07	999	+	**4**	**50**	NA	Intergenic	MYSERV6-int|LTR|ERVK	572097	NM_007882	13507	Mm.89935	NM_007882	ENSMUSG00000059898	**Dsc3**	5430426I24Rik	desmocollin 3	LOSS	protein-coding
899_T12,T1,T2,T32	chr18	1.9E+07	2E+07	5999	+	**4**	**50**	NA	Intergenic	ORR1A2|LTR|ERVL-MaLR	563597	NM_007882	13507	Mm.89935	NM_007882	ENSMUSG00000059898	**Dsc3**	5430426I24Rik	desmocollin 3	LOSS	protein-coding
903_T12,T1,T2,T32	chr18	1.9E+07	2E+07	88999	+	**4**	**50**	NA	Intergenic	B3A|SINE|B2	509097	NM_007882	13507	Mm.89935	NM_007882	ENSMUSG00000059898	**Dsc3**	5430426I24Rik	desmocollin 3	LOSS	protein-coding
909_T12,T1,T2,T42	chr18	2E+07	2E+07	999	+	**4**	**50**	NA	Intergenic	RLTR47|LTR|ERVK	411097	NM_007882	13507	Mm.89935	NM_007882	ENSMUSG00000059898	**Dsc3**	5430426I24Rik	desmocollin 3	LOSS	protein-coding
917_T12,T1,T2,T32	chr18	2E+07	2E+07	999	+	**4**	**50**	NA	Intergenic	L1_Mus3|LINE|L1	126097	NM_007882	13507	Mm.89935	NM_007882	ENSMUSG00000059898	**Dsc3**	5430426I24Rik	desmocollin 3	LOSS	protein-coding

927_T12,T1,T2,T32	chr18	2.1E+07	2E+07	2999	+	**4**	**50**	NA	Intergenic	B4|SINE|B4	-1304	NM_030596	13512	Mm.106811	NM_030596	ENSMUSG00000056632	**Dsg3**	bal	desmoglein 3	LOSS	protein-coding
925_T12,T1,T2,T32	chr18	2E+07	2E+07	112999	+	**4**	**50**	NA	intron (NM_181564, intron 1 of 15)	intron (NM_181564, intron 1 of 15)	5825	NM_181564	16769	Mm.358619	NM_181564	ENSMUSG00000001804	**Dsg4**	CDHF13|lah	desmoglein 4	LOSS	protein-coding
977_T12,T1,T2,T32	chr18	2.3E+07	2E+07	379999	+	**4**	**50**	NA	intron (NM_207650, intron 10 of 20)	RLTR17B_Mm|LTR|ERVK	101018	NM_001285810	13527	Mm.94371	NM_010087	ENSMUSG00000024302	**Dtna**	2210407P21Rik|DTN-A|Dtn|Gm19389|a-DB-1|adbn	dystrobrevin alpha	LOSS	protein-coding
102_T12,T22,T2,T3	chr13	8.7E+07	9E+07	3999	+	**4**	**50**	NA	Intergenic	L1Md_T|LINE|L1	-1E+06	NM_010103	13612	Mm.125580	NM_010103	ENSMUSG00000034488	**Edil3**	Del-1|Del1	EGF-like repeats and discoidin I-like domains 3	LOSS	protein-coding
2017_T1,T32,T3,T42	chr18	7.8E+07	8E+07	999	+	**4**	**50**	NA	3' UTR (NM_013831, exon 15 of 15)	3' UTR (NM_013831, exon 15 of 15)	-56467	NM_001195633	1E+08	Mm.297782	NM_001195633	ENSMUSG00000039840	**Epg5**	4732475F16|5430411K18Rik|AI661957|AW456499|mKIAA1632	ectopic P-granules autophagy protein 5 homolog (C. elegans)	LOSS	protein-coding
1492_T1,T2,T32,T3	chr18	5E+07	5E+07	999	+	**4**	**50**	NA	Intergenic	Intergenic	-66369	NM_001004061	225497	Mm.329435	NM_001004061	ENSMUSG00000035420	**Fam170a**	Gm93|Znfd	family with sequence similarity 170, member A	LOSS	protein-coding
1495_T22,T2,T32,T3	chr18	5E+07	5E+07	999	+	**4**	**50**	NA	Intergenic	Intergenic	-53369	NM_001004061	225497	Mm.329435	NM_001004061	ENSMUSG00000035420	**Fam170a**	Gm93|Znfd	family with sequence similarity 170, member A	LOSS	protein-coding
1498_T12,T1,T2,T32	chr18	5E+07	5E+07	262999	+	**4**	**50**	NA	Intergenic	Intergenic	121631	NM_001004061	225497	Mm.329435	NM_001004061	ENSMUSG00000035420	**Fam170a**	Gm93|Znfd	family with sequence similarity 170, member A	LOSS	protein-coding
1659_T12,T22,T2,T3	chr18	5.8E+07	6E+07	4999	+	**4**	**50**	NA	Intergenic	L1Md_F3|LINE|L1	-47074	NM_010181	14119	Mm.20271	NM_010181	ENSMUSG00000024598	**Fbn2**	BC063774|Fib-2|mKIAA4226|sne|sy	fibrillin 2	LOSS	protein-coding
1665_T1,T32,T3,T42	chr18	5.8E+07	6E+07	999	+	**4**	**50**	NA	Intergenic	Intergenic	-162074	NM_010181	14119	Mm.20271	NM_010181	ENSMUSG00000024598	**Fbn2**	BC063774|Fib-2|mKIAA4226|sne|sy	fibrillin 2	LOSS	protein-coding
1739_T12,T1,T2,T32	chr18	6.2E+07	6E+07	325999	+	**4**	**50**	NA	intron (NM_008313, intron 6 of 6)	intron (NM_008313, intron 6 of 6)	92243	NM_134136	107035	Mm.21295	NM_134136	ENSMUSG00000042211	**Fbxo38**	6030410I24Rik|AU044865|AW214031|Moka|SP329	F-box protein 38	LOSS	protein-coding
467_T12,T22,T3,T4	chr14	4.6E+07	5E+07	2999	+	**4**	**50**	NA	promoter-TSS (NM_146054)	promoter-TSS (NM_146054)	65	NM_146054	218952	Mm.210018	NM_146054	ENSMUSG00000037712	**Fermt2**	AA960555|Kindlin-2|Mig2|Plekhc1	fermitin family member 2	LOSS	protein-coding
134_T22,T3,T42,T4	chr14	1E+07	1E+07	999	+	**4**	**50**	NA	intron (NM_001308286, intron 2 of 6)	ORR1E|LTR|ERVL-MaLR	275458	NM_001308286	14198	Mm.397619	NM_010210	ENSMUSG00000060579	**Fhit**	AW045638|Fra14A2	fragile histidine triad gene	LOSS	protein-coding
988_T12,T1,T2,T4	chr18	2.5E+07	2E+07	13999	+	**4**	**50**	NA	intron (NM_175276, intron 1 of 26)	Lx2A|LINE|L1	12877	NM_001289655	225288	Mm.329322	NM_175276	ENSMUSG00000034295	**Fhod3**	A930009H06Rik|FHOS2|mKIAA1695	formin homology 2 domain containing 3	LOSS	protein-coding

2094_T12,T1,T2,T32	chr18	8.2E+07	8E+07	19999	+	**4**	**50**	NA	Intergenic	Intergenic	151277	NM_008082	14427	Mm.6219	NM_008082	ENSMUSG00000024553	**Galr1**	Galnr1	galanin receptor 1	LOSS	protein-coding
2097_T12,T1,T2,T32	chr18	8.2E+07	8E+07	177999	+	**4**	**50**	NA	Intergenic	B4|SINE|B4	46277	NM_008082	14427	Mm.6219	NM_008082	ENSMUSG00000024553	**Galr1**	Galnr1	galanin receptor 1	LOSS	protein-coding
944_T12,T2,T32,T42	chr18	2.1E+07	2E+07	999	+	**4**	**50**	NA	intron (NM_001033445, intron 2 of 5)	B2_Mm1a|SINE|B2	103139	NM_001033445	381126	Mm.312276	NM_001033445	ENSMUSG00000042680	**Garem1**	C86169|Fam59a|Garem|Gm944|mKIAA4238	GRB2 associated regulator of MAPK1 subtype 1	LOSS	protein-coding
728_T12,T1,T2,T4	chr18	1.1E+07	1E+07	1999	+	**4**	**50**	NA	Intergenic	RMER19A|LTR|ERVK	-33010	NM_010258	14465	Mm.329287	NM_010258	ENSMUSG00000005836	**Gata6**	AA410133|GATA-6	GATA binding protein 6	LOSS	protein-coding
731_T1,T22,T2,T4	chr18	1.1E+07	1E+07	999	+	**4**	**50**	NA	Intergenic	Intergenic	-22510	NM_010258	14465	Mm.329287	NM_010258	ENSMUSG00000005836	**Gata6**	AA410133|GATA-6	GATA binding protein 6	LOSS	protein-coding
733_T12,T22,T2,T4	chr18	1.1E+07	1E+07	999	+	**4**	**50**	NA	TTS (NR_105023)	TTS (NR_105023)	-10510	NM_010258	14465	Mm.329287	NM_010258	ENSMUSG00000005836	**Gata6**	AA410133|GATA-6	GATA binding protein 6	LOSS	protein-coding
361_T12,T22,T2,T3	chr14	3.8E+07	4E+07	3999	+	**4**	**50**	NA	Intergenic	Intergenic	-502178	NM_078478	66092	Mm.182912	NM_078478	ENSMUSG00000041028	**Ghitm**	1010001P14Rik|C77840|MICS1|PTD010|Tmbim5	growth hormone inducible transmembrane protein	LOSS	protein-coding
363_T12,T22,T2,T3	chr14	3.8E+07	4E+07	88999	+	**4**	**50**	NA	Intergenic	Intergenic	-586678	NM_078478	66092	Mm.182912	NM_078478	ENSMUSG00000041028	**Ghitm**	1010001P14Rik|C77840|MICS1|PTD010|Tmbim5	growth hormone inducible transmembrane protein	LOSS	protein-coding
365_T12,T2,T3,T4	chr14	3.8E+07	4E+07	4999	+	**4**	**50**	NA	Intergenic	Intergenic	-661678	NM_078478	66092	Mm.182912	NM_078478	ENSMUSG00000041028	**Ghitm**	1010001P14Rik|C77840|MICS1|PTD010|Tmbim5	growth hormone inducible transmembrane protein	LOSS	protein-coding
707_T12,T1,T2,T32	chr18	9269501	9E+06	188999	+	**4**	**50**	NA	intron (NM_026484, intron 3 of 9)	L1_Mus3|LINE|L1	-81191	NM_153086	225152	Mm.119273	NM_153086	ENSMUSG00000036855	**Gjd4**	9430022F06Rik|Cx39	gap junction protein, delta 4	LOSS	protein-coding
275_T22,T2,T3,T42	chr14	3.1E+07	3E+07	64999	+	**4**	**50**	NA	intron (NM_001308328, intron 11 of 13)	intron (NM_001308328, intron 11 of 13)	-19393	NM_001165930	76485	Mm.8766	NM_029626	ENSMUSG00000021916	**Glt8d1**	2410004H05Rik|5430414N14Rik|AI450005	glycosyltransferase 8 domain containing 1	LOSS	protein-coding
453_T12,T22,T2,T4	chr14	4.5E+07	5E+07	1999	+	**4**	**50**	NA	intron (NM_027518, intron 1 of 6)	intron (NM_027518, intron 1 of 6)	18783	NM_027518	70713	Mm.343793	NM_027518	ENSMUSG00000049092	**Gpr137c**	6330416L11Rik|Gm908|TM7SF1L2	G protein-coupled receptor 137C	LOSS	protein-coding
1716_T12,T1,T2,T32	chr18	6.1E+07	6E+07	707999	+	**4**	**50**	NA	exon (NM_134134, exon 8 of 21)	exon (NM_134134, exon 8 of 21)	19549	NM_178277	106894	Mm.22574	NM_134134	ENSMUSG00000024622	**Hmgxb3**	2510002C16Rik|A630042L21Rik|AI413166|mKIAA0194	HMG box domain containing 3	LOSS	protein-coding
764_T12,T1,T2,T32	chr18	1.3E+07	1E+07	241999	+	**4**	**50**	NA	Intergenic	ORR1B2|LTR|ERVL-MaLR	447510	NM_153087	225192	Mm.207073	NM_153087	ENSMUSG00000037346	**Hrh4**	AXOR35|BG26|GPCR105|GPRv53|H4|H4R|HH4R	histamine receptor H4	LOSS	protein-coding

1202_T12,T1,T32,T3	chr18	3.6E+07	4E+07	999	+	**4**	**50**	NA	Intergenic	Intergenic	-2101	NM_001267796	109169	Mm.426423	NM_001267796	ENSMUSG00000110185	**Igip**	6330403M23Rik	IgA inducing protein	LOSS	protein-coding
1729_T12,T1,T2,T32	chr18	6.2E+07	6E+07	168999	+	**4**	**50**	NA	intron (NM_019508, intron 1 of 2)	intron (NM_019508, intron 1 of 2)	1065	NM_019508	56069	Mm.59313	NM_019508	ENSMUSG00000024578	**Il17b**	1110006O16Rik|1700006N07Rik|Zcyto7	interleukin 17B	LOSS	protein-coding
1346_T12,T1,T2,T32	chr18	4.3E+07	4E+07	230999	+	**4**	**50**	NA	intron (NM_001163637, intron 1 of 21)	Lx2|LINE|L1	93773	NM_001163637	76217	Mm.134602	NM_001163637	ENSMUSG00000024502	**Jakmip2**	6430702L21Rik|AI850334|D930046L20Rik	janus kinase and microtubule interacting protein 2	LOSS	protein-coding
223_T22,T2,T3,T4	chr14	2.2E+07	2E+07	6999	+	**4**	**50**	NA	intron (NM_001205241, intron 2 of 16)	intron (NM_001205241, intron 2 of 16)	90215	NM_017479	54169	Mm.248967	NM_017479	ENSMUSG00000021767	**Kat6b**	AI507552|B130044K16Rik|MYST-4|Morf|Myst4|mKIAA0383|qkf|querkopf	K(lysine) acetyltransferase 6B	LOSS	protein-coding
216_T22,T2,T3,T42	chr14	2.1E+07	2E+07	999	+	**4**	**50**	NA	intron (NM_134079, intron 7 of 10)	intron (NM_134079, intron 7 of 10)	-155770	NM_001205241	54169	Mm.248967	NM_017479	ENSMUSG00000021767	**Kat6b**	AI507552|B130044K16Rik|MYST-4|Morf|Myst4|mKIAA0383|qkf|querkopf	K(lysine) acetyltransferase 6B	LOSS	protein-coding
218_T22,T2,T3,T42	chr14	2.1E+07	2E+07	1999	+	**4**	**50**	NA	intron (NM_134079, intron 7 of 10)	ORR1D1|LTR|ERVL-MaLR	-146270	NM_001205241	54169	Mm.248967	NM_017479	ENSMUSG00000021767	**Kat6b**	AI507552|B130044K16Rik|MYST-4|Morf|Myst4|mKIAA0383|qkf|querkopf	K(lysine) acetyltransferase 6B	LOSS	protein-coding
221_T22,T2,T42,T4	chr14	2.1E+07	2E+07	999	+	**4**	**50**	NA	intron (NM_134079, intron 7 of 10)	intron (NM_134079, intron 7 of 10)	-132770	NM_001205241	54169	Mm.248967	NM_017479	ENSMUSG00000021767	**Kat6b**	AI507552|B130044K16Rik|MYST-4|Morf|Myst4|mKIAA0383|qkf|querkopf	K(lysine) acetyltransferase 6B	LOSS	protein-coding
1387_T1,T2,T32,T3	chr18	4.5E+07	5E+07	999	+	**4**	**50**	NA	intron (NM_001312905, intron 1 of 10)	B3A|SINE|B2	41140	NM_001312905	140492	Mm.411614	NM_080465	ENSMUSG00000054477	**Kcnn2**	KCa2.2|SK2|SKCA2|bc|fri	potassium intermediate/small conductance calcium-activated channel, subfamily N, member 2	LOSS	protein-coding
1388_T12,T1,T2,T32	chr18	4.5E+07	5E+07	999	+	**4**	**50**	NA	intron (NM_001312905, intron 1 of 10)	URR1A|DNA|hAT-Charlie	46140	NM_001312905	140492	Mm.411614	NM_080465	ENSMUSG00000054477	**Kcnn2**	KCa2.2|SK2|SKCA2|bc|fri	potassium intermediate/small conductance calcium-activated channel, subfamily N, member 2	LOSS	protein-coding
1396_T12,T1,T2,T32	chr18	4.6E+07	5E+07	293999	+	**4**	**50**	NA	intron (NR_040342, intron 3 of 3)	Lx|LINE|L1	130346	NM_080465	140492	Mm.411614	NM_080465	ENSMUSG00000054477	**Kcnn2**	KCa2.2|SK2|SKCA2|bc|fri	potassium intermediate/small conductance calcium-activated channel, subfamily N, member 2	LOSS	protein-coding
783_T12,T1,T2,T32	chr18	1.5E+07	2E+07	999	+	**4**	**50**	NA	Intergenic	Intergenic	-29554	NM_134112	106931	Mm.329299	NM_134112	ENSMUSG00000036225	**Kctd1**	4933402K10Rik|AI661543|AW553000	potassium channel tetramerisation domain containing 1	LOSS	protein-coding

785_T12,T1,T22,T2	chr18	1.5E+07	2E+07	16999	+	**4**	**50**	NA	Intergenic	MamTip2|DNA|hAT-Tip100	-64554	NM_134112	106931	Mm.329299	NM_134112	ENSMUSG00000036225	**Kctd1**	4933402K10Rik|AI661543|AW553000	potassium channel tetramerisation domain containing 1	LOSS	protein-coding
1275_T12,T1,T22,T2	chr18	4.1E+07	4E+07	4999	+	**4**	**50**	NA	Intergenic	(TTA)n|Simple_repeat|Simple_repeat	308639	NM_026135	383348	Mm.76366	NM_026135	ENSMUSG00000051401	**Kctd16**	4930434H12Rik|Gm1267	potassium channel tetramerisation domain containing 16	LOSS	protein-coding
1277_T12,T1,T22,T2	chr18	4.1E+07	4E+07	24999	+	**4**	**50**	NA	Intergenic	Intergenic	324639	NM_026135	383348	Mm.76366	NM_026135	ENSMUSG00000051401	**Kctd16**	4930434H12Rik|Gm1267	potassium channel tetramerisation domain containing 16	LOSS	protein-coding
1279_T1,T22,T2,T3	chr18	4.1E+07	4E+07	999	+	**4**	**50**	NA	Intergenic	Lx5b|LINE|L1	338639	NM_026135	383348	Mm.76366	NM_026135	ENSMUSG00000051401	**Kctd16**	4930434H12Rik|Gm1267	potassium channel tetramerisation domain containing 16	LOSS	protein-coding
1289_T12,T1,T2,T32	chr18	4.1E+07	4E+07	21999	+	**4**	**50**	NA	Intergenic	Intergenic	643139	NM_026135	383348	Mm.76366	NM_026135	ENSMUSG00000051401	**Kctd16**	4930434H12Rik|Gm1267	potassium channel tetramerisation domain containing 16	LOSS	protein-coding
1291_T12,T1,T2,T32	chr18	4.1E+07	4E+07	247999	+	**4**	**50**	NA	Intergenic	Intergenic	782139	NM_026135	383348	Mm.76366	NM_026135	ENSMUSG00000051401	**Kctd16**	4930434H12Rik|Gm1267	potassium channel tetramerisation domain containing 16	LOSS	protein-coding
1266_T12,T2,T32,T42	chr18	4E+07	4E+07	999	+	**4**	**50**	NA	intron (NM_026135, intron 1 of 1)	intron (NM_026135, intron 1 of 1)	162639	NM_026135	383348	Mm.76366	NM_026135	ENSMUSG00000051401	**Kctd16**	4930434H12Rik|Gm1267	potassium channel tetramerisation domain containing 16	LOSS	protein-coding
1268_T12,T1,T2,T32	chr18	4E+07	4E+07	26999	+	**4**	**50**	NA	intron (NM_026135, intron 1 of 1)	L2c|LINE|L2	180639	NM_026135	383348	Mm.76366	NM_026135	ENSMUSG00000051401	**Kctd16**	4930434H12Rik|Gm1267	potassium channel tetramerisation domain containing 16	LOSS	protein-coding
951_T12,T1,T2,T32	chr18	2.2E+07	2E+07	571999	+	**4**	**50**	NA	intron (NM_027616, intron 22 of 22)	intron (NM_027616, intron 22 of 22)	-176132	NM_001081403	225266	Mm.436836	NM_001081403	ENSMUSG00000042514	**Klhl14**	6330403N15Rik|AI785250|printor	kelch-like 14	LOSS	protein-coding
20_T22,T3,T42,T4	chr10	2.8E+07	3E+07	999	+	**4**	**50**	NA	Intergenic	L1Md_A|LINE|L1	-208058	NM_008481	16773	Mm.256087	NM_008481	ENSMUSG00000019899	**Lama2**	5830440B04|dy|mKIAA4087|mer|merosin	laminin, alpha 2	LOSS	protein-coding
1336_T12,T2,T32,T4	chr18	4.2E+07	4E+07	999	+	**4**	**50**	NA	exon (NM_134137, exon 10 of 32)	exon (NM_134137, exon 10 of 32)	20071	NM_134137	107045	Mm.312170	NM_134137	ENSMUSG00000024493	**Lars**	2310045K21Rik|3110009L02Rik|AW536573|mKIAA1352	leucyl-tRNA synthetase	LOSS	protein-coding
1330_T12,T1,T22,T32	chr18	4.2E+07	4E+07	999	+	**4**	**50**	NA	exon (NM_134137, exon 16 of 32)	exon (NM_134137, exon 16 of 32)	32071	NM_134137	107045	Mm.312170	NM_134137	ENSMUSG00000024493	**Lars**	2310045K21Rik|3110009L02Rik|AW536573|mKIAA1352	leucyl-tRNA synthetase	LOSS	protein-coding
317_T12,T22,T2,T4	chr14	3.5E+07	3E+07	93999	+	**4**	**50**	NA	intron (NM_001039073, intron 4 of 14)	B3|SINE|B2	13107	NM_001039074	24131	Mm.29733	NM_011918	ENSMUSG00000021798	**Ldb3**	AW742271|PDLIM6|ZASP	LIM domain binding 3	LOSS	protein-coding

1817_T12,T22,T3,T42	chr18	6.8E+07	7E+07	11999	+	**4**	**50**	NA	intron (NM_172631, intron 2 of 5)	Lx7|LINE|L1	147243	NM_172631	52662	Mm.440183	NM_172631	ENSMUSG00000024544	**Ldlrad4**	8230401C20Rik|A430083H02|A430108L08Rik|C18orf1|D18Ertd653e|D330030L18Rik	low density lipoprotein receptor class A domain containing 4	LOSS	protein-coding
1561_T12,T1,T2,T32	chr18	5.2E+07	5E+07	46999	+	**4**	**50**	NA	TTS (NM_010728)	TTS (NM_010728)	13721	NM_001286181	16948	Mm.172	NM_010728	ENSMUSG00000024529	**Lox**	AI893619|TSC-160|rrg	lysyl oxidase	LOSS	protein-coding
1860_T12,T1,T2,T32	chr18	7.1E+07	7E+07	84999	+	**4**	**50**	NA	Intergenic	L1_Mus1|LINE|L1	90708	NM_010773	17191	Mm.322	NM_010773	ENSMUSG00000024513	**Mbd2**	MBD2a	methyl-CpG binding domain protein 2	LOSS	protein-coding
1864_T12,T1,T2,T42	chr18	7.1E+07	7E+07	28999	+	**4**	**50**	NA	Intergenic	Intergenic	192708	NM_010773	17191	Mm.322	NM_010773	ENSMUSG00000024513	**Mbd2**	MBD2a	methyl-CpG binding domain protein 2	LOSS	protein-coding
1870_T12,T1,T2,T32	chr18	7.1E+07	7E+07	182999	+	**4**	**50**	NA	Intergenic	Intergenic	372708	NM_010773	17191	Mm.322	NM_010773	ENSMUSG00000024513	**Mbd2**	MBD2a	methyl-CpG binding domain protein 2	LOSS	protein-coding
1871_T12,T1,T2,T32	chr18	7.1E+07	7E+07	98999	+	**4**	**50**	NA	Intergenic	MLT2B1|LTR|ERVL	520708	NM_010773	17191	Mm.322	NM_010773	ENSMUSG00000024513	**Mbd2**	MBD2a	methyl-CpG binding domain protein 2	LOSS	protein-coding
1874_T12,T1,T2,T32	chr18	7.1E+07	7E+07	112999	+	**4**	**50**	NA	Intergenic	RLTR10-int|LTR|ERVK	641708	NM_010773	17191	Mm.322	NM_010773	ENSMUSG00000024513	**Mbd2**	MBD2a	methyl-CpG binding domain protein 2	LOSS	protein-coding
1885_T12,T1,T2,T32	chr18	7.1E+07	7E+07	28999	+	**4**	**50**	NA	intron (NM_007831, intron 13 of 28)	intron (NM_007831, intron 13 of 28)	885708	NM_010773	17191	Mm.322	NM_010773	ENSMUSG00000024513	**Mbd2**	MBD2a	methyl-CpG binding domain protein 2	LOSS	protein-coding
1883_T12,T1,T2,T32	chr18	7.1E+07	7E+07	999	+	**4**	**50**	NA	intron (NM_007831, intron 14 of 28)	intron (NM_007831, intron 14 of 28)	856708	NM_010773	17191	Mm.322	NM_010773	ENSMUSG00000024513	**Mbd2**	MBD2a	methyl-CpG binding domain protein 2	LOSS	protein-coding
1881_T12,T1,T2,T32	chr18	7.1E+07	7E+07	13999	+	**4**	**50**	NA	intron (NM_007831, intron 21 of 28)	intron (NM_007831, intron 21 of 28)	793208	NM_010773	17191	Mm.322	NM_010773	ENSMUSG00000024513	**Mbd2**	MBD2a	methyl-CpG binding domain protein 2	LOSS	protein-coding
1827_T12,T1,T2,T32	chr18	6.8E+07	7E+07	12999	+	**4**	**50**	NA	Intergenic	L1_Mur3|LINE|L1	-4680	NM_001271716	17200	Mm.426053	NM_008560	ENSMUSG00000045569	**Mc2r**	ACTH-R|ACTHR|MC2-R	melanocortin 2 receptor	LOSS	protein-coding
1822_T12,T2,T32,T3	chr18	6.8E+07	7E+07	999	+	**4**	**50**	NA	Intergenic	L1Md_T|LINE|L1	44397	NM_013596	17203	Mm.8003	NM_013596	ENSMUSG00000007480	**Mc5r**	-	melanocortin 5 receptor	LOSS	protein-coding
1954_T12,T1,T22,T2	chr18	7.4E+07	7E+07	221999	+	**4**	**50**	NA	intron (NM_145494, intron 2 of 15)	intron (NM_145494, intron 2 of 15)	12892	NM_145494	107029	Mm.36817	NM_145494	ENSMUSG00000024556	**Me2**	AW120568|D030040L20Rik|NAD-ME	malic enzyme 2, NAD(+)-dependent, mitochondrial	LOSS	protein-coding

86_T12,T1,T2,T42	chr12	1.1E+08	1E+08	999	+	**4**	**50**	NA	intron (NR_027651, intron 5 of 8)	intron (NR_027651, intron 5 of 8)	1602	NR_003633	17263	Mm.289645	NM_144513	ENSMUSG00000021268	**Meg3**	2900016C05Rik|3110050O07Rik|6330408G06Rik|AI425946|AW108224|D12Bwg1266e|Gtl2|R74756|R75394	maternally expressed 3	LOSS	ncRNA
1645_T12,T1,T2,T32	chr18	5.7E+07	6E+07	199999	+	**4**	**50**	NA	Intergenic	Intergenic	-18590	NM_001001979	70417	Mm.297863	NM_001001979	ENSMUSG00000024593	**Megf10**	3000002B06Rik|Gm331	multiple EGF-like-domains 10	LOSS	protein-coding
942_T1,T2,T32,T42	chr18	2.1E+07	2E+07	999	+	**4**	**50**	NA	intron (NM_001033445, intron 3 of 5)	intron (NM_001033445, intron 3 of 5)	95656	NM_008586	17288	Mm.2682	NM_008586	ENSMUSG00000024313	**Mep1b**	Mep-1b	meprin 1 beta	LOSS	protein-coding
546_T12,T2,T42,T4	chr14	5.2E+07	5E+07	5999	+	**4**	**50**	NA	Intergenic	Intergenic	-13342	NM_001029990	52535	Mm.5750	NM_001029990	ENSMUSG00000004561	**Mettl17**	2310032K15Rik|D14Ertd209e|Mett11d1	methyltransferase like 17	LOSS	protein-coding
1935_T12,T1,T2,T32	chr18	7.3E+07	7E+07	161999	+	**4**	**50**	NA	Intergenic	Intergenic	-543205	NM_001039214	240396	Mm.24131	NM_001039214	ENSMUSG00000037253	**Mex3c**	A130001D14Rik|BC035207|BM-013|Rkhd2	mex3 RNA binding family member C	LOSS	protein-coding
1936_T12,T1,T2,T32	chr18	7.3E+07	7E+07	22999	+	**4**	**50**	NA	Intergenic	Lx8|LINE|L1	-444705	NM_001039214	240396	Mm.24131	NM_001039214	ENSMUSG00000037253	**Mex3c**	A130001D14Rik|BC035207|BM-013|Rkhd2	mex3 RNA binding family member C	LOSS	protein-coding
1938_T12,T1,T2,T32	chr18	7.3E+07	7E+07	77999	+	**4**	**50**	NA	Intergenic	L1M2|LINE|L1	-385205	NM_001039214	240396	Mm.24131	NM_001039214	ENSMUSG00000037253	**Mex3c**	A130001D14Rik|BC035207|BM-013|Rkhd2	mex3 RNA binding family member C	LOSS	protein-coding
1940_T12,T1,T2,T32	chr18	7.3E+07	7E+07	21999	+	**4**	**50**	NA	Intergenic	Intergenic	-324205	NM_001039214	240396	Mm.24131	NM_001039214	ENSMUSG00000037253	**Mex3c**	A130001D14Rik|BC035207|BM-013|Rkhd2	mex3 RNA binding family member C	LOSS	protein-coding
1946_T12,T1,T2,T32	chr18	7.3E+07	7E+07	4999	+	**4**	**50**	NA	Intergenic	Intergenic	-232705	NM_001039214	240396	Mm.24131	NM_001039214	ENSMUSG00000037253	**Mex3c**	A130001D14Rik|BC035207|BM-013|Rkhd2	mex3 RNA binding family member C	LOSS	protein-coding
1948_T12,T1,T2,T32	chr18	7.3E+07	7E+07	102999	+	**4**	**50**	NA	Intergenic	Lx2B|LINE|L1	-171705	NM_001039214	240396	Mm.24131	NM_001039214	ENSMUSG00000037253	**Mex3c**	A130001D14Rik|BC035207|BM-013|Rkhd2	mex3 RNA binding family member C	LOSS	protein-coding
1950_T12,T1,T2,T32	chr18	7.3E+07	7E+07	226999	+	**4**	**50**	NA	Intergenic	ORR1F|LTR|ERVL-MaLR	-5705	NM_001039214	240396	Mm.24131	NM_001039214	ENSMUSG00000037253	**Mex3c**	A130001D14Rik|BC035207|BM-013|Rkhd2	mex3 RNA binding family member C	LOSS	protein-coding
669_T12,T1,T2,T32	chr18	6868501	7E+06	222999	+	**4**	**50**	NA	intron (NM_177595, intron 5 of 6)	intron (NM_177595, intron 5 of 6)	24779	NM_177595	210719	Mm.257186	NM_177595	ENSMUSG00000061013	**Mkx**	9430023B20Rik|Irxl1	mohawk homeobox	LOSS	protein-coding
311_T12,T2,T42,T4	chr14	3.4E+07	3E+07	5999	+	**4**	**50**	NA	intron (NM_009758, intron 9 of 12)	intron (NM_009758, intron 9 of 12)	48996	NM_153127	105450	Mm.272673	NM_153127	ENSMUSG00000041445	**Mmrn2**	AA986839|ENDOGLYX1|Emilin3|EndoGlyx-1	multimerin 2	LOSS	protein-coding
675_T12,T1,T22,T2	chr18	7535501	8E+06	71999	+	**4**	**50**	NA	intron (NM_001161620, intron 1 of 12)	L1M2|LINE|L1	55363	NM_001081287	75739	Mm.133293	NM_001081287	ENSMUSG00000057440	**Mpp7**	1110068J02Rik|2810038M04Rik|5430426E14Rik|AI415104|Gm955	membrane protein, palmitoylated 7 (MAGUK p55 subfamily member 7)	LOSS	protein-coding

204_T22,T2,T3,T42	chr14	2.1E+07	2E+07	6999	+	**4**	**50**	NA	intron (NM_001310427, intron 10 of 12)	intron (NM_001310427, intron 10 of 12)	-16099	NM_029104	74843	Mm.295241	NM_029104	ENSMUSG00000021815	**Mss51**	4833444M15Rik|Zmynd17	MSS51 mitochondrial translational activator	LOSS	protein-coding
2293_T22,T2,T3,T4	chr2	2.2E+07	2E+07	3999	+	**4**	**50**	NA	Intergenic	L1Md_A|LINE|L1	-223003	NM_148413	667663	Mm.330033	NM_148413	ENSMUSG00000025716	**Myo3a**	9030416P08Rik	myosin IIIA	LOSS	protein-coding
210_T22,T2,T3,T42	chr14	2.1E+07	2E+07	16999	+	**4**	**50**	NA	intron (NM_021508, intron 3 of 5)	intron (NM_021508, intron 3 of 5)	4540	NM_021508	59011	Mm.439911	NM_021508	ENSMUSG00000068697	**Myoz1**	2310001N11Rik|AV090278|FATZ|Myoz	myozenin 1	LOSS	protein-coding
1775_T12,T1,T2,T32	chr18	6.4E+07	7E+07	948999	+	**4**	**50**	NA	intron (NM_001114386, intron 1 of 30)	(TG)n|Simple_repeat|Simple_repeat	37244	NM_001114386	83814	Mm.98668	NM_031881	ENSMUSG00000024589	**Nedd4l**	1300012C07Rik|Nedd4-2|Nedd4b	neural precursor cell expressed, developmentally down-regulated gene 4-like	LOSS	protein-coding
2150_T12,T1,T2,T32	chr18	8.6E+07	9E+07	18999	+	**4**	**50**	NA	Intergenic	Intergenic	-582952	NM_144946	246317	Mm.329578	NM_144946	ENSMUSG00000050321	**Neto1**	AI851453|Btcl1|C130005O10Rik	neuropilin (NRP) and tolloid (TLL)-like 1	LOSS	protein-coding
2155_T12,T1,T2,T32	chr18	8.6E+07	9E+07	45999	+	**4**	**50**	NA	Intergenic	Intergenic	-534452	NM_144946	246317	Mm.329578	NM_144946	ENSMUSG00000050321	**Neto1**	AI851453|Btcl1|C130005O10Rik	neuropilin (NRP) and tolloid (TLL)-like 1	LOSS	protein-coding
970_T12,T1,T2,T32	chr18	2.3E+07	2E+07	78999	+	**4**	**50**	NA	intron (NM_001161483, intron 2 of 10)	Lx9|LINE|L1	114663	NM_001161483	319211	Mm.209896	NM_199024	ENSMUSG00000041923	**Nol4**	1700013J13Rik|4930568N03Rik|Gm1262	nucleolar protein 4	LOSS	protein-coding
968_T12,T1,T2,T32	chr18	2.3E+07	2E+07	302999	+	**4**	**50**	NA	intron (NM_001161483, intron 9 of 10)	B1_Mm|SINE|Alu	312663	NM_001161483	319211	Mm.209896	NM_199024	ENSMUSG00000041923	**Nol4**	1700013J13Rik|4930568N03Rik|Gm1262	nucleolar protein 4	LOSS	protein-coding
973_T12,T1,T2,T32	chr18	2.3E+07	2E+07	57999	+	**4**	**50**	NA	intron (NM_199024, intron 1 of 9)	intron (NM_199024, intron 1 of 9)	13163	NM_001161483	319211	Mm.209896	NM_199024	ENSMUSG00000041923	**Nol4**	1700013J13Rik|4930568N03Rik|Gm1262	nucleolar protein 4	LOSS	protein-coding
757_T12,T1,T2,T3	chr18	1.2E+07	1E+07	174999	+	**4**	**50**	NA	intron (NM_008720, intron 4 of 24)	RMER16B|LTR|ERVK	18386	NM_008720	18145	Mm.3484	NM_008720	ENSMUSG00000024413	**Npc1**	A430089E03Rik|C85354|D18Ertd139e|D18Ertd723e|lcsd|nmf164|spm	Niemann-Pick type C1	LOSS	protein-coding
1368_T12,T1,T22,T2	chr18	4.4E+07	4E+07	61999	+	**4**	**50**	NA	Intergenic	IAPEz-int|LTR|ERVK	-5627	NM_010935	18169	Mm.57234	NM_010935	ENSMUSG00000038071	**Npy6r**	-	neuropeptide Y receptor Y6	LOSS	protein-coding
1182_T12,T1,T2,T32	chr18	3.3E+07	3E+07	63999	+	**4**	**50**	NA	Intergenic	Intergenic	101134	NM_001267717	27528	Mm.407415	NM_053078	ENSMUSG00000042834	**Nrep**	AI325076|D0H4S114|Harp|P311|PTZ17|SEZ17	neuronal regeneration related protein	LOSS	protein-coding
1184_T12,T1,T2,T32	chr18	3.3E+07	3E+07	57999	+	**4**	**50**	NA	Intergenic	Charlie2a|DNA|hAT-Charlie	33134	NM_001267717	27528	Mm.407415	NM_053078	ENSMUSG00000042834	**Nrep**	AI325076|D0H4S114|Harp|P311|PTZ17|SEZ17	neuronal regeneration related protein	LOSS	protein-coding

1188_T22,T2,T3,T4	chr18	3.3E+07	3E+07	2999	+	**4**	**50**	NA	promoter-TSS (NM_001109989)	promoter-TSS (NM_001109989)	29	NM_001109988	27528	Mm.407415	NM_053078	ENSMUSG00000042834	**Nrep**	AI325076|D0H4S114|Harp|P311|PTZ17|SEZ17	neuronal regeneration related protein	LOSS	protein-coding
202_T22,T3,T42,T4	chr14	2E+07	2E+07	999	+	**4**	**50**	NA	Intergenic	Intergenic	-5690	NM_026341	67725	Mm.317636	NM_026341	ENSMUSG00000021809	**Nudt13**	4933433B15Rik	nudix (nucleoside diphosphate linked moiety X)-type motif 13	LOSS	protein-coding
1773_T12,T1,T2,T32	chr18	6.4E+07	6E+07	99999	+	**4**	**50**	NA	3' UTR (NM_194268, exon 2 of 2)	3' UTR (NM_194268, exon 2 of 2)	57136	NM_194268	225631	Mm.234723	NM_194268	ENSMUSG00000045991	**Onecut2**	C730009D12|OC-2|Oc2	one cut domain, family member 2	LOSS	protein-coding
1771_T12,T1,T2,T32	chr18	6.4E+07	6E+07	17999	+	**4**	**50**	NA	Intergenic	Intergenic	-2864	NM_194268	225631	Mm.234723	NM_194268	ENSMUSG00000045991	**Onecut2**	C730009D12|OC-2|Oc2	one cut domain, family member 2	LOSS	protein-coding
2057_T22,T2,T32,T42	chr18	8E+07	8E+07	999	+	**4**	**50**	NA	Intergenic	IAPEz-int|LTR|ERVK	-336895	NM_053117	93737	Mm.24678	NM_053117	ENSMUSG00000056214	**Pard6g**	2410049N21Rik|Par6a	par-6 family cell polarity regulator gamma	LOSS	protein-coding
291_T22,T2,T3,T4	chr14	3.2E+07	3E+07	24999	+	**4**	**50**	NA	intron (NM_011960, intron 6 of 17)	B4A|SINE|B4	15029	NM_011960	26430	Mm.15962	NM_011960	ENSMUSG00000021911	**Parg**	AI413217	poly (ADP-ribose) glycohydrolase	LOSS	protein-coding
1232_T12,T1,T2,T32	chr18	3.7E+07	4E+07	41999	+	**4**	**50**	NA	promoter-TSS (NM_009960)	promoter-TSS (NM_009960)	-358	NM_009960	12942	Mm.308500	NM_009960	ENSMUSG00000102206	**Pcdha11**	A830022B16Rik|Cnr7|Crnr7	protocadherin alpha 11	LOSS	protein-coding
1235_T12,T2,T3,T4	chr18	3.7E+07	4E+07	4999	+	**4**	**50**	NA	intron (NM_138663, intron 1 of 3)	intron (NM_138663, intron 1 of 3)	28770	NM_138663	192164	Mm.308500	NM_138663	ENSMUSG00000103310	**Pcdha12**	Cnr5|Crnr5|Pcdha13	protocadherin alpha 12	LOSS	protein-coding
1230_T1,T22,T2,T32	chr18	3.7E+07	4E+07	999	+	**4**	**50**	NA	intron (NM_001301259, intron 1 of 3)	intron (NM_001301259, intron 1 of 3)	6076	NM_009957	12939	Mm.308500	NM_009957	ENSMUSG00000104318	**Pcdha7**	Cnr4|Crnr4	protocadherin alpha 7	LOSS	protein-coding
1237_T12,T1,T2,T3	chr18	3.7E+07	4E+07	44999	+	**4**	**50**	NA	intron (NM_001003671, intron 1 of 3)	intron (NM_001003671, intron 1 of 3)	6864	NM_001003671	353236	Mm.308500	NM_001003671	ENSMUSG00000103255	**Pcdhac1**	CNRc1	protocadherin alpha subfamily C, 1	LOSS	protein-coding
1211_T12,T1,T32,T3	chr18	3.6E+07	4E+07	999	+	**4**	**50**	NA	Intergenic	Intergenic	-18505	NM_026027	67199	Mm.30184	NM_026027	ENSMUSG00000024346	**Pfdn1**	2700086I23Rik|AA408327|AU044714	prefoldin 1	LOSS	protein-coding
1995_T1,T2,T3,T4	chr18	7.7E+07	8E+07	999	+	**4**	**50**	NA	intron (NM_001164170, intron 1 of 12)	L1Md_A|LINE|L1	22320	NM_001164168	17344	Mm.6370	NM_008602	ENSMUSG00000025423	**Pias2**	6330408K17Rik|AI462206|ARIP3|AU018068|Dib|Miz1|PIASxalpha|PIASxbeta|SIZ2	protein inhibitor of activated STAT 2	LOSS	protein-coding

1744_T12,T1,T2,T32	chr18	6.3E+07	6E+07	401999	+	**4**	**50**	NA	intron (NM_001039485, intron 3 of 53)	intron (NM_001039485, intron 3 of 53)	145683	NM_001039485	667742	Mm.158720	NM_172629	ENSMUSG00000041482	**Piezo2**	5930434P17|9030411M15Rik|9430028L06Rik|Fam38b|Fam38b2	piezo-type mechanosensitive ion channel component 2	LOSS	protein-coding
1108_T12,T1,T2,T32	chr18	3E+07	3E+07	39999	+	**4**	**50**	NA	Intergenic	Intergenic	-322396	NM_181414	225326	Mm.194127	NM_181414	ENSMUSG00000033628	**Pik3c3**	5330434F23Rik|Vps34	phosphoinositide-3-kinase, class 3	LOSS	protein-coding
1109_T12,T1,T2,T32	chr18	3E+07	3E+07	751999	+	**4**	**50**	NA	Intergenic	Intergenic	80604	NM_181414	225326	Mm.194127	NM_181414	ENSMUSG00000033628	**Pik3c3**	5330434F23Rik|Vps34	phosphoinositide-3-kinase, class 3	LOSS	protein-coding
1112_T12,T1,T32,T3	chr18	3.1E+07	3E+07	5999	+	**4**	**50**	NA	Intergenic	L1Md_T|LINE|L1	517604	NM_181414	225326	Mm.194127	NM_181414	ENSMUSG00000033628	**Pik3c3**	5330434F23Rik|Vps34	phosphoinositide-3-kinase, class 3	LOSS	protein-coding
1326_T12,T1,T2,T32	chr18	4.2E+07	4E+07	44999	+	**4**	**50**	NA	Intergenic	Intergenic	36709	NM_027072	69401	Mm.34841	NM_027072	ENSMUSG00000059455	**Plac8l1**	1700009N18Rik|1700015M15Rik	PLAC8-like 1	LOSS	protein-coding
1328_T12,T1,T2,T32	chr18	4.2E+07	4E+07	3999	+	**4**	**50**	NA	intron (NM_027072, intron 2 of 3)	B1_Mus2|SINE|Alu	8209	NM_027072	69401	Mm.34841	NM_027072	ENSMUSG00000059455	**Plac8l1**	1700009N18Rik|1700015M15Rik	PLAC8-like 1	LOSS	protein-coding
1785_T12,T1,T2,T3	chr18	6.6E+07	7E+07	2999	+	**4**	**50**	NA	Intergenic	Intergenic	-42604	NM_021451	58801	Mm.271878	NM_021451	ENSMUSG00000024521	**Pmaip1**	Noxa	phorbol-12-myristate-13-acetate-induced protein 1	LOSS	protein-coding
1341_T12,T1,T2,T32	chr18	4.3E+07	4E+07	400999	+	**4**	**50**	NA	intron (NM_028392, intron 1 of 8)	intron (NM_028392, intron 1 of 8)	103506	NR_073583	72930	Mm.26134	NM_027531	ENSMUSG00000024500	**Ppp2r2b**	2900026H06Rik|6330404L05Rik|E130009M08Rik|PP2A-PR55B|PR55-BETA|SCA12	protein phosphatase 2, regulatory subunit B, beta	LOSS	protein-coding
1299_T1,T22,T2,T32	chr18	4.1E+07	4E+07	1999	+	**4**	**50**	NA	Intergenic	Lx7|LINE|L1	626694	NM_029942	77619	Mm.30649	NM_029942	ENSMUSG00000056671	**Prelid2**	1700003A01Rik|C330008K14Rik	PRELI domain containing 2	LOSS	protein-coding
1302_T1,T22,T2,T32	chr18	4.1E+07	4E+07	999	+	**4**	**50**	NA	Intergenic	Intergenic	589194	NM_029942	77619	Mm.30649	NM_029942	ENSMUSG00000056671	**Prelid2**	1700003A01Rik|C330008K14Rik	PRELI domain containing 2	LOSS	protein-coding
1305_T12,T22,T32,T42	chr18	4.1E+07	4E+07	999	+	**4**	**50**	NA	Intergenic	Lx9|LINE|L1	563194	NM_029942	77619	Mm.30649	NM_029942	ENSMUSG00000056671	**Prelid2**	1700003A01Rik|C330008K14Rik	PRELI domain containing 2	LOSS	protein-coding
1313_T12,T1,T2,T32	chr18	4.2E+07	4E+07	34999	+	**4**	**50**	NA	Intergenic	Intergenic	417194	NM_029942	77619	Mm.30649	NM_029942	ENSMUSG00000056671	**Prelid2**	1700003A01Rik|C330008K14Rik	PRELI domain containing 2	LOSS	protein-coding
1317_T12,T1,T22,T2	chr18	4.2E+07	4E+07	1999	+	**4**	**50**	NA	Intergenic	Intergenic	123694	NM_029942	77619	Mm.30649	NM_029942	ENSMUSG00000056671	**Prelid2**	1700003A01Rik|C330008K14Rik	PRELI domain containing 2	LOSS	protein-coding
1513_T12,T1,T2,T32	chr18	5.1E+07	5E+07	15999	+	**4**	**50**	NA	Intergenic	Lx8|LINE|L1	-211398	NM_001081224	71373	Mm.63546	NM_001081224	ENSMUSG00000073565	**Prr16**	5430406M13Rik|AI607429	proline rich 16	LOSS	protein-coding
1517_T12,T1,T2,T32	chr18	5.1E+07	5E+07	39999	+	**4**	**50**	NA	Intergenic	RMER17C-int|LTR|ERVK	-173398	NM_001081224	71373	Mm.63546	NM_001081224	ENSMUSG00000073565	**Prr16**	5430406M13Rik|AI607429	proline rich 16	LOSS	protein-coding
1520_T12,T1,T2,T32	chr18	5.1E+07	5E+07	68999	+	**4**	**50**	NA	Intergenic	RLTR31D_MM|LTR|ERVK	-80898	NM_001081224	71373	Mm.63546	NM_001081224	ENSMUSG00000073565	**Prr16**	5430406M13Rik|AI607429	proline rich 16	LOSS	protein-coding

1526_T12,T1,T22,T2	chr18	5.1E+07	5E+07	999	+	**4**	**50**	NA	Intergenic	L1M4|LINE|L1	261102	NM_001081224	71373	Mm.63546	NM_001081224	ENSMUSG00000073565	**Prr16**	5430406M13Rik|AI607429	proline rich 16	LOSS	protein-coding
1533_T12,T1,T22,T32	chr18	5.1E+07	5E+07	8999	+	**4**	**50**	NA	Intergenic	Intergenic	341102	NM_001081224	71373	Mm.63546	NM_001081224	ENSMUSG00000073565	**Prr16**	5430406M13Rik|AI607429	proline rich 16	LOSS	protein-coding
1524_T12,T1,T2,T32	chr18	5.1E+07	5E+07	210999	+	**4**	**50**	NA	intron (NM_001081224, intron 1 of 1)	L2|LINE|L2	89102	NM_001081224	71373	Mm.63546	NM_001081224	ENSMUSG00000073565	**Prr16**	5430406M13Rik|AI607429	proline rich 16	LOSS	protein-coding
1851_T12,T1,T2,T32	chr18	7E+07	7E+07	244999	+	**4**	**50**	NA	Intergenic	Lx9|LINE|L1	-16395	NM_001082553	80718	Mm.246753	NM_030554	ENSMUSG00000024511	**Rab27b**	2310021G14Rik|B130064M09Rik	RAB27B, member RAS oncogene family	LOSS	protein-coding
743_T12,T1,T2,T32	chr18	1.1E+07	1E+07	330999	+	**4**	**50**	NA	Intergenic	Lx2|LINE|L1	-173276	NM_001252495	225182	Mm.154275	NM_175458	ENSMUSG00000041238	**Rbbp8**	9930104E21Rik|CtIP|RBBP-8|RIM|SAE2	retinoblastoma binding protein 8	LOSS	protein-coding
756_T12,T1,T3,T4	chr18	1.2E+07	1E+07	999	+	**4**	**50**	NA	intron (NM_024182, intron 1 of 12)	B1_Mus2|SINE|Alu	1150	NM_024182	66878	Mm.28551	NM_024182	ENSMUSG00000024404	**Riok3**	1200013N13Rik|D18Ertd331e|E130306C24Rik|Sudd	RIO kinase 3	LOSS	protein-coding
1118_T12,T1,T2,T3	chr18	3.1E+07	3E+07	6999	+	**4**	**50**	NA	Intergenic	L1Md_T|LINE|L1	488128	NM_009065	19762	Mm.5163	NM_009065	ENSMUSG00000057455	**Rit2**	RIBA|Rin|Roc2	Ras-like without CAAX 2	LOSS	protein-coding
1139_T12,T2,T32,T42	chr18	3.1E+07	3E+07	3999	+	**4**	**50**	NA	intron (NM_009065, intron 1 of 4)	intron (NM_009065, intron 1 of 4)	69628	NM_009065	19762	Mm.5163	NM_009065	ENSMUSG00000057455	**Rit2**	RIBA|Rin|Roc2	Ras-like without CAAX 2	LOSS	protein-coding
1137_T12,T1,T2,T32	chr18	3.1E+07	3E+07	11999	+	**4**	**50**	NA	intron (NM_009065, intron 2 of 4)	intron (NM_009065, intron 2 of 4)	86628	NM_009065	19762	Mm.5163	NM_009065	ENSMUSG00000057455	**Rit2**	RIBA|Rin|Roc2	Ras-like without CAAX 2	LOSS	protein-coding
1141_T12,T1,T2,T32	chr18	3.1E+07	3E+07	73999	+	**4**	**50**	NA	promoter-TSS (NM_009065)	promoter-TSS (NM_009065)	-372	NM_009065	19762	Mm.5163	NM_009065	ENSMUSG00000057455	**Rit2**	RIBA|Rin|Roc2	Ras-like without CAAX 2	LOSS	protein-coding
2007_T12,T1,T2,T32	chr18	7.8E+07	8E+07	64999	+	**4**	**50**	NA	Intergenic	RMER12|LTR|ERVK	-24864	NM_001164504	225743	Mm.23293	NM_001164504	ENSMUSG00000025427	**Rnf165**	2900024M11Rik|AI427432|Akd2|Ark2c|G630064H08Rik|Gm96	ring finger protein 165	LOSS	protein-coding
2005_T12,T1,T2,T32	chr18	7.7E+07	8E+07	9999	+	**4**	**50**	NA	intron (NM_001164504, intron 1 of 7)	intron (NM_001164504, intron 1 of 7)	76636	NM_001164504	225743	Mm.23293	NM_001164504	ENSMUSG00000025427	**Rnf165**	2900024M11Rik|AI427432|Akd2|Ark2c|G630064H08Rik|Gm96	ring finger protein 165	LOSS	protein-coding
984_T12,T1,T32,T3	chr18	2.5E+07	2E+07	9999	+	**4**	**50**	NA	Intergenic	Intergenic	-34296	NM_144861	225283	Mm.28427	NM_144861	ENSMUSG00000040446	**Rprd1a**	C77387|mKIAA4077	regulation of nuclear pre-mRNA domain containing 1A	LOSS	protein-coding

1960_T12,T1,T2,T3	chr18	7.5E+07	7E+07	17999	+	**4**	**50**	NA	intron (NM_201600, intron 38 of 38)	L1M4|LINE|L1	9955	NR_028560	1E+08		NR_028560		**Scarna17**	MBII-119	small Cajal body-specific RNA 17	LOSS	ncRNA
1348_T12,T1,T2,T32	chr18	4.4E+07	4E+07	78999	+	**4**	**50**	NA	Intergenic	L1_Mus3|LINE|L1	-7301	NM_001289643	117158	Mm.210437	NM_054038	ENSMUSG00000038791	**Scgb3a2**	LuLeu1|Pnsp1|UGRP1|Utgrp1	secretoglobin, family 3A, member 2	LOSS	protein-coding
1780_T12,T1,T2,T32	chr18	6.6E+07	7E+07	181999	+	**4**	**50**	NA	intron (NM_025468, intron 2 of 5)	intron (NM_025468, intron 2 of 5)	9922	NM_025468	66286	Mm.27800	NM_025468	ENSMUSG00000024516	**Sec11c**	1810029G24Rik|Sec11l3	SEC11 homolog C, signal peptidase complex subunit	LOSS	protein-coding
2039_T12,T1,T22,T2	chr18	7.9E+07	8E+07	8999	+	**4**	**50**	NA	Intergenic	Intergenic	-130609	NM_053099	240427	Mm.312871	NM_053099	ENSMUSG00000024548	**Setbp1**	C130092E12|Seb|mKIAA0437	SET binding protein 1	LOSS	protein-coding
2041_T12,T1,T22,T2	chr18	7.9E+07	8E+07	3999	+	**4**	**50**	NA	Intergenic	Lx8|LINE|L1	-223109	NM_053099	240427	Mm.312871	NM_053099	ENSMUSG00000024548	**Setbp1**	C130092E12|Seb|mKIAA0437	SET binding protein 1	LOSS	protein-coding
2043_T1,T22,T2,T32	chr18	7.9E+07	8E+07	999	+	**4**	**50**	NA	Intergenic	(TTTTG)n|Simple_repeat|Simple_repeat	-240609	NM_053099	240427	Mm.312871	NM_053099	ENSMUSG00000024548	**Setbp1**	C130092E12|Seb|mKIAA0437	SET binding protein 1	LOSS	protein-coding
2050_T12,T1,T2,T32	chr18	7.9E+07	8E+07	999	+	**4**	**50**	NA	Intergenic	L1Md_T|LINE|L1	-311609	NM_053099	240427	Mm.312871	NM_053099	ENSMUSG00000024548	**Setbp1**	C130092E12|Seb|mKIAA0437	SET binding protein 1	LOSS	protein-coding
2031_T12,T1,T22,T2	chr18	7.9E+07	8E+07	22999	+	**4**	**50**	NA	intron (NM_053099, intron 3 of 5)	intron (NM_053099, intron 3 of 5)	230391	NM_053099	240427	Mm.312871	NM_053099	ENSMUSG00000024548	**Setbp1**	C130092E12|Seb|mKIAA0437	SET binding protein 1	LOSS	protein-coding
1731_T12,T1,T2,T32	chr18	6.2E+07	6E+07	458999	+	**4**	**50**	NA	intron (NM_172628, intron 12 of 16)	ORR1G|LTR|ERVL-MaLR	52925	NM_172628	225608	Mm.262320	NM_172628	ENSMUSG00000045629	**Sh3tc2**	D430044G18Rik	SH3 domain and tetratricopeptide repeats 2	LOSS	protein-coding
1982_T12,T1,T2,T32	chr18	7.7E+07	8E+07	199999	+	**4**	**50**	NA	Intergenic	Intergenic	-169905	NM_001109743	664805	Mm.329543	NM_001109743	ENSMUSG00000091519	**Skor2**	Corl2|EG664805|Fussel18|Gm7348	SKI family transcriptional corepressor 2	LOSS	protein-coding
1985_T12,T1,T2,T32	chr18	7.7E+07	8E+07	7999	+	**4**	**50**	NA	Intergenic	Lx6|LINE|L1	-59905	NM_001109743	664805	Mm.329543	NM_001109743	ENSMUSG00000091519	**Skor2**	Corl2|EG664805|Fussel18|Gm7348	SKI family transcriptional corepressor 2	LOSS	protein-coding
2028_T12,T1,T2,T32	chr18	7.8E+07	8E+07	267999	+	**4**	**50**	NA	intron (NM_207651, intron 2 of 21)	(TCTA)n|Simple_repeat|Simple_repeat	33450	NM_207651	27411	Mm.44158	NM_030683	ENSMUSG00000024552	**Slc14a2**	UT-A1|UT-A3	solute carrier family 14 (urea transporter), member 2	LOSS	protein-coding
1145_T12,T1,T2,T32	chr18	3.2E+07	3E+07	21999	+	**4**	**50**	NA	Intergenic	Intergenic	39402	NM_026165	67453	Mm.23896	NM_026165	ENSMUSG00000024259	**Slc25a46**	1200007B05Rik|AI325987	solute carrier family 25, member 46	LOSS	protein-coding

498_T12,T22,T2,T3	chr14	4.9E+07	5E+07	12999	+	**4**	**50**	NA	intron (NM_029238, intron 1 of 7)	intron (NM_029238, intron 1 of 7)	72837	NM_029238	75288	Mm.78738	NM_029238	ENSMUSG00000021852	**Slc35f4**	4930550L21Rik	solute carrier family 35, member F4	LOSS	protein-coding
1214_T12,T1,T2,T3	chr18	3.7E+07	4E+07	3999	+	**4**	**50**	NA	intron (NM_001271547, intron 19 of 19)	intron (NM_001271547, intron 19 of 19)	15348	NM_001271544	240215	Mm.135463	NM_172830	ENSMUSG00000024485	**Slc4a9**	AE4|D630003B07|D630024F24Rik	solute carrier family 4, sodium bicarbonate cotransporter, member 9	LOSS	protein-coding
1978_T1,T2,T32,T3	chr18	7.6E+07	8E+07	1999	+	**4**	**50**	NA	Intergenic	Intergenic	96055	NM_001311070	17126	Mm.152699	NM_010754	ENSMUSG00000024563	**Smad2**	7120426M23Rik|Madh2|Madr2|Smad-2|mMad2	SMAD family member 2	LOSS	protein-coding
1964_T12,T2,T32,T3	chr18	7.5E+07	8E+07	999	+	**4**	**50**	NA	intron (NM_001042660, intron 3 of 3)	intron (NM_001042660, intron 3 of 3)	14635	NM_001042660	17131	Mm.34407	NM_008543	ENSMUSG00000025880	**Smad7**	Madh7	SMAD family member 7	LOSS	protein-coding
1966_T12,T2,T32,T3	chr18	7.5E+07	8E+07	999	+	**4**	**50**	NA	intron (NM_001042660, intron 3 of 3)	intron (NM_001042660, intron 3 of 3)	20635	NM_001042660	17131	Mm.34407	NM_008543	ENSMUSG00000025880	**Smad7**	Madh7	SMAD family member 7	LOSS	protein-coding
1711_T12,T1,T2,T32	chr18	6E+07	6E+07	116999	+	**4**	**50**	NA	Intergenic	MERVK26-int|LTR|ERVK	31983	NM_134133	106878	Mm.273197	NM_134133	ENSMUSG00000038059	**Smim3**	2010002N04Rik|AI195350|AW561905|Nid67|cI-41	small integral membrane protein 3	LOSS	protein-coding
277_T22,T2,T3,T42	chr14	3.1E+07	3E+07	999	+	**4**	**50**	NA	intron (NM_001308091, intron 1 of 1)	intron (NM_001308091, intron 1 of 1)	3930	NM_001308466	66487	Mm.379116	NM_001308091	ENSMUSG00000058351	**Smim4**	2010107H07Rik|Gm41122	small integral membrane protein 4	LOSS	protein-coding
1569_T12,T1,T2,T32	chr18	5.3E+07	5E+07	33999	+	**4**	**50**	NA	Intergenic	Intergenic	-131865	NM_026386	67804	Mm.252171	NM_026386	ENSMUSG00000034484	**Snx2**	0610030A03Rik	sorting nexin 2	LOSS	protein-coding
2200_T12,T1,T22,T2	chr18	8.8E+07	9E+07	999	+	**4**	**50**	NA	Intergenic	Intergenic	1070207	NM_018821	54607	Mm.91920	NM_018821	ENSMUSG00000056153	**Socs6**	1500012M23Rik|5830401B18Rik|AI447482|Cis4|Cish4|HSPC060|SOCS-4|SOCS-6|SSI4|STAI4|STATI4|Socs4	suppressor of cytokine signaling 6	LOSS	protein-coding
2212_T12,T1,T22,T2	chr18	8.8E+07	9E+07	65999	+	**4**	**50**	NA	Intergenic	Intergenic	557707	NM_018821	54607	Mm.91920	NM_018821	ENSMUSG00000056153	**Socs6**	1500012M23Rik|5830401B18Rik|AI447482|Cis4|Cish4|HSPC060|SOCS-4|SOCS-6|SSI4|STAI4|STATI4|Socs4	suppressor of cytokine signaling 6	LOSS	protein-coding

2218_T12,T1,T22,T2	chr18	8.8E+07	9E+07	13999	+	**4**	**50**	NA	Intergenic	L1_Mus2|LINE|L1	443707	NM_018821	54607	Mm.91920	NM_018821	ENSMUSG00000056153	**Socs6**	1500012M23Rik|5830401B18Rik|AI447482|Cis4|Cish4|HSPC060|SOCS-4|SOCS-6|SSI4|STAI4|STATI4|Socs4	suppressor of cytokine signaling 6	LOSS	protein-coding
2222_T12,T1,T2,T32	chr18	8.8E+07	9E+07	129999	+	**4**	**50**	NA	Intergenic	Intergenic	339707	NM_018821	54607	Mm.91920	NM_018821	ENSMUSG00000056153	**Socs6**	1500012M23Rik|5830401B18Rik|AI447482|Cis4|Cish4|HSPC060|SOCS-4|SOCS-6|SSI4|STAI4|STATI4|Socs4	suppressor of cytokine signaling 6	LOSS	protein-coding
2224_T12,T1,T2,T32	chr18	8.9E+07	9E+07	314999	+	**4**	**50**	NA	Intergenic	Intergenic	112207	NM_018821	54607	Mm.91920	NM_018821	ENSMUSG00000056153	**Socs6**	1500012M23Rik|5830401B18Rik|AI447482|Cis4|Cish4|HSPC060|SOCS-4|SOCS-6|SSI4|STAI4|STATI4|Socs4	suppressor of cytokine signaling 6	LOSS	protein-coding
1740_T12,T1,T2,T32	chr18	6.3E+07	6E+07	126999	+	**4**	**50**	NA	intron (NM_001168423, intron 1 of 4)	B4|SINE|B4	52387	NM_001168423	1E+08	Mm.387557	NM_001168423	ENSMUSG00000073551	**Spink13**	ENSMUSG00000073551|Gm10534|Spink5l3	serine peptidase inhibitor, Kazal type 13	LOSS	protein-coding
1357_T12,T1,T2,T32	chr18	4.4E+07	4E+07	131999	+	**4**	**50**	NA	intron (NM_001081180, intron 2 of 31)	intron (NM_001081180, intron 2 of 31)	3259	NM_001081180	72432	Mm.35369	NM_001081180	ENSMUSG00000055561	**Spink5**	2310065D10Rik|AV238912|LEKT1|LETK1|VAKT1	serine peptidase inhibitor, Kazal type 5	LOSS	protein-coding
1359_T12,T1,T2,T32	chr18	4.4E+07	4E+07	88999	+	**4**	**50**	NA	intron (NM_001013797, intron 3 of 4)	intron (NM_001013797, intron 3 of 4)	9607	NM_001013797	433180	Mm.57302	NM_001013797	ENSMUSG00000055095	**Spink6**	EG433180	serine peptidase inhibitor, Kazal type 6	LOSS	protein-coding
772_T12,T1,T2,T32	chr18	1.4E+07	1E+07	88999	+	**4**	**50**	NA	Intergenic	RSINE1|SINE|B4	180914	NM_001161369	268996	Mm.31761	NM_009280	ENSMUSG00000037013	**Ss18**	D130059H17|Ssxt|Syt	SS18, nBAF chromatin remodeling complex subunit	LOSS	protein-coding
774_T12,T1,T2,T32	chr18	1.5E+07	1E+07	31999	+	**4**	**50**	NA	Intergenic	Intergenic	101414	NM_001161369	268996	Mm.31761	NM_009280	ENSMUSG00000037013	**Ss18**	D130059H17|Ssxt|Syt	SS18, nBAF chromatin remodeling complex subunit	LOSS	protein-coding
1767_T12,T1,T2,T32	chr18	6.4E+07	6E+07	31999	+	**4**	**50**	NA	Intergenic	Intergenic	-222859	NM_009182	20451	Mm.252110	NM_009182	ENSMUSG00000056812	**St8sia3**	AI847333|ST8SiaIII|Siat8c	ST8 alpha-N-acetyl-neuraminide alpha-2,8-sialyltransferase 3	LOSS	protein-coding
2001_T12,T1,T32,T4	chr18	7.7E+07	8E+07	31999	+	**4**	**50**	NA	Intergenic	Intergenic	-28347	NM_153124	225742	Mm.365178	NM_013666	ENSMUSG00000025425	**St8sia5**	ST8SiaV|Siat8e	ST8 alpha-N-acetyl-neuraminide alpha-2,8-sialyltransferase 5	LOSS	protein-coding
1999_T1,T2,T32,T4	chr18	7.7E+07	8E+07	999	+	**4**	**50**	NA	intron (NM_001164167, intron 8 of 13)	intron (NM_001164167, intron 8 of 13)	-52847	NM_153124	225742	Mm.365178	NM_013666	ENSMUSG00000025425	**St8sia5**	ST8SiaV|Siat8e	ST8 alpha-N-acetyl-neuraminide alpha-2,8-sialyltransferase 5	LOSS	protein-coding

1177_T12,T1,T22,T2	chr18	3.3E+07	3E+07	66999	+	**4**	**50**	NA	Intergenic	Lx7|LINE|L1	-61184	NM_133774	170459	Mm.127058	NM_133774	ENSMUSG00000024378	**Stard4**	4632419C16Rik|9030213J02Rik	StAR-related lipid transfer (START) domain containing 4	LOSS	protein-coding
1179_T12,T1,T22,T2	chr18	3.3E+07	3E+07	10999	+	**4**	**50**	NA	Intergenic	Lx8b|LINE|L1	-104184	NM_133774	170459	Mm.127058	NM_133774	ENSMUSG00000024378	**Stard4**	4632419C16Rik|9030213J02Rik	StAR-related lipid transfer (START) domain containing 4	LOSS	protein-coding
1342_T12,T1,T2,T32	chr18	4.3E+07	4E+07	324999	+	**4**	**50**	NA	Intergenic	URR1B|DNA|hAT-Charlie	-41697	NM_178749	269019	Mm.150401	NM_178749	ENSMUSG00000039954	**Stk32a**	A930015B13Rik|YANK1	serine/threonine kinase 32A	LOSS	protein-coding
1837_T12,T1,T2,T32	chr18	6.9E+07	7E+07	9999	+	**4**	**50**	NA	Intergenic	Lx7|LINE|L1	-107992	NM_013685	21413	Mm.4269	NM_013685	ENSMUSG00000053477	**Tcf4**	5730422P05Rik|ASP-I2|E2-2|E2.2|ITF-2|ITF-2b|ITF2|ME2|MITF-2A|MITF-2B|SEF-2|SEF2|SEF2-1|TFE|Tcf-4|bHLHb19	transcription factor 4	LOSS	protein-coding
597_T12,T22,T2,T3	chr14	8.8E+07	9E+07	3999	+	**4**	**50**	NA	Intergenic	L1Md_A|LINE|L1	436917	NM_001253755	219249	Mm.35168	NM_172605	ENSMUSG00000022019	**Tdrd3**	4732418C03Rik|6720468N18	tudor domain containing 3	LOSS	protein-coding
599_T1,T22,T2,T4	chr14	8.8E+07	9E+07	3999	+	**4**	**50**	NA	Intergenic	L1Md_F|LINE|L1	496917	NM_001253755	219249	Mm.35168	NM_172605	ENSMUSG00000022019	**Tdrd3**	4732418C03Rik|6720468N18	tudor domain containing 3	LOSS	protein-coding
146_T1,T2,T3,T42	chr14	1.4E+07	1E+07	1999	+	**4**	**50**	NA	Intergenic	Intergenic	67813	NM_001285780	66231	Mm.295875	NM_025435	ENSMUSG00000053453	**Thoc7**	Nif3l1bp1	THO complex 7	LOSS	protein-coding
2138_T12,T1,T22,T2	chr18	8.5E+07	9E+07	66999	+	**4**	**50**	NA	Intergenic	L1Md_T|LINE|L1	-50476	NM_025969	67105	Mm.250617	NM_025969	ENSMUSG00000024645	**Timm21**	1700034H14Rik|2700002I20Rik	tranlocase of inner mitochondrial membrane 21	LOSS	protein-coding
2140_T12,T1,T22,T2	chr18	8.5E+07	9E+07	19999	+	**4**	**50**	NA	Intergenic	L1_Mus1|LINE|L1	-112976	NM_025969	67105	Mm.250617	NM_025969	ENSMUSG00000024645	**Timm21**	1700034H14Rik|2700002I20Rik	tranlocase of inner mitochondrial membrane 21	LOSS	protein-coding
752_T12,T1,T2,T3	chr18	1.2E+07	1E+07	12999	+	**4**	**50**	NA	intron (NM_001289666, intron 4 of 14)	MMERVK9E_I-int|LTR|ERVK	32537	NR_110356	338363	Mm.271567	NM_178801	ENSMUSG00000049411	**Tmem241**	6030446N20Rik	transmembrane protein 241	LOSS	protein-coding
754_T12,T1,T2,T3	chr18	1.2E+07	1E+07	999	+	**4**	**50**	NA	intron (NM_001289666, intron 4 of 14)	L1M4|LINE|L1	24537	NR_110356	338363	Mm.271567	NM_178801	ENSMUSG00000049411	**Tmem241**	6030446N20Rik	transmembrane protein 241	LOSS	protein-coding
935_T12,T22,T32,T3	chr18	2.1E+07	2E+07	999	+	**4**	**50**	NA	Intergenic	ORR1B2|LTR|ERVL-MaLR	-5922	NM_029491	75964	Mm.273769	NM_029491	ENSMUSG00000033382	**Trappc8**	5033403J15Rik|AI845423|D030074E01Rik|Trs85|mKIAA1012	trafficking protein particle complex 8	LOSS	protein-coding
2132_T12,T1,T2,T32	chr18	8.4E+07	8E+07	448999	+	**4**	**50**	NA	Intergenic	Intergenic	153562	NM_001081300	110796	Mm.102136	NM_001081300	ENSMUSG00000046982	**Tshz1**	5730407I04Rik|D18Bwg1409e|Mtsh1|NY-CO-33|Sdccag33|Tsh1|mKIAA4206|teashirt1	teashirt zinc finger family member 1	LOSS	protein-coding

1170_T12,T1,T22,T2	chr18	3.3E+07	3E+07	2999	+	**4**	**50**	NA	intron (NR_045381, intron 2 of 4)	B1_Mur3|SINE|Alu	-40383	NM_021367	53603	Mm.143716	NM_021367	ENSMUSG00000024379	**Tslp**	-	thymic stromal lymphopoietin	LOSS	protein-coding
1172_T12,T1,T22,T2	chr18	3.3E+07	3E+07	11999	+	**4**	**50**	NA	intron (NR_045381, intron 2 of 4)	Lx5c|LINE|L1	-31883	NM_021367	53603	Mm.143716	NM_021367	ENSMUSG00000024379	**Tslp**	-	thymic stromal lymphopoietin	LOSS	protein-coding
1753_T12,T1,T32,T3	chr18	6.4E+07	6E+07	999	+	**4**	**50**	NA	Intergenic	B1_Mur2|SINE|Alu	31359	NM_016792	53382	Mm.19169	NM_016792	ENSMUSG00000024583	**Txnl1**	32kDa|TRP32|Txnl	thioredoxin-like 1	LOSS	protein-coding
172_T22,T2,T3,T4	chr14	1.8E+07	2E+07	209999	+	**4**	**50**	NA	intron (NM_009455, intron 3 of 5)	intron (NM_009455, intron 3 of 5)	24344	NM_009455	22194	Mm.4429	NM_009455	ENSMUSG00000021774	**Ube2e1**	UbcM3|Ubce5|ubcM2	ubiquitin-conjugating enzyme E2E 1	LOSS	protein-coding
212_T22,T2,T3,T42	chr14	2.1E+07	2E+07	97999	+	**4**	**50**	NA	intron (NM_009502, intron 6 of 20)	intron (NM_009502, intron 6 of 20)	61067	NM_009502	22330	Mm.279361	NM_009502	ENSMUSG00000021823	**Vcl**	9430097D22|AA571387|AI462105|AW545629	vinculin	LOSS	protein-coding
680_T12,T1,T2,T32	chr18	7788501	8E+06	1999	+	**4**	**50**	NA	Intergenic	Intergenic	-79332	NM_001282093	225131	Mm.204524	NM_153085	ENSMUSG00000024283	**Wac**	1110067P07Rik|A230035H12Rik|AI256735|AI256776|Wwp4	WW domain containing adaptor with coiled-coil	LOSS	protein-coding
683_T12,T1,T22,T4	chr18	7851501	8E+06	999	+	**4**	**50**	NA	Intergenic	Lx3A|LINE|L1	-16832	NM_001282093	225131	Mm.204524	NM_153085	ENSMUSG00000024283	**Wac**	1110067P07Rik|A230035H12Rik|AI256735|AI256776|Wwp4	WW domain containing adaptor with coiled-coil	LOSS	protein-coding
688_T12,T1,T2,T32	chr18	8018501	8E+06	39999	+	**4**	**50**	NA	Intergenic	RLTR13C2|LTR|ERVK	169303	NM_153085	225131	Mm.204524	NM_153085	ENSMUSG00000024283	**Wac**	1110067P07Rik|A230035H12Rik|AI256735|AI256776|Wwp4	WW domain containing adaptor with coiled-coil	LOSS	protein-coding
319_T12,T2,T42,T4	chr14	3.5E+07	3E+07	999	+	**4**	**50**	NA	Intergenic	Intergenic	-1928	NM_001004436	218914	Mm.27957	NM_001004436	ENSMUSG00000041408	**Wapl**	A530089A20Rik|BC037674|DIF-2|FOE|Wapal	WAPL cohesin release factor	LOSS	protein-coding
326_T12,T22,T32,T4	chr14	3.5E+07	3E+07	7999	+	**4**	**50**	NA	intron (NM_001004436, intron 12 of 18)	intron (NM_001004436, intron 12 of 18)	61572	NM_001301330	218914	Mm.27957	NM_001004436	ENSMUSG00000041408	**Wapl**	A530089A20Rik|BC037674|DIF-2|FOE|Wapal	WAPL cohesin release factor	LOSS	protein-coding
324_T22,T2,T32,T4	chr14	3.5E+07	3E+07	999	+	**4**	**50**	NA	intron (NM_001004436, intron 9 of 18)	intron (NM_001004436, intron 9 of 18)	56072	NM_001301330	218914	Mm.27957	NM_001004436	ENSMUSG00000041408	**Wapl**	A530089A20Rik|BC037674|DIF-2|FOE|Wapal	WAPL cohesin release factor	LOSS	protein-coding
1765_T12,T1,T2,T32	chr18	6.4E+07	6E+07	90999	+	**4**	**50**	NA	intron (NM_001014981, intron 26 of 27)	intron (NM_001014981, intron 26 of 27)	254305	NM_001014981	104082	Mm.30850	NM_001014981	ENSMUSG00000040560	**Wdr7**	AI462727|TRAG|mKIAA0541	WD repeat domain 7	LOSS	protein-coding

263_T22,T2,T3,T42	chr14	2.9E+07	3E+07	1999	+	**4**	**50**	NA	intron (NM_009524, intron 1 of 4)	intron (NM_009524, intron 1 of 4)	-2905	NM_001256224	22418	Mm.287544	NM_009524	ENSMUSG00000021994	**Wnt5a**	8030457G12Rik|Wnt-5a	wingless-type MMTV integration site family, member 5A	LOSS	protein-coding
1256_T12,T1,T2,T32	chr18	4E+07	4E+07	168999	+	**4**	**50**	NA	Intergenic	Intergenic	139399	NM_023311	67180	Mm.270382	NM_023311	ENSMUSG00000024487	**Yipf5**	2610311I19Rik|AA408236|Yip1a	Yip1 domain family, member 5	LOSS	protein-coding
1732_T12,T1,T32	chr18	6.2E+07	6E+07	999	+	**3**	**37.5**	NA	Intergenic	CT-rich|Low_complexity|Low_complexity	-56019	NM_007420	11555	Mm.5598	NM_007420	ENSMUSG00000045730	**Adrb2**	Adrb-2|Badm|Gpcr7	adrenergic receptor, beta 2	LOSS	protein-coding
1735_T12,T1,T2	chr18	6.2E+07	6E+07	4999	+	**3**	**37.5**	NA	Intergenic	L1Md_F2|LINE|L1	-66019	NM_007420	11555	Mm.5598	NM_007420	ENSMUSG00000045730	**Adrb2**	Adrb-2|Badm|Gpcr7	adrenergic receptor, beta 2	LOSS	protein-coding
1217_T12,T1,T2	chr18	3.7E+07	4E+07	1999	+	**3**	**37.5**	NA	Intergenic	ID_B1|SINE|B4	-3103	NM_175375	108857	Mm.482287	NM_175375	ENSMUSG00000024483	**Ankhd1**	1110004O12Rik|4933432B13Rik|9130019P20Rik|A530027J04Rik|A630021B20Rik|AA571404|Mask|mFLJ00246|mKIAA1085	ankyrin repeat and KH domain containing 1	LOSS	protein-coding
1215_T12,T1,T2	chr18	3.7E+07	4E+07	9999	+	**3**	**37.5**	NA	intron (NM_001271547, intron 19 of 19)	B3A|SINE|B2	-10103	NM_175375	108857	Mm.482287	NM_175375	ENSMUSG00000024483	**Ankhd1**	1110004O12Rik|4933432B13Rik|9130019P20Rik|A530027J04Rik|A630021B20Rik|AA571404|Mask|mFLJ00246|mKIAA1085	ankyrin repeat and KH domain containing 1	LOSS	protein-coding
1222_T1,T3,T4	chr18	3.7E+07	4E+07	999	+	**3**	**37.5**	NA	intron (NM_175375, intron 10 of 33)	intron (NM_175375, intron 10 of 33)	49397	NM_175375	108857	Mm.482287	NM_175375	ENSMUSG00000024483	**Ankhd1**	1110004O12Rik|4933432B13Rik|9130019P20Rik|A530027J04Rik|A630021B20Rik|AA571404|Mask|mFLJ00246|mKIAA1085	ankyrin repeat and KH domain containing 1	LOSS	protein-coding
758_T12,T1,T3	chr18	1.2E+07	1E+07	2999	+	**3**	**37.5**	NA	Intergenic	L1_Mur2|LINE|L1	-1280	NM_001190371	225187	Mm.53865	NM_001190371	ENSMUSG00000057766	**Ankrd29**	G630054C21|G630054C21Rik|Gm95	ankyrin repeat domain 29	LOSS	protein-coding
241_T22,T3,T4	chr14	2.6E+07	3E+07	7999	+	**3**	**37.5**	NA	Intergenic	Intergenic	-5655	NM_013469	11744	Mm.294083	NM_013469	ENSMUSG00000021866	**Anxa11**	A830099O17Rik|Anx11	annexin A11	LOSS	protein-coding
788_T12,T1,T32	chr18	1.5E+07	2E+07	1999	+	**3**	**37.5**	NA	Intergenic	Intergenic	37516	NM_001308647	11829	Mm.250786	NM_009700	ENSMUSG00000024411	**Aqp4**	WCH4	aquaporin 4	LOSS	protein-coding
790_T12,T1,T2	chr18	1.5E+07	2E+07	104999	+	**3**	**37.5**	NA	Intergenic	Intergenic	-14018	NM_001308646	11829	Mm.250786	NM_009700	ENSMUSG00000024411	**Aqp4**	WCH4	aquaporin 4	LOSS	protein-coding
792_T12,T1,T2	chr18	1.5E+07	2E+07	42999	+	**3**	**37.5**	NA	intron (NM_199055, intron 2 of 3)	intron (NM_199055, intron 2 of 3)	-99018	NM_001308646	11829	Mm.250786	NM_009700	ENSMUSG00000024411	**Aqp4**	WCH4	aquaporin 4	LOSS	protein-coding

252_T2,T32,T3	chr14	2.7E+07	3E+07	18999	+	**3**	**37.5**	NA	Intergenic	B3A|SINE|B2	-30993	NM_001289686	71704	Mm.248606	NM_027871	ENSMUSG00000021895	**Arhgef3**	1200004I24Rik|9830169H03Rik|C76747|Xpln	Rho guanine nucleotide exchange factor (GEF) 3	LOSS	protein-coding
254_T2,T32,T4	chr14	2.7E+07	3E+07	5999	+	**3**	**37.5**	NA	intron (NM_027871, intron 1 of 10)	intron (NM_027871, intron 1 of 10)	21490	NM_027871	71704	Mm.248606	NM_027871	ENSMUSG00000021895	**Arhgef3**	1200004I24Rik|9830169H03Rik|C76747|Xpln	Rho guanine nucleotide exchange factor (GEF) 3	LOSS	protein-coding
965_T12,T2,T32	chr18	2.3E+07	2E+07	1999	+	**3**	**37.5**	NA	Intergenic	L1Md_A|LINE|L1	210411	NM_001167777	211961	Mm.392310	NM_001167777	ENSMUSG00000045215	**Asxl3**	C230079D11Rik|D430002O22Rik|D930044O18Rik|Gm329|Gm945	additional sex combs like 3 (Drosophila)	LOSS	protein-coding
967_T12,T1,T2	chr18	2.3E+07	2E+07	1999	+	**3**	**37.5**	NA	Intergenic	L1Md_F|LINE|L1	228411	NM_001167777	211961	Mm.392310	NM_001167777	ENSMUSG00000045215	**Asxl3**	C230079D11Rik|D430002O22Rik|D930044O18Rik|Gm329|Gm945	additional sex combs like 3 (Drosophila)	LOSS	protein-coding
960_T12,T1,T3	chr18	2.2E+07	2E+07	999	+	**3**	**37.5**	NA	intron (NM_001167777, intron 2 of 12)	intron (NM_001167777, intron 2 of 12)	36911	NM_001167777	211961	Mm.392310	NM_001167777	ENSMUSG00000045215	**Asxl3**	C230079D11Rik|D430002O22Rik|D930044O18Rik|Gm329|Gm945	additional sex combs like 3 (Drosophila)	LOSS	protein-coding
963_T12,T1,T2	chr18	2.2E+07	2E+07	32999	+	**3**	**37.5**	NA	intron (NM_001167777, intron 5 of 12)	intron (NM_001167777, intron 5 of 12)	77911	NM_001167777	211961	Mm.392310	NM_001167777	ENSMUSG00000045215	**Asxl3**	C230079D11Rik|D430002O22Rik|D930044O18Rik|Gm329|Gm945	additional sex combs like 3 (Drosophila)	LOSS	protein-coding
2074_T12,T1,T2	chr18	8.1E+07	8E+07	50999	+	**3**	**37.5**	NA	intron (NM_001201569, intron 8 of 29)	intron (NM_001201569, intron 8 of 29)	80058	NM_001201569	50771	Mm.247138	NM_015805	ENSMUSG00000024566	**Atp9b**	AA934181|Atpc2b|IIb|MMR	ATPase, class II, type 9B	LOSS	protein-coding
154_T2,T3,T4	chr14	1.4E+07	1E+07	999	+	**3**	**37.5**	NA	intron (NM_139227, intron 3 of 11)	intron (NM_139227, intron 3 of 11)	2509	NM_139227	246103	Mm.133625	NM_139227	ENSMUSG00000021738	**Atxn7**	A430107N12Rik|AI627028|Sca7|ataxin-7	ataxin 7	LOSS	protein-coding
211_T2,T3,T42	chr14	2.1E+07	2E+07	280999	+	**3**	**37.5**	NA	Intergenic	Intergenic	-6912	NM_178597	12325	Mm.235182	NM_178597	ENSMUSG00000021820	**Camk2g**	Camkg	calcium/calmodulin-dependent protein kinase II gamma	LOSS	protein-coding
2171_T12,T1,T2	chr18	8.7E+07	9E+07	73999	+	**3**	**37.5**	NA	Intergenic	MER92-int|LTR|ERV1	-79610	NM_001302356	12405	Mm.70775	NM_172633	ENSMUSG00000024647	**Cbln2**	6330593N19Rik|A730004O05	cerebellin 2 precursor protein	LOSS	protein-coding
2173_T12,T1,T2	chr18	8.7E+07	9E+07	9999	+	**3**	**37.5**	NA	Intergenic	(TG)n|Simple_repeat|Simple_repeat	57435	NM_172633	12405	Mm.70775	NM_172633	ENSMUSG00000024647	**Cbln2**	6330593N19Rik|A730004O05	cerebellin 2 precursor protein	LOSS	protein-coding
2177_T12,T1,T2	chr18	8.7E+07	9E+07	96999	+	**3**	**37.5**	NA	Intergenic	MTD|LTR|ERVL-MaLR	153935	NM_172633	12405	Mm.70775	NM_172633	ENSMUSG00000024647	**Cbln2**	6330593N19Rik|A730004O05	cerebellin 2 precursor protein	LOSS	protein-coding
2181_T12,T1,T2	chr18	8.7E+07	9E+07	31999	+	**3**	**37.5**	NA	Intergenic	L1_Mus3|LINE|L1	242435	NM_172633	12405	Mm.70775	NM_172633	ENSMUSG00000024647	**Cbln2**	6330593N19Rik|A730004O05	cerebellin 2 precursor protein	LOSS	protein-coding

2187_T1,T2,T32	chr18	8.7E+07	9E+07	999	+	**3**	**37.5**	NA	Intergenic	Lx6|LINE|L1	475935	NM_172633	12405	Mm.70775	NM_172633	ENSMUSG00000024647	**Cbln2**	6330593N19Rik|A730004O05	cerebellin 2 precursor protein	LOSS	protein-coding
2195_T12,T1,T2	chr18	8.8E+07	9E+07	26999	+	**3**	**37.5**	NA	Intergenic	L1_Rod|LINE|L1	932935	NM_172633	12405	Mm.70775	NM_172633	ENSMUSG00000024647	**Cbln2**	6330593N19Rik|A730004O05	cerebellin 2 precursor protein	LOSS	protein-coding
2197_T12,T1,T2	chr18	8.8E+07	9E+07	111999	+	**3**	**37.5**	NA	Intergenic	Intergenic	1008435	NM_172633	12405	Mm.70775	NM_172633	ENSMUSG00000024647	**Cbln2**	6330593N19Rik|A730004O05	cerebellin 2 precursor protein	LOSS	protein-coding
952_T1,T2,T32	chr18	2.2E+07	2E+07	27999	+	**3**	**37.5**	NA	intron (NM_027616, intron 8 of 22)	L1Md_F2|LINE|L1	42896	NM_027616	70950	Mm.235716	NM_027616	ENSMUSG00000024306	**Ccdc178**	4921528I01Rik	coiled coil domain containing 178	LOSS	protein-coding
1845_T12,T22,T32	chr18	7E+07	7E+07	999	+	**3**	**37.5**	NA	TTS (NM_030554)	TTS (NM_030554)	53441	NM_201362	381175	Mm.266831	NM_201362	ENSMUSG00000038903	**Ccdc68**	BC046343	coiled-coil domain containing 68	LOSS	protein-coding
709_T1,T2,T42	chr18	9529501	1E+07	1999	+	**3**	**37.5**	NA	Intergenic	Intergenic	-80350	NM_026484	67974	Mm.86523	NM_026484	ENSMUSG00000024286	**Ccny**	1700025H17Rik|3110050L10Rik|4631402G10Rik|5730405I09Rik	cyclin Y	LOSS	protein-coding
2236_T12,T1,T2	chr18	8.9E+07	9E+07	106999	+	**3**	**37.5**	NA	intron (NM_001039173, intron 5 of 7)	intron (NM_001039173, intron 5 of 7)	264573	NM_001039149	225825	Mm.139293	NM_178687	ENSMUSG00000034028	**Cd226**	BC051526|DNAM-1|DNAM1|Pta1|TLiSA1	CD226 antigen	LOSS	protein-coding
2234_T12,T1,T3	chr18	8.9E+07	9E+07	39999	+	**3**	**37.5**	NA	intron (NM_001039173, intron 7 of 7)	RMER17C|LTR|ERVK	153073	NM_001039149	225825	Mm.139293	NM_178687	ENSMUSG00000034028	**Cd226**	BC051526|DNAM-1|DNAM1|Pta1|TLiSA1	CD226 antigen	LOSS	protein-coding
1715_T12,T1,T32	chr18	6.1E+07	6E+07	999	+	**3**	**37.5**	NA	promoter-TSS (NM_001042605)	promoter-TSS (NM_001042605)	-849	NM_001042605	16149	Mm.439737	NM_010545	ENSMUSG00000024610	**Cd74**	CLIP|DHLAG|HLADG|Ia-GAMMA|Ii	CD74 antigen (invariant polypeptide of major histocompatibility complex, class II antigen-associated)	LOSS	protein-coding
814_T12,T1,T32	chr18	1.6E+07	2E+07	5999	+	**3**	**37.5**	NA	Intergenic	Intergenic	316746	NM_007664	12558	Mm.257437	NM_007664	ENSMUSG00000024304	**Cdh2**	CDHN|N-CAD|Ncad	cadherin 2	LOSS	protein-coding
1013_T12,T1,T32	chr18	2.6E+07	3E+07	20999	+	**3**	**37.5**	NA	Intergenic	L2a|LINE|L2	-16017	NM_001174074	108013	Mm.266435	NM_133195	ENSMUSG00000024268	**Celf4**	A230070D14Rik|BRUNOL-4|Brul4|Brunol4|C130060B05Rik	CUGBP, Elav-like family member 4	LOSS	protein-coding
1015_T12,T1,T2	chr18	2.6E+07	3E+07	354999	+	**3**	**37.5**	NA	Intergenic	Intergenic	-211017	NM_001174074	108013	Mm.266435	NM_133195	ENSMUSG00000024268	**Celf4**	A230070D14Rik|BRUNOL-4|Brul4|Brunol4|C130060B05Rik	CUGBP, Elav-like family member 4	LOSS	protein-coding
1018_T12,T1,T2	chr18	2.6E+07	3E+07	121999	+	**3**	**37.5**	NA	Intergenic	Intergenic	-649517	NM_001174074	108013	Mm.266435	NM_133195	ENSMUSG00000024268	**Celf4**	A230070D14Rik|BRUNOL-4|Brul4|Brunol4|C130060B05Rik	CUGBP, Elav-like family member 4	LOSS	protein-coding

1020_T12,T1,T2	chr18	2.6E+07	3E+07	29999	+	**3**	**37.5**	NA	Intergenic	Intergenic	-726517	NM_001174074	108013	Mm.266435	NM_133195	ENSMUSG00000024268	**Celf4**	A230070D14Rik|BRUNOL-4|Brul4|Brunol4|C130060B05Rik	CUGBP, Elav-like family member 4	LOSS	protein-coding
1022_T12,T1,T2	chr18	2.6E+07	3E+07	106999	+	**3**	**37.5**	NA	Intergenic	MTA_Mm-int|LTR|ERVL-MaLR	-796017	NM_001174074	108013	Mm.266435	NM_133195	ENSMUSG00000024268	**Celf4**	A230070D14Rik|BRUNOL-4|Brul4|Brunol4|C130060B05Rik	CUGBP, Elav-like family member 4	LOSS	protein-coding
1025_T12,T1,T2	chr18	2.7E+07	3E+07	131999	+	**3**	**37.5**	NA	Intergenic	Intergenic	-1E+06	NM_001174074	108013	Mm.266435	NM_133195	ENSMUSG00000024268	**Celf4**	A230070D14Rik|BRUNOL-4|Brul4|Brunol4|C130060B05Rik	CUGBP, Elav-like family member 4	LOSS	protein-coding
1029_T12,T1,T2	chr18	2.7E+07	3E+07	8999	+	**3**	**37.5**	NA	Intergenic	MusHAL1|LINE|L1	-1E+06	NM_001174074	108013	Mm.266435	NM_133195	ENSMUSG00000024268	**Celf4**	A230070D14Rik|BRUNOL-4|Brul4|Brunol4|C130060B05Rik	CUGBP, Elav-like family member 4	LOSS	protein-coding
1030_T12,T1,T2	chr18	2.7E+07	3E+07	56999	+	**3**	**37.5**	NA	Intergenic	Intergenic	-1E+06	NM_001174074	108013	Mm.266435	NM_133195	ENSMUSG00000024268	**Celf4**	A230070D14Rik|BRUNOL-4|Brul4|Brunol4|C130060B05Rik	CUGBP, Elav-like family member 4	LOSS	protein-coding
1032_T12,T1,T2	chr18	2.7E+07	3E+07	28999	+	**3**	**37.5**	NA	Intergenic	L1_Mus3|LINE|L1	-1E+06	NM_001174074	108013	Mm.266435	NM_133195	ENSMUSG00000024268	**Celf4**	A230070D14Rik|BRUNOL-4|Brul4|Brunol4|C130060B05Rik	CUGBP, Elav-like family member 4	LOSS	protein-coding
1011_T12,T12,T32	chr18	2.5E+07	3E+07	999	+	**3**	**37.5**	NA	intron (NM_001146292, intron 12 of 12)	intron (NM_001146292, intron 12 of 12)	270983	NM_133195	108013	Mm.266435	NM_133195	ENSMUSG00000024268	**Celf4**	A230070D14Rik|BRUNOL-4|Brul4|Brunol4|C130060B05Rik	CUGBP, Elav-like family member 4	LOSS	protein-coding
1009_T12,T12,T32	chr18	2.5E+07	3E+07	999	+	**3**	**37.5**	NA	TTS (NM_133195)	TTS (NM_133195)	276983	NM_133195	108013	Mm.266435	NM_133195	ENSMUSG00000024268	**Celf4**	A230070D14Rik|BRUNOL-4|Brul4|Brunol4|C130060B05Rik	CUGBP, Elav-like family member 4	LOSS	protein-coding
711_T12,T1,T2	chr18	9536501	1E+07	195999	+	**3**	**37.5**	NA	Intergenic	Intergenic	-15031	NM_007593	26369	Mm.195831	NM_007593	ENSMUSG00000050996	**Cetn1**	caltractin	centrin 1	LOSS	protein-coding
807_T12,T1,T32	chr18	1.6E+07	2E+07	22999	+	**3**	**37.5**	NA	Intergenic	Intergenic	-202954	NM_199055	71367	Mm.329304	NM_199055	ENSMUSG00000047161	**Chst9**	5430438D01Rik	carbohydrate (N-acetylgalactosamine 4-0) sulfotransferase 9	LOSS	protein-coding
811_T12,T1,T2	chr18	1.6E+07	2E+07	3999	+	**3**	**37.5**	NA	Intergenic	Intergenic	-261454	NM_199055	71367	Mm.329304	NM_199055	ENSMUSG00000047161	**Chst9**	5430438D01Rik	carbohydrate (N-acetylgalactosamine 4-0) sulfotransferase 9	LOSS	protein-coding
794_T12,T1,T2	chr18	1.6E+07	2E+07	49999	+	**3**	**37.5**	NA	intron (NM_199055, intron 1 of 3)	intron (NM_199055, intron 1 of 3)	95546	NM_199055	71367	Mm.329304	NM_199055	ENSMUSG00000047161	**Chst9**	5430438D01Rik	carbohydrate (N-acetylgalactosamine 4-0) sulfotransferase 9	LOSS	protein-coding

797_T12,T1,T2	chr18	1.6E+07	2E+07	999	+	**3**	**37.5**	NA	intron (NM_199055, intron 1 of 3)	intron (NM_199055, intron 1 of 3)	63046	NM_199055	71367	Mm.329304	NM_199055	ENSMUSG00000047161	**Chst9**	5430438D01Rik	carbohydrate (N-acetylgalactosamine 4-0) sulfotransferase 9	LOSS	protein-coding
799_T12,T1,T2	chr18	1.6E+07	2E+07	8999	+	**3**	**37.5**	NA	intron (NM_199055, intron 1 of 3)	L1_Mus2|LINE|L1	27046	NM_199055	71367	Mm.329304	NM_199055	ENSMUSG00000047161	**Chst9**	5430438D01Rik	carbohydrate (N-acetylgalactosamine 4-0) sulfotransferase 9	LOSS	protein-coding
713_T12,T1,T2	chr18	9735501	1E+07	36999	+	**3**	**37.5**	NA	intron (NM_130449, intron 2 of 9)	intron (NM_130449, intron 2 of 9)	46352	NM_130449	140792	Mm.218571	NM_130449	ENSMUSG00000036103	**Colec12**	CL-P1|SRCL|Scara4	collectin sub-family member 12	LOSS	protein-coding
715_T12,T1,T2	chr18	9773501	1E+07	51999	+	**3**	**37.5**	NA	intron (NM_130449, intron 2 of 9)	PB1D10|SINE|Alu	91852	NM_130449	140792	Mm.218571	NM_130449	ENSMUSG00000036103	**Colec12**	CL-P1|SRCL|Scara4	collectin sub-family member 12	LOSS	protein-coding
1598_T22,T2,T3	chr18	5.4E+07	5E+07	999	+	**3**	**37.5**	NA	Intergenic	Intergenic	-29113	NM_152809	70425	Mm.368668	NM_152809	ENSMUSG00000073563	**Csnk1g3**	3300002K07Rik|C330049O21Rik	casein kinase 1, gamma 3	LOSS	protein-coding
2136_T1,T2,T32	chr18	8.5E+07	8E+07	2999	+	**3**	**37.5**	NA	Intergenic	(CCTG)n|Simple_repeat|Simple_repeat	-19414	NM_025797	109672	Mm.31018	NM_025797	ENSMUSG00000024646	**Cyb5a**	0610009N12Rik|Cyb5	cytochrome b5 type A (microsomal)	LOSS	protein-coding
1922_T12,T1,T32	chr18	7.3E+07	7E+07	63999	+	**3**	**37.5**	NA	Intergenic	Intergenic	-194431	NM_007831	13176	Mm.167882	NM_007831	ENSMUSG00000060534	**Dcc**	C030036D22Rik|Igdcc1	deleted in colorectal carcinoma	LOSS	protein-coding
1926_T12,T1,T2	chr18	7.3E+07	7E+07	22999	+	**3**	**37.5**	NA	Intergenic	Intergenic	-288931	NM_007831	13176	Mm.167882	NM_007831	ENSMUSG00000060534	**Dcc**	C030036D22Rik|Igdcc1	deleted in colorectal carcinoma	LOSS	protein-coding
1928_T12,T1,T2	chr18	7.3E+07	7E+07	103999	+	**3**	**37.5**	NA	Intergenic	L1_Mus3|LINE|L1	-361431	NM_007831	13176	Mm.167882	NM_007831	ENSMUSG00000060534	**Dcc**	C030036D22Rik|Igdcc1	deleted in colorectal carcinoma	LOSS	protein-coding
1929_T1,T22,T2	chr18	7.3E+07	7E+07	1999	+	**3**	**37.5**	NA	Intergenic	Intergenic	-424431	NM_007831	13176	Mm.167882	NM_007831	ENSMUSG00000060534	**Dcc**	C030036D22Rik|Igdcc1	deleted in colorectal carcinoma	LOSS	protein-coding
1931_T12,T1,T2	chr18	7.3E+07	7E+07	1999	+	**3**	**37.5**	NA	Intergenic	Lx|LINE|L1	-445431	NM_007831	13176	Mm.167882	NM_007831	ENSMUSG00000060534	**Dcc**	C030036D22Rik|Igdcc1	deleted in colorectal carcinoma	LOSS	protein-coding
1913_T12,T1,T2	chr18	7.2E+07	7E+07	327999	+	**3**	**37.5**	NA	intron (NM_007831, intron 1 of 28)	B3|SINE|B2	242569	NM_007831	13176	Mm.167882	NM_007831	ENSMUSG00000060534	**Dcc**	C030036D22Rik|Igdcc1	deleted in colorectal carcinoma	LOSS	protein-coding
1917_T12,T2,T42	chr18	7.2E+07	7E+07	2999	+	**3**	**37.5**	NA	intron (NM_007831, intron 1 of 28)	intron (NM_007831, intron 1 of 28)	36069	NM_007831	13176	Mm.167882	NM_007831	ENSMUSG00000060534	**Dcc**	C030036D22Rik|Igdcc1	deleted in colorectal carcinoma	LOSS	protein-coding

1908_T1,T2,T32	chr18	7.2E+07	7E+07	999	+	**3**	**37.5**	NA	intron (NM_007831, intron 5 of 28)	intron (NM_007831, intron 5 of 28)	559069	NM_007831	13176	Mm.167882	NM_007831	ENSMUSG00000060534	**Dcc**	C030036D22Rik|Igdcc1	deleted in colorectal carcinoma	LOSS	protein-coding
1910_T12,T2,T32	chr18	7.2E+07	7E+07	999	+	**3**	**37.5**	NA	intron (NM_007831, intron 5 of 28)	intron (NM_007831, intron 5 of 28)	550069	NM_007831	13176	Mm.167882	NM_007831	ENSMUSG00000060534	**Dcc**	C030036D22Rik|Igdcc1	deleted in colorectal carcinoma	LOSS	protein-coding
1894_T12,T1,T2	chr18	7.2E+07	7E+07	16999	+	**3**	**37.5**	NA	intron (NM_007831, intron 7 of 28)	Lx2B|LINE|L1	728069	NM_007831	13176	Mm.167882	NM_007831	ENSMUSG00000060534	**Dcc**	C030036D22Rik|Igdcc1	deleted in colorectal carcinoma	LOSS	protein-coding
1897_T12,T22,T2	chr18	7.2E+07	7E+07	999	+	**3**	**37.5**	NA	intron (NM_007831, intron 7 of 28)	intron (NM_007831, intron 7 of 28)	683069	NM_007831	13176	Mm.167882	NM_007831	ENSMUSG00000060534	**Dcc**	C030036D22Rik|Igdcc1	deleted in colorectal carcinoma	LOSS	protein-coding
593_T22,T2,T4	chr14	5.5E+07	6E+07	3999	+	**3**	**37.5**	NA	intron (NM_027790, intron 2 of 10)	IAPEz-int|LTR|ERVK	6493	NM_027790	71412	Mm.85484	NM_027790	ENSMUSG00000022209	**Dhrs2**	5430405K24Rik|Hep27	dehydrogenase/reductase member 2	LOSS	protein-coding
2252_T1,T22,T2	chr18	9E+07	9E+07	999	+	**3**	**37.5**	NA	Intergenic	Intergenic	-100864	NM_001039173	623279	Mm.447164	NM_001039173	ENSMUSG00000073514	**Dok6**	Dok-6	docking protein 6	LOSS	protein-coding
2256_T12,T1,T32	chr18	9E+07	9E+07	64999	+	**3**	**37.5**	NA	Intergenic	Intergenic	-180864	NM_001039173	623279	Mm.447164	NM_001039173	ENSMUSG00000073514	**Dok6**	Dok-6	docking protein 6	LOSS	protein-coding
2266_T12,T2,T3	chr18	9E+07	9E+07	4999	+	**3**	**37.5**	NA	Intergenic	L1Md_A|LINE|L1	-287864	NM_001039173	623279	Mm.447164	NM_001039173	ENSMUSG00000073514	**Dok6**	Dok-6	docking protein 6	LOSS	protein-coding
2268_T12,T2,T32	chr18	9E+07	9E+07	9999	+	**3**	**37.5**	NA	Intergenic	L1Md_T|LINE|L1	-318364	NM_001039173	623279	Mm.447164	NM_001039173	ENSMUSG00000073514	**Dok6**	Dok-6	docking protein 6	LOSS	protein-coding
2241_T12,T1,T2	chr18	9E+07	9E+07	42999	+	**3**	**37.5**	NA	intron (NM_001039173, intron 1 of 7)	Lx3C|LINE|L1	108136	NM_001039173	623279	Mm.447164	NM_001039173	ENSMUSG00000073514	**Dok6**	Dok-6	docking protein 6	LOSS	protein-coding
2242_T12,T1,T32	chr18	9E+07	9E+07	13999	+	**3**	**37.5**	NA	intron (NM_001039173, intron 1 of 7)	intron (NM_001039173, intron 1 of 7)	72636	NM_001039173	623279	Mm.447164	NM_001039173	ENSMUSG00000073514	**Dok6**	Dok-6	docking protein 6	LOSS	protein-coding
2239_T12,T1,T2	chr18	9E+07	9E+07	108999	+	**3**	**37.5**	NA	intron (NM_001039173, intron 2 of 7)	B4A|SINE|B4	189136	NM_001039173	623279	Mm.447164	NM_001039173	ENSMUSG00000073514	**Dok6**	Dok-6	docking protein 6	LOSS	protein-coding

1343_T12,T1,T32	chr18	4.3E+07	4E+07	9999	+	**3**	**37.5**	NA	intron (NM_001136086, intron 9 of 13)	intron (NM_001136086, intron 9 of 13)	39772	NM_001136086	22240	Mm.8180	NM_009468	ENSMUSG00000024501	**Dpysl3**	CRMP-4|DRP-3|TUC4|ULIP-1|Ulip|Ulip1	dihydropyrimidinase-like 3	LOSS	protein-coding
879_T1,T2,T32	chr18	1.9E+07	2E+07	15999	+	**3**	**37.5**	NA	Intergenic	Lx2B|LINE|L1	1134597	NM_007882	13507	Mm.89935	NM_007882	ENSMUSG00000059898	**Dsc3**	5430426I24Rik	desmocollin 3	LOSS	protein-coding
888_T1,T2,T32	chr18	1.9E+07	2E+07	1999	+	**3**	**37.5**	NA	Intergenic	ERVL-B4-int|LTR|ERVL	779597	NM_007882	13507	Mm.89935	NM_007882	ENSMUSG00000059898	**Dsc3**	5430426I24Rik	desmocollin 3	LOSS	protein-coding
898_T12,T1,T2	chr18	1.9E+07	2E+07	999	+	**3**	**37.5**	NA	Intergenic	ORR1A2|LTR|ERVL-MaLR	567097	NM_007882	13507	Mm.89935	NM_007882	ENSMUSG00000059898	**Dsc3**	5430426I24Rik	desmocollin 3	LOSS	protein-coding
900_T12,T2,T32	chr18	1.9E+07	2E+07	999	+	**3**	**37.5**	NA	Intergenic	Intergenic	560097	NM_007882	13507	Mm.89935	NM_007882	ENSMUSG00000059898	**Dsc3**	5430426I24Rik	desmocollin 3	LOSS	protein-coding
902_T12,T2,T32	chr18	1.9E+07	2E+07	999	+	**3**	**37.5**	NA	Intergenic	L1Md_F2|LINE|L1	554097	NM_007882	13507	Mm.89935	NM_007882	ENSMUSG00000059898	**Dsc3**	5430426I24Rik	desmocollin 3	LOSS	protein-coding
904_T12,T1,T32	chr18	2E+07	2E+07	1999	+	**3**	**37.5**	NA	Intergenic	L1_Mur1|LINE|L1	463597	NM_007882	13507	Mm.89935	NM_007882	ENSMUSG00000059898	**Dsc3**	5430426I24Rik	desmocollin 3	LOSS	protein-coding
913_T12,T1,T2	chr18	2E+07	2E+07	190999	+	**3**	**37.5**	NA	Intergenic	L1Md_F3|LINE|L1	284097	NM_007882	13507	Mm.89935	NM_007882	ENSMUSG00000059898	**Dsc3**	5430426I24Rik	desmocollin 3	LOSS	protein-coding
915_T12,T1,T2	chr18	2E+07	2E+07	43999	+	**3**	**37.5**	NA	Intergenic	L1_Mur3|LINE|L1	161597	NM_007882	13507	Mm.89935	NM_007882	ENSMUSG00000059898	**Dsc3**	5430426I24Rik	desmocollin 3	LOSS	protein-coding
916_T12,T1,T32	chr18	2E+07	2E+07	6999	+	**3**	**37.5**	NA	Intergenic	L1_Mus1|LINE|L1	130097	NM_007882	13507	Mm.89935	NM_007882	ENSMUSG00000059898	**Dsc3**	5430426I24Rik	desmocollin 3	LOSS	protein-coding
918_T1,T2,T32	chr18	2E+07	2E+07	999	+	**3**	**37.5**	NA	Intergenic	Intergenic	125097	NM_007882	13507	Mm.89935	NM_007882	ENSMUSG00000059898	**Dsc3**	5430426I24Rik	desmocollin 3	LOSS	protein-coding
920_T1,T2,T32	chr18	2E+07	2E+07	999	+	**3**	**37.5**	NA	Intergenic	Lx2B2|LINE|L1	115097	NM_007882	13507	Mm.89935	NM_007882	ENSMUSG00000059898	**Dsc3**	5430426I24Rik	desmocollin 3	LOSS	protein-coding
926_T12,T1,T2	chr18	2E+07	2E+07	8999	+	**3**	**37.5**	NA	Intergenic	Intergenic	-7304	NM_030596	13512	Mm.106811	NM_030596	ENSMUSG00000056632	**Dsg3**	bal	desmoglein 3	LOSS	protein-coding
976_T12,T2,T32	chr18	2.3E+07	2E+07	2999	+	**3**	**37.5**	NA	intron (NM_001285813, intron 1 of 21)	L1Md_A|LINE|L1	-8409	NM_010087	13527	Mm.94371	NM_010087	ENSMUSG00000024302	**Dtna**	2210407P21Rik|DTN-A|Dtn|Gm19389|a-DB-1|adbn	dystrobrevin alpha	LOSS	protein-coding
1488_T12,T2,T32	chr18	5E+07	5E+07	6999	+	**3**	**37.5**	NA	Intergenic	Intergenic	89601	NM_026854	68857	Mm.96010	NM_026854	ENSMUSG00000024505	**Dtwd2**	1190002H09Rik|8030470C17Rik|AI414673|BB115449	DTW domain containing 2	LOSS	protein-coding
1852_T12,T1,T2	chr18	7E+07	7E+07	30999	+	**3**	**37.5**	NA	Intergenic	L1Md_F3|LINE|L1	-51416	NM_029346	75577	Mm.58214	NM_029346	ENSMUSG00000024512	**Dynap**	2310002L13Rik	dynactin associated protein	LOSS	protein-coding

282_T22,T2,T3	chr14	3.1E+07	3E+07	390999	+	**3**	**37.5**	NA	intron (NM_009937, intron 12 of 16)	intron (NM_009937, intron 12 of 16)	38921	NM_028932	74427	Mm.491208	NM_028932	ENSMUSG00000021890	**Eaf1**	4933403C17Rik|AI666536	ELL associated factor 1	LOSS	protein-coding
985_T12,T1,T2	chr18	2.5E+07	2E+07	93999	+	**3**	**37.5**	NA	intron (NM_021448, intron 13 of 21)	B1_Mur1|SINE|Alu	19539	NM_021448	58523	Mm.25298	NM_021448	ENSMUSG00000024271	**Elp2**	AU023723|Epl2|StIP1|Statip1	elongator acetyltransferase complex subunit 2	LOSS	protein-coding
2022_T12,T1,T2	chr18	7.8E+07	8E+07	5999	+	**3**	**37.5**	NA	intron (NM_001195633, intron 26 of 43)	B3A|SINE|B2	58033	NM_001195633	1E+08	Mm.297782	NM_001195633	ENSMUSG00000039840	**Epg5**	4732475F16|5430411K18Rik|AI661957|AW456499|mKIAA1632	ectopic P-granules autophagy protein 5 homolog (C. elegans)	LOSS	protein-coding
1162_T12,T1,T22	chr18	3.2E+07	3E+07	4999	+	**3**	**37.5**	NA	Intergenic	Intergenic	56669	NM_133658	13872	Mm.282335	NM_133658	ENSMUSG00000024382	**Ercc3**	BTF2 p89|Ercc-3|XPB	excision repair cross-complementing rodent repair deficiency, complementation group 3	LOSS	protein-coding
1494_T22,T2,T3	chr18	5E+07	5E+07	1999	+	**3**	**37.5**	NA	Intergenic	L1_Mus2|LINE|L1	-54869	NM_001004061	225497	Mm.329435	NM_001004061	ENSMUSG00000035420	**Fam170a**	Gm93|Znfd	family with sequence similarity 170, member A	LOSS	protein-coding
1774_T12,T1,T2	chr18	6.4E+07	6E+07	2999	+	**3**	**37.5**	NA	Intergenic	L3|LINE|CR1	40066	NM_007998	14151	Mm.1070	NM_007998	ENSMUSG00000024588	**Fech**	AI894116|Fcl|fch	ferrochelatase	LOSS	protein-coding
987_T12,T1,T2	chr18	2.5E+07	2E+07	39999	+	**3**	**37.5**	NA	intron (NM_026779, intron 12 of 14)	intron (NM_026779, intron 12 of 14)	-14123	NM_001289654	225288	Mm.329322	NM_175276	ENSMUSG00000034295	**Fhod3**	A930009H06Rik|FHOS2|mKIAA1695	formin homology 2 domain containing 3	LOSS	protein-coding
989_T12,T1,T2	chr18	2.5E+07	2E+07	999	+	**3**	**37.5**	NA	intron (NM_175276, intron 1 of 26)	intron (NM_175276, intron 1 of 26)	20377	NM_001289655	225288	Mm.329322	NM_175276	ENSMUSG00000034295	**Fhod3**	A930009H06Rik|FHOS2|mKIAA1695	formin homology 2 domain containing 3	LOSS	protein-coding
991_T12,T1,T2	chr18	2.5E+07	3E+07	387999	+	**3**	**37.5**	NA	intron (NM_175276, intron 5 of 26)	intron (NM_175276, intron 5 of 26)	215877	NM_001289655	225288	Mm.329322	NM_175276	ENSMUSG00000034295	**Fhod3**	A930009H06Rik|FHOS2|mKIAA1695	formin homology 2 domain containing 3	LOSS	protein-coding
2462_T22,T42,T4	chr9	8E+07	8E+07	3999	+	**3**	**37.5**	NA	intron (NM_001081243, intron 1 of 5)	L1Md_A|LINE|L1	76382	NM_001081243	70598	Mm.23049	NM_001081243	ENSMUSG00000034898	**Filip1**	5730485H21Rik|AI843021	filamin A interacting protein 1	LOSS	protein-coding
698_T12,T1,T2	chr18	8583501	9E+06	71999	+	**3**	**37.5**	NA	Intergenic	BGLII_B|LTR|ERVK	-593356	NM_008058	14370	Mm.184289	NM_008058	ENSMUSG00000036904	**Fzd8**	Fz8	frizzled class receptor 8	LOSS	protein-coding
700_T12,T1,T2	chr18	8656501	9E+06	454999	+	**3**	**37.5**	NA	Intergenic	Intergenic	-328856	NM_008058	14370	Mm.184289	NM_008058	ENSMUSG00000036904	**Fzd8**	Fz8	frizzled class receptor 8	LOSS	protein-coding

702_T12,T1,T2	chr18	9116501	9E+06	55999	+	**3**	**37.5**	NA	Intergenic	RodERV21-int|LTR|ERV1	-68356	NM_008058	14370	Mm.184289	NM_008058	ENSMUSG00000036904	**Fzd8**	Fz8	frizzled class receptor 8	LOSS	protein-coding
704_T12,T1,T2	chr18	9177501	9E+06	84999	+	**3**	**37.5**	NA	Intergenic	ORR1D1|LTR|ERVL-MaLR	7144	NM_008058	14370	Mm.184289	NM_008058	ENSMUSG00000036904	**Fzd8**	Fz8	frizzled class receptor 8	LOSS	protein-coding
290_T22,T2,T3	chr14	3.2E+07	3E+07	359999	+	**3**	**37.5**	NA	Intergenic	Intergenic	-4603	NM_030166	78754	Mm.40681	NM_030166	ENSMUSG00000021903	**Galnt15**	4631401E18Rik|Galntl2|mpp-GalNAc-T15	UDP-N-acetyl-alpha-D-galactosamine:polypeptide N-acetylgalactosaminyltransferase 15	LOSS	protein-coding
2093_T12,T1,T2	chr18	8.2E+07	8E+07	84999	+	**3**	**37.5**	NA	Intergenic	Intergenic	203777	NM_008082	14427	Mm.6219	NM_008082	ENSMUSG00000024553	**Galr1**	Galnr1	galanin receptor 1	LOSS	protein-coding
2096_T12,T1,T2	chr18	8.2E+07	8E+07	2999	+	**3**	**37.5**	NA	Intergenic	L1Md_F2|LINE|L1	136777	NM_008082	14427	Mm.6219	NM_008082	ENSMUSG00000024553	**Galr1**	Galnr1	galanin receptor 1	LOSS	protein-coding
947_T12,T1,T2	chr18	2.1E+07	2E+07	197999	+	**3**	**37.5**	NA	Intergenic	L1Md_F3|LINE|L1	-68361	NM_001033445	381126	Mm.312276	NM_001033445	ENSMUSG00000042680	**Garem1**	C86169|Fam59a|Garem|Gm944|mKIAA4238	GRB2 associated regulator of MAPK1 subtype 1	LOSS	protein-coding
946_T1,T32,T42	chr18	2.1E+07	2E+07	999	+	**3**	**37.5**	NA	intron (NM_001033445, intron 1 of 5)	IAPLTR1_Mm|LTR|ERVK	38139	NM_001033445	381126	Mm.312276	NM_001033445	ENSMUSG00000042680	**Garem1**	C86169|Fam59a|Garem|Gm944|mKIAA4238	GRB2 associated regulator of MAPK1 subtype 1	LOSS	protein-coding
727_T12,T1,T2	chr18	1.1E+07	1E+07	136999	+	**3**	**37.5**	NA	Intergenic	Intergenic	-102510	NM_010258	14465	Mm.329287	NM_010258	ENSMUSG00000005836	**Gata6**	AA410133|GATA-6	GATA binding protein 6	LOSS	protein-coding
729_T12,T1,T4	chr18	1.1E+07	1E+07	999	+	**3**	**37.5**	NA	Intergenic	Lx6|LINE|L1	-31510	NM_010258	14465	Mm.329287	NM_010258	ENSMUSG00000005836	**Gata6**	AA410133|GATA-6	GATA binding protein 6	LOSS	protein-coding
362_T12,T22,T3	chr14	3.8E+07	4E+07	37999	+	**3**	**37.5**	NA	Intergenic	L1_Mus1|LINE|L1	-523178	NM_078478	66092	Mm.182912	NM_078478	ENSMUSG00000041028	**Ghitm**	1010001P14Rik|C77840|MICS1|PTD010|Tmbim5	growth hormone inducible transmembrane protein	LOSS	protein-coding
706_T12,T1,T2	chr18	9268501	9E+06	999	+	**3**	**37.5**	NA	Intergenic	L1Md_T|LINE|L1	13809	NM_153086	225152	Mm.119273	NM_153086	ENSMUSG00000036855	**Gjd4**	9430022F06Rik|Cx39	gap junction protein, delta 4	LOSS	protein-coding
198_T22,T2,T42	chr14	2E+07	2E+07	534999	+	**3**	**37.5**	NA	Intergenic	MLT1F|LTR|ERVL-MaLR	-7751	NM_001285909	14702	Mm.41737	NM_010315	ENSMUSG00000043004	**Gng2**	-	guanine nucleotide binding protein (G protein), gamma 2	LOSS	protein-coding
464_T12,T22,T3	chr14	4.5E+07	5E+07	999	+	**3**	**37.5**	NA	intron (NM_019637, intron 9 of 10)	intron (NM_019637, intron 9 of 10)	16796	NM_019425	54342	Mm.312945	NM_019425	ENSMUSG00000037722	**Gnpnat1**	AU017428|AU040593|EMeg32|Gpnat1|Gsnpat	glucosamine-phosphate N-acetyltransferase 1	LOSS	protein-coding
2457_T2,T42,T4	chr8	5.5E+07	6E+07	3999	+	**3**	**37.5**	NA	Intergenic	(GA)n|Simple_repeat|Simple_repeat	413772	NM_001253756	234267	Mm.241700	NM_153581	ENSMUSG00000031517	**Gpm6a**	Gpm6|M6A	glycoprotein m6a	LOSS	protein-coding
1339_T12,T1,T22	chr18	4.3E+07	4E+07	999	+	**3**	**37.5**	NA	Intergenic	Intergenic	-14348	NM_181543	240239	Mm.186779	NM_181543	ENSMUSG00000042816	**Gpr151**	C130082O03Rik|GalRL|PGR7|nGPCR-2037	G protein-coupled receptor 151	LOSS	protein-coding

1150_T12,T1,T32	chr18	3.2E+07	3E+07	999	+	**3**	**37.5**	NA	intron (NM_144862, intron 1 of 9)	Lx4A|LINE|L1	8636	NM_001025381	574402	Mm.391108	NM_001025381	ENSMUSG00000052229	**Gpr17**	AI853548	G protein-coupled receptor 17	LOSS	protein-coding
1153_T12,T1,T32	chr18	3.2E+07	3E+07	19999	+	**3**	**37.5**	NA	intron (NM_144862, intron 4 of 9)	intron (NM_144862, intron 4 of 9)	-3864	NM_001025381	574402	Mm.391108	NM_001025381	ENSMUSG00000052229	**Gpr17**	AI853548	G protein-coupled receptor 17	LOSS	protein-coding
81_T12,T1,T42	chr12	1E+08	1E+08	3999	+	**3**	**37.5**	NA	exon (NM_010351, exon 3 of 3)	exon (NM_010351, exon 3 of 3)	1736	NM_010351	14836	Mm.129	NM_010351	ENSMUSG00000021095	**Gsc**	-	goosecoid homeobox	LOSS	protein-coding
1213_T12,T1,T2	chr18	3.6E+07	4E+07	58999	+	**3**	**37.5**	NA	intron (NM_010415, intron 3 of 5)	intron (NM_010415, intron 3 of 5)	3805	NM_010415	15200	Mm.289681	NM_010415	ENSMUSG00000024486	**Hbegf**	AW047313|Dtr|Dts|Hegfl	heparin-binding EGF-like growth factor	LOSS	protein-coding
763_T12,T1,T32	chr18	1.3E+07	1E+07	32999	+	**3**	**37.5**	NA	Intergenic	LTR72_RN|LTR|ERV1	310010	NM_153087	225192	Mm.207073	NM_153087	ENSMUSG00000037346	**Hrh4**	AXOR35|BG26|GPCR105|GPRv53|H4|H4R|HH4R	histamine receptor H4	LOSS	protein-coding
1737_T12,T1,T2	chr18	6.2E+07	6E+07	30999	+	**3**	**37.5**	NA	Intergenic	L1MB7|LINE|L1	-53204	NM_008313	15562	Mm.20440	NM_008313	ENSMUSG00000026322	**Htr4**	5-HT-4|5-HT4|5-HT<4L>|5HTR4	5 hydroxytryptamine (serotonin) receptor 4	LOSS	protein-coding
1738_T12,T2,T32	chr18	6.2E+07	6E+07	999	+	**3**	**37.5**	NA	Intergenic	L1Md_T|LINE|L1	-31204	NM_008313	15562	Mm.20440	NM_008313	ENSMUSG00000026322	**Htr4**	5-HT-4|5-HT4|5-HT<4L>|5HTR4	5 hydroxytryptamine (serotonin) receptor 4	LOSS	protein-coding
1200_T12,T32,T3	chr18	3.6E+07	4E+07	1999	+	**3**	**37.5**	NA	Intergenic	Intergenic	-8601	NM_001267796	109169	Mm.426423	NM_001267796	ENSMUSG00000110185	**Igip**	6330403M23Rik	IgA inducing protein	LOSS	protein-coding
219_T22,T2,T42	chr14	2.1E+07	2E+07	4999	+	**3**	**37.5**	NA	intron (NM_134079, intron 7 of 10)	intron (NM_134079, intron 7 of 10)	-142770	NM_001205241	54169	Mm.248967	NM_017479	ENSMUSG00000021767	**Kat6b**	AI507552|B130044K16Rik|MYST-4|Morf|Myst4|mKIAA0383|qkf|querkopf	K(lysine) acetyltransferase 6B	LOSS	protein-coding
2067_T12,T1,T2	chr18	8E+07	8E+07	198999	+	**3**	**37.5**	NA	Intergenic	Intergenic	-41746	NM_001190373	240444	Mm.279568	NM_001190373	ENSMUSG00000059852	**Kcng2**	AA591524	potassium voltage-gated channel, subfamily G, member 2	LOSS	protein-coding
200_T22,T2,T42	chr14	2E+07	2E+07	19999	+	**3**	**37.5**	NA	intron (NM_029006, intron 1 of 4)	intron (NM_029006, intron 1 of 4)	1662	NM_029006	74571	Mm.105571	NM_029006	ENSMUSG00000023387	**Kcnk16**	4731413G05Rik|TALK-1|TALK1	potassium channel, subfamily K, member 16	LOSS	protein-coding
1383_T12,T1,T2	chr18	4.5E+07	5E+07	258999	+	**3**	**37.5**	NA	Intergenic	L1Md_F2|LINE|L1	-106860	NM_001312905	140492	Mm.411614	NM_080465	ENSMUSG00000054477	**Kcnn2**	KCa2.2|SK2|SKCA2|bc|fri	potassium intermediate/small conductance calcium-activated channel, subfamily N, member 2	LOSS	protein-coding

1395_T12,T1,T2	chr18	4.6E+07	5E+07	999	+	**3**	**37.5**	NA	intron (NM_001312905, intron 2 of 10)	L1Md_T|LINE|L1	-17154	NM_080465	140492	Mm.411614	NM_080465	ENSMUSG00000054477	**Kcnn2**	KCa2.2|SK2|SKCA2|bc|fri	potassium intermediate/small conductance calcium-activated channel, subfamily N, member 2	LOSS	protein-coding
784_T12,T1,T2	chr18	1.5E+07	2E+07	25999	+	**3**	**37.5**	NA	Intergenic	MLT1H|LTR|ERVL-MaLR	-43054	NM_134112	106931	Mm.329299	NM_134112	ENSMUSG00000036225	**Kctd1**	4933402K10Rik|AI661543|AW553000	potassium channel tetramerisation domain containing 1	LOSS	protein-coding
786_T1,T22,T2	chr18	1.5E+07	2E+07	999	+	**3**	**37.5**	NA	Intergenic	RLTR10F|LTR|ERVK	-73554	NM_134112	106931	Mm.329299	NM_134112	ENSMUSG00000036225	**Kctd1**	4933402K10Rik|AI661543|AW553000	potassium channel tetramerisation domain containing 1	LOSS	protein-coding
1274_T1,T22,T2	chr18	4.1E+07	4E+07	3999	+	**3**	**37.5**	NA	Intergenic	L1M2|LINE|L1	304139	NM_026135	383348	Mm.76366	NM_026135	ENSMUSG00000051401	**Kctd16**	4930434H12Rik|Gm1267	potassium channel tetramerisation domain containing 16	LOSS	protein-coding
1276_T1,T22,T2	chr18	4.1E+07	4E+07	999	+	**3**	**37.5**	NA	Intergenic	L1MD3|LINE|L1	311639	NM_026135	383348	Mm.76366	NM_026135	ENSMUSG00000051401	**Kctd16**	4930434H12Rik|Gm1267	potassium channel tetramerisation domain containing 16	LOSS	protein-coding
1283_T12,T1,T2	chr18	4.1E+07	4E+07	30999	+	**3**	**37.5**	NA	Intergenic	Intergenic	373639	NM_026135	383348	Mm.76366	NM_026135	ENSMUSG00000051401	**Kctd16**	4930434H12Rik|Gm1267	potassium channel tetramerisation domain containing 16	LOSS	protein-coding
1286_T12,T1,T2	chr18	4.1E+07	4E+07	16999	+	**3**	**37.5**	NA	Intergenic	Intergenic	463639	NM_026135	383348	Mm.76366	NM_026135	ENSMUSG00000051401	**Kctd16**	4930434H12Rik|Gm1267	potassium channel tetramerisation domain containing 16	LOSS	protein-coding
1290_T12,T2,T32	chr18	4.1E+07	4E+07	3999	+	**3**	**37.5**	NA	Intergenic	Intergenic	656139	NM_026135	383348	Mm.76366	NM_026135	ENSMUSG00000051401	**Kctd16**	4930434H12Rik|Gm1267	potassium channel tetramerisation domain containing 16	LOSS	protein-coding
1269_T1,T2,T32	chr18	4E+07	4E+07	5999	+	**3**	**37.5**	NA	intron (NM_026135, intron 1 of 1)	intron (NM_026135, intron 1 of 1)	197139	NM_026135	383348	Mm.76366	NM_026135	ENSMUSG00000051401	**Kctd16**	4930434H12Rik|Gm1267	potassium channel tetramerisation domain containing 16	LOSS	protein-coding
1271_T1,T22,T2	chr18	4.1E+07	4E+07	47999	+	**3**	**37.5**	NA	intron (NM_026135, intron 1 of 1)	intron (NM_026135, intron 1 of 1)	270139	NM_026135	383348	Mm.76366	NM_026135	ENSMUSG00000051401	**Kctd16**	4930434H12Rik|Gm1267	potassium channel tetramerisation domain containing 16	LOSS	protein-coding
2397_T2,T32,T3	chr6	1.3E+08	1E+08	6999	+	**3**	**37.5**	NA	TTS (NM_001098669)	TTS (NM_001098669)	4556	NM_010653	16642	Mm.347927	NM_010653	ENSMUSG00000052736	**Klrc2**	NKG2C	killer cell lectin-like receptor subfamily C, member 2	LOSS	protein-coding
486_T12,T22,T2	chr14	4.8E+07	5E+07	2999	+	**3**	**37.5**	NA	Intergenic	RLTR10-int|LTR|ERVK	-40311	NM_001293636	16709	Mm.3110	NM_008477	ENSMUSG00000021843	**Ktn1**	-	kinectin 1	LOSS	protein-coding
1562_T12,T1,T2	chr18	5.3E+07	5E+07	9999	+	**3**	**37.5**	NA	Intergenic	L1Md_T|LINE|L1	-14633	NM_001286182	16948	Mm.172	NM_010728	ENSMUSG00000024529	**Lox**	AI893619|TSC-160|rrg	lysyl oxidase	LOSS	protein-coding
2004_T12,T1,T2	chr18	7.7E+07	8E+07	211999	+	**3**	**37.5**	NA	intron (NM_172834, intron 19 of 38)	intron (NM_172834, intron 19 of 38)	95542	NM_172834	240411	Mm.380247	NM_172834	ENSMUSG00000032818	**Loxhd1**	1700096C21Rik|4932417K07|sba	lipoxygenase homology domains 1	LOSS	protein-coding
1956_T12,T1,T2	chr18	7.4E+07	7E+07	405999	+	**3**	**37.5**	NA	Intergenic	MTEa|LTR|ERVL-MaLR	-57551	NM_172632	225724	Mm.254517	NM_172632	ENSMUSG00000024558	**Mapk4**	A330097D03Rik|Erk3|Prkm4|p63Mapk	mitogen-activated protein kinase 4	LOSS	protein-coding

298_T12,T2,T32	chr14	3.3E+07	3E+07	61999	+	**3**	**37.5**	NA	promoter-TSS (NM_016700)	promoter-TSS (NM_016700)	-342	NM_016700	26419	Mm.21495	NM_016700	ENSMUSG00000021936	**Mapk8**	AI849689|JNK|JNK1|Prkm8|SAPK1	mitogen-activated protein kinase 8	LOSS	protein-coding
1863_T12,T2,T42	chr18	7.1E+07	7E+07	999	+	**3**	**37.5**	NA	Intergenic	Intergenic	177708	NM_010773	17191	Mm.322	NM_010773	ENSMUSG00000024513	**Mbd2**	MBD2a	methyl-CpG binding domain protein 2	LOSS	protein-coding
1865_T12,T1,T32	chr18	7.1E+07	7E+07	6999	+	**3**	**37.5**	NA	Intergenic	Intergenic	216708	NM_010773	17191	Mm.322	NM_010773	ENSMUSG00000024513	**Mbd2**	MBD2a	methyl-CpG binding domain protein 2	LOSS	protein-coding
1867_T12,T1,T32	chr18	7.1E+07	7E+07	12999	+	**3**	**37.5**	NA	Intergenic	Intergenic	231708	NM_010773	17191	Mm.322	NM_010773	ENSMUSG00000024513	**Mbd2**	MBD2a	methyl-CpG binding domain protein 2	LOSS	protein-coding
1869_T12,T1,T32	chr18	7.1E+07	7E+07	20999	+	**3**	**37.5**	NA	Intergenic	Lx8|LINE|L1	262708	NM_010773	17191	Mm.322	NM_010773	ENSMUSG00000024513	**Mbd2**	MBD2a	methyl-CpG binding domain protein 2	LOSS	protein-coding
1873_T12,T2,T32	chr18	7.1E+07	7E+07	1999	+	**3**	**37.5**	NA	Intergenic	BC1_Mm|scRNA|scRNA	584208	NM_010773	17191	Mm.322	NM_010773	ENSMUSG00000024513	**Mbd2**	MBD2a	methyl-CpG binding domain protein 2	LOSS	protein-coding
1884_T12,T2,T32	chr18	7.1E+07	7E+07	13999	+	**3**	**37.5**	NA	intron (NM_007831, intron 14 of 28)	ORR1A2-int|LTR|ERVL-MaLR	864208	NM_010773	17191	Mm.322	NM_010773	ENSMUSG00000024513	**Mbd2**	MBD2a	methyl-CpG binding domain protein 2	LOSS	protein-coding
1880_T12,T2,T32	chr18	7.1E+07	7E+07	6999	+	**3**	**37.5**	NA	intron (NM_007831, intron 21 of 28)	RMER15|LTR|ERVL	782708	NM_010773	17191	Mm.322	NM_010773	ENSMUSG00000024513	**Mbd2**	MBD2a	methyl-CpG binding domain protein 2	LOSS	protein-coding
1876_T12,T1,T3	chr18	7.1E+07	7E+07	15999	+	**3**	**37.5**	NA	intron (NM_007831, intron 23 of 28)	intron (NM_007831, intron 23 of 28)	760208	NM_010773	17191	Mm.322	NM_010773	ENSMUSG00000024513	**Mbd2**	MBD2a	methyl-CpG binding domain protein 2	LOSS	protein-coding
1859_T12,T2,T32	chr18	7.1E+07	7E+07	999	+	**3**	**37.5**	NA	intron (NM_010773, intron 3 of 6)	intron (NM_010773, intron 3 of 6)	47708	NM_010773	17191	Mm.322	NM_010773	ENSMUSG00000024513	**Mbd2**	MBD2a	methyl-CpG binding domain protein 2	LOSS	protein-coding
2099_T12,T1,T2	chr18	8.2E+07	8E+07	187999	+	**3**	**37.5**	NA	intron (NM_010777, intron 3 of 6)	(TGGA)n|Simple_repeat|Simple_repeat	-9963	NM_001025256	17196	Mm.252063	NM_010777	ENSMUSG00000041607	**Mbp**	C76307|Hmbpr|R75289|golli-mbp|jve|mld|shi	myelin basic protein	LOSS	protein-coding
1828_T12,T1,T2	chr18	6.8E+07	7E+07	18999	+	**3**	**37.5**	NA	Intergenic	Intergenic	-20680	NM_001271716	17200	Mm.426053	NM_008560	ENSMUSG00000045569	**Mc2r**	ACTH-R|ACTHR|MC2-R	melanocortin 2 receptor	LOSS	protein-coding
1830_T12,T1,T2	chr18	6.8E+07	7E+07	10999	+	**3**	**37.5**	NA	Intergenic	Intergenic	-39680	NM_001271716	17200	Mm.426053	NM_008560	ENSMUSG00000045569	**Mc2r**	ACTH-R|ACTHR|MC2-R	melanocortin 2 receptor	LOSS	protein-coding

1826_T12,T1,T2	chr18	6.8E+07	7E+07	4999	+	**3**	**37.5**	NA	intron (NM_001301372, intron 1 of 4)	L1Md_F2|LINE|L1	2840	NM_001271717	17200	Mm.426053	NM_008560	ENSMUSG00000045569	**Mc2r**	ACTH-R|ACTHR|MC2-R	melanocortin 2 receptor	LOSS	protein-coding
26_T12,T32,T3	chr11	1.9E+07	2E+07	3999	+	**3**	**37.5**	NA	Intergenic	Intergenic	141469	NM_001193271	17268	Mm.356578	NM_010789	ENSMUSG00000020160	**Meis1**	C530044H18Rik|Evi8	Meis homeobox 1	LOSS	protein-coding
1937_T12,T2,T32	chr18	7.3E+07	7E+07	8999	+	**3**	**37.5**	NA	Intergenic	ID_B1|SINE|B4	-428705	NM_001039214	240396	Mm.24131	NM_001039214	ENSMUSG00000037253	**Mex3c**	A130001D14Rik|BC035207|BM-013|Rkhd2	mex3 RNA binding family member C	LOSS	protein-coding
1941_T12,T1,T2	chr18	7.3E+07	7E+07	22999	+	**3**	**37.5**	NA	Intergenic	Intergenic	-296705	NM_001039214	240396	Mm.24131	NM_001039214	ENSMUSG00000037253	**Mex3c**	A130001D14Rik|BC035207|BM-013|Rkhd2	mex3 RNA binding family member C	LOSS	protein-coding
1943_T12,T1,T2	chr18	7.3E+07	7E+07	30999	+	**3**	**37.5**	NA	Intergenic	Intergenic	-261705	NM_001039214	240396	Mm.24131	NM_001039214	ENSMUSG00000037253	**Mex3c**	A130001D14Rik|BC035207|BM-013|Rkhd2	mex3 RNA binding family member C	LOSS	protein-coding
1949_T1,T2,T32	chr18	7.3E+07	7E+07	999	+	**3**	**37.5**	NA	Intergenic	Intergenic	-119705	NM_001039214	240396	Mm.24131	NM_001039214	ENSMUSG00000037253	**Mex3c**	A130001D14Rik|BC035207|BM-013|Rkhd2	mex3 RNA binding family member C	LOSS	protein-coding
670_T1,T2,T32	chr18	7091501	7E+06	2999	+	**3**	**37.5**	NA	intron (NM_001081393, intron 20 of 20)	(CA)n|Simple_repeat|Simple_repeat	-88221	NM_177595	210719	Mm.257186	NM_177595	ENSMUSG00000061013	**Mkx**	9430023B20Rik|Irxl1	mohawk homeobox	LOSS	protein-coding
678_T12,T1,T2	chr18	7613501	8E+06	144999	+	**3**	**37.5**	NA	Intergenic	L1_Mus3|LINE|L1	-59137	NM_001161620	75739	Mm.133293	NM_001081287	ENSMUSG00000057440	**Mpp7**	1110068J02Rik|2810038M04Rik|5430426E14Rik|AI415104|Gm955	membrane protein, palmitoylated 7 (MAGUK p55 subfamily member 7)	LOSS	protein-coding
676_T12,T1,T2	chr18	7607501	8E+06	2999	+	**3**	**37.5**	NA	intron (NM_001161620, intron 1 of 12)	SUBTEL_sa|Satellite|Satellite	17863	NM_001081287	75739	Mm.133293	NM_001081287	ENSMUSG00000057440	**Mpp7**	1110068J02Rik|2810038M04Rik|5430426E14Rik|AI415104|Gm955	membrane protein, palmitoylated 7 (MAGUK p55 subfamily member 7)	LOSS	protein-coding
672_T1,T2,T32	chr18	7526501	8E+06	999	+	**3**	**37.5**	NA	intron (NM_001161620, intron 2 of 12)	intron (NM_001161620, intron 2 of 12)	99863	NM_001081287	75739	Mm.133293	NM_001081287	ENSMUSG00000057440	**Mpp7**	1110068J02Rik|2810038M04Rik|5430426E14Rik|AI415104|Gm955	membrane protein, palmitoylated 7 (MAGUK p55 subfamily member 7)	LOSS	protein-coding
1743_T12,T2,T32	chr18	6.3E+07	6E+07	999	+	**3**	**37.5**	NA	intron (NM_001039485, intron 40 of 53)	intron (NM_001039485, intron 40 of 53)	56084	NM_028017	108123	Mm.292687	NM_028017	ENSMUSG00000024581	**Napg**	2400003O04Rik|SNARE	N-ethylmaleimide sensitive fusion protein attachment protein gamma	LOSS	protein-coding
274_T22,T3,T42	chr14	3.1E+07	3E+07	999	+	**3**	**37.5**	NA	Intergenic	L1MB3|LINE|L1	-2377	NM_001308328	23955	Mm.251494	NM_011849	ENSMUSG00000021918	**Nek4**	Stk2	NIMA (never in mitosis gene a)-related expressed kinase 4	LOSS	protein-coding
62_T2,T3,T4	chr12	6.9E+07	7E+07	3999	+	**3**	**37.5**	NA	intron (NM_025441, intron 7 of 32)	B3|SINE|B2	8676	NM_025441	66244	Mm.46705	NM_025441	ENSMUSG00000020982	**Nemf**	1500011I12Rik|4933405E14Rik|Sdccag1	nuclear export mediator factor	LOSS	protein-coding

2149_T12,T1,T2	chr18	8.6E+07	9E+07	83999	+	**3**	**37.5**	NA	Intergenic	L1_Mus1|LINE|L1	-653452	NM_144946	246317	Mm.329578	NM_144946	ENSMUSG00000050321	**Neto1**	AI851453|Btcl1|C130005O10Rik	neuropilin (NRP) and tolloid (TLL)-like 1	LOSS	protein-coding
2151_T1,T2,T32	chr18	8.6E+07	9E+07	999	+	**3**	**37.5**	NA	Intergenic	L1Md_T|LINE|L1	-572952	NM_144946	246317	Mm.329578	NM_144946	ENSMUSG00000050321	**Neto1**	AI851453|Btcl1|C130005O10Rik	neuropilin (NRP) and tolloid (TLL)-like 1	LOSS	protein-coding
2153_T1,T2,T32	chr18	8.6E+07	9E+07	3999	+	**3**	**37.5**	NA	Intergenic	L1_Mus3|LINE|L1	-566452	NM_144946	246317	Mm.329578	NM_144946	ENSMUSG00000050321	**Neto1**	AI851453|Btcl1|C130005O10Rik	neuropilin (NRP) and tolloid (TLL)-like 1	LOSS	protein-coding
2156_T12,T1,T2	chr18	8.6E+07	9E+07	13999	+	**3**	**37.5**	NA	Intergenic	Lx9|LINE|L1	-504452	NM_144946	246317	Mm.329578	NM_144946	ENSMUSG00000050321	**Neto1**	AI851453|Btcl1|C130005O10Rik	neuropilin (NRP) and tolloid (TLL)-like 1	LOSS	protein-coding
2160_T12,T1,T2	chr18	8.6E+07	9E+07	129999	+	**3**	**37.5**	NA	Intergenic	L1Md_F2|LINE|L1	-278452	NM_144946	246317	Mm.329578	NM_144946	ENSMUSG00000050321	**Neto1**	AI851453|Btcl1|C130005O10Rik	neuropilin (NRP) and tolloid (TLL)-like 1	LOSS	protein-coding
2164_T12,T1,T2	chr18	8.6E+07	9E+07	10999	+	**3**	**37.5**	NA	Intergenic	RLTR6-int|LTR|ERV1	-198952	NM_144946	246317	Mm.329578	NM_144946	ENSMUSG00000050321	**Neto1**	AI851453|Btcl1|C130005O10Rik	neuropilin (NRP) and tolloid (TLL)-like 1	LOSS	protein-coding
2072_T12,T1,T2	chr18	8.1E+07	8E+07	110999	+	**3**	**37.5**	NA	intron (NM_001201569, intron 15 of 29)	intron (NM_001201569, intron 15 of 29)	-58929	NM_001164109	18018	Mm.329560	NM_016791	ENSMUSG00000033016	**Nfatc1**	2210017P03Rik|AI449492|AV076380|NF-ATc|NFAT2|NFATc|Nfatcb	nuclear factor of activated T cells, cytoplasmic, calcineurin dependent 1	LOSS	protein-coding
2070_T12,T1,T2	chr18	8.1E+07	8E+07	15999	+	**3**	**37.5**	NA	intron (NM_198429, intron 1 of 9)	intron (NM_198429, intron 1 of 9)	673	NM_001164111	18018	Mm.329560	NM_016791	ENSMUSG00000033016	**Nfatc1**	2210017P03Rik|AI449492|AV076380|NF-ATc|NFAT2|NFATc|Nfatcb	nuclear factor of activated T cells, cytoplasmic, calcineurin dependent 1	LOSS	protein-coding
974_T12,T2,T32	chr18	2.3E+07	2E+07	5999	+	**3**	**37.5**	NA	Intergenic	L1Md_T|LINE|L1	-15847	NM_199024	319211	Mm.209896	NM_199024	ENSMUSG00000041923	**Nol4**	1700013J13Rik|4930568N03Rik|Gm1262	nucleolar protein 4	LOSS	protein-coding
969_T12,T1,T2	chr18	2.3E+07	2E+07	999	+	**3**	**37.5**	NA	intron (NM_001161483, intron 5 of 10)	L1Md_T|LINE|L1	160663	NM_001161483	319211	Mm.209896	NM_199024	ENSMUSG00000041923	**Nol4**	1700013J13Rik|4930568N03Rik|Gm1262	nucleolar protein 4	LOSS	protein-coding
304_T12,T3,T4	chr14	3.4E+07	3E+07	5999	+	**3**	**37.5**	NA	Intergenic	ORR1F|LTR|ERVL-MaLR	-15081	NM_008919	19065	Mm.57059	NM_008919	ENSMUSG00000048337	**Npy4r**	NYYR-D|Ppyr1|Y4	neuropeptide Y receptor Y4	LOSS	protein-coding
171_T22,T2,T3	chr14	1.8E+07	2E+07	54999	+	**3**	**37.5**	NA	Intergenic	Intergenic	64106	NM_011584	353187	Mm.26587	NM_011584	ENSMUSG00000021775	**Nr1d2**	RVR|Rev-erb	nuclear receptor subfamily 1, group D, member 2	LOSS	protein-coding
1185_T12,T1,T32	chr18	3.3E+07	3E+07	999	+	**3**	**37.5**	NA	intron (NM_001267717, intron 3 of 4)	intron (NM_001267717, intron 3 of 4)	3634	NM_001267717	27528	Mm.407415	NM_053078	ENSMUSG00000042834	**Nrep**	AI325076|D0H4S114|Harp|P311|PTZ17|SEZ17	neuronal regeneration related protein	LOSS	protein-coding
292_T22,T2,T3	chr14	3.2E+07	3E+07	70999	+	**3**	**37.5**	NA	intron (NM_011960, intron 13 of 17)	intron (NM_011960, intron 13 of 17)	-57019	NM_001081130	239017	Mm.318302	NM_001081130	ENSMUSG00000021913	**Ogdhl**	-	oxoglutarate dehydrogenase-like	LOSS	protein-coding

1772_T12,T1,T2	chr18	6.4E+07	6E+07	999	+	**3**	**37.5**	NA	intron (NM_194268, intron 1 of 1)	intron (NM_194268, intron 1 of 1)	6636	NM_194268	225631	Mm.234723	NM_194268	ENSMUSG00000045991	**Onecut2**	C730009D12|OC-2|Oc2	one cut domain, family member 2	LOSS	protein-coding
2056_T2,T32,T42	chr18	8E+07	8E+07	999	+	**3**	**37.5**	NA	Intergenic	IAPEz-int|LTR|ERVK	-337895	NM_053117	93737	Mm.24678	NM_053117	ENSMUSG00000056214	**Pard6g**	2410049N21Rik|Par6a	par-6 family cell polarity regulator gamma	LOSS	protein-coding
2063_T1,T2,T32	chr18	8E+07	8E+07	999	+	**3**	**37.5**	NA	Intergenic	L1Md_T|LINE|L1	-296895	NM_053117	93737	Mm.24678	NM_053117	ENSMUSG00000056214	**Pard6g**	2410049N21Rik|Par6a	par-6 family cell polarity regulator gamma	LOSS	protein-coding
2065_T12,T1,T2	chr18	8E+07	8E+07	2999	+	**3**	**37.5**	NA	Intergenic	Intergenic	-32895	NM_053117	93737	Mm.24678	NM_053117	ENSMUSG00000056214	**Pard6g**	2410049N21Rik|Par6a	par-6 family cell polarity regulator gamma	LOSS	protein-coding
1234_T12,T2,T3	chr18	3.7E+07	4E+07	7999	+	**3**	**37.5**	NA	intron (NM_138663, intron 1 of 3)	L1Md_T|LINE|L1	22270	NM_138663	192164	Mm.308500	NM_138663	ENSMUSG00000103310	**Pcdha12**	Cnr5|Crnr5|Pcdha13	protocadherin alpha 12	LOSS	protein-coding
1236_T12,T2,T3	chr18	3.7E+07	4E+07	22999	+	**3**	**37.5**	NA	intron (NM_138663, intron 1 of 3)	L1_Mur2|LINE|L1	-27136	NM_001003671	353236	Mm.308500	NM_001003671	ENSMUSG00000103255	**Pcdhac1**	CNRc1	protocadherin alpha subfamily C, 1	LOSS	protein-coding
1240_T12,T1,T2	chr18	3.7E+07	4E+07	15999	+	**3**	**37.5**	NA	intron (NM_001301259, intron 1 of 3)	Lx|LINE|L1	-21355	NM_053134	93880	Mm.286019	NM_053134	ENSMUSG00000051242	**Pcdhb9**	Pcdhb4C|PcdhbI	protocadherin beta 9	LOSS	protein-coding
2371_T22,T32,T4	chr6	4749501	5E+06	3999	+	**3**	**37.5**	NA	intron (NM_130877, intron 1 of 1)	intron (NM_130877, intron 1 of 1)	4194	NM_130877	170676	Mm.320575	NM_130877	ENSMUSG00000092035	**Peg10**	AA407948|Edr|HB-1|MEF3L|Mar2|Mart2|MyEF-3	paternally expressed 10	LOSS	protein-coding
1745_T12,T1,T2	chr18	6.3E+07	6E+07	999	+	**3**	**37.5**	NA	Intergenic	Intergenic	-55817	NM_001039485	667742	Mm.158720	NM_172629	ENSMUSG00000041482	**Piezo2**	5930434P17|9030411M15Rik|9430028L06Rik|Fam38b|Fam38b2	piezo-type mechanosensitive ion channel component 2	LOSS	protein-coding
1746_T12,T1,T32	chr18	6.3E+07	6E+07	23999	+	**3**	**37.5**	NA	Intergenic	LTR37-int|LTR|ERV1	-74317	NM_001039485	667742	Mm.158720	NM_172629	ENSMUSG00000041482	**Piezo2**	5930434P17|9030411M15Rik|9430028L06Rik|Fam38b|Fam38b2	piezo-type mechanosensitive ion channel component 2	LOSS	protein-coding
1750_T12,T1,T32	chr18	6.3E+07	6E+07	51999	+	**3**	**37.5**	NA	Intergenic	IMPB_01|Satellite|Satellite	-121317	NM_001039485	667742	Mm.158720	NM_172629	ENSMUSG00000041482	**Piezo2**	5930434P17|9030411M15Rik|9430028L06Rik|Fam38b|Fam38b2	piezo-type mechanosensitive ion channel component 2	LOSS	protein-coding
1107_T1,T2,T32	chr18	3E+07	3E+07	5999	+	**3**	**37.5**	NA	Intergenic	L1MD|LINE|L1	-345396	NM_181414	225326	Mm.194127	NM_181414	ENSMUSG00000033628	**Pik3c3**	5330434F23Rik|Vps34	phosphoinositide-3-kinase, class 3	LOSS	protein-coding
1327_T12,T2,T32	chr18	4.2E+07	4E+07	3999	+	**3**	**37.5**	NA	intron (NM_027072, intron 2 of 3)	RLTR10-int|LTR|ERVK	12209	NM_027072	69401	Mm.34841	NM_027072	ENSMUSG00000059455	**Plac8l1**	1700009N18Rik|1700015M15Rik	PLAC8-like 1	LOSS	protein-coding

1784_T12,T1,T2	chr18	6.6E+07	7E+07	37999	+	**3**	**37.5**	NA	Intergenic	B1_Mus2|SINE|Alu	-63104	NM_021451	58801	Mm.271878	NM_021451	ENSMUSG00000024521	**Pmaip1**	Noxa	phorbol-12-myristate-13-acetate-induced protein 1	LOSS	protein-coding
1786_T12,T1,T3	chr18	6.6E+07	7E+07	999	+	**3**	**37.5**	NA	Intergenic	Intergenic	-40604	NM_021451	58801	Mm.271878	NM_021451	ENSMUSG00000024521	**Pmaip1**	Noxa	phorbol-12-myristate-13-acetate-induced protein 1	LOSS	protein-coding
1297_T22,T2,T32	chr18	4.1E+07	4E+07	999	+	**3**	**37.5**	NA	Intergenic	Intergenic	641194	NM_029942	77619	Mm.30649	NM_029942	ENSMUSG00000056671	**Prelid2**	1700003A01Rik|C330008K14Rik	PRELI domain containing 2	LOSS	protein-coding
1310_T1,T2,T32	chr18	4.1E+07	4E+07	999	+	**3**	**37.5**	NA	Intergenic	Intergenic	479194	NM_029942	77619	Mm.30649	NM_029942	ENSMUSG00000056671	**Prelid2**	1700003A01Rik|C330008K14Rik	PRELI domain containing 2	LOSS	protein-coding
1315_T12,T1,T2	chr18	4.2E+07	4E+07	263999	+	**3**	**37.5**	NA	Intergenic	L1Md_F2|LINE|L1	261694	NM_029942	77619	Mm.30649	NM_029942	ENSMUSG00000056671	**Prelid2**	1700003A01Rik|C330008K14Rik	PRELI domain containing 2	LOSS	protein-coding
583_T22,T3,T4	chr14	5.5E+07	5E+07	1999	+	**3**	**37.5**	NA	Intergenic	RLTR4_MM-int|LTR|ERV1	-14030	NM_001313907	27374	Mm.196585	NM_013768	ENSMUSG00000023110	**Prmt5**	Jbp1|Skb1	protein arginine N-methyltransferase 5	LOSS	protein-coding
1512_T1,T2,T32	chr18	5.1E+07	5E+07	999	+	**3**	**37.5**	NA	Intergenic	Intergenic	-219898	NM_001081224	71373	Mm.63546	NM_001081224	ENSMUSG00000073565	**Prr16**	5430406M13Rik|AI607429	proline rich 16	LOSS	protein-coding
1516_T12,T1,T32	chr18	5.1E+07	5E+07	999	+	**3**	**37.5**	NA	Intergenic	MTE2a|LTR|ERVL-MaLR	-193898	NM_001081224	71373	Mm.63546	NM_001081224	ENSMUSG00000073565	**Prr16**	5430406M13Rik|AI607429	proline rich 16	LOSS	protein-coding
1523_T12,T1,T2	chr18	5.1E+07	5E+07	22999	+	**3**	**37.5**	NA	Intergenic	RMER6C|LTR|ERVK	-27898	NM_001081224	71373	Mm.63546	NM_001081224	ENSMUSG00000073565	**Prr16**	5430406M13Rik|AI607429	proline rich 16	LOSS	protein-coding
1525_T12,T1,T2	chr18	5.1E+07	5E+07	65999	+	**3**	**37.5**	NA	Intergenic	Lx2B|LINE|L1	227602	NM_001081224	71373	Mm.63546	NM_001081224	ENSMUSG00000073565	**Prr16**	5430406M13Rik|AI607429	proline rich 16	LOSS	protein-coding
776_T12,T1,T2	chr18	1.5E+07	1E+07	999	+	**3**	**37.5**	NA	Intergenic	Intergenic	-7151	NM_001163609	73677	Mm.87277	NM_001163609	ENSMUSG00000036743	**Psma8**	2410072D24Rik	proteasome (prosome, macropain) subunit, alpha type, 8	LOSS	protein-coding
778_T12,T1,T2	chr18	1.5E+07	1E+07	999	+	**3**	**37.5**	NA	intron (NM_001163609, intron 1 of 6)	intron (NM_001163609, intron 1 of 6)	1849	NM_001163609	73677	Mm.87277	NM_001163609	ENSMUSG00000036743	**Psma8**	2410072D24Rik	proteasome (prosome, macropain) subunit, alpha type, 8	LOSS	protein-coding
668_T12,T1,T2	chr18	6780501	7E+06	79999	+	**3**	**37.5**	NA	Intergenic	Intergenic	55333	NM_001278447	19330	Mm.132802	NM_181070	ENSMUSG00000073639	**Rab18**	AA959686	RAB18, member RAS oncogene family	LOSS	protein-coding
1847_T12,T1,T32	chr18	7E+07	7E+07	23999	+	**3**	**37.5**	NA	intron (NM_001302799, intron 2 of 5)	intron (NM_001302799, intron 2 of 5)	58088	NM_001302798	80718	Mm.246753	NM_030554	ENSMUSG00000024511	**Rab27b**	2310021G14Rik|B130064M09Rik	RAB27B, member RAS oncogene family	LOSS	protein-coding
1782_T12,T1,T2	chr18	6.6E+07	7E+07	2999	+	**3**	**37.5**	NA	Intergenic	Intergenic	25089	NM_013833	19434	Mm.431047	NM_013833	ENSMUSG00000024518	**Rax**	7530406A22Rik|E130303K03Rik|Rx|ey-1|ey1	retina and anterior neural fold homeobox	LOSS	protein-coding

1135_T12,T1,T32	chr18	3.1E+07	3E+07	999	+	**3**	**37.5**	NA	intron (NM_009065, intron 3 of 4)	L1MA8|LINE|L1	131128	NM_009065	19762	Mm.5163	NM_009065	ENSMUSG00000057455	**Rit2**	RIBA|Rin|Roc2	Ras-like without CAAX 2	LOSS	protein-coding
1127_T12,T1,T2	chr18	3.1E+07	3E+07	26999	+	**3**	**37.5**	NA	intron (NM_009065, intron 4 of 4)	L1Md_F|LINE|L1	329128	NM_009065	19762	Mm.5163	NM_009065	ENSMUSG00000057455	**Rit2**	RIBA|Rin|Roc2	Ras-like without CAAX 2	LOSS	protein-coding
2008_T1,T2,T32	chr18	7.8E+07	8E+07	999	+	**3**	**37.5**	NA	Intergenic	Intergenic	-57864	NM_001164504	225743	Mm.23293	NM_001164504	ENSMUSG00000025427	**Rnf165**	2900024M11Rik|AI427432|Akd2|Ark2c|G630064H08Rik|Gm96	ring finger protein 165	LOSS	protein-coding
2006_T12,T1,T2	chr18	7.7E+07	8E+07	63999	+	**3**	**37.5**	NA	intron (NM_001164504, intron 1 of 7)	intron (NM_001164504, intron 1 of 7)	39636	NM_001164504	225743	Mm.23293	NM_001164504	ENSMUSG00000025427	**Rnf165**	2900024M11Rik|AI427432|Akd2|Ark2c|G630064H08Rik|Gm96	ring finger protein 165	LOSS	protein-coding
517_T12,T2,T42	chr14	5.1E+07	5E+07	153999	+	**3**	**37.5**	NA	Intergenic	Intergenic	4271	NR_002142	85029		NR_002142	ENSMUSG00000092837	**Rpph1**	Gm24821|H1RNA|Rmrp5|Rpr	ribonuclease P RNA component H1	LOSS	ncRNA
980_T12,T1,T22	chr18	2.5E+07	2E+07	1999	+	**3**	**37.5**	NA	Intergenic	Intergenic	-1296	NM_144861	225283	Mm.28427	NM_144861	ENSMUSG00000040446	**Rprd1a**	C77387|mKIAA4077	regulation of nuclear pre-mRNA domain containing 1A	LOSS	protein-coding
1713_T12,T1,T32	chr18	6.1E+07	6E+07	999	+	**3**	**37.5**	NA	Intergenic	B3A|SINE|B2	7404	NM_020600	20044	Mm.43778	NM_020600	ENSMUSG00000024608	**Rps14**	2600014J02Rik|AL023078	ribosomal protein S14	LOSS	protein-coding
2085_T12,T1,T32	chr18	8.1E+07	8E+07	60999	+	**3**	**37.5**	NA	Intergenic	Intergenic	-37422	NM_178280	20689	Mm.215917	NM_178280	ENSMUSG00000024565	**Sall3**	B130022O04Rik|Msal|Msal-1|Sal|Salt|Spalt	sal-like 3 (Drosophila)	LOSS	protein-coding
2087_T12,T1,T32	chr18	8.1E+07	8E+07	241999	+	**3**	**37.5**	NA	Intergenic	Intergenic	-191922	NM_178280	20689	Mm.215917	NM_178280	ENSMUSG00000024565	**Sall3**	B130022O04Rik|Msal|Msal-1|Sal|Salt|Spalt	sal-like 3 (Drosophila)	LOSS	protein-coding
225_T2,T32,T3	chr14	2.2E+07	2E+07	33999	+	**3**	**37.5**	NA	intron (NM_026283, intron 1 of 5)	intron (NM_026283, intron 1 of 5)	13969	NM_026283	67630	Mm.102765	NM_026283	ENSMUSG00000021770	**Samd8**	1110053F04Rik|1700010P07Rik|SMSr	sterile alpha motif domain containing 8	LOSS	protein-coding
2051_T12,T1,T2	chr18	7.9E+07	8E+07	85999	+	**3**	**37.5**	NA	Intergenic	Intergenic	-355109	NM_053099	240427	Mm.312871	NM_053099	ENSMUSG00000024548	**Setbp1**	C130092E12|Seb|mKIAA0437	SET binding protein 1	LOSS	protein-coding
2037_T12,T22,T2	chr18	7.9E+07	8E+07	3999	+	**3**	**37.5**	NA	promoter-TSS (NM_053099)	promoter-TSS (NM_053099)	-109	NM_053099	240427	Mm.312871	NM_053099	ENSMUSG00000024548	**Setbp1**	C130092E12|Seb|mKIAA0437	SET binding protein 1	LOSS	protein-coding
2023_T12,T1,T2	chr18	7.8E+07	8E+07	53999	+	**3**	**37.5**	NA	intron (NM_001195633, intron 43 of 43)	intron (NM_001195633, intron 43 of 43)	26768	NM_001101038	620235	Mm.35607	NM_001101038	ENSMUSG00000091055	**Siglec15**	Cd33l3|EG620235|SIGLEC-I	sialic acid binding Ig-like lectin 15	LOSS	protein-coding
1984_T12,T2,T32	chr18	7.7E+07	8E+07	999	+	**3**	**37.5**	NA	Intergenic	L1Md_T|LINE|L1	-64405	NM_001109743	664805	Mm.329543	NM_001109743	ENSMUSG00000091519	**Skor2**	Corl2|EG664805|Fussel18|Gm7348	SKI family transcriptional corepressor 2	LOSS	protein-coding

1987_T1,T32,T42	chr18	7.7E+07	8E+07	999	+	**3**	**37.5**	NA	Intergenic	Lx6|LINE|L1	-53405	NM_001109743	664805	Mm.329543	NM_001109743	ENSMUSG00000091519	**Skor2**	Corl2|EG664805|Fussel18|Gm7348	SKI family transcriptional corepressor 2	LOSS	protein-coding
2025_T12,T1,T2	chr18	7.8E+07	8E+07	352999	+	**3**	**37.5**	NA	intron (NM_207651, intron 3 of 21)	intron (NM_207651, intron 3 of 21)	-30560	NM_001110274	27411	Mm.44158	NM_030683	ENSMUSG00000024552	**Slc14a2**	UT-A1|UT-A3	solute carrier family 14 (urea transporter), member 2	LOSS	protein-coding
501_T12,T22,T2	chr14	5E+07	5E+07	16999	+	**3**	**37.5**	NA	Intergenic	L1MEf|LINE|L1	-94163	NM_029238	75288	Mm.78738	NM_029238	ENSMUSG00000021852	**Slc35f4**	4930550L21Rik	solute carrier family 35, member F4	LOSS	protein-coding
1980_T12,T1,T2	chr18	7.6E+07	8E+07	204999	+	**3**	**37.5**	NA	Intergenic	Intergenic	213555	NM_001311070	17126	Mm.152699	NM_010754	ENSMUSG00000024563	**Smad2**	7120426M23Rik|Madh2|Madr2|Smad-2|mMad2	SMAD family member 2	LOSS	protein-coding
1951_T12,T1,T32	chr18	7.4E+07	7E+07	999	+	**3**	**37.5**	NA	intron (NM_008540, intron 1 of 11)	intron (NM_008540, intron 1 of 11)	22741	NM_008540	17128	Mm.100399	NM_008540	ENSMUSG00000024515	**Smad4**	AW743858|D18Wsu70e|DPC4|Madh4	SMAD family member 4	LOSS	protein-coding
1953_T12,T1,T2	chr18	7.4E+07	7E+07	2999	+	**3**	**37.5**	NA	intron (NM_008540, intron 1 of 11)	intron (NM_008540, intron 1 of 11)	13741	NM_008540	17128	Mm.100399	NM_008540	ENSMUSG00000024515	**Smad4**	AW743858|D18Wsu70e|DPC4|Madh4	SMAD family member 4	LOSS	protein-coding
278_T22,T2,T3	chr14	3.1E+07	3E+07	6999	+	**3**	**37.5**	NA	promoter-TSS (NM_001308466)	promoter-TSS (NM_001308466)	-70	NM_001308464	66487	Mm.379116	NM_001308091	ENSMUSG00000058351	**Smim4**	2010107H07Rik|Gm41122	small integral membrane protein 4	LOSS	protein-coding
310_T12,T2,T4	chr14	3.4E+07	3E+07	101999	+	**3**	**37.5**	NA	3' UTR (NM_011430, exon 5 of 5)	3' UTR (NM_011430, exon 5 of 5)	4169	NM_011430	20618	Mm.282800	NM_011430	ENSMUSG00000023064	**Sncg**	C79089|persyn	synuclein, gamma	LOSS	protein-coding
2199_T1,T22,T2	chr18	8.8E+07	9E+07	999	+	**3**	**37.5**	NA	Intergenic	Intergenic	1071207	NM_018821	54607	Mm.91920	NM_018821	ENSMUSG00000056153	**Socs6**	1500012M23Rik|5830401B18Rik|AI447482|Cis4|Cish4|HSPC060|SOCS-4|SOCS-6|SSI4|STAI4|STATI4|Socs4	suppressor of cytokine signaling 6	LOSS	protein-coding
2215_T12,T1,T2	chr18	8.8E+07	9E+07	16999	+	**3**	**37.5**	NA	Intergenic	Intergenic	510207	NM_018821	54607	Mm.91920	NM_018821	ENSMUSG00000056153	**Socs6**	1500012M23Rik|5830401B18Rik|AI447482|Cis4|Cish4|HSPC060|SOCS-4|SOCS-6|SSI4|STAI4|STATI4|Socs4	suppressor of cytokine signaling 6	LOSS	protein-coding
2217_T12,T1,T2	chr18	8.8E+07	9E+07	39999	+	**3**	**37.5**	NA	Intergenic	Intergenic	470707	NM_018821	54607	Mm.91920	NM_018821	ENSMUSG00000056153	**Socs6**	1500012M23Rik|5830401B18Rik|AI447482|Cis4|Cish4|HSPC060|SOCS-4|SOCS-6|SSI4|STAI4|STATI4|Socs4	suppressor of cytokine signaling 6	LOSS	protein-coding

2219_T12,T1,T2	chr18	8.8E+07	9E+07	20999	+	**3**	**37.5**	NA	Intergenic	Intergenic	426207	NM_018821	54607	Mm.91920	NM_018821	ENSMUSG00000056153	**Socs6**	1500012M23Rik|5830401B18Rik|AI447482|Cis4|Cish4|HSPC060|SOCS-4|SOCS-6|SSI4|STAI4|STATI4|Socs4	suppressor of cytokine signaling 6	LOSS	protein-coding
2221_T1,T2,T32	chr18	8.8E+07	9E+07	999	+	**3**	**37.5**	NA	Intergenic	Intergenic	405207	NM_018821	54607	Mm.91920	NM_018821	ENSMUSG00000056153	**Socs6**	1500012M23Rik|5830401B18Rik|AI447482|Cis4|Cish4|HSPC060|SOCS-4|SOCS-6|SSI4|STAI4|STATI4|Socs4	suppressor of cytokine signaling 6	LOSS	protein-coding
2223_T12,T1,T2	chr18	8.9E+07	9E+07	4999	+	**3**	**37.5**	NA	Intergenic	L1Md_Gf|LINE|L1	272207	NM_018821	54607	Mm.91920	NM_018821	ENSMUSG00000056153	**Socs6**	1500012M23Rik|5830401B18Rik|AI447482|Cis4|Cish4|HSPC060|SOCS-4|SOCS-6|SSI4|STAI4|STATI4|Socs4	suppressor of cytokine signaling 6	LOSS	protein-coding
773_T12,T1,T2	chr18	1.5E+07	1E+07	18999	+	**3**	**37.5**	NA	Intergenic	L1_Mus2|LINE|L1	126914	NM_001161369	268996	Mm.31761	NM_009280	ENSMUSG00000037013	**Ss18**	D130059H17|Ssxt|Syt	SS18, nBAF chromatin remodeling complex subunit	LOSS	protein-coding
1768_T1,T2,T32	chr18	6.4E+07	6E+07	1999	+	**3**	**37.5**	NA	Intergenic	L1_Rod|LINE|L1	-205859	NM_009182	20451	Mm.252110	NM_009182	ENSMUSG00000056812	**St8sia3**	AI847333|ST8SiaIII|Siat8c	ST8 alpha-N-acetyl-neuraminide alpha-2,8-sialyltransferase 3	LOSS	protein-coding
1770_T12,T1,T2	chr18	6.4E+07	6E+07	272999	+	**3**	**37.5**	NA	intron (NR_045366, intron 2 of 5)	intron (NR_045366, intron 2 of 5)	-62359	NM_009182	20451	Mm.252110	NM_009182	ENSMUSG00000056812	**St8sia3**	AI847333|ST8SiaIII|Siat8c	ST8 alpha-N-acetyl-neuraminide alpha-2,8-sialyltransferase 3	LOSS	protein-coding
2002_T12,T1,T32	chr18	7.7E+07	8E+07	90999	+	**3**	**37.5**	NA	intron (NM_013666, intron 2 of 7)	intron (NM_013666, intron 2 of 7)	33153	NM_013666	225742	Mm.365178	NM_013666	ENSMUSG00000025425	**St8sia5**	ST8SiaV|Siat8e	ST8 alpha-N-acetyl-neuraminide alpha-2,8-sialyltransferase 5	LOSS	protein-coding
1178_T12,T22,T2	chr18	3.3E+07	3E+07	3999	+	**3**	**37.5**	NA	Intergenic	L1Md_T|LINE|L1	-96684	NM_133774	170459	Mm.127058	NM_133774	ENSMUSG00000024378	**Stard4**	4632419C16Rik|9030213J02Rik	StAR-related lipid transfer (START) domain containing 4	LOSS	protein-coding
1854_T12,T1,T2	chr18	7E+07	7E+07	173999	+	**3**	**37.5**	NA	Intergenic	(TG)n|Simple_repeat|Simple_repeat	-49954	NM_029019	170461	Mm.83623	NM_029019	ENSMUSG00000079608	**Stard6**	1700011K09Rik|4833424I06Rik|4933429L05Rik|AI644424	StAR-related lipid transfer (START) domain containing 6	LOSS	protein-coding
1143_T12,T1,T32	chr18	3.1E+07	3E+07	85999	+	**3**	**37.5**	NA	Intergenic	L1Md_A|LINE|L1	-56085	NM_009308	20983	Mm.233846	NM_009308	ENSMUSG00000024261	**Syt4**	SytIV	synaptotagmin IV	LOSS	protein-coding
145_T2,T3,T42	chr14	1.4E+07	1E+07	23999	+	**3**	**37.5**	NA	Intergenic	Intergenic	80813	NM_001285780	66231	Mm.295875	NM_025435	ENSMUSG00000053453	**Thoc7**	Nif3l1bp1	THO complex 7	LOSS	protein-coding
2139_T12,T22,T2	chr18	8.5E+07	9E+07	18999	+	**3**	**37.5**	NA	Intergenic	L1MEc|LINE|L1	-93476	NM_025969	67105	Mm.250617	NM_025969	ENSMUSG00000024645	**Timm21**	1700034H14Rik|2700002I20Rik	tranlocase of inner mitochondrial membrane 21	LOSS	protein-coding

2147_T12,T1,T2	chr18	8.5E+07	9E+07	306999	+	**3**	**37.5**	NA	Intergenic	Lx9|LINE|L1	-584476	NM_025969	67105	Mm.250617	NM_025969	ENSMUSG00000024645	**Timm21**	1700034H14Rik|2700002I20Rik	tranlocase of inner mitochondrial membrane 21	LOSS	protein-coding
753_T12,T1,T3	chr18	1.2E+07	1E+07	999	+	**3**	**37.5**	NA	intron (NM_001289666, intron 4 of 14)	RMER1B|Other|Other	25537	NR_110356	338363	Mm.271567	NM_178801	ENSMUSG00000049411	**Tmem241**	6030446N20Rik	transmembrane protein 241	LOSS	protein-coding
751_T12,T1,T2	chr18	1.2E+07	1E+07	190999	+	**3**	**37.5**	NA	intron (NM_178801, intron 14 of 14)	intron (NM_178801, intron 14 of 14)	134537	NR_110356	338363	Mm.271567	NM_178801	ENSMUSG00000049411	**Tmem241**	6030446N20Rik	transmembrane protein 241	LOSS	protein-coding
2272_T12,T1,T2	chr18	9E+07	9E+07	5999	+	**3**	**37.5**	NA	Intergenic	Intergenic	-347654	NM_198295	67988	Mm.268041	NM_198295	ENSMUSG00000024614	**Tmx3**	6430411B10Rik|A730024F05Rik|AV259382|Txndc10|mKIAA1830	thioredoxin-related transmembrane protein 3	LOSS	protein-coding
2280_T12,T1,T2	chr18	9E+07	9E+07	96999	+	**3**	**37.5**	NA	Intergenic	L1Md_F2|LINE|L1	-184154	NM_198295	67988	Mm.268041	NM_198295	ENSMUSG00000024614	**Tmx3**	6430411B10Rik|A730024F05Rik|AV259382|Txndc10|mKIAA1830	thioredoxin-related transmembrane protein 3	LOSS	protein-coding
2285_T12,T22,T3	chr18	9E+07	9E+07	999	+	**3**	**37.5**	NA	Intergenic	ORR1D1-int|LTR|ERVL-MaLR	-46154	NM_198295	67988	Mm.268041	NM_198295	ENSMUSG00000024614	**Tmx3**	6430411B10Rik|A730024F05Rik|AV259382|Txndc10|mKIAA1830	thioredoxin-related transmembrane protein 3	LOSS	protein-coding
993_T12,T1,T2	chr18	2.5E+07	3E+07	4999	+	**3**	**37.5**	NA	intron (NM_175276, intron 24 of 26)	intron (NM_175276, intron 24 of 26)	43965	NM_001142697	66648	Mm.263189	NM_001004361	ENSMUSG00000024269	**Tpgs2**	5730437P09Rik|5730494M16Rik|AI666318|Pgs2	tubulin polyglutamylase complex subunit 2	LOSS	protein-coding
995_T12,T1,T2	chr18	2.5E+07	3E+07	13999	+	**3**	**37.5**	NA	intron (NM_175276, intron 26 of 26)	intron (NM_175276, intron 26 of 26)	33465	NM_001142697	66648	Mm.263189	NM_001004361	ENSMUSG00000024269	**Tpgs2**	5730437P09Rik|5730494M16Rik|AI666318|Pgs2	tubulin polyglutamylase complex subunit 2	LOSS	protein-coding
1171_T1,T22,T2	chr18	3.3E+07	3E+07	999	+	**3**	**37.5**	NA	intron (NR_045381, intron 2 of 4)	ORR1D2|LTR|ERVL-MaLR	-38383	NM_021367	53603	Mm.143716	NM_021367	ENSMUSG00000024379	**Tslp**	-	thymic stromal lymphopoietin	LOSS	protein-coding
1167_T12,T1,T4	chr18	3.3E+07	3E+07	8999	+	**3**	**37.5**	NA	intron (NR_045381, intron 4 of 4)	intron (NR_045381, intron 4 of 4)	-121383	NM_021367	53603	Mm.143716	NM_021367	ENSMUSG00000024379	**Tslp**	-	thymic stromal lymphopoietin	LOSS	protein-coding
1169_T12,T1,T22	chr18	3.3E+07	3E+07	71999	+	**3**	**37.5**	NA	intron (NR_045381, intron 4 of 4)	Lx7|LINE|L1	-77883	NM_021367	53603	Mm.143716	NM_021367	ENSMUSG00000024379	**Tslp**	-	thymic stromal lymphopoietin	LOSS	protein-coding
403_T12,T2,T42	chr14	4.1E+07	4E+07	202999	+	**3**	**37.5**	NA	intron (NM_145928, intron 1 of 8)	intron (NM_145928, intron 1 of 8)	-4791	NM_001316748	52588	Mm.28876	NM_145928	ENSMUSG00000037824	**Tspan14**	AA958793|D14Ertd226e|Tm4sf14|tspan-14	tetraspanin 14	LOSS	protein-coding

1751_T12,T1,T2	chr18	6.4E+07	6E+07	101999	+	**3**	**37.5**	NA	Intergenic	Intergenic	100859	NM_016792	53382	Mm.19169	NM_016792	ENSMUSG00000024583	**Txnl1**	32kDa|TRP32|Txnl	thioredoxin-like 1	LOSS	protein-coding
173_T22,T2,T3	chr14	1.8E+07	2E+07	999	+	**3**	**37.5**	NA	Intergenic	(TTTC)n|Simple_repeat|Simple_repeat	-81156	NM_009455	22194	Mm.4429	NM_009455	ENSMUSG00000021774	**Ube2e1**	UbcM3|Ubce5|ubcM2	ubiquitin-conjugating enzyme E2E 1	LOSS	protein-coding
180_T1,T22,T4	chr14	1.9E+07	2E+07	11999	+	**3**	**37.5**	NA	Intergenic	B3|SINE|B2	-87873	NM_144839	218793	Mm.23551	NM_144839	ENSMUSG00000058317	**Ube2e2**	BC016265	ubiquitin-conjugating enzyme E2E 2	LOSS	protein-coding
183_T3,T42,T4	chr14	1.9E+07	2E+07	13999	+	**3**	**37.5**	NA	Intergenic	Intergenic	-284873	NM_144839	218793	Mm.23551	NM_144839	ENSMUSG00000058317	**Ube2e2**	BC016265	ubiquitin-conjugating enzyme E2E 2	LOSS	protein-coding
690_T12,T1,T2	chr18	8066501	8E+06	143999	+	**3**	**37.5**	NA	Intergenic	BC1_Mm|scRNA|scRNA	269303	NM_153085	225131	Mm.204524	NM_153085	ENSMUSG00000024283	**Wac**	1110067P07Rik|A230035H12Rik|AI256735|AI256776|Wwp4	WW domain containing adaptor with coiled-coil	LOSS	protein-coding
692_T12,T1,T2	chr18	8215501	8E+06	26999	+	**3**	**37.5**	NA	Intergenic	ORR1E|LTR|ERVL-MaLR	359803	NM_153085	225131	Mm.204524	NM_153085	ENSMUSG00000024283	**Wac**	1110067P07Rik|A230035H12Rik|AI256735|AI256776|Wwp4	WW domain containing adaptor with coiled-coil	LOSS	protein-coding
694_T12,T1,T2	chr18	8278501	8E+06	12999	+	**3**	**37.5**	NA	Intergenic	Lx|LINE|L1	415803	NM_153085	225131	Mm.204524	NM_153085	ENSMUSG00000024283	**Wac**	1110067P07Rik|A230035H12Rik|AI256735|AI256776|Wwp4	WW domain containing adaptor with coiled-coil	LOSS	protein-coding
696_T12,T1,T2	chr18	8294501	9E+06	287999	+	**3**	**37.5**	NA	Intergenic	L1Md_F2|LINE|L1	569303	NM_153085	225131	Mm.204524	NM_153085	ENSMUSG00000024283	**Wac**	1110067P07Rik|A230035H12Rik|AI256735|AI256776|Wwp4	WW domain containing adaptor with coiled-coil	LOSS	protein-coding
327_T12,T32,T4	chr14	3.5E+07	3E+07	1999	+	**3**	**37.5**	NA	intron (NM_001004436, intron 16 of 18)	L1M4|LINE|L1	66572	NM_001301330	218914	Mm.27957	NM_001004436	ENSMUSG00000041408	**Wapl**	A530089A20Rik|BC037674|DIF-2|FOE|Wapal	WAPL cohesin release factor	LOSS	protein-coding
1372_T12,T2,T32	chr18	4.5E+07	4E+07	999	+	**3**	**37.5**	NA	Intergenic	B1F|SINE|Alu	-3665	NM_001163013	240255	Mm.244482	NM_001163013	ENSMUSG00000034653	**Ythdc2**	3010002F02Rik|BC037178	YTH domain containing 2	LOSS	protein-coding
2133_T12,T2,T32	chr18	8.4E+07	8E+07	2999	+	**3**	**37.5**	NA	Intergenic	(CA)n|Simple_repeat|Simple_repeat	70842	NM_146090	225791	Mm.147052	NM_146090	ENSMUSG00000049090	**Zadh2**	C530046K17Rik|PTGR-3|Pthr3	zinc binding alcohol dehydrogenase, domain containing 2	LOSS	protein-coding
1671_T12,T3	chr18	5.9E+07	6E+07	3999	+	**2**	**25**	NA	Intergenic	L1Md_T|LINE|L1	-29264	NM_175506	240322	Mm.71963	NM_175506	ENSMUSG00000053441	**Adamts19**	4831407I23Rik|AU015154|D230034E10Rik	a disintegrin-like and metallopeptidase (reprolysin type) with thrombospondin type 1 motif, 19	LOSS	protein-coding
667_T12,T2	chr17	9.3E+07	9E+07	3999	+	**2**	**25**	NA	Intergenic	L1Md_A|LINE|L1	-253517	NM_001315503	11516	Mm.3407	NM_009625	ENSMUSG00000024256	**Adcyap1**	PACAP	adenylate cyclase activating polypeptide 1	LOSS	protein-coding
1734_T12,T1	chr18	6.2E+07	6E+07	1999	+	**2**	**25**	NA	Intergenic	L1_Mus3|LINE|L1	-62519	NM_007420	11555	Mm.5598	NM_007420	ENSMUSG00000045730	**Adrb2**	Adrb-2|Badm|Gpcr7	adrenergic receptor, beta 2	LOSS	protein-coding

2454_T2,T4	chr8	5.2E+07	5E+07	3999	+	**2**	**25**	NA	Intergenic	L1Md_T|LINE|L1	-1E+06	NM_001205054	11593	Mm.334535	NM_001005847	ENSMUSG00000031521	**Aga**	AW060726	aspartylglucosaminidase	LOSS	protein-coding
1216_T1,T2	chr18	3.7E+07	4E+07	999	+	**2**	**25**	NA	3' UTR (NM_001271547, exon 20 of 20)	3' UTR (NM_001271547, exon 20 of 20)	-4603	NM_175375	108857	Mm.482287	NM_175375	ENSMUSG00000024483	**Ankhd1**	1110004O12Rik|4933432B13Rik|9130019P20Rik|A530027J04Rik|A630021B20Rik|AA571404|Mask|mFLJ00246|mKIAA1085	ankyrin repeat and KH domain containing 1	LOSS	protein-coding
23_T12,T3	chr10	9E+07	9E+07	4999	+	**2**	**25**	NA	intron (NM_001128086, intron 1 of 24)	L1Md_T|LINE|L1	80491	NM_001128086	77531	Mm.440605	NM_181398	ENSMUSG00000058589	**Anks1b**	AIDA-1b|C030032C09Rik|E530015N03Rik|ENSMUSG00000078431|Gm10937|Gm1555	ankyrin repeat and sterile alpha motif domain containing 1B	LOSS	protein-coding
240_T3,T4	chr14	2.6E+07	3E+07	36999	+	**2**	**25**	NA	Intergenic	(GAAA)n|Simple_repeat|Simple_repeat	-28155	NM_013469	11744	Mm.294083	NM_013469	ENSMUSG00000021866	**Anxa11**	A830099O17Rik|Anx11	annexin A11	LOSS	protein-coding
242_T22,T3	chr14	2.6E+07	3E+07	999	+	**2**	**25**	NA	Intergenic	(TTTA)n|Simple_repeat|Simple_repeat	-1155	NM_013469	11744	Mm.294083	NM_013469	ENSMUSG00000021866	**Anxa11**	A830099O17Rik|Anx11	annexin A11	LOSS	protein-coding
253_T2,T32	chr14	2.7E+07	3E+07	133999	+	**2**	**25**	NA	intron (NM_001289686, intron 2 of 12)	intron (NM_001289686, intron 2 of 12)	45507	NM_001289686	71704	Mm.248606	NM_027871	ENSMUSG00000021895	**Arhgef3**	1200004I24Rik|9830169H03Rik|C76747|Xpln	Rho guanine nucleotide exchange factor (GEF) 3	LOSS	protein-coding
1717_T2,T32	chr18	6.2E+07	6E+07	999	+	**2**	**25**	NA	intron (NM_177828, intron 4 of 12)	(GA)n|Simple_repeat|Simple_repeat	24536	NM_177828	328967	Mm.488098	NM_177828	ENSMUSG00000045094	**Arhgef37**	4933429F08Rik	Rho guanine nucleotide exchange factor (GEF) 37	LOSS	protein-coding
966_T12,T2	chr18	2.3E+07	2E+07	7999	+	**2**	**25**	NA	Intergenic	L1Md_A|LINE|L1	215411	NM_001167777	211961	Mm.392310	NM_001167777	ENSMUSG00000045215	**Asxl3**	C230079D11Rik|D430002O22Rik|D930044O18Rik|Gm329|Gm945	additional sex combs like 3 (Drosophila)	LOSS	protein-coding
961_T12,T1	chr18	2.2E+07	2E+07	999	+	**2**	**25**	NA	intron (NM_001167777, intron 2 of 12)	intron (NM_001167777, intron 2 of 12)	37911	NM_001167777	211961	Mm.392310	NM_001167777	ENSMUSG00000045215	**Asxl3**	C230079D11Rik|D430002O22Rik|D930044O18Rik|Gm329|Gm945	additional sex combs like 3 (Drosophila)	LOSS	protein-coding
962_T1,T2	chr18	2.2E+07	2E+07	6999	+	**2**	**25**	NA	intron (NM_001167777, intron 3 of 12)	intron (NM_001167777, intron 3 of 12)	57911	NM_001167777	211961	Mm.392310	NM_001167777	ENSMUSG00000045215	**Asxl3**	C230079D11Rik|D430002O22Rik|D930044O18Rik|Gm329|Gm945	additional sex combs like 3 (Drosophila)	LOSS	protein-coding

2073_T12,T2	chr18	8.1E+07	8E+07	999	+	**2**	**25**	NA	intron (NM_001201569, intron 11 of 29)	intron (NM_001201569, intron 11 of 29)	106058	NM_001201569	50771	Mm.247138	NM_015805	ENSMUSG00000024566	**Atp9b**	AA934181|Atpc2b|IIb|MMR	ATPase, class II, type 9B	LOSS	protein-coding
2075_T12,T2	chr18	8.1E+07	8E+07	999	+	**2**	**25**	NA	intron (NM_001201569, intron 5 of 29)	intron (NM_001201569, intron 5 of 29)	54058	NM_001201569	50771	Mm.247138	NM_015805	ENSMUSG00000024566	**Atp9b**	AA934181|Atpc2b|IIb|MMR	ATPase, class II, type 9B	LOSS	protein-coding
156_T2,T4	chr14	1.4E+07	1E+07	11999	+	**2**	**25**	NA	intron (NM_139227, intron 3 of 11)	(TA)n|Simple_repeat|Simple_repeat	10009	NM_139227	246103	Mm.133625	NM_139227	ENSMUSG00000021738	**Atxn7**	A430107N12Rik|AI627028|Sca7|ataxin-7	ataxin 7	LOSS	protein-coding
616_T1,T42	chr16	2.4E+07	2E+07	3999	+	**2**	**25**	NA	intron (NM_009744, intron 1 of 9)	CpG	1112	NM_009744	12053	Mm.347398	NM_009744	ENSMUSG00000022508	**Bcl6**	Bcl5	B cell leukemia/lymphoma 6	LOSS	protein-coding
761_T12,T1	chr18	1.2E+07	1E+07	812999	+	**2**	**25**	NA	intron (NR_045524, intron 5 of 11)	intron (NR_045524, intron 5 of 11)	24645	NM_027687	71132	Mm.179740	NM_027687	ENSMUSG00000024430	**Cabyr**	1700016C01Rik|4933421A18Rik|CBP86|FSP-2	calcium-binding tyrosine-(Y)-phosphorylation regulated (fibrousheathin 2)	LOSS	protein-coding
1174_T2,T32	chr18	3.3E+07	3E+07	6999	+	**2**	**25**	NA	intron (NM_009793, intron 1 of 10)	URR1B|DNA|hAT-Charlie	101959	NM_009793	12326	Mm.222329	NM_009793	ENSMUSG00000038128	**Camk4**	A430110E23Rik|AI666733|CaMKIV|CaMKIV/Gr|D18Bwg0362e	calcium/calmodulin-dependent protein kinase IV	LOSS	protein-coding
1175_T12,T2	chr18	3.3E+07	3E+07	999	+	**2**	**25**	NA	intron (NM_009793, intron 1 of 10)	L1Md_T|LINE|L1	110959	NM_009793	12326	Mm.222329	NM_009793	ENSMUSG00000038128	**Camk4**	A430110E23Rik|AI666733|CaMKIV|CaMKIV/Gr|D18Bwg0362e	calcium/calmodulin-dependent protein kinase IV	LOSS	protein-coding
281_T22,T3	chr14	3.1E+07	3E+07	4999	+	**2**	**25**	NA	promoter-TSS (NM_009796)	promoter-TSS (NM_009796)	-724	NM_009796	12339	Mm.201535	NM_009796	ENSMUSG00000021893	**Capn7**	AU022319|PalBH	calpain 7	LOSS	protein-coding
2169_T12,T2	chr18	8.7E+07	9E+07	3999	+	**2**	**25**	NA	Intergenic	L1Md_F2|LINE|L1	-126610	NM_001302356	12405	Mm.70775	NM_172633	ENSMUSG00000024647	**Cbln2**	6330593N19Rik|A730004O05	cerebellin 2 precursor protein	LOSS	protein-coding
2176_T12,T2	chr18	8.7E+07	9E+07	999	+	**2**	**25**	NA	Intergenic	Lx8|LINE|L1	104935	NM_172633	12405	Mm.70775	NM_172633	ENSMUSG00000024647	**Cbln2**	6330593N19Rik|A730004O05	cerebellin 2 precursor protein	LOSS	protein-coding
2178_T12,T2	chr18	8.7E+07	9E+07	999	+	**2**	**25**	NA	Intergenic	Intergenic	202935	NM_172633	12405	Mm.70775	NM_172633	ENSMUSG00000024647	**Cbln2**	6330593N19Rik|A730004O05	cerebellin 2 precursor protein	LOSS	protein-coding
2182_T12,T1	chr18	8.7E+07	9E+07	999	+	**2**	**25**	NA	Intergenic	Intergenic	258935	NM_172633	12405	Mm.70775	NM_172633	ENSMUSG00000024647	**Cbln2**	6330593N19Rik|A730004O05	cerebellin 2 precursor protein	LOSS	protein-coding
2185_T1,T32	chr18	8.7E+07	9E+07	5999	+	**2**	**25**	NA	Intergenic	L1Md_A|LINE|L1	445435	NM_172633	12405	Mm.70775	NM_172633	ENSMUSG00000024647	**Cbln2**	6330593N19Rik|A730004O05	cerebellin 2 precursor protein	LOSS	protein-coding

2188_T1,T32	chr18	8.7E+07	9E+07	4999	+	**2**	**25**	NA	Intergenic	Intergenic	478935	NM_172633	12405	Mm.70775	NM_172633	ENSMUSG00000024647	**Cbln2**	6330593N19Rik|A730004O05	cerebellin 2 precursor protein	LOSS	protein-coding
2191_T32,T3	chr18	8.7E+07	9E+07	999	+	**2**	**25**	NA	Intergenic	L1Md_A|LINE|L1	638935	NM_172633	12405	Mm.70775	NM_172633	ENSMUSG00000024647	**Cbln2**	6330593N19Rik|A730004O05	cerebellin 2 precursor protein	LOSS	protein-coding
2194_T12,T2	chr18	8.8E+07	9E+07	1999	+	**2**	**25**	NA	Intergenic	Lx|LINE|L1	918435	NM_172633	12405	Mm.70775	NM_172633	ENSMUSG00000024647	**Cbln2**	6330593N19Rik|A730004O05	cerebellin 2 precursor protein	LOSS	protein-coding
708_T1,T2	chr18	9514501	1E+07	14999	+	**2**	**25**	NA	Intergenic	MuLV-int|LTR|ERV1	-71850	NM_026484	67974	Mm.86523	NM_026484	ENSMUSG00000024286	**Ccny**	1700025H17Rik|3110050L10Rik|4631402G10Rik|5730405I09Rik	cyclin Y	LOSS	protein-coding
353_T12,T2	chr14	3.7E+07	4E+07	20999	+	**2**	**25**	NA	intron (NM_028407, intron 7 of 7)	intron (NM_028407, intron 7 of 7)	48568	NM_028407	72972	Mm.27621	NM_027045	ENSMUSG00000058690	**Ccser2**	1700012P13Rik|2900054P12Rik|AI035535|AW108503|Fam190b|Gcap14	coiled-coil serine rich 2	LOSS	protein-coding
2230_T12,T1	chr18	8.9E+07	9E+07	72999	+	**2**	**25**	NA	Intergenic	Intergenic	-17427	NM_178687	225825	Mm.139293	NM_178687	ENSMUSG00000034028	**Cd226**	BC051526|DNAM-1|DNAM1|Pta1|TLiSA1	CD226 antigen	LOSS	protein-coding
2233_T12,T1	chr18	8.9E+07	9E+07	5999	+	**2**	**25**	NA	intron (NM_001039173, intron 7 of 7)	(TATATG)n|Simple_repeat|Simple_repeat	123073	NM_001039149	225825	Mm.139293	NM_178687	ENSMUSG00000034028	**Cd226**	BC051526|DNAM-1|DNAM1|Pta1|TLiSA1	CD226 antigen	LOSS	protein-coding
2232_T12,T1	chr18	8.9E+07	9E+07	89999	+	**2**	**25**	NA	intron (NM_178687, intron 5 of 6)	MTEa|LTR|ERVL-MaLR	70073	NM_001039149	225825	Mm.139293	NM_178687	ENSMUSG00000034028	**Cd226**	BC051526|DNAM-1|DNAM1|Pta1|TLiSA1	CD226 antigen	LOSS	protein-coding
483_T12,T22	chr14	4.7E+07	5E+07	1999	+	**2**	**25**	NA	Intergenic	L1Md_T|LINE|L1	-9041	NM_028222	72391	Mm.272394	NM_028222	ENSMUSG00000037628	**Cdkn3**	2410006H10Rik|KAP	cyclin-dependent kinase inhibitor 3	LOSS	protein-coding
1016_T1,T2	chr18	2.6E+07	3E+07	999	+	**2**	**25**	NA	Intergenic	L1M5|LINE|L1	-389017	NM_001174074	108013	Mm.266435	NM_133195	ENSMUSG00000024268	**Celf4**	A230070D14Rik|BRUNOL-4|Brul4|Brunol4|C130060B05Rik	CUGBP, Elav-like family member 4	LOSS	protein-coding
1017_T12,T1	chr18	2.6E+07	3E+07	184999	+	**2**	**25**	NA	Intergenic	Lx2|LINE|L1	-496017	NM_001174074	108013	Mm.266435	NM_133195	ENSMUSG00000024268	**Celf4**	A230070D14Rik|BRUNOL-4|Brul4|Brunol4|C130060B05Rik	CUGBP, Elav-like family member 4	LOSS	protein-coding
1019_T1,T2	chr18	2.6E+07	3E+07	999	+	**2**	**25**	NA	Intergenic	RMER15|LTR|ERVL	-711017	NM_001174074	108013	Mm.266435	NM_133195	ENSMUSG00000024268	**Celf4**	A230070D14Rik|BRUNOL-4|Brul4|Brunol4|C130060B05Rik	CUGBP, Elav-like family member 4	LOSS	protein-coding
1021_T1,T2	chr18	2.6E+07	3E+07	999	+	**2**	**25**	NA	Intergenic	RMER1A|Other|Other	-742017	NM_001174074	108013	Mm.266435	NM_133195	ENSMUSG00000024268	**Celf4**	A230070D14Rik|BRUNOL-4|Brul4|Brunol4|C130060B05Rik	CUGBP, Elav-like family member 4	LOSS	protein-coding
1023_T12,T1	chr18	2.7E+07	3E+07	225999	+	**2**	**25**	NA	Intergenic	Intergenic	-962517	NM_001174074	108013	Mm.266435	NM_133195	ENSMUSG00000024268	**Celf4**	A230070D14Rik|BRUNOL-4|Brul4|Brunol4|C130060B05Rik	CUGBP, Elav-like family member 4	LOSS	protein-coding

1026_T12,T1	chr18	2.7E+07	3E+07	2999	+	**2**	**25**	NA	Intergenic	Intergenic	-1E+06	NM_001174074	108013	Mm.266435	NM_133195	ENSMUSG00000024268	**Celf4**	A230070D14Rik|BRUNOL-4|Brul4|Brunol4|C130060B05Rik	CUGBP, Elav-like family member 4	LOSS	protein-coding
1031_T12,T1	chr18	2.7E+07	3E+07	5999	+	**2**	**25**	NA	Intergenic	L1Md_T|LINE|L1	-1E+06	NM_001174074	108013	Mm.266435	NM_133195	ENSMUSG00000024268	**Celf4**	A230070D14Rik|BRUNOL-4|Brul4|Brunol4|C130060B05Rik	CUGBP, Elav-like family member 4	LOSS	protein-coding
1813_T32,T42	chr18	6.8E+07	7E+07	999	+	**2**	**25**	NA	intron (NM_027556, intron 16 of 45)	ID_B1|SINE|B4	33893	NM_027556	70799	Mm.30857	NM_027556	ENSMUSG00000024542	**Cep192**	4631422C13Rik|D430014P18Rik	centrosomal protein 192	LOSS	protein-coding
1957_T1,T2	chr18	7.4E+07	7E+07	3999	+	**2**	**25**	NA	intron (NR_029424, intron 7 of 8)	L1_Rod|LINE|L1	44400	NM_028948	74453	Mm.182293	NM_028948	ENSMUSG00000035394	**Cfap53**	4933415I03Rik|Ccdc11	cilia and flagella associated protein 53	LOSS	protein-coding
808_T12,T32	chr18	1.6E+07	2E+07	7999	+	**2**	**25**	NA	Intergenic	Intergenic	-218454	NM_199055	71367	Mm.329304	NM_199055	ENSMUSG00000047161	**Chst9**	5430438D01Rik	carbohydrate (N-acetylgalactosamine 4-0) sulfotransferase 9	LOSS	protein-coding
810_T12,T1	chr18	1.6E+07	2E+07	26999	+	**2**	**25**	NA	Intergenic	L1Md_F2|LINE|L1	-245954	NM_199055	71367	Mm.329304	NM_199055	ENSMUSG00000047161	**Chst9**	5430438D01Rik	carbohydrate (N-acetylgalactosamine 4-0) sulfotransferase 9	LOSS	protein-coding
801_T12,T1	chr18	1.6E+07	2E+07	3999	+	**2**	**25**	NA	intron (NM_199055, intron 1 of 3)	MIRb|SINE|MIR	13546	NM_199055	71367	Mm.329304	NM_199055	ENSMUSG00000047161	**Chst9**	5430438D01Rik	carbohydrate (N-acetylgalactosamine 4-0) sulfotransferase 9	LOSS	protein-coding
712_T1,T2	chr18	9732501	1E+07	2999	+	**2**	**25**	NA	intron (NM_130449, intron 2 of 9)	intron (NM_130449, intron 2 of 9)	26352	NM_130449	140792	Mm.218571	NM_130449	ENSMUSG00000036103	**Colec12**	CL-P1|SRCL|Scara4	collectin sub-family member 12	LOSS	protein-coding
714_T1,T2	chr18	9772501	1E+07	999	+	**2**	**25**	NA	intron (NM_130449, intron 2 of 9)	L1_Mus4|LINE|L1	65352	NM_130449	140792	Mm.218571	NM_130449	ENSMUSG00000036103	**Colec12**	CL-P1|SRCL|Scara4	collectin sub-family member 12	LOSS	protein-coding
228_T2,T3	chr14	2.2E+07	2E+07	69999	+	**2**	**25**	NA	intron (NM_011695, intron 8 of 9)	(CAGC)n|Simple_repeat|Simple_repeat	5410	NM_026965	69156	Mm.11827	NM_026965	ENSMUSG00000021773	**Comtd1**	1810030M08Rik|MT773	catechol-O-methyltransferase domain containing 1	LOSS	protein-coding
100_T2,T3	chr13	8.6E+07	9E+07	7999	+	**2**	**25**	NA	Intergenic	L1Md_F|LINE|L1	-149705	NM_007749	12867	Mm.378898	NM_007749	ENSMUSG00000017778	**Cox7c**	COXVIIc|Cox7c1	cytochrome c oxidase subunit VIIc	LOSS	protein-coding
2431_T1,T32	chr7	8.8E+07	9E+07	3999	+	**2**	**25**	NA	Intergenic	Intergenic	-10585	NM_009982	13032	Mm.89295	NM_009982	ENSMUSG00000030560	**Ctsc**	AI047818|CatC|DPP1|DPPI	cathepsin C	LOSS	protein-coding
1925_T12,T1	chr18	7.3E+07	7E+07	999	+	**2**	**25**	NA	Intergenic	RLTR50B|LTR|ERVK	-276931	NM_007831	13176	Mm.167882	NM_007831	ENSMUSG00000060534	**Dcc**	C030036D22Rik|Igdcc1	deleted in colorectal carcinoma	LOSS	protein-coding

1927_T1,T2	chr18	7.3E+07	7E+07	8999	+	**2**	**25**	NA	Intergenic	Lx7|LINE|L1	-304931	NM_007831	13176	Mm.167882	NM_007831	ENSMUSG00000060534	**Dcc**	C030036D22Rik|Igdcc1	deleted in colorectal carcinoma	LOSS	protein-coding
1933_T12,T1	chr18	7.3E+07	7E+07	128999	+	**2**	**25**	NA	Intergenic	Intergenic	-517931	NM_007831	13176	Mm.167882	NM_007831	ENSMUSG00000060534	**Dcc**	C030036D22Rik|Igdcc1	deleted in colorectal carcinoma	LOSS	protein-coding
1934_T12,T2	chr18	7.3E+07	7E+07	7999	+	**2**	**25**	NA	Intergenic	Intergenic	-593431	NM_007831	13176	Mm.167882	NM_007831	ENSMUSG00000060534	**Dcc**	C030036D22Rik|Igdcc1	deleted in colorectal carcinoma	LOSS	protein-coding
1914_T12,T2	chr18	7.2E+07	7E+07	6999	+	**2**	**25**	NA	intron (NM_007831, intron 1 of 28)	intron (NM_007831, intron 1 of 28)	75069	NM_007831	13176	Mm.167882	NM_007831	ENSMUSG00000060534	**Dcc**	C030036D22Rik|Igdcc1	deleted in colorectal carcinoma	LOSS	protein-coding
1916_T12,T2	chr18	7.2E+07	7E+07	11999	+	**2**	**25**	NA	intron (NM_007831, intron 1 of 28)	intron (NM_007831, intron 1 of 28)	43569	NM_007831	13176	Mm.167882	NM_007831	ENSMUSG00000060534	**Dcc**	C030036D22Rik|Igdcc1	deleted in colorectal carcinoma	LOSS	protein-coding
1890_T2,T32	chr18	7.2E+07	7E+07	1999	+	**2**	**25**	NA	intron (NM_007831, intron 12 of 28)	Lx8|LINE|L1	811569	NM_007831	13176	Mm.167882	NM_007831	ENSMUSG00000060534	**Dcc**	C030036D22Rik|Igdcc1	deleted in colorectal carcinoma	LOSS	protein-coding
1909_T12,T2	chr18	7.2E+07	7E+07	999	+	**2**	**25**	NA	intron (NM_007831, intron 5 of 28)	CT-rich|Low_complexity|Low_complexity	551069	NM_007831	13176	Mm.167882	NM_007831	ENSMUSG00000060534	**Dcc**	C030036D22Rik|Igdcc1	deleted in colorectal carcinoma	LOSS	protein-coding
1893_T1,T2	chr18	7.2E+07	7E+07	11999	+	**2**	**25**	NA	intron (NM_007831, intron 7 of 28)	intron (NM_007831, intron 7 of 28)	742569	NM_007831	13176	Mm.167882	NM_007831	ENSMUSG00000060534	**Dcc**	C030036D22Rik|Igdcc1	deleted in colorectal carcinoma	LOSS	protein-coding
1895_T1,T3	chr18	7.2E+07	7E+07	999	+	**2**	**25**	NA	intron (NM_007831, intron 7 of 28)	IAPEz-int|LTR|ERVK	713069	NM_007831	13176	Mm.167882	NM_007831	ENSMUSG00000060534	**Dcc**	C030036D22Rik|Igdcc1	deleted in colorectal carcinoma	LOSS	protein-coding
472_T12,T4	chr14	4.6E+07	5E+07	3999	+	**2**	**25**	NA	Intergenic	MERVL_2A-int|LTR|ERVL	-71357	NM_001042719	114874	Mm.121918	NM_176845	ENSMUSG00000037697	**Ddhd1**	Mir5131|PA-PLA1	DDHD domain containing 1	LOSS	protein-coding
2253_T1,T2	chr18	9E+07	9E+07	4999	+	**2**	**25**	NA	Intergenic	ORR1D1|LTR|ERVL-MaLR	-103864	NM_001039173	623279	Mm.447164	NM_001039173	ENSMUSG00000073514	**Dok6**	Dok-6	docking protein 6	LOSS	protein-coding
2257_T32,T3	chr18	9E+07	9E+07	999	+	**2**	**25**	NA	Intergenic	MERVL-int|LTR|ERVL	-219864	NM_001039173	623279	Mm.447164	NM_001039173	ENSMUSG00000073514	**Dok6**	Dok-6	docking protein 6	LOSS	protein-coding
2267_T12,T2	chr18	9E+07	9E+07	15999	+	**2**	**25**	NA	Intergenic	L1Md_F2|LINE|L1	-298364	NM_001039173	623279	Mm.447164	NM_001039173	ENSMUSG00000073514	**Dok6**	Dok-6	docking protein 6	LOSS	protein-coding

2270_T12,T2	chr18	9E+07	9E+07	5999	+	**2**	**25**	NA	Intergenic	IAPEz-int|LTR|ERVK	-346364	NM_001039173	623279	Mm.447164	NM_001039173	ENSMUSG00000073514	**Dok6**	Dok-6	docking protein 6	LOSS	protein-coding
2240_T12,T2	chr18	9E+07	9E+07	4999	+	**2**	**25**	NA	intron (NM_001039173, intron 1 of 7)	intron (NM_001039173, intron 1 of 7)	132136	NM_001039173	623279	Mm.447164	NM_001039173	ENSMUSG00000073514	**Dok6**	Dok-6	docking protein 6	LOSS	protein-coding
2237_T12,T1	chr18	9E+07	9E+07	999	+	**2**	**25**	NA	intron (NM_001039173, intron 3 of 7)	L1VL4|LINE|L1	253136	NM_001039173	623279	Mm.447164	NM_001039173	ENSMUSG00000073514	**Dok6**	Dok-6	docking protein 6	LOSS	protein-coding
2238_T12,T1	chr18	9E+07	9E+07	999	+	**2**	**25**	NA	intron (NM_001039173, intron 3 of 7)	Lx|LINE|L1	244136	NM_001039173	623279	Mm.447164	NM_001039173	ENSMUSG00000073514	**Dok6**	Dok-6	docking protein 6	LOSS	protein-coding
619_T12,T42	chr16	4.8E+07	5E+07	3999	+	**2**	**25**	NA	promoter-TSS (NM_028610)	promoter-TSS (NM_028610)	-235	NM_028610	73693	Mm.35597	NM_028610	ENSMUSG00000058550	**Dppa4**	2410091M23Rik|C76608|ECAT15-1	developmental pluripotency associated 4	LOSS	protein-coding
884_T1,T22	chr18	1.9E+07	2E+07	999	+	**2**	**25**	NA	Intergenic	Intergenic	832097	NM_007882	13507	Mm.89935	NM_007882	ENSMUSG00000059898	**Dsc3**	5430426I24Rik	desmocollin 3	LOSS	protein-coding
886_T1,T22	chr18	1.9E+07	2E+07	10999	+	**2**	**25**	NA	Intergenic	Lx4A|LINE|L1	796097	NM_007882	13507	Mm.89935	NM_007882	ENSMUSG00000059898	**Dsc3**	5430426I24Rik	desmocollin 3	LOSS	protein-coding
901_T12,T2	chr18	1.9E+07	2E+07	4999	+	**2**	**25**	NA	Intergenic	L1Md_F2|LINE|L1	557097	NM_007882	13507	Mm.89935	NM_007882	ENSMUSG00000059898	**Dsc3**	5430426I24Rik	desmocollin 3	LOSS	protein-coding
905_T12,T1	chr18	2E+07	2E+07	22999	+	**2**	**25**	NA	Intergenic	L1Md_T|LINE|L1	443097	NM_007882	13507	Mm.89935	NM_007882	ENSMUSG00000059898	**Dsc3**	5430426I24Rik	desmocollin 3	LOSS	protein-coding
907_T12,T32	chr18	2E+07	2E+07	999	+	**2**	**25**	NA	Intergenic	Intergenic	422097	NM_007882	13507	Mm.89935	NM_007882	ENSMUSG00000059898	**Dsc3**	5430426I24Rik	desmocollin 3	LOSS	protein-coding
912_T12,T1	chr18	2E+07	2E+07	2999	+	**2**	**25**	NA	Intergenic	Intergenic	381097	NM_007882	13507	Mm.89935	NM_007882	ENSMUSG00000059898	**Dsc3**	5430426I24Rik	desmocollin 3	LOSS	protein-coding
914_T1,T2	chr18	2E+07	2E+07	4999	+	**2**	**25**	NA	Intergenic	L1Md_A|LINE|L1	186097	NM_007882	13507	Mm.89935	NM_007882	ENSMUSG00000059898	**Dsc3**	5430426I24Rik	desmocollin 3	LOSS	protein-coding
1489_T2,T32	chr18	5E+07	5E+07	23999	+	**2**	**25**	NA	Intergenic	MLT1H1|LTR|ERVL-MaLR	74101	NM_026854	68857	Mm.96010	NM_026854	ENSMUSG00000024505	**Dtwd2**	1190002H09Rik|8030470C17Rik|AI414673|BB115449	DTW domain containing 2	LOSS	protein-coding
1490_T12,T32	chr18	5E+07	5E+07	999	+	**2**	**25**	NA	Intergenic	IAPEz-int|LTR|ERVK	61601	NM_026854	68857	Mm.96010	NM_026854	ENSMUSG00000024505	**Dtwd2**	1190002H09Rik|8030470C17Rik|AI414673|BB115449	DTW domain containing 2	LOSS	protein-coding
2021_T12,T1	chr18	7.8E+07	8E+07	999	+	**2**	**25**	NA	intron (NM_001195633, intron 25 of 43)	RLTR21|LTR|ERVK	54533	NM_001195633	1E+08	Mm.297782	NM_001195633	ENSMUSG00000039840	**Epg5**	4732475F16|5430411K18Rik|AI661957|AW456499|mKIAA1632	ectopic P-granules autophagy protein 5 homolog (C. elegans)	LOSS	protein-coding

608_T2,T3	chr15	7.1E+07	7E+07	3999	+	**2**	**25**	NA	Intergenic	L1Md_A|LINE|L1	830338	NM_177819	70363	Mm.126450	NM_177819	ENSMUSG00000036800	**Fam135b**	1700010C24Rik|A830008O07Rik	family with sequence similarity 135, member B	LOSS	protein-coding
1493_T2,T3	chr18	5E+07	5E+07	9999	+	**2**	**25**	NA	Intergenic	L1Md_T|LINE|L1	-60869	NM_001004061	225497	Mm.329435	NM_001004061	ENSMUSG00000035420	**Fam170a**	Gm93|Znfd	family with sequence similarity 170, member A	LOSS	protein-coding
28_T22,T3	chr11	2.6E+07	3E+07	3999	+	**2**	**25**	NA	intron (NM_001277273, intron 8 of 13)	L1Md_T|LINE|L1	79416	NR_102382	67030	Mm.18875	NM_025923	ENSMUSG00000004018	**Fancl**	2010322C19Rik|AW554273|B230118H11Rik|Phf9|Pog|gcd	Fanconi anemia, complementation group L	LOSS	protein-coding
1664_T12,T1	chr18	5.8E+07	6E+07	22999	+	**2**	**25**	NA	Intergenic	AT_rich|Low_complexity|Low_complexity	-144074	NM_010181	14119	Mm.20271	NM_010181	ENSMUSG00000024598	**Fbn2**	BC063774|Fib-2|mKIAA4226|sne|sy	fibrillin 2	LOSS	protein-coding
2327_T12,T3	chr3	8.7E+07	9E+07	8999	+	**2**	**25**	NA	intron (NM_001113238, intron 11 of 11)	ETnERV2-int|LTR|ERVK	33218	NM_001113238	329693	Mm.331122	NM_183222	ENSMUSG00000048031	**Fcrl5**	Fcrh3|mBXMH2	Fc receptor-like 5	LOSS	protein-coding
136_T2,T4	chr14	1E+07	1E+07	21999	+	**2**	**25**	NA	intron (NM_001308286, intron 2 of 6)	intron (NM_001308286, intron 2 of 6)	167958	NM_001308286	14198	Mm.397619	NM_010210	ENSMUSG00000060579	**Fhit**	AW045638|Fra14A2	fragile histidine triad gene	LOSS	protein-coding
990_T1,T2	chr18	2.5E+07	2E+07	999	+	**2**	**25**	NA	intron (NM_175276, intron 1 of 26)	L1_Mus1|LINE|L1	21377	NM_001289655	225288	Mm.329322	NM_175276	ENSMUSG00000034295	**Fhod3**	A930009H06Rik|FHOS2|mKIAA1695	formin homology 2 domain containing 3	LOSS	protein-coding
697_T12,T2	chr18	8582501	9E+06	999	+	**2**	**25**	NA	Intergenic	Intergenic	-629856	NM_008058	14370	Mm.184289	NM_008058	ENSMUSG00000036904	**Fzd8**	Fz8	frizzled class receptor 8	LOSS	protein-coding
699_T12,T2	chr18	8655501	9E+06	999	+	**2**	**25**	NA	Intergenic	MTC|LTR|ERVL-MaLR	-556856	NM_008058	14370	Mm.184289	NM_008058	ENSMUSG00000036904	**Fzd8**	Fz8	frizzled class receptor 8	LOSS	protein-coding
701_T12,T2	chr18	9111501	9E+06	4999	+	**2**	**25**	NA	Intergenic	L1Md_T|LINE|L1	-98856	NM_008058	14370	Mm.184289	NM_008058	ENSMUSG00000036904	**Fzd8**	Fz8	frizzled class receptor 8	LOSS	protein-coding
703_T12,T2	chr18	9172501	9E+06	4999	+	**2**	**25**	NA	Intergenic	L1Md_T|LINE|L1	-37856	NM_008058	14370	Mm.184289	NM_008058	ENSMUSG00000036904	**Fzd8**	Fz8	frizzled class receptor 8	LOSS	protein-coding
2095_T12,T2	chr18	8.2E+07	8E+07	2999	+	**2**	**25**	NA	Intergenic	L1Md_F2|LINE|L1	139777	NM_008082	14427	Mm.6219	NM_008082	ENSMUSG00000024553	**Galr1**	Galnr1	galanin receptor 1	LOSS	protein-coding
948_T12,T1	chr18	2.1E+07	2E+07	11999	+	**2**	**25**	NA	Intergenic	Intergenic	-173361	NM_001033445	381126	Mm.312276	NM_001033445	ENSMUSG00000042680	**Garem1**	C86169|Fam59a|Garem|Gm944|mKIAA4238	GRB2 associated regulator of MAPK1 subtype 1	LOSS	protein-coding
358_T12,T3	chr14	3.8E+07	4E+07	82999	+	**2**	**25**	NA	Intergenic	L1Md_F2|LINE|L1	-415678	NM_078478	66092	Mm.182912	NM_078478	ENSMUSG00000041028	**Ghitm**	1010001P14Rik|C77840|MICS1|PTD010|Tmbim5	growth hormone inducible transmembrane protein	LOSS	protein-coding

360_T12,T3	chr14	3.8E+07	4E+07	12999	+	**2**	**25**	NA	Intergenic	Intergenic	-476678	NM_078478	66092	Mm.182912	NM_078478	ENSMUSG00000041028	**Ghitm**	1010001P14Rik|C77840|MICS1|PTD010|Tmbim5	growth hormone inducible transmembrane protein	LOSS	protein-coding
366_T12,T2	chr14	3.8E+07	4E+07	30999	+	**2**	**25**	NA	Intergenic	Intergenic	-686678	NM_078478	66092	Mm.182912	NM_078478	ENSMUSG00000041028	**Ghitm**	1010001P14Rik|C77840|MICS1|PTD010|Tmbim5	growth hormone inducible transmembrane protein	LOSS	protein-coding
368_T12,T2	chr14	3.8E+07	4E+07	26999	+	**2**	**25**	NA	Intergenic	Intergenic	-770678	NM_078478	66092	Mm.182912	NM_078478	ENSMUSG00000041028	**Ghitm**	1010001P14Rik|C77840|MICS1|PTD010|Tmbim5	growth hormone inducible transmembrane protein	LOSS	protein-coding
372_T12,T42	chr14	3.8E+07	4E+07	80999	+	**2**	**25**	NA	Intergenic	L1Md_F2|LINE|L1	-860678	NM_078478	66092	Mm.182912	NM_078478	ENSMUSG00000041028	**Ghitm**	1010001P14Rik|C77840|MICS1|PTD010|Tmbim5	growth hormone inducible transmembrane protein	LOSS	protein-coding
705_T12,T2	chr18	9262501	9E+06	5999	+	**2**	**25**	NA	Intergenic	L1Md_T|LINE|L1	17309	NM_153086	225152	Mm.119273	NM_153086	ENSMUSG00000036855	**Gjd4**	9430022F06Rik|Cx39	gap junction protein, delta 4	LOSS	protein-coding
1340_T2,T32	chr18	4.3E+07	4E+07	999	+	**2**	**25**	NA	Intergenic	ORR1B1|LTR|ERVL-MaLR	-15348	NM_181543	240239	Mm.186779	NM_181543	ENSMUSG00000042816	**Gpr151**	C130082O03Rik|GalRL|PGR7|nGPCR-2037	G protein-coupled receptor 151	LOSS	protein-coding
1151_T12,T32	chr18	3.2E+07	3E+07	999	+	**2**	**25**	NA	TTS (NM_001025381)	TTS (NM_001025381)	7636	NM_001025381	574402	Mm.391108	NM_001025381	ENSMUSG00000052229	**Gpr17**	AI853548	G protein-coupled receptor 17	LOSS	protein-coding
1152_T1,T32	chr18	3.2E+07	3E+07	999	+	**2**	**25**	NA	TTS (NM_001025381)	TTS (NM_001025381)	6636	NM_001025381	574402	Mm.391108	NM_001025381	ENSMUSG00000052229	**Gpr17**	AI853548	G protein-coupled receptor 17	LOSS	protein-coding
305_T12,T4	chr14	3.4E+07	3E+07	58999	+	**2**	**25**	NA	intron (NM_183209, intron 1 of 1)	intron (NM_183209, intron 1 of 1)	1633	NM_183209	432839	Mm.294643	NM_183209	ENSMUSG00000071531	**Gprin2**	C130040D06Rik|C230073P13|Gm286|mKIAA0514	G protein regulated inducer of neurite outgrowth 2	LOSS	protein-coding
328_T12,T32	chr14	3.5E+07	3E+07	38999	+	**2**	**25**	NA	Intergenic	Intergenic	-59136	NM_008166	14803	Mm.121569	NM_008166	ENSMUSG00000041078	**Grid1**	GluD1|GluRdelta1	glutamate receptor, ionotropic, delta 1	LOSS	protein-coding
82_T12,T42	chr12	1E+08	1E+08	4999	+	**2**	**25**	NA	Intergenic	Intergenic	-2764	NM_010351	14836	Mm.129	NM_010351	ENSMUSG00000021095	**Gsc**	-	goosecoid homeobox	LOSS	protein-coding
1164_T12,T1	chr18	3.2E+07	3E+07	353999	+	**2**	**25**	NA	Intergenic	L1_Mus2|LINE|L1	57534	NM_001048207	71683	Mm.292145	NM_027863	ENSMUSG00000090523	**Gypc**	0610037F22Rik|Cd236r	glycophorin C	LOSS	protein-coding
762_T12,T1	chr18	1.3E+07	1E+07	115999	+	**2**	**25**	NA	Intergenic	Intergenic	230510	NM_153087	225192	Mm.207073	NM_153087	ENSMUSG00000037346	**Hrh4**	AXOR35|BG26|GPCR105|GPRv53|H4|H4R|HH4R	histamine receptor H4	LOSS	protein-coding
627_T1,T32	chr16	6.5E+07	7E+07	3999	+	**2**	**25**	NA	intron (NM_008310, intron 2 of 2)	LTRIS2|LTR|ERV1	67284	NM_008310	15557	Mm.5040	NM_008310	ENSMUSG00000050783	**Htr1f**	Htr1eb	5-hydroxytryptamine (serotonin) receptor 1F	LOSS	protein-coding
2303_T12,T2	chr2	8.4E+07	8E+07	3999	+	**2**	**25**	NA	Intergenic	Intergenic	-8897	NM_008402	16410	Mm.227	NM_008402	ENSMUSG00000027087	**Itgav**	1110004F14Rik|2610028E01Rik|CD51|D430040G12Rik	integrin alpha V	LOSS	protein-coding

273_T22,T3	chr14	3.1E+07	3E+07	102999	+	**2**	**25**	NA	intron (NM_001289632, intron 16 of 21)	intron (NM_001289632, intron 16 of 21)	10524	NM_001289632	16427	Mm.34819	NM_018746	ENSMUSG00000021922	**Itih4**	ITI-HC4|Itih-4|PK-120	inter alpha-trypsin inhibitor, heavy chain 4	LOSS	protein-coding
199_T2,T42	chr14	2E+07	2E+07	4999	+	**2**	**25**	NA	Intergenic	(GGA)n|Simple_repeat|Simple_repeat	14162	NM_029006	74571	Mm.105571	NM_029006	ENSMUSG00000023387	**Kcnk16**	4731413G05Rik|TALK-1|TALK1	potassium channel, subfamily K, member 16	LOSS	protein-coding
1385_T22,T32	chr18	4.5E+07	5E+07	999	+	**2**	**25**	NA	intron (NM_001312905, intron 1 of 10)	IAPLTR2_Mm|LTR|ERVK	27140	NM_001312905	140492	Mm.411614	NM_080465	ENSMUSG00000054477	**Kcnn2**	KCa2.2|SK2|SKCA2|bc|fri	potassium intermediate/small conductance calcium-activated channel, subfamily N, member 2	LOSS	protein-coding
1393_T12,T1	chr18	4.6E+07	5E+07	22999	+	**2**	**25**	NA	intron (NM_001312905, intron 2 of 10)	intron (NM_001312905, intron 2 of 10)	-34154	NM_080465	140492	Mm.411614	NM_080465	ENSMUSG00000054477	**Kcnn2**	KCa2.2|SK2|SKCA2|bc|fri	potassium intermediate/small conductance calcium-activated channel, subfamily N, member 2	LOSS	protein-coding
1272_T1,T22	chr18	4.1E+07	4E+07	999	+	**2**	**25**	NA	Intergenic	Intergenic	294639	NM_026135	383348	Mm.76366	NM_026135	ENSMUSG00000051401	**Kctd16**	4930434H12Rik|Gm1267	potassium channel tetramerisation domain containing 16	LOSS	protein-coding
1282_T12,T2	chr18	4.1E+07	4E+07	7999	+	**2**	**25**	NA	Intergenic	L1Md_F2|LINE|L1	354139	NM_026135	383348	Mm.76366	NM_026135	ENSMUSG00000051401	**Kctd16**	4930434H12Rik|Gm1267	potassium channel tetramerisation domain containing 16	LOSS	protein-coding
1285_T12,T1	chr18	4.1E+07	4E+07	53999	+	**2**	**25**	NA	Intergenic	Intergenic	423139	NM_026135	383348	Mm.76366	NM_026135	ENSMUSG00000051401	**Kctd16**	4930434H12Rik|Gm1267	potassium channel tetramerisation domain containing 16	LOSS	protein-coding
1287_T12,T1	chr18	4.1E+07	4E+07	150999	+	**2**	**25**	NA	Intergenic	L1Md_F2|LINE|L1	547639	NM_026135	383348	Mm.76366	NM_026135	ENSMUSG00000051401	**Kctd16**	4930434H12Rik|Gm1267	potassium channel tetramerisation domain containing 16	LOSS	protein-coding
1270_T1,T2	chr18	4E+07	4E+07	45999	+	**2**	**25**	NA	intron (NM_026135, intron 1 of 1)	MYSERV16_I-int|LTR|ERVK	223139	NM_026135	383348	Mm.76366	NM_026135	ENSMUSG00000051401	**Kctd16**	4930434H12Rik|Gm1267	potassium channel tetramerisation domain containing 16	LOSS	protein-coding
606_T1,T2	chr14	9.7E+07	1E+08	3999	+	**2**	**25**	NA	Intergenic	L1Md_F3|LINE|L1	-640466	NM_053105	93688	Mm.308735	NM_053105	ENSMUSG00000022076	**Klhl1**	mKIAA1490	kelch-like 1	LOSS	protein-coding
603_T22,T2	chr14	9.6E+07	1E+08	3999	+	**2**	**25**	NA	intron (NM_053105, intron 1 of 10)	L1Md_T|LINE|L1	111534	NM_053105	93688	Mm.308735	NM_053105	ENSMUSG00000022076	**Klhl1**	mKIAA1490	kelch-like 1	LOSS	protein-coding
2396_T2,T32	chr6	1.3E+08	1E+08	1999	+	**2**	**25**	NA	Intergenic	Intergenic	-8212	NM_021378	58179	Mm.358684	NM_021378	ENSMUSG00000033027	**Klrc3**	Klrc2|Nkg2e	killer cell lectin-like receptor subfamily C, member 3	LOSS	protein-coding
759_T12,T1	chr18	1.2E+07	1E+07	41999	+	**2**	**25**	NA	Intergenic	Intergenic	-4524	NM_010680	16774	Mm.42012	NM_010680	ENSMUSG00000024421	**Lama3**	Lama3B|[a]3B	laminin, alpha 3	LOSS	protein-coding

2427_T22,T3	chr7	5.5E+07	5E+07	3999	+	**2**	**25**	NA	Intergenic	Intergenic	-171745	NM_178705	233271	Mm.387344	NM_178705	ENSMUSG00000063297	**Luzp2**	9330154K17Rik|D130073E01	leucine zipper protein 2	LOSS	protein-coding
299_T2,T32	chr14	3.3E+07	3E+07	4999	+	**2**	**25**	NA	Intergenic	L1MCa|LINE|L1	-33842	NM_016700	26419	Mm.21495	NM_016700	ENSMUSG00000021936	**Mapk8**	AI849689|JNK|JNK1|Prkm8|SAPK1	mitogen-activated protein kinase 8	LOSS	protein-coding
297_T2,T32	chr14	3.3E+07	3E+07	14999	+	**2**	**25**	NA	intron (NM_001310453, intron 2 of 11)	intron (NM_001310453, intron 2 of 11)	2024	NM_001310452	26419	Mm.21495	NM_016700	ENSMUSG00000021936	**Mapk8**	AI849689|JNK|JNK1|Prkm8|SAPK1	mitogen-activated protein kinase 8	LOSS	protein-coding
1862_T12,T2	chr18	7.1E+07	7E+07	21999	+	**2**	**25**	NA	Intergenic	Lx6|LINE|L1	166208	NM_010773	17191	Mm.322	NM_010773	ENSMUSG00000024513	**Mbd2**	MBD2a	methyl-CpG binding domain protein 2	LOSS	protein-coding
1866_T12,T1	chr18	7.1E+07	7E+07	4999	+	**2**	**25**	NA	Intergenic	L1Md_A|LINE|L1	222708	NM_010773	17191	Mm.322	NM_010773	ENSMUSG00000024513	**Mbd2**	MBD2a	methyl-CpG binding domain protein 2	LOSS	protein-coding
1868_T12,T1	chr18	7.1E+07	7E+07	13999	+	**2**	**25**	NA	Intergenic	MIR|SINE|MIR	245208	NM_010773	17191	Mm.322	NM_010773	ENSMUSG00000024513	**Mbd2**	MBD2a	methyl-CpG binding domain protein 2	LOSS	protein-coding
1872_T12,T2	chr18	7.1E+07	7E+07	12999	+	**2**	**25**	NA	Intergenic	L1Md_T|LINE|L1	576708	NM_010773	17191	Mm.322	NM_010773	ENSMUSG00000024513	**Mbd2**	MBD2a	methyl-CpG binding domain protein 2	LOSS	protein-coding
1879_T12,T32	chr18	7.1E+07	7E+07	5999	+	**2**	**25**	NA	intron (NM_007831, intron 21 of 28)	intron (NM_007831, intron 21 of 28)	776208	NM_010773	17191	Mm.322	NM_010773	ENSMUSG00000024513	**Mbd2**	MBD2a	methyl-CpG binding domain protein 2	LOSS	protein-coding
1877_T12,T3	chr18	7.1E+07	7E+07	999	+	**2**	**25**	NA	intron (NM_007831, intron 22 of 28)	intron (NM_007831, intron 22 of 28)	768708	NM_010773	17191	Mm.322	NM_010773	ENSMUSG00000024513	**Mbd2**	MBD2a	methyl-CpG binding domain protein 2	LOSS	protein-coding
1875_T12,T1	chr18	7.1E+07	7E+07	46999	+	**2**	**25**	NA	intron (NM_007831, intron 27 of 28)	intron (NM_007831, intron 27 of 28)	728708	NM_010773	17191	Mm.322	NM_010773	ENSMUSG00000024513	**Mbd2**	MBD2a	methyl-CpG binding domain protein 2	LOSS	protein-coding
1857_T1,T32	chr18	7.1E+07	7E+07	999	+	**2**	**25**	NA	intron (NM_010773, intron 3 of 6)	intron (NM_010773, intron 3 of 6)	41708	NM_010773	17191	Mm.322	NM_010773	ENSMUSG00000024513	**Mbd2**	MBD2a	methyl-CpG binding domain protein 2	LOSS	protein-coding
2098_T12,T2	chr18	8.2E+07	8E+07	999	+	**2**	**25**	NA	Intergenic	RLTR17B_Mm|LTR|ERVK	-25123	NM_010777	17196	Mm.252063	NM_010777	ENSMUSG00000041607	**Mbp**	C76307|Hmbpr|R75289|golli-mbp|jve|mld|shi	myelin basic protein	LOSS	protein-coding
1829_T12,T1	chr18	6.8E+07	7E+07	3999	+	**2**	**25**	NA	Intergenic	L1_Mm|LINE|L1	-32180	NM_001271716	17200	Mm.426053	NM_008560	ENSMUSG00000045569	**Mc2r**	ACTH-R|ACTHR|MC2-R	melanocortin 2 receptor	LOSS	protein-coding
1832_T2,T32	chr18	6.8E+07	7E+07	999	+	**2**	**25**	NA	Intergenic	Intergenic	-52680	NM_001271716	17200	Mm.426053	NM_008560	ENSMUSG00000045569	**Mc2r**	ACTH-R|ACTHR|MC2-R	melanocortin 2 receptor	LOSS	protein-coding

1825_T12,T1	chr18	6.8E+07	7E+07	999	+	**2**	**25**	NA	intron (NM_001301372, intron 2 of 4)	L1MC3|LINE|L1	5840	NM_001271717	17200	Mm.426053	NM_008560	ENSMUSG00000045569	**Mc2r**	ACTH-R|ACTHR|MC2-R	melanocortin 2 receptor	LOSS	protein-coding
87_T12,T2	chr12	1.1E+08	1E+08	1999	+	**2**	**25**	NA	intron (NR_027651, intron 5 of 8)	intron (NR_027651, intron 5 of 8)	3102	NR_003633	17263	Mm.289645	NM_144513	ENSMUSG00000021268	**Meg3**	2900016C05Rik|3110050O07Rik|6330408G06Rik|AI425946|AW108224|D12Bwg1266e|Gtl2|R74756|R75394	maternally expressed 3	LOSS	ncRNA
1939_T12,T2	chr18	7.3E+07	7E+07	10999	+	**2**	**25**	NA	Intergenic	L1Md_T|LINE|L1	-340705	NM_001039214	240396	Mm.24131	NM_001039214	ENSMUSG00000037253	**Mex3c**	A130001D14Rik|BC035207|BM-013|Rkhd2	mex3 RNA binding family member C	LOSS	protein-coding
1942_T12,T1	chr18	7.3E+07	7E+07	7999	+	**2**	**25**	NA	Intergenic	Intergenic	-281205	NM_001039214	240396	Mm.24131	NM_001039214	ENSMUSG00000037253	**Mex3c**	A130001D14Rik|BC035207|BM-013|Rkhd2	mex3 RNA binding family member C	LOSS	protein-coding
1945_T12,T2	chr18	7.3E+07	7E+07	999	+	**2**	**25**	NA	Intergenic	L1Md_F2|LINE|L1	-235705	NM_001039214	240396	Mm.24131	NM_001039214	ENSMUSG00000037253	**Mex3c**	A130001D14Rik|BC035207|BM-013|Rkhd2	mex3 RNA binding family member C	LOSS	protein-coding
1947_T2,T32	chr18	7.3E+07	7E+07	999	+	**2**	**25**	NA	Intergenic	Intergenic	-229705	NM_001039214	240396	Mm.24131	NM_001039214	ENSMUSG00000037253	**Mex3c**	A130001D14Rik|BC035207|BM-013|Rkhd2	mex3 RNA binding family member C	LOSS	protein-coding
677_T12,T2	chr18	7610501	8E+06	2999	+	**2**	**25**	NA	intron (NM_001161620, intron 1 of 12)	(GGAGAA)n|Simple_repeat|Simple_repeat	14863	NM_001081287	75739	Mm.133293	NM_001081287	ENSMUSG00000057440	**Mpp7**	1110068J02Rik|2810038M04Rik|5430426E14Rik|AI415104|Gm955	membrane protein, palmitoylated 7 (MAGUK p55 subfamily member 7)	LOSS	protein-coding
1955_T1,T2	chr18	7.4E+07	7E+07	5999	+	**2**	**25**	NA	Intergenic	L1Md_F2|LINE|L1	57115	NM_001305882	71263	Mm.126220	NM_027741	ENSMUSG00000064036	**Mro**	4930507C04Rik|4933435E20Rik	maestro	LOSS	protein-coding
95_T22,T3	chr13	6.9E+07	7E+07	4999	+	**2**	**25**	NA	Intergenic	L1Md_A|LINE|L1	75104	NM_001308475	210009	Mm.205514	NM_172480	ENSMUSG00000034617	**Mtrr**	4732420G08|MSR	5-methyltetrahydrofolate-homocysteine methyltransferase reductase	LOSS	protein-coding
1154_T12,T1	chr18	3.2E+07	3E+07	78999	+	**2**	**25**	NA	intron (NM_032394, intron 9 of 46)	URR1B|DNA|hAT-Charlie	33931	NM_032394	17922	Mm.440013	NM_032394	ENSMUSG00000024388	**Myo7b**	-	myosin VIIB	LOSS	protein-coding
2423_T3,T4	chr7	4.5E+07	4E+07	4999	+	**2**	**25**	NA	intron (NM_008437, intron 1 of 8)	(GA)n|Simple_repeat|Simple_repeat	5555	NM_008437	16541	Mm.383181	NM_008437	ENSMUSG00000002204	**Napsa**	KAP|Kdap|NAP1|SNAPA|pronapsin	napsin A aspartic peptidase	LOSS	protein-coding
61_T3,T4	chr12	6.9E+07	7E+07	999	+	**2**	**25**	NA	intron (NM_025441, intron 8 of 32)	intron (NM_025441, intron 8 of 32)	11176	NM_025441	66244	Mm.46705	NM_025441	ENSMUSG00000020982	**Nemf**	1500011I12Rik|4933405E14Rik|Sdccag1	nuclear export mediator factor	LOSS	protein-coding
2148_T1,T2	chr18	8.6E+07	9E+07	9999	+	**2**	**25**	NA	Intergenic	Intergenic	-700452	NM_144946	246317	Mm.329578	NM_144946	ENSMUSG00000050321	**Neto1**	AI851453|Btcl1|C130005O10Rik	neuropilin (NRP) and tolloid (TLL)-like 1	LOSS	protein-coding

2152_T1,T2	chr18	8.6E+07	9E+07	3999	+	**2**	**25**	NA	Intergenic	L1Md_T|LINE|L1	-570452	NM_144946	246317	Mm.329578	NM_144946	ENSMUSG00000050321	**Neto1**	AI851453|Btcl1|C130005O10Rik	neuropilin (NRP) and tolloid (TLL)-like 1	LOSS	protein-coding
2154_T1,T2	chr18	8.6E+07	9E+07	6999	+	**2**	**25**	NA	Intergenic	Intergenic	-560952	NM_144946	246317	Mm.329578	NM_144946	ENSMUSG00000050321	**Neto1**	AI851453|Btcl1|C130005O10Rik	neuropilin (NRP) and tolloid (TLL)-like 1	LOSS	protein-coding
2157_T12,T1	chr18	8.6E+07	9E+07	5999	+	**2**	**25**	NA	Intergenic	MTD|LTR|ERVL-MaLR	-487452	NM_144946	246317	Mm.329578	NM_144946	ENSMUSG00000050321	**Neto1**	AI851453|Btcl1|C130005O10Rik	neuropilin (NRP) and tolloid (TLL)-like 1	LOSS	protein-coding
2159_T12,T1	chr18	8.6E+07	9E+07	126999	+	**2**	**25**	NA	Intergenic	Intergenic	-413952	NM_144946	246317	Mm.329578	NM_144946	ENSMUSG00000050321	**Neto1**	AI851453|Btcl1|C130005O10Rik	neuropilin (NRP) and tolloid (TLL)-like 1	LOSS	protein-coding
2161_T1,T2	chr18	8.6E+07	9E+07	999	+	**2**	**25**	NA	Intergenic	Intergenic	-212952	NM_144946	246317	Mm.329578	NM_144946	ENSMUSG00000050321	**Neto1**	AI851453|Btcl1|C130005O10Rik	neuropilin (NRP) and tolloid (TLL)-like 1	LOSS	protein-coding
2163_T1,T2	chr18	8.6E+07	9E+07	999	+	**2**	**25**	NA	Intergenic	Intergenic	-204952	NM_144946	246317	Mm.329578	NM_144946	ENSMUSG00000050321	**Neto1**	AI851453|Btcl1|C130005O10Rik	neuropilin (NRP) and tolloid (TLL)-like 1	LOSS	protein-coding
2165_T12,T2	chr18	8.6E+07	9E+07	999	+	**2**	**25**	NA	Intergenic	L1Md_T|LINE|L1	-187952	NM_144946	246317	Mm.329578	NM_144946	ENSMUSG00000050321	**Neto1**	AI851453|Btcl1|C130005O10Rik	neuropilin (NRP) and tolloid (TLL)-like 1	LOSS	protein-coding
2071_T12,T2	chr18	8.1E+07	8E+07	999	+	**2**	**25**	NA	Intergenic	Intergenic	-2929	NM_001164109	18018	Mm.329560	NM_016791	ENSMUSG00000033016	**Nfatc1**	2210017P03Rik|AI449492|AV076380|NF-ATc|NFAT2|NFATc|Nfatcb	nuclear factor of activated T cells, cytoplasmic, calcineurin dependent 1	LOSS	protein-coding
2069_T12,T2	chr18	8.1E+07	8E+07	999	+	**2**	**25**	NA	intron (NM_198429, intron 1 of 9)	intron (NM_198429, intron 1 of 9)	9173	NM_001164111	18018	Mm.329560	NM_016791	ENSMUSG00000033016	**Nfatc1**	2210017P03Rik|AI449492|AV076380|NF-ATc|NFAT2|NFATc|Nfatcb	nuclear factor of activated T cells, cytoplasmic, calcineurin dependent 1	LOSS	protein-coding
972_T2,T3	chr18	2.3E+07	2E+07	999	+	**2**	**25**	NA	intron (NM_199024, intron 1 of 9)	L1Md_T|LINE|L1	48663	NM_001161483	319211	Mm.209896	NM_199024	ENSMUSG00000041923	**Nol4**	1700013J13Rik|4930568N03Rik|Gm1262	nucleolar protein 4	LOSS	protein-coding
303_T12,T4	chr14	3.4E+07	3E+07	58999	+	**2**	**25**	NA	Intergenic	Intergenic	17419	NM_008919	19065	Mm.57059	NM_008919	ENSMUSG00000048337	**Npy4r**	NYYR-D|Ppyr1|Y4	neuropeptide Y receptor Y4	LOSS	protein-coding
1248_T22,T3	chr18	4E+07	4E+07	999	+	**2**	**25**	NA	Intergenic	L1Md_A|LINE|L1	-111755	NM_008173	14815	Mm.129481	NM_008173	ENSMUSG00000024431	**Nr3c1**	GR|Grl-1|Grl1	nuclear receptor subfamily 3, group C, member 1	LOSS	protein-coding
1183_T2,T32	chr18	3.3E+07	3E+07	999	+	**2**	**25**	NA	Intergenic	L1Md_T|LINE|L1	68634	NM_001267717	27528	Mm.407415	NM_053078	ENSMUSG00000042834	**Nrep**	AI325076|D0H4S114|Harp|P311|PTZ17|SEZ17	neuronal regeneration related protein	LOSS	protein-coding
279_T22,T3	chr14	3.1E+07	3E+07	6999	+	**2**	**25**	NA	intron (NM_027289, intron 6 of 12)	intron (NM_027289, intron 6 of 12)	1147	NM_027289	70021	Mm.261681	NM_027289	ENSMUSG00000071547	**Nt5dc2**	2510015F01Rik	5'-nucleotidase domain containing 2	LOSS	protein-coding
201_T42,T4	chr14	2E+07	2E+07	999	+	**2**	**25**	NA	Intergenic	Intergenic	-6690	NM_026341	67725	Mm.317636	NM_026341	ENSMUSG00000021809	**Nudt13**	4933433B15Rik	nudix (nucleoside diphosphate linked moiety X)-type motif 13	LOSS	protein-coding

293_T22,T2	chr14	3.2E+07	3E+07	2999	+	**2**	**25**	NA	Intergenic	Intergenic	-20019	NM_001081130	239017	Mm.318302	NM_001081130	ENSMUSG00000021913	**Ogdhl**	-	oxoglutarate dehydrogenase-like	LOSS	protein-coding
2329_T22,T3	chr3	1.2E+08	1E+08	3999	+	**2**	**25**	NA	Intergenic	L1Md_F2|LINE|L1	207535	NM_153157	229759	Mm.54183	NM_153157	ENSMUSG00000027965	**Olfm3**	B230206G02Rik	olfactomedin 3	LOSS	protein-coding
2054_T22,T32	chr18	8E+07	8E+07	1999	+	**2**	**25**	NA	Intergenic	IAPEz-int|LTR|ERVK	-342395	NM_053117	93737	Mm.24678	NM_053117	ENSMUSG00000056214	**Pard6g**	2410049N21Rik|Par6a	par-6 family cell polarity regulator gamma	LOSS	protein-coding
2060_T2,T32	chr18	8E+07	8E+07	999	+	**2**	**25**	NA	Intergenic	L1_Mur3|LINE|L1	-314895	NM_053117	93737	Mm.24678	NM_053117	ENSMUSG00000056214	**Pard6g**	2410049N21Rik|Par6a	par-6 family cell polarity regulator gamma	LOSS	protein-coding
1233_T12,T3	chr18	3.7E+07	4E+07	999	+	**2**	**25**	NA	intron (NM_138663, intron 1 of 3)	L1Md_T|LINE|L1	17770	NM_138663	192164	Mm.308500	NM_138663	ENSMUSG00000103310	**Pcdha12**	Cnr5|Crnr5|Pcdha13	protocadherin alpha 12	LOSS	protein-coding
1241_T12,T1	chr18	3.7E+07	4E+07	999	+	**2**	**25**	NA	intron (NM_001301259, intron 1 of 3)	L1Md_F|LINE|L1	-12855	NM_053134	93880	Mm.286019	NM_053134	ENSMUSG00000051242	**Pcdhb9**	Pcdhb4C|Pcdhbl	protocadherin beta 9	LOSS	protein-coding
2370_T22,T4	chr6	4746501	5E+06	2999	+	**2**	**25**	NA	promoter-TSS (NM_011360)	promoter-TSS (NM_011360)	694	NM_130877	170676	Mm.320575	NM_130877	ENSMUSG00000092035	**Peg10**	AA407948|Edr|HB-1|MEF3L|Mar2|Mart2|MyEF-3	paternally expressed 10	LOSS	protein-coding
1212_T12,T1	chr18	3.6E+07	4E+07	999	+	**2**	**25**	NA	Intergenic	PB1D7|SINE|Alu	-27505	NM_026027	67199	Mm.30184	NM_026027	ENSMUSG00000024346	**Pfdn1**	2700086I23Rik|AA408327|AU044714	prefoldin 1	LOSS	protein-coding
1996_T1,T3	chr18	7.7E+07	8E+07	4999	+	**2**	**25**	NA	intron (NM_001164170, intron 1 of 12)	L1Md_A|LINE|L1	25320	NM_001164168	17344	Mm.6370	NM_008602	ENSMUSG00000025423	**Pias2**	6330408K17Rik|AI462206|ARIP3|AU018068|Dib|Miz1|PIASxalpha|PIASxb|PIASxbeta|SIZ2	protein inhibitor of activated STAT 2	LOSS	protein-coding
1747_T12,T32	chr18	6.3E+07	6E+07	1999	+	**2**	**25**	NA	Intergenic	Intergenic	-87317	NM_001039485	667742	Mm.158720	NM_172629	ENSMUSG00000041482	**Piezo2**	5930434P17|9030411M15Rik|9430028L06Rik|Fam38b|Fam38b2	piezo-type mechanosensitive ion channel component 2	LOSS	protein-coding
239_T22,T2	chr14	2.4E+07	2E+07	3999	+	**2**	**25**	NA	Intergenic	Intergenic	-1454	NM_001081247	218832	Mm.343610	NM_183157	ENSMUSG00000025280	**Polr3a**	9330175N20Rik|BC053071|RPC1|RPC155	polymerase (RNA) III (DNA directed) polypeptide A	LOSS	protein-coding
1293_T22,T32	chr18	4.1E+07	4E+07	999	+	**2**	**25**	NA	Intergenic	Intergenic	780194	NM_029942	77619	Mm.30649	NM_029942	ENSMUSG00000056671	**Prelid2**	1700003A01Rik|C330008K14Rik	PRELI domain containing 2	LOSS	protein-coding
1296_T2,T32	chr18	4.1E+07	4E+07	17999	+	**2**	**25**	NA	Intergenic	(TAA)n|Simple_repeat|Simple_repeat	650694	NM_029942	77619	Mm.30649	NM_029942	ENSMUSG00000056671	**Prelid2**	1700003A01Rik|C330008K14Rik	PRELI domain containing 2	LOSS	protein-coding
1304_T12,T32	chr18	4.1E+07	4E+07	5999	+	**2**	**25**	NA	Intergenic	L1Md_T|LINE|L1	566694	NM_029942	77619	Mm.30649	NM_029942	ENSMUSG00000056671	**Prelid2**	1700003A01Rik|C330008K14Rik	PRELI domain containing 2	LOSS	protein-coding

1312_T12,T2	chr18	4.2E+07	4E+07	5999	+	**2**	**25**	NA	Intergenic	L1Md_A|LINE|L1	437694	NM_029942	77619	Mm.30649	NM_029942	ENSMUSG00000056671	**Prelid2**	1700003A01Rik|C330008K14Rik	PRELI domain containing 2	LOSS	protein-coding
1514_T2,T32	chr18	5.1E+07	5E+07	999	+	**2**	**25**	NA	Intergenic	L1MA4|LINE|L1	-202898	NM_001081224	71373	Mm.63546	NM_001081224	ENSMUSG00000073565	**Prr16**	5430406M13Rik|AI607429	proline rich 16	LOSS	protein-coding
1515_T12,T1	chr18	5.1E+07	5E+07	2999	+	**2**	**25**	NA	Intergenic	RMER6A|LTR|ERVK	-195898	NM_001081224	71373	Mm.63546	NM_001081224	ENSMUSG00000073565	**Prr16**	5430406M13Rik|AI607429	proline rich 16	LOSS	protein-coding
1519_T2,T32	chr18	5.1E+07	5E+07	999	+	**2**	**25**	NA	Intergenic	Intergenic	-115898	NM_001081224	71373	Mm.63546	NM_001081224	ENSMUSG00000073565	**Prr16**	5430406M13Rik|AI607429	proline rich 16	LOSS	protein-coding
1522_T12,T2	chr18	5.1E+07	5E+07	999	+	**2**	**25**	NA	Intergenic	L1MA4|LINE|L1	-39898	NM_001081224	71373	Mm.63546	NM_001081224	ENSMUSG00000073565	**Prr16**	5430406M13Rik|AI607429	proline rich 16	LOSS	protein-coding
1530_T12,T22	chr18	5.1E+07	5E+07	4999	+	**2**	**25**	NA	Intergenic	L1Md_A|LINE|L1	279102	NM_001081224	71373	Mm.63546	NM_001081224	ENSMUSG00000073565	**Prr16**	5430406M13Rik|AI607429	proline rich 16	LOSS	protein-coding
169_T2,T3	chr14	1.7E+07	2E+07	48999	+	**2**	**25**	NA	intron (NM_001289760, intron 1 of 9)	intron (NM_001289760, intron 1 of 9)	200331	NM_001289760	218772	Mm.259318	NM_011243	ENSMUSG00000017491	**Rarb**	A830025K23|Hap|Nr1b2	retinoic acid receptor, beta	LOSS	protein-coding
634_T42,T4	chr16	7.6E+07	8E+07	7999	+	**2**	**25**	NA	Intergenic	L1Md_A|LINE|L1	61609	NM_198302	224344	Mm.273214	NM_198302	ENSMUSG00000032940	**Rbm11**	A330018F01	RNA binding motif protein 11	LOSS	protein-coding
1114_T2,T3	chr18	3.1E+07	3E+07	999	+	**2**	**25**	NA	Intergenic	L1Md_A|LINE|L1	514128	NM_009065	19762	Mm.5163	NM_009065	ENSMUSG00000057455	**Rit2**	RIBA|Rin|Roc2	Ras-like without CAAX 2	LOSS	protein-coding
1124_T12,T1	chr18	3.1E+07	3E+07	84999	+	**2**	**25**	NA	Intergenic	Intergenic	394128	NM_009065	19762	Mm.5163	NM_009065	ENSMUSG00000057455	**Rit2**	RIBA|Rin|Roc2	Ras-like without CAAX 2	LOSS	protein-coding
1126_T12,T1	chr18	3.1E+07	3E+07	3999	+	**2**	**25**	NA	Intergenic	Intergenic	344628	NM_009065	19762	Mm.5163	NM_009065	ENSMUSG00000057455	**Rit2**	RIBA|Rin|Roc2	Ras-like without CAAX 2	LOSS	protein-coding
1133_T12,T32	chr18	3.1E+07	3E+07	999	+	**2**	**25**	NA	intron (NM_009065, intron 3 of 4)	intron (NM_009065, intron 3 of 4)	138128	NM_009065	19762	Mm.5163	NM_009065	ENSMUSG00000057455	**Rit2**	RIBA|Rin|Roc2	Ras-like without CAAX 2	LOSS	protein-coding
1128_T1,T2	chr18	3.1E+07	3E+07	999	+	**2**	**25**	NA	intron (NM_009065, intron 4 of 4)	intron (NM_009065, intron 4 of 4)	315128	NM_009065	19762	Mm.5163	NM_009065	ENSMUSG00000057455	**Rit2**	RIBA|Rin|Roc2	Ras-like without CAAX 2	LOSS	protein-coding
519_T12,T2	chr14	5.1E+07	5E+07	434999	+	**2**	**25**	NA	TTS (NM_001161731)	TTS (NM_001161731)	6000	NM_201239	58809	Mm.202665	NM_021472	ENSMUSG00000021876	**Rnase4**	C730049F20Rik|Rab1	ribonuclease, RNase A family 4	LOSS	protein-coding
982_T12,T1	chr18	2.5E+07	2E+07	13999	+	**2**	**25**	NA	Intergenic	RSINE1|SINE|B4	-17296	NM_144861	225283	Mm.28427	NM_144861	ENSMUSG00000040446	**Rprd1a**	C77387|mKIAA4077	regulation of nuclear pre-mRNA domain containing 1A	LOSS	protein-coding
983_T12,T3	chr18	2.5E+07	2E+07	999	+	**2**	**25**	NA	Intergenic	Intergenic	-28796	NM_144861	225283	Mm.28427	NM_144861	ENSMUSG00000040446	**Rprd1a**	C77387|mKIAA4077	regulation of nuclear pre-mRNA domain containing 1A	LOSS	protein-coding

2086_T12,T32	chr18	8.1E+07	8E+07	2999	+	**2**	**25**	NA	Intergenic	RLTR17B_Mm|LTR|ERVK	-69422	NM_178280	20689	Mm.215917	NM_178280	ENSMUSG00000024565	**Sall3**	B130022O04Rik|Msal|Msal-1|Sal|Salt|Spalt	sal-like 3 (Drosophila)	LOSS	protein-coding
1959_T1,T2	chr18	7.5E+07	7E+07	999	+	**2**	**25**	NA	intron (NM_201600, intron 35 of 38)	intron (NM_201600, intron 35 of 38)	19455	NR_028560	1E+08		NR_028560		**Scarna17**	MBII-119	small Cajal body-specific RNA 17	LOSS	ncRNA
398_T12,T2	chr14	4E+07	4E+07	213999	+	**2**	**25**	NA	Intergenic	L1M4|LINE|L1	478465	NM_177816	328381	Mm.235694	NM_177816	ENSMUSG00000037833	**Sh2d4b**	A430109M18Rik|D030001E08	SH2 domain containing 4B	LOSS	protein-coding
400_T12,T2	chr14	4.1E+07	4E+07	20999	+	**2**	**25**	NA	Intergenic	Intergenic	355965	NM_177816	328381	Mm.235694	NM_177816	ENSMUSG00000037833	**Sh2d4b**	A430109M18Rik|D030001E08	SH2 domain containing 4B	LOSS	protein-coding
2024_T12,T2	chr18	7.8E+07	8E+07	2999	+	**2**	**25**	NA	Intergenic	Intergenic	-1732	NM_001101038	620235	Mm.35607	NM_001101038	ENSMUSG00000091055	**Siglec15**	Cd33l3|EG620235|SIGLEC-I	sialic acid binding Ig-like lectin 15	LOSS	protein-coding
1981_T12,T2	chr18	7.7E+07	8E+07	999	+	**2**	**25**	NA	Intergenic	Intergenic	-277405	NM_001109743	664805	Mm.329543	NM_001109743	ENSMUSG00000091519	**Skor2**	Corl2|EG664805|Fussel18|Gm7348	SKI family transcriptional corepressor 2	LOSS	protein-coding
1983_T12,T2	chr18	7.7E+07	8E+07	4999	+	**2**	**25**	NA	Intergenic	L1Md_T|LINE|L1	-67405	NM_001109743	664805	Mm.329543	NM_001109743	ENSMUSG00000091519	**Skor2**	Corl2|EG664805|Fussel18|Gm7348	SKI family transcriptional corepressor 2	LOSS	protein-coding
2027_T2,T32	chr18	7.8E+07	8E+07	999	+	**2**	**25**	NA	intron (NM_207651, intron 2 of 21)	intron (NM_207651, intron 2 of 21)	167950	NM_207651	27411	Mm.44158	NM_030683	ENSMUSG00000024552	**Slc14a2**	UT-A1|UT-A3	solute carrier family 14 (urea transporter), member 2	LOSS	protein-coding
1144_T1,T32	chr18	3.2E+07	3E+07	12999	+	**2**	**25**	NA	Intergenic	L1VL2|LINE|L1	56902	NM_026165	67453	Mm.23896	NM_026165	ENSMUSG00000024259	**Slc25a46**	1200007B05Rik|AI325987	solute carrier family 25, member 46	LOSS	protein-coding
500_T12,T2	chr14	5E+07	5E+07	2999	+	**2**	**25**	NA	Intergenic	Lx7|LINE|L1	-84163	NM_029238	75288	Mm.78738	NM_029238	ENSMUSG00000021852	**Slc35f4**	4930550L21Rik	solute carrier family 35, member F4	LOSS	protein-coding
502_T12,T22	chr14	5E+07	5E+07	999	+	**2**	**25**	NA	Intergenic	L1Md_T|LINE|L1	-103163	NM_029238	75288	Mm.78738	NM_029238	ENSMUSG00000021852	**Slc35f4**	4930550L21Rik	solute carrier family 35, member F4	LOSS	protein-coding
1979_T1,T2	chr18	7.6E+07	8E+07	2999	+	**2**	**25**	NA	Intergenic	Intergenic	109555	NM_001311070	17126	Mm.152699	NM_010754	ENSMUSG00000024563	**Smad2**	7120426M23Rik|Madh2|Madr2|Smad-2|mMad2	SMAD family member 2	LOSS	protein-coding
1952_T12,T1	chr18	7.4E+07	7E+07	999	+	**2**	**25**	NA	intron (NM_008540, intron 1 of 11)	B2_Mm2|SINE|B2	15741	NM_008540	17128	Mm.100399	NM_008540	ENSMUSG00000024515	**Smad4**	AW743858|D18Wsu70e|DPC4|Madh4	SMAD family member 4	LOSS	protein-coding
2207_T12,T1	chr18	8.8E+07	9E+07	95999	+	**2**	**25**	NA	Intergenic	Intergenic	947707	NM_018821	54607	Mm.91920	NM_018821	ENSMUSG00000056153	**Socs6**	1500012M23Rik|5830401B18Rik|AI447482|Cis4|Cish4|HSPC060|SOCS-4|SOCS-6|SSI4|STAI4|STATI4|Socs4	suppressor of cytokine signaling 6	LOSS	protein-coding

2209_T12,T1	chr18	8.8E+07	9E+07	234999	+	**2**	**25**	NA	Intergenic	Intergenic	771207	NM_018821	54607	Mm.91920	NM_018821	ENSMUSG00000056153	**Socs6**	1500012M23Rik|5830401B18Rik|AI447482|Cis4|Cish4|HSPC060|SOCS-4|SOCS-6|SSI4|STAI4|STATI4|Socs4	suppressor of cytokine signaling 6	LOSS	protein-coding
2211_T12,T1	chr18	8.8E+07	9E+07	47999	+	**2**	**25**	NA	Intergenic	Intergenic	620707	NM_018821	54607	Mm.91920	NM_018821	ENSMUSG00000056153	**Socs6**	1500012M23Rik|5830401B18Rik|AI447482|Cis4|Cish4|HSPC060|SOCS-4|SOCS-6|SSI4|STAI4|STATI4|Socs4	suppressor of cytokine signaling 6	LOSS	protein-coding
2213_T1,T2	chr18	8.8E+07	9E+07	999	+	**2**	**25**	NA	Intergenic	Intergenic	524207	NM_018821	54607	Mm.91920	NM_018821	ENSMUSG00000056153	**Socs6**	1500012M23Rik|5830401B18Rik|AI447482|Cis4|Cish4|HSPC060|SOCS-4|SOCS-6|SSI4|STAI4|STATI4|Socs4	suppressor of cytokine signaling 6	LOSS	protein-coding
2216_T12,T2	chr18	8.8E+07	9E+07	10999	+	**2**	**25**	NA	Intergenic	Lx8b|LINE|L1	496207	NM_018821	54607	Mm.91920	NM_018821	ENSMUSG00000056153	**Socs6**	1500012M23Rik|5830401B18Rik|AI447482|Cis4|Cish4|HSPC060|SOCS-4|SOCS-6|SSI4|STAI4|STATI4|Socs4	suppressor of cytokine signaling 6	LOSS	protein-coding
2220_T12,T2	chr18	8.8E+07	9E+07	999	+	**2**	**25**	NA	Intergenic	Intergenic	415207	NM_018821	54607	Mm.91920	NM_018821	ENSMUSG00000056153	**Socs6**	1500012M23Rik|5830401B18Rik|AI447482|Cis4|Cish4|HSPC060|SOCS-4|SOCS-6|SSI4|STAI4|STATI4|Socs4	suppressor of cytokine signaling 6	LOSS	protein-coding
2294_T22,T2	chr2	2.4E+07	2E+07	6999	+	**2**	**25**	NA	Intergenic	L1_Mus2|LINE|L1	-223896	NM_001165997	76857	Mm.276415	NM_029773	ENSMUSG00000026771	**Spopl**	4921517N04Rik|AU014935|BB233739|E430033K04Rik	speckle-type POZ protein-like	LOSS	protein-coding
1559_T12,T1	chr18	5.2E+07	5E+07	90999	+	**2**	**25**	NA	Intergenic	L1MA4|LINE|L1	-24693	NM_026040	67222	Mm.100617	NM_026040	ENSMUSG00000024528	**Srfbp1**	2810036K01Rik|p49/STRAP	serum response factor binding protein 1	LOSS	protein-coding
1180_T22,T2	chr18	3.3E+07	3E+07	999	+	**2**	**25**	NA	Intergenic	L1Md_F2|LINE|L1	-110184	NM_133774	170459	Mm.127058	NM_133774	ENSMUSG00000024378	**Stard4**	4632419C16Rik|9030213J02Rik	StAR-related lipid transfer (START) domain containing 4	LOSS	protein-coding
1836_T2,T32	chr18	6.9E+07	7E+07	2999	+	**2**	**25**	NA	Intergenic	Intergenic	-114492	NM_013685	21413	Mm.4269	NM_013685	ENSMUSG00000053477	**Tcf4**	5730422P05Rik|ASP-I2|E2-2|E2.2|ITF-2|ITF-2b|ITF2|ME2|MITF-2A|MITF-2B|SEF-2|SEF2|SEF2-1|TFE|Tcf-4|bHLHb19	transcription factor 4	LOSS	protein-coding

1840_T1,T2	chr18	6.9E+07	7E+07	2999	+	**2**	**25**	NA	intron (NM_001083967, intron 1 of 19)	intron (NM_001083967, intron 1 of 19)	1279	NM_001083967	21413	Mm.4269	NM_013685	ENSMUSG00000053477	**Tcf4**	5730422P05Rik|ASP-I2|E2-2|E2.2|ITF-2|ITF-2b|ITF2|ME2|MITF-2A|MITF-2B|SEF-2|SEF2|SEF2-1|TFE|Tcf-4|bHLHb19	transcription factor 4	LOSS	protein-coding
144_T3,T42	chr14	1.4E+07	1E+07	999	+	**2**	**25**	NA	Intergenic	Intergenic	93313	NM_001285780	66231	Mm.295875	NM_025435	ENSMUSG00000053453	**Thoc7**	Nif3l1bp1	THO complex 7	LOSS	protein-coding
2141_T12,T1	chr18	8.5E+07	9E+07	28999	+	**2**	**25**	NA	Intergenic	Lx5b|LINE|L1	-143476	NM_025969	67105	Mm.250617	NM_025969	ENSMUSG00000024645	**Timm21**	1700034H14Rik|2700002I20Rik	tranlocase of inner mitochondrial membrane 21	LOSS	protein-coding
2143_T12,T1	chr18	8.5E+07	9E+07	204999	+	**2**	**25**	NA	Intergenic	Intergenic	-265476	NM_025969	67105	Mm.250617	NM_025969	ENSMUSG00000024645	**Timm21**	1700034H14Rik|2700002I20Rik	tranlocase of inner mitochondrial membrane 21	LOSS	protein-coding
271_T2,T3	chr14	3E+07	3E+07	503999	+	**2**	**25**	NA	TTS (NM_133761)	TTS (NM_133761)	-21631	NM_009388	21881	Mm.290692	NM_009388	ENSMUSG00000021957	**Tkt**	p68	transketolase	LOSS	protein-coding
2279_T12,T2	chr18	9E+07	9E+07	6999	+	**2**	**25**	NA	Intergenic	L1Md_A|LINE|L1	-236154	NM_198295	67988	Mm.268041	NM_198295	ENSMUSG00000024614	**Tmx3**	6430411B10Rik|A730024F05Rik|AV259382|Txndc10|mKIAA1830	thioredoxin-related transmembrane protein 3	LOSS	protein-coding
2281_T12,T1	chr18	9E+07	9E+07	42999	+	**2**	**25**	NA	Intergenic	RMER6C|LTR|ERVK	-108154	NM_198295	67988	Mm.268041	NM_198295	ENSMUSG00000024614	**Tmx3**	6430411B10Rik|A730024F05Rik|AV259382|Txndc10|mKIAA1830	thioredoxin-related transmembrane protein 3	LOSS	protein-coding
2368_T22,T2	chr5	1.4E+08	1E+08	3999	+	**2**	**25**	NA	promoter-TSS (NM_178242)	promoter-TSS (NM_178242)	-113	NM_001122730	231861	Mm.354649	NM_178242	ENSMUSG00000039477	**Tnrc18**	BC025631|EG381742|Zfp469	trinucleotide repeat containing 18	LOSS	protein-coding
992_T1,T2	chr18	2.5E+07	3E+07	999	+	**2**	**25**	NA	intron (NM_175276, intron 23 of 26)	L1M4|LINE|L1	46965	NM_001142697	66648	Mm.263189	NM_001004361	ENSMUSG00000024269	**Tpgs2**	5730437P09Rik|5730494M16Rik|AI666318|Pgs2	tubulin polyglutamylase complex subunit 2	LOSS	protein-coding
1166_T12,T4	chr18	3.3E+07	3E+07	999	+	**2**	**25**	NA	intron (NR_045381, intron 4 of 4)	L1Md_T|LINE|L1	-126383	NM_021367	53603	Mm.143716	NM_021367	ENSMUSG00000024379	**Tslp**	-	thymic stromal lymphopoietin	LOSS	protein-coding
174_T22,T3	chr14	1.8E+07	2E+07	16999	+	**2**	**25**	NA	Intergenic	MTEa|LTR|ERVL-MaLR	-90156	NM_009455	22194	Mm.4429	NM_009455	ENSMUSG00000021774	**Ube2e1**	UbcM3|Ubce5|ubcM2	ubiquitin-conjugating enzyme E2E 1	LOSS	protein-coding
176_T22,T2	chr14	1.8E+07	2E+07	52999	+	**2**	**25**	NA	Intergenic	Intergenic	-130156	NM_009455	22194	Mm.4429	NM_009455	ENSMUSG00000021774	**Ube2e1**	UbcM3|Ubce5|ubcM2	ubiquitin-conjugating enzyme E2E 1	LOSS	protein-coding
179_T22,T4	chr14	1.9E+07	2E+07	999	+	**2**	**25**	NA	Intergenic	MusHAL1|LINE|L1	-81373	NM_144839	218793	Mm.23551	NM_144839	ENSMUSG00000058317	**Ube2e2**	BC016265	ubiquitin-conjugating enzyme E2E 2	LOSS	protein-coding
181_T22,T4	chr14	1.9E+07	2E+07	17999	+	**2**	**25**	NA	Intergenic	Intergenic	-102873	NM_144839	218793	Mm.23551	NM_144839	ENSMUSG00000058317	**Ube2e2**	BC016265	ubiquitin-conjugating enzyme E2E 2	LOSS	protein-coding

184_T3,T42	chr14	1.9E+07	2E+07	999	+	**2**	**25**	NA	Intergenic	Looper|DNA|PiggyBac	-292373	NM_144839	218793	Mm.23551	NM_144839	ENSMUSG00000058317	**Ube2e2**	BC016265	ubiquitin-conjugating enzyme E2E 2	LOSS	protein-coding
637_T2,T4	chr16	7.7E+07	8E+07	6999	+	**2**	**25**	NA	intron (NM_013918, intron 6 of 23)	intron (NM_013918, intron 6 of 23)	44931	NM_013918	30940	Mm.40986	NM_013918	ENSMUSG00000022867	**Usp25**	-	ubiquitin specific peptidase 25	LOSS	protein-coding
226_T2,T3	chr14	2.2E+07	2E+07	25999	+	**2**	**25**	NA	3' UTR (NM_026283, exon 6 of 6)	3' UTR (NM_026283, exon 6 of 6)	-37061	NM_011695	22334	Mm.262327	NM_011695	ENSMUSG00000021771	**Vdac2**	Vdac6|mVDAC2|mVDAC6	voltage-dependent anion channel 2	LOSS	protein-coding
679_T1,T2	chr18	7758501	8E+06	22999	+	**2**	**25**	NA	Intergenic	Intergenic	-98832	NM_001282093	225131	Mm.204524	NM_153085	ENSMUSG00000024283	**Wac**	1110067P07Rik|A230035H12Rik|AI256735|AI256776|Wwp4	WW domain containing adaptor with coiled-coil	LOSS	protein-coding
685_T12,T22	chr18	7856501	8E+06	999	+	**2**	**25**	NA	Intergenic	MTD|LTR|ERVL-MaLR	-11832	NM_001282093	225131	Mm.204524	NM_153085	ENSMUSG00000024283	**Wac**	1110067P07Rik|A230035H12Rik|AI256735|AI256776|Wwp4	WW domain containing adaptor with coiled-coil	LOSS	protein-coding
689_T12,T2	chr18	8058501	8E+06	7999	+	**2**	**25**	NA	Intergenic	Intergenic	193303	NM_153085	225131	Mm.204524	NM_153085	ENSMUSG00000024283	**Wac**	1110067P07Rik|A230035H12Rik|AI256735|AI256776|Wwp4	WW domain containing adaptor with coiled-coil	LOSS	protein-coding
691_T12,T2	chr18	8210501	8E+06	4999	+	**2**	**25**	NA	Intergenic	L1Md_T|LINE|L1	343803	NM_153085	225131	Mm.204524	NM_153085	ENSMUSG00000024283	**Wac**	1110067P07Rik|A230035H12Rik|AI256735|AI256776|Wwp4	WW domain containing adaptor with coiled-coil	LOSS	protein-coding
693_T12,T2	chr18	8242501	8E+06	35999	+	**2**	**25**	NA	Intergenic	L1_Mus3|LINE|L1	391303	NM_153085	225131	Mm.204524	NM_153085	ENSMUSG00000024283	**Wac**	1110067P07Rik|A230035H12Rik|AI256735|AI256776|Wwp4	WW domain containing adaptor with coiled-coil	LOSS	protein-coding
695_T12,T2	chr18	8291501	8E+06	2999	+	**2**	**25**	NA	Intergenic	MTA_Mm-int|LTR|ERVL-MaLR	423803	NM_153085	225131	Mm.204524	NM_153085	ENSMUSG00000024283	**Wac**	1110067P07Rik|A230035H12Rik|AI256735|AI256776|Wwp4	WW domain containing adaptor with coiled-coil	LOSS	protein-coding
261_T22,T3	chr14	2.8E+07	3E+07	190999	+	**2**	**25**	NA	intron (NM_177814, intron 14 of 15)	intron (NM_177814, intron 14 of 15)	-221473	NM_009524	22418	Mm.287544	NM_009524	ENSMUSG00000021994	**Wnt5a**	8030457G12Rik|Wnt-5a	wingless-type MMTV integration site family, member 5A	LOSS	protein-coding
666_T12	chr17	9.2E+07	9E+07	3999	+	**1**	**12.5**	NA	Intergenic	L1Md_F2|LINE|L1	-762517	NM_001315503	11516	Mm.3407	NM_009625	ENSMUSG00000024256	**Adcyap1**	PACAP	adenylate cyclase activating polypeptide 1	LOSS	protein-coding
1733_T12	chr18	6.2E+07	6E+07	4999	+	**1**	**12.5**	NA	Intergenic	L1Md_F2|LINE|L1	-59019	NM_007420	11555	Mm.5598	NM_007420	ENSMUSG00000045730	**Adrb2**	Adrb-2|Badm|Gpcr7	adrenergic receptor, beta 2	LOSS	protein-coding
1730_T12	chr18	6.2E+07	6E+07	2999	+	**1**	**12.5**	NA	intron (NM_178928, intron 1 of 18)	(GGGAGA)n|Simple_repeat|Simple_repeat	11662	NM_178928	106877	Mm.26632	NM_178928	ENSMUSG00000033032	**Afap1l1**	AI173486	actin filament associated protein 1-like 1	LOSS	protein-coding

2455_T2	chr8	5.2E+07	5E+07	999	+	**1**	**12.5**	NA	Intergenic	L1Md_T|LINE|L1	-1E+06	NM_001205054	11593	Mm.334535	NM_001005847	ENSMUSG00000031521	**Aga**	AW060726	aspartylglucosaminidase	LOSS	protein-coding
1776_T2	chr18	6.5E+07	7E+07	999	+	**1**	**12.5**	NA	intron (NR_105742, intron 3 of 6)	intron (NR_105742, intron 3 of 6)	-6112	NM_001037294	225638	Mm.296528	NM_001037294	ENSMUSG00000032845	**Alpk2**	Gm549|Hak	alpha-kinase 2	LOSS	protein-coding
302_T12	chr14	3.4E+07	3E+07	71999	+	**1**	**12.5**	NA	intron (NM_172808, intron 13 of 16)	intron (NM_172808, intron 13 of 16)	16030	NM_172808	239029	Mm.477093	NM_172808	ENSMUSG00000047441	**Antxrl**	1700112N15Rik|4933430J11	anthrax toxin receptor-like	LOSS	protein-coding
243_T3	chr14	2.6E+07	3E+07	7999	+	**1**	**12.5**	NA	intron (NM_013469, intron 1 of 14)	B2_Mm2|SINE|B2	3345	NM_013469	11744	Mm.294083	NM_013469	ENSMUSG00000021866	**Anxa11**	A830099O17Rik|Anx11	annexin A11	LOSS	protein-coding
789_T12	chr18	1.5E+07	2E+07	5999	+	**1**	**12.5**	NA	Intergenic	L1Md_A|LINE|L1	33516	NM_001308647	11829	Mm.250786	NM_009700	ENSMUSG00000024411	**Aqp4**	WCH4	aquaporin 4	LOSS	protein-coding
38_T1	chr12	3470501	3E+06	3999	+	**1**	**12.5**	NA	intron (NM_172421, intron 5 of 10)	B3A|SINE|B2	45643	NM_172421	75302	Mm.120475	NM_172421	ENSMUSG00000037486	**Asxl2**	4930556B16Rik|mKIAA1685	additional sex combs like 2 (Drosophila)	LOSS	protein-coding
155_T4	chr14	1.4E+07	1E+07	999	+	**1**	**12.5**	NA	intron (NM_139227, intron 3 of 11)	intron (NM_139227, intron 3 of 11)	3509	NM_139227	246103	Mm.133625	NM_139227	ENSMUSG00000021738	**Atxn7**	A430107N12Rik|AI627028|Sca7|ataxin-7	ataxin 7	LOSS	protein-coding
615_T42	chr16	2.4E+07	2E+07	3999	+	**1**	**12.5**	NA	intron (NM_009744, intron 1 of 9)	CpG	5112	NM_009744	12053	Mm.347398	NM_009744	ENSMUSG00000022508	**Bcl6**	Bcl5	B cell leukemia/lymphoma 6	LOSS	protein-coding
1163_T12	chr18	3.2E+07	3E+07	24999	+	**1**	**12.5**	NA	Intergenic	(TA)n|Simple_repeat|Simple_repeat	-64217	NM_001083334	30948	Mm.4383	NM_009668	ENSMUSG00000024381	**Bin1**	ALP-1|Amphl|BRAMP-2|SH3P9	bridging integrator 1	LOSS	protein-coding
270_T2	chr14	2.9E+07	3E+07	927999	+	**1**	**12.5**	NA	Intergenic	Intergenic	-89636	NM_009785	12294	Mm.386754	NM_009785	ENSMUSG00000021991	**Cacna2d3**	Cacnad3|alapha2delta3	calcium channel, voltage-dependent, alpha2/delta subunit 3	LOSS	protein-coding
629_T12	chr16	6.8E+07	7E+07	12999	+	**1**	**12.5**	NA	Intergenic	Intergenic	-34092	NM_178721	239857	Mm.394300	NM_178721	ENSMUSG00000064115	**Cadm2**	2900078E11Rik|9330131D06|A830029E02Rik|Igdf4d|Igsf4d|NECL3|SynCAM 2|SynCAM2	cell adhesion molecule 2	LOSS	protein-coding
2167_T2	chr18	8.7E+07	9E+07	999	+	**1**	**12.5**	NA	Intergenic	L1Md_T|LINE|L1	-150110	NM_001302356	12405	Mm.70775	NM_172633	ENSMUSG00000024647	**Cbln2**	6330593N19Rik|A730004O05	cerebellin 2 precursor protein	LOSS	protein-coding
2168_T2	chr18	8.7E+07	9E+07	4999	+	**1**	**12.5**	NA	Intergenic	Intergenic	-131110	NM_001302356	12405	Mm.70775	NM_172633	ENSMUSG00000024647	**Cbln2**	6330593N19Rik|A730004O05	cerebellin 2 precursor protein	LOSS	protein-coding

2174_T12	chr18	8.7E+07	9E+07	18999	+	**1**	**12.5**	NA	Intergenic	L1Md_A|LINE|L1	71935	NM_172633	12405	Mm.70775	NM_172633	ENSMUSG00000024647	**Cbln2**	6330593N19Rik|A730004O05	cerebellin 2 precursor protein	LOSS	protein-coding
2175_T12	chr18	8.7E+07	9E+07	15999	+	**1**	**12.5**	NA	Intergenic	Lx7|LINE|L1	96435	NM_172633	12405	Mm.70775	NM_172633	ENSMUSG00000024647	**Cbln2**	6330593N19Rik|A730004O05	cerebellin 2 precursor protein	LOSS	protein-coding
2179_T2	chr18	8.7E+07	9E+07	1999	+	**1**	**12.5**	NA	Intergenic	MTC|LTR|ERVL-MaLR	211435	NM_172633	12405	Mm.70775	NM_172633	ENSMUSG00000024647	**Cbln2**	6330593N19Rik|A730004O05	cerebellin 2 precursor protein	LOSS	protein-coding
2183_T1	chr18	8.7E+07	9E+07	7999	+	**1**	**12.5**	NA	Intergenic	Intergenic	263435	NM_172633	12405	Mm.70775	NM_172633	ENSMUSG00000024647	**Cbln2**	6330593N19Rik|A730004O05	cerebellin 2 precursor protein	LOSS	protein-coding
2190_T2	chr18	8.7E+07	9E+07	999	+	**1**	**12.5**	NA	Intergenic	L1Md_A|LINE|L1	633935	NM_172633	12405	Mm.70775	NM_172633	ENSMUSG00000024647	**Cbln2**	6330593N19Rik|A730004O05	cerebellin 2 precursor protein	LOSS	protein-coding
2193_T2	chr18	8.8E+07	9E+07	2999	+	**1**	**12.5**	NA	Intergenic	(GA)n|Simple_repeat|Simple_repeat	911935	NM_172633	12405	Mm.70775	NM_172633	ENSMUSG00000024647	**Cbln2**	6330593N19Rik|A730004O05	cerebellin 2 precursor protein	LOSS	protein-coding
2196_T12	chr18	8.8E+07	9E+07	5999	+	**1**	**12.5**	NA	Intergenic	Intergenic	949435	NM_172633	12405	Mm.70775	NM_172633	ENSMUSG00000024647	**Cbln2**	6330593N19Rik|A730004O05	cerebellin 2 precursor protein	LOSS	protein-coding
2198_T3	chr18	8.8E+07	9E+07	6999	+	**1**	**12.5**	NA	Intergenic	Intergenic	1089935	NM_172633	12405	Mm.70775	NM_172633	ENSMUSG00000024647	**Cbln2**	6330593N19Rik|A730004O05	cerebellin 2 precursor protein	LOSS	protein-coding
953_T1	chr18	2.2E+07	2E+07	7999	+	**1**	**12.5**	NA	intron (NM_027616, intron 4 of 22)	intron (NM_027616, intron 4 of 22)	24896	NM_027616	70950	Mm.235716	NM_027616	ENSMUSG00000024306	**Ccdc178**	4921528I01Rik	coiled coil domain containing 178	LOSS	protein-coding
99_T12	chr13	8.5E+07	8E+07	14999	+	**1**	**12.5**	NA	Intergenic	Intergenic	-367477	NM_023243	66671	Mm.18474	NM_023243	ENSMUSG00000021548	**Ccnh**	6330408H09Rik|AI661354|AV102684|AW538719	cyclin H	LOSS	protein-coding
351_T12	chr14	3.7E+07	4E+07	78999	+	**1**	**12.5**	NA	Intergenic	L2|LINE|L2	292568	NM_028407	72972	Mm.27621	NM_027045	ENSMUSG00000058690	**Ccser2**	1700012P13Rik|2900054P12Rik|AI035535|AW108503|Fam190b|Gcap14	coiled-coil serine rich 2	LOSS	protein-coding
352_T12	chr14	3.7E+07	4E+07	184999	+	**1**	**12.5**	NA	Intergenic	Intergenic	151568	NM_028407	72972	Mm.27621	NM_027045	ENSMUSG00000058690	**Ccser2**	1700012P13Rik|2900054P12Rik|AI035535|AW108503|Fam190b|Gcap14	coiled-coil serine rich 2	LOSS	protein-coding
2231_T1	chr18	8.9E+07	9E+07	5999	+	**1**	**12.5**	NA	intron (NM_178687, intron 3 of 6)	CR1_Mam|LINE|CR1	22073	NM_001039149	225825	Mm.139293	NM_178687	ENSMUSG00000034028	**Cd226**	BC051526|DNAM-1|DNAM1|Pta1|TLiSA1	CD226 antigen	LOSS	protein-coding
607_T22	chr15	2.2E+07	2E+07	4999	+	**1**	**12.5**	NA	Intergenic	L1Md_A|LINE|L1	756548	NM_001008420	215654	Mm.300909	NM_001008420	ENSMUSG00000040452	**Cdh12**	Cdhb	cadherin 12	LOSS	protein-coding
2364_T22	chr5	3252501	3E+06	3999	+	**1**	**12.5**	NA	Intergenic	MMETn-int|LTR|ERVK	-89393	NM_009873	12571	Mm.31672	NM_009873	ENSMUSG00000040274	**Cdk6**	AI504062|Crk2	cyclin-dependent kinase 6	LOSS	protein-coding

482_T12	chr14	4.7E+07	5E+07	4999	+	**1**	**12.5**	NA	Intergenic	L1Md_T|LINE|L1	-12541	NM_028222	72391	Mm.272394	NM_028222	ENSMUSG00000037628	**Cdkn3**	2410006H10Rik|KAP	cyclin-dependent kinase inhibitor 3	LOSS	protein-coding
1014_T32	chr18	2.6E+07	3E+07	999	+	**1**	**12.5**	NA	Intergenic	L1Md_T|LINE|L1	-27017	NM_001174074	108013	Mm.266435	NM_133195	ENSMUSG00000024268	**Celf4**	A230070D14Rik|BRUNOL-4|Brul4|Brunol4|C130060B05Rik	CUGBP, Elav-like family member 4	LOSS	protein-coding
1024_T1	chr18	2.7E+07	3E+07	5999	+	**1**	**12.5**	NA	Intergenic	IAPEz-int|LTR|ERVK	-1E+06	NM_001174074	108013	Mm.266435	NM_133195	ENSMUSG00000024268	**Celf4**	A230070D14Rik|BRUNOL-4|Brul4|Brunol4|C130060B05Rik	CUGBP, Elav-like family member 4	LOSS	protein-coding
1027_T12	chr18	2.7E+07	3E+07	1999	+	**1**	**12.5**	NA	Intergenic	Lx9|LINE|L1	-1E+06	NM_001174074	108013	Mm.266435	NM_133195	ENSMUSG00000024268	**Celf4**	A230070D14Rik|BRUNOL-4|Brul4|Brunol4|C130060B05Rik	CUGBP, Elav-like family member 4	LOSS	protein-coding
9_T3	chr1	1.4E+08	1E+08	3999	+	**1**	**12.5**	NA	Intergenic	L1Md_F2|LINE|L1	-107278	NM_015780	50702	Mm.220362	NM_015780	ENSMUSG00000057037	**Cfhr1**	AI194696|CFHRB|Cfhl1	complement factor H-related 1	LOSS	protein-coding
809_T12	chr18	1.6E+07	2E+07	9999	+	**1**	**12.5**	NA	Intergenic	L1Md_A|LINE|L1	-227454	NM_199055	71367	Mm.329304	NM_199055	ENSMUSG00000047161	**Chst9**	5430438D01Rik	carbohydrate (N-acetylgalactosamine 4-0) sulfotransferase 9	LOSS	protein-coding
800_T12	chr18	1.6E+07	2E+07	999	+	**1**	**12.5**	NA	intron (NM_199055, intron 1 of 3)	B1F|SINE|Alu	16046	NM_199055	71367	Mm.329304	NM_199055	ENSMUSG00000047161	**Chst9**	5430438D01Rik	carbohydrate (N-acetylgalactosamine 4-0) sulfotransferase 9	LOSS	protein-coding
802_T12	chr18	1.6E+07	2E+07	4999	+	**1**	**12.5**	NA	intron (NM_199055, intron 1 of 3)	L1Md_T|LINE|L1	9046	NM_199055	71367	Mm.329304	NM_199055	ENSMUSG00000047161	**Chst9**	5430438D01Rik	carbohydrate (N-acetylgalactosamine 4-0) sulfotransferase 9	LOSS	protein-coding
2390_T4	chr6	1.1E+08	1E+08	4999	+	**1**	**12.5**	NA	intron (NM_001109751, intron 3 of 18)	intron (NM_001109751, intron 3 of 18)	30235	NM_001109751	269784	Mm.321683	NM_173004	ENSMUSG00000064293	**Cntn4**	9630050B05|Axcam|BIG-2	contactin 4	LOSS	protein-coding
229_T3	chr14	2.2E+07	2E+07	5999	+	**1**	**12.5**	NA	Intergenic	L1Md_T|LINE|L1	-32590	NM_026965	69156	Mm.11827	NM_026965	ENSMUSG00000021773	**Comtd1**	1810030M08Rik|MT773	catechol-O-methyltransferase domain containing 1	LOSS	protein-coding
5_T4	chr1	6.8E+07	7E+07	3999	+	**1**	**12.5**	NA	Intergenic	MMERVK10C-int|LTR|ERVK	788473	NM_001080809	227231	Mm.343942	NM_001080809	ENSMUSG00000025991	**Cps1**	4732433M03Rik|CPS|D1Ucla3	carbamoyl-phosphate synthetase 1	LOSS	protein-coding
625_T1	chr16	6.4E+07	6E+07	6999	+	**1**	**12.5**	NA	Intergenic	L1_Mur2|LINE|L1	73134	NM_173861	224291	Mm.246580	NM_173861	ENSMUSG00000068167	**Csnka2ip**	Ckt2	casein kinase 2, alpha prime interacting protein	LOSS	protein-coding
574_T12	chr14	5.4E+07	5E+07	93999	+	**1**	**12.5**	NA	Intergenic	ORR1A3|LTR|ERVL-MaLR	634429	NM_001113358	13135	Mm.319038	NM_010015	ENSMUSG00000022174	**Dad1**	AI323713	defender against cell death 1	LOSS	protein-coding
575_T12	chr14	5.4E+07	5E+07	261999	+	**1**	**12.5**	NA	Intergenic	ORR1F|LTR|ERVL-MaLR	455429	NM_001113358	13135	Mm.319038	NM_010015	ENSMUSG00000022174	**Dad1**	AI323713	defender against cell death 1	LOSS	protein-coding

576_T12	chr14	5.4E+07	5E+07	193999	+	**1**	**12.5**	NA	Intergenic	MLT1E1A-int|LTR|ERVL-MaLR	221429	NM_001113358	13135	Mm.319038	NM_010015	ENSMUSG00000022174	**Dad1**	AI323713	defender against cell death 1	LOSS	protein-coding
1921_T1	chr18	7.2E+07	7E+07	71999	+	**1**	**12.5**	NA	Intergenic	RLTR11A2|LTR|ERVK	-119431	NM_007831	13176	Mm.167882	NM_007831	ENSMUSG00000060534	**Dcc**	C030036D22Rik|Igdcc1	deleted in colorectal carcinoma	LOSS	protein-coding
1932_T1	chr18	7.3E+07	7E+07	999	+	**1**	**12.5**	NA	Intergenic	Intergenic	-452931	NM_007831	13176	Mm.167882	NM_007831	ENSMUSG00000060534	**Dcc**	C030036D22Rik|Igdcc1	deleted in colorectal carcinoma	LOSS	protein-coding
1915_T12	chr18	7.2E+07	7E+07	15999	+	**1**	**12.5**	NA	intron (NM_007831, intron 1 of 28)	intron (NM_007831, intron 1 of 28)	57569	NM_007831	13176	Mm.167882	NM_007831	ENSMUSG00000060534	**Dcc**	C030036D22Rik|Igdcc1	deleted in colorectal carcinoma	LOSS	protein-coding
1891_T1	chr18	7.2E+07	7E+07	999	+	**1**	**12.5**	NA	intron (NM_007831, intron 10 of 28)	intron (NM_007831, intron 10 of 28)	802069	NM_007831	13176	Mm.167882	NM_007831	ENSMUSG00000060534	**Dcc**	C030036D22Rik|Igdcc1	deleted in colorectal carcinoma	LOSS	protein-coding
1886_T32	chr18	7.1E+07	7E+07	999	+	**1**	**12.5**	NA	intron (NM_007831, intron 12 of 28)	intron (NM_007831, intron 12 of 28)	882069	NM_007831	13176	Mm.167882	NM_007831	ENSMUSG00000060534	**Dcc**	C030036D22Rik|Igdcc1	deleted in colorectal carcinoma	LOSS	protein-coding
1898_T22	chr18	7.2E+07	7E+07	7999	+	**1**	**12.5**	NA	intron (NM_007831, intron 7 of 28)	Lx9|LINE|L1	678569	NM_007831	13176	Mm.167882	NM_007831	ENSMUSG00000060534	**Dcc**	C030036D22Rik|Igdcc1	deleted in colorectal carcinoma	LOSS	protein-coding
2362_T12	chr4	1.4E+08	1E+08	4999	+	**1**	**12.5**	NA	Intergenic	MMETn-int|LTR|ERVK	-38827	NM_001172424	20148	Mm.14063	NM_011303	ENSMUSG00000066026	**Dhrs3**	Rsdr1|retSDR1	dehydrogenase/reductase (SDR family) member 3	LOSS	protein-coding
39_T3	chr12	4102501	4E+06	3999	+	**1**	**12.5**	NA	intron (NM_153082, intron 6 of 6)	intron (NM_153082, intron 6 of 6)	21926	NM_153082	217378	Mm.271656	NM_153082	ENSMUSG00000020657	**Dnajc27**	AI639580|C330021A05Rik|Rabj|Rbj	DnaJ heat shock protein family (Hsp40) member C27	LOSS	protein-coding
2248_T2	chr18	9E+07	9E+07	10999	+	**1**	**12.5**	NA	Intergenic	Lx6|LINE|L1	-76864	NM_001039173	623279	Mm.447164	NM_001039173	ENSMUSG00000073514	**Dok6**	Dok-6	docking protein 6	LOSS	protein-coding
2243_T1	chr18	9E+07	9E+07	999	+	**1**	**12.5**	NA	intron (NM_001039173, intron 1 of 7)	intron (NM_001039173, intron 1 of 7)	65136	NM_001039173	623279	Mm.447164	NM_001039173	ENSMUSG00000073514	**Dok6**	Dok-6	docking protein 6	LOSS	protein-coding
618_T3	chr16	4.8E+07	5E+07	3999	+	**1**	**12.5**	NA	Intergenic	B4A|SINE|B4	-61235	NM_028610	73693	Mm.35597	NM_028610	ENSMUSG00000058550	**Dppa4**	2410091M23Rik|C76608|ECAT15-1	developmental pluripotency associated 4	LOSS	protein-coding
1345_T12	chr18	4.3E+07	4E+07	999	+	**1**	**12.5**	NA	Intergenic	ORR1E|LTR|ERVL-MaLR	-39714	NM_001291455	22240	Mm.8180	NM_009468	ENSMUSG00000024501	**Dpysl3**	CRMP-4|DRP-3|TUC4|ULIP-1|Ulip|Ulip1	dihydropyrimidinase-like 3	LOSS	protein-coding

885_T1	chr18	1.9E+07	2E+07	29999	+	**1**	**12.5**	NA	Intergenic	Lx8|LINE|L1	816597	NM_007882	13507	Mm.89935	NM_007882	ENSMUSG00000059898	**Dsc3**	5430426I24Rik	desmocollin 3	LOSS	protein-coding
887_T1	chr18	1.9E+07	2E+07	9999	+	**1**	**12.5**	NA	Intergenic	RMER16-int|LTR|ERVK	785597	NM_007882	13507	Mm.89935	NM_007882	ENSMUSG00000059898	**Dsc3**	5430426I24Rik	desmocollin 3	LOSS	protein-coding
906_T12	chr18	2E+07	2E+07	999	+	**1**	**12.5**	NA	Intergenic	AT_rich|Low_complexity|Low_complexity	431097	NM_007882	13507	Mm.89935	NM_007882	ENSMUSG00000059898	**Dsc3**	5430426I24Rik	desmocollin 3	LOSS	protein-coding
7_T22	chr1	1.1E+08	1E+08	3999	+	**1**	**12.5**	NA	Intergenic	L1Md_T|LINE|L1	430418	NM_001081316	319901	Mm.103468	NM_001081316	ENSMUSG00000038702	**Dsel**	9330132E09Rik|DS-epi2	dermatan sulfate epimerase-like	LOSS	protein-coding
8_T22	chr1	1.1E+08	1E+08	6999	+	**1**	**12.5**	NA	Intergenic	L1Md_A|LINE|L1	401918	NM_001081316	319901	Mm.103468	NM_001081316	ENSMUSG00000038702	**Dsel**	9330132E09Rik|DS-epi2	dermatan sulfate epimerase-like	LOSS	protein-coding
224_T3	chr14	2.2E+07	2E+07	149999	+	**1**	**12.5**	NA	TTS (NM_017479)	TTS (NM_017479)	42076	NM_001013826	435391	Mm.295860	NM_001013826	ENSMUSG00000063821	**Dupd1**	EG435391	dual specificity phosphatase and pro isomerase domain containing 1	LOSS	protein-coding
404_T12	chr14	4.1E+07	4E+07	7999	+	**1**	**12.5**	NA	intron (NM_027717, intron 3 of 3)	IAPEY4_I-int|LTR|ERVK	10574	NM_027717	71200	Mm.443973	NM_027717	ENSMUSG00000021791	**Dydc2**	4933428D01Rik	DPY30 domain containing 2	LOSS	protein-coding
1853_T1	chr18	7E+07	7E+07	11999	+	**1**	**12.5**	NA	Intergenic	L1Md_F2|LINE|L1	-84916	NM_029346	75577	Mm.58214	NM_029346	ENSMUSG00000024512	**Dynap**	2310002L13Rik	dynactin associated protein	LOSS	protein-coding
644_T2	chr16	9.5E+07	9E+07	9999	+	**1**	**12.5**	NA	promoter-TSS (NM_001113389)	promoter-TSS (NM_001113389)	294	NM_007890	13548	Mm.310973	NM_007890	ENSMUSG00000022897	**Dyrk1a**	2310043O08Rik|D16Ertd272e|D16Ertd493e|Dyrk|ENSMUSG00000074897|Gm10783|Mnbh|Mp86|mmb	dual-specificity tyrosine-(Y)-phosphorylation regulated kinase 1a	LOSS	protein-coding
101_T2	chr13	8.7E+07	9E+07	999	+	**1**	**12.5**	NA	Intergenic	L1Md_T|LINE|L1	-1E+06	NM_010103	13612	Mm.125580	NM_010103	ENSMUSG00000034488	**Edil3**	Del-1|Del1	EGF-like repeats and discoidin I-like domains 3	LOSS	protein-coding
624_T2	chr16	6.1E+07	6E+07	5999	+	**1**	**12.5**	NA	Intergenic	RLTR22_Mur|LTR|ERVK	-603969	NM_007938	13840	Mm.455790	NM_007938	ENSMUSG00000055540	**Epha6**	Ehk2|Hek12|m-ehk2	Eph receptor A6	LOSS	protein-coding
2334_T3	chr4	2.8E+07	3E+07	3999	+	**1**	**12.5**	NA	Intergenic	L1VL4|LINE|L1	-952631	NM_001290434	13841	Mm.257266	NM_010141	ENSMUSG00000028289	**Epha7**	Cek11|Ebk|Ehk3|Hek11|Mdk1	Eph receptor A7	LOSS	protein-coding
646_T1	chr16	9.6E+07	1E+08	4999	+	**1**	**12.5**	NA	intron (NM_133659, intron 2 of 10)	intron (NM_133659, intron 2 of 10)	22365	NM_001302179	13876	Mm.164531	NM_133659	ENSMUSG00000040732	**Erg**	D030036I24Rik	avian erythroblastosis virus E-26 (v-ets) oncogene related	LOSS	protein-coding
14_T2	chr1	1.9E+08	2E+08	3999	+	**1**	**12.5**	NA	intron (NM_001243792, intron 1 of 7)	intron (NM_001243792, intron 1 of 7)	44494	NM_001243792	26381	Mm.89989	NM_011935	ENSMUSG00000026610	**Esrrg**	ERR3|Errg|NR3B3|mKIAA0832	estrogen-related receptor gamma	LOSS	protein-coding

2330_T12	chr3	1.2E+08	1E+08	3999	+	**1**	**12.5**	NA	Intergenic	IAPEz-int|LTR|ERVK	25963	NM_010171	14066	Mm.273188	NM_010171	ENSMUSG00000028128	**F3**	AA409063|CD142|Cf-3|Cf3|TF	coagulation factor III	LOSS	protein-coding
609_T3	chr15	7.1E+07	7E+07	999	+	**1**	**12.5**	NA	Intergenic	L1Md_A|LINE|L1	827838	NM_177819	70363	Mm.126450	NM_177819	ENSMUSG00000036800	**Fam135b**	1700010C24Rik|A830008O07Rik	family with sequence similarity 135, member B	LOSS	protein-coding
2433_T3	chr7	9.4E+07	9E+07	3999	+	**1**	**12.5**	NA	Intergenic	L1Md_T|LINE|L1	893621	NM_021427	58238	Mm.113787	NM_021427	ENSMUSG00000051515	**Fam181b**	A830059I20Rik|AB041569	family with sequence similarity 181, member B	LOSS	protein-coding
2434_T22	chr7	9.4E+07	9E+07	3999	+	**1**	**12.5**	NA	Intergenic	L1Md_F2|LINE|L1	1097621	NM_021427	58238	Mm.113787	NM_021427	ENSMUSG00000051515	**Fam181b**	A830059I20Rik|AB041569	family with sequence similarity 181, member B	LOSS	protein-coding
27_T3	chr11	2.6E+07	3E+07	999	+	**1**	**12.5**	NA	intron (NM_001277273, intron 8 of 13)	L1Md_T|LINE|L1	76916	NR_102382	67030	Mm.18875	NM_025923	ENSMUSG00000004018	**Fancl**	2010322C19Rik|AW554273|B230118H11Rik|Phf9|Pog|gcd	Fanconi anemia, complementation group L	LOSS	protein-coding
1663_T12	chr18	5.8E+07	6E+07	19999	+	**1**	**12.5**	NA	Intergenic	L1Md_F2|LINE|L1	-122574	NM_010181	14119	Mm.20271	NM_010181	ENSMUSG00000024598	**Fbn2**	BC063774|Fib-2|mKIAA4226|sne|sy	fibrillin 2	LOSS	protein-coding
141_T2	chr14	1.2E+07	1E+07	141999	+	**1**	**12.5**	NA	exon (NM_080433, exon 5 of 5)	exon (NM_080433, exon 5 of 5)	3365	NM_080433	54713	Mm.34644	NM_080433	ENSMUSG00000021743	**Fezf2**	AI451466|AI852056|Fez|Fezl|Zfp312	Fez family zinc finger 2	LOSS	protein-coding
142_T42	chr14	1.3E+07	1E+07	8999	+	**1**	**12.5**	NA	intron (NM_012061, intron 11 of 28)	B4A|SINE|B4	-166135	NM_080433	54713	Mm.34644	NM_080433	ENSMUSG00000021743	**Fezf2**	AI451466|AI852056|Fez|Fezl|Zfp312	Fez family zinc finger 2	LOSS	protein-coding
137_T2	chr14	1E+07	1E+07	111999	+	**1**	**12.5**	NA	intron (NM_001308285, intron 4 of 9)	intron (NM_001308285, intron 4 of 9)	-99042	NM_001308286	14198	Mm.397619	NM_010210	ENSMUSG00000060579	**Fhit**	AW045638|Fra14A2	fragile histidine triad gene	LOSS	protein-coding
135_T2	chr14	1E+07	1E+07	999	+	**1**	**12.5**	NA	intron (NM_001308286, intron 2 of 6)	MuRRS-int|LTR|ERV1	179458	NM_001308286	14198	Mm.397619	NM_010210	ENSMUSG00000060579	**Fhit**	AW045638|Fra14A2	fragile histidine triad gene	LOSS	protein-coding
120_T22	chr14	7707501	8E+06	20999	+	**1**	**12.5**	NA	Intergenic	Intergenic	-99957	NM_001081427	286940	Mm.489652	NM_134080	ENSMUSG00000025278	**Flnb**	AL024016|Fln-b	filamin, beta	LOSS	protein-coding
121_T3	chr14	7741501	8E+06	13999	+	**1**	**12.5**	NA	Intergenic	Intergenic	-69457	NM_001081427	286940	Mm.489652	NM_134080	ENSMUSG00000025278	**Flnb**	AL024016|Fln-b	filamin, beta	LOSS	protein-coding
2388_T2	chr6	1E+08	1E+08	7999	+	**1**	**12.5**	NA	Intergenic	Intergenic	-84155	NM_053202	108655	Mm.234965	NM_053202	ENSMUSG00000030067	**Foxp1**	3110052D19Rik|4932443N09Rik|AI461938|AW494214	forkhead box P1	LOSS	protein-coding
1558_T12	chr18	5.2E+07	5E+07	999	+	**1**	**12.5**	NA	Intergenic	L1Md_F2|LINE|L1	55464	NM_026286	67634	Mm.179144	NM_026286	ENSMUSG00000024510	**Ftmt**	4930447C24Rik|FerH|Fth3|MtF	ferritin mitochondrial	LOSS	protein-coding

2428_T22	chr7	5.7E+07	6E+07	3999	+	**1**	**12.5**	NA	intron (NM_008074, intron 5 of 9)	intron (NM_008074, intron 5 of 9)	534371	NM_008074	14407	Mm.434343	NM_008074	ENSMUSG00000055026	**Gabrg3**	B230362M20Rik|Gabrg-3	gamma-aminobutyric acid (GABA) A receptor, subunit gamma 3	LOSS	protein-coding
72_T12	chr12	9.8E+07	1E+08	6999	+	**1**	**12.5**	NA	Intergenic	L1Md_F2|LINE|L1	491459	NM_008079	14420	Mm.5120	NM_008079	ENSMUSG00000021003	**Galc**	2310068B06Rik|A930008M05Rik|AW212969|AW413532|Gacy|twi|twitcher	galactosylceramidase	LOSS	protein-coding
356_T12	chr14	3.7E+07	4E+07	17999	+	**1**	**12.5**	NA	Intergenic	Lx10|LINE|L1	-110178	NM_078478	66092	Mm.182912	NM_078478	ENSMUSG00000041028	**Ghitm**	1010001P14Rik|C77840|MICS1|PTD010|Tmbim5	growth hormone inducible transmembrane protein	LOSS	protein-coding
357_T12	chr14	3.7E+07	4E+07	247999	+	**1**	**12.5**	NA	Intergenic	L1_Mur2|LINE|L1	-250178	NM_078478	66092	Mm.182912	NM_078478	ENSMUSG00000041028	**Ghitm**	1010001P14Rik|C77840|MICS1|PTD010|Tmbim5	growth hormone inducible transmembrane protein	LOSS	protein-coding
359_T12	chr14	3.8E+07	4E+07	12999	+	**1**	**12.5**	NA	Intergenic	RMER15-int|LTR|ERVL	-463678	NM_078478	66092	Mm.182912	NM_078478	ENSMUSG00000041028	**Ghitm**	1010001P14Rik|C77840|MICS1|PTD010|Tmbim5	growth hormone inducible transmembrane protein	LOSS	protein-coding
364_T2	chr14	3.8E+07	4E+07	999	+	**1**	**12.5**	NA	Intergenic	Intergenic	-631678	NM_078478	66092	Mm.182912	NM_078478	ENSMUSG00000041028	**Ghitm**	1010001P14Rik|C77840|MICS1|PTD010|Tmbim5	growth hormone inducible transmembrane protein	LOSS	protein-coding
367_T12	chr14	3.8E+07	4E+07	48999	+	**1**	**12.5**	NA	Intergenic	L1Md_F3|LINE|L1	-732678	NM_078478	66092	Mm.182912	NM_078478	ENSMUSG00000041028	**Ghitm**	1010001P14Rik|C77840|MICS1|PTD010|Tmbim5	growth hormone inducible transmembrane protein	LOSS	protein-coding
369_T12	chr14	3.8E+07	4E+07	17999	+	**1**	**12.5**	NA	Intergenic	Intergenic	-793178	NM_078478	66092	Mm.182912	NM_078478	ENSMUSG00000041028	**Ghitm**	1010001P14Rik|C77840|MICS1|PTD010|Tmbim5	growth hormone inducible transmembrane protein	LOSS	protein-coding
370_T12	chr14	3.8E+07	4E+07	7999	+	**1**	**12.5**	NA	Intergenic	RMER5|LTR|ERV1	-810178	NM_078478	66092	Mm.182912	NM_078478	ENSMUSG00000041028	**Ghitm**	1010001P14Rik|C77840|MICS1|PTD010|Tmbim5	growth hormone inducible transmembrane protein	LOSS	protein-coding
371_T12	chr14	3.8E+07	4E+07	999	+	**1**	**12.5**	NA	Intergenic	MIR|SINE|MIR	-819678	NM_078478	66092	Mm.182912	NM_078478	ENSMUSG00000041028	**Ghitm**	1010001P14Rik|C77840|MICS1|PTD010|Tmbim5	growth hormone inducible transmembrane protein	LOSS	protein-coding
329_T12	chr14	3.5E+07	3E+07	18999	+	**1**	**12.5**	NA	Intergenic	L1_Mur2|LINE|L1	-30136	NM_008166	14803	Mm.121569	NM_008166	ENSMUSG00000041078	**Grid1**	GluD1|GluRdelta1	glutamate receptor, ionotropic, delta 1	LOSS	protein-coding
2383_T1	chr6	6.4E+07	6E+07	5999	+	**1**	**12.5**	NA	intron (NM_008167, intron 2 of 15)	L1_Mus2|LINE|L1	303643	NM_008167	14804	Mm.439651	NM_008167	ENSMUSG00000071424	**Grid2**	B230104L07Rik|GluD2|GluRdelta2|Lc|Lc<J>|MMS10-AC|Ms10ac|cpr|ho|nmf408|tpr	glutamate receptor, ionotropic, delta 2	LOSS	protein-coding
83_T12	chr12	1E+08	1E+08	999	+	**1**	**12.5**	NA	Intergenic	Intergenic	-5764	NM_010351	14836	Mm.129	NM_010351	ENSMUSG00000021095	**Gsc**	-	goosecoid homeobox	LOSS	protein-coding

<table>
<tr><th></th><th></th><th></th><th></th><th></th><th></th><th></th><th></th><th></th><th></th><th></th><th></th><th></th><th></th><th></th><th></th><th></th><th></th><th></th><th></th><th></th><th></th></tr>
<tr><td>1165_T12</td><td>chr18</td><td>3.3E+07</td><td>3E+07</td><td>8999</td><td>+</td><td>1</td><td>12.5</td><td>NA</td><td>intron (NR_045381, intron 4 of 4)</td><td>L1Md_T|LINE|L1</td><td>-123966</td><td>NM_001048207</td><td>71683</td><td>Mm.292145</td><td>NM_027863</td><td>ENSMUSG00000090523</td><td>Gypc</td><td>0610037F22Rik|Cd236r</td><td>glycophorin C</td><td>LOSS</td><td>protein-coding</td></tr>
<tr><td>626_T1</td><td>chr16</td><td>6.5E+07</td><td>7E+07</td><td>999</td><td>+</td><td>1</td><td>12.5</td><td>NA</td><td>intron (NM_008310, intron 2 of 2)</td><td>L1MC|LINE|L1</td><td>69784</td><td>NM_008310</td><td>15557</td><td>Mm.5040</td><td>NM_008310</td><td>ENSMUSG00000050783</td><td>Htr1f</td><td>Htr1eb</td><td>5-hydroxytryptamine (serotonin) receptor 1F</td><td>LOSS</td><td>protein-coding</td></tr>
<tr><td>628_T1</td><td>chr16</td><td>6.5E+07</td><td>7E+07</td><td>6999</td><td>+</td><td>1</td><td>12.5</td><td>NA</td><td>intron (NM_008310, intron 2 of 2)</td><td>B1_Mur1|SINE|Alu</td><td>54784</td><td>NM_008310</td><td>15557</td><td>Mm.5040</td><td>NM_008310</td><td>ENSMUSG00000050783</td><td>Htr1f</td><td>Htr1eb</td><td>5-hydroxytryptamine (serotonin) receptor 1F</td><td>LOSS</td><td>protein-coding</td></tr>
<tr><td>2442_T42</td><td>chr7</td><td>1.4E+08</td><td>1E+08</td><td>3999</td><td>+</td><td>1</td><td>12.5</td><td>NA</td><td>intron (NR_002855, intron 1 of 3)</td><td>intron (NR_002855, intron 1 of 3)</td><td>-2229</td><td>NM_001122736</td><td>16002</td><td>Mm.3862</td><td>NM_010514</td><td>ENSMUSG00000048583</td><td>Igf2</td><td>AL033362|Igf-2|Igf-II|M6pr|Mpr|Peg2</td><td>insulin-like growth factor 2</td><td>LOSS</td><td>protein-coding</td></tr>
<tr><td>1201_T32</td><td>chr18</td><td>3.6E+07</td><td>4E+07</td><td>999</td><td>+</td><td>1</td><td>12.5</td><td>NA</td><td>Intergenic</td><td>Intergenic</td><td>-3101</td><td>NM_001267796</td><td>109169</td><td>Mm.426423</td><td>NM_001267796</td><td>ENSMUSG00000110185</td><td>Igip</td><td>6330403M23Rik</td><td>IgA inducing protein</td><td>LOSS</td><td>protein-coding</td></tr>
<tr><td>220_T2</td><td>chr14</td><td>2.1E+07</td><td>2E+07</td><td>999</td><td>+</td><td>1</td><td>12.5</td><td>NA</td><td>intron (NM_134079, intron 7 of 10)</td><td>intron (NM_134079, intron 7 of 10)</td><td>-139770</td><td>NM_001205241</td><td>54169</td><td>Mm.248967</td><td>NM_017479</td><td>ENSMUSG00000021767</td><td>Kat6b</td><td>AI507552|B130044K16Rik|MYST-4|Morf|Myst4|mKIAA0383|qkf|querkopf</td><td>K(lysine) acetyltransferase 6B</td><td>LOSS</td><td>protein-coding</td></tr>
<tr><td>645_T1</td><td>chr16</td><td>9.5E+07</td><td>1E+08</td><td>6999</td><td>+</td><td>1</td><td>12.5</td><td>NA</td><td>Intergenic</td><td>L1MB3|LINE|L1</td><td>-94304</td><td>NM_010606</td><td>16522</td><td>Mm.328720</td><td>NM_010606</td><td>ENSMUSG00000043301</td><td>Kcnj6</td><td>BIR1|GIRK2|KATP2|KCNJ7|Kir3.2|weaver|wv</td><td>potassium inwardly-rectifying channel, subfamily J, member 6</td><td>LOSS</td><td>protein-coding</td></tr>
<tr><td>1384_T32</td><td>chr18</td><td>4.5E+07</td><td>5E+07</td><td>999</td><td>+</td><td>1</td><td>12.5</td><td>NA</td><td>intron (NM_001312905, intron 1 of 10)</td><td>IAPEz-int|LTR|ERVK</td><td>26140</td><td>NM_001312905</td><td>140492</td><td>Mm.411614</td><td>NM_080465</td><td>ENSMUSG00000054477</td><td>Kcnn2</td><td>KCa2.2|SK2|SKCA2|bc|fri</td><td>potassium intermediate/small conductance calcium-activated channel, subfamily N, member 2</td><td>LOSS</td><td>protein-coding</td></tr>
<tr><td>1391_T3</td><td>chr18</td><td>4.6E+07</td><td>5E+07</td><td>999</td><td>+</td><td>1</td><td>12.5</td><td>NA</td><td>intron (NM_001312905, intron 2 of 10)</td><td>MT2_Mm|LTR|ERVL</td><td>-53154</td><td>NM_080465</td><td>140492</td><td>Mm.411614</td><td>NM_080465</td><td>ENSMUSG00000054477</td><td>Kcnn2</td><td>KCa2.2|SK2|SKCA2|bc|fri</td><td>potassium intermediate/small conductance calcium-activated channel, subfamily N, member 2</td><td>LOSS</td><td>protein-coding</td></tr>
<tr><td>1392_T1</td><td>chr18</td><td>4.6E+07</td><td>5E+07</td><td>999</td><td>+</td><td>1</td><td>12.5</td><td>NA</td><td>intron (NM_001312905, intron 2 of 10)</td><td>intron (NM_001312905, intron 2 of 10)</td><td>-46154</td><td>NM_080465</td><td>140492</td><td>Mm.411614</td><td>NM_080465</td><td>ENSMUSG00000054477</td><td>Kcnn2</td><td>KCa2.2|SK2|SKCA2|bc|fri</td><td>potassium intermediate/small conductance calcium-activated channel, subfamily N, member 2</td><td>LOSS</td><td>protein-coding</td></tr>
<tr><td>1394_T1</td><td>chr18</td><td>4.6E+07</td><td>5E+07</td><td>4999</td><td>+</td><td>1</td><td>12.5</td><td>NA</td><td>intron (NM_001312905, intron 2 of 10)</td><td>L1Md_T|LINE|L1</td><td>-20154</td><td>NM_080465</td><td>140492</td><td>Mm.411614</td><td>NM_080465</td><td>ENSMUSG00000054477</td><td>Kcnn2</td><td>KCa2.2|SK2|SKCA2|bc|fri</td><td>potassium intermediate/small conductance calcium-activated channel, subfamily N, member 2</td><td>LOSS</td><td>protein-coding</td></tr>
</table>

1273_T1	chr18	4.1E+07	4E+07	6999	+	**1**	**12.5**	NA	Intergenic	Intergenic	298639	NM_026135	383348	Mm.76366	NM_026135	ENSMUSG00000051401	**Kctd16**	4930434H12Rik|Gm1267	potassium channel tetramerisation domain containing 16	LOSS	protein-coding
1281_T12	chr18	4.1E+07	4E+07	999	+	**1**	**12.5**	NA	Intergenic	Intergenic	349639	NM_026135	383348	Mm.76366	NM_026135	ENSMUSG00000051401	**Kctd16**	4930434H12Rik|Gm1267	potassium channel tetramerisation domain containing 16	LOSS	protein-coding
1284_T12	chr18	4.1E+07	4E+07	6999	+	**1**	**12.5**	NA	Intergenic	L1Md_T|LINE|L1	392639	NM_026135	383348	Mm.76366	NM_026135	ENSMUSG00000051401	**Kctd16**	4930434H12Rik|Gm1267	potassium channel tetramerisation domain containing 16	LOSS	protein-coding
1288_T12	chr18	4.1E+07	4E+07	999	+	**1**	**12.5**	NA	Intergenic	Intergenic	631639	NM_026135	383348	Mm.76366	NM_026135	ENSMUSG00000051401	**Kctd16**	4930434H12Rik|Gm1267	potassium channel tetramerisation domain containing 16	LOSS	protein-coding
605_T2	chr14	9.7E+07	1E+08	999	+	**1**	**12.5**	NA	Intergenic	MER72B|LTR|ERV1	-637966	NM_053105	93688	Mm.308735	NM_053105	ENSMUSG00000022076	**Klhl1**	mKIAA1490	kelch-like 1	LOSS	protein-coding
604_T22	chr14	9.6E+07	1E+08	999	+	**1**	**12.5**	NA	intron (NM_053105, intron 1 of 10)	L1Md_T|LINE|L1	109034	NM_053105	93688	Mm.308735	NM_053105	ENSMUSG00000022076	**Klhl1**	mKIAA1490	kelch-like 1	LOSS	protein-coding
518_T12	chr14	5.1E+07	5E+07	3999	+	**1**	**12.5**	NA	Intergenic	IAPEz-int|LTR|ERVK	10755	NM_001166651	546611	Mm.390692	NM_001166651	ENSMUSG00000090799	**Klhl33**	EG546611	kelch-like 33	LOSS	protein-coding
2398_T2	chr6	1.3E+08	1E+08	1999	+	**1**	**12.5**	NA	promoter-TSS (NM_001098669)	promoter-TSS (NM_001098669)	56	NM_010653	16642	Mm.347927	NM_010653	ENSMUSG00000052736	**Klrc2**	NKG2C	killer cell lectin-like receptor subfamily C, member 2	LOSS	protein-coding
2395_T2	chr6	1.3E+08	1E+08	2999	+	**1**	**12.5**	NA	Intergenic	Intergenic	-5712	NM_021378	58179	Mm.358684	NM_021378	ENSMUSG00000033027	**Klrc3**	Klrc2|Nkg2e	killer cell lectin-like receptor subfamily C, member 3	LOSS	protein-coding
2380_T3	chr6	4.8E+07	5E+07	3999	+	**1**	**12.5**	NA	Intergenic	tRNA-Cys-TGY|tRNA|tRNA	-90086	NM_133922	77827	Mm.107646	NM_133922	ENSMUSG00000042810	**Krba1**	A930040G15Rik|AI448780	KRAB-A domain containing 1	LOSS	protein-coding
18_T4	chr10	2.8E+07	3E+07	999	+	**1**	**12.5**	NA	Intergenic	L1Md_A|LINE|L1	-202058	NM_008481	16773	Mm.256087	NM_008481	ENSMUSG00000019899	**Lama2**	5830440B04|dy|mKIAA4087|mer|merosin	laminin, alpha 2	LOSS	protein-coding
760_T12	chr18	1.2E+07	1E+07	1999	+	**1**	**12.5**	NA	intron (NM_010680, intron 1 of 74)	intron (NM_010680, intron 1 of 74)	24476	NM_010680	16774	Mm.42012	NM_010680	ENSMUSG00000024421	**Lama3**	Lama3B|[a]3B	laminin, alpha 3	LOSS	protein-coding
2003_T1	chr18	7.7E+07	8E+07	999	+	**1**	**12.5**	NA	Intergenic	MERVL-int|LTR|ERVL	-16958	NM_172834	240411	Mm.380247	NM_172834	ENSMUSG00000032818	**Loxhd1**	1700096C21Rik|4932417K07|sba	lipoxygenase homology domains 1	LOSS	protein-coding
296_T1	chr14	3.3E+07	3E+07	4999	+	**1**	**12.5**	NA	intron (NM_001146022, intron 36 of 58)	Charlie1b|DNA|hAT-Charlie	58618	NM_026253	67580	Mm.46115	NM_026253	ENSMUSG00000041673	**Lrrc18**	4930442L21Rik|MTLR1	leucine rich repeat containing 18	LOSS	protein-coding
160_T42	chr14	1.5E+07	2E+07	31999	+	**1**	**12.5**	NA	Intergenic	Intergenic	-48513	NM_146052	218763	Mm.138039	NM_146052	ENSMUSG00000045201	**Lrrc3b**	BC019794|LRP15	leucine rich repeat containing 3B	LOSS	protein-coding

161_T32	chr14	1.6E+07	2E+07	63999	+	**1**	**12.5**	NA	Intergenic	Intergenic	-202513	NM_146052	218763	Mm.138039	NM_146052	ENSMUSG00000045201	**Lrrc3b**	BC019794|LRP15	leucine rich repeat containing 3B	LOSS	protein-coding
162_T3	chr14	1.6E+07	2E+07	8999	+	**1**	**12.5**	NA	Intergenic	L1Md_F3|LINE|L1	-391013	NM_146052	218763	Mm.138039	NM_146052	ENSMUSG00000045201	**Lrrc3b**	BC019794|LRP15	leucine rich repeat containing 3B	LOSS	protein-coding
269_T2	chr14	2.9E+07	3E+07	5999	+	**1**	**12.5**	NA	intron (NM_009785, intron 11 of 37)	intron (NM_009785, intron 11 of 37)	215292	NM_176920	319476	Mm.95780	NM_176920	ENSMUSG00000045776	**Lrtm1**	A930016D02Rik	leucine-rich repeats and transmembrane domains 1	LOSS	protein-coding
4_T1	chr1	6.6E+07	7E+07	4999	+	**1**	**12.5**	NA	Intergenic	L1_Mm|LINE|L1	-101329	NM_008632	17756	Mm.256966	NM_008632	ENSMUSG00000015222	**Map2**	G1-397-34|MAP-2|Mtap-2|Mtap2|repro4	microtubule-associated protein 2	LOSS	protein-coding
300_T32	chr14	3.3E+07	3E+07	29999	+	**1**	**12.5**	NA	Intergenic	Intergenic	-51342	NM_016700	26419	Mm.21495	NM_016700	ENSMUSG00000021936	**Mapk8**	AI849689|JNK|JNK1|Prkm8|SAPK1	mitogen-activated protein kinase 8	LOSS	protein-coding
1861_T32	chr18	7.1E+07	7E+07	16999	+	**1**	**12.5**	NA	Intergenic	MLT1A1|LTR|ERVL-MaLR	141708	NM_010773	17191	Mm.322	NM_010773	ENSMUSG00000024513	**Mbd2**	MBD2a	methyl-CpG binding domain protein 2	LOSS	protein-coding
1878_T12	chr18	7.1E+07	7E+07	3999	+	**1**	**12.5**	NA	intron (NM_007831, intron 22 of 28)	RSINE1|SINE|B4	771208	NM_010773	17191	Mm.322	NM_010773	ENSMUSG00000024513	**Mbd2**	MBD2a	methyl-CpG binding domain protein 2	LOSS	protein-coding
1858_T32	chr18	7.1E+07	7E+07	4999	+	**1**	**12.5**	NA	intron (NM_010773, intron 3 of 6)	MTE2a|LTR|ERVL-MaLR	44708	NM_010773	17191	Mm.322	NM_010773	ENSMUSG00000024513	**Mbd2**	MBD2a	methyl-CpG binding domain protein 2	LOSS	protein-coding
1831_T12	chr18	6.8E+07	7E+07	999	+	**1**	**12.5**	NA	Intergenic	Intergenic	-45680	NM_001271716	17200	Mm.426053	NM_008560	ENSMUSG00000045569	**Mc2r**	ACTH-R|ACTHR|MC2-R	melanocortin 2 receptor	LOSS	protein-coding
84_T12	chr12	1.1E+08	1E+08	1999	+	**1**	**12.5**	NA	intron (NR_027651, intron 1 of 8)	intron (NR_027651, intron 1 of 8)	504	NR_027651	17263	Mm.289645	NM_144513	ENSMUSG00000021268	**Meg3**	2900016C05Rik|3110050O07Rik|6330408G06Rik|AI425946|AW108224|D12Bwg1266e|Gtl2|R74756|R75394	maternally expressed 3	LOSS	ncRNA
1646_T2	chr18	5.7E+07	6E+07	999	+	**1**	**12.5**	NA	intron (NM_001001979, intron 5 of 24)	intron (NM_001001979, intron 5 of 24)	87910	NM_001001979	70417	Mm.297863	NM_001001979	ENSMUSG00000024593	**Megf10**	3000002B06Rik|Gm331	multiple EGF-like-domains 10	LOSS	protein-coding
25_T32	chr11	1.9E+07	2E+07	3999	+	**1**	**12.5**	NA	Intergenic	Intergenic	145469	NM_001193271	17268	Mm.356578	NM_010789	ENSMUSG00000020160	**Meis1**	C530044H18Rik|Evi8	Meis homeobox 1	LOSS	protein-coding
2309_T12	chr2	1.2E+08	1E+08	3999	+	**1**	**12.5**	NA	intron (NM_001159567, intron 1 of 12)	intron (NM_001159567, intron 1 of 12)	1121	NM_001159569	17536	Mm.247566	NM_010825	ENSMUSG00000027210	**Meis2**	A430109D20Rik|Mrg1|Stra10	Meis homeobox 2	LOSS	protein-coding

1944_T12	chr18	7.3E+07	7E+07	5999	+	**1**	**12.5**	NA	Intergenic	L1Md_F2|LINE|L1	-239205	NM_001039214	240396	Mm.24131	NM_001039214	ENSMUSG00000037253	**Mex3c**	A130001D14Rik|BC035207|BM-013|Rkhd2	mex3 RNA binding family member C	LOSS	protein-coding
2381_T42	chr6	5.1E+07	5E+07	10999	+	**1**	**12.5**	NA	Intergenic	Intergenic	91910	NR_029719	387166		NR_029719	ENSMUSG00000065505	**Mir148a**	Mirn148|Mirn148a|mir-148a	microRNA 148a	LOSS	ncRNA
330_T12	chr14	3.5E+07	4E+07	395999	+	**1**	**12.5**	NA	intron (NM_008166, intron 3 of 15)	intron (NM_008166, intron 3 of 15)	106891	NR_029774	723847		NR_029774	ENSMUSG00000065481	**Mir346**	Mirn346|mir-346|mmu-mir-346	microRNA 346	LOSS	ncRNA
331_T12	chr14	3.5E+07	4E+07	68999	+	**1**	**12.5**	NA	intron (NM_008166, intron 4 of 15)	intron (NM_008166, intron 4 of 15)	346391	NR_029774	723847		NR_029774	ENSMUSG00000065481	**Mir346**	Mirn346|mir-346|mmu-mir-346	microRNA 346	LOSS	ncRNA
332_T12	chr14	3.5E+07	4E+07	115999	+	**1**	**12.5**	NA	intron (NM_008166, intron 8 of 15)	RLTR20B3A_MM|LTR|ERVK	444891	NR_029774	723847		NR_029774	ENSMUSG00000065481	**Mir346**	Mirn346|mir-346|mmu-mir-346	microRNA 346	LOSS	ncRNA
986_T2	chr18	2.5E+07	2E+07	3999	+	**1**	**12.5**	NA	intron (NM_026779, intron 5 of 14)	intron (NM_026779, intron 5 of 14)	18809	NM_026779	68591	Mm.28252	NM_026779	ENSMUSG00000039616	**Mocos**	1110018O12Rik|HMCS|MCS|MOS	molybdenum cofactor sulfurase	LOSS	protein-coding
673_T2	chr18	7527501	8E+06	999	+	**1**	**12.5**	NA	intron (NM_001161620, intron 2 of 12)	IAPEz-int|LTR|ERVK	98863	NM_001081287	75739	Mm.133293	NM_001081287	ENSMUSG00000057440	**Mpp7**	1110068J02Rik|2810038M04Rik|5430426E14Rik|AI415104|Gm955	membrane protein, palmitoylated 7 (MAGUK p55 subfamily member 7)	LOSS	protein-coding
674_T1	chr18	7534501	8E+06	999	+	**1**	**12.5**	NA	intron (NM_001161620, intron 2 of 12)	intron (NM_001161620, intron 2 of 12)	91863	NM_001081287	75739	Mm.133293	NM_001081287	ENSMUSG00000057440	**Mpp7**	1110068J02Rik|2810038M04Rik|5430426E14Rik|AI415104|Gm955	membrane protein, palmitoylated 7 (MAGUK p55 subfamily member 7)	LOSS	protein-coding
94_T3	chr13	6.9E+07	7E+07	1999	+	**1**	**12.5**	NA	Intergenic	L1Md_A|LINE|L1	78604	NM_001308475	210009	Mm.205514	NM_172480	ENSMUSG00000034617	**Mtrr**	4732420G08|MSR	5-methyltetrahydrofolate-homocysteine methyltransferase reductase	LOSS	protein-coding
96_T3	chr13	6.9E+07	7E+07	999	+	**1**	**12.5**	NA	Intergenic	Lx8|LINE|L1	72104	NM_001308475	210009	Mm.205514	NM_172480	ENSMUSG00000034617	**Mtrr**	4732420G08|MSR	5-methyltetrahydrofolate-homocysteine methyltransferase reductase	LOSS	protein-coding
1155_T22	chr18	3.2E+07	3E+07	2999	+	**1**	**12.5**	NA	Intergenic	L1Md_T|LINE|L1	-12069	NM_032394	17922	Mm.440013	NM_032394	ENSMUSG00000024388	**Myo7b**	-	myosin VIIB	LOSS	protein-coding
2424_T3	chr7	4.5E+07	4E+07	999	+	**1**	**12.5**	NA	intron (NM_008437, intron 1 of 8)	intron (NM_008437, intron 1 of 8)	8555	NM_008437	16541	Mm.383181	NM_008437	ENSMUSG00000002204	**Napsa**	KAP|Kdap|NAP1|SNAPA|pronapsin	napsin A aspartic peptidase	LOSS	protein-coding

159_T42	chr14	1.5E+07	2E+07	11999	+	**1**	**12.5**	NA	Intergenic	Intergenic	279685	NM_001195229	674895	Mm.317563	NM_001034865	ENSMUSG00000042567	**Nek10**	Gm282	NIMA (never in mitosis gene a)-related kinase 10	LOSS	protein-coding
2158_T1	chr18	8.6E+07	9E+07	999	+	**1**	**12.5**	NA	Intergenic	Intergenic	-483952	NM_144946	246317	Mm.329578	NM_144946	ENSMUSG00000050321	**Neto1**	AI851453|Btcl1|C130005O10Rik	neuropilin (NRP) and tolloid (TLL)-like 1	LOSS	protein-coding
2162_T2	chr18	8.6E+07	9E+07	6999	+	**1**	**12.5**	NA	Intergenic	Intergenic	-208952	NM_144946	246317	Mm.329578	NM_144946	ENSMUSG00000050321	**Neto1**	AI851453|Btcl1|C130005O10Rik	neuropilin (NRP) and tolloid (TLL)-like 1	LOSS	protein-coding
163_T2	chr14	1.6E+07	2E+07	24999	+	**1**	**12.5**	NA	Intergenic	Lx|LINE|L1	-213314	NM_021504	59007	Mm.258381	NM_021504	ENSMUSG00000021785	**Ngly1**	1110002C09Rik|PNGase|Png1	N-glycanase 1	LOSS	protein-coding
164_T22	chr14	1.6E+07	2E+07	21999	+	**1**	**12.5**	NA	Intergenic	Intergenic	-181814	NM_021504	59007	Mm.258381	NM_021504	ENSMUSG00000021785	**Ngly1**	1110002C09Rik|PNGase|Png1	N-glycanase 1	LOSS	protein-coding
165_T4	chr14	1.6E+07	2E+07	6999	+	**1**	**12.5**	NA	Intergenic	Lx7|LINE|L1	-101314	NM_021504	59007	Mm.258381	NM_021504	ENSMUSG00000021785	**Ngly1**	1110002C09Rik|PNGase|Png1	N-glycanase 1	LOSS	protein-coding
57_T2	chr12	4.6E+07	5E+07	4999	+	**1**	**12.5**	NA	Intergenic	Intergenic	386775	NM_021361	664883	Mm.247195	NM_021361	ENSMUSG00000021047	**Nova1**	9430099M15Rik|G630039L02|Nova-1	neuro-oncological ventral antigen 1	LOSS	protein-coding
170_T22	chr14	1.8E+07	2E+07	999	+	**1**	**12.5**	NA	Intergenic	RLTR13A|LTR|ERVK	92106	NM_011584	353187	Mm.26587	NM_011584	ENSMUSG00000021775	**Nr1d2**	RVR|Rev-erb	nuclear receptor subfamily 1, group D, member 2	LOSS	protein-coding
2422_T3	chr7	4.5E+07	4E+07	3999	+	**1**	**12.5**	NA	Intergenic	B1_Mus1|SINE|Alu	-1535	NM_001285517	22260	Mm.968	NM_009473	ENSMUSG00000060601	**Nr1h2**	AI194859|LXR|LXRB|LXRBSV|LXRbeta|NER1|OR-1|RIP15|UR|Unr|Unr2	nuclear receptor subfamily 1, group H, member 2	LOSS	protein-coding
382_T12	chr14	4E+07	4E+07	5999	+	**1**	**12.5**	NA	Intergenic	MLT1D|LTR|ERVL-MaLR	-83412	NM_008734	18183	Mm.258705	NM_008734	ENSMUSG00000041014	**Nrg3**	ska	neuregulin 3	LOSS	protein-coding
21_T42	chr10	8.1E+07	8E+07	4999	+	**1**	**12.5**	NA	Intergenic	L1Md_F2|LINE|L1	-5656	NM_001301034	18245	Mm.398119	NM_008753	ENSMUSG00000035242	**Oaz1**	AZ-1|AZ1|Antizyme|ODC-Az|Oaz	ornithine decarboxylase antizyme 1	LOSS	protein-coding
294_T22	chr14	3.2E+07	3E+07	88999	+	**1**	**12.5**	NA	TTS (NM_001081130)	TTS (NM_001081130)	25981	NM_001081130	239017	Mm.318302	NM_001081130	ENSMUSG00000021913	**Ogdhl**	-	oxoglutarate dehydrogenase-like	LOSS	protein-coding
2055_T32	chr18	8E+07	8E+07	2999	+	**1**	**12.5**	NA	Intergenic	IAPEz-int|LTR|ERVK	-339895	NM_053117	93737	Mm.24678	NM_053117	ENSMUSG00000056214	**Pard6g**	2410049N21Rik|Par6a	par-6 family cell polarity regulator gamma	LOSS	protein-coding
2062_T2	chr18	8E+07	8E+07	5999	+	**1**	**12.5**	NA	Intergenic	L1Md_T|LINE|L1	-300395	NM_053117	93737	Mm.24678	NM_053117	ENSMUSG00000056214	**Pard6g**	2410049N21Rik|Par6a	par-6 family cell polarity regulator gamma	LOSS	protein-coding
1231_T2	chr18	3.7E+07	4E+07	999	+	**1**	**12.5**	NA	intron (NM_001301259, intron 1 of 3)	intron (NM_001301259, intron 1 of 3)	-3467	NM_201243	353235	Mm.308500	NM_201243	ENSMUSG00000103800	**Pcdha8**	-	protocadherin alpha 8	LOSS	protein-coding
1239_T12	chr18	3.7E+07	4E+07	999	+	**1**	**12.5**	NA	intron (NM_001301259, intron 1 of 3)	Lx2|LINE|L1	8729	NM_053133	93879	Mm.348052	NM_053133	ENSMUSG00000045876	**Pcdhb8**	Pcdhb5C|PcdhbH	protocadherin beta 8	LOSS	protein-coding

1_T1	chr1	7389501	7E+06	3999	+	**1**	**12.5**	NA	Intergenic	Intergenic	302580	NM_183028	319263	Mm.21539	NM_183028	ENSMUSG00000051285	**Pcmtd1**	8430411F12Rik|A030012M09Rik	protein-L-isoaspartate (D-aspartate) O-methyltransferase domain containing 1	LOSS	protein-coding
246_T3	chr14	2.7E+07	3E+07	33999	+	**1**	**12.5**	NA	Intergenic	RLTR20C1_MM|LTR|ERVK	-52654	NM_178668	211948	Mm.290758	NM_178668	ENSMUSG00000043702	**Pde12**	2'-PDE|E430028B21Rik	phosphodiesterase 12	LOSS	protein-coding
280_T22	chr14	3.1E+07	3E+07	193999	+	**1**	**12.5**	NA	Intergenic	Intergenic	14718	NM_027949	71838	Mm.5348	NM_027949	ENSMUSG00000021902	**Phf7**	1700006H01Rik|1700010P14Rik|AI427892|AW555949	PHD finger protein 7	LOSS	protein-coding
2459_T2	chr8	8.6E+07	9E+07	3999	+	**1**	**12.5**	NA	Intergenic	L1Md_F3|LINE|L1	336498	NM_199446	102093	Mm.237296	NM_199446	ENSMUSG00000036879	**Phkb**	AI463271	phosphorylase kinase beta	LOSS	protein-coding
1748_T32	chr18	6.3E+07	6E+07	999	+	**1**	**12.5**	NA	Intergenic	L1Md_A|LINE|L1	-88817	NM_001039485	667742	Mm.158720	NM_172629	ENSMUSG00000041482	**Piezo2**	5930434P17|9030411M15Rik|9430028L06Rik|Fam38b|Fam38b2	piezo-type mechanosensitive ion channel component 2	LOSS	protein-coding
1749_T32	chr18	6.3E+07	6E+07	999	+	**1**	**12.5**	NA	Intergenic	Intergenic	-94817	NM_001039485	667742	Mm.158720	NM_172629	ENSMUSG00000041482	**Piezo2**	5930434P17|9030411M15Rik|9430028L06Rik|Fam38b|Fam38b2	piezo-type mechanosensitive ion channel component 2	LOSS	protein-coding
1110_T12	chr18	3.1E+07	3E+07	5999	+	**1**	**12.5**	NA	Intergenic	L1Md_F2|LINE|L1	459604	NM_181414	225326	Mm.194127	NM_181414	ENSMUSG00000033628	**Pik3c3**	5330434F23Rik|Vps34	phosphoinositide-3-kinase, class 3	LOSS	protein-coding
2393_T32	chr6	1.3E+08	1E+08	11999	+	**1**	**12.5**	NA	Intergenic	MTD|LTR|ERVL-MaLR	-15996	NM_198604	213522	Mm.333396	NM_198604	ENSMUSG00000038167	**Plekhg6**	BC026778|Gm155	pleckstrin homology domain containing, family G (with RhoGef domain) member 6	LOSS	protein-coding
617_T1	chr16	3.7E+07	4E+07	12999	+	**1**	**12.5**	NA	intron (NM_172440, intron 25 of 27)	(TA)n|Simple_repeat|Simple_repeat	116214	NM_001159369	77782	Mm.259114	NM_029977	ENSMUSG00000034206	**Polq**	A430110D14Rik	polymerase (DNA directed), theta	LOSS	protein-coding
2382_T2	chr6	5.7E+07	6E+07	4999	+	**1**	**12.5**	NA	Intergenic	MTA_Mm-int|LTR|ERVL-MaLR	43426	NM_175523	243382	Mm.396893	NM_175523	ENSMUSG00000037826	**Ppm1k**	2900063A19Rik|A930026L03Rik|PP2Cm	protein phosphatase 1K (PP2C domain containing)	LOSS	protein-coding
1292_T32	chr18	4.1E+07	4E+07	5999	+	**1**	**12.5**	NA	Intergenic	Intergenic	783694	NM_029942	77619	Mm.30649	NM_029942	ENSMUSG00000056671	**Prelid2**	1700003A01Rik|C330008K14Rik	PRELI domain containing 2	LOSS	protein-coding
1300_T1	chr18	4.1E+07	4E+07	12999	+	**1**	**12.5**	NA	Intergenic	Intergenic	606194	NM_029942	77619	Mm.30649	NM_029942	ENSMUSG00000056671	**Prelid2**	1700003A01Rik|C330008K14Rik	PRELI domain containing 2	LOSS	protein-coding
1301_T2	chr18	4.1E+07	4E+07	999	+	**1**	**12.5**	NA	Intergenic	RLTR17B_Mm|LTR|ERVK	590194	NM_029942	77619	Mm.30649	NM_029942	ENSMUSG00000056671	**Prelid2**	1700003A01Rik|C330008K14Rik	PRELI domain containing 2	LOSS	protein-coding
1309_T2	chr18	4.1E+07	4E+07	1999	+	**1**	**12.5**	NA	Intergenic	L1Md_A|LINE|L1	480694	NM_029942	77619	Mm.30649	NM_029942	ENSMUSG00000056671	**Prelid2**	1700003A01Rik|C330008K14Rik	PRELI domain containing 2	LOSS	protein-coding
1314_T1	chr18	4.2E+07	4E+07	999	+	**1**	**12.5**	NA	Intergenic	L1Md_T|LINE|L1	394194	NM_029942	77619	Mm.30649	NM_029942	ENSMUSG00000056671	**Prelid2**	1700003A01Rik|C330008K14Rik	PRELI domain containing 2	LOSS	protein-coding

1316_T12	chr18	4.2E+07	4E+07	999	+	**1**	**12.5**	NA	Intergenic	L1Md_T|LINE|L1	129194	NM_029942	77619	Mm.30649	NM_029942	ENSMUSG00000056671	**Prelid2**	1700003A01Rik|C330008K14Rik	PRELI domain containing 2	LOSS	protein-coding
1518_T12	chr18	5.1E+07	5E+07	999	+	**1**	**12.5**	NA	Intergenic	L1Md_F2|LINE|L1	-152898	NM_001081224	71373	Mm.63546	NM_001081224	ENSMUSG00000073565	**Prr16**	5430406M13Rik|AI607429	proline rich 16	LOSS	protein-coding
1521_T2	chr18	5.1E+07	5E+07	5999	+	**1**	**12.5**	NA	Intergenic	IAPEz-int|LTR|ERVK	-43398	NM_001081224	71373	Mm.63546	NM_001081224	ENSMUSG00000073565	**Prr16**	5430406M13Rik|AI607429	proline rich 16	LOSS	protein-coding
2374_T12	chr6	4.1E+07	4E+07	999	+	**1**	**12.5**	NA	Intergenic	L1Md_T|LINE|L1	9613	NM_011645	22073	Mm.425318	NM_011645	ENSMUSG00000071519	**Prss3**	MTG|TRY4|Tb|Try3	protease, serine 3	LOSS	protein-coding
157_T4	chr14	1.4E+07	1E+07	92999	+	**1**	**12.5**	NA	intron (NM_139227, intron 4 of 11)	MERVL-int|LTR|ERVL	45904	NM_025550	66413	Mm.27591	NM_025550	ENSMUSG00000021737	**Psmd6**	2400006A19Rik	proteasome (prosome, macropain) 26S subunit, non-ATPase, 6	LOSS	protein-coding
449_T12	chr14	4.5E+07	5E+07	370999	+	**1**	**12.5**	NA	Intergenic	Intergenic	11375	NM_008962	19214	Mm.5105	NM_008962	ENSMUSG00000071489	**Ptgdr**	DP|PGD	prostaglandin D receptor	LOSS	protein-coding
140_T3	chr14	1.1E+07	1E+07	373999	+	**1**	**12.5**	NA	intron (NM_008981, intron 1 of 29)	intron (NM_008981, intron 1 of 29)	77947	NM_008981	19270	Mm.431266	NM_008981	ENSMUSG00000021745	**Ptprg**	5430405N12Rik|AW046354|AW549872|RPTPgamma	protein tyrosine phosphatase, receptor type, G	LOSS	protein-coding
1848_T12	chr18	7E+07	7E+07	5999	+	**1**	**12.5**	NA	intron (NM_001082553, intron 2 of 6)	L1Md_T|LINE|L1	43088	NM_001302798	80718	Mm.246753	NM_030554	ENSMUSG00000024511	**Rab27b**	2310021G14Rik|B130064M09Rik	RAB27B, member RAS oncogene family	LOSS	protein-coding
1850_T12	chr18	7E+07	7E+07	4999	+	**1**	**12.5**	NA	intron (NM_001082553, intron 2 of 6)	L1Md_A|LINE|L1	20588	NM_001302798	80718	Mm.246753	NM_030554	ENSMUSG00000024511	**Rab27b**	2310021G14Rik|B130064M09Rik	RAB27B, member RAS oncogene family	LOSS	protein-coding
2312_T32	chr2	1.5E+08	2E+08	3999	+	**1**	**12.5**	NA	intron (NM_001139511, intron 1 of 7)	(TAAA)n|Simple_repeat|Simple_repeat	11390	NM_001139511	19383	Mm.221440	NM_023130	ENSMUSG00000027593	**Raly**	AI663842|Merc	hnRNP-associated with lethal yellow	LOSS	protein-coding
168_T4	chr14	1.7E+07	2E+07	20999	+	**1**	**12.5**	NA	intron (NM_001289760, intron 2 of 9)	L1_Rod|LINE|L1	-143524	NM_001289762	218772	Mm.259318	NM_011243	ENSMUSG00000017491	**Rarb**	A830025K23|Hap|Nr1b2	retinoic acid receptor, beta	LOSS	protein-coding
167_T4	chr14	1.7E+07	2E+07	8999	+	**1**	**12.5**	NA	intron (NM_001289761, intron 2 of 8)	intron (NM_001289761, intron 2 of 8)	3476	NM_011243	218772	Mm.259318	NM_011243	ENSMUSG00000017491	**Rarb**	A830025K23|Hap|Nr1b2	retinoic acid receptor, beta	LOSS	protein-coding
1781_T1	chr18	6.6E+07	7E+07	999	+	**1**	**12.5**	NA	Intergenic	Intergenic	27089	NM_013833	19434	Mm.431047	NM_013833	ENSMUSG00000024518	**Rax**	7530406A22Rik|E130303K03Rik|Rx|ey-1|ey1	retina and anterior neural fold homeobox	LOSS	protein-coding

635_T42	chr16	7.6E+07	8E+07	999	+	**1**	**12.5**	NA	Intergenic	Intergenic	66109	NM_198302	224344	Mm.273214	NM_198302	ENSMUSG00000032940	**Rbm11**	A330018F01	RNA binding motif protein 11	LOSS	protein-coding
354_T12	chr14	3.7E+07	4E+07	293999	+	**1**	**12.5**	NA	intron (NM_001301692, intron 3 of 5)	intron (NM_001301692, intron 3 of 5)	4514	NM_001301694	57811	Mm.20460	NM_021340	ENSMUSG00000021804	**Rgr**	-	retinal G protein coupled receptor	LOSS	protein-coding
15_T22	chr10	6170501	6E+06	3999	+	**1**	**12.5**	NA	Intergenic	L1Md_F2|LINE|L1	-250100	NM_019958	56533	Mm.44606	NM_019958	ENSMUSG00000019775	**Rgs17**	6430507P11Rik|Rgsz2	regulator of G-protein signaling 17	LOSS	protein-coding
1115_T3	chr18	3.1E+07	3E+07	999	+	**1**	**12.5**	NA	Intergenic	L1Md_A|LINE|L1	513128	NM_009065	19762	Mm.5163	NM_009065	ENSMUSG00000057455	**Rit2**	RIBA|Rin|Roc2	Ras-like without CAAX 2	LOSS	protein-coding
1122_T12	chr18	3.1E+07	3E+07	2999	+	**1**	**12.5**	NA	Intergenic	L1Md_F2|LINE|L1	444128	NM_009065	19762	Mm.5163	NM_009065	ENSMUSG00000057455	**Rit2**	RIBA|Rin|Roc2	Ras-like without CAAX 2	LOSS	protein-coding
1123_T12	chr18	3.1E+07	3E+07	999	+	**1**	**12.5**	NA	Intergenic	Lx9|LINE|L1	437128	NM_009065	19762	Mm.5163	NM_009065	ENSMUSG00000057455	**Rit2**	RIBA|Rin|Roc2	Ras-like without CAAX 2	LOSS	protein-coding
1125_T12	chr18	3.1E+07	3E+07	4999	+	**1**	**12.5**	NA	Intergenic	Intergenic	349128	NM_009065	19762	Mm.5163	NM_009065	ENSMUSG00000057455	**Rit2**	RIBA|Rin|Roc2	Ras-like without CAAX 2	LOSS	protein-coding
1134_T12	chr18	3.1E+07	3E+07	5999	+	**1**	**12.5**	NA	intron (NM_009065, intron 3 of 4)	intron (NM_009065, intron 3 of 4)	134628	NM_009065	19762	Mm.5163	NM_009065	ENSMUSG00000057455	**Rit2**	RIBA|Rin|Roc2	Ras-like without CAAX 2	LOSS	protein-coding
1129_T1	chr18	3.1E+07	3E+07	999	+	**1**	**12.5**	NA	intron (NM_009065, intron 4 of 4)	L1_Rod|LINE|L1	309128	NM_009065	19762	Mm.5163	NM_009065	ENSMUSG00000057455	**Rit2**	RIBA|Rin|Roc2	Ras-like without CAAX 2	LOSS	protein-coding
981_T1	chr18	2.5E+07	2E+07	1999	+	**1**	**12.5**	NA	Intergenic	MMERGLN-int|LTR|ERV1	-9296	NM_144861	225283	Mm.28427	NM_144861	ENSMUSG00000040446	**Rprd1a**	C77387|mKIAA4077	regulation of nuclear pre-mRNA domain containing 1A	LOSS	protein-coding
2304_T4	chr2	8.5E+07	8E+07	4999	+	**1**	**12.5**	NA	Intergenic	RLTR6-int|LTR|ERV1	-19308	NM_199223	269295	Mm.86447	NM_199223	ENSMUSG00000050896	**Rtn4rl2**	Ngr2|Ngrh1|Ngrl3	reticulon 4 receptor-like 2	LOSS	protein-coding
2225_T2	chr18	8.9E+07	9E+07	999	+	**1**	**12.5**	NA	Intergenic	L1Md_A|LINE|L1	-31790	NM_175542	246102	Mm.772	NM_175542	ENSMUSG00000023066	**Rttn**	4921538A15Rik|AI666264|C530033I08Rik|D230040K24	rotatin	LOSS	protein-coding
2439_T1	chr7	1.1E+08	1E+08	3999	+	**1**	**12.5**	NA	intron (NM_177324, intron 1 of 40)	intron (NM_177324, intron 1 of 40)	25420	NM_177324	319934	Mm.102970	NM_177324	ENSMUSG00000038371	**Sbf2**	4833411B01Rik|AA414977|AI317167|B430219L04Rik|Mtmr13|mMTMH1	SET binding factor 2	LOSS	protein-coding
58_T22	chr12	5.9E+07	6E+07	3999	+	**1**	**12.5**	NA	Intergenic	L1Md_T|LINE|L1	323517	NM_009147	20334	Mm.491137	NM_009147	ENSMUSG00000020986	**Sec23a**	Msec23|Sec23r	SEC23 homolog A, COPII coat complex component	LOSS	protein-coding

2311_T22	chr2	1.2E+08	1E+08	9999	+	**1**	**12.5**	NA	Intergenic	IAPLTR2_Mm|LTR|ERVK	-570469	NM_001290997	214968	Mm.330536	NM_172537	ENSMUSG00000027200	**Sema6d**	1110067B02Rik|AA409156|D330011G23|mKIAA1479	sema domain, transmembrane domain (TM), and cytoplasmic domain, (semaphorin) 6D	LOSS	protein-coding
2044_T32	chr18	7.9E+07	8E+07	8999	+	**1**	**12.5**	NA	Intergenic	L1Md_T|LINE|L1	-251609	NM_053099	240427	Mm.312871	NM_053099	ENSMUSG00000024548	**Setbp1**	C130092E12|Seb|mKIAA0437	SET binding protein 1	LOSS	protein-coding
406_T12	chr14	4.1E+07	4E+07	4999	+	**1**	**12.5**	NA	Intergenic	Intergenic	-69802	NM_009160	20390	Mm.1321	NM_009160	ENSMUSG00000021795	**Sftpd**	AI573415|SP-D|Sfpd|Sftp4	surfactant associated protein D	LOSS	protein-coding
407_T12	chr14	4.1E+07	4E+07	33999	+	**1**	**12.5**	NA	Intergenic	L1_Mus3|LINE|L1	-90302	NM_009160	20390	Mm.1321	NM_009160	ENSMUSG00000021795	**Sftpd**	AI573415|SP-D|Sfpd|Sftp4	surfactant associated protein D	LOSS	protein-coding
408_T12	chr14	4.1E+07	4E+07	157999	+	**1**	**12.5**	NA	Intergenic	RMER13A1|LTR|ERVK	-193302	NM_009160	20390	Mm.1321	NM_009160	ENSMUSG00000021795	**Sftpd**	AI573415|SP-D|Sfpd|Sftp4	surfactant associated protein D	LOSS	protein-coding
2369_T4	chr6	4745501	5E+06	999	+	**1**	**12.5**	NA	intron (NM_001130188, intron 1 of 11)	intron (NM_001130188, intron 1 of 11)	1204	NM_001130188	20392	Mm.8739	NM_011360	ENSMUSG00000004631	**Sgce**	e-SG	sarcoglycan, epsilon	LOSS	protein-coding
399_T12	chr14	4.1E+07	4E+07	4999	+	**1**	**12.5**	NA	Intergenic	Lx7|LINE|L1	368965	NM_177816	328381	Mm.235694	NM_177816	ENSMUSG00000037833	**Sh2d4b**	A430109M18Rik|D030001E08	SH2 domain containing 4B	LOSS	protein-coding
401_T12	chr14	4.1E+07	4E+07	227999	+	**1**	**12.5**	NA	Intergenic	RLTR13B1|LTR|ERVK	224465	NM_177816	328381	Mm.235694	NM_177816	ENSMUSG00000037833	**Sh2d4b**	A430109M18Rik|D030001E08	SH2 domain containing 4B	LOSS	protein-coding
402_T12	chr14	4.1E+07	4E+07	54999	+	**1**	**12.5**	NA	intron (NM_177816, intron 7 of 7)	L1ME3G|LINE|L1	75965	NM_177816	328381	Mm.235694	NM_177816	ENSMUSG00000037833	**Sh2d4b**	A430109M18Rik|D030001E08	SH2 domain containing 4B	LOSS	protein-coding
2029_T32	chr18	7.9E+07	8E+07	999	+	**1**	**12.5**	NA	Intergenic	L1_Mus2|LINE|L1	-107050	NM_030683	27411	Mm.44158	NM_030683	ENSMUSG00000024552	**Slc14a2**	UT-A1|UT-A3	solute carrier family 14 (urea transporter), member 2	LOSS	protein-coding
2026_T2	chr18	7.8E+07	8E+07	999	+	**1**	**12.5**	NA	intron (NM_207651, intron 2 of 21)	L1Md_T|LINE|L1	182950	NM_207651	27411	Mm.44158	NM_030683	ENSMUSG00000024552	**Slc14a2**	UT-A1|UT-A3	solute carrier family 14 (urea transporter), member 2	LOSS	protein-coding
2288_T2	chr19	8506501	9E+06	5999	+	**1**	**12.5**	NA	Intergenic	Lx3C|LINE|L1	-81754	NM_001164634	19879	Mm.285294	NM_031194	ENSMUSG00000063796	**Slc22a8**	Oat3|Roct	solute carrier family 22 (organic anion transporter), member 8	LOSS	protein-coding
56_T4	chr12	3.2E+07	3E+07	3999	+	**1**	**12.5**	NA	intron (NM_011867, intron 15 of 20)	PB1D7|SINE|Alu	28469	NM_011867	23985	Mm.100187	NM_011867	ENSMUSG00000020651	**Slc26a4**	Pds|pendrin	solute carrier family 26, member 4	LOSS	protein-coding

499_T12	chr14	4.9E+07	5E+07	148999	+	**1**	**12.5**	NA	Intergenic	MTA_Mm-int|LTR|ERVL-MaLR	-8163	NM_029238	75288	Mm.78738	NM_029238	ENSMUSG00000021852	**Slc35f4**	4930550L21Rik	solute carrier family 35, member F4	LOSS	protein-coding
143_T3	chr14	1.4E+07	1E+07	165999	+	**1**	**12.5**	NA	Intergenic	RLTR4_MM-int|LTR|ERV1	113624	NM_177624	218739	Mm.39919	NM_177624	ENSMUSG00000044772	**Sntn**	A430083B19Rik	sentan, cilia apical structure protein	LOSS	protein-coding
1570_T22	chr18	5.3E+07	5E+07	999	+	**1**	**12.5**	NA	Intergenic	MTD|LTR|ERVL-MaLR	-111365	NM_026386	67804	Mm.252171	NM_026386	ENSMUSG00000034484	**Snx2**	0610030A03Rik	sorting nexin 2	LOSS	protein-coding
2206_T12	chr18	8.8E+07	9E+07	999	+	**1**	**12.5**	NA	Intergenic	L1Md_T|LINE|L1	996207	NM_018821	54607	Mm.91920	NM_018821	ENSMUSG00000056153	**Socs6**	1500012M23Rik|5830401B18Rik|AI447482|Cis4|Cish4|HSPC060|SOCS-4|SOCS-6|SSI4|STAI4|STAI4|Socs4	suppressor of cytokine signaling 6	LOSS	protein-coding
2208_T1	chr18	8.8E+07	9E+07	10999	+	**1**	**12.5**	NA	Intergenic	Intergenic	894207	NM_018821	54607	Mm.91920	NM_018821	ENSMUSG00000056153	**Socs6**	1500012M23Rik|5830401B18Rik|AI447482|Cis4|Cish4|HSPC060|SOCS-4|SOCS-6|SSI4|STAI4|STAI4|Socs4	suppressor of cytokine signaling 6	LOSS	protein-coding
2210_T1	chr18	8.8E+07	9E+07	999	+	**1**	**12.5**	NA	Intergenic	MTD|LTR|ERVL-MaLR	653207	NM_018821	54607	Mm.91920	NM_018821	ENSMUSG00000056153	**Socs6**	1500012M23Rik|5830401B18Rik|AI447482|Cis4|Cish4|HSPC060|SOCS-4|SOCS-6|SSI4|STAI4|STAI4|Socs4	suppressor of cytokine signaling 6	LOSS	protein-coding
2214_T2	chr18	8.8E+07	9E+07	4999	+	**1**	**12.5**	NA	Intergenic	Intergenic	521207	NM_018821	54607	Mm.91920	NM_018821	ENSMUSG00000056153	**Socs6**	1500012M23Rik|5830401B18Rik|AI447482|Cis4|Cish4|HSPC060|SOCS-4|SOCS-6|SSI4|STAI4|STAI4|Socs4	suppressor of cytokine signaling 6	LOSS	protein-coding
63_T2	chr12	7E+07	7E+07	5999	+	**1**	**12.5**	NA	Intergenic	Intergenic	-1648	NM_001135559	20663	Mm.3770	NM_001135559	ENSMUSG00000034801	**Sos2**	SOS-2|mSOS-2	son of sevenless homolog 2 (Drosophila)	LOSS	protein-coding
1347_T2	chr18	4.4E+07	4E+07	999	+	**1**	**12.5**	NA	Intergenic	Intergenic	20237	NM_009258	20730	Mm.272	NM_009258	ENSMUSG00000024503	**Spink1**	Spink3|p12	serine peptidase inhibitor, Kazal type 1	LOSS	protein-coding
1358_T1	chr18	4.4E+07	4E+07	999	+	**1**	**12.5**	NA	Intergenic	Intergenic	8131	NM_001039218	433178	Mm.120009	NM_001039218	ENSMUSG00000051050	**Spink14**	EG433178|Gm5505	serine peptidase inhibitor, Kazal type 14	LOSS	protein-coding
2295_T2	chr2	2.4E+07	2E+07	2999	+	**1**	**12.5**	NA	Intergenic	L1Md_F2|LINE|L1	-228896	NM_001165997	76857	Mm.276415	NM_029773	ENSMUSG00000026771	**Spopl**	4921517N04Rik|AU014935|BB233739|E430033K04Rik	speckle-type POZ protein-like	LOSS	protein-coding

2392_T3	chr6	1.2E+08	1E+08	999	+	**1**	**12.5**	NA	Intergenic	MMETn-int|LTR|ERVK	-6661	NM_001306051	14794	Mm.261906	NM_013539	ENSMUSG00000038451	**Spsb2**	AI461677|C9|Grcc9|SSB2	splA/ryanodine receptor domain and SOCS box containing 2	LOSS	protein-coding
1560_T12	chr18	5.2E+07	5E+07	5999	+	**1**	**12.5**	NA	intron (NM_026040, intron 6 of 7)	intron (NM_026040, intron 6 of 7)	23807	NM_026040	67222	Mm.100617	NM_026040	ENSMUSG00000024528	**Srfbp1**	2810036K01Rik|p49/STRAP	serum response factor binding protein 1	LOSS	protein-coding
614_T1	chr16	2.3E+07	2E+07	7999	+	**1**	**12.5**	NA	Intergenic	Intergenic	-1240	NM_145933	20440	Mm.149029	NM_145933	ENSMUSG00000022885	**St6gal1**	AW742324|Siat1|St6Gal-I|St6gal|St6galI	beta galactoside alpha 2,6 sialyltransferase 1	LOSS	protein-coding
665_T2	chr17	5.5E+07	6E+07	3999	+	**1**	**12.5**	NA	Intergenic	L1Md_A|LINE|L1	-144217	NM_172829	240119	Mm.311687	NM_172829	ENSMUSG00000024172	**St6gal2**	C230064G14Rik|St6galII|mKIAA1877	beta galactoside alpha 2,6 sialyltransferase 2	LOSS	protein-coding
1769_T2	chr18	6.4E+07	6E+07	5999	+	**1**	**12.5**	NA	Intergenic	L1Md_A|LINE|L1	-201859	NM_009182	20451	Mm.252110	NM_009182	ENSMUSG00000056812	**St8sia3**	AI847333|ST8SiaIII|Siat8c	ST8 alpha-N-acetyl-neuraminide alpha-2,8-sialyltransferase 3	LOSS	protein-coding
1181_T2	chr18	3.3E+07	3E+07	999	+	**1**	**12.5**	NA	Intergenic	L1Md_F2|LINE|L1	-111184	NM_133774	170459	Mm.127058	NM_133774	ENSMUSG00000024378	**Stard4**	4632419C16Rik|9030213J02Rik	StAR-related lipid transfer (START) domain containing 4	LOSS	protein-coding
29_T12	chr11	3.1E+07	3E+07	3999	+	**1**	**12.5**	NA	Intergenic	RLTR1B-int|LTR|ERV1	-67439	NM_011491	20856	Mm.32506	NM_011491	ENSMUSG00000020303	**Stc2**	AW125853|Stc2l|mustc2	stanniocalcin 2	LOSS	protein-coding
2331_T2	chr3	1.4E+08	1E+08	3999	+	**1**	**12.5**	NA	intron (NM_198659, intron 12 of 13)	IAPEy-int|LTR|ERVK	357607	NM_198659	381476	Mm.188334	NM_198659	ENSMUSG00000047940	**Stpg2**	B930007M17Rik|Gm1017	sperm tail PG rich repeat containing 2	LOSS	protein-coding
2384_T4	chr6	7.3E+07	7E+07	3999	+	**1**	**12.5**	NA	Intergenic	B3|SINE|B2	57995	NM_019879	56451	Mm.29845	NM_019879	ENSMUSG00000052738	**Suclg1**	1500000I01Rik|Sucla1	succinate-CoA ligase, GDP-forming, alpha subunit	LOSS	protein-coding
306_T4	chr14	3.4E+07	3E+07	999	+	**1**	**12.5**	NA	3' UTR (NM_181529, exon 8 of 8)	3' UTR (NM_181529, exon 8 of 8)	9954	NM_181529	319508	Mm.228006	NM_176931	ENSMUSG00000041479	**Syt15**	CHR10SYT|E230025K04Rik|sytXV	synaptotagmin XV	LOSS	protein-coding
55_T2	chr12	2.4E+07	2E+07	10999	+	**1**	**12.5**	NA	Intergenic	Intergenic	-149581	NM_020614	21340	Mm.39082	NM_020614	ENSMUSG00000059669	**Taf1b**	4930408G01Rik|A230108M10Rik|TAFI68|Tafi86|p63	TATA-box binding protein associated factor, RNA polymerase I, B	LOSS	protein-coding
73_T42	chr12	1E+08	1E+08	4999	+	**1**	**12.5**	NA	intron (NM_001286364, intron 7 of 12)	L1VL4|LINE|L1	24573	NM_028924	74413	Mm.275939	NM_028924	ENSMUSG00000021187	**Tc2n**	4933406D09Rik|Mtac2d1|Tac2-N	tandem C2 domains, nuclear	LOSS	protein-coding
2292_T22	chr19	5.6E+07	6E+07	3999	+	**1**	**12.5**	NA	intron (NM_001142919, intron 3 of 13)	intron (NM_001142919, intron 3 of 13)	1690	NM_001142923	21416	Mm.139815	NM_009333	ENSMUSG00000024985	**Tcf7l2**	TCF4B|TCF4E|Tcf-4|Tcf4	transcription factor 7 like 2, T cell specific, HMG box	LOSS	protein-coding
598_T1	chr14	8.8E+07	9E+07	999	+	**1**	**12.5**	NA	Intergenic	L1Md_F|LINE|L1	494417	NM_001253755	219249	Mm.35168	NM_172605	ENSMUSG00000022019	**Tdrd3**	4732418C03Rik|6720468N18	tudor domain containing 3	LOSS	protein-coding

2372_T42	chr6	1.7E+07	2E+07	4999	+	**1**	**12.5**	NA	Intergenic	L1_Mur3|LINE|L1	336441	NM_031198	21426	Mm.36217	NM_031198	ENSMUSG00000029553	**Tfec**	BB107417|Tcfec|bHLHe34	transcription factor EC	LOSS	protein-coding
2142_T1	chr18	8.5E+07	9E+07	4999	+	**1**	**12.5**	NA	Intergenic	L1Md_T|LINE|L1	-160476	NM_025969	67105	Mm.250617	NM_025969	ENSMUSG00000024645	**Timm21**	1700034H14Rik|2700002I20Rik	tranlocase of inner mitochondrial membrane 21	LOSS	protein-coding
2432_T42	chr7	8.9E+07	9E+07	3999	+	**1**	**12.5**	NA	Intergenic	Lx3C|LINE|L1	236287	NM_028343	72759	Mm.208477	NM_028343	ENSMUSG00000039428	**Tmem135**	2810439K08Rik|AW319712|PMP52	transmembrane protein 135	LOSS	protein-coding
2387_T3	chr6	9.7E+07	1E+08	4999	+	**1**	**12.5**	NA	TTS (NM_001301859)	TTS (NM_001301859)	-5876	NM_001081111	232286	Mm.30893	NM_001081111	ENSMUSG00000030059	**Tmf1**	7030402D04Rik|Gm153|Tmf1`	TATA element modulatory factor 1	LOSS	protein-coding
2367_T22	chr5	1.4E+08	1E+08	999	+	**1**	**12.5**	NA	intron (NM_001122730, intron 2 of 27)	intron (NM_001122730, intron 2 of 27)	2387	NM_178242	231861	Mm.354649	NM_178242	ENSMUSG00000039477	**Tnrc18**	BC025631|EG381742|Zfp469	trinucleotide repeat containing 18	LOSS	protein-coding
166_T4	chr14	1.6E+07	2E+07	284999	+	**1**	**12.5**	NA	exon (NM_021504, exon 12 of 12)	exon (NM_021504, exon 12 of 12)	-54206	NM_009409	21974	Mm.130362	NM_009409	ENSMUSG00000017485	**Top2b**	D230016L12Rik|Top-2	topoisomerase (DNA) II beta	LOSS	protein-coding
994_T12	chr18	2.5E+07	3E+07	999	+	**1**	**12.5**	NA	intron (NM_175276, intron 24 of 26)	intron (NM_175276, intron 24 of 26)	40965	NM_001142697	66648	Mm.263189	NM_001004361	ENSMUSG00000024269	**Tpgs2**	5730437P09Rik|5730494M16Rik|AI666318|Pgs2	tubulin polyglutamylase complex subunit 2	LOSS	protein-coding
1168_T1	chr18	3.3E+07	3E+07	2999	+	**1**	**12.5**	NA	intron (NR_045381, intron 4 of 4)	intron (NR_045381, intron 4 of 4)	-115383	NM_021367	53603	Mm.143716	NM_021367	ENSMUSG00000024379	**Tslp**	-	thymic stromal lymphopoietin	LOSS	protein-coding
2310_T12	chr2	1.2E+08	1E+08	3999	+	**1**	**12.5**	NA	intron (NM_080788, intron 4 of 15)	MMETn-int|LTR|ERVK	50918	NM_001024857	140810	Mm.275698	NM_080788	ENSMUSG00000090100	**Ttbk2**	2610507N02Rik|AI326283|B930008N24Rik|TTK|Ttbk|Ttbk1|mKIAA0847	tau tubulin kinase 2	LOSS	protein-coding
1752_T2	chr18	6.4E+07	6E+07	7999	+	**1**	**12.5**	NA	Intergenic	Intergenic	45859	NM_016792	53382	Mm.19169	NM_016792	ENSMUSG00000024583	**Txnl1**	32kDa|TRP32|Txnl	thioredoxin-like 1	LOSS	protein-coding
175_T22	chr14	1.8E+07	2E+07	4999	+	**1**	**12.5**	NA	Intergenic	L1Md_A|LINE|L1	-101156	NM_009455	22194	Mm.4429	NM_009455	ENSMUSG00000021774	**Ube2e1**	UbcM3|Ubce5|ubcM2	ubiquitin-conjugating enzyme E2E 1	LOSS	protein-coding
178_T22	chr14	1.9E+07	2E+07	34999	+	**1**	**12.5**	NA	Intergenic	Lx2|LINE|L1	-63373	NM_144839	218793	Mm.23551	NM_144839	ENSMUSG00000058317	**Ube2e2**	BC016265	ubiquitin-conjugating enzyme E2E 2	LOSS	protein-coding
182_T42	chr14	1.9E+07	2E+07	6999	+	**1**	**12.5**	NA	Intergenic	RMER10A|LTR|ERVL	-274373	NM_144839	218793	Mm.23551	NM_144839	ENSMUSG00000058317	**Ube2e2**	BC016265	ubiquitin-conjugating enzyme E2E 2	LOSS	protein-coding
185_T42	chr14	1.9E+07	2E+07	64999	+	**1**	**12.5**	NA	Intergenic	MIRc|SINE|MIR	-325373	NM_144839	218793	Mm.23551	NM_144839	ENSMUSG00000058317	**Ube2e2**	BC016265	ubiquitin-conjugating enzyme E2E 2	LOSS	protein-coding

177_T4	chr14	1.9E+07	2E+07	54999	+	**1**	**12.5**	NA	intron (NM_144839, intron 3 of 5)	intron (NM_144839, intron 3 of 5)	79627	NM_144839	218793	Mm.23551	NM_144839	ENSMUSG00000058317	**Ube2e2**	BC016265	ubiquitin-conjugating enzyme E2E 2	LOSS	protein-coding
2299_T2	chr2	7E+07	7E+07	3999	+	**1**	**12.5**	NA	intron (NM_001303033, intron 18 of 38)	intron (NM_001303033, intron 18 of 38)	62254	NM_177783	68795	Mm.314576	NM_177783	ENSMUSG00000044308	**Ubr3**	1110059H15Rik|4833421P10Rik|A130030D10Rik|AA409735|AA414972|AA422631|AI646861|AU016126|Zfp650|Znf650	ubiquitin protein ligase E3 component n-recognin 3	LOSS	protein-coding
636_T2	chr16	7.7E+07	8E+07	1999	+	**1**	**12.5**	NA	intron (NM_013918, intron 5 of 23)	B1_Mur4|SINE|Alu	40431	NM_013918	30940	Mm.40986	NM_013918	ENSMUSG00000022867	**Usp25**	-	ubiquitin specific peptidase 25	LOSS	protein-coding
227_T3	chr14	2.2E+07	2E+07	999	+	**1**	**12.5**	NA	Intergenic	Intergenic	-23561	NM_011695	22334	Mm.262327	NM_011695	ENSMUSG00000021771	**Vdac2**	Vdac6|mVDAC2|mVDAC6	voltage-dependent anion channel 2	LOSS	protein-coding
2456_T2	chr8	5.4E+07	5E+07	5999	+	**1**	**12.5**	NA	Intergenic	L1Md_T|LINE|L1	-102032	NM_009506	22341	Mm.1402	NM_009506	ENSMUSG00000031520	**Vegfc**	AW228853|VEGF-C	vascular endothelial growth factor C	LOSS	protein-coding
2461_T32	chr9	7.4E+07	7E+07	4999	+	**1**	**12.5**	NA	Intergenic	Intergenic	-52334	NM_001033500	546144	Mm.335289	NM_177908	ENSMUSG00000044976	**Wdr72**	AW558070|D230040E23	WD repeat domain 72	LOSS	protein-coding
268_T2	chr14	2.9E+07	3E+07	5999	+	**1**	**12.5**	NA	Intergenic	Intergenic	16095	NM_001256224	22418	Mm.287544	NM_009524	ENSMUSG00000021994	**Wnt5a**	8030457G12Rik|Wnt-5a	wingless-type MMTV integration site family, member 5A	LOSS	protein-coding
262_T3	chr14	2.8E+07	3E+07	3999	+	**1**	**12.5**	NA	intron (NM_177814, intron 15 of 15)	intron (NM_177814, intron 15 of 15)	-123973	NM_009524	22418	Mm.287544	NM_009524	ENSMUSG00000021994	**Wnt5a**	8030457G12Rik|Wnt-5a	wingless-type MMTV integration site family, member 5A	LOSS	protein-coding
1257_T1	chr18	4E+07	4E+07	999	+	**1**	**12.5**	NA	Intergenic	L1Md_A|LINE|L1	54399	NM_023311	67180	Mm.270382	NM_023311	ENSMUSG00000024487	**Yipf5**	2610311I19Rik|AA408236|Yip1a	Yip1 domain family, member 5	LOSS	protein-coding
1380_T22	chr18	4.5E+07	4E+07	999	+	**1**	**12.5**	NA	Intergenic	L1Md_T|LINE|L1	157335	NM_001163013	240255	Mm.244482	NM_001163013	ENSMUSG00000034653	**Ythdc2**	3010002F02Rik|BC037178	YTH domain containing 2	LOSS	protein-coding
1969_T32	chr18	7.6E+07	8E+07	999	+	**1**	**12.5**	NA	intron (NM_145356, intron 1 of 4)	L1Md_T|LINE|L1	69822	NM_145356	207259	Mm.159332	NM_145356	ENSMUSG00000044646	**Zbtb7c**	B230208J24Rik|Kr-pok|Zbtb36	zinc finger and BTB domain containing 7C	LOSS	protein-coding
620_T42	chr16	5.4E+07	5E+07	4999	+	**1**	**12.5**	NA	Intergenic	Intergenic	1077237	NM_178720	239852	Mm.245045	NM_178720	ENSMUSG00000064310	**Zpld1**	9430016A21Rik	zona pellucida like domain containing 1	LOSS	protein-coding

Supplementary Table S4. Selection of PPAPA/TP53I Patient Cohort

Breast cancer subtypes are indicated, including HER2+, HR+ and TN;
PTEN and TP53 gene copy number variation (CNV) are listed: 0, no change; -1 & -2, heterozygous or homozygous copy number deletion (CND); 1, heterozygous allele gain.
PTEN and TP53 inactivating and AKT1 and PIK3CA activating mutations (Mut) are listed. NA indicates no mutation.

For gene copy number, the table in allthresholded.bygenes.txt is obtained by applying both low- and hihg-level thresholds to the gene copy levels of all the samples. The Allthresholded.bygenes.txt is the part of the GISTIC output that is used to determine the copy-number status of each gene in each sample in Cbioportal. The entries with value +/- 2 exceed the high-level thresholds for amplifications or deep deletions, and those with +/- 1 exceed the low-level thresholds but not the high-level thresholds.

PPAPA/TP53I Patient Cohort (n=244)

Subtype	SAMPLE_ID	PTEN CNV	TP53 CNV	PTEN Mut	TP53 Mut	AKT1 Mut	PIK3CA Mut	Age at Diagnosis	Overall Survival (Months)	Overall Survival Status	Survival code (1 dead; 0 living)
HER2 +	MB-2895	0	0	NA	R306*	NA	H1047R	66.84	196.57	DECEASED	1
HER2 +	MB-0378	0	-1	NA	S20Qfs*24	NA	H1047R	57.15	97.30	DECEASED	1
HER2 +	MB-5593	0	0	NA	-125fs	NA	H1047R	40.53	92.50	DECEASED	1
HER2 +	MB-5166	0	-1	NA	R280K	NA	E545K	69.23	80.73	DECEASED	1
HER2 +	MB-7020	0	-1	NA	E349_G356 del	NA	H1047R	44.47	71.63	DECEASED	1
HER2 +	MB-7207	-1	-1	NA	P250L	NA	H1047R	43.28	55.73	DECEASED	1
HER2 +	MB-7250	0	0	NA	A83Gfs*66	NA	H1047R	48.45	51.00	DECEASED	1
HER2 +	MB-5292	0	-1	NA	R175H	NA	E545K	82.06	48.43	DECEASED	1
HER2 +	MB-0165	0	-1	NA	H214R	NA	H1047R	53.75	47.63	DECEASED	1
HER2 +	MB-5259	0	-1	NA	R175H	NA	E545K	41.28	46.17	DECEASED	1
HER2 +	MB-5315	0	0	NA	N239D	NA	H1047R	52.13	43.03	DECEASED	1
HER2 +	MB-5483	0	-1	NA	R248W	NA	H1047R	44.5	42.43	DECEASED	1
HER2 +	MB-2758	0	0	NA	V197E	NA	H1047R	38.16	35.00	DECEASED	1
HER2 +	TCGA-A8-A06U-01	-1	-1	NA	R209Kfs*6	NA	H1047R	NA	29.01	DECEASED	1
HER2 +	MB-0373	0	-1	NA	Y234H	NA	H1047R	63.31	27.00	DECEASED	1
HER2 +	MB-2742	0	-1	NA	L111P	NA	H1047R	48.25	24.90	DECEASED	1
HER2 +	MB-0046	0	-1	NA	C135R	NA	H1047R	83.02	14.13	DECEASED	1
HER2 +	MB-4360	-1	-1	NA	G262V	NA	E545K	59.16	11.87	DECEASED	1
HER2 +	TCGA-BH-A18T-01	-2	-1	NA	Y107D	NA	NA	NA	7.36	DECEASED	1
HER2 +	MB-5190	0	-1	NA	R273C	NA	H1047R	53.07	240.00	LIVING	0
HER2 +	MB-4663	0	0	NA	Y220C	NA	H1047R	61.97	239.33	LIVING	0
HER2 +	MB-0236	-2	-1	NA	N239D	NA	NA	43.63	205.03	LIVING	0
HER2 +	MB-5409	0	0	NA	K132E	NA	H1047R	51.62	198.13	LIVING	0
HER2 +	MB-5054	0	0	NA	R248Q	NA	E545K	52.57	187.70	LIVING	0
HER2 +	MB-5458	0	-1	NA	E285K	NA	H1047R	54.96	183.43	LIVING	0
HER2 +	MB-4724	0	-1	NA	I50Mfs*73	NA	H1047R	60.44	146.07	LIVING	0
HER2 +	MB-6334	0	-1	NA	E198*	NA	H1047R	60.12	128.10	LIVING	0
HER2 +	MB-3606	0	-1	NA	Q331Rfs*1 4	NA	E545K	68.61	110.63	LIVING	0
HER2 +	MB-5417	0	0	NA	F54Kfs*67	NA	H1047R	45.8	107.07	LIVING	0
HER2 +	MB-7067	0	0	NA	L194R	NA	H1047R	69.64	105.00	LIVING	0
HER2 +	MB-4763	-1	-1	NA	R248Q	NA	H1047R	44.07	102.10	LIVING	0
HER2 +	TCGA-A2-A04W-01	-1	-1	NA	V272M	NA	H1047R	NA	101.91	LIVING	0
HER2 +	TCGA-A2-A0EQ-01	1	-1	NA	I162_Y163d elinsN	NA	H1047R	NA	79.70	LIVING	0
HER2 +	MB-6114	-1	-1	L57*	Y234C	NA	NA	70.76	66.83	LIVING	0

HER2 +	MB-0152	0	-1	NA	G266Rfs*74	NA	H1047R	63.77	63.03	LIVING	0
HER2 +	MB-4930	0	-1	NA	F134C	NA	H1047R	62.43	58.43	LIVING	0
HER2 +	TCGA-E9-A22E-01	-2	0	NA	G245S	NA	NA	NA	41.69	LIVING	0
HER2 +	TCGA-E9-A1ND-01	-2	-1	NA	Y220C	NA	NA	NA	41.59	LIVING	0
HER2 +	TCGA-BH-A202-01	-1	-1	NA	R213*	NA	H1047R<br>E365V	NA	26.12	LIVING	0
HER2 +	MB-5558	-1	-1	NA	G266V	NA	H1047R	35.13	25.03	LIVING	0
HER2 +	TCGA-A2-A0YG-01	-2	-1	NA	X125_splice	NA	NA	NA	21.88	LIVING	0
HER2 +	TCGA-AC-A23C-01	-1	-1	K183Efs*6	R174Sfs*73	NA	Q546R	NA	19.22	LIVING	0
HER2 +	TCGA-A8-A06R-01	-1	-2	T319*	NA	NA	E545K	NA	17.97	LIVING	0
HER2 +	TCGA-A8-A075-01	1	-1	NA	X187_splice	NA	E545K<br>E453K	NA	17.02	LIVING	0
HER2 +	TCGA-C8-A1HF-01	0	-1	NA	H179Y	NA	H1047R	NA	10.91	LIVING	0
HER2 +	TCGA-AN-A0AJ-01	-1	-1	NA	G108Vfs*15	NA	H1047R	NA	9.95	LIVING	0
HR+	MB-3545	0	0	NA	D393Tfs*29	NA	H1047R	49.84	232.73	DECEASED	1
HR+	MB-3388	-1	0	NA	R249M	NA	E545K	59.3	228.60	DECEASED	1
HR+	MB-6044	0	0	NA	G334V	NA	H1047R	70.64	219.10	DECEASED	1
HR+	TCGA-B6-A0RH-01	0	-1	NA	Y220S	NA	E545K	NA	212.09	DECEASED	1
HR+	MB-5272	0	0	NA	H193Y	NA	E545K	40.71	201.93	DECEASED	1
HR+	MB-4343	1	-1	NA	R342*	NA	H1047R<br>H1048R	68.09	198.60	DECEASED	1
HR+	MB-6184	1	-1	NA	R280K	NA	H1047R	35.02	185.70	DECEASED	1
HR+	MB-5228	0	-1	NA	X225_splice	E17K	NA	72.56	180.73	DECEASED	1
HR+	MB-5397	-1	-1	NA	Y163C	NA	E545K	61.16	172.87	DECEASED	1
HR+	MB-4426	-1	-1	NA	P153Afs*28	NA	H1047R	70.84	170.90	DECEASED	1
HR+	MB-5045	-2	-1	NA	A74Pfs*49	NA	NA	70.11	168.30	DECEASED	1
HR+	MB-3295	-1	0	NA	P278R	NA	H1047R<br>E365K	60.42	158.53	DECEASED	1
HR+	MB-2846	0	-1	NA	Y234N	NA	H1047R	58.85	149.70	DECEASED	1
HR+	MB-2858	0	-1	NA	R267P	NA	H1047R	63.94	141.57	DECEASED	1
HR+	MB-0102	0	0	NA	R175H	E17K	NA	51.38	140.77	DECEASED	1
HR+	MB-5288	0	-1	NA	E285K	NA	E545K<br>M1043I	70.1	128.40	DECEASED	1
HR+	MB-5614	0	-1	NA	X187_splice	NA	H1047R	62.87	116.43	DECEASED	1
HR+	MB-0109	-1	-1	X70_splice	C277F	NA	NA	82.53	112.40	DECEASED	1
HR+	MB-7196	0	0	NA	X224_splice	NA	H1047R	67.99	107.10	DECEASED	1
HR+	MB-0609	0	0	NA	R249S	E17K	NA	51.15	98.70	DECEASED	1
HR+	MB-0884	0	-1	NA	V218Hfs*5	NA	H1047R	56.47	93.67	DECEASED	1
HR+	MB-5584	-1	-1	NA	P301Qfs*44	NA	H1047R	69.55	91.10	DECEASED	1

HR+	MB-2617	0	-1	NA	H179R	NA	H1047R P471L	63.3	89.10	DECEASED	1
HR+	MB-3536	0	-1	NA	Q192*	NA	H1047R	48.12	86.23	DECEASED	1
HR+	MB-3526	0	-1	NA	R282W	NA	H1047R	60.3	86.07	DECEASED	1
HR+	MB-6135	0	-1	NA	K305Sfs*40	NA	H1047R	69.38	78.60	DECEASED	1
HR+	MB-6007	0	-1	NA	R175H	NA	H1047R E726K	70.65	70.53	DECEASED	1
HR+	MB-6179	0	-1	NA	G245S	NA	H1047R	59.08	58.47	DECEASED	1
HR+	MB-6071	0	0	NA	R110H	NA	H1047R	72.81	57.40	DECEASED	1
HR+	MB-6204	0	-1	NA	H179R	NA	H1047R	41.08	56.77	DECEASED	1
HR+	MB-3253	0	0	NA	V272M	NA	H1047R	56.81	55.83	DECEASED	1
HR+	MB-6194	0	-1	NA	R280K	NA	H1047R K111N	44.51	52.47	DECEASED	1
HR+	MB-7072	-2	0	NA	E349*	NA	NA	71.24	52.33	DECEASED	1
HR+	MB-5338	-1	-1	NA	S215G	NA	H1047R	61.24	51.67	DECEASED	1
HR+	MB-3266	-1	-1	NA	Y220C	NA	H1047R	52.91	51.20	DECEASED	1
HR+	MB-0506	0	-1	NA	C135W	NA	H1047R	60.74	45.80	DECEASED	1
HR+	MB-5329	0	-1	NA	R280T	NA	H1047R	64.64	45.73	DECEASED	1
HR+	MB-0398	0	-1	NA	D148Hfs*31	NA	H1047R	56.41	43.20	DECEASED	1
HR+	MB-0167	1	0	D92E	R282W	NA	E545K	76.4	43.10	DECEASED	1
HR+	MB-0306	0	-1	NA	G266E	NA	H1047R E726K	82.53	42.97	DECEASED	1
HR+	MB-6192	0	0	NA	E287Ifs*63	NA	H1047R	78.45	42.63	DECEASED	1
HR+	MB-0353	0	-1	NA	Q136H	NA	H1047R G118D	86.26	41.83	DECEASED	1
HR+	MB-5243	-2	-1	NA	G262_N263 del	NA	NA	62.79	38.43	DECEASED	1
HR+	MB-5019	-1	-1	NA	S183*	NA	H1047R	49.88	37.80	DECEASED	1
HR+	MB-5513	0	-1	NA	R209Kfs*6	NA	H1047R	86.55	36.63	DECEASED	1
HR+	MB-4887	0	-1	NA	I255Nfs*9	NA	H1047R	52.09	35.40	DECEASED	1
HR+	MB-5543	0	-1	NA	Y107_F109 del	NA	E545K	79.11	34.43	DECEASED	1
HR+	MB-0636	-2	-1	NA	R273H	NA	NA	26.36	32.63	DECEASED	1
HR+	MB-2953	1	-1	NA	Y234C	NA	H1047R	43.08	30.13	DECEASED	1
HR+	MB-6200	0	-1	NA	R213*	NA	H1047R	65.52	30.07	DECEASED	1
HR+	MB-0601	0	-1	NA	Y126C	E17K	NA	32.99	29.67	DECEASED	1
HR+	TCGA-A2-A0SV-01	-1	-1	H64_Y65del	F113_H115 del	NA	NA	NA	27.10	DECEASED	1
HR+	TCGA-A2-A0YT-01	0	-1	NA	I255S	NA	E545K	NA	23.75	DECEASED	1
HR+	TCGA-E2-A14Z-01	-1	-1	D252Rfs*42	R282W	NA	NA	NA	18.50	DECEASED	1
HR+	MB-5482	0	-1	NA	R337C	NA	H1047R E365K	40.75	17.67	DECEASED	1
HR+	MB-4649	0	-1	NA	Q331*	NA	H1047R	61.67	16.83	DECEASED	1
HR+	MB-5520	0	-1	NA	C141Y	NA	H1047R	38.49	16.30	DECEASED	1
HR+	MB-5431	-2	-1	NA	A83Pfs*35	NA	NA	49.91	15.87	DECEASED	1

HR+	MB-6019	0	-1	NA	R248Q	NA	H1047R E110del Y1021C	67	13.53	DECEASED	1
HR+	MB-2964	0	-1	NA	R248W	NA	E545K	69.48	9.60	DECEASED	1
HR+	MB-0010	0	-1	NA	P67Qfs*56	NA	H1047R	78.77	7.80	DECEASED	1
HR+	MB-5179	0	-1	NA	R213*	NA	H1047R I459T	68.6	240.00	LIVING	0
HR+	MB-4712	-1	-1	NA	C176Y	NA	H1047R	46.59	240.00	LIVING	0
HR+	MB-5130	0	-1	NA	R280T	NA	H1047R	58.61	240.00	LIVING	0
HR+	MB-4281	0	-1	NA	L194R	NA	E545K	60	240.00	LIVING	0
HR+	MB-2779	0	-1	NA	G245C	NA	E545K	36.8	240.00	LIVING	0
HR+	MB-4079	0	-1	NA	V274L	NA	E545K	63.17	240.00	LIVING	0
HR+	MB-4860	0	-1	NA	R175H	NA	E545K	44.95	234.60	LIVING	0
HR+	MB-6010	0	-1	NA	R175H	E17K	NA	70.28	218.23	LIVING	0
HR+	MB-4949	0	-1	NA	R175H	NA	H1047R	69.75	216.97	LIVING	0
HR+	MB-5004	-1	-1	Y76*	I255del	NA	NA	42.84	216.73	LIVING	0
HR+	MB-7293	0	-1	NA	I255S	NA	E545K	56.9	199.23	LIVING	0
HR+	MB-5502	0	-1	NA	A159D	NA	E545K	51.62	189.73	LIVING	0
HR+	MB-4300	0	0	NA	R209Kfs*6	E17K	NA	70.58	189.03	LIVING	0
HR+	MB-4005	-2	-1	NA	R175H	NA	NA	66.37	188.37	LIVING	0
HR+	MB-3874	-1	0	Y336*	R110H	NA	E545K	35.5	186.43	LIVING	0
HR+	MB-0325	0	-1	NA	R213Dfs*34	NA	H1047R	79.34	177.53	LIVING	0
HR+	MB-5511	0	-1	NA	C238Y	NA	H1047R E81A	54.18	158.97	LIVING	0
HR+	MB-5533	0	0	NA	E171*	NA	H1047R	71.64	158.03	LIVING	0
HR+	MB-2844	0	0	NA	I162delinsRL I162_Y163insC I162M	NA	H1047R	60.76	146.73	LIVING	0
HR+	MB-0172	0	-2	NA	R110P	NA	E545K H1047R	48.11	138.10	LIVING	0
HR+	MB-7150	0	-1	NA	R175H	NA	E545K	70.88	128.97	LIVING	0
HR+	MB-0202	0	-1	NA	D281H	E17K	NA	46	128.37	LIVING	0
HR+	MB-0574	0	-1	NA	Y236C	NA	E545K	71.21	119.80	LIVING	0
HR+	MB-6225	0	-1	NA	I254Sfs*91	NA	E545K	68.43	117.87	LIVING	0
HR+	TCGA-GM-A2DL-01	0	-1	NA	D281E	E17K	NA	NA	115.60	LIVING	0
HR+	MB-0150	-1	-1	D92A	K132N	NA	N345K	70.22	111.20	LIVING	0
HR+	MB-7091	0	-1	NA	Y236C	NA	H1047R	55.71	110.27	LIVING	0
HR+	MB-7070	0	-1	NA	N239_S240del	NA	H1047R	68.76	108.07	LIVING	0
HR+	TCGA-AR-A24T-01	0	-1	NA	R273H	NA	H1047R	NA	105.19	LIVING	0
HR+	TCGA-AR-A1AJ-01	-1	-1	S10R	R273H	NA	NA	NA	100.92	LIVING	0
HR+	TCGA-A2-A0CL-01	-2	0	NA	R273L	NA	NA	NA	99.05	LIVING	0
HR+	MB-7048	-2	-1	NA	R110L	NA	NA	36.27	88.47	LIVING	0
HR+	TCGA-AR-A1AW-01	1	1	NA	R273C	NA	H1047R	NA	86.47	LIVING	0
HR+	TCGA-BH-A0B0-01	0	-1	NA	R175H	NA	E545K	NA	81.37	LIVING	0
HR+	TCGA-BH-A0DL-01	0	-1	NA	E286A	NA	H1047R	NA	78.22	LIVING	0
HR+	MB-7029	0	-1	NA	Q331*	NA	E545K	43.45	76.87	LIVING	0
HR+	TCGA-BH-A0BR-01	0	-1	NA	V274F	E17K	NA	NA	76.54	LIVING	0

HR+	TCGA-E2-A14Y-01	-1	-1	NA	Q136E	NA	H1047R	NA	69.28	LIVING	0
HR+	TCGA-A2-A3Y0-01	-2	-1	NA	R342*	NA	NA	NA	50.79	LIVING	0
HR+	MB-0358	-1	-1	NA	X225_splice	NA	H1047R	57.87	32.87	LIVING	0
HR+	TCGA-A8-A092-01	1	-1	NA	C238Y	NA	H1047R	NA	30.95	LIVING	0
HR+	TCGA-A7-A0CJ-01	-2	-1	NA	R175H	NA	Q546R	NA	30.58	LIVING	0
HR+	TCGA-BH-A0GY-01	0	0	NA	R175H	NA	H1047R	NA	30.32	LIVING	0
HR+	TCGA-A7-A26H-01	0	-2	NA	NA	NA	H1047R	NA	23.78	LIVING	0
HR+	TCGA-A1-A0SI-01	0	-1	NA	R175H	NA	H1047R<br>E726K	NA	20.86	LIVING	0
HR+	TCGA-E2-A15E-01	0	-1	NA	H193R	NA	H1047R	NA	20.70	LIVING	0
HR+	TCGA-C8-A26V-01	-1	-1	NA	I232S	E17K	NA	NA	20.24	LIVING	0
HR+	TCGA-D8-A1JK-01	-1	-1	NA	X331_splice	NA	E545K	NA	20.11	LIVING	0
HR+	TCGA-D8-A1JJ-01	0	-1	NA	C141Y	NA	H1047R	NA	20.07	LIVING	0
HR+	MB-5590	0	0	NA	S127F	NA	E545K	64.93	17.83	LIVING	0
HR+	TCGA-A7-A4SA-01	-2	-1	NA	P151A	NA	NA	NA	14.91	LIVING	0
HR+	TCGA-AC-A3OD-01	0	-1	NA	Q144*	NA	E545K	NA	14.82	LIVING	0
HR+	TCGA-A8-A06O-01	-1	-1	E288Rfs*7	G279E	NA	NA	NA	13.01	LIVING	0
HR+	TCGA-C8-A26W-01	-1	-1	NA	R273L	NA	H1047R	NA	12.52	LIVING	0
HR+	TCGA-C8-A1HM-01	-2	-1	NA	L194P	NA	NA	NA	12.32	LIVING	0
HR+	TCGA-C8-A130-01	1	-1	NA	N131Tfs*39	NA	H1047R	NA	12.16	LIVING	0
HR+	TCGA-A8-A08I-01	-2	0	NA	Y163C	NA	NA	NA	11.99	LIVING	0
HR+	TCGA-EW-A1PC-01	1	-1	NA	R273C	NA	H1047R	NA	6.14	LIVING	0
HR+	MB-7186	-1	-2	NA	NA	E17K	NA	48.92	4.87	LIVING	0
HR+	MB-5525	0	-1	NA	R248Q	NA	H1047R	63.2	2.00	LIVING	0
HR+	TCGA-A8-A06Q-01	0	-2	NA	C238Y	NA	H1047R	NA	1.02	LIVING	0
HR+	TCGA-A8-A081-01	0	-1	NA	C141Y	NA	H1047R	NA	0.10	LIVING	0
HR+	TCGA-A8-A094-01	-1	-1	NA	R209Kfs*6	E17K	NA	NA	0.10	LIVING	0
TN	MB-5223	-2	-1	NA	Y234C	NA	NA	42.05	144.67	DECEASED	1
TN	MB-6318	0	-1	NA	Q331*	E17K	NA	81.37	109.23	DECEASED	1
TN	MB-7154	-2	0	NA	R273H	M227T	NA	77.54	108.47	DECEASED	1
TN	MB-7045	0	-1	NA	R306*	E17K	NA	61.39	94.93	DECEASED	1
TN	MB-0436	-1	-1	NA	R273H	E17K	NA	88.29	75.50	DECEASED	1
TN	MB-4993	0	-1	NA	R174Sfs*67	E17K	NA	33.55	75.23	DECEASED	1
TN	TCGA-B6-A0IE-01	1	-1	NA	X307_splice	NA	H1047R	NA	65.47	DECEASED	1
TN	MB-0608	-1	-1	I33del	R175H	NA	NA	76.81	63.97	DECEASED	1
TN	MB-0906	0	0	NA	L111P	NA	H1047R<br>G118D	70.6	63.87	DECEASED	1
TN	MB-5634	-2	-1	NA	N239*	NA	NA	55.53	62.90	DECEASED	1
TN	MB-5577	0	-1	NA	V216M	E17K	NA	68.64	55.40	DECEASED	1
TN	MB-5072	-1	-1	NA	N239I	NA	H1047R<br>E542A	52.23	50.23	DECEASED	1
TN	MB-0292	-1	-1	NA	I195Yfs*14	NA	E545K	80.34	49.43	DECEASED	1
TN	MB-0211	0	-1	NA	R248Q	NA	E545K	42.95	44.80	DECEASED	1
TN	MB-7057	0	-1	NA	S241F	E17K	NA	72.81	43.83	DECEASED	1
TN	MB-5213	0	0	NA	N131I	NA	H1047R	74.98	43.40	DECEASED	1

TN	MB-0464	1	-1	NA	E285K	E17K	NA	58.98	39.30	DECEASED	1
TN	MB-5138	-1	-1	NA	R273H	NA	H1047R	44.59	37.00	DECEASED	1
TN	MB-0494	-1	-1	NA	P278S	NA	E545K	77.22	36.63	DECEASED	1
TN	MB-7252	0	0	NA	F113del	NA	E545K	74.8	35.70	DECEASED	1
TN	MB-3277	-2	-1	NA	R273C	NA	NA	29.92	32.93	DECEASED	1
TN	MB-3453	0	-1	NA	G262V	NA	E545K	51.36	32.83	DECEASED	1
TN	MB-2917	-2	-1	NA	X126_splice	NA	NA	33.9	31.93	DECEASED	1
TN	MB-4911	-2	0	NA	C238Y	NA	NA	43.51	31.43	DECEASED	1
TN	MB-7023	0	0	NA	H193R	NA	H1047R	66.66	27.97	DECEASED	1
TN	MB-0221	-1	-1	X343_splice	V197G	NA	H1047R	77.72	20.20	DECEASED	1
TN	MB-4733	0	0	NA	G154S	NA	H1047R	56.27	19.73	DECEASED	1
TN	MB-4982	0	0	NA	S166Hfs*4	NA	H1047R	62.99	19.17	DECEASED	1
TN	TCGA-B6-A0IK-01	-1	-1	NA	L194R	NA	H1047R	NA	18.76	DECEASED	1
TN	TCGA-AR-A1AR-01	-2	-1	NA	S90Pfs*33	NA	NA	NA	17.21	DECEASED	1
TN	MB-5299	-2	-1	NA	C176S	NA	Y361C	53.35	16.70	DECEASED	1
TN	MB-0259	-2	-1	NA	R273H	NA	NA	38.99	11.87	DECEASED	1
TN	MB-0354	-1	-1	NA	H178P	NA	H1047R	73.01	11.60	DECEASED	1
TN	TCGA-E2-A1LK-01	-2	1	NA	A86Cfs*63	NA	NA	NA	8.74	DECEASED	1
TN	TCGA-A2-A0T2-01	-2	0	NA	Y220C	NA	NA	NA	8.38	DECEASED	1
TN	MB-0581	-1	-1	NA	L194R	NA	H1047R	96.29	7.87	DECEASED	1
TN	TCGA-EW-A1P8-01	-2	-1	NA	R282W	NA	NA	NA	7.85	DECEASED	1
TN	TCGA-B6-A0RU-01	-2	-1	NA	R196*	NA	NA	NA	240.00	LIVING	0
TN	MB-4667	0	-1	NA	E271K	E17K	NA	65.58	240.00	LIVING	0
TN	MB-5392	0	0	NA	M246V	NA	H1047R	57.34	237.27	LIVING	0
TN	MB-3567	-1	-1	C71F	Y126N	NA	NA	55.95	236.03	LIVING	0
TN	MB-6152	-1	-1	NA	P278S	NA	H1047R	60.62	228.90	LIVING	0
TN	MB-3153	-1	-1	G44Afs*10	S241F	NA	NA	42.17	227.93	LIVING	0
TN	MB-3396	-1	-1	S10_K13del	A69Vfs*54	NA	C420R	59.29	226.73	LIVING	0
TN	MB-5008	-1	-1	Y346D	X225_splice	NA	NA	65.76	223.30	LIVING	0
TN	MB-5346	-1	-1	Y176C	V173G	NA	NA	52.09	194.30	LIVING	0
TN	MB-5450	-1	-1	I122F	C141Y	NA	NA	38.92	190.20	LIVING	0
TN	MB-0249	-1	-1	NA	Y205S	NA	H1047R	53.45	188.33	LIVING	0
TN	MB-3123	-1	-1	NA	S241Y	NA	E545K	50.26	167.43	LIVING	0
TN	MB-7205	-1	-1	S370Tfs*45	R273H	NA	NA	38.17	166.03	LIVING	0
TN	MB-6242	-1	-1	F215_Q219 del	X225_splice	NA	NA	41.29	165.43	LIVING	0
TN	MB-7119	-2	-1	NA	R110Pfs*39	NA	NA	54.53	128.37	LIVING	0
TN	MB-7090	-1	-1	NA	E294*	NA	E545K	48.52	109.77	LIVING	0
TN	MB-0174	-2	0	NA	G154Afs*16	NA	NA	59.18	78.77	LIVING	0
TN	TCGA-GM-A2DD-01	-1	-1	NA	R175H	NA	H1047R	NA	74.97	LIVING	0
TN	MB-0446	-2	0	NA	C141R X126_splice	NA	NA	56.96	72.27	LIVING	0

TN	TCGA-GM-A3XL-01	-2	-1	NA	R156del	NA	NA	NA	69.25	LIVING	0
TN	MB-0525	-1	-1	F341Lfs*3	R110P	NA	NA	48.47	65.87	LIVING	0
TN	TCGA-E2-A14N-01	-2	0	NA	R196*	NA	NA	NA	47.11	LIVING	0
TN	TCGA-BH-A0B3-01	-2	-1	NA	V216M	NA	NA	NA	39.52	LIVING	0
TN	TCGA-E2-A573-01	-2	-1	NA	Y220C	NA	NA	NA	34.89	LIVING	0
TN	TCGA-A2-A0D2-01	-2	0	NA	P318Tfs*15	NA	NA	NA	33.74	LIVING	0
TN	TCGA-AR-A1AY-01	-2	-1	NA	V122Dfs*2 6	NA	NA	NA	33.71	LIVING	0
TN	TCGA-AQ-A04J-01	-2	-1	NA	L111P	NA	NA	NA	26.91	LIVING	0
TN	TCGA-E2-A159-01	-2	-1	NA	V216M	NA	NA	NA	25.03	LIVING	0
TN	TCGA-A7-A26G-01	-1	-1	I135K	Y220H	NA	NA	NA	23.72	LIVING	0
TN	TCGA-A7-A4SD-01	-2	-1	NA	M237I	NA	NA	NA	14.49	LIVING	0
TN	TCGA-D8-A143-01	0	1	NA	X331_splice	NA	H1047R	NA	14.16	LIVING	0
TN	TCGA-C8-A131-01	0	-1	NA	R175H	NA	H1047R R108H	NA	13.50	LIVING	0
TN	TCGA-C8-A134-01	-2	-1	NA	R273H	NA	NA	NA	12.58	LIVING	0
TN	TCGA-D8-A1JF-01	-1	1	NA	H179R	NA	E545K R398H	NA	12.02	LIVING	0
TN	TCGA-C8-A12L-01	-1	-1	NA	C238F	NA	H1047R	NA	11.93	LIVING	0
TN	TCGA-AN-A0AL-01	-1	-1	V9Kfs*7	R175H	NA	NA	NA	7.46	LIVING	0
TN	TCGA-AN-A0XU-01	-2	-1	NA	C275Lfs*67	NA	NA	NA	0.33	LIVING	0

Supplementary Table S5. The Genes that Are Altered in both Mouse PtenI/Tp53I Mammary Tumors (n = 8) and Human PAPPA/TP53I Breast Cancer Cohort (n = 244)

Part I. The Genes Commonly Mutated by NSPMs/InDels in Mouse PtenI/Tp53I Tumors and Human PAPPA/TP53I Breast Cancer Cohort

Gene name	% in mouse tumors	% in human BCs
KMT2C	12.5	10.66
KMT2D	12.5	6.15
NOTCH1	25	2.87
COL22A1	12.5	2.87
MUC4	62.5	2.46
PTPRM	25	2.05
PCLO	25	1.64
STK11	25	1.23
ABCA12	12.5	1.23
BRWD1	12.5	1.23
CSMD1	12.5	1.23
FANCA	12.5	1.23
OBSCN	12.5	1.23
CHD4	100	0.82
NOP2	100	0.82
VWF	100	0.82
PCDHB14	75	0.82
PCDHB4	75	0.82
MUC5B	62.5	0.82
ITPR3	50	0.82
NEB	37.5	0.82
ABCA9	12.5	0.82
CPSF1	12.5	0.82
DMXL2	12.5	0.82
ERGIC3	12.5	0.82
FGD5	12.5	0.82
HRG	12.5	0.82
IGF2R	12.5	0.82
ITPR2	12.5	0.82

Part II. The Genes Commonly Mutated by CNAs in Mouse PtenI/Tp53I Tumors and Human PAPPA/TP53I Breast Cancer Cohort

Gene name	% in mouse tumors	% in human BCs
MYC	25	37.23
TRPS1	100	35.52
MED30	12.5	35.23
CSMD3	100	33.82
HAS2	87.5	33.82
KHDRBS3	50	33.23
ADCY8	12.5	32.93
NSMCE2	62.5	32.70
EFR3A	100	32.23
FAM135B	37.5	32.23
KCNK9	25	32.23
PKHD1L1	100	31.64
TMEM74	100	31.64
KCNV1	87.5	31.64
ANGPT1	50	31.64
EBAG9	50	31.64
RSPO2	12.5	31.64
SDC2	100	28.00
MMP16	100	25.34
CNGB3	37.5	24.75
RALYL	62.5	24.64
ZC2HC1A	87.5	24.16
RMDN1	37.5	24.16
PEX2	25	22.46
HNF4G	100	21.23
NKAIN3	25	20.52
YTHDF3	100	19.93
SNTG1	12.5	17.75
COX20	87.5	15.23

Part III. The Genes Commonly Mutated by CNDs in Mouse PtenI/Tp53I Tumors and Human PAPPA/TP53I Breast Cancer Cohort

Gene name	% in mouse tumors	% in human BCs
POLR3A	25	8.59
SH2D4B	62.5	7.89
ANXA11	37.5	7.48
SFTPD	12.5	7.48
TSPAN14	37.5	7.18
ADK	75	6.59
DYDC2	12.5	6.48
AP3M1	62.5	6.30
KAT6B	62.5	6.30
VCL	50	6.30
SAMD8	37.5	6.30
COMTD1	25	6.30
VDAC2	25	6.30
DUPD1	12.5	6.30
SOS2	12.5	5.76
CAMK2G	37.5	5.41
KLRC2	37.5	5.30
KLRC3	25	5.30
ANTXRL	12.5	5.06
PPP3CB	75	4.70
USP54	75	4.70
MRPS16	62.5	4.70
MYOZ1	62.5	4.70
MSS51	50	4.70
NUDT13	50	4.70
MMRN2	62.5	3.89
SNCG	37.5	3.89
BMPR1A	75	3.81
USP25	25	3.65

JUN	12.5	0.82	RHOU	62.5	15.23	NRG3	12.5	3.56
KRT84	12.5	0.82	CFH	25	12.64	CADM2	12.5	3.39
NLRC5	12.5	0.82	BRINP3	100	11.93	LDB3	50	3.22
TCHH	12.5	0.82	RGS18	100	11.75	RBM11	25	3.22
TRPV2	12.5	0.82	B3GALT2	75	11.75	RTTN	100	3.21
UNC80	12.5	0.82	PLA2G4A	100	11.64	SOCS6	100	3.21
A2M	100	0.41	PDC	12.5	11.52	CD226	87.5	3.21
ANKRD26	100	0.41	NLGN1	100	10.89	DOK6	75	3.21
ATP2B2	100	0.41	SLC7A14	12.5	10.30	FOXP1	12.5	2.97
CACNA1C	100	0.41	SPRR2B	12.5	10.11	SYT15	87.5	2.94
KLRB1	100	0.41	PDCD10	87.5	10.00	NPY4R	37.5	2.94
NAV2	100	0.41	PGLYRP3	62.5	10.00	GPRIN2	25	2.94
SFI1	100	0.41	PGLYRP4	12.5	10.00	DSEL	12.5	2.91
TTLL3	100	0.41	SERPINI2	12.5	10.00	VEGFC	12.5	2.91
BNC2	87.5	0.41	ZBBX	12.5	10.00	TMX3	87.5	2.62
SP140	75	0.41	HRNR	87.5	9.41	TIMM21	100	2.49
PHC2	62.5	0.41	FLG2	50	9.41	PARD6G	87.5	2.49
GNAI2	50	0.41	STX16	100	9.30	ATP9B	75	2.49
FAM83C	37.5	0.41	MBNL1	25	9.30	CBLN2	75	2.49
THOC1	37.5	0.41	SUCNR1	37.5	8.41	SALL3	75	2.49
ANO8	25	0.41	IGSF10	25	8.41	FBXO15	62.5	2.49
CDH13	25	0.41	CRP	12.5	8.41	NETO1	62.5	2.49
CIT	25	0.41	PFN2	100	8.11	ADNP2	50	2.49
DSCAML1	25	0.41	ANKUB1	87.5	8.11	TSHZ1	50	2.49
DTWD2	25	0.41	UNC5D	87.5	8.07	CYB5A	37.5	2.49
PCDHB7	25	0.41	SLITRK3	100	8.00	GPM6A	37.5	2.49
PPP1R26	25	0.41	FCRL5	12.5	8.00	KCNG2	37.5	2.49
TACC3	25	0.41	PDZK1	12.5	8.00	NFATC1	37.5	2.49
UBAP2	25	0.41	FGF12	12.5	7.89	ZADH2	37.5	2.49
UTP20	25	0.41	TM4SF1	100	7.88	AGA	25	2.49
ABL2	12.5	0.41	CTNND2	50	7.70	CTNNA1	100	2.19
BPNT1	12.5	0.41	GPR52	25	7.70	WDR7	100	2.19
C1QTNF7	12.5	0.41	MLF1	25	7.70	CCSER2	75	2.19
CADM1	12.5	0.41	RSRC1	12.5	7.70	GHITM	62.5	2.19
DNAH17	12.5	0.41	WWTR1	100	7.52	RGR	12.5	2.19
FLNB	12.5	0.41	TSC22D2	100	7.41	DCC	100	2.03
FNTB	12.5	0.41	NKAIN2	12.5	7.18	MC4R	100	2.03
GABRE	12.5	0.41	TM4SF4	100	7.17	NEDD4L	50	2.03
GPR179	12.5	0.41	DNAH5	25	6.59	THOC7	75	1.67
LAMP2	12.5	0.41	DOK5	12.5	6.48	MAPK8	37.5	1.67
LCP2	12.5	0.41	CD300LD	100	6.47	FEZF2	12.5	1.67

MAP1A	12.5	0.41	CDH18	87.5	6.30	LRRC18	12.5	1.67
MLC1	12.5	0.41	SGCZ	100	6.18	PTPRG	12.5	1.67
OSM	12.5	0.41	OTOL1	25	6.11	SNTN	12.5	1.67
PTPRO	12.5	0.41	LUC7L	100	6.05	TTBK2	12.5	1.62
REV1	12.5	0.41	GPR158	25	6.00	PPP2R2B	50	1.61
SHCBP1L	12.5	0.41	MSR1	100	5.95	CACNA2D3	12.5	1.61
SLC25A53	12.5	0.41	ZIC1	12.5	5.76	CCDC68	87.5	1.60
TIMELESS	12.5	0.41	POTEG	12.5	5.64	LRRTM2	87.5	1.60
YBX3	12.5	0.41	KIF2B	100	5.41	MALT1	87.5	1.60
ZBTB39	12.5	0.41	MACROD2	25	5.41	PMAIP1	87.5	1.60
			NFIA	12.5	5.35	RAB27B	75	1.60
			CDH10	100	5.30	ME2	50	1.60
			RGS17	75	5.30	MEX3C	50	1.60
			PRH1	25	5.30	ONECUT2	50	1.60
			HACE1	12.5	5.30	SEC11C	50	1.60
			THNSL1	12.5	5.30	DYNAP	37.5	1.60
			TEX15	37.5	5.18	FECH	37.5	1.60
			SUGCT	50	5.06	OGDHL	37.5	1.60
			ST8SIA2	12.5	5.06	RAX	37.5	1.60
			GHR	62.5	4.70	SMAD4	37.5	1.60
			FGF10	12.5	4.70	STARD6	37.5	1.60
			MYO3A	12.5	4.70	ALPK2	12.5	1.60
			TMEM126B	12.5	4.64	CPS1	12.5	1.60
			CNTNAP2	100	4.59	MAP2	12.5	1.60
			CDH6	87.5	4.59	ASB14	87.5	1.26
			RNF217	87.5	4.59	ARHGEF3	75	1.26
			FOXD4	87.5	4.48	PDE12	75	1.26
			POP4	62.5	4.40	SLMAP	75	1.26
			CACNG3	12.5	4.23	FHIT	62.5	1.26
			LIPI	37.5	4.11	FLNB	12.5	1.26
			CDH12	100	4.00	GRID2	12.5	1.26
			CDH9	100	4.00	TMF1	12.5	1.26
			DAB2	87.5	4.00	TXNL1	100	1.25
			CST3	100	3.94	TCF4	75	1.25
			AGMO	87.5	3.94	MBD2	62.5	1.25
			HS3ST5	100	3.89	PBRM1	62.5	1.25
			GJA1	12.5	3.89	ST8SIA3	62.5	1.25
			LRRC4C	100	3.81	PARG	50	1.25
			TYRP1	37.5	3.81	SMIM4	50	1.25
			GRM7	25	3.81	MAPK4	37.5	1.25
			DCLK1	100	3.69	GRID1	25	1.25

DZIP3	100	3.69
PTPRD	75	3.69
MAGI2	100	3.65
VSTM2A	50	3.65
TRIML1	12.5	3.56
INHBA	37.5	3.52
FRK	62.5	3.39
PCDH15	62.5	3.39
TKTL2	62.5	3.39
CADM2	12.5	3.39
ARHGAP5	75	3.30
NCAM2	12.5	3.30
ANXA10	100	3.26
GALNTL6	100	3.26
TMEM196	100	3.23
ETV1	75	3.23
EPHA6	87.5	3.22
LRFN5	75	3.22
FLRT3	50	3.22
HGF	12.5	3.22
LURAP1L	12.5	3.22
CDH7	100	2.97
FSTL5	100	2.97
MAB21L1	100	2.97
CRIM1	87.5	2.97
CTSO	87.5	2.97
KCNA4	87.5	2.94
NT5C1B	37.5	2.94
PHF3	37.5	2.94
DSEL	100	2.91
CDH20	87.5	2.91
VEGFC	62.5	2.91
GRIK2	100	2.87
LRRC7	62.5	2.87
ATP8A2	50	2.87
STEAP1	50	2.87
STXBP6	50	2.87
NOVA1	37.5	2.87
BTBD3	25	2.87
NXPH1	100	2.82
EEPD1	12.5	2.82

MRO	25	1.25
NT5DC2	25	1.25
PHF7	12.5	1.25
OAZ1	12.5	1.18
COMMD10	75	0.85
GLT8D1	50	0.84
NEK4	37.5	0.84
ITIH4	25	0.84
TKT	25	0.84
LRTM1	12.5	0.84
WAPL	100	0.83
SLC25A46	50	0.83

FGF14	100	2.80
FUT9	100	2.80
MANEA	100	2.80
PCLO	50	2.80
DNAJB9	37.5	2.80
RAG2	37.5	2.80
LRRN3	25	2.80
CD200	12.5	2.80
STEAP4	12.5	2.80
UFL1	12.5	2.80
NBEA	100	2.62
SERTM1	100	2.62
PDGFC	62.5	2.62
GRIA2	12.5	2.62
RAPGEF2	12.5	2.62
LARGE1	25	2.53
MAP1S	25	2.53
JAG1	12.5	2.53
TRPC6	12.5	2.53
AGA	100	2.49
SPOCK3	100	2.49
TENM3	37.5	2.49
ADAM29	25	2.49
GPM6A	25	2.49
FBXO8	12.5	2.49
CNTN5	100	2.46
CHORDC1	87.5	2.46
FAF1	87.5	2.46
KCND2	87.5	2.46
DLGAP1	75	2.46
ALKBH5	62.5	2.46
TECRL	12.5	2.46
CFTR	100	2.45
TES	100	2.45
TFEC	100	2.45
TNFRSF19	100	2.45
WNT2	100	2.45
HCK	87.5	2.45
BDNF	50	2.45
FOXP2	25	2.45
METTL15	25	2.45

MTPN	25	2.45
SLITRK5	25	2.45
GRM5	12.5	2.45
ERBB4	100	2.38
CNTN6	62.5	2.38
PDGFD	62.5	2.38
CHL1	50	2.38
MMS22L	37.5	2.38
DDI1	12.5	2.38
ADCYAP1	100	2.11
LRP1B	100	2.07
SELENOT	100	2.07
HAO1	87.5	2.04
GABRB1	12.5	2.04
CTTNBP2	100	2.03
EPHA5	100	2.03
PABPC4L	100	2.03
SPANXN4	100	2.03
KCTD8	75	2.03
LLGL1	62.5	2.03
NRAP	62.5	2.03
SORCS3	62.5	2.03
EFNB2	50	2.03
GABRA1	50	2.03
ETS2	25	2.03
LINGO2	25	2.03
MMP3	25	2.03
BEND5	12.5	2.03
BIRC2	12.5	2.03
MMP10	12.5	2.03
MMP12	12.5	2.03
MMP8	12.5	2.03
PIEZO2	12.5	2.03
PNPT1	12.5	2.03
SNX9	12.5	2.03
YAP1	12.5	2.03
POSTN	100	2.03
EPHA7	87.5	2.03
FAM83B	87.5	2.03
CRBN	25	2.03
SLC6A15	25	2.03

CNTN4	12.5	2.03
MAPRE2	12.5	2.03
GRM3	100	2.02
GUCY1A2	100	2.02
SLITRK1	100	2.02
SLITRK6	100	2.02
ANKRD7	87.5	2.02
SEMA3A	75	2.02
ST7	75	2.02
ATRNL1	62.5	2.02
MGAT4C	37.5	2.02
CAPZA2	100	1.67
CAV1	100	1.67
DOCK2	100	1.67
CASP12	25	1.67
KBTBD8	25	1.67
EPYC	12.5	1.67
FNDC3A	12.5	1.67
GRIA4	12.5	1.67
SLC22A2	12.5	1.67
CDH11	100	1.62
NRXN3	100	1.62
GNAZ	62.5	1.62
LRRTM4	62.5	1.62
VWC2	37.5	1.62
GABRG1	25	1.62
GNPDA2	12.5	1.62
LRRTM1	12.5	1.62
TMEM17	12.5	1.62
SEMA3D	100	1.61
SLC10A2	100	1.61
SORCS1	100	1.61
GABRG2	87.5	1.61
CASP7	62.5	1.61
GABRB2	50	1.61
API5	12.5	1.61
DYNC2H1	12.5	1.61
MDFIC	12.5	1.61
NT5E	12.5	1.61
SNX14	12.5	1.61
TTLL7	12.5	1.61

CDH8	87.5	1.60
ACSS3	50	1.60
FST	50	1.60
SPATA5	37.5	1.60
SMAD4	12.5	1.60
SNRPN	12.5	1.60
PCDH10	100	1.26
FOXN3	87.5	1.26
PPP6C	87.5	1.26
PCDH20	75	1.26
TBC1D9	75	1.26
ABLIM1	62.5	1.26
HABP2	62.5	1.26
NHLRC2	62.5	1.26
RBBP8	62.5	1.26
VWA2	62.5	1.26
AFAP1L2	50	1.26
TAGAP	50	1.26
TDRD1	50	1.26
DCUN1D5	12.5	1.26
NR2E3	12.5	1.26
PHKB	12.5	1.26
COL19A1	100	1.25
NUP35	12.5	1.25
RARB	12.5	1.25
ZNRF4	12.5	1.25
DACH1	100	1.20
MAML3	100	1.20
PCDH17	100	1.20
RORB	50	1.20
PCDH7	37.5	1.20
KLF5	12.5	1.20
OSTF1	12.5	1.20
SNHG14	75	1.18
PRR30	12.5	1.18
CDKN1A	100	0.85
ELF2	100	0.85
KLHL1	100	0.85
MGST2	100	0.85
PCDH9	100	0.85
PRMT6	100	0.85

MEI4	87.5	0.85
CACUL1	62.5	0.85
CCDC172	62.5	0.85
NANOS1	62.5	0.85
DPYD	50	0.85
EIF3A	12.5	0.85
TDRD3	12.5	0.85
COL3A1	100	0.84
SLIT2	100	0.84
SUSD5	87.5	0.84
TLR4	50	0.84
NOL4	12.5	0.84
PCSK5	12.5	0.84
GAREM2	75	0.83

Supplementary Table S6. Commonly altered genes between mouse PtenI/Tp53I tumors and human PPAPA/TP53I breast cancers that have a neural connection

AFG3L2	EPHA6	ISL1	PPP3CB
APBA2	FEZF2	KCND2	RIT2
ATP2B2	GABRA1	LRRC4C	ROCK1
BDNF	GABRA2	MAP1S	SBF2
CACNA1C	GABRB2	MBP	SEMA3A
CACNA1F	GABRE	NCAM2	SEMA3D
CAMK4	GABRG1	NLGN1	SEMA6D
CTNNA2	GABRG2	NOVA1	SLC12A2
CTTNBP2	GABRG3	NRXN3	SLITRK1
CUL3	GRIA2	PCDHB11	SLITRK3
DCC	GRIA4	PCDHB14	SLITRK5
DCLK1	GRID2	PCDHB4	SLITRK6
DLGAP1	GRIK2	PCDHB5	TOP2B
DSCAML1	GRM3	PCDHB6	UNC5D
DTNA	GRM5	PCDHB9	USP14
EFNB2	GRM7	PCLO	SYT4
EPHA5	HTR1F	POU4F3	
